# Global, regional, and national levels and trends in older child, adolescent, and youth (5-24 years) all cause mortality from 1990 to 2024: modelling study 

**DOI:** 10.1136/bmj-2025-088685

**Published:** 2026-06-04

**Authors:** Danzhen You, Lucia Hug, Graeme Wilson Fell, Yang Liu, David Sharrow, Patrick Gerland, Bruno Masquelier, Leontine Alkema, Fengqing Chao, Michel Guillot, Thomas Spoorenberg, Bochen Cao, Kathleen Strong, Haidong Wang, Helena Cruz Castanheira, Emi Suzuki

**Affiliations:** 1 Unicef Office of Strategy and Evidence - Innocenti, New York, NY, USA; 2 Unicef Office of Strategy and Evidence - Innocenti, Florence, Italy; 3 Population Estimates and Projections, United Nations Department of Economic and Social Affairs, NY, USA; 4 Louvain University, Louvain-la-Neuve, Belgium; 5 University of Massachusetts Amherst, Amherst, MA, USA; 6 Chinese University of Hong Kong, Shenzhen, Shenzhen, Guangdong, China; 7 University of Pennsylvania, Philadelphia, PA, USA; 8 French Institute for Demographic Studies, Paris, France; 9 Demographic Analysis Section, Population Division, United Nations Department of Economic and Social Affairs, NY, USA; 10 Department of Data, Digital Health, Analytics and AI, World Health Organization, Geneva, Switzerland; 11 Science, Innovation and Monitoring Unit, Department of Sexual, Reproductive, Maternal, Child and Adolescent Health and Ageing, World Health Organization, Geneva, Switzerland; 12 CELADE - Population Division of United Nations Economic Commission for Latin America and the Caribbean, Santiago, Chile; 13 Development Data Group, World Bank, Washington DC, USA

## Abstract

**Objective:**

To estimate all cause mortality among children, adolescents, and youths aged 5-24 years for 200 countries and areas from 1990 to 2024, to assess mortality levels and trends, and to identify which regions and countries require the greatest investment.

**Design:**

Database construction of empirical data on mortality, mortality estimation, and assessment of levels and trends.

**Data and methods:**

Mortality databases were constructed from all available nationally representative data including vital registration data, sample vital registration data, household surveys, and population censuses to estimate mortality risk in the age groups in people aged 5 to 24 years.

**Results:**

In 2024, an estimated 2.1 million (90% uncertainty interval (UI) 2.1 million to 2.4 million) people aged 5-24 years died worldwide, representing 31% of all 7.0 million deaths under 25 years of age. This figure, an increase from 21% (3.3 million of 16.3 million) in 1990, reflects this age group’s increasing epidemiological importance as mortality in children under 5 years old declines faster. Globally, mortality risk was lowest at ages 10-14 years (2.7 (90% UI 2.5 to 3.3) deaths per 1000) and increased at ages 15-19 years (4.3 (4.1 to 4.6)) and 20-24 years (6.1 (5.7 to 7.7)). Male mortality was consistently higher than female mortality, with the male to female ratio increasing with age. Progress has been uneven: mortality fell by 64% for people aged 5-9 years from 1990 to 2024, compared with 33% for people aged 20-24 years, with slower declines in male mortality, particularly in older youth (26% reduction in male mortality *v* a 43% reduction in female mortality in people aged 20-24 years). Mortality declines have slowed since 2015, with increases in some low mortality regions, including North America, in people aged 10-24 years. In West and Central Africa, population growth outpaced mortality decline, increasing the absolute number of deaths. Deaths were increasingly concentrated in high mortality regions. Nearly half of deaths in 2024 occurred in just two regions: West and Central Africa, and Eastern and Southern Africa, which accounted for 23% (0.6 billion of 2.6 billion) of the global population of 5-24 year olds. This figure is an increase from 12% in 1990 (0.2 billion of 2.1 billion) and is projected to exceed 30% (0.9 billion of 2.6 billion) by 2050.

**Conclusion:**

Progress in reducing mortality in people aged 5-24 years has been uneven and has slowed. Urgent, context specific investments—particularly in high mortality regions—are needed to reduce preventable deaths and strengthen mortality monitoring data systems.

## Introduction

Although the global health community has historically prioritised mortality in children under 5 years of age, attention is increasingly being given to mortality in older children, adolescents, and youths. This shift is reflected in global health frameworks, including the sustainable development goals,^1^ the Global Strategy for Women’s, Children’s and Adolescents’ Health (2016-2030),^2^ Countdown to 2030,^3^ Global Action for Measurement of Adolescent Health,^4^ and the Lancet Commission on Adolescent Health and Wellbeing 2018,^5^ that recognise the importance of these age groups to public health, human capital, and the realisation of a demographic dividend.^3^ ^6^ ^7^ ^8^ ^9^ ^10^ ^11^ ^12^ Moreover, the global population of 5-24 year olds has grown by 25% since 1990, from 2.1 billion to 2.6 billion in 2024, increasing the number of people exposed to mortality risk.

Mortality in the 5-24 year old group remains lower than in children under 5 years of age.^7^ ^8^ Yet recent patterns show important changes: globally, the probability of dying between the ages of 15 and 25 years now exceeds the probability of dying between the ages of 1 and 5 years, reflecting faster declines in child mortality, alongside slower progress in people in the 5-24 year age group.^7^ These patterns align with the epidemiological transition in which mortality declines substantially at younger ages, followed by slower and more heterogeneous changes during adolescence and youth.^10^

Past gains in survival for people aged 5-24 years, driven by improved health, participation in the labour market later in life, and delayed parenthood, are increasingly threatened by climate change, migration and conflict, economic instability, and exposure to unhealthy commercial and social influences.^7^ ^11^ ^12^ Mortality increases among older children, adolescents, and youth in several high income countries^12^ underscore the need for systematic assessment of mortality. However, few studies provide recent, comparable estimates across countries, analyse trends by age, sex, and region, or assess future mortality burdens.

Using the latest estimates from the United Nations Interagency Group for Child Mortality Estimation (UN IGME)—produced in consultation with countries and based on the most recent and comprehensive data^13^—we provide global, regional, and national mortality estimates for people aged 5-24 years from 1990 to 2024. We assess differences by age, sex, and region, identify where progress has slowed or reversed, examine how population growth and age structure (ie, the distribution of various age groups and genders in a population) shape the distribution of deaths, and present scenario based projections from 2025 to 2030 to illustrate potential future mortality burdens. Together, these estimates and analyses aim to guide governments, researchers, and global partners in identifying priority areas for policy and investment to reduce mortality in older children, adolescents, and youth. 

## Data and methods

In this study, we estimated probabilities of dying for people in age groups 5-9 years, 10-14 years, 15-19 years, and 20-24 years, defined as the probability of dying within 5 year age interval and expressed as deaths per 1000 population at the start of the interval. We use the term older children for those aged 5-9 years, young adolescents for ages 10-14 years, older adolescents for ages 15-19 years, and youths for individuals aged 20-24 years. We compiled all available, nationally representative data on age specific probabilities of dying at ages 5-24 years, disaggregated by 5 year age groups and sex, for 200 countries and areas and applied bayesian models to estimate country specific trends until 2024. Estimates are produced annually in consultation with countries and using a common methodology, ensuring robust, timely, and comparable results.

### Databases

We obtained empirical data on the probabilities of dying at ages 5-24 years from vital registration and sample vital registration systems, population censuses, and household surveys.^7^ ^8^ ^9^ ^13^

For vital registration data, we derived probabilities from standard period abridged life tables, with sex specific completeness evaluated using death distribution methods.^14^ ^15^ In countries lacking sufficiently reliable vital registration and sample vital registration systems, we derived mortality probabilities from full birth histories for ages 5-14 years and sibling survival histories for ages 15-24 years, collected in household surveys and censuses. To a lesser extent, data on household deaths from censuses or surveys were also used to derive mortality estimates in some countries.

In total, 64 000 data points from more than 900 data series across 198 locations from 1950 to 2024 were used to estimate the probabilities of dying in people aged 5-24 years, grouped by sex. A detailed list of data sources is available in the supplementary appendix, and all empirical data and estimates are publicly available on the UN IGME web portal.^13^

### Estimation of mortality among older children, adolescents, and youth

To reconcile the possibly heterogenous data sources within a country, we used a two step bayesian approach, consistent with the approach applied in mortality estimation in children under 5 years of age.^7^ ^8^ ^16^ ^17^ Because combined sex data are generally more robust, we firstly estimated total (non-sex specific) probabilities of dying by using a bayesian B-spline bias reduction model (B3), which adjusts for source specific biases (including recall and selection bias) and accounts for sampling and non-sampling error.^17^ We estimated broader 10 year probabilities for ages 5-14 years and 15-24 years first, because the aggregated empirical data are generally more reliable. Estimates of 5-9 years and 15-19 years were then modelled and constrained using the 10 year probabilities, to ensure internal consistency. We calculated estimates of the 10-14 years and 20-24 years age groups from the 10 year and 5 year estimates. When recent empirical data were unavailable, we used a weighted combination of earlier country specific trends and global patterns to produce short term extrapolations of the probabilities from the most recent available empirical data point to 2024. On average, the extrapolation period was 3.9 years for the age group 5-14 years and 4.0 years for the age group 15-24 years.

For countries with insufficient data (ie, fewer than five observations or coverage of less than 10 years between 1990 and 2024) probabilities are modelled as a function of mortality in children under 5 years of age using multilevel regression models with regional (based on M49 regional classification^18^) effects. This is the case for 35 countries for people aged 5-14 years and 39 countries for people aged 15-24 years, representing 5% (38 000 of 835 000) and 10% (129 000 of 1.3 million) of the total burden of deaths in 2024, respectively. 

Secondly, we obtained sex/age specific estimates using a bayesian hierarchical time series model of sex ratios, ensuring consistency with total mortality.^9^ Available data by sex are firstly used to estimate the trend in the expected male to female sex ratio for each country-year. The estimated sex ratio is the product of the expected ratio and a country specific multiplier that is modelled from empirical data and represents the relative advantage or disadvantage of girls to boys compared to other countries at similar levels of mortality. For countries without empirical data, we used the expected ratio. A more detailed description of the modelling approaches is provided in the supplementary appendix sections on estimation.

### Crisis adjustments

Because data from vital registration systems, surveys, and censuses may not capture sudden mortality shocks, sex specific mortality estimates were adjusted for deaths from crisis events, such as conflicts, natural disasters, famines, and epidemics. We obtained numbers of crisis deaths from multiple databases^19^ ^20^ ^21^ ^22^ ^23^ ^24^ and reports from the United Nations and other organisations. Adjustments were applied to 69 countries for people aged 5-14 years and 62 countries for people aged 15-24 years. If detail about the sex and age was unavailable in crisis deaths, modelled age/sex patterns were applied.^25^ No adjustment was made for the covid-19 pandemic, as available evidence does not support such adjustment.^26^ ^27^

### Scenario based projections

We projected probability of death for all age groups (5-9 years, 10-14 years, 15-19 years, and 20-24 years) by sex for 2025-30 under five scenarios: constant 2024, continuing current trends, continuing current trends (lower bound), continuing current trends (upper bound), and achieving the 2024 high income country levels by 2030. The constant 2024 scenario maintains mortality at 2024 levels. The continuing current trends scenario applies country specific, crisis free median annual rates of reductions in the probabilities of dying for 2015-24; if projected mortality reaches the lowest observed level in 2024, mortality remains constant for the remainder of the projection period thereafter. Continuing current trends (lower bound) and continuing current trends (upper bound) use the continuing current trends approach, but apply the lower and upper bound of the 90% uncertainty interval (UI) of the annual rates of reductions. In the achieving high income country scenario, every country is projected to reach the average 2024 high income country probabilities of dying by 2030. Deaths were calculated using the estimated and projected probabilities and medium fertility variant population projections from the World Population Prospects 2024 revision.^28^ Regional and global deaths were calculated by aggregating country level estimates and projections.

### Analysis

We assessed all cause mortality levels and number of deaths from 1990 to 2024 by age, sex, region, and country. We evaluated progress using annual rates of reductions based on crisis free estimates to avoid short term volatility due to crisis events that may obscure underlying mortality trends. We assessed variation in mortality patterns across the early life course, comparing levels, trends and current mortality burden in young people aged 5-24 years with children under 5 years of age using the latest UN IGME estimates for that age group.^13^ Finally, we calculated the respective mortality and number of deaths under different scenarios from 2025-30 to assess the future mortality burden. Unless explicitly stated, analysis refers to the point estimate and estimates are presented including crisis adjustment.

### Patient and public involvement

This study uses publicly available nationally representative civil registration, survey, and population census data, so patients and the public were not involved in its design or conduct. Since no participants were recruited, no assessment of burden or participation experience was done.

## Results

### Current levels of older child, adolescent, and youth mortality

**Fig 1:**
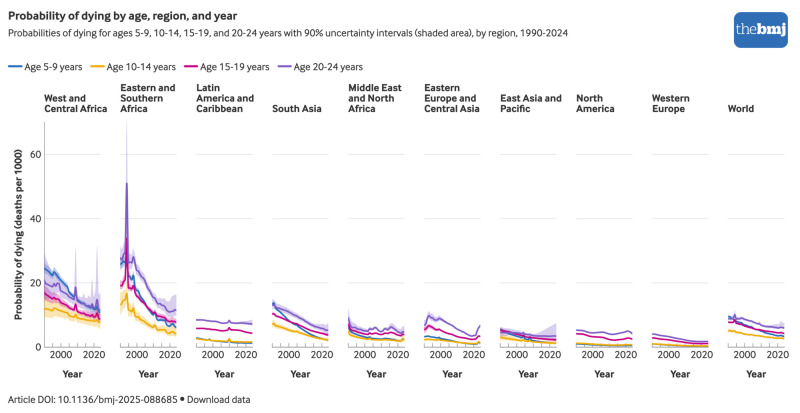
Probability of dying by age, region, and year. An interactive version of this figure can be accessed at https://public.flourish.studio/visualisation/28954515/

In 2024, the global probability of dying at ages 5-9 years was 3.4 deaths (90% UI 3.2 to 3.6) per 1000 children aged 5 years. Mortality was lowest at ages 10-14 years at 2.7 deaths (2.5 to 3.3) per 1000 adolescents aged 10 years, but not significantly (ie, uncertainty intervals overlap). Thereafter, the probability of dying increased with age to 4.3 deaths (90% UI 4.1 to 4.6) per 1000 adolescents aged 15 years for people aged 15-19 years and to 6.1 deaths (5.7 to 7.7) per 1000 youths aged 20 years for people aged 20-24 years (fig 1, table 1).

**Table 1:** Probabilities of dying for people aged 5-9 years, 10-14 years, 15-19 years, and 20-24 years expressed per 1000 survivors, by sex and region, in 1990 and 2024. Data are number (90% uncertainty interval) unless otherwise stated

Region	Probability of dying in people aged 5-9 years		Probability of dying in people aged 10-14 years		Probability of dying in people aged 15-19 years		Probability of dying in people aged 20-24 years	
	1990	2024	1990	2024	1990	2024	1990	2024
**West and Central Africa**								
Total	24.7 (23.2 to 26.4)	10.9 (9.9 to 11.9)	12.1 (9.3 to 16.4)	7.7 (5.7 to 11.0)	17.1 (15.0 to 19.9)	8.8 (7.8 to 10.3)	21.0 (15.9 to 30.1)	11.6 (9.4 to 16.9)
Female	24.0 (22.4 to 25.8)	10.2 (9.2 to 11.3)	12.1 (9.3 to 16.4)	7.3 (5.4 to 10.5)	17.8 (15.4 to 20.8)	7.8 (6.7 to 9.4)	19.9 (14.7 to 29.5)	10.4 (8.3 to 15.7)
Male	25.5 (23.9 to 27.4)	11.5 (10.4 to 12.7)	12.1 (9.3 to 16.4)	8.1 (6.0 to 11.6)	16.5 (14.3 to 19.4)	9.7 (8.5 to 11.6)	22.0 (16.6 to 31.5)	12.7 (10.3 to 18.6)
**Eastern and Southern Africa**								
Total	25.8 (24.0 to 27.8)	6.2 (5.6 to 6.7)	13.2 (10.0 to 17.1)	4.2 (3.3 to 6.6)	19.2 (18.1 to 21.0)	7.8 (7.0 to 8.8)	28.0 (25.0 to 31.8)	11.4 (9.6 to 16.4)
Female	24.6 (22.8 to 26.6)	5.8 (5.2 to 6.3)	13.0 (9.8 to 17.0)	3.7 (2.9 to 5.8)	18.5 (17.2 to 20.4)	5.8 (5.2 to 6.7)	24.3 (21.6 to 27.8)	7.9 (6.5 to 11.6)
Male	27.0 (25.0 to 29.2)	6.6 (6.0 to 7.2)	13.4 (10.2 to 17.4)	4.7 (3.6 to 7.4)	19.9 (18.5 to 22.0)	9.7 (8.6 to 11.2)	31.8 (27.9 to 36.6)	14.8 (12.2 to 21.7)
**Latin America and Caribbean**								
Total	3.0 (2.9 to 3.0)	1.3 (1.2 to 1.4)	2.7 (2.6 to 2.9)	1.7 (1.5 to 1.9)	5.9 (5.7 to 6.1)	4.4 (4.2 to 4.6)	8.6 (8.2 to 9.0)	7.2 (6.7 to 8.6)
Female	2.6 (2.6 to 2.7)	1.1 (1.1 to 1.2)	2.2 (2.1 to 2.4)	1.4 (1.3 to 1.6)	3.8 (3.7 to 4.1)	2.3 (2.2 to 2.5)	4.7 (4.3 to 5.1)	3.2 (3.0 to 3.9)
Male	3.3 (3.2 to 3.4)	1.4 (1.4 to 1.5)	3.2 (3.0 to 3.4)	1.9 (1.8 to 2.2)	7.9 (7.7 to 8.2)	6.4 (6.1 to 6.7)	12.5 (11.9 to 13.0)	11.2 (10.3 to 13.2)
**South Asia**								
Total	13.4 (12.9 to 13.8)	2.2 (2.1 to 2.4)	7.1 (6.0 to 8.2)	2.3 (1.8 to 3.1)	10.4 (10.0 to 10.9)	3.8 (3.4 to 4.4)	13.1 (11.8 to 15.0)	5.3 (3.9 to 7.6)
Female	13.9 (13.2 to 14.6)	2.0 (1.8 to 2.2)	7.1 (6.0 to 8.3)	2.0 (1.6 to 2.7)	11.4 (10.7 to 12.2)	3.4 (2.9 to 4.0)	13.6 (12.1 to 15.6)	4.1 (2.9 to 6.3)
Male	12.9 (12.3 to 13.5)	2.4 (2.2 to 2.7)	7.0 (6.0 to 8.2)	2.6 (2.0 to 3.4)	9.4 (8.8 to 10.1)	4.2 (3.6 to 4.9)	12.6 (11.3 to 14.7)	6.4 (4.6 to 9.3)
**Middle East and North Africa**								
Total	6.7 (6.5 to 7.0)	2.2 (2.0 to 2.5)	5.1 (4.1 to 6.3)	2.3 (1.8 to 3.5)	7.4 (6.7 to 8.2)	3.9 (3.7 to 4.3)	9.4 (7.7 to 12.4)	4.8 (4.0 to 6.6)
Female	6.3 (6.0 to 6.7)	2.0 (1.8 to 2.3)	4.7 (3.7 to 5.9)	1.9 (1.4 to 3.0)	5.1 (4.6 to 5.9)	2.4 (2.1 to 2.7)	5.4 (4.2 to 7.5)	2.6 (2.1 to 3.7)
Male	7.1 (6.8 to 7.4)	2.4 (2.2 to 2.8)	5.4 (4.5 to 6.8)	2.6 (2.0 to 3.9)	9.4 (8.6 to 10.5)	5.4 (5.0 to 6.0)	13.0 (10.7 to 17.1)	6.8 (5.7 to 9.5)
**Eastern Europe and Central Asia**								
Total	3.4 (3.3 to 3.4)	1.1 (1.1 to 1.1)	2.4 (2.0 to 2.7)	1.3 (1.3 to 1.4)	5.6 (4.6 to 6.4)	3.3 (3.2 to 3.4)	6.9 (5.9 to 8.5)	6.8 (6.2 to 7.5)
Female	2.7 (2.6 to 2.8)	1.0 (0.9 to 1.0)	1.7 (1.5 to 2.0)	1.1 (1.0 to 1.1)	3.8 (2.9 to 4.6)	1.9 (1.8 to 2.0)	3.4 (2.9 to 4.3)	2.5 (2.3 to 2.9)
Male	4.0 (3.9 to 4.1)	1.3 (1.3 to 1.3)	3.0 (2.6 to 3.4)	1.5 (1.5 to 1.6)	7.3 (6.1 to 8.2)	4.7 (4.5 to 5.0)	10.2 (8.7 to 12.5)	11.0 (9.8 to 12.5)
**East Asia and Pacific**								
Total	5.6 (5.1 to 6.2)	1.3 (1.1 to 1.5)	3.1 (2.2 to 4.4)	1.3 (0.9 to 2.1)	5.4 (4.8 to 6.0)	2.3 (1.9 to 2.8)	4.6 (3.4 to 6.3)	3.5 (2.3 to 7.5)
Female	5.2 (4.7 to 5.7)	1.1 (0.9 to 1.2)	2.7 (1.9 to 3.7)	1.0 (0.7 to 1.6)	3.8 (3.3 to 4.4)	1.4 (1.2 to 1.8)	2.9 (2.1 to 4.1)	1.9 (1.3 to 4.0)
Male	6.0 (5.4 to 6.7)	1.5 (1.3 to 1.7)	3.5 (2.5 to 5.0)	1.5 (1.1 to 2.5)	6.9 (6.1 to 7.8)	3.1 (2.5 to 3.8)	6.3 (4.5 to 8.6)	4.9 (3.1 to 10.7)
**North America**								
Total	1.1 (1.1 to 1.1)	0.6 (0.6 to 0.6)	1.3 (1.3 to 1.3)	0.8 (0.8 to 0.9)	4.3 (4.2 to 4.3)	2.5 (2.4 to 2.6)	5.4 (5.2 to 5.6)	4.3 (3.7 to 4.8)
Female	0.9 (0.9 to 1.0)	0.5 (0.5 to 0.6)	1.0 (0.9 to 1.0)	0.7 (0.6 to 0.8)	2.3 (2.2 to 2.3)	1.5 (1.3 to 1.6)	2.5 (2.4 to 2.6)	2.4 (2.0 to 2.8)
Male	1.3 (1.2 to 1.3)	0.7 (0.6 to 0.7)	1.6 (1.5 to 1.7)	1.0 (0.9 to 1.1)	6.1 (6.0 to 6.2)	3.5 (3.3 to 3.7)	8.1 (7.8 to 8.5)	6.0 (5.2 to 6.8)
**Western Europe**								
Total	1.1 (1.1 to 1.1)	0.4 (0.4 to 0.4)	1.1 (1.1 to 1.1)	0.5 (0.4 to 0.5)	3.0 (2.9 to 3.0)	1.2 (1.2 to 1.2)	4.1 (4.1 to 4.2)	1.8 (1.8 to 1.9)
Female	0.9 (0.9 to 0.9)	0.3 (0.3 to 0.4)	0.9 (0.8 to 0.9)	0.4 (0.4 to 0.4)	1.6 (1.6 to 1.6)	0.8 (0.8 to 0.8)	1.9 (1.9 to 2.0)	1.0 (1.0 to 1.1)
Male	1.3 (1.2 to 1.3)	0.4 (0.4 to 0.4)	1.3 (1.3 to 1.3)	0.5 (0.5 to 0.5)	4.2 (4.2 to 4.3)	1.6 (1.5 to 1.6)	6.3 (6.2 to 6.4)	2.6 (2.5 to 2.7)
**World**								
Total	9.6 (9.3 to 9.9)	3.4 (3.2 to 3.6)	5.1 (4.7 to 5.8)	2.7 (2.5 to 3.3)	7.8 (7.6 to 8.2)	4.3 (4.1 to 4.6)	9.0 (8.4 to 10.0)	6.1 (5.7 to 7.7)
Female	9.3 (9.1 to 9.7)	3.2 (3.0 to 3.3)	4.9 (4.5 to 5.5)	2.4 (2.2 to 3.0)	6.9 (6.7 to 7.3)	3.3 (3.1 to 3.6)	7.2 (6.7 to 8.1)	4.1 (3.8 to 5.4)
Male	9.8 (9.5 to 10.1)	3.6 (3.5 to 3.8)	5.4 (4.9 to 6.1)	3.0 (2.7 to 3.7)	8.7 (8.4 to 9.1)	5.3 (5.0 to 5.6)	10.7 (10.0 to 12.0)	7.9 (7.4 to 10.0)

**Fig 9:**
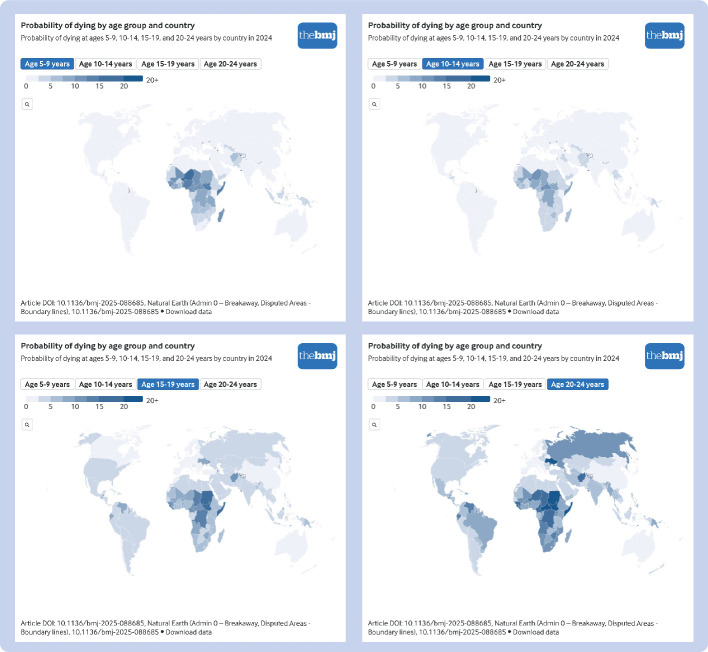
Probability of dying in 2024 by age group and country. An interactive version of this figure can be accessed at https://public.flourish.studio/visualisation/28960360/

Regional disparities were substantial. West and Central Africa and Eastern and Southern Africa had the highest and second highest probabilities of dying, respectively, across all age groups in 2024, followed by South Asia and the Middle East and North Africa for age groups 5-9 years and 10-14 years, and Latin America and the Caribbean for the age group 15-19 years. Western Europe had the lowest levels across ages (fig 1). Disparities were larger in younger age groups. In 2024, for example, probability of dying at ages 5-9 years in West and Central Africa was 29 times higher than in Western Europe, while probability of dying at ages 20-24 years was about six times higher (fig 1, table 1). Extremely high mortality levels were often associated with crises such as conflict or natural disasters. In 2024, the highest probabilities of dying across all age groups were estimated for the State of Palestine (fig 9).

**Fig 2:**
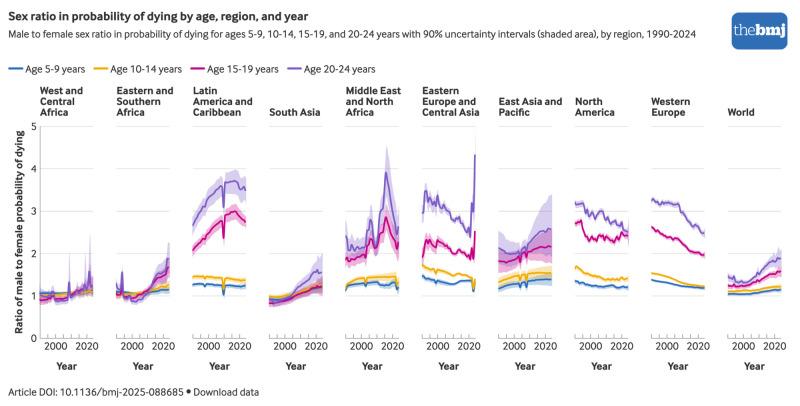
Sex ratio in probability of dying by age, region, and year. An interactive version of this figure can be accessed at https://public.flourish.studio/visualisation/28955315/

Globally, male mortality was higher than female mortality across all age groups, with sex differentials widening with age (fig 2). The male to female mortality ratio increased from 1.15 (90% UI 1.10 to 1.20) at ages 5-9 years to 1.23 (1.16 to 1.29) at ages 10-14 years, 1.59 (1.47 to 1.71) at ages 15-19 years, and by ages 20-24 years, the male mortality risk was almost double that of the female mortality risk, with a ratio of 1.90 (1.67 to 2.12) at ages 20-24 years. This pattern was broadly consistent across regions, except in South Asia where female mortality exceeded male mortality at ages 5-9 years and 10-14 years until recently. The highest sex ratios were observed for ages 20-24 years in Eastern Europe and Central Asia, Latin America and Caribbean, and Middle East and North Africa (fig 2), where the male probability of dying at 20-24 years of age was estimated to be at least three times that of the female probability of dying in the same age group. Sex ratios exceed 1.5 in more than 80% of countries at ages 15-19 years and 20-24 years (167 and 176 countries of 200, respectively), compared to 26% (51 countries) at ages 10-14 years and 4% (7 countries) at ages 5-9 years.

**Fig 3:**
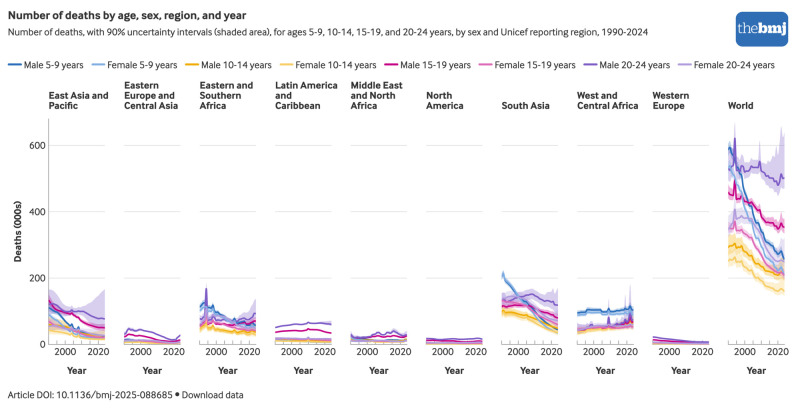
Number of deaths by age, sex, region, and year. An interactive version of this figure can be accessed at https://public.flourish.studio/visualisation/28955498/

**Fig 4:**
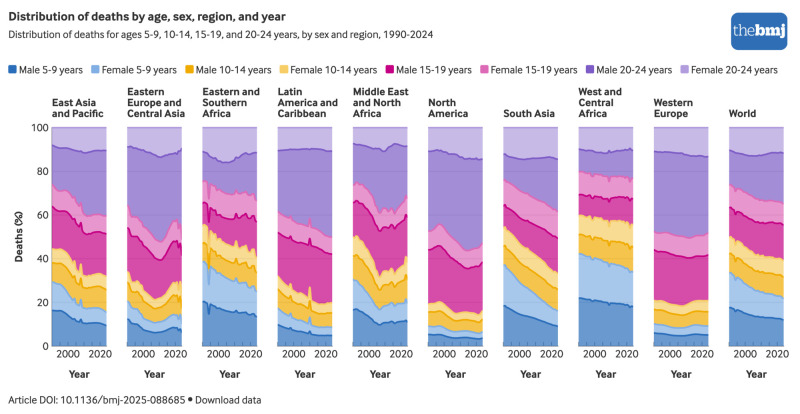
Distribution of deaths by age, sex, region, and year. An interactive version of this figure can be accessed at https://public.flourish.studio/visualisation/28971761/

Deaths were concentrated in older age groups, in men, and in high mortality regions. In 2024, an estimated 2.1 (90% UI 2.1 to 2.4) million deaths occurred in people aged 5-24 years. Of these, 0.8 million (90% UI 0.7 to 1.0) occurred at ages 20-24 years of age, 0.6 million (0.5 to 0.6) at 15-19 years, 0.5 million (0.4 to 0.5) at 5-9 years of age, and 0.4 million (0.3 to 0.5) at 10-14 years of age. The proportion of deaths attributed to men increased with age, from 55% (90% UI 54% to 56%) at ages 5-9 years to 57% (55% to 58%) at ages 10-14 years, 63% (61% to 65%) at ages 15-19 years, and 67% (64% to 69%) at ages 20-24 years. Nearly 70% (1.5 million, 90% UI 1.4 million to 1.8 million) of deaths at ages 5-24 years in 2024 occurred in West and Central Africa, Eastern and Southern Africa, and South Asia (table 2, fig 3, fig 4).

**Table 2:** Number of deaths (thousands) for ages 5-9 years, 10-14 years, 15-19 years, and 20-24 years, by sex and region in 1990 and 2024. Data are number (90% uncertainty interval) unless otherwise stated

Region		Number of deaths in age group 5-9 years		Number of deaths in age group 10-14 years		Number of deaths in age group 15-19 years		Number of deaths in age group 20-24 years	
		1990	2024	1990	2024	1990	2024	1990	2024
**West and Central Africa**									
Total		183 (172 to 195)	194 (176 to 213)	75 (58 to 102)	124 (92 to 177)	88 (77 to 102)	122 (107 to 143)	89 (67 to 129)	134 (109 to 197)
Female		88 (82 to 95)	90 (81 to 100)	37 (29 to 50)	58 (43 to 83)	45 (39 to 53)	53 (46 to 64)	42 (31 to 63)	60 (47 to 91)
Male		95 (89 to 102)	104 (93 to 114)	38 (29 to 51)	66 (48 to 94)	42 (37 to 50)	68 (59 to 81)	47 (35 to 67)	75 (61 to 110)
**Eastern and Southern Africa**									
Total		214 (199 to 230)	107 (97 to 115)	91 (69 to 118)	66 (51 to 104)	113 (106 to 123)	112 (101 to 127)	139 (124 to 158)	142 (120 to 206)
Female		101 (94 to 109)	49 (44 to 54)	45 (34 to 58)	29 (23 to 45)	54 (50 to 60)	41 (37 to 48)	60 (54 to 69)	50 (41 to 73)
Male		113 (104 to 122)	58 (52 to 63)	46 (35 to 60)	37 (29 to 59)	59 (55 to 65)	70 (62 to 81)	79 (69 to 91)	93 (77 to 137)
**Latin America and Caribbean**									
Total		32 (31 to 33)	13 (12 to 14)	27 (26 to 29)	17 (16 to 20)	54 (53 to 55)	46 (44 to 48)	70 (67 to 74)	77 (71 to 91)
Female		14 (13 to 14)	6 (5 to 6)	11 (10 to 12)	7 (7 to 8)	17 (17 to 18)	12 (11 to 13)	19 (18 to 21)	17 (15 to 20)
Male		18 (18 to 18)	7 (7 to 8)	16 (15 to 17)	10 (9 to 12)	36 (35 to 38)	34 (32 to 36)	51 (49 to 53)	60 (55 to 71)
**South Asia**									
Total		405 (391 to 420)	79 (72 to 85)	186 (159 to 216)	84 (64 to 111)	242 (232 to 253)	139 (122 to 158)	268 (242 to 306)	189 (139 to 274)
Female		203 (193 to 213)	34 (31 to 38)	90 (77 to 105)	35 (27 to 47)	129 (121 to 137)	60 (50 to 70)	135 (120 to 154)	71 (50 to 109)
Male		202 (193 to 212)	44 (40 to 49)	96 (82 to 112)	48 (37 to 65)	113 (106 to 121)	79 (68 to 93)	133 (119 to 155)	118 (85 to 172)
**Middle East and North Africa**									
Total		50 (48 to 52)	23 (21 to 26)	31 (26 to 39)	23 (17 to 34)	38 (35 to 43)	35 (32 to 38)	43 (35 to 57)	39 (33 to 54)
Female		23 (22 to 24)	10 (9 to 12)	14 (11 to 18)	9 (7 to 15)	13 (11 to 15)	10 (9 to 12)	12 (9 to 16)	10 (8 to 14)
Male		27 (26 to 28)	13 (12 to 14)	17 (14 to 22)	13 (10 to 20)	25 (23 to 28)	25 (22 to 27)	31 (25 to 41)	29 (24 to 40)
**Eastern Europe and Central Asia**									
Total		24 (24 to 24)	7 (7 to 7)	16 (14 to 18)	8 (8 to 8)	36 (29 to 40)	18 (18 to 19)	42 (35 to 51)	35 (32 to 39)
Female		10 (9 to 10)	3 (3 to 3)	6 (5 to 7)	3 (3 to 3)	12 (9 to 14)	5 (5 to 5)	10 (9 to 13)	6 (6 to 7)
Male		14 (14 to 15)	4 (4 to 4)	10 (9 to 12)	5 (5 to 5)	24 (20 to 27)	13 (13 to 14)	31 (27 to 39)	28 (26 to 32)
**East Asia and Pacific**									
Total		200 (181 to 220)	39 (34 to 45)	103 (73 to 144)	41 (29 to 67)	204 (184 to 229)	71 (58 to 87)	176 (128 to 240)	104 (68 to 221)
Female		89 (80 to 98)	16 (13 to 18)	43 (31 to 60)	15 (11 to 25)	70 (61 to 82)	21 (17 to 27)	54 (39 to 77)	27 (18 to 57)
Male		111 (100 to 123)	24 (20 to 28)	60 (42 to 84)	26 (18 to 43)	134 (119 to 152)	50 (40 to 62)	121 (88 to 166)	77 (49 to 167)
**North America**									
Total		4 (4 to 5)	3 (3 to 3)	5 (5 to 5)	4 (4 to 4)	17 (16 to 17)	13 (12 to 13)	23 (22 to 24)	21 (18 to 24)
Female		2 (2 to 2)	1 (1 to 1)	2 (2 to 2)	2 (1 to 2)	4 (4 to 4)	4 (3 to 4)	5 (5 to 6)	6 (5 to 7)
Male		3 (3 to 3)	1 (1 to 2)	3 (3 to 3)	2 (2 to 3)	12 (12 to 12)	9 (9 to 9)	18 (17 to 19)	16 (13 to 18)
**Western Europe**									
Total		6 (6 to 6)	2 (2 to 2)	7 (6 to 7)	2 (2 to 3)	19 (19 to 19)	6 (6 to 7)	29 (29 to 29)	10 (10 to 10)
Female		3 (3 to 3)	1 (1 to 1)	3 (2 to 3)	1 (1 to 1)	5 (5 to 5)	2 (2 to 2)	7 (7 to 7)	3 (3 to 3)
Male		4 (4 to 4)	1 (1 to 1)	4 (4 to 4)	1 (1 to 1)	14 (14 to 14)	4 (4 to 4)	22 (22 to 23)	7 (7 to 8)
**World**									
Total		1,118 (1,088 to 1,150)	466 (443 to 488)	540 (496 to 611)	369 (337 to 456)	810 (784 to 845)	561 (536 to 600)	879 (822 to 979)	752 (708 to 955)
Female		531 (515 to 549)	210 (199 to 221)	250 (229 to 283)	160 (145 to 199)	350 (336 to 369)	208 (196 to 227)	346 (322 to 388)	249 (231 to 323)
Male		586 (568 to 605)	256 (243 to 270)	291 (266 to 329)	209 (191 to 259)	460 (441 to 483)	353 (335 to 379)	533 (496 to 599)	503 (471 to 641)

### Trends in older child, adolescent, and youth mortality

**Fig 5:**
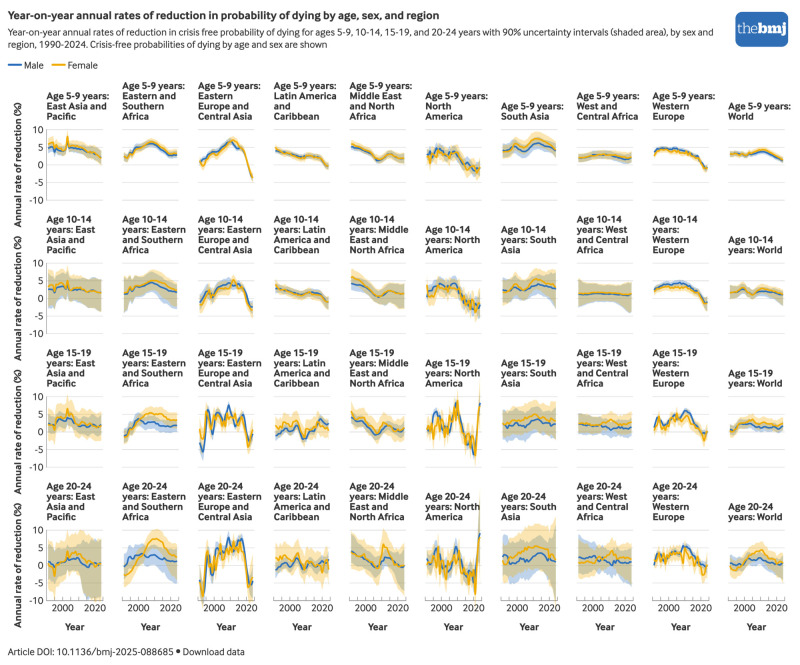
Year-on-year annual rates of reduction in probability of dying by age, sex, and region. An interactive version of this figure can be accessed at https://public.flourish.studio/visualisation/28956897/

**Fig 6:**
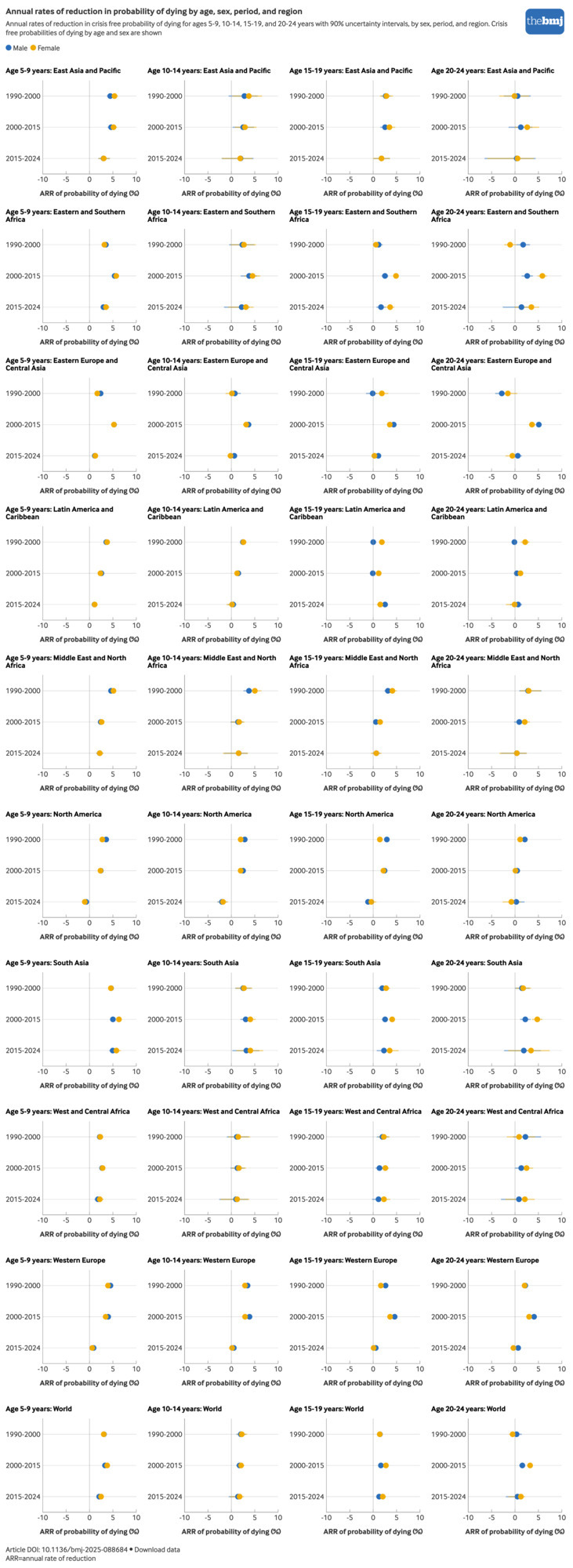
Annual rates of reduction in probability of dying by age, sex, period, and region. An interactive version of this figure can be accessed at https://public.flourish.studio/visualisation/28956993/

Global mortality in people aged 5-24 years declined substantially between 1990 and 2024 (table 1), with the probability of dying between the ages of 5 years and 24 years falling from 31.2 (90% UI 30.7 to 32.5) to 16.4 (16.1 to 18.4) per 1000 survivors to age 5 years. Mortality declined faster in people aged 5-14 years than in people aged 15-24 years, with variation by region and sex (fig 1, fig 5, fig 6). Female mortality declined more rapidly than male mortality, particularly at ages 15-24 years where annual rates of reductions for people in the 15-19 years and 20-24 years age groups were more than 1.5 times higher for female mortality. Differences in pace of decline were smaller in people aged 5-14 years, with slightly faster progress in reducing female mortality.

Sex differences in trends varied by region. Female mortality declined faster than male mortality in Eastern and Southern Africa and South Asia, especially at older ages, increasing the mortality sex ratio. In contrast, Western Europe, North America and Eastern Europe and Central Asia had smaller differences by sex, including some age groups with greater male mortality decline.

Globally, progress has not accelerated since 1990 (fig 5, fig 6) and slowed in several regions after excluding crisis related shocks. For people aged 5-9 years, the global annual rates of reductions fluctuated between 1.3% and 4.0% and slowed moderately after 2012 (fig 5). Reductions were slower in Western and Central Africa than in Eastern and Southern Africa, despite similar mortality levels in 1990. In the last decade, lower mortality regions like North America, Western Europe, Eastern Europe and Central Asia, and Latin America and Caribbean recorded the lowest annual rates of reductions, with negative annual rates of reductions in some cases, particularly in North America, indicating an increase in mortality levels. Conversely, South Asia maintained the fastest declines, especially for female mortality, and was also the only region with notable sex differences in annual rates of reductions, with male mortality declining slower than female mortality (fig 5, fig 6). Mortality was higher in girls than in boys aged 5-9 years in South Asia in the 1990s but has since reversed.

In people aged 10-14 years, the global annual rates of reductions fluctuated between 1.0% and 2.8% between 1990 and 2024 (fig 5) and remained stable across the three periods (ie, 1990-2000, 2000-2015, and 2015-2024), while slowing slightly (fig 6). As at younger ages, declines in mortality were slower in Western and Central Africa than Eastern and Southern Africa. In regions with lower mortality, progress has slowed considerably in recent years, with stagnation or increases in mortality in North America, Western Europe, Eastern Europe and Central Asia, and Latin America and Caribbean. Mortality increased in North America from 2015 for both sexes. Declines in female mortality were slightly faster in higher mortality regions, and slower in lower mortality regions.

In people aged 15-19 years, the global annual rates of reductions varied between 0.3% and 2.8% between 1990 and 2024 and slowed moderately after 2006. Declines slowed recently in lower mortality regions of North America, Western Europe, and Eastern Europe and Central Asia. Globally, female mortality declined faster than male mortality during 1990-2024 (2.2% (90% UI 1.9% to 2.4%) *v* 1.5% (1.2% to 1.7%)), driven by high mortality regions, such as West and Central Africa, Eastern and Southern Africa, and South Asia, where mortality sex ratios remained lower than in lower mortality regions.

For people aged 20-24 years, global annual rates of reductions fluctuated between −0.6% and 2.9% between 1990 and 2024. Reductions were lower than at younger ages, slowing markedly during 2015-24 to 0.6% (90% UI −1.8% to 1.2%), from 2.1% (1.4% to 2.6%) during 2000-15 (fig 6). In low mortality regions, declines stagnated or reversed, most noticeably in North America, although annual rates of reductions increased slightly after 2022. Sex differences mirrored those at ages 15-19 years but were more pronounced, especially from 2000 to 2015. For example, in Eastern and Southern Africa during 2000-15, female annual rate of reduction was 5.7% (90% UI 4.7% to 6.7%) compared with male rates of reduction of 2.4% (1.3% to 3.6%). 

Most countries experienced substantial mortality declines across all four age groups between 1990 and 2024. Of 200 countries, 142 achieved reductions of more than 25% across all four age groups (135 for female mortality and 128 for male mortality) and 57 achieved reductions exceeding 50% (54 for female mortality and 54 for male). However, 30 countries showed no progress in at least one age group since 1990. More countries achieved reductions among the younger age groups than among the older: 160 countries reduced the probability of dying in the 5-9 years age group by at least half, compared with 83 for the probability of dying between at 20-24 years of age. Since 2015, more countries have experienced stagnation or increases than in the earlier periods. For example, during 2015-24, more than 50 countries had increased total mortality in people aged 20-24, compared to 27 during 2000-15.

Despite declines in probabilities of dying, reductions in the numbers of deaths were offset by population growth in some regions. The regions of sub-Saharan Africa accounted for almost 50% (1.0 million of 2.1 million) of deaths in people aged 5-24 years in 2024 (up from about 30% in 1990, or 1.0 million of 3.3 million), while their share of the global population in this age group increased from 12% (0.2 billion of 2.1 billion) to 23% (0.6 billion of 2.6 billion) and is projected to exceed 30% (0.9 billion of 2.6 billion) by 2050.^28^ In the regions of sub-Saharan Africa, mortality declines in people aged 15-24 years were not sufficient to overcome population growth, resulting in an increase in the absolute number of deaths in 2024 compared to 1990 (fig 3, fig 4, fig 5, and table 2). For Western and Central Africa, this was also the case for the younger age groups. In West and Central Africa, deaths increased in people older than 10 years and remained stable at children aged 5-9 years (fig 3, fig 4). In Eastern and Southern Africa, deaths in people aged 5-14 years declined because of larger declines in the mortality levels, but deaths remained stable in people aged 15-24 years. 

**Fig 7:**
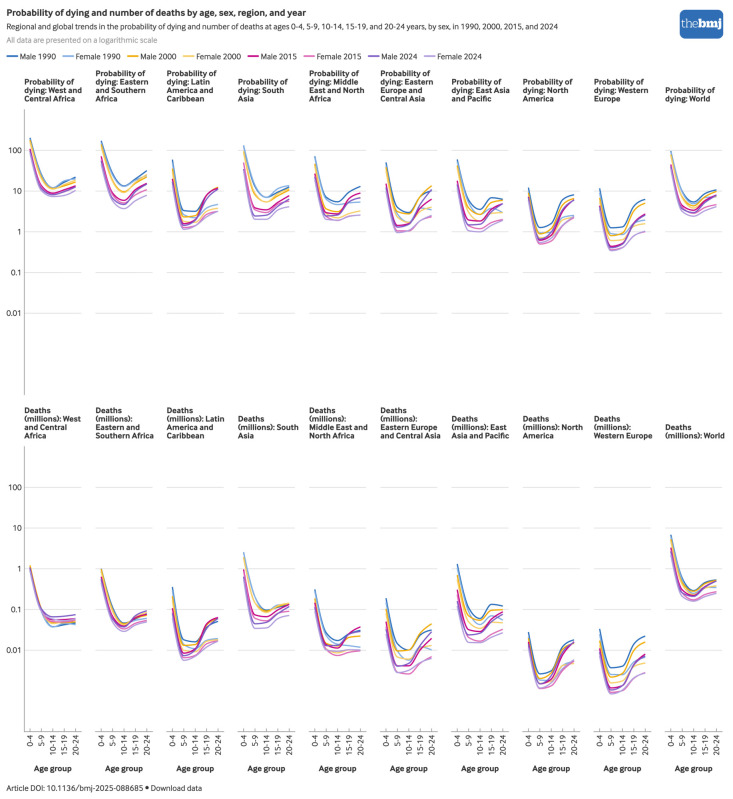
Probability of dying and number of deaths by age, sex, region, and year. An interactive version of this figure can be accessed at https://public.flourish.studio/visualisation/28959061/

**Fig 8:**
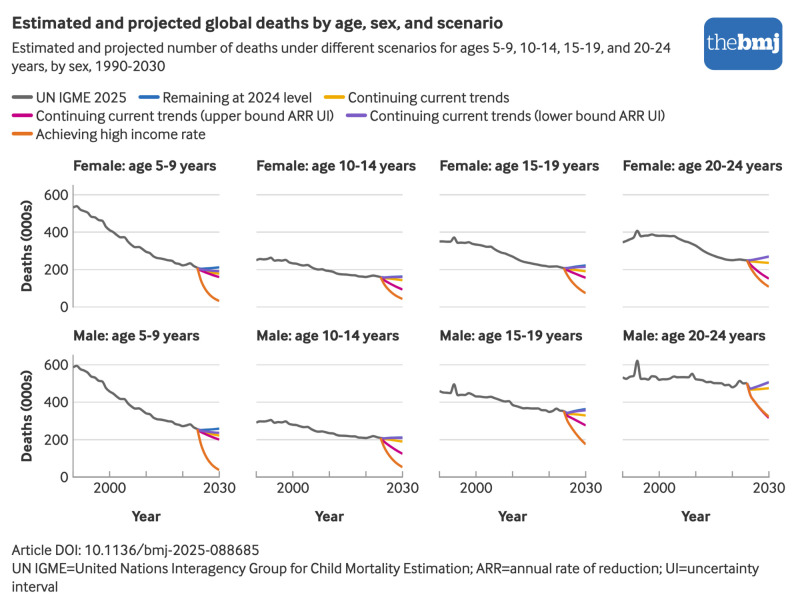
Estimated and projected global deaths by age, sex, and scenario. An interactive version of this figure can be accessed at https://public.flourish.studio/visualisation/28972066/

Faster declines in mortality in people younger than 5 years of age than in people aged 5-24 years have altered age patterns of mortality. Mortality in children younger than 5 years old fell by 60% since 1990, compared to 47% in people aged 5-24 years, reducing the ratio of mortality in children younger than 5 years to mortality in people aged 5-24 years from 3.0 (90% UI 2.9 to 3.1) in 1990 to 2.3 (2.0 to 2.4) in 2024. Regional mortality differences are more pronounced: for example, Latin America and the Caribbean has higher mortality in men aged 15-24 years than East Asia and the Pacific, a region that has similar levels of mortality in children under 5 years. In North America, Eastern Europe and Central Asia, Western Europe, and Latin America and the Caribbean, mortality levels in people aged 20-24 years are now similar to levels of mortality in children under 5 years (fig 7, fig 8).

In terms of overall burden, deaths in 5-24 year olds accounted for 31% (90% UI 30% to 33%) of all deaths in people under the age of 25 years in 2024 (2.1 million of 7.0 million deaths), up from 21% (90% UI 20% to 21%) in 1990 (3.3 million of 16.3 million deaths). The share of deaths in people aged 15-24 years increased in most regions, with larger numbers of male deaths. 

### Projection scenarios

The scenario based projections show that if mortality were to remain at 2024 levels, the projected annual number of deaths from 2025 through 2030 will be slightly higher than in 2024 owing to population increases in regions with higher mortality levels, with almost 12.9 million deaths over the years 2025 to 2030 (fig 8). If the rate of decline observed in each country between 2015 and 2024 was to continue, the number of deaths in people in the age group 5-24 years will decline slightly, leading to 12.0 million projected deaths among older children, adolescents, and youths between 2025 and 2030 (7.4 million male and 4.6 million female). The largest share of these deaths—23% (2.8 million deaths)—will be in men in the age group 20-24 years. Between 2025 and 2030, 4.8 million deaths could be averted compared to the scenario wherein past trends continue, if all countries achieve the average mortality levels in high income countries by 2030. The vast majority of these deaths (84%, or 4.0 million) would be averted in the regions of West and Central Africa, Eastern and Southern Africa, and South Asia. Taking the uncertainty in the estimates into account under the scenario where the upper bound of the UIs for the rate of decline in each country between 2015 and 2024 continued, 10.2 million deaths are projected and 1.8 million deaths could be averted compared to the scenario using the median rate of decline. If all countries performed at the lower bound of the UIs for the rate of decline from the 2015-24 period, 12.7 million deaths will occur, only slightly better than the scenario with no change to the current mortality levels.

## Discussion

Globally, mortality declined substantially among children, adolescents, and youths from 1990 to 2024, yet progress has been highly uneven across regions, age groups, and sexes. It is estimated that 2.1 million deaths occurred among children, adolescents, and young adults aged 5-24 years in 2024, a high burden, increasingly concentrated in the regions of sub-Saharan Africa, where the number of deaths has increased since 1990 because mortality declines have been outpaced by population growth as noted by Masquelier et al.^7^ The increasing share stems from slow progress in reducing mortality, particularly in Western and Central Africa, and regional demographic patterns; sub-Saharan Africa represents 23% (0.6 billion of 2.6 billion) of the world’s population of people aged 5-24 years old in 2024, up from 12% (0.2 billion of 2.1 billion) in 1990. Population projections indicate that this proportion will continue to rise, surpassing 30% (0.9 billion of 2.6 billion) by 2050.^28^

The data reveal a persistent and widening gap between male mortality and female mortality, particularly in older adolescence and youth, as found in other studies.^9^ ^11^ Globally, the annual rate of reduction for female mortality in the 15-24 years age group was more than 1.5 times that of male mortality. By 2024, the probability of dying for men aged 20-24 was nearly double that of women. In Latin America and Caribbean, this disparity is most extreme, with male mortality risk reaching more than three times that of female mortality in the 20-24 years age group. The higher risk for men in the region in particular is often associated with the increased contribution of violent deaths and injuries at these ages.^9^ ^29^ These findings underscore the need for gender responsive health interventions that specifically address the distinct behavioural and environmental risks faced by young men.

Apart from the risk in early childhood, adolescents, and youth show an increased risk of mortality, and this pattern is more pronounced in regions and countries with lower mortality risks. Deaths in the 5-24 years age range are also a growing share of all deaths in people under the age of 25 years: mortality declines in children under 5 years of age have outpaced that of the older age groups. Globally, the risk of dying at 5-24 years of age is slightly less than half of the risk of dying before reaching 5 years of age, but in several lower mortality regions, the risk of dying between ages 5 and 24 years is higher than for children under 5 years.

A key finding of this study is the stagnation—and in some cases, reversal—of mortality improvements in regions that had previously achieved low mortality levels, most noticeably in North America, Western Europe, Eastern Europe and Central Asia, and Latin America and the Caribbean. This alarming trend aligns with recent warnings from the Global Burden of Disease 2023^12^ study regarding a potential health crisis in youths in North America.

Given the current trends of slow decline in probability of dying, and no acceleration in the rate of reduction of mortality risk**,** we do not expect large reductions in the number of deaths until 2030 without increased investments. With no change in current levels of mortality risk, 12.9 million older children, adolescents, and youth are expected to die between 2025 and 2030. If current rates of progress are maintained the number of deaths would amount to 12.0 million. The high income scenario illustrates the potential in saving 4.8 million lives with accelerated progress compared to the current trend scenario over the same period. More realistic scenarios using uncertainty in the estimates with the upper bound of the estimated annual rates of reduction between 2015 and 2024 suggests that the number of deaths could be reduced to 10.2 million deaths, while using the lower bound of the annual rates of reduction could increase the number of deaths to 12.7 million deaths.

Understanding the underlying factors driving mortality risks and how they evolve with age is another important element of global health monitoring. In adolescents and youth, injuries, violence, and non-communicable diseases are understood to be major contributors to mortality. For older children aged 5-9 years, childhood illnesses, diarrhoea, malaria, and lower respiratory infections remain the leading causes of death. Because these factors are more readily treatable with traditional public health interventions in children aged 5-9 years, combined with a lower contribution from injuries and accidents, likely explains the faster decline in mortality in children aged 5-9 years in recent decades. The reversals for older adolescents and youths suggest that emerging threats such as interpersonal violence, self-harm, and road traffic injuries—causes of death less responsive to traditional infectious disease control measures—are becoming dominant drivers of mortality in these age groups. A detailed analysis on causes of death for children and adolescents younger than 20 years old was conducted by the Child and Adolescent Causes of Death Estimation (CA-CoDE) group.^29^ ^30^

This study offers comprehensive analysis of all cause mortality in people aged 5-9 years, 10-14 years, 15-19 years, and 20-24 years by sex, building on the standardised modelling approach used by the UN IGME and provides timely estimates up to the year 2024. Moreover, the UN IGME updates its estimates of mortality in 5-24 year olds annually in consultation with national governments, ensuring the data compiled are complete and nationally representative. However, the lack or sparsity of empirical data in several high mortality countries limited the study’s precision, particularly in more recent estimates with only 37 countries with data for 2024 and an average short term extrapolation period of around four years, increasing the uncertainty in recent years. Based on the lower bound of the 90% UIs, only 75 countries reduced mortality by at least 25% in all four age groups compared to 142 countries when the point estimates are used. In the most recent period, based on the point estimates, more than 50 countries showed a negative annual rate of reduction in the mortality risk for the people in the age group 20-24 years, but based on the upper bound of the 90% UIs, only 10 countries showed increases.

To fill persistent data gaps, sustained and increased investment in timely data collection, along with the expansion and strengthening of data systems, is essential in regions and countries where uncertainty in estimates remains high. Between 2000 and 2024, more than 50 countries relied exclusively on household survey and census data to estimate mortality in people aged 5-24 years. Sufficient source data to model trend estimates directly were not available in 35 countries for people in the age group 5-14 years and 39 countries for age people in the age group 15-24 years. As of 2024, less than one third of older children, adolescents, and young adults aged 5-24 years lived in countries with high quality vital registration systems and less than 20% of the deaths of children, adolescents, and youths occurred in these countries. In the absence of well functioning vital registration systems, data from household surveys or censuses are crucial for assessing mortality levels specific to age and sex, as well as trends in many high burden countries. Without these sources, little information would be available to inform the estimates directly from country data. Given current funding constraints for data collection, the development and use of cost efficient tools to measure mortality, together with the strengthening of administrative systems for the registration of vital events, are critical for accurately monitoring current patterns and trends.

In comparison with other global estimates our estimates differ from those of the 2023 Global Burden of Disease study, because of differences in underlying data inputs, crisis adjustments, and modelling strategies. Globally, the number of deaths in children, adolescents, and youths from the Global Burden of Disease study (2.3 million), is included in the 90% UIs around our estimates. At the country level, the correlation between UN IGME estimates and the most recent Global Burden of Disease 2023 estimates for the years 1990 to 2023 is 0.82. Correlations are highest for the 5-9 years and 10-14 years age groups, with correlations between 1990 and 2023 of 0.84 for both groups, respectively, and weaken in the 15-19 years and 20-24 years age groups, which show correlations of 0.83 and 0.79, respectively. Our latest UN IGME estimates are based on more recent data inputs than the Global Burden of Disease 2023, which also contributes to differences in the estimates. Estimates became closer since Global Burden of Disease study also started using survey data for the age groups 5-14 years and 15-24 years in their latest iteration. 

Discrepancies are highest in country-years with crisis adjustments and in the regions of sub-Saharan Africa where less empirical data are available to inform the estimates. In terms of modelling, our approach differs from Global Burden of Disease study since our estimates are specific to the 5-14 years and 15-24 years age groups and do not use information regarding other age groups, except for the few countries where we infer death risks from mortalityrisks for children younger than 5 years old. This means that the difficulty of measuring adult mortality, particularly in countries affected by HIV or crisis, does not affect our estimates. The current Global Burden of Disease model incorporates covariates to smooth trends and fill in data gaps: a socio-demographic index, the mortality rate from HIV, the age standardised covid-19 mortality rate, a binary covariate indicating whether each location is an island, and an all risk factor summary covariate calculated as the total population attributable fraction for all risk factors combined for age standardised, all cause mortality, excluding deaths from HIV/AIDS and crisis deaths. These covariates are likely to introduce a certain degree of circularity into the estimation process, as several of them involve measures of mortality. Furthermore, in several countries, considerable uncertainty surrounding these covariates exists.

## Conclusion

Without accelerated progress, the absolute number of deaths will remain stagnant or rise by 2030. Our findings underscore the urgent need for increased investment to counteract rising mortality in high income regions in older adolescents and youths, and highlight the risk that similar reversals could emerge in other parts of the world without targeted action. In addition, sustained and expanded investment is essential in sub-Saharan Africa, where rapid population growth is placing increasing pressure on health systems and could exacerbate the overall mortality burden. Progress is needed particularly in people aged 5-14 years in West and Central Africa, for whom progress has been slow.

The past three decades have seen substantial progress in mortality for older children, adolescents, and young adults aged 5-24 years. As the gap in mortality between children under 5 years of age and people aged 5-24 years narrows, reducing mortality in older children demands a shift beyond the traditional child survival agenda of reducing mortality in children under 5 years of age, as seen in the sustainable development agenda and the millennium development goals. Tackling the remaining burden of 2.1 million annual deaths requires age specific strategies that address leading causes in adolescence and young adulthood—such as violence, accidents, and injuries—while sustaining progress against infectious diseases in high mortality regions. With most deaths concentrated in sub-Saharan Africa and South Asia, urgent investment in health systems and robust data infrastructure is critical. Strengthening civil registration and vital statistics systems is not just a technical step but a foundation for equitable, evidence based policies that ensure no child or young person is left behind.

**What is already known on this topic**

- Global health initiatives have historically prioritised mortality in children under 5 years of age, leading to substantial reductions in mortality for that age group, while mortality among older children, adolescents, and youth has received less attention in global monitoring
- Progress has been steady in reducing mortality risk for ages 5-24 years since 1990, but was slower than in children under 5 years of age
- Recent studies, including Global Burden of Disease Study 2023, have warned of a potential health crisis with rising mortality risk among adolescents in specific high income regions, but evidence for this across all regions has been limited

**What are the new findings?**

- This study provides the most comprehensive annual estimates of all cause mortality for 200 countries and areas from 1990 to 2024, by five year age group (5-9, 10-14, 15-19, and 20-24 years), sex, and region
- Male mortality is declining slower than female mortality, particularly in people aged 15-24 years, where the risk of death for men is nearly double that of women
- The burden of deaths is shifting heavily to sub-Saharan Africa, which, now accounts for nearly 50 per cent of global deaths in age group 5-24 years driven by population growth and slower mortality reductions
- Despite global estimates, high quality vital registration data remains scarce; precision is often lowest in the regions where the estimated mortality burden is highest

**What do the new findings imply?**

- A child survival approach that focuses mainly on infectious diseases is insufficient for older children, adolescents, and youth, and policies targeting causes of death less amenable to traditional health interventions, such as violence and injuries, are needed
- Without accelerated mortality reductions, the absolute number of deaths in this age group will remain stagnant or rise by 2030, owing to population growth in high mortality regions and slow mortality reductions
- The lack of high quality data in high mortality countries and uncertainties around funding for large scale demographic surveys limits the ability to monitor trends in mortality and emerging health crises effectively, necessitating urgent investment in civil registration and vital statistics systems to fill critical knowledge gaps

## Data Availability

The data underlying the findings in this paper are openly and publicly available and can be found here: https://childmortality.org/. If you encounter problems accessing the data, please contact the corresponding author.

## Web extra

Extra material supplied by authors

Web appendix: Supplementary appendix

## Notes

Contributors: DY, LH, and DS conceived the study and completed the data analysis. LH, DS, YL, GF, BM, TS, and PG contributed to the database construction and all authors reviewed and had access to the underlying data for this study and YL and GF verified the raw data. LA, BM, and FC led the development of the bayesian models; DS, LH, YL, and GF produced the estimates, and all authors reviewed the final results. LH, GF, DY, and DS drafted the original manuscript. All authors reviewed the manuscript and provided input and edits to the article and approved its final version for submission.

Transparency: The lead author (the manuscript’s guarantor) affirms that the manuscript is an honest, accurate, and transparent account of the study being reported; that no important aspects of the study have been omitted; and that any discrepancies from the study as planned (and, if relevant, registered) have been explained.

Dissemination to participants and related patient and public communities: Estimates and underlying data are publicly available on https://childmortality.org/. All estimates have been shared with national governments.

Provenance and peer review: Not commissioned; externally peer reviewed.

Maps: These maps do not reflect the positions of United Nations Interagency Group for Child Mortality Estimation agencies or those of the institutions to which the authors are affiliated on the legal status of any country or territory, or on the delimitation of any frontiers. Published maps are provided without any warranty of any kind, either express or implied. BMJ remains neutral with regard to jurisdictional claims in published maps.

This article is part of a collection proposed by Unicef, WHO, and Johns Hopkins University. The three organisations provided funding for the collection. Unicef funded the open access fees for this article. *The BMJ* peer reviewed, edited, and decided to publish this article. Elizabeth Loder, Navjoyt Ladher, and Paul Simpson were the lead editors for *The BMJ*.
