# Supplementary material for: Global, regional, and national levels and trends in older child, adolescent, and youth (5-24 years) all cause mortality from 1990 to 2024: modelling study
Source: BMJ. 2026 Jun 4;393:e088685. doi: 10.1136/bmj-2025-088685 (PMC13327470; doi:10.1136/bmj-2025-088685)
Supplement: Supplementary file 1 — Web appendix: Supplementary appendix [file youd088685.ww.pdf]

Supplemental appendix to Global, regional and national levels and trends in older child, adolescent and young adult (5–24 years) all-cause mortality from 1990 to 2024: a systematic analysis

Contents

Methods 3

Overview 3

Civil registration data 4

Survey data 6

Systematic and random measurement error 7

Estimation of older child, adolescent and young adult mortality rates 7

Estimation of mortality rates among children aged 5–14 years and youth aged 15–24 years by sex 8

Estimation of older child, adolescent and young adult mortality due to conflict and natural disasters 9

Estimation of uncertainty intervals 10

Extrapolation to common reference year 10

Calculating number of deaths 10

Regional Classifications 11

Supplementary tables 12

Table A.1: Global and regional probability of dying by sex, 1990–2024 12

Table A.2: Global and regional deaths by sex, 1990–2024 14

Table A.3: Global and regional annual rate of reduction 16

Table A.4: Country probability of dying, 1990–2024 17

Table A.5: Country deaths, 1990–2024 28

Table A.6: Country annual rate of reduction 38

Table A.7: Data series in mortality database for older children 47

Supplementary figures 82

Country-level probability of dying with underlying data 82

Methods

Overview

The UN IGME employs the following broad strategy to arrive at annual estimates of older child (age 5-9), adolescent (ages 10-14 and 15-19) and young adult (age 20-24) mortality:

1. Compile and assess the quality of all available nationally representative data relevant to the estimation of older child, adolescent and young adult mortality, including data from vital registration systems, population censuses, household surveys and sample registration systems;
2. Assess data quality, recalculate data inputs and make adjustments as needed by applying standard methods;
3. Fit a statistical model to these data to generate a smooth trend curve that averages possibly disparate estimates from the different data sources for a country;
4. Extrapolate the model to a target year (in this case, 2024).

To increase the transparency of the estimation process, the UN IGME has developed a child mortality web portal, Child Mortality Estimation (CME) Info, available at <childmortality.org>. It includes all available data and shows estimates for each country as well as which data are currently officially used by the UN IGME. Once new estimates are finalized, CME Info is updated accordingly.

The UN IGME applies a common methodology across countries and uses empirical data from each country to produce comparable estimates, i.e., country values for the same reference year produced using a common method. Applying a consistent methodology allows for comparisons between countries, despite the varied number and types of data sources. UN IGME estimates are based on nationally available data from censuses, surveys or vital registration systems. The UN IGME does not use covariates to derive its estimates, but, rather, applies a curve-fitting method to empirical data after data quality assessment.

Countries may use a single data source for their official estimates or apply valid methods different from those used by the UN IGME. The UN IGME does not report figures produced by individual countries using other methods, as these estimates would not be comparable across countries. The differences between UN IGME and national official estimates are usually not large if the empirical data are of good quality. The UN IGME aims to minimize errors for each estimate, harmonize trends over time, and produce up-to-date and comparable estimates of older child, adolescent and young adult mortality. Because errors are inevitable in data, there will always be uncertainty around data and estimates. To allow for added comparability, the UN IGME generates all older child, adolescent and young adult mortality estimates with uncertainty bounds.

Starting in 2017, UN IGME began publishing mortality estimates for older children and young adolescents aged 5–14. In 2020, mortality estimates were expanded further to include older adolescents and young adults aged 15–24, and since 2024, they are differentiated by sex.

Data sources

The first step in the process of arriving at estimates of levels and recent trends of older child, adolescent and young adult mortality is to compile all newly available data and add the data to the UN IGME database (newly available may include newer, recently released data and occasionally, results from older censuses or surveys not previously available). Nationally representative estimates of older child, adolescent and young adult mortality can be derived from several different sources, including civil registration and sample surveys. Demographic surveillance sites and hospital data are excluded as they are not nationally representative. The preferred source of data is a civil registration system that records births and deaths on a continuous basis. If registration is complete and this system functions efficiently, the resulting estimates will be accurate and timely. However, many low- and middle-income countries do not have well-functioning vital registration systems. Therefore, household surveys such as the UNICEF-supported Multiple Indicator Cluster Surveys, the Demographic and Health Surveys, and periodic population censuses have become the primary sources of data on mortality among older children, adolescents and young adults aged 5–24 years. These surveys ask women about the survival of their children and about the survival of their siblings, and it is these reports, or microdata upon availability, that provide the basis for childhood, adolescent and youth mortality estimates for a majority of low- and middle-income countries.

Databases were constructed for the probabilities of dying for age groups 5–14 years (${}_{10}q_5$), 5–9 years (${}_5q_5$), 15–24 years (${}_{10}q_{15}$) and 15–19 years (${}_{15}q_5$) for total mortality and by sex. The probabilities of dying for the age groups 10–14 and 20–24 are derived from the estimated broader age groups using the following formula:

${}_5q_{10} = 1{,}000 \times (1 - (1{,}000 - {}_{10}q_5) / (1{,}000 - {}_5q_5))$,

${}_5q_{20} = 1{,}000 \times (1 - (1{,}000 - {}_{10}q_{15}) / (1{,}000 - {}_5q_{15}))$,

when the estimated ${}_nq_x$, are expressed per 1,000 survivors to age *x* years.

Whatever the method used to derive the estimates, data quality is critical. The UN IGME assesses data quality and does not include data sources with substantial non-sampling errors or omissions as underlying empirical data in its statistical model.

A detailed list of the data sources used is available in table XX in the annex. Empirical data sources and annual estimates are available publicly on the UN IGME web portal~~The full set of empirical data used in this analysis is publicly available from the UN IGME web portal~~, CME Info <www.childmortality.org>.

Civil registration data

Data from civil registration systems are the preferred data source for older child, adolescent and young adult mortality estimation. The calculation of probabilities of dying among older children and adolescents

aged 5–14 years (${}_{10}q_5$, the probability a five-year-old would die before reaching age 15, expressed per 1,000 children aged 5 years) and probabilities of dying among adolescents and young adults aged 15–24 years (${}_{10}q_{15}$, the probability a 15-year-old would die before reaching age 25, expressed per 1,000 youths aged 15 years) are derived from a standard period abridged life table.. The inputs for ${}_{10}q_5$ are the number of deaths for age group 5-9 years (noted D_{5-9}) and for the age group 10-14 years (D_{10-14}), as well as the mid-year population for the same age groups (P_{5-9} and P_{10-14}).

- The death rate for age group 5-9, ${}_5M_5$ is obtained by dividing D_{5-9} by P_{5-9}.
- The probability ${}_5q_5$, which is the probability a five-year-old would die before reaching age 10, is obtained as ${}_5q_5$ = (5 * ${}_5M_5$)/[1+(5-${}_5a_5$) * ${}_5M_5$], where ${}_5a_5$ is the average number of years lived by children who died in the age group 5-9 (set at 2.5 for all countries)
- The same calculation is applied for ${}_5q_{10}$, the probability a 10-year-old would die before reaching age 15.
- Finally, ${}_{10}q_5$ = 1-(1-${}_5q_5$)(1-${}_5q_{10}$)

The calculation of the probability ${}_{10}q_{15}$ is also derived from the number of deaths for the age groups 15-19 years (noted D_{15-19}) and 20-24 years (D_{20-24}), as well as the mid-year population for the same age groups (P_{15-19} and P_{20-24}), using the approach detailed above.

To select country-years for which vital registration data are included for older children aged 5–14 and youth aged 15–24, and compute adjustment factors in case of incomplete registration, we used a hybrid of the generalized growth balance method (GGB) and the synthetic extinct generation method (SEG), the GGBSEG method, which is one several demographic methods known as "death distribution methods"[1] and has been shown to perform better than the GGB and SEG methods applied separately. The GGBSEG method is implemented in the DDM package of the R statistical software[2]. Completeness was estimated for each sex separately, for periods between pairs of recent censuses for which an age distribution of the population was available in the Demographic Yearbook[3]. The sex-specific completeness estimates were combined to obtain an estimate for both sexes. When the estimated completeness was less than 80%, mortality rates derived from vital registration data were excluded from the model fit. When completeness was greater than or equal to 95%, the registration was considered virtually complete, and no adjustment was used to adjust mortality estimates upwards. If completeness was between 80 and 95%, we multiplied the number of deaths by the inverse of the completeness rate to obtain adjusted estimates. These adjustments are only applied to mortality data above age 5 as the death distribution methods cannot be applied to estimate completeness of registration of under-five deaths. No adjustment was applied for countries included in the Human Mortality Database[4], as their death registration data were considered complete. A more detailed description of the estimation of death registration completeness is provided elsewhere[5].

For civil registration data (with available data on the number of deaths and mid-year populations), annual observations were initially constructed for all observation years in a country. For country-years in which

the coefficient of variation exceeded 20% for those aged 5–14 or 15-24 years, deaths and midyear populations were pooled over longer periods. Starting from the most recent years, deaths and population were combined with adjacent previous years to reduce spurious fluctuations in countries where small numbers of births and deaths were observed. The stochastic standard errors of the mortality rates were calculated using a Poisson approximation, based on the number of children turning 5 or 15 in each year, estimated from the 2024 World Population Prospects.

Vital registration (VR) data were obtained from the WHO Mortality Database, the United Nations Department of Economic and Social Affairs, Population Division Data Portal, Human Mortality Database and country-specific sources.

Survey data

Survey data on mortality among those aged 5-14 years comes in the form of the full birth history (FBH),

Mortality estimates of children aged 5–14 years can be derived from the full birth history module (FBH), whereby women are asked for the date of birth of each of their children, whether the child is still alive, and if not, the child's age at death. The probability of dying among children in this age group (${}_{10}q_{5}$) is estimated for the period 0–12 years before the survey and divided into intervals of various length (6, 4, 3, 2, 1 years) depending on the coefficient of the variation of the estimates (< 20%).[6]

Mortality estimates of adolescents and young adults aged 15-24 years were derived from the sibling survival histories (SSH). In SSH, women aged 15-49 years are asked to list all their siblings born to the same mother by birth order, and to report on each sibling's gender, survival status, current age, if alive, or age at death and years since death, if deceased. Sibling histories have been extensively used to model adult mortality in countries lacking vital registration and to monitor trends in maternal mortality.[7, 8, 9]

As with birth histories, sibling histories can be affected by under-reporting of deaths—living and deceased siblings can be omitted and ages at survey or at death can be misreported. The time of death can also be affected by heaping or systematic misstatement. However, the magnitudes of these biases are likely to vary by age, and few studies have explored this issue for the 15-24 age group in particular. To account for the possibility of non-sampling bias in sibling histories, we used the Bayesian penalized B-splines bias-reduction model (or B3 model) developed by Alkema and New[6], as detailed below. This model includes a data model that estimates the bias in SSH, both in terms of the level and the trend, as a function of the retrospective period. This bias is estimated by contrasting the sibling-based estimates with vital registration data for overlapping time periods. Before adding the SSH estimates in the database, we also examined the age pattern of mortality contained in each DHS, contrasting the mortality rate of 15–24-year-olds (${}_{10}q_{15}$) from the SSH with the under-five mortality rate (${}_{5}q_{0}$) obtained from the FBH. We compared the ${}_{10}q_{15}$-to-${}_{5}q_{0}$ relationship observed in the DHS with an expected pattern from life tables computed in complete vital registration data or other high-quality sources, such as Health and Demographic Surveillance Sites. In about 25 surveys, the ${}_{10}q_{15}$ probability obtained from sibling histories

was inconsistent with the level of under-five mortality, and these surveys were excluded from the model fitting.

Systematic and random measurement error

Data from these different sources require varied calculation methods and may suffer from different errors, such as random errors in sample surveys or systematic errors due to misreporting. Thus, different surveys often yield widely divergent estimates of mortality for a given period. To reconcile these differences and take better account of the systematic biases associated with the various types of data inputs, an estimation method was developed to fit a smoothed trend curve to a set of observations and to extrapolate that trend to a defined time point, in this case, 2024. This method is described in the following section.

Estimation of older child, adolescent and young adult mortality rates

Estimation of older child, adolescent and young adult mortality rates was undertaken using the Bayesian B-splines bias-adjusted model, referred to as the B3 model.[6] This model was developed, validated and used to produce previous rounds of UN IGME child mortality estimates.[10, 11]

In the B3 model, log($_{10}q_{5}$) or log($_{10}q_{15}$) is estimated with a flexible splines regression model. The spline regression model is fitted to all observations in the country. An observed value for $_{10}q_{5}$ or $_{10}q_{15}$ is considered to be the true value multiplied by an error multiplier, e.g., observed $_{10}q_{5}$ = true $_{10}q_{5}$ * error multiplier, or on the log scale, log(observed $_{10}q_{5}$) = log(true $_{10}q_{5}$) + log(error multiplier). The error multiplier refers to the relative difference between an observation and the truth with error multiplier equal to 1 (and log(error multiplier) equal to zero) meaning no error. The B3 model is also used to estimate 10q15, 5q5, and 5q15.

While estimating the true mortality rate, properties of the errors that provide information about the quality of the observation or in other words, the extent of error that we expect, are taken into account. These properties include: the standard error of the observation; its source type (e.g., Demographic and Health Surveys versus census); and whether the observation is part of a data series from a specific survey (and how far the data series is from other series with overlapping observation periods). These properties are summarized in the “data model.” When estimating the probability of dying, the data model adjusts for the errors in the observations, including the average systematic biases associated with different types of data sources, using information on data quality for different source types from all countries. Mean biases within surveys are modelled as a linear function of the retrospective period of observation. Biases are estimated on the basis of consistency between each observation and other observations relating to the same periods, using a hierarchical model. The model takes into account differences in sampling and nonsampling variance between observations.

For all non-vital registration data series, non-sampling biases specific to data series are estimated with the B3 model. Full birth histories from surveys tend to slightly underestimate mortality in the age group 5–14

when compared to other data series. Sibling histories used to model the probability $_{10}q_{15}$ also tend to underestimate mortality in the age group 15–24, especially for reference periods that are located further in the past from the survey date. This is likely due to omissions of some deaths or systematic age misstatements. As a result, in countries where the trend in mortality is largely informed by survey data, the final estimates are adjusted upwards and therefore, the final estimated series may fall slightly above the original survey data points.

There were not enough data inputs from vital registration, surveys or censuses to estimate the probability $_{10}q_5$ in 35 countries and $_{10}q_{15}$ in 40 countries. For these cases, the probability, $_{10}q_5$ or $_{10}q_{15}$ was modelled on the draft estimates of the under-five mortality rate (U5MR) and an expected relationship between mortality in the 0–4 and 5–14 or 15–24 age groups, as observed in countries with sufficient data series. A hierarchical linear regression was used to regress log($_{10}q_5$) or log($_{10}q_{15}$) against log(U5MR) and the coefficients of this regression were used to predict the probability $_{10}q_5$ and $_{10}q_{15}$ between 1990 and 2024 for countries with insufficient data sources. The advantage of this approach is that no model life tables are used (such life tables are based on the historical experience of countries with high-quality vital registration data and do not always adequately reflect mortality age patterns in low- and middle-income countries). A more complete technical description of the application of the B3 model to older children, adolescents and young adults is available elsewhere.[12]

~~Compared with the previously applied LOESS (locally estimated scatterplot smoothing) estimation approach,[13] the B3 model better accounts for data errors, including biases and sampling and non-sampling errors in the data. It can more accurately capture short-term fluctuations in a given mortality rate and its annual rate of reduction and, thus, is better able to account for evidence of acceleration in the decline of mortality from new surveys. Validation exercises show that the B3 model also performs better in short-term projections.~~The B3 method was developed and implemented for the UN IGME by Leontine Alkema and Jin Rou New with guidance and review by the UN IGME's Technical Advisory Group. A more complete technical description of the B3 model is available elsewhere.[6, 12] In general, the B3 model described above is used for all countries (except the Democratic People's Republic of Korea where a non-standard method was employed).

Estimation of mortality rates among children aged 5–14 years and youth aged 15–24 years by sex

Since 2022, the UN IGME has produced estimates of mortality in children aged 5–14 and youth aged 15–24 by sex. The estimation model builds upon the main model structure of the sex ratio for IMR, CMR and U5MR[14] but with reconsideration of model choices. In particular, the expected sex ratio (denoted as *W(c,t)*), is modelled with a second-order random walk (RW2) model instead of a B-splines model. The within-country fluctuation time series *P(c,t)* is modelled with a first-order random walk (RW1) model rather than an AR(1) model. Furthermore, the statistical computing is carried out using Integrated Nested Laplace Approximations (INLA) instead of Markov chain Monte Carlo (MCMC). A more complete technical description of this model is available elsewhere.[15]

Estimation of older child, adolescent and young adult mortality due to conflict and natural disasters

Estimated deaths from major crises were derived from various data sources from 1950 to the present. Data on natural disasters were obtained from the Centre for Research on the Epidemiology of Disasters' International Disaster Database.[16] Conflict death data were taken from the Uppsala Conflict Data Program/Peace Research Institute Oslo datasets,[17, 18] Armed Conflict Location & Event Data Project[19] Center for Systemic Peace/Integrated Network for Societal Conflict Research dataset,[20] as well as from reports prepared by the UN and other organizations.

For crises where deaths were adequately recorded in death registration data, age-specific deaths were obtained directly from the data. For many countries, age-sex specific data on crisis deaths is not available and the UN IGME undertook a comprehensive analysis of more than 1,000 articles and books on crisis mortality compiled over the years by the UNPD and WHO to identify studies and datasets with age-patterns for crisis deaths. Additionally, death registration data in the WHO Mortality Database, UNDESA Population Division Data Portal and the Human Mortality Database, DHS, MICS and World Fertility Surveys for the period 1960 to 2017 were analysed for regions and years determined to have experienced crisis events. From all these sources, information on age-sex distributions was obtained for 174 events: 51 conflicts, 32 earthquakes, 35 famines, 30 epidemics, 10 floods, 9 tsunamis, 4 genocides and 3 cyclones. These data were analysed to prepare age-sex distributions by five-year age groups and for more detailed age groups under 5 for each of the event types as described elsewhere.[21]

Estimated deaths due to major crises were included if they met the following criteria: ~~(1) the crisis was isolated to a few years;~~ (~~2~~1) crisis deaths among those aged 5–14 years or aged 15–24 years were greater than 10% of non-crisis deaths in the age group; (~~3~~2) crisis $_{10}q_5$ or crisis $_{10}q_{15}$ was > 0.2 deaths per 1,000; (~~4~~3) the number of crisis deaths among those 5–14 or 15–24 years old was > 10 deaths.

Crisis deaths were included in the estimates by first excluding data points from crisis years if the crisis was isolated over a few years due to natural disasters, then fitting the B3 model to the remaining data and adding the crisis-specific mortality rate to the fitted B3 curve. Crisis death estimates are uncertain but, presently, no uncertainty around crisis deaths is included in the uncertainty intervals of the estimates. Instead, we assume the relative uncertainty in the adjusted estimates is equal to the relative uncertainty in the non-adjusted estimates; this assumption will be revisited in the future.

The UN IGME has assessed recent humanitarian crises and, based on the scarcity of currently available data and the difficulties of estimating the broader impact of these crises on health systems, decided to hold the estimates constant from the start of the crisis while increasing the uncertainty over the crisis time for two countries: South Sudan and Venezuela (Bolivarian Republic of). Where applicable, direct crisis deaths have been added to the constant trend estimate. The UN IGME will review new data, if available, in the next estimation round and revise estimates accordingly.

The 2025 UN IGME estimates do not include any adjustment in the years 2020 to 2024 for COVID-19-related mortality as the evidence is insufficient to support an adjustment at this time. First, direct COVID-19 deaths in the age groups estimated are rare, and thus unlikely to impact national-level estimates. Second, a UN IGME analysis of excess mortality using empirical data on deaths from civil registration and vital statistics systems and health management information systems (HMIS) found no evidence of systematic excess mortality among children, adolescents or youth from 2020 to 2024. It should be noted that geographic and income variation in the data on excess deaths analysed by the UN IGME thus far is limited, and the pandemic continues to evolve in unpredictable ways. Thus, the UN IGME will continue to collect data for assessing excess deaths, revisiting this issue and generating adjustments where applicable and as needed based on evidence as it becomes available.

Estimation of uncertainty intervals

Given the inherent uncertainty in older child, adolescent and young adult mortality estimates, 90% uncertainty intervals are used by the UN IGME instead of the more conventional 95% intervals. Reporting intervals based on higher levels of uncertainty (i.e., 95% instead of 90%) has the advantage that the chance of not having included the true value in the interval is smaller. The disadvantage of choosing higher uncertainty levels, however, is that intervals lose their utility to present meaningful summaries of a range of likely outcomes if the indicator of interest is highly uncertain. Given this trade-off and the substantial uncertainty associated with older child, adolescent and young adult mortality estimates, the UN IGME chose to report 90% uncertainty intervals or in other words, intervals for which there is a 90% chance that they contain the true value, to encourage wider use and interpretation of uncertainty intervals.

Extrapolation to common reference year

If the underlying empirical data refer to an earlier reference period than the end year of the period the estimates are reported, the UN IGME extrapolates the estimates to the common end year; in this round, to 2024. The UN IGME does not use covariates to derive the estimates but uses the past trend in a country and the global trend to extrapolate to the target year.

Calculating number of deaths

The absolute number of deaths among those aged 5–14 years in a given year and country is calculated using the central death rates of age groups 5–9 and 10–14 years, ${}_5M_5$ and ${}_5M_{10}$, computed from the estimated ${}_5q_5$ and ${}_5q_{10}$. The central death rates are then multiplied by the country population estimates for the respective age groups from the *World Population Prospects 2024*[22] to calculate the number of deaths. A similar approach is used for calculating the number of deaths in the age group 15–24: the estimated ${}_5q_{15}$ and ${}_5q_{20}$ are converted in central death rates ${}_5M_{15}$ and ${}_5M_{20}$ and multiplied by the population estimates.

Assessing the annual rate of reduction in the mortality risk

The pace of change in probabilities of dying is measured using the annual rate of reduction (ARR), defined as the annual percentage decrease in the probability of dying over a specified period. ARR is calculated as $ARR = \log(q_{t2} / q_{t1}) / (t_1 - t_2)$, where q refers to probability of dying and $t_1 < t_2$. Uncertainty in the ARR is derived from the distribution of ARRs calculated across the posterior trajectories for each country and indicator.

Regional Classifications

Estimates at the regional level in the paper were based on the UNICEF regional classifications.

Map A1: UNICEF regional classifications.

a

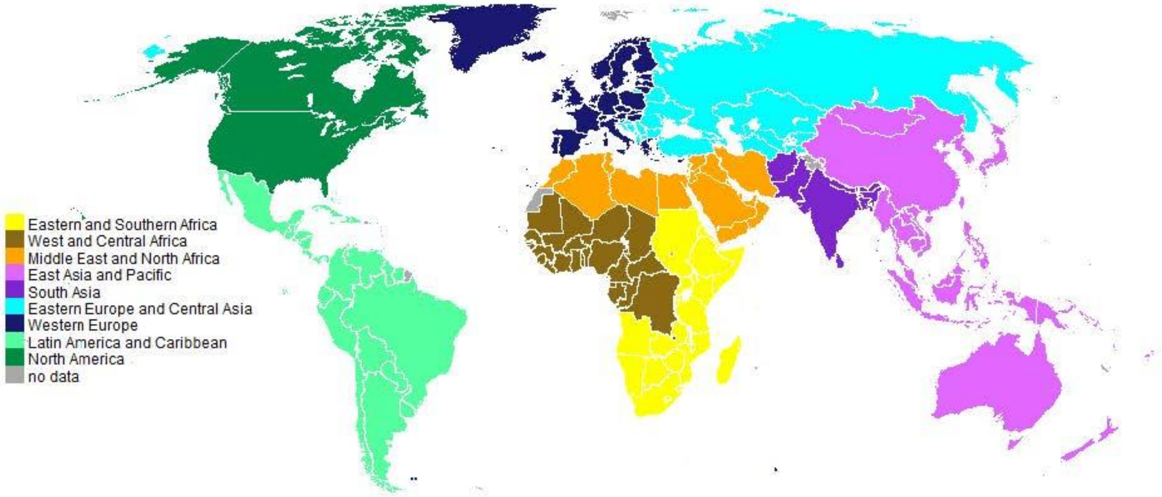

Note: This map does not reflect a position by UN IGME agencies or those of the institutions to which the authors are affiliated on the legal status of any country or territory or on the delimitation of any frontiers.

East Asia and Pacific

Australia; Brunei Darussalam; Cambodia; China; Cook Islands; Democratic People's Republic of Korea; Fiji; Indonesia; Japan; Kiribati; Lao People's Democratic Republic; Malaysia; Marshall Islands; Micronesia (Federated States of); Mongolia; Myanmar; Nauru; New Zealand; Niue; Palau; Papua New Guinea; Philippines; Republic of Korea; Samoa; Singapore; Solomon Islands; Thailand; Timor-Leste; Tonga; Tuvalu; Vanuatu; Viet Nam

Eastern and Southern Africa

Angola; Botswana; Burundi; Comoros; Djibouti; Eritrea; Eswatini; Ethiopia; Kenya; Lesotho; Madagascar; Malawi; Mauritius; Mozambique; Namibia; Rwanda; Seychelles; Somalia; South Africa; South Sudan; Sudan; Uganda; United Republic of Tanzania; Zambia; Zimbabwe

Eastern Europe and Central Asia

Albania; Armenia; Azerbaijan; Belarus; Bosnia and Herzegovina; Bulgaria; Croatia; Georgia; Kazakhstan; Kosovo (UNSCR 1244); Kyrgyzstan; Montenegro; North Macedonia; Republic of Moldova; Romania; Russian Federation; Serbia; Tajikistan; Türkiye; Turkmenistan; Ukraine; Uzbekistan

Latin America and Caribbean

Anguilla; Antigua and Barbuda; Argentina; Bahamas; Barbados; Belize; Bolivia (Plurinational State of); Brazil; British Virgin Islands; Chile; Colombia; Costa Rica; Cuba; Dominica; Dominican Republic; Ecuador; El Salvador; Grenada; Guatemala; Guyana; Haiti; Honduras; Jamaica; Mexico; Montserrat; Nicaragua; Panama; Paraguay; Peru; Saint Kitts and Nevis; Saint Lucia; Saint Vincent and the Grenadines; Suriname; Trinidad and Tobago; Turks and Caicos Islands; Uruguay; Venezuela (Bolivarian Republic of)

Middle East and North Africa

Algeria; Bahrain; Egypt; Iran (Islamic Republic of); Iraq; Israel; Jordan; Kuwait; Lebanon; Libya; Morocco; Oman; Qatar; Saudi Arabia; State of Palestine; Syrian Arab Republic; Tunisia; United Arab Emirates; Yemen

North America

Canada; United States

South Asia

Afghanistan; Bangladesh; Bhutan; India; Maldives; Nepal; Pakistan; Sri Lanka

West and Central Africa

Benin; Burkina Faso; Cabo Verde; Cameroon; Central African Republic; Chad; Congo; Côte d'Ivoire; Democratic Republic of the Congo; Equatorial Guinea; Gabon; Gambia; Ghana; Guinea; Guinea-Bissau; Liberia; Mali; Mauritania; Niger; Nigeria; Sao Tome and Principe; Senegal; Sierra Leone; Togo

Western Europe

Andorra; Austria; Belgium; Cyprus; Czechia; Denmark; Estonia; Finland; France; Germany; Greece; Hungary; Iceland; Ireland; Italy; Latvia; Liechtenstein; Lithuania; Luxembourg; Malta; Monaco; Netherlands (Kingdom of the); Norway; Poland; Portugal; San Marino; Slovakia; Slovenia; Spain; Sweden; Switzerland; United Kingdom

Supplementary tables

Table A.1: Global and regional probability of dying by sex, 1990–2024

Probability of dying $_5q_5$: the probability of dying between exact age 5 and exact age 10, expressed per 1,000 children aged 5. Probability of dying $_5q_{10}$: the probability of dying between exact age 10 and exact age 15, expressed per 1,000 children aged 10. Probability of dying $_5q_{15}$: the probability of dying between exact age 15 and exact age 20, expressed per 1,000 youths aged 15. Probability of dying $_5q_{20}$: the probability of dying between exact age 20 and exact age 25, expressed per 1,000 youths aged 20. Values shown for 1990, 2000, 2015, and 2024, by UNICEF reporting region and World. Medians with 90% uncertainty intervals in parentheses.

		Probability of dying $_5q_5$ (per 1,000 children aged 5)				Probability of dying $_5q_{10}$ (per 1,000 children aged 10)				Probability of dying $_5q_{15}$ (per 1,000 youths aged 15)				Probability of dying $_5q_{20}$ (per 1,000 youths aged 20)			
		1990	2000	2015	2024	1990	2000	2015	2024	1990	2000	2015	2024	1990	2000	2015	2024
West and Central Africa	Total	24.7 (23.2–26.4)	20.9 (20.0–22.2)	13.1 (12.4–13.9)	10.9 (9.9–11.9)	12.1 (9.3–16.4)	11.4 (9.3–13.6)	8.6 (7.1–10.1)	7.7 (5.7–11.0)	17.1 (15.0–19.9)	14.0 (12.9–15.2)	10.2 (9.5–11.2)	8.8 (7.8–10.3)	21.0 (15.9–30.1)	17.6 (15.3–20.3)	13.0 (11.3–15.3)	11.6 (9.4–16.9)

		Probability of dying $_5q_5$ (per 1,000 children aged 5)				Probability of dying $_5q_{10}$ (per 1,000 children aged 10)				Probability of dying $_5q_{15}$ (per 1,000 youths aged 15)				Probability of dying $_5q_{20}$ (per 1,000 youths aged 20)			
		1990	2000	2015	2024	1990	2000	2015	2024	1990	2000	2015	2024	1990	2000	2015	2024
	Female	24.0 (22.4–25.8)	20.3 (19.2–21.6)	12.5 (11.8–13.4)	10.2 (9.2–11.3)	12.1 (9.3–16.4)	11.2 (9.2–13.4)	8.2 (6.8–9.7)	7.3 (5.4–10.5)	17.8 (15.4–20.8)	14.5 (13.3–16.0)	9.6 (8.8–10.7)	7.8 (6.7–9.4)	19.9 (14.7–29.5)	18.6 (16.0–21.6)	12.6 (10.7–14.9)	10.4 (8.3–15.7)
	Male	25.5 (23.9–27.4)	21.6 (20.5–23.0)	13.6 (12.8–14.5)	11.5 (10.4–12.7)	12.1 (9.3–16.4)	11.5 (9.4–13.9)	8.9 (7.4–10.6)	8.1 (6.0–11.6)	16.5 (14.3–19.4)	13.4 (12.3–14.8)	10.7 (9.9–11.9)	9.7 (8.5–11.6)	22.0 (16.6–31.5)	16.7 (14.3–19.5)	13.5 (11.6–15.9)	12.7 (10.3–18.6)
Eastern and Southern Africa	Total	25.8 (24.0–27.8)	17.7 (16.9–18.7)	8.5 (8.0–9.1)	6.2 (5.6–6.7)	13.2 (10.0–17.1)	9.4 (8.0–11.1)	5.5 (4.6–6.7)	4.2 (3.3–6.6)	19.2 (18.1–21.0)	16.0 (15.2–16.9)	9.5 (8.8–10.4)	7.8 (7.0–8.8)	28.0 (25.0–31.8)	24.0 (22.4–26.0)	13.2 (11.6–15.2)	11.4 (9.6–16.4)
	Female	24.6 (22.8–26.6)	17.2 (16.3–18.2)	8.1 (7.6–8.7)	5.8 (5.2–6.3)	13.0 (9.8–17.0)	9.2 (7.8–10.8)	5.1 (4.3–6.2)	3.7 (2.9–5.8)	18.5 (17.2–20.4)	16.4 (15.5–17.5)	8.1 (7.4–9.0)	5.8 (5.2–6.7)	24.3 (21.6–27.8)	25.6 (23.7–27.9)	10.9 (9.6–12.6)	7.9 (6.5–11.6)
	Male	27.0 (25.0–29.2)	18.2 (17.4–19.3)	8.9 (8.4–9.6)	6.6 (6.0–7.2)	13.4 (10.2–17.4)	9.6 (8.2–11.4)	5.9 (4.9–7.3)	4.7 (3.6–7.4)	19.9 (18.5–22.0)	15.5 (14.7–16.6)	10.9 (9.9–12.1)	9.7 (8.6–11.2)	31.8 (27.9–36.6)	22.3 (20.6–24.4)	15.5 (13.3–18.2)	14.8 (12.2–21.7)
Latin America and Caribbean	Total	3.0 (2.9–3.0)	2.1 (2.0–2.1)	1.4 (1.4–1.5)	1.3 (1.2–1.4)	2.7 (2.6–2.9)	2.1 (2.0–2.2)	1.7 (1.6–1.8)	1.7 (1.5–1.9)	5.9 (5.7–6.1)	5.5 (5.5–5.6)	5.4 (5.3–5.5)	4.4 (4.2–4.6)	8.6 (8.2–9.0)	8.1 (7.9–8.3)	7.5 (7.3–7.9)	7.2 (6.7–8.6)
	Female	2.6 (2.6–2.7)	1.8 (1.8–1.9)	1.3 (1.2–1.3)	1.1 (1.1–1.2)	2.2 (2.1–2.4)	1.7 (1.6–1.8)	1.4 (1.3–1.5)	1.4 (1.3–1.6)	3.8 (3.7–4.1)	3.2 (3.1–3.3)	2.7 (2.5–2.8)	2.3 (2.2–2.5)	4.7 (4.3–5.1)	3.8 (3.6–4.0)	3.2 (3.0–3.5)	3.2 (3.0–3.9)
	Male	3.3 (3.2–3.4)	2.3 (2.3–2.4)	1.6 (1.5–1.6)	1.4 (1.4–1.5)	3.2 (3.0–3.4)	2.5 (2.4–2.6)	2.0 (1.9–2.1)	1.9 (1.8–2.2)	7.9 (7.7–8.2)	7.9 (7.7–8.0)	8.0 (7.8–8.2)	6.4 (6.1–6.7)	12.5 (11.9–13.0)	12.5 (12.1–12.8)	11.8 (11.4–12.3)	11.2 (10.3–13.2)
South Asia	Total	13.4 (12.9–13.8)	8.4 (8.2–8.7)	3.7 (3.5–3.8)	2.2 (2.1–2.4)	7.1 (6.0–8.2)	5.4 (4.8–6.0)	3.2 (2.9–3.6)	2.3 (1.8–3.1)	10.4 (10.0–10.9)	8.1 (7.9–8.5)	5.0 (4.8–5.3)	3.8 (3.4–4.4)	13.1 (11.8–15.0)	11.0 (10.1–12.1)	6.7 (6.0–7.5)	5.3 (3.9–7.6)
	Female	13.9 (13.2–14.6)	8.7 (8.4–9.2)	3.5 (3.3–3.7)	2.0 (1.8–2.2)	7.1 (6.0–8.3)	5.4 (4.8–6.0)	3.0 (2.6–3.4)	2.0 (1.6–2.7)	11.4 (10.7–12.2)	8.7 (8.1–9.2)	4.7 (4.4–5.1)	3.4 (2.9–4.0)	13.6 (12.1–15.6)	11.4 (10.3–12.7)	5.6 (5.0–6.4)	4.1 (2.9–6.3)
	Male	12.9 (12.3–13.5)	8.1 (7.7–8.5)	3.9 (3.7–4.1)	2.4 (2.2–2.7)	7.0 (6.0–8.2)	5.4 (4.8–6.1)	3.5 (3.0–3.9)	2.6 (2.0–3.4)	9.4 (8.8–10.1)	7.7 (7.2–8.2)	5.3 (4.9–5.7)	4.2 (3.6–4.9)	12.6 (11.3–14.7)	10.7 (9.6–11.8)	7.7 (6.8–8.7)	6.4 (4.6–9.3)
Middle East and North Africa	Total	6.7 (6.5–7.0)	3.2 (3.1–3.4)	2.7 (2.5–2.8)	2.2 (2.0–2.5)	5.1 (4.1–6.3)	2.7 (2.4–3.0)	2.3 (2.0–2.9)	2.3 (1.8–3.5)	7.4 (6.7–8.2)	4.2 (3.9–4.4)	4.6 (4.3–4.9)	3.9 (3.7–4.3)	9.4 (7.7–12.4)	5.2 (4.6–5.9)	6.0 (5.3–7.1)	4.8 (4.0–6.6)
	Female	6.3 (6.0–6.7)	2.8 (2.7–3.0)	2.4 (2.2–2.5)	2.0 (1.8–2.3)	4.7 (3.7–5.9)	2.2 (2.0–2.5)	1.9 (1.6–2.4)	1.9 (1.4–3.0)	5.1 (4.6–5.9)	2.8 (2.6–3.1)	2.4 (2.2–2.7)	2.4 (2.1–2.7)	5.4 (4.2–7.5)	3.3 (2.9–3.8)	2.6 (2.3–3.0)	2.6 (2.1–3.7)
	Male	7.1 (6.8–7.4)	3.6 (3.5–3.8)	2.9 (2.8–3.1)	2.4 (2.2–2.8)	5.4 (4.5–6.8)	3.1 (2.8–3.5)	2.8 (2.4–3.4)	2.6 (2.0–3.9)	9.4 (8.6–10.5)	5.4 (5.1–5.9)	6.5 (6.1–7.1)	5.4 (5.0–6.0)	13.0 (10.7–17.1)	6.9 (6.1–8.0)	8.9 (7.8–10.8)	6.8 (5.7–9.5)
Eastern Europe and Central Asia	Total	3.4 (3.3–3.4)	2.7 (2.7–2.8)	1.2 (1.2–1.3)	1.1 (1.1–1.1)	2.4 (2.0–2.7)	2.2 (2.0–2.4)	1.3 (1.3–1.3)	1.3 (1.3–1.4)	5.6 (4.6–6.4)	5.3 (5.0–5.6)	2.9 (2.8–2.9)	3.3 (3.2–3.4)	6.9 (5.9–8.5)	8.8 (8.2–9.6)	4.4 (4.3–4.5)	6.8 (6.2–7.5)
	Female	2.7 (2.6–2.8)	2.3 (2.2–2.4)	1.1 (1.0–1.1)	1.0 (0.9–1.0)	1.7 (1.5–2.0)	1.7 (1.5–1.9)	1.1 (1.0–1.1)	1.1 (1.0–1.1)	3.8 (2.9–4.6)	3.2 (3.0–3.4)	1.9 (1.8–1.9)	1.9 (1.8–2.0)	3.4 (2.9–4.3)	4.0 (3.7–4.5)	2.3 (2.3–2.4)	2.5 (2.3–2.9)
	Male	4.0 (3.9–4.1)	3.1 (3.1–3.2)	1.4 (1.4–1.4)	1.3 (1.3–1.3)	3.0 (2.6–3.4)	2.8 (2.5–3.0)	1.6 (1.6–1.6)	1.5 (1.5–1.6)	7.3 (6.1–8.2)	7.4 (7.0–7.8)	3.8 (3.8–3.9)	4.7 (4.5–5.0)	10.2 (8.7–12.5)	13.6 (12.7–14.6)	6.3 (6.2–6.5)	11.0 (9.8–12.5)

		Probability of dying $_5q_5$ (per 1,000 children aged 5)				Probability of dying $_5q_{10}$ (per 1,000 children aged 10)				Probability of dying $_5q_{15}$ (per 1,000 youths aged 15)				Probability of dying $_5q_{20}$ (per 1,000 youths aged 20)			
		1990	2000	2015	2024	1990	2000	2015	2024	1990	2000	2015	2024	1990	2000	2015	2024
East Asia and Pacific	Total	5.6 (5.1–6.2)	3.5 (3.3–3.7)	1.7 (1.6–1.8)	1.3 (1.1–1.5)	3.1 (2.2–4.4)	2.3 (1.9–2.9)	1.5 (1.2–2.0)	1.3 (0.9–2.1)	5.4 (4.8–6.0)	4.1 (3.8–4.4)	2.7 (2.4–3.1)	2.3 (1.9–2.8)	4.6 (3.4–6.3)	4.5 (3.9–5.4)	3.5 (2.6–5.0)	3.5 (2.3–7.5)
	Female	5.2 (4.7–5.7)	3.0 (2.8–3.2)	1.4 (1.3–1.6)	1.1 (0.9–1.2)	2.7 (1.9–3.7)	1.8 (1.5–2.4)	1.2 (1.0–1.5)	1.0 (0.7–1.6)	3.8 (3.3–4.4)	2.8 (2.6–3.2)	1.7 (1.5–2.0)	1.4 (1.2–1.8)	2.9 (2.1–4.1)	3.0 (2.5–3.7)	2.0 (1.5–2.9)	1.9 (1.3–4.0)
	Male	6.0 (5.4–6.7)	3.9 (3.6–4.1)	1.9 (1.8–2.1)	1.5 (1.3–1.7)	3.5 (2.5–5.0)	2.7 (2.2–3.4)	1.8 (1.5–2.4)	1.5 (1.1–2.5)	6.9 (6.1–7.8)	5.2 (4.9–5.7)	3.6 (3.1–4.1)	3.1 (2.5–3.8)	6.3 (4.5–8.6)	5.9 (5.1–7.1)	4.9 (3.6–7.0)	4.9 (3.1–10.7)
North America	Total	1.1 (1.1–1.1)	0.8 (0.8–0.8)	0.6 (0.6–0.6)	0.6 (0.6–0.6)	1.3 (1.3–1.3)	1.0 (1.0–1.0)	0.7 (0.7–0.7)	0.8 (0.8–0.9)	4.3 (4.2–4.3)	3.3 (3.3–3.4)	2.3 (2.3–2.4)	2.5 (2.4–2.6)	5.4 (5.2–5.6)	4.5 (4.3–4.7)	4.3 (4.1–4.4)	4.3 (3.7–4.8)
	Female	0.9 (0.9–1.0)	0.7 (0.7–0.7)	0.5 (0.5–0.5)	0.5 (0.5–0.6)	1.0 (0.9–1.0)	0.8 (0.8–0.8)	0.6 (0.6–0.6)	0.7 (0.6–0.8)	2.3 (2.2–2.3)	2.0 (1.9–2.0)	1.4 (1.4–1.4)	1.5 (1.3–1.6)	2.5 (2.4–2.6)	2.3 (2.2–2.4)	2.2 (2.2–2.3)	2.4 (2.0–2.8)
	Male	1.3 (1.2–1.3)	0.9 (0.9–0.9)	0.6 (0.6–0.6)	0.7 (0.6–0.7)	1.6 (1.5–1.7)	1.2 (1.2–1.2)	0.8 (0.8–0.9)	1.0 (0.9–1.1)	6.1 (6.0–6.2)	4.6 (4.5–4.7)	3.2 (3.1–3.2)	3.5 (3.3–3.7)	8.1 (7.8–8.5)	6.6 (6.3–6.8)	6.1 (5.9–6.4)	6.0 (5.2–6.8)
Western Europe	Total	1.1 (1.1–1.1)	0.7 (0.7–0.7)	0.4 (0.4–0.4)	0.4 (0.4–0.4)	1.1 (1.1–1.1)	0.8 (0.8–0.8)	0.5 (0.5–0.5)	0.5 (0.4–0.5)	3.0 (2.9–3.0)	2.3 (2.3–2.4)	1.2 (1.2–1.2)	1.2 (1.2–1.2)	4.1 (4.1–4.2)	3.3 (3.3–3.4)	1.9 (1.9–1.9)	1.8 (1.8–1.9)
	Female	0.9 (0.9–0.9)	0.6 (0.6–0.6)	0.4 (0.4–0.4)	0.3 (0.3–0.4)	0.9 (0.8–0.9)	0.6 (0.6–0.7)	0.4 (0.4–0.4)	0.4 (0.4–0.4)	1.6 (1.6–1.6)	1.4 (1.4–1.4)	0.8 (0.8–0.8)	0.8 (0.8–0.8)	1.9 (1.9–2.0)	1.6 (1.5–1.6)	1.0 (1.0–1.0)	1.0 (1.0–1.1)
	Male	1.3 (1.2–1.3)	0.8 (0.8–0.8)	0.4 (0.4–0.4)	0.4 (0.4–0.4)	1.3 (1.3–1.3)	0.9 (0.9–0.9)	0.5 (0.5–0.5)	0.5 (0.5–0.5)	4.2 (4.2–4.3)	3.3 (3.2–3.3)	1.6 (1.6–1.7)	1.6 (1.5–1.6)	6.3 (6.2–6.4)	5.0 (5.0–5.1)	2.7 (2.7–2.8)	2.6 (2.5–2.7)
World	Total	9.6 (9.3–9.9)	7.0 (6.9–7.2)	4.2 (4.1–4.3)	3.4 (3.2–3.6)	5.1 (4.7–5.8)	4.1 (3.9–4.4)	3.1 (2.9–3.4)	2.7 (2.5–3.3)	7.8 (7.6–8.2)	6.6 (6.5–6.8)	4.9 (4.8–5.1)	4.3 (4.1–4.6)	9.0 (8.4–10.0)	8.7 (8.4–9.1)	6.4 (6.0–6.9)	6.1 (5.7–7.7)
	Female	9.3 (9.1–9.7)	6.8 (6.7–7.0)	4.0 (3.9–4.1)	3.2 (3.0–3.3)	4.9 (4.5–5.5)	3.8 (3.6–4.2)	2.8 (2.7–3.1)	2.4 (2.2–3.0)	6.9 (6.7–7.3)	5.9 (5.8–6.2)	4.0 (3.8–4.2)	3.3 (3.1–3.6)	7.2 (6.7–8.1)	7.5 (7.1–8.0)	4.6 (4.4–5.1)	4.1 (3.8–5.4)
	Male	9.8 (9.5–10.1)	7.2 (7.0–7.4)	4.4 (4.3–4.6)	3.6 (3.5–3.8)	5.4 (4.9–6.1)	4.3 (4.1–4.7)	3.4 (3.2–3.7)	3.0 (2.7–3.7)	8.7 (8.4–9.1)	7.3 (7.1–7.5)	5.8 (5.6–6.1)	5.3 (5.0–5.6)	10.7 (10.0–12.0)	9.8 (9.4–10.4)	8.0 (7.5–8.8)	7.9 (7.4–10.0)

Table A.2: Global and regional deaths by sex, 1990–2024

Number of deaths age 5–9, deaths age 10–14, deaths age 15–19, and deaths age 20–24 (thousands) for 1990, 2000, 2015, and 2024, by UNICEF reporting region and World. Values shown are medians with 90% uncertainty intervals in parentheses.

		Number of deaths age 5–9 (thousands)				Number of deaths age 10–14 (thousands)				Number of deaths age 15–19 (thousands)				Number of deaths age 20–24 (thousands)			
		1990	2000	2015	2024	1990	2000	2015	2024	1990	2000	2015	2024	1990	2000	2015	2024
West and Central Africa	Total	183 (172–195)	199 (190–211)	193 (183–205)	194 (176–213)	75 (58–102)	92 (76–111)	106 (88–125)	124 (92–177)	88 (77–102)	98 (91–107)	106 (98–116)	122 (107–143)	89 (67–129)	105 (91–121)	115 (99–135)	134 (109–197)

		Number of deaths age 5–9 (thousands)				Number of deaths age 10–14 (thousands)				Number of deaths age 15–19 (thousands)				Number of deaths age 20–24 (thousands)			
		1990	2000	2015	2024	1990	2000	2015	2024	1990	2000	2015	2024	1990	2000	2015	2024
	Female	88 (82–95)	96 (91–102)	91 (86–98)	90 (81–100)	37 (29–50)	45 (37–54)	50 (42–60)	58 (43–83)	45 (39–53)	51 (46–56)	49 (45–55)	53 (46–64)	42 (31–63)	55 (47–64)	55 (47–65)	60 (47–91)
	Male	95 (89–102)	104 (99–111)	101 (96–108)	104 (93–114)	38 (29–51)	47 (39–57)	56 (46–66)	66 (48–94)	42 (37–50)	48 (44–52)	57 (52–63)	68 (59–81)	47 (35–67)	50 (43–58)	60 (51–71)	75 (61–110)
Eastern and Southern Africa	Total	214 (199–230)	186 (178–197)	128 (120–137)	107 (97–115)	91 (69–118)	87 (74–102)	72 (60–89)	66 (51–104)	113 (106–123)	126 (120–133)	108 (100–119)	112 (101–127)	139 (124–158)	157 (147–170)	130 (114–150)	142 (120–206)
	Female	101 (94–109)	90 (85–95)	60 (56–65)	49 (44–54)	45 (34–58)	42 (36–49)	33 (28–41)	29 (23–45)	54 (50–60)	65 (61–69)	46 (42–51)	41 (37–48)	60 (54–69)	85 (79–93)	54 (47–62)	50 (41–73)
	Male	113 (104–122)	97 (92–103)	67 (63–73)	58 (52–63)	46 (35–60)	45 (38–53)	39 (32–48)	37 (29–59)	59 (55–65)	61 (58–65)	62 (57–69)	70 (62–81)	79 (69–91)	72 (66–79)	76 (65–90)	93 (77–137)
Latin America and Caribbean	Total	32 (31–33)	23 (23–24)	15 (15–16)	13 (12–14)	27 (26–29)	23 (22–24)	18 (17–20)	17 (16–20)	54 (53–55)	58 (57–59)	59 (58–60)	46 (44–48)	70 (67–74)	78 (76–80)	80 (78–84)	77 (71–91)
	Female	14 (13–14)	10 (10–10)	7 (6–7)	6 (5–6)	11 (10–12)	9 (9–10)	7 (7–8)	7 (7–8)	17 (17–18)	17 (16–17)	14 (14–15)	12 (11–13)	19 (18–21)	18 (17–19)	17 (16–18)	17 (15–20)
	Male	18 (18–18)	13 (13–13)	8 (8–9)	7 (7–8)	16 (15–17)	14 (13–14)	11 (10–12)	10 (9–12)	36 (35–38)	41 (41–42)	44 (43–45)	34 (32–36)	51 (49–53)	60 (58–61)	64 (61–66)	60 (55–71)
South Asia	Total	405 (391–420)	291 (283–300)	134 (129–140)	79 (72–85)	186 (159–216)	176 (157–197)	119 (106–134)	84 (64–111)	242 (232–253)	242 (233–251)	178 (170–190)	139 (122–158)	268 (242–306)	285 (261–312)	223 (199–249)	189 (139–274)
	Female	203 (193–213)	145 (139–152)	61 (57–65)	34 (31–38)	90 (77–105)	85 (75–95)	53 (46–59)	35 (27–47)	129 (121–137)	124 (116–132)	81 (75–87)	60 (50–70)	135 (120–154)	143 (128–158)	90 (80–102)	71 (50–109)
	Male	202 (193–212)	145 (139–152)	74 (70–78)	44 (40–49)	96 (82–112)	92 (81–103)	67 (59–75)	48 (37–65)	113 (106–121)	118 (110–125)	98 (91–106)	79 (68–93)	133 (119–155)	143 (129–158)	133 (117–151)	118 (85–172)
Middle East and North Africa	Total	50 (48–52)	25 (25–26)	24 (23–26)	23 (21–26)	31 (26–39)	21 (19–24)	19 (16–23)	23 (17–34)	38 (35–43)	31 (29–33)	34 (33–37)	35 (32–38)	43 (35–57)	32 (29–37)	47 (42–56)	39 (33–54)
	Female	23 (22–24)	11 (10–11)	10 (10–11)	10 (9–12)	14 (11–18)	9 (8–10)	7 (6–9)	9 (7–15)	13 (11–15)	10 (9–11)	9 (8–10)	10 (9–12)	12 (9–16)	10 (9–11)	10 (9–11)	10 (8–14)
	Male	27 (26–28)	15 (14–15)	14 (13–15)	13 (12–14)	17 (14–22)	13 (11–14)	11 (10–14)	13 (10–20)	25 (23–28)	21 (19–22)	26 (24–28)	25 (22–27)	31 (25–41)	22 (20–26)	37 (33–45)	29 (24–40)
Eastern Europe and Central Asia	Total	24 (24–24)	16 (16–17)	7 (7–7)	7 (7–7)	16 (14–18)	16 (15–18)	7 (7–7)	8 (8–8)	36 (29–40)	37 (35–39)	15 (15–15)	18 (18–19)	42 (35–51)	58 (54–62)	26 (26–27)	35 (32–39)
	Female	10 (9–10)	7 (7–7)	3 (3–3)	3 (3–3)	6 (5–7)	6 (5–7)	3 (3–3)	3 (3–3)	12 (9–14)	11 (10–12)	5 (5–5)	5 (5–5)	10 (9–13)	13 (12–14)	7 (7–7)	6 (6–7)
	Male	14 (14–15)	10 (9–10)	4 (4–4)	4 (4–4)	10 (9–12)	10 (9–11)	4 (4–4)	5 (5–5)	24 (20–27)	26 (25–28)	10 (10–10)	13 (13–14)	31 (27–39)	45 (42–48)	20 (19–20)	28 (26–32)

		Number of deaths age 5–9 (thousands)				Number of deaths age 10–14 (thousands)				Number of deaths age 15–19 (thousands)				Number of deaths age 20–24 (thousands)			
		1990	2000	2015	2024	1990	2000	2015	2024	1990	2000	2015	2024	1990	2000	2015	2024
East Asia and Pacific	Total	200 (181–220)	120 (113–127)	52 (48–57)	39 (34–45)	103 (73–144)	89 (73–114)	45 (36–59)	41 (29–67)	204 (184–229)	144 (135–155)	83 (73–94)	71 (58–87)	176 (128–240)	146 (126–175)	120 (89–171)	104 (68–221)
	Female	89 (80–98)	50 (46–53)	21 (19–23)	16 (13–18)	43 (31–60)	34 (28–45)	17 (13–22)	15 (11–25)	70 (61–82)	49 (44–54)	25 (21–29)	21 (17–27)	54 (39–77)	47 (40–59)	33 (24–47)	27 (18–57)
	Male	111 (100–123)	70 (66–75)	32 (29–35)	24 (20–28)	60 (42–84)	54 (45–69)	29 (23–38)	26 (18–43)	134 (119–152)	95 (89–104)	58 (51–66)	50 (40–62)	121 (88–166)	99 (84–118)	88 (64–125)	77 (49–167)
North America	Total	4 (4–5)	4 (4–4)	3 (3–3)	3 (3–3)	5 (5–5)	5 (4–5)	3 (3–3)	4 (4–4)	17 (16–17)	15 (15–15)	11 (11–11)	13 (12–13)	23 (22–24)	19 (19–20)	22 (21–22)	21 (18–24)
	Female	2 (2–2)	2 (1–2)	1 (1–1)	1 (1–1)	2 (2–2)	2 (2–2)	1 (1–1)	2 (1–2)	4 (4–4)	4 (4–4)	3 (3–3)	4 (3–4)	5 (5–6)	5 (5–5)	5 (5–6)	6 (5–7)
	Male	3 (3–3)	2 (2–2)	1 (1–2)	1 (1–2)	3 (3–3)	3 (3–3)	2 (2–2)	2 (2–3)	12 (12–12)	11 (10–11)	8 (8–8)	9 (9–9)	18 (17–19)	15 (14–15)	16 (16–17)	16 (13–18)
Western Europe	Total	6 (6–6)	4 (4–4)	2 (2–2)	2 (2–2)	7 (6–7)	4 (4–5)	2 (2–2)	2 (2–3)	19 (19–19)	14 (14–14)	6 (6–7)	6 (6–7)	29 (29–29)	21 (20–21)	11 (11–11)	10 (10–10)
	Female	3 (3–3)	2 (2–2)	1 (1–1)	1 (1–1)	3 (2–3)	2 (2–2)	1 (1–1)	1 (1–1)	5 (5–5)	4 (4–4)	2 (2–2)	2 (2–2)	7 (7–7)	5 (5–5)	3 (3–3)	3 (3–3)
	Male	4 (4–4)	2 (2–2)	1 (1–1)	1 (1–1)	4 (4–4)	3 (3–3)	1 (1–1)	1 (1–1)	14 (14–14)	10 (10–10)	4 (4–4)	4 (4–4)	22 (22–23)	16 (16–16)	8 (8–8)	7 (7–8)
World	Total	1,118 (1,088–1,150)	868 (853–889)	558 (544–576)	466 (443–488)	540 (496–611)	513 (482–556)	392 (368–427)	369 (337–456)	810 (784–845)	765 (750–785)	600 (586–624)	561 (536–600)	879 (822–979)	900 (865–948)	775 (733–842)	752 (708–955)
	Female	531 (515–549)	411 (401–423)	255 (248–264)	210 (199–221)	250 (229–283)	233 (219–254)	173 (161–188)	160 (145–199)	350 (336–369)	334 (324–346)	234 (226–246)	208 (196–227)	346 (322–388)	380 (363–405)	273 (258–298)	249 (231–323)
	Male	586 (568–605)	457 (447–470)	303 (295–313)	256 (243–270)	291 (266–329)	280 (263–303)	220 (206–240)	209 (191–259)	460 (441–483)	431 (420–445)	367 (355–383)	353 (335–379)	533 (496–599)	520 (498–548)	502 (471–550)	503 (471–641)

Table A.3: Global and regional annual rate of reduction

Annual rate of reduction (ARR,%) for probability of dying $_5q_5$, $_5q_{10}$, $_5q_{15}$, and $_5q_{20}$ for the periods 1990-2000, 2000-2015, and 2015-2024, by UNICEF reporting region and World. Values shown are medians with 90% uncertainty intervals in parentheses.

	Annual rate of reduction in probability of dying $_5q_5$ (%)			Annual rate of reduction in probability of dying $_5q_{10}$ (%)			Annual rate of reduction in probability of dying $_5q_{15}$ (%)			Annual rate of reduction in probability of dying $_5q_{20}$ (%)		
Region	1990-2000	2000-2015	2015-2024	1990-2000	2000-2015	2015-2024	1990-2000	2000-2015	2015-2024	1990-2000	2000-2015	2015-2024
West and Central Africa	1.7 (1.3–2.0)	3.1 (2.7–3.6)	2.0 (1.4–2.7)	0.6 (-1.5–3.1)	1.9 (0.4–3.4)	1.2 (-2.4–3.7)	2.1 (1.1–3.1)	2.1 (1.4–2.8)	1.6 (0.4–2.7)	1.8 (-0.5–4.9)	2.0 (0.7–3.3)	1.3 (-2.4–3.1)
Eastern and Southern Africa	3.8 (3.2–4.2)	4.9 (4.5–5.3)	3.5 (3.0–4.3)	3.4 (0.9–5.7)	3.6 (1.9–5.2)	3.0 (-0.9–4.8)	1.9 (1.3–2.6)	3.5 (2.9–4.0)	2.2 (1.4–3.0)	1.6 (0.4–2.8)	4.0 (3.0–5.0)	1.6 (-1.8–3.2)

Region	Annual rate of reduction in probability of dying $_5q_5$ (%)			Annual rate of reduction in probability of dying $_5q_{10}$ (%)			Annual rate of reduction in probability of dying $_5q_{15}$ (%)			Annual rate of reduction in probability of dying $_5q_{20}$ (%)		
	1990-2000	2000-2015	2015-2024	1990-2000	2000-2015	2015-2024	1990-2000	2000-2015	2015-2024	1990-2000	2000-2015	2015-2024
Latin America and Caribbean	3.7 (3.5–3.8)	2.5 (2.3–2.6)	1.1 (0.8–1.4)	2.5 (2.0–3.1)	1.4 (0.9–1.8)	0.3 (-0.8–0.9)	0.6 (0.4–0.9)	0.2 (0.1–0.4)	2.2 (1.7–2.6)	0.5 (0.1–1.0)	0.5 (0.2–0.8)	0.4 (-1.3–1.2)
South Asia	4.6 (4.2–4.9)	5.5 (5.2–5.8)	5.5 (4.9–6.2)	2.7 (1.0–4.4)	3.4 (2.4–4.5)	3.7 (0.8–6.4)	2.4 (2.0–2.9)	3.2 (2.7–3.6)	3.0 (1.8–4.4)	1.7 (0.4–3.3)	3.3 (2.4–4.3)	2.6 (-1.5–6.1)
Middle East and North Africa	7.3 (6.9–7.6)	1.3 (1.0–1.6)	1.9 (0.8–3.0)	6.4 (4.7–8.4)	0.9 (-0.7–2.0)	0.4 (-3.5–2.8)	5.7 (5.0–6.4)	-0.6 (-1.1—0.2)	1.6 (0.9–2.4)	6.0 (4.3–8.3)	-1.0 (-2.3–0.1)	2.5 (-0.7–4.4)
Eastern Europe and Central Asia	2.1 (2.0–2.2)	5.2 (5.1–5.3)	1.1 (0.9–1.2)	0.5 (-0.7–1.8)	3.5 (2.9–4.1)	0.2 (-0.4–0.7)	0.5 (-1.1–1.4)	4.1 (3.8–4.4)	-1.7 (-2.0—1.3)	-2.5 (-3.9—0.6)	4.7 (4.2–5.2)	-4.8 (-6.1—3.8)
East Asia and Pacific	4.8 (4.2–5.4)	4.8 (4.1–5.4)	3.1 (2.1–4.3)	3.2 (-0.2–5.9)	2.6 (0.5–4.9)	2.0 (-2.0–4.7)	2.7 (1.9–3.6)	2.8 (1.9–3.8)	1.6 (0.1–3.2)	0.3 (-2.6–3.1)	1.6 (-0.9–4.0)	0.1 (-6.5–3.7)
North America	3.2 (3.1–3.4)	2.4 (2.2–2.5)	-0.8 (-1.2—0.5)	2.5 (2.1–3.0)	2.3 (2.0–2.6)	-1.9 (-2.9—0.9)	2.5 (2.3–2.7)	2.4 (2.2–2.5)	-0.9 (-1.4—0.4)	1.8 (1.3–2.4)	0.4 (0.0–0.7)	0.0 (-1.5–1.7)
Western Europe	4.3 (4.2–4.3)	3.8 (3.7–3.8)	0.7 (0.5–0.9)	3.2 (3.0–3.5)	3.5 (3.3–3.7)	0.4 (-0.3–0.9)	2.4 (2.3–2.4)	4.3 (4.2–4.3)	0.4 (0.2–0.6)	2.1 (2.0–2.3)	3.8 (3.7–3.9)	0.4 (-0.0–0.8)
World	3.1 (2.9–3.3)	3.4 (3.2–3.6)	2.4 (2.0–2.8)	2.3 (1.4–3.4)	1.8 (1.1–2.4)	1.6 (-0.4–2.4)	1.7 (1.4–2.0)	2.0 (1.7–2.2)	1.5 (1.0–2.0)	0.4 (-0.3–1.4)	2.1 (1.4–2.6)	0.6 (-1.8–1.2)

Table A.4: Country probability of dying, 1990–2024

Probability of dying $_5q_5$: the probability of dying between exact age 5 and exact age 10, expressed per 1,000 children aged 5. Probability of dying $_5q_{10}$: the probability of dying between exact age 10 and exact age 15, expressed per 1,000 children aged 10. Probability of dying $_5q_{15}$: the probability of dying between exact age 15 and exact age 20, expressed per 1,000 youths aged 15. Probability of dying $_5q_{20}$: the probability of dying between exact age 20 and exact age 25, expressed per 1,000 youths aged 20. Values shown for 1990, 2000, 2015, and 2024, by country. Medians with 90% uncertainty intervals in parentheses.

Country	Probability of dying $_5q_5$ (per 1,000 children aged 5)				Probability of dying $_5q_{10}$ (per 1,000 children aged 10)				Probability of dying $_5q_{15}$ (per 1,000 youths aged 15)				Probability of dying $_5q_{20}$ (per 1,000 youths aged 20)			
	1990	2000	2015	2024	1990	2000	2015	2024	1990	2000	2015	2024	1990	2000	2015	2024
Afghanistan	17.9 (13.8–21.4)	5.7 (5.0–6.6)	5.1 (4.4–6.0)	5.1 (4.0–6.3)	11.8 (0.0–45.1)	4.4 (1.6–7.8)	3.7 (1.4–6.2)	4.2 (0.0–12.6)	26.6 (18.0–35.7)	10.1 (8.0–12.6)	14.5 (10.6–19.9)	11.4 (6.5–17.9)	49.3 (11.8–153.5)	17.9 (11.3–26.7)	20.3 (8.1–37.4)	16.6 (1.8–56.2)
Albania	3.8 (3.6–3.9)	2.2 (2.1–2.2)	1.1 (1.0–1.2)	0.7 (0.6–0.8)	2.6 (2.3–2.9)	1.8 (1.6–2.0)	1.2 (1.0–1.4)	0.9 (0.6–1.3)	3.3 (3.1–3.4)	3.4 (3.2–3.6)	2.1 (2.0–2.3)	1.4 (1.1–1.8)	4.8 (4.4–5.1)	5.3 (5.0–5.7)	2.4 (2.2–2.7)	1.9 (1.1–3.0)
Algeria	5.5 (5.3–5.6)	3.6 (3.6–3.7)	2.0 (2.0–2.0)	1.5 (1.4–1.5)	3.8 (3.0–4.6)	2.8 (2.6–2.9)	1.9 (1.8–1.9)	1.6 (1.4–1.7)	5.2 (4.8–5.6)	3.9 (3.8–4.0)	2.7 (2.7–2.8)	2.6 (2.5–2.7)	6.0 (4.5–7.8)	5.2 (5.0–5.4)	3.5 (3.3–3.6)	3.5 (3.1–4.0)
Andorra	1.4 (1.1–1.7)	0.8 (0.7–1.1)	0.4 (0.3–0.5)	0.3 (0.2–0.4)	1.4 (1.1–1.8)	0.9 (0.7–1.2)	0.5 (0.4–0.6)	0.4 (0.3–0.5)	3.6 (2.8–4.6)	2.5 (2.0–3.2)	1.5 (1.2–1.9)	1.2 (0.9–1.5)	5.3 (4.1–6.7)	3.7 (2.9–4.7)	2.2 (1.7–2.8)	1.8 (1.4–2.2)
Angola	36.3 (28.3–46.2)	26.7 (20.8–34.0)	8.4 (6.5–10.7)	6.6 (5.1–8.4)	17.9 (13.9–23.1)	13.6 (10.5–17.4)	5.7 (4.4–7.3)	4.7 (3.7–6.0)	34.8 (27.1–44.3)	26.9 (21.0–34.2)	11.2 (8.7–14.2)	9.5 (7.4–12.1)	55.2 (42.7–71.1)	39.0 (30.2–50.1)	16.6 (12.9–21.2)	14.4 (11.2–18.3)
Anguilla	1.6 (1.2–2.0)	1.1 (0.8–1.4)	0.7 (0.6–0.9)	0.6 (0.5–0.8)	1.8 (1.4–2.3)	1.4 (1.1–1.8)	1.0 (0.8–1.3)	0.9 (0.7–1.1)	4.4 (3.4–5.6)	3.4 (2.7–4.4)	2.7 (2.1–3.4)	2.3 (1.8–3.0)	6.5 (5.1–8.3)	5.1 (4.0–6.6)	4.0 (3.1–5.2)	3.5 (2.7–4.5)

Country	Probability of dying $_{5}q_{5}$ (per 1,000 children aged 5) 1990	2000	2015	2024	Probability of dying $_{5}q_{10}$ (per 1,000 children aged 10) 1990	2000	2015	2024	Probability of dying $_{5}q_{15}$ (per 1,000 youths aged 15) 1990	2000	2015	2024	Probability of dying $_{5}q_{20}$ (per 1,000 youths aged 20) 1990	2000	2015	2024
Antigua and Barbuda	1.2 (0.9–1.5)	1.3 (1.0–1.6)	1.0 (0.8–1.3)	0.9 (0.7–1.1)	1.5 (1.2–1.9)	1.6 (1.2–2.0)	1.3 (1.0–1.7)	1.2 (0.9–1.5)	2.9 (1.7–4.2)	4.3 (3.5–5.2)	2.8 (2.2–3.6)	1.7 (1.0–2.8)	3.4 (0.6–7.5)	5.1 (3.5–6.8)	3.8 (2.5–5.2)	3.0 (1.0–6.0)
Argentina	1.6 (1.6–1.7)	1.3 (1.3–1.3)	0.9 (0.9–0.9)	0.9 (0.9–0.9)	1.8 (1.7–1.9)	1.5 (1.4–1.6)	1.2 (1.1–1.2)	1.2 (1.1–1.4)	3.7 (3.6–3.7)	3.9 (3.9–4.0)	3.8 (3.7–3.8)	2.9 (2.7–3.1)	5.0 (4.8–5.2)	5.3 (5.1–5.5)	5.0 (4.8–5.2)	4.4 (3.5–5.5)
Armenia	1.8 (1.7–1.9)	1.0 (0.9–1.0)	1.0 (1.0–1.1)	1.0 (0.9–1.0)	1.5 (1.4–1.7)	0.9 (0.8–1.0)	1.0 (0.9–1.2)	1.0 (0.8–1.2)	2.4 (2.2–2.5)	2.0 (1.9–2.1)	2.8 (2.6–3.0)	1.7 (1.5–2.0)	3.9 (3.5–4.3)	2.2 (1.9–2.5)	3.1 (2.6–3.6)	2.8 (2.2–3.4)
Australia	0.9 (0.9–0.9)	0.6 (0.6–0.6)	0.4 (0.4–0.4)	0.4 (0.3–0.4)	1.0 (0.9–1.0)	0.7 (0.7–0.7)	0.5 (0.4–0.5)	0.5 (0.4–0.5)	3.3 (3.2–3.3)	2.6 (2.6–2.7)	1.5 (1.5–1.5)	1.3 (1.2–1.4)	4.5 (4.3–4.7)	3.7 (3.6–3.9)	2.0 (1.9–2.1)	1.9 (1.6–2.2)
Austria	0.9 (0.9–0.9)	0.6 (0.6–0.6)	0.4 (0.4–0.4)	0.3 (0.3–0.4)	0.9 (0.9–1.0)	0.7 (0.6–0.7)	0.5 (0.4–0.5)	0.4 (0.4–0.5)	3.6 (3.5–3.6)	2.7 (2.7–2.8)	1.4 (1.4–1.5)	1.7 (1.6–1.8)	4.4 (4.1–4.6)	3.4 (3.2–3.6)	1.8 (1.7–2.0)	2.0 (1.7–2.3)
Azerbaijan	3.0 (2.9–3.1)	3.0 (3.0–3.1)	1.5 (1.5–1.5)	1.6 (1.5–1.6)	2.0 (1.8–2.2)	2.2 (2.1–2.4)	1.4 (1.3–1.4)	1.6 (1.3–1.8)	2.6 (2.5–2.7)	3.7 (3.6–3.8)	2.6 (2.6–2.7)	2.7 (2.5–2.9)	3.7 (3.4–4.0)	5.5 (5.2–5.7)	3.7 (3.4–4.0)	3.7 (2.2–5.5)
Bahamas	2.5 (2.1–3.0)	2.5 (2.3–2.8)	1.4 (1.2–1.7)	1.0 (0.7–1.4)	1.5 (0.6–2.7)	1.9 (1.5–2.4)	1.6 (1.1–2.2)	1.3 (0.5–2.5)	3.9 (3.2–4.7)	5.0 (4.6–5.5)	5.6 (5.1–6.2)	3.0 (2.1–4.1)	7.9 (6.2–9.8)	8.9 (7.8–10.1)	11.7 (10.4–13.0)	6.9 (3.8–11.8)
Bahrain	2.0 (1.7–2.3)	1.8 (1.6–2.0)	1.1 (0.9–1.2)	0.9 (0.8–1.1)	1.7 (1.2–2.2)	1.4 (1.1–1.8)	1.0 (0.8–1.2)	0.9 (0.6–1.3)	3.0 (2.6–3.4)	2.7 (2.5–2.9)	2.1 (2.0–2.3)	1.7 (1.4–2.0)	3.2 (2.4–4.0)	3.3 (2.9–3.8)	2.7 (2.4–3.1)	2.1 (1.5–2.7)
Bangladesh	16.5 (15.8–17.2)	6.9 (6.6–7.1)	3.4 (3.3–3.6)	2.4 (2.2–2.7)	8.2 (5.5–11.0)	4.1 (3.2–5.2)	2.6 (2.1–3.1)	2.0 (0.7–3.9)	10.1 (8.7–11.3)	5.4 (4.7–6.3)	5.7 (5.3–6.3)	5.3 (2.4–7.1)	13.3 (11.0–16.1)	9.1 (7.7–10.3)	5.1 (4.2–5.9)	4.4 (1.5–8.2)
Barbados	1.3 (1.0–1.7)	1.0 (0.9–1.2)	0.6 (0.4–0.9)	0.5 (0.2–0.8)	1.6 (0.9–2.3)	1.6 (1.2–2.0)	1.3 (0.7–2.1)	1.1 (0.4–2.3)	4.4 (3.7–5.2)	3.4 (3.0–3.9)	2.6 (1.9–3.5)	2.0 (1.1–3.1)	5.7 (4.2–7.1)	5.0 (4.1–6.1)	3.8 (2.0–6.2)	3.4 (0.8–10.6)
Belarus	2.1 (2.1–2.2)	1.5 (1.5–1.6)	0.8 (0.7–0.8)	0.4 (0.4–0.5)	1.9 (1.7–2.0)	1.4 (1.3–1.6)	0.8 (0.7–0.9)	0.5 (0.3–0.8)	4.2 (4.1–4.3)	4.3 (4.2–4.4)	2.1 (2.1–2.2)	1.4 (1.1–1.7)	7.1 (6.8–7.4)	8.7 (8.3–9.0)	3.7 (3.4–3.9)	2.2 (1.1–4.0)
Belgium	1.0 (1.0–1.1)	0.7 (0.7–0.7)	0.4 (0.4–0.4)	0.3 (0.3–0.4)	1.0 (1.0–1.1)	0.7 (0.7–0.8)	0.5 (0.4–0.5)	0.4 (0.3–0.5)	3.0 (3.0–3.1)	2.5 (2.5–2.6)	1.3 (1.2–1.3)	1.0 (0.9–1.0)	4.5 (4.3–4.7)	3.9 (3.7–4.1)	2.0 (1.9–2.2)	1.6 (1.4–1.8)
Belize	2.6 (2.2–2.9)	2.6 (2.4–2.8)	1.6 (1.5–1.8)	1.1 (0.9–1.3)	2.3 (1.5–3.3)	2.6 (2.1–3.1)	1.9 (1.5–2.2)	1.4 (0.8–2.0)	3.0 (2.3–3.9)	6.5 (6.0–7.1)	6.1 (5.6–6.6)	5.4 (4.5–6.4)	6.2 (4.3–8.5)	12.0 (10.7–13.3)	10.1 (9.1–11.2)	9.7 (7.4–12.1)
Benin	28.2 (25.2–31.4)	20.6 (18.5–22.7)	14.2 (12.3–16.2)	11.4 (9.2–13.8)	13.3 (7.0–20.1)	10.5 (6.4–14.9)	7.9 (4.3–11.7)	6.7 (2.1–13.0)	13.5 (10.5–17.4)	9.9 (8.1–12.0)	10.5 (7.9–14.0)	9.7 (5.8–14.7)	14.1 (7.6–20.9)	11.7 (7.5–15.9)	11.0 (5.2–17.7)	10.5 (0.9–30.3)
Bhutan	14.2 (8.3–16.6)	11.2 (8.0–14.6)	3.4 (1.8–6.5)	2.3 (0.9–5.0)	2.7 (0.0–15.0)	4.8 (0.0–10.6)	5.4 (1.2–10.2)	4.5 (0.7–10.6)	16.5 (10.1–23.8)	9.4 (7.4–12.0)	6.1 (3.7–9.6)	4.8 (2.3–8.2)	13.8 (0.0–34.8)	8.4 (3.4–14.2)	7.1 (0.8–15.8)	6.5 (0.0–24.2)
Bolivia (Plurinational State of)	7.2 (6.1–8.4)	4.5 (3.8–5.3)	2.4 (1.6–3.5)	1.8 (0.9–2.7)	4.6 (2.4–6.8)	3.5 (1.9–5.0)	2.4 (0.3–5.3)	1.9 (0.1–5.6)	11.0 (9.0–13.3)	8.2 (6.8–9.9)	5.0 (2.9–7.4)	3.9 (1.8–6.4)	11.9 (7.1–16.6)	8.0 (5.2–11.0)	5.3 (0.4–16.2)	4.8 (0.1–23.1)
Bosnia and Herzegovina	1.4 (1.3–1.5)	0.8 (0.7–0.8)	0.6 (0.5–0.6)	0.8 (0.7–0.9)	1.2 (1.0–1.4)	0.8 (0.7–0.9)	0.7 (0.6–0.8)	0.9 (0.6–1.3)	2.6 (2.4–2.8)	1.6 (1.5–1.7)	1.6 (1.5–1.8)	1.7 (1.5–2.0)	3.9 (3.6–4.3)	2.2 (2.0–2.4)	2.5 (2.3–2.8)	2.3 (1.7–3.0)
Botswana	12.9 (9.9–16.9)	8.3 (6.7–10.5)	4.1 (3.2–5.3)	2.6 (1.7–3.8)	6.9 (1.1–13.2)	5.3 (1.7–9.0)	3.7 (1.9–5.7)	3.0 (1.0–5.3)	12.7 (9.3–16.9)	9.5 (6.8–11.9)	5.2 (4.1–6.6)	4.6 (3.1–6.9)	25.1 (15.5–37.6)	21.9 (14.9–31.1)	10.7 (6.5–15.6)	9.1 (3.5–17.8)
Brazil	2.0 (1.9–2.0)	1.7 (1.6–1.7)	1.3 (1.3–1.3)	1.2 (1.2–1.2)	2.2 (2.0–2.4)	2.0 (1.9–2.0)	1.7 (1.6–1.7)	1.6 (1.5–1.7)	5.5 (5.2–5.7)	5.8 (5.7–5.9)	7.0 (6.9–7.2)	4.7 (4.6–4.9)	8.5 (7.7–9.3)	8.6 (8.3–9.0)	9.1 (8.7–9.5)	7.8 (7.4–8.3)

Country	Probability of dying $_5q_5$ (per 1,000 children aged 5)				Probability of dying $_5q_{10}$ (per 1,000 children aged 10)				Probability of dying $_5q_{15}$ (per 1,000 youths aged 15)				Probability of dying $_5q_{20}$ (per 1,000 youths aged 20)			
	1990	2000	2015	2024	1990	2000	2015	2024	1990	2000	2015	2024	1990	2000	2015	2024
British Virgin Islands	1.8 (1.4–2.3)	1.4 (1.1–1.8)	1.3 (1.0–1.7)	1.1 (0.9–1.4)	2.0 (1.6–2.6)	1.7 (1.3–2.2)	1.6 (1.3–2.1)	1.4 (1.1–1.8)	4.8 (3.7–6.2)	4.1 (3.2–5.2)	3.9 (3.1–5.0)	3.5 (2.7–4.5)	7.1 (5.5–9.2)	6.1 (4.7–7.8)	5.8 (4.5–7.5)	5.2 (4.1–6.7)
Brunei Darussalam	2.0 (1.8–2.2)	1.5 (1.4–1.6)	1.2 (1.0–1.3)	1.0 (0.8–1.2)	1.9 (1.5–2.4)	1.4 (1.1–1.7)	1.0 (0.7–1.3)	0.8 (0.4–1.3)	4.4 (3.7–5.2)	3.2 (2.9–3.5)	1.6 (1.4–1.8)	1.8 (1.4–2.3)	5.5 (4.1–7.0)	4.2 (3.4–5.0)	2.6 (2.1–3.2)	2.5 (1.6–3.6)
Bulgaria	2.1 (2.0–2.1)	1.7 (1.6–1.7)	0.9 (0.9–1.0)	0.7 (0.6–0.7)	1.9 (1.8–2.0)	1.6 (1.5–1.8)	1.0 (0.9–1.1)	0.8 (0.7–1.0)	3.5 (3.4–3.6)	2.9 (2.8–3.0)	2.2 (2.2–2.3)	2.1 (2.0–2.2)	4.7 (4.4–4.9)	4.1 (3.9–4.3)	3.1 (2.9–3.4)	3.1 (2.7–3.4)
Burkina Faso	26.9 (23.7–30.5)	24.0 (21.4–27.1)	9.0 (7.1–11.1)	4.7 (3.1–6.2)	10.0 (3.2–17.1)	10.2 (4.3–16.6)	4.9 (1.0–10.1)	2.9 (0.0–8.9)	17.3 (14.7–20.4)	13.0 (11.3–14.9)	7.3 (6.0–8.8)	6.7 (4.7–9.0)	19.9 (13.9–26.3)	15.3 (11.6–19.3)	9.2 (6.2–12.5)	10.4 (4.6–18.9)
Burundi	37.7 (33.1–43.1)	36.8 (32.4–41.8)	12.3 (9.8–15.1)	9.7 (6.7–12.7)	20.9 (8.5–35.1)	22.9 (11.5–35.9)	9.2 (3.0–16.9)	8.0 (0.0–26.8)	29.3 (22.0–40.7)	27.2 (22.1–33.9)	8.9 (6.5–11.7)	7.4 (4.3–10.8)	43.3 (12.3–92.1)	37.3 (22.3–54.5)	9.1 (3.7–16.1)	8.1 (0.3–28.0)
Cabo Verde	2.7 (2.4–3.1)	2.3 (2.1–2.4)	1.2 (1.1–1.4)	0.9 (0.7–1.1)	2.4 (1.6–3.3)	2.2 (1.8–2.5)	1.3 (1.0–1.6)	1.0 (0.5–1.5)	2.9 (2.4–3.6)	3.6 (3.3–3.9)	2.8 (2.4–3.2)	2.2 (1.5–3.0)	5.0 (3.7–6.4)	5.5 (4.8–6.1)	3.9 (2.9–5.0)	2.6 (1.1–4.3)
Cambodia	22.3 (17.7–27.3)	12.0 (10.4–13.8)	3.4 (2.2–4.5)	2.3 (1.2–3.2)	9.7 (1.3–18.9)	5.6 (2.6–8.8)	2.0 (0.0–4.7)	1.5 (0.0–4.9)	17.1 (13.0–22.5)	8.4 (7.0–9.9)	4.8 (3.7–6.3)	4.9 (3.0–7.2)	19.5 (10.1–29.8)	11.2 (8.2–14.4)	5.2 (2.7–7.9)	4.4 (0.0–12.6)
Cameroon	21.3 (19.1–23.9)	19.5 (17.6–21.7)	14.2 (11.8–16.9)	10.9 (7.9–13.7)	10.8 (6.1–15.7)	10.5 (6.5–15.2)	9.0 (3.7–14.3)	7.5 (0.7–16.3)	13.1 (10.8–15.6)	13.4 (11.4–15.5)	12.1 (9.2–15.9)	10.3 (6.2–16.0)	16.9 (11.5–22.3)	19.3 (15.0–24.7)	17.6 (10.5–25.1)	16.2 (4.1–38.3)
Canada	1.0 (1.0–1.0)	0.6 (0.6–0.7)	0.4 (0.4–0.4)	0.5 (0.4–0.5)	1.1 (1.0–1.2)	0.8 (0.7–0.8)	0.6 (0.5–0.6)	0.6 (0.5–0.7)	3.4 (3.4–3.5)	2.5 (2.4–2.5)	1.7 (1.7–1.7)	1.7 (1.6–1.8)	4.1 (3.9–4.3)	3.1 (3.0–3.2)	2.6 (2.5–2.7)	2.9 (2.6–3.3)
Central African Republic	17.4 (14.7–21.0)	15.7 (13.0–18.8)	17.5 (13.0–23.0)	11.2 (7.1–15.7)	12.2 (5.0–20.0)	11.8 (4.7–19.3)	13.8 (4.1–25.9)	10.3 (0.6–25.6)	17.5 (14.0–21.9)	17.6 (12.8–24.1)	17.1 (8.4–30.0)	10.1 (3.8–19.8)	26.8 (17.8–36.9)	27.6 (13.6–45.8)	33.3 (5.0–110.3)	22.4 (2.5–103.2)
Chad	34.8 (29.0–40.9)	24.8 (21.8–28.3)	14.8 (12.3–17.8)	10.7 (7.6–14.1)	14.4 (3.9–25.8)	12.8 (6.9–19.1)	11.2 (5.9–16.7)	9.7 (3.3–18.3)	26.6 (21.1–33.7)	20.7 (17.2–24.7)	17.0 (11.9–23.6)	13.5 (7.4–21.0)	30.4 (18.2–43.0)	19.3 (12.4–27.0)	19.5 (7.6–32.7)	17.9 (2.1–56.2)
Chile	1.5 (1.5–1.6)	1.0 (1.0–1.1)	0.7 (0.6–0.7)	0.6 (0.6–0.6)	1.6 (1.5–1.7)	1.1 (1.1–1.2)	0.8 (0.8–0.9)	0.8 (0.7–0.9)	3.3 (3.2–3.4)	2.6 (2.5–2.6)	2.1 (2.1–2.2)	2.1 (2.0–2.2)	5.1 (4.8–5.3)	4.1 (3.9–4.3)	3.1 (3.0–3.3)	3.4 (3.2–3.7)
China	4.4 (3.7–5.3)	2.4 (2.2–2.6)	1.1 (0.9–1.3)	0.8 (0.6–1.1)	2.7 (1.2–4.4)	1.8 (1.3–2.3)	1.1 (0.6–1.7)	0.9 (0.3–2.0)	5.0 (4.2–5.9)	3.2 (2.9–3.6)	1.6 (1.2–2.0)	1.2 (0.7–1.9)	3.6 (1.8–5.9)	3.3 (2.4–4.3)	2.6 (1.4–4.6)	2.4 (0.6–7.7)
Colombia	2.1 (2.1–2.2)	1.8 (1.8–1.8)	1.2 (1.2–1.2)	1.0 (1.0–1.1)	2.4 (2.3–2.5)	2.2 (2.1–2.2)	1.5 (1.5–1.6)	1.4 (1.3–1.5)	7.8 (7.6–8.0)	9.5 (9.4–9.7)	5.0 (4.9–5.1)	4.4 (4.2–4.5)	12.5 (12.0–13.1)	14.9 (14.3–15.5)	7.5 (7.2–7.8)	7.4 (7.0–7.8)
Comoros	11.1 (8.9–14.1)	8.2 (6.7–10.1)	4.5 (3.5–5.9)	3.8 (2.4–5.1)	5.3 (0.3–11.3)	4.2 (1.1–7.8)	2.8 (0.8–5.1)	2.5 (0.0–6.1)	12.0 (8.3–16.3)	7.9 (5.7–10.1)	4.6 (3.5–6.0)	4.3 (2.7–6.3)	11.1 (3.2–22.9)	8.2 (3.9–13.8)	4.8 (2.2–7.7)	4.4 (0.5–10.9)
Congo	22.3 (11.4–28.3)	12.6 (10.1–15.8)	4.3 (2.7–6.2)	3.0 (1.5–4.6)	8.5 (0.0–33.3)	6.4 (1.6–11.6)	3.7 (0.3–7.6)	2.8 (0.0–8.2)	26.4 (13.9–40.2)	14.2 (11.3–17.8)	5.2 (2.9–8.7)	3.9 (1.7–7.5)	32.2 (1.2–70.4)	21.7 (15.1–29.1)	11.2 (4.4–20.9)	9.9 (2.2–30.6)
Cook Islands	2.7 (2.1–3.5)	2.2 (1.7–2.8)	1.6 (1.3–2.1)	1.6 (1.2–2.0)	2.3 (1.8–2.9)	1.9 (1.5–2.5)	1.5 (1.2–1.9)	1.5 (1.2–1.9)	7.7 (5.6–10.7)	8.8 (6.8–11.5)	6.2 (3.4–8.3)	5.0 (2.1–7.2)	9.6 (5.1–14.3)	7.0 (3.0–11.4)	3.3 (0.0–14.4)	3.1 (0.0–21.0)
Costa Rica	1.4 (1.3–1.5)	1.1 (1.1–1.1)	0.8 (0.8–0.8)	0.8 (0.7–0.9)	1.5 (1.3–1.7)	1.3 (1.2–1.4)	1.1 (1.0–1.2)	1.2 (0.9–1.4)	2.7 (2.6–2.9)	3.1 (3.0–3.2)	2.8 (2.7–2.9)	3.5 (3.3–3.7)	3.7 (3.4–4.1)	4.3 (4.0–4.6)	4.4 (4.1–4.7)	6.0 (5.5–6.6)
Croatia	1.5 (1.4–1.6)	0.9 (0.8–0.9)	0.6 (0.6–0.7)	1.1 (1.0–1.2)	1.4 (1.3–1.6)	0.8 (0.7–0.9)	0.5 (0.4–0.6)	0.9 (0.5–1.4)	3.5 (3.4–3.7)	2.6 (2.5–2.8)	1.5 (1.4–1.6)	2.6 (2.2–3.0)	6.4 (6.0–6.8)	3.7 (3.4–4.0)	2.1 (1.9–2.4)	2.5 (1.3–4.0)

Country	Probability of dying $_5q_5$ (per 1,000 children aged 5)				Probability of dying $_5q_{10}$ (per 1,000 children aged 10)				Probability of dying $_5q_{15}$ (per 1,000 youths aged 15)				Probability of dying $_5q_{20}$ (per 1,000 youths aged 20)			
	1990	2000	2015	2024	1990	2000	2015	2024	1990	2000	2015	2024	1990	2000	2015	2024
Cuba	1.8 (1.7–1.9)	1.3 (1.3–1.4)	0.9 (0.9–1.0)	1.1 (1.0–1.2)	2.1 (1.9–2.2)	1.5 (1.4–1.6)	1.0 (1.0–1.1)	1.2 (1.0–1.4)	4.6 (4.5–4.7)	2.9 (2.9–3.0)	1.9 (1.9–2.0)	2.5 (2.4–2.6)	5.9 (5.7–6.2)	4.1 (4.0–4.3)	2.6 (2.5–2.8)	3.6 (3.3–3.9)
Cyprus	1.1 (0.9–1.2)	1.0 (0.9–1.1)	0.6 (0.5–0.7)	0.4 (0.3–0.5)	0.7 (0.4–1.0)	0.8 (0.6–1.0)	0.6 (0.4–0.7)	0.5 (0.3–0.8)	2.9 (2.5–3.3)	3.4 (3.1–3.6)	1.0 (0.9–1.2)	0.7 (0.5–1.0)	3.6 (2.8–4.4)	4.2 (3.7–4.8)	1.9 (1.6–2.2)	2.0 (1.4–2.8)
Czechia	1.3 (1.2–1.3)	0.8 (0.8–0.9)	0.4 (0.4–0.5)	0.4 (0.4–0.5)	1.1 (1.0–1.2)	0.9 (0.8–1.0)	0.5 (0.4–0.6)	0.5 (0.4–0.6)	2.9 (2.9–3.0)	2.4 (2.4–2.5)	1.6 (1.5–1.6)	1.4 (1.3–1.4)	3.9 (3.8–4.1)	3.4 (3.3–3.6)	2.4 (2.3–2.6)	2.2 (2.0–2.5)
Côte d'Ivoire	17.9 (15.5–20.5)	14.7 (12.9–16.6)	11.1 (9.3–13.3)	8.2 (5.9–10.6)	11.3 (6.1–17.0)	10.0 (5.8–14.2)	8.4 (4.5–12.7)	7.0 (1.2–13.2)	12.1 (9.5–14.9)	12.4 (10.0–14.9)	8.9 (6.9–11.6)	6.5 (4.1–9.7)	18.0 (12.2–24.3)	18.3 (12.8–25.4)	11.4 (6.8–16.3)	8.3 (2.0–16.8)
Democratic People's Republic of Korea	4.5 (3.5–5.7)	10.3 (8.0–13.1)	2.5 (1.9–3.2)	2.0 (1.5–2.5)	3.4 (2.6–4.3)	6.3 (4.9–8.1)	2.1 (1.7–2.7)	1.8 (1.4–2.3)	6.3 (5.0–8.1)	10.4 (8.1–13.3)	4.4 (3.5–5.7)	3.9 (3.0–5.0)	7.9 (6.2–10.1)	12.4 (9.7–15.9)	5.7 (4.5–7.3)	5.0 (3.9–6.5)
Democratic Republic of the Congo	25.7 (21.2–31.3)	27.5 (23.7–32.3)	13.0 (11.1–15.3)	9.9 (7.4–12.5)	11.5 (1.0–24.9)	15.4 (7.1–24.5)	8.9 (4.9–13.1)	7.9 (2.6–13.8)	20.4 (13.3–28.2)	20.3 (16.4–25.1)	13.7 (11.1–16.7)	13.9 (9.8–19.1)	26.0 (7.0–62.9)	25.0 (16.3–35.4)	16.9 (9.9–23.1)	16.9 (7.0–31.5)
Denmark	0.9 (0.9–1.0)	0.6 (0.6–0.7)	0.3 (0.3–0.3)	0.3 (0.2–0.3)	0.9 (0.8–1.1)	0.7 (0.6–0.8)	0.4 (0.3–0.4)	0.3 (0.2–0.4)	2.3 (2.2–2.4)	2.1 (2.0–2.2)	1.0 (0.9–1.0)	0.9 (0.8–1.0)	3.1 (2.9–3.3)	2.7 (2.5–2.9)	1.5 (1.3–1.6)	1.3 (1.1–1.6)
Djibouti	16.2 (12.6–20.7)	13.7 (10.7–17.5)	8.9 (6.9–11.3)	6.5 (5.1–8.4)	9.4 (7.3–12.1)	8.3 (6.4–10.6)	6.0 (4.6–7.6)	4.7 (3.7–6.0)	17.2 (13.4–22.0)	15.5 (12.0–19.7)	11.6 (9.0–14.8)	9.5 (7.4–12.1)	24.3 (18.9–31.2)	22.1 (17.1–28.3)	17.1 (13.3–21.9)	14.3 (11.1–18.4)
Dominica	11.5 (9.3–12.9)	8.2 (7.3–9.4)	3.0 (2.1–4.1)	1.5 (0.6–2.3)	1.7 (0.0–5.4)	1.9 (0.2–3.4)	1.6 (0.1–3.3)	1.1 (0.0–3.0)	5.0 (3.3–6.8)	3.9 (3.2–4.6)	2.7 (2.0–3.5)	2.9 (1.7–4.8)	4.7 (0.7–10.3)	5.3 (3.8–6.9)	6.0 (4.4–7.8)	7.4 (3.1–14.3)
Dominican Republic	4.1 (3.5–4.6)	2.7 (2.3–3.2)	1.8 (1.3–2.4)	1.4 (0.9–2.0)	2.9 (1.6–4.2)	2.3 (1.3–3.2)	1.9 (0.8–3.4)	1.6 (0.4–3.8)	6.2 (4.0–8.7)	7.1 (5.9–8.7)	4.8 (3.2–7.2)	3.6 (1.8–6.4)	6.8 (1.8–12.3)	8.9 (5.9–12.2)	8.7 (3.2–15.8)	7.8 (1.5–24.2)
Ecuador	3.8 (3.7–3.8)	2.5 (2.5–2.5)	1.6 (1.5–1.6)	1.5 (1.5–1.6)	3.5 (3.3–3.7)	2.6 (2.5–2.8)	1.9 (1.8–2.0)	2.0 (1.8–2.2)	5.9 (5.7–6.1)	5.4 (5.3–5.6)	4.3 (4.2–4.4)	7.5 (7.2–7.9)	8.5 (7.9–9.0)	8.1 (7.7–8.6)	6.5 (6.2–6.7)	13.7 (12.3–15.1)
Egypt	6.0 (5.9–6.1)	2.9 (2.9–3.0)	2.1 (2.1–2.2)	1.7 (1.6–1.8)	5.1 (4.9–5.4)	2.7 (2.6–2.8)	2.1 (2.0–2.2)	1.8 (1.4–2.1)	6.2 (6.1–6.4)	4.0 (3.9–4.1)	3.7 (3.6–3.8)	3.7 (3.3–4.1)	6.8 (6.5–7.1)	4.4 (4.2–4.6)	4.2 (4.0–4.4)	4.4 (3.0–6.3)
El Salvador	3.0 (2.9–3.1)	1.6 (1.5–1.6)	1.2 (1.1–1.2)	1.5 (1.3–1.8)	3.1 (2.9–3.3)	2.0 (1.9–2.1)	2.1 (2.0–2.2)	3.2 (2.3–4.5)	9.9 (9.6–10.2)	6.5 (6.3–6.6)	7.9 (7.7–8.1)	3.6 (2.7–4.6)	17.8 (16.9–18.6)	10.9 (10.5–11.3)	9.5 (9.1–9.9)	6.7 (3.4–12.6)
Equatorial Guinea	22.8 (17.8–29.3)	19.6 (15.3–25.2)	11.0 (8.6–14.1)	7.8 (6.1–10.1)	12.0 (9.3–15.5)	10.9 (8.5–14.1)	7.5 (5.9–9.7)	6.0 (4.7–7.7)	18.8 (14.7–24.2)	17.2 (13.5–22.1)	12.2 (9.5–15.6)	9.9 (7.8–12.7)	22.2 (17.3–28.6)	20.5 (16.0–26.5)	15.2 (11.9–19.6)	12.8 (10.0–16.4)
Eritrea	30.0 (24.7–35.6)	13.6 (11.6–16.0)	4.2 (2.8–5.8)	3.1 (1.7–4.6)	12.5 (3.1–23.4)	7.5 (2.7–12.8)	3.5 (0.0–11.4)	2.8 (0.0–11.6)	23.2 (18.1–29.6)	16.4 (12.8–20.9)	9.2 (7.1–11.7)	7.4 (5.8–9.5)	35.8 (27.8–45.9)	25.5 (19.8–32.7)	13.9 (10.9–17.8)	11.6 (9.0–14.8)
Estonia	2.5 (2.4–2.6)	1.6 (1.5–1.7)	0.7 (0.6–0.7)	0.5 (0.4–0.6)	2.3 (2.0–2.6)	1.6 (1.3–1.8)	0.8 (0.6–0.9)	0.6 (0.4–0.9)	5.4 (5.1–5.6)	4.0 (3.8–4.3)	2.0 (1.8–2.2)	2.0 (1.6–2.4)	8.0 (7.4–8.6)	7.6 (7.0–8.2)	3.2 (2.7–3.6)	2.4 (1.6–3.3)
Eswatini	9.0 (4.6–10.9)	8.4 (6.5–10.7)	5.5 (3.7–8.4)	3.6 (1.5–7.6)	2.4 (0.0–12.2)	4.2 (0.8–7.6)	7.3 (2.7–12.4)	7.9 (1.5–16.8)	11.5 (9.0–14.7)	9.1 (7.1–11.7)	9.3 (7.2–11.9)	8.5 (6.6–10.9)	16.9 (13.2–21.7)	13.7 (10.7–17.7)	14.0 (10.9–18.0)	12.9 (10.1–16.6)
Ethiopia	51.5 (42.0–61.3)	27.4 (24.3–31.2)	6.6 (5.2–8.2)	4.2 (2.7–5.6)	22.6 (4.5–42.2)	14.1 (7.6–21.2)	4.7 (1.3–8.6)	3.3 (0.0–8.5)	29.2 (23.9–36.3)	20.2 (17.2–23.4)	9.0 (6.2–12.1)	6.7 (3.7–10.0)	43.7 (29.2–60.5)	25.2 (18.5–32.5)	9.3 (3.0–16.7)	7.8 (0.4–26.3)
Fiji	6.8 (6.1–7.6)	4.3 (4.0–4.6)	2.2 (2.0–2.4)	2.2 (1.8–2.5)	5.6 (2.1–10.2)	4.0 (3.3–4.7)	2.6 (2.2–2.9)	2.9 (1.8–4.3)	6.9 (3.1–9.8)	5.8 (5.2–6.3)	4.0 (3.7–4.3)	3.7 (2.8–4.7)	10.0 (1.0–79.7)	8.2 (7.0–9.3)	6.1 (5.5–6.7)	5.9 (3.0–10.9)

Country	Probability of dying $_5q_5$ (per 1,000 children aged 5)				Probability of dying $_5q_{10}$ (per 1,000 children aged 10)				Probability of dying $_5q_{15}$ (per 1,000 youths aged 15)				Probability of dying $_5q_{20}$ (per 1,000 youths aged 20)			
	1990	2000	2015	2024	1990	2000	2015	2024	1990	2000	2015	2024	1990	2000	2015	2024
Finland	0.9 (0.9–1.0)	0.7 (0.7–0.7)	0.4 (0.4–0.4)	0.4 (0.3–0.4)	0.9 (0.8–1.0)	0.7 (0.6–0.8)	0.4 (0.4–0.5)	0.4 (0.3–0.6)	3.4 (3.4–3.6)	2.4 (2.3–2.5)	1.5 (1.5–1.6)	1.7 (1.6–1.8)	4.8 (4.4–5.2)	3.7 (3.4–4.0)	2.5 (2.3–2.8)	2.8 (2.5–3.2)
France	1.0 (1.0–1.0)	0.7 (0.6–0.7)	0.4 (0.4–0.4)	0.4 (0.4–0.4)	1.0 (1.0–1.0)	0.7 (0.7–0.7)	0.4 (0.4–0.4)	0.4 (0.3–0.5)	2.8 (2.7–2.8)	2.3 (2.2–2.3)	1.1 (1.1–1.1)	1.1 (1.0–1.1)	4.9 (4.7–5.1)	3.6 (3.5–3.7)	2.0 (1.9–2.1)	2.1 (1.9–2.3)
Gabon	9.9 (6.2–14.5)	8.7 (6.8–11.2)	5.7 (3.6–8.3)	4.3 (2.2–6.6)	9.3 (1.5–18.6)	7.8 (3.7–12.9)	5.0 (0.5–10.4)	3.9 (0.0–11.1)	8.9 (5.7–13.7)	10.6 (8.1–13.9)	6.0 (3.7–9.0)	4.6 (2.2–7.7)	13.7 (5.6–23.2)	12.9 (7.2–19.9)	6.6 (1.1–13.2)	5.9 (0.0–20.4)
Gambia	21.3 (16.7–27.3)	13.8 (10.8–17.7)	6.7 (5.2–8.6)	4.6 (3.6–5.9)	11.5 (8.9–14.8)	8.7 (6.8–11.2)	5.3 (4.2–6.9)	4.1 (3.2–5.3)	18.1 (14.2–23.2)	13.9 (10.9–17.9)	9.0 (7.1–11.6)	7.2 (5.7–9.3)	21.4 (16.7–27.6)	17.1 (13.3–22.0)	11.7 (9.2–15.1)	9.7 (7.6–12.5)
Georgia	1.7 (1.6–1.7)	1.1 (1.0–1.1)	1.2 (1.1–1.2)	0.7 (0.7–0.8)	1.4 (1.2–1.6)	1.0 (0.9–1.1)	1.3 (1.2–1.5)	0.9 (0.7–1.1)	2.8 (2.7–3.0)	2.3 (2.1–2.4)	2.9 (2.7–3.1)	2.4 (2.1–2.6)	4.8 (4.5–5.2)	3.6 (3.3–3.9)	4.3 (3.8–4.8)	3.4 (2.9–4.0)
Germany	1.0 (1.0–1.0)	0.6 (0.6–0.6)	0.4 (0.4–0.4)	0.4 (0.4–0.5)	0.9 (0.9–1.0)	0.7 (0.6–0.7)	0.4 (0.4–0.5)	0.5 (0.4–0.5)	2.8 (2.8–2.9)	2.3 (2.2–2.3)	1.2 (1.1–1.2)	1.2 (1.2–1.3)	3.6 (3.5–3.8)	3.0 (2.9–3.1)	1.6 (1.6–1.7)	1.6 (1.4–1.8)
Ghana	15.9 (14.0–18.1)	11.6 (10.5–12.8)	7.0 (6.0–8.0)	5.4 (4.2–6.7)	8.9 (5.0–13.1)	7.3 (4.9–10.1)	5.2 (3.2–7.4)	4.5 (1.6–8.9)	12.2 (6.4–20.3)	8.8 (6.4–12.1)	7.7 (4.4–12.1)	6.2 (2.8–10.8)	16.6 (1.9–39.2)	13.1 (6.9–20.0)	9.1 (1.1–19.7)	8.2 (0.0–29.4)
Greece	0.9 (0.9–1.0)	0.7 (0.7–0.7)	0.4 (0.4–0.5)	0.3 (0.3–0.3)	1.0 (0.9–1.0)	0.8 (0.7–0.8)	0.5 (0.4–0.5)	0.4 (0.3–0.4)	2.7 (2.7–2.8)	2.4 (2.4–2.5)	1.3 (1.2–1.3)	1.3 (1.2–1.4)	4.1 (3.9–4.2)	3.6 (3.4–3.8)	2.1 (2.0–2.3)	2.1 (1.7–2.4)
Grenada	2.2 (1.7–2.8)	1.8 (1.5–2.1)	1.8 (1.4–2.3)	1.9 (1.2–2.7)	1.6 (0.6–2.7)	1.5 (0.9–2.2)	2.2 (1.3–3.0)	2.6 (1.0–4.9)	3.2 (2.4–4.3)	3.0 (2.5–3.5)	2.7 (2.2–3.4)	2.3 (1.5–3.2)	5.6 (3.8–7.8)	4.0 (3.0–5.1)	3.1 (2.0–4.2)	2.4 (0.5–5.4)
Guatemala	7.9 (7.8–8.1)	3.5 (3.4–3.5)	2.0 (1.9–2.0)	1.7 (1.7–1.8)	5.5 (5.2–5.8)	3.0 (2.9–3.1)	2.3 (2.2–2.4)	2.3 (2.1–2.5)	8.3 (8.1–8.4)	6.9 (6.8–7.1)	6.5 (6.4–6.7)	5.3 (5.0–5.5)	12.8 (12.3–13.3)	11.6 (11.1–12.0)	8.9 (8.6–9.3)	8.5 (7.6–9.5)
Guinea	28.6 (24.8–33.7)	20.5 (18.2–23.1)	13.6 (11.0–16.1)	10.7 (7.6–13.3)	15.1 (6.7–24.9)	10.6 (5.9–15.4)	7.3 (2.7–12.6)	5.9 (0.5–15.2)	17.5 (13.5–22.4)	18.0 (15.2–21.2)	15.1 (10.3–20.9)	11.9 (6.4–18.6)	15.2 (7.0–23.9)	17.3 (11.8–24.1)	18.6 (6.9–32.5)	17.2 (2.6–53.9)
Guinea-Bissau	36.9 (13.5–42.3)	23.2 (18.2–27.4)	9.7 (7.0–13.0)	6.1 (3.3–9.2)	7.2 (0.0–90.0)	7.5 (0.0–19.1)	6.9 (1.6–12.9)	5.6 (0.0–14.4)	22.1 (17.2–28.4)	18.7 (14.5–24.1)	12.1 (9.4–15.6)	9.9 (7.7–12.7)	25.3 (19.6–32.7)	21.9 (17.0–28.3)	15.1 (11.7–19.4)	12.6 (9.8–16.3)
Guyana	2.7 (2.5–2.9)	2.0 (1.9–2.1)	1.9 (1.7–2.0)	1.3 (1.1–1.5)	2.7 (2.3–3.2)	2.3 (2.1–2.6)	2.6 (2.3–3.0)	2.0 (1.3–3.0)	5.5 (5.1–6.0)	5.3 (5.0–5.7)	6.5 (6.1–6.9)	5.0 (3.9–6.2)	9.6 (8.5–10.7)	10.5 (9.4–11.6)	10.1 (9.0–11.1)	7.3 (2.5–17.5)
Haiti	17.3 (15.0–20.0)	12.0 (10.4–13.9)	6.8 (5.2–8.7)	5.1 (3.4–6.8)	10.5 (5.5–16.5)	8.0 (4.5–11.8)	5.0 (1.2–9.5)	4.0 (0.2–10.1)	17.8 (12.8–24.3)	11.7 (9.4–14.4)	7.2 (4.9–10.5)	6.5 (3.4–10.9)	22.5 (11.5–34.9)	16.5 (11.4–22.0)	11.8 (6.1–18.5)	12.6 (3.1–32.6)
Honduras	5.4 (4.6–6.5)	3.1 (2.7–3.6)	2.1 (1.5–2.8)	1.7 (1.0–2.5)	3.4 (1.4–5.3)	2.7 (1.5–3.9)	3.1 (1.6–4.9)	3.0 (1.0–6.8)	7.8 (6.1–10.0)	6.2 (4.9–7.9)	4.5 (3.5–5.8)	3.8 (3.0–4.9)	11.4 (9.0–14.7)	9.1 (7.1–11.7)	6.7 (5.2–8.6)	5.7 (4.5–7.3)
Hungary	1.2 (1.2–1.3)	0.9 (0.8–0.9)	0.5 (0.5–0.6)	0.4 (0.4–0.5)	1.3 (1.2–1.4)	1.0 (0.9–1.1)	0.6 (0.6–0.7)	0.5 (0.4–0.6)	3.4 (3.3–3.5)	2.1 (2.1–2.2)	1.4 (1.3–1.5)	1.2 (1.1–1.3)	4.9 (4.7–5.1)	3.1 (2.9–3.3)	2.0 (1.8–2.1)	2.1 (1.7–2.4)
Iceland	1.0 (0.9–1.1)	0.6 (0.6–0.8)	0.3 (0.2–0.4)	0.2 (0.1–0.2)	0.9 (0.6–1.2)	0.7 (0.5–1.0)	0.4 (0.3–0.6)	0.3 (0.1–0.6)	3.4 (3.0–3.8)	2.4 (2.1–2.7)	1.3 (1.1–1.6)	1.2 (0.8–1.7)	3.4 (2.6–4.2)	3.0 (2.3–3.7)	1.8 (1.3–2.3)	1.8 (0.9–2.9)
India	13.7 (13.0–14.3)	9.0 (8.7–9.4)	3.5 (3.3–3.6)	1.9 (1.7–2.1)	7.1 (5.8–8.3)	5.7 (4.9–6.5)	3.2 (2.8–3.6)	2.2 (1.6–2.8)	10.2 (9.7–10.8)	8.7 (8.3–9.1)	4.7 (4.5–5.0)	3.5 (3.1–4.0)	12.9 (11.3–14.5)	11.5 (10.3–12.8)	6.7 (6.0–7.4)	5.2 (3.3–7.8)
Indonesia	8.7 (7.9–9.5)	5.7 (5.1–6.4)	3.3 (2.7–4.0)	2.5 (1.8–3.2)	4.8 (3.2–6.5)	3.6 (2.4–4.9)	2.4 (1.1–4.1)	2.0 (0.5–4.7)	6.4 (5.3–7.8)	5.9 (5.0–7.0)	5.2 (3.6–7.1)	4.2 (2.2–6.3)	5.8 (3.4–8.4)	5.4 (3.6–7.5)	5.2 (1.2–12.0)	4.9 (0.3–19.4)

Country	Probability of dying $_5q_5$ (per 1,000 children aged 5) 1990	2000	2015	2024	Probability of dying $_5q_{10}$ (per 1,000 children aged 10) 1990	2000	2015	2024	Probability of dying $_5q_{15}$ (per 1,000 youths aged 15) 1990	2000	2015	2024	Probability of dying $_5q_{20}$ (per 1,000 youths aged 20) 1990	2000	2015	2024
Iran (Islamic Republic of)	7.3 (6.8–7.8)	2.5 (2.4–2.6)	2.1 (2.1–2.1)	1.5 (1.3–1.8)	6.3 (3.2–10.1)	2.3 (1.7–3.1)	2.1 (2.0–2.2)	1.7 (1.1–2.6)	8.9 (6.3–12.1)	3.9 (3.2–4.7)	4.4 (4.3–4.5)	4.7 (3.6–5.9)	11.6 (3.9–21.4)	6.3 (4.2–9.2)	5.4 (5.2–5.7)	5.8 (2.3–13.0)
Iraq	14.8 (12.0–18.0)	3.7 (3.0–4.4)	2.9 (2.1–3.8)	2.2 (1.4–3.2)	8.8 (0.5–20.4)	3.3 (1.6–5.0)	3.1 (0.9–7.0)	2.6 (0.4–8.1)	16.0 (12.5–20.4)	4.9 (3.9–6.3)	4.2 (3.3–5.3)	3.7 (2.9–4.7)	27.0 (21.0–34.7)	5.4 (4.2–6.9)	4.7 (3.6–6.0)	4.2 (3.3–5.4)
Ireland	1.0 (0.9–1.0)	0.7 (0.6–0.7)	0.3 (0.3–0.4)	0.3 (0.2–0.3)	1.0 (0.9–1.1)	0.8 (0.7–0.9)	0.4 (0.4–0.5)	0.3 (0.2–0.5)	2.5 (2.4–2.6)	2.8 (2.7–2.9)	1.2 (1.1–1.3)	1.1 (1.0–1.3)	3.6 (3.4–3.9)	4.1 (3.9–4.4)	2.1 (1.9–2.2)	1.5 (1.1–2.1)
Israel	1.0 (1.0–1.0)	0.8 (0.7–0.8)	0.5 (0.5–0.5)	0.4 (0.4–0.4)	1.0 (0.9–1.0)	0.8 (0.7–0.8)	0.5 (0.5–0.6)	0.4 (0.4–0.5)	2.2 (2.1–2.3)	2.0 (1.9–2.1)	1.2 (1.2–1.3)	1.2 (1.1–1.3)	3.0 (2.8–3.2)	3.0 (2.8–3.3)	1.8 (1.7–2.0)	1.8 (1.4–2.3)
Italy	0.9 (0.8–0.9)	0.6 (0.6–0.6)	0.4 (0.4–0.4)	0.3 (0.3–0.4)	1.0 (0.9–1.0)	0.7 (0.7–0.8)	0.5 (0.4–0.5)	0.5 (0.4–0.6)	2.7 (2.7–2.8)	2.1 (2.1–2.1)	1.1 (1.1–1.1)	1.0 (0.9–1.0)	3.6 (3.4–3.7)	3.0 (2.8–3.1)	1.6 (1.5–1.6)	1.4 (1.2–1.6)
Jamaica	2.1 (1.7–2.7)	1.7 (1.3–2.1)	1.5 (1.2–2.0)	1.5 (1.2–1.9)	2.3 (1.8–2.9)	1.9 (1.5–2.4)	1.8 (1.4–2.3)	1.7 (1.4–2.2)	5.3 (4.2–6.8)	4.5 (3.5–5.8)	4.3 (3.4–5.5)	4.2 (3.3–5.3)	7.9 (6.2–10.0)	6.7 (5.3–8.6)	6.4 (5.0–8.2)	6.3 (4.9–8.0)
Japan	0.9 (0.9–0.9)	0.6 (0.6–0.7)	0.4 (0.4–0.4)	0.4 (0.4–0.4)	0.7 (0.7–0.8)	0.6 (0.5–0.6)	0.4 (0.4–0.4)	0.5 (0.4–0.5)	2.1 (2.1–2.2)	1.7 (1.6–1.7)	1.0 (1.0–1.0)	1.2 (1.2–1.3)	2.7 (2.6–2.8)	2.3 (2.3–2.4)	1.8 (1.8–1.9)	1.9 (1.8–2.0)
Jordan	2.6 (2.3–2.8)	2.1 (2.0–2.2)	1.2 (1.2–1.3)	0.7 (0.6–0.7)	2.2 (1.3–3.4)	1.8 (1.5–2.2)	1.1 (1.0–1.2)	0.6 (0.5–0.7)	3.2 (2.3–4.4)	2.9 (2.5–3.2)	1.9 (1.9–2.0)	1.3 (1.2–1.4)	4.3 (2.1–7.0)	4.4 (3.5–5.4)	2.4 (2.3–2.5)	1.4 (1.2–1.7)
Kazakhstan	3.0 (3.0–3.1)	2.5 (2.4–2.5)	1.5 (1.5–1.5)	1.2 (1.2–1.3)	2.7 (2.5–2.8)	2.4 (2.3–2.5)	1.6 (1.5–1.6)	1.4 (1.2–1.5)	5.4 (5.3–5.5)	5.7 (5.7–5.9)	3.3 (3.2–3.4)	2.7 (2.6–2.9)	8.5 (8.2–8.9)	11.4 (11.0–11.7)	5.1 (4.9–5.2)	3.7 (3.3–4.2)
Kenya	11.0 (9.8–12.3)	10.2 (9.1–11.4)	4.5 (3.7–5.5)	2.4 (1.7–3.1)	5.3 (2.4–8.3)	5.9 (3.0–9.3)	3.4 (1.6–5.4)	2.0 (0.1–4.7)	8.7 (6.7–11.1)	10.4 (8.8–12.2)	7.0 (4.8–9.8)	5.4 (2.8–8.6)	14.0 (9.0–19.3)	17.0 (12.9–21.7)	10.2 (3.7–18.3)	9.1 (1.4–29.1)
Kiribati	9.4 (7.3–12.0)	7.0 (5.4–8.9)	6.3 (4.9–8.0)	5.7 (4.5–7.3)	6.0 (4.7–7.7)	4.8 (3.7–6.1)	4.4 (3.4–5.6)	4.1 (3.2–5.2)	9.9 (7.7–12.6)	8.2 (6.4–10.6)	7.7 (6.0–9.9)	7.3 (5.7–9.4)	11.9 (9.2–15.2)	10.1 (7.9–12.9)	9.5 (7.4–12.2)	9.0 (7.1–11.6)
Kosovo (UNSCR 1244)	4.1 (3.2–5.2)	2.6 (2.0–3.3)	1.3 (1.0–1.6)	0.9 (0.7–1.2)	3.3 (2.6–4.2)	2.3 (1.8–2.9)	1.3 (1.0–1.7)	1.0 (0.8–1.3)	5.0 (3.9–6.4)	3.9 (3.1–5.0)	2.7 (2.1–3.4)	2.3 (1.8–2.9)	8.1 (6.3–10.3)	6.2 (4.8–7.9)	4.1 (3.2–5.2)	3.4 (2.7–4.4)
Kuwait	2.9 (2.7–3.1)	1.3 (1.3–1.4)	0.9 (0.9–1.0)	0.8 (0.7–0.9)	2.4 (2.0–2.9)	1.3 (1.1–1.5)	1.0 (0.8–1.1)	0.9 (0.6–1.2)	5.1 (4.6–5.6)	2.9 (2.7–3.1)	2.3 (2.2–2.5)	1.5 (1.4–1.8)	9.3 (8.1–10.6)	3.4 (3.1–3.8)	2.7 (2.4–3.0)	2.1 (1.6–2.6)
Kyrgyzstan	3.1 (3.1–3.2)	2.4 (2.3–2.4)	1.5 (1.4–1.5)	1.3 (1.2–1.3)	2.6 (2.4–2.7)	2.2 (2.1–2.3)	1.7 (1.6–1.8)	1.6 (1.4–1.8)	4.2 (4.1–4.3)	3.7 (3.6–3.8)	2.9 (2.8–3.0)	2.7 (2.6–2.9)	7.1 (6.8–7.5)	6.8 (6.5–7.0)	4.2 (4.0–4.5)	3.2 (2.7–3.7)
Lao People's Democratic Republic	28.2 (20.8–34.9)	14.2 (11.9–16.7)	4.2 (3.4–5.2)	2.1 (1.4–3.0)	15.0 (0.0–36.8)	9.6 (5.5–14.1)	4.2 (2.4–6.2)	2.7 (0.9–5.1)	14.7 (5.1–24.5)	13.6 (9.4–19.6)	5.7 (3.4–10.1)	4.2 (1.9–8.3)	12.9 (0.0–34.0)	11.6 (2.7–21.5)	7.7 (1.2–15.8)	6.7 (0.0–21.6)
Latvia	2.9 (2.8–3.0)	1.9 (1.8–2.0)	0.8 (0.8–0.9)	0.5 (0.4–0.6)	2.6 (2.3–2.9)	1.7 (1.4–1.9)	0.8 (0.6–1.0)	0.5 (0.3–0.8)	5.7 (5.5–6.0)	4.5 (4.2–4.7)	2.4 (2.3–2.6)	1.9 (1.6–2.1)	8.3 (7.7–8.8)	7.8 (7.4–8.4)	3.8 (3.3–4.2)	3.0 (2.4–3.6)
Lebanon	2.9 (2.3–3.7)	2.0 (1.6–2.5)	1.4 (1.1–1.8)	3.3 (2.6–4.2)	3.4 (2.7–4.4)	2.2 (1.7–2.8)	1.4 (1.1–1.8)	2.8 (2.2–3.6)	12.0 (9.3–15.3)	3.5 (2.8–4.5)	2.9 (2.3–3.7)	5.2 (4.0–6.6)	27.8 (21.6–35.6)	4.1 (3.2–5.2)	3.5 (2.7–4.4)	7.6 (5.9–9.7)
Lesotho	8.7 (7.2–10.4)	7.0 (6.0–8.2)	5.0 (4.0–6.3)	4.0 (2.7–5.3)	7.7 (3.5–12.3)	6.5 (4.0–9.0)	5.1 (2.9–7.5)	4.3 (1.5–8.3)	12.1 (7.9–18.8)	14.1 (11.7–16.9)	8.4 (6.3–11.4)	3.4 (1.8–5.9)	15.9 (1.5–37.7)	28.8 (22.5–36.2)	16.4 (11.1–22.1)	6.2 (1.1–13.3)
Liberia	19.2 (16.3–22.7)	16.7 (14.5–19.2)	11.7 (9.3–14.5)	8.5 (5.9–11.3)	12.7 (4.0–25.2)	12.0 (6.1–19.7)	9.4 (3.7–15.9)	7.4 (0.6–21.2)	33.5 (21.3–46.5)	14.1 (11.2–17.7)	14.1 (10.3–19.1)	11.1 (6.3–17.4)	78.3 (43.7–128.3)	20.0 (13.4–27.5)	17.6 (9.0–26.8)	14.4 (2.4–36.3)

Country	Probability of dying $_5q_5$ (per 1,000 children aged 5)				Probability of dying $_5q_{10}$ (per 1,000 children aged 10)				Probability of dying $_5q_{15}$ (per 1,000 youths aged 15)				Probability of dying $_5q_{20}$ (per 1,000 youths aged 20)			
	1990	2000	2015	2024	1990	2000	2015	2024	1990	2000	2015	2024	1990	2000	2015	2024
Libya	4.7 (3.4–6.5)	2.5 (1.7–3.9)	1.8 (0.7–3.4)	1.1 (0.3–2.1)	3.0 (0.1–6.6)	2.1 (0.1–4.2)	1.8 (0.0–6.5)	1.2 (0.0–5.6)	4.8 (3.8–6.2)	4.1 (3.2–5.2)	4.5 (3.5–5.7)	2.6 (2.1–3.4)	5.3 (4.1–6.8)	4.6 (3.6–5.8)	7.6 (5.9–9.7)	3.1 (2.4–4.0)
Lithuania	2.3 (2.3–2.4)	1.5 (1.4–1.6)	0.8 (0.8–0.9)	0.5 (0.5–0.6)	2.0 (1.8–2.2)	1.4 (1.3–1.6)	0.9 (0.8–1.0)	0.6 (0.4–0.8)	4.9 (4.7–5.1)	4.4 (4.3–4.6)	2.7 (2.5–2.9)	1.6 (1.4–1.8)	7.3 (6.9–7.7)	7.9 (7.5–8.3)	4.3 (3.9–4.7)	2.7 (2.2–3.2)
Luxembourg	0.9 (0.8–1.0)	0.4 (0.4–0.5)	0.2 (0.2–0.3)	0.2 (0.2–0.3)	0.8 (0.5–1.1)	0.4 (0.2–0.6)	0.3 (0.1–0.4)	0.3 (0.1–0.6)	4.2 (3.7–4.7)	1.9 (1.7–2.2)	1.0 (0.8–1.2)	0.8 (0.6–1.1)	5.5 (4.5–6.5)	3.4 (2.7–4.0)	1.3 (0.9–1.7)	0.8 (0.3–1.4)
Madagascar	22.9 (19.9–26.2)	15.1 (13.6–16.9)	10.1 (8.6–11.7)	11.6 (9.2–14.2)	14.2 (7.8–21.6)	9.5 (5.5–14.1)	6.4 (3.3–9.8)	7.3 (0.0–21.7)	14.1 (11.3–17.4)	12.1 (10.1–14.2)	9.5 (7.2–12.8)	7.3 (4.4–11.0)	17.0 (11.2–23.4)	14.1 (9.9–18.4)	10.9 (5.1–17.1)	9.4 (1.7–23.2)
Malawi	26.4 (23.8–29.2)	16.3 (14.9–17.7)	8.6 (7.5–9.8)	6.2 (4.8–7.6)	11.7 (6.7–16.8)	8.8 (5.9–11.6)	6.0 (3.8–8.4)	4.9 (2.0–9.0)	18.1 (15.1–21.4)	20.0 (17.4–22.8)	8.7 (6.9–10.9)	6.3 (3.9–9.2)	22.2 (15.1–30.0)	29.0 (22.2–36.6)	13.3 (8.4–19.0)	10.4 (2.6–25.9)
Malaysia	2.4 (2.3–2.5)	1.6 (1.5–1.6)	1.2 (1.2–1.2)	0.9 (0.9–0.9)	2.6 (2.4–2.8)	1.8 (1.7–1.9)	1.5 (1.4–1.5)	1.1 (1.0–1.3)	4.6 (4.3–5.0)	4.0 (3.9–4.1)	3.5 (3.4–3.5)	3.2 (3.0–3.5)	6.9 (5.8–8.2)	5.2 (4.9–5.5)	3.7 (3.5–3.8)	3.3 (2.7–3.9)
Maldives	5.5 (5.0–6.1)	2.9 (2.7–3.1)	1.2 (1.0–1.4)	0.5 (0.4–0.7)	3.5 (2.5–4.5)	2.4 (1.9–2.9)	1.5 (1.2–1.9)	0.8 (0.4–1.4)	5.4 (4.6–6.2)	2.5 (2.2–2.8)	2.7 (2.4–3.0)	2.0 (1.5–2.4)	6.9 (5.5–8.4)	3.5 (2.9–4.1)	2.5 (1.9–3.2)	1.3 (0.2–2.8)
Mali	27.9 (24.9–31.4)	21.9 (19.9–24.1)	14.7 (12.8–16.8)	10.2 (8.0–12.4)	12.7 (6.7–18.9)	11.3 (7.1–15.8)	9.5 (5.6–13.5)	7.7 (2.2–13.2)	17.9 (14.6–22.3)	14.1 (12.1–16.4)	10.0 (7.5–13.1)	9.1 (5.3–13.6)	18.1 (10.7–26.1)	15.7 (11.5–20.5)	11.9 (6.6–17.9)	12.3 (2.8–31.8)
Malta	0.5 (0.3–0.8)	0.5 (0.4–0.7)	0.4 (0.3–0.5)	0.3 (0.2–0.5)	0.7 (0.3–1.4)	0.7 (0.5–1.0)	0.6 (0.3–0.8)	0.5 (0.2–0.9)	2.0 (1.6–2.4)	1.9 (1.7–2.2)	1.1 (0.9–1.3)	0.9 (0.6–1.4)	2.5 (1.7–3.4)	2.4 (1.8–2.9)	1.6 (1.2–2.1)	1.6 (0.7–2.8)
Marshall Islands	5.2 (4.0–6.7)	4.7 (3.6–6.0)	3.9 (3.0–5.0)	3.0 (2.4–3.9)	3.8 (3.0–4.9)	3.5 (2.7–4.5)	3.0 (2.4–3.9)	2.5 (1.9–3.2)	6.9 (5.4–8.9)	6.5 (5.1–8.3)	5.8 (4.6–7.5)	5.0 (3.9–6.5)	8.6 (6.7–11.0)	8.1 (6.3–10.4)	7.3 (5.7–9.4)	6.4 (5.0–8.2)
Mauritania	13.2 (11.6–15.1)	9.9 (8.6–11.5)	5.6 (4.3–7.1)	4.0 (2.5–5.3)	6.9 (3.0–10.6)	5.3 (2.3–8.5)	3.5 (1.0–6.2)	2.7 (0.0–6.5)	9.2 (6.6–12.6)	6.1 (4.3–8.2)	6.2 (4.4–8.6)	6.1 (3.4–9.5)	11.5 (4.0–21.0)	7.9 (2.8–14.3)	7.4 (3.3–11.9)	7.4 (0.7–20.5)
Mauritius	1.6 (1.4–1.8)	1.3 (1.2–1.4)	0.9 (0.8–1.0)	0.8 (0.6–0.9)	1.9 (1.6–2.2)	1.5 (1.3–1.7)	1.1 (0.9–1.3)	0.9 (0.7–1.2)	3.5 (3.2–3.9)	2.9 (2.7–3.1)	2.9 (2.7–3.1)	3.1 (2.7–3.5)	4.9 (4.3–5.5)	4.1 (3.7–4.6)	4.6 (4.1–5.0)	4.5 (3.6–5.4)
Mexico	2.7 (2.6–2.7)	1.6 (1.5–1.6)	1.1 (1.1–1.1)	1.1 (1.1–1.1)	2.5 (2.4–2.7)	1.7 (1.7–1.8)	1.4 (1.4–1.5)	1.5 (1.4–1.7)	4.6 (4.5–4.7)	3.7 (3.7–3.8)	3.7 (3.6–3.7)	3.9 (3.7–4.1)	6.9 (6.6–7.2)	5.4 (5.2–5.7)	5.8 (5.5–6.0)	6.2 (5.4–7.0)
Micronesia (Federated States of)	5.3 (4.1–6.8)	4.3 (3.3–5.4)	3.3 (2.6–4.3)	2.6 (2.0–3.3)	3.9 (3.0–5.0)	3.3 (2.5–4.2)	2.7 (2.1–3.5)	2.2 (1.7–2.8)	7.0 (5.5–9.0)	6.1 (4.8–7.8)	5.3 (4.1–6.8)	4.6 (3.5–5.8)	8.7 (6.8–11.1)	7.7 (6.0–9.9)	6.8 (5.3–8.7)	5.9 (4.6–7.5)
Monaco	0.9 (0.7–1.1)	0.6 (0.5–0.8)	0.4 (0.3–0.5)	0.3 (0.3–0.4)	0.9 (0.7–1.2)	0.7 (0.5–0.9)	0.5 (0.4–0.6)	0.4 (0.3–0.5)	2.5 (2.0–3.3)	1.9 (1.5–2.5)	1.5 (1.2–1.9)	1.2 (1.0–1.6)	3.7 (2.9–4.8)	2.9 (2.2–3.6)	2.2 (1.7–2.8)	1.8 (1.4–2.3)
Mongolia	6.6 (6.4–6.8)	3.7 (3.6–3.7)	2.2 (2.1–2.2)	1.3 (1.3–1.4)	4.7 (3.9–5.5)	3.1 (2.9–3.4)	2.5 (2.3–2.7)	1.8 (1.5–2.1)	6.1 (5.6–6.6)	4.9 (4.8–5.1)	4.2 (4.1–4.4)	3.5 (3.2–3.9)	10.9 (9.5–12.4)	8.4 (8.0–8.8)	5.9 (5.5–6.3)	4.8 (4.0–5.6)
Montenegro	1.3 (1.1–1.5)	1.0 (0.9–1.1)	0.5 (0.4–0.7)	0.4 (0.3–0.6)	1.2 (0.8–1.8)	1.0 (0.8–1.3)	0.7 (0.5–0.9)	0.6 (0.3–0.9)	2.4 (2.0–2.8)	2.3 (2.1–2.5)	1.4 (1.2–1.6)	2.4 (1.9–2.9)	4.0 (3.3–4.8)	3.5 (3.0–4.0)	2.1 (1.7–2.5)	3.2 (2.2–4.2)
Montserrat	1.7 (1.3–2.1)	1.2 (1.0–1.6)	0.8 (0.6–1.0)	0.6 (0.5–0.8)	1.9 (1.5–2.4)	1.5 (1.2–1.9)	1.1 (0.9–1.4)	0.9 (0.7–1.2)	4.5 (3.5–5.8)	3.7 (2.9–4.7)	2.9 (2.2–3.6)	2.5 (1.9–3.1)	6.7 (5.2–8.6)	5.5 (4.3–7.0)	4.3 (3.3–5.5)	3.7 (2.9–4.7)
Morocco	5.6 (4.9–6.4)	3.4 (2.8–4.1)	1.8 (1.2–2.3)	1.3 (0.7–1.8)	3.7 (2.2–5.1)	2.2 (1.0–3.4)	1.1 (0.0–2.6)	0.9 (0.0–2.9)	5.2 (4.1–6.6)	3.7 (2.8–4.6)	2.5 (1.4–3.7)	1.9 (0.9–3.3)	6.6 (4.1–9.2)	4.6 (2.8–6.6)	3.1 (0.5–7.8)	2.8 (0.1–11.3)

Country	Probability of dying $_5q_5$ (per 1,000 children aged 5)				Probability of dying $_5q_{10}$ (per 1,000 children aged 10)				Probability of dying $_5q_{15}$ (per 1,000 youths aged 15)				Probability of dying $_5q_{20}$ (per 1,000 youths aged 20)			
	1990	2000	2015	2024	1990	2000	2015	2024	1990	2000	2015	2024	1990	2000	2015	2024
Mozambique	39.8 (30.3–49.1)	21.2 (18.2–24.6)	7.9 (6.3–9.8)	4.7 (3.2–6.4)	21.7 (2.3–44.3)	12.5 (6.8–18.7)	5.5 (2.2–9.0)	3.6 (0.3–8.0)	17.9 (13.4–23.5)	16.8 (13.8–20.5)	8.7 (6.5–11.3)	7.3 (4.6–11.4)	20.4 (10.5–30.9)	19.5 (13.2–26.8)	13.5 (8.5–18.7)	10.7 (3.3–20.4)
Myanmar	22.2 (14.1–28.4)	14.6 (10.6–19.0)	3.1 (2.1–4.6)	1.9 (0.9–3.0)	7.2 (0.0–26.5)	6.6 (0.0–26.4)	2.7 (0.2–5.9)	2.0 (0.0–6.0)	14.1 (9.0–22.3)	11.3 (7.7–16.9)	3.9 (2.7–5.7)	3.9 (2.0–6.7)	12.3 (0.3–28.0)	11.8 (2.2–30.2)	6.3 (2.7–10.5)	8.4 (1.9–24.4)
Namibia	8.7 (7.0–10.6)	7.9 (6.9–9.2)	6.5 (5.2–7.9)	3.6 (2.5–4.7)	5.8 (2.0–9.8)	5.7 (3.0–8.4)	5.3 (2.3–8.8)	3.2 (0.5–8.8)	11.1 (9.2–13.3)	11.5 (9.9–13.3)	8.6 (7.1–10.5)	5.9 (4.2–8.2)	16.0 (10.8–21.4)	18.8 (14.6–23.8)	15.5 (10.3–21.7)	11.4 (5.1–21.1)
Nauru	7.1 (5.5–9.1)	4.5 (3.5–5.8)	2.0 (1.6–2.6)	1.0 (0.8–1.3)	4.8 (3.7–6.2)	3.4 (2.6–4.4)	1.8 (1.4–2.3)	1.0 (0.8–1.3)	8.3 (6.5–10.7)	6.4 (5.0–8.2)	4.0 (3.1–5.1)	2.6 (2.1–3.4)	10.1 (7.9–13.0)	7.9 (6.2–10.2)	5.2 (4.0–6.6)	3.5 (2.8–4.6)
Nepal	18.1 (16.0–20.5)	8.8 (8.0–9.8)	4.9 (4.1–5.9)	2.3 (1.7–3.0)	8.2 (4.1–12.7)	4.9 (2.9–7.0)	3.9 (2.1–6.1)	2.0 (0.5–5.5)	10.0 (7.9–12.9)	6.7 (5.5–8.1)	6.0 (4.2–8.7)	3.9 (2.1–6.2)	10.0 (5.6–14.7)	8.4 (5.7–11.2)	7.3 (3.0–12.5)	5.5 (0.8–16.7)
Netherlands (Kingdom of the)	0.9 (0.9–0.9)	0.6 (0.6–0.7)	0.4 (0.3–0.4)	0.4 (0.3–0.4)	0.9 (0.9–1.0)	0.7 (0.7–0.8)	0.5 (0.4–0.5)	0.5 (0.4–0.6)	2.0 (1.9–2.0)	1.8 (1.7–1.8)	1.0 (1.0–1.0)	1.1 (1.0–1.1)	2.6 (2.5–2.7)	2.3 (2.2–2.5)	1.4 (1.3–1.5)	1.5 (1.4–1.7)
New Zealand	1.2 (1.2–1.2)	0.8 (0.8–0.9)	0.5 (0.4–0.5)	0.4 (0.3–0.4)	1.4 (1.2–1.5)	1.0 (0.9–1.1)	0.7 (0.6–0.7)	0.6 (0.4–0.8)	5.0 (4.8–5.2)	3.6 (3.4–3.7)	2.2 (2.1–2.3)	1.8 (1.5–2.1)	6.4 (6.0–6.8)	4.0 (3.7–4.4)	2.8 (2.6–3.1)	2.6 (1.7–3.9)
Nicaragua	4.6 (3.5–6.0)	3.0 (2.5–3.7)	1.5 (0.7–2.5)	1.1 (0.5–2.1)	2.7 (0.6–4.9)	2.8 (1.7–4.0)	2.4 (0.5–5.3)	1.9 (0.3–5.6)	8.9 (7.0–11.4)	6.2 (4.9–8.0)	4.1 (3.2–5.2)	3.7 (2.9–4.8)	13.9 (10.8–17.8)	9.1 (7.1–11.7)	6.0 (4.7–7.7)	5.5 (4.3–7.1)
Niger	45.8 (40.1–53.0)	33.6 (29.2–38.1)	21.6 (17.9–26.2)	17.8 (13.0–24.5)	18.4 (6.9–29.6)	17.7 (10.3–25.7)	14.3 (6.9–22.4)	12.2 (1.8–24.3)	19.0 (15.0–23.8)	16.1 (13.3–19.5)	11.7 (7.5–17.6)	9.1 (4.5–15.3)	19.6 (10.6–28.3)	19.5 (13.6–25.9)	16.9 (5.3–34.5)	15.3 (2.2–54.7)
Nigeria	24.5 (21.5–28.2)	19.9 (18.1–22.3)	14.1 (12.6–15.6)	13.5 (11.3–15.7)	12.8 (6.3–19.7)	10.7 (6.9–14.9)	8.9 (5.9–12.0)	8.8 (3.8–15.9)	17.2 (12.5–23.2)	12.3 (10.1–14.9)	8.2 (6.9–9.7)	6.9 (5.1–9.5)	20.4 (9.5–34.5)	16.2 (11.6–21.2)	11.0 (8.2–13.8)	8.7 (4.3–14.0)
Niue	2.9 (2.3–3.7)	3.7 (2.9–4.7)	3.5 (2.8–4.5)	2.7 (2.1–3.5)	2.4 (1.9–3.1)	2.9 (2.3–3.7)	2.8 (2.2–3.6)	2.3 (1.8–2.9)	4.9 (3.9–6.3)	5.6 (4.4–7.3)	5.5 (4.3–7.1)	4.7 (3.7–6.1)	6.2 (4.9–8.0)	7.1 (5.6–9.1)	6.9 (5.4–8.9)	6.0 (4.7–7.7)
North Macedonia	1.8 (1.7–1.9)	1.3 (1.2–1.3)	0.7 (0.6–0.8)	0.5 (0.5–0.6)	1.6 (1.3–1.9)	1.2 (1.0–1.4)	0.8 (0.7–0.9)	0.7 (0.5–0.9)	2.3 (2.2–2.5)	2.3 (2.2–2.4)	1.4 (1.4–1.5)	1.6 (1.4–1.8)	3.1 (2.8–3.5)	3.1 (2.8–3.4)	1.8 (1.6–2.1)	2.1 (1.7–2.7)
Norway	0.9 (0.9–1.0)	0.6 (0.6–0.7)	0.4 (0.3–0.4)	0.3 (0.3–0.3)	0.9 (0.8–1.0)	0.7 (0.6–0.7)	0.4 (0.4–0.5)	0.4 (0.3–0.5)	2.8 (2.7–2.9)	2.5 (2.4–2.6)	1.3 (1.2–1.3)	1.4 (1.2–1.5)	3.3 (3.1–3.6)	3.6 (3.3–3.9)	1.9 (1.7–2.1)	1.8 (1.5–2.1)
Oman	3.4 (2.7–4.3)	1.7 (1.4–2.2)	1.3 (1.0–1.6)	1.2 (0.9–1.5)	4.2 (3.3–5.3)	1.8 (1.4–2.3)	1.3 (1.0–1.6)	1.2 (0.9–1.5)	4.7 (3.7–6.0)	3.3 (2.6–4.2)	2.8 (2.2–3.5)	2.7 (2.1–3.4)	5.2 (4.1–6.6)	3.8 (3.0–4.9)	3.3 (2.6–4.2)	3.2 (2.5–4.1)
Pakistan	8.2 (7.8–8.5)	6.9 (6.4–7.3)	4.5 (3.8–5.2)	3.0 (2.3–3.7)	5.7 (4.8–6.5)	5.2 (4.0–6.5)	3.8 (2.2–5.9)	2.8 (0.6–5.7)	10.5 (9.1–12.0)	7.2 (6.2–8.1)	4.5 (3.4–6.2)	3.3 (2.0–5.3)	10.8 (7.7–14.4)	9.3 (7.1–11.6)	6.1 (3.0–9.4)	4.8 (1.3–9.4)
Palau	3.9 (3.1–5.0)	3.0 (2.3–3.8)	2.5 (1.9–3.1)	2.5 (1.9–3.2)	3.1 (2.4–4.0)	2.5 (1.9–3.2)	2.1 (1.7–2.7)	2.1 (1.7–2.7)	6.0 (4.1–8.7)	7.8 (5.9–10.5)	7.6 (3.7–12.7)	5.9 (2.3–10.9)	10.2 (5.9–14.6)	10.6 (6.1–15.2)	10.7 (0.9–34.6)	9.7 (0.5–46.4)
Panama	2.6 (2.5–2.7)	2.0 (2.0–2.1)	1.7 (1.6–1.8)	1.4 (1.3–1.5)	2.3 (1.9–2.6)	2.0 (1.8–2.1)	1.9 (1.8–2.1)	1.8 (1.4–2.1)	4.6 (4.3–4.9)	4.1 (4.0–4.3)	4.8 (4.6–4.9)	3.5 (3.2–3.9)	6.6 (5.9–7.3)	6.2 (5.9–6.6)	6.9 (6.5–7.3)	5.8 (4.5–7.4)
Papua New Guinea	8.8 (6.9–11.2)	7.6 (5.9–9.6)	6.8 (5.3–8.7)	4.3 (3.4–5.5)	5.7 (4.5–7.3)	5.1 (4.0–6.5)	4.8 (3.8–6.2)	3.3 (2.6–4.2)	9.5 (7.4–12.1)	8.7 (6.8–11.0)	7.2 (5.7–9.2)	6.2 (4.9–7.9)	11.4 (9.0–14.7)	10.5 (8.2–13.5)	8.9 (7.0–11.4)	7.8 (6.1–10.0)
Paraguay	2.6 (2.4–2.8)	1.5 (1.4–1.6)	1.1 (1.1–1.1)	0.8 (0.8–0.9)	2.1 (1.1–3.4)	1.5 (1.2–1.8)	1.5 (1.4–1.6)	1.3 (1.0–1.6)	6.9 (5.4–8.8)	5.9 (4.6–7.5)	4.7 (3.7–6.0)	4.0 (3.1–5.2)	10.0 (7.8–12.9)	8.6 (6.7–11.1)	6.9 (5.4–8.9)	5.9 (4.6–7.7)

Country	Probability of dying $_5q_5$ (per 1,000 children aged 5)				Probability of dying $_5q_{10}$ (per 1,000 children aged 10)				Probability of dying $_5q_{15}$ (per 1,000 youths aged 15)				Probability of dying $_5q_{20}$ (per 1,000 youths aged 20)			
	1990	2000	2015	2024	1990	2000	2015	2024	1990	2000	2015	2024	1990	2000	2015	2024
Peru	5.2 (4.8–5.6)	2.5 (2.4–2.6)	1.3 (1.3–1.4)	1.3 (1.1–1.5)	4.4 (3.1–5.8)	2.3 (1.8–2.8)	1.4 (1.3–1.5)	1.5 (1.0–2.3)	8.7 (7.5–10.0)	4.6 (4.1–5.2)	2.7 (2.6–2.7)	2.8 (2.1–3.6)	9.7 (7.1–12.6)	5.9 (4.6–7.4)	3.7 (3.6–3.9)	4.2 (1.8–8.9)
Philippines	5.2 (5.2–5.3)	3.1 (3.0–3.1)	2.3 (2.2–2.3)	2.1 (2.0–2.2)	3.9 (3.6–4.1)	2.6 (2.5–2.8)	2.2 (2.2–2.3)	2.3 (2.0–2.6)	5.3 (5.2–5.4)	4.1 (4.0–4.2)	3.8 (3.7–3.9)	4.3 (4.0–4.7)	8.5 (8.2–8.9)	6.5 (6.2–6.7)	6.0 (5.8–6.2)	6.1 (4.9–7.5)
Poland	1.4 (1.4–1.4)	0.9 (0.9–0.9)	0.5 (0.5–0.5)	0.4 (0.4–0.5)	1.4 (1.3–1.4)	1.0 (0.9–1.1)	0.6 (0.6–0.7)	0.6 (0.6–0.7)	3.5 (3.4–3.5)	2.7 (2.7–2.7)	1.9 (1.9–2.0)	1.8 (1.7–1.8)	5.1 (4.8–5.3)	3.9 (3.8–4.1)	2.9 (2.8–3.0)	2.8 (2.6–2.9)
Portugal	1.9 (1.9–2.0)	1.2 (1.2–1.2)	0.4 (0.4–0.4)	0.4 (0.4–0.4)	1.9 (1.8–2.0)	1.3 (1.2–1.4)	0.5 (0.4–0.6)	0.5 (0.4–0.6)	4.8 (4.7–4.9)	3.1 (3.0–3.2)	1.1 (1.0–1.1)	1.2 (1.1–1.4)	6.3 (6.1–6.6)	4.7 (4.5–5.0)	1.7 (1.6–1.9)	1.8 (1.6–2.1)
Qatar	1.8 (1.6–2.1)	1.4 (1.3–1.5)	0.9 (0.8–0.9)	0.8 (0.7–0.9)	2.1 (1.5–2.7)	1.5 (1.2–1.8)	0.9 (0.7–1.0)	0.7 (0.5–1.0)	3.6 (3.1–4.3)	3.6 (3.0–3.9)	2.5 (2.3–2.7)	1.8 (1.5–2.1)	3.3 (2.3–4.4)	3.7 (3.0–4.5)	2.0 (1.6–2.4)	1.5 (1.0–2.0)
Republic of Korea	2.6 (2.5–2.6)	1.2 (1.2–1.3)	0.4 (0.4–0.4)	0.4 (0.4–0.4)	1.9 (1.8–2.0)	1.0 (1.0–1.1)	0.5 (0.4–0.5)	0.5 (0.4–0.6)	3.8 (3.7–3.9)	2.4 (2.3–2.4)	1.1 (1.1–1.1)	1.2 (1.1–1.3)	5.0 (4.8–5.2)	3.1 (2.9–3.2)	1.6 (1.6–1.7)	1.9 (1.6–2.1)
Republic of Moldova	3.1 (3.0–3.2)	2.3 (2.2–2.3)	1.2 (1.2–1.3)	0.9 (0.8–1.0)	2.3 (2.1–2.6)	2.0 (1.8–2.2)	1.5 (1.3–1.6)	1.3 (1.0–1.5)	4.7 (4.5–4.9)	3.5 (3.4–3.6)	2.8 (2.7–2.9)	2.9 (2.6–3.1)	8.0 (7.6–8.5)	5.5 (5.2–5.8)	4.0 (3.7–4.3)	4.1 (3.6–4.6)
Romania	3.0 (2.9–3.1)	2.3 (2.2–2.4)	0.9 (0.9–1.0)	0.6 (0.6–0.7)	2.3 (2.1–2.5)	3.0 (2.8–3.2)	1.1 (1.0–1.2)	0.7 (0.6–0.8)	3.6 (3.5–3.7)	2.9 (2.8–3.0)	2.2 (2.1–2.2)	1.6 (1.5–1.7)	5.1 (4.8–5.3)	3.7 (3.5–3.9)	2.9 (2.7–3.1)	2.5 (2.3–2.7)
Russian Federation	2.8 (2.7–2.8)	2.3 (2.3–2.4)	1.1 (1.1–1.1)	0.7 (0.7–0.7)	2.4 (2.3–2.5)	2.3 (2.2–2.4)	1.4 (1.3–1.4)	1.1 (1.0–1.2)	5.6 (5.5–5.7)	7.2 (7.0–7.3)	3.5 (3.4–3.6)	3.5 (3.2–3.7)	8.6 (8.2–8.9)	14.3 (13.7–14.8)	6.2 (6.0–6.5)	11.1 (9.8–12.5)
Rwanda	39.8 (36.0–44.1)	34.3 (31.4–37.4)	7.3 (6.4–8.4)	2.4 (1.9–2.9)	21.6 (9.9–35.2)	21.0 (13.2–29.5)	5.3 (2.5–8.9)	1.9 (0.0–6.3)	31.3 (24.0–40.2)	23.3 (20.2–26.9)	5.9 (4.8–7.1)	3.7 (2.5–5.1)	35.7 (17.4–56.7)	28.9 (20.7–37.9)	7.8 (4.9–11.3)	5.3 (1.3–11.8)
Saint Kitts and Nevis	2.3 (1.8–2.9)	1.9 (1.5–2.4)	1.6 (1.2–2.0)	1.3 (1.1–1.7)	2.4 (1.9–3.1)	2.1 (1.6–2.7)	1.8 (1.4–2.4)	1.6 (1.3–2.1)	3.2 (1.9–4.7)	5.7 (4.7–6.9)	7.0 (5.1–9.3)	5.7 (3.1–9.0)	3.8 (0.8–8.1)	7.2 (5.0–9.6)	10.8 (6.6–15.6)	10.3 (2.7–28.3)
Saint Lucia	1.9 (1.5–2.3)	2.0 (1.8–2.3)	1.5 (1.2–1.9)	1.2 (0.8–1.6)	1.8 (1.0–2.8)	1.8 (1.2–2.4)	1.2 (0.6–1.8)	0.8 (0.1–1.9)	4.1 (3.4–5.0)	3.7 (3.2–4.2)	3.3 (2.9–3.8)	5.6 (4.1–7.4)	6.5 (4.9–8.3)	6.5 (5.4–7.6)	6.0 (4.9–7.2)	11.9 (8.1–16.3)
Saint Vincent and the Grenadines	2.2 (1.8–2.7)	2.0 (1.7–2.3)	1.6 (1.2–2.0)	1.1 (0.7–1.5)	1.4 (0.6–2.3)	1.9 (1.2–2.5)	2.6 (1.8–3.3)	2.5 (1.4–3.8)	3.7 (3.1–4.5)	3.4 (2.9–4.0)	4.7 (4.0–5.5)	3.8 (2.7–5.2)	5.1 (3.7–6.6)	6.7 (5.4–8.0)	7.2 (5.6–8.8)	5.6 (3.1–8.5)
Samoa	3.9 (1.7–4.8)	3.1 (2.3–3.9)	1.6 (1.1–2.5)	1.1 (0.5–1.9)	1.0 (0.0–6.5)	1.2 (0.0–2.8)	1.3 (0.2–2.5)	1.1 (0.0–2.8)	8.4 (2.3–16.9)	5.4 (3.3–7.8)	3.6 (2.3–5.3)	3.0 (1.5–4.9)	16.9 (0.0–66.3)	9.0 (4.4–14.6)	4.4 (1.5–7.8)	3.8 (0.1–11.7)
San Marino	1.3 (1.0–1.7)	0.6 (0.5–0.8)	0.2 (0.2–0.3)	0.2 (0.1–0.2)	1.4 (1.1–1.8)	0.7 (0.5–0.9)	0.3 (0.2–0.4)	0.2 (0.2–0.3)	3.6 (2.8–4.5)	2.0 (1.5–2.5)	1.0 (0.8–1.3)	0.7 (0.6–1.0)	5.2 (4.1–6.7)	2.9 (2.3–3.7)	1.5 (1.2–1.9)	1.1 (0.9–1.4)
Sao Tome and Principe	13.2 (10.4–16.9)	9.7 (7.6–12.4)	2.4 (1.9–3.0)	1.3 (1.0–1.7)	8.5 (6.6–10.9)	6.9 (5.4–8.8)	2.6 (2.0–3.3)	1.6 (1.3–2.1)	10.1 (2.6–19.0)	7.2 (4.4–11.5)	6.8 (3.7–10.8)	5.2 (2.2–9.6)	14.4 (0.0–81.7)	12.0 (4.4–21.2)	12.6 (3.3–26.6)	11.5 (1.7–38.2)
Saudi Arabia	3.7 (2.9–4.7)	2.2 (1.7–2.8)	1.1 (0.8–1.4)	0.8 (0.6–1.0)	4.6 (3.6–5.9)	2.4 (1.9–3.1)	1.0 (0.8–1.3)	0.7 (0.5–0.9)	7.4 (2.6–11.7)	6.1 (4.6–8.1)	4.9 (3.5–6.9)	4.0 (2.2–6.5)	6.9 (0.0–41.5)	6.0 (2.5–10.3)	5.3 (1.9–9.3)	4.9 (0.2–14.4)
Senegal	22.8 (20.9–25.0)	21.1 (19.6–22.8)	6.8 (6.1–7.6)	4.2 (3.3–5.1)	10.1 (5.0–15.5)	11.6 (7.5–16.4)	5.5 (3.8–7.4)	4.3 (1.4–9.7)	11.6 (9.2–14.5)	10.6 (9.0–12.4)	6.2 (5.0–7.7)	4.1 (2.7–5.7)	13.4 (8.0–18.8)	10.9 (7.6–14.7)	6.2 (3.8–8.6)	4.2 (1.1–8.1)
Serbia	1.6 (1.6–1.7)	1.2 (1.1–1.2)	0.6 (0.5–0.6)	0.5 (0.5–0.6)	1.5 (1.3–1.6)	1.2 (1.1–1.3)	0.7 (0.6–0.8)	0.8 (0.6–0.9)	3.0 (2.9–3.1)	2.8 (2.7–2.9)	1.5 (1.5–1.6)	1.7 (1.6–1.8)	4.1 (3.8–4.4)	4.1 (3.8–4.4)	2.3 (2.1–2.5)	2.5 (2.1–2.9)

Country	Probability of dying $_5q_5$ (per 1,000 children aged 5)				Probability of dying $_5q_{10}$ (per 1,000 children aged 10)				Probability of dying $_5q_{15}$ (per 1,000 youths aged 15)				Probability of dying $_5q_{20}$ (per 1,000 youths aged 20)			
	1990	2000	2015	2024	1990	2000	2015	2024	1990	2000	2015	2024	1990	2000	2015	2024
Seychelles	2.1 (1.7–2.6)	1.7 (1.4–2.1)	1.2 (0.9–1.6)	1.0 (0.6–1.4)	1.6 (0.6–2.8)	1.5 (0.9–2.2)	1.3 (0.5–2.4)	1.1 (0.1–3.0)	2.8 (2.1–3.8)	3.8 (3.2–4.5)	4.2 (3.2–5.4)	4.1 (2.4–5.8)	5.2 (3.4–7.2)	4.9 (3.5–6.3)	3.7 (1.7–6.0)	3.8 (0.0–11.7)
Sierra Leone	28.3 (17.8–35.9)	20.8 (16.9–24.8)	15.7 (13.2–18.6)	13.7 (9.9–17.5)	15.1 (0.0–86.4)	11.9 (4.4–20.7)	10.2 (5.3–15.6)	9.5 (1.3–22.8)	24.0 (11.8–39.6)	21.5 (16.5–27.3)	18.6 (14.5–23.4)	14.3 (8.1–21.0)	26.5 (4.3–54.2)	25.4 (15.4–35.3)	20.5 (12.1–29.1)	17.4 (2.9–41.2)
Singapore	1.0 (0.9–1.1)	0.6 (0.6–0.6)	0.3 (0.3–0.4)	0.3 (0.3–0.4)	1.3 (1.1–1.5)	0.8 (0.7–0.9)	0.5 (0.4–0.6)	0.5 (0.4–0.7)	2.4 (2.3–2.6)	1.6 (1.5–1.7)	1.0 (0.9–1.0)	1.2 (1.1–1.4)	3.5 (3.1–3.8)	2.4 (2.2–2.7)	1.4 (1.2–1.5)	1.4 (1.1–1.7)
Slovakia	1.3 (1.3–1.4)	1.1 (1.1–1.1)	0.7 (0.6–0.7)	0.5 (0.5–0.6)	1.2 (1.1–1.3)	1.1 (1.0–1.2)	0.7 (0.6–0.8)	0.6 (0.5–0.8)	2.9 (2.8–2.9)	2.3 (2.3–2.4)	1.8 (1.7–1.9)	1.5 (1.4–1.6)	4.0 (3.8–4.2)	3.4 (3.2–3.6)	2.6 (2.4–2.8)	2.3 (2.0–2.5)
Slovenia	1.2 (1.1–1.3)	0.8 (0.7–0.8)	0.4 (0.3–0.4)	0.3 (0.2–0.3)	1.1 (0.9–1.3)	0.8 (0.7–0.9)	0.5 (0.4–0.6)	0.4 (0.3–0.6)	3.4 (3.2–3.5)	2.8 (2.7–3.0)	1.4 (1.3–1.5)	1.0 (0.9–1.2)	5.2 (4.8–5.5)	4.1 (3.8–4.4)	2.1 (1.9–2.4)	1.4 (1.1–1.8)
Solomon Islands	4.2 (3.3–5.4)	3.4 (2.7–4.4)	2.8 (2.2–3.6)	2.3 (1.8–3.0)	3.2 (2.5–4.1)	2.7 (2.1–3.5)	2.4 (1.9–3.0)	2.0 (1.6–2.6)	6.1 (4.8–7.8)	5.4 (4.2–6.9)	4.8 (3.8–6.2)	4.3 (3.4–5.5)	7.6 (6.0–9.8)	6.8 (5.4–8.8)	6.2 (4.8–7.9)	5.5 (4.3–7.1)
Somalia	26.0 (20.3–33.3)	24.9 (19.4–31.9)	19.0 (14.8–24.4)	14.1 (11.0–18.1)	13.4 (10.4–17.3)	13.0 (10.1–16.8)	10.6 (8.3–13.7)	8.5 (6.6–11.0)	31.0 (24.2–39.7)	22.9 (17.9–29.4)	19.2 (15.0–24.6)	16.9 (13.2–21.7)	55.1 (42.7–71.4)	31.3 (24.3–40.5)	26.8 (20.8–34.6)	25.8 (20.1–33.3)
South Africa	5.1 (3.7–5.6)	6.4 (5.9–6.8)	3.7 (3.5–3.9)	2.4 (2.2–2.6)	2.3 (0.1–5.7)	3.9 (2.8–5.1)	3.5 (3.0–4.1)	3.0 (2.3–3.7)	7.8 (5.7–10.6)	9.7 (8.9–10.5)	7.6 (7.2–8.1)	7.1 (6.4–7.8)	13.2 (7.8–19.4)	25.9 (22.6–29.3)	14.6 (13.1–16.2)	11.6 (9.5–14.0)
South Sudan	89.7 (69.7–115.1)	31.3 (24.3–40.1)	51.1 (39.7–65.6)	13.5 (10.4–17.2)	74.9 (56.9–98.8)	19.1 (14.7–24.7)	31.4 (24.1–40.9)	8.2 (6.4–10.6)	87.5 (68.0–112.2)	29.3 (22.8–37.6)	36.2 (28.1–46.4)	15.3 (11.9–19.6)	114.4 (87.0–150.7)	39.6 (30.5–51.2)	56.8 (43.7–73.6)	21.9 (16.9–28.2)
Spain	1.1 (1.1–1.1)	0.8 (0.7–0.8)	0.4 (0.4–0.4)	0.4 (0.3–0.4)	1.1 (1.1–1.2)	0.8 (0.8–0.9)	0.4 (0.4–0.5)	0.4 (0.4–0.5)	3.3 (3.2–3.3)	2.3 (2.2–2.3)	0.9 (0.9–0.9)	0.9 (0.8–0.9)	5.1 (4.9–5.3)	3.0 (2.9–3.1)	1.3 (1.2–1.4)	1.4 (1.3–1.6)
Sri Lanka	3.6 (3.5–3.6)	2.3 (2.3–2.4)	1.1 (1.0–1.1)	0.7 (0.6–0.7)	3.0 (2.8–3.1)	2.3 (2.2–2.3)	1.3 (1.2–1.4)	0.9 (0.7–1.2)	8.5 (8.3–8.7)	6.2 (6.1–6.3)	2.2 (2.1–2.4)	1.5 (1.0–2.0)	16.5 (15.9–17.1)	11.5 (11.1–11.9)	3.2 (2.8–3.6)	2.4 (0.8–6.2)
State of Palestine	2.8 (2.2–3.6)	2.2 (1.8–2.6)	1.9 (1.3–2.6)	28.1 (16.2–42.9)	2.5 (1.1–4.1)	2.2 (1.4–3.2)	2.0 (0.7–3.7)	31.8 (4.6–79.0)	5.4 (4.2–6.8)	5.2 (4.0–6.6)	3.4 (2.6–4.4)	36.2 (28.1–46.2)	6.6 (5.1–8.4)	7.6 (5.9–9.8)	3.9 (3.1–5.0)	41.2 (31.7–53.2)
Sudan	18.1 (15.9–20.7)	11.6 (10.0–13.5)	5.5 (4.0–7.0)	8.6 (5.3–11.4)	8.1 (2.6–15.2)	5.8 (2.2–10.2)	3.4 (0.0–7.6)	4.9 (0.0–16.9)	18.8 (14.7–24.2)	15.8 (12.4–20.3)	11.4 (8.9–14.6)	15.4 (12.1–19.8)	26.3 (20.5–33.9)	22.6 (17.6–29.1)	16.8 (13.1–21.7)	27.3 (21.3–35.2)
Suriname	2.1 (1.8–2.3)	1.7 (1.6–1.8)	1.5 (1.4–1.7)	2.0 (1.8–2.3)	2.3 (1.8–2.9)	1.8 (1.5–2.2)	1.6 (1.3–1.9)	2.1 (1.4–2.9)	4.9 (4.3–5.5)	3.8 (3.5–4.1)	3.8 (3.5–4.1)	4.4 (3.7–5.1)	7.4 (6.3–8.7)	6.7 (5.9–7.4)	5.6 (4.9–6.3)	5.8 (4.2–7.5)
Sweden	0.7 (0.7–0.7)	0.5 (0.5–0.6)	0.3 (0.3–0.4)	0.3 (0.3–0.3)	0.7 (0.7–0.8)	0.6 (0.6–0.7)	0.4 (0.4–0.5)	0.4 (0.4–0.5)	2.2 (2.1–2.2)	1.7 (1.6–1.7)	1.2 (1.2–1.3)	1.0 (0.9–1.1)	3.0 (2.8–3.2)	2.5 (2.3–2.7)	2.1 (2.0–2.3)	1.8 (1.6–2.0)
Switzerland	1.0 (0.9–1.0)	0.6 (0.6–0.6)	0.4 (0.3–0.4)	0.3 (0.3–0.4)	1.0 (0.9–1.1)	0.7 (0.6–0.8)	0.4 (0.4–0.5)	0.3 (0.3–0.4)	3.2 (3.1–3.3)	2.1 (2.0–2.1)	1.1 (1.1–1.2)	1.1 (1.0–1.2)	5.3 (5.0–5.5)	3.2 (3.0–3.4)	1.5 (1.4–1.6)	1.5 (1.3–1.7)
Syrian Arab Republic	5.6 (5.4–5.8)	3.5 (3.4–3.5)	8.1 (7.3–8.8)	2.2 (1.6–2.8)	3.6 (2.2–5.3)	2.6 (2.4–2.7)	4.9 (3.1–7.3)	2.0 (0.8–4.7)	8.3 (6.4–10.2)	3.6 (3.5–3.7)	15.2 (12.6–17.9)	3.0 (1.9–4.2)	5.8 (1.3–14.0)	3.5 (3.4–3.7)	34.1 (19.2–58.6)	4.7 (1.0–16.1)
Tajikistan	3.5 (3.5–3.6)	2.3 (2.2–2.3)	1.3 (1.2–1.3)	0.9 (0.8–0.9)	2.7 (2.5–2.9)	2.0 (1.9–2.1)	1.3 (1.1–1.4)	0.9 (0.8–1.1)	3.6 (3.4–3.7)	3.5 (3.4–3.6)	2.1 (1.9–2.2)	1.6 (1.3–1.8)	5.6 (5.3–5.9)	6.3 (6.0–6.6)	3.0 (2.7–3.4)	2.4 (1.6–3.4)
Thailand	3.6 (3.5–3.6)	3.2 (3.2–3.2)	2.0 (2.0–2.0)	1.6 (1.6–1.7)	3.0 (2.7–3.2)	2.9 (2.8–3.0)	2.5 (2.4–2.6)	2.7 (2.5–2.9)	7.2 (6.9–7.4)	6.7 (6.6–6.8)	6.2 (6.1–6.3)	4.8 (4.5–5.0)	9.2 (8.6–9.8)	11.8 (11.3–12.2)	6.8 (6.5–7.1)	6.6 (5.9–7.4)

Country	Probability of dying $_5q_5$ (per 1,000 children aged 5) 1990	2000	2015	2024	Probability of dying $_5q_{10}$ (per 1,000 children aged 10) 1990	2000	2015	2024	Probability of dying $_5q_{15}$ (per 1,000 youths aged 15) 1990	2000	2015	2024	Probability of dying $_5q_{20}$ (per 1,000 youths aged 20) 1990	2000	2015	2024
Timor-Leste	29.1 (22.8–37.1)	11.3 (8.9–14.4)	6.4 (5.0–8.2)	5.2 (4.0–6.6)	17.0 (13.3–21.9)	6.8 (5.4–8.8)	4.5 (3.5–5.7)	3.8 (3.0–4.8)	13.5 (7.1–22.4)	9.1 (6.9–12.2)	12.0 (8.7–16.0)	13.9 (7.6–20.5)	17.5 (0.0–50.4)	12.2 (6.1–19.9)	11.8 (3.5–22.3)	14.7 (0.0–64.9)
Togo	24.9 (20.9–29.8)	17.5 (15.2–20.6)	8.1 (6.2–10.4)	5.6 (3.5–7.7)	8.7 (1.3–16.4)	7.8 (2.8–13.9)	5.8 (2.1–9.9)	4.7 (0.6–10.6)	11.7 (9.3–14.8)	12.5 (10.1–15.6)	8.4 (5.6–11.8)	6.4 (3.3–10.2)	12.3 (6.4–18.8)	13.1 (7.3–20.5)	10.4 (3.0–20.0)	9.4 (1.0–32.1)
Tonga	1.5 (0.9–2.2)	2.6 (2.2–3.0)	1.7 (1.3–2.2)	1.1 (0.7–1.4)	1.5 (0.4–2.9)	2.0 (1.3–2.7)	1.0 (0.3–1.8)	0.6 (0.0–1.7)	2.3 (1.5–3.5)	3.8 (3.2–4.3)	3.3 (2.7–4.1)	2.7 (1.6–4.1)	4.4 (2.0–7.6)	4.8 (3.6–6.1)	5.3 (4.0–6.6)	5.4 (2.0–11.5)
Trinidad and Tobago	2.0 (1.8–2.1)	1.6 (1.5–1.7)	1.2 (1.1–1.3)	1.0 (0.8–1.2)	2.0 (1.7–2.3)	1.8 (1.6–2.0)	1.5 (1.2–1.7)	1.3 (0.7–2.0)	4.1 (3.8–4.3)	4.2 (4.0–4.3)	4.4 (4.2–4.7)	4.9 (3.8–6.1)	6.8 (6.1–7.5)	7.1 (6.5–7.6)	7.7 (7.0–8.4)	9.2 (4.6–16.9)
Tunisia	3.8 (3.7–3.9)	2.4 (2.3–2.5)	1.7 (1.7–1.8)	1.4 (1.3–1.4)	3.0 (2.3–3.7)	2.1 (1.9–2.2)	1.7 (1.6–1.9)	1.5 (1.2–1.7)	3.7 (3.1–4.3)	3.2 (3.0–3.3)	2.7 (2.5–2.8)	3.1 (2.9–3.4)	4.9 (3.1–7.2)	4.2 (3.9–4.5)	4.0 (3.7–4.3)	3.6 (2.7–4.5)
Turkmenistan	3.6 (3.5–3.7)	3.6 (3.5–3.7)	2.1 (2.1–2.2)	1.8 (1.5–2.2)	2.9 (2.6–3.1)	3.1 (2.9–3.3)	2.1 (1.8–2.3)	1.9 (1.0–3.3)	4.6 (4.4–4.8)	5.8 (5.6–6.1)	4.1 (3.9–4.4)	3.2 (2.1–4.5)	7.0 (6.6–7.4)	10.5 (10.1–10.9)	5.9 (5.5–6.4)	5.1 (1.8–13.0)
Turks and Caicos Islands	1.7 (1.3–2.1)	1.1 (0.8–1.4)	0.6 (0.5–0.8)	0.5 (0.4–0.6)	1.9 (1.5–2.4)	1.4 (1.1–1.7)	0.9 (0.7–1.2)	0.8 (0.6–1.0)	4.6 (3.6–5.8)	3.4 (2.6–4.3)	2.4 (1.9–3.1)	2.1 (1.6–2.7)	6.7 (5.3–8.6)	5.0 (3.9–6.5)	3.6 (2.8–4.7)	3.1 (2.5–4.0)
Tuvalu	5.8 (4.5–7.4)	4.7 (3.7–6.0)	3.0 (2.3–3.8)	2.2 (1.7–2.9)	4.1 (3.2–5.3)	3.5 (2.8–4.5)	2.5 (1.9–3.2)	2.0 (1.5–2.5)	7.4 (5.8–9.5)	6.5 (5.1–8.3)	4.9 (3.9–6.4)	4.2 (3.3–5.4)	9.1 (7.1–11.8)	8.1 (6.4–10.5)	6.3 (5.0–8.2)	5.4 (4.3–7.0)
Türkiye	5.9 (5.6–6.2)	4.4 (4.2–4.6)	1.3 (1.2–1.3)	0.9 (0.9–0.9)	2.5 (0.9–4.4)	2.6 (1.5–3.7)	1.2 (1.1–1.2)	1.1 (1.1–1.2)	9.5 (4.1–13.6)	5.3 (3.9–6.7)	2.4 (2.4–2.5)	2.3 (2.2–2.4)	5.8 (0.0–14.6)	4.1 (1.2–7.8)	2.7 (2.6–2.8)	2.8 (2.6–3.0)
Uganda	19.1 (16.8–21.9)	14.8 (13.3–16.4)	9.1 (7.6–10.8)	6.8 (5.0–8.6)	9.9 (5.3–14.8)	8.0 (5.1–11.5)	5.6 (2.6–8.7)	4.4 (1.0–8.4)	18.0 (14.9–21.6)	17.0 (14.8–19.5)	10.3 (8.3–13.3)	7.0 (4.8–10.9)	28.5 (20.8–37.1)	26.2 (20.3–32.9)	14.2 (8.7–20.4)	10.2 (3.5–19.2)
Ukraine	2.4 (2.4–2.4)	1.9 (1.9–2.0)	1.0 (0.9–1.0)	0.8 (0.8–0.9)	2.1 (2.0–2.2)	1.8 (1.7–1.9)	1.1 (1.0–1.1)	1.1 (0.9–1.4)	4.5 (4.4–4.5)	4.5 (4.4–4.6)	2.7 (2.7–2.8)	9.2 (8.2–10.3)	7.2 (6.9–7.5)	8.7 (8.4–9.1)	4.6 (4.4–4.8)	23.1 (16.3–32.5)
United Arab Emirates	1.6 (1.3–2.1)	1.2 (1.0–1.6)	1.0 (0.7–1.2)	0.7 (0.5–0.8)	1.7 (1.3–2.2)	1.2 (1.0–1.6)	0.9 (0.7–1.1)	0.6 (0.4–0.7)	3.2 (2.5–4.1)	2.7 (2.1–3.5)	2.4 (1.8–3.0)	2.0 (1.5–2.5)	3.7 (2.9–4.8)	3.3 (2.5–4.2)	2.9 (2.3–3.7)	2.4 (1.9–3.1)
United Kingdom	0.9 (0.8–0.9)	0.6 (0.6–0.6)	0.4 (0.4–0.4)	0.3 (0.3–0.4)	1.0 (0.9–1.0)	0.7 (0.7–0.8)	0.5 (0.4–0.5)	0.4 (0.4–0.5)	2.5 (2.5–2.6)	2.1 (2.1–2.1)	1.2 (1.2–1.2)	1.2 (1.2–1.3)	3.1 (3.0–3.2)	2.9 (2.8–3.1)	1.8 (1.7–1.9)	1.9 (1.7–2.1)
United Republic of Tanzania	18.5 (16.5–20.9)	13.5 (12.2–15.0)	9.9 (8.0–11.6)	8.2 (5.7–10.0)	8.8 (4.5–13.9)	6.1 (3.0–9.3)	4.2 (0.3–10.2)	3.6 (0.0–15.3)	12.3 (10.2–14.6)	13.0 (11.1–14.9)	6.4 (5.1–7.7)	5.4 (3.7–7.6)	14.9 (9.4–20.8)	16.4 (11.8–21.8)	7.6 (4.9–10.7)	6.4 (1.4–15.3)
United States	1.1 (1.1–1.1)	0.8 (0.8–0.8)	0.6 (0.6–0.6)	0.6 (0.6–0.6)	1.3 (1.3–1.4)	1.0 (1.0–1.1)	0.7 (0.7–0.8)	0.9 (0.8–0.9)	4.3 (4.3–4.4)	3.4 (3.3–3.4)	2.4 (2.3–2.4)	2.6 (2.5–2.7)	5.5 (5.3–5.8)	4.7 (4.5–4.8)	4.4 (4.3–4.6)	4.4 (3.8–5.0)
Uruguay	1.5 (1.4–1.7)	1.2 (1.2–1.3)	0.8 (0.7–0.9)	0.6 (0.6–0.7)	1.7 (1.5–1.9)	1.4 (1.3–1.5)	1.0 (0.9–1.1)	0.9 (0.7–1.1)	3.9 (3.7–4.1)	3.6 (3.5–3.8)	3.6 (3.5–3.8)	3.7 (3.3–4.1)	4.4 (4.0–4.8)	4.7 (4.4–5.1)	5.2 (4.8–5.5)	6.1 (4.7–7.8)
Uzbekistan	3.6 (3.5–3.6)	2.7 (2.6–2.7)	1.9 (1.9–1.9)	2.9 (2.8–3.0)	3.0 (2.9–3.2)	2.4 (2.3–2.5)	1.9 (1.8–1.9)	2.9 (2.6–3.2)	4.2 (4.1–4.3)	4.2 (4.1–4.3)	3.4 (3.4–3.5)	4.3 (4.1–4.5)	6.6 (6.3–6.9)	7.0 (6.7–7.3)	4.2 (4.1–4.4)	4.3 (3.7–5.0)
Vanuatu	3.9 (3.0–5.0)	3.0 (2.4–3.9)	2.2 (1.7–2.8)	2.0 (1.6–2.6)	3.0 (2.4–3.9)	2.5 (1.9–3.2)	1.9 (1.5–2.5)	1.8 (1.4–2.3)	5.8 (4.6–7.5)	5.0 (3.9–6.4)	4.1 (3.2–5.3)	3.9 (3.1–5.0)	7.3 (5.7–9.4)	6.4 (5.0–8.2)	5.3 (4.2–6.8)	5.1 (4.0–6.6)
Venezuela (Bolivarian Republic of)	2.2 (2.1–2.2)	1.8 (1.8–1.8)	1.4 (1.4–1.4)	1.3 (1.0–1.7)	2.3 (2.2–2.5)	2.1 (2.0–2.1)	1.9 (1.7–2.0)	1.9 (0.7–3.8)	5.2 (5.0–5.4)	6.5 (6.4–6.6)	9.5 (9.1–10.0)	7.6 (5.0–10.7)	7.1 (6.6–7.6)	10.2 (9.8–10.6)	15.2 (13.6–16.9)	14.4 (5.1–36.3)

Country	Probability of dying $_5q_5$ (per 1,000 children aged 5) 1990	2000	2015	2024	Probability of dying $_5q_{10}$ (per 1,000 children aged 10) 1990	2000	2015	2024	Probability of dying $_5q_{15}$ (per 1,000 youths aged 15) 1990	2000	2015	2024	Probability of dying $_5q_{20}$ (per 1,000 youths aged 20) 1990	2000	2015	2024
Viet Nam	7.5 (6.4–8.7)	3.8 (3.2–4.3)	1.5 (1.3–1.7)	1.0 (0.7–1.3)	2.7 (0.7–5.3)	2.3 (1.2–3.5)	1.8 (1.4–2.3)	1.6 (0.9–2.8)	5.9 (4.4–7.7)	4.2 (3.3–5.3)	3.1 (2.7–3.6)	2.6 (1.8–3.4)	6.5 (2.9–11.1)	5.1 (2.8–7.5)	3.7 (2.7–4.8)	3.2 (1.2–6.2)
Yemen	10.8 (9.6–12.3)	7.1 (6.3–8.0)	5.2 (4.2–6.3)	4.3 (3.1–5.7)	6.9 (3.9–10.0)	5.0 (3.1–7.1)	4.2 (2.1–6.5)	4.1 (0.8–9.3)	7.6 (6.0–9.7)	6.7 (5.3–8.6)	7.2 (5.6–9.1)	4.6 (3.6–5.9)	7.9 (6.2–10.1)	7.1 (5.5–9.1)	11.1 (8.7–14.2)	5.2 (4.0–6.6)
Zambia	17.1 (14.8–19.8)	14.2 (12.7–16.5)	7.1 (5.7–8.4)	5.8 (4.3–7.4)	9.5 (5.0–14.3)	7.4 (3.6–11.8)	4.3 (1.9–6.7)	3.5 (0.5–7.5)	18.1 (14.9–21.9)	16.2 (13.9–18.7)	7.9 (6.6–9.5)	5.0 (3.5–7.1)	30.3 (22.1–38.7)	29.1 (23.2–35.8)	12.5 (9.2–16.1)	7.1 (3.0–12.2)
Zimbabwe	7.9 (6.7–9.2)	8.0 (7.1–9.1)	6.1 (5.1–7.2)	4.0 (2.9–5.2)	5.2 (2.4–7.9)	6.4 (3.8–9.2)	6.8 (4.4–9.3)	5.4 (1.9–10.0)	7.4 (6.1–8.9)	10.8 (9.2–12.3)	9.3 (7.8–11.1)	9.3 (6.6–13.2)	14.1 (10.3–17.9)	23.3 (19.0–28.6)	15.5 (10.9–20.1)	13.7 (4.4–26.6)

Table A.5: Country deaths, 1990–2024

Number of deaths age 5–9, deaths age 10–14, deaths age 15–19, and deaths age 20–24 for 1990, 2000, 2015, and 2024, by country. Values shown are medians with 90% uncertainty intervals in parentheses.

Country	Number of deaths age 5–9 1990	2000	2015	2024	Number of deaths age 10–14 1990	2000	2015	2024	Number of deaths age 15–19 1990	2000	2015	2024	Number of deaths age 20–24 1990	2000	2015	2024
Afghanistan	6,515 (5,003–7,840)	3,817 (3,337–4,399)	5,275 (4,514–6,167)	6,265 (4,843–7,680)	3,665 (0–14,026)	2,239 (813–4,027)	3,425 (1,326–5,802)	4,512 (0–13,705)	6,981 (4,715–9,447)	4,122 (3,234–5,113)	11,674 (8,572–16,130)	10,959 (6,252–17,286)	10,582 (2,414–35,175)	6,167 (3,880–9,271)	13,167 (5,160–24,524)	14,240 (1,536–49,304)
Albania	269 (259–280)	137 (133–142)	39 (36–42)	24 (20–26)	168 (150–188)	117 (105–131)	48 (41–56)	31 (20–45)	208 (197–221)	203 (192–212)	105 (99–112)	48 (37–59)	298 (274–321)	262 (246–279)	129 (115–144)	72 (42–113)
Algeria	4,054 (3,958–4,155)	2,557 (2,523–2,593)	1,542 (1,524–1,559)	1,463 (1,427–1,502)	2,397 (1,907–2,911)	2,127 (2,014–2,244)	1,125 (1,075–1,176)	1,417 (1,271–1,572)	2,956 (2,729–3,195)	2,955 (2,890–3,022)	1,630 (1,595–1,666)	1,906 (1,801–2,007)	2,950 (2,208–3,832)	3,419 (3,261–3,578)	2,430 (2,318–2,543)	2,052 (1,809–2,313)
Andorra	1 (0–1)	0 (0–0)	0 (0–0)	0 (0–0)	1 (0–1)	0 (0–0)	0 (0–0)	0 (0–0)	3 (3–4)	2 (1–3)	1 (1–1)	1 (1–1)	4 (4–6)	3 (3–4)	1 (1–2)	1 (1–2)
Angola	12,444 (9,664–15,926)	12,983 (10,095–16,598)	7,116 (5,544–9,075)	7,374 (5,746–9,402)	4,851 (3,743–6,261)	5,504 (4,260–7,077)	3,884 (3,022–4,963)	4,605 (3,583–5,879)	7,920 (6,122–10,185)	8,905 (6,917–11,407)	6,252 (4,869–7,975)	7,681 (5,984–9,797)	11,386 (8,688–14,906)	10,679 (8,209–13,843)	8,111 (6,298–10,387)	9,341 (7,258–11,954)
Anguilla	0 (0–0)	0 (0–0)	0 (0–0)	0 (0–0)	0 (0–0)	0 (0–0)	0 (0–0)	0 (0–0)	1 (0–1)	0 (0–1)	0 (0–1)	0 (0–1)	1 (1–1)	1 (0–1)	1 (0–1)	1 (0–1)
Antigua and Barbuda	2 (1–2)	2 (2–2)	2 (1–2)	1 (0–1)	2 (2–2)	2 (2–3)	2 (2–2)	2 (1–2)	3 (2–5)	6 (4–7)	4 (3–5)	3 (1–4)	4 (1–9)	6 (4–8)	5 (4–8)	4 (1–8)
Argentina	1,096 (1,076–1,117)	926 (916–938)	671 (661–683)	641 (616–666)	1,151 (1,100–1,206)	1,018 (982–1,057)	830 (800–860)	912 (786–1,057)	2,071 (2,027–2,117)	2,589 (2,548–2,634)	2,643 (2,602–2,687)	2,077 (1,932–2,237)	2,480 (2,372–2,587)	3,397 (3,257–3,541)	3,542 (3,405–3,678)	3,063 (2,438–3,807)
Armenia	132 (125–139)	55 (53–58)	42 (39–44)	37 (34–40)	96 (83–108)	59 (52–67)	35 (31–40)	40 (31–50)	138 (129–148)	122 (115–129)	102 (95–110)	65 (56–75)	239 (215–264)	109 (95–124)	150 (126–174)	101 (79–122)
Australia	233 (225–238)	170 (165–175)	127 (122–132)	118 (111–126)	245 (231–259)	189 (177–200)	136 (126–145)	150 (128–179)	916 (899–932)	699 (685–713)	441 (431–453)	423 (400–445)	1,234 (1,185–1,286)	967 (929–1,007)	689 (654–723)	609 (528–696)
Austria	85 (82–87)	58 (56–60)	32 (30–34)	30 (28–33)	81 (75–88)	63 (58–69)	40 (36–44)	37 (30–44)	367 (357–375)	265 (259–273)	133 (128–138)	147 (139–159)	561 (529–594)	320 (299–340)	204 (188–222)	188 (162–215)
Azerbaijan	491 (478–503)	543 (534–552)	234 (228–240)	233 (223–244)	278 (255–303)	416 (391–443)	167 (155–178)	269 (229–312)	349 (333–364)	596 (577–615)	342 (330–355)	407 (378–436)	512 (472–554)	763 (726–802)	661 (614–711)	476 (280–714)
Bahamas	13 (11–15)	16 (15–18)	8 (7–10)	5 (3–7)	8 (3–14)	11 (8–14)	8 (6–12)	7 (3–13)	22 (18–26)	26 (24–29)	34 (31–38)	17 (12–24)	46 (36–56)	47 (41–53)	78 (69–87)	40 (22–68)

Country	Number of deaths age 5–9				Number of deaths age 10–14				Number of deaths age 15–19				Number of deaths age 20–24			
	1990	2000	2015	2024	1990	2000	2015	2024	1990	2000	2015	2024	1990	2000	2015	2024
Bahrain	24 (20–27)	24 (23–27)	20 (18–22)	19 (16–22)	15 (11–20)	17 (13–21)	17 (14–20)	19 (13–27)	23 (20–26)	29 (26–31)	32 (30–35)	30 (25–34)	32 (24–40)	41 (36–46)	57 (51–65)	45 (33–57)
Bangladesh	54,052 (51,736–56,482)	22,582 (21,648–23,351)	11,322 (10,831–11,799)	7,473 (6,842–8,338)	23,011 (15,385–31,125)	13,311 (10,178–16,726)	9,212 (7,366–11,088)	6,563 (2,121–12,770)	23,393 (20,040–26,279)	16,498 (14,305–19,052)	19,133 (17,674–20,997)	17,992 (8,192–24,498)	25,411 (20,966–30,699)	24,038 (20,447–27,292)	15,090 (12,585–17,634)	14,064 (4,909–26,456)
Barbados	5 (4–7)	4 (3–5)	2 (2–3)	2 (0–2)	7 (3–10)	6 (5–8)	5 (3–8)	3 (2–8)	21 (17–25)	13 (12–16)	10 (7–13)	7 (4–11)	27 (20–33)	20 (16–23)	14 (8–23)	13 (3–40)
Belarus	343 (336–351)	182 (177–187)	78 (74–81)	47 (41–53)	278 (258–297)	228 (208–249)	69 (62–78)	57 (31–91)	614 (600–630)	689 (670–707)	198 (189–206)	138 (109–168)	974 (933–1,017)	1,253 (1,206–1,304)	452 (423–483)	187 (91–349)
Belgium	123 (119–127)	85 (81–88)	55 (53–58)	41 (38–46)	127 (118–135)	91 (84–99)	59 (54–64)	53 (43–62)	402 (393–411)	307 (300–315)	159 (154–164)	135 (126–143)	660 (629–692)	493 (466–523)	282 (265–301)	213 (189–237)
Belize	14 (12–16)	17 (15–18)	12 (11–14)	9 (7–10)	11 (7–16)	15 (13–19)	15 (11–18)	10 (6–14)	12 (9–15)	34 (31–37)	46 (43–50)	42 (35–50)	21 (14–28)	54 (49–61)	73 (65–80)	77 (60–98)
Benin	4,568 (4,073–5,096)	4,482 (4,034–4,946)	4,642 (4,025–5,315)	4,658 (3,765–5,623)	1,739 (910–2,642)	1,942 (1,173–2,746)	2,195 (1,206–3,266)	2,394 (736–4,656)	1,489 (1,149–1,919)	1,549 (1,268–1,872)	2,517 (1,890–3,369)	2,990 (1,791–4,542)	1,298 (698–1,937)	1,471 (950–2,008)	2,294 (1,085–3,679)	2,738 (236–8,021)
Bhutan	239 (141–281)	178 (128–234)	45 (23–86)	25 (9–53)	39 (0–215)	73 (0–162)	77 (18–148)	58 (9–137)	196 (119–284)	128 (101–163)	90 (55–141)	65 (31–111)	136 (0–347)	96 (39–163)	116 (13–261)	96 (0–357)
Bolivia (Plurinational State of)	1,360 (1,155–1,582)	983 (829–1,170)	579 (374–832)	433 (230–675)	786 (415–1,162)	696 (386–1,019)	544 (73–1,231)	462 (31–1,366)	1,649 (1,348–1,987)	1,458 (1,200–1,758)	1,130 (638–1,652)	928 (416–1,504)	1,530 (907–2,141)	1,247 (811–1,723)	1,119 (78–3,452)	1,086 (13–5,247)
Bosnia and Herzegovina	104 (96–113)	40 (38–43)	19 (17–21)	21 (19–25)	91 (76–106)	52 (44–60)	26 (21–30)	27 (18–39)	191 (178–205)	116 (109–123)	69 (64–74)	57 (48–66)	304 (278–330)	161 (147–176)	99 (88–108)	79 (58–102)
Botswana	526 (401–690)	359 (290–455)	206 (163–266)	139 (89–202)	245 (37–467)	234 (76–397)	175 (88–266)	147 (51–262)	361 (263–477)	395 (286–497)	233 (185–295)	226 (154–341)	597 (365–899)	774 (525–1,104)	489 (296–710)	448 (171–883)
Brazil	7,174 (7,036–7,309)	5,661 (5,605–5,715)	3,828 (3,790–3,866)	3,404 (3,345–3,465)	7,459 (6,844–8,107)	6,978 (6,728–7,227)	5,285 (5,101–5,465)	4,512 (4,254–4,772)	16,498 (15,713–17,284)	20,902 (20,509–21,318)	23,939 (23,497–24,393)	14,147 (13,705–14,603)	23,596 (21,454–25,804)	28,565 (27,462–29,683)	30,303 (29,044–31,568)	24,673 (23,245–26,143)
British Virgin Islands	0 (0–0)	0 (0–0)	0 (0–1)	0 (0–0)	0 (0–0)	0 (0–0)	0 (0–1)	0 (0–0)	1 (1–2)	1 (1–2)	2 (1–2)	2 (1–3)	3 (1–3)	1 (1–3)	3 (2–4)	3 (2–3)
Brunei Darussalam	11 (10–13)	10 (9–11)	7 (6–8)	7 (5–8)	9 (8–12)	9 (7–11)	7 (5–9)	5 (3–8)	21 (17–25)	19 (17–21)	12 (10–13)	11 (9–14)	29 (21–37)	25 (21–30)	21 (16–24)	18 (12–26)
Bulgaria	244 (239–250)	134 (131–139)	64 (60–66)	44 (40–48)	249 (232–265)	168 (150–182)	65 (57–72)	57 (47–69)	451 (441–461)	316 (306–324)	140 (136–147)	143 (133–152)	550 (524–575)	485 (460–509)	246 (228–263)	195 (174–219)
Burkina Faso	7,641 (6,729–8,692)	8,703 (7,730–9,837)	5,169 (4,046–6,378)	3,177 (2,103–4,155)	2,332 (734–3,996)	3,234 (1,354–5,284)	2,339 (471–4,779)	1,842 (0–5,597)	3,252 (2,757–3,847)	3,527 (3,066–4,054)	2,908 (2,380–3,514)	3,614 (2,542–4,893)	3,100 (2,165–4,108)	3,337 (2,523–4,216)	3,107 (2,095–4,196)	4,625 (2,011–8,458)
Burundi	7,023 (6,152–8,037)	7,635 (6,710–8,689)	4,344 (3,470–5,332)	4,078 (2,827–5,337)	3,005 (1,213–5,066)	4,373 (2,191–6,913)	2,422 (792–4,481)	3,197 (0–10,855)	3,427 (2,565–4,784)	4,541 (3,678–5,676)	1,858 (1,358–2,455)	2,390 (1,406–3,525)	4,167 (1,169–9,081)	4,526 (2,693–6,678)	1,753 (707–3,098)	1,940 (78–6,751)
Cabo Verde	31 (27–35)	29 (28–32)	12 (11–14)	9 (7–10)	23 (16–32)	28 (23–32)	13 (10–16)	10 (5–15)	24 (19–30)	38 (34–41)	29 (24–33)	21 (15–29)	35 (26–45)	46 (41–52)	44 (32–56)	22 (10–36)
Cambodia	5,294 (4,179–6,490)	4,492 (3,909–5,188)	1,148 (744–1,503)	833 (436–1,153)	1,128 (153–2,208)	1,921 (903–3,025)	611 (0–1,424)	502 (0–1,636)	2,605 (1,969–3,436)	2,553 (2,126–3,017)	1,430 (1,098–1,878)	1,596 (970–2,373)	3,085 (1,594–4,737)	2,256 (1,644–2,900)	1,658 (864–2,519)	1,232 (0–3,547)
Cameroon	7,426 (6,658–8,321)	8,693 (7,840–9,673)	9,417 (7,816–11,194)	8,919 (6,480–11,188)	3,101 (1,763–4,542)	4,123 (2,526–5,982)	5,032 (2,068–8,040)	5,356 (531–11,710)	3,132 (2,593–3,746)	4,480 (3,820–5,216)	5,781 (4,425–7,638)	6,516 (3,943–10,167)	3,265 (2,209–4,320)	5,330 (4,128–6,825)	7,530 (4,490–10,813)	8,594 (2,181–20,550)

Country	Number of deaths age 5–9				Number of deaths age 10–14				Number of deaths age 15–19				Number of deaths age 20–24			
	1990	2000	2015	2024	1990	2000	2015	2024	1990	2000	2015	2024	1990	2000	2015	2024
Canada	384 (375–392)	266 (259–272)	165 (160–169)	182 (174–195)	415 (394–438)	325 (307–343)	211 (199–225)	268 (230–312)	1,331 (1,313–1,350)	1,051 (1,035–1,068)	712 (699–726)	734 (704–768)	1,774 (1,699–1,849)	1,297 (1,244–1,353)	1,250 (1,200–1,300)	1,435 (1,256–1,617)
Central African Republic	1,456 (1,228–1,759)	1,811 (1,502–2,179)	2,549 (1,883–3,367)	1,868 (1,172–2,617)	795 (324–1,303)	1,196 (478–1,961)	1,823 (539–3,443)	1,648 (102–4,127)	997 (796–1,249)	1,442 (1,045–1,989)	1,932 (941–3,409)	1,321 (495–2,609)	1,415 (935–1,955)	1,769 (866–2,962)	2,817 (423–9,712)	2,463 (273–11,810)
Chad	6,232 (5,184–7,348)	6,759 (5,921–7,712)	6,985 (5,775–8,403)	6,576 (4,688–8,683)	2,194 (583–3,951)	2,715 (1,449–4,057)	4,217 (2,231–6,340)	5,089 (1,743–9,613)	3,512 (2,776–4,457)	3,518 (2,926–4,205)	5,320 (3,714–7,406)	6,021 (3,297–9,398)	3,201 (1,890–4,594)	2,848 (1,829–3,999)	4,976 (1,938–8,403)	6,663 (794–21,361)
Chile	406 (397–413)	294 (288–300)	157 (152–161)	142 (136–151)	401 (382–422)	323 (306–341)	194 (182–207)	195 (172–221)	834 (819–850)	679 (663–694)	571 (558–586)	511 (489–533)	1,268 (1,214–1,322)	1,041 (997–1,084)	912 (866–956)	913 (849–976)
China	94,985 (79,077–113,492)	48,567 (44,096–53,665)	18,865 (16,116–21,859)	14,142 (9,466–18,842)	53,145 (24,130–85,805)	44,988 (31,729–58,805)	16,986 (10,321–27,112)	15,593 (5,770–36,312)	123,192 (104,456–146,243)	68,364 (61,402–76,691)	26,144 (20,157–32,948)	19,985 (11,184–31,325)	92,815 (46,063–152,284)	63,673 (46,710–83,109)	52,782 (27,603–92,720)	38,232 (10,041–122,965)
Colombia	1,676 (1,650–1,702)	1,589 (1,573–1,605)	942 (930–954)	730 (713–747)	1,754 (1,677–1,831)	1,747 (1,684–1,812)	1,261 (1,219–1,307)	1,016 (959–1,076)	5,292 (5,180–5,411)	7,443 (7,299–7,586)	4,263 (4,183–4,343)	3,476 (3,366–3,589)	8,334 (7,999–8,680)	10,630 (10,225–11,034)	6,339 (6,098–6,594)	6,447 (6,079–6,827)
Comoros	155 (124–197)	140 (116–174)	86 (67–112)	81 (53–110)	60 (3–129)	65 (17–120)	48 (14–88)	50 (1–120)	112 (77–152)	95 (68–121)	71 (54–94)	75 (48–110)	87 (25–181)	71 (33–118)	62 (29–99)	70 (8–172)
Congo	1,549 (785–1,970)	1,123 (895–1,406)	610 (384–884)	502 (250–781)	518 (0–2,059)	503 (123–907)	422 (33–884)	466 (0–1,342)	1,396 (732–2,147)	972 (770–1,222)	509 (285–844)	530 (229–1,008)	1,476 (53–3,290)	1,321 (915–1,781)	972 (377–1,823)	1,071 (234–3,356)
Cook Islands	1 (0–2)	1 (0–1)	0 (0–0)	0 (0–0)	1 (0–1)	0 (0–1)	0 (0–0)	0 (0–0)	3 (3–4)	3 (2–3)	2 (1–3)	1 (0–2)	4 (1–5)	1 (0–3)	1 (0–4)	0 (0–3)
Costa Rica	107 (101–113)	91 (88–94)	58 (55–61)	55 (50–61)	99 (88–110)	110 (101–118)	79 (71–87)	82 (66–99)	156 (147–164)	238 (231–245)	221 (215–229)	253 (239–267)	213 (193–233)	296 (277–317)	363 (340–388)	455 (416–499)
Croatia	98 (94–102)	42 (40–44)	27 (25–29)	37 (33–41)	95 (84–105)	40 (35–47)	22 (17–26)	35 (19–55)	235 (225–247)	150 (143–158)	72 (67–77)	106 (89–124)	438 (411–465)	214 (198–231)	100 (90–111)	98 (52–157)
Cuba	274 (262–284)	205 (200–213)	109 (105–113)	125 (119–135)	323 (300–349)	255 (237–273)	138 (127–150)	149 (128–173)	1,008 (981–1,037)	440 (426–451)	274 (267–284)	292 (277–308)	1,405 (1,341–1,470)	615 (588–643)	394 (373–416)	473 (432–514)
Cyprus	14 (12–16)	15 (13–16)	7 (7–9)	7 (5–8)	8 (5–12)	11 (8–15)	7 (5–9)	7 (4–11)	36 (32–42)	54 (50–58)	15 (13–17)	9 (7–13)	50 (39–60)	71 (62–80)	37 (31–43)	30 (21–41)
Czechia	176 (170–183)	99 (94–104)	50 (47–53)	48 (43–54)	197 (179–215)	116 (104–128)	47 (42–54)	52 (41–64)	496 (486–505)	339 (332–348)	145 (141–150)	157 (148–166)	540 (517–562)	597 (568–627)	294 (275–312)	238 (215–263)
Côte d'Ivoire	6,877 (5,943–7,876)	7,842 (6,876–8,860)	8,019 (6,677–9,558)	7,402 (5,294–9,485)	3,620 (1,959–5,473)	4,584 (2,670–6,541)	4,913 (2,646–7,392)	5,514 (960–10,476)	3,183 (2,485–3,914)	4,887 (3,931–5,903)	4,380 (3,384–5,693)	4,470 (2,787–6,639)	3,983 (2,695–5,384)	6,481 (4,521–8,999)	5,220 (3,101–7,450)	4,611 (1,099–9,440)
Democratic People's Republic of Korea	1,666 (1,304–2,135)	4,262 (3,334–5,465)	809 (633–1,036)	663 (519–849)	1,169 (914–1,499)	2,482 (1,938–3,190)	750 (587–961)	572 (447–733)	2,847 (2,228–3,650)	3,829 (2,995–4,909)	1,747 (1,368–2,238)	1,273 (997–1,631)	3,722 (2,909–4,781)	4,254 (3,319–5,472)	2,309 (1,805–2,962)	1,817 (1,422–2,331)
Democratic Republic of the Congo	27,331 (22,434–33,286)	41,773 (35,919–49,139)	31,891 (27,073–37,364)	33,158 (24,703–41,888)	9,905 (858–21,507)	19,136 (8,768–30,609)	17,937 (9,909–26,555)	22,038 (7,129–38,611)	15,119 (9,845–21,001)	20,602 (16,578–25,486)	23,578 (19,066–28,938)	31,921 (22,496–44,005)	16,712 (4,457–41,240)	20,589 (13,376–29,285)	24,144 (14,047–33,068)	32,301 (13,260–60,552)
Denmark	50 (47–52)	43 (41–46)	20 (19–22)	17 (15–20)	60 (53–67)	43 (38–48)	25 (21–29)	22 (16–28)	167 (160–174)	117 (112–122)	68 (64–72)	61 (56–68)	246 (229–264)	181 (166–196)	112 (101–123)	99 (83–115)
Djibouti	271 (211–348)	272 (212–349)	206 (160–263)	148 (116–190)	138 (107–177)	146 (114–188)	139 (109–179)	107 (83–137)	220 (171–282)	247 (193–317)	242 (189–310)	220 (171–281)	257 (199–331)	328 (254–422)	330 (256–424)	330 (256–424)

Country	Number of deaths age 5–9				Number of deaths age 10–14				Number of deaths age 15–19				Number of deaths age 20–24			
	1990	2000	2015	2024	1990	2000	2015	2024	1990	2000	2015	2024	1990	2000	2015	2024
Dominica	19 (15–21)	12 (11–14)	3 (2–4)	1 (0–2)	2 (0–8)	3 (0–5)	2 (0–3)	1 (0–3)	7 (5–10)	5 (4–6)	3 (3–4)	3 (1–4)	7 (1–15)	5 (4–7)	8 (6–10)	7 (3–13)
Dominican Republic	753 (653–861)	565 (484–658)	359 (266–474)	280 (178–400)	500 (274–736)	444 (258–629)	375 (156–688)	319 (80–780)	997 (644–1,401)	1,299 (1,067–1,591)	960 (637–1,436)	711 (353–1,241)	977 (253–1,767)	1,471 (974–2,017)	1,699 (629–3,094)	1,520 (288–4,738)
Ecuador	1,026 (1,004–1,048)	756 (744–768)	498 (488–508)	444 (428–458)	882 (824–940)	738 (698–781)	593 (566–620)	636 (573–702)	1,353 (1,308–1,399)	1,449 (1,406–1,492)	1,337 (1,311–1,365)	2,387 (2,269–2,508)	1,710 (1,604–1,818)	1,936 (1,827–2,046)	1,916 (1,844–1,987)	4,286 (3,850–4,746)
Egypt	9,691 (9,545–9,837)	5,221 (5,173–5,271)	4,732 (4,683–4,783)	4,404 (4,182–4,624)	7,024 (6,677–7,384)	4,774 (4,578–4,975)	4,179 (4,017–4,346)	4,388 (3,586–5,304)	7,290 (7,129–7,453)	6,348 (6,234–6,464)	6,750 (6,620–6,893)	7,892 (7,045–8,787)	6,990 (6,647–7,336)	6,057 (5,785–6,334)	7,253 (6,921–7,584)	8,618 (5,792–12,357)
El Salvador	448 (435–462)	241 (236–246)	135 (129–141)	160 (134–188)	422 (396–449)	280 (263–297)	260 (245–277)	359 (258–500)	1,177 (1,142–1,215)	805 (786–825)	1,125 (1,100–1,150)	403 (302–518)	1,883 (1,794–1,975)	1,240 (1,196–1,286)	1,214 (1,159–1,267)	808 (405–1,525)
Equatorial Guinea	284 (222–366)	390 (305–502)	415 (324–533)	372 (291–478)	118 (92–153)	183 (143–237)	222 (173–286)	256 (199–329)	163 (127–209)	223 (174–286)	302 (236–389)	360 (282–463)	182 (142–236)	229 (178–297)	385 (299–496)	377 (293–485)
Eritrea	1,741 (1,430–2,067)	912 (776–1,075)	377 (246–520)	274 (146–406)	636 (159–1,193)	428 (157–736)	281 (0–930)	249 (0–1,033)	987 (767–1,263)	743 (578–951)	662 (517–847)	635 (495–811)	1,293 (997–1,682)	997 (771–1,286)	778 (605–997)	839 (653–1,074)
Estonia	60 (57–62)	25 (24–27)	10 (9–11)	7 (7–9)	51 (45–57)	33 (27–38)	9 (7–12)	9 (6–14)	117 (111–124)	82 (78–88)	24 (22–26)	30 (25–36)	171 (158–185)	149 (138–161)	48 (42–56)	32 (21–43)
Eswatini	255 (130–307)	263 (203–338)	154 (103–237)	101 (43–210)	56 (0–283)	124 (23–228)	198 (73–335)	213 (42–458)	217 (170–279)	238 (186–305)	238 (186–305)	221 (172–283)	263 (204–339)	279 (218–360)	331 (258–427)	311 (242–400)
Ethiopia	71,886 (58,333–86,029)	59,246 (52,294–67,375)	19,937 (15,745–24,673)	14,365 (9,299–19,047)	26,728 (5,226–50,301)	23,962 (12,804–36,201)	12,904 (3,538–23,617)	10,025 (0–25,811)	28,685 (23,394–35,816)	26,726 (22,807–31,144)	21,618 (14,984–29,221)	19,606 (10,745–29,450)	37,509 (24,684–52,386)	28,833 (21,075–37,420)	18,935 (6,133–34,090)	20,690 (1,170–70,375)
Fiji	140 (125–156)	82 (77–88)	41 (38–44)	37 (32–43)	99 (37–182)	75 (62–88)	43 (37–49)	48 (31–72)	103 (47–147)	106 (95–116)	65 (60–69)	60 (46–77)	148 (15–1,222)	129 (111–148)	99 (89–109)	88 (45–163)
Finland	62 (59–64)	46 (45–49)	24 (22–26)	19 (18–22)	59 (52–66)	46 (41–52)	26 (22–30)	28 (21–37)	208 (203–215)	161 (155–166)	92 (89–96)	107 (100–115)	335 (309–360)	242 (224–260)	172 (155–188)	176 (153–199)
France	764 (751–777)	478 (467–488)	319 (311–327)	277 (263–291)	762 (732–792)	548 (523–575)	330 (312–349)	320 (278–367)	2,402 (2,360–2,444)	1,756 (1,721–1,802)	871 (852–890)	895 (847–945)	4,201 (4,061–4,348)	2,773 (2,666–2,876)	1,466 (1,415–1,515)	1,683 (1,523–1,854)
Gabon	275 (172–405)	304 (236–390)	281 (175–411)	271 (139–416)	216 (34–431)	247 (116–411)	211 (20–440)	222 (0–629)	169 (108–261)	295 (224–385)	229 (141–346)	218 (105–366)	218 (88–370)	298 (165–461)	252 (41–502)	242 (0–848)
Gambia	670 (524–863)	636 (497–818)	431 (337–554)	341 (267–439)	282 (220–365)	330 (257–426)	294 (230–378)	293 (229–377)	378 (296–487)	423 (331–544)	443 (347–571)	442 (346–569)	417 (325–540)	403 (314–521)	507 (396–654)	500 (391–646)
Georgia	158 (149–166)	68 (66–71)	58 (55–61)	40 (37–44)	119 (103–134)	76 (67–84)	56 (50–62)	49 (40–59)	229 (216–244)	162 (151–173)	131 (122–139)	110 (99–122)	392 (361–423)	225 (203–245)	228 (203–254)	145 (123–169)
Germany	859 (845–877)	491 (477–503)	276 (270–284)	352 (335–372)	768 (731–804)	610 (580–640)	318 (299–337)	346 (297–403)	2,551 (2,513–2,585)	2,084 (2,056–2,114)	974 (957–990)	951 (914–991)	4,702 (4,524–4,885)	2,741 (2,640–2,850)	1,489 (1,435–1,544)	1,352 (1,203–1,511)
Ghana	7,348 (6,456–8,372)	6,435 (5,827–7,103)	5,100 (4,411–5,892)	4,492 (3,519–5,574)	3,531 (1,991–5,208)	3,663 (2,451–5,120)	3,446 (2,109–4,895)	3,565 (1,295–7,066)	4,080 (2,145–6,795)	3,926 (2,830–5,359)	4,396 (2,505–6,926)	4,344 (1,946–7,582)	4,862 (554–11,596)	4,948 (2,597–7,582)	4,797 (571–10,491)	5,119 (0–18,582)
Greece	127 (123–131)	79 (75–81)	48 (45–50)	27 (25–29)	147 (135–159)	93 (86–101)	53 (47–58)	36 (27–44)	424 (408–436)	361 (351–375)	138 (132–143)	133 (122–146)	639 (611–669)	594 (563–623)	243 (226–259)	211 (178–246)
Grenada	5 (3–6)	5 (3–5)	3 (2–4)	3 (2–4)	3 (1–5)	5 (3–6)	3 (2–5)	5 (2–9)	7 (5–9)	7 (6–8)	5 (4–6)	4 (3–6)	11 (7–15)	7 (5–8)	7 (4–9)	4 (1–9)

Country	Number of deaths age 5–9				Number of deaths age 10–14				Number of deaths age 15–19				Number of deaths age 20–24			
	1990	2000	2015	2024	1990	2000	2015	2024	1990	2000	2015	2024	1990	2000	2015	2024
Guatemala	2,175 (2,144–2,208)	1,196 (1,183–1,208)	756 (745–766)	687 (667–706)	1,274 (1,202–1,349)	923 (884–961)	871 (839–902)	881 (804–961)	1,571 (1,535–1,605)	1,826 (1,791–1,860)	2,395 (2,351–2,441)	1,996 (1,898–2,098)	2,012 (1,932–2,095)	2,489 (2,400–2,579)	2,938 (2,823–3,056)	3,175 (2,833–3,536)
Guinea	5,156 (4,464–6,084)	5,168 (4,590–5,842)	4,618 (3,744–5,470)	4,337 (3,080–5,354)	2,307 (1,024–3,824)	2,235 (1,244–3,235)	2,143 (781–3,733)	2,157 (170–5,524)	2,315 (1,783–2,970)	3,113 (2,624–3,680)	4,052 (2,769–5,633)	3,848 (2,051–6,022)	1,723 (791–2,712)	2,469 (1,681–3,438)	4,297 (1,591–7,558)	4,832 (727–15,422)
Guinea-Bissau	1,093 (394–1,254)	840 (657–995)	490 (354–658)	347 (185–524)	175 (0–2,295)	237 (0–604)	304 (71–570)	304 (0–784)	457 (354–589)	516 (400–665)	466 (362–600)	476 (370–613)	391 (302–508)	504 (389–653)	501 (388–648)	527 (408–681)
Guyana	48 (45–53)	40 (37–42)	28 (25–30)	21 (17–24)	48 (40–56)	38 (33–42)	38 (33–44)	31 (20–47)	94 (86–102)	77 (72–82)	105 (99–112)	72 (56–90)	149 (132–166)	145 (130–160)	147 (131–162)	105 (37–252)
Haiti	3,277 (2,843–3,786)	2,726 (2,349–3,146)	1,646 (1,255–2,094)	1,250 (840–1,684)	1,749 (919–2,753)	1,662 (944–2,466)	1,173 (288–2,205)	975 (57–2,462)	2,580 (1,853–3,536)	2,117 (1,704–2,618)	1,610 (1,092–2,358)	1,506 (791–2,566)	2,772 (1,413–4,330)	2,564 (1,758–3,429)	2,475 (1,281–3,905)	2,791 (681–7,278)
Honduras	832 (701–998)	592 (513–687)	455 (328–607)	366 (216–555)	453 (193–715)	453 (261–660)	669 (338–1,047)	634 (214–1,465)	848 (666–1,088)	930 (732–1,195)	927 (729–1,190)	817 (644–1,050)	998 (783–1,286)	1,175 (923–1,512)	1,241 (975–1,596)	1,211 (952–1,557)
Hungary	159 (155–166)	105 (100–109)	51 (48–54)	41 (37–50)	215 (200–228)	125 (117–135)	60 (53–66)	45 (34–56)	537 (524–549)	291 (282–299)	143 (138–150)	122 (110–133)	679 (651–710)	514 (487–544)	249 (229–267)	208 (169–246)
Iceland	4 (3–5)	3 (2–3)	2 (1–2)	0 (0–1)	3 (2–5)	3 (2–5)	2 (1–3)	2 (0–3)	14 (13–16)	10 (9–12)	6 (5–7)	5 (4–8)	14 (11–18)	12 (10–16)	9 (7–12)	9 (5–15)
India	305,687 (291,647–319,822)	226,073 (218,426–235,029)	89,162 (85,330–93,021)	45,212 (40,236–49,499)	139,015 (114,163–164,106)	135,916 (117,919–155,493)	83,953 (74,050–93,839)	54,351 (40,708–70,702)	180,423 (171,397–189,806)	190,823 (182,959–198,832)	121,498 (115,846–127,120)	89,863 (78,055–103,105)	201,699 (177,054–227,296)	222,085 (198,829–246,981)	164,201 (145,887–181,966)	134,327 (86,937–202,667)
Indonesia	39,039 (35,651–42,889)	25,315 (22,694–28,221)	15,640 (12,665–19,062)	11,576 (8,300–14,966)	20,250 (13,517–27,569)	15,623 (10,440–21,169)	10,919 (5,129–18,411)	9,746 (2,473–23,006)	25,469 (21,029–30,851)	26,335 (22,102–31,069)	23,353 (15,979–31,787)	19,797 (10,457–29,731)	21,047 (12,314–30,559)	22,852 (15,046–31,627)	22,561 (5,340–52,033)	21,398 (1,179–86,107)
Iran (Islamic Republic of)	13,401 (12,505–14,312)	3,840 (3,699–3,983)	2,699 (2,654–2,743)	2,269 (1,933–2,623)	8,729 (4,430–14,042)	4,441 (3,197–5,924)	2,459 (2,357–2,564)	2,363 (1,497–3,629)	9,571 (6,809–13,063)	7,078 (5,815–8,544)	5,226 (5,126–5,329)	6,015 (4,578–7,550)	11,454 (3,958–21,193)	8,521 (5,626–12,456)	8,087 (7,738–8,437)	6,745 (2,642–15,253)
Iraq	7,770 (6,298–9,499)	2,639 (2,187–3,142)	2,854 (2,109–3,753)	2,499 (1,526–3,599)	4,080 (266–9,438)	1,985 (997–3,063)	2,700 (792–6,137)	2,962 (466–9,243)	6,502 (5,080–8,370)	2,608 (2,038–3,337)	3,326 (2,600–4,255)	3,525 (2,755–4,509)	9,134 (7,092–11,905)	2,501 (1,953–3,205)	3,306 (2,582–4,236)	3,574 (2,792–4,577)
Ireland	63 (59–66)	36 (33–37)	24 (23–27)	17 (14–19)	70 (63–77)	45 (41–52)	28 (24–32)	24 (18–33)	165 (157–172)	184 (175–192)	71 (66–76)	81 (70–94)	194 (181–208)	259 (241–276)	113 (103–123)	105 (74–142)
Israel	95 (93–99)	85 (83–88)	71 (68–74)	66 (61–70)	87 (80–95)	82 (75–89)	71 (65–78)	72 (60–88)	187 (182–194)	212 (205–219)	155 (149–161)	185 (171–199)	220 (203–237)	314 (289–339)	212 (195–231)	260 (203–323)
Italy	520 (510–527)	344 (338–350)	205 (199–211)	165 (157–174)	702 (662–740)	412 (387–437)	259 (240–278)	245 (196–307)	2,384 (2,351–2,414)	1,294 (1,276–1,313)	634 (622–645)	575 (550–602)	3,292 (3,157–3,432)	2,158 (2,076–2,241)	971 (933–1,011)	850 (749–961)
Jamaica	122 (96–156)	100 (78–127)	69 (53–87)	51 (40–65)	123 (96–156)	108 (85–138)	87 (68–111)	68 (53–87)	272 (213–346)	251 (196–319)	233 (182–296)	188 (147–239)	365 (286–466)	325 (254–414)	365 (286–466)	294 (230–375)
Japan	1,351 (1,336–1,367)	766 (756–778)	432 (423–440)	356 (337–370)	1,259 (1,203–1,315)	735 (702–769)	491 (465–517)	489 (446–538)	4,419 (4,353–4,486)	2,448 (2,412–2,486)	1,218 (1,195–1,240)	1,387 (1,347–1,430)	4,677 (4,504–4,852)	3,960 (3,823–4,100)	2,164 (2,094–2,232)	2,321 (2,193–2,458)
Jordan	280 (254–309)	312 (295–331)	283 (276–291)	158 (150–167)	212 (125–317)	227 (182–275)	216 (202–232)	145 (120–173)	270 (194–369)	309 (274–347)	369 (356–382)	304 (280–329)	316 (155–510)	423 (333–522)	455 (428–481)	289 (240–341)
Kazakhstan	1,095 (1,075–1,114)	716 (707–725)	512 (503–521)	497 (482–513)	880 (835–926)	788 (755–825)	416 (398–434)	525 (470–582)	1,649 (1,613–1,685)	1,716 (1,688–1,749)	789 (772–809)	878 (835–923)	2,404 (2,308–2,501)	3,046 (2,948–3,147)	1,464 (1,407–1,519)	906 (802–1,016)

Country	Number of deaths age 5–9				Number of deaths age 10–14				Number of deaths age 15–19				Number of deaths age 20–24			
	1990	2000	2015	2024	1990	2000	2015	2024	1990	2000	2015	2024	1990	2000	2015	2024
Kenya	8,225 (7,325–9,231)	9,418 (8,376–10,551)	6,198 (5,099–7,484)	3,222 (2,348–4,185)	3,297 (1,529–5,223)	4,992 (2,561–7,876)	4,042 (1,922–6,443)	2,775 (74–6,486)	4,613 (3,568–5,905)	7,564 (6,391–8,880)	7,044 (4,811–9,914)	7,011 (3,632–11,261)	6,172 (3,948–8,557)	10,170 (7,708–12,991)	9,045 (3,224–16,262)	10,150 (1,587–32,940)
Kiribati	19 (15–24)	17 (13–21)	17 (13–22)	18 (14–23)	10 (8–12)	11 (8–13)	11 (8–13)	12 (9–16)	14 (11–18)	16 (12–20)	19 (15–24)	18 (14–23)	18 (14–23)	14 (11–19)	21 (17–28)	20 (15–25)
Kosovo (UNSCR 1244)	198 (154–252)	107 (83–136)	38 (30–48)	20 (16–26)	148 (116–190)	117 (92–150)	44 (34–56)	29 (22–36)	212 (165–270)	158 (123–201)	90 (70–115)	64 (51–83)	315 (246–403)	174 (136–223)	131 (103–168)	101 (79–129)
Kuwait	125 (117–134)	51 (49–54)	52 (49–55)	49 (45–55)	92 (75–110)	47 (40–55)	47 (40–54)	58 (39–79)	155 (140–172)	100 (92–106)	102 (96–107)	88 (77–100)	230 (198–262)	135 (121–151)	105 (95–116)	101 (76–125)
Kyrgyzstan	346 (340–352)	286 (281–292)	185 (180–190)	204 (196–214)	243 (228–258)	268 (251–285)	177 (166–188)	241 (210–274)	362 (352–373)	391 (381–402)	316 (307–327)	332 (312–352)	540 (517–566)	627 (602–651)	473 (449–499)	341 (286–394)
Lao People's Democratic Republic	3,515 (2,579–4,366)	2,310 (1,941–2,730)	630 (506–777)	342 (226–482)	1,596 (0–3,960)	1,368 (774–2,007)	619 (349–909)	407 (136–771)	1,358 (473–2,276)	1,614 (1,109–2,335)	824 (502–1,481)	623 (276–1,223)	1,004 (0–2,684)	1,128 (262–2,107)	1,051 (169–2,174)	967 (6–3,121)
Latvia	110 (106–114)	52 (50–55)	18 (16–19)	11 (9–12)	90 (80–101)	61 (51–71)	15 (12–18)	10 (7–15)	211 (203–222)	161 (152–168)	42 (40–46)	39 (34–44)	307 (286–328)	253 (238–270)	89 (79–100)	53 (42–64)
Lebanon	258 (201–329)	185 (144–236)	148 (116–190)	332 (258–424)	277 (215–353)	207 (161–264)	154 (120–197)	318 (247–406)	889 (687–1,139)	313 (244–400)	313 (243–399)	543 (423–694)	1,959 (1,498–2,544)	324 (252–414)	378 (294–483)	725 (563–933)
Lesotho	451 (373–541)	364 (311–428)	254 (202–318)	220 (150–295)	346 (156–553)	352 (218–490)	240 (140–357)	230 (83–445)	454 (297–705)	699 (578–837)	378 (280–510)	163 (88–283)	510 (47–1,220)	1,138 (884–1,435)	736 (498–996)	274 (47–592)
Liberia	1,240 (1,050–1,472)	1,363 (1,181–1,565)	1,574 (1,254–1,955)	1,244 (873–1,663)	693 (218–1,389)	854 (435–1,406)	1,077 (418–1,828)	1,043 (79–3,017)	1,524 (952–2,146)	891 (706–1,120)	1,372 (1,003–1,865)	1,433 (810–2,253)	3,137 (1,661–5,494)	1,072 (719–1,479)	1,430 (725–2,183)	1,538 (250–3,913)
Libya	598 (428–819)	278 (191–429)	248 (92–472)	146 (44–289)	363 (13–811)	260 (8–509)	221 (0–811)	176 (0–789)	505 (395–647)	514 (401–658)	506 (395–650)	356 (278–456)	447 (349–573)	552 (431–708)	812 (628–1,049)	380 (296–486)
Lithuania	130 (127–134)	72 (69–74)	23 (21–24)	15 (14–17)	106 (97–116)	79 (70–88)	24 (20–28)	18 (13–24)	270 (260–281)	233 (224–243)	90 (84–95)	45 (39–52)	408 (384–432)	375 (354–394)	173 (158–189)	85 (70–101)
Luxembourg	4 (3–5)	2 (2–3)	2 (1–2)	2 (1–2)	3 (2–5)	2 (2–3)	2 (1–2)	2 (0–4)	18 (17–21)	10 (8–11)	7 (6–8)	6 (4–8)	31 (26–37)	17 (14–21)	10 (7–12)	6 (2–11)
Madagascar	8,314 (7,226–9,549)	7,485 (6,697–8,340)	7,221 (6,183–8,404)	9,659 (7,610–11,817)	4,479 (2,475–6,850)	3,801 (2,189–5,684)	4,064 (2,077–6,226)	5,526 (16–16,493)	3,759 (3,011–4,653)	4,204 (3,533–4,963)	5,457 (4,152–7,394)	4,966 (2,981–7,560)	3,662 (2,396–5,062)	4,273 (2,997–5,610)	5,125 (2,419–8,086)	5,755 (1,058–14,251)
Malawi	7,535 (6,786–8,340)	5,642 (5,148–6,145)	4,594 (3,999–5,254)	3,567 (2,804–4,427)	2,705 (1,543–3,882)	2,530 (1,705–3,369)	2,680 (1,700–3,751)	2,737 (1,136–5,082)	3,485 (2,911–4,124)	4,959 (4,308–5,663)	3,260 (2,581–4,081)	3,234 (2,024–4,763)	3,796 (2,575–5,159)	5,905 (4,501–7,496)	4,241 (2,667–6,079)	4,443 (1,101–11,133)
Malaysia	1,086 (1,062–1,111)	834 (823–845)	619 (609–629)	480 (465–495)	1,029 (939–1,124)	906 (871–943)	827 (796–859)	640 (579–704)	1,664 (1,533–1,801)	1,824 (1,776–1,873)	2,010 (1,965–2,055)	1,803 (1,669–1,949)	2,270 (1,902–2,711)	2,017 (1,905–2,133)	2,180 (2,069–2,288)	2,043 (1,709–2,409)
Maldives	40 (37–44)	22 (21–24)	8 (7–10)	3 (3–5)	20 (14–25)	21 (16–25)	9 (6–11)	6 (3–11)	25 (22–29)	18 (16–20)	19 (17–21)	13 (10–16)	29 (22–35)	18 (15–21)	25 (19–31)	8 (1–18)
Mali	7,906 (7,037–8,899)	7,629 (6,932–8,417)	8,741 (7,617–9,986)	7,714 (6,039–9,363)	3,030 (1,583–4,527)	3,465 (2,174–4,846)	4,606 (2,726–6,571)	5,072 (1,425–8,794)	3,483 (2,828–4,352)	3,772 (3,227–4,399)	3,863 (2,916–5,067)	5,061 (2,964–7,641)	2,743 (1,618–3,964)	3,420 (2,492–4,471)	3,805 (2,112–5,717)	5,476 (1,266–14,335)
Malta	3 (2–5)	3 (2–4)	2 (1–2)	2 (1–2)	5 (2–8)	4 (3–5)	2 (2–3)	2 (0–4)	11 (8–13)	11 (10–13)	6 (4–7)	4 (3–7)	12 (8–17)	15 (11–18)	9 (7–13)	8 (4–14)
Marshall Islands	8 (6–10)	6 (5–7)	5 (4–7)	3 (2–3)	5 (4–6)	5 (4–7)	3 (3–5)	2 (2–3)	6 (5–8)	9 (6–11)	6 (4–7)	4 (4–6)	6 (5–8)	8 (6–10)	5 (4–7)	4 (3–5)
Mauritania	779 (683–893)	770 (669–897)	663 (505–838)	595 (373–786)	347 (152–535)	347 (154–563)	338 (102–605)	355 (0–863)	394 (281–541)	347 (245–468)	506 (358–707)	689 (385–1,069)	409 (140–751)	374 (135–683)	515 (229–830)	689 (64–1,911)

Country	Number of deaths age 5–9				Number of deaths age 10–14				Number of deaths age 15–19				Number of deaths age 20–24			
	1990	2000	2015	2024	1990	2000	2015	2024	1990	2000	2015	2024	1990	2000	2015	2024
Mauritius	34 (30–38)	28 (26–30)	14 (13–16)	9 (8–11)	43 (36–51)	30 (27–35)	21 (17–24)	13 (9–17)	71 (65–78)	61 (56–65)	55 (52–60)	52 (46–60)	105 (91–119)	92 (83–103)	98 (87–108)	84 (67–101)
Mexico	5,699 (5,610–5,785)	3,574 (3,539–3,609)	2,543 (2,517–2,569)	2,371 (2,319–2,422)	5,294 (5,053–5,554)	3,780 (3,645–3,920)	3,293 (3,183–3,407)	3,444 (3,155–3,749)	8,983 (8,796–9,177)	7,596 (7,477–7,715)	8,168 (8,027–8,297)	8,720 (8,356–9,140)	11,163 (10,669–11,660)	10,106 (9,701–10,511)	12,583 (12,054–13,102)	13,816 (12,075–15,648)
Micronesia (Federated States of)	16 (13–21)	12 (10–16)	8 (7–11)	6 (5–8)	11 (9–14)	10 (7–12)	7 (5–8)	5 (4–7)	16 (13–21)	17 (13–22)	13 (10–17)	10 (8–14)	16 (13–21)	16 (12–20)	15 (12–19)	13 (10–16)
Monaco	0 (0–0)	0 (0–0)	0 (0–0)	0 (0–0)	0 (0–0)	0 (0–0)	0 (0–0)	0 (0–0)	1 (0–1)	0 (0–0)	0 (0–0)	0 (0–0)	1 (1–1)	1 (0–1)	0 (0–1)	0 (0–1)
Mongolia	385 (372–397)	211 (206–216)	127 (122–131)	106 (101–113)	249 (208–292)	203 (188–220)	109 (102–118)	136 (114–159)	290 (264–316)	273 (264–283)	205 (196–213)	192 (174–211)	456 (398–518)	417 (397–438)	316 (294–337)	206 (172–243)
Montenegro	14 (12–17)	9 (8–10)	4 (3–5)	3 (2–5)	13 (8–19)	10 (8–13)	5 (3–7)	5 (2–8)	25 (21–29)	25 (22–27)	11 (10–13)	19 (15–23)	41 (34–50)	35 (30–40)	18 (14–22)	25 (18–34)
Montserrat	0 (0–0)	0 (0–0)	0 (0–0)	0 (0–0)	0 (0–0)	0 (0–0)	0 (0–0)	0 (0–0)	1 (0–1)	0 (0–0)	0 (0–0)	0 (0–0)	1 (1–2)	0 (0–0)	0 (0–0)	0 (0–0)
Morocco	3,775 (3,308–4,314)	2,196 (1,785–2,645)	1,163 (780–1,534)	842 (472–1,172)	2,231 (1,362–3,145)	1,448 (672–2,251)	691 (18–1,575)	601 (0–1,931)	2,818 (2,224–3,561)	2,312 (1,776–2,933)	1,497 (881–2,253)	1,238 (574–2,078)	3,100 (1,897–4,322)	2,577 (1,529–3,695)	1,893 (298–4,797)	1,655 (83–6,664)
Mozambique	15,801 (11,963–19,579)	11,338 (9,755–13,178)	6,300 (5,067–7,806)	4,816 (3,302–6,504)	7,224 (765–14,898)	5,817 (3,167–8,708)	3,732 (1,502–6,146)	3,234 (310–7,259)	5,051 (3,791–6,670)	6,691 (5,502–8,191)	5,148 (3,873–6,696)	5,515 (3,440–8,614)	4,545 (2,340–6,925)	6,413 (4,335–8,870)	6,626 (4,174–9,224)	6,896 (2,115–13,276)
Myanmar	22,066 (14,000–28,311)	14,085 (10,206–18,423)	2,788 (1,865–4,069)	1,703 (822–2,695)	6,560 (0–24,319)	6,247 (0–25,447)	2,532 (161–5,438)	1,717 (0–5,289)	11,976 (7,598–19,004)	10,868 (7,382–16,325)	3,574 (2,448–5,262)	3,443 (1,757–5,929)	9,460 (240–21,643)	10,268 (1,943–26,572)	5,687 (2,436–9,530)	7,633 (1,741–22,036)
Namibia	318 (258–389)	419 (363–487)	368 (297–451)	273 (191–364)	200 (71–339)	262 (140–387)	267 (117–444)	211 (33–586)	356 (292–426)	448 (385–518)	433 (354–527)	335 (240–466)	450 (302–601)	665 (517–843)	792 (523–1,111)	597 (266–1,110)
Nauru	2 (2–2)	2 (1–2)	0 (0–0)	0 (0–0)	1 (0–2)	0 (0–1)	0 (0–0)	0 (0–0)	2 (1–2)	1 (1–2)	1 (0–1)	0 (0–1)	1 (1–2)	1 (1–2)	1 (1–1)	0 (0–1)
Nepal	9,731 (8,613–11,040)	6,106 (5,519–6,761)	2,954 (2,444–3,540)	1,304 (942–1,703)	3,812 (1,881–5,886)	2,938 (1,761–4,209)	2,523 (1,401–3,963)	1,167 (259–3,180)	4,006 (3,158–5,142)	3,492 (2,843–4,215)	4,018 (2,793–5,824)	2,269 (1,211–3,665)	3,375 (1,880–4,979)	3,653 (2,449–4,869)	4,160 (1,684–7,100)	3,188 (489–9,752)
Netherlands (Kingdom of the)	162 (157–166)	132 (127–135)	67 (64–70)	65 (61–70)	168 (156–181)	145 (135–157)	92 (83–100)	93 (78–109)	432 (422–442)	337 (329–347)	204 (198–211)	215 (204–228)	660 (628–691)	449 (426–474)	303 (285–320)	357 (323–393)
New Zealand	62 (59–64)	50 (47–51)	29 (27–31)	24 (22–27)	71 (64–78)	59 (54–66)	38 (34–43)	40 (28–55)	292 (281–304)	195 (187–204)	141 (133–147)	119 (99–140)	351 (328–374)	206 (190–223)	189 (173–206)	173 (114–254)
Nicaragua	599 (455–769)	417 (342–507)	202 (98–336)	150 (60–277)	309 (72–568)	369 (224–527)	301 (69–677)	256 (44–750)	873 (682–1,121)	746 (583–956)	521 (407–668)	485 (379–621)	1,083 (843–1,394)	940 (733–1,208)	747 (582–959)	683 (533–877)
Niger	11,430 (9,977–13,294)	11,675 (10,137–13,252)	14,083 (11,685–17,121)	14,976 (10,879–20,700)	3,825 (1,432–6,164)	4,975 (2,885–7,237)	7,629 (3,642–11,954)	8,937 (1,283–17,929)	3,270 (2,574–4,112)	3,842 (3,159–4,656)	4,959 (3,161–7,482)	5,610 (2,783–9,485)	2,861 (1,538–4,159)	3,878 (2,684–5,148)	5,596 (1,752–11,541)	7,663 (1,078–27,889)
Nigeria	71,487 (62,467–82,433)	71,683 (65,207–80,134)	79,407 (71,229–88,196)	86,235 (72,186–100,692)	31,663 (15,560–48,947)	32,924 (20,978–45,825)	42,078 (27,867–57,172)	52,496 (22,765–95,382)	34,259 (24,848–46,392)	33,852 (27,653–40,818)	32,199 (27,110–38,165)	36,104 (26,429–49,773)	33,097 (15,242–56,246)	38,380 (27,333–50,231)	36,190 (27,120–45,708)	38,113 (18,728–61,683)
Niue	0 (0–0)	0 (0–0)	0 (0–0)	0 (0–0)	0 (0–0)	0 (0–0)	0 (0–0)	0 (0–0)	0 (0–0)	0 (0–0)	0 (0–0)	0 (0–0)	0 (0–0)	0 (0–0)	0 (0–0)	0 (0–0)
North Macedonia	66 (62–71)	42 (40–44)	16 (14–17)	12 (10–13)	59 (48–70)	40 (35–45)	20 (16–23)	15 (10–21)	86 (80–93)	77 (74–82)	40 (37–42)	34 (29–38)	106 (94–120)	101 (91–112)	52 (45–59)	45 (35–57)

Country	Number of deaths age 5–9				Number of deaths age 10–14				Number of deaths age 15–19				Number of deaths age 20–24			
	1990	2000	2015	2024	1990	2000	2015	2024	1990	2000	2015	2024	1990	2000	2015	2024
Norway	48 (46–50)	39 (37–41)	23 (21–24)	17 (16–20)	48 (43–52)	39 (35–43)	28 (23–31)	26 (20–33)	174 (168–182)	135 (129–140)	82 (78–86)	92 (84–101)	227 (208–245)	198 (182–216)	131 (118–144)	124 (105–145)
Oman	179 (140–228)	100 (78–127)	78 (61–99)	115 (90–146)	174 (136–222)	104 (81–132)	61 (48–78)	94 (73–119)	163 (128–208)	173 (135–219)	131 (103–167)	160 (125–203)	163 (127–208)	180 (140–229)	262 (205–334)	274 (214–348)
Pakistan	27,755 (26,522–29,019)	31,072 (29,091–33,168)	25,162 (21,348–29,241)	18,291 (14,114–22,562)	15,559 (13,188–17,870)	20,860 (15,844–25,838)	19,411 (11,114–29,806)	16,555 (3,621–34,298)	24,218 (20,910–27,753)	24,098 (20,843–27,313)	20,987 (15,744–29,058)	17,321 (10,872–27,965)	21,453 (15,259–28,721)	24,950 (19,079–31,188)	25,301 (12,526–39,275)	22,705 (6,151–44,340)
Palau	1 (1–2)	1 (0–2)	0 (0–0)	0 (0–0)	1 (0–1)	0 (0–1)	0 (0–0)	0 (0–0)	1 (1–3)	3 (1–3)	2 (1–3)	1 (0–3)	3 (1–4)	3 (1–4)	3 (0–9)	1 (0–9)
Panama	155 (148–163)	132 (128–136)	125 (120–129)	108 (101–115)	124 (106–143)	123 (112–133)	137 (126–147)	134 (107–164)	245 (231–263)	245 (236–253)	323 (314–335)	258 (233–285)	328 (294–361)	342 (322–363)	454 (429–480)	419 (321–530)
Papua New Guinea	957 (749–1,223)	1,098 (860–1,403)	1,434 (1,123–1,833)	1,027 (805–1,313)	540 (422–691)	669 (523–856)	935 (732–1,197)	743 (582–951)	788 (616–1,007)	1,011 (792–1,292)	1,303 (1,020–1,665)	1,303 (1,020–1,665)	843 (658–1,081)	1,109 (867–1,422)	1,494 (1,168–1,914)	1,520 (1,189–1,946)
Paraguay	297 (274–322)	203 (193–214)	133 (128–138)	113 (103–123)	200 (100–318)	192 (159–228)	175 (163–188)	165 (130–204)	557 (435–717)	654 (511–842)	586 (458–755)	473 (369–608)	754 (588–972)	773 (603–997)	843 (658–1,087)	691 (539–890)
Peru	2,996 (2,765–3,232)	1,559 (1,490–1,631)	773 (756–790)	718 (609–826)	2,353 (1,673–3,116)	1,405 (1,115–1,720)	772 (738–806)	835 (538–1,270)	4,217 (3,627–4,889)	2,586 (2,289–2,898)	1,429 (1,401–1,458)	1,617 (1,205–2,067)	4,080 (2,969–5,293)	3,058 (2,353–3,816)	1,966 (1,886–2,048)	2,344 (1,018–4,989)
Philippines	9,175 (9,036–9,316)	6,382 (6,319–6,443)	5,395 (5,340–5,453)	4,743 (4,593–4,896)	5,906 (5,555–6,266)	5,145 (4,932–5,362)	5,073 (4,864–5,282)	5,305 (4,602–6,075)	7,188 (7,033–7,351)	7,047 (6,915–7,182)	8,252 (8,095–8,407)	9,791 (9,045–10,564)	10,683 (10,266–11,114)	9,607 (9,267–9,953)	12,052 (11,616–12,486)	13,176 (10,608–16,258)
Poland	948 (938–959)	454 (448–461)	196 (191–200)	172 (166–179)	882 (834–935)	590 (556–623)	226 (211–241)	266 (239–297)	2,004 (1,977–2,033)	1,807 (1,777–1,832)	789 (773–803)	707 (684–733)	2,494 (2,382–2,615)	2,432 (2,334–2,534)	1,458 (1,398–1,520)	1,002 (939–1,068)
Portugal	263 (257–268)	131 (128–135)	43 (41–45)	33 (31–36)	304 (285–325)	146 (135–158)	54 (48–60)	43 (35–53)	802 (783–822)	434 (420–448)	121 (115–127)	129 (117–142)	977 (937–1,019)	752 (716–790)	189 (175–204)	203 (175–232)
Qatar	14 (13–16)	15 (13–16)	21 (18–22)	25 (22–28)	14 (10–18)	14 (11–17)	17 (13–20)	20 (13–28)	25 (21–29)	33 (28–36)	50 (46–54)	45 (37–51)	27 (19–36)	42 (34–51)	104 (82–125)	47 (32–65)
Republic of Korea	2,047 (2,015–2,080)	869 (858–880)	198 (193–203)	150 (143–157)	1,526 (1,441–1,613)	647 (619–677)	230 (214–245)	220 (188–257)	3,497 (3,418–3,575)	1,828 (1,793–1,863)	716 (698–736)	551 (516–587)	4,529 (4,316–4,747)	2,446 (2,336–2,555)	1,149 (1,100–1,198)	1,053 (915–1,196)
Republic of Moldova	259 (250–269)	149 (145–153)	48 (46–50)	39 (36–42)	174 (156–192)	166 (151–181)	52 (46–58)	54 (44–65)	325 (310–339)	273 (265–281)	109 (104–113)	106 (97–114)	488 (461–514)	366 (348–383)	202 (187–218)	123 (108–140)
Romania	978 (946–1,011)	569 (540–599)	197 (184–210)	129 (114–146)	903 (836–972)	1,022 (956–1,087)	235 (213–258)	143 (116–171)	1,376 (1,341–1,413)	932 (905–959)	455 (437–474)	356 (332–383)	1,989 (1,895–2,085)	1,374 (1,307–1,443)	653 (614–692)	501 (457–546)
Russian Federation	6,415 (6,346–6,491)	3,780 (3,736–3,822)	1,786 (1,766–1,809)	1,302 (1,263–1,339)	5,213 (4,966–5,463)	5,499 (5,260–5,741)	1,967 (1,892–2,044)	2,006 (1,814–2,208)	11,674 (11,448–11,904)	17,326 (16,984–17,670)	4,734 (4,640–4,826)	5,500 (5,133–5,856)	16,521 (15,800–17,233)	31,877 (30,652–33,156)	11,058 (10,631–11,493)	16,234 (14,331–18,305)
Rwanda	9,969 (8,992–11,071)	8,341 (7,635–9,119)	2,392 (2,074–2,750)	851 (667–1,041)	4,219 (1,918–6,937)	5,279 (3,303–7,445)	1,560 (739–2,621)	624 (0–2,088)	4,792 (3,663–6,171)	5,225 (4,521–6,051)	1,419 (1,161–1,709)	1,179 (808–1,621)	4,453 (2,146–7,154)	4,614 (3,293–6,072)	1,705 (1,073–2,466)	1,449 (366–3,215)
Saint Kitts and Nevis	2 (2–3)	2 (2–2)	1 (0–2)	0 (0–1)	2 (2–3)	2 (2–3)	1 (1–2)	1 (0–1)	3 (1–4)	4 (4–6)	6 (4–7)	4 (2–6)	3 (0–6)	5 (4–8)	8 (5–12)	7 (2–18)
Saint Lucia	7 (5–8)	7 (6–8)	3 (3–5)	2 (2–3)	6 (3–10)	6 (5–8)	3 (2–5)	2 (0–4)	13 (11–16)	13 (11–14)	10 (9–12)	14 (10–18)	18 (14–24)	19 (16–22)	20 (16–24)	33 (22–46)
Saint Vincent and the Grenadines	7 (5–8)	5 (4–5)	3 (2–3)	2 (1–2)	5 (2–7)	5 (3–6)	5 (3–6)	4 (2–6)	10 (8–12)	8 (7–10)	8 (7–10)	6 (4–8)	11 (8–14)	14 (11–17)	12 (10–16)	8 (4–12)

Country	Number of deaths age 5–9				Number of deaths age 10–14				Number of deaths age 15–19				Number of deaths age 20–24			
	1990	2000	2015	2024	1990	2000	2015	2024	1990	2000	2015	2024	1990	2000	2015	2024
Samoa	18 (8–22)	15 (11–19)	8 (5–13)	7 (3–11)	5 (0–29)	5 (0–12)	6 (1–11)	6 (0–15)	35 (9–71)	19 (12–28)	14 (9–21)	14 (7–22)	58 (0–231)	28 (14–46)	15 (5–26)	13 (0–39)
San Marino	0 (0–0)	0 (0–0)	0 (0–0)	0 (0–0)	0 (0–0)	0 (0–0)	0 (0–0)	0 (0–0)	1 (1–1)	0 (0–0)	0 (0–0)	0 (0–0)	3 (1–3)	1 (1–1)	0 (0–0)	0 (0–0)
Sao Tome and Principe	49 (38–62)	40 (31–51)	13 (10–17)	7 (6–10)	27 (21–35)	27 (21–34)	13 (10–16)	10 (7–12)	26 (7–48)	26 (16–42)	28 (15–44)	29 (12–53)	34 (0–200)	35 (13–62)	43 (11–93)	51 (8–174)
Saudi Arabia	1,150 (901–1,468)	903 (708–1,153)	546 (428–698)	434 (340–554)	1,085 (850–1,388)	937 (734–1,198)	448 (352–573)	367 (288–469)	1,377 (483–2,165)	2,075 (1,555–2,751)	2,195 (1,580–3,093)	1,971 (1,064–3,187)	1,209 (0–7,431)	1,733 (716–2,950)	3,208 (1,151–5,586)	2,585 (125–7,606)
Senegal	5,507 (5,034–6,044)	6,079 (5,657–6,572)	2,804 (2,497–3,139)	1,978 (1,544–2,395)	2,085 (1,022–3,216)	3,035 (1,950–4,285)	1,993 (1,350–2,677)	1,946 (607–4,366)	1,960 (1,557–2,445)	2,451 (2,080–2,882)	1,925 (1,555–2,378)	1,681 (1,101–2,341)	1,801 (1,074–2,527)	2,107 (1,452–2,836)	1,643 (995–2,289)	1,518 (378–2,920)
Serbia	169 (161–177)	98 (94–101)	38 (37–41)	36 (33–39)	159 (140–177)	112 (100–125)	50 (44–57)	50 (40–61)	319 (306–333)	289 (279–298)	112 (107–117)	115 (106–124)	430 (398–463)	445 (414–478)	190 (171–210)	175 (149–203)
Seychelles	3 (3–5)	3 (2–3)	2 (2–3)	2 (1–2)	3 (1–5)	2 (1–3)	2 (0–3)	2 (0–5)	4 (3–6)	5 (5–7)	6 (5–9)	7 (4–9)	8 (5–11)	8 (5–10)	7 (4–11)	6 (0–20)
Sierra Leone	3,327 (2,085–4,244)	2,630 (2,144–3,148)	3,096 (2,613–3,684)	2,997 (2,161–3,837)	1,509 (0–8,943)	1,279 (474–2,237)	1,802 (929–2,763)	1,982 (264–4,756)	2,052 (998–3,410)	1,988 (1,520–2,530)	2,880 (2,241–3,629)	2,705 (1,526–3,994)	1,922 (308–3,988)	1,978 (1,197–2,762)	2,679 (1,568–3,821)	2,955 (492–7,087)
Singapore	44 (39–47)	32 (30–33)	15 (14–17)	16 (14–18)	55 (46–63)	40 (35–44)	24 (20–28)	23 (18–30)	125 (116–135)	95 (90–101)	83 (78–88)	82 (74–92)	208 (186–230)	180 (164–197)	160 (142–178)	151 (121–185)
Slovakia	120 (117–124)	80 (77–82)	39 (36–40)	31 (29–34)	117 (106–127)	89 (80–98)	39 (34–43)	36 (29–45)	246 (240–253)	211 (204–217)	103 (99–108)	85 (78–91)	297 (284–311)	321 (305–337)	186 (174–198)	121 (107–136)
Slovenia	34 (32–36)	16 (15–17)	9 (7–9)	6 (5–7)	33 (28–38)	20 (16–23)	9 (7–11)	9 (6–13)	98 (93–103)	78 (75–83)	27 (25–29)	22 (19–26)	154 (144–166)	125 (114–135)	45 (39–50)	29 (22–36)
Solomon Islands	41 (33–53)	42 (34–55)	48 (37–61)	48 (37–61)	28 (22–37)	30 (23–39)	36 (28–46)	38 (30–50)	47 (37–60)	52 (41–67)	64 (50–81)	72 (56–92)	46 (35–58)	59 (46–75)	67 (52–86)	84 (66–108)
Somalia	5,663 (4,408–7,297)	6,569 (5,115–8,464)	8,099 (6,309–10,426)	8,401 (6,548–10,807)	2,319 (1,796–3,005)	2,835 (2,198–3,673)	3,911 (3,037–5,057)	4,093 (3,182–5,283)	4,403 (3,415–5,695)	4,327 (3,369–5,573)	5,913 (4,606–7,611)	6,981 (5,439–8,999)	6,679 (5,090–8,808)	4,796 (3,711–6,226)	6,312 (4,892–8,181)	9,082 (7,013–11,821)
South Africa	5,472 (4,034–5,997)	7,066 (6,499–7,510)	4,096 (3,875–4,261)	2,575 (2,386–2,814)	1,972 (97–4,763)	4,574 (3,297–6,001)	3,436 (2,938–3,986)	3,161 (2,481–3,892)	6,278 (4,601–8,547)	10,555 (9,707–11,493)	7,856 (7,415–8,368)	7,614 (6,845–8,376)	9,953 (5,888–14,686)	23,315 (20,379–26,454)	16,506 (14,795–18,279)	11,947 (9,762–14,445)
South Sudan	13,295 (10,234–17,295)	5,829 (4,514–7,508)	16,806 (12,993–21,735)	3,893 (3,019–5,001)	9,622 (7,248–12,860)	2,514 (1,937–3,265)	8,668 (6,634–11,350)	2,753 (2,130–3,548)	9,673 (7,456–12,597)	3,686 (2,858–4,742)	7,793 (6,015–10,133)	4,659 (3,613–5,987)	10,110 (7,578–13,630)	4,573 (3,516–5,950)	10,511 (7,879–14,048)	5,393 (4,165–6,967)
Spain	559 (549–569)	303 (296–309)	187 (182–193)	148 (141–159)	713 (679–750)	368 (348–389)	205 (191–218)	203 (170–239)	2,166 (2,127–2,203)	1,186 (1,161–1,210)	386 (374–398)	447 (417–479)	3,356 (3,228–3,494)	2,012 (1,931–2,099)	603 (573–634)	719 (623–814)
Sri Lanka	1,305 (1,281–1,330)	797 (786–807)	393 (382–403)	223 (203–243)	1,121 (1,062–1,180)	800 (767–833)	467 (439–495)	330 (254–422)	2,904 (2,840–2,970)	2,389 (2,342–2,436)	759 (717–803)	535 (356–741)	5,287 (5,090–5,493)	4,206 (4,052–4,360)	1,068 (950–1,198)	817 (276–2,120)
State of Palestine	193 (150–247)	226 (181–269)	224 (155–312)	4,089 (2,368–6,319)	142 (65–232)	190 (115–271)	212 (70–392)	4,255 (561–10,654)	249 (193–319)	358 (278–459)	332 (258–425)	4,162 (3,240–5,381)	249 (193–319)	434 (337–558)	370 (287–474)	4,104 (3,154–5,385)
Sudan	12,272 (10,734–14,043)	9,923 (8,499–11,563)	6,366 (4,675–8,161)	11,636 (7,131–15,662)	4,500 (1,455–8,485)	4,150 (1,537–7,281)	3,521 (15–7,964)	5,921 (0–19,693)	8,848 (6,905–11,386)	9,561 (7,464–12,299)	10,249 (8,004–13,177)	16,899 (13,118–21,959)	10,282 (7,987–13,319)	10,839 (8,426–14,021)	12,626 (9,830–16,300)	26,696 (20,343–35,196)
Suriname	22 (19–24)	17 (16–18)	17 (15–18)	22 (19–25)	20 (15–25)	19 (15–22)	18 (15–22)	23 (15–32)	43 (38–49)	36 (34–40)	41 (38–45)	47 (40–56)	67 (56–78)	55 (49–61)	53 (46–59)	64 (46–83)

Country	Number of deaths age 5–9				Number of deaths age 10–14				Number of deaths age 15–19				Number of deaths age 20–24			
	1990	2000	2015	2024	1990	2000	2015	2024	1990	2000	2015	2024	1990	2000	2015	2024
Sweden	68 (65–70)	65 (62–67)	39 (37–42)	39 (36–43)	74 (67–82)	72 (65–80)	47 (42–53)	57 (47–69)	244 (237–252)	167 (162–173)	128 (123–133)	124 (116–133)	364 (340–387)	260 (242–276)	285 (265–304)	219 (195–244)
Switzerland	73 (69–76)	52 (49–55)	31 (28–33)	31 (27–35)	74 (68–82)	59 (53–65)	33 (29–37)	31 (23–39)	272 (263–280)	172 (165–178)	98 (94–103)	99 (90–107)	536 (509–566)	267 (250–284)	149 (136–163)	134 (114–154)
Syrian Arab Republic	2,181 (2,089–2,272)	1,616 (1,589–1,643)	4,588 (4,165–5,013)	887 (635–1,145)	1,228 (740–1,808)	1,116 (1,058–1,173)	2,482 (1,574–3,668)	1,076 (415–2,550)	2,373 (1,808–2,896)	1,378 (1,352–1,406)	6,250 (5,142–7,394)	1,760 (1,095–2,466)	1,348 (291–3,248)	1,151 (1,096–1,206)	9,579 (5,300–16,904)	2,715 (581–9,385)
Tajikistan	536 (523–549)	407 (400–415)	259 (251–266)	231 (220–243)	333 (308–356)	342 (322–362)	220 (199–242)	220 (181–264)	391 (375–408)	489 (472–505)	368 (344–392)	293 (251–339)	544 (514–574)	715 (682–749)	521 (465–578)	422 (289–602)
Thailand	3,917 (3,853–3,984)	3,306 (3,275–3,338)	1,667 (1,650–1,685)	1,154 (1,125–1,182)	3,368 (3,134–3,600)	3,115 (2,987–3,241)	2,326 (2,247–2,406)	2,146 (1,983–2,323)	8,506 (8,236–8,780)	7,437 (7,293–7,584)	6,239 (6,125–6,351)	4,093 (3,899–4,296)	10,978 (10,255–11,718)	12,245 (11,819–12,675)	7,014 (6,704–7,311)	6,184 (5,494–6,934)
Timor-Leste	616 (482–790)	231 (181–296)	203 (159–260)	172 (135–220)	274 (213–354)	128 (100–164)	141 (110–180)	115 (90–148)	198 (106–335)	140 (105–186)	336 (241–445)	439 (241–652)	254 (0–729)	144 (72–234)	269 (79–512)	435 (0–1,972)
Togo	2,977 (2,499–3,572)	2,552 (2,213–3,001)	1,745 (1,337–2,228)	1,400 (872–1,941)	894 (139–1,694)	996 (362–1,788)	1,050 (372–1,791)	1,096 (131–2,464)	1,016 (805–1,284)	1,409 (1,142–1,761)	1,298 (861–1,827)	1,300 (666–2,073)	873 (455–1,340)	1,290 (720–2,021)	1,435 (405–2,765)	1,624 (166–5,599)
Tonga	5 (3–6)	7 (6–8)	5 (3–6)	3 (2–3)	3 (1–7)	5 (3–6)	3 (0–5)	2 (0–5)	5 (3–8)	8 (7–10)	7 (6–9)	6 (4–10)	9 (4–16)	9 (7–11)	9 (7–12)	10 (4–22)
Trinidad and Tobago	60 (55–64)	36 (34–38)	23 (21–25)	18 (15–21)	52 (44–61)	48 (42–54)	27 (23–31)	25 (14–39)	93 (86–99)	119 (113–123)	83 (78–88)	93 (72–116)	147 (133–163)	173 (159–185)	164 (149–179)	175 (87–324)
Tunisia	807 (778–837)	474 (462–485)	324 (315–332)	286 (273–300)	590 (461–736)	446 (409–484)	286 (262–311)	307 (256–362)	640 (539–745)	680 (652–707)	459 (437–483)	555 (505–605)	812 (511–1,183)	817 (764–871)	773 (716–830)	571 (439–721)
Turkmenistan	370 (360–381)	418 (411–425)	252 (242–262)	301 (242–361)	242 (222–261)	371 (350–393)	212 (190–235)	279 (151–495)	352 (336–369)	596 (574–619)	487 (460–516)	352 (234–487)	483 (455–513)	869 (834–904)	763 (700–826)	536 (183–1,367)
Turks and Caicos Islands	0 (0–0)	0 (0–0)	0 (0–0)	0 (0–0)	0 (0–0)	0 (0–0)	0 (0–0)	0 (0–0)	1 (1–1)	1 (0–1)	1 (1–2)	1 (1–2)	1 (1–2)	1 (1–2)	1 (1–2)	2 (1–3)
Tuvalu	2 (1–2)	1 (1–2)	0 (0–1)	0 (0–0)	0 (0–0)	0 (0–1)	0 (0–0)	0 (0–0)	1 (0–1)	1 (1–1)	1 (1–1)	1 (0–1)	1 (1–2)	1 (1–1)	1 (1–2)	1 (0–1)
Türkiye	7,953 (7,525–8,363)	5,763 (5,542–5,982)	1,553 (1,533–1,571)	1,181 (1,150–1,213)	3,167 (1,116–5,636)	3,342 (2,002–4,867)	1,496 (1,436–1,557)	1,520 (1,418–1,626)	11,427 (4,876–16,340)	7,006 (5,196–8,877)	3,260 (3,198–3,322)	2,866 (2,767–2,969)	6,264 (0–15,840)	5,219 (1,559–9,872)	3,615 (3,455–3,777)	3,653 (3,382–3,930)
Uganda	10,336 (9,067–11,869)	11,777 (10,582–13,112)	11,011 (9,156–13,128)	9,740 (7,217–12,434)	4,492 (2,418–6,726)	5,228 (3,318–7,472)	5,936 (2,794–9,254)	5,785 (1,370–10,951)	7,107 (5,888–8,575)	8,781 (7,625–10,077)	9,233 (7,437–11,945)	8,117 (5,533–12,737)	9,361 (6,794–12,231)	11,070 (8,558–13,932)	10,355 (6,374–14,952)	10,259 (3,547–19,432)
Ukraine	1,827 (1,809–1,844)	1,120 (1,107–1,132)	460 (451–469)	286 (269–304)	1,501 (1,435–1,566)	1,395 (1,339–1,451)	434 (415–455)	492 (404–599)	3,155 (3,105–3,207)	3,457 (3,394–3,512)	1,160 (1,138–1,184)	3,799 (3,367–4,278)	5,210 (4,991–5,450)	6,147 (5,909–6,410)	2,612 (2,491–2,739)	8,219 (5,708–11,716)
United Arab Emirates	62 (48–80)	55 (43–71)	99 (77–127)	82 (63–105)	53 (41–67)	58 (45–75)	79 (62–102)	65 (51–84)	99 (77–127)	178 (139–229)	201 (156–258)	198 (154–254)	147 (114–188)	264 (205–339)	459 (357–590)	455 (353–584)
United Kingdom	646 (633–659)	451 (439–461)	303 (295–312)	277 (259–293)	686 (660–713)	544 (522–568)	330 (313–347)	383 (337–440)	2,006 (1,979–2,035)	1,566 (1,542–1,589)	930 (913–946)	1,007 (967–1,050)	2,778 (2,665–2,894)	2,121 (2,041–2,202)	1,559 (1,502–1,619)	1,526 (1,365–1,693)
United Republic of Tanzania	14,815 (13,189–16,746)	13,398 (12,076–14,917)	15,275 (12,335–17,954)	16,110 (11,192–19,613)	6,152 (3,128–9,686)	5,298 (2,599–8,114)	5,663 (410–13,810)	6,128 (0–25,779)	7,150 (5,911–8,499)	9,965 (8,505–11,485)	7,236 (5,848–8,805)	8,113 (5,515–11,362)	6,586 (4,169–9,232)	10,816 (7,742–14,418)	7,140 (4,556–10,095)	8,206 (1,830–19,637)
United States	4,077 (4,023–4,130)	3,289 (3,243–3,333)	2,464 (2,431–2,501)	2,493 (2,414–2,569)	4,500 (4,337–4,662)	4,202 (4,048–4,352)	3,078 (2,974–3,185)	3,678 (3,375–3,979)	15,189 (14,967–15,430)	13,785 (13,568–13,992)	10,142 (9,975–10,306)	11,808 (11,340–12,327)	21,453 (20,552–22,378)	18,141 (17,405–18,877)	20,336 (19,619–21,090)	19,941 (17,063–22,889)

Country	Number of deaths age 5–9				Number of deaths age 10–14				Number of deaths age 15–19				Number of deaths age 20–24			
	1990	2000	2015	2024	1990	2000	2015	2024	1990	2000	2015	2024	1990	2000	2015	2024
Uruguay	79 (74–84)	65 (62–68)	38 (35–40)	28 (25–31)	93 (80–105)	74 (68–80)	50 (45–55)	39 (31–51)	198 (188–209)	181 (173–187)	187 (180–195)	175 (155–193)	205 (186–223)	247 (230–266)	264 (245–283)	296 (228–378)
Uzbekistan	1,941 (1,911–1,971)	1,715 (1,697–1,732)	1,101 (1,089–1,114)	2,140 (2,093–2,187)	1,406 (1,335–1,480)	1,512 (1,450–1,573)	932 (896–968)	1,833 (1,657–2,022)	1,775 (1,736–1,814)	2,232 (2,192–2,272)	1,887 (1,854–1,922)	2,400 (2,298–2,503)	2,496 (2,391–2,602)	3,126 (3,007–3,246)	2,669 (2,554–2,785)	2,129 (1,820–2,466)
Vanuatu	17 (14–23)	17 (13–21)	15 (12–19)	17 (13–22)	10 (8–12)	13 (10–16)	11 (8–14)	14 (11–18)	19 (15–24)	20 (16–26)	20 (16–26)	25 (20–32)	20 (15–25)	18 (14–23)	28 (21–35)	27 (21–34)
Venezuela (Bolivarian Republic of)	1,090 (1,067–1,112)	1,004 (992–1,015)	804 (783–827)	646 (492–815)	1,069 (1,002–1,137)	1,123 (1,082–1,164)	1,073 (977–1,173)	1,056 (386–2,067)	2,148 (2,081–2,217)	3,254 (3,194–3,317)	5,341 (5,076–5,614)	4,180 (2,715–5,875)	2,678 (2,508–2,859)	4,659 (4,485–4,838)	8,176 (7,302–9,135)	6,709 (2,345–17,071)
Viet Nam	13,029 (11,156–15,120)	6,509 (5,584–7,514)	2,211 (1,910–2,561)	1,512 (1,124–2,099)	4,271 (1,142–8,250)	3,983 (2,032–5,981)	2,538 (1,953–3,153)	2,760 (1,521–4,742)	8,164 (6,101–10,810)	7,243 (5,585–9,095)	4,415 (3,862–5,056)	3,769 (2,701–4,997)	7,607 (3,422–12,971)	7,807 (4,318–11,534)	6,455 (4,737–8,359)	4,149 (1,534–8,046)
Yemen	5,092 (4,506–5,791)	4,682 (4,148–5,290)	4,498 (3,635–5,491)	4,859 (3,494–6,363)	2,521 (1,431–3,659)	2,843 (1,758–4,001)	3,246 (1,607–5,030)	3,865 (780–8,778)	2,199 (1,713–2,808)	3,093 (2,410–3,948)	4,939 (3,834–6,318)	3,897 (3,037–4,971)	1,804 (1,403–2,308)	2,508 (1,951–3,207)	7,264 (5,619–9,369)	3,798 (2,956–4,851)
Zambia	4,197 (3,629–4,853)	4,474 (3,995–5,208)	3,564 (2,878–4,242)	3,406 (2,528–4,318)	2,029 (1,068–3,056)	2,011 (989–3,203)	1,801 (815–2,839)	1,879 (283–4,086)	3,283 (2,707–3,983)	3,783 (3,242–4,358)	2,829 (2,352–3,412)	2,400 (1,698–3,416)	4,385 (3,188–5,619)	5,761 (4,578–7,092)	3,848 (2,823–4,959)	2,856 (1,189–4,946)
Zimbabwe	2,592 (2,213–3,040)	2,508 (2,230–2,840)	2,518 (2,123–2,965)	1,761 (1,290–2,279)	1,502 (706–2,293)	2,092 (1,233–2,993)	2,549 (1,654–3,483)	2,427 (847–4,565)	1,714 (1,421–2,080)	3,689 (3,146–4,220)	2,823 (2,368–3,370)	3,543 (2,518–5,004)	2,590 (1,901–3,299)	6,197 (5,028–7,605)	3,719 (2,613–4,849)	4,426 (1,429–8,678)

Table A.6: Country annual rate of reduction

Annual rate of reduction (ARR,%) for probability of dying $_5q_5$, probability of dying $_5q_{10}$, probability of dying $_5q_{15}$, and probability of dying $_5q_{20}$ for the periods 1990-2000, 2000-2015, and 2015-2024, by country. Values shown are medians with 90% uncertainty intervals in parentheses.

Country	Annual rate of reduction in probability of dying $_5q_5$ (%)			Annual rate of reduction in probability of dying $_5q_{10}$ (%)			Annual rate of reduction in probability of dying $_5q_{15}$ (%)			Annual rate of reduction in probability of dying $_5q_{20}$ (%)		
	1990-2000	2000-2015	2015-2024	1990-2000	2000-2015	2015-2024	1990-2000	2000-2015	2015-2024	1990-2000	2000-2015	2015-2024
Afghanistan	11.3 (10.4–12.3)	0.8 (-0.2–1.9)	-0.0 (-1.0–1.4)	10.0 (-5.5–26.2)	1.1 (-5.3–7.6)	-1.3 (-19.8–19.5)	9.6 (7.0–11.9)	-2.4 (-5.1—0.1)	2.7 (-0.6–7.1)	10.1 (-1.3–20.4)	-0.8 (-5.6–5.5)	2.3 (-9.5–17.0)
Albania	5.6 (5.2–5.9)	4.3 (3.9–4.7)	4.9 (4.2–5.7)	3.9 (2.3–5.4)	2.5 (1.4–3.7)	3.1 (-1.1–8.3)	-0.4 (-1.2–0.4)	3.1 (2.5–3.6)	4.4 (2.0–7.2)	-1.1 (-2.2—0.1)	5.2 (4.4–6.1)	2.8 (-2.4–9.2)
Algeria	4.1 (4.0–4.3)	4.0 (3.9–4.1)	3.5 (3.2–3.7)	3.1 (0.8–5.1)	2.7 (2.2–3.1)	2.0 (0.7–3.3)	2.9 (2.0–3.7)	2.4 (2.2–2.6)	0.5 (-0.1–1.2)	1.4 (-1.6–4.1)	2.7 (2.2–3.1)	-0.1 (-1.6–1.3)
Andorra	4.8 (4.8–4.8)	4.5 (4.5–4.5)	3.3 (3.3–3.3)	4.3 (4.3–4.3)	4.1 (4.1–4.1)	3.0 (3.0–3.0)	3.7 (3.7–3.7)	3.5 (3.5–3.5)	2.5 (2.5–2.5)	3.6 (3.6–3.6)	3.4 (3.4–3.4)	2.5 (2.5–2.5)
Angola	3.1 (3.1–3.1)	7.7 (7.7–7.7)	2.7 (2.7–2.7)	2.8 (2.7–2.8)	5.8 (5.8–5.8)	2.1 (2.1–2.1)	2.6 (2.6–2.6)	5.9 (5.9–5.9)	1.8 (1.8–1.8)	3.5 (3.5–3.5)	5.7 (5.7–5.7)	1.6 (1.6–1.6)
Anguilla	3.8 (3.8–3.8)	2.5 (2.5–2.5)	2.5 (2.5–2.5)	2.8 (2.8–2.8)	1.9 (1.9–1.9)	1.9 (1.9–1.9)	2.4 (2.4–2.4)	1.6 (1.6–1.6)	1.6 (1.6–1.6)	2.4 (2.4–2.4)	1.6 (1.6–1.6)	1.6 (1.6–1.6)
Antigua and Barbuda	-0.8 (-0.8—0.8)	1.6 (1.6–1.6)	1.7 (1.7–1.7)	-0.6 (-0.6—0.6)	1.2 (1.2–1.2)	1.3 (1.3–1.3)	-4.1 (-9.0—0.4)	2.9 (0.7–5.0)	5.2 (1.5–10.1)	-4.1 (-18.0–4.8)	1.9 (-1.4–5.4)	2.9 (-5.3–13.8)
Argentina	2.4 (2.2–2.5)	2.2 (2.1–2.3)	0.4 (0.0–0.8)	1.7 (1.1–2.3)	1.5 (1.2–1.9)	-0.3 (-2.0–1.3)	-0.7 (-1.0—0.4)	0.3 (0.1–0.4)	2.8 (2.0–3.7)	-0.6 (-1.2–0.0)	0.4 (-0.0–0.8)	1.4 (-1.1–4.0)
Armenia	6.2 (5.8–6.5)	-0.4 (-0.7—0.1)	0.9 (0.5–1.3)	5.6 (3.8–7.4)	-0.9 (-2.2–0.3)	0.4 (-2.5–3.6)	1.8 (0.9–2.8)	-2.3 (-3.0—1.7)	5.4 (3.6–7.3)	5.9 (4.3–7.6)	-2.3 (-3.7—0.9)	1.3 (-1.7–4.4)
Australia	3.8 (3.4–4.0)	2.9 (2.6–3.1)	1.1 (0.6–1.7)	3.4 (2.5–4.1)	2.6 (2.0–3.2)	0.5 (-1.6–2.4)	2.1 (1.9–2.4)	3.8 (3.6–4.0)	1.5 (0.9–2.2)	1.9 (1.3–2.4)	4.0 (3.6–4.5)	0.9 (-0.7–2.6)

Country	Annual rate of reduction in probability of dying $_5q_5$ (%)			Annual rate of reduction in probability of dying $_5q_{10}$ (%)			Annual rate of reduction in probability of dying $_5q_{15}$ (%)			Annual rate of reduction in probability of dying $_5q_{20}$ (%)		
	1990-2000	2000-2015	2015-2024	1990-2000	2000-2015	2015-2024	1990-2000	2000-2015	2015-2024	1990-2000	2000-2015	2015-2024
Austria	4.0 (3.8–4.3)	2.9 (2.6–3.2)	1.9 (1.3–2.3)	3.3 (2.3–4.3)	2.2 (1.4–3.1)	1.3 (-0.7–3.3)	2.6 (2.3–2.9)	4.3 (4.0–4.6)	-1.7 (-2.7—1.1)	2.5 (1.6–3.3)	4.1 (3.4–4.8)	-0.9 (-2.7–1.0)
Azerbaijan	-0.0 (-0.2–0.2)	4.6 (4.5–4.8)	-0.4 (-0.7—0.1)	-1.2 (-2.2—0.1)	3.3 (2.7–4.0)	-1.7 (-3.5–0.3)	-3.4 (-3.9—3.0)	2.2 (1.9–2.5)	-0.1 (-1.0–0.9)	-4.0 (-4.9—3.1)	2.6 (2.0–3.2)	-0.0 (-4.6–5.9)
Bahamas	0.1 (-1.0–1.0)	3.8 (2.4–5.2)	3.9 (2.0–6.2)	-2.3 (-10.9–3.6)	1.3 (-1.6–4.4)	2.1 (-3.4–9.3)	-2.5 (-4.8—0.3)	-0.7 (-1.7–0.3)	7.0 (3.6–10.9)	-1.2 (-4.1–1.4)	-1.8 (-2.9—0.7)	5.8 (-0.3–12.6)
Bahrain	1.5 (-1.1–2.4)	3.4 (2.7–4.5)	1.9 (0.1–3.2)	1.6 (-2.8–5.4)	2.2 (0.1–4.3)	1.1 (-3.5–5.3)	0.8 (-0.5–2.3)	1.6 (0.9–2.3)	2.7 (0.9–4.3)	-0.5 (-3.6–2.0)	1.3 (0.2–2.5)	2.8 (-0.1–6.7)
Bangladesh	8.7 (8.4–9.2)	4.7 (4.3–5.0)	4.0 (2.9–4.7)	6.8 (2.7–10.6)	3.1 (1.0–5.1)	2.8 (-5.1–14.8)	6.2 (4.5–8.0)	-0.3 (-1.6–0.9)	0.9 (-2.6–10.1)	3.8 (1.5–6.4)	3.9 (2.2–5.5)	1.7 (-6.0–13.1)
Barbados	2.2 (-0.2–4.4)	3.3 (0.9–6.1)	3.6 (0.2–7.7)	0.0 (-5.7–4.4)	1.1 (-2.6–5.5)	2.4 (-3.3–8.2)	2.4 (0.3–4.5)	1.8 (-0.4–4.1)	3.0 (-0.5–7.7)	1.2 (-2.3–4.4)	1.8 (-1.6–6.4)	1.3 (-9.6–13.6)
Belarus	3.3 (3.1–3.5)	4.6 (4.4–5.0)	5.9 (4.8–7.3)	2.8 (1.7–3.9)	3.9 (3.0–4.9)	5.0 (-0.5–11.7)	-0.1 (-0.5–0.3)	4.6 (4.3–5.0)	4.6 (2.4–7.1)	-2.0 (-2.5—1.4)	5.7 (5.2–6.2)	5.9 (-1.1–14.0)
Belgium	4.0 (3.8–4.3)	3.3 (2.8–3.6)	2.7 (1.9–3.1)	3.3 (2.5–4.1)	3.0 (2.2–3.7)	2.5 (0.7–4.3)	1.9 (1.6–2.2)	4.6 (4.4–4.9)	2.7 (2.0–3.3)	1.4 (0.7–2.1)	4.3 (3.8–4.9)	2.7 (1.4–4.1)
Belize	-0.2 (-1.0–0.5)	3.1 (2.2–3.9)	4.4 (3.5–5.4)	-1.0 (-5.5–2.8)	2.2 (0.3–4.2)	3.5 (-0.9–8.6)	-7.7 (-10.5—5.0)	0.5 (-0.3–1.3)	1.2 (-0.9–3.4)	-6.6 (-10.6—3.2)	1.1 (0.1–2.2)	0.5 (-2.4–3.7)
Benin	3.1 (2.5–3.8)	2.5 (1.7–3.2)	2.4 (1.5–3.5)	2.3 (-2.1–6.4)	1.9 (-1.6–5.6)	1.8 (-3.8–10.0)	3.1 (0.8–5.5)	-0.3 (-2.6–1.7)	0.9 (-1.8–4.7)	1.9 (-4.0–6.7)	0.4 (-3.8–5.5)	0.6 (-10.5–15.6)
Bhutan	2.4 (-0.5–3.3)	7.9 (3.2–13.5)	4.2 (1.3–9.2)	-5.6 (-20.1–13.6)	-0.8 (-13.1–9.0)	2.0 (-4.5–9.2)	5.6 (1.0–8.8)	2.8 (-0.3–6.5)	2.7 (-0.4–7.2)	5.0 (-12.6–17.5)	1.1 (-6.8–11.3)	1.0 (-12.2–15.1)
Bolivia (Plurinational State of)	4.8 (3.1–6.3)	4.1 (2.3–6.4)	3.5 (1.0–7.0)	2.9 (-2.8–8.2)	2.5 (-3.4–11.2)	2.4 (-5.9–11.1)	2.9 (0.9–5.0)	3.2 (1.1–6.5)	2.8 (-0.3–7.4)	4.0 (-1.6–8.4)	2.8 (-5.0–13.5)	1.0 (-12.8–15.1)
Bosnia and Herzegovina	5.8 (5.2–6.3)	2.1 (1.6–2.7)	-3.6 (-4.2—2.8)	4.6 (2.5–6.6)	0.9 (-0.6–2.4)	-3.4 (-7.5–1.4)	5.1 (4.2–6.0)	-0.4 (-1.0–0.3)	-0.6 (-2.5–1.5)	5.9 (4.7–7.1)	-1.0 (-1.9—0.0)	0.9 (-2.3–4.5)
Botswana	4.4 (2.9–5.5)	4.8 (3.2–6.6)	5.0 (3.1–8.4)	2.6 (-6.5–9.4)	2.4 (-3.8–7.0)	2.4 (-3.6–10.3)	3.0 (0.8–6.2)	4.0 (1.3–5.8)	1.4 (-2.4–4.8)	1.4 (-3.6–5.3)	4.7 (1.3–9.2)	1.9 (-7.2–11.1)
Brazil	1.8 (1.6–1.9)	1.7 (1.6–1.8)	0.8 (0.6–1.0)	1.2 (0.3–2.1)	1.1 (0.8–1.4)	0.7 (-0.0–1.4)	-0.6 (-1.1—0.1)	-1.3 (-1.5—1.1)	4.4 (4.0–4.8)	-0.1 (-1.2–0.9)	-0.4 (-0.7–0.0)	1.7 (0.9–2.5)
British Virgin Islands	2.5 (2.5–2.5)	0.5 (0.5–0.5)	2.0 (2.0–2.0)	1.8 (1.8–1.8)	0.3 (0.3–0.3)	1.5 (1.5–1.5)	1.6 (1.6–1.6)	0.3 (0.3–0.3)	1.3 (1.3–1.3)	1.6 (1.6–1.6)	0.3 (0.3–0.3)	1.3 (1.3–1.3)
Brunei Darussalam	2.7 (2.0–3.4)	1.7 (1.1–2.3)	1.8 (1.2–2.5)	3.2 (0.4–6.1)	2.2 (-0.1–4.7)	2.3 (-3.3–9.6)	3.2 (1.1–5.3)	4.7 (3.5–5.9)	-1.6 (-4.2–1.3)	2.7 (-0.8–5.9)	3.1 (1.3–4.9)	0.5 (-4.3–6.0)
Bulgaria	2.3 (2.1–2.5)	3.9 (3.7–4.3)	3.3 (3.0–4.0)	1.6 (0.7–2.9)	3.0 (2.2–4.0)	2.4 (-0.5–4.6)	1.9 (1.6–2.3)	1.7 (1.3–2.0)	0.8 (0.1–1.7)	1.3 (0.6–1.9)	1.8 (1.2–2.4)	0.3 (-1.2–1.8)
Burkina Faso	1.1 (0.2–2.0)	6.5 (5.5–7.9)	7.2 (6.2–9.3)	-0.2 (-10.2–7.9)	4.8 (-2.3–13.5)	5.8 (-5.1–21.2)	2.8 (1.5–4.2)	3.9 (2.6–5.2)	0.9 (-1.5–3.6)	2.6 (-0.8–5.4)	3.4 (1.0–6.2)	-1.4 (-7.7–6.0)
Burundi	0.3 (-0.6–1.1)	7.3 (6.4–8.5)	2.6 (1.0–4.9)	-0.9 (-8.9–5.4)	6.1 (0.7–12.8)	1.6 (-9.6–17.3)	0.7 (-1.4–3.1)	7.5 (5.7–9.8)	2.1 (-0.6–5.9)	1.5 (-9.5–8.9)	9.4 (4.6–15.5)	1.3 (-10.9–18.5)
Cabo Verde	1.7 (0.9–2.5)	4.1 (3.3–4.9)	3.7 (2.9–4.7)	1.1 (-2.9–4.5)	3.5 (1.6–5.5)	3.1 (-1.8–9.3)	-2.0 (-4.2–0.3)	1.7 (0.4–2.9)	2.6 (-0.1–5.9)	-1.0 (-4.2–1.9)	2.2 (0.3–4.4)	4.6 (-1.5–13.3)
Cambodia	6.2 (4.2–7.7)	8.4 (6.8–11.2)	4.2 (2.5–7.3)	5.5 (-7.5–13.3)	6.8 (-0.3–17.9)	3.2 (-7.7–14.2)	7.1 (4.5–10.1)	3.7 (1.6–5.7)	-0.1 (-3.0–3.3)	5.5 (-0.8–10.3)	5.1 (1.6–9.7)	1.9 (-9.6–18.5)
Cameroon	0.9 (0.1–1.6)	2.1 (1.3–3.2)	2.9 (1.6–5.0)	0.2 (-4.3–3.9)	1.1 (-2.6–6.9)	2.0 (-4.4–12.8)	-0.2 (-1.8–1.4)	0.7 (-1.3–2.5)	1.7 (-1.3–5.6)	-1.3 (-5.5–1.5)	0.6 (-2.1–4.4)	0.9 (-7.8–12.3)

Country	Annual rate of reduction in probability of dying $_5q_5$ (%)			Annual rate of reduction in probability of dying $_5q_{10}$ (%)			Annual rate of reduction in probability of dying $_5q_{15}$ (%)			Annual rate of reduction in probability of dying $_5q_{20}$ (%)		
	1990-2000	2000-2015	2015-2024	1990-2000	2000-2015	2015-2024	1990-2000	2000-2015	2015-2024	1990-2000	2000-2015	2015-2024
Canada	4.3 (4.1–4.5)	3.0 (2.7–3.2)	-1.0 (-1.6–-0.6)	3.4 (2.7–4.2)	2.3 (1.8–2.9)	-1.5 (-3.4–0.3)	3.2 (3.0–3.4)	2.6 (2.4–2.8)	-0.0 (-0.6–0.5)	2.8 (2.2–3.4)	1.2 (0.8–1.6)	-1.4 (-2.8–0.1)
Central African Republic	1.0 (-0.0–2.3)	-0.7 (-1.9–0.8)	4.9 (3.5–7.4)	0.4 (-6.7–6.2)	-1.1 (-7.6–5.6)	3.3 (-4.5–15.1)	-0.0 (-3.0–2.7)	0.2 (-2.6–3.9)	5.9 (1.9–11.3)	-0.3 (-5.8–6.2)	-1.3 (-9.1–8.3)	4.4 (-7.0–16.0)
Chad	3.4 (2.3–4.2)	3.4 (2.2–4.8)	3.6 (2.1–5.9)	1.2 (-7.8–6.2)	0.9 (-3.5–5.1)	1.6 (-3.5–8.8)	2.5 (0.5–4.6)	1.3 (-1.1–3.8)	2.6 (-0.5–6.9)	4.6 (-0.3–8.7)	-0.1 (-4.3–6.1)	1.0 (-11.3–15.3)
Chile	4.0 (3.9–4.2)	3.0 (2.8–3.2)	0.9 (0.4–1.2)	3.2 (2.5–4.0)	2.3 (1.7–2.9)	0.3 (-1.2–1.9)	2.4 (2.1–2.7)	1.3 (1.0–1.5)	0.2 (-0.3–0.8)	2.1 (1.5–2.7)	1.8 (1.4–2.3)	-1.1 (-2.0–-0.1)
China	6.1 (5.1–7.1)	5.1 (3.8–6.6)	3.2 (0.9–6.7)	4.1 (-1.9–8.1)	3.5 (-0.9–7.5)	2.3 (-4.7–10.4)	4.4 (3.1–5.7)	4.8 (3.0–6.8)	2.9 (-1.1–7.9)	0.9 (-4.7–5.5)	1.5 (-3.0–6.4)	1.0 (-9.6–12.5)
Colombia	1.5 (1.4–1.7)	2.7 (2.6–2.8)	1.8 (1.6–2.0)	1.1 (0.5–1.6)	2.3 (1.9–2.6)	1.2 (0.4–1.9)	-2.0 (-2.3–-1.7)	4.3 (4.1–4.4)	1.6 (1.2–2.0)	-1.7 (-2.3–-1.2)	4.6 (4.2–4.9)	0.2 (-0.6–1.0)
Comoros	3.1 (2.0–4.1)	3.9 (2.8–5.7)	2.0 (0.8–4.6)	2.4 (-7.3–8.8)	2.8 (-4.3–9.9)	0.9 (-6.9–13.0)	4.2 (2.3–6.7)	3.6 (1.1–5.7)	0.7 (-2.4–4.0)	3.0 (-4.4–8.7)	3.6 (-1.5–9.4)	0.9 (-8.4–14.3)
Congo	5.7 (1.4–7.0)	7.2 (4.3–11.0)	4.0 (1.8–7.7)	2.7 (-13.3–16.3)	3.8 (-6.2–14.3)	2.8 (-6.2–12.2)	6.2 (0.7–9.9)	6.7 (3.0–10.9)	3.2 (-0.8–8.4)	4.0 (-10.7–11.9)	4.4 (-0.5–10.8)	1.4 (-9.0–12.3)
Cook Islands	2.2 (2.2–2.2)	2.0 (2.0–2.0)	0.0 (0.0–0.0)	1.8 (1.8–1.8)	1.6 (1.6–1.6)	0.0 (0.0–0.0)	-1.3 (-5.2–2.2)	2.4 (0.8–5.4)	2.3 (0.2–6.1)	3.1 (-4.4–11.9)	5.1 (-6.4–18.3)	0.6 (-17.1–18.0)
Costa Rica	2.3 (1.8–2.7)	2.2 (1.8–2.6)	-0.2 (-0.9–0.4)	1.4 (0.0–2.7)	1.4 (0.6–2.3)	-0.9 (-3.4–1.6)	-1.2 (-1.8–-0.6)	0.7 (0.4–1.0)	-2.5 (-3.3–-1.8)	-1.5 (-2.6–-0.3)	-0.1 (-0.8–0.5)	-3.5 (-4.7–-2.2)
Croatia	5.4 (5.0–5.7)	2.0 (1.7–2.3)	-5.5 (-6.0–-4.9)	5.8 (4.1–7.6)	2.6 (1.1–4.2)	-5.6 (-10.8–1.0)	2.9 (2.2–3.6)	3.7 (3.1–4.3)	-5.8 (-7.7–-3.7)	5.5 (4.5–6.5)	3.7 (2.9–4.6)	-1.7 (-7.3–5.5)
Cuba	3.2 (2.5–3.5)	2.4 (2.2–2.9)	-2.0 (-3.1–-1.6)	3.4 (2.4–4.5)	2.4 (1.6–3.0)	-1.6 (-3.4–0.4)	4.5 (4.1–4.9)	2.8 (2.5–3.1)	-3.0 (-3.6–-2.2)	3.6 (3.0–4.3)	3.0 (2.5–3.5)	-3.3 (-4.5–-2.2)
Cyprus	0.3 (-0.5–1.0)	3.8 (2.9–4.8)	3.2 (2.2–4.4)	-1.2 (-6.4–3.0)	2.2 (-0.3–4.7)	1.6 (-3.8–8.2)	-1.5 (-3.2–0.1)	7.8 (6.8–8.8)	4.4 (0.7–8.2)	-1.6 (-4.4–0.8)	5.2 (3.9–6.6)	-0.6 (-4.6–4.0)
Czechia	4.1 (3.6–4.7)	4.5 (4.0–4.9)	0.2 (-1.1–1.4)	2.4 (1.0–3.8)	3.8 (2.8–4.9)	0.8 (-2.1–4.0)	1.8 (1.6–2.1)	3.0 (2.8–3.2)	1.6 (1.0–2.2)	1.4 (0.7–2.0)	2.4 (1.8–2.9)	0.9 (-0.4–2.2)
Côte d'Ivoire	2.0 (1.0–2.8)	1.8 (0.8–2.9)	3.3 (2.1–5.6)	1.3 (-3.3–5.7)	1.1 (-2.9–5.1)	2.1 (-3.3–13.8)	-0.2 (-2.2–1.6)	2.2 (0.1–3.9)	3.4 (0.7–6.9)	-0.2 (-4.6–3.3)	3.1 (0.1–7.1)	3.6 (-3.6–15.1)
Democratic People's Republic of Korea	-8.3 (-8.3–-8.3)	9.5 (9.5–9.5)	2.5 (2.5–2.5)	-6.3 (-6.4–-6.3)	7.3 (7.3–7.3)	2.0 (2.0–2.0)	-4.9 (-4.9–-4.9)	5.7 (5.7–5.7)	1.5 (1.5–1.5)	-4.5 (-4.5–-4.5)	5.2 (5.2–5.2)	1.4 (1.4–1.4)
Democratic Republic of the Congo	-0.7 (-1.5–0.1)	5.0 (4.0–6.2)	3.0 (1.9–5.1)	-2.9 (-13.0–2.7)	3.7 (-1.1–7.9)	1.3 (-3.3–9.3)	0.0 (-2.8–2.1)	2.7 (0.9–4.4)	-0.2 (-2.7–2.4)	0.4 (-8.6–8.0)	2.6 (-0.7–7.0)	-0.0 (-8.7–7.8)
Denmark	3.9 (3.6–4.3)	4.7 (4.1–5.1)	1.3 (0.2–1.9)	2.9 (1.4–4.4)	4.2 (3.0–5.8)	0.9 (-2.6–4.6)	0.9 (0.2–1.4)	5.0 (4.6–5.6)	1.1 (-0.3–2.2)	1.3 (0.3–2.4)	4.1 (3.2–5.0)	1.0 (-1.0–3.2)
Djibouti	1.7 (1.7–1.7)	2.9 (2.9–2.9)	3.4 (3.4–3.4)	1.2 (1.2–1.3)	2.2 (2.2–2.2)	2.6 (2.6–2.6)	1.1 (1.1–1.1)	1.9 (1.9–1.9)	2.2 (2.2–2.2)	1.0 (1.0–1.0)	1.7 (1.7–1.7)	2.0 (2.0–2.0)
Dominica	3.4 (1.7–4.0)	6.6 (4.6–9.6)	8.1 (6.3–14.7)	-0.9 (-18.3–15.5)	1.0 (-9.9–12.0)	4.2 (-4.6–15.6)	2.5 (-1.6–5.6)	2.4 (0.3–4.6)	-1.0 (-5.1–3.7)	-1.2 (-15.8–7.9)	-0.9 (-3.8–1.9)	-2.2 (-9.5–6.9)
Dominican Republic	3.9 (2.9–4.9)	2.8 (1.5–4.2)	3.0 (0.7–6.0)	2.3 (-2.1–6.3)	1.3 (-4.3–6.8)	2.0 (-4.6–10.0)	-1.5 (-5.7–1.4)	2.6 (-0.2–5.8)	3.1 (-0.7–8.2)	-2.7 (-14.6–3.9)	0.1 (-4.8–7.3)	1.2 (-9.4–13.0)
Ecuador	4.1 (3.9–4.2)	3.2 (3.0–3.3)	0.4 (0.1–0.6)	3.0 (2.1–3.8)	2.1 (1.7–2.6)	-0.6 (-1.8–0.7)	0.8 (0.4–1.3)	1.5 (1.3–1.8)	-6.2 (-6.8–-5.6)	0.4 (-0.5–1.2)	1.5 (1.1–2.0)	-8.3 (-9.5–-7.1)
Egypt	7.2 (7.0–7.3)	2.1 (2.1–2.2)	2.6 (2.1–3.1)	6.4 (5.8–7.0)	1.6 (1.2–2.0)	2.0 (-0.1–4.3)	4.5 (4.2–4.8)	0.5 (0.3–0.7)	0.1 (-1.1–1.4)	4.2 (3.6–4.9)	0.4 (0.0–0.9)	-0.6 (-4.7–3.8)
El Salvador	6.4 (6.1–6.6)	2.0 (1.7–2.2)	-2.9 (-4.6–-1.1)	4.3 (3.5–5.2)	-0.2 (-0.8–0.3)	-4.7 (-8.5–-1.0)	4.3 (3.9–4.7)	-1.4 (-1.6–-1.1)	8.7 (6.0–12.0)	4.8 (4.3–5.4)	0.9 (0.6–1.3)	3.9 (-3.2–11.6)

Country	Annual rate of reduction in probability of dying $_5q_5$ (%)			Annual rate of reduction in probability of dying $_5q_{10}$ (%)			Annual rate of reduction in probability of dying $_5q_{15}$ (%)			Annual rate of reduction in probability of dying $_5q_{20}$ (%)		
	1990-2000	2000-2015	2015-2024	1990-2000	2000-2015	2015-2024	1990-2000	2000-2015	2015-2024	1990-2000	2000-2015	2015-2024
Equatorial Guinea	1.5 (1.5–1.5)	3.9 (3.9–3.9)	3.8 (3.8–3.8)	0.9 (0.9–0.9)	2.5 (2.5–2.5)	2.5 (2.5–2.6)	0.9 (0.9–0.9)	2.3 (2.3–2.3)	2.3 (2.3–2.3)	0.8 (0.8–0.8)	2.0 (2.0–2.0)	2.0 (2.0–2.0)
Eritrea	7.9 (6.1–9.4)	7.8 (6.1–10.3)	3.4 (1.1–6.8)	5.2 (-5.7–14.5)	5.1 (-3.8–15.0)	2.4 (-7.2–11.9)	3.5 (3.5–3.5)	3.9 (3.9–3.9)	2.3 (2.3–2.3)	3.4 (3.4–3.4)	4.0 (4.0–4.0)	2.1 (2.1–2.1)
Estonia	4.6 (4.2–4.9)	5.9 (5.4–6.3)	2.9 (2.4–3.5)	3.7 (1.7–5.7)	4.9 (3.3–6.7)	2.0 (-2.6–7.2)	2.8 (2.0–3.6)	4.7 (3.9–5.4)	0.2 (-2.0–2.6)	0.5 (-0.6–1.6)	5.8 (4.8–6.9)	3.1 (-1.0–7.8)
Eswatini	0.8 (-2.7–1.6)	2.9 (-0.7–6.2)	4.5 (0.6–10.8)	-5.3 (-20.7–12.0)	-3.8 (-13.1–4.0)	-0.9 (-6.7–8.0)	2.3 (2.3–2.3)	-0.1 (-0.1—0.1)	1.0 (1.0–1.0)	2.1 (2.1–2.1)	-0.1 (-0.1—0.1)	0.9 (0.9–0.9)
Ethiopia	6.3 (4.9–7.3)	9.5 (8.0–11.3)	5.0 (3.4–7.6)	4.7 (-6.3–11.2)	7.3 (1.5–15.6)	4.0 (-3.7–16.3)	3.7 (1.8–5.8)	5.4 (3.3–7.9)	3.3 (0.4–7.3)	5.5 (1.3–9.5)	6.6 (2.1–13.8)	2.0 (-10.5–16.4)
Fiji	4.7 (4.1–5.2)	4.4 (3.8–5.1)	0.1 (-1.1–1.5)	3.2 (-5.2–8.6)	3.0 (1.4–4.5)	-1.2 (-5.7–3.7)	1.8 (-5.0–5.0)	2.4 (1.6–3.2)	1.0 (-1.8–3.9)	2.0 (-11.6–24.4)	1.9 (0.8–3.1)	0.3 (-6.7–7.9)
Finland	2.8 (2.5–3.1)	4.0 (3.6–4.3)	0.8 (0.2–1.3)	2.1 (0.5–3.6)	3.3 (2.0–4.7)	0.1 (-3.7–3.8)	3.5 (3.2–3.9)	3.1 (2.8–3.4)	-1.2 (-2.0—0.5)	2.6 (1.6–3.7)	2.5 (1.7–3.4)	-1.4 (-3.1–0.4)
France	4.1 (3.9–4.3)	3.4 (3.1–3.6)	0.4 (-0.1–1.0)	3.5 (2.8–4.1)	3.6 (3.1–4.1)	0.3 (-1.4–2.0)	2.1 (1.8–2.4)	4.8 (4.6–5.1)	0.1 (-0.6–0.8)	3.0 (2.5–3.5)	4.0 (3.6–4.3)	-0.4 (-1.6–0.7)
Gabon	1.2 (-2.6–3.7)	2.8 (0.3–6.1)	3.1 (1.2–6.4)	1.9 (-10.2–8.7)	2.9 (-3.8–13.0)	2.7 (-5.9–13.0)	-1.8 (-6.2–1.9)	3.8 (0.9–7.5)	3.0 (-0.0–7.4)	0.6 (-8.8–7.5)	4.4 (-1.7–14.1)	1.4 (-11.5–16.1)
Gambia	4.3 (4.3–4.3)	4.8 (4.8–4.8)	4.1 (4.1–4.1)	2.7 (2.7–2.8)	3.2 (3.2–3.3)	2.9 (2.9–2.9)	2.6 (2.6–2.6)	2.9 (2.9–2.9)	2.5 (2.5–2.5)	2.2 (2.2–2.3)	2.5 (2.5–2.5)	2.1 (2.1–2.1)
Georgia	4.4 (4.1–4.7)	-0.7 (-1.0—0.4)	5.6 (5.2–6.1)	3.3 (1.5–4.9)	-1.9 (-2.9—0.9)	4.4 (2.1–7.0)	2.2 (1.4–3.0)	-1.7 (-2.2—1.1)	2.3 (1.0–3.8)	3.0 (1.8–4.2)	-1.2 (-2.1—0.3)	2.5 (0.4–4.8)
Germany	5.3 (5.0–5.7)	2.8 (2.5–3.0)	-1.1 (-1.7—0.5)	3.6 (3.0–4.3)	2.8 (2.3–3.3)	-0.6 (-2.5–1.2)	2.2 (2.0–2.4)	4.4 (4.3–4.6)	-0.6 (-1.1—0.1)	2.0 (1.4–2.5)	4.1 (3.8–4.5)	0.0 (-1.3–1.4)
Ghana	3.1 (2.3–3.9)	3.4 (2.6–4.3)	2.8 (1.7–4.2)	2.0 (-2.4–5.5)	2.2 (-0.9–5.9)	1.6 (-4.6–9.1)	3.2 (-2.4–8.9)	0.9 (-2.8–5.1)	2.4 (-0.6–7.1)	2.4 (-11.1–12.0)	2.5 (-4.4–12.1)	1.1 (-11.4–15.5)
Greece	2.9 (2.7–3.1)	3.1 (2.7–3.3)	4.1 (3.6–4.5)	2.5 (1.4–3.5)	2.8 (2.0–3.6)	3.7 (1.5–6.8)	1.2 (0.6–1.6)	4.3 (4.0–4.7)	0.2 (-0.9–1.2)	1.1 (0.5–1.9)	3.5 (2.9–4.1)	0.4 (-1.5–2.5)
Grenada	2.1 (0.5–3.2)	-0.2 (-1.9–1.7)	-0.3 (-2.0–2.2)	0.2 (-8.6–6.3)	-2.3 (-6.0–1.4)	-2.2 (-8.6–6.4)	0.7 (-2.3–3.7)	0.5 (-1.3–2.2)	2.0 (-0.7–5.8)	3.5 (-1.3–7.9)	1.7 (-1.2–5.0)	2.6 (-6.5–16.0)
Guatemala	8.3 (8.1–8.4)	3.8 (3.7–3.9)	1.6 (1.3–1.8)	6.0 (5.3–6.7)	1.9 (1.5–2.2)	0.1 (-0.9–1.2)	1.7 (1.5–2.1)	0.4 (0.2–0.6)	2.4 (1.8–3.0)	1.0 (0.5–1.6)	1.7 (1.4–2.1)	0.5 (-0.8–1.8)
Guinea	3.4 (2.6–4.4)	2.7 (1.9–4.1)	2.6 (1.5–4.6)	3.5 (-2.3–8.7)	2.5 (-2.2–8.4)	2.2 (-5.7–14.0)	-0.3 (-2.8–2.0)	1.2 (-1.1–3.8)	2.6 (-0.6–7.1)	-1.3 (-8.8–3.8)	-0.5 (-4.8–6.0)	0.9 (-11.0–14.3)
Guinea-Bissau	4.7 (1.4–5.2)	5.8 (3.0–8.5)	5.2 (3.1–9.1)	-0.5 (-12.3–27.5)	0.5 (-12.6–11.6)	2.3 (-5.7–14.2)	1.7 (1.7–1.7)	2.9 (2.9–2.9)	2.3 (2.3–2.3)	1.4 (1.4–1.4)	2.5 (2.5–2.5)	2.0 (2.0–2.0)
Guyana	2.9 (2.4–3.3)	0.6 (0.0–1.1)	4.2 (2.9–5.6)	1.5 (-0.5–3.4)	-0.8 (-1.9–0.4)	2.9 (-1.4–7.7)	0.4 (-0.7–1.6)	-1.3 (-1.9—0.7)	2.9 (0.4–5.6)	-0.9 (-2.5–0.7)	0.3 (-0.7–1.3)	3.5 (-6.2–15.3)
Haiti	3.6 (2.7–4.7)	3.8 (2.7–5.1)	3.3 (1.6–5.8)	2.8 (-2.1–7.4)	3.0 (-1.2–10.5)	2.6 (-5.3–12.6)	4.2 (1.2–7.1)	3.2 (0.6–6.0)	1.2 (-2.2–5.8)	3.1 (-3.1–8.2)	2.3 (-1.6–6.9)	-0.8 (-10.4–10.8)
Honduras	5.5 (4.3–6.7)	2.6 (0.9–4.7)	2.7 (0.4–6.0)	2.3 (-4.4–7.9)	-1.0 (-6.1–4.3)	0.6 (-5.8–7.3)	2.3 (2.3–2.3)	2.1 (2.1–2.1)	1.8 (1.8–1.8)	2.3 (2.3–2.3)	2.1 (2.1–2.1)	1.7 (1.7–1.7)
Hungary	3.2 (3.0–3.9)	3.5 (3.1–3.9)	2.1 (-0.4–2.8)	2.5 (1.6–3.3)	3.2 (2.4–4.1)	2.7 (-0.1–6.0)	4.6 (4.3–4.9)	2.9 (2.5–3.2)	1.6 (0.6–2.9)	4.6 (3.9–5.3)	2.9 (2.3–3.6)	-0.3 (-2.4–2.1)

Country	Annual rate of reduction in probability of dying $_5q_5$ (%)			Annual rate of reduction in probability of dying $_5q_{10}$ (%)			Annual rate of reduction in probability of dying $_5q_{15}$ (%)			Annual rate of reduction in probability of dying $_5q_{20}$ (%)		
	1990-2000	2000-2015	2015-2024	1990-2000	2000-2015	2015-2024	1990-2000	2000-2015	2015-2024	1990-2000	2000-2015	2015-2024
Iceland	4.1 (3.2–5.0)	5.6 (4.6–6.9)	5.6 (4.0–7.7)	1.7 (-2.2–5.6)	3.3 (0.2–6.7)	3.7 (-1.6–10.0)	3.5 (2.0–5.0)	3.8 (2.4–5.2)	1.5 (-2.1–5.5)	1.1 (-2.2–4.4)	3.3 (0.9–5.9)	0.1 (-6.1–8.4)
India	4.2 (3.7–4.5)	6.3 (6.0–6.7)	6.7 (6.0–7.9)	2.2 (0.0–4.1)	3.9 (2.7–5.1)	4.2 (1.2–7.3)	1.6 (1.1–2.2)	4.0 (3.7–4.4)	3.3 (1.8–4.8)	1.2 (-0.5–2.8)	3.6 (2.6–4.7)	2.9 (-2.0–7.9)
Indonesia	4.1 (3.4–5.0)	3.7 (2.8–4.8)	3.0 (1.5–5.1)	2.8 (-0.4–6.0)	2.6 (-1.1–6.8)	2.2 (-4.3–10.7)	0.8 (-0.8–2.5)	0.9 (-1.0–3.3)	2.3 (-0.6–6.7)	0.7 (-4.6–5.0)	0.3 (-5.8–8.9)	0.8 (-12.1–16.1)
Iran (Islamic Republic of)	10.6 (10.2–10.9)	1.3 (1.0–1.6)	3.5 (1.9–5.3)	9.8 (4.8–13.4)	0.6 (-1.6–2.6)	2.7 (-2.0–7.9)	8.2 (5.9–10.5)	-0.9 (-2.2–0.4)	-0.7 (-3.2–2.3)	6.1 (-2.3–11.2)	1.0 (-1.8–3.5)	-0.7 (-9.7–9.7)
Iraq	13.9 (12.7–15.3)	1.6 (0.0–3.4)	2.7 (0.3–6.1)	9.9 (-2.4–18.8)	0.4 (-7.4–8.2)	1.8 (-5.7–10.6)	11.7 (11.7–11.7)	1.2 (1.2–1.2)	1.4 (1.4–1.4)	16.1 (16.1–16.1)	1.0 (1.0–1.0)	1.2 (1.2–1.2)
Ireland	3.7 (3.3–4.1)	4.3 (3.6–4.8)	3.7 (2.4–4.4)	2.7 (0.9–3.8)	3.7 (2.7–5.2)	3.0 (-0.7–6.0)	-1.2 (-1.9—0.5)	5.7 (5.1–6.2)	0.5 (-1.3–2.5)	-1.3 (-2.3—0.3)	4.6 (3.9–5.4)	3.2 (-0.4–7.3)
Israel	2.9 (2.7–3.2)	3.1 (2.9–3.4)	2.5 (2.1–3.0)	2.4 (1.2–3.5)	2.5 (1.7–3.4)	1.9 (-0.7–4.1)	0.9 (0.6–1.4)	3.2 (2.9–3.5)	-0.1 (-0.8–0.9)	-0.1 (-1.2–1.1)	3.4 (2.6–4.2)	-0.2 (-2.7–2.7)
Italy	3.3 (3.1–3.4)	3.7 (3.4–3.8)	0.3 (-0.1–0.6)	2.7 (2.0–3.5)	3.3 (2.6–3.9)	-0.1 (-2.6–2.4)	2.6 (2.4–2.8)	4.3 (4.1–4.4)	1.3 (0.7–1.8)	1.9 (1.4–2.5)	4.2 (3.8–4.5)	1.3 (-0.1–2.8)
Jamaica	2.5 (2.5–2.5)	0.5 (0.5–0.5)	0.5 (0.5–0.5)	1.8 (1.8–1.8)	0.4 (0.4–0.4)	0.3 (0.3–0.3)	1.6 (1.6–1.6)	0.3 (0.3–0.3)	0.3 (0.3–0.3)	1.6 (1.6–1.6)	0.3 (0.3–0.3)	0.3 (0.3–0.3)
Japan	3.6 (3.4–3.7)	3.3 (3.1–3.4)	0.8 (0.4–1.4)	2.5 (1.9–3.1)	2.0 (1.5–2.5)	-0.9 (-2.1–0.2)	2.6 (2.3–2.8)	3.3 (3.2–3.5)	-2.2 (-2.6—1.8)	1.4 (0.8–1.9)	1.7 (1.3–2.0)	-0.5 (-1.2–0.2)
Jordan	2.1 (1.6–2.6)	3.5 (3.0–3.9)	6.7 (6.3–7.2)	2.1 (-2.5–5.6)	3.4 (1.9–4.8)	6.7 (4.6–9.0)	1.3 (-1.6–3.9)	2.7 (1.8–3.5)	4.0 (3.0–5.0)	-0.2 (-6.6–4.3)	4.1 (2.4–5.6)	5.6 (3.6–7.8)
Kazakhstan	2.0 (1.8–2.1)	3.5 (3.3–3.6)	2.1 (1.8–2.3)	1.1 (0.5–1.8)	2.8 (2.4–3.2)	1.5 (0.2–2.8)	-0.6 (-0.9—0.4)	3.7 (3.6–3.9)	2.0 (1.3–2.6)	-2.9 (-3.4—2.4)	5.4 (5.1–5.7)	3.4 (2.1–4.8)
Kenya	0.7 (-0.0–1.5)	5.4 (4.5–6.4)	7.3 (6.0–8.9)	-1.1 (-8.7–5.2)	3.7 (-1.2–9.1)	5.7 (-2.7–21.7)	-1.8 (-4.2–0.6)	2.7 (0.2–5.2)	2.9 (-0.5–7.6)	-2.0 (-6.8–2.1)	3.4 (-1.1–10.4)	1.3 (-9.8–14.2)
Kiribati	3.0 (3.0–3.0)	0.7 (0.7–0.7)	1.0 (1.0–1.0)	2.3 (2.3–2.3)	0.5 (0.5–0.5)	0.8 (0.8–0.8)	1.8 (1.8–1.8)	0.4 (0.4–0.4)	0.6 (0.6–0.6)	1.6 (1.6–1.6)	0.4 (0.4–0.4)	0.6 (0.5–0.6)
Kosovo (UNSCR 1244)	4.6 (4.6–4.6)	4.8 (4.8–4.8)	3.3 (3.3–3.3)	3.6 (3.6–3.6)	3.8 (3.8–3.8)	2.6 (2.6–2.6)	2.4 (2.4–2.4)	2.6 (2.6–2.6)	1.7 (1.7–1.7)	2.7 (2.7–2.7)	2.8 (2.8–2.8)	1.9 (1.9–1.9)
Kuwait	7.8 (7.3–8.3)	2.5 (2.1–2.9)	1.7 (1.0–2.3)	6.0 (3.4–8.5)	1.9 (0.6–3.2)	1.2 (-2.6–5.4)	5.6 (4.4–6.9)	1.5 (0.8–2.0)	4.6 (3.0–6.2)	10.1 (8.3–11.8)	1.6 (0.6–2.6)	2.9 (0.2–6.1)
Kyrgyzstan	2.8 (2.7–3.0)	3.1 (2.9–3.3)	1.8 (1.5–2.1)	1.6 (0.7–2.4)	1.8 (1.2–2.4)	0.5 (-1.1–2.2)	1.2 (0.9–1.6)	1.6 (1.3–1.9)	0.9 (0.1–1.7)	0.5 (-0.1–1.1)	3.1 (2.7–3.5)	3.0 (1.3–5.1)
Lao People's Democratic Republic	6.9 (5.1–7.8)	8.1 (6.5–9.9)	7.4 (5.5–10.2)	4.4 (-10.3–13.0)	5.5 (1.2–9.9)	5.1 (-1.2–13.4)	0.8 (-8.9–4.3)	5.9 (1.2–10.4)	3.3 (0.0–8.5)	1.1 (-16.1–14.3)	2.7 (-6.8–12.4)	1.4 (-10.5–14.5)
Latvia	4.3 (4.0–4.7)	5.4 (5.0–6.2)	5.5 (5.0–7.3)	4.3 (2.4–6.7)	4.9 (3.2–6.6)	4.9 (-0.1–9.7)	2.5 (1.8–3.2)	4.0 (3.4–4.6)	2.9 (1.4–4.6)	0.5 (-0.5–1.5)	4.9 (4.0–5.8)	2.7 (0.1–5.5)
Lebanon	3.7 (3.7–3.7)	2.4 (2.4–2.4)	-9.6 (-9.6—9.6)	4.5 (4.5–4.5)	2.9 (2.9–2.9)	-7.5 (-7.5—7.5)	12.2 (12.2–12.2)	1.3 (1.3–1.3)	-6.4 (-6.4—6.4)	19.2 (19.2–19.2)	1.1 (1.1–1.1)	-8.7 (-8.7—8.7)
Lesotho	2.1 (1.0–3.1)	2.2 (1.1–3.5)	2.6 (1.0–5.0)	1.7 (-3.4–5.4)	1.6 (-2.0–5.1)	1.9 (-3.4–9.0)	-1.5 (-5.5–3.4)	3.4 (1.0–5.7)	10.2 (5.6–15.5)	-5.9 (-21.7–3.0)	3.8 (1.2–6.7)	10.8 (2.5–26.5)
Liberia	1.4 (0.4–2.4)	2.4 (1.3–3.7)	3.6 (2.1–5.6)	0.5 (-9.0–6.9)	1.6 (-3.6–8.5)	2.7 (-8.4–15.1)	8.6 (5.1–10.9)	0.0 (-2.5–2.4)	2.7 (-0.1–6.5)	13.7 (8.9–18.1)	0.8 (-2.8–5.6)	2.2 (-7.0–14.8)
Libya	6.2 (1.7–10.5)	2.3 (0.2–7.4)	5.9 (3.2–10.3)	3.2 (-10.8–19.2)	1.3 (-10.6–11.5)	3.8 (-6.6–11.5)	1.7 (1.7–1.7)	-0.6 (-0.6—0.6)	5.9 (5.9–5.9)	1.5 (1.5–1.5)	-3.4 (-3.4—3.4)	9.9 (9.9–9.9)

Country	Annual rate of reduction in probability of dying $_5q_5$ (%)			Annual rate of reduction in probability of dying $_5q_{10}$ (%)			Annual rate of reduction in probability of dying $_5q_{15}$ (%)			Annual rate of reduction in probability of dying $_5q_{20}$ (%)		
	1990-2000	2000-2015	2015-2024	1990-2000	2000-2015	2015-2024	1990-2000	2000-2015	2015-2024	1990-2000	2000-2015	2015-2024
Lithuania	4.3 (4.1–4.6)	4.1 (3.8–4.4)	5.2 (4.8–5.6)	3.5 (2.0–4.9)	3.1 (1.9–4.4)	4.3 (0.8–8.0)	1.0 (0.4–1.6)	3.4 (2.9–3.9)	5.7 (4.0–7.5)	-0.8 (-1.6—0.0)	4.1 (3.4–4.8)	5.1 (2.8–7.6)
Luxembourg	7.9 (7.1–8.7)	4.0 (3.1–5.0)	-0.3 (-1.1–1.0)	6.8 (2.9–11.2)	3.0 (-0.5–6.7)	-1.2 (-7.7–8.0)	7.8 (6.1–9.4)	4.3 (2.9–5.8)	2.3 (-0.5–5.8)	4.9 (2.2–7.6)	6.3 (4.0–8.8)	6.0 (-1.7–18.0)
Madagascar	4.1 (3.2–5.1)	2.7 (1.9–3.6)	-1.6 (-2.7—0.3)	4.0 (-1.7–9.9)	2.6 (-1.4–7.5)	-1.5 (-15.4–15.5)	1.6 (-0.6–3.7)	1.6 (-0.3–3.4)	2.9 (0.2–7.0)	1.9 (-2.4–6.0)	1.7 (-1.7–6.4)	1.6 (-7.7–13.9)
Malawi	4.8 (4.2–5.5)	4.3 (3.4–5.1)	3.7 (2.4–5.2)	2.9 (-1.2–6.2)	2.6 (-0.5–5.5)	2.3 (-3.2–9.0)	-1.0 (-2.6–0.6)	5.5 (3.9–7.2)	3.6 (0.6–7.3)	-2.7 (-6.9–1.1)	5.2 (2.2–8.6)	2.7 (-6.8–15.4)
Malaysia	4.3 (4.1–4.5)	1.9 (1.8–2.0)	3.2 (3.0–3.5)	3.6 (2.7–4.6)	1.4 (1.0–1.7)	2.7 (1.6–3.9)	1.4 (0.6–2.3)	1.0 (0.7–1.2)	0.8 (-0.1–1.7)	2.8 (0.9–4.7)	2.4 (1.9–2.8)	1.2 (-0.7–3.3)
Maldives	6.6 (5.9–7.2)	5.8 (4.9–6.8)	9.4 (8.2–10.7)	3.8 (0.2–7.1)	2.9 (0.9–5.1)	6.7 (1.6–13.0)	7.8 (5.9–9.7)	-0.6 (-1.8–0.5)	3.5 (1.5–6.3)	6.8 (3.8–9.5)	2.2 (0.2–4.4)	7.4 (-1.7–23.1)
Mali	2.5 (1.8–3.1)	2.6 (1.9–3.5)	4.0 (3.1–5.5)	1.2 (-3.4–5.0)	1.2 (-2.0–4.5)	2.4 (-2.1–13.1)	2.4 (0.4–4.4)	2.3 (0.4–4.3)	1.1 (-1.7–5.0)	1.4 (-3.6–5.3)	1.8 (-1.4–6.0)	-0.3 (-9.9–11.7)
Malta	0.6 (-2.4–2.5)	1.8 (-0.7–4.2)	2.9 (1.0–5.6)	0.3 (-8.4–6.9)	1.4 (-2.1–5.4)	2.4 (-3.3–10.4)	0.2 (-2.3–2.7)	3.9 (2.3–5.3)	1.5 (-2.3–6.3)	0.7 (-3.8–4.6)	2.6 (0.2–5.2)	0.0 (-6.9–9.3)
Marshall Islands	1.0 (1.0–1.0)	1.2 (1.2–1.2)	2.8 (2.8–2.8)	0.8 (0.8–0.8)	0.9 (0.9–0.9)	2.2 (2.2–2.2)	0.6 (0.6–0.6)	0.7 (0.7–0.7)	1.7 (1.7–1.7)	0.6 (0.6–0.6)	0.6 (0.6–0.7)	1.5 (1.5–1.5)
Mauritania	2.9 (1.9–3.9)	3.7 (2.8–5.3)	3.7 (2.6–6.5)	2.7 (-3.9–7.9)	2.8 (-2.4–9.5)	2.8 (-4.2–14.4)	4.1 (1.8–6.5)	-0.1 (-2.9–2.3)	0.1 (-2.7–4.0)	3.8 (-3.2–10.7)	0.4 (-6.5–6.6)	-0.1 (-10.1–14.0)
Mauritius	2.2 (1.4–3.0)	2.3 (1.6–3.0)	1.9 (1.1–3.0)	2.2 (0.3–4.0)	2.2 (1.0–3.6)	1.8 (-1.4–4.7)	2.0 (0.9–3.2)	0.0 (-0.7–0.7)	-0.8 (-2.5–1.0)	1.7 (-0.1–3.4)	-0.7 (-1.7–0.4)	0.2 (-2.3–3.0)
Mexico	5.4 (5.2–5.5)	2.1 (2.1–2.2)	0.1 (-0.1–0.3)	3.9 (3.3–4.5)	1.1 (0.8–1.5)	-0.6 (-1.6–0.4)	2.2 (1.9–2.5)	0.1 (-0.1–0.2)	-0.6 (-1.2—0.1)	2.4 (1.8–3.0)	-0.4 (-0.8–0.0)	-0.8 (-2.3–0.7)
Micronesia (Federated States of)	2.2 (2.2–2.2)	1.6 (1.6–1.6)	2.9 (2.9–2.9)	1.7 (1.7–1.7)	1.2 (1.2–1.2)	2.3 (2.3–2.3)	1.3 (1.3–1.3)	0.9 (0.9–0.9)	1.7 (1.7–1.7)	1.2 (1.2–1.2)	0.9 (0.9–0.9)	1.6 (1.6–1.6)
Monaco	3.6 (3.6–3.6)	2.3 (2.3–2.3)	2.7 (2.7–2.7)	3.2 (3.2–3.2)	2.0 (2.0–2.0)	2.5 (2.5–2.5)	2.8 (2.8–2.8)	1.7 (1.7–1.7)	2.1 (2.1–2.1)	2.7 (2.7–2.7)	1.7 (1.7–1.7)	2.1 (2.1–2.1)
Mongolia	5.9 (5.7–6.1)	3.5 (3.2–3.7)	5.4 (5.1–5.8)	4.1 (2.1–5.8)	1.6 (0.9–2.3)	3.6 (1.6–5.7)	2.1 (1.1–3.1)	1.0 (0.6–1.4)	2.0 (0.8–3.3)	2.6 (1.2–4.0)	2.3 (1.8–2.9)	2.4 (0.4–4.5)
Montenegro	2.9 (1.6–4.0)	3.8 (2.6–5.1)	2.2 (1.0–3.8)	2.1 (-3.1–6.3)	2.9 (0.4–5.6)	1.2 (-3.9–7.9)	0.3 (-1.6–2.1)	3.4 (2.3–4.6)	-6.0 (-8.6–3.4)	1.4 (-1.2–3.8)	3.4 (1.7–5.1)	-4.6 (-8.6–0.0)
Montserrat	3.1 (3.1–3.1)	2.7 (2.7–2.7)	2.5 (2.5–2.5)	2.3 (2.3–2.3)	2.0 (2.0–2.0)	1.9 (1.9–1.9)	2.0 (2.0–2.0)	1.7 (1.7–1.7)	1.6 (1.6–1.6)	1.9 (1.9–1.9)	1.7 (1.7–1.7)	1.6 (1.6–1.6)
Morocco	5.0 (3.9–6.4)	4.3 (3.4–6.0)	3.5 (1.5–6.4)	5.0 (1.1–10.8)	4.5 (-1.0–13.0)	2.6 (-7.8–13.1)	3.5 (1.2–6.1)	2.6 (0.1–5.7)	2.7 (-0.7–7.3)	3.6 (-1.1–8.4)	2.7 (-3.8–12.0)	0.9 (-11.4–14.6)
Mozambique	6.3 (4.5–7.5)	6.6 (5.1–8.2)	5.7 (4.3–7.9)	5.5 (-6.4–12.2)	5.5 (0.8–11.4)	4.8 (-2.4–15.8)	0.6 (-2.2–3.1)	4.4 (2.5–6.6)	1.9 (-2.8–5.8)	0.5 (-6.4–5.4)	2.5 (-0.8–6.3)	2.6 (-4.8–13.0)
Myanmar	4.2 (1.5–5.3)	10.3 (7.4–13.9)	5.5 (3.3–9.7)	1.0 (-20.3–19.2)	5.8 (-7.3–21.3)	3.7 (-5.5–16.2)	2.2 (-1.6–5.5)	7.1 (3.7–11.0)	-0.1 (-3.7–5.0)	0.5 (-15.8–11.5)	4.2 (-5.3–13.3)	-3.3 (-14.0–8.1)
Namibia	0.9 (-0.4–2.0)	1.3 (0.1–2.8)	6.7 (5.4–8.8)	0.1 (-8.3–6.4)	0.4 (-4.9–6.0)	5.8 (-4.8–21.4)	-0.3 (-1.9–1.3)	1.9 (0.5–3.3)	4.2 (1.5–7.1)	-1.6 (-6.0–1.7)	1.3 (-1.3–4.4)	3.4 (-2.3–11.9)
Nauru	4.5 (4.5–4.5)	5.3 (5.3–5.3)	7.7 (7.7–7.7)	3.4 (3.4–3.5)	4.1 (4.1–4.1)	6.2 (6.1–6.2)	2.7 (2.7–2.7)	3.1 (3.1–3.1)	4.6 (4.6–4.6)	2.4 (2.4–2.4)	2.9 (2.8–2.9)	4.2 (4.2–4.2)
Nepal	7.2 (6.3–8.0)	3.9 (2.9–5.1)	8.3 (6.9–10.3)	5.2 (-0.6–10.5)	1.6 (-3.0–5.8)	7.1 (-1.5–17.9)	4.0 (1.8–6.7)	0.8 (-2.1–3.4)	4.9 (1.8–9.4)	1.7 (-3.7–6.2)	0.9 (-3.4–6.9)	3.2 (-8.3–16.4)
Netherlands (Kingdom of the)	3.3 (3.1–3.5)	4.0 (3.7–4.2)	-0.1 (-0.7–0.2)	2.3 (1.3–3.4)	3.2 (2.4–4.0)	-0.9 (-3.0–1.3)	1.1 (0.8–1.4)	3.9 (3.6–4.2)	-0.7 (-1.4—0.1)	1.0 (0.3–1.7)	3.4 (2.9–4.0)	-0.9 (-2.2–0.4)

Country	Annual rate of reduction in probability of dying $_5q_5$ (%)			Annual rate of reduction in probability of dying $_5q_{10}$ (%)			Annual rate of reduction in probability of dying $_5q_{15}$ (%)			Annual rate of reduction in probability of dying $_5q_{20}$ (%)		
	1990-2000	2000-2015	2015-2024	1990-2000	2000-2015	2015-2024	1990-2000	2000-2015	2015-2024	1990-2000	2000-2015	2015-2024
New Zealand	3.9 (3.6–4.1)	3.9 (3.6–4.2)	1.7 (1.1–2.5)	2.9 (1.5–4.3)	3.0 (1.9–4.0)	0.8 (-2.9–4.9)	3.3 (2.8–3.9)	3.2 (2.7–3.6)	2.3 (0.4–4.5)	4.6 (3.5–5.6)	2.4 (1.6–3.2)	0.8 (-3.7–5.6)
Nicaragua	4.2 (1.1–7.0)	4.6 (1.8–9.0)	3.3 (0.1–7.8)	-0.4 (-12.3–7.0)	1.2 (-4.0–8.6)	2.3 (-4.8–9.4)	3.6 (3.6–3.6)	2.8 (2.8–2.8)	1.0 (1.0–1.0)	4.2 (4.2–4.2)	2.8 (2.8–2.8)	1.0 (1.0–1.0)
Niger	3.1 (2.1–4.9)	3.0 (1.7–4.1)	2.1 (-0.7–4.1)	0.4 (-7.3–5.1)	1.4 (-2.1–5.9)	1.8 (-4.5–12.9)	1.6 (-0.5–3.7)	2.1 (-0.5–5.0)	2.8 (-0.7–7.6)	0.0 (-6.0–4.0)	1.0 (-4.1–8.3)	1.1 (-10.7–14.4)
Nigeria	2.1 (1.2–2.8)	2.3 (1.6–3.3)	0.5 (-0.8–1.7)	1.7 (-3.9–5.5)	1.3 (-1.9–4.5)	0.1 (-6.7–7.4)	3.3 (1.2–5.6)	2.7 (1.1–4.2)	1.9 (-0.9–4.4)	2.3 (-3.6–6.6)	2.6 (0.1–5.0)	2.6 (-2.0–8.9)
Niue	-2.4 (-2.4—2.4)	0.3 (0.3–0.3)	2.9 (2.9–2.9)	-1.9 (-1.9—1.9)	0.2 (0.2–0.2)	2.3 (2.3–2.3)	-1.4 (-1.4—1.4)	0.2 (0.2–0.2)	1.7 (1.7–1.7)	-1.3 (-1.3—1.3)	0.2 (0.2–0.2)	1.6 (1.6–1.6)
North Macedonia	3.7 (3.2–4.2)	3.9 (3.4–4.5)	2.6 (2.0–3.3)	2.6 (0.3–4.8)	2.8 (1.4–4.3)	1.4 (-2.4–5.6)	0.2 (-0.6–1.1)	3.1 (2.5–3.6)	-1.3 (-2.5–0.3)	0.2 (-1.5–1.8)	3.4 (2.3–4.6)	-1.7 (-4.9–1.5)
Norway	3.9 (3.5–4.2)	3.8 (3.4–4.1)	2.3 (1.5–2.8)	2.9 (1.6–4.0)	2.7 (1.8–4.1)	1.4 (-2.3–4.0)	0.9 (0.4–1.4)	4.7 (4.3–5.1)	-0.9 (-2.1–0.1)	-0.7 (-1.9–0.5)	4.2 (3.2–5.2)	0.6 (-1.4–2.8)
Oman	6.7 (6.7–6.7)	2.1 (2.1–2.1)	0.7 (0.7–0.7)	8.2 (8.2–8.2)	2.5 (2.5–2.5)	0.8 (0.8–0.8)	3.6 (3.6–3.6)	1.1 (1.1–1.1)	0.4 (0.4–0.4)	3.1 (3.1–3.1)	1.0 (1.0–1.0)	0.3 (0.3–0.3)
Pakistan	1.7 (1.1–2.3)	2.9 (2.2–3.7)	4.4 (3.5–5.6)	0.8 (-1.8–3.6)	2.1 (-1.0–5.6)	3.6 (-1.8–15.5)	3.8 (2.1–5.5)	3.1 (0.8–5.0)	3.5 (0.3–7.3)	1.4 (-2.1–5.1)	2.8 (-0.2–7.5)	2.6 (-3.6–11.6)
Palau	2.7 (2.7–2.7)	1.3 (1.3–1.3)	-0.1 (-0.1—0.1)	2.1 (2.1–2.1)	1.1 (1.1–1.1)	-0.1 (-0.1—0.1)	-2.7 (-7.1–1.6)	0.2 (-2.4–4.2)	2.8 (-0.7–7.6)	-0.3 (-6.2–5.5)	-0.1 (-8.4–10.7)	1.1 (-11.2–13.5)
Panama	2.4 (2.1–2.7)	1.2 (1.0–1.5)	2.2 (1.7–2.7)	1.4 (-0.3–3.1)	0.1 (-0.6–0.9)	1.0 (-1.4–3.8)	1.1 (0.4–1.9)	-0.9 (-1.3—0.6)	3.4 (2.2–4.6)	0.6 (-0.6–1.8)	-0.7 (-1.3—0.2)	1.9 (-0.8–4.9)
Papua New Guinea	1.5 (1.5–1.5)	0.7 (0.7–0.7)	5.1 (5.1–5.1)	1.2 (1.2–1.2)	0.3 (0.3–0.3)	4.2 (4.2–4.2)	0.9 (0.9–0.9)	1.2 (1.2–1.2)	1.7 (1.7–1.7)	0.8 (0.8–0.8)	1.1 (1.1–1.1)	1.5 (1.5–1.5)
Paraguay	5.5 (5.1–5.9)	2.1 (1.7–2.5)	3.0 (2.3–3.8)	3.5 (-2.3–7.4)	0.2 (-1.2–1.5)	1.2 (-1.4–4.0)	1.6 (1.6–1.6)	1.5 (1.5–1.5)	1.7 (1.7–1.7)	1.6 (1.5–1.6)	1.5 (1.5–1.5)	1.7 (1.7–1.7)
Peru	7.3 (6.9–7.7)	4.1 (3.8–4.5)	0.2 (-1.3–1.9)	6.4 (3.3–9.2)	3.3 (1.8–4.7)	-0.6 (-5.3–4.3)	6.3 (4.8–7.9)	3.6 (2.7–4.3)	-0.4 (-3.1–2.9)	4.9 (1.4–8.2)	3.1 (1.3–4.6)	-1.3 (-9.7–8.0)
Philippines	5.4 (5.2–5.5)	2.0 (1.9–2.1)	0.9 (0.6–1.2)	3.8 (3.1–4.5)	1.1 (0.7–1.5)	-0.0 (-1.6–1.6)	2.6 (2.3–2.9)	0.4 (0.3–0.6)	-1.4 (-2.3—0.5)	2.8 (2.2–3.3)	0.5 (0.2–0.9)	-0.3 (-2.6–2.1)
Poland	4.2 (4.1–4.3)	4.3 (4.2–4.4)	1.0 (0.7–1.2)	3.1 (2.3–4.0)	3.1 (2.5–3.6)	-0.2 (-1.6–1.2)	2.6 (2.4–2.8)	2.2 (2.1–2.4)	1.0 (0.5–1.5)	2.6 (2.0–3.2)	2.0 (1.6–2.4)	0.6 (-0.2–1.5)
Portugal	5.0 (4.8–5.1)	6.8 (6.5–7.0)	1.4 (1.1–1.8)	4.1 (3.1–5.1)	6.1 (5.2–7.1)	0.9 (-1.8–3.8)	4.3 (3.9–4.7)	7.0 (6.6–7.4)	-1.5 (-2.7—0.2)	2.9 (2.3–3.5)	6.7 (6.1–7.4)	-0.6 (-2.3–1.3)
Qatar	2.8 (1.8–3.9)	3.2 (2.5–3.8)	1.4 (0.4–2.2)	3.2 (-0.3–6.4)	3.8 (2.1–6.0)	2.2 (-3.3–7.1)	0.1 (-1.6–2.9)	2.4 (1.1–3.2)	3.4 (1.9–5.7)	-1.1 (-5.8–2.6)	4.2 (2.3–6.2)	3.3 (-1.2–7.9)
Republic of Korea	7.2 (7.1–7.4)	7.0 (6.8–7.1)	1.2 (0.8–1.5)	5.9 (5.2–6.6)	5.5 (4.9–6.0)	-0.5 (-2.5–1.4)	4.7 (4.4–5.0)	5.1 (4.8–5.3)	-0.8 (-1.6—0.0)	4.8 (4.2–5.5)	4.2 (3.8–4.6)	-1.4 (-2.9–0.2)
Republic of Moldova	3.1 (2.9–3.4)	4.0 (3.7–4.3)	3.6 (3.1–4.0)	1.3 (-0.0–2.7)	2.2 (1.3–3.1)	1.7 (-0.7–4.2)	2.8 (2.3–3.3)	1.6 (1.2–1.9)	-0.3 (-1.1–0.7)	3.8 (3.1–4.5)	2.1 (1.6–2.8)	-0.2 (-1.9–1.5)
Romania	2.7 (2.1–3.4)	6.1 (5.5–6.6)	4.1 (2.6–5.7)	-2.6 (-3.6—1.6)	6.4 (5.7–7.2)	5.5 (3.2–8.1)	2.2 (1.8–2.6)	2.0 (1.7–2.3)	3.1 (2.2–4.0)	3.1 (2.4–3.8)	1.6 (1.1–2.2)	1.7 (0.6–2.9)
Russian Federation	1.7 (1.5–1.8)	5.0 (4.9–5.1)	4.7 (4.4–5.1)	0.4 (-0.2–1.1)	3.4 (3.0–3.8)	2.6 (1.4–3.8)	-2.4 (-2.7—2.1)	4.8 (4.6–5.0)	0.1 (-0.6–0.9)	-5.1 (-5.7—4.5)	5.5 (5.2–5.9)	-6.4 (-7.8—5.0)
Rwanda	1.5 (0.8–2.1)	10.3 (9.5–11.1)	12.5 (11.5–13.7)	0.3 (-7.0–6.1)	9.2 (4.7–14.3)	11.4 (-5.2–32.6)	3.0 (0.8–5.2)	9.2 (7.7–10.7)	5.2 (2.6–8.4)	2.1 (-4.9–7.4)	8.7 (5.5–12.2)	4.3 (-5.9–18.5)
Saint Kitts and Nevis	1.9 (1.9–1.9)	1.1 (1.1–1.1)	1.8 (1.8–1.8)	1.4 (1.4–1.4)	0.8 (0.8–0.8)	1.3 (1.3–1.3)	-5.7 (-10.3—2.2)	-1.4 (-3.8–1.2)	2.2 (-1.3–7.1)	-6.3 (-19.8–2.3)	-2.7 (-6.3–1.2)	0.5 (-9.7–12.9)

Country	Annual rate of reduction in probability of dying $_5q_5$ (%)			Annual rate of reduction in probability of dying $_5q_{10}$ (%)			Annual rate of reduction in probability of dying $_5q_{15}$ (%)			Annual rate of reduction in probability of dying $_5q_{20}$ (%)		
	1990-2000	2000-2015	2015-2024	1990-2000	2000-2015	2015-2024	1990-2000	2000-2015	2015-2024	1990-2000	2000-2015	2015-2024
Saint Lucia	-0.8 (-2.7–0.7)	1.9 (0.5–3.4)	2.8 (2.0–4.4)	0.3 (-5.6–5.5)	2.8 (-0.6–7.4)	3.7 (-4.7–18.2)	1.2 (-1.1–3.4)	0.7 (-0.6–1.9)	-5.8 (-9.0—2.4)	0.1 (-3.1–3.2)	0.5 (-1.2–2.3)	-7.7 (-12.1—2.7)
Saint Vincent and the Grenadines	1.2 (0.1–1.9)	1.6 (0.2–3.1)	4.1 (2.3–6.2)	-2.6 (-9.8–2.7)	-2.2 (-5.2–0.6)	0.5 (-4.3–6.0)	0.9 (-1.5–3.2)	-2.1 (-3.5—0.7)	2.2 (-1.1–6.0)	-2.7 (-6.5–0.7)	-0.5 (-2.5–1.5)	2.9 (-2.7–9.9)
Samoa	2.5 (-3.0–3.3)	4.4 (0.8–7.6)	4.6 (2.4–9.1)	-1.5 (-16.2–18.8)	-0.4 (-12.5–9.5)	1.6 (-5.8–11.3)	4.4 (-5.5–9.8)	2.7 (-1.5–6.9)	1.9 (-0.9–6.3)	6.2 (-11.5–19.8)	4.8 (-1.6–12.4)	1.6 (-9.6–16.5)
San Marino	7.8 (7.8–7.8)	6.0 (6.0–6.0)	4.1 (4.1–4.1)	7.0 (7.0–7.0)	5.4 (5.4–5.4)	3.7 (3.7–3.7)	6.0 (6.0–6.0)	4.6 (4.6–4.6)	3.1 (3.1–3.1)	5.9 (5.9–5.9)	4.5 (4.5–4.5)	3.1 (3.1–3.1)
Sao Tome and Principe	3.1 (3.1–3.1)	9.4 (9.4–9.4)	6.8 (6.8–6.8)	2.1 (2.1–2.1)	6.6 (6.6–6.6)	5.0 (5.0–5.0)	3.4 (-6.4–7.5)	0.4 (-4.6–6.0)	2.9 (-1.1–8.1)	1.9 (-16.4–18.0)	-0.4 (-9.1–9.2)	1.1 (-10.6–12.9)
Saudi Arabia	5.4 (5.4–5.4)	4.6 (4.6–4.6)	3.6 (3.6–3.6)	6.6 (6.6–6.6)	5.7 (5.7–5.7)	4.4 (4.4–4.4)	2.0 (-6.4–5.5)	1.5 (-1.8–4.6)	2.2 (-0.6–6.6)	1.3 (-15.9–20.7)	0.8 (-6.4–8.4)	0.9 (-10.5–15.2)
Senegal	0.8 (0.2–1.3)	7.5 (6.8–8.4)	5.3 (4.2–7.0)	-1.4 (-9.1–4.5)	4.9 (1.4–8.4)	2.8 (-6.8–14.2)	0.9 (-0.9–2.6)	3.5 (2.0–5.1)	4.7 (2.5–8.1)	2.1 (-2.8–5.8)	3.8 (0.7–7.5)	4.3 (-2.4–15.8)
Serbia	3.4 (3.1–3.8)	4.8 (4.4–5.1)	0.4 (-0.2–1.0)	2.2 (0.6–3.7)	3.5 (2.4–4.7)	-0.8 (-3.6–1.9)	0.8 (0.3–1.4)	4.0 (3.6–4.3)	-1.2 (-2.0—0.2)	-0.0 (-1.1–1.0)	3.9 (3.1–4.8)	-0.9 (-2.9–1.3)
Seychelles	1.8 (0.7–3.0)	2.3 (1.2–4.1)	2.6 (0.9–5.2)	0.6 (-8.0–6.7)	1.0 (-4.3–7.7)	1.6 (-4.7–11.8)	-3.1 (-6.0—0.2)	-0.6 (-2.6–1.6)	0.3 (-2.1–4.0)	0.7 (-4.4–5.3)	1.8 (-2.2–7.2)	-0.1 (-12.2–16.8)
Sierra Leone	3.1 (0.5–4.2)	1.9 (0.1–3.5)	1.5 (0.1–3.7)	2.4 (-14.3–18.9)	1.0 (-5.8–6.5)	0.7 (-7.8–12.9)	1.1 (-4.6–5.9)	1.0 (-1.4–3.3)	3.0 (0.1–7.6)	0.4 (-11.2–7.6)	1.4 (-2.7–5.6)	1.8 (-7.1–14.4)
Singapore	5.5 (4.9–6.0)	3.7 (3.2–4.3)	-0.4 (-1.0–0.3)	5.1 (3.1–7.0)	3.3 (2.0–4.6)	-0.8 (-3.9–2.5)	4.2 (3.2–5.1)	3.3 (2.8–3.9)	-2.5 (-3.8—1.2)	3.5 (2.0–4.9)	3.9 (3.0–4.9)	-0.4 (-3.1–2.4)
Slovakia	2.0 (1.7–2.2)	3.3 (3.0–3.5)	2.8 (2.5–3.4)	1.3 (-0.1–2.7)	2.5 (1.5–3.6)	2.0 (-0.7–4.6)	2.0 (1.7–2.4)	1.8 (1.5–2.1)	1.8 (1.1–2.7)	1.7 (1.0–2.3)	1.8 (1.2–2.3)	1.4 (-0.0–2.9)
Slovenia	4.5 (3.9–5.0)	4.5 (3.9–5.1)	3.4 (2.5–4.4)	3.2 (1.6–5.0)	3.2 (1.9–4.9)	2.2 (-2.2–5.3)	1.8 (1.0–2.4)	4.6 (4.0–5.3)	3.5 (1.7–4.9)	2.3 (1.2–3.4)	4.4 (3.4–5.5)	4.3 (1.4–7.3)
Solomon Islands	2.0 (2.0–2.0)	1.2 (1.2–1.2)	2.3 (2.3–2.3)	1.6 (1.6–1.6)	1.0 (1.0–1.0)	1.8 (1.8–1.8)	1.2 (1.2–1.2)	0.7 (0.7–0.7)	1.3 (1.3–1.3)	1.1 (1.1–1.1)	0.7 (0.7–0.7)	1.2 (1.2–1.2)
Somalia	0.4 (0.4–0.4)	1.8 (1.8–1.8)	3.3 (3.3–3.3)	0.3 (0.3–0.3)	1.3 (1.3–1.3)	2.5 (2.5–2.5)	3.0 (3.0–3.0)	1.2 (1.2–1.2)	1.4 (1.4–1.4)	5.6 (5.6–5.7)	1.0 (1.0–1.1)	0.4 (0.4–0.4)
South Africa	-2.4 (-4.8—1.9)	3.7 (3.1–4.1)	4.8 (3.5–5.6)	-5.1 (-19.2–3.8)	0.6 (-1.7–2.6)	2.0 (-1.0–5.2)	-2.2 (-5.1–0.7)	1.6 (0.9–2.3)	0.8 (-0.5–2.3)	-6.7 (-11.7—3.0)	3.8 (2.7–4.9)	2.6 (0.0–5.2)
South Sudan	10.5 (10.5–10.5)	-3.3 (-3.3—3.3)	14.8 (14.8–14.8)	13.7 (13.5–13.9)	-3.3 (-3.4—3.3)	14.9 (14.8–15.0)	10.9 (10.9–10.9)	-1.4 (-1.4—1.4)	9.6 (9.6–9.6)	10.6 (10.5–10.8)	-2.4 (-2.4—2.4)	10.6 (10.5–10.7)
Spain	3.8 (3.7–4.0)	4.6 (4.4–4.8)	0.7 (0.2–1.1)	3.1 (2.4–3.8)	4.3 (3.7–4.9)	0.4 (-1.7–2.5)	3.7 (3.4–4.0)	6.2 (6.0–6.5)	0.1 (-0.8–1.0)	5.3 (4.7–5.8)	5.7 (5.2–6.1)	-1.3 (-2.8–0.4)
Sri Lanka	4.3 (4.1–4.5)	5.1 (4.9–5.3)	5.4 (4.6–6.4)	2.8 (2.2–3.5)	3.6 (3.1–4.0)	3.9 (1.1–7.0)	3.2 (2.9–3.5)	6.8 (6.4–7.2)	4.6 (1.2–8.9)	3.6 (3.1–4.1)	8.5 (7.7–9.3)	3.2 (-7.0–14.9)
State of Palestine	2.4 (0.5–4.7)	1.1 (-1.5–3.5)	-30.2 (-32.5—26.8)	1.1 (-5.3–6.2)	0.7 (-4.6–7.6)	-30.7 (-37.7—22.2)	0.3 (0.3–0.3)	2.8 (2.8–2.8)	-26.2 (-26.2—26.2)	-1.5 (-1.5—1.5)	4.4 (4.4–4.4)	-26.1 (-26.2—26.0)
Sudan	4.5 (3.5–5.5)	5.0 (4.0–6.6)	-5.0 (-6.7—2.3)	3.3 (-4.3–11.4)	3.6 (-3.6–13.8)	-4.1 (-14.6–9.1)	1.7 (1.7–1.7)	2.2 (2.2–2.2)	-3.4 (-3.4—3.4)	1.5 (1.5–1.5)	1.9 (1.9–2.0)	-5.4 (-5.4—5.4)
Suriname	2.1 (1.4–2.7)	0.7 (0.0–1.3)	-3.0 (-3.6—2.4)	2.3 (-0.7–5.0)	0.8 (-0.8–2.6)	-2.8 (-6.9–1.9)	2.6 (1.0–4.1)	-0.0 (-0.8–0.8)	-1.6 (-3.6–0.4)	1.1 (-1.0–3.1)	1.2 (0.1–2.3)	-0.4 (-3.7–3.5)

Country	Annual rate of reduction in probability of dying $_5q_5$ (%)			Annual rate of reduction in probability of dying $_5q_{10}$ (%)			Annual rate of reduction in probability of dying $_5q_{15}$ (%)			Annual rate of reduction in probability of dying $_5q_{20}$ (%)		
	1990-2000	2000-2015	2015-2024	1990-2000	2000-2015	2015-2024	1990-2000	2000-2015	2015-2024	1990-2000	2000-2015	2015-2024
Sweden	2.6 (2.3–2.9)	3.1 (2.7–3.3)	0.8 (0.2–1.2)	1.8 (0.4–3.0)	2.2 (1.3–3.3)	-0.0 (-2.7–2.5)	2.7 (2.3–3.1)	2.0 (1.7–2.4)	2.2 (1.3–2.9)	1.8 (0.9–2.7)	1.0 (0.4–1.7)	1.6 (0.3–3.1)
Switzerland	4.4 (3.9–4.9)	3.3 (2.7–3.8)	1.1 (-0.4–2.1)	3.6 (2.4–4.8)	3.4 (2.5–4.5)	2.2 (-0.9–5.5)	4.3 (3.9–4.7)	4.0 (3.7–4.4)	-0.0 (-0.9–1.0)	4.9 (4.1–5.8)	5.1 (4.4–5.8)	0.2 (-1.7–2.2)
Syrian Arab Republic	4.7 (4.4–5.0)	-5.6 (-6.2—5.0)	14.5 (12.1–17.7)	3.5 (-1.6–7.4)	-4.4 (-7.0—1.4)	10.2 (2.6–18.2)	8.4 (5.7–10.4)	-9.6 (-10.7—8.4)	18.0 (15.0–22.3)	5.0 (-8.2–13.9)	-15.1 (-18.8—11.3)	22.0 (10.7–35.8)
Tajikistan	4.4 (4.2–4.6)	3.9 (3.8–4.1)	4.1 (3.7–4.5)	3.3 (2.4–4.1)	3.0 (2.3–3.8)	3.3 (0.9–5.8)	0.2 (-0.3–0.8)	3.5 (3.0–4.0)	3.0 (1.1–5.1)	-1.2 (-2.0—0.5)	4.9 (4.1–5.7)	2.6 (-1.5–7.1)
Thailand	1.1 (1.0–1.3)	3.2 (3.1–3.3)	2.0 (1.8–2.3)	0.1 (-0.7–0.9)	0.9 (0.6–1.3)	-0.5 (-1.5–0.4)	0.7 (0.3–1.0)	0.5 (0.3–0.7)	3.0 (2.4–3.6)	-2.4 (-3.2—1.7)	3.6 (3.3–4.0)	0.3 (-1.0–1.7)
Timor-Leste	9.4 (9.4–9.4)	3.8 (3.8–3.8)	2.4 (2.4–2.4)	9.1 (9.1–9.2)	2.9 (2.9–2.9)	1.8 (1.8–1.9)	3.9 (-1.2–8.9)	-1.8 (-4.5–1.1)	-1.6 (-4.3–2.4)	3.6 (-14.7–16.7)	0.2 (-5.7–8.0)	-2.4 (-17.0–15.7)
Togo	3.5 (2.4–4.4)	5.1 (3.7–7.2)	4.2 (2.5–7.2)	1.1 (-9.2–7.1)	1.9 (-4.7–9.2)	2.3 (-4.6–11.8)	-0.6 (-2.6–1.1)	2.6 (0.5–5.6)	3.0 (-0.4–7.5)	-0.7 (-7.4–4.9)	1.5 (-4.3–9.8)	1.1 (-11.2–15.1)
Tonga	-5.3 (-9.2—3.1)	2.8 (0.8–5.3)	5.0 (3.9–7.2)	-3.3 (-14.5–3.7)	4.5 (-0.6–12.6)	5.5 (-3.1–19.6)	-4.8 (-9.4—1.0)	0.8 (-1.0–2.5)	2.3 (-1.2–6.7)	-1.0 (-9.3–5.4)	-0.6 (-3.1–1.9)	-0.2 (-8.6–10.0)
Trinidad and Tobago	1.9 (1.4–2.4)	2.0 (1.5–2.6)	2.0 (0.8–3.3)	1.3 (-0.8–3.2)	1.3 (-0.0–2.7)	1.2 (-3.6–7.1)	-0.2 (-1.0–0.5)	-0.5 (-0.9–0.0)	-1.1 (-3.4–1.6)	-0.4 (-1.6–0.9)	-0.5 (-1.4–0.2)	-2.0 (-9.0–5.8)
Tunisia	4.6 (4.4–4.9)	2.1 (1.9–2.3)	2.7 (2.3–3.1)	3.6 (1.0–6.0)	1.2 (0.4–1.9)	1.8 (-0.3–4.0)	1.5 (-0.3–3.2)	1.1 (0.7–1.5)	-1.7 (-2.9—0.5)	1.6 (-3.0–5.5)	0.4 (-0.2–1.0)	1.2 (-1.5–4.2)
Turkmenistan	0.0 (-0.2–0.2)	3.4 (3.2–3.7)	1.8 (-0.1–4.1)	-0.8 (-1.8–0.2)	2.7 (1.9–3.5)	1.0 (-5.0–7.4)	-2.3 (-2.9—1.7)	2.3 (1.9–2.8)	2.7 (-0.7–7.1)	-4.1 (-4.8—3.3)	3.8 (3.2–4.4)	1.7 (-8.5–13.4)
Turks and Caicos Islands	4.6 (4.6–4.6)	3.4 (3.4–3.4)	2.6 (2.6–2.6)	3.4 (3.4–3.4)	2.6 (2.6–2.6)	2.0 (2.0–2.0)	3.0 (3.0–3.0)	2.2 (2.2–2.2)	1.7 (1.7–1.7)	2.9 (2.9–2.9)	2.2 (2.2–2.2)	1.6 (1.6–1.6)
Tuvalu	2.1 (2.1–2.1)	3.1 (3.1–3.1)	3.1 (3.1–3.1)	1.6 (1.6–1.7)	2.4 (2.4–2.4)	2.5 (2.5–2.5)	1.3 (1.3–1.3)	1.8 (1.8–1.8)	1.9 (1.9–1.9)	1.2 (1.2–1.2)	1.7 (1.7–1.7)	1.7 (1.7–1.7)
Türkiye	2.9 (2.7–3.0)	8.4 (8.2–8.6)	3.6 (3.3–3.9)	-0.4 (-8.8–5.3)	5.1 (1.7–7.7)	0.5 (-0.4–1.4)	5.9 (-0.3–8.1)	5.2 (3.2–6.8)	0.6 (0.2–1.1)	3.4 (-11.4–13.8)	2.8 (-4.7–7.1)	-0.3 (-1.2–0.7)
Uganda	2.6 (1.6–3.5)	3.2 (2.3–4.4)	3.3 (2.3–4.9)	2.1 (-2.8–5.8)	2.4 (-0.9–7.1)	2.6 (-2.2–11.3)	0.5 (-1.2–2.3)	3.3 (1.5–4.8)	4.4 (1.3–8.0)	0.8 (-2.6–4.0)	4.1 (1.3–7.5)	3.7 (-3.5–13.9)
Ukraine	2.1 (2.0–2.2)	4.8 (4.6–4.9)	1.9 (1.3–2.6)	1.3 (0.7–1.8)	3.4 (3.0–3.8)	-0.3 (-2.5–2.0)	-0.1 (-0.3–0.2)	3.4 (3.2–3.5)	-13.6 (-14.8—12.2)	-1.9 (-2.5—1.4)	4.3 (3.9–4.7)	-18.0 (-21.9—14.1)
United Arab Emirates	2.7 (2.7–2.7)	1.8 (1.8–1.8)	4.0 (4.0–4.0)	3.3 (3.3–3.3)	2.2 (2.2–2.2)	5.0 (5.0–5.0)	1.4 (1.4–1.4)	0.9 (0.9–0.9)	2.2 (2.2–2.2)	1.2 (1.2–1.2)	0.8 (0.8–0.8)	1.9 (1.9–1.9)
United Kingdom	3.9 (3.6–4.2)	3.0 (2.7–3.2)	1.1 (0.5–1.9)	3.1 (2.5–3.7)	3.0 (2.6–3.5)	0.3 (-1.5–1.9)	1.8 (1.7–2.1)	3.7 (3.5–3.8)	-0.2 (-0.7–0.3)	0.6 (0.0–1.1)	3.3 (2.9–3.6)	-0.7 (-1.9–0.6)
United Republic of Tanzania	3.1 (2.2–4.1)	2.1 (1.4–3.3)	2.0 (0.8–4.0)	3.7 (-2.7–10.9)	2.5 (-5.2–12.3)	1.6 (-10.6–17.1)	-0.5 (-2.0–1.0)	4.8 (3.2–6.2)	1.8 (-0.7–4.6)	-1.0 (-5.9–3.3)	5.1 (2.0–8.6)	1.9 (-7.9–15.3)
United States	3.1 (3.0–3.3)	2.4 (2.2–2.5)	-0.9 (-1.2—0.4)	2.5 (2.0–3.0)	2.3 (2.0–2.7)	-2.0 (-2.9—0.9)	2.5 (2.2–2.7)	2.4 (2.2–2.5)	-0.9 (-1.5—0.4)	1.7 (1.2–2.3)	0.3 (-0.1–0.7)	0.1 (-1.5–1.9)
Uruguay	2.4 (1.9–2.9)	2.8 (2.3–3.2)	2.6 (1.8–3.3)	1.8 (0.3–3.1)	2.1 (1.3–3.0)	2.0 (-0.8–4.3)	0.6 (-0.0–1.3)	0.0 (-0.4–0.4)	-0.2 (-1.4–1.1)	-0.8 (-2.1–0.3)	-0.6 (-1.2–0.1)	-1.8 (-4.7–1.2)
Uzbekistan	2.9 (2.8–3.1)	2.3 (2.2–2.4)	-4.8 (-5.0—4.6)	2.2 (1.5–2.8)	1.8 (1.4–2.2)	-4.9 (-6.0—3.7)	0.1 (-0.2–0.4)	1.4 (1.2–1.5)	-2.6 (-3.1—2.0)	-0.6 (-1.1–0.0)	3.3 (3.0–3.7)	-0.1 (-1.8–1.7)
Vanuatu	2.6 (2.6–2.6)	2.2 (2.2–2.2)	0.8 (0.8–0.8)	2.0 (2.0–2.0)	1.7 (1.7–1.7)	0.7 (0.7–0.7)	1.5 (1.5–1.5)	1.3 (1.3–1.3)	0.5 (0.5–0.5)	1.4 (1.4–1.4)	1.2 (1.2–1.2)	0.5 (0.5–0.5)

Country	Annual rate of reduction in probability of dying $_5q_5$ (%)			Annual rate of reduction in probability of dying $_5q_{10}$ (%)			Annual rate of reduction in probability of dying $_5q_{15}$ (%)			Annual rate of reduction in probability of dying $_5q_{20}$ (%)		
	1990-2000	2000-2015	2015-2024	1990-2000	2000-2015	2015-2024	1990-2000	2000-2015	2015-2024	1990-2000	2000-2015	2015-2024
Venezuela (Bolivarian Republic of)	1.9 (1.7–2.0)	1.7 (1.5–1.9)	0.6 (-1.9–3.5)	1.1 (0.4–1.8)	0.7 (0.1–1.4)	-0.4 (-7.3–9.8)	-2.2 (-2.6–-1.9)	-2.6 (-2.9–-2.2)	2.5 (-1.1–7.1)	-3.6 (-4.4–-2.8)	-2.7 (-3.4–-1.9)	0.6 (-9.2–11.7)
Viet Nam	6.9 (6.2–8.3)	6.1 (4.9–7.3)	4.9 (1.8–7.2)	1.7 (-7.7–9.1)	1.5 (-3.0–4.6)	1.4 (-4.3–6.7)	3.2 (1.5–5.5)	2.0 (0.1–3.7)	2.1 (-0.5–5.3)	2.5 (-3.3–8.3)	2.1 (-2.1–5.3)	1.7 (-5.1–11.2)
Yemen	4.2 (3.3–5.1)	2.1 (1.2–3.3)	2.0 (0.8–3.6)	3.1 (-1.6–7.5)	1.2 (-2.6–5.6)	0.3 (-9.2–11.9)	1.2 (1.2–1.2)	-0.4 (-0.4–-0.4)	4.8 (4.8–4.8)	1.1 (1.1–1.1)	-3.0 (-3.0–-3.0)	8.5 (8.5–8.5)
Zambia	1.9 (0.1–2.9)	4.6 (3.6–6.6)	2.2 (-0.4–4.1)	2.5 (-4.0–8.7)	3.7 (-1.8–9.8)	2.4 (-8.1–15.2)	1.1 (-0.6–3.1)	4.8 (3.3–6.1)	5.1 (2.1–7.8)	0.4 (-3.0–3.3)	5.6 (3.5–8.1)	6.3 (0.4–15.1)
Zimbabwe	-0.2 (-1.2–0.6)	1.8 (0.8–3.1)	4.7 (3.4–6.7)	-2.1 (-9.1–2.9)	-0.4 (-4.2–3.2)	2.6 (-3.9–12.7)	-3.8 (-5.7–-1.4)	0.9 (-0.7–2.3)	-0.0 (-2.9–3.0)	-5.1 (-8.8–-2.0)	2.7 (0.5–5.5)	1.4 (-6.4–12.4)

Table A.7: Data series in mortality database for older children

Data series included in the mortality database for children and youths aged 5–24, by country.

Country	Data series	Inclusion
Afghanistan	National Demographic and Family Guidance Survey 1972 (Household Deaths)	1
	Census 1979 (Household Deaths)	1
	Afghanistan Mortality Survey (AMS) 2010 (Direct)	1
	Afghanistan Mortality Survey (AMS) Excluding South Zone 2010 (Direct)	1
	Afghanistan Mortality Survey (AMS) Excluding South Zone (Household data) 2010 (Household Deaths)	1
	Demographic and Health Survey 2015 (Direct)	1
	Afghanistan Health Survey 2018 (Direct)	1
	Multiple Indicator Cluster Survey 2022-2023 (Direct)	1
Angola	Malaria Indicator Survey 2011 (Direct)	0
	Census 2014 (Household Deaths)	0
	Demographic and Health Survey 2015-2016 (Direct)	0
	Demographic and Health Survey 2023-2024 (Direct)	0
Albania	Reproductive Health Survey 2002 (Direct)	1
	Demographic and Health Survey 2008-2009 (Direct)	1
	Census 2011 (Household Deaths)	1
	Demographic and Health Survey 2017-2018 (Direct)	1
	WHO VR (recalculated - unadjusted) 2025 (VR)	1
Andorra	WHO VR (recalculated - unadjusted) 2025 (VR)	0
United Arab Emirates	World Health Survey 2003 (Direct)	0
	WHO VR (recalculated - unadjusted) 2025 (VR)	0

Country	Data series	Inclusion
Argentina	WHO VR (recalculated - unadjusted) 2025 (VR)	1
Armenia	Demographic and Health Survey 2000 (Direct)	1
	Demographic and Health Survey 2005 (Direct)	1
	Demographic and Health Survey 2010 (Direct)	1
	Demographic and Health Survey 2015-2016 (Direct)	0
	WHO VR (recalculated - unadjusted) 2025 (VR)	1
Antigua and Barbuda	WHO VR (recalculated - unadjusted) 2025 (VR)	1 for 10q15, 0 for 10q5
Australia	WHO/HMD VR (recalculated - unadjusted) 2025 (VR)	1
Austria	WHO/HMD VR (recalculated - unadjusted) 2025 (VR)	1
Azerbaijan	Demographic and Health Survey 2006 (Direct)	1
	Azerbaijan National DHS 2011 (Direct)	1
	Multiple Indicator Cluster Survey 2023 (Direct)	0
	Recalculated based on WHO - adjusted for incompleteness 2025 (VR)	1
	WHO VR (recalculated - unadjusted) 2025 (VR)	0
Burundi	Population Survey 1965 (Household Deaths)	1
	Demographic Survey 1970 (Household Deaths)	1
	Demographic and Health Survey 1987 (Direct)	1
	Demographic Survey 2002 (Direct)	1
	Census 2008 (Household Deaths)	1 for 10q15, 0 for 10q5
	Demographic and Health Survey 2010-2011 (Direct)	1
	Demographic and Health Survey 2016-2017 (Direct)	1
Belgium	WHO/HMD VR (recalculated - unadjusted) 2025 (VR)	1
Benin	World Fertility Survey 1981-1982 (Direct)	1
	Demographic and Health Survey 1996 (Direct)	1
	Demographic and Health Survey 2001 (Direct)	1
	Demographic and Health Survey 2006 (Direct)	1
	Demographic and Health Survey 2011-2012 (Direct)	0
	Multiple Indicator Cluster Survey 2014 (Direct)	1
	Demographic and Health Survey 2017-2018 (Direct)	1
	Multiple Indicator Cluster Survey 2021-2022 (Direct)	1

Country	Data series	Inclusion
Burkina Faso	Survey 1960-1961 (Household Deaths)	1
	Recensement General de la Population et de l'Habitat (RGPH) 1985 (Household Deaths)	1 for 10q15, 0 for 10q5
	Recensement General de la Population et de l'Habitat (RGPH) 1996 (Household Deaths)	1 for 10q15, 0 for 10q5
	Demographic and Health Survey 1993 (Direct)	1
	Demographic and Health Survey 1998-1999 (Direct)	1
	World Health Survey 2003 (Direct)	1
	Demographic and Health Survey 2003 (Direct)	1
	Recensement General de la Population et de l'Habitat (RGPH) 2006 (Household Deaths)	1 for 10q15, 0 for 10q5
	Global Fund Evaluation Survey 2008 (Household Deaths)	1
	Demographic and Health Survey 2010 (Direct)	1
	Recensement General de la Population et de l'Habitat (RGPH) 2019 (Household Deaths)	1
	Enquete Demographique et de Sante 2021 (Direct)	1 for 10q15, 0 for 10q5
Bangladesh	Population Growth Estimation Experiment 1962-1965 (Household Deaths)	1
	Retrospective Fertility and Mortality Survey (UN SA) 1974 (Household Deaths)	1
	World Fertility Survey 1975-1976 (Direct)	1
	Demographic and Health Survey 1993-1994 (Direct)	1
	Demographic and Health Survey 1996-1997 (Direct)	1
	Demographic and Health Survey 1999-2000 (Direct)	1
	Demographic and Health Survey 1999-2000 (Household Deaths)	1
	Maternal Health Services and Maternal Mortality Survey 2001 (Direct)	0
	World Health Survey 2003 (Direct)	1
	Demographic and Health Survey 2004 (Direct)	1
	Demographic and Health Survey 2007 (Direct)	1
	Maternal Mortality and Health Care Survey 2010 (Household Deaths)	1
	Census 2011 (Household Deaths)	1
	Demographic and Health Survey 2011 (Direct)	1
	Demographic and Health Survey 2014 (Direct)	1
	Maternal Mortality and Health Care Survey 2016 (Household Deaths)	1
	Demographic and Health Survey 2017-2018 (Direct)	1

Country	Data series	Inclusion
	Multiple Indicator Cluster Survey 2019 (Direct)	1
	Demographic and Health Survey 2022 (Direct)	1
	SVR (from published reports) 2021 (VR)	1
	SVR (from published reports) 2022 (VR)	1
Bulgaria	WHO/HMD VR (recalculated - unadjusted) 2025 (VR)	1
Bahrain	WHO/UNPD VR (recalculated - unadjusted) 2025 (VR)	1
Bahamas	Recalculated based on WHO - adjusted for incompleteness 2025 (VR)	1
	WHO VR (recalculated - unadjusted) 2025 (VR)	0
Bosnia and Herzegovina	World Health Survey 2003 (Direct)	0
	WHO VR (recalculated - unadjusted) 2025 (VR)	1
Belarus	WHO/HMD VR (recalculated - unadjusted) 2025 (VR)	1
Belize	Family Health Survey 1991 (Direct)	1
	Multiple Indicator Cluster Survey 2015-2016 (Direct)	1
	Recalculated based on WHO - adjusted for incompleteness 2025 (VR)	1
	WHO VR (recalculated - unadjusted) 2025 (VR)	0
Bolivia (Plurinational State of)	Encuesta Demografica Nacional 1980 (Household Deaths)	1
	Demographic and Health Survey 1989 (Direct)	1
	Census 1992 (Household Deaths)	1 for 10q15, 0 for 10q5
	Demographic and Health Survey 1993-1994 (Direct)	1
	Demographic and Health Survey 1998 (Direct)	1
	Census 2001 (Household Deaths)	1 for 10q15, 0 for 10q5
	Demographic and Health Survey 2003 (Direct)	1
	Demographic and Health Survey 2008 (Direct)	1
	Census 2012 (Household Deaths)	0
	Demographic and Health Survey 2016 (Direct)	1
	Demographic and Health Survey (EDSA) 2023 (Direct)	0
	WHO VR (recalculated - unadjusted) 2025 (VR)	0
Brazil	Demographic and Health Survey 1986 (Direct)	1
	Demographic and Health Survey 1991 (Direct)	0
	Demographic and Health Survey 1996 (Direct)	1

Country	Data series	Inclusion
	Census 2010 (Household Deaths)	1
	Recalculated based on WHO - adjusted for incompleteness 2025 (VR)	1
	WHO VR (recalculated - unadjusted) 2025 (VR)	0
Barbados	WHO VR (recalculated - unadjusted) 2025 (VR)	1
Brunei Darussalam	WHO VR (recalculated - unadjusted) 2025 (VR)	1
Bhutan	National Health Survey 1994 (Household Deaths)	1
	National Health Survey 2000 (Household Deaths)	1
	Census 2005 (Household Deaths)	1
	Multiple Indicator Cluster Survey 2010 (Direct)	0
	National Health Survey 2012 (Household Deaths)	1
Botswana	Census 1981 (Household Deaths)	1
	Census 1991 (Household Deaths)	1
	Demographic Survey 1998 (Household Deaths)	1 for 10q15, 0 for 10q5
	Census 2001 (Household Deaths)	1
	Demographic Survey 2006 (Household Deaths)	1
	Family Health Survey 2008 (Direct)	1
	Census 2011 (Household Deaths)	1
	Demographic Survey 2017 (Household Deaths)	1
	Census 2022 (Household Deaths)	1
Central African Republic	Survey 1959-1960 (Household Deaths)	1
	Census 1988 (Household Deaths)	1
	Demographic and Health Survey 1994-1995 (Direct)	1
	Census 2003 (Household Deaths)	1
	Multiple Indicator Cluster Survey 2018-2019 (Direct)	1
Canada	WHO/HMD VR (recalculated - unadjusted) 2025 (VR)	1
Switzerland	WHO/HMD VR (recalculated - unadjusted) 2025 (VR)	1
Chile	WHO/HMD/UNPD VR (recalculated - unadjusted) 2025 (VR)	1
China	Adjusted Census Deaths (1964-1982) 1964-2000 (Household Deaths)	1
	Adjusted Census Deaths (1982-1990) 1964-2000 (Household Deaths)	1
	Adjusted Census Deaths (1990-2000) 1964-2000 (Household Deaths)	1
	Adjusted Census Deaths (1999-2000) 1964-2000 (Household Deaths)	1

Country	Data series	Inclusion
	Adjusted Census Deaths (2000-2010) 1964-2000 (Household Deaths)	1
	Population 1% Sample Survey 1964-2000 (Household Deaths)	0
	Census 1982 (Household Deaths)	0
	Intercensal Population Sample Survey of One-Percent 1987 (Household Deaths)	1 for 10q15, 0 for 10q5
	Census 1990 (Household Deaths)	0
	1994 Annual Population Change Survey 1994 (Household Deaths)	0
	Intercensal Population Sample Survey of One-Percent 1995 (Household Deaths)	1 for 10q15, 0 for 10q5
	1996 Annual Population Change Survey 1996 (Household Deaths)	0
	1997 Annual Population Change Survey 1997 (Household Deaths)	0
	1998 Annual Population Change Survey 1998 (Household Deaths)	0
	1999 Annual Population Change Survey 1999 (Household Deaths)	0
	Census 2000 (Household Deaths)	0
	2001 Annual Population Change Survey 2001 (Household Deaths)	0
	2002 Annual Population Change Survey 2002 (Household Deaths)	0
	2003 Annual Population Change Survey 2003 (Household Deaths)	0
	World Health Survey 2003 (Direct)	0
	2004 Annual Population Change Survey 2004 (Household Deaths)	0
	2006 Annual Population Change Survey 2006 (Household Deaths)	0
	China 2007 Annual Population Change Survey 2007 (Household Deaths)	1 for 10q15, 0 for 10q5
	China 2008 Annual Population Change Survey 2008 (Household Deaths)	1 for 10q15, 0 for 10q5
	China 2009 Annual Population Change Survey 2009 (Household Deaths)	0
	Census 2010 (Household Deaths)	0
	2011 Annual Population Change Survey 2011 (Household Deaths)	0
	2012 Annual Population Change Survey 2012 (Household Deaths)	0
	2013 Annual Population Change Survey 2013 (Household Deaths)	0
	2014 Annual Population Change Survey 2014 (Household Deaths)	0
	Intercensal Population Sample Survey of One-Percent 2015 (Household Deaths)	0
	Population Sample Survey 2015 (Household Deaths)	0
	2016 Annual Population Change Survey 2016 (Household Deaths)	0

Country	Data series	Inclusion
	2017 Annual Population Change Survey 2017 (Household Deaths)	0
	2018 Annual Population Change Survey 2018 (Household Deaths)	0
	China CDC Surveillance surveys 2013 (VR)	1
Côte d'Ivoire	Demographic Survey Repeated Passages 1978-1979 (Household Deaths)	1
	World Fertility Survey 1980-1981 (Direct)	1
	Demographic and Health Survey 1994 (Direct)	1
	Census 1998 (Household Deaths)	0
	Demographic and Health Survey 1998-1999 (Direct)	1
	World Health Survey 2003 (Direct)	1
	AIDS Indicator Survey 2005 (Direct)	1
	Demographic and Health Survey 2011-2012 (Direct)	1
	Multiple Indicator Cluster Survey 2016 (Direct)	1
	Enquete Demographique et de Sante 2021 (Direct)	1
Cameroon	Census 1976 (Household Deaths)	1
	World Fertility Survey 1978 (Direct)	1
	Census 1987 (Household Deaths)	1
	Demographic and Health Survey 1991 (Direct)	1
	Demographic and Health Survey 1998 (Direct)	1
	Demographic and Health Survey 2004 (Direct)	1
	Census 2005 (Household Deaths)	1
	Demographic and Health Survey 2011 (Direct)	1
	Multiple Indicator Cluster Survey 2014 (Direct)	1
	Demographic and Health Survey 2018 (Direct)	1
Democratic Republic of the Congo	SPS 1955-1957 (Household Deaths)	1
	Demographic and Health Survey 2007 (Direct)	1
	Demographic and Health Survey 2013-2014 (Direct)	1
	Multiple Indicator Cluster Survey 2017-2018 (Direct)	1
	Demographic and Health Survey 2023-2024 (Direct)	1
Congo	Census 1974 (Household Deaths)	0
	Census 1984 (Household Deaths)	0
	World Health Survey 2003 (Direct)	0

Country	Data series	Inclusion
	Demographic and Health Survey 2005 (Direct)	1
	Demographic and Health Survey 2011-2012 (Direct)	1
	Multiple Indicator Cluster Survey 2014-2015 (Direct)	1
Cook Islands	WHO VR (recalculated - unadjusted) 2025 (VR)	1 for 10q15, 0 for 10q5
Colombia	Demographic and Health Survey 1986 (Direct)	1
	Demographic and Health Survey 1990 (Direct)	1
	Demographic and Health Survey 1995 (Direct)	1
	Demographic and Health Survey 2000 (Direct)	1
	Census 2005 (Household Deaths)	0 for 10q15, 1 for 10q5
	Demographic and Health Survey 2005 (Direct)	1
	Demographic and Health Survey 2010 (Direct)	1
	Demographic and Health Survey 2015 (Direct)	1
	Census 2018 (Household Deaths)	0
	Recalculated based on WHO - adjusted for incompleteness 2025 (VR)	1
	WHO VR (recalculated - unadjusted) 2025 (VR)	0
Comoros	Census 1958 (Household Deaths)	1
	Census 1980 (Household Deaths)	1
	Demographic and Health Survey 1996 (Direct)	1
	Census 2003 (Household Deaths)	1
	World Health Survey 2003 (Direct)	0
	Demographic and Health Survey 2012 (Direct)	1
	Census 2017 (Household Deaths)	1
	Multiple Indicator Cluster Survey 2022 (Direct)	1
Cabo Verde	Demographic and Reproductive Health Survey 1998 (Direct)	1
	Census 2000 (Household Deaths)	1
	Census 2010 (Household Deaths)	1
	Recalculated based on WHO/UNPD - adjusted for incompleteness 2025 (VR)	1
	WHO/UNPD VR (recalculated - unadjusted) 2025 (VR)	0
Costa Rica	WHO VR (recalculated - unadjusted) 2025 (VR)	1
Cuba	WHO VR (recalculated - unadjusted) 2025 (VR)	1

Country	Data series	Inclusion
Cyprus	Recalculated based on WHO - adjusted for incompleteness 2025 (VR)	1
	WHO VR (recalculated - unadjusted) 2025 (VR)	0
Czechia	WHO/HMD VR (recalculated - unadjusted) 2025 (VR)	1
Germany	WHO/HMD VR (recalculated - unadjusted) 2025 (VR)	1
Djibouti	PAPFAM Family Health Survey 2002 (Direct)	0
	PAPFAM Family Health Survey 2012 (Direct)	0
Dominica	WHO VR (recalculated - unadjusted) 2025 (VR)	1
Denmark	WHO/HMD VR (recalculated - unadjusted) 2025 (VR)	1
Dominican Republic	World Fertility Survey 1975 (Direct)	1
	World Fertility Survey 1980 (Direct)	1
	Demographic and Health Survey 1986 (Direct)	1
	Demographic and Health Survey 1991 (Direct)	1
	Demographic and Health Survey 1996 (Direct)	1
	Demographic and Health Survey 1999 (Direct)	0
	Demographic and Health Survey 2002 (Direct)	1
	World Health Survey 2003 (Direct)	1
	Demographic and Health Survey 2007 (Direct)	1
	Census 2010 (Household Deaths)	1 for 10q15, 0 for 10q5
	Demographic and Health Survey 2013 (Household Deaths)	1
	Demographic and Health Survey 2013 (Direct)	1
	Multiple Indicator Cluster Survey 2014 (Direct)	1
	Multiple Indicator Cluster Survey 2019 (Direct)	1
	WHO VR (recalculated - unadjusted) 2025 (VR)	0
Algeria	Demographic Survey 1970 (Household Deaths)	1
	PAPCHILD Maternal and Child Health Survey 1992 (Direct)	1
	PAPFAM Family Health Survey 2002 (Direct)	1
	Multiple Indicator Cluster Survey 2012-2013 (Direct)	0
	Multiple Indicator Cluster Survey 2018-2019 (Direct)	0
	WHO VR (recalculated - unadjusted) 2025 (VR)	1
Ecuador	Demographic and Family Health Survey 1987 (Direct)	1
	Demographic and Maternal and Child Health Survey 1994 (Direct)	1

Country	Data series	Inclusion
	Demographic and Maternal and Child Health Survey 1999 (Direct)	1
	World Health Survey 2003 (Direct)	1
	Demographic and Maternal and Child Health Survey 2004 (Direct)	1
	National Health and Nutrition Survey 2012 (Household Deaths)	1
	National Health and Nutrition Survey 2012 (Direct)	1
	Encuesta Nacional de Salud y Nutricion (ENSANUT) 2018 (Direct)	1
	Census 2022 (Household Deaths)	0
	Recalculated based on WHO - adjusted for incompleteness 2025 (VR)	1
	WHO VR (recalculated - unadjusted) 2025 (VR)	0
Egypt	World Fertility Survey 1980 (Direct)	1
	Demographic and Health Survey 1988 (Direct)	1
	PAPCHILD Maternal and Child Health Survey 1991 (Direct)	1
	Demographic and Health Survey 1992 (Direct)	1
	Demographic and Health Survey 1995 (Direct)	1
	Demographic and Health Survey 2000 (Direct)	1
	Demographic and Health Survey 2003 (Direct)	1
	Demographic and Health Survey 2005 (Direct)	1
	Demographic and Health Survey 2008 (Direct)	1
	Demographic and Health Survey 2014 (Direct)	1
	Family Health Survey 2021 (Direct)	1
	Recalculated based on WHO - adjusted for incompleteness 2025 (VR)	1
	WHO VR (recalculated - unadjusted) 2025 (VR)	0
Eritrea	Demographic and Health Survey 1995-1996 (Direct)	1
	Demographic and Health Survey 2002 (Direct)	1
	Population and Health Survey 2010 (Direct)	1
Spain	WHO/HMD VR (recalculated - unadjusted) 2025 (VR)	1
Estonia	Census 2011-2012 (Household Deaths)	1
	WHO/HMD VR (recalculated - unadjusted) 2025 (VR)	1
Ethiopia	Demographic Survey 1981 (Household Deaths)	0
	Census 1984 (Household Deaths)	1 for 10q15, 0 for 10q5
	Demographic and Health Survey 2000 (Direct)	1

Country	Data series	Inclusion
	World Health Survey 2003 (Direct)	1
	Demographic and Health Survey 2005 (Direct)	1
	Census 2007 (Household Deaths)	1 for 10q15, 0 for 10q5
	Demographic and Health Survey 2011 (Direct)	1
	Mini Demographic and Health Survey 2014 (Direct)	1
	Demographic and Health Survey 2016 (Direct)	1
	Mini Demographic and Health Survey 2019 (Direct)	1
Finland	WHO/HMD VR (recalculated - unadjusted) 2025 (VR)	1
Fiji	World Fertility Survey 1974 (Direct)	1
	Multiple Indicator Cluster Survey 2021 (Direct)	1
	WHO VR (recalculated - unadjusted) 2025 (VR)	1
France	WHO/HMD VR (recalculated - unadjusted) 2025 (VR)	1
Micronesia (Federated States of)	WHO VR (recalculated - unadjusted) 2025 (VR)	0
Gabon	Demographic and Health Survey 2000 (Direct)	1
	Demographic and Health Survey 2012 (Direct)	1
	Demographic and Health Survey 2019-2021 (Direct)	1
United Kingdom	WHO/HMD VR (recalculated - unadjusted) 2025 (VR)	1
Georgia	Reproductive Health Survey 1999-2000 (Direct)	1
	World Health Survey 2003 (Direct)	0
	Reproductive Health Survey 2005 (Direct)	1
	Reproductive Health Survey 2010 (Direct)	1
	WHO VR (recalculated - unadjusted) 2025 (VR)	1
Ghana	World Fertility Survey 1979-1980 (Direct)	0
	Demographic and Health Survey 1988 (Direct)	1
	Demographic and Health Survey 1993-1994 (Direct)	1
	Demographic and Health Survey 1998-1999 (Direct)	1
	Demographic and Health Survey 2003 (Direct)	1
	World Health Survey 2003 (Direct)	1
	Maternal Health Survey 2007 (Direct)	1
	Demographic and Health Survey 2008 (Direct)	1

Country	Data series	Inclusion
	Census 2010 (Household Deaths)	1
	Multiple Indicator Cluster Survey 2011 (Direct)	1
	Demographic and Health Survey 2014 (Direct)	1
	Maternal Health Survey 2017 (Direct)	1
	Maternal Health Survey 2017 (Others)	1
	Multiple Indicator Cluster Survey 2017-2018 (Direct)	1
	Census 2021 (Household Deaths)	0
	Demographic and Health Survey 2022 (Direct)	1
Guinea	Survey 1954-1955 (Household Deaths)	1
	Census 1983 (Household Deaths)	0
	Census 1996 (Household Deaths)	1
	Demographic and Health Survey 1999 (Direct)	1
	Demographic and Health Survey 2005 (Direct)	1
	Demographic and Health Survey 2012 (Direct)	1
	Census 2014 (Household Deaths)	1
	Multiple Indicator Cluster Survey 2016 (Direct)	0
	Demographic and Health Survey 2018 (Direct)	1
Gambia	Census 2003 (Household Deaths)	0
	Demographic and Health Survey 2013 (Direct)	0
	Census 2013 (Household Deaths)	0
	Multiple Indicator Cluster Survey 2018 (Direct)	0
	Demographic and Health Survey 2019-2020 (Direct)	0
Guinea-Bissau	Census 2009 (Household Deaths)	0
	Multiple Indicator Cluster Survey 2010 (Direct)	1
	Multiple Indicator Cluster Survey 2014 (Direct)	1 for 10q5, 0 for 10q15
	Multiple Indicator Cluster Survey 2018-2019 (Direct)	1
Equatorial Guinea	Census 1994 (Household Deaths)	0
Greece	WHO/HMD VR (recalculated - unadjusted) 2025 (VR)	1
Grenada	WHO VR (recalculated - unadjusted) 2025 (VR)	1
Guatemala	Demographic and Health Survey 1987 (Direct)	1
	Demographic and Health Survey 1995 (Direct)	1

Country	Data series	Inclusion
	Demographic and Health Survey 1998-1999 (Direct)	1
	Encuesta Nacional de Salud Materno Infantil (ENSMI) 2002 (Direct)	1
	Encuesta Nacional de Salud Materno Infantil (ENSMI) 2008-2009 (Direct)	1
	Demographic and Health Survey 2014-2015 (Direct)	1
	WHO VR (recalculated - unadjusted) 2025 (VR)	1
Guyana	World Fertility Survey 1975 (Direct)	1
	AIDS Indicator Survey 2005 (Direct)	1
	Demographic and Health Survey 2009 (Direct)	1
	Census 2012 (Household Deaths)	0 for 10q15, 1 for 10q5
	Multiple Indicator Cluster Survey 2014 (Direct)	1
	Multiple Indicator Cluster Survey 2019-2020 (Direct)	0
	WHO VR (recalculated - unadjusted) 2025 (VR)	1
Honduras	Encuesta Demografica Nacional 1972 (Household Deaths)	0 for 10q15, 1 for 10q5
	National Demographic Survey 1983 (Household Deaths)	1 for 10q5, 0 for 10q15
	Reproductive Health Survey 1996 (Direct)	1
	Census 2001 (Household Deaths)	0 for 10q15, 1 for 10q5
	Reproductive Health Survey 2001 (Direct)	1
	Demographic and Health Survey 2005-2006 (Direct)	1
	Demographic and Health Survey 2011-2012 (Direct)	1
	Census 2013 (Household Deaths)	0 for 10q15, 1 for 10q5
	Multiple Indicator Cluster Survey 2019 (Direct)	1
	WHO VR (recalculated - unadjusted) 2025 (VR)	0
Croatia	Census 2011 (Household Deaths)	1
	WHO/HMD VR (recalculated - unadjusted) 2025 (VR)	1
Haiti	Census 2003 (Household Deaths)	0
	Census 1971 (Household Deaths)	0
	World Fertility Survey 1977 (Direct)	1
	Demographic and Health Survey 1994-1995 (Direct)	1
	Demographic and Health Survey 2000 (Direct)	1

Country	Data series	Inclusion
	Demographic and Health Survey 2005-2006 (Direct)	1
	Demographic and Health Survey 2012 (Direct)	1
	Demographic and Health Survey 2016-2017 (Direct)	1
	WHO VR (recalculated - unadjusted) 2025 (VR)	0
Hungary	WHO/HMD VR (recalculated - unadjusted) 2025 (VR)	1
Indonesia	National Socio-economic Survey 1964-1965 (Household Deaths)	1
	World Fertility Survey 1976 (Direct)	1
	Demographic and Health Survey 1987 (Direct)	1
	Demographic and Health Survey 1991 (Direct)	1
	Demographic and Health Survey 1994 (Direct)	1
	Demographic and Health Survey 1997 (Direct)	0 for 10q15, 1 for 10q5
	Demographic and Health Survey 2002-2003 (Direct)	1
	Demographic and Health Survey 2007 (Direct)	1
	Census 2010 (Household Deaths)	1
	Demographic and Health Survey 2012 (Direct)	1
	Demographic and Health Survey 2017 (Direct)	1
India	Census 1971 (Household Deaths)	1
	Census 1981 (Household Deaths)	1
	National Family Health Survey 1992-1993 (Direct)	1
	National Family Health Survey 1992-1993 (Household Deaths)	1
	National Family Health Survey 1998-1999 (Direct)	1
	National Family Health Survey 1998-1999 (Household Deaths)	1
	World Health Survey 2003 (Direct)	1
	National Family Health Survey 2005-2006 (Direct)	1
	National Family Health Survey 2015-2016 (Direct)	0
	National Family Health Survey 2020 (Direct)	0
	Sample Registration System 2023 (VR)	1
Ireland	Census 2011 (Household Deaths)	1 for 10q15, 0 for 10q5
	WHO/HMD VR (recalculated - unadjusted) 2025 (VR)	1
Iran (Islamic Republic of)	Population Growth Survey 1973-1976 (Household Deaths)	0 for 10q15, 1 for 10q5

Country	Data series	Inclusion
	Census 1986 (Household Deaths)	1
	Census 1991 (Household Deaths)	1
	Demographic and Health Survey 2000 (Household Deaths)	1
	Census 1976 (Household Deaths)	1
	Recalculated based on WHO - adjusted for incompleteness 2025 (VR)	1
	WHO VR (recalculated - unadjusted) 2025 (VR)	0
Iraq	Demographic Sample Survey and Sample Registration System 1973 (Household Deaths)	0 for 10q15, 1 for 10q5
	Iraq Family Health Survey 2006 (Direct)	0
	Multiple Indicator Cluster Survey 2006 (Direct)	1
	Multiple Indicator Cluster Survey 2011 (Direct)	1
	Multiple Indicator Cluster Survey 2018 (Direct)	1
	WHO VR (recalculated - unadjusted) 2025 (VR)	0
Iceland	WHO/HMD VR (recalculated - unadjusted) 2025 (VR)	1
Israel	WHO/HMD VR (recalculated - unadjusted) 2025 (VR)	1
Italy	WHO/HMD VR (recalculated - unadjusted) 2025 (VR)	1
Jamaica	World Fertility Survey 1975-1976 (Direct)	0
	Census 2011 (Household Deaths)	0
	WHO VR (recalculated - unadjusted) 2025 (VR)	0
Jordan	Demographic and Health Survey 1990 (Direct)	1
	Demographic and Health Survey 1997 (Direct)	1
	Demographic and Health Survey 2002 (Direct)	1
	Demographic and Health Survey 2007 (Direct)	1
	Demographic and Health Survey 2009 (Direct)	1
	Demographic and Health Survey 2012 (Direct)	1
	Census 2015 (Household Deaths)	0
	Demographic and Health Survey 2017 (Direct)	1
	Demographic and Health Survey 2023 (Direct)	1
	Recalculated based on WHO - adjusted for incompleteness 2025 (VR)	1
	WHO VR (recalculated - unadjusted) 2025 (VR)	0
Japan	WHO/HMD VR (recalculated - unadjusted) 2025 (VR)	1
Kazakhstan	Demographic and Health Survey 1995 (Direct)	1

Country	Data series	Inclusion
	Demographic and Health Survey 1999 (Direct)	1
	World Health Survey 2003 (Direct)	1
	Multiple Indicator Cluster Survey 2024 (Direct)	0
	WHO VR (recalculated - unadjusted) 2025 (VR)	1
Kenya	World Fertility Survey 1977-1978 (Direct)	1
	Demographic and Health Survey 1989 (Direct)	1
	Demographic and Health Survey 1993 (Direct)	1
	Demographic and Health Survey 1998 (Direct)	1
	Demographic and Health Survey 2003 (Direct)	1
	World Health Survey 2003 (Direct)	1
	Demographic and Health Survey 2008-2009 (Direct)	1
	Census 2009 (Household Deaths)	1 for 10q15, 0 for 10q5
	Demographic and Health Survey 2014 (Direct)	1
	Census 2019 (Household Deaths)	0
	Demographic and Health Survey 2022 (Direct)	1
	WHO VR (recalculated - unadjusted) 2025 (VR)	0
Kyrgyzstan	Demographic and Health Survey 1997 (Direct)	1
	Demographic and Health Survey 2012 (Direct)	1
	Multiple Indicator Cluster Survey 2014 (Direct)	1
	Multiple Indicator Cluster Survey 2018 (Direct)	1
	Multiple Indicator Cluster Survey 2023 (Direct)	1
	WHO VR (recalculated - unadjusted) 2025 (VR)	1
Cambodia	Demographic and Health Survey 2000 (Direct)	1
	Demographic and Health Survey 2005 (Direct)	1
	Demographic and Health Survey 2010 (Direct)	1
	Demographic and Health Survey 2014 (Direct)	1
	Demographic and Health Survey 2021-2022 (Direct)	1 for 10q15, 0 for 10q5
Kiribati	Multiple Indicator Cluster Survey 2018-2019 (Direct)	0
	WHO VR (recalculated - unadjusted) 2025 (VR)	0
Saint Kitts and Nevis	WHO VR (recalculated - unadjusted) 2025 (VR)	1 for 10q15, 0 for 10q5

Country	Data series	Inclusion
Republic of Korea	World Fertility Survey 1974 (Direct)	1
	National Life Table 1970-2015 2017 (VR)	0
	WHO/HMD VR (recalculated - unadjusted) 2025 (VR)	1
Kuwait	WHO VR (recalculated - unadjusted) 2025 (VR)	1
Lao People's Democratic Republic	Census 1995 (Household Deaths)	1
	World Health Survey 2003 (Direct)	1
	Census 2005 (Household Deaths)	1
	Reproductive Health Survey 2005 (Direct)	1
	Lao Social Indicator Survey (combined MICS4/DHS) 2012 (Direct)	1
	Multiple Indicator Cluster Survey 2012 (Direct)	0
	Census 2015 (Household Deaths)	1 for 10q15, 0 for 10q5
	Lao Social Indicator Survey II 2017 (Direct)	1
	Lao Social Indicator Survey III 2023 (Direct)	1
Lebanon	Maternal and Child Health Survey 1996 (Direct)	0
	PAPFAM Family Health Survey 2004 (Direct)	0
	WHO VR (recalculated - unadjusted) 2025 (VR)	0
Liberia	Population Growth Survey 1969-1970 (Household Deaths)	1
	Demographic and Health Survey 1986 (Direct)	1
	Demographic and Health Survey 2006-2007 (Direct)	1
	Population and Housing Census 2008 (Household Deaths)	0
	Malaria Indicator Survey 2008-2009 (Direct)	1
	Demographic and Health Survey 2013 (Direct)	1
	Demographic and Health Survey 2019-2020 (Direct)	1
Libya	PAPCHILD Maternal and Child Health Survey 1995 (Direct)	1
	PAPFAM Family Health Survey 2007 (Direct)	1
	WHO VR (recalculated - unadjusted) 2025 (VR)	0
Saint Lucia	WHO VR (recalculated - unadjusted) 2025 (VR)	1
Sri Lanka	Demographic and Health Survey 1987 (Direct)	1
	Demographic and Health Survey 1993 (Direct)	1
	Demographic and Health Survey 2000 (Direct)	1
	World Health Survey 2003 (Direct)	1

Country	Data series	Inclusion
	Demographic and Health Survey 2007 (Direct)	1
	Demographic and Health Survey 2016 (Direct)	1
	WHO VR (recalculated - unadjusted) 2025 (VR)	1
Lesotho	Demographic Survey 1971-1973 (Household Deaths)	0 for 10q15, 1 for 10q5
	World Fertility Survey 1977 (Household Deaths)	0
	World Fertility Survey 1977 (Direct)	1
	Census 1986 (Household Deaths)	0
	Census 1996 (Household Deaths)	1 for 10q15, 0 for 10q5
	Demographic Survey 2001 (Household Deaths)	1
	Demographic and Health Survey 2004 (Direct)	1
	Census 2006 (Household Deaths)	0
	Demographic and Health Survey 2009 (Direct)	1
	Demographic and Health Survey 2014 (Direct)	1
	Census 2016 (Household Deaths)	1
	Multiple Indicator Cluster Survey 2018 (Direct)	1
	Demographic and Health Survey 2023-2024 (Direct)	1
Lithuania	Census 2011 (Household Deaths)	1
	Statistical Survey 2018 (Household Deaths)	1
	WHO/HMD VR (recalculated - unadjusted) 2025 (VR)	1
Luxembourg	WHO/HMD VR (recalculated - unadjusted) 2025 (VR)	1
Latvia	WHO/HMD VR (recalculated - unadjusted) 2025 (VR)	1
Morocco	World Fertility Survey 1980 (Direct)	1
	Demographic and Health Survey 1987 (Direct)	1
	Demographic and Health Survey 1992 (Direct)	1
	Demographic and Health Survey 1995 (Direct)	1
	PAPCHILD Maternal and Child Health Survey 1997 (Direct)	1
	World Health Survey 2003 (Direct)	1
	Demographic and Health Survey 2003-2004 (Direct)	1
	National Demographic Survey with repeated passages 2009-2010 (Household Deaths)	1
	WHO VR (recalculated - unadjusted) 2025 (VR)	0

Country	Data series	Inclusion
Monaco	WHO VR (recalculated - unadjusted) 2025 (VR)	0
Republic of Moldova	Demographic and Health Survey 2005 (Direct)	1
	Multiple Indicator Cluster Survey 2012 (Direct)	1
	WHO VR (recalculated - unadjusted) 2025 (VR)	1
Madagascar	Enquete demographique 1966 (Household Deaths)	0
	Demographic and Health Survey 1992 (Direct)	1
	Census 1993 (Household Deaths)	1
	Demographic and Health Survey 1997 (Direct)	1
	Demographic and Health Survey 2003-2004 (Direct)	1
	Demographic and Health Survey 2008-2009 (Direct)	1
	Census 2018 (Household Deaths)	1
	Multiple Indicator Cluster Survey 2018 (Direct)	1
	Enquete Demographique et de Sante (EDSMDV) 2021 (Direct)	1
Maldives	Demographic and Health Survey 2009 (Direct)	1
	Demographic and Health Survey 2016-2017 (Direct)	1
	Recalculated based on WHO - adjusted for incompleteness 2025 (VR)	1
	WHO VR (recalculated - unadjusted) 2025 (VR)	0
Mexico	Demographic and Health Survey 1987 (Direct)	1
	World Health Survey 2003 (Direct)	0
	Encuesta Nacional de la Dinamica Demografica (ENADID) 2009 (Direct)	1
	Encuesta Nacional de la Dinamica Demografica (ENADID) 2014 (Direct)	1
	Encuesta Nacional de la Dinamica Demografica (ENADID) 2018 (Direct)	1
	Encuesta Nacional de la Dinamica Demografica (ENADID) 2023 (Direct)	1
	WHO VR (recalculated - unadjusted) 2025 (VR)	1
Marshall Islands	WHO VR (recalculated - unadjusted) 2025 (VR)	0
North Macedonia	Multiple Indicator Cluster Survey 2018-2019 (Direct)	1
	Recalculated based on WHO - adjusted for incompleteness 2025 (VR)	1
	WHO VR (recalculated - unadjusted) 2025 (VR)	0
Mali	Census 1976 (Household Deaths)	0 for 10q15, 1 for 10q5
	Census 1987 (Household Deaths)	1 for 10q15, 0 for 10q5

Country	Data series	Inclusion
	Demographic and Health Survey 1987 (Direct)	1
	Demographic and Health Survey 1995-1996 (Direct)	1 for 10q5, 0 for 10q15
	Census 1998 (Household Deaths)	0 for 10q15, 1 for 10q5
	Demographic and Health Survey 2001 (Direct)	1
	World Health Survey 2003 (Direct)	1
	Demographic and Health Survey 2006 (Direct)	1
	Census 2009 (Household Deaths)	0
	Demographic and Health Survey 2012-2013 (Direct)	1 for 10q15, 0 for 10q5
	Multiple Indicator Cluster Survey 2015 (Direct)	1
	Demographic and Health Survey 2018 (Direct)	1
	Demographic and Health Survey 2023-2024 (Direct)	1
Malta	WHO VR (recalculated - unadjusted) 2025 (VR)	1
Myanmar	Population Change and Fertility Survey 1991 (Household Deaths)	1
	Census 2014 (Household Deaths)	1
	Demographic and Health Survey 2015-2016 (Direct)	1
	WHO VR (recalculated - unadjusted) 2025 (VR)	0
Montenegro	WHO VR (recalculated - unadjusted) 2025 (VR)	1
Mongolia	Census 1989 (Household Deaths)	0
	Reproductive Health Survey 1998 (Direct)	1
	Census 2000 (Household Deaths)	0
	Census 2010 (Household Deaths)	1
	Social Indicator Sample Survey (SISS) 2013-2014 (Direct)	1
	Multiple Indicator Cluster Survey 2018 (Direct)	1
	Recalculated based on WHO - adjusted for incompleteness 2025 (VR)	1
	WHO VR (recalculated - unadjusted) 2025 (VR)	0
Mozambique	Demographic and Health Survey 1997 (Direct)	1 for 10q15, 0 for 10q5
	Census 1997 (Household Deaths)	1
	Demographic and Health Survey 2003-2004 (Direct)	1
	Census 2007 (Household Deaths)	0

Country	Data series	Inclusion
	Multiple Indicator Cluster Survey 2008 (Direct)	1
	Demographic and Health Survey 2011 (Direct)	1
	Census 2017 (Household Deaths)	0
	Demographic and Health Survey 2022-2023 (Direct)	1
Mauritania	Census 1977 (Household Deaths)	1
	World Fertility Survey 1981-1982 (Direct)	1
	Census 1988 (Household Deaths)	1
	Maternal and Child Health Survey 1990 (Direct)	1
	Demographic and Health Survey 2000-2001 (Direct)	1
	World Health Survey 2003 (Direct)	0
	Multiple Indicator Cluster Survey 2011 (Direct)	1
	Census 2013 (Household Deaths)	0
	Multiple Indicator Cluster Survey 2015 (Direct)	1
	Demographic and Health Survey 2019-2021 (Direct)	1
Montserrat	WHO VR (recalculated - unadjusted) 2025 (VR)	0
Mauritius	WHO VR (recalculated - unadjusted) 2025 (VR)	1
Malawi	Population Change Survey 1970-1972 (Household Deaths)	1
	Census 1977 (Household Deaths)	0 for 10q15, 1 for 10q5
	Family Formation Survey 1984 (Household Deaths)	0 for 10q15, 1 for 10q5
	Census 1987 (Household Deaths)	0 for 10q15, 1 for 10q5
	Demographic and Health Survey 1992 (Direct)	1
	Census 1998 (Household Deaths)	1 for 10q15, 0 for 10q5
	Demographic and Health Survey 2000 (Direct)	1
	World Health Survey 2003 (Direct)	1
	Demographic and Health Survey 2004 (Direct)	1
	Second Integrated Household Survey 2004-2005 (Household Deaths)	1
	Multiple Indicator Cluster Survey 2006 (Direct)	1
	Census 2008 (Household Deaths)	1 for 10q15, 0 for 10q5
	Demographic and Health Survey 2010 (Household Deaths)	0

Country	Data series	Inclusion
	Demographic and Health Survey 2010 (Direct)	1
	MDG Endline Survey 2013-2014 (Direct)	1
	Malaria Indicator Survey 2014 (Direct)	1
	Demographic and Health Survey 2015-2016 (Direct)	1
	Census 2018 (Household Deaths)	1
	Multiple Indicator Cluster Survey 2019-2020 (Direct)	1
Malaysia	World Fertility Survey 1974 (Direct)	1
	World Health Survey 2003 (Direct)	0
	WHO VR (recalculated - unadjusted) 2025 (VR)	1
Namibia	Demographic and Health Survey 1992 (Direct)	1
	Demographic and Health Survey 2000 (Direct)	1
	Census 2001 (Household Deaths)	1
	World Health Survey 2003 (Direct)	1
	Demographic and Health Survey 2006-2007 (Direct)	1
	Census 2011 (Household Deaths)	1
	Demographic and Health Survey 2013 (Direct)	1
	Inter-censal Demographic Survey 2016 (Household Deaths)	1
	Census 2023 (Household Deaths)	1
	WHO VR (recalculated - unadjusted) 2025 (VR)	0
Niger	Demographic and Health Survey 1992 (Direct)	1
	Demographic and Health Survey 1998 (Direct)	1
	Census 2001 (Household Deaths)	1
	Demographic and Health Survey 2006 (Direct)	1
	Demographic and Health Survey 2012 (Direct)	1
	Enquete Nationale sur la Fecondite et la Mortalite des Enfants de moins de 5 ans (ENAFEME) 2021 (Direct)	1
Nigeria	Rural Demographic Sample Survey 1965-1966 (Household Deaths)	1
	World Fertility Survey 1981-1982 (Direct)	0
	Demographic and Health Survey 1990 (Direct)	1
	Demographic and Health Survey 1999 (Direct)	0
	Demographic and Health Survey 2003 (Direct)	1
	Demographic and Health Survey 2008 (Direct)	1

Country	Data series	Inclusion
	Demographic and Health Survey 2008 (Household Deaths)	0
	Malaria Indicator Survey 2010 (Direct)	1
	GHS Panel Survey 2010 (Household Deaths)	1 for 10q15, 0 for 10q5
	GHS Panel Survey 2012-2013 (Household Deaths)	1 for 10q15, 0 for 10q5
	Demographic and Health Survey 2013 (Direct)	1
	Demographic and Health Survey 2013 (Household Deaths)	0
	Multiple Indicator Cluster Survey 2017 (Direct)	0
	Demographic and Health Survey 2018 (Direct)	1
	Multiple Indicator Cluster Survey 2021 (Direct)	1
	Demographic and Health Survey 2024 (Direct)	1
Nicaragua	Census 1995 (Household Deaths)	0
	Demographic and Health Survey 1998 (Direct)	1
	Demographic and Health Survey 2001 (Household Deaths)	0
	Living Standards Measurement Survey 2001 (Household Deaths)	0 for 10q15, 1 for 10q5
	Demographic and Health Survey 2001 (Direct)	1
	Census 2005 (Household Deaths)	0
	Nicaraguense de Demografia y Salud (ENDESA) 2006-2007 (Direct)	1
	Encuesta Nicaraguense de Demografia y Salud 2011-2012 (Direct)	1
	WHO VR (recalculated - unadjusted) 2025 (VR)	0
Niue	WHO VR (recalculated - unadjusted) 2025 (VR)	0
Netherlands (Kingdom of the)	WHO/HMD VR (recalculated - unadjusted) 2025 (VR)	1
Norway	WHO/HMD VR (recalculated - unadjusted) 2025 (VR)	1
Nepal	Census 1981 (Household Deaths)	0
	Census 1991 (Household Deaths)	0
	Fertility and Family Planning Survey 1991 (Direct)	1
	Demographic and Health Survey 1996 (Direct)	1
	Census 2001 (Household Deaths)	1
	Census 2011 (Household Deaths)	1
	Demographic and Health Survey 2001 (Direct)	1
	World Health Survey 2003 (Direct)	1

Country	Data series	Inclusion
	Demographic and Health Survey 2006 (Direct)	1
	Demographic and Health Survey 2011 (Direct)	1
	Multiple Indicator Cluster Survey 2014 (Direct)	1
	Demographic and Health Survey 2016 (Direct)	1
	Multiple Indicator Cluster Survey 2019 (Direct)	1
	Demographic and Health Survey 2022 (Direct)	1
Nauru	Census 2021 (Household Deaths)	0
	Census 2011 (Household Deaths)	0
	WHO VR (recalculated - unadjusted) 2025 (VR)	0
New Zealand	Census 2006 (Household Deaths)	1
	WHO/HMD VR (recalculated - unadjusted) 2025 (VR)	1
Oman	Recalculated based on WHO - adjusted for incompleteness 2025 (VR)	0
	WHO VR (recalculated - unadjusted) 2025 (VR)	0
Pakistan	Population Growth Estimation Experiment 1962-1965 (Household Deaths)	1
	Population Growth Survey I 1971 (Household Deaths)	1
	Demographic and Health Survey 1990-1991 (Direct)	1
	World Health Survey 2003 (Direct)	0
	Demographic Survey 2000-2007 (Household Deaths)	1
	Demographic and Health Survey 2006-2007 (Direct)	1
	Demographic and Health Survey 2006-2007 (Household Deaths)	1
	Demographic and Health Survey 2012-2013 (Direct)	1
	Demographic and Health Survey 2017-2018 (Direct)	1
	Pakistan 2020 Demographic Survey 2021 (Household Deaths)	1
	Household Integrated Economic Survey 2024-2025 (Direct)	0
	Pakistan Demographic Survey 1984-1999 (VR)	1
Panama	Census 2000 (Household Deaths)	0
	Census 2023 (Household Deaths)	0
	TABLAS DE VIDA NACIONALES (VR)	0
	Recalculated based on WHO - adjusted for incompleteness 2025 (VR)	1
	WHO VR (recalculated - unadjusted) 2025 (VR)	0
Peru	Demographic and Health Survey 1986 (Direct)	1

Country	Data series	Inclusion
	Demographic and Health Survey 1991-1992 (Direct)	1
	Demographic and Health Survey 1996 (Direct)	1
	Demographic and Health Survey 2000 (Direct)	1
	Demographic and Health Survey 2006 (Direct)	1
	Demographic and Health Survey 2004-2008 (Direct)	1
	Demographic and Health Survey 2008 (Direct)	1
	Encuesta Demografica y de Salud Familiar (ENDES) 2009 (Direct)	0 for 10q15, 1 for 10q5
	Encuesta Demografica y de Salud Familiar (ENDES) 2010 (Direct)	0 for 10q15, 1 for 10q5
	Encuesta Demografica y de Salud Familiar (ENDES) 2011 (Direct)	1
	Demographic and Health Survey 2012 (Direct)	1 for 10q5, 0 for 10q15
	Demographic and Health Survey 2013 (Direct)	1
	Encuesta Demografica y de Salud Familiar 2014-2015 (Direct)	1
	Encuesta Demografica y de Salud Familiar 2016-2017 (Direct)	1
	Encuesta Demografica y de Salud Familiar 2018-2019 (Direct)	1
	Encuesta Demografica y de Salud Familiar 2020-2021 (Direct)	1
	Encuesta Demografica y de Salud Familiar 2022-2023 (Direct)	1
	Recalculated based on WHO - adjusted for incompleteness 2025 (VR)	1
	WHO VR (recalculated - unadjusted) 2025 (VR)	0
Philippines	World Fertility Survey 1978 (Direct)	1
	Demographic and Health Survey 1993 (Direct)	1
	Demographic and Health Survey 1998 (Direct)	1
	Demographic and Health Survey 2003 (Direct)	1
	World Health Survey 2003 (Direct)	0
	Demographic and Health Survey 2008 (Direct)	1
	National Demographic and Health Survey 2013 (Direct)	1
	Demographic and Health Survey 2017 (Direct)	1
	Demographic and Health Survey 2022 (Direct)	1
	WHO VR (recalculated - unadjusted) 2025 (VR)	1
Palau	WHO/UNPD VR (recalculated - unadjusted) 2025 (VR)	1 for 10q15, 0 for 10q5

Country	Data series	Inclusion
Papua New Guinea	Demographic and Health Survey 2016-2018 (Direct)	0
	WHO VR (recalculated - unadjusted) 2025 (VR)	0
Poland	WHO/HMD VR (recalculated - unadjusted) 2025 (VR)	1
Democratic People's Republic of Korea	Census 1993 (Household Deaths)	0
	Census 2008 (Household Deaths)	0
Portugal	World Fertility Survey 1979-1980 (Direct)	0
	Census 2011 (Household Deaths)	1
	WHO/HMD VR (recalculated - unadjusted) 2025 (VR)	1
Paraguay	World Fertility Survey 1979 (Direct)	1
	Demographic and Health Survey 1990 (Direct)	1
	National Survey of Demography and Reproductive Health 1995-1996 (Direct)	1
	Census 2002 (Household Deaths)	0 for 10q15, 1 for 10q5
	World Health Survey 2003 (Direct)	0
	Reproductive Health Survey 2004 (Direct)	1
	Encuesta Nacional de Demografia y Salud Sexual y Reproductiva 2008 (Direct)	1
	Multiple Indicator Cluster Survey 2016 (Direct)	1
	WHO VR (recalculated - unadjusted) 2025 (VR)	0 for 10q15, 1 for 10q5
State of Palestine	Demographic Survey 1995 (Direct)	1
	PAPFAM Family Health Survey 2006 (Direct)	1
	Multiple Indicator Cluster Survey-Family Health Survey 2010 (Direct)	1
	Multiple Indicator Cluster Survey 2014 (Direct)	1
	Census 2017 (Household Deaths)	0
	Multiple Indicator Cluster Survey 2019-2020 (Direct)	1
	WHO VR (recalculated - unadjusted) 2025 (VR)	0
Qatar	WHO VR (recalculated - unadjusted) 2025 (VR)	1
Kosovo (UNSCR 1244)	Census 2011 (Household Deaths)	0
	Multiple Indicator Cluster Survey 2013-2014 (Direct)	0
	Multiple Indicator Cluster Survey 2019-2020 (Direct)	0
	WHO VR (recalculated - unadjusted) 2025 (VR)	0
Romania	WHO VR (recalculated - unadjusted) 2025 (VR)	1

Country	Data series	Inclusion
Russian Federation	WHO/HMD VR (recalculated - unadjusted) 2025 (VR)	1
Rwanda	World Fertility Survey 1983 (Direct)	1
	Demographic and Health Survey 1992 (Direct)	1
	Demographic and Health Survey 2000 (Direct)	1
	Census 2002 (Household Deaths)	1 for 10q15, 0 for 10q5
	Demographic and Health Survey 2005 (Direct)	1
	Demographic and Health Survey 2008 (Direct)	1
	Demographic and Health Survey 2010 (Direct)	1
	Census 2012 (Household Deaths)	1
	Demographic and Health Survey 2014-2015 (Direct)	1
	Demographic and Health Survey 2019-2020 (Direct)	1
	Census 2022 (Household Deaths)	1
Saudi Arabia	Demographic Survey 1999 (Household Deaths)	1 for 10q15, 0 for 10q5
	Census 2004 (Household Deaths)	1 for 10q15, 0 for 10q5
	Demographic Survey 2007 (Household Deaths)	1 for 10q15, 0 for 10q5
	Census 2010 (Household Deaths)	1 for 10q15, 0 for 10q5
	Demographic Survey 2016 (Household Deaths)	0
	Household Health Survey 2018 (Household Deaths)	1 for 10q15, 0 for 10q5
	WHO VR (recalculated - unadjusted) 2025 (VR)	0
Sudan	World Fertility Survey 1979 (Direct)	1
	Demographic and Health Survey 1989-1990 (Direct)	1
	Maternal and Child Health Survey 1992 (Direct)	1
	Census 1993 (Household Deaths)	0
	Census 2008 (Household Deaths)	0
	Multiple Indicator Cluster Survey 2010 (Direct)	1
	Multiple Indicator Cluster Survey 2014 (Direct)	1
Senegal	World Fertility Survey 1978 (Direct)	1
	Demographic and Health Survey 1986 (Direct)	1

Country	Data series	Inclusion
	Demographic and Health Survey 1992-1993 (Direct)	1
	Demographic and Health Survey 1997 (Direct)	1
	Demographic and Health Survey 1999-2000 (Direct)	1
	Census 2002 (Household Deaths)	1
	World Health Survey 2003 (Direct)	0
	Demographic and Health Survey 2005 (Direct)	1
	Malaria Indicator Survey 2008-2009 (Direct)	1
	Demographic and Health Survey 2010-2011 (Direct)	1
	Census 2013 (Household Deaths)	1
	Demographic and Health Survey 2012-2013 (Direct)	1
	Demographic and Health Survey 2014 (Direct)	1
	Demographic and Health Survey 2015 (Direct)	1
	Demographic and Health Survey 2016 (Direct)	1
	Demographic and Health Survey 2017 (Direct)	1
	Demographic and Health Survey 2018 (Direct)	1
	Demographic and Health Survey 2019 (Direct)	1
	Demographic and Health Survey 2023 (Direct)	1
Singapore	WHO VR (recalculated - unadjusted) 2025 (VR)	1
Solomon Islands	Census 2019 (Household Deaths)	0
	Census 2009 (Household Deaths)	0
	Demographic and Health Survey 2015 (Direct)	0
	WHO VR (recalculated - unadjusted) 2025 (VR)	0
Sierra Leone	National Survey 1977 (Household Deaths)	1
	Census 2004 (Household Deaths)	1 for 10q15, 0 for 10q5
	Demographic and Health Survey 2008 (Direct)	1
	Demographic and Health Survey 2013 (Direct)	1
	Census 2015 (Household Deaths)	1 for 10q15, 0 for 10q5
	Multiple Indicator Cluster Survey 2017 (Direct)	1
	Demographic and Health Survey 2019 (Direct)	1
	Countrywide Mortality Surveillance for Action (COMSA) 2018-2020 (VR)	0

Country	Data series	Inclusion
El Salvador	Census 1992 (Household Deaths)	1
	National Family Health Survey 1993 (Direct)	1
	National Family Health Survey 1998 (Direct)	1
	National Family Health Survey 2002-2003 (Direct)	1
	Census 2007 (Household Deaths)	0 for 10q15, 1 for 10q5
	National Family Health Survey 2008 (Direct)	1
	Multiple Indicator Cluster Survey 2014 (Direct)	1
	Encuesta Nacional de Salud (ENS) 2021 (Direct)	1
	WHO/UNPD VR (recalculated - unadjusted) 2025 (VR)	1
San Marino	WHO VR (recalculated - unadjusted) 2025 (VR)	0
Somalia	Multiple Indicator Cluster Survey 2006 (Direct)	0
Serbia	WHO VR (recalculated - unadjusted) 2025 (VR)	1
South Sudan	Household Health Survey 2010 (Direct)	0
Sao Tome and Principe	Demographic and Health Survey 2008-2009 (Direct)	1 for 10q15, 0 for 10q5
	Census 2012 (Household Deaths)	1 for 10q15, 0 for 10q5
	Multiple Indicator Cluster Survey 2014 (Direct)	1 for 10q15, 0 for 10q5
	Multiple Indicator Cluster Survey 2019 (Direct)	0
	WHO VR (recalculated - unadjusted) 2025 (VR)	0
Suriname	Multiple Indicator Cluster Survey 2018 (Direct)	1
	WHO/UNPD VR (recalculated - unadjusted) 2025 (VR)	1
Slovakia	WHO/HMD VR (recalculated - unadjusted) 2025 (VR)	1
Slovenia	WHO/HMD VR (recalculated - unadjusted) 2025 (VR)	1
Sweden	WHO/HMD VR (recalculated - unadjusted) 2025 (VR)	1
Eswatini	Census 1997 (Household Deaths)	0 for 10q15, 1 for 10q5
	World Health Survey 2003 (Direct)	0
	Demographic and Health Survey 2006-2007 (Direct)	0 for 10q15, 1 for 10q5
	Census 2007 (Household Deaths)	0
	Multiple Indicator Cluster Survey 2010 (Direct)	1

Country	Data series	Inclusion
	Multiple Indicator Cluster Survey 2014 (Direct)	1
	Multiple Indicator Cluster Survey 2021-2022 (Direct)	1
Seychelles	Census 1960 (Household Deaths)	1
	WHO VR (recalculated - unadjusted) 2025 (VR)	1
Syrian Arab Republic	World Fertility Survey 1978 (Direct)	1
	PAPCHILD Maternal and Child Health Survey 1993 (Household Deaths)	1
	PAPCHILD Maternal and Child Health Survey 1993 (Direct)	1
	PAPFAM Family Health Survey 2001 (Direct)	1
	WHO VR (recalculated - unadjusted) 2025 (VR)	1
Turks and Caicos Islands	Census 2001 (Household Deaths)	0
	Multiple Indicator Cluster Survey 2019-2020 (Direct)	0
	WHO VR (recalculated - unadjusted) 2025 (VR)	0
Chad	Demographic and Health Survey 1996-1997 (Direct)	1
	World Health Survey 2003 (Direct)	0
	Demographic and Health Survey 2004 (Direct)	1
	Census 2009 (Household Deaths)	1
	Demographic and Health Survey 2014-2015 (Direct)	1
	Multiple Indicator Cluster Survey 2019 (Direct)	1
Togo	Demographic survey 1961 (Household Deaths)	1
	Census 1981 (Household Deaths)	1 for 10q15, 0 for 10q5
	Demographic and Health Survey 1988 (Direct)	1
	Demographic and Health Survey 1998 (Direct)	1
	Census 2010 (Household Deaths)	1
	Demographic and Health Survey 2013-2014 (Direct)	1
	Multiple Indicator Cluster Survey 2017 (Direct)	1
Thailand	Demographic and Health Survey 1987 (Direct)	1
	Survey of Population Change 1989 (Household Deaths)	1
	Survey of Population Change 1995-1996 (Household Deaths)	1
	Survey of Population Change 2005-2006 (Household Deaths)	1
	National Life Tables (VR)	0
	Recalculated based on WHO - adjusted for incompleteness 2025 (VR)	1

Country	Data series	Inclusion
	WHO VR (recalculated - unadjusted) 2025 (VR)	0
Tajikistan	Demographic and Health Survey 2012 (Direct)	1
	Demographic and Health Survey 2017 (Direct)	1
	Demographic and Health Survey 2023 (Direct)	1
	WHO VR (recalculated - unadjusted) 2025 (VR)	1
Turkmenistan	Demographic and Health Survey 2000 (Direct)	1
	Multiple Indicator Cluster Survey 2015-2016 (Direct)	1
	Multiple Indicator Cluster Survey 2019 (Direct)	1
	Recalculated based on WHO - adjusted for incompleteness 2025 (VR)	1
	WHO VR (recalculated - unadjusted) 2025 (VR)	0
Timor-Leste	Demographic and Health Survey 1997 (Direct)	1
	Demographic and Health Survey 2009-2010 (Direct)	1 for 10q15, 0 for 10q5
	Census 2015 (Household Deaths)	1 for 10q15, 0 for 10q5
	Demographic and Health Survey 2016 (Direct)	0 for 10q5, 1 for 10q15
Tonga	Census 2006 (Household Deaths)	1
	Multiple Indicator Cluster Survey 2019 (Direct)	1
	WHO VR (recalculated - unadjusted) 2025 (VR)	1
Trinidad and Tobago	World Fertility Survey 1977 (Direct)	1
	Demographic and Health Survey 1987 (Direct)	1
	Multiple Indicator Cluster Survey 2022 (Direct)	1
	WHO/UNPD VR (recalculated - unadjusted) 2025 (VR)	1
Tunisia	National Demographic Survey 1968-1969 (Household Deaths)	0 for 10q15, 1 for 10q5
	World Fertility Survey 1978 (Direct)	0
	Demographic and Health Survey 1988 (Direct)	1
	PAPCHILD Maternal and Child Health Survey 1994 (Direct)	1
	PAPFAM Family Health Survey 2001 (Direct)	1
	World Health Survey 2003 (Direct)	1
	Multiple Indicator Cluster Survey 2011-2012 (Direct)	1
	Multiple Indicator Cluster Survey 2018 (Direct)	1

Country	Data series	Inclusion
	Multiple Indicator Cluster Survey 2023 (Direct)	1
	Recalculated based on WHO - adjusted for incompleteness 2025 (VR)	1
	WHO VR (recalculated - unadjusted) 2025 (VR)	0
Türkiye	World Fertility Survey 1978 (Direct)	1
	Demographic Survey 1989 (Household Deaths)	1
	Demographic and Health Survey 1993 (Direct)	1
	Demographic and Health Survey 1998 (Direct)	1
	National Verbal Autopsy Survey 2003 (Household Deaths)	1
	Demographic and Health Survey 2003-2004 (Direct)	1
	Demographic and Health Survey 2008 (Direct)	1
	Demographic and Health Survey 2013 (Direct)	1
	Demographic and Health Survey 2018 (Direct)	1
	WHO VR (recalculated - unadjusted) 2025 (VR)	1
Tuvalu	Multiple Indicator Cluster Survey 2019-2020 (Direct)	0
	Recalculated based on WHO - adjusted for incompleteness 2025 (VR)	0
	WHO VR (recalculated - unadjusted) 2025 (VR)	0
United Republic of Tanzania	Census 1967 (Household Deaths)	1 for 10q15, 0 for 10q5
	National Demographic Survey 1973 (Household Deaths)	1
	Census 1988 (Household Deaths)	1 for 10q15, 0 for 10q5
	Demographic and Health Survey 1991-1992 (Direct)	1
	Demographic and Health Survey 1996 (Direct)	1
	Demographic and Health Survey 1999 (Direct)	1
	Census 2002 (Household Deaths)	1 for 10q15, 0 for 10q5
	Demographic and Health Survey 2004-2005 (Direct)	1
	AIDS Indicator Survey 2007-2008 (Direct)	1
	Demographic and Health Survey 2010 (Direct)	1
	National Panel Survey 2010-2011 (Household Deaths)	1
	National Panel Survey 2012-2013 (Household Deaths)	1
	Demographic and Health Survey 2015-2016 (Direct)	1

Country	Data series	Inclusion
	Demographic and Health Survey 2022 (Direct)	1 for 10q15, 0 for 10q5
Uganda	Demographic and Health Survey 1988-1989 (Direct)	1
	Demographic and Health Survey 1995 (Direct)	1
	Demographic and Health Survey 2000-2001 (Direct)	1
	Census 2002 (Household Deaths)	1 for 10q15, 0 for 10q5
	Demographic and Health Survey 2006 (Direct)	1
	Demographic and Health Survey 2006 (Household Deaths)	0
	Malaria Indicator Survey 2009-2010 (Direct)	1
	Demographic and Health Survey 2011 (Direct)	1
	Census 2014 (Household Deaths)	1 for 10q15, 0 for 10q5
	Demographic and Health Survey 2016 (Direct)	1
	Census 2024 (Household Deaths)	1
Ukraine	Reproductive Health Survey 1999 (Direct)	1
	World Health Survey 2003 (Direct)	0
	Demographic and Health Survey 2007 (Direct)	1
	Multiple Indicator Cluster Survey 2012 (Direct)	1
	WHO/HMD VR (recalculated - unadjusted) 2025 (VR)	1
Uruguay	World Health Survey 2003 (Direct)	0
	WHO VR (recalculated - unadjusted) 2025 (VR)	1
United States	WHO/HMD VR (recalculated - unadjusted) 2025 (VR)	1
Uzbekistan	Demographic and Health Survey 1996 (Direct)	0
	Demographic and Health Survey 2002 (Direct)	1
	Multiple Indicator Cluster Survey 2021-2022 (Direct)	1
	Recalculated based on WHO - adjusted for incompleteness 2025 (VR)	1
	WHO VR (recalculated - unadjusted) 2025 (VR)	0
Saint Vincent and the Grenadines	WHO VR (recalculated - unadjusted) 2025 (VR)	1
Venezuela (Bolivarian Republic of)	World Fertility Survey 1977 (Direct)	1
	Recalculated based on WHO - adjusted for incompleteness 2025 (VR)	1
	WHO VR (recalculated - unadjusted) 2025 (VR)	0

Country	Data series	Inclusion
Viet Nam	Census 1979 (Household Deaths)	1
	Census 1989 (Household Deaths)	1
	National Demographic and Health Survey 1988 (Direct)	1
	Demographic and Health Survey 1997 (Direct)	1
	Population and Housing Census 1999 (Household Deaths)	1
	Demographic and Health Survey 2002 (Direct)	1
	World Health Survey 2003 (Direct)	1
	Population Change and Family Planning Survey 2007 (Household Deaths)	1
	Population Change and Family Planning Survey 2008 (Household Deaths)	1
	Census 2009 (Household Deaths)	0
	National Sample Mortality Surveillance Programme 2009 (Household Deaths)	0
	Population Change and Family Planning Survey 2011 (Household Deaths)	1
	Population Change and Family Planning Survey 2012 (Household Deaths)	1
	Population Change and Family Planning Survey 2013 (Household Deaths)	1
	Intercensal Population and Housing Survey 2014 (Household Deaths)	1
	Multiple Indicator Cluster Survey 2013-2014 (Direct)	1
	Population Change and Family Planning Survey 2015 (Household Deaths)	1
	Population Change and Family Planning Survey 2017 (Household Deaths)	1
	Population Change and Family Planning Survey 2018 (Household Deaths)	1
	Population Change and Family Planning Survey 2020 (Household Deaths)	1
	Population Change and Family Planning Survey 2021 (Direct)	1
Vanuatu	Census 2009 (Household Deaths)	0
	Multiple Indicator Cluster Survey 2023 (Direct)	0
Samoa	Demographic and Health Survey 1999 (Household Deaths)	1
	Demographic and Vital Statistics Survey 2000 (Household Deaths)	1
	Population and Housing Census 2006 (Household Deaths)	1
	Population and Housing Census 2011 (Household Deaths)	1
	Population and Housing Census 2016 (Household Deaths)	1
	Multiple Indicator Cluster Survey 2019-2020 (Direct)	1
Yemen	World Fertility Survey 1979 (Direct)	1
	Demographic and Health Survey 1991-1992 (Direct)	1

Country	Data series	Inclusion
	Demographic and Health Survey 1997 (Direct)	1
	PAPFAM Family Health Survey 2003 (Direct)	1
	Multiple Indicator Cluster Survey 2006 (Direct)	1
	Demographic and Health Survey 2013 (Direct)	1
	Multiple Indicator Cluster Survey 2022-2023 (Direct)	1
South Africa	Household Survey 1993 (Household Deaths)	0
	Household Survey 1995 (Household Deaths)	0
	Household Survey 1996 (Household Deaths)	0
	Household Survey 1997 (Household Deaths)	0
	Household Survey 1998 (Household Deaths)	0
	Demographic and Health Survey 1998 (Direct)	1
	Census 2001 (Household Deaths)	1
	World Health Survey 2003 (Direct)	1
	Community Survey 2007 (Household Deaths)	0
	Census 2011 (Household Deaths)	1
	Demographic and Health Survey 2016 (Direct)	1
	Rapid Mortality Surveillance 2024 (VR)	1
	WHO VR (recalculated - unadjusted) 2025 (VR)	0
Zambia	Demographic and Health Survey 1992 (Direct)	1
	Demographic and Health Survey 1996-1997 (Direct)	1
	Demographic and Health Survey 2001-2002 (Direct)	1
	World Health Survey 2003 (Direct)	1
	Demographic and Health Survey 2007 (Direct)	1
	Demographic and Health Survey 2007 (Household Deaths)	0
	Global Fund Evaluation Survey 2008 (Household Deaths)	0
	Census 2010 (Household Deaths)	1 for 10q15, 0 for 10q5
	Demographic and Health Survey 2013-2014 (Direct)	1
	Demographic and Health Survey 2018 (Direct)	1
	Demographic and Health Survey 2024 (Direct)	1
Zimbabwe	Census 2022 (Household Deaths)	1
	Demographic and Health Survey 1988-1989 (Direct)	1

Country	Data series	Inclusion
	Census 1992 (Household Deaths)	1
	Demographic and Health Survey 1994 (Direct)	1
	Inter-censal Demographic Survey 1997 (Household Deaths)	1 for 10q15, 0 for 10q5
	Demographic and Health Survey 1999 (Direct)	1
	Census 2002 (Household Deaths)	1 for 10q15, 0 for 10q5
	World Health Survey 2003 (Direct)	1
	Demographic and Health Survey 2005-2006 (Direct)	1 for 10q15, 0 for 10q5
	Multiple Indicator Cluster Survey 2009 (Direct)	1
	Demographic and Health Survey 2010-2011 (Direct)	1
	Census 2012 (Household Deaths)	1
	Multiple Indicator Cluster Survey 2014 (Direct)	1
	Demographic and Health Survey 2015 (Direct)	1
	Inter-censal Demographic Survey 2017 (Household Deaths)	1
	Multiple Indicator Cluster Survey 2019 (Direct)	1
	WHO VR (recalculated - unadjusted) 2025 (VR)	0

Supplementary figures

Country-level probability of dying with underlying data

Probability of dying by age group (${}_{5}q_{5}$, ${}_{5}q_{10}$, ${}_{10}q_{5}$, ${}_{5}q_{15}$, ${}_{5}q_{20}$, ${}_{10}q_{15}$) with underlying data sources, by country.

Afghanistan (AFG)

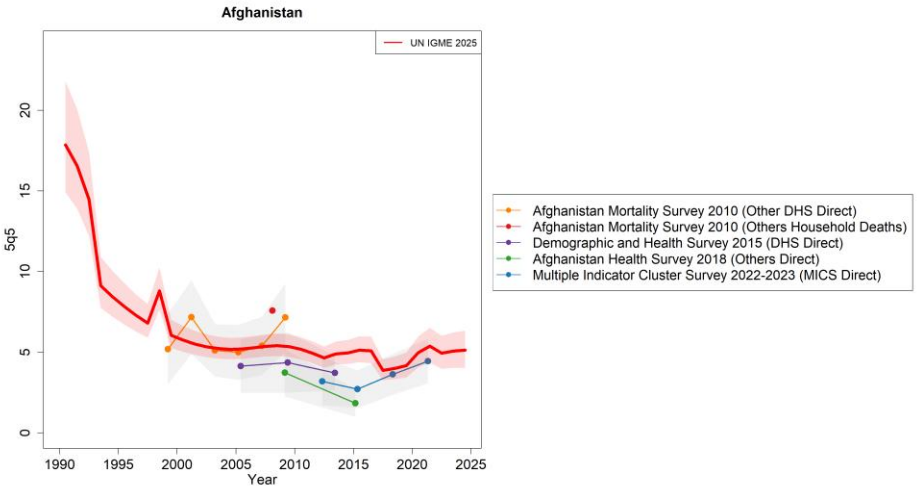

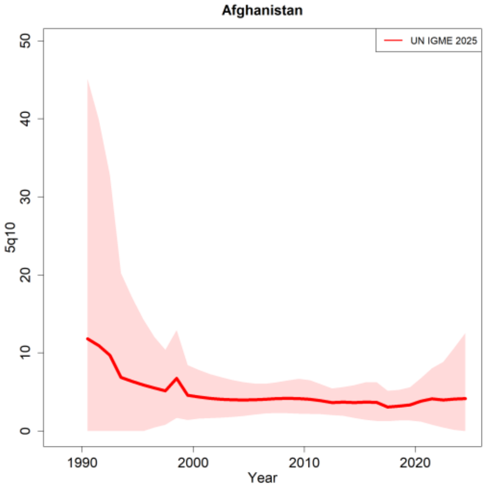

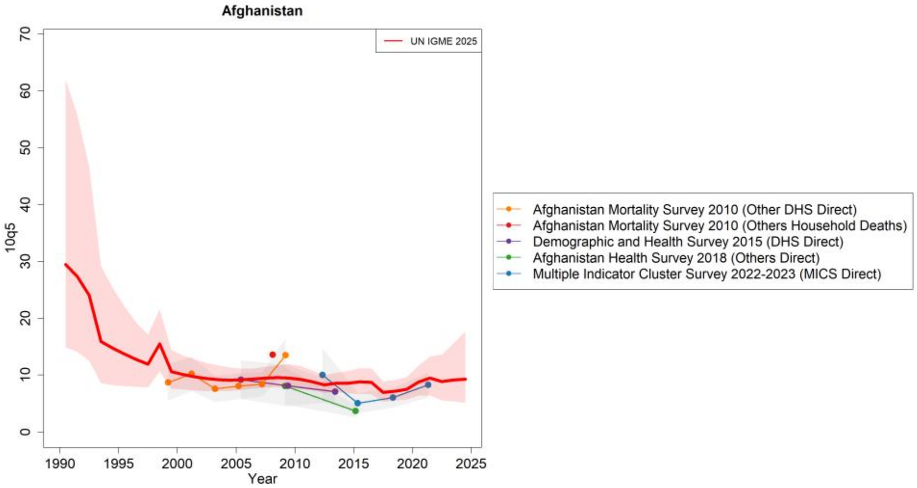

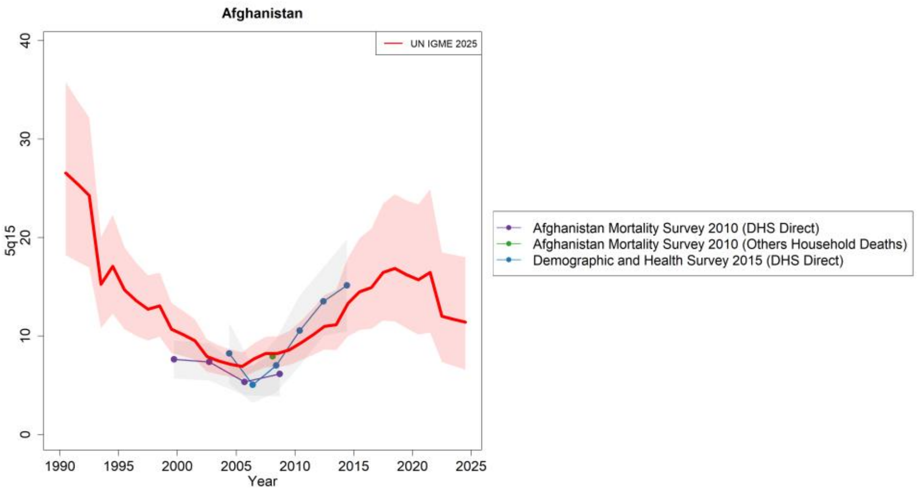
Afghanistan
UN IGME 2025
5q15
Year
Afghanistan Mortality Survey 2010 (DHS Direct)
Afghanistan Mortality Survey 2010 (Others Household Deaths)
Demographic and Health Survey 2015 (DHS Direct)

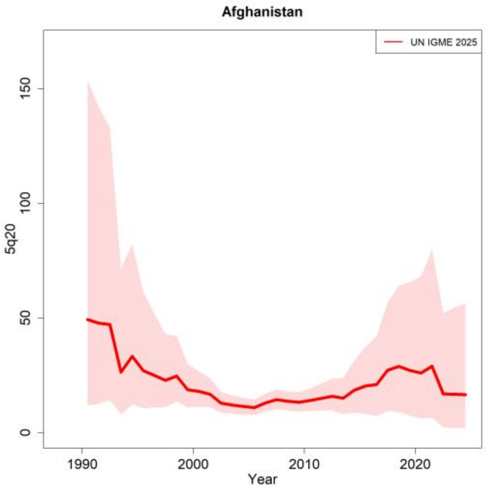
Afghanistan
UN IGME 2025
5q20
Year

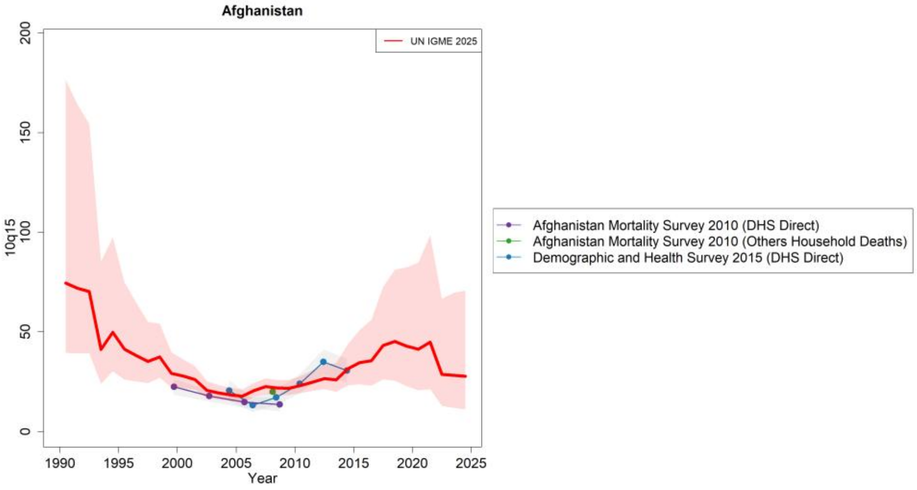
Afghanistan
UN IGME 2025
10q15
Year
Afghanistan Mortality Survey 2010 (DHS Direct)
Afghanistan Mortality Survey 2010 (Others Household Deaths)
Demographic and Health Survey 2015 (DHS Direct)

Albania (ALB)

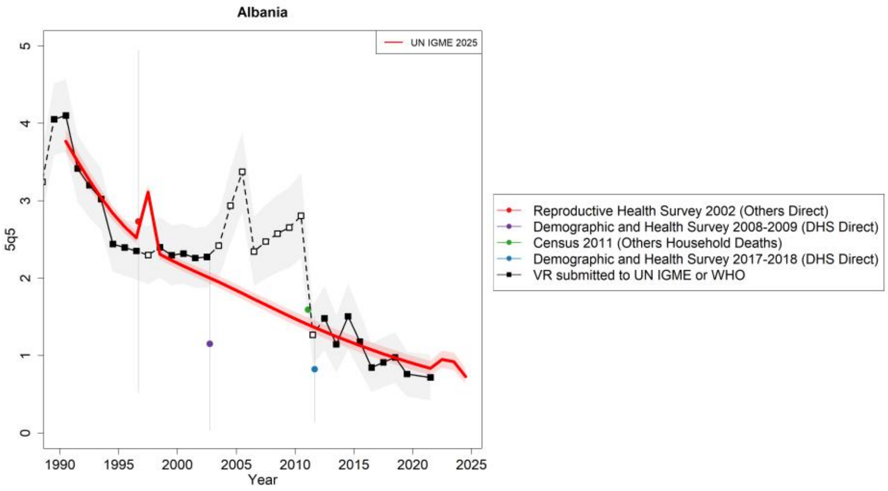
Albania
UN IGME 2025
5q5
Year
0
1
2
3
4
5
1990
1995
2000
2005
2010
2015
2020
2025
Reproductive Health Survey 2002 (Others Direct)
Demographic and Health Survey 2008-2009 (DHS Direct)
Census 2011 (Others Household Deaths)
Demographic and Health Survey 2017-2018 (DHS Direct)
VR submitted to UN IGME or WHO

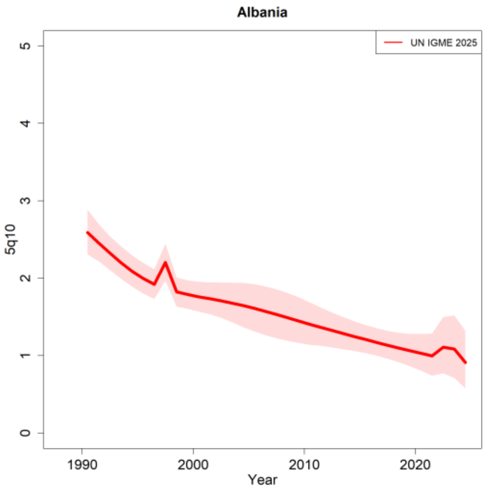
Albania
UN IGME 2025
5q10
Year
0
1
2
3
4
5
1990
2000
2010
2020

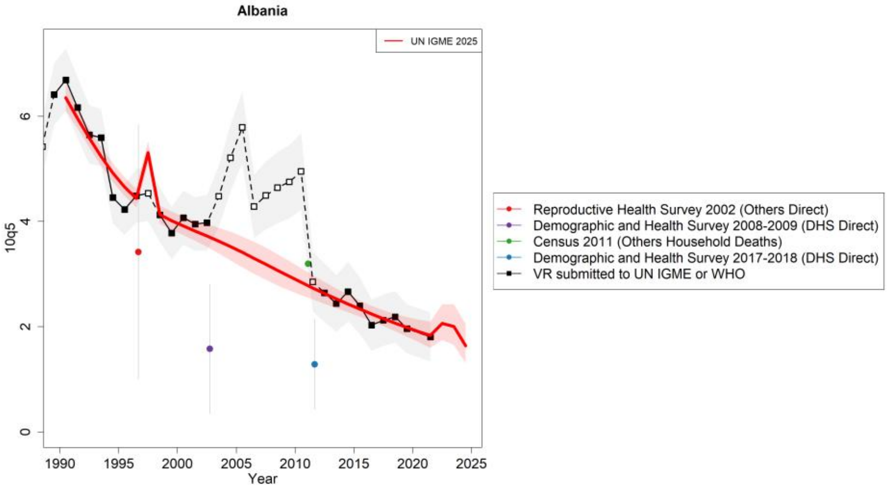
Albania
UN IGME 2025
10q5
Year
0
2
4
6
1990
1995
2000
2005
2010
2015
2020
2025
Reproductive Health Survey 2002 (Others Direct)
Demographic and Health Survey 2008-2009 (DHS Direct)
Census 2011 (Others Household Deaths)
Demographic and Health Survey 2017-2018 (DHS Direct)
VR submitted to UN IGME or WHO

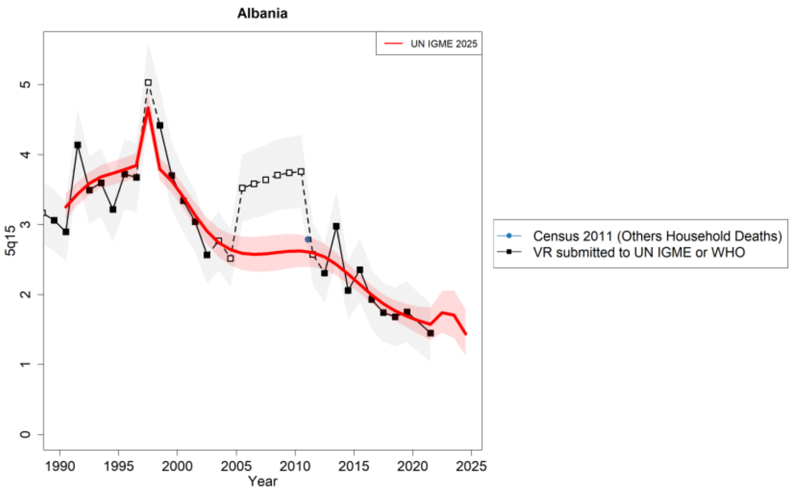
Albania
UN IGME 2025
5q15
Year
Census 2011 (Others Household Deaths)
VR submitted to UN IGME or WHO

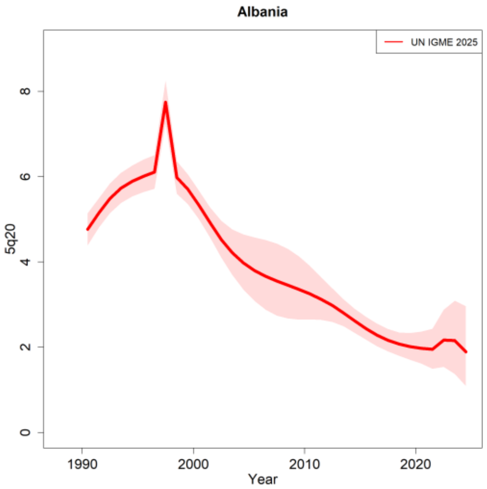
Albania
UN IGME 2025
5q20
Year

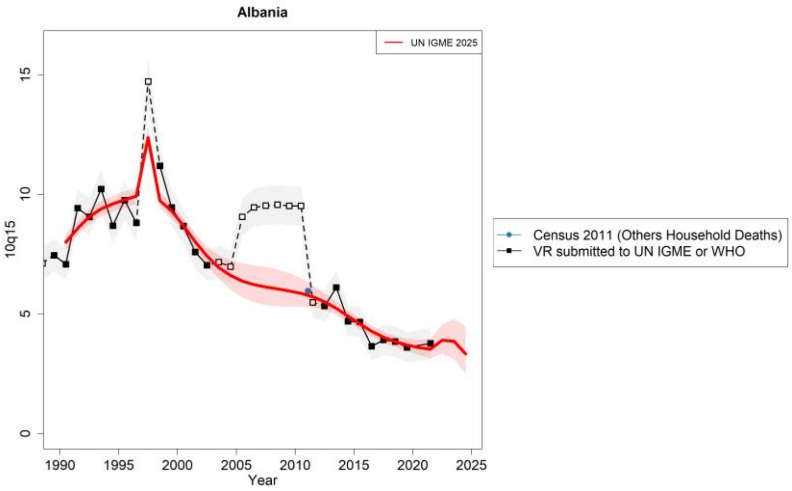
Albania
UN IGME 2025
10q15
Year
Census 2011 (Others Household Deaths)
VR submitted to UN IGME or WHO

Algeria (DZA)

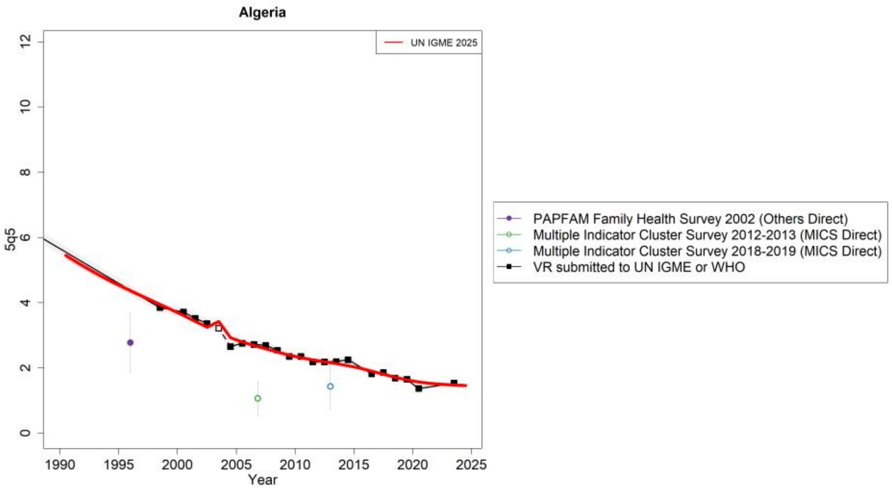
Algeria
UN IGME 2025
5q5
0
2
4
6
8
10
12
1990
1995
2000
2005
2010
2015
2020
2025
Year
PAPFAM Family Health Survey 2002 (Others Direct)
Multiple Indicator Cluster Survey 2012-2013 (MICS Direct)
Multiple Indicator Cluster Survey 2018-2019 (MICS Direct)
VR submitted to UN IGME or WHO

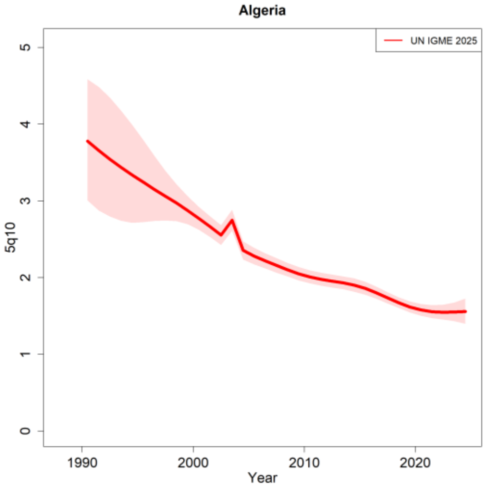
Algeria
UN IGME 2025
5q10
0
1
2
3
4
5
1990
2000
2010
2020
Year

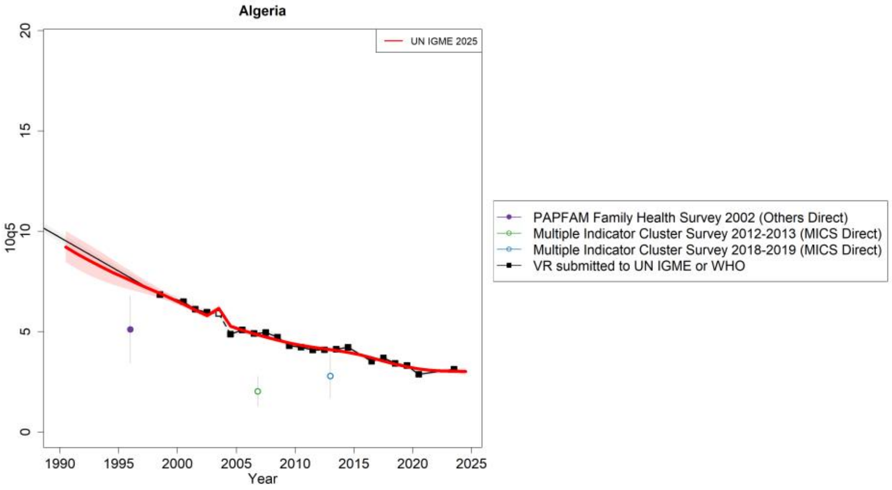
Algeria
UN IGME 2025
10q5
0
5
10
15
20
1990
1995
2000
2005
2010
2015
2020
2025
Year
PAPFAM Family Health Survey 2002 (Others Direct)
Multiple Indicator Cluster Survey 2012-2013 (MICS Direct)
Multiple Indicator Cluster Survey 2018-2019 (MICS Direct)
VR submitted to UN IGME or WHO

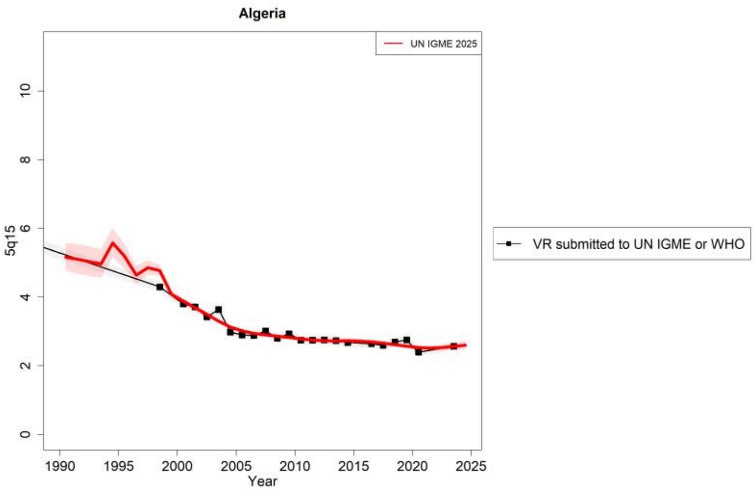
Algeria
UN IGME 2025
VR submitted to UN IGME or WHO
5q15
Year

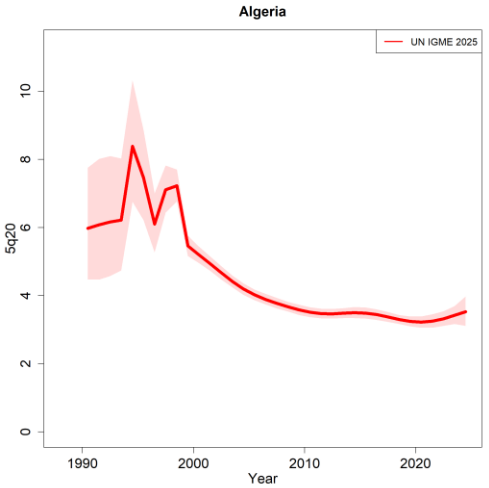
Algeria
UN IGME 2025
5q20
Year

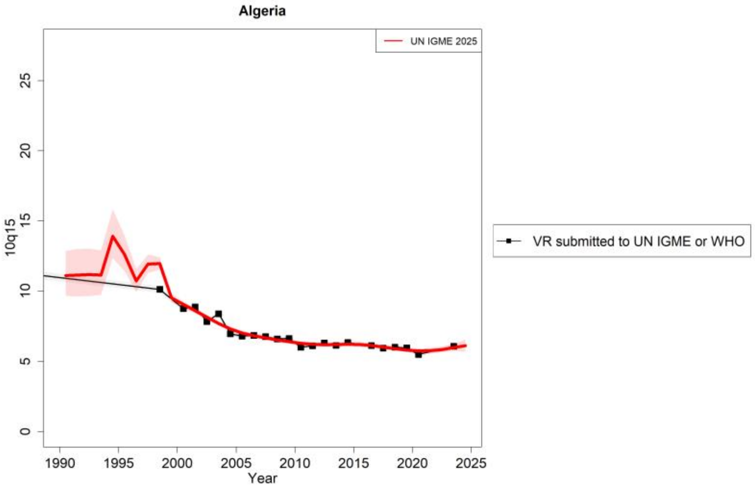
Algeria
UN IGME 2025
VR submitted to UN IGME or WHO
10q15
Year

Andorra (AND)

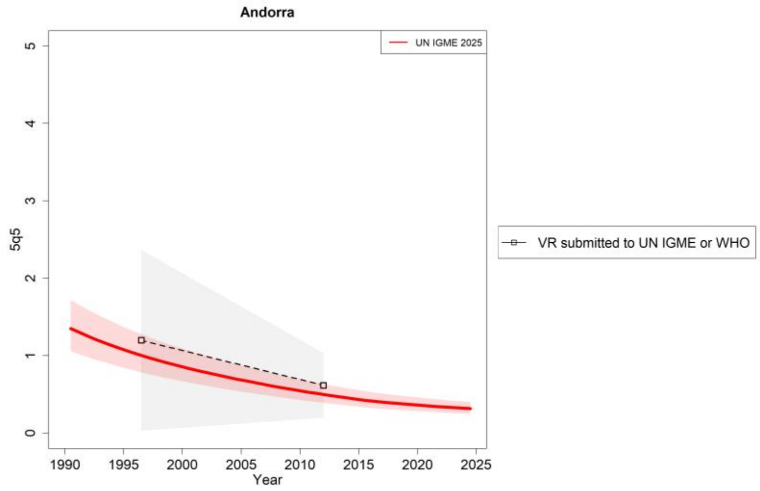
Andorra
UN IGME 2025
VR submitted to UN IGME or WHO
5q5
Year

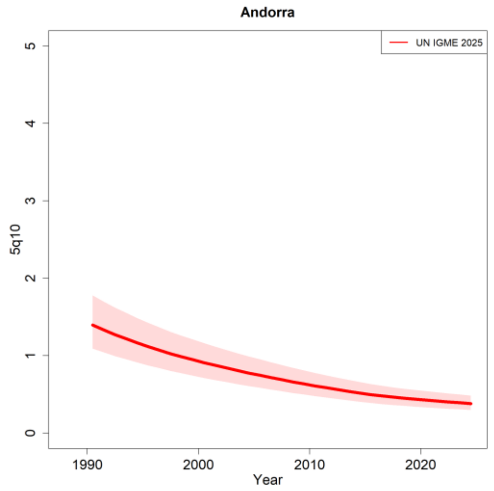
Andorra
UN IGME 2025
5q10
Year

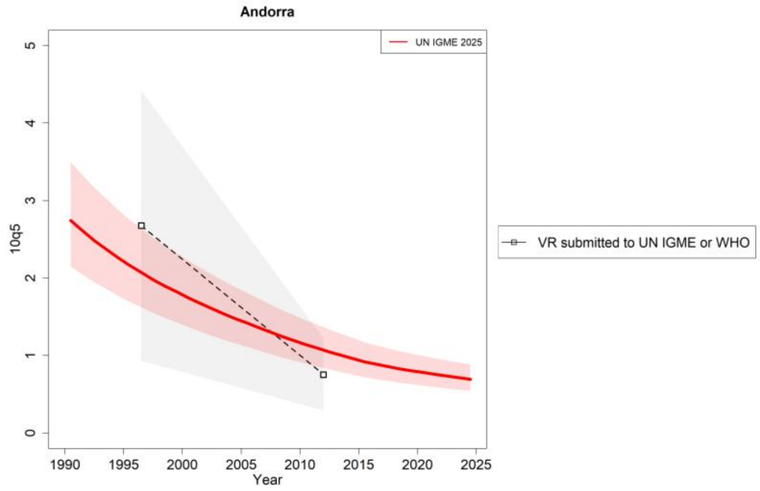
Andorra
UN IGME 2025
VR submitted to UN IGME or WHO
10q5
Year

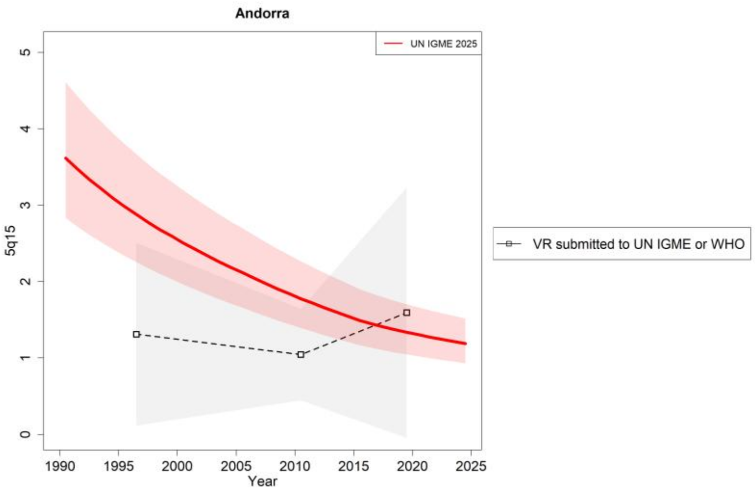
Andorra
UN IGME 2025
VR submitted to UN IGME or WHO
5q15
0
1
2
3
4
5
1990
1995
2000
2005
2010
2015
2020
2025
Year

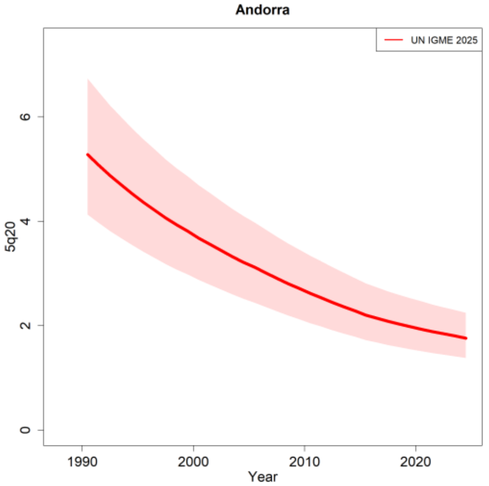
Andorra
UN IGME 2025
5q20
0
2
4
6
1990
2000
2010
2020
Year

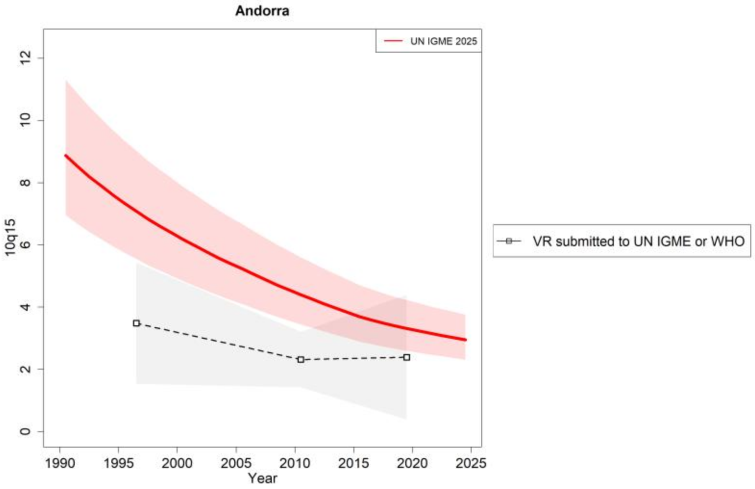
Andorra
UN IGME 2025
VR submitted to UN IGME or WHO
10q15
0
2
4
6
8
10
12
1990
1995
2000
2005
2010
2015
2020
2025
Year

Angola (AGO)

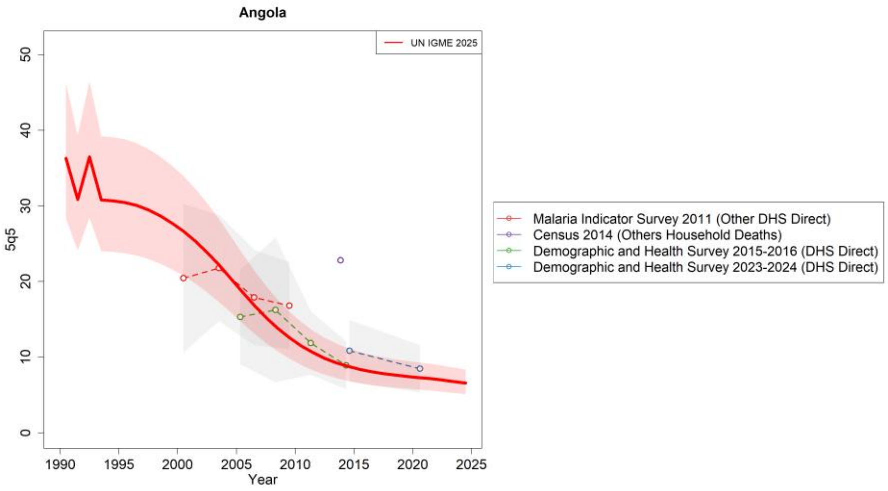
Angola
UN IGME 2025
5q5
Year
Malaria Indicator Survey 2011 (Other DHS Direct)
Census 2014 (Others Household Deaths)
Demographic and Health Survey 2015-2016 (DHS Direct)
Demographic and Health Survey 2023-2024 (DHS Direct)

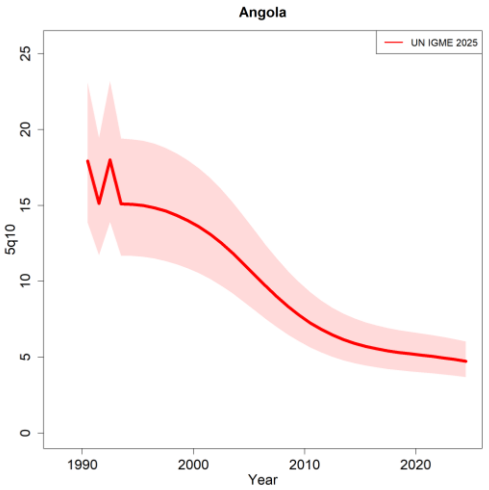
Angola
UN IGME 2025
5q10
Year

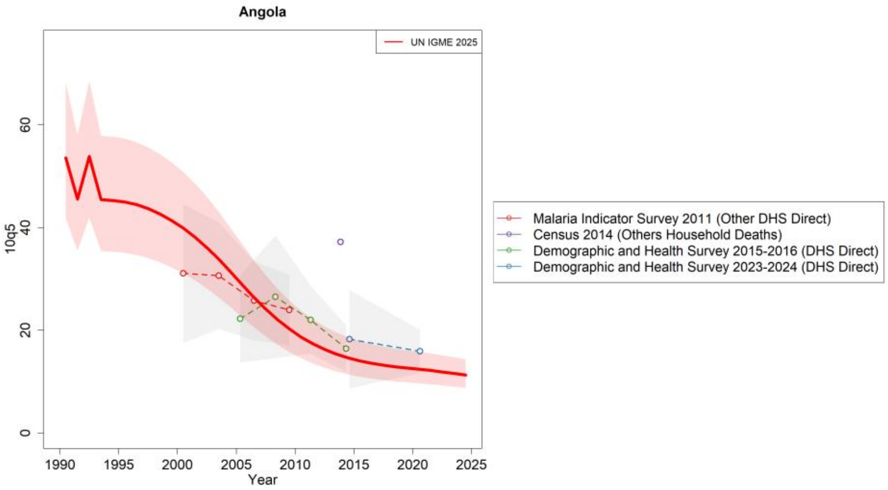
Angola
UN IGME 2025
10q5
Year
Malaria Indicator Survey 2011 (Other DHS Direct)
Census 2014 (Others Household Deaths)
Demographic and Health Survey 2015-2016 (DHS Direct)
Demographic and Health Survey 2023-2024 (DHS Direct)

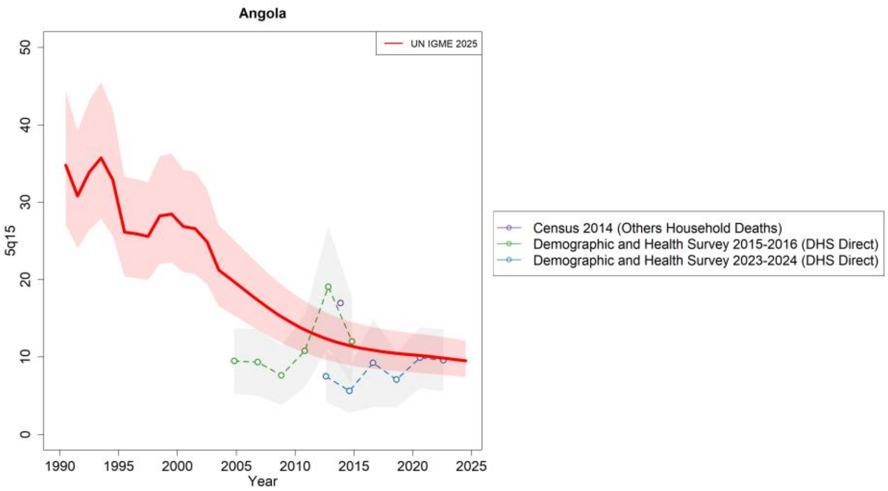
Angola
UN IGME 2025
5q15
Year
Census 2014 (Others Household Deaths)
Demographic and Health Survey 2015-2016 (DHS Direct)
Demographic and Health Survey 2023-2024 (DHS Direct)

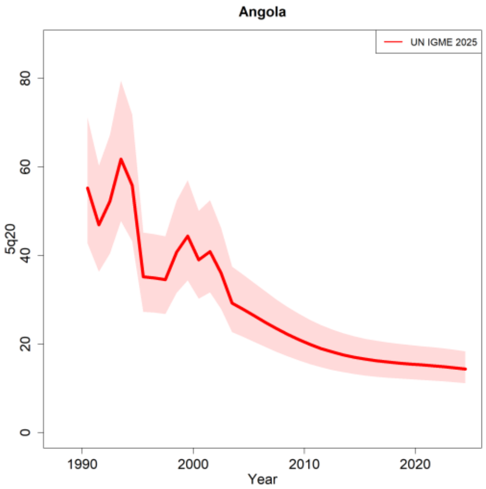
Angola
UN IGME 2025
5q20
Year

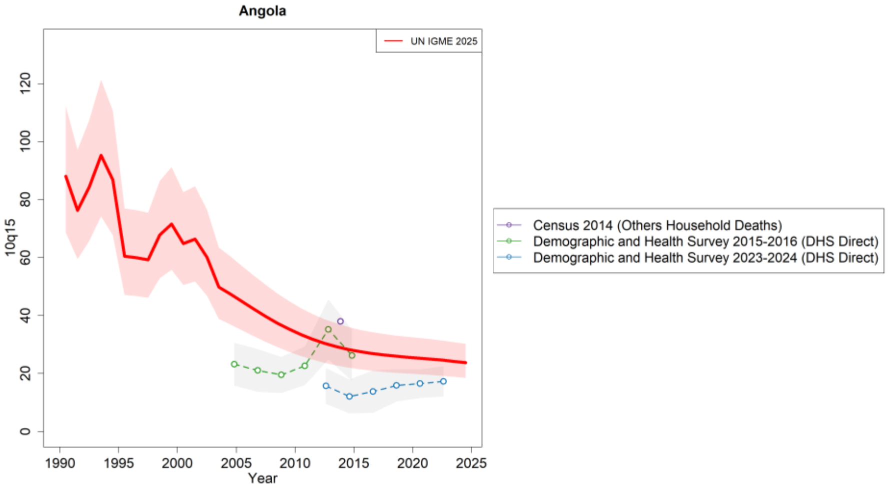
Angola
UN IGME 2025
10q15
Year
Census 2014 (Others Household Deaths)
Demographic and Health Survey 2015-2016 (DHS Direct)
Demographic and Health Survey 2023-2024 (DHS Direct)

Anguilla (AIA)

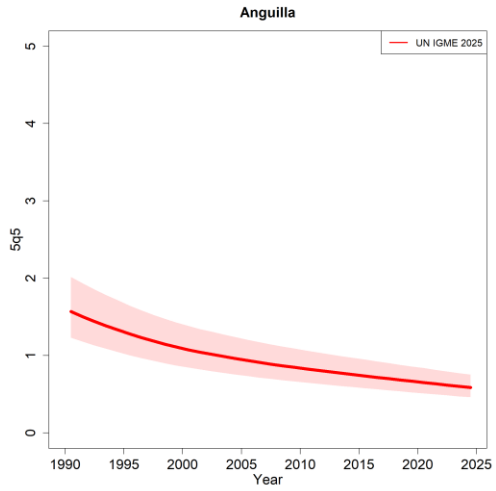

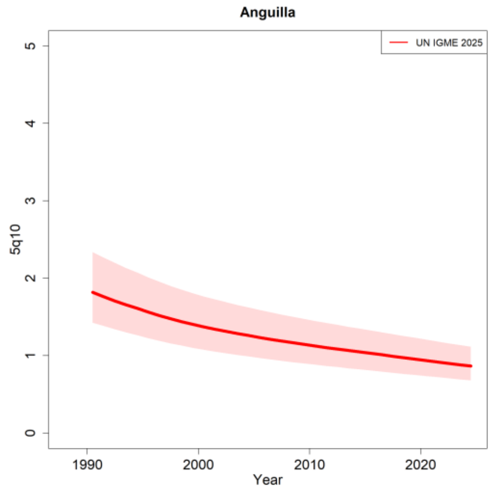

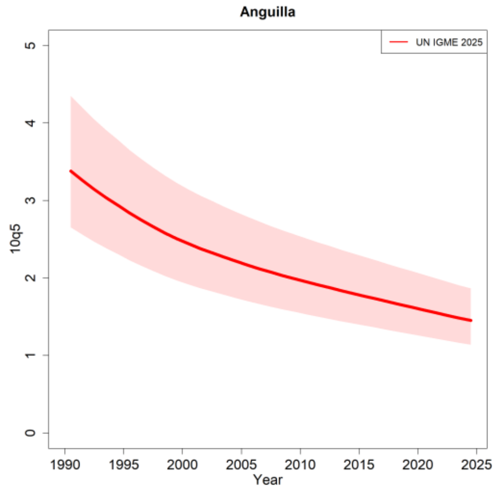

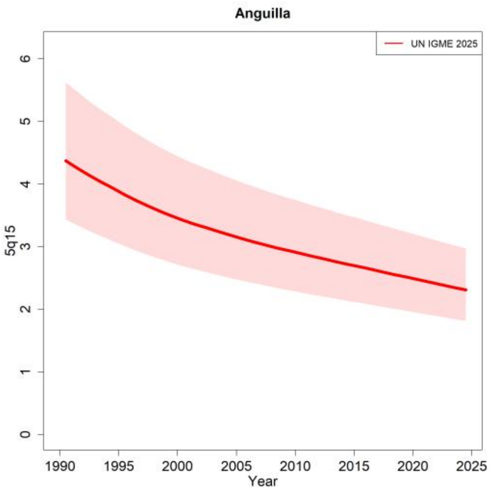
Anguilla
UN IGME 2025
5q15
Year

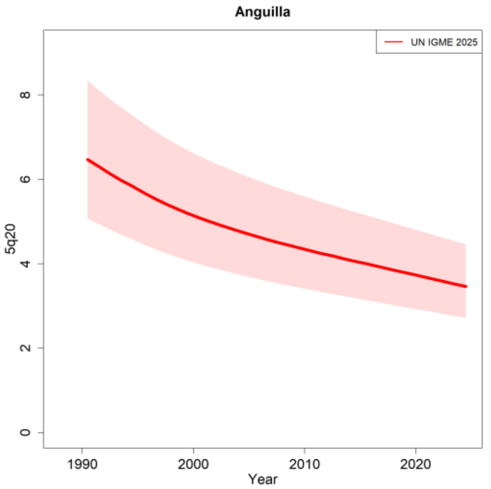
Anguilla
UN IGME 2025
5q20
Year

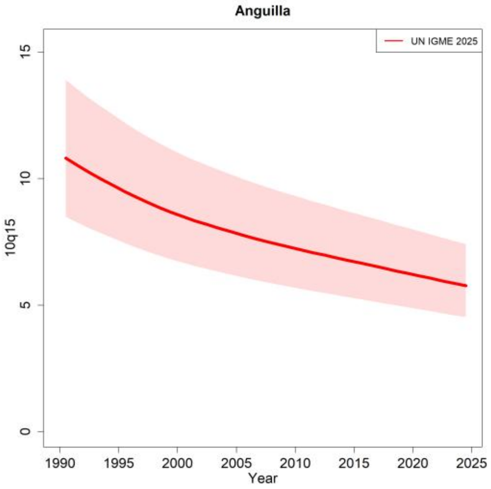
Anguilla
UN IGME 2025
10q15
Year

Antigua and Barbuda (ATG)

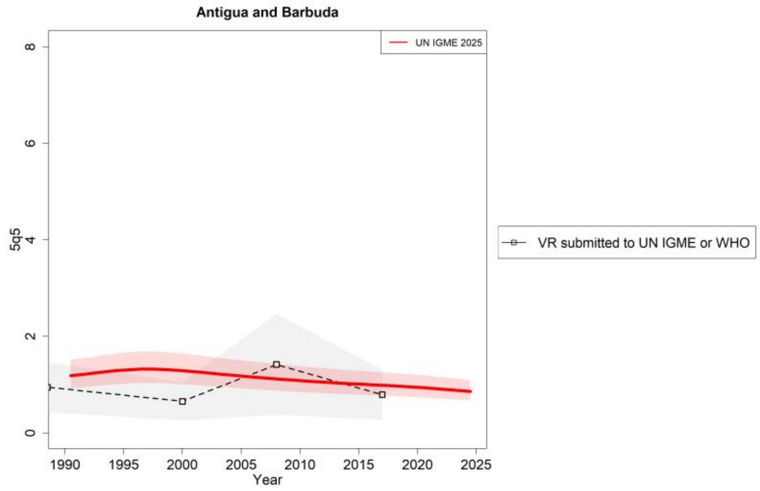

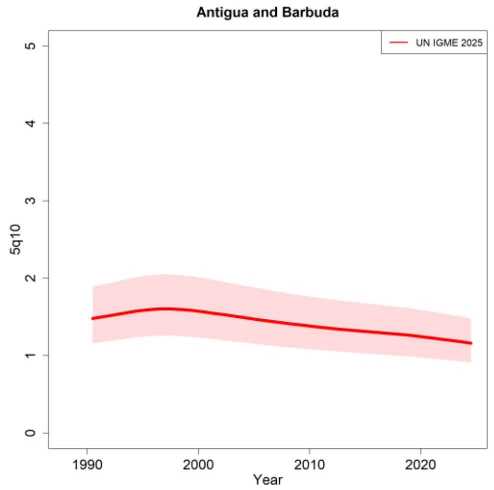

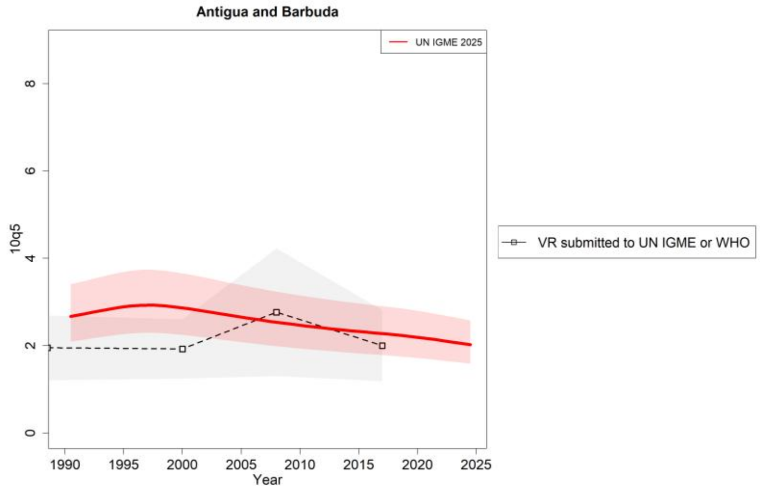

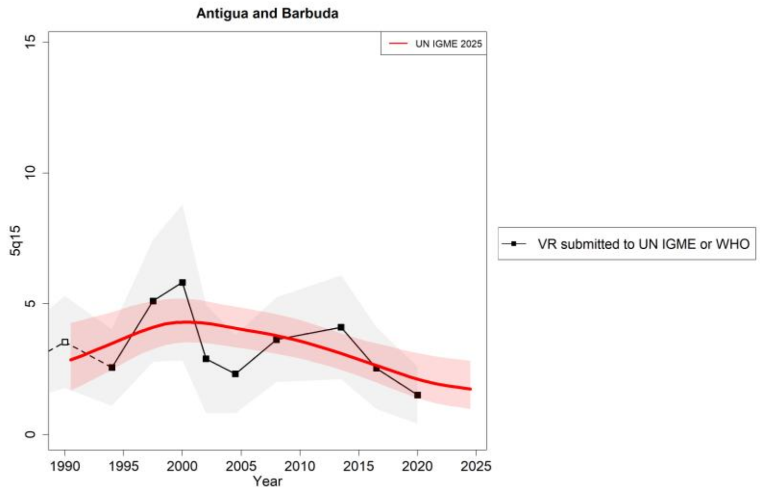
Antigua and Barbuda
UN IGME 2025
VR submitted to UN IGME or WHO
5q15
Year

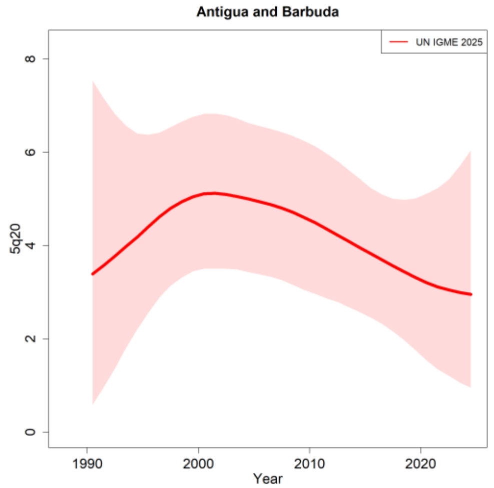
Antigua and Barbuda
UN IGME 2025
5q20
Year

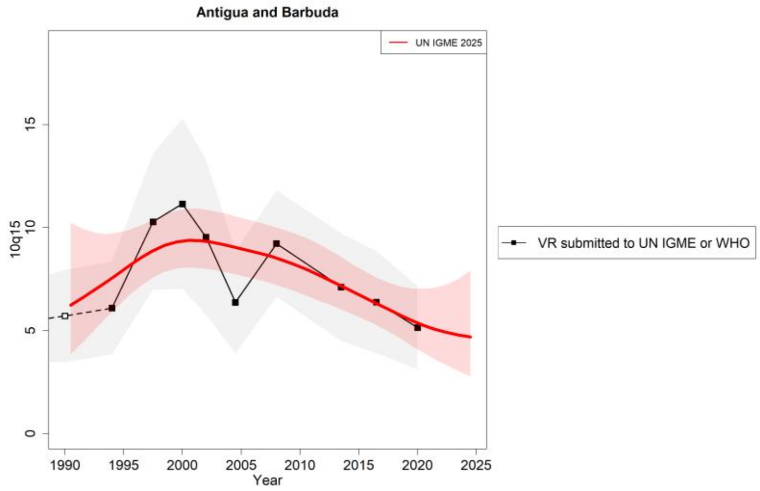
Antigua and Barbuda
UN IGME 2025
VR submitted to UN IGME or WHO
10q15
Year

Argentina (ARG)

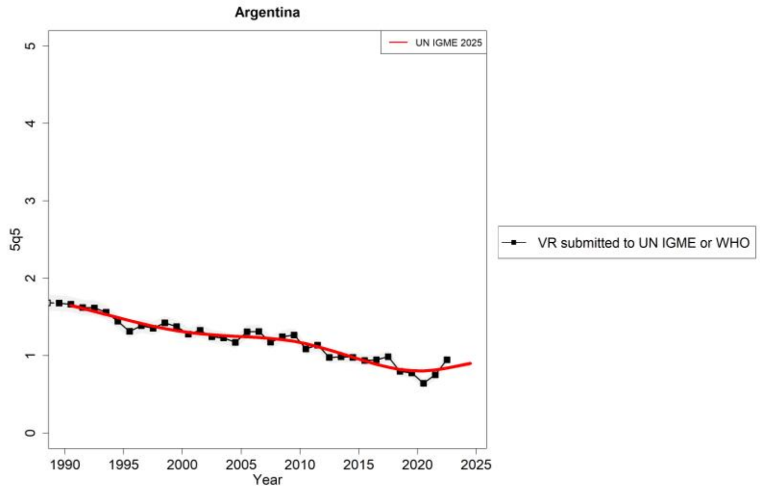

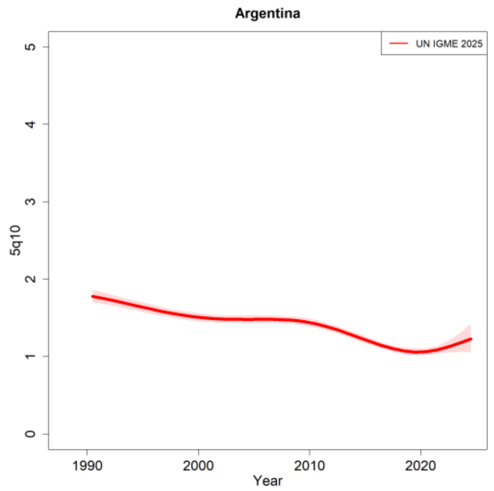

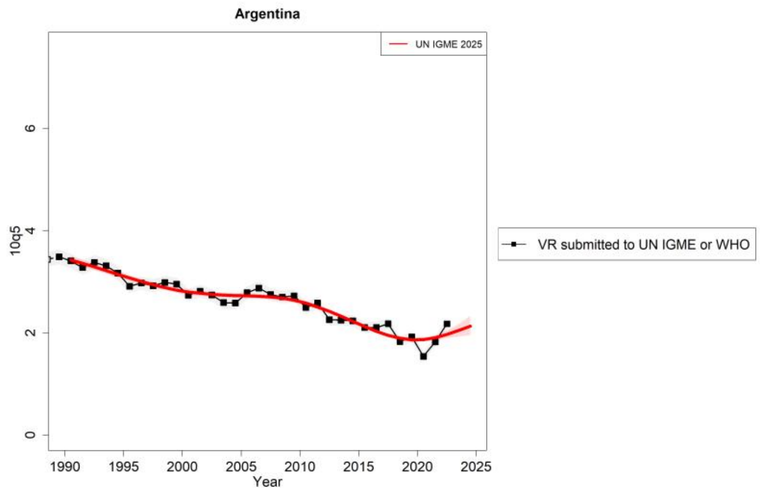

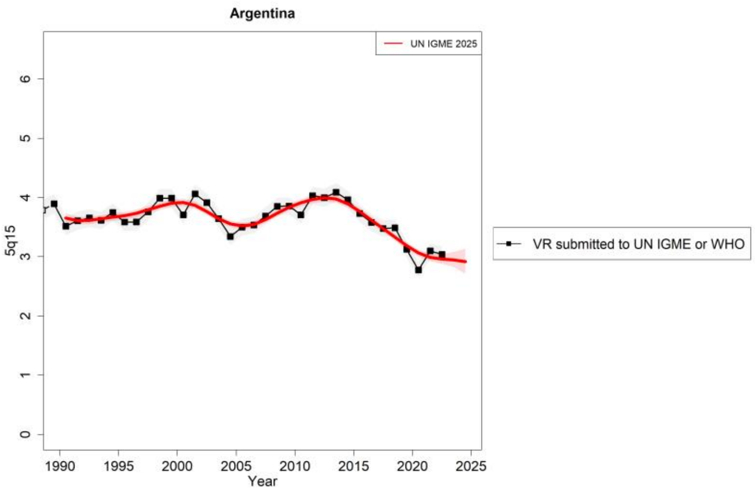
Argentina
UN IGME 2025
VR submitted to UN IGME or WHO
5q15
0
1
2
3
4
5
6
1990
1995
2000
2005
2010
2015
2020
2025
Year

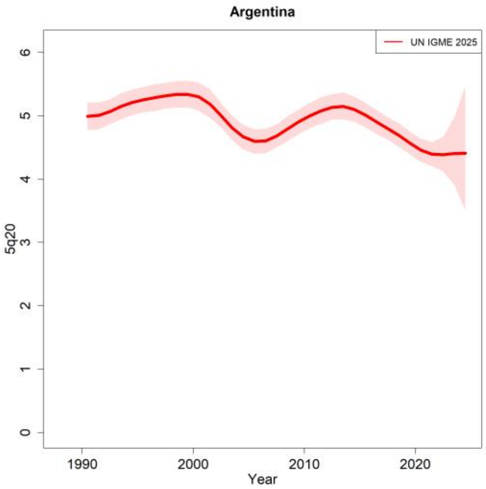
Argentina
UN IGME 2025
5q20
0
1
2
3
4
5
6
1990
2000
2010
2020
Year

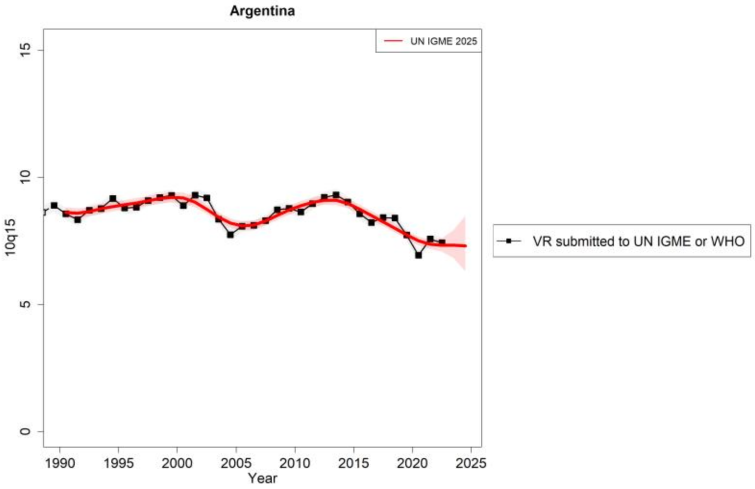
Argentina
UN IGME 2025
VR submitted to UN IGME or WHO
10q15
0
5
10
15
1990
1995
2000
2005
2010
2015
2020
2025
Year

Armenia (ARM)

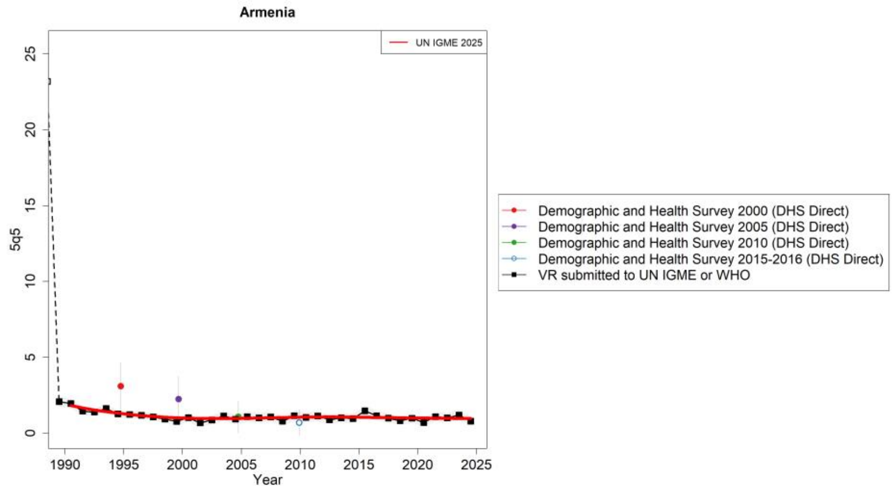
Armenia
UN IGME 2025
5q5
Year
Demographic and Health Survey 2000 (DHS Direct)
Demographic and Health Survey 2005 (DHS Direct)
Demographic and Health Survey 2010 (DHS Direct)
Demographic and Health Survey 2015-2016 (DHS Direct)
VR submitted to UN IGME or WHO

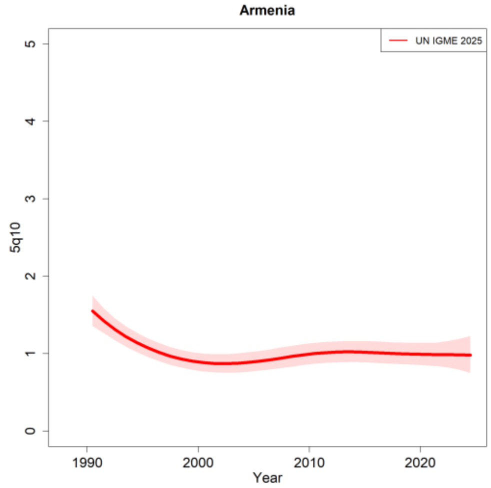
Armenia
UN IGME 2025
5q10
Year

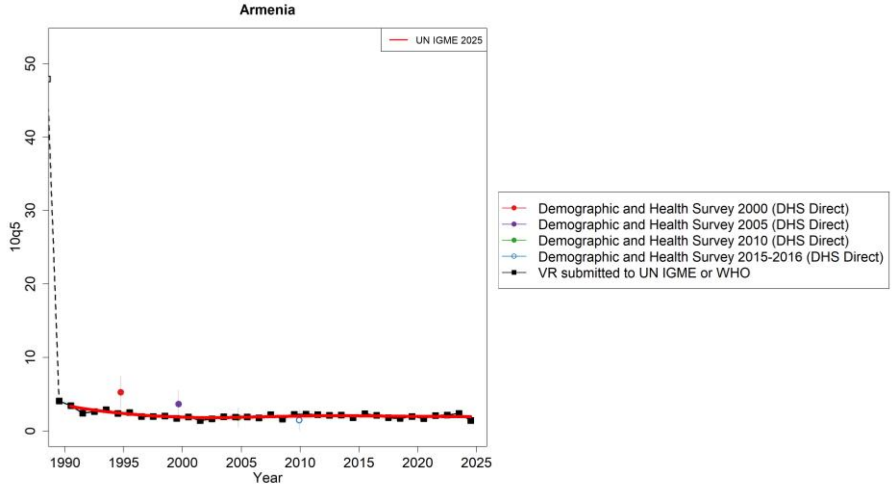
Armenia
UN IGME 2025
10q5
Year
Demographic and Health Survey 2000 (DHS Direct)
Demographic and Health Survey 2005 (DHS Direct)
Demographic and Health Survey 2010 (DHS Direct)
Demographic and Health Survey 2015-2016 (DHS Direct)
VR submitted to UN IGME or WHO

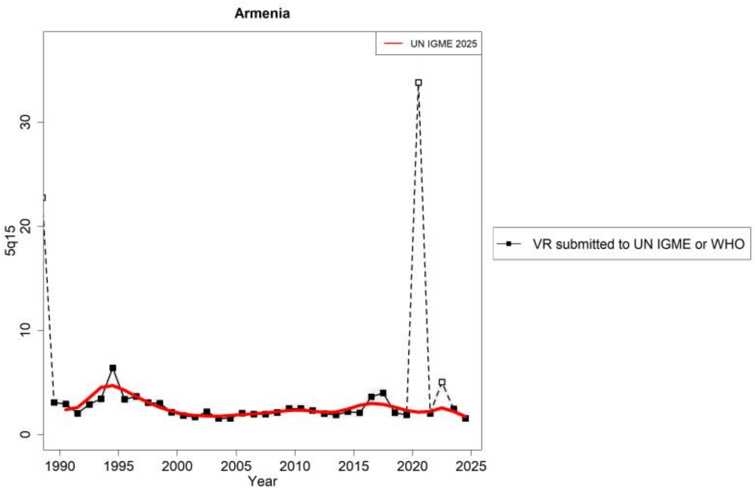
Armenia
UN IGME 2025
VR submitted to UN IGME or WHO
5q15
0
10
20
30
1990
1995
2000
2005
2010
2015
2020
2025
Year

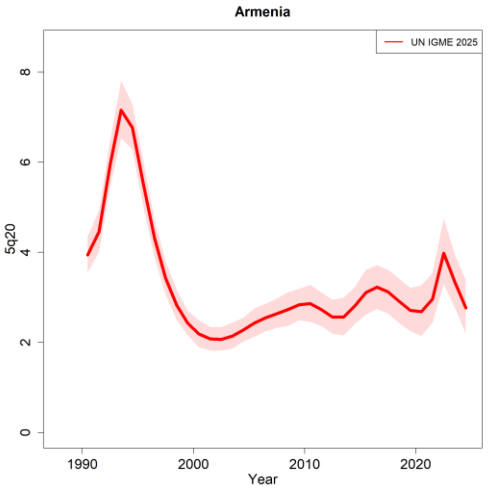
Armenia
UN IGME 2025
5q20
0
2
4
6
8
1990
2000
2010
2020
Year

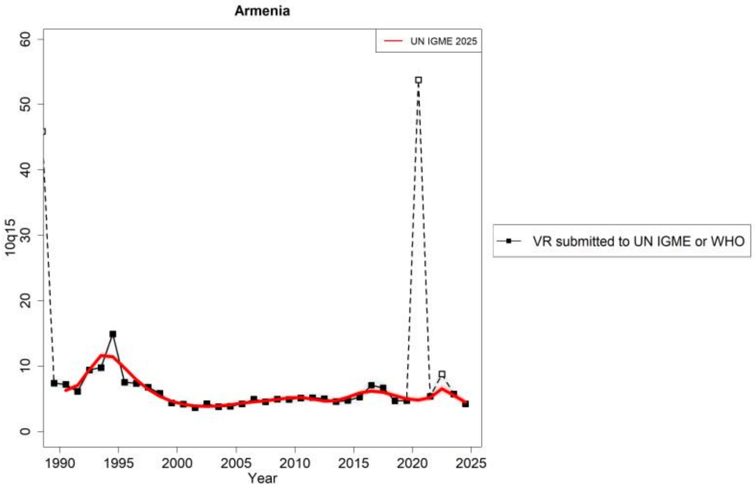
Armenia
UN IGME 2025
VR submitted to UN IGME or WHO
10q15
0
10
20
30
40
50
60
1990
1995
2000
2005
2010
2015
2020
2025
Year

Australia (AUS)

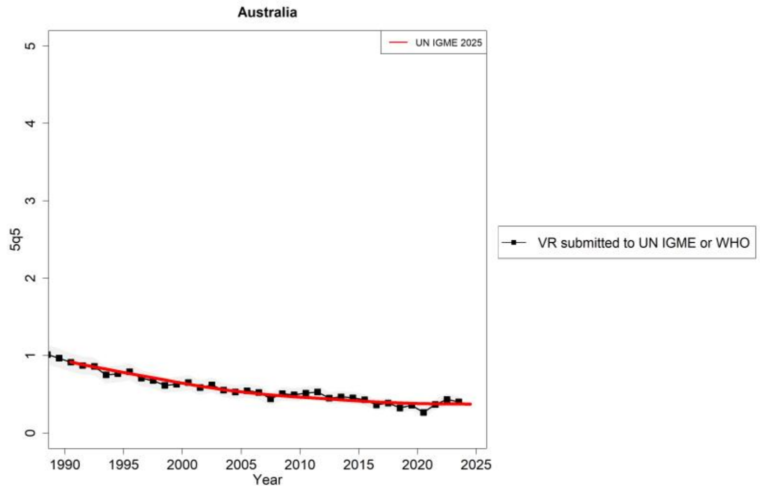

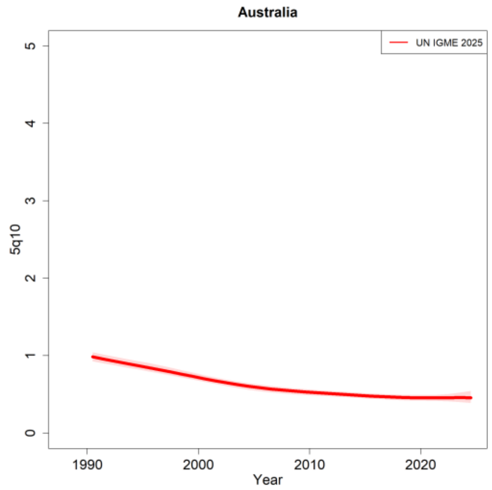

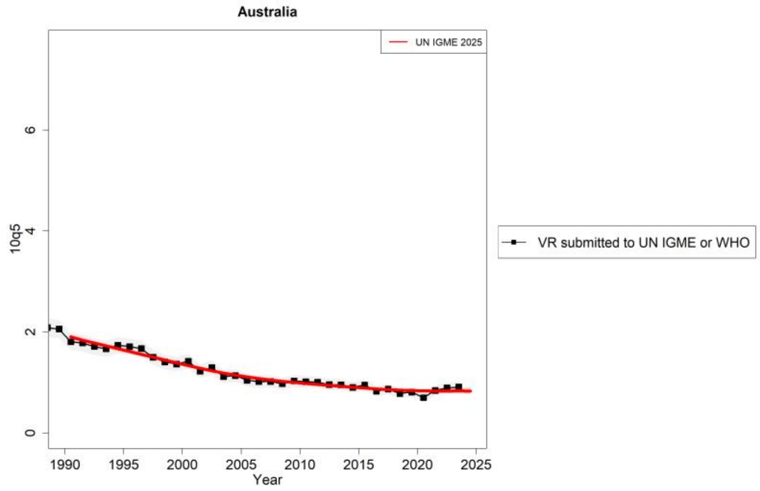

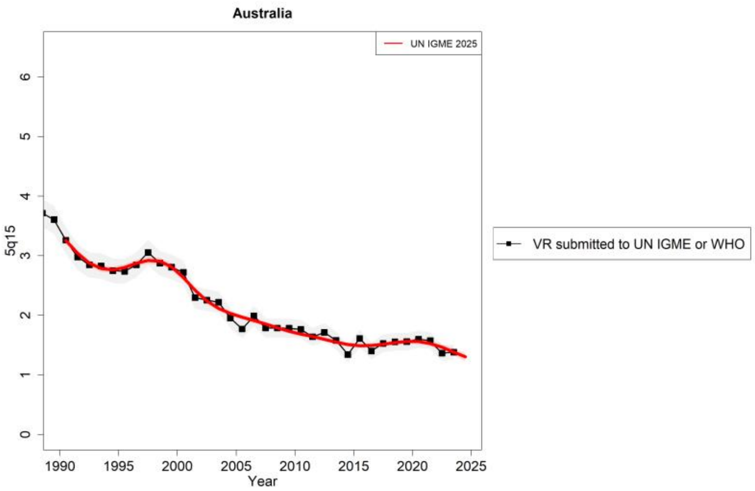
Australia
UN IGME 2025
5q15
0
1
2
3
4
5
6
1990
1995
2000
2005
2010
2015
2020
2025
Year
VR submitted to UN IGME or WHO

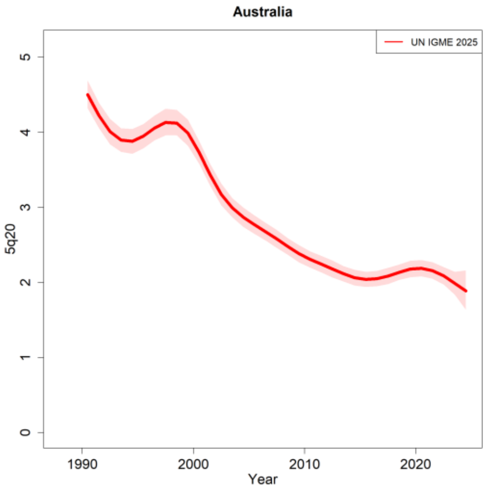
Australia
UN IGME 2025
5q20
0
1
2
3
4
5
1990
2000
2010
2020
Year

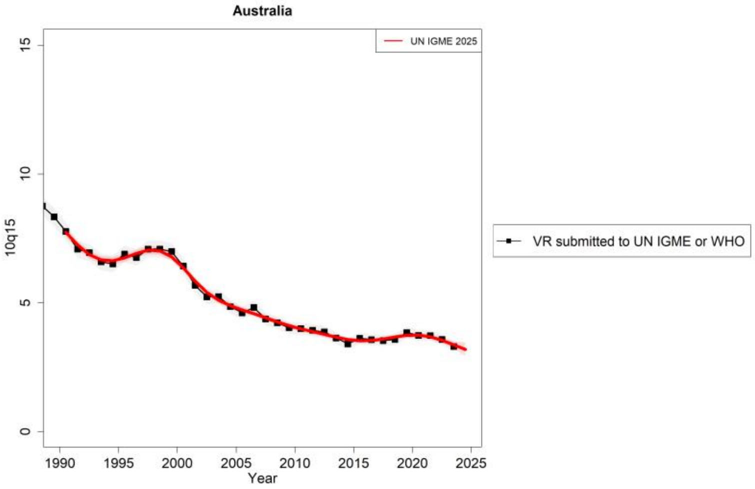
Australia
UN IGME 2025
10q15
0
5
10
15
1990
1995
2000
2005
2010
2015
2020
2025
Year
VR submitted to UN IGME or WHO

Austria (AUT)

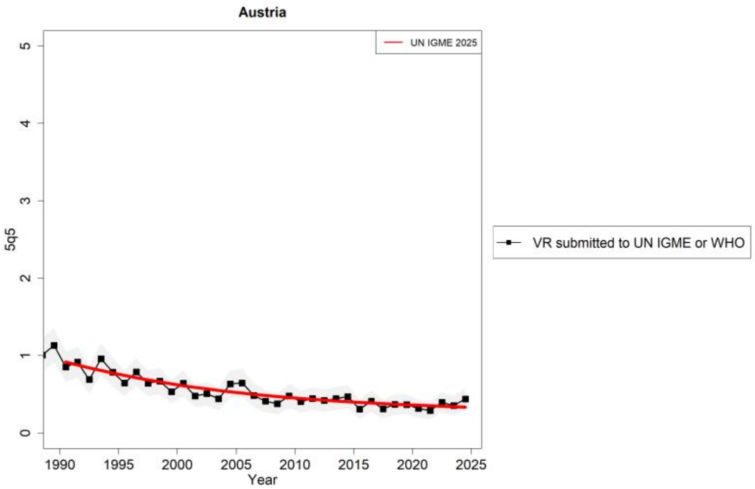

Austria
UN IGME 2025
VR submitted to UN IGME or WHO
5q5
Year

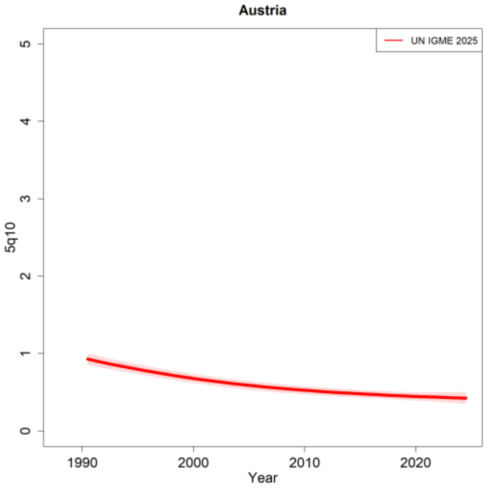

Austria
UN IGME 2025
5q10
Year

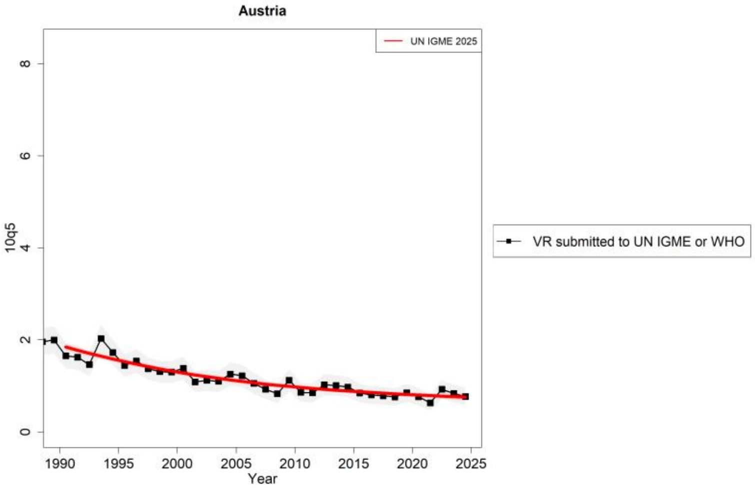

Austria
UN IGME 2025
VR submitted to UN IGME or WHO
10q5
Year

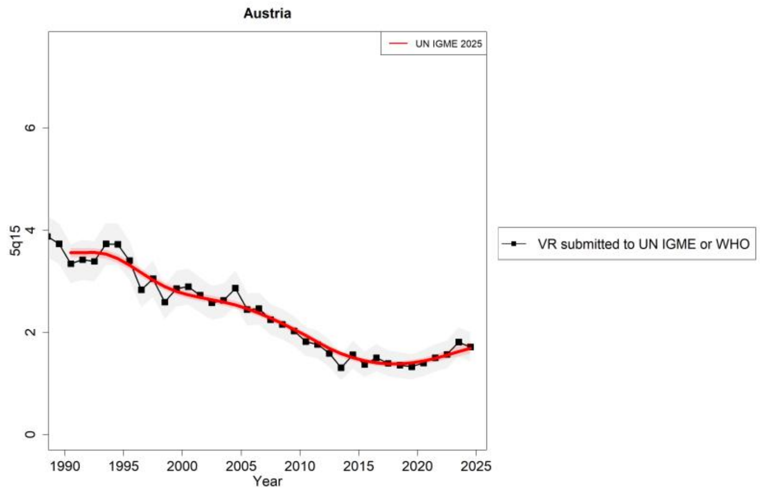
Austria
UN IGME 2025
VR submitted to UN IGME or WHO
5q15
Year

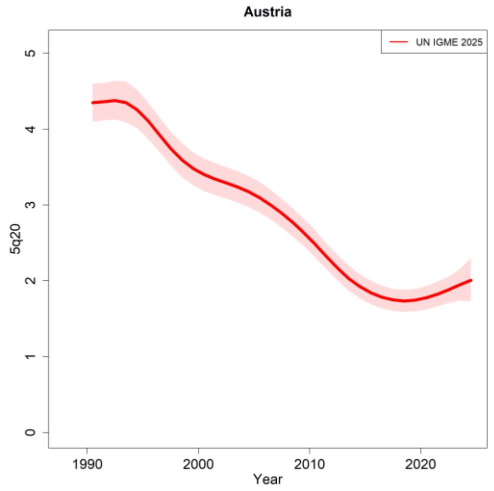
Austria
UN IGME 2025
5q20
Year

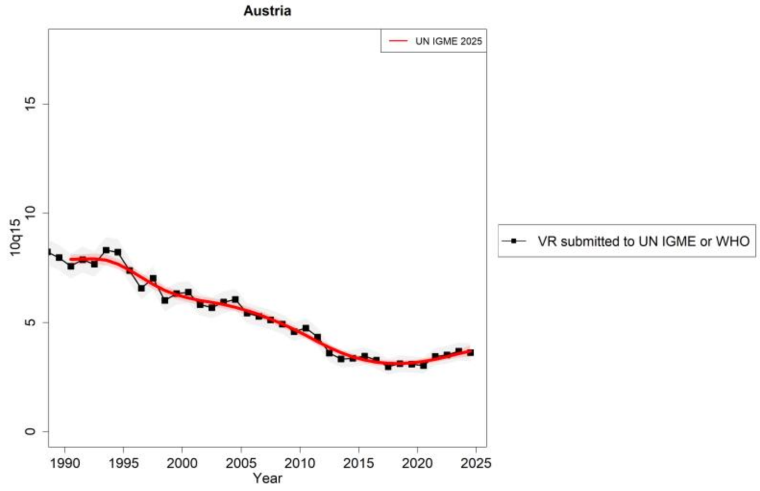
Austria
UN IGME 2025
VR submitted to UN IGME or WHO
10q15
Year

Azerbaijan (AZE)

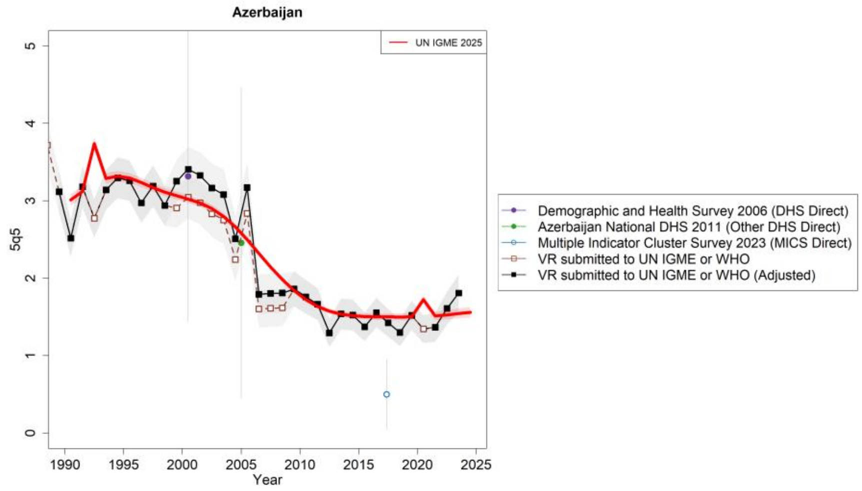

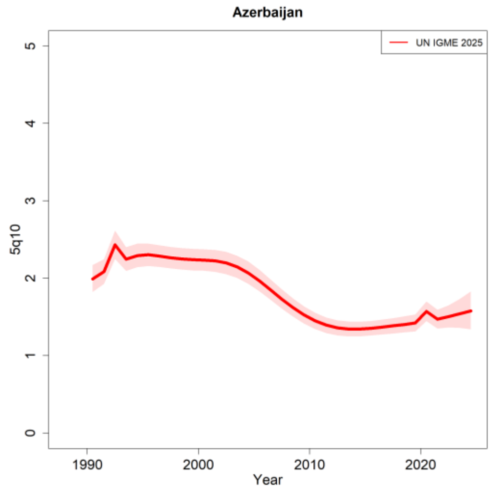

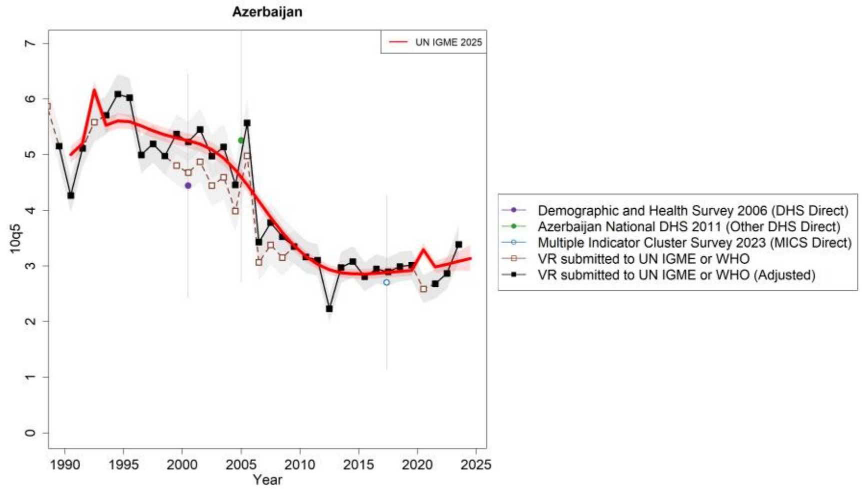

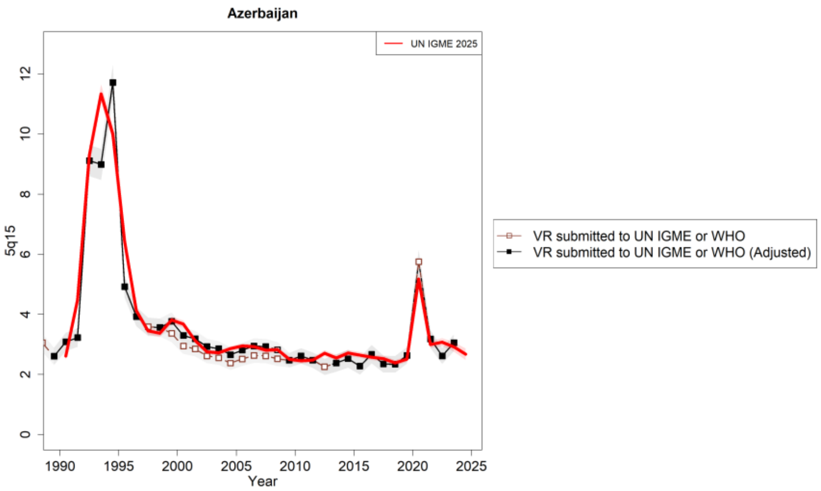
Azerbaijan
UN IGME 2025
VR submitted to UN IGME or WHO
VR submitted to UN IGME or WHO (Adjusted)
5q15
Year

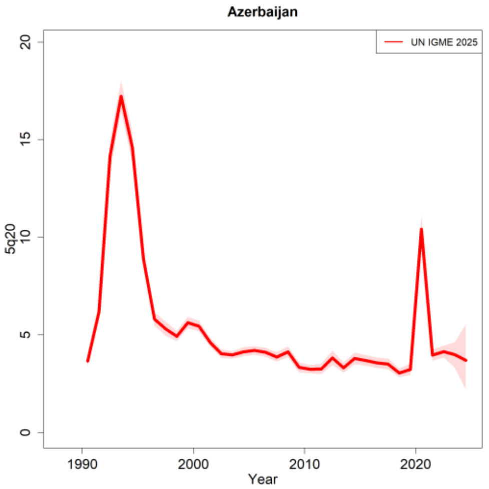
Azerbaijan
UN IGME 2025
5q20
Year

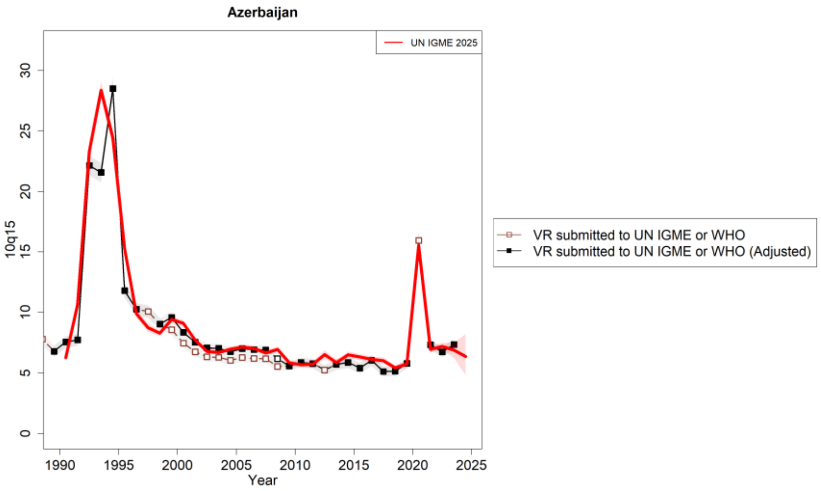
Azerbaijan
UN IGME 2025
VR submitted to UN IGME or WHO
VR submitted to UN IGME or WHO (Adjusted)
10q15
Year

Bahamas (BHS)

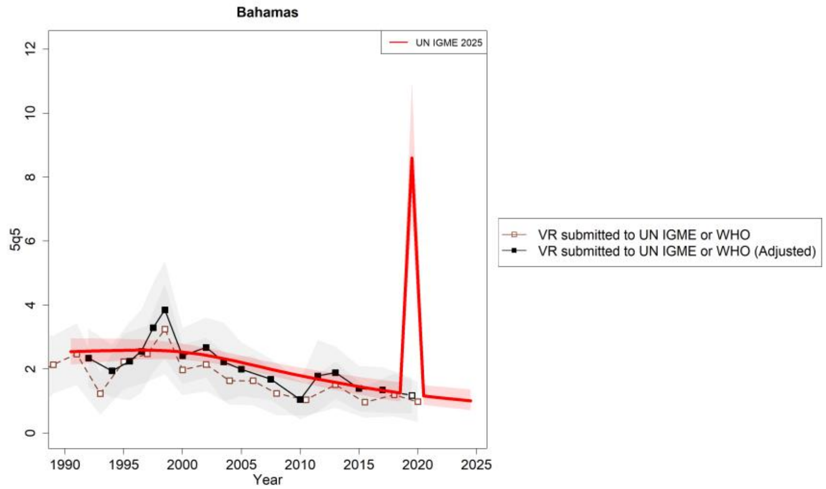
Bahamas
UN IGME 2025
VR submitted to UN IGME or WHO
VR submitted to UN IGME or WHO (Adjusted)
5q5
Year

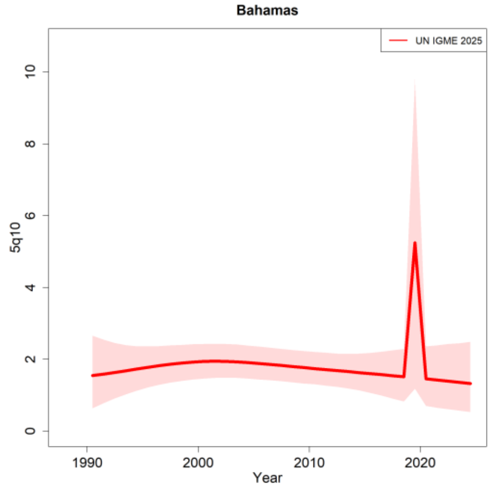
Bahamas
UN IGME 2025
5q10
Year

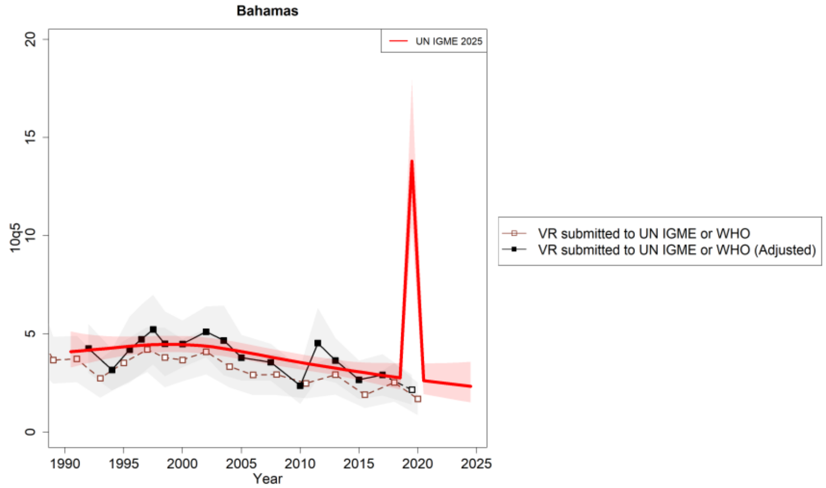
Bahamas
UN IGME 2025
VR submitted to UN IGME or WHO
VR submitted to UN IGME or WHO (Adjusted)
10q5
Year

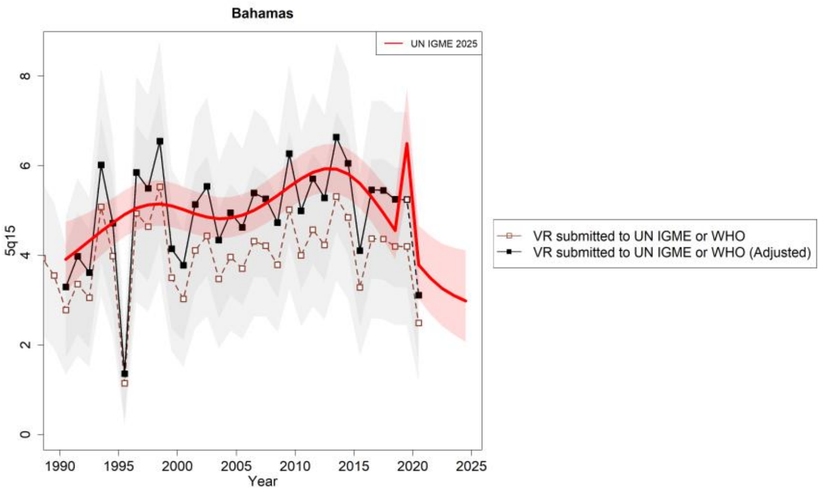
Bahamas
UN IGME 2025
VR submitted to UN IGME or WHO
VR submitted to UN IGME or WHO (Adjusted)
5q15
Year
0
2
4
6
8
1990
1995
2000
2005
2010
2015
2020
2025

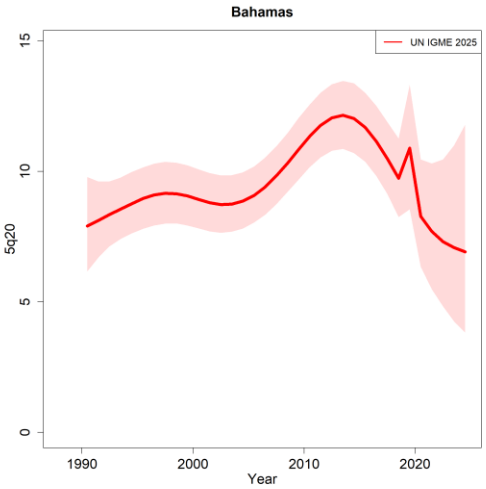
Bahamas
UN IGME 2025
5q20
Year
0
5
10
15
1990
2000
2010
2020

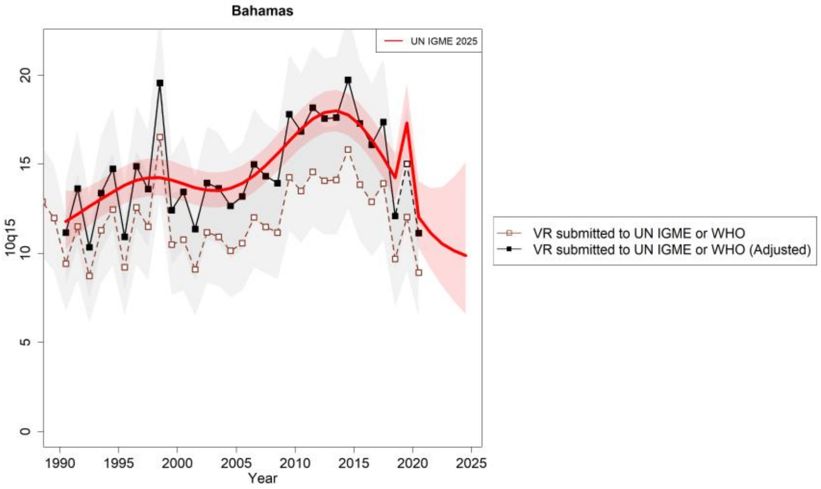
Bahamas
UN IGME 2025
VR submitted to UN IGME or WHO
VR submitted to UN IGME or WHO (Adjusted)
10q15
Year
0
5
10
15
20
1990
1995
2000
2005
2010
2015
2020
2025

Bahrain (BHR)

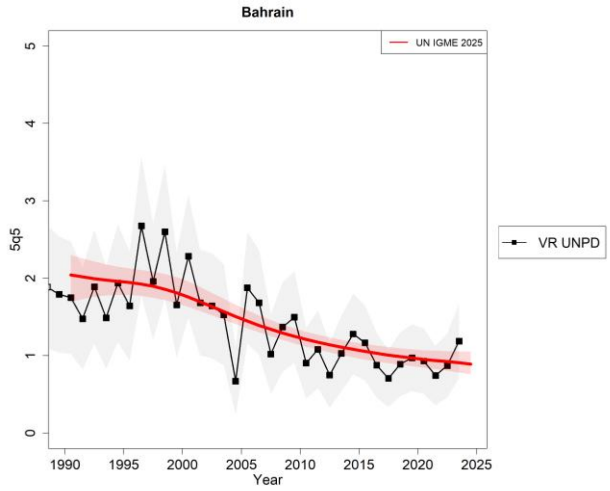
Bahrain
UN IGME 2025
VR UNPD
5q5
Year

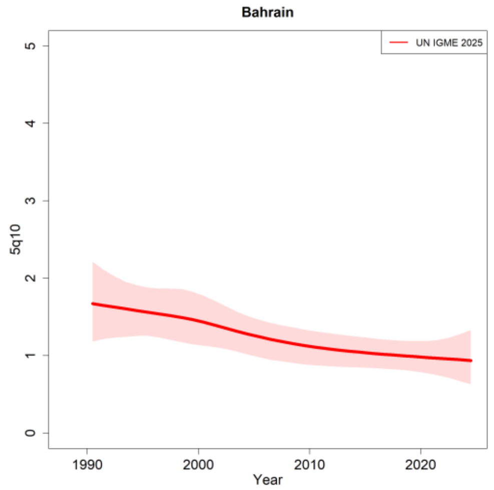
Bahrain
UN IGME 2025
5q10
Year

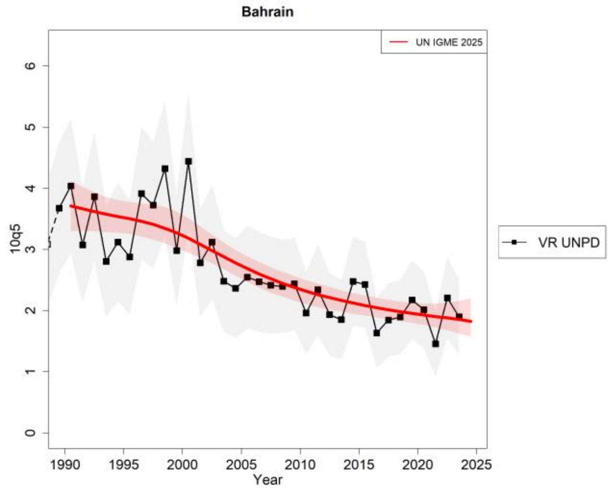
Bahrain
UN IGME 2025
VR UNPD
10q5
Year

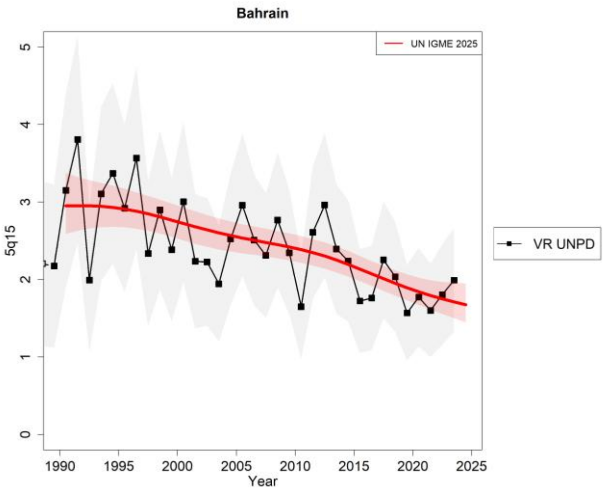
Bahrain
UN IGME 2025
VR UNPD
5q15
Year
0
1
2
3
4
5
1990
1995
2000
2005
2010
2015
2020
2025

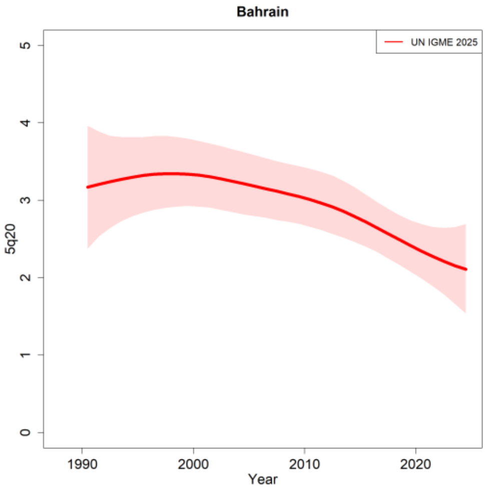
Bahrain
UN IGME 2025
5q20
Year
0
1
2
3
4
5
1990
2000
2010
2020

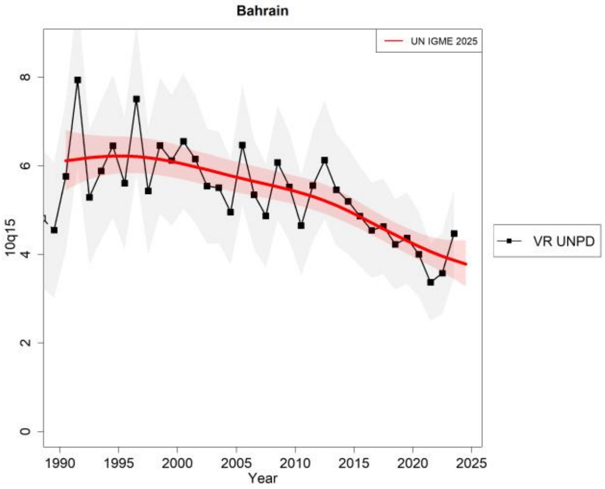
Bahrain
UN IGME 2025
VR UNPD
10q15
Year
0
2
4
6
8
1990
1995
2000
2005
2010
2015
2020
2025

Bangladesh (BGD)

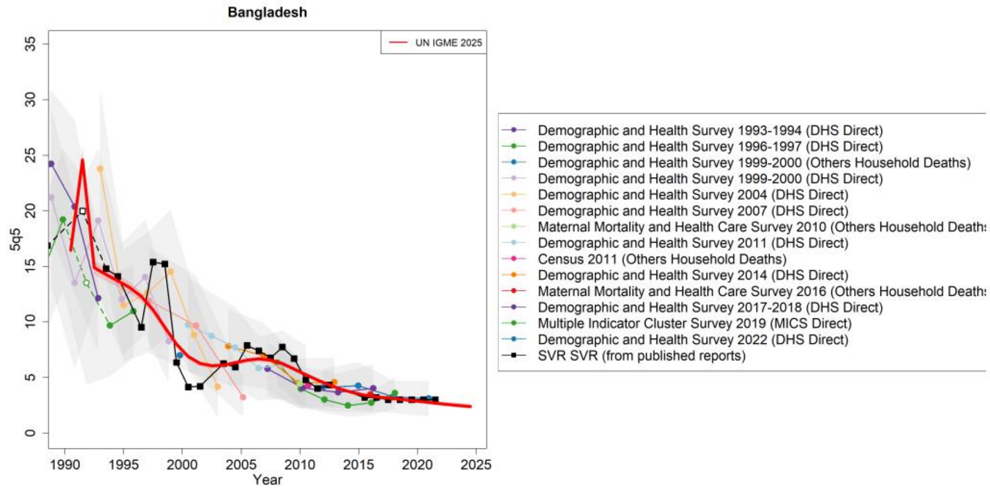

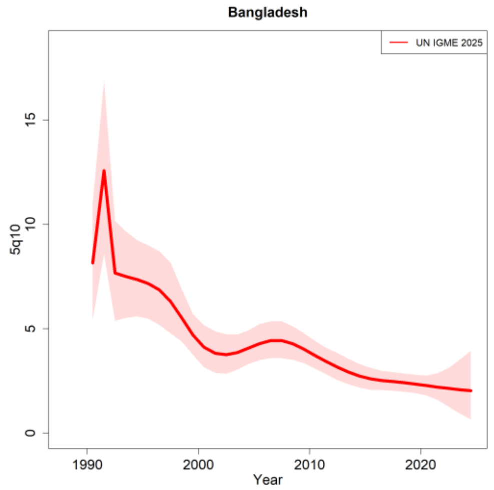

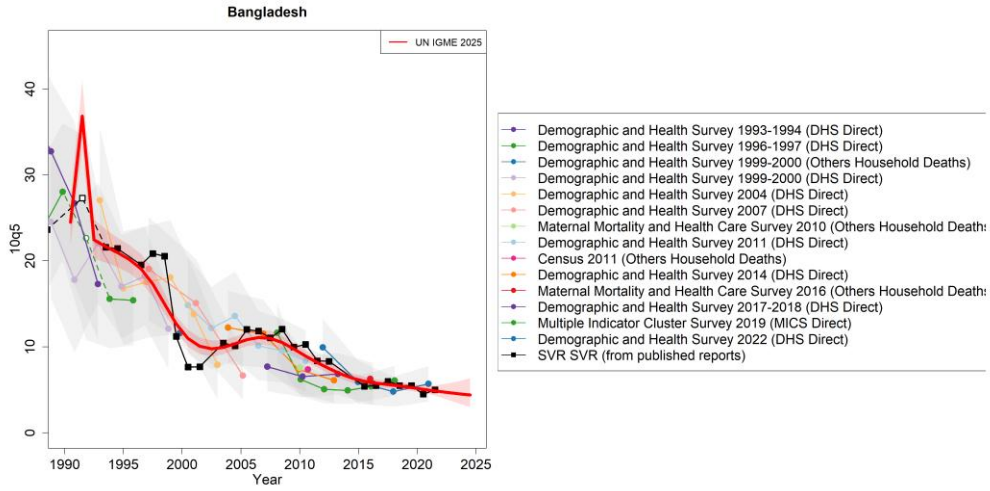

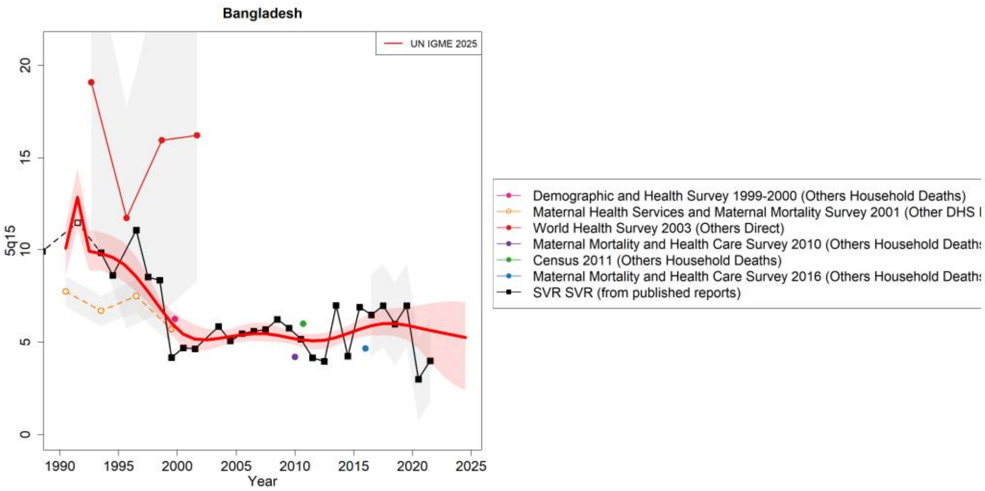
Bangladesh
UN IGME 2025
5q15
Year
Demographic and Health Survey 1999-2000 (Others Household Deaths)
Maternal Health Services and Maternal Mortality Survey 2001 (Other DHS I
World Health Survey 2003 (Others Direct)
Maternal Mortality and Health Care Survey 2010 (Others Household Death
Census 2011 (Others Household Deaths)
Maternal Mortality and Health Care Survey 2016 (Others Household Death
SVR SVR (from published reports)

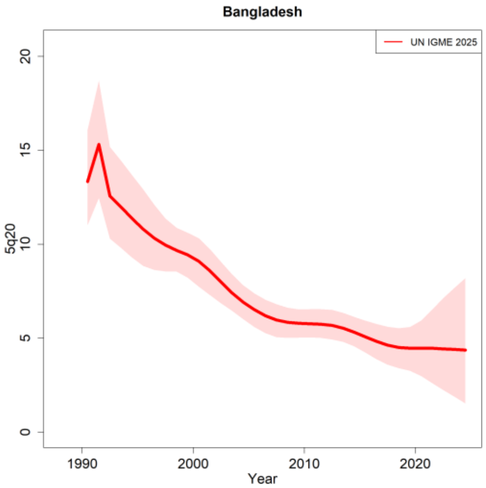
Bangladesh
UN IGME 2025
5q20
Year

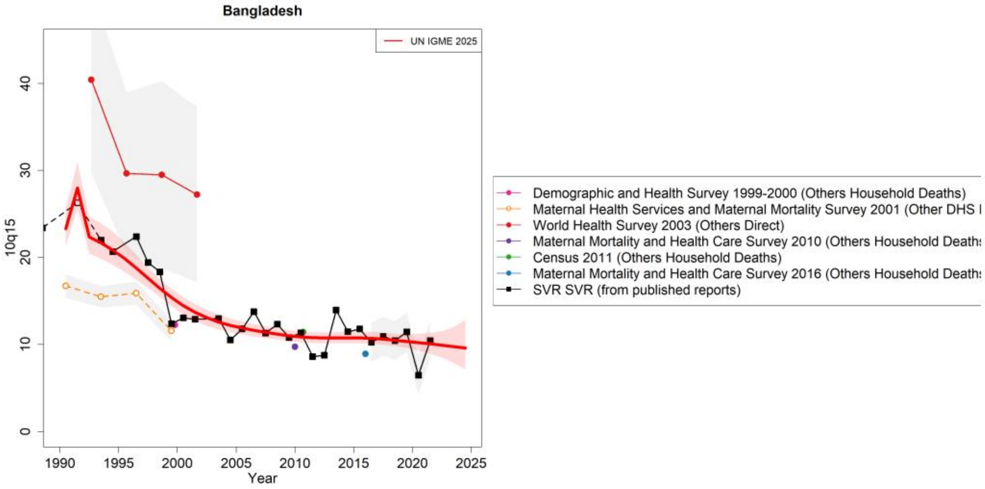
Bangladesh
UN IGME 2025
10q15
Year
Demographic and Health Survey 1999-2000 (Others Household Deaths)
Maternal Health Services and Maternal Mortality Survey 2001 (Other DHS I
World Health Survey 2003 (Others Direct)
Maternal Mortality and Health Care Survey 2010 (Others Household Death
Census 2011 (Others Household Deaths)
Maternal Mortality and Health Care Survey 2016 (Others Household Death
SVR SVR (from published reports)

Barbados (BRB)

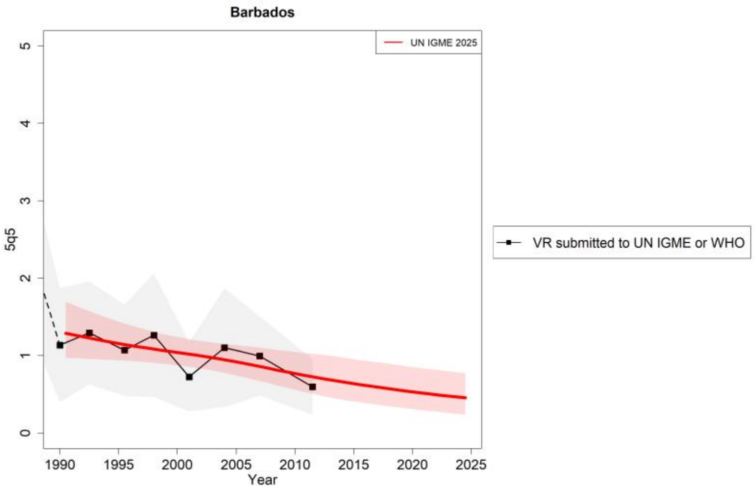

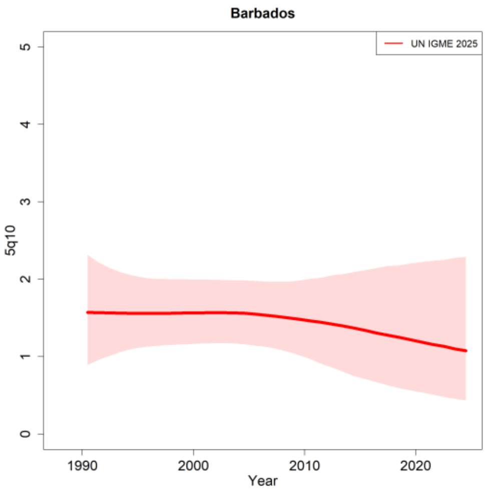

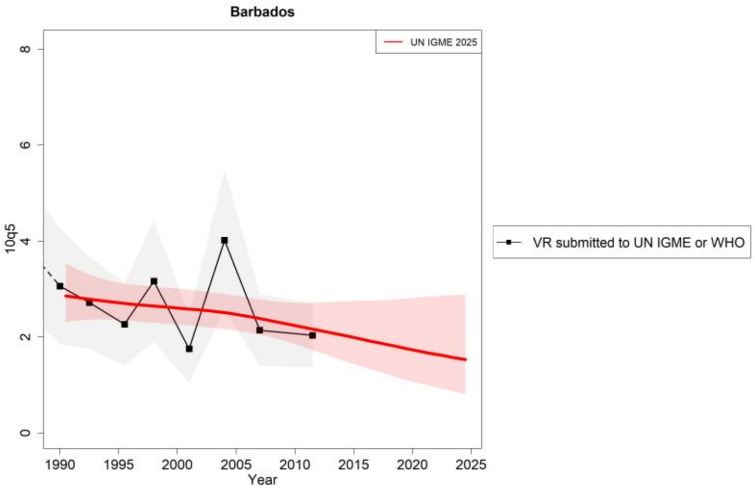

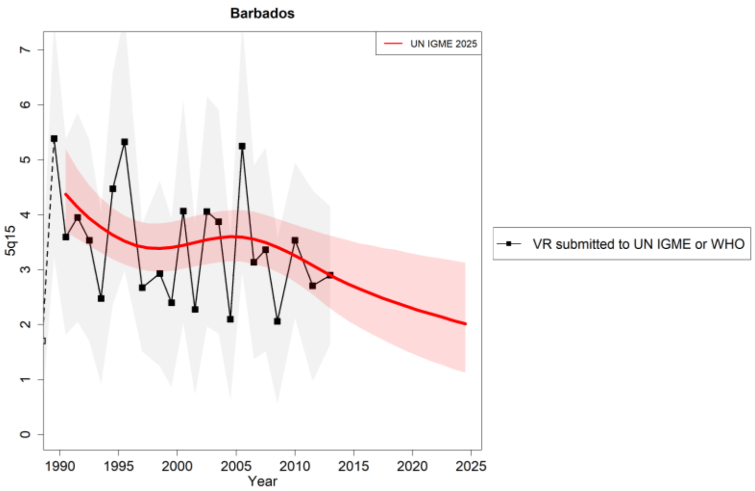
Barbados
UN IGME 2025
VR submitted to UN IGME or WHO
5q15
Year

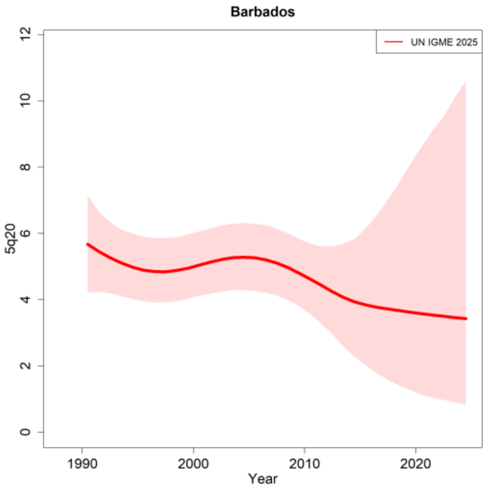
Barbados
UN IGME 2025
5q20
Year

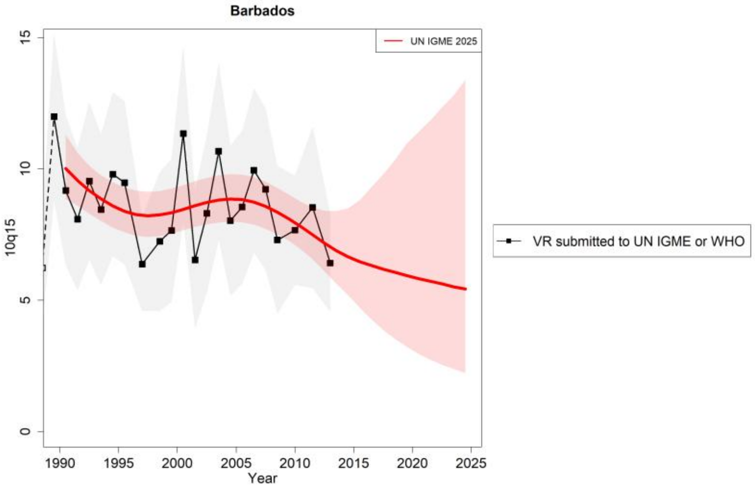
Barbados
UN IGME 2025
VR submitted to UN IGME or WHO
10q15
Year

Belarus (BLR)

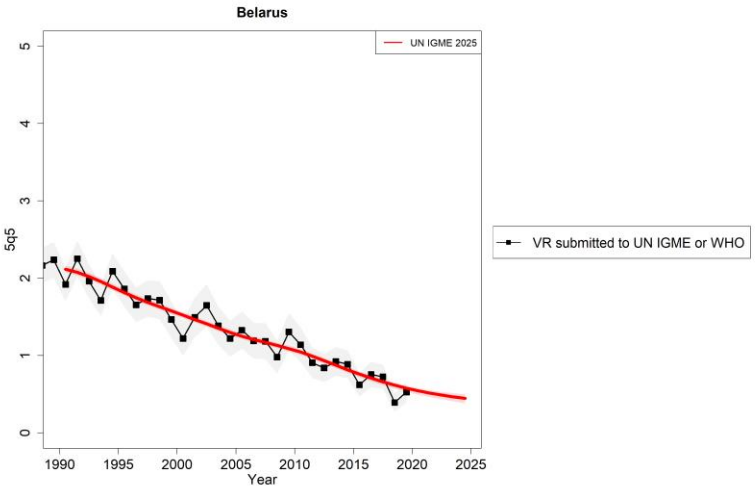
Belarus
UN IGME 2025
VR submitted to UN IGME or WHO
5q5
Year
0
1
2
3
4
5
1990
1995
2000
2005
2010
2015
2020
2025

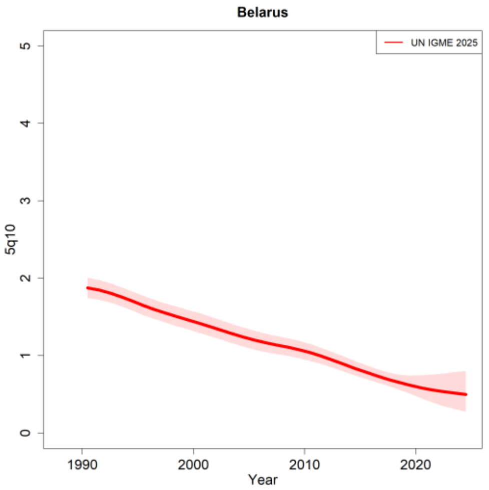
Belarus
UN IGME 2025
5q10
Year
0
1
2
3
4
5
1990
2000
2010
2020

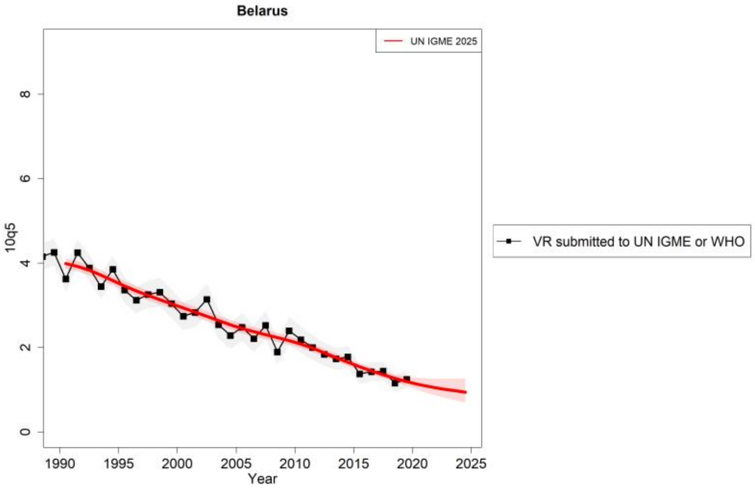
Belarus
UN IGME 2025
VR submitted to UN IGME or WHO
10q5
Year
0
2
4
6
8
1990
1995
2000
2005
2010
2015
2020
2025

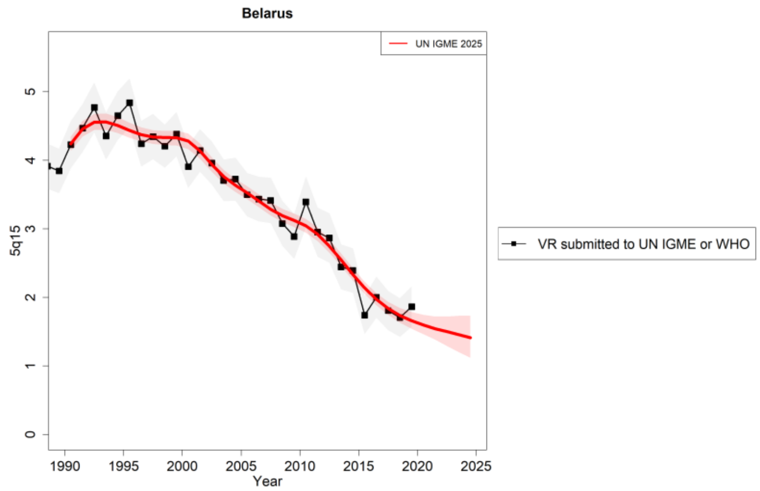
Belarus
UN IGME 2025
VR submitted to UN IGME or WHO
5q15
Year
0
1
2
3
4
5
1990
1995
2000
2005
2010
2015
2020
2025

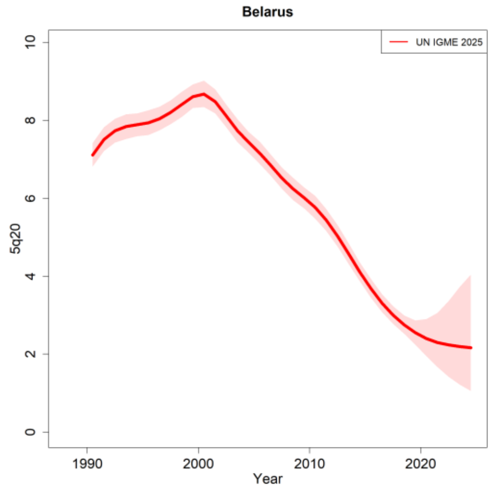
Belarus
UN IGME 2025
5q20
Year
0
2
4
6
8
10
1990
2000
2010
2020

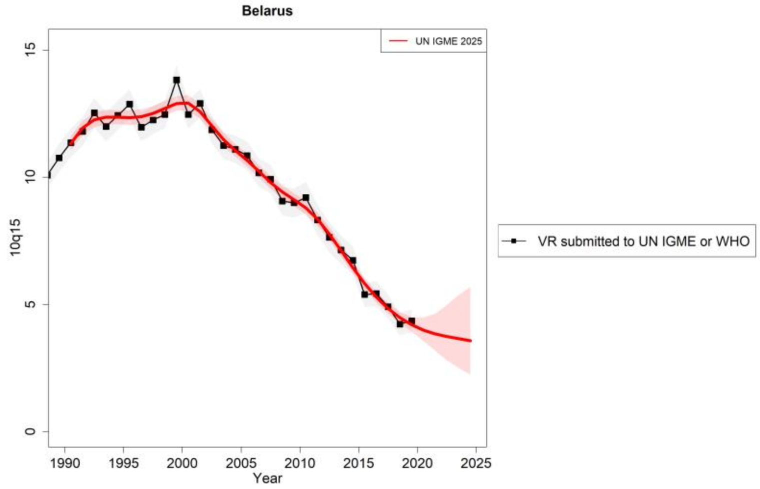
Belarus
UN IGME 2025
VR submitted to UN IGME or WHO
10q15
Year
0
5
10
15
1990
1995
2000
2005
2010
2015
2020
2025

Belgium (BEL)

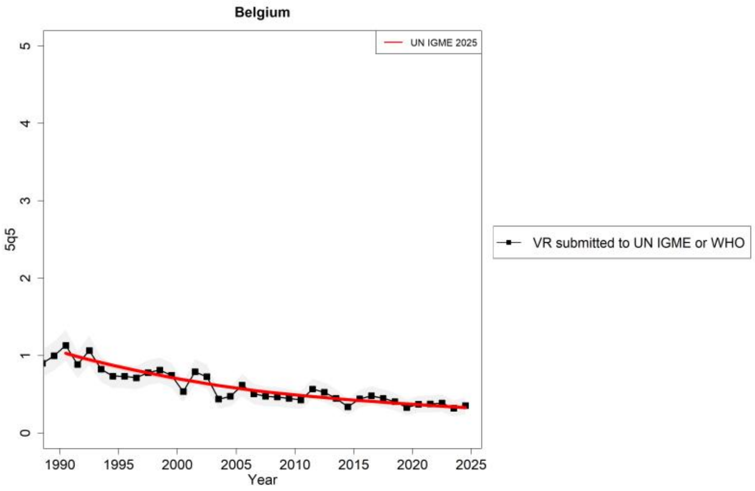
Belgium
UN IGME 2025
VR submitted to UN IGME or WHO
5q5
0
1
2
3
4
5
1990
1995
2000
2005
2010
2015
2020
2025
Year

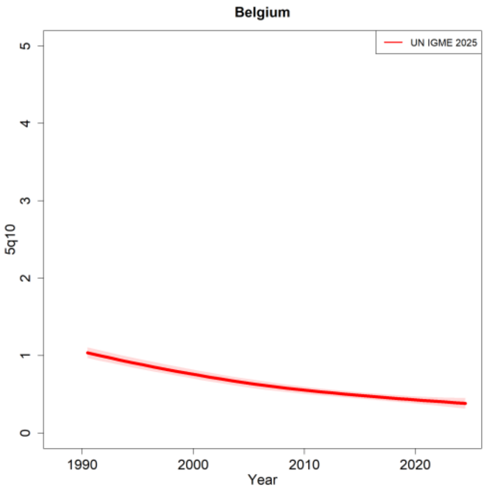
Belgium
UN IGME 2025
5q10
0
1
2
3
4
5
1990
2000
2010
2020
Year

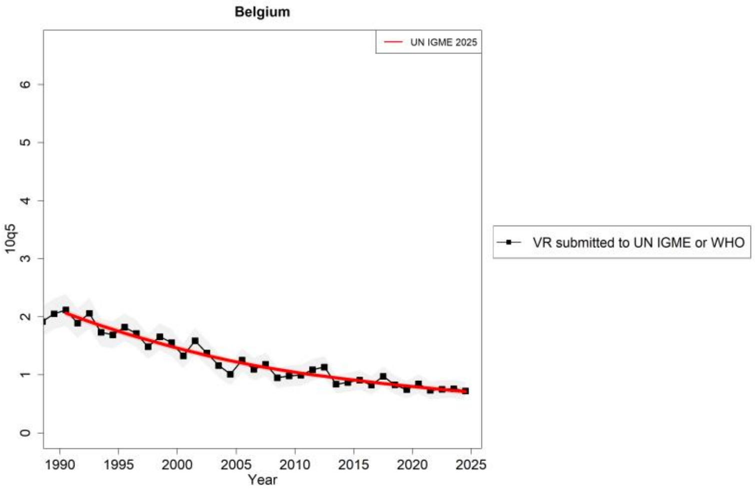
Belgium
UN IGME 2025
VR submitted to UN IGME or WHO
10q5
0
1
2
3
4
5
6
1990
1995
2000
2005
2010
2015
2020
2025
Year

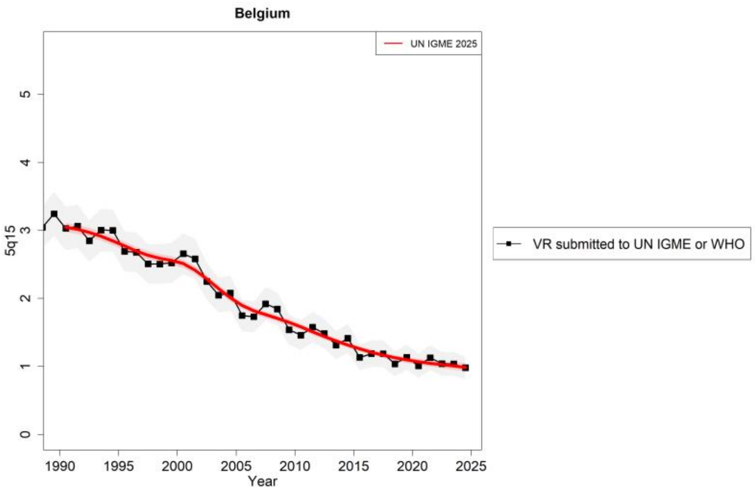
Belgium
UN IGME 2025
VR submitted to UN IGME or WHO
5q15
0
1
2
3
4
5
1990
1995
2000
2005
2010
2015
2020
2025
Year

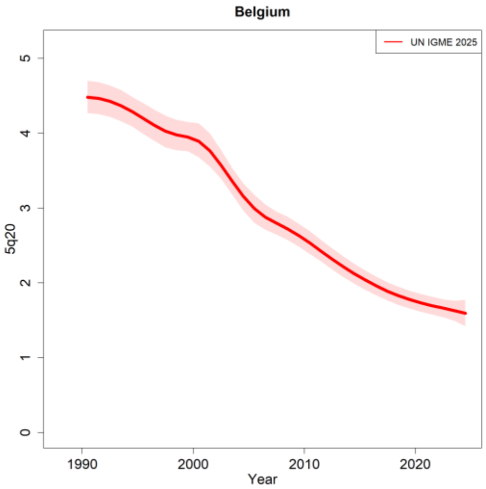
Belgium
UN IGME 2025
5q20
0
1
2
3
4
5
1990
2000
2010
2020
Year

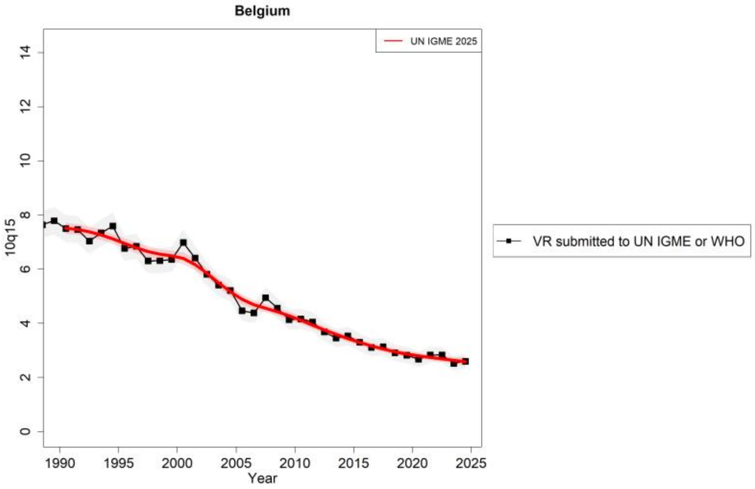
Belgium
UN IGME 2025
VR submitted to UN IGME or WHO
10q15
0
2
4
6
8
10
12
14
1990
1995
2000
2005
2010
2015
2020
2025
Year

Belize (BLZ)

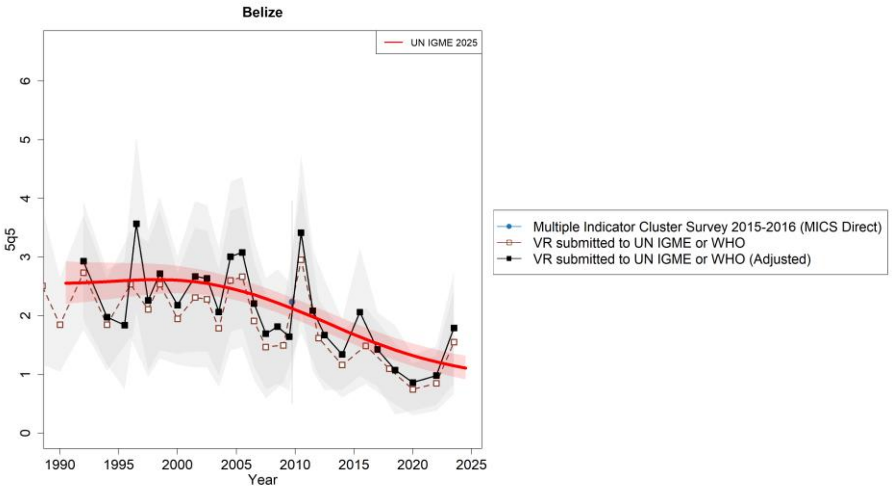
Belize
UN IGME 2025
5q5
Year
0
1
2
3
4
5
6
1990
1995
2000
2005
2010
2015
2020
2025
Multiple Indicator Cluster Survey 2015-2016 (MICS Direct)
VR submitted to UN IGME or WHO
VR submitted to UN IGME or WHO (Adjusted)

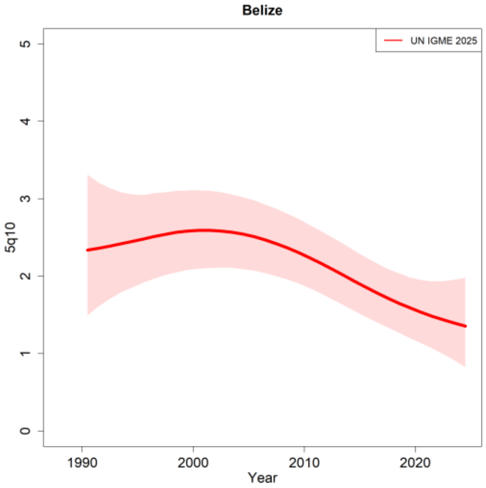
Belize
UN IGME 2025
5q10
Year
0
1
2
3
4
5
1990
2000
2010
2020

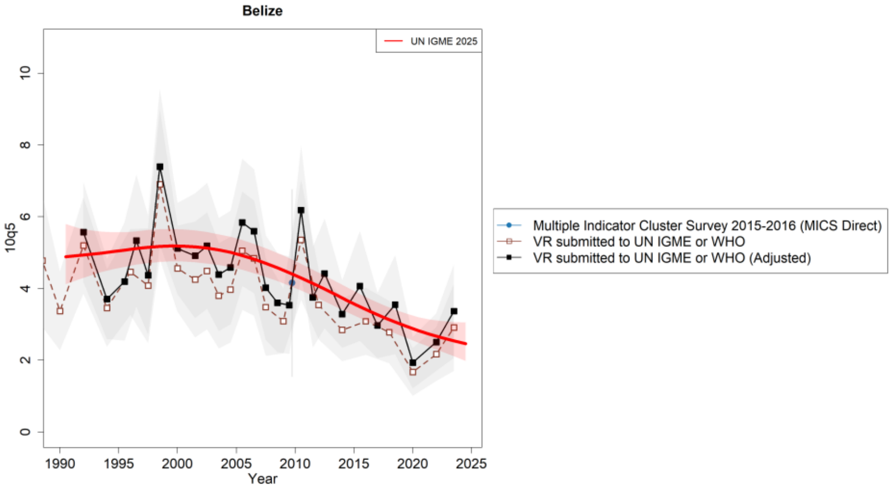
Belize
UN IGME 2025
10q5
Year
0
2
4
6
8
10
1990
1995
2000
2005
2010
2015
2020
2025
Multiple Indicator Cluster Survey 2015-2016 (MICS Direct)
VR submitted to UN IGME or WHO
VR submitted to UN IGME or WHO (Adjusted)

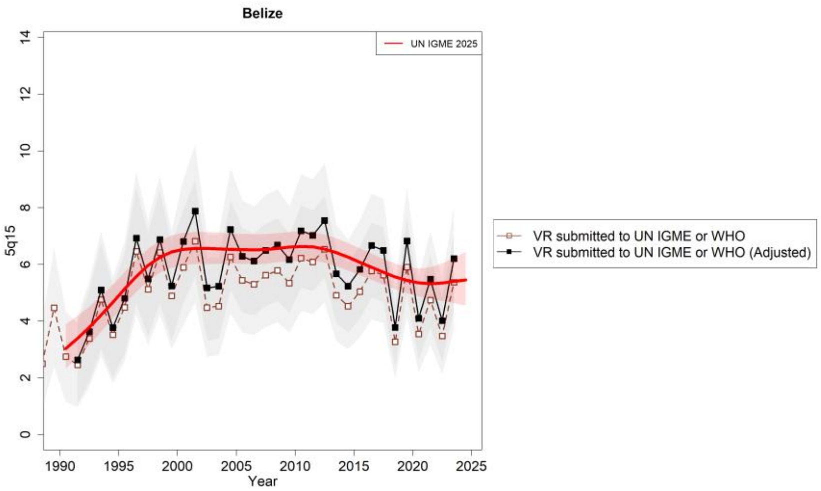
Belize
UN IGME 2025
VR submitted to UN IGME or WHO
VR submitted to UN IGME or WHO (Adjusted)
5q15
Year

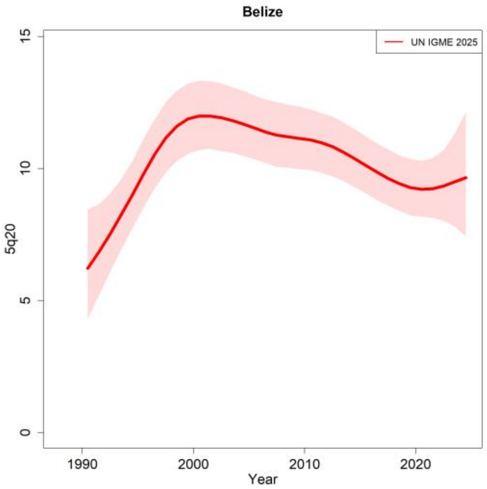
Belize
UN IGME 2025
5q20
Year

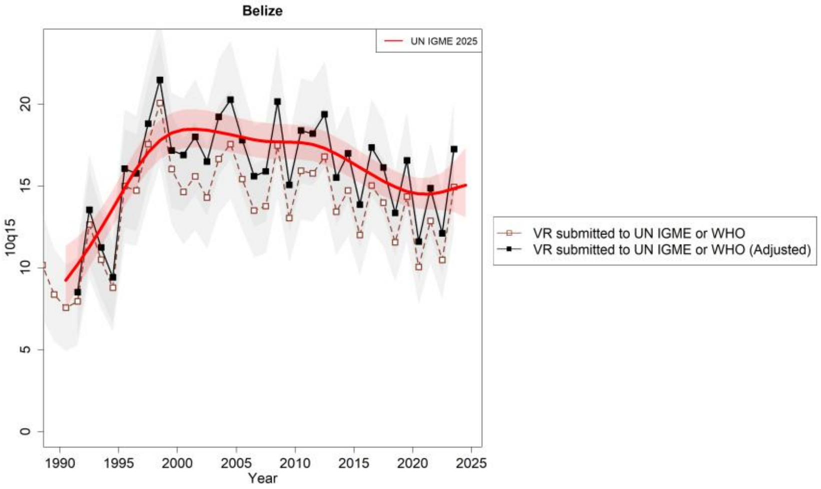
Belize
UN IGME 2025
VR submitted to UN IGME or WHO
VR submitted to UN IGME or WHO (Adjusted)
10q15
Year

Benin (BEN)

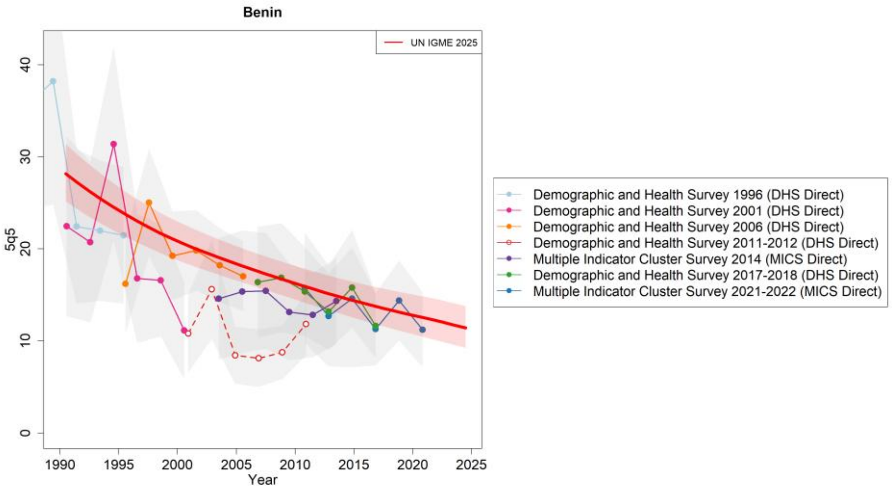
Benin
UN IGME 2025
5q5
Year
Demographic and Health Survey 1996 (DHS Direct)
Demographic and Health Survey 2001 (DHS Direct)
Demographic and Health Survey 2006 (DHS Direct)
Demographic and Health Survey 2011-2012 (DHS Direct)
Multiple Indicator Cluster Survey 2014 (MICS Direct)
Demographic and Health Survey 2017-2018 (DHS Direct)
Multiple Indicator Cluster Survey 2021-2022 (MICS Direct)

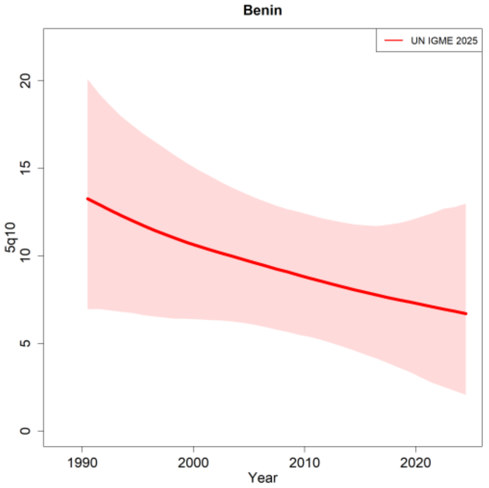
Benin
UN IGME 2025
5q10
Year

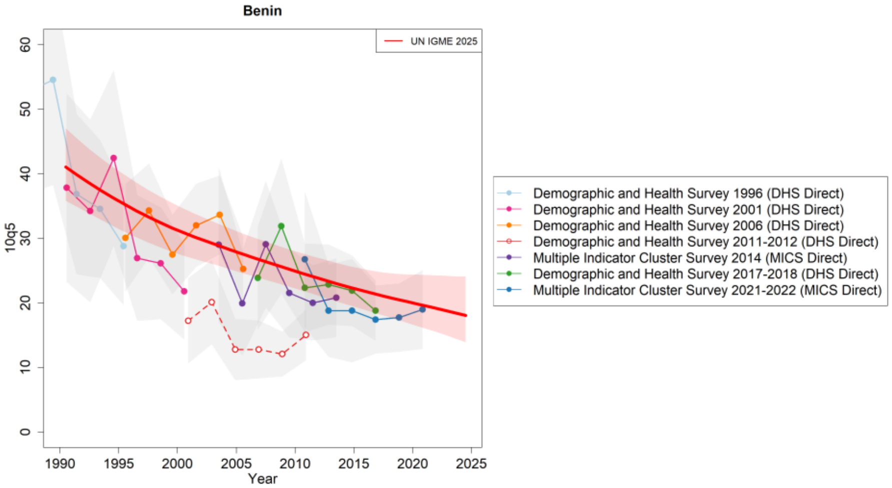
Benin
UN IGME 2025
10q5
Year
Demographic and Health Survey 1996 (DHS Direct)
Demographic and Health Survey 2001 (DHS Direct)
Demographic and Health Survey 2006 (DHS Direct)
Demographic and Health Survey 2011-2012 (DHS Direct)
Multiple Indicator Cluster Survey 2014 (MICS Direct)
Demographic and Health Survey 2017-2018 (DHS Direct)
Multiple Indicator Cluster Survey 2021-2022 (MICS Direct)

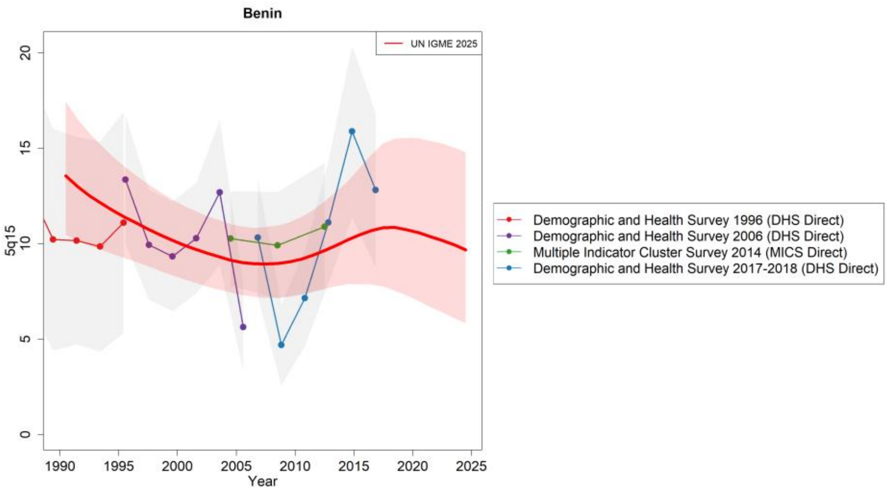
Benin
UN IGME 2025
5q15
Year
Demographic and Health Survey 1996 (DHS Direct)
Demographic and Health Survey 2006 (DHS Direct)
Multiple Indicator Cluster Survey 2014 (MICS Direct)
Demographic and Health Survey 2017-2018 (DHS Direct)

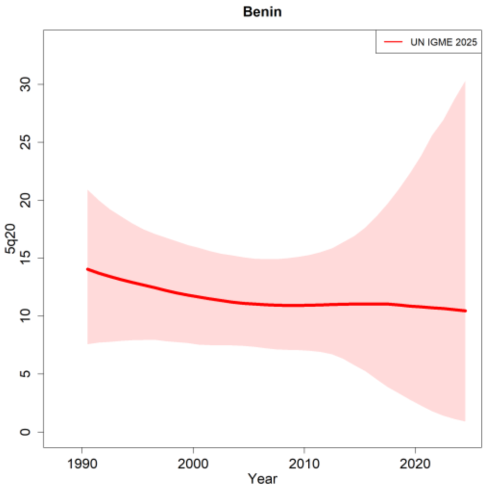
Benin
UN IGME 2025
5q20
Year

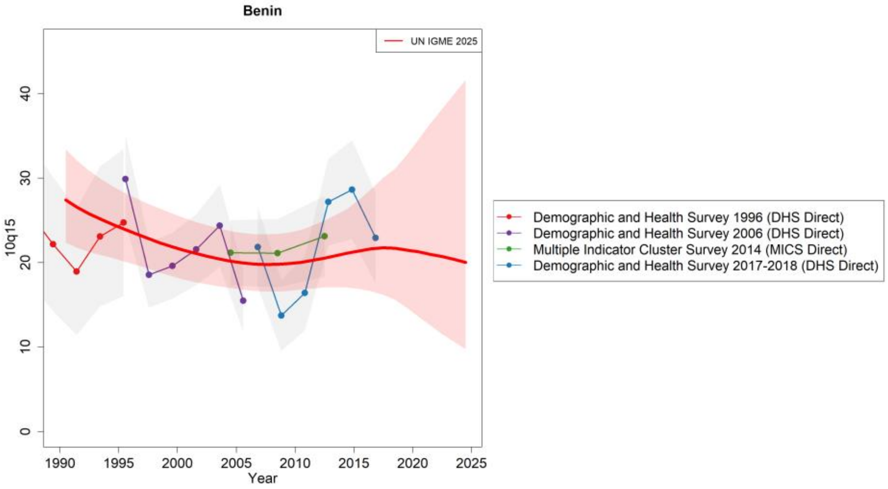
Benin
UN IGME 2025
10q15
Year
Demographic and Health Survey 1996 (DHS Direct)
Demographic and Health Survey 2006 (DHS Direct)
Multiple Indicator Cluster Survey 2014 (MICS Direct)
Demographic and Health Survey 2017-2018 (DHS Direct)

Bhutan (BTN)

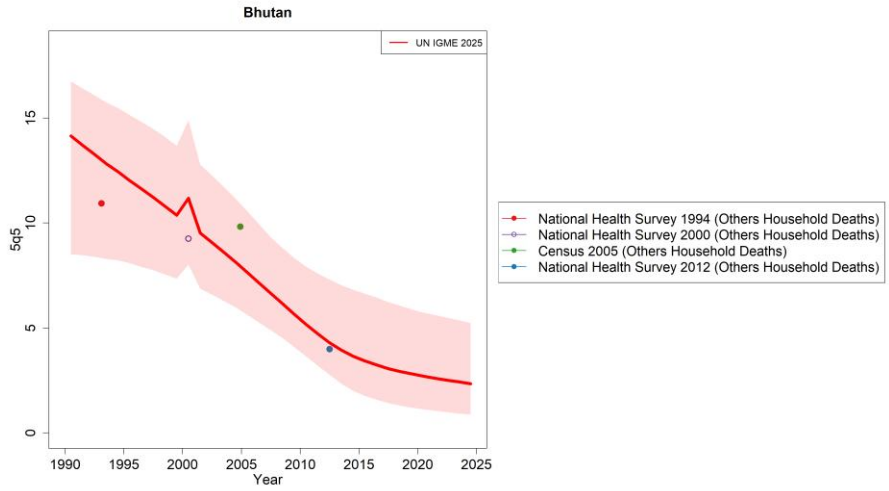
Bhutan
UN IGME 2025
5q5
Year
National Health Survey 1994 (Others Household Deaths)
National Health Survey 2000 (Others Household Deaths)
Census 2005 (Others Household Deaths)
National Health Survey 2012 (Others Household Deaths)

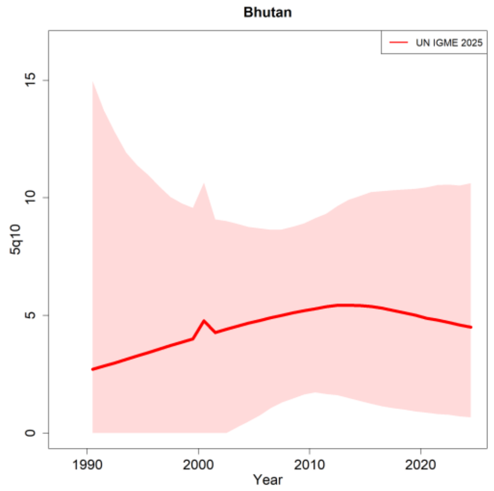
Bhutan
UN IGME 2025
5q10
Year

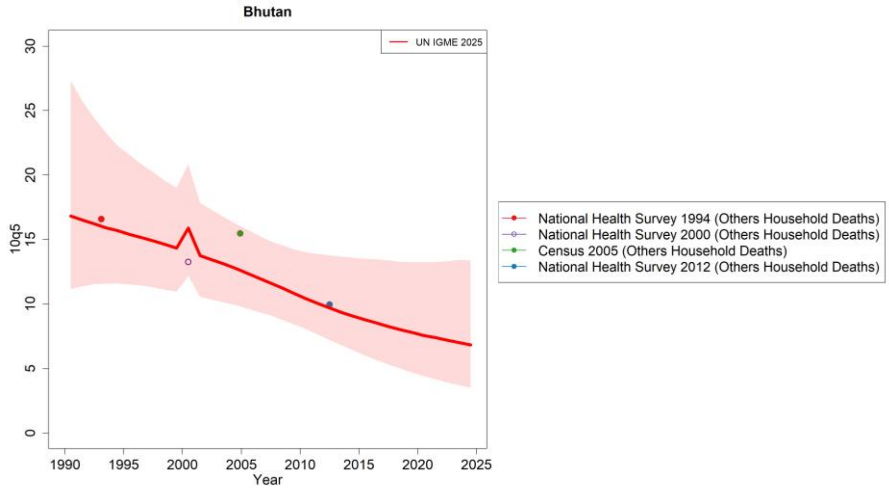
Bhutan
UN IGME 2025
10q5
Year
National Health Survey 1994 (Others Household Deaths)
National Health Survey 2000 (Others Household Deaths)
Census 2005 (Others Household Deaths)
National Health Survey 2012 (Others Household Deaths)

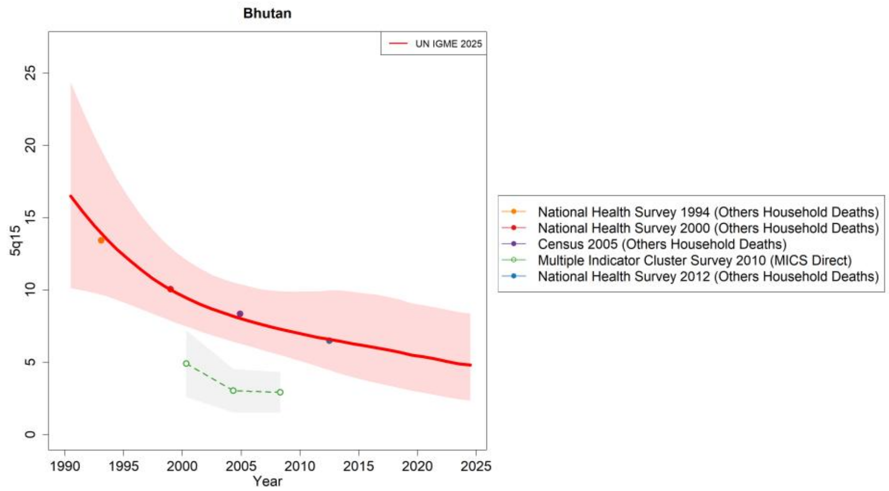
Bhutan
UN IGME 2025
5q15
Year
National Health Survey 1994 (Others Household Deaths)
National Health Survey 2000 (Others Household Deaths)
Census 2005 (Others Household Deaths)
Multiple Indicator Cluster Survey 2010 (MICS Direct)
National Health Survey 2012 (Others Household Deaths)

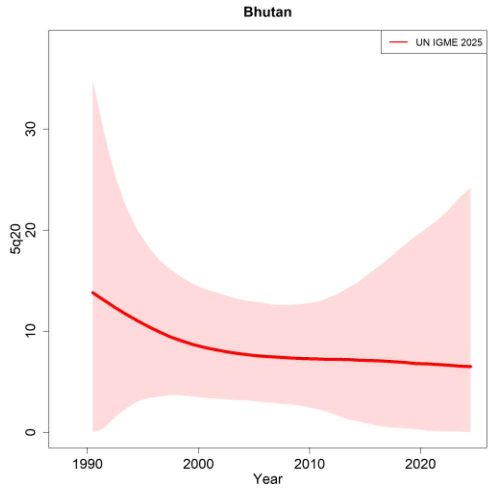
Bhutan
UN IGME 2025
5q20
Year

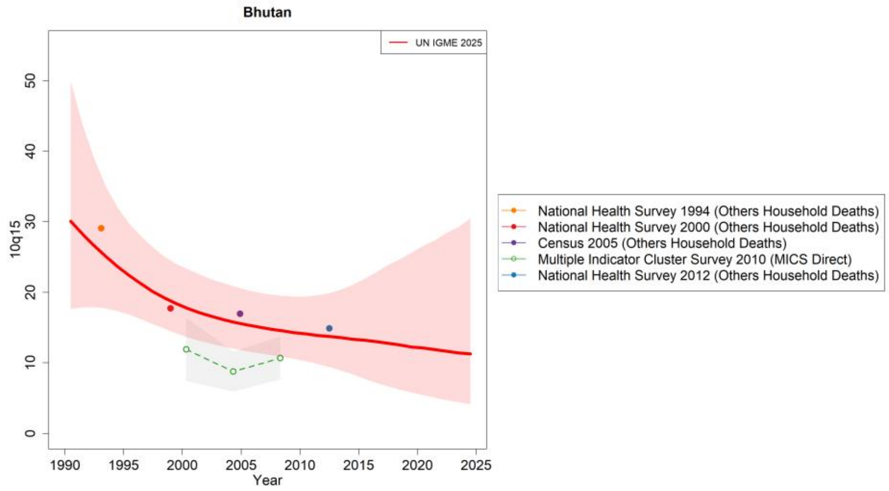
Bhutan
UN IGME 2025
10q15
Year
National Health Survey 1994 (Others Household Deaths)
National Health Survey 2000 (Others Household Deaths)
Census 2005 (Others Household Deaths)
Multiple Indicator Cluster Survey 2010 (MICS Direct)
National Health Survey 2012 (Others Household Deaths)

Bolivia (Plurinational State of) (BOL)

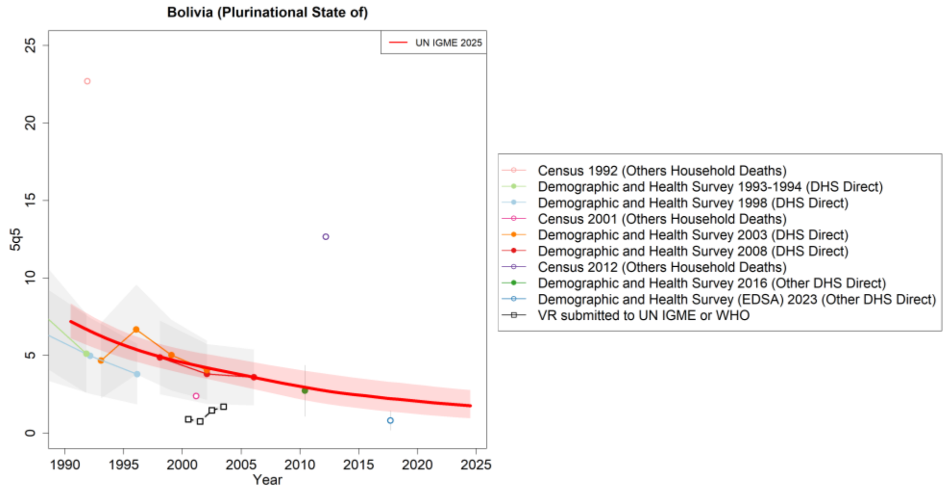

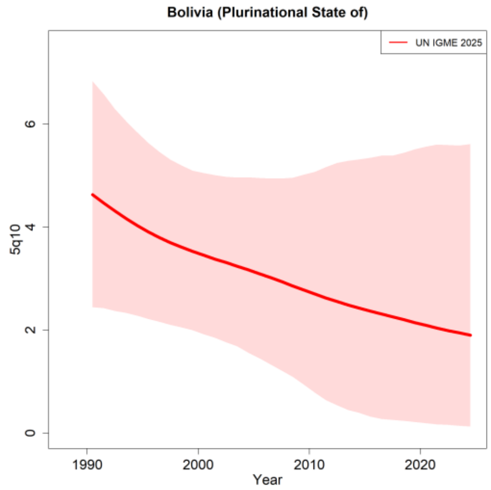

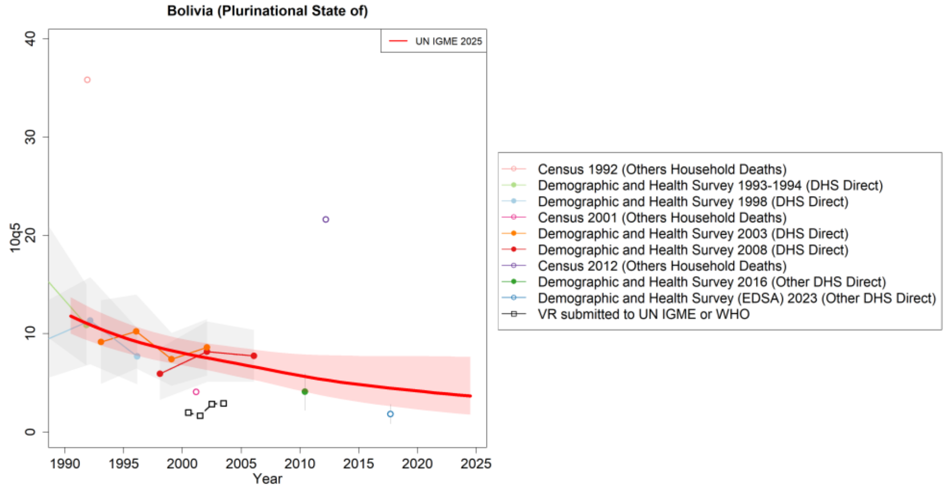

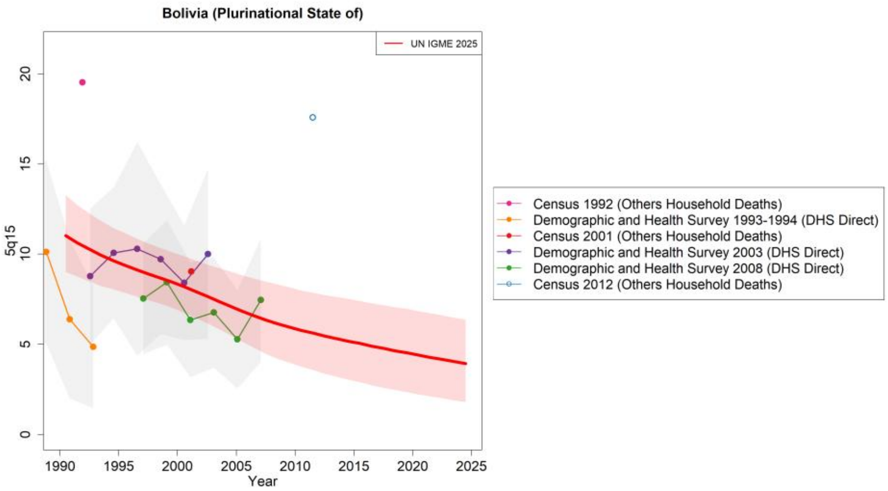
Bolivia (Plurinational State of)
UN IGME 2025
5q15
Year
Census 1992 (Others Household Deaths)
Demographic and Health Survey 1993-1994 (DHS Direct)
Census 2001 (Others Household Deaths)
Demographic and Health Survey 2003 (DHS Direct)
Demographic and Health Survey 2008 (DHS Direct)
Census 2012 (Others Household Deaths)

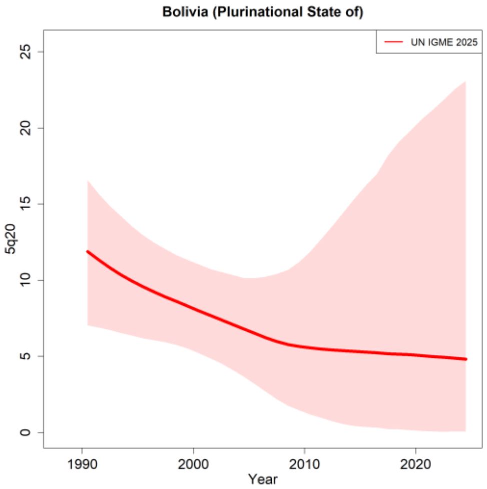
Bolivia (Plurinational State of)
UN IGME 2025
5q20
Year

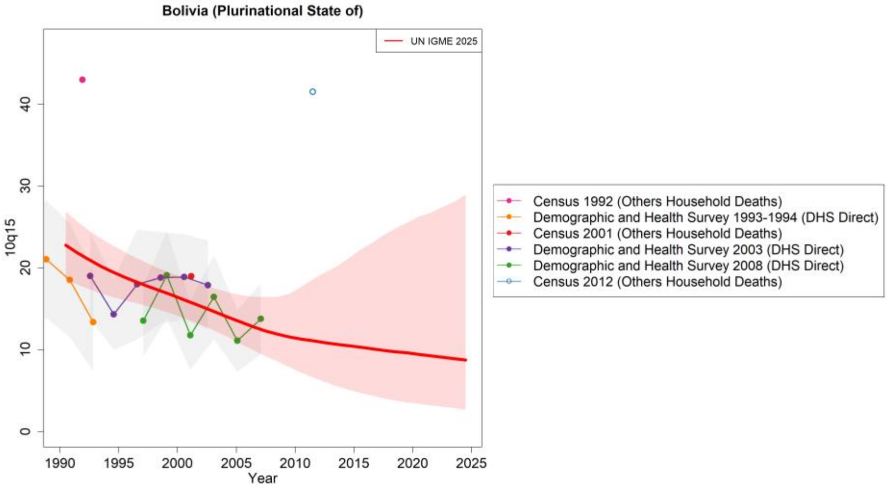
Bolivia (Plurinational State of)
UN IGME 2025
10q15
Year
Census 1992 (Others Household Deaths)
Demographic and Health Survey 1993-1994 (DHS Direct)
Census 2001 (Others Household Deaths)
Demographic and Health Survey 2003 (DHS Direct)
Demographic and Health Survey 2008 (DHS Direct)
Census 2012 (Others Household Deaths)

Bosnia and Herzegovina (BIH)

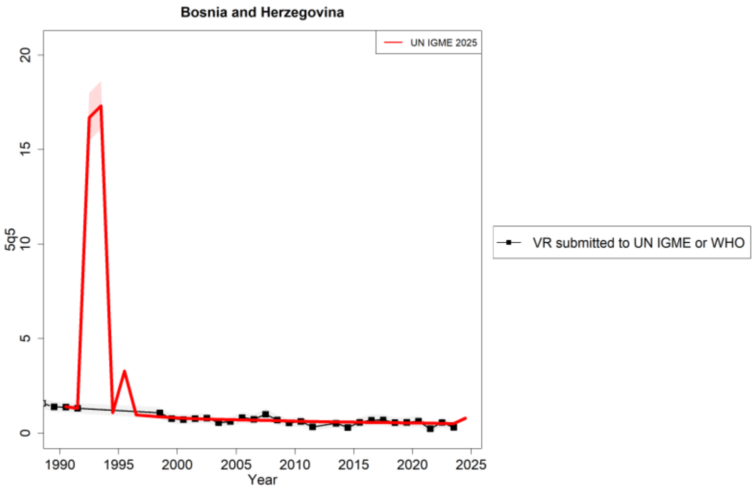

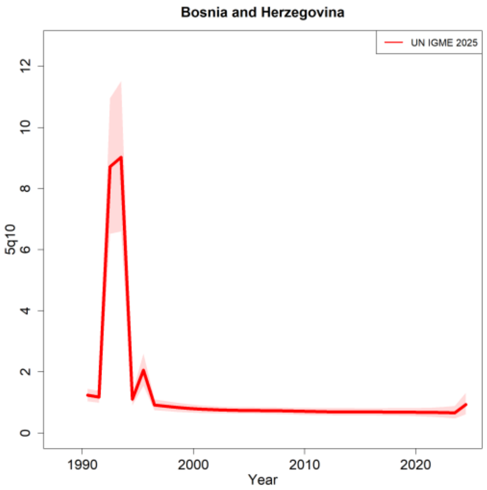

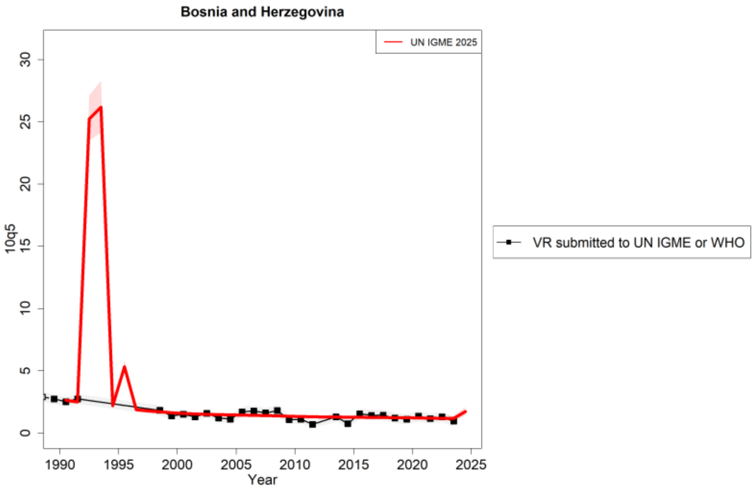

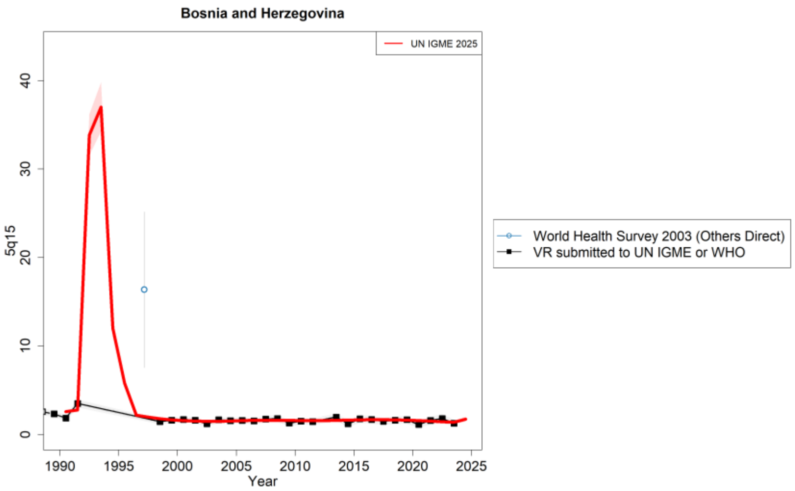
Bosnia and Herzegovina
UN IGME 2025
5q15
0
10
20
30
40
1990
1995
2000
2005
2010
2015
2020
2025
Year
World Health Survey 2003 (Others Direct)
VR submitted to UN IGME or WHO

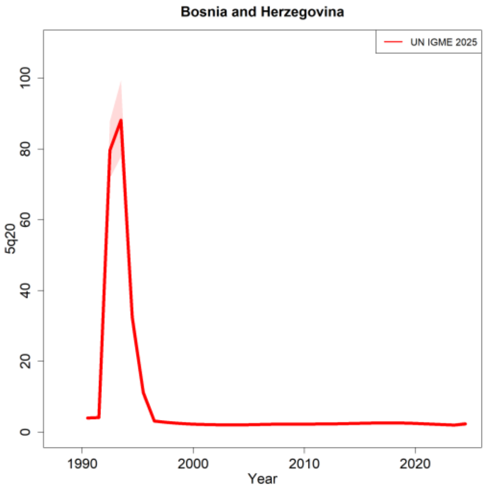
Bosnia and Herzegovina
UN IGME 2025
5q20
0
20
40
60
80
100
1990
2000
2010
2020
Year

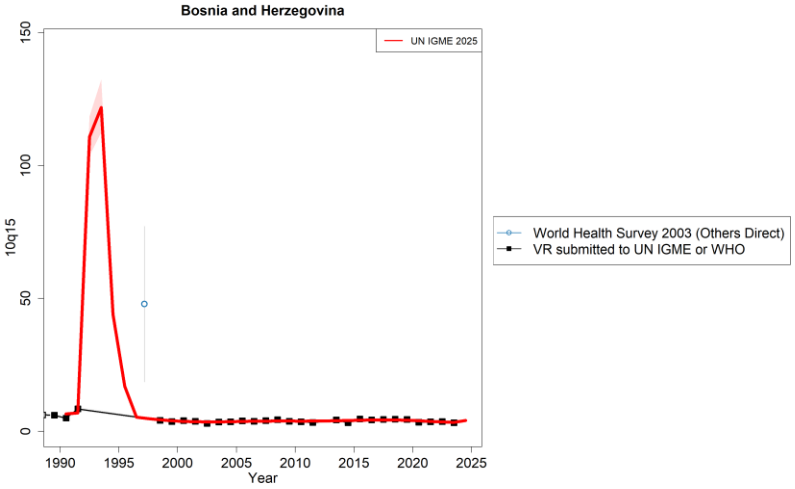
Bosnia and Herzegovina
UN IGME 2025
10q15
0
50
100
150
1990
1995
2000
2005
2010
2015
2020
2025
Year
World Health Survey 2003 (Others Direct)
VR submitted to UN IGME or WHO

Botswana (BWA)

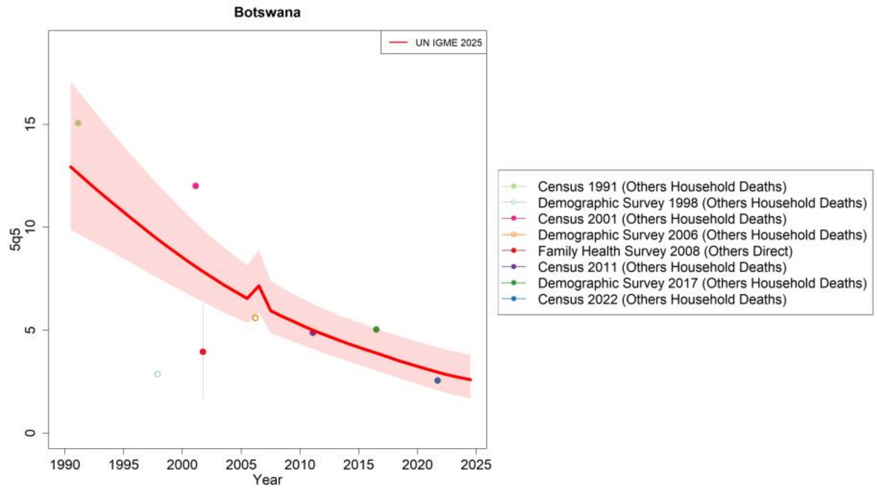

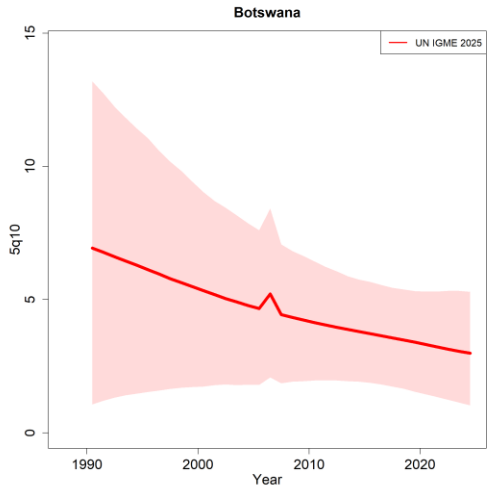

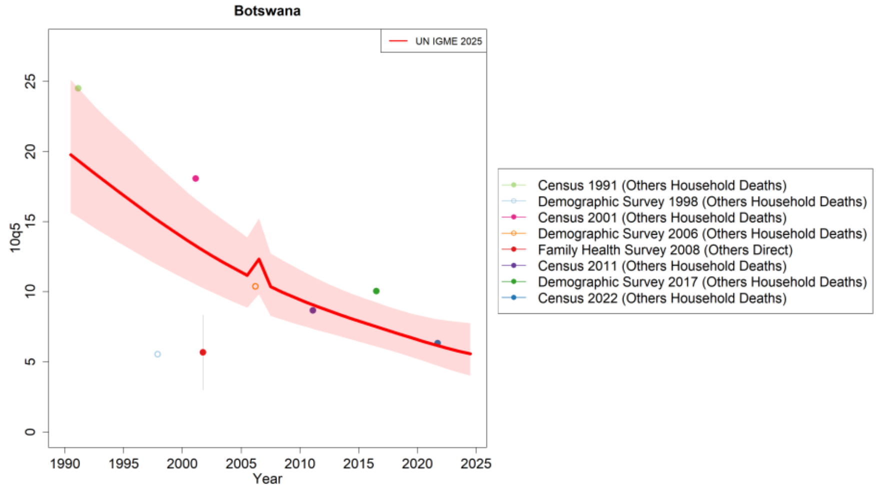

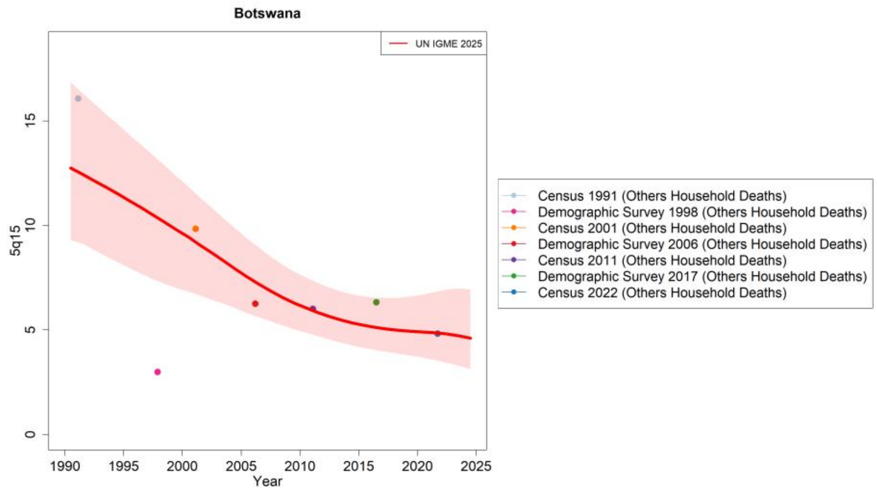
Botswana
UN IGME 2025
Census 1991 (Others Household Deaths)
Demographic Survey 1998 (Others Household Deaths)
Census 2001 (Others Household Deaths)
Demographic Survey 2006 (Others Household Deaths)
Census 2011 (Others Household Deaths)
Demographic Survey 2017 (Others Household Deaths)
Census 2022 (Others Household Deaths)
5q15
Year

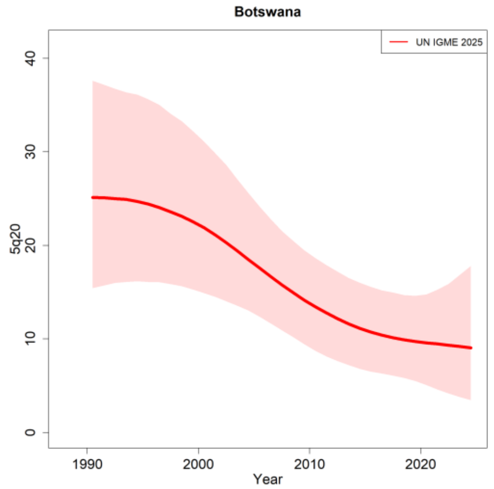
Botswana
UN IGME 2025
5q20
Year

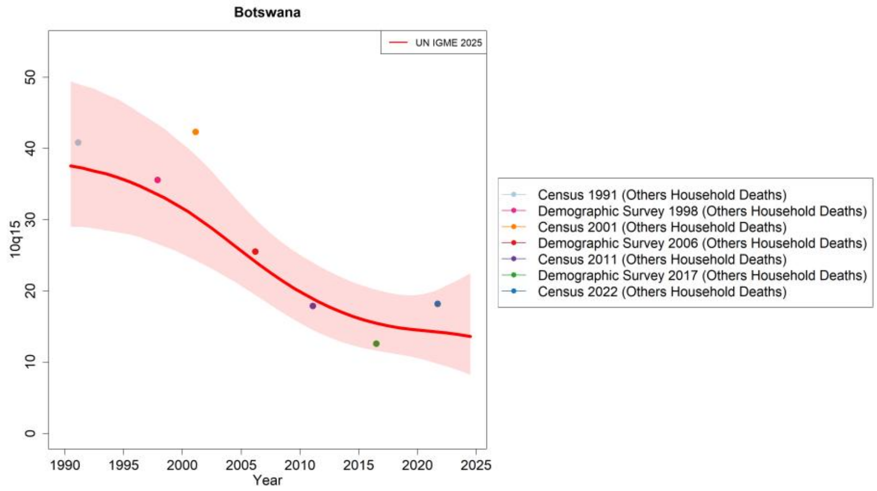
Botswana
UN IGME 2025
Census 1991 (Others Household Deaths)
Demographic Survey 1998 (Others Household Deaths)
Census 2001 (Others Household Deaths)
Demographic Survey 2006 (Others Household Deaths)
Census 2011 (Others Household Deaths)
Demographic Survey 2017 (Others Household Deaths)
Census 2022 (Others Household Deaths)
10q15
Year

Brazil (BRA)

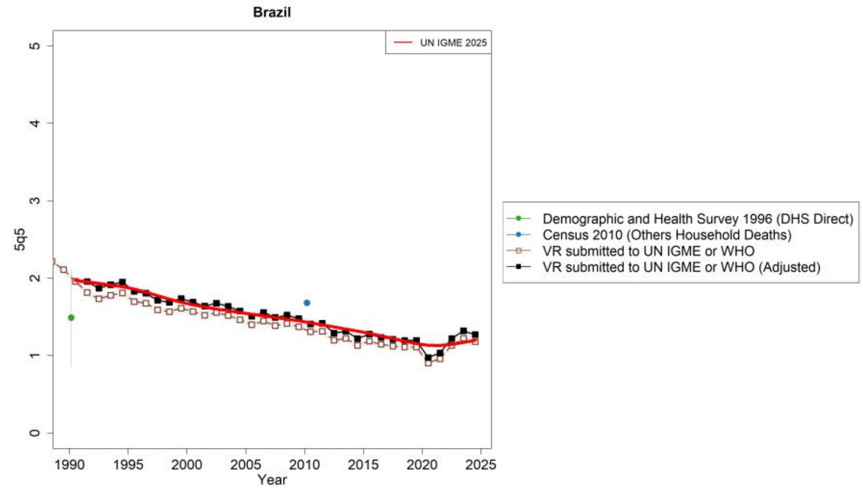
Brazil
UN IGME 2025
5q5
Year
Demographic and Health Survey 1996 (DHS Direct)
Census 2010 (Others Household Deaths)
VR submitted to UN IGME or WHO
VR submitted to UN IGME or WHO (Adjusted)

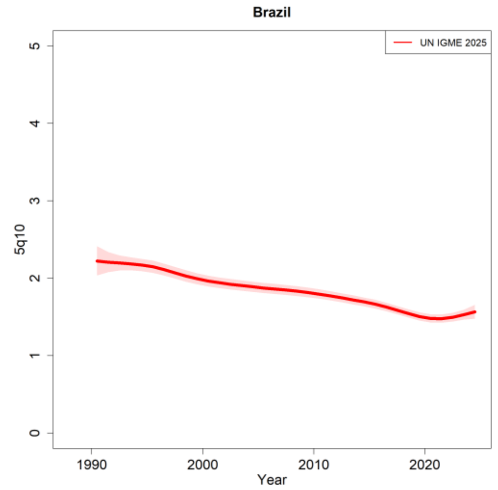
Brazil
UN IGME 2025
5q10
Year

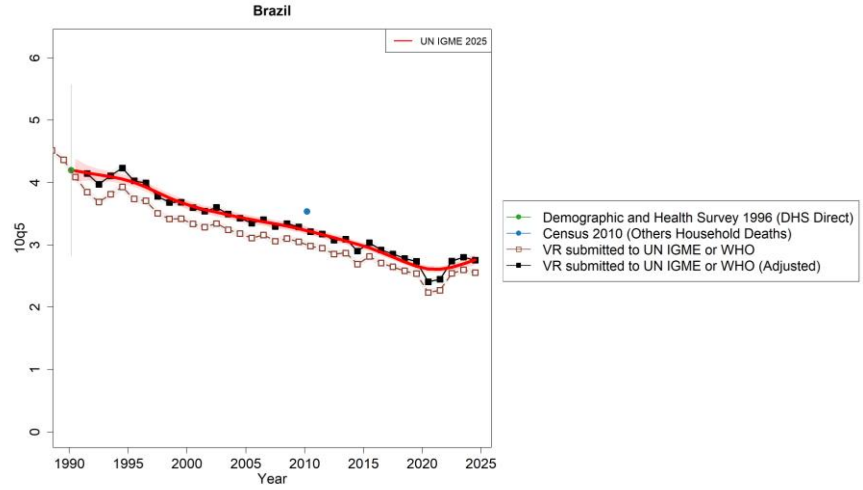
Brazil
UN IGME 2025
10q5
Year
Demographic and Health Survey 1996 (DHS Direct)
Census 2010 (Others Household Deaths)
VR submitted to UN IGME or WHO
VR submitted to UN IGME or WHO (Adjusted)

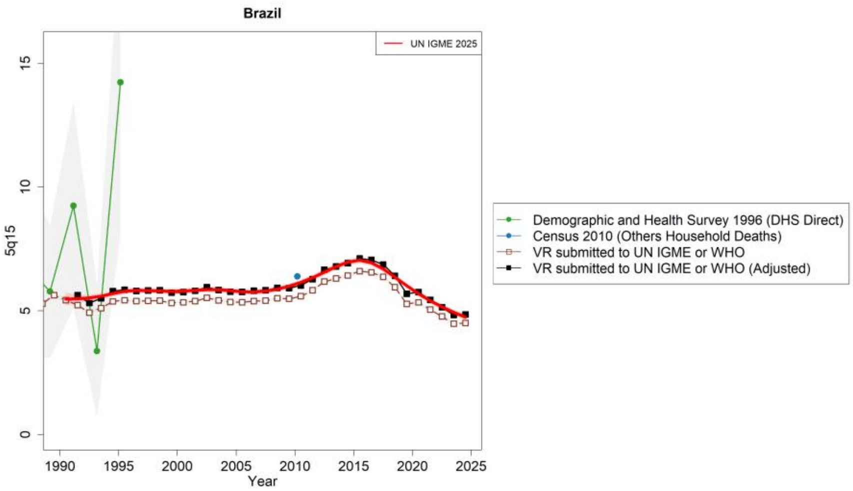
Brazil
UN IGME 2025
5q15
Year
Demographic and Health Survey 1996 (DHS Direct)
Census 2010 (Others Household Deaths)
VR submitted to UN IGME or WHO
VR submitted to UN IGME or WHO (Adjusted)

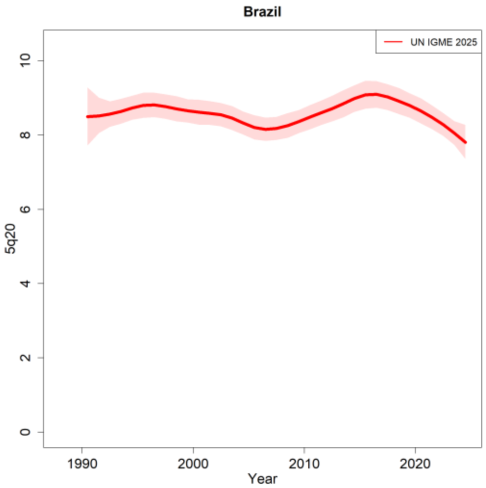
Brazil
UN IGME 2025
5q20
Year

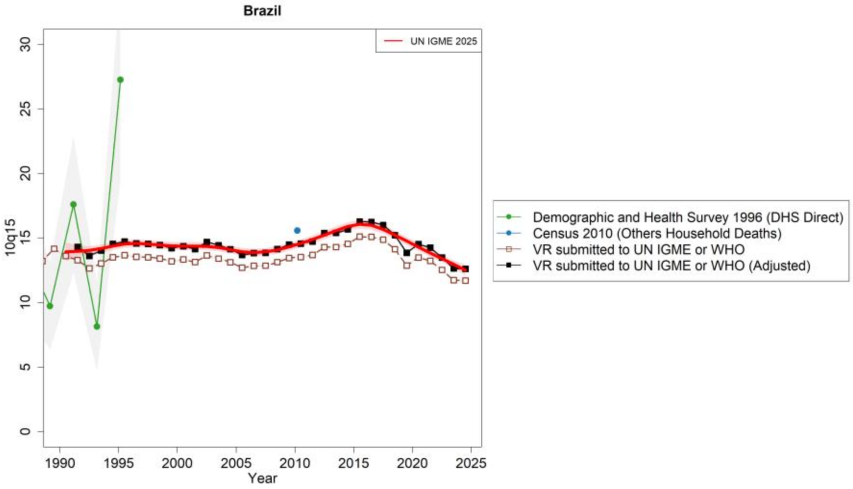
Brazil
UN IGME 2025
10q15
Year
Demographic and Health Survey 1996 (DHS Direct)
Census 2010 (Others Household Deaths)
VR submitted to UN IGME or WHO
VR submitted to UN IGME or WHO (Adjusted)

British Virgin Islands (VGB)

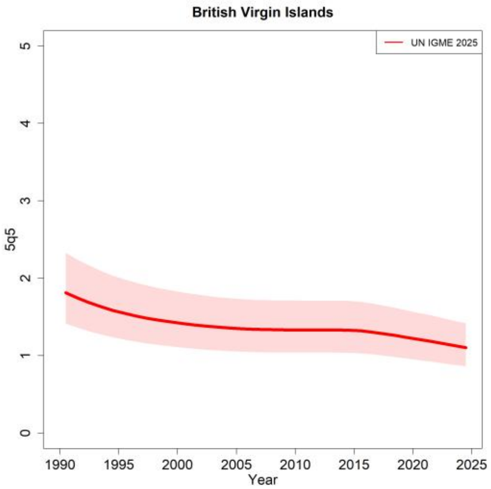

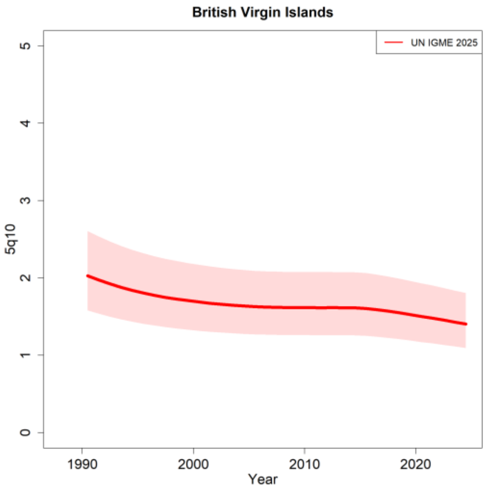

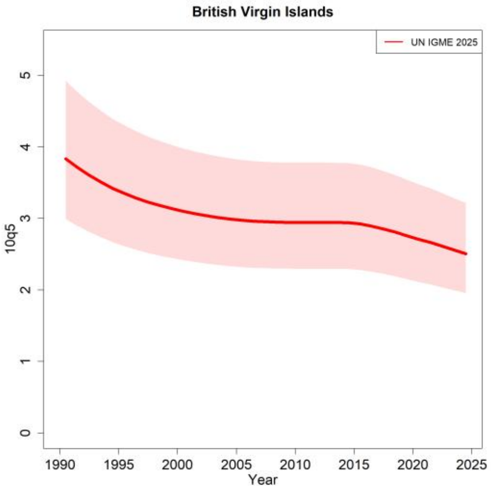

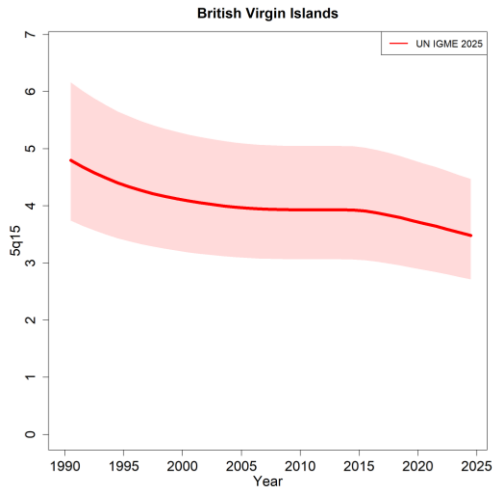
British Virgin Islands
UN IGME 2025
5q15
Year

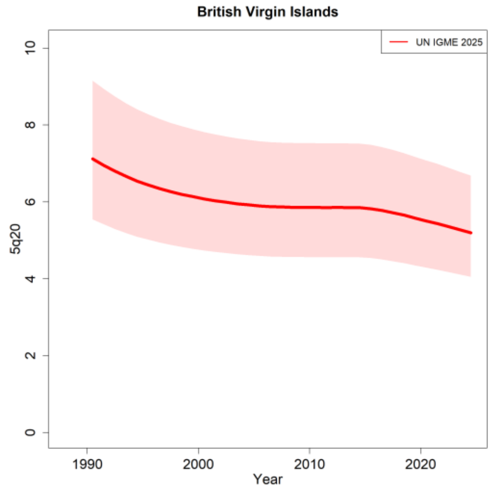
British Virgin Islands
UN IGME 2025
5q20
Year

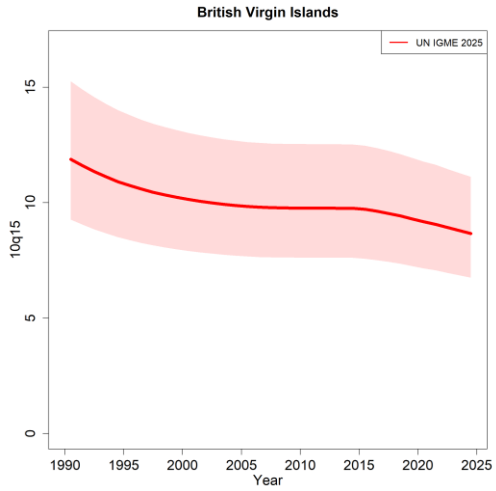
British Virgin Islands
UN IGME 2025
10q15
Year

Brunei Darussalam (BRN)

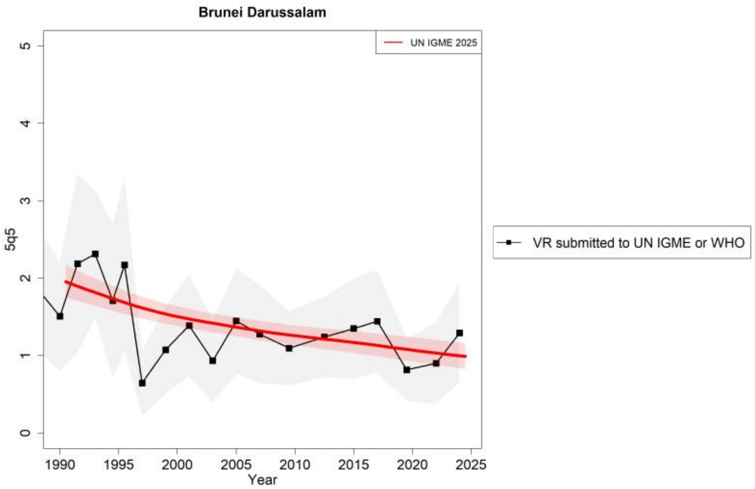

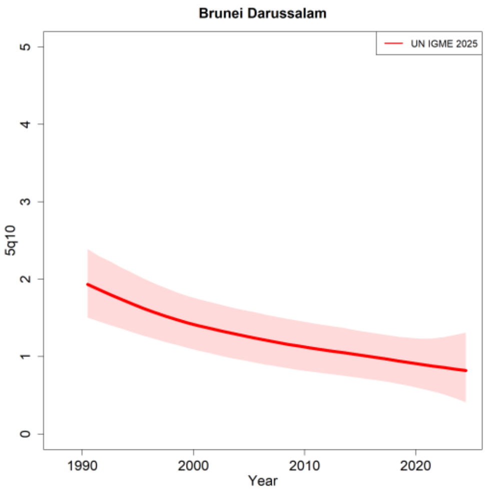

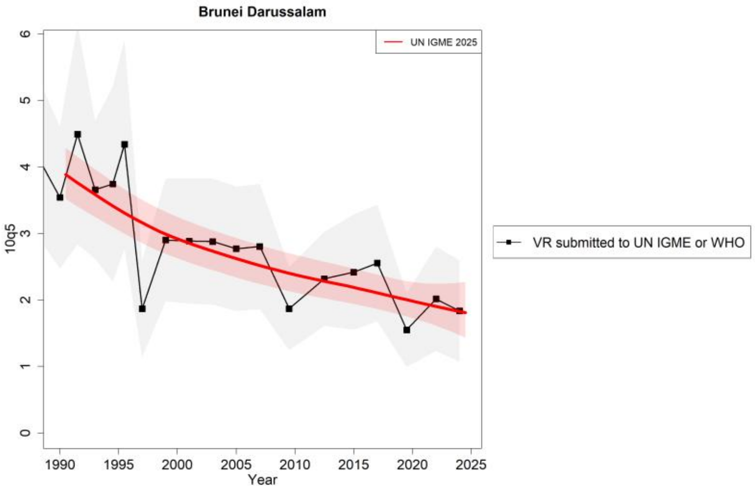

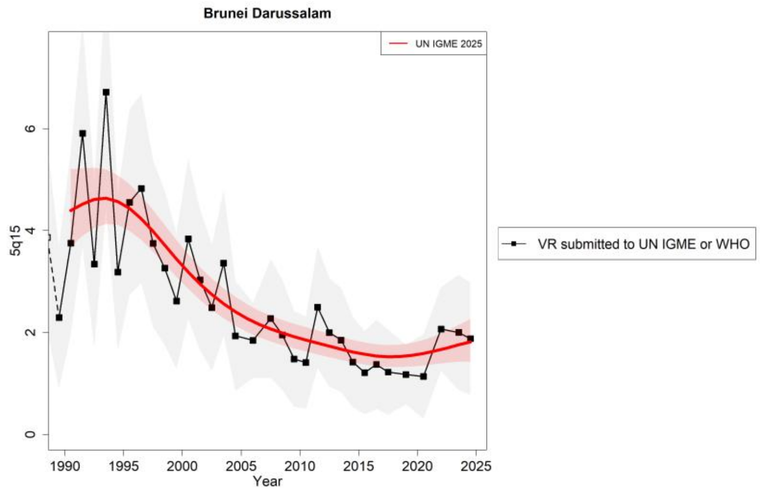
Brunei Darussalam
UN IGME 2025
VR submitted to UN IGME or WHO
5q15
Year

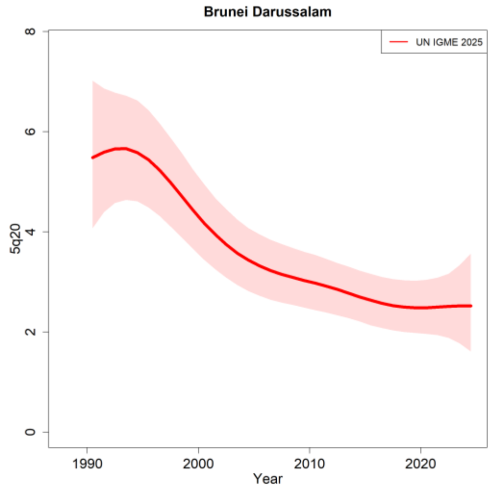
Brunei Darussalam
UN IGME 2025
5q20
Year

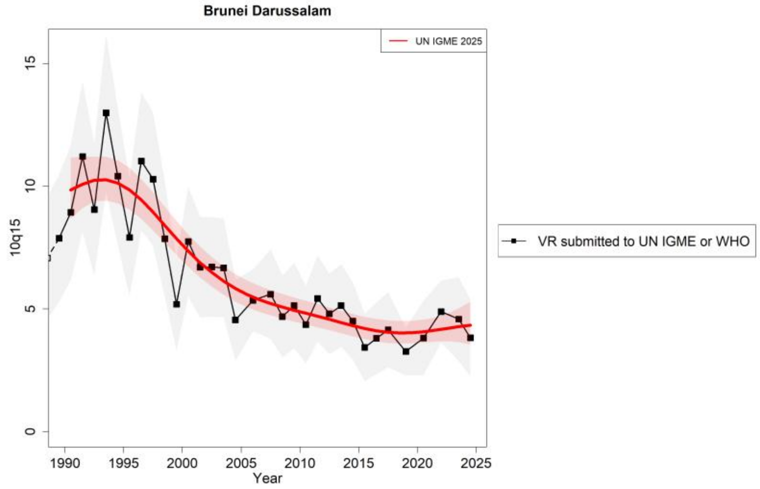
Brunei Darussalam
UN IGME 2025
VR submitted to UN IGME or WHO
10q15
Year

Bulgaria (BGR)

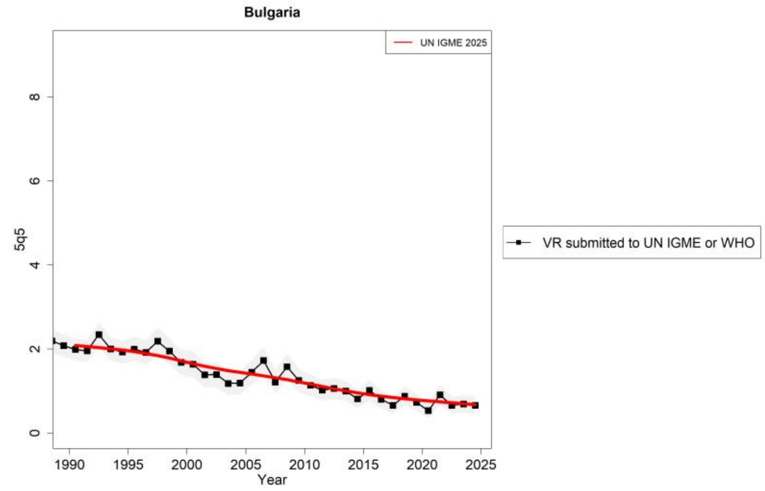

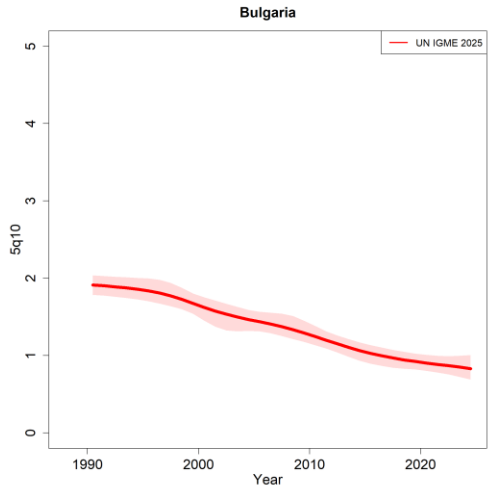

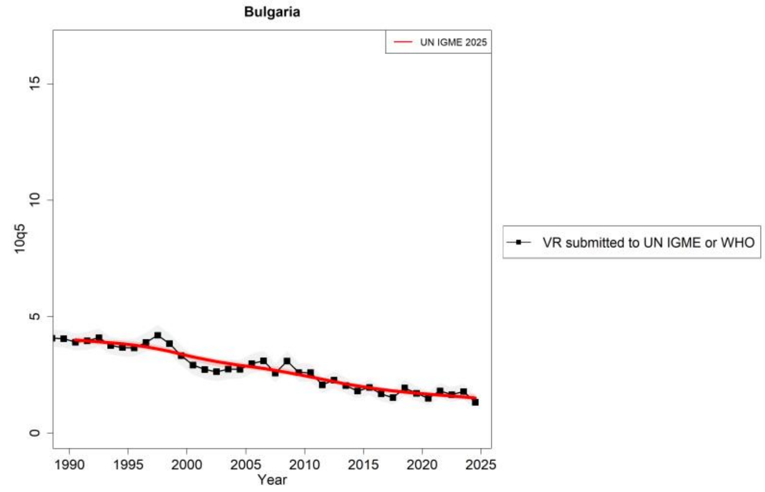

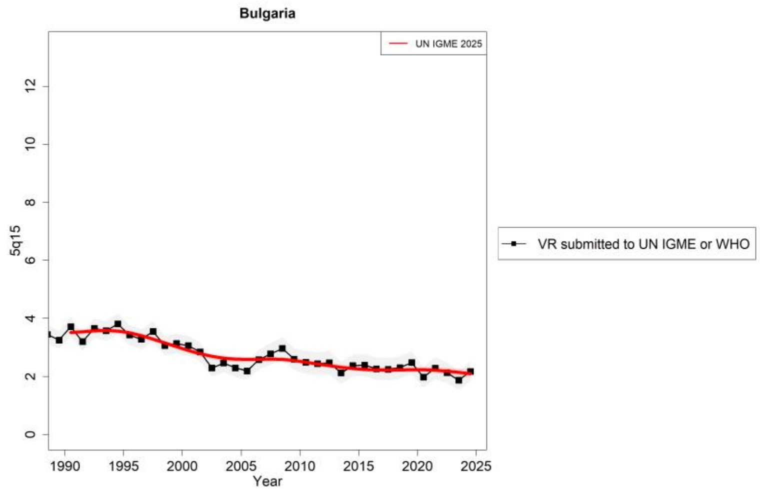
Bulgaria
UN IGME 2025
VR submitted to UN IGME or WHO
5q15
Year

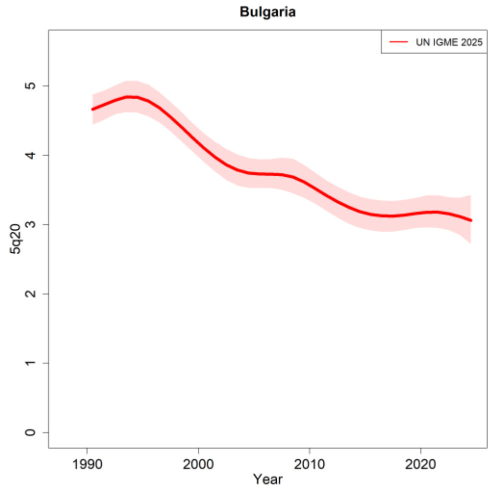
Bulgaria
UN IGME 2025
5q20
Year

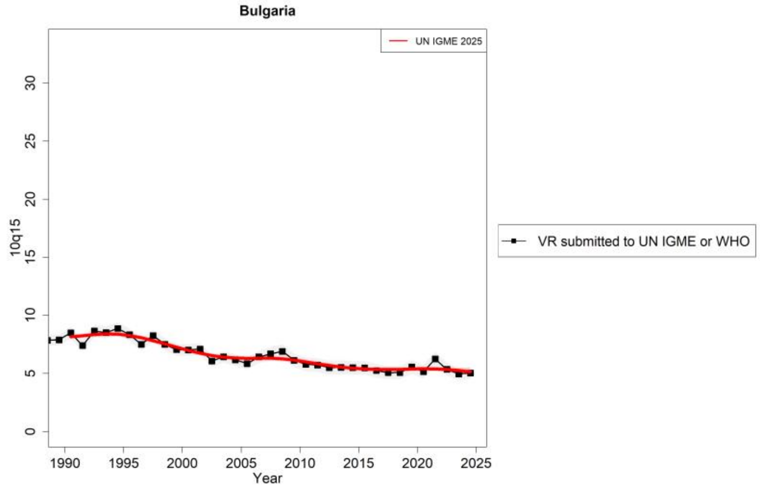
Bulgaria
UN IGME 2025
VR submitted to UN IGME or WHO
10q15
Year

Burkina Faso (BFA)

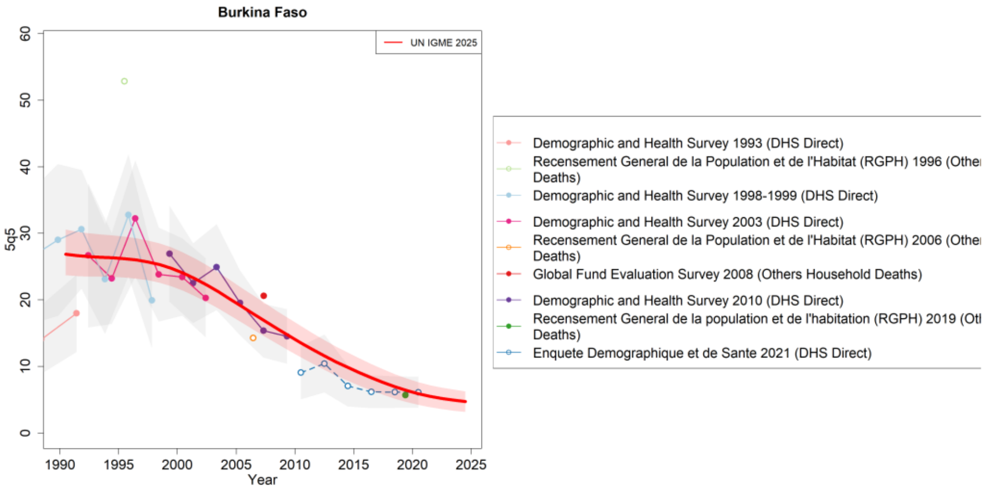

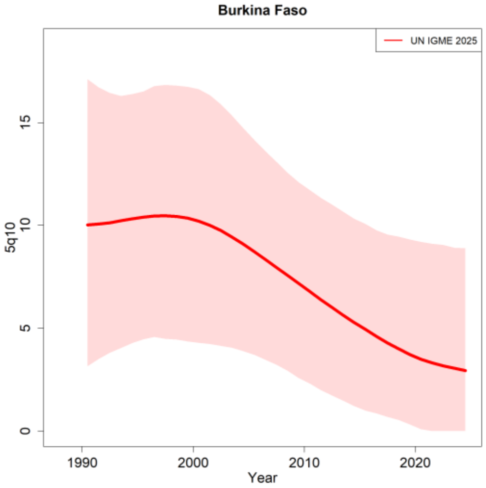

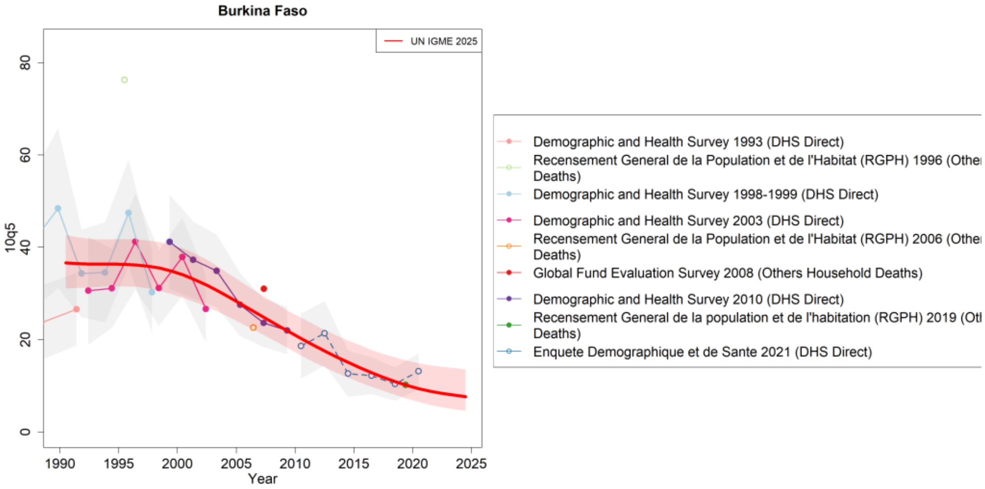

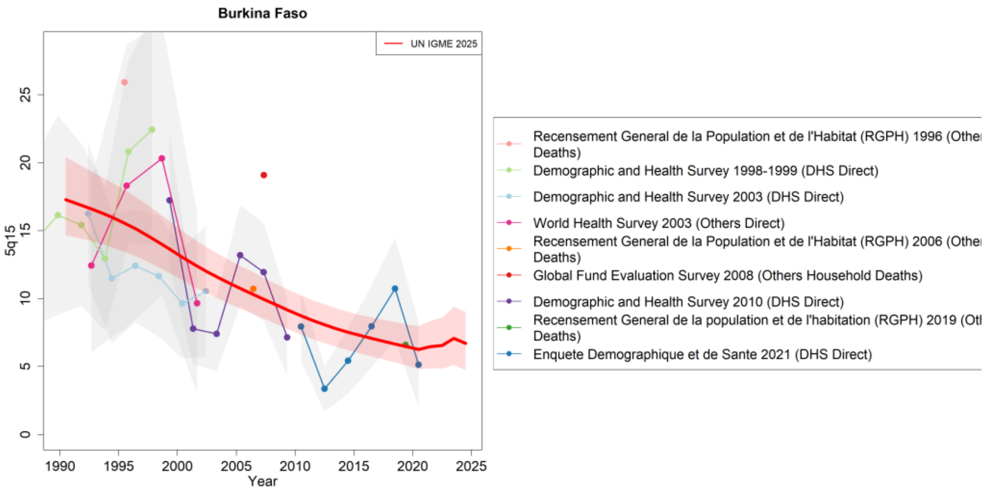
Burkina Faso
UN IGME 2025
5q15
Year
0
5
10
15
20
25
1990
1995
2000
2005
2010
2015
2020
2025
Recensement General de la Population et de l'Habitat (RGPH) 1996 (Othe Deaths)
Demographic and Health Survey 1998-1999 (DHS Direct)
Demographic and Health Survey 2003 (DHS Direct)
World Health Survey 2003 (Others Direct)
Recensement General de la Population et de l'Habitat (RGPH) 2006 (Othe Deaths)
Global Fund Evaluation Survey 2008 (Others Household Deaths)
Demographic and Health Survey 2010 (DHS Direct)
Recensement General de la population et de l'habitation (RGPH) 2019 (Otl Deaths)
Enquete Demographique et de Sante 2021 (DHS Direct)

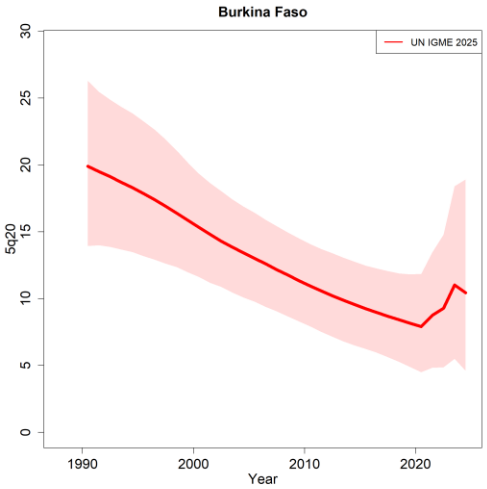
Burkina Faso
UN IGME 2025
5q20
Year
0
5
10
15
20
25
30
1990
2000
2010
2020

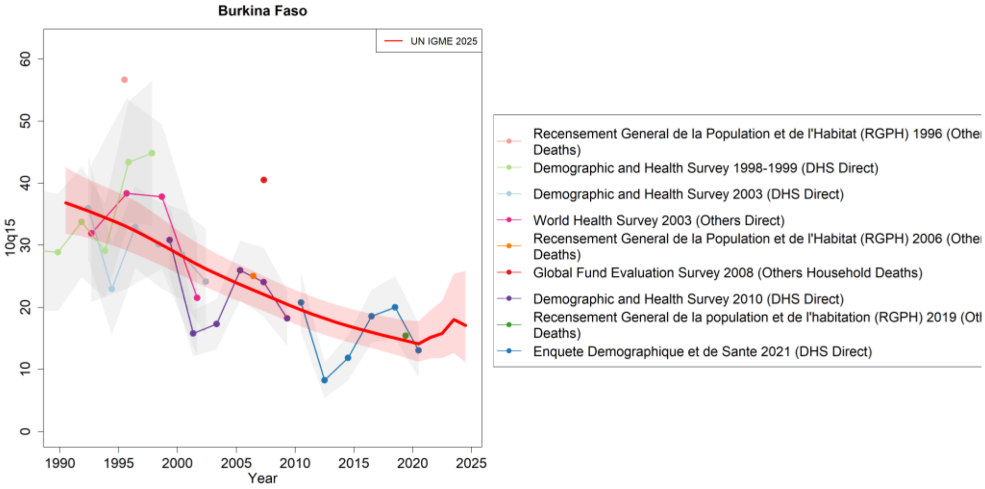
Burkina Faso
UN IGME 2025
10q15
Year
0
10
20
30
40
50
60
1990
1995
2000
2005
2010
2015
2020
2025
Recensement General de la Population et de l'Habitat (RGPH) 1996 (Othe Deaths)
Demographic and Health Survey 1998-1999 (DHS Direct)
Demographic and Health Survey 2003 (DHS Direct)
World Health Survey 2003 (Others Direct)
Recensement General de la Population et de l'Habitat (RGPH) 2006 (Othe Deaths)
Global Fund Evaluation Survey 2008 (Others Household Deaths)
Demographic and Health Survey 2010 (DHS Direct)
Recensement General de la population et de l'habitation (RGPH) 2019 (Otl Deaths)
Enquete Demographique et de Sante 2021 (DHS Direct)

Burundi (BDI)

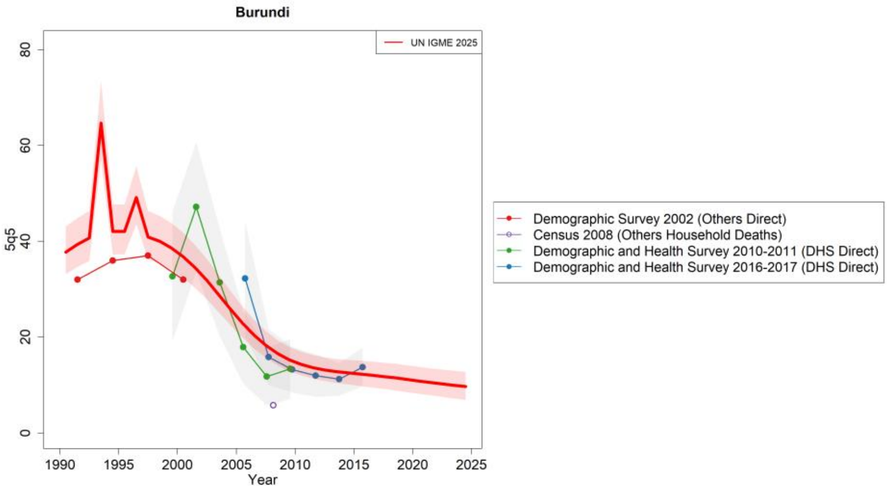
Burundi
UN IGME 2025
5q5
0
20
40
60
80
1990
1995
2000
2005
2010
2015
2020
2025
Year
Demographic Survey 2002 (Others Direct)
Census 2008 (Others Household Deaths)
Demographic and Health Survey 2010-2011 (DHS Direct)
Demographic and Health Survey 2016-2017 (DHS Direct)

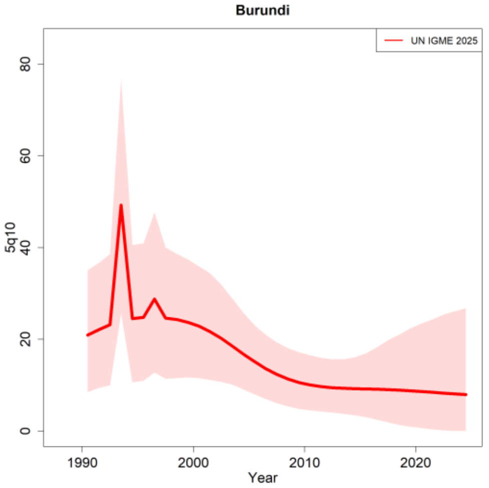
Burundi
UN IGME 2025
5q10
0
20
40
60
80
1990
2000
2010
2020
Year

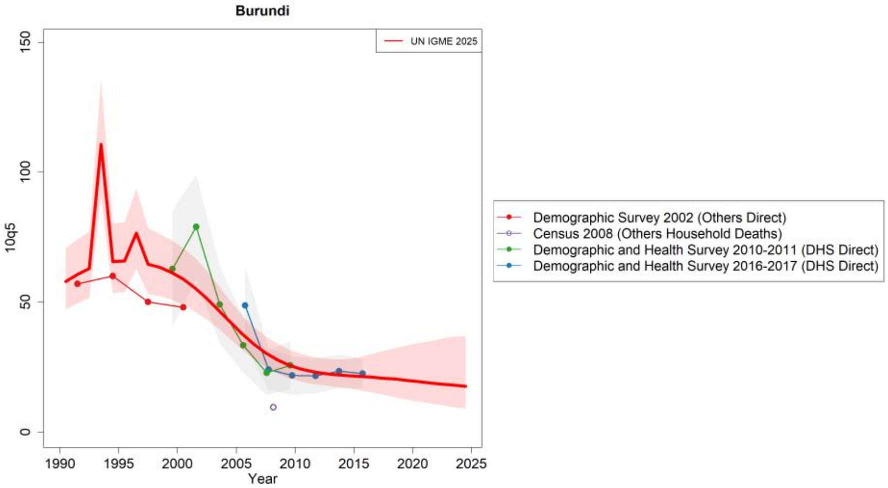
Burundi
UN IGME 2025
10q5
0
50
100
150
1990
1995
2000
2005
2010
2015
2020
2025
Year
Demographic Survey 2002 (Others Direct)
Census 2008 (Others Household Deaths)
Demographic and Health Survey 2010-2011 (DHS Direct)
Demographic and Health Survey 2016-2017 (DHS Direct)

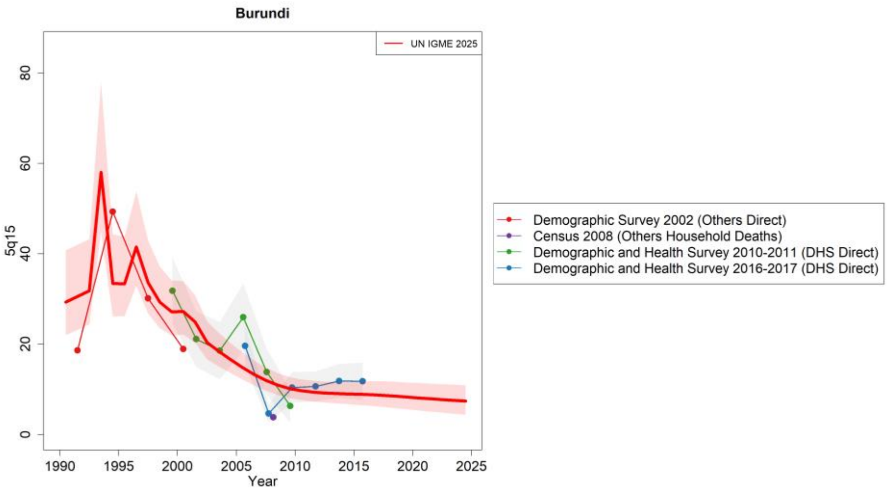
Burundi
UN IGME 2025
5q15
Year
Demographic Survey 2002 (Others Direct)
Census 2008 (Others Household Deaths)
Demographic and Health Survey 2010-2011 (DHS Direct)
Demographic and Health Survey 2016-2017 (DHS Direct)

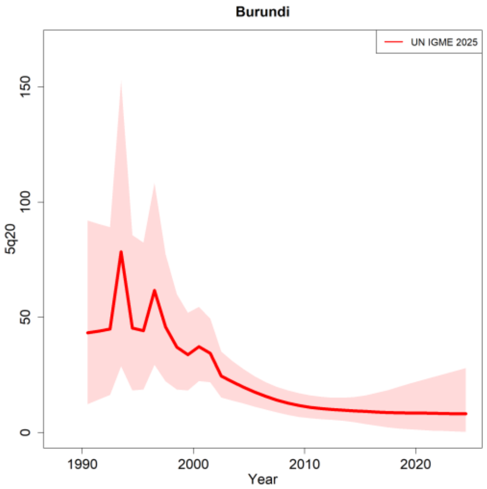
Burundi
UN IGME 2025
5q20
Year

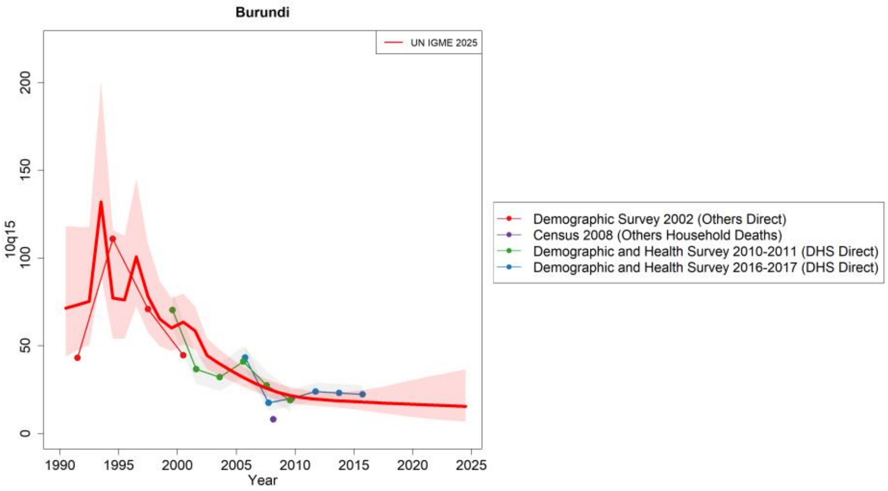
Burundi
UN IGME 2025
10q15
Year
Demographic Survey 2002 (Others Direct)
Census 2008 (Others Household Deaths)
Demographic and Health Survey 2010-2011 (DHS Direct)
Demographic and Health Survey 2016-2017 (DHS Direct)

Cabo Verde (CPV)

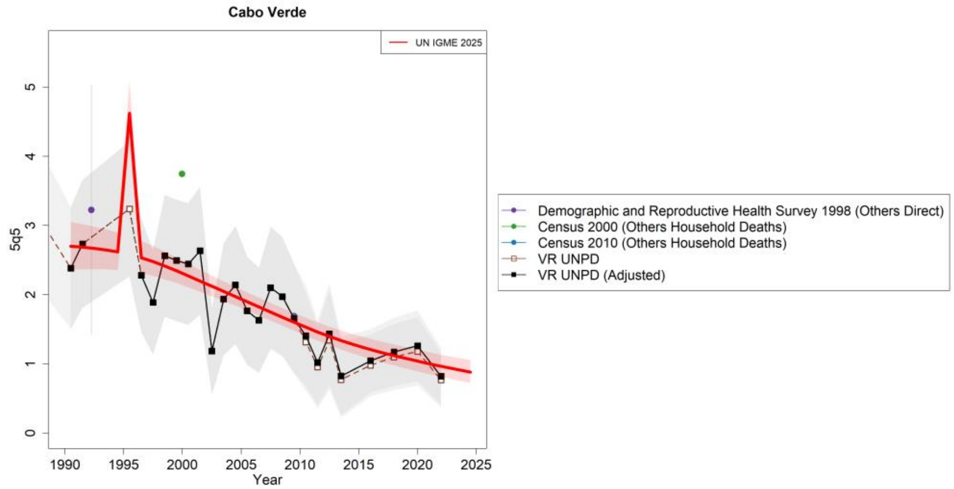

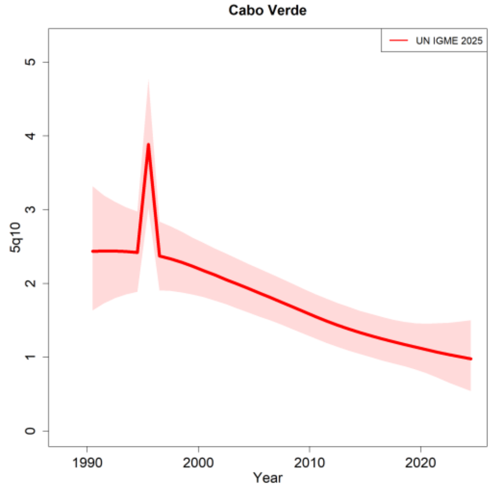

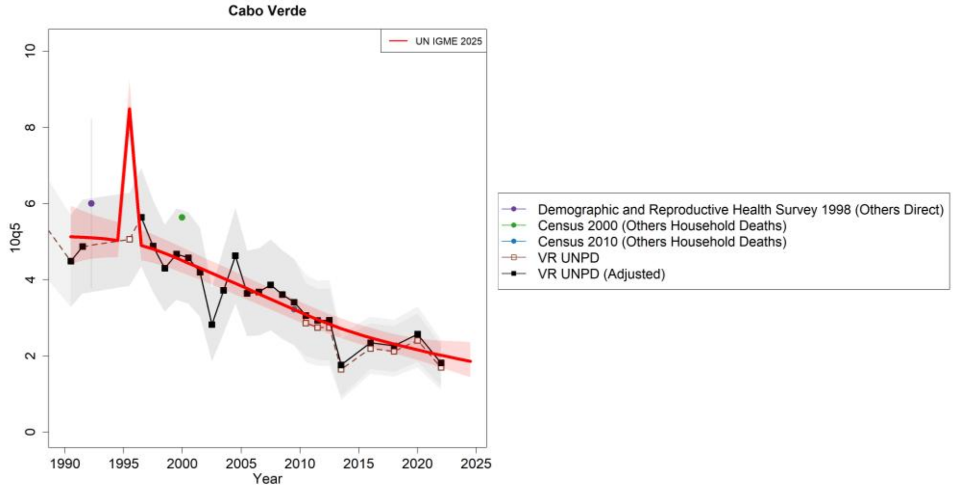

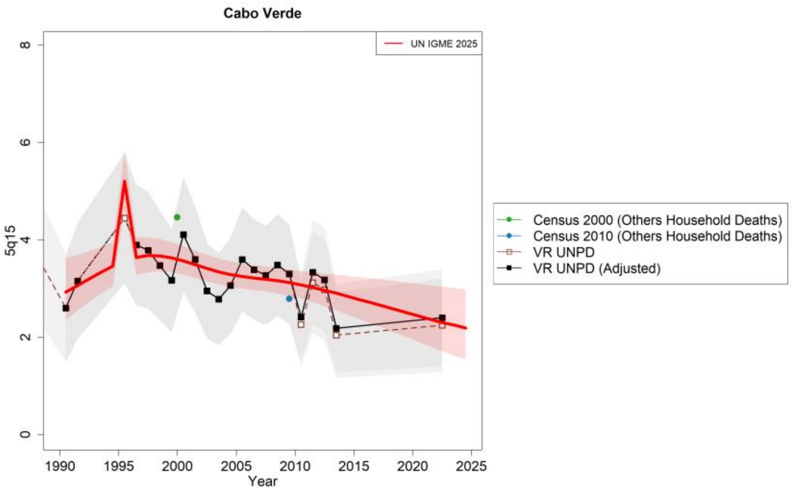
Cabo Verde
UN IGME 2025
5q15
Year
Census 2000 (Others Household Deaths)
Census 2010 (Others Household Deaths)
VR UNPD
VR UNPD (Adjusted)

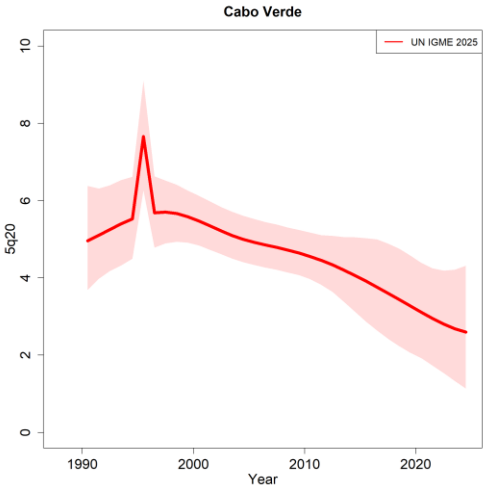
Cabo Verde
UN IGME 2025
5q20
Year

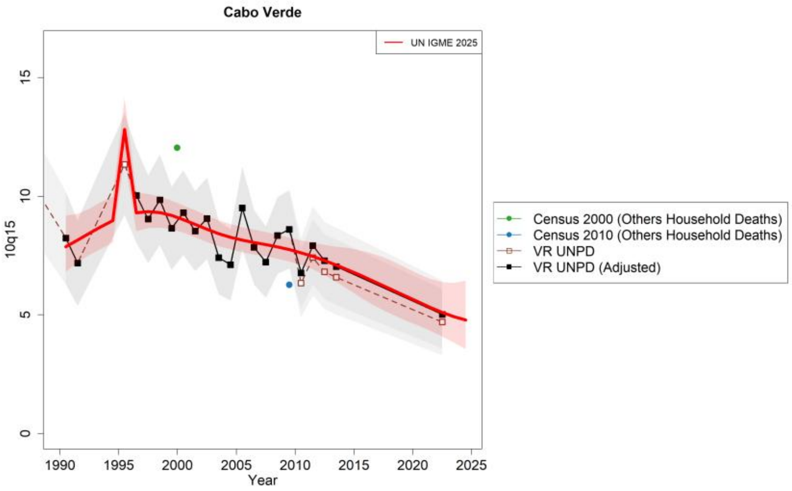
Cabo Verde
UN IGME 2025
10q15
Year
Census 2000 (Others Household Deaths)
Census 2010 (Others Household Deaths)
VR UNPD
VR UNPD (Adjusted)

Cambodia (KHM)

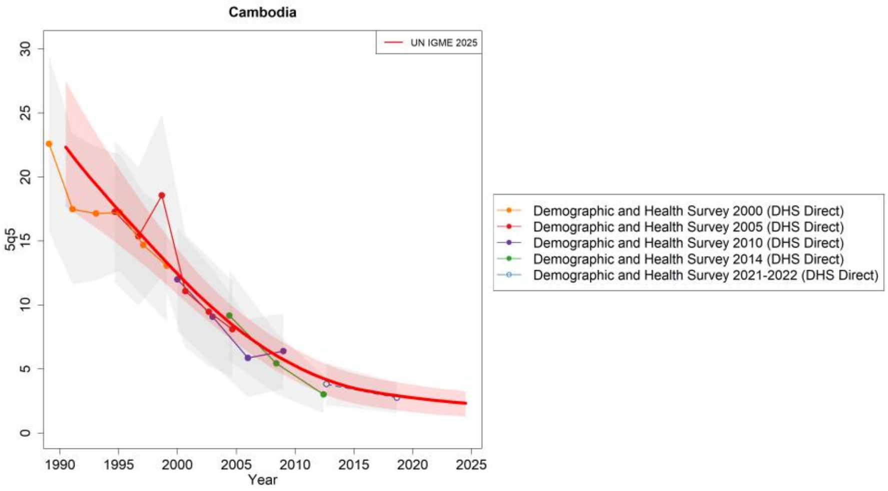

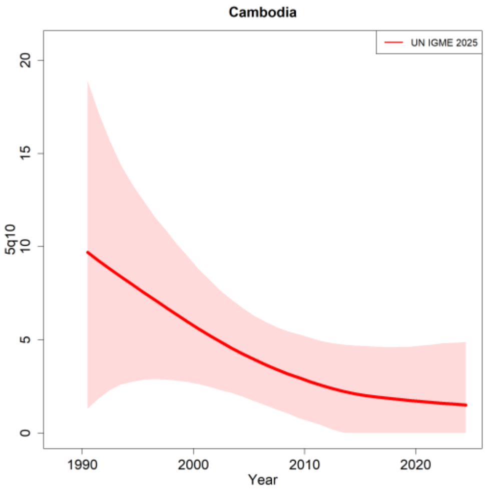

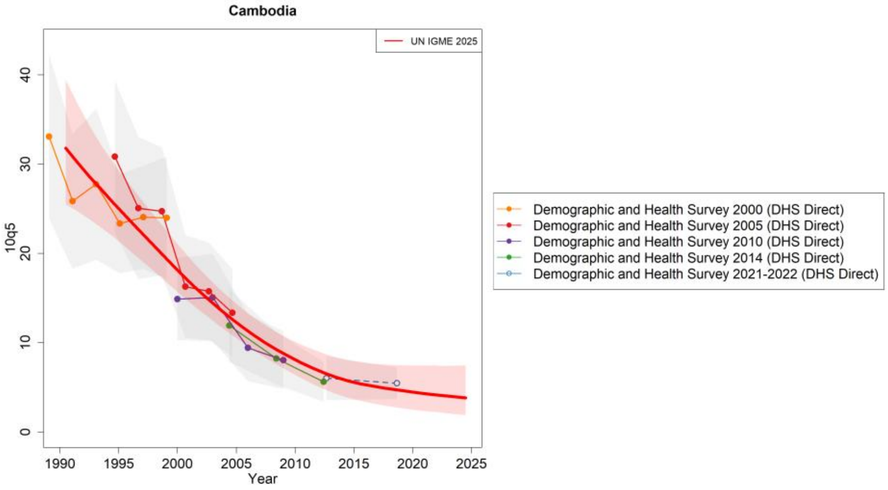

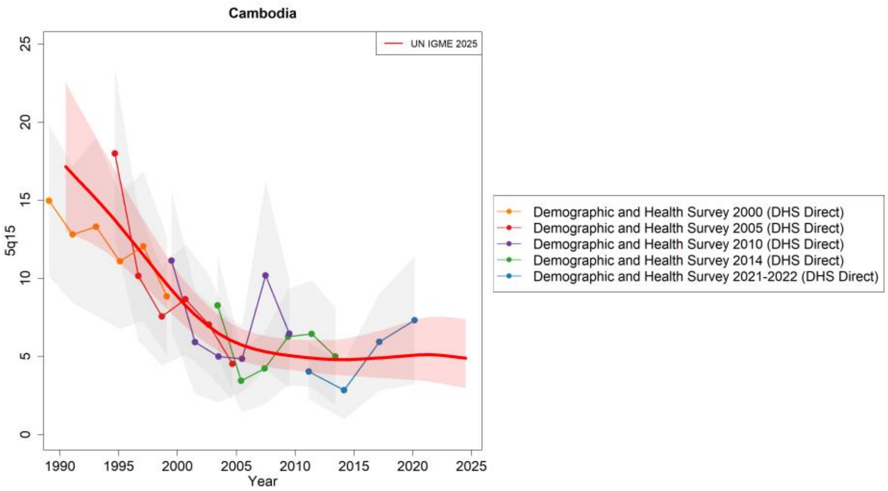
Cambodia
UN IGME 2025
5q15
Year
Demographic and Health Survey 2000 (DHS Direct)
Demographic and Health Survey 2005 (DHS Direct)
Demographic and Health Survey 2010 (DHS Direct)
Demographic and Health Survey 2014 (DHS Direct)
Demographic and Health Survey 2021-2022 (DHS Direct)

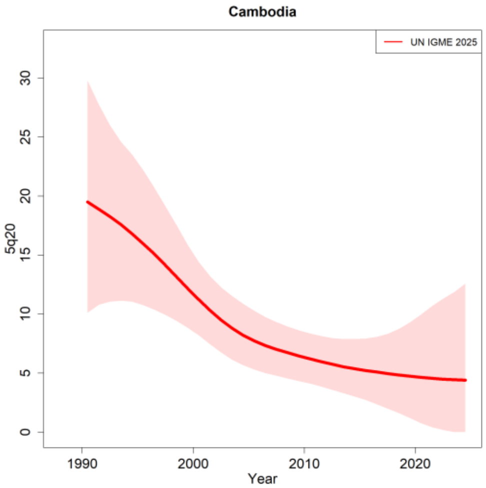
Cambodia
UN IGME 2025
5q20
Year

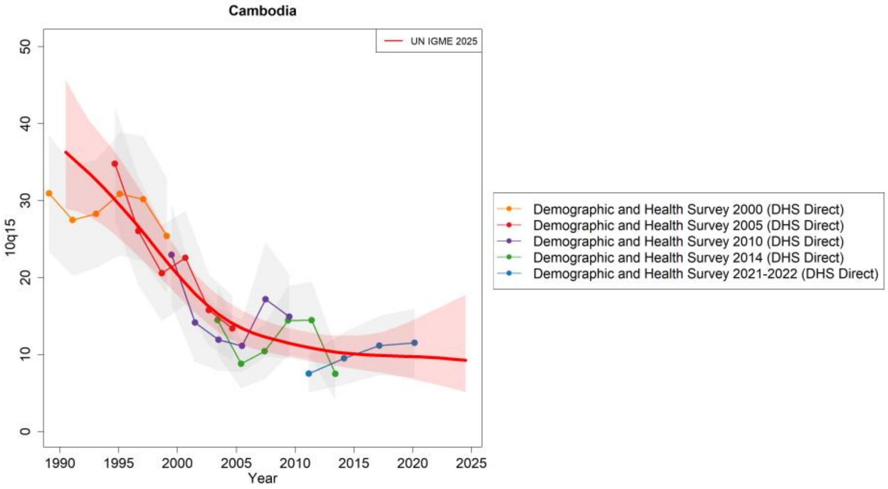
Cambodia
UN IGME 2025
10q15
Year
Demographic and Health Survey 2000 (DHS Direct)
Demographic and Health Survey 2005 (DHS Direct)
Demographic and Health Survey 2010 (DHS Direct)
Demographic and Health Survey 2014 (DHS Direct)
Demographic and Health Survey 2021-2022 (DHS Direct)

Cameroon (CMR)

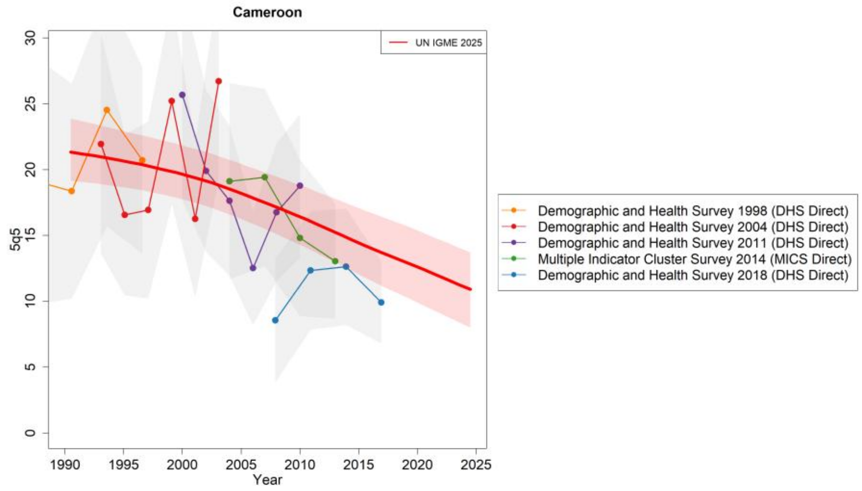

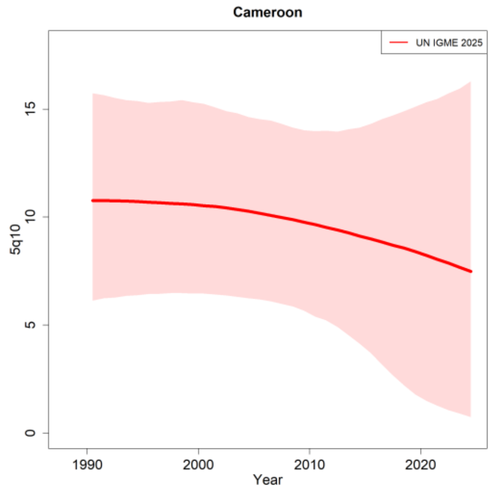

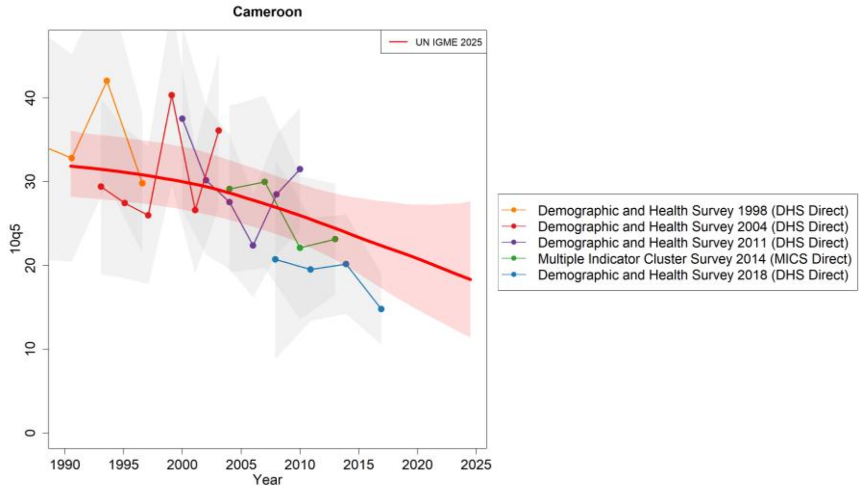

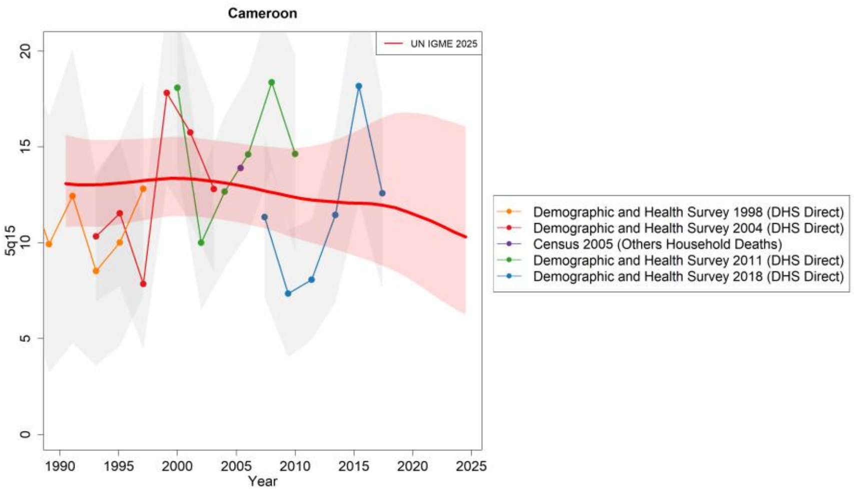
Cameroon
UN IGME 2025
5q15
Year
Demographic and Health Survey 1998 (DHS Direct)
Demographic and Health Survey 2004 (DHS Direct)
Census 2005 (Others Household Deaths)
Demographic and Health Survey 2011 (DHS Direct)
Demographic and Health Survey 2018 (DHS Direct)

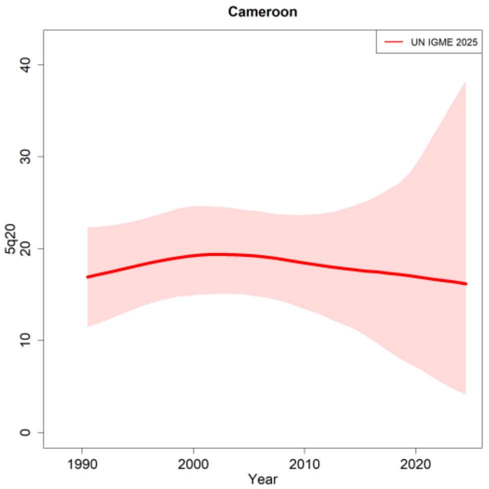
Cameroon
UN IGME 2025
5q20
Year

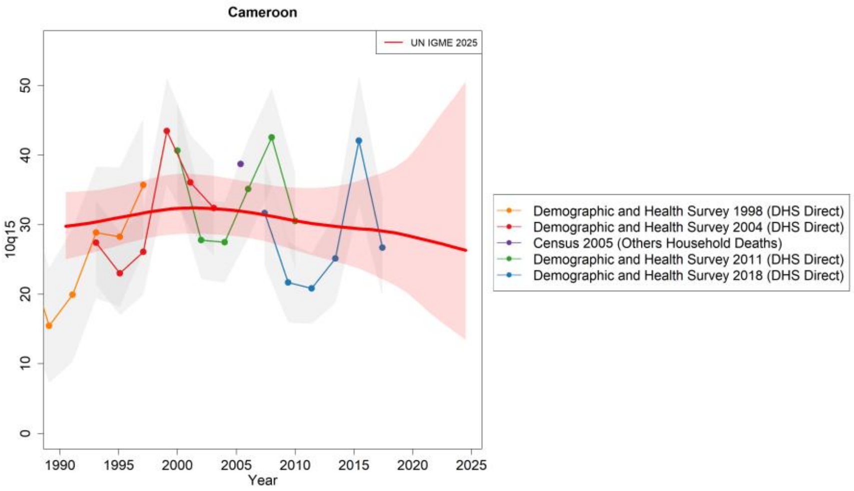
Cameroon
UN IGME 2025
10q15
Year
Demographic and Health Survey 1998 (DHS Direct)
Demographic and Health Survey 2004 (DHS Direct)
Census 2005 (Others Household Deaths)
Demographic and Health Survey 2011 (DHS Direct)
Demographic and Health Survey 2018 (DHS Direct)

Canada (CAN)

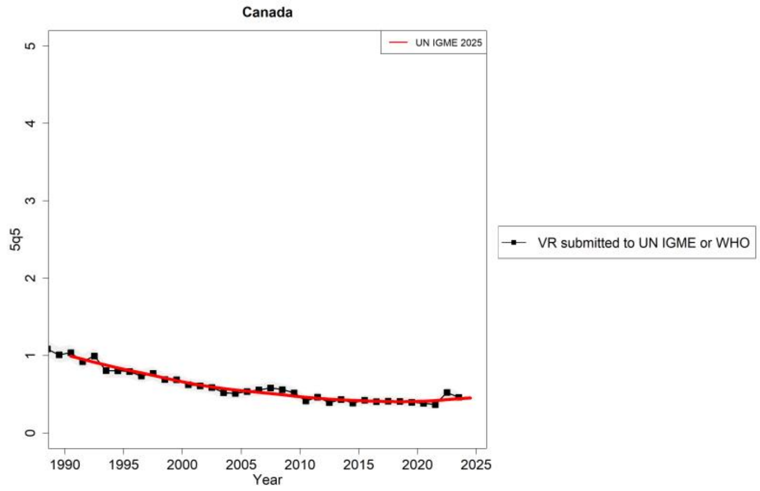
Canada
UN IGME 2025
VR submitted to UN IGME or WHO
5q5
Year

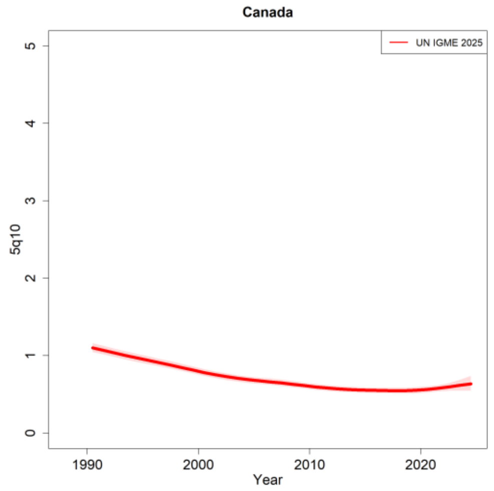
Canada
UN IGME 2025
5q10
Year

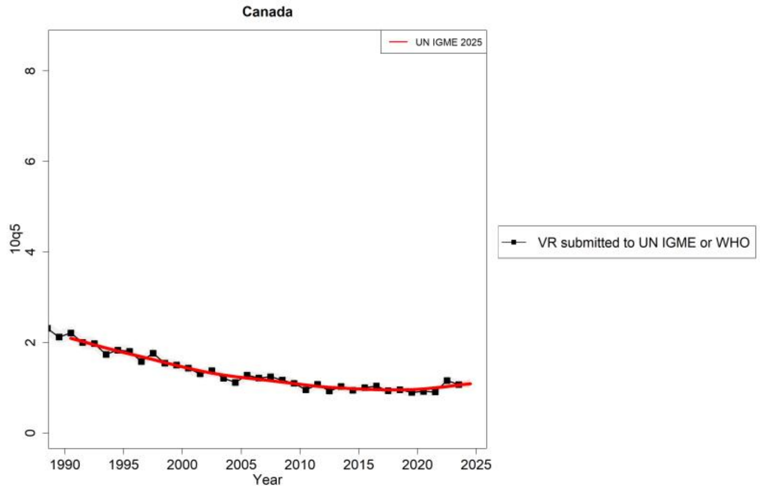
Canada
UN IGME 2025
VR submitted to UN IGME or WHO
10q5
Year

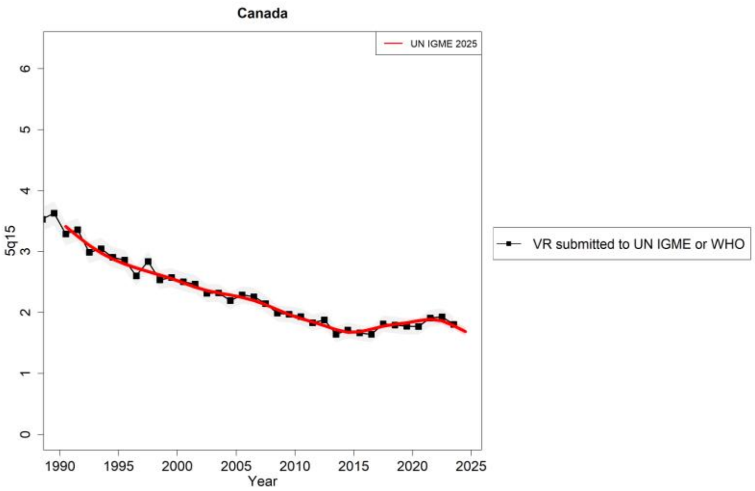
Canada
UN IGME 2025
VR submitted to UN IGME or WHO
5q15
Year
0
1
2
3
4
5
6
1990
1995
2000
2005
2010
2015
2020
2025

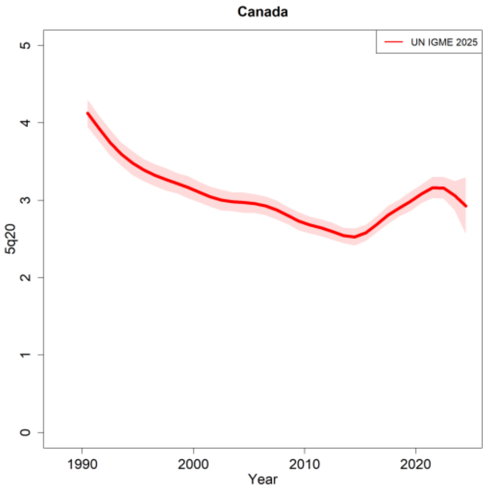
Canada
UN IGME 2025
5q20
Year
0
1
2
3
4
5
1990
2000
2010
2020

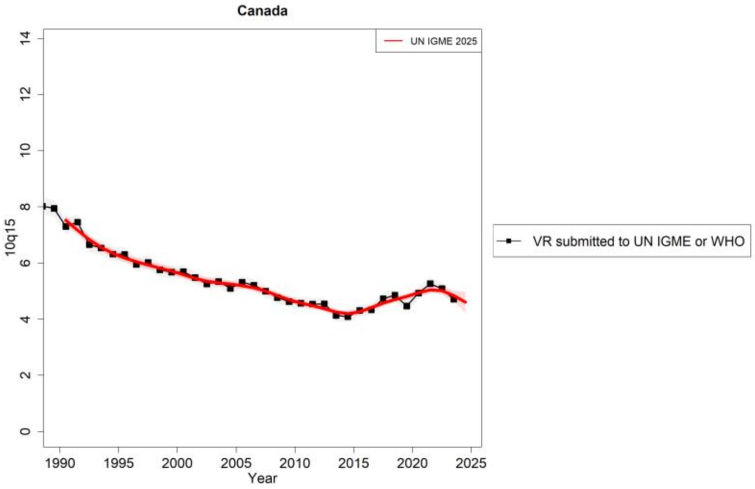
Canada
UN IGME 2025
VR submitted to UN IGME or WHO
10q15
Year
0
2
4
6
8
10
12
14
1990
1995
2000
2005
2010
2015
2020
2025

Central African Republic (CAF)

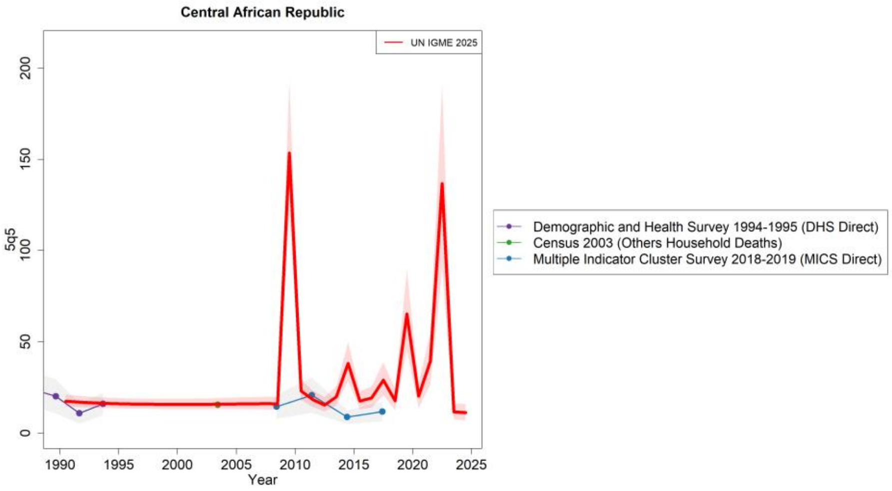

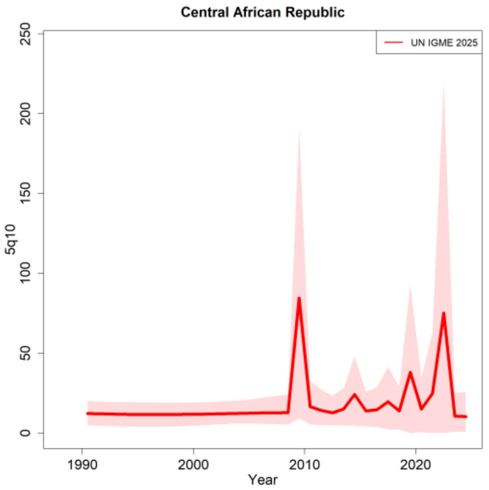

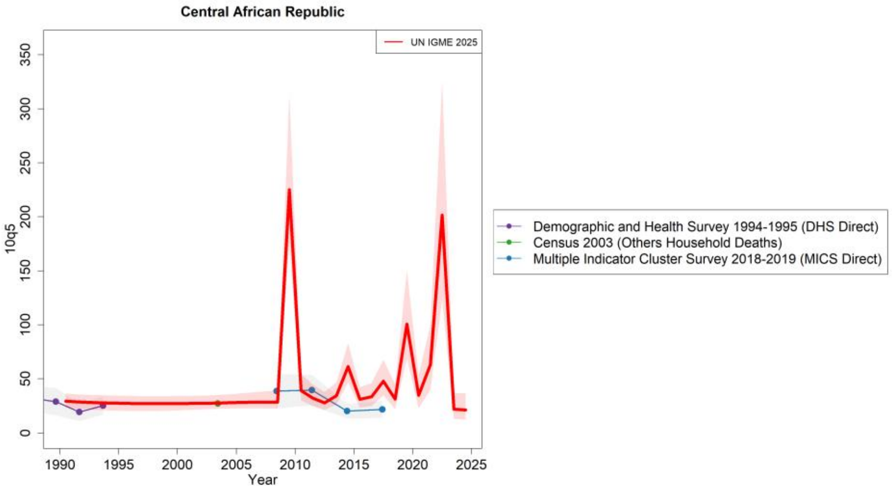

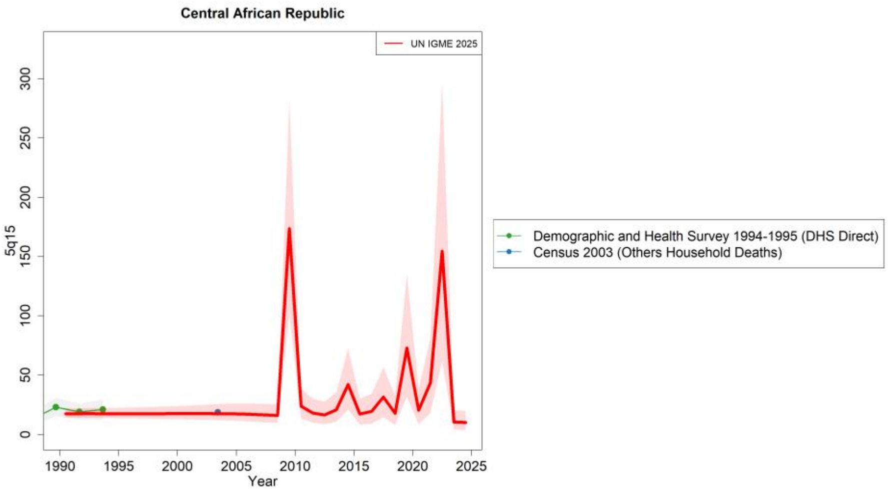
Central African Republic
UN IGME 2025
5q15
Year
Demographic and Health Survey 1994-1995 (DHS Direct)
Census 2003 (Others Household Deaths)

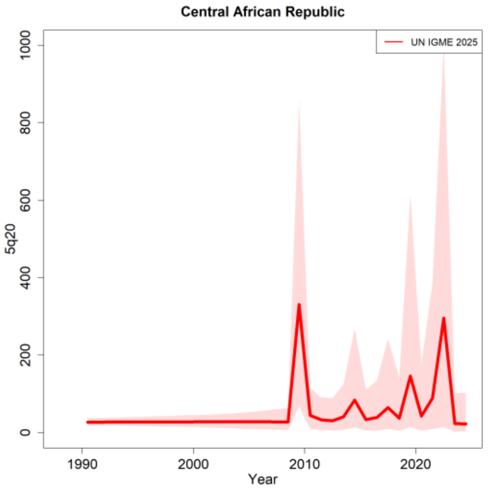
Central African Republic
UN IGME 2025
5q20
Year

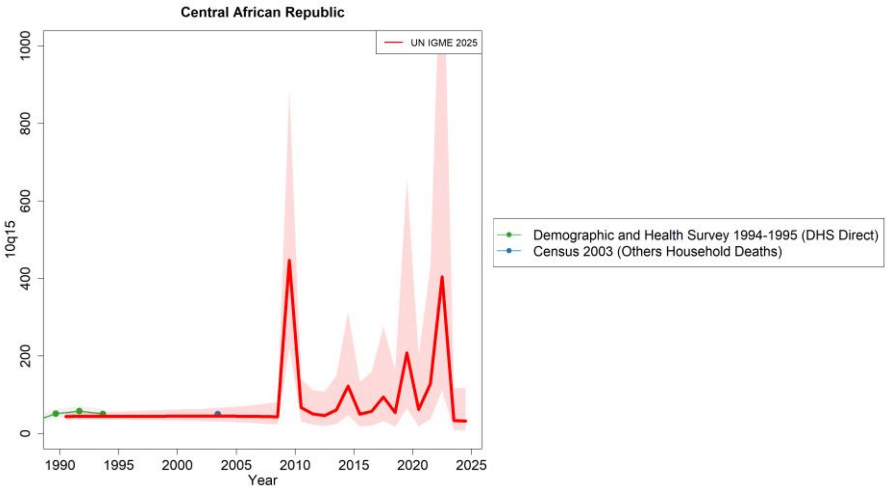
Central African Republic
UN IGME 2025
10q15
Year
Demographic and Health Survey 1994-1995 (DHS Direct)
Census 2003 (Others Household Deaths)

Chad (TCD)

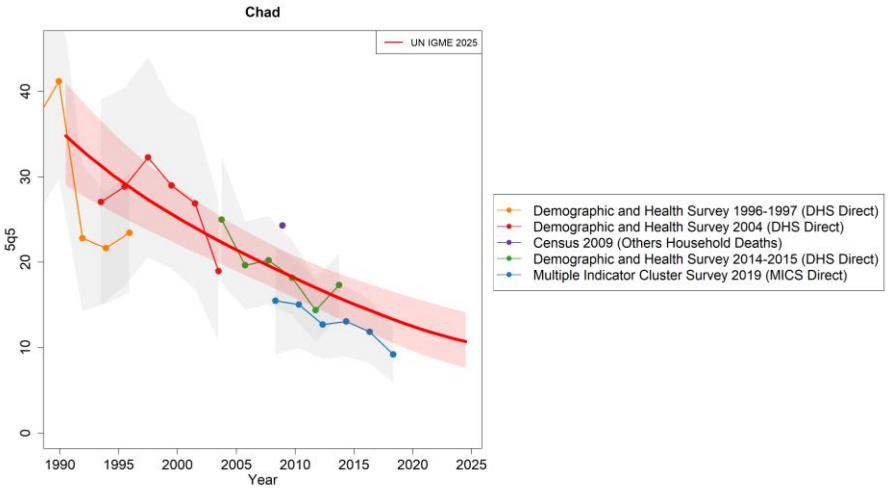
Chad
UN IGME 2025
Demographic and Health Survey 1996-1997 (DHS Direct)
Demographic and Health Survey 2004 (DHS Direct)
Census 2009 (Others Household Deaths)
Demographic and Health Survey 2014-2015 (DHS Direct)
Multiple Indicator Cluster Survey 2019 (MICS Direct)
5q5
Year

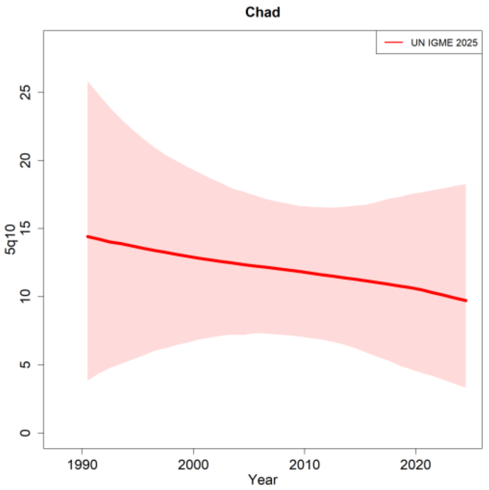
Chad
UN IGME 2025
5q10
Year

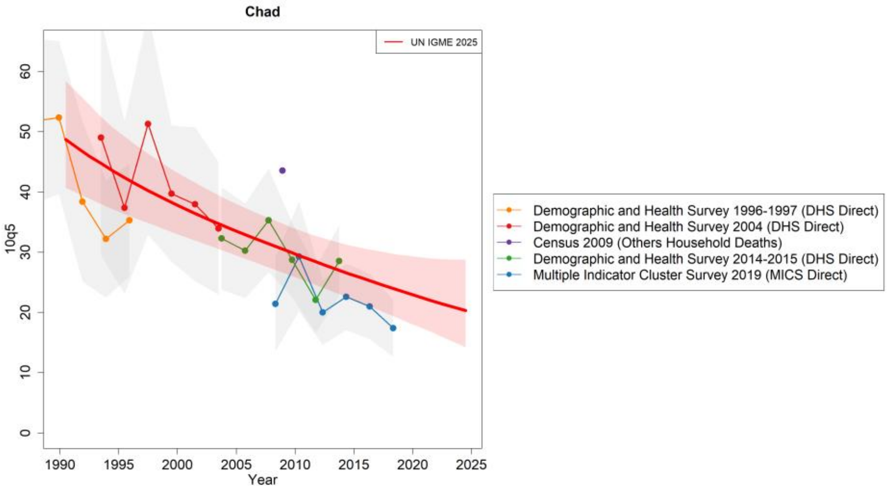
Chad
UN IGME 2025
Demographic and Health Survey 1996-1997 (DHS Direct)
Demographic and Health Survey 2004 (DHS Direct)
Census 2009 (Others Household Deaths)
Demographic and Health Survey 2014-2015 (DHS Direct)
Multiple Indicator Cluster Survey 2019 (MICS Direct)
10q5
Year

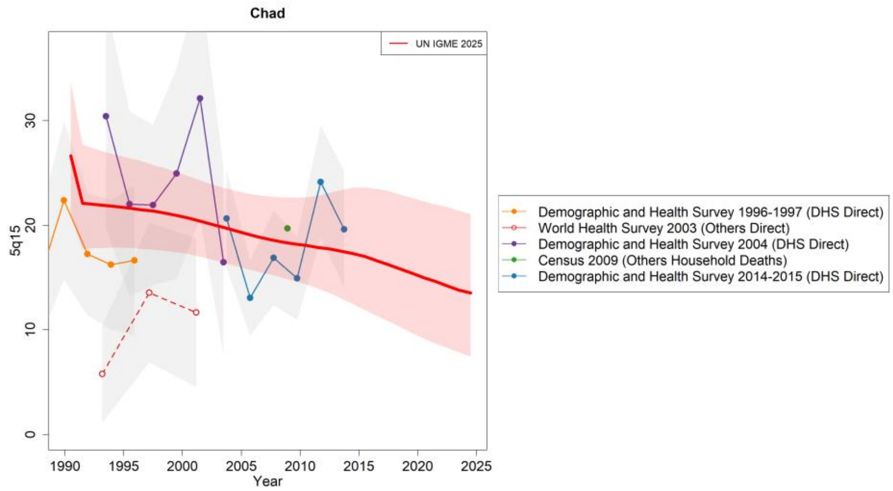
Chad
UN IGME 2025
5q15
Year
Demographic and Health Survey 1996-1997 (DHS Direct)
World Health Survey 2003 (Others Direct)
Demographic and Health Survey 2004 (DHS Direct)
Census 2009 (Others Household Deaths)
Demographic and Health Survey 2014-2015 (DHS Direct)

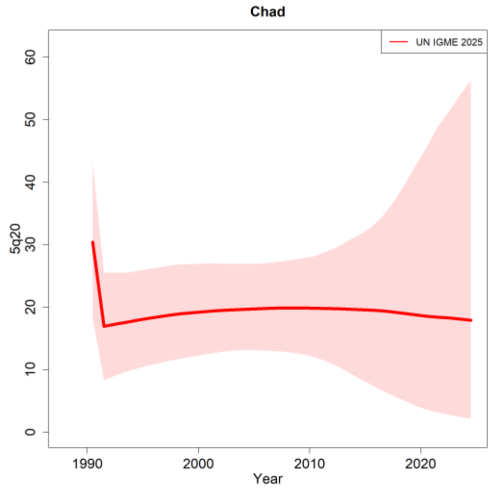
Chad
UN IGME 2025
5q20
Year

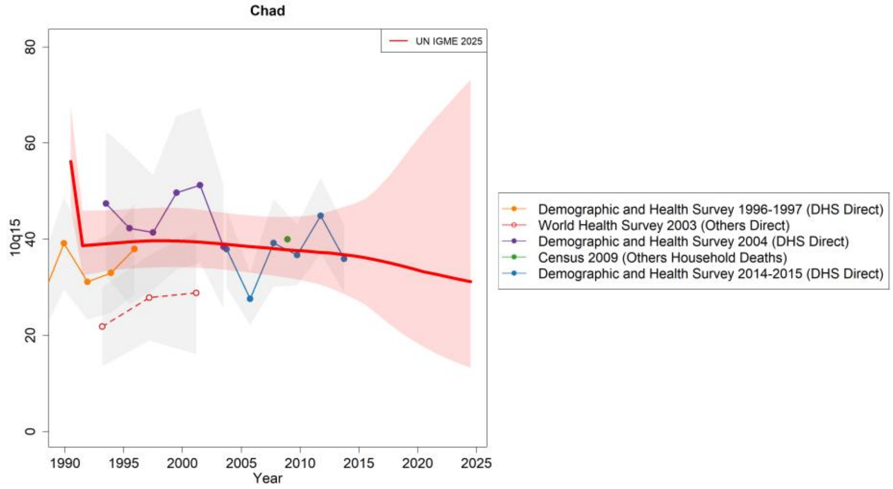
Chad
UN IGME 2025
10q15
Year
Demographic and Health Survey 1996-1997 (DHS Direct)
World Health Survey 2003 (Others Direct)
Demographic and Health Survey 2004 (DHS Direct)
Census 2009 (Others Household Deaths)
Demographic and Health Survey 2014-2015 (DHS Direct)

Chile (CHL)

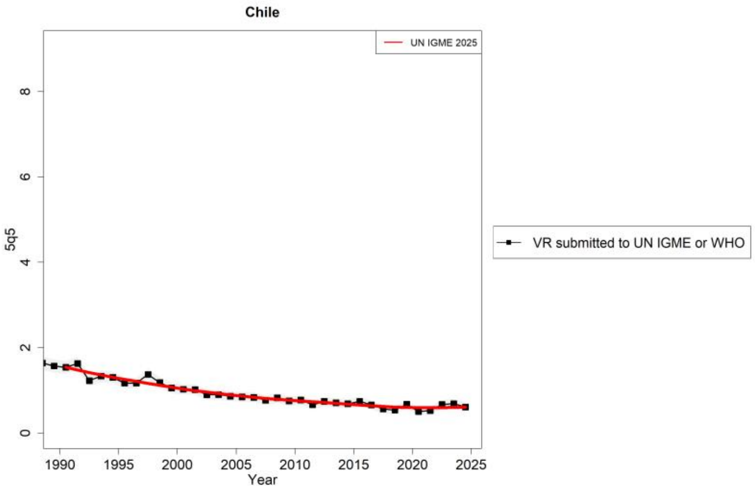
Chile
UN IGME 2025
VR submitted to UN IGME or WHO
5q5
0
2
4
6
8
1990
1995
2000
2005
2010
2015
2020
2025
Year

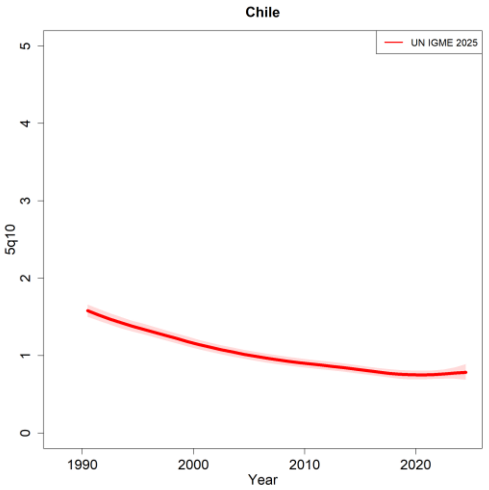
Chile
UN IGME 2025
5q10
0
1
2
3
4
5
1990
2000
2010
2020
Year

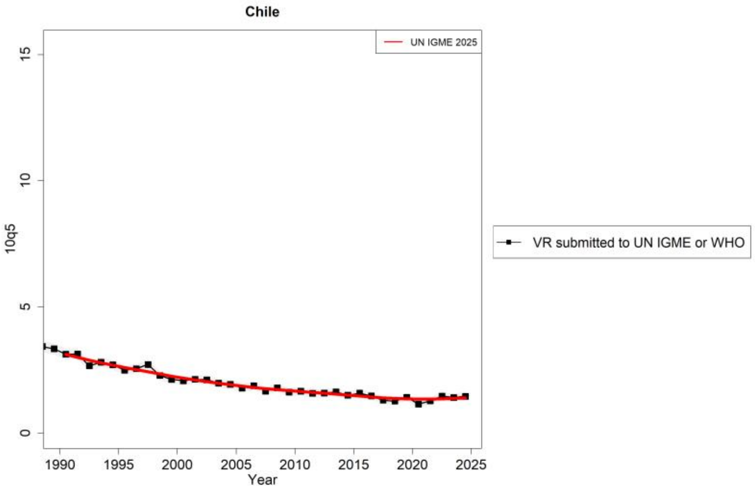
Chile
UN IGME 2025
VR submitted to UN IGME or WHO
10q5
0
5
10
15
1990
1995
2000
2005
2010
2015
2020
2025
Year

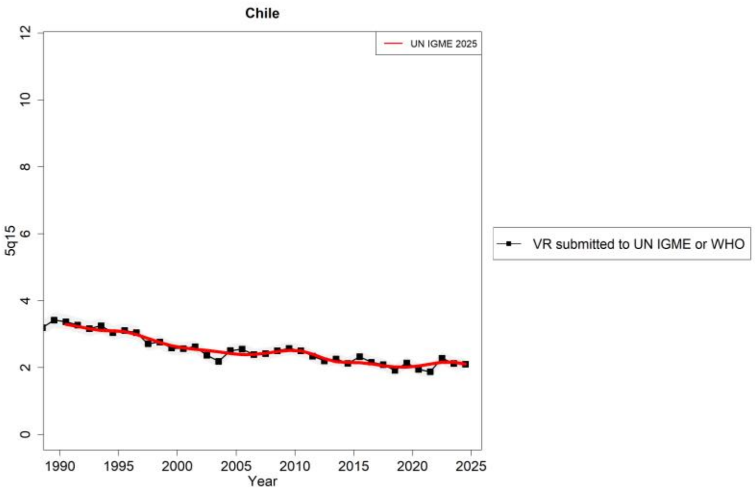
Chile
UN IGME 2025
VR submitted to UN IGME or WHO
5q15
Year

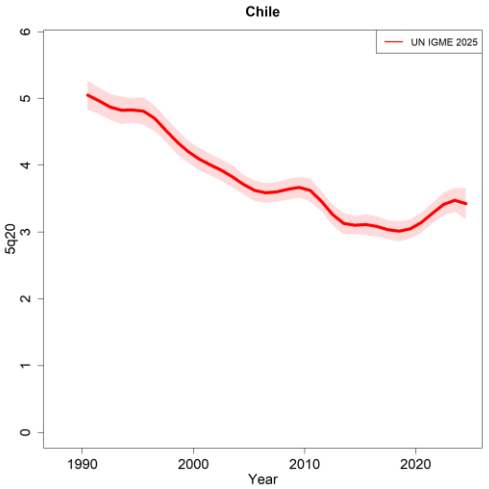
Chile
UN IGME 2025
5q20
Year

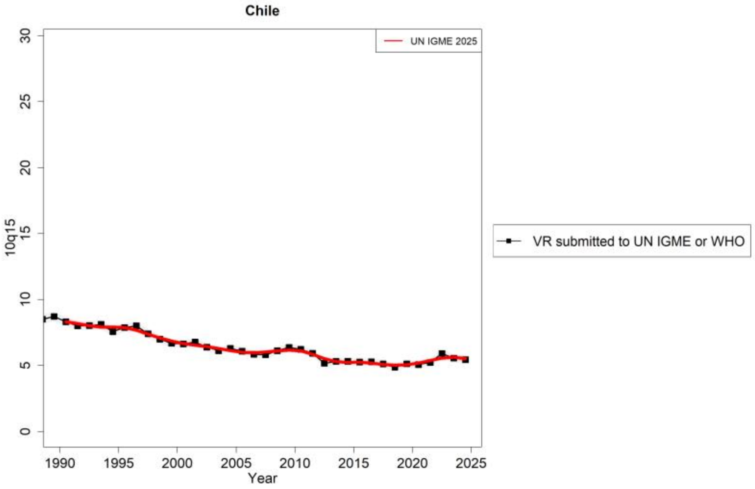
Chile
UN IGME 2025
VR submitted to UN IGME or WHO
10q15
Year

China (CHN)

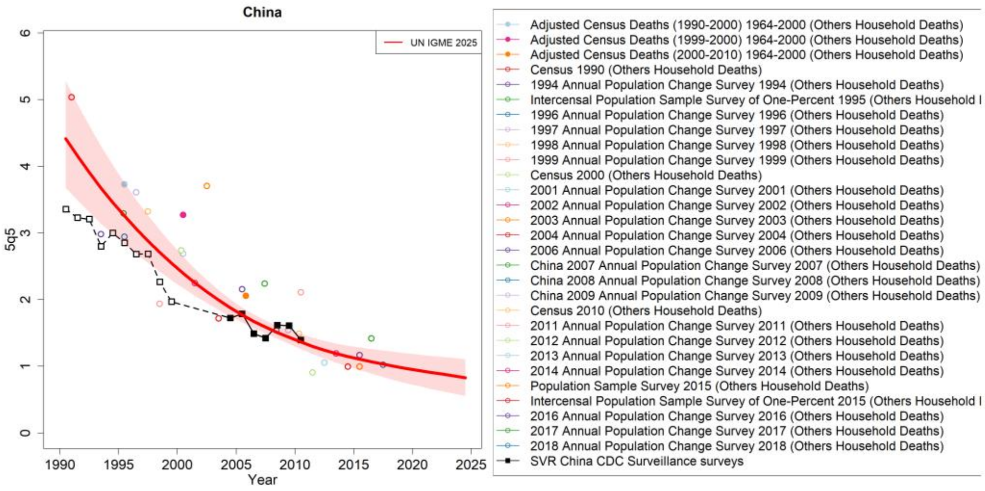
China
UN IGME 2025
5q5
Year
0
1
2
3
4
5
6
1990
1995
2000
2005
2010
2015
2020
2025
Adjusted Census Deaths (1990-2000) 1964-2000 (Others Household Deaths)
Adjusted Census Deaths (1999-2000) 1964-2000 (Others Household Deaths)
Adjusted Census Deaths (2000-2010) 1964-2000 (Others Household Deaths)
Census 1990 (Others Household Deaths)
1994 Annual Population Change Survey 1994 (Others Household Deaths)
Intercensal Population Sample Survey of One-Percent 1995 (Others Household I
1996 Annual Population Change Survey 1996 (Others Household Deaths)
1997 Annual Population Change Survey 1997 (Others Household Deaths)
1998 Annual Population Change Survey 1998 (Others Household Deaths)
1999 Annual Population Change Survey 1999 (Others Household Deaths)
Census 2000 (Others Household Deaths)
2001 Annual Population Change Survey 2001 (Others Household Deaths)
2002 Annual Population Change Survey 2002 (Others Household Deaths)
2003 Annual Population Change Survey 2003 (Others Household Deaths)
2004 Annual Population Change Survey 2004 (Others Household Deaths)
2006 Annual Population Change Survey 2006 (Others Household Deaths)
China 2007 Annual Population Change Survey 2007 (Others Household Deaths)
China 2008 Annual Population Change Survey 2008 (Others Household Deaths)
China 2009 Annual Population Change Survey 2009 (Others Household Deaths)
Census 2010 (Others Household Deaths)
2011 Annual Population Change Survey 2011 (Others Household Deaths)
2012 Annual Population Change Survey 2012 (Others Household Deaths)
2013 Annual Population Change Survey 2013 (Others Household Deaths)
2014 Annual Population Change Survey 2014 (Others Household Deaths)
Population Sample Survey 2015 (Others Household Deaths)
Intercensal Population Sample Survey of One-Percent 2015 (Others Household I
2016 Annual Population Change Survey 2016 (Others Household Deaths)
2017 Annual Population Change Survey 2017 (Others Household Deaths)
2018 Annual Population Change Survey 2018 (Others Household Deaths)
SVR China CDC Surveillance surveys

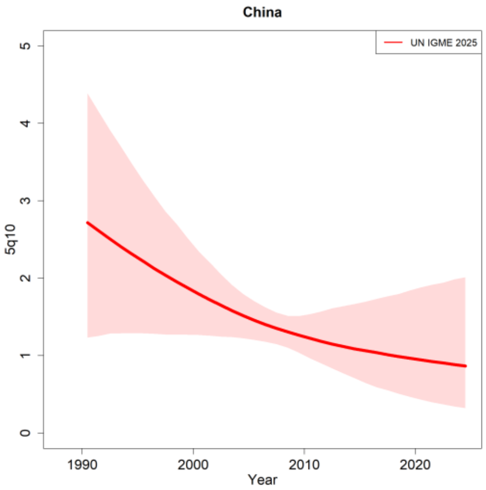
China
UN IGME 2025
5q10
Year
0
1
2
3
4
5
1990
2000
2010
2020

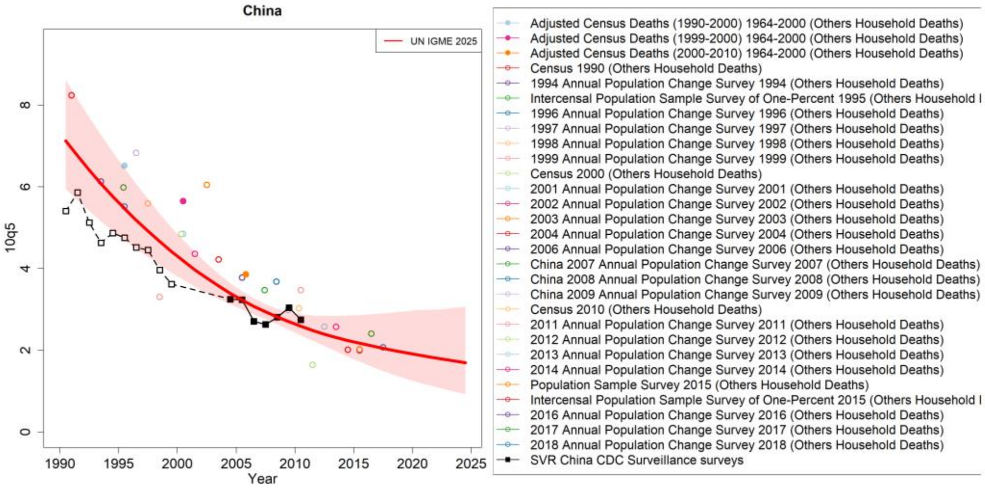
China
UN IGME 2025
10q5
Year
0
2
4
6
8
1990
1995
2000
2005
2010
2015
2020
2025
Adjusted Census Deaths (1990-2000) 1964-2000 (Others Household Deaths)
Adjusted Census Deaths (1999-2000) 1964-2000 (Others Household Deaths)
Adjusted Census Deaths (2000-2010) 1964-2000 (Others Household Deaths)
Census 1990 (Others Household Deaths)
1994 Annual Population Change Survey 1994 (Others Household Deaths)
Intercensal Population Sample Survey of One-Percent 1995 (Others Household I
1996 Annual Population Change Survey 1996 (Others Household Deaths)
1997 Annual Population Change Survey 1997 (Others Household Deaths)
1998 Annual Population Change Survey 1998 (Others Household Deaths)
1999 Annual Population Change Survey 1999 (Others Household Deaths)
Census 2000 (Others Household Deaths)
2001 Annual Population Change Survey 2001 (Others Household Deaths)
2002 Annual Population Change Survey 2002 (Others Household Deaths)
2003 Annual Population Change Survey 2003 (Others Household Deaths)
2004 Annual Population Change Survey 2004 (Others Household Deaths)
2006 Annual Population Change Survey 2006 (Others Household Deaths)
China 2007 Annual Population Change Survey 2007 (Others Household Deaths)
China 2008 Annual Population Change Survey 2008 (Others Household Deaths)
China 2009 Annual Population Change Survey 2009 (Others Household Deaths)
Census 2010 (Others Household Deaths)
2011 Annual Population Change Survey 2011 (Others Household Deaths)
2012 Annual Population Change Survey 2012 (Others Household Deaths)
2013 Annual Population Change Survey 2013 (Others Household Deaths)
2014 Annual Population Change Survey 2014 (Others Household Deaths)
Population Sample Survey 2015 (Others Household Deaths)
Intercensal Population Sample Survey of One-Percent 2015 (Others Household I
2016 Annual Population Change Survey 2016 (Others Household Deaths)
2017 Annual Population Change Survey 2017 (Others Household Deaths)
2018 Annual Population Change Survey 2018 (Others Household Deaths)
SVR China CDC Surveillance surveys

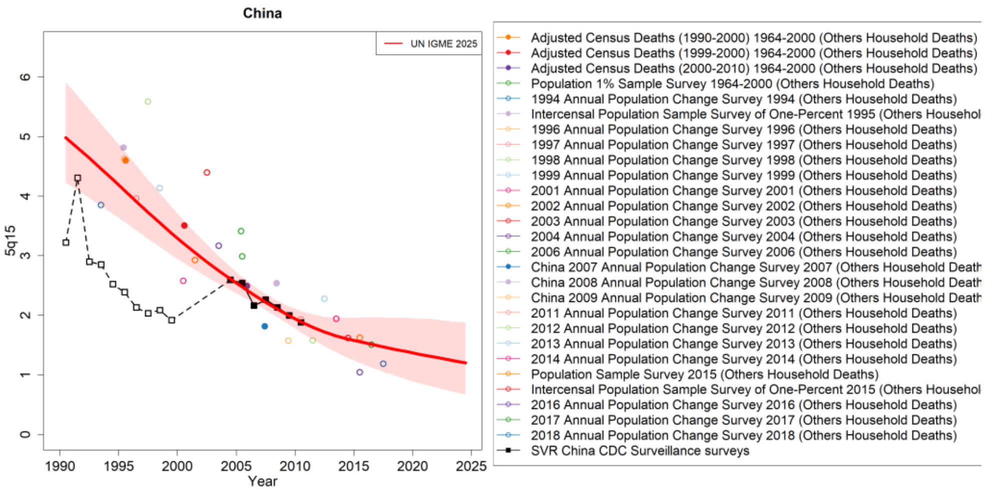
China
UN IGME 2025
5q15
Year
Adjusted Census Deaths (1990-2000) 1964-2000 (Others Household Deaths)
Adjusted Census Deaths (1999-2000) 1964-2000 (Others Household Deaths)
Adjusted Census Deaths (2000-2010) 1964-2000 (Others Household Deaths)
Population 1% Sample Survey 1964-2000 (Others Household Deaths)
1994 Annual Population Change Survey 1994 (Others Household Deaths)
Intercensal Population Sample Survey of One-Percent 1995 (Others Househol
1996 Annual Population Change Survey 1996 (Others Household Deaths)
1997 Annual Population Change Survey 1997 (Others Household Deaths)
1998 Annual Population Change Survey 1998 (Others Household Deaths)
1999 Annual Population Change Survey 1999 (Others Household Deaths)
2001 Annual Population Change Survey 2001 (Others Household Deaths)
2002 Annual Population Change Survey 2002 (Others Household Deaths)
2003 Annual Population Change Survey 2003 (Others Household Deaths)
2004 Annual Population Change Survey 2004 (Others Household Deaths)
2006 Annual Population Change Survey 2006 (Others Household Deaths)
China 2007 Annual Population Change Survey 2007 (Others Household Death
China 2008 Annual Population Change Survey 2008 (Others Household Death
China 2009 Annual Population Change Survey 2009 (Others Household Death
2011 Annual Population Change Survey 2011 (Others Household Deaths)
2012 Annual Population Change Survey 2012 (Others Household Deaths)
2013 Annual Population Change Survey 2013 (Others Household Deaths)
2014 Annual Population Change Survey 2014 (Others Household Deaths)
Population Sample Survey 2015 (Others Household Deaths)
Intercensal Population Sample Survey of One-Percent 2015 (Others Househol
2016 Annual Population Change Survey 2016 (Others Household Deaths)
2017 Annual Population Change Survey 2017 (Others Household Deaths)
2018 Annual Population Change Survey 2018 (Others Household Deaths)
SVR China CDC Surveillance surveys

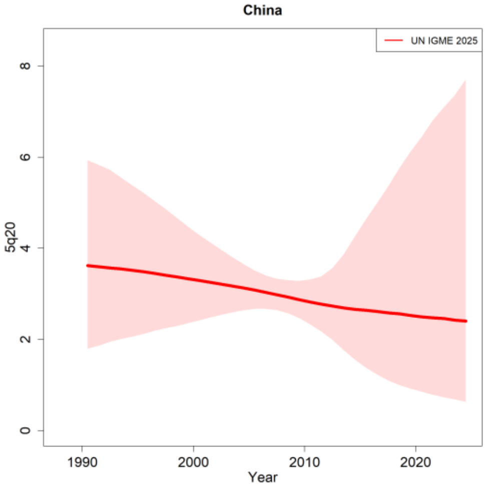
China
UN IGME 2025
5q20
Year

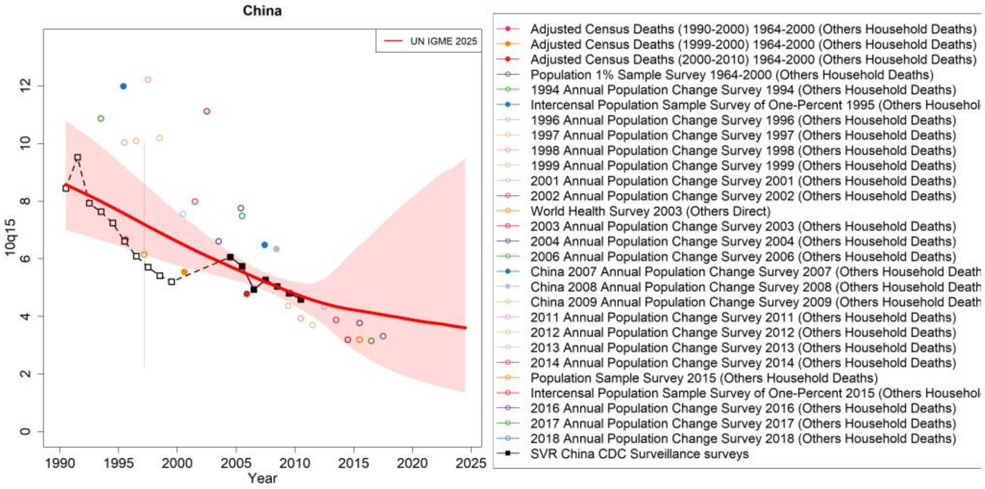
China
UN IGME 2025
10q15
Year
Adjusted Census Deaths (1990-2000) 1964-2000 (Others Household Deaths)
Adjusted Census Deaths (1999-2000) 1964-2000 (Others Household Deaths)
Adjusted Census Deaths (2000-2010) 1964-2000 (Others Household Deaths)
Population 1% Sample Survey 1964-2000 (Others Household Deaths)
1994 Annual Population Change Survey 1994 (Others Household Deaths)
Intercensal Population Sample Survey of One-Percent 1995 (Others Househol
1996 Annual Population Change Survey 1996 (Others Household Deaths)
1997 Annual Population Change Survey 1997 (Others Household Deaths)
1998 Annual Population Change Survey 1998 (Others Household Deaths)
1999 Annual Population Change Survey 1999 (Others Household Deaths)
2001 Annual Population Change Survey 2001 (Others Household Deaths)
2002 Annual Population Change Survey 2002 (Others Household Deaths)
World Health Survey 2003 (Others Direct)
2003 Annual Population Change Survey 2003 (Others Household Deaths)
2004 Annual Population Change Survey 2004 (Others Household Deaths)
2006 Annual Population Change Survey 2006 (Others Household Deaths)
China 2007 Annual Population Change Survey 2007 (Others Household Death
China 2008 Annual Population Change Survey 2008 (Others Household Death
China 2009 Annual Population Change Survey 2009 (Others Household Death
2011 Annual Population Change Survey 2011 (Others Household Deaths)
2012 Annual Population Change Survey 2012 (Others Household Deaths)
2013 Annual Population Change Survey 2013 (Others Household Deaths)
2014 Annual Population Change Survey 2014 (Others Household Deaths)
Population Sample Survey 2015 (Others Household Deaths)
Intercensal Population Sample Survey of One-Percent 2015 (Others Househol
2016 Annual Population Change Survey 2016 (Others Household Deaths)
2017 Annual Population Change Survey 2017 (Others Household Deaths)
2018 Annual Population Change Survey 2018 (Others Household Deaths)
SVR China CDC Surveillance surveys

Colombia (COL)

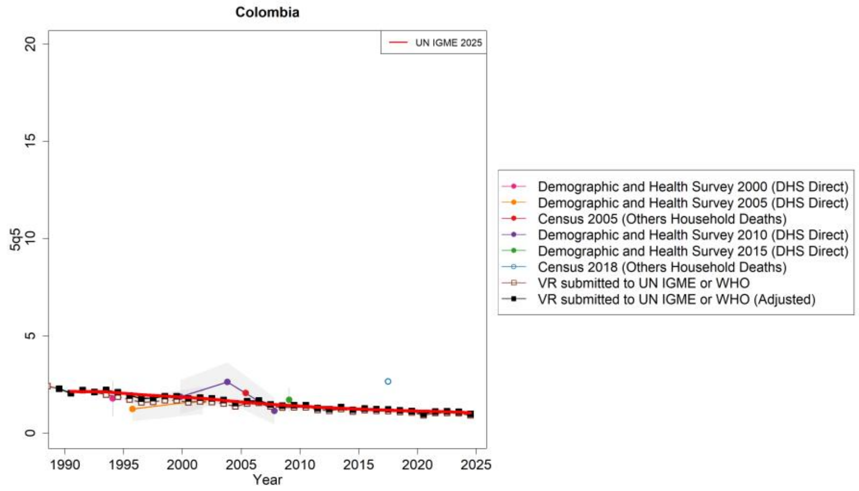

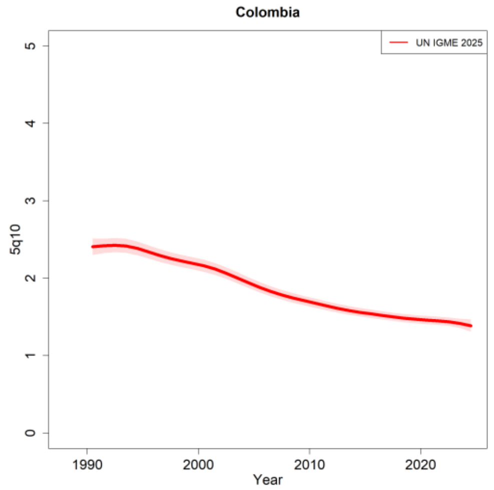

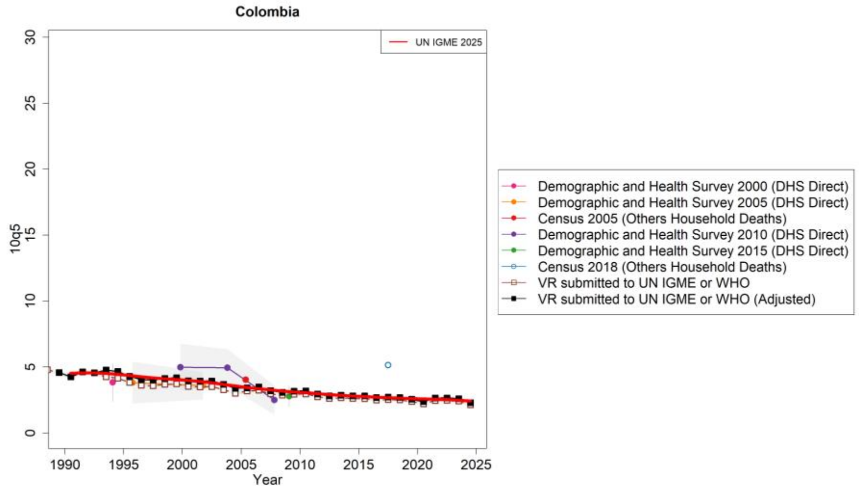

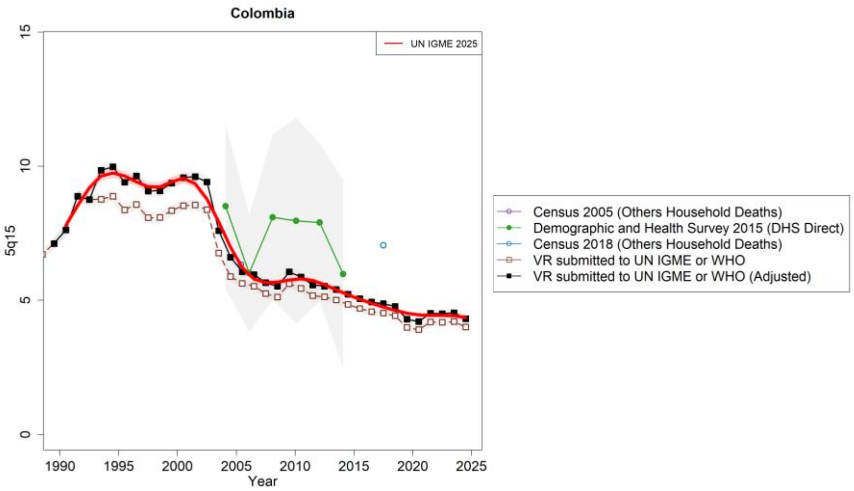
Colombia
UN IGME 2025
Census 2005 (Others Household Deaths)
Demographic and Health Survey 2015 (DHS Direct)
Census 2018 (Others Household Deaths)
VR submitted to UN IGME or WHO
VR submitted to UN IGME or WHO (Adjusted)
5q15
Year

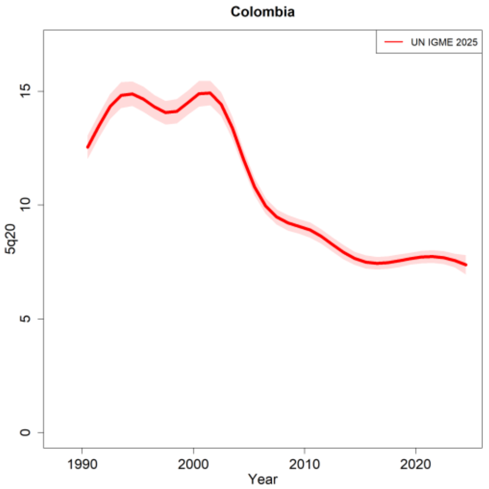
Colombia
UN IGME 2025
5q20
Year

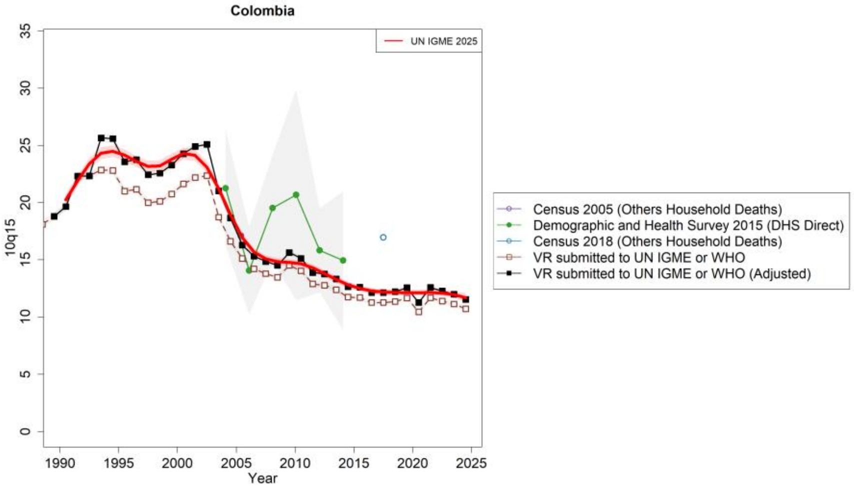
Colombia
UN IGME 2025
Census 2005 (Others Household Deaths)
Demographic and Health Survey 2015 (DHS Direct)
Census 2018 (Others Household Deaths)
VR submitted to UN IGME or WHO
VR submitted to UN IGME or WHO (Adjusted)
10q15
Year

Comoros (COM)

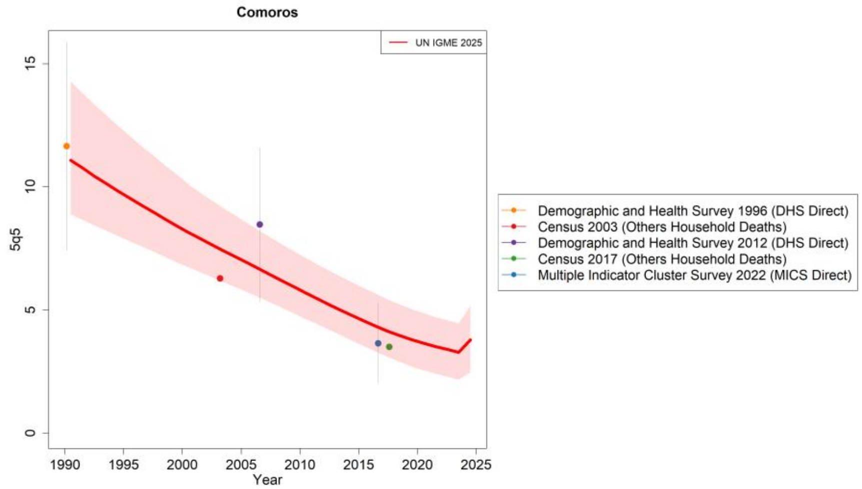
Comoros
UN IGME 2025
5q5
Year
Demographic and Health Survey 1996 (DHS Direct)
Census 2003 (Others Household Deaths)
Demographic and Health Survey 2012 (DHS Direct)
Census 2017 (Others Household Deaths)
Multiple Indicator Cluster Survey 2022 (MICS Direct)

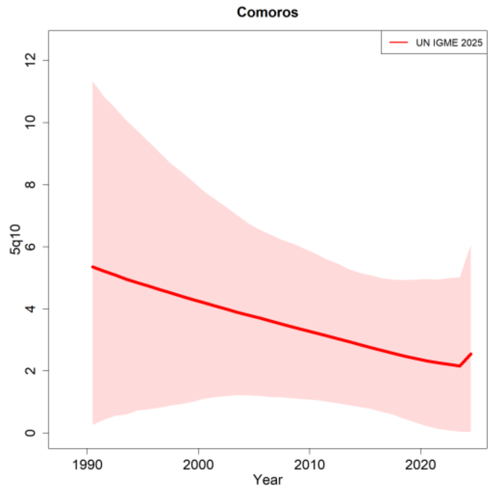
Comoros
UN IGME 2025
5q10
Year

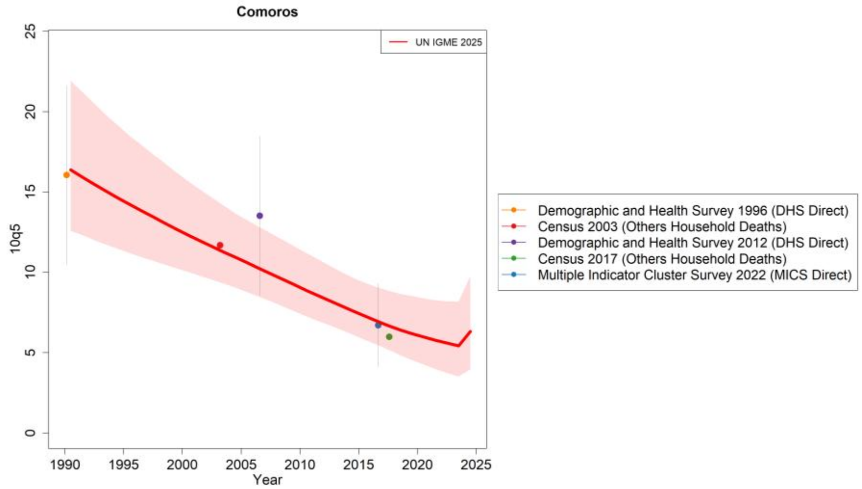
Comoros
UN IGME 2025
10q5
Year
Demographic and Health Survey 1996 (DHS Direct)
Census 2003 (Others Household Deaths)
Demographic and Health Survey 2012 (DHS Direct)
Census 2017 (Others Household Deaths)
Multiple Indicator Cluster Survey 2022 (MICS Direct)

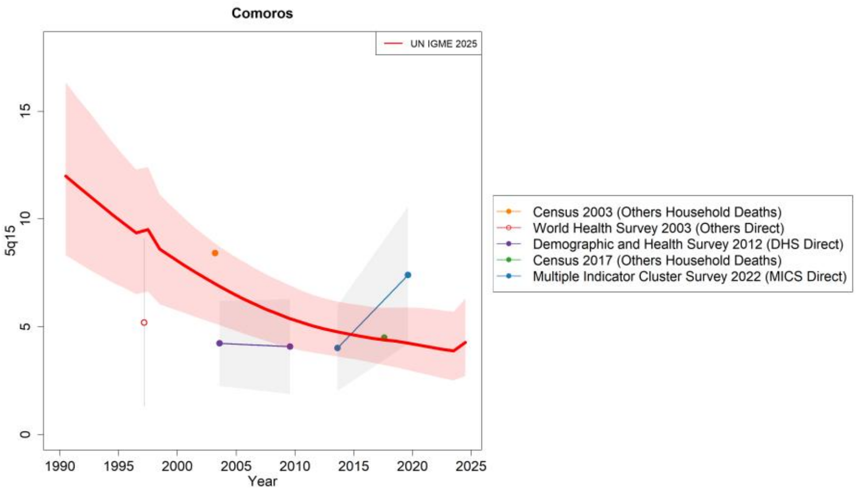
Comoros
UN IGME 2025
5q15
Year
Census 2003 (Others Household Deaths)
World Health Survey 2003 (Others Direct)
Demographic and Health Survey 2012 (DHS Direct)
Census 2017 (Others Household Deaths)
Multiple Indicator Cluster Survey 2022 (MICS Direct)

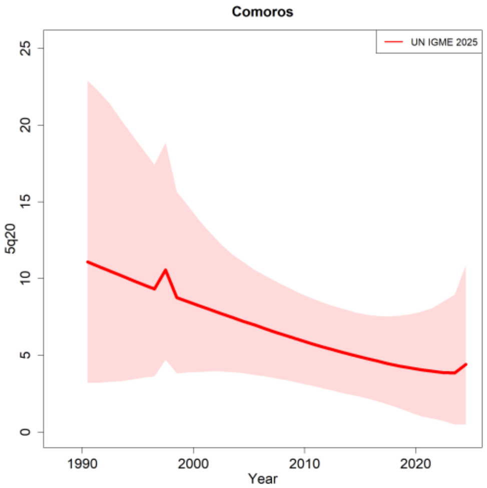
Comoros
UN IGME 2025
5q20
Year

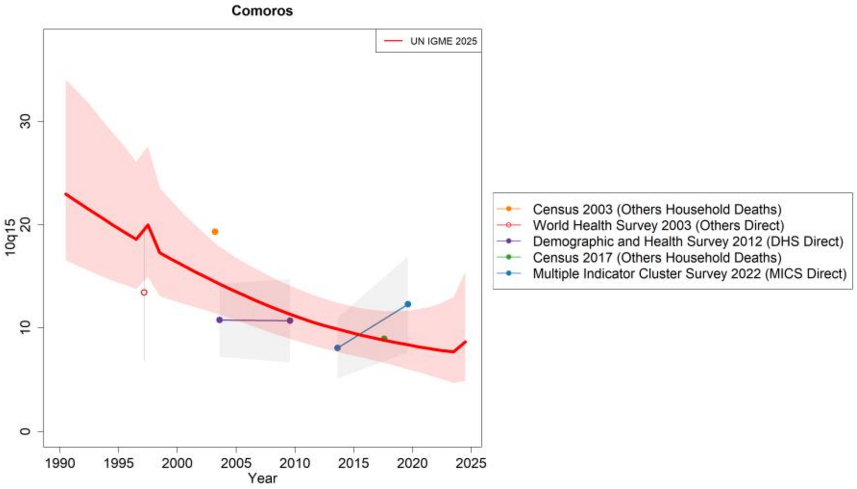
Comoros
UN IGME 2025
10q15
Year
Census 2003 (Others Household Deaths)
World Health Survey 2003 (Others Direct)
Demographic and Health Survey 2012 (DHS Direct)
Census 2017 (Others Household Deaths)
Multiple Indicator Cluster Survey 2022 (MICS Direct)

Congo (COG)

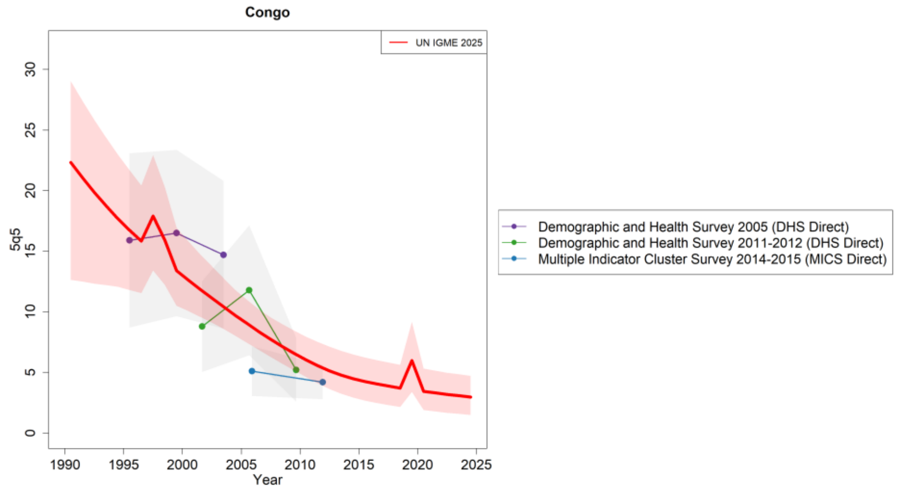
Congo
UN IGME 2025
5q5
Year
Demographic and Health Survey 2005 (DHS Direct)
Demographic and Health Survey 2011-2012 (DHS Direct)
Multiple Indicator Cluster Survey 2014-2015 (MICS Direct)

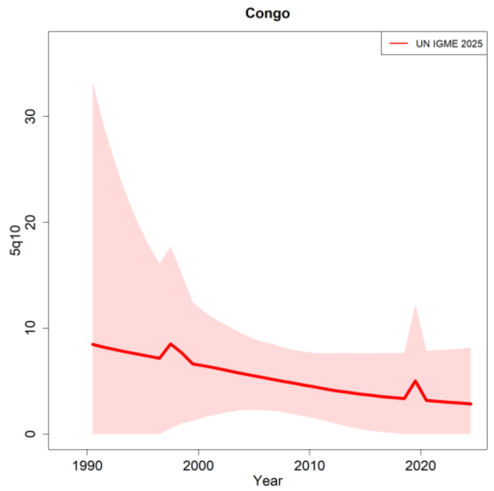
Congo
UN IGME 2025
5q10
Year

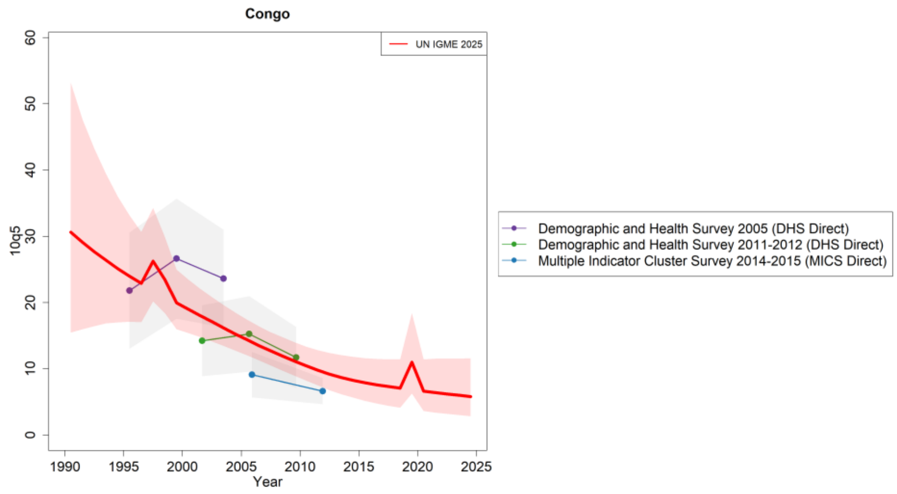
Congo
UN IGME 2025
10q5
Year
Demographic and Health Survey 2005 (DHS Direct)
Demographic and Health Survey 2011-2012 (DHS Direct)
Multiple Indicator Cluster Survey 2014-2015 (MICS Direct)

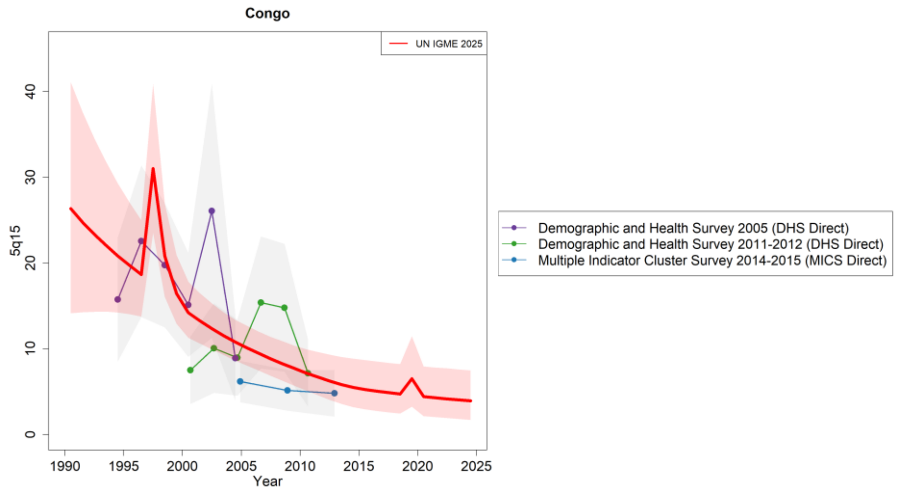
Congo
UN IGME 2025
5q15
Year
Demographic and Health Survey 2005 (DHS Direct)
Demographic and Health Survey 2011-2012 (DHS Direct)
Multiple Indicator Cluster Survey 2014-2015 (MICS Direct)

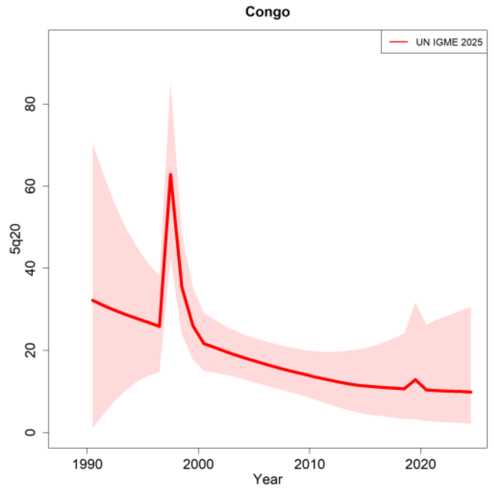
Congo
UN IGME 2025
5q20
Year

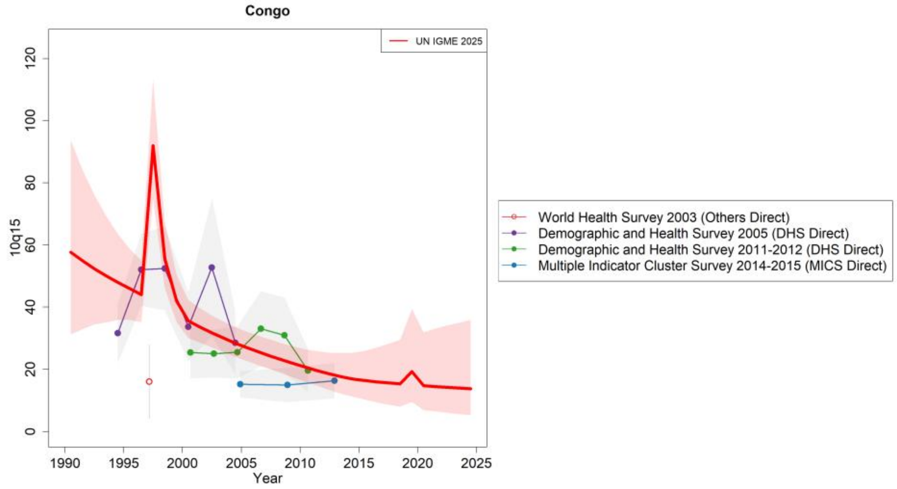
Congo
UN IGME 2025
10q15
Year
World Health Survey 2003 (Others Direct)
Demographic and Health Survey 2005 (DHS Direct)
Demographic and Health Survey 2011-2012 (DHS Direct)
Multiple Indicator Cluster Survey 2014-2015 (MICS Direct)

Cook Islands (COK)

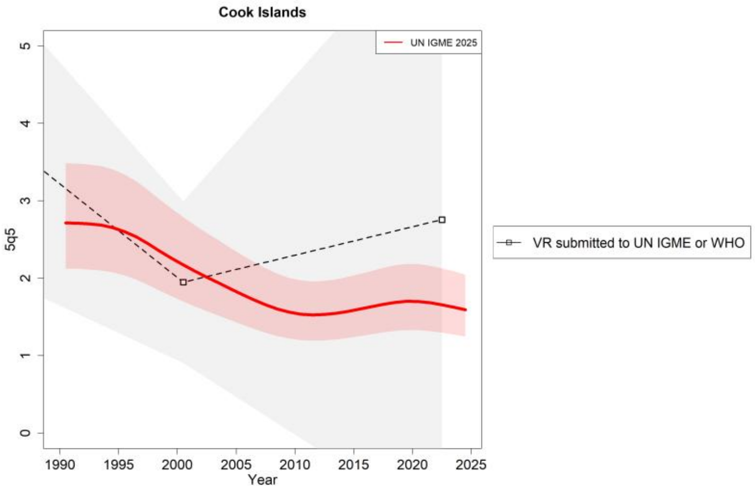

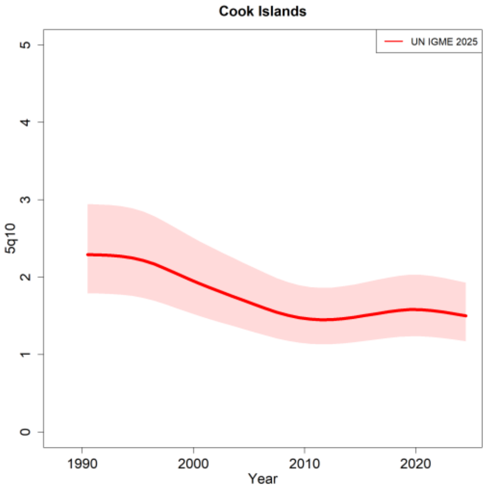

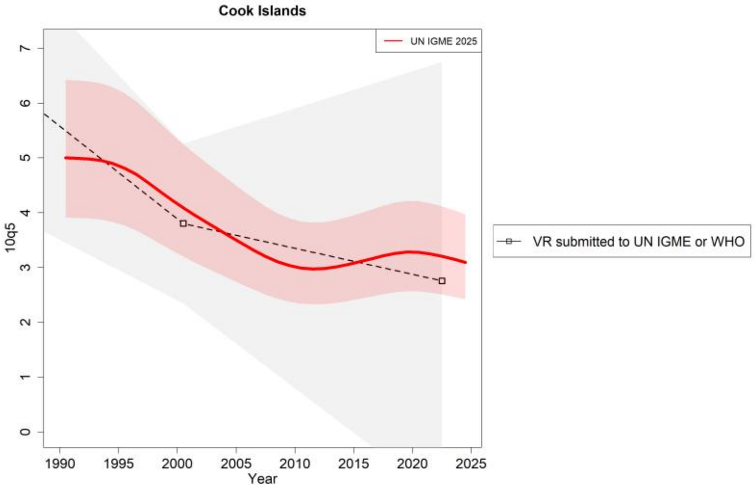

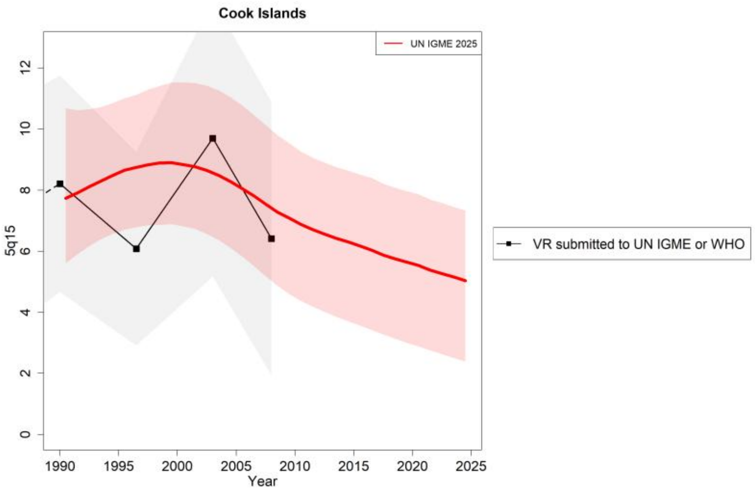

Cook Islands
UN IGME 2025
VR submitted to UN IGME or WHO
5q15
Year

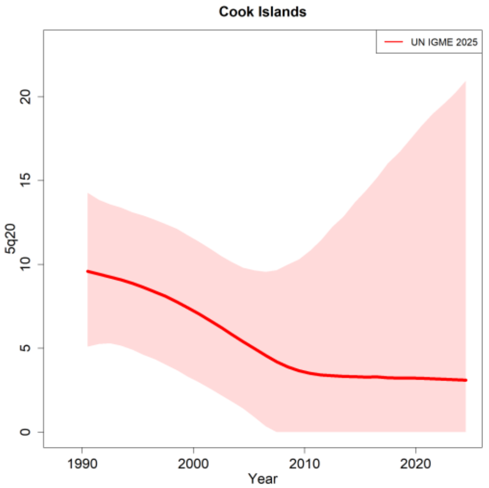

Cook Islands
UN IGME 2025
5q20
Year

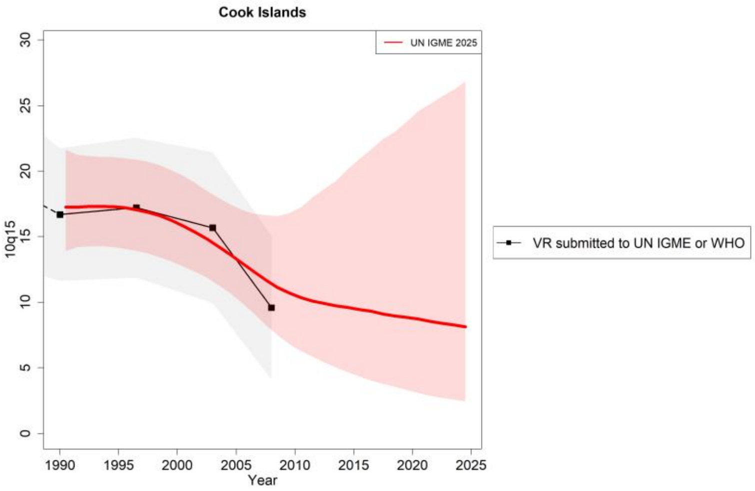

Cook Islands
UN IGME 2025
VR submitted to UN IGME or WHO
10q15
Year

Costa Rica (CRI)

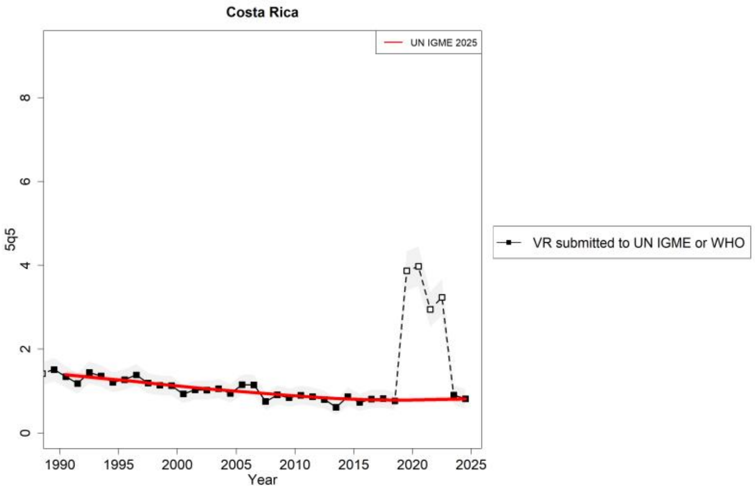

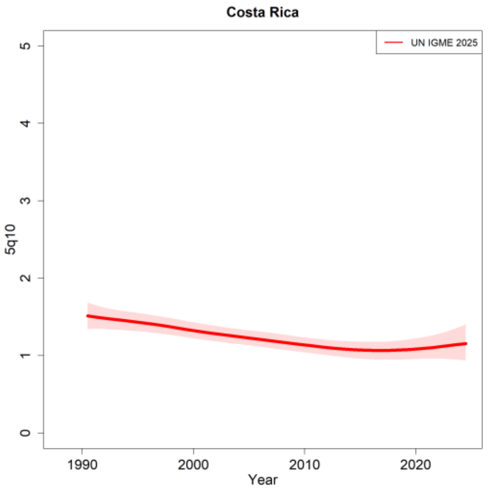

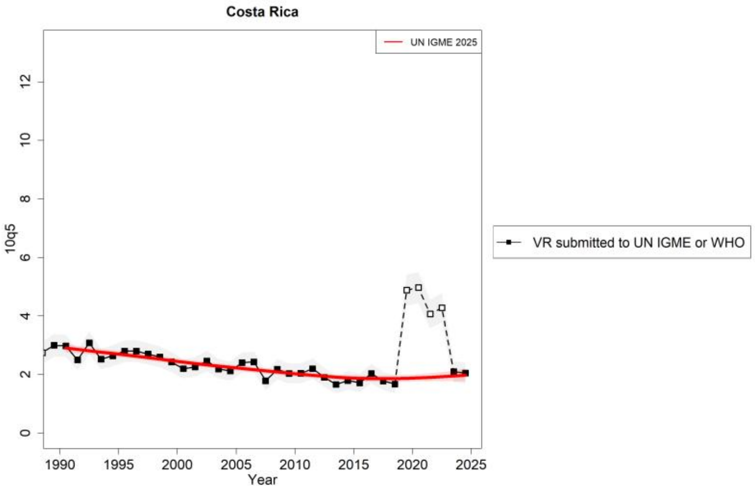

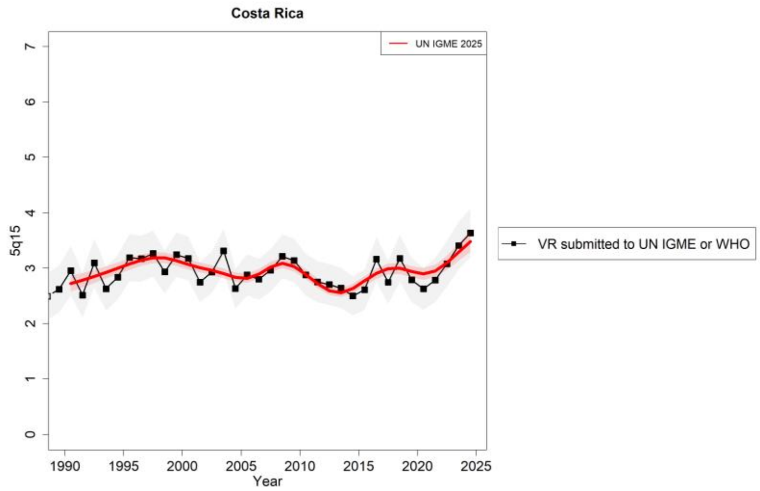
Costa Rica
UN IGME 2025
VR submitted to UN IGME or WHO
5q15
0
1
2
3
4
5
6
7
1990
1995
2000
2005
2010
2015
2020
2025
Year

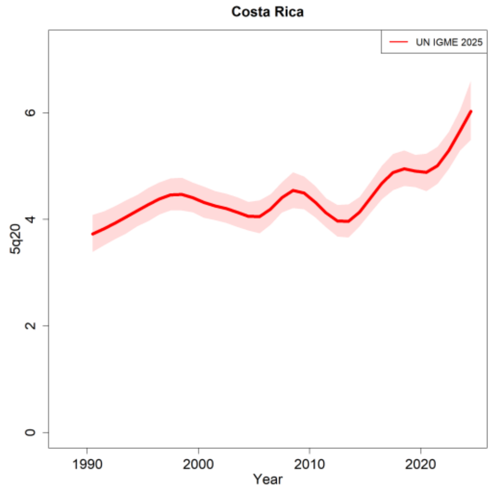
Costa Rica
UN IGME 2025
5q20
0
2
4
6
1990
2000
2010
2020
Year

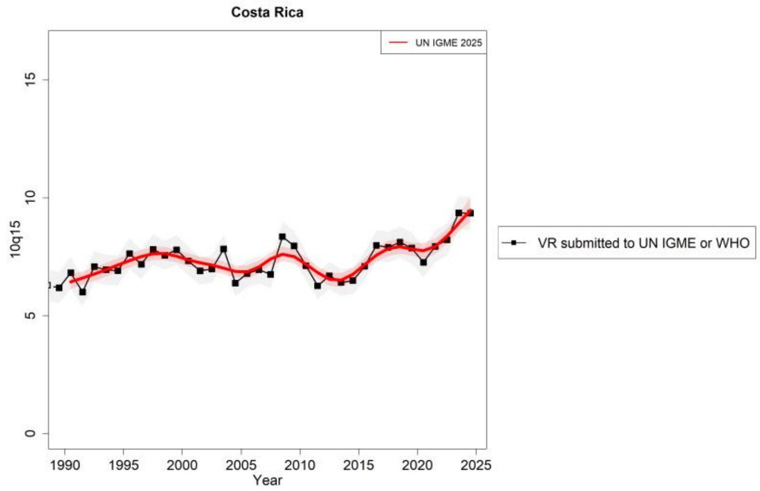
Costa Rica
UN IGME 2025
VR submitted to UN IGME or WHO
10q15
0
5
10
15
1990
1995
2000
2005
2010
2015
2020
2025
Year

Croatia (HRV)

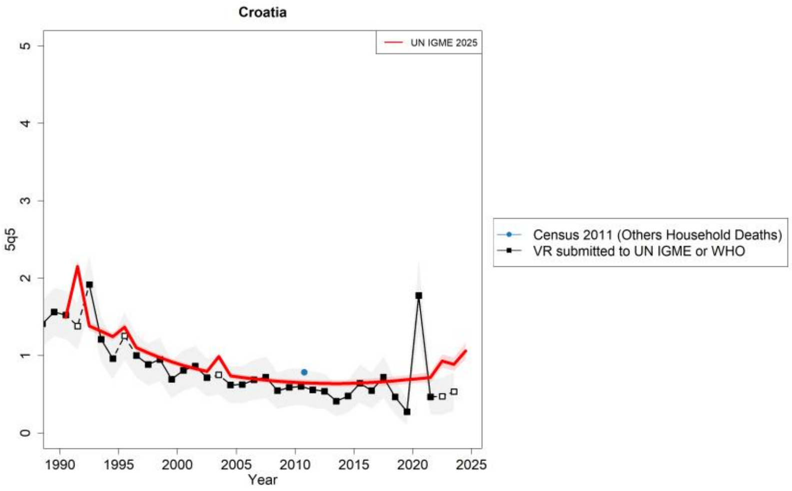
Croatia
UN IGME 2025
5q5
0
1
2
3
4
5
1990
1995
2000
2005
2010
2015
2020
2025
Year
Census 2011 (Others Household Deaths)
VR submitted to UN IGME or WHO

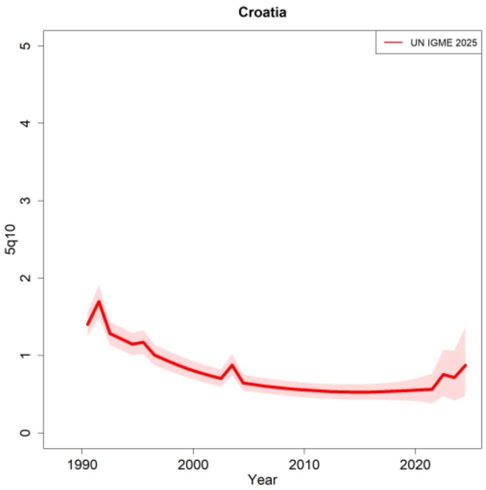
Croatia
UN IGME 2025
5q10
0
1
2
3
4
5
1990
2000
2010
2020
Year

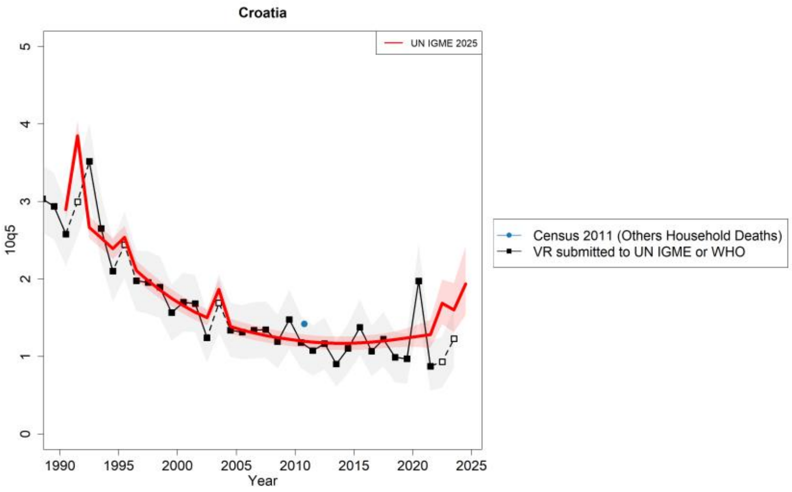
Croatia
UN IGME 2025
10q5
0
1
2
3
4
5
1990
1995
2000
2005
2010
2015
2020
2025
Year
Census 2011 (Others Household Deaths)
VR submitted to UN IGME or WHO

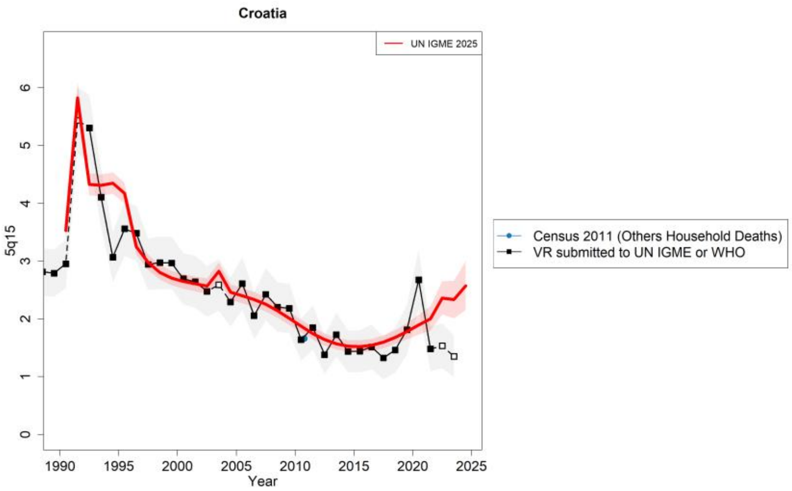
Croatia
UN IGME 2025
Census 2011 (Others Household Deaths)
VR submitted to UN IGME or WHO
5q15
Year
0
1
2
3
4
5
6
1990
1995
2000
2005
2010
2015
2020
2025

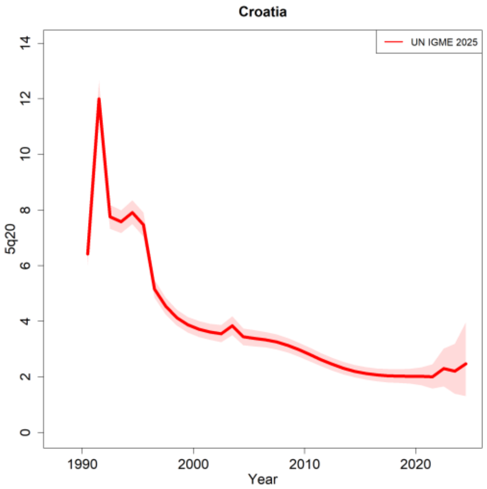
Croatia
UN IGME 2025
5q20
Year
0
2
4
6
8
10
12
14
1990
2000
2010
2020

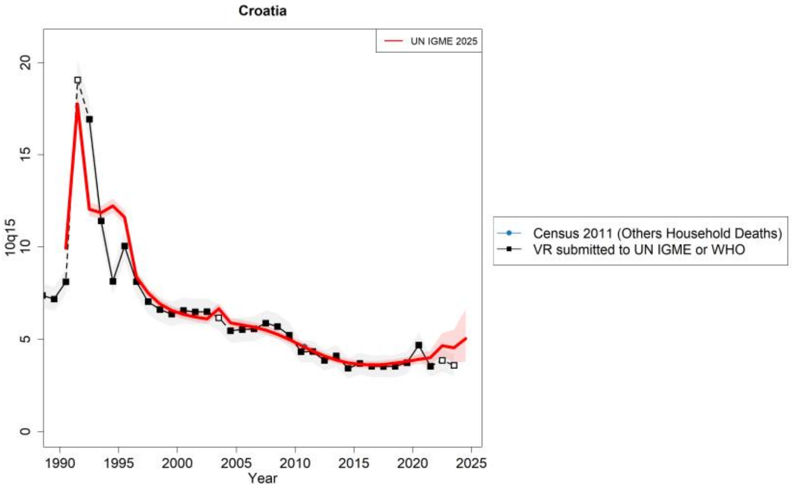
Croatia
UN IGME 2025
Census 2011 (Others Household Deaths)
VR submitted to UN IGME or WHO
10q15
Year
0
5
10
15
20
1990
1995
2000
2005
2010
2015
2020
2025

Cuba (CUB)

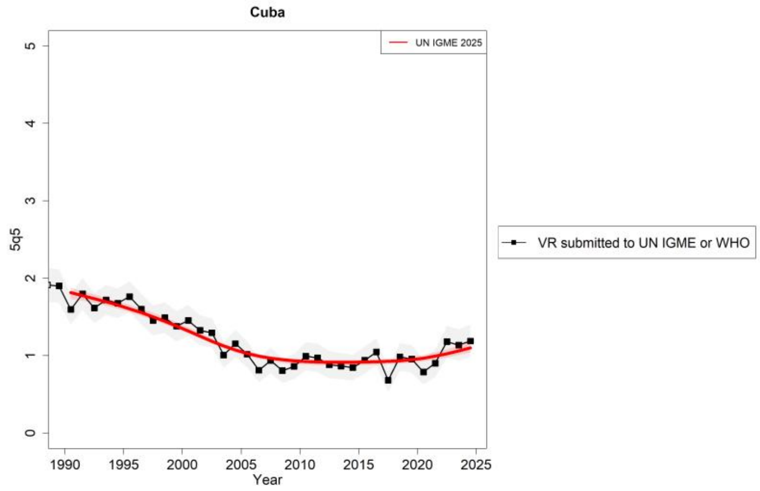
Cuba
UN IGME 2025
VR submitted to UN IGME or WHO
5q5
0
1
2
3
4
5
1990
1995
2000
2005
2010
2015
2020
2025
Year

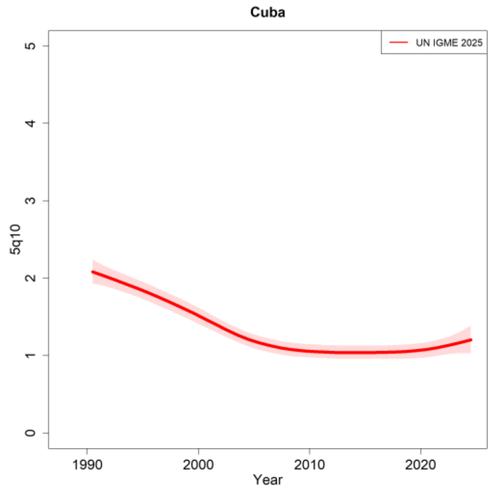
Cuba
UN IGME 2025
5q10
0
1
2
3
4
5
1990
2000
2010
2020
Year

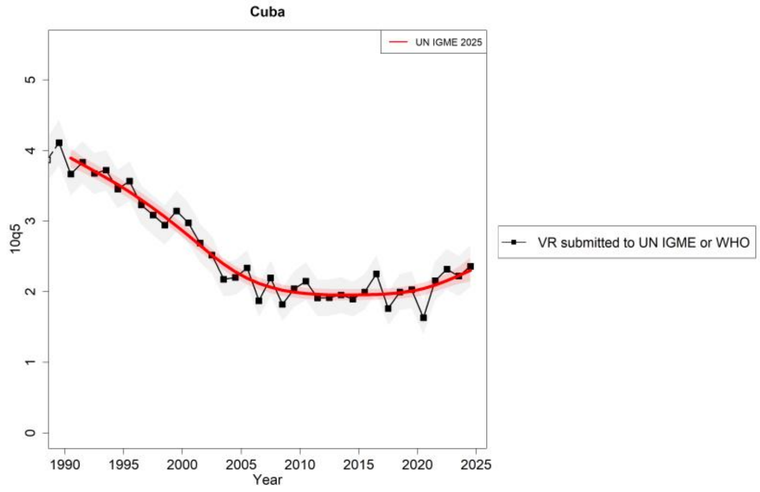
Cuba
UN IGME 2025
VR submitted to UN IGME or WHO
10q5
0
1
2
3
4
5
1990
1995
2000
2005
2010
2015
2020
2025
Year

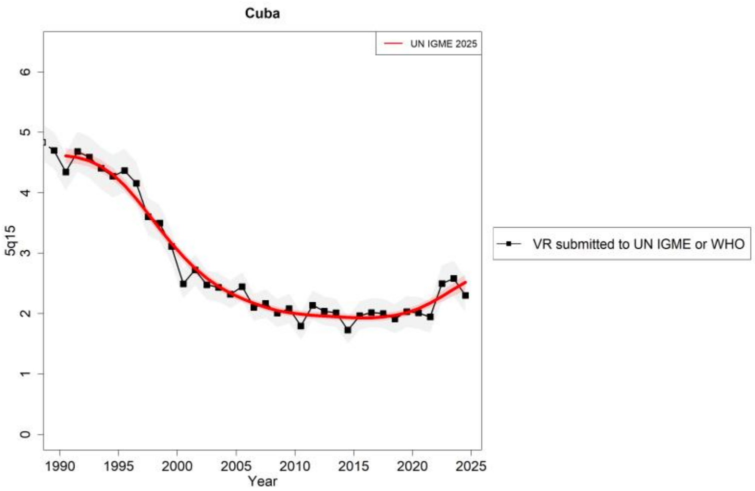
Cuba
UN IGME 2025
5q15
Year
VR submitted to UN IGME or WHO

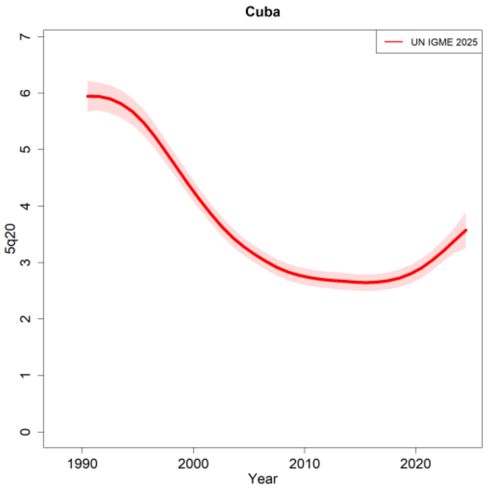
Cuba
UN IGME 2025
5q20
Year

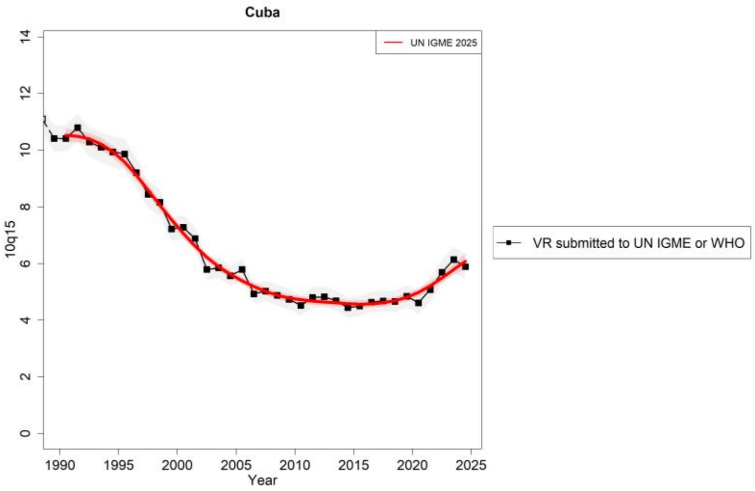
Cuba
UN IGME 2025
10q15
Year
VR submitted to UN IGME or WHO

Cyprus (CYP)

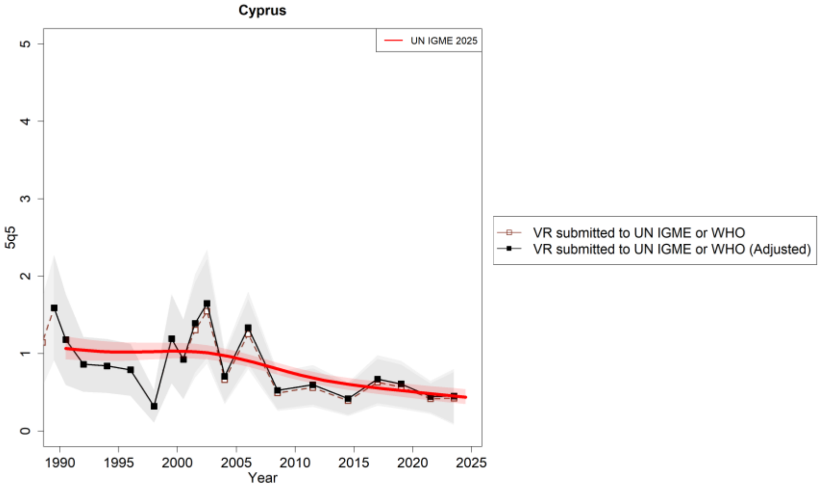
Cyprus
UN IGME 2025
5q5
Year
VR submitted to UN IGME or WHO
VR submitted to UN IGME or WHO (Adjusted)

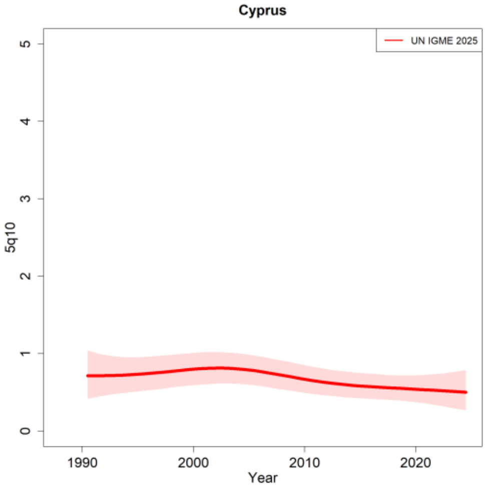
Cyprus
UN IGME 2025
5q10
Year

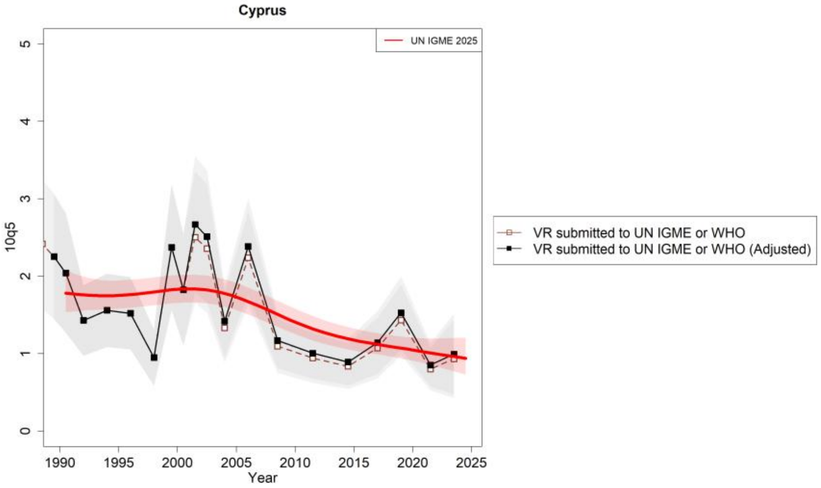
Cyprus
UN IGME 2025
10q5
Year
VR submitted to UN IGME or WHO
VR submitted to UN IGME or WHO (Adjusted)

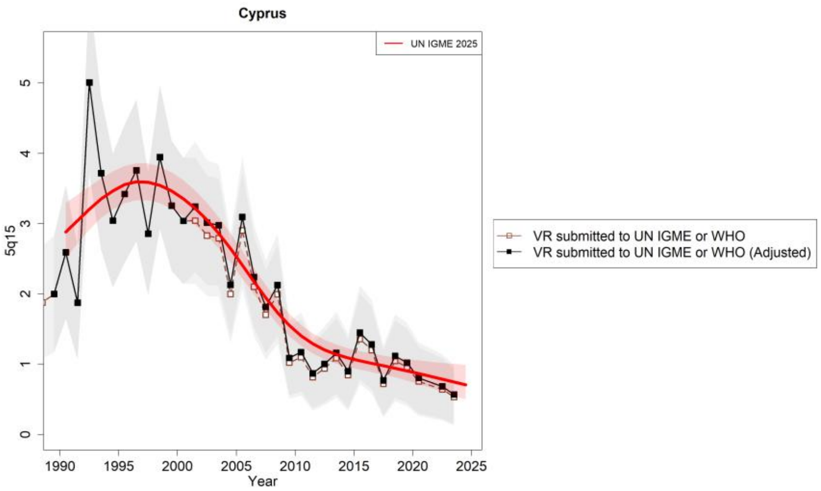
Cyprus
UN IGME 2025
VR submitted to UN IGME or WHO
VR submitted to UN IGME or WHO (Adjusted)
5q15
Year
0
1
2
3
4
5
1990
1995
2000
2005
2010
2015
2020
2025

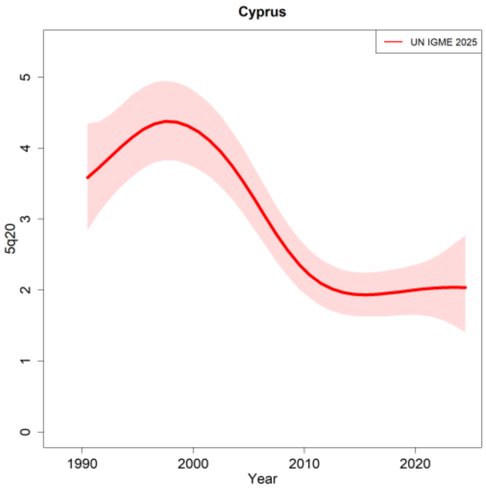
Cyprus
UN IGME 2025
5q20
Year
0
1
2
3
4
5
1990
2000
2010
2020

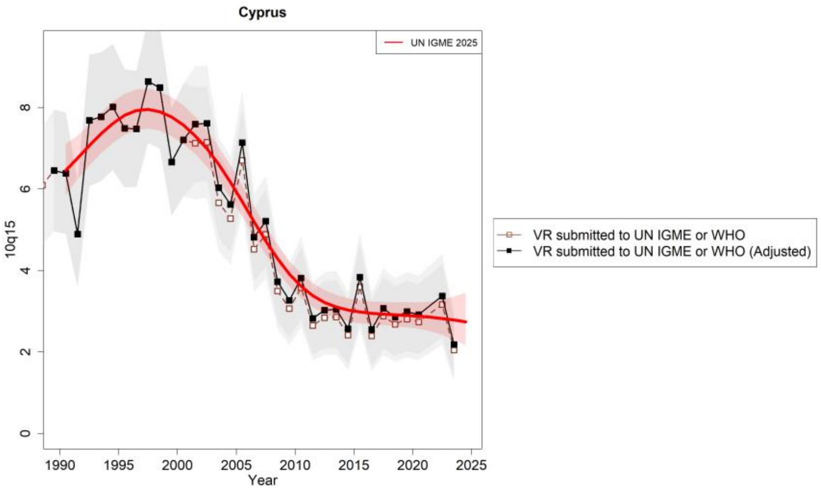
Cyprus
UN IGME 2025
VR submitted to UN IGME or WHO
VR submitted to UN IGME or WHO (Adjusted)
10q15
Year
0
2
4
6
8
1990
1995
2000
2005
2010
2015
2020
2025

Czechia (CZE)

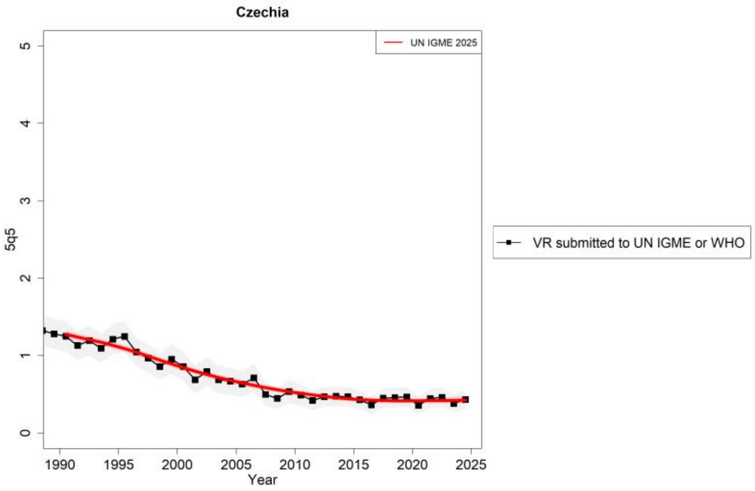
Czechia
UN IGME 2025
VR submitted to UN IGME or WHO
5q5
Year
0
1
2
3
4
5
1990
1995
2000
2005
2010
2015
2020
2025

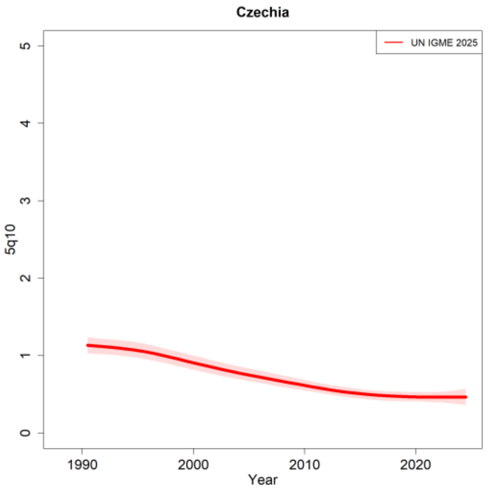
Czechia
UN IGME 2025
5q10
Year
0
1
2
3
4
5
1990
2000
2010
2020

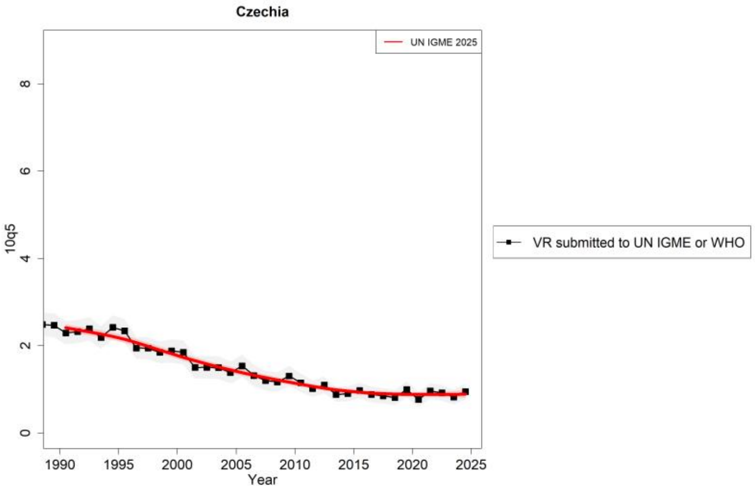
Czechia
UN IGME 2025
VR submitted to UN IGME or WHO
10q5
Year
0
2
4
6
8
1990
1995
2000
2005
2010
2015
2020
2025

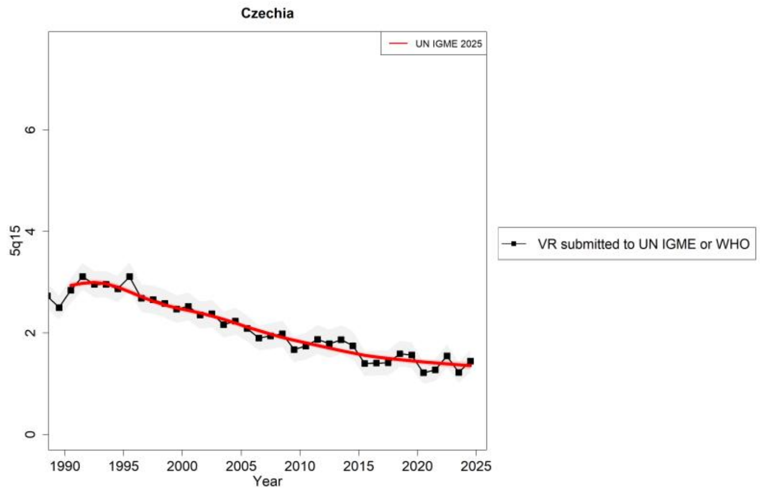
Czechia
UN IGME 2025
VR submitted to UN IGME or WHO
5q15
0
2
4
6
1990
1995
2000
2005
2010
2015
2020
2025
Year

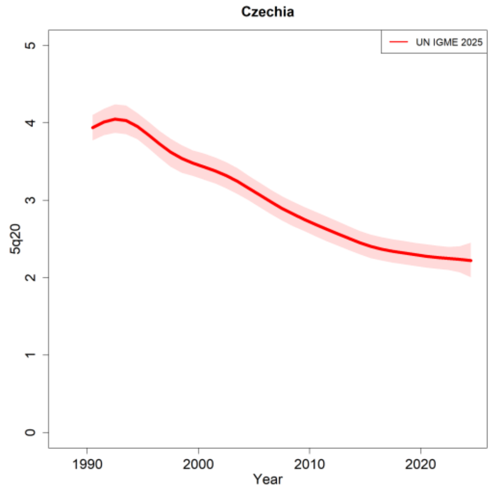
Czechia
UN IGME 2025
5q20
0
1
2
3
4
5
1990
2000
2010
2020
Year

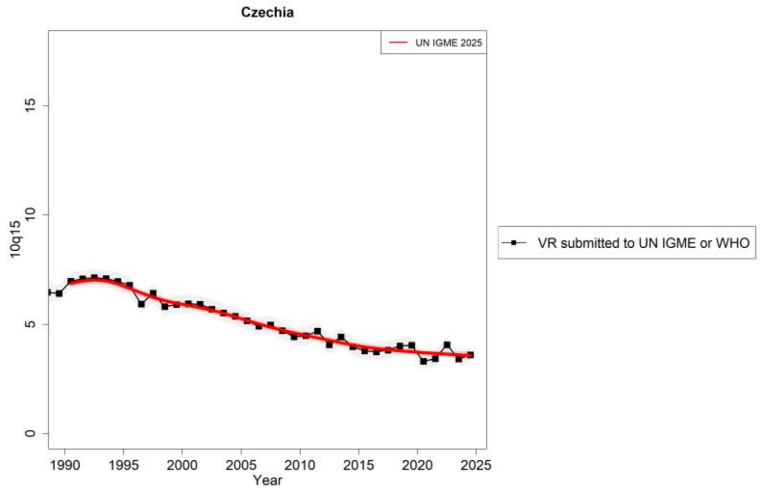
Czechia
UN IGME 2025
VR submitted to UN IGME or WHO
10q15
0
5
10
15
1990
1995
2000
2005
2010
2015
2020
2025
Year

Côte d’Ivoire (CIV)

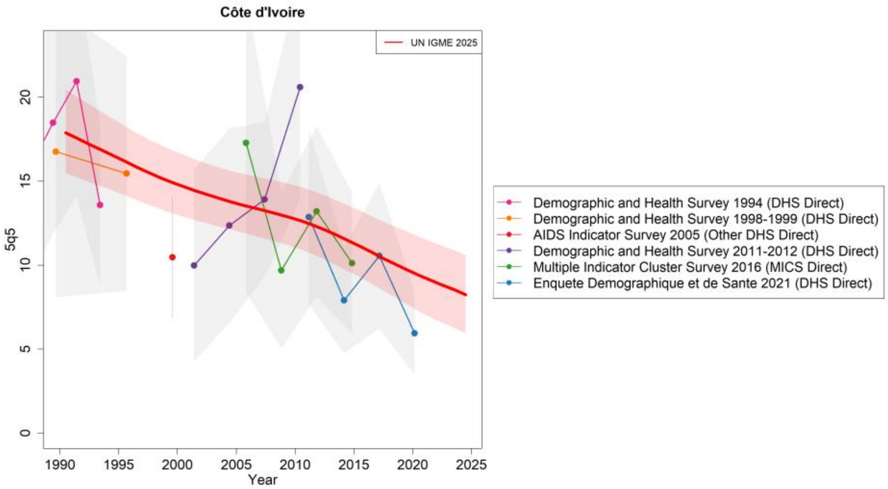

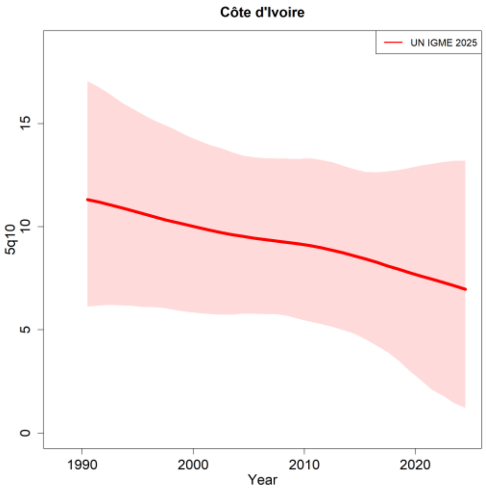

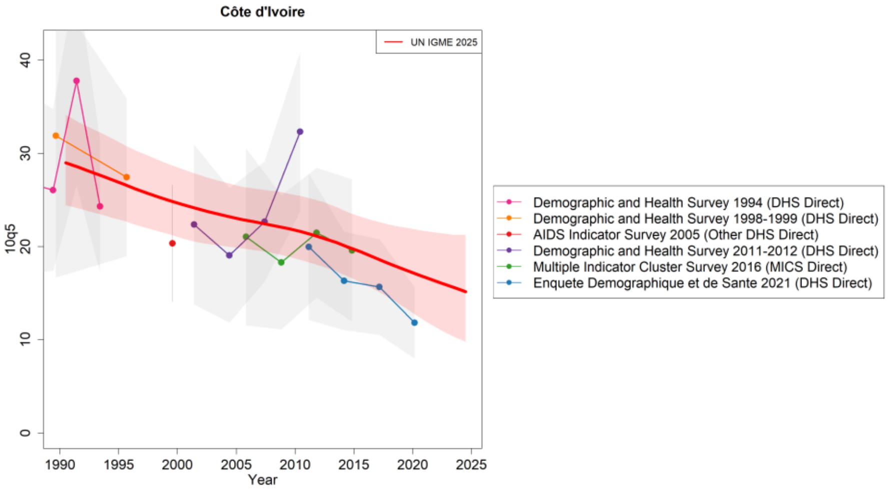

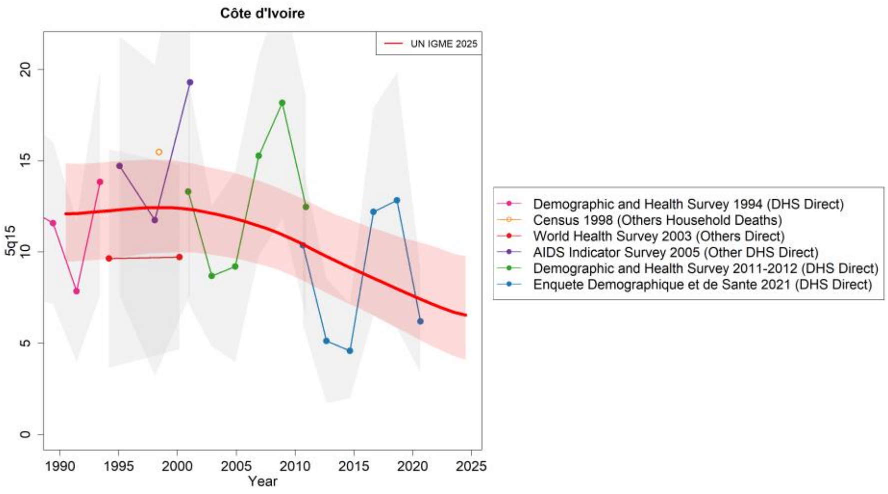

Côte d'Ivoire
UN IGME 2025
5q15
Year
Demographic and Health Survey 1994 (DHS Direct)
Census 1998 (Others Household Deaths)
World Health Survey 2003 (Others Direct)
AIDS Indicator Survey 2005 (Other DHS Direct)
Demographic and Health Survey 2011-2012 (DHS Direct)
Enquete Demographique et de Sante 2021 (DHS Direct)

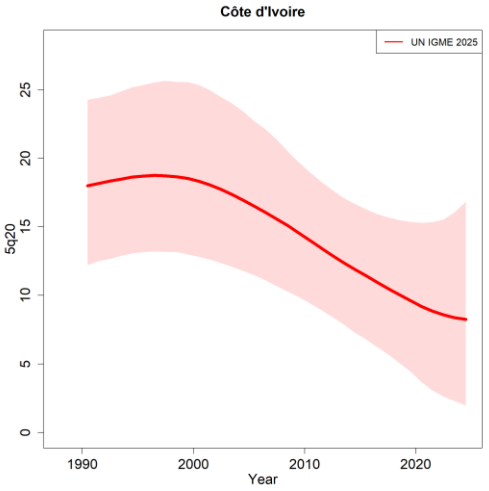

Côte d'Ivoire
UN IGME 2025
5q20
Year

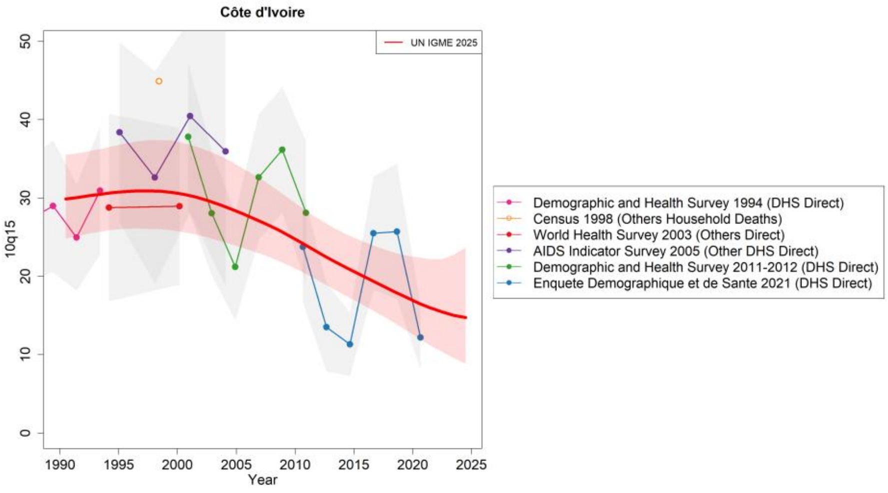

Côte d'Ivoire
UN IGME 2025
10q15
Year
Demographic and Health Survey 1994 (DHS Direct)
Census 1998 (Others Household Deaths)
World Health Survey 2003 (Others Direct)
AIDS Indicator Survey 2005 (Other DHS Direct)
Demographic and Health Survey 2011-2012 (DHS Direct)
Enquete Demographique et de Sante 2021 (DHS Direct)

Democratic People's Republic of Korea (PRK)

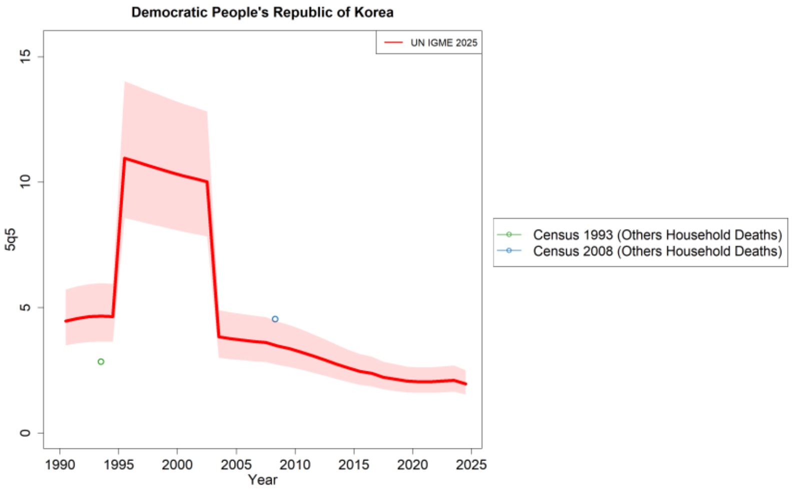

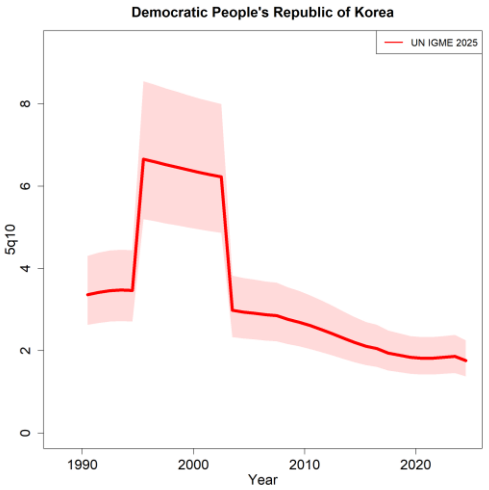

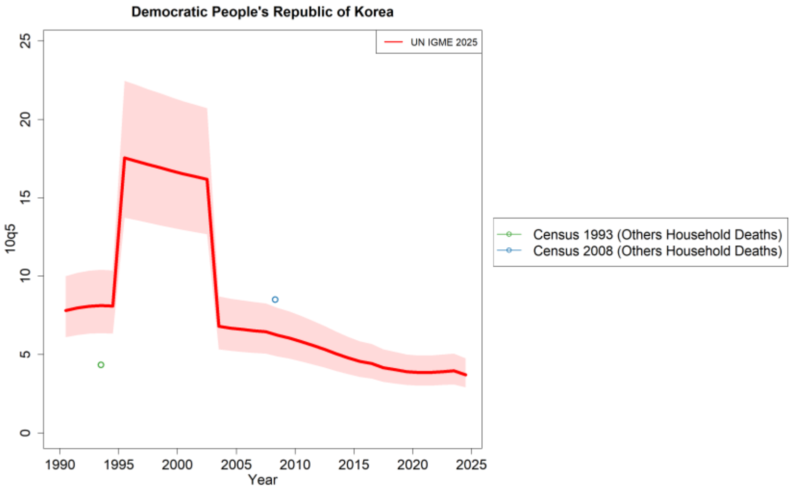

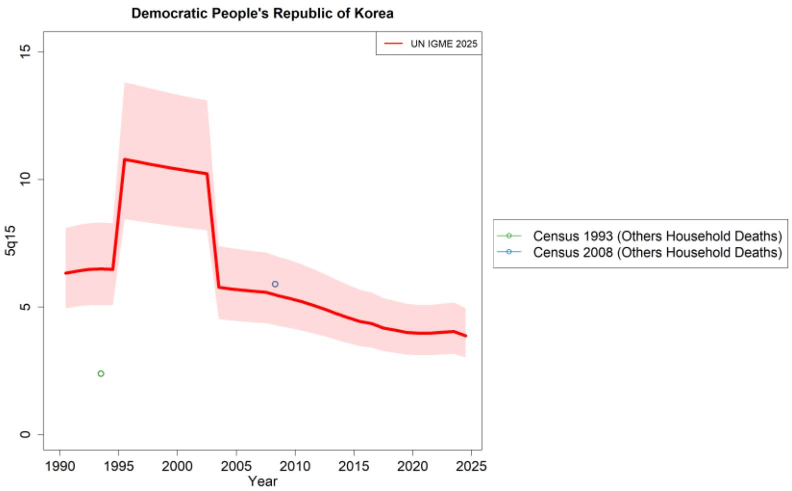
Democratic People's Republic of Korea
UN IGME 2025
Census 1993 (Others Household Deaths)
Census 2008 (Others Household Deaths)
5q15
0
5
10
15
1990
1995
2000
2005
2010
2015
2020
2025
Year

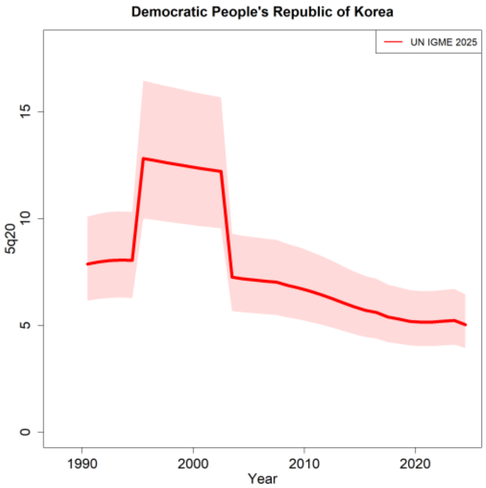
Democratic People's Republic of Korea
UN IGME 2025
5q20
0
5
10
15
1990
2000
2010
2020
Year

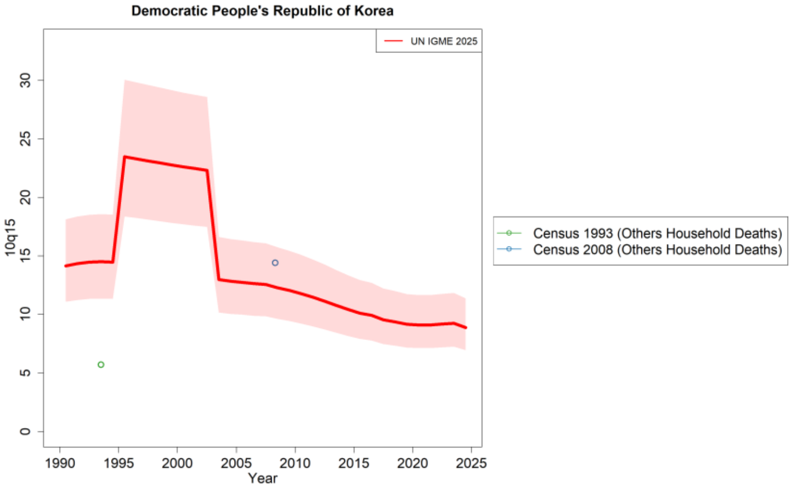
Democratic People's Republic of Korea
UN IGME 2025
Census 1993 (Others Household Deaths)
Census 2008 (Others Household Deaths)
10q15
0
5
10
15
20
25
30
1990
1995
2000
2005
2010
2015
2020
2025
Year

Democratic Republic of the Congo (COD)

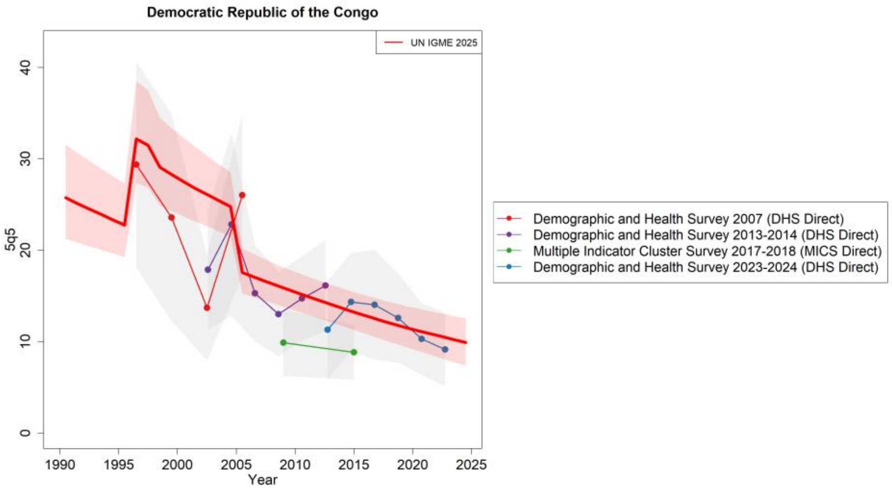

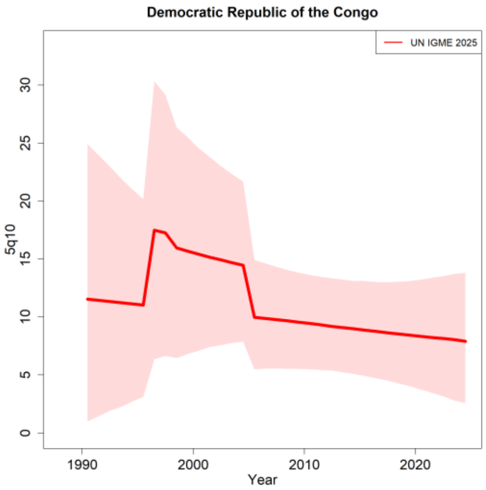

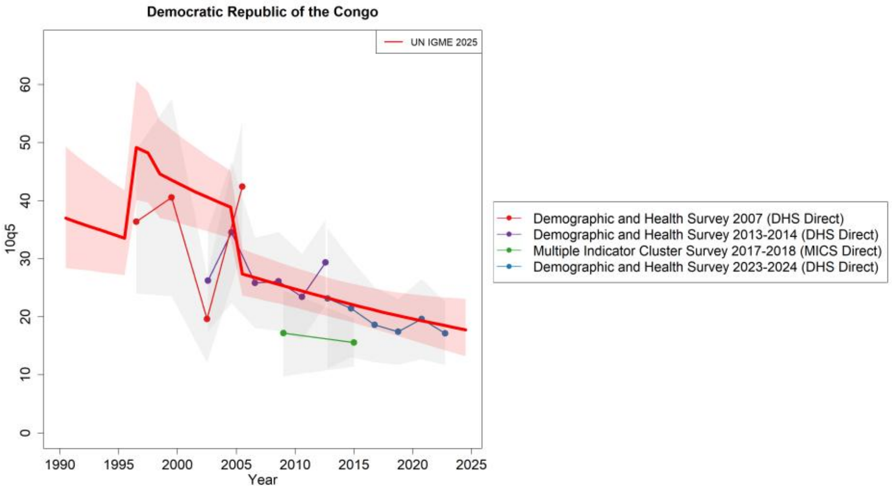

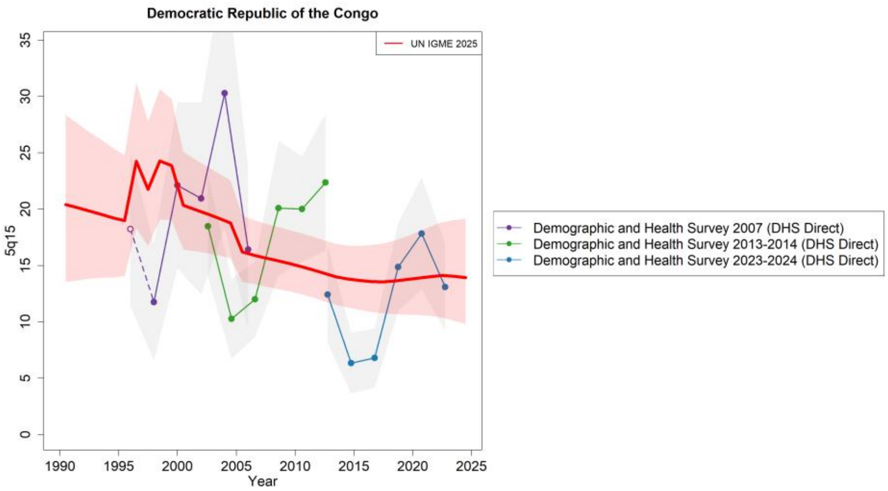
Democratic Republic of the Congo
UN IGME 2025
5q15
Year
Demographic and Health Survey 2007 (DHS Direct)
Demographic and Health Survey 2013-2014 (DHS Direct)
Demographic and Health Survey 2023-2024 (DHS Direct)

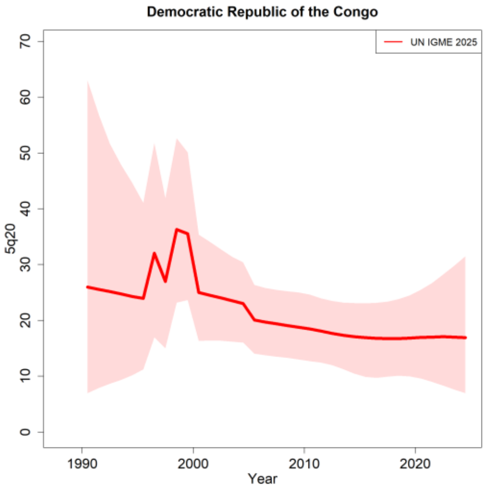
Democratic Republic of the Congo
UN IGME 2025
5q20
Year

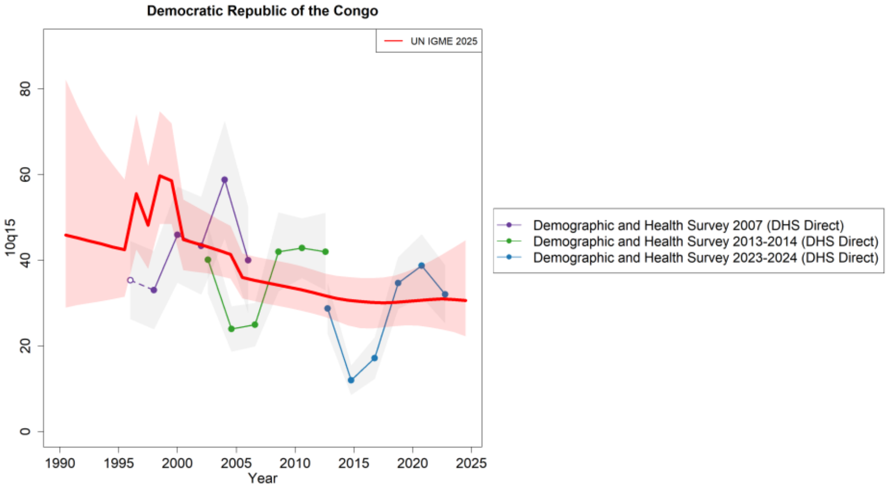
Democratic Republic of the Congo
UN IGME 2025
10q15
Year
Demographic and Health Survey 2007 (DHS Direct)
Demographic and Health Survey 2013-2014 (DHS Direct)
Demographic and Health Survey 2023-2024 (DHS Direct)

Denmark (DNK)

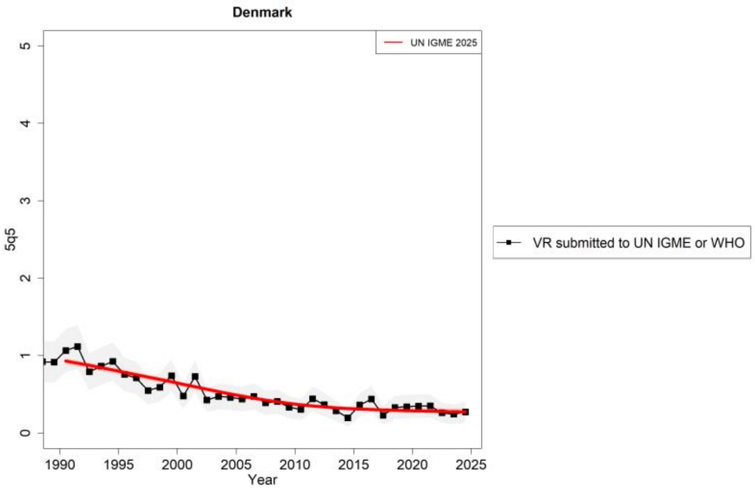
Denmark
UN IGME 2025
VR submitted to UN IGME or WHO
5q5
0
1
2
3
4
5
1990
1995
2000
2005
2010
2015
2020
2025
Year

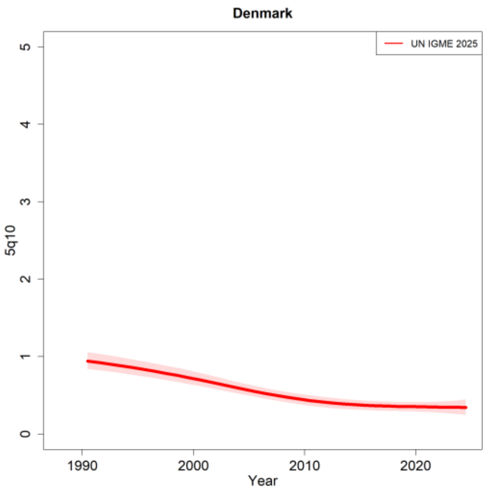
Denmark
UN IGME 2025
5q10
0
1
2
3
4
5
1990
2000
2010
2020
Year

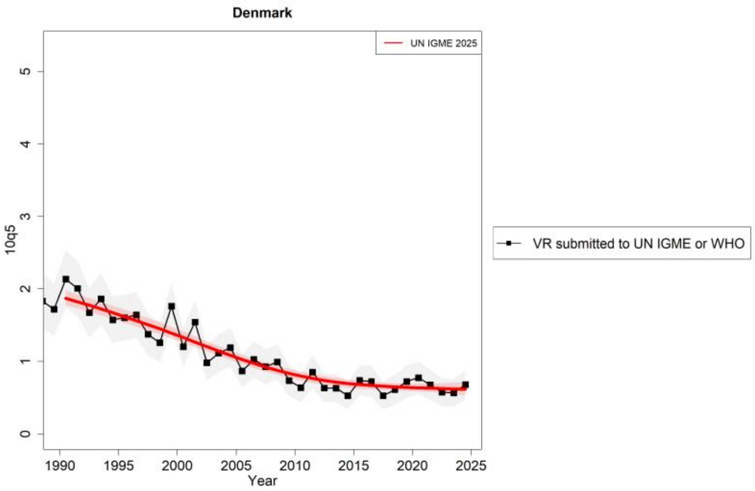
Denmark
UN IGME 2025
VR submitted to UN IGME or WHO
10q5
0
1
2
3
4
5
1990
1995
2000
2005
2010
2015
2020
2025
Year

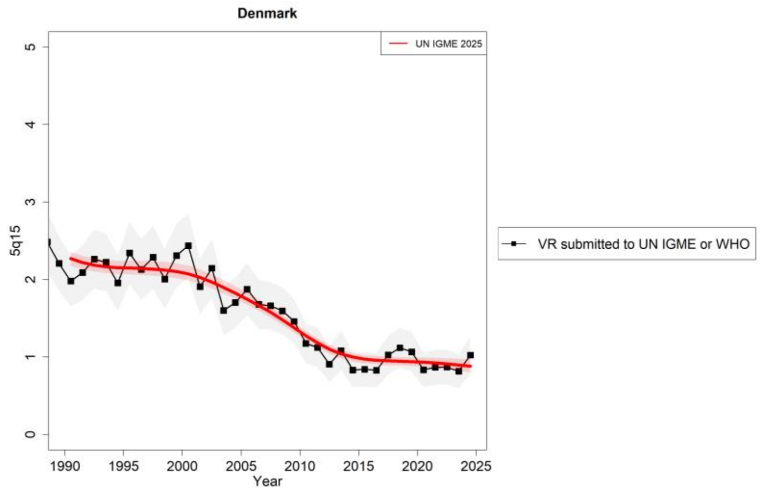
Denmark
UN IGME 2025
VR submitted to UN IGME or WHO
5q15
Year

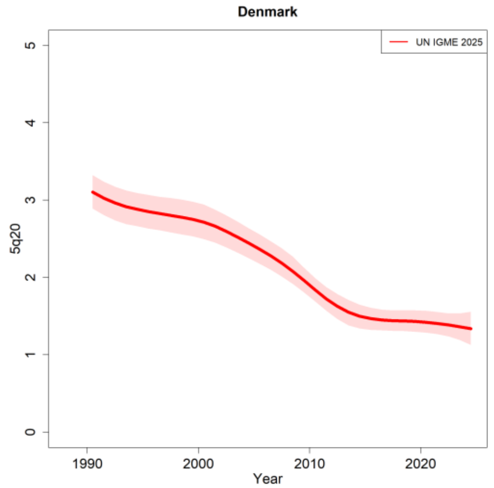
Denmark
UN IGME 2025
5q20
Year

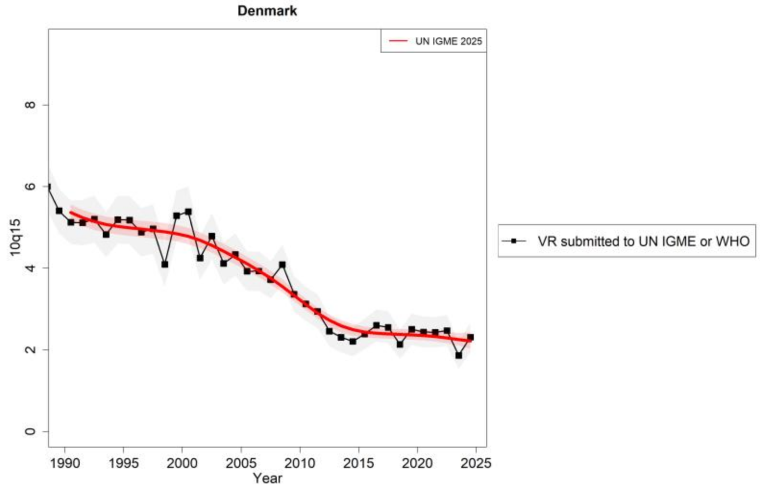
Denmark
UN IGME 2025
VR submitted to UN IGME or WHO
10q15
Year

Djibouti (DJI)

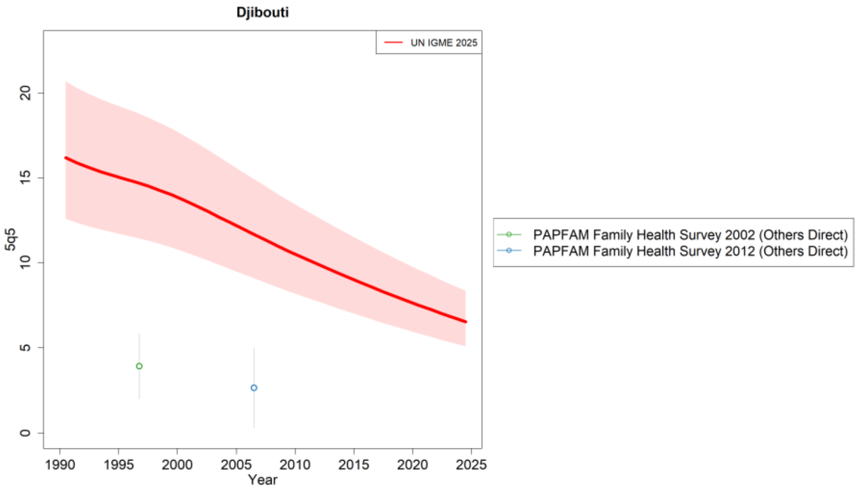

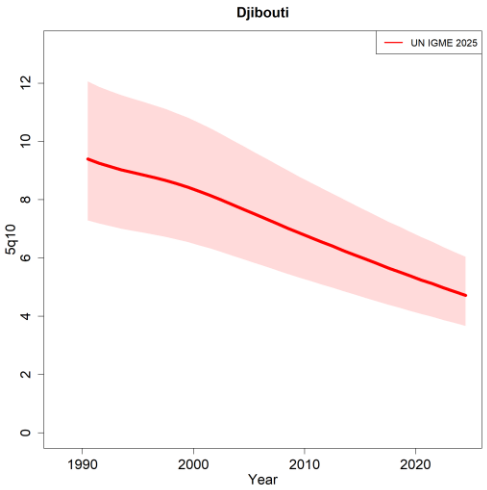

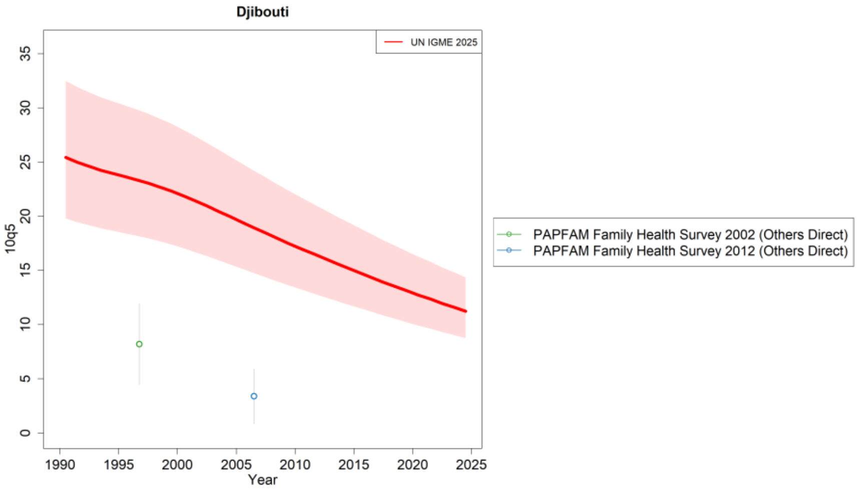

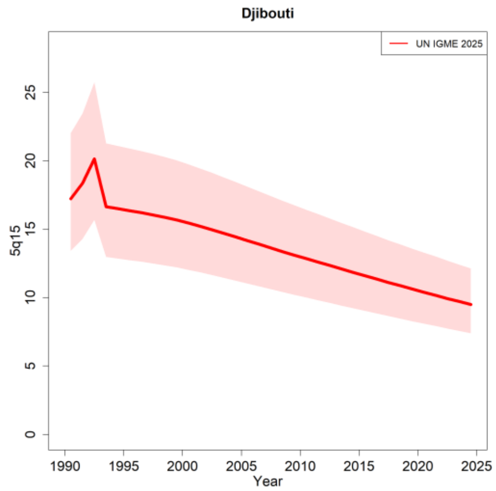
Djibouti
UN IGME 2025
5q15
0
5
10
15
20
25
1990
1995
2000
2005
2010
2015
2020
2025
Year

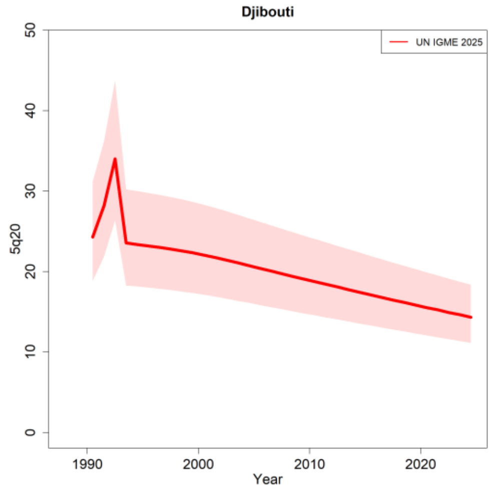
Djibouti
UN IGME 2025
5q20
0
10
20
30
40
50
1990
2000
2010
2020
Year

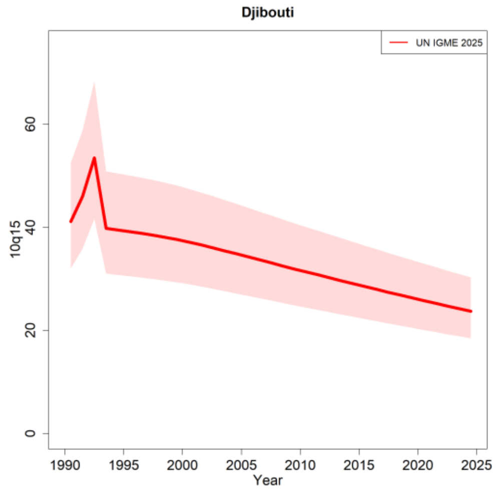
Djibouti
UN IGME 2025
10q15
0
20
40
60
1990
1995
2000
2005
2010
2015
2020
2025
Year

Dominica (DMA)

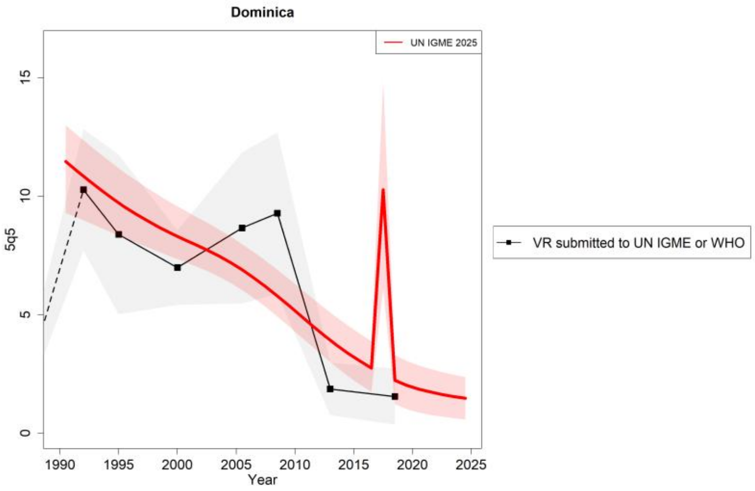

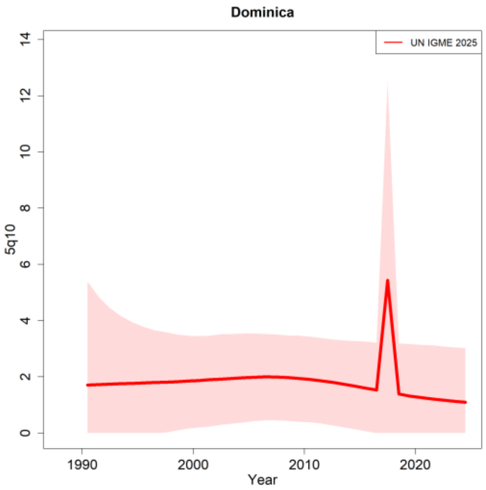

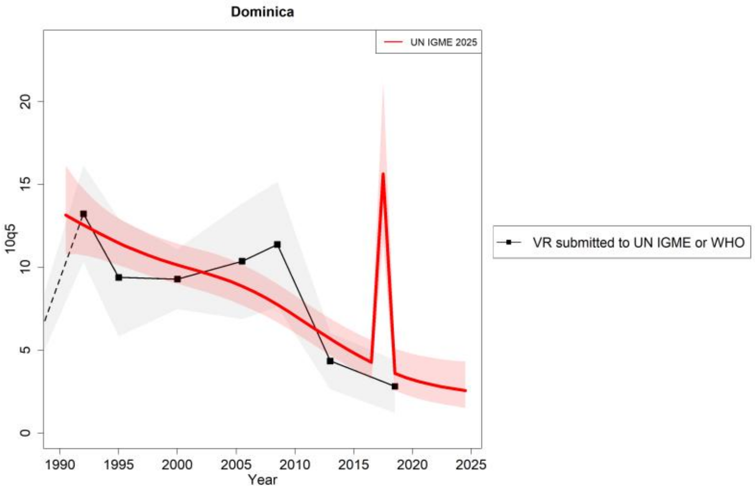

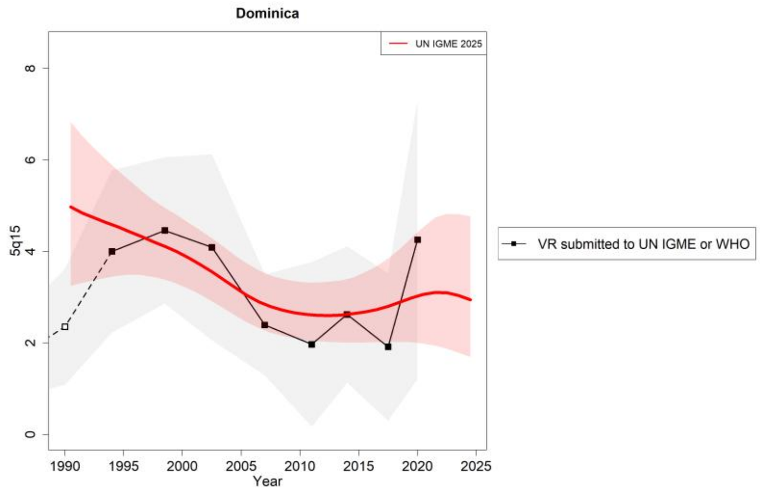
Dominica
UN IGME 2025
VR submitted to UN IGME or WHO
5q15
0
2
4
6
8
1990
1995
2000
2005
2010
2015
2020
2025
Year

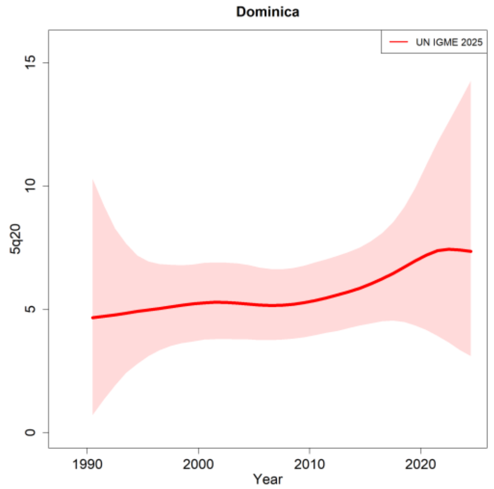
Dominica
UN IGME 2025
5q20
0
5
10
15
1990
2000
2010
2020
Year

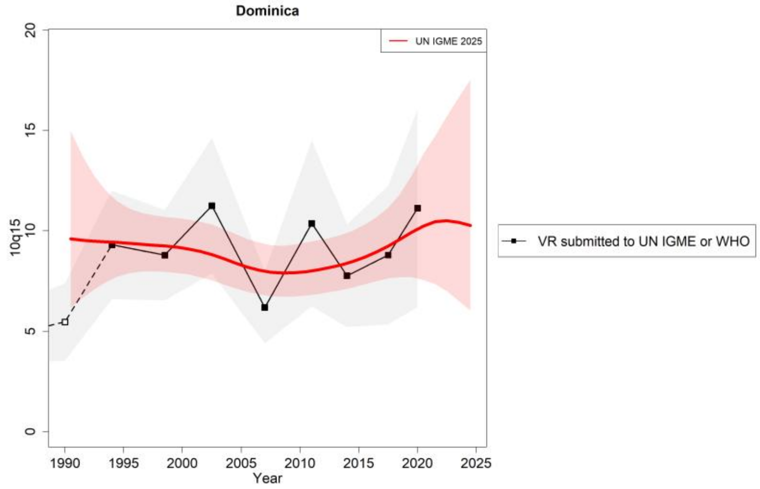
Dominica
UN IGME 2025
VR submitted to UN IGME or WHO
10q15
0
5
10
15
20
1990
1995
2000
2005
2010
2015
2020
2025
Year

Dominican Republic (DOM)

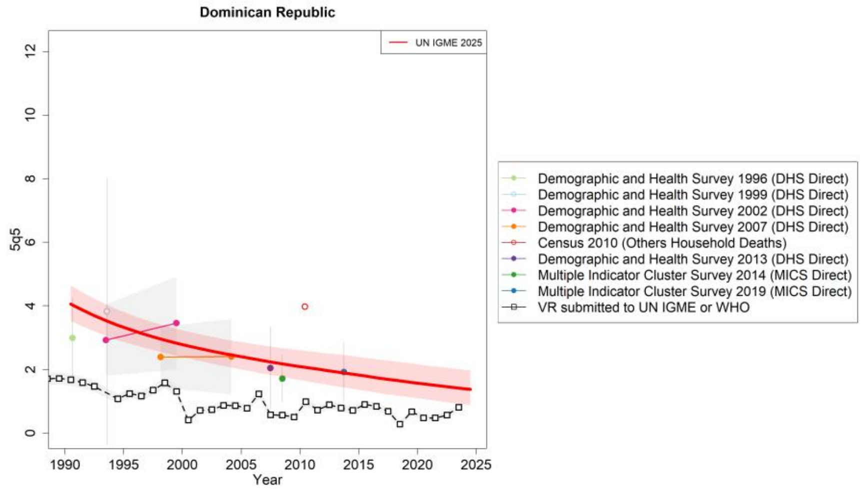

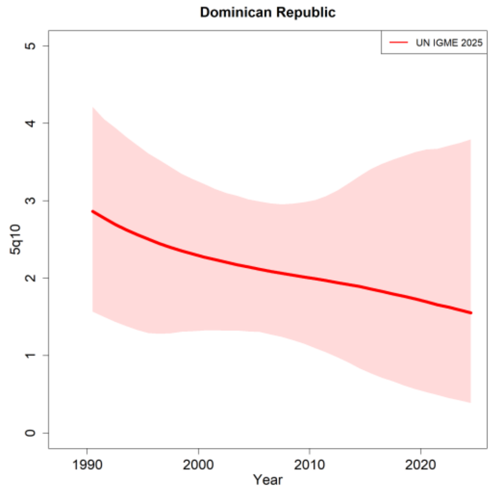

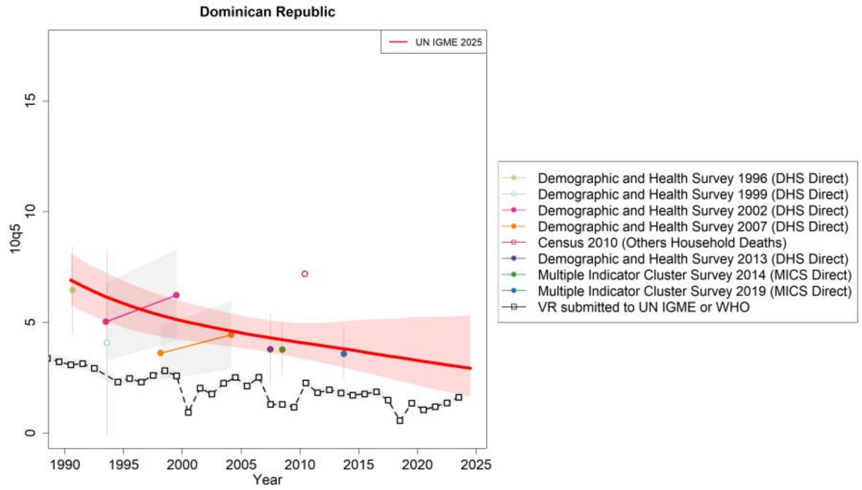

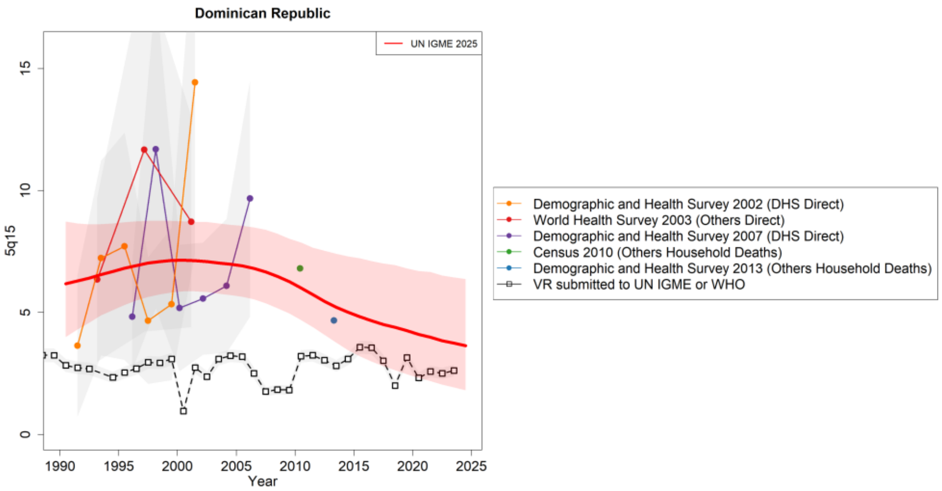
Dominican Republic
UN IGME 2025
5q15
Year
0
5
10
15
1990
1995
2000
2005
2010
2015
2020
2025
Demographic and Health Survey 2002 (DHS Direct)
World Health Survey 2003 (Others Direct)
Demographic and Health Survey 2007 (DHS Direct)
Census 2010 (Others Household Deaths)
Demographic and Health Survey 2013 (Others Household Deaths)
VR submitted to UN IGME or WHO

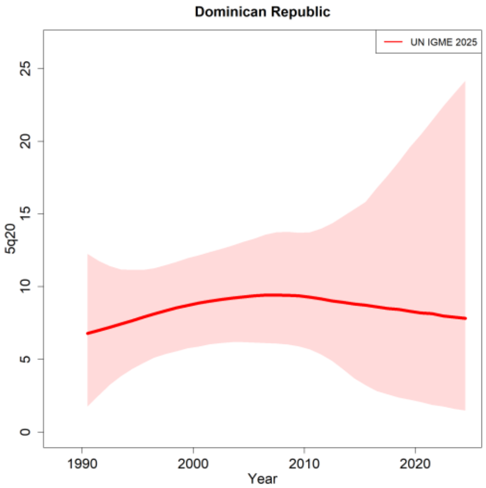
Dominican Republic
UN IGME 2025
5q20
Year
0
5
10
15
20
25
1990
2000
2010
2020

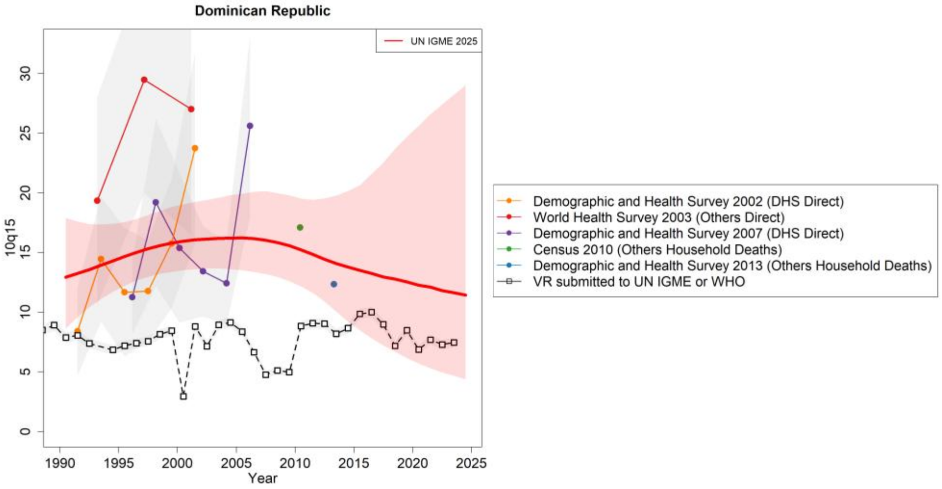
Dominican Republic
UN IGME 2025
10q15
Year
0
5
10
15
20
25
30
1990
1995
2000
2005
2010
2015
2020
2025
Demographic and Health Survey 2002 (DHS Direct)
World Health Survey 2003 (Others Direct)
Demographic and Health Survey 2007 (DHS Direct)
Census 2010 (Others Household Deaths)
Demographic and Health Survey 2013 (Others Household Deaths)
VR submitted to UN IGME or WHO

Ecuador (ECU)

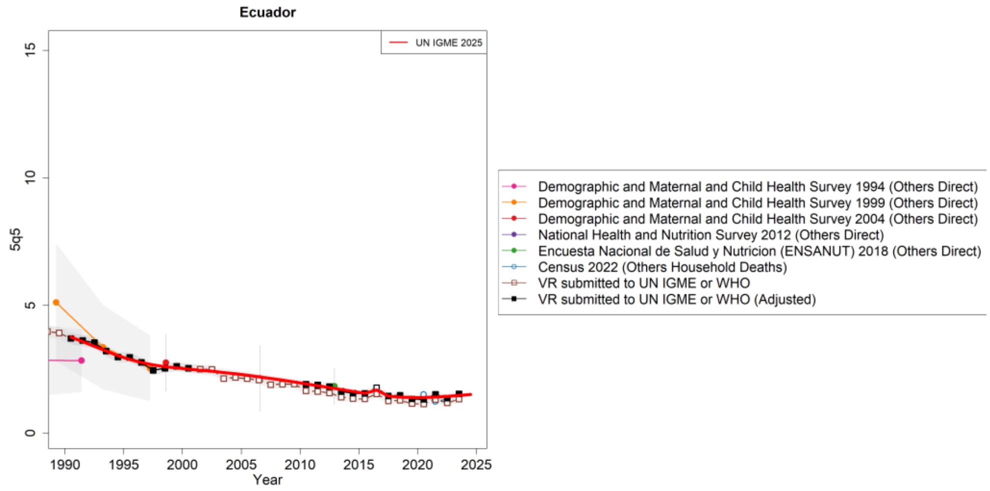

Ecuador
UN IGME 2025
5q5
Year
Demographic and Maternal and Child Health Survey 1994 (Others Direct)
Demographic and Maternal and Child Health Survey 1999 (Others Direct)
Demographic and Maternal and Child Health Survey 2004 (Others Direct)
National Health and Nutrition Survey 2012 (Others Direct)
Encuesta Nacional de Salud y Nutricion (ENSANUT) 2018 (Others Direct)
Census 2022 (Others Household Deaths)
VR submitted to UN IGME or WHO
VR submitted to UN IGME or WHO (Adjusted)

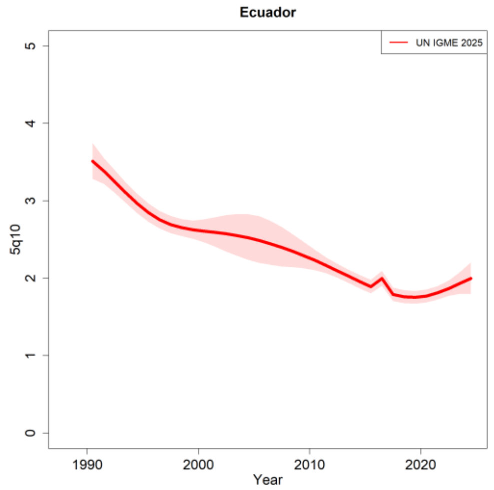

Ecuador
UN IGME 2025
5q10
Year

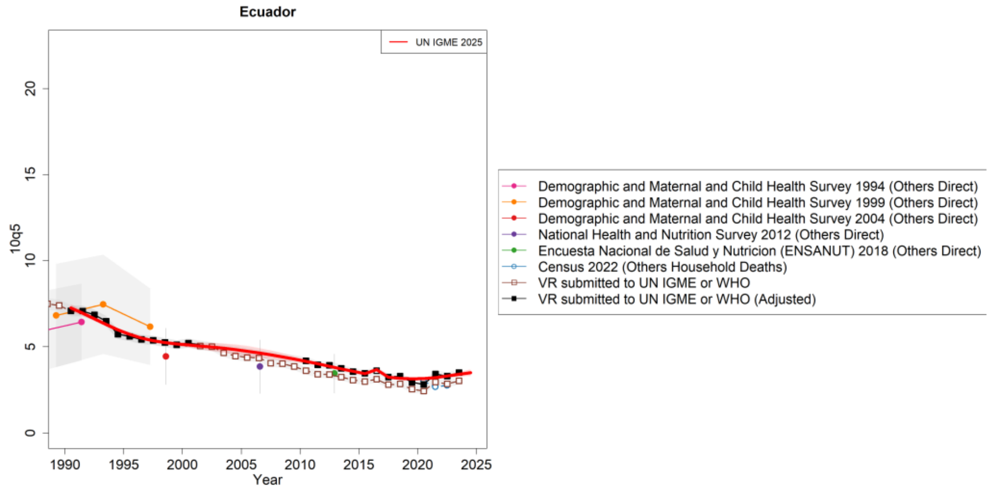

Ecuador
UN IGME 2025
10q5
Year
Demographic and Maternal and Child Health Survey 1994 (Others Direct)
Demographic and Maternal and Child Health Survey 1999 (Others Direct)
Demographic and Maternal and Child Health Survey 2004 (Others Direct)
National Health and Nutrition Survey 2012 (Others Direct)
Encuesta Nacional de Salud y Nutricion (ENSANUT) 2018 (Others Direct)
Census 2022 (Others Household Deaths)
VR submitted to UN IGME or WHO
VR submitted to UN IGME or WHO (Adjusted)

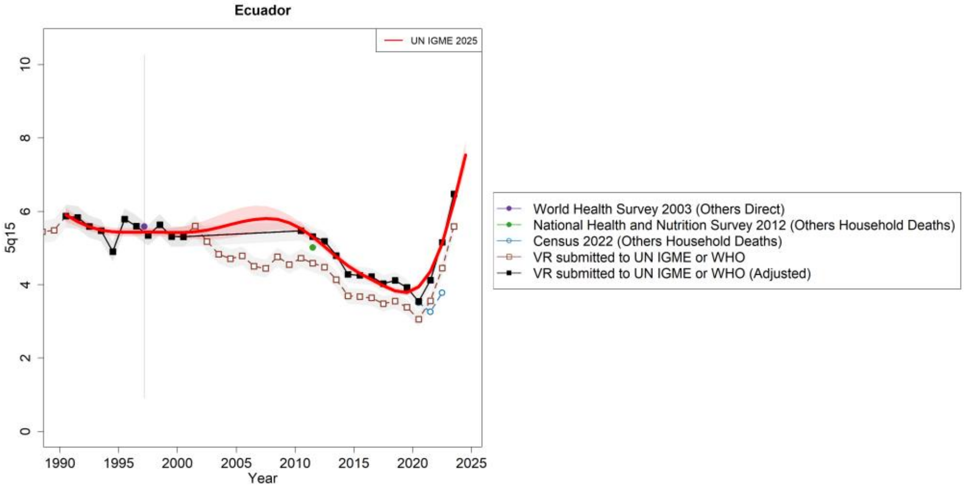
Ecuador
UN IGME 2025
5q15
Year
World Health Survey 2003 (Others Direct)
National Health and Nutrition Survey 2012 (Others Household Deaths)
Census 2022 (Others Household Deaths)
VR submitted to UN IGME or WHO
VR submitted to UN IGME or WHO (Adjusted)

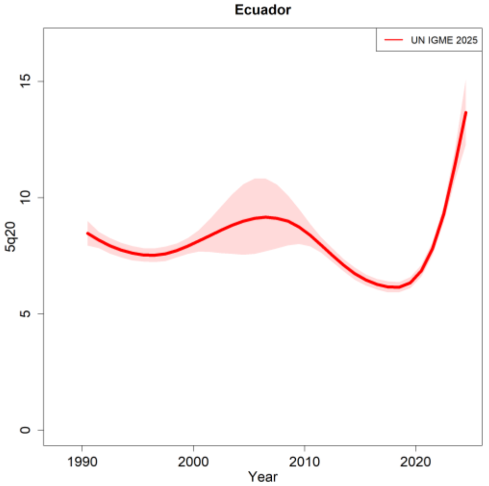
Ecuador
UN IGME 2025
5q20
Year

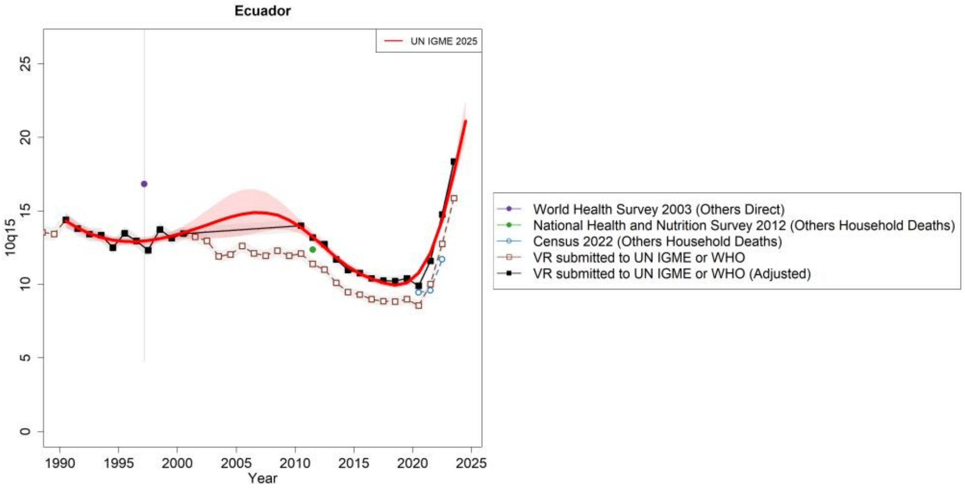
Ecuador
UN IGME 2025
10q15
Year
World Health Survey 2003 (Others Direct)
National Health and Nutrition Survey 2012 (Others Household Deaths)
Census 2022 (Others Household Deaths)
VR submitted to UN IGME or WHO
VR submitted to UN IGME or WHO (Adjusted)

Egypt (EGY)

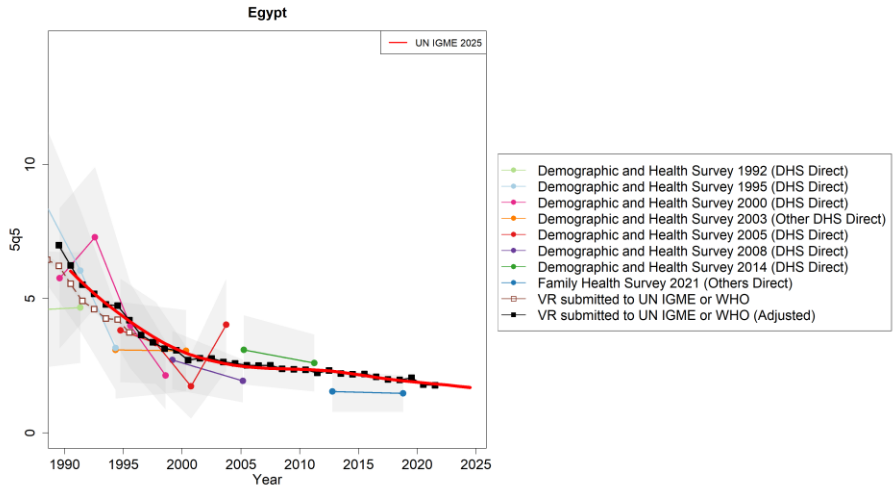
Egypt
UN IGME 2025
5q5
0
5
10
1990
1995
2000
2005
2010
2015
2020
2025
Year
Demographic and Health Survey 1992 (DHS Direct)
Demographic and Health Survey 1995 (DHS Direct)
Demographic and Health Survey 2000 (DHS Direct)
Demographic and Health Survey 2003 (Other DHS Direct)
Demographic and Health Survey 2005 (DHS Direct)
Demographic and Health Survey 2008 (DHS Direct)
Demographic and Health Survey 2014 (DHS Direct)
Family Health Survey 2021 (Others Direct)
VR submitted to UN IGME or WHO
VR submitted to UN IGME or WHO (Adjusted)

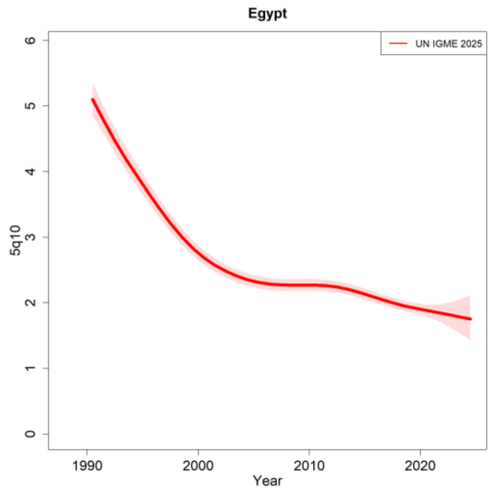
Egypt
UN IGME 2025
5q10
0
1
2
3
4
5
6
1990
2000
2010
2020
Year

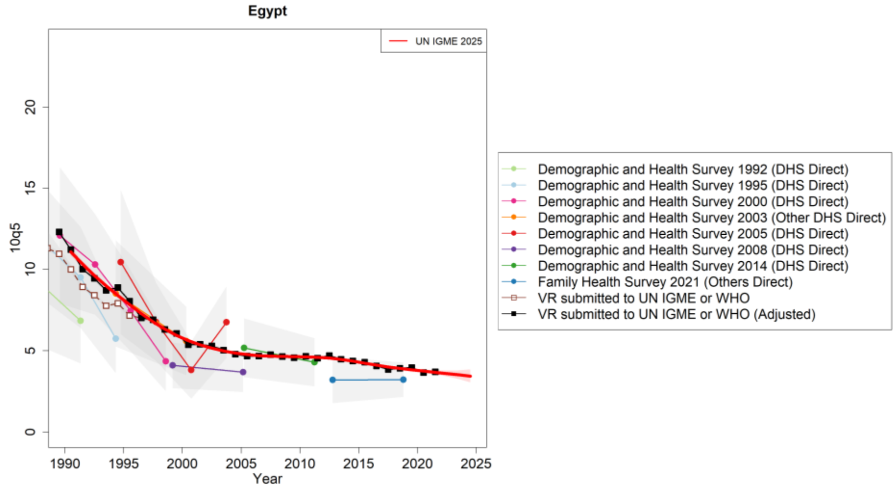
Egypt
UN IGME 2025
10q5
0
5
10
15
20
1990
1995
2000
2005
2010
2015
2020
2025
Year
Demographic and Health Survey 1992 (DHS Direct)
Demographic and Health Survey 1995 (DHS Direct)
Demographic and Health Survey 2000 (DHS Direct)
Demographic and Health Survey 2003 (Other DHS Direct)
Demographic and Health Survey 2005 (DHS Direct)
Demographic and Health Survey 2008 (DHS Direct)
Demographic and Health Survey 2014 (DHS Direct)
Family Health Survey 2021 (Others Direct)
VR submitted to UN IGME or WHO
VR submitted to UN IGME or WHO (Adjusted)

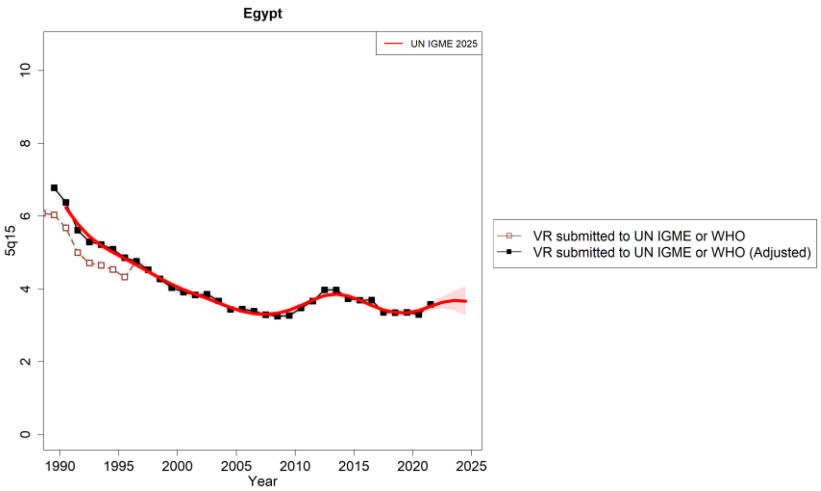
Egypt
UN IGME 2025
VR submitted to UN IGME or WHO
VR submitted to UN IGME or WHO (Adjusted)
5q15
0
2
4
6
8
10
1990
1995
2000
2005
2010
2015
2020
2025
Year

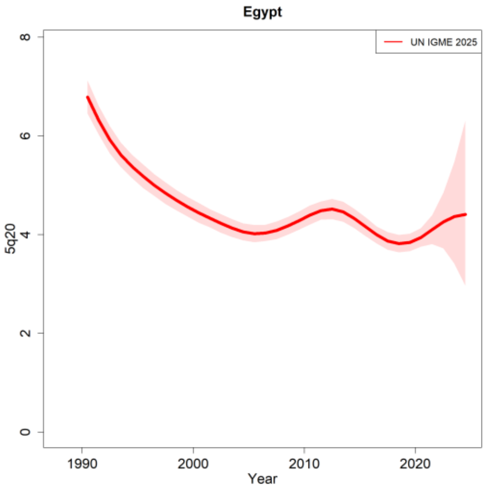
Egypt
UN IGME 2025
5q20
0
2
4
6
8
1990
2000
2010
2020
Year

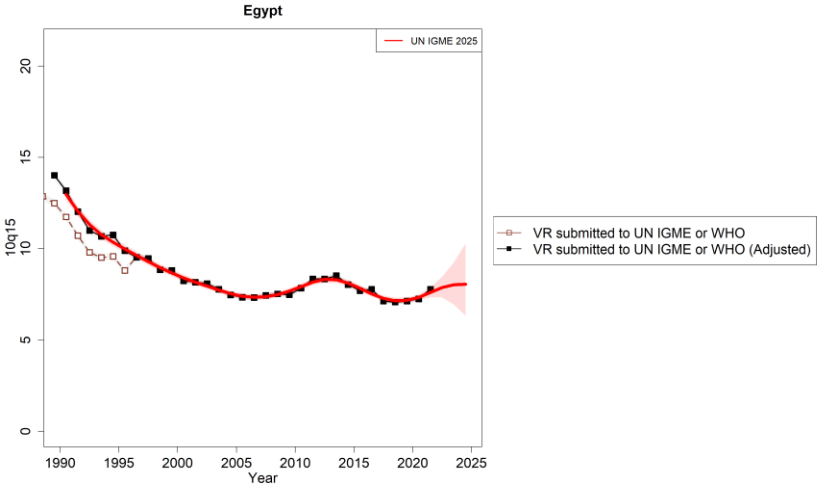
Egypt
UN IGME 2025
VR submitted to UN IGME or WHO
VR submitted to UN IGME or WHO (Adjusted)
10q15
0
5
10
15
20
1990
1995
2000
2005
2010
2015
2020
2025
Year

El Salvador (SLV)

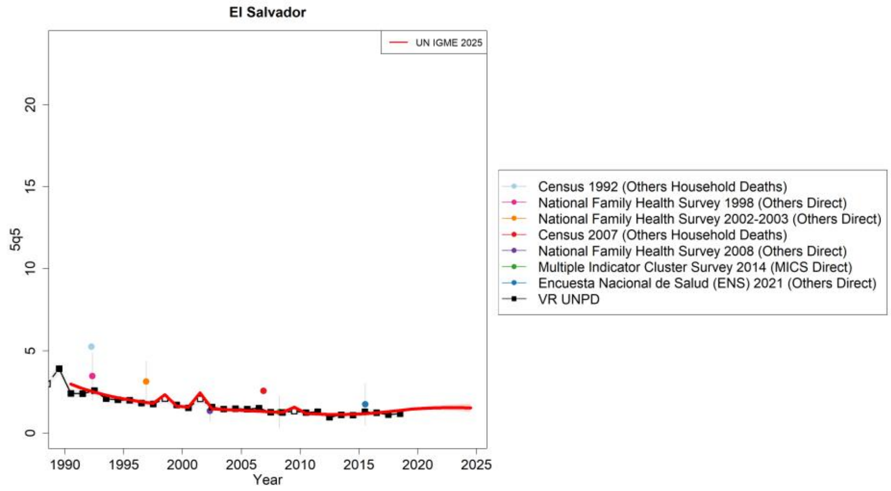

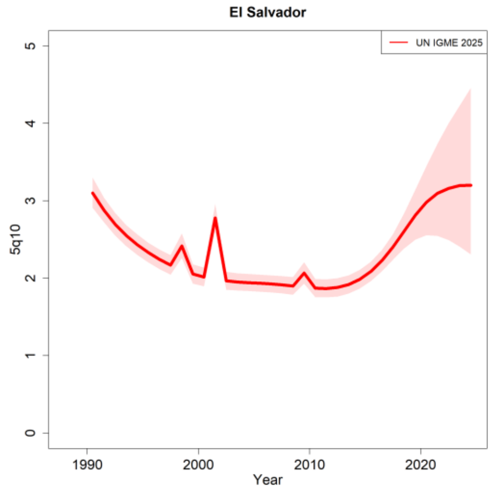

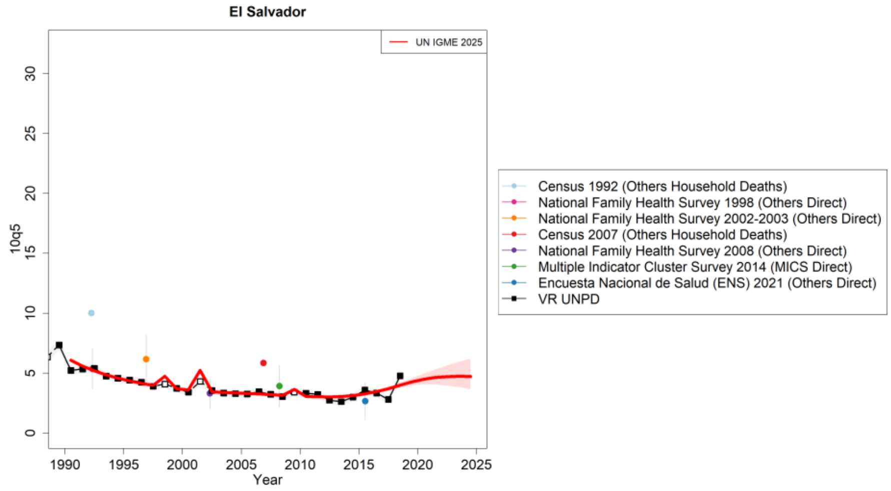

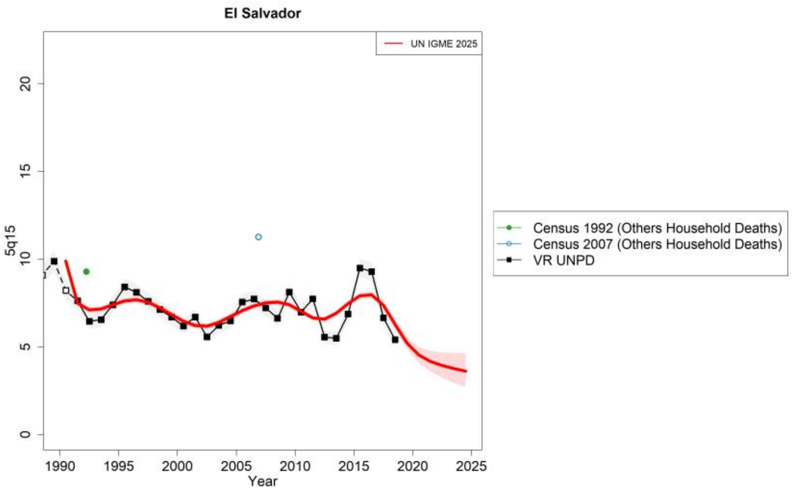
El Salvador
UN IGME 2025
Census 1992 (Others Household Deaths)
Census 2007 (Others Household Deaths)
VR UNPD
5q15
0
5
10
15
20
1990
1995
2000
2005
2010
2015
2020
2025
Year

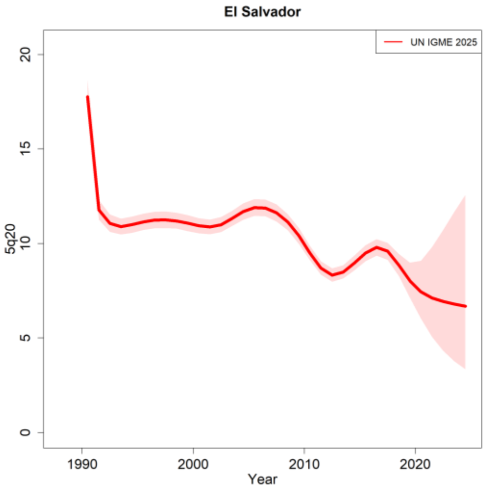
El Salvador
UN IGME 2025
5q20
0
5
10
15
20
1990
2000
2010
2020
Year

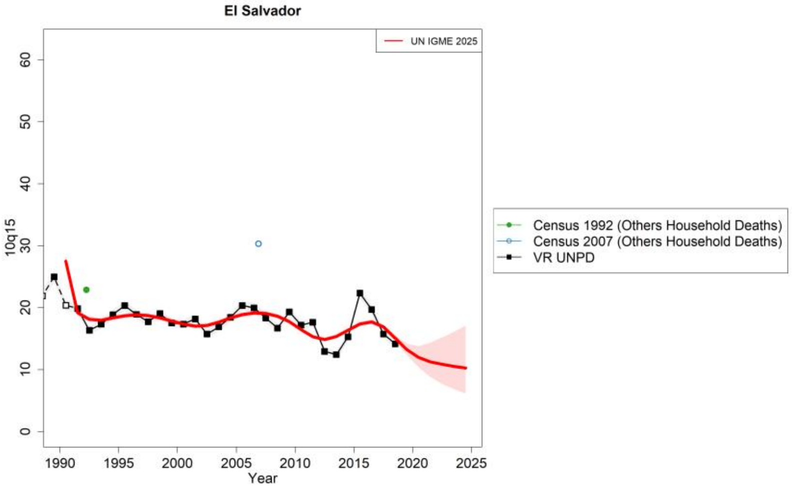
El Salvador
UN IGME 2025
Census 1992 (Others Household Deaths)
Census 2007 (Others Household Deaths)
VR UNPD
10q15
0
10
20
30
40
50
60
1990
1995
2000
2005
2010
2015
2020
2025
Year

Equatorial Guinea (GNQ)

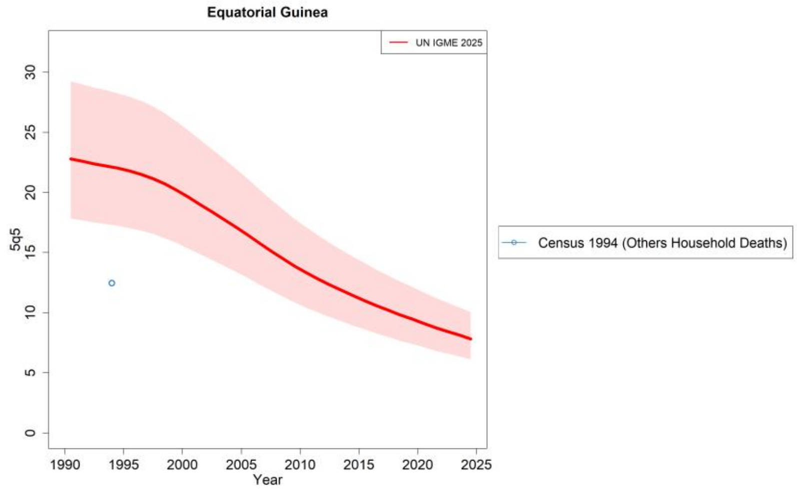

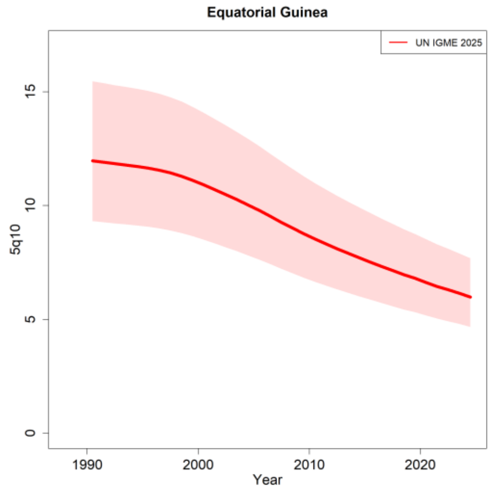

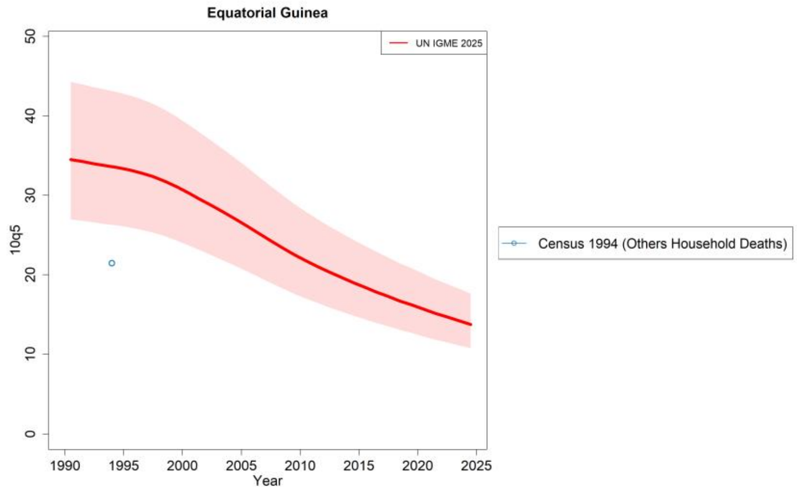

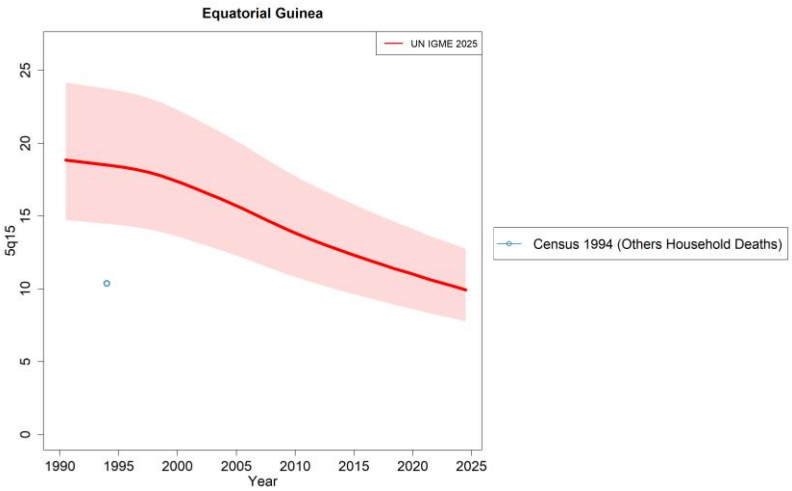
Equatorial Guinea
UN IGME 2025
Census 1994 (Others Household Deaths)
5q15
Year
0
5
10
15
20
25
1990
1995
2000
2005
2010
2015
2020
2025

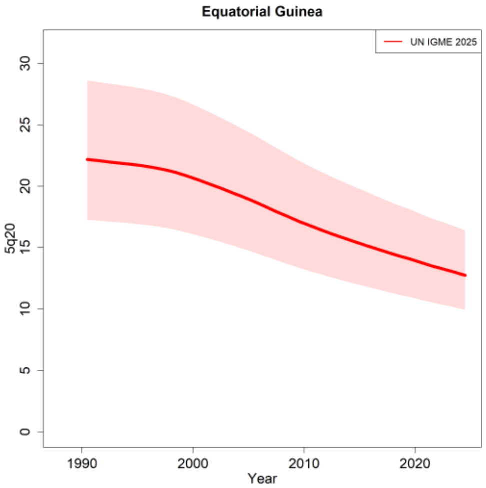
Equatorial Guinea
UN IGME 2025
5q20
Year
0
5
10
15
20
25
30
1990
2000
2010
2020

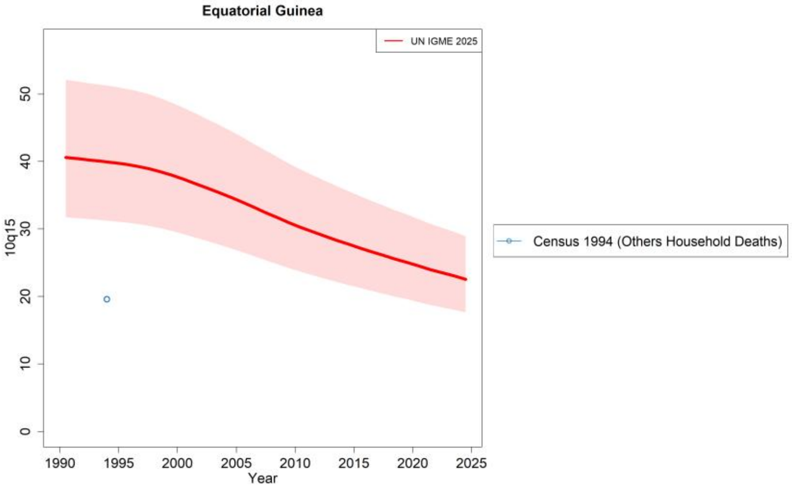
Equatorial Guinea
UN IGME 2025
Census 1994 (Others Household Deaths)
10q15
Year
0
10
20
30
40
50
1990
1995
2000
2005
2010
2015
2020
2025

Eritrea (ERI)

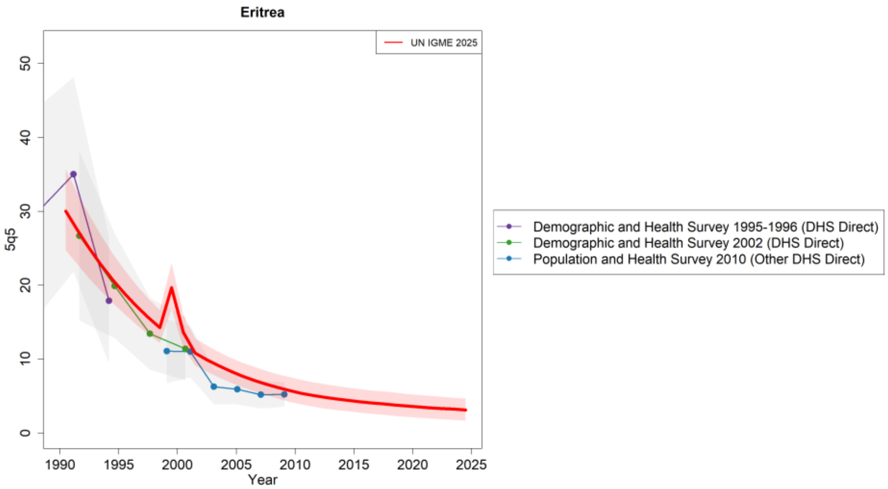
Eritrea
UN IGME 2025
5q5
0
10
20
30
40
50
1990
1995
2000
2005
2010
2015
2020
2025
Year
Demographic and Health Survey 1995-1996 (DHS Direct)
Demographic and Health Survey 2002 (DHS Direct)
Population and Health Survey 2010 (Other DHS Direct)

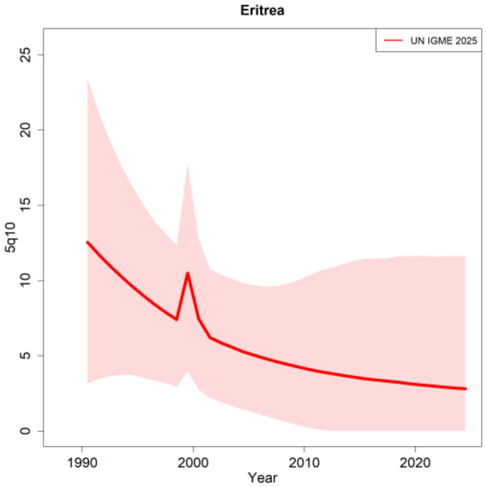
Eritrea
UN IGME 2025
5q10
0
5
10
15
20
25
1990
2000
2010
2020
Year

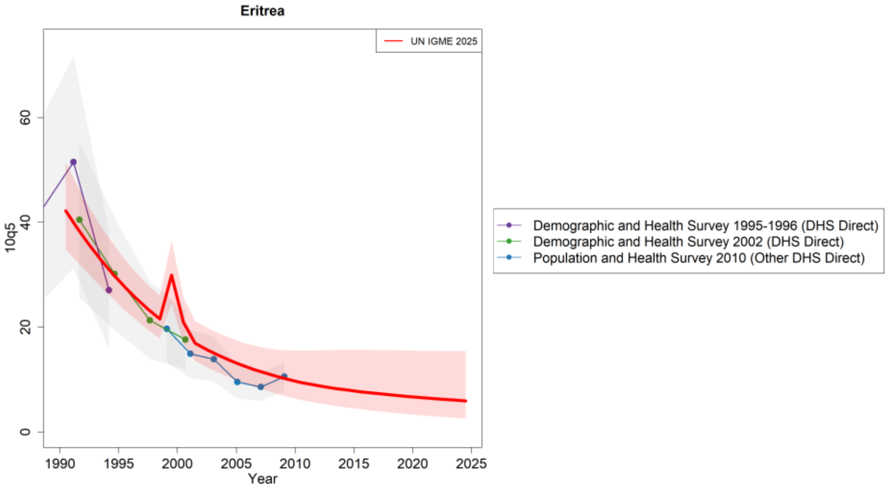
Eritrea
UN IGME 2025
10q5
0
20
40
60
1990
1995
2000
2005
2010
2015
2020
2025
Year
Demographic and Health Survey 1995-1996 (DHS Direct)
Demographic and Health Survey 2002 (DHS Direct)
Population and Health Survey 2010 (Other DHS Direct)

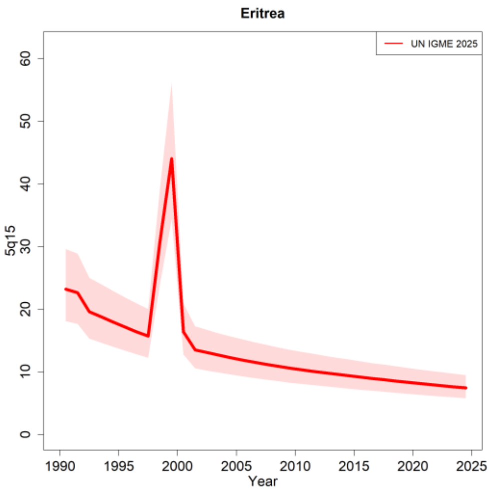
Eritrea
UN IGME 2025
5q15
0
10
20
30
40
50
60
1990
1995
2000
2005
2010
2015
2020
2025
Year

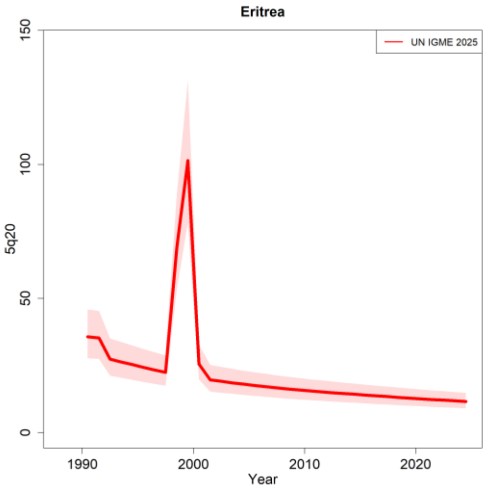
Eritrea
UN IGME 2025
5q20
0
50
100
150
1990
2000
2010
2020
Year

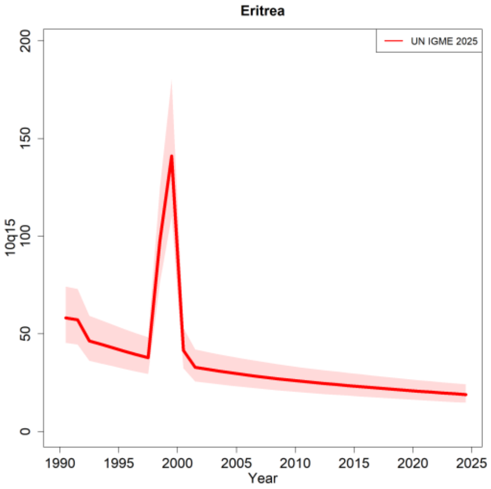
Eritrea
UN IGME 2025
10q15
0
50
100
150
200
1990
1995
2000
2005
2010
2015
2020
2025
Year

Estonia (EST)

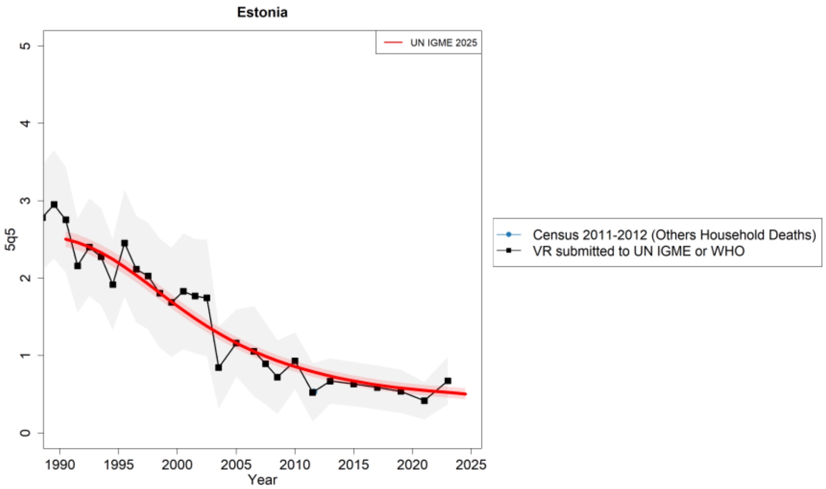
Estonia
UN IGME 2025
5q5
Year
Census 2011-2012 (Others Household Deaths)
VR submitted to UN IGME or WHO

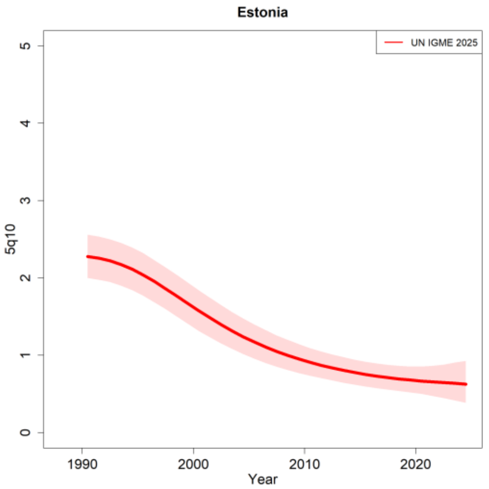
Estonia
UN IGME 2025
5q10
Year

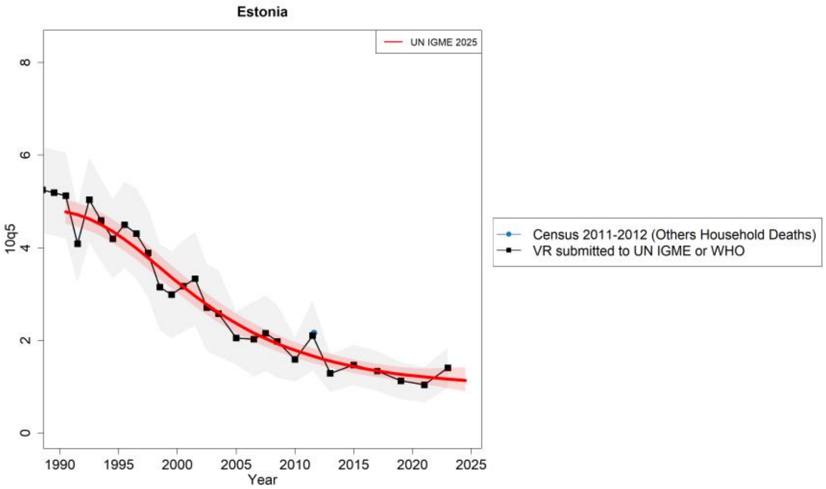
Estonia
UN IGME 2025
10q5
Year
Census 2011-2012 (Others Household Deaths)
VR submitted to UN IGME or WHO

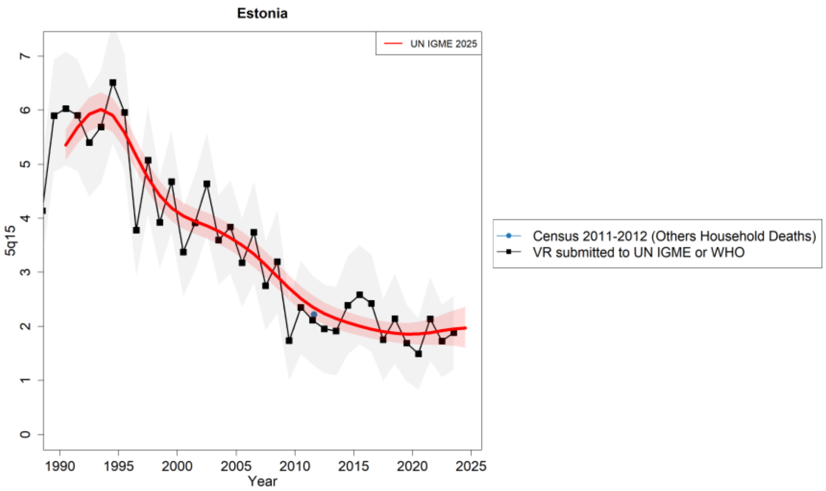
Estonia
UN IGME 2025
5q15
Year
Census 2011-2012 (Others Household Deaths)
VR submitted to UN IGME or WHO

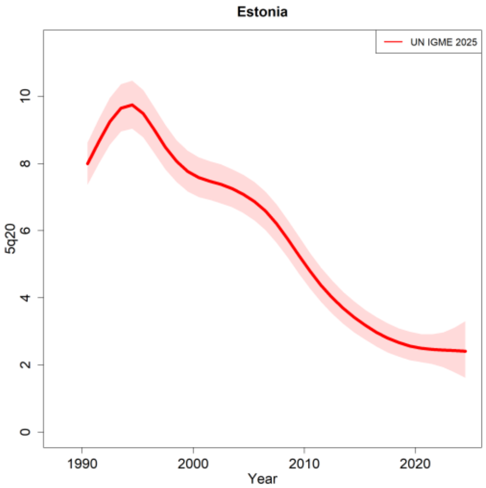
Estonia
UN IGME 2025
5q20
Year

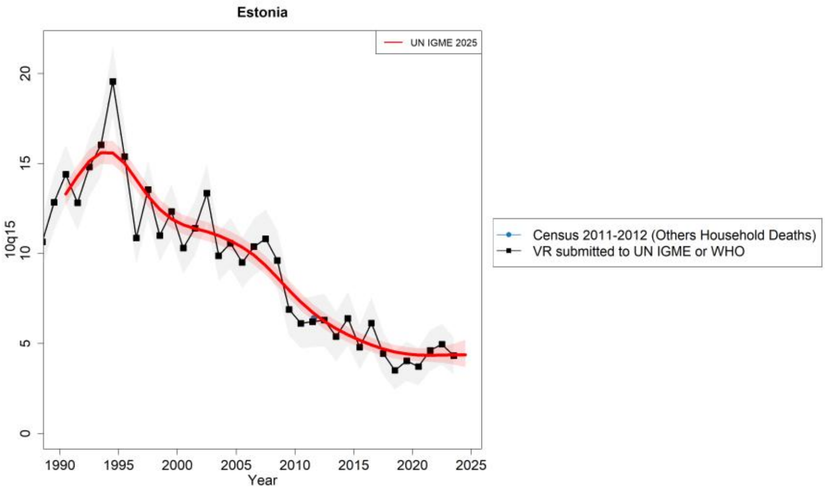
Estonia
UN IGME 2025
10q15
Year
Census 2011-2012 (Others Household Deaths)
VR submitted to UN IGME or WHO

Eswatini (SWZ)

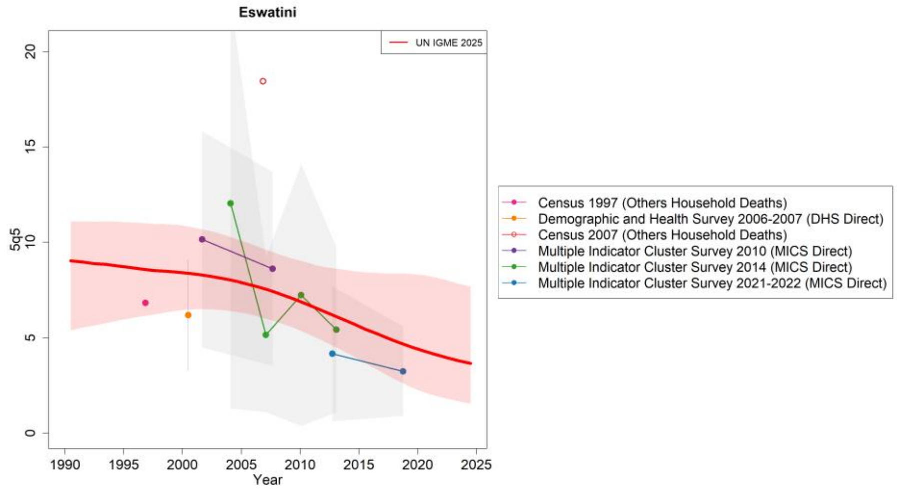

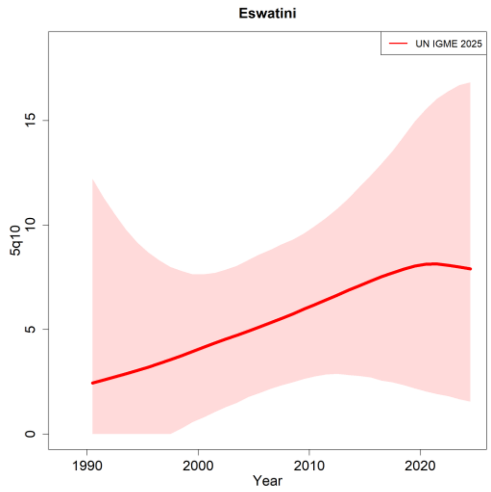

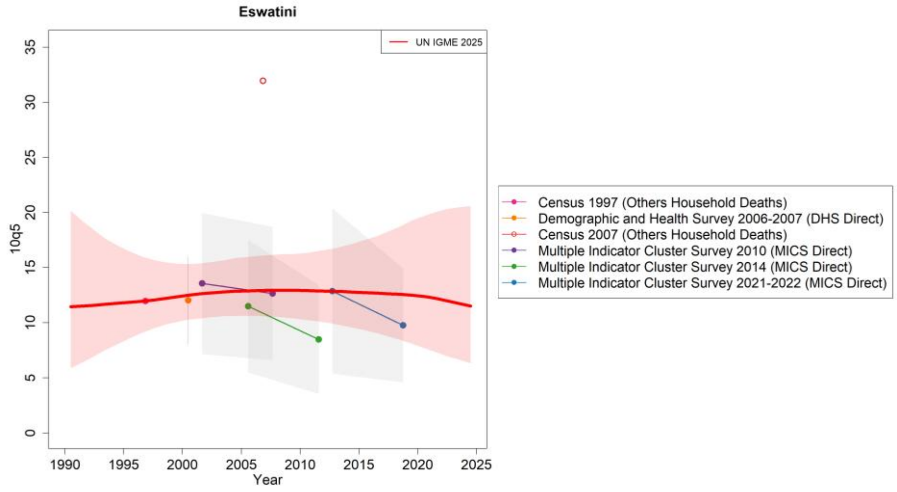

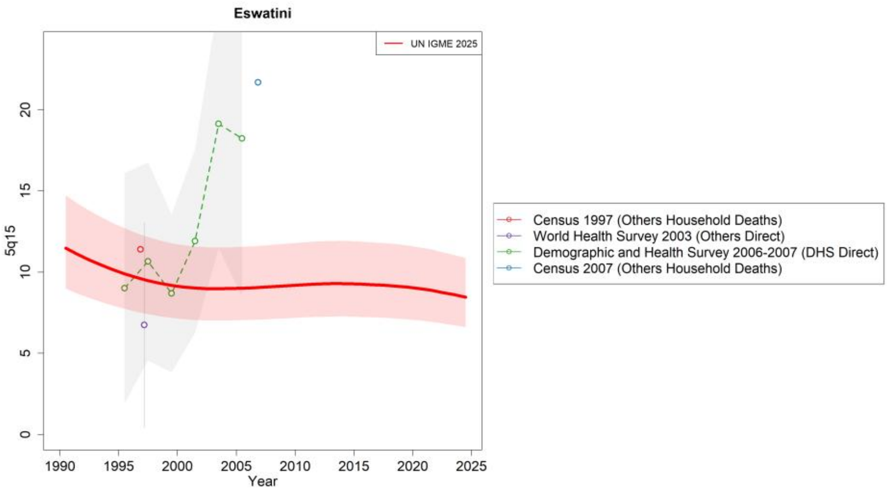
Eswatini
UN IGME 2025
5q15
Year
Census 1997 (Others Household Deaths)
World Health Survey 2003 (Others Direct)
Demographic and Health Survey 2006-2007 (DHS Direct)
Census 2007 (Others Household Deaths)

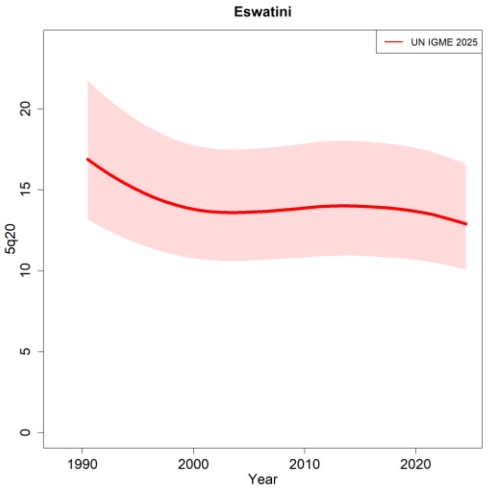
Eswatini
UN IGME 2025
5q20
Year

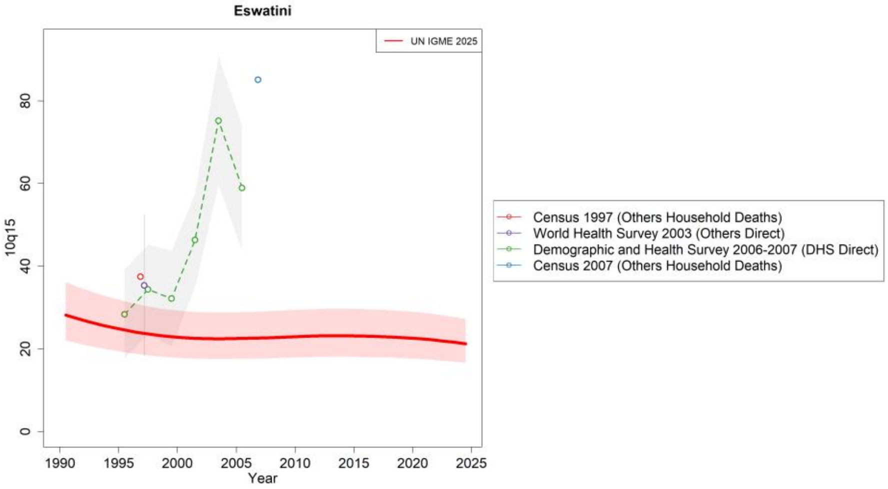
Eswatini
UN IGME 2025
10q15
Year
Census 1997 (Others Household Deaths)
World Health Survey 2003 (Others Direct)
Demographic and Health Survey 2006-2007 (DHS Direct)
Census 2007 (Others Household Deaths)

Ethiopia (ETH)

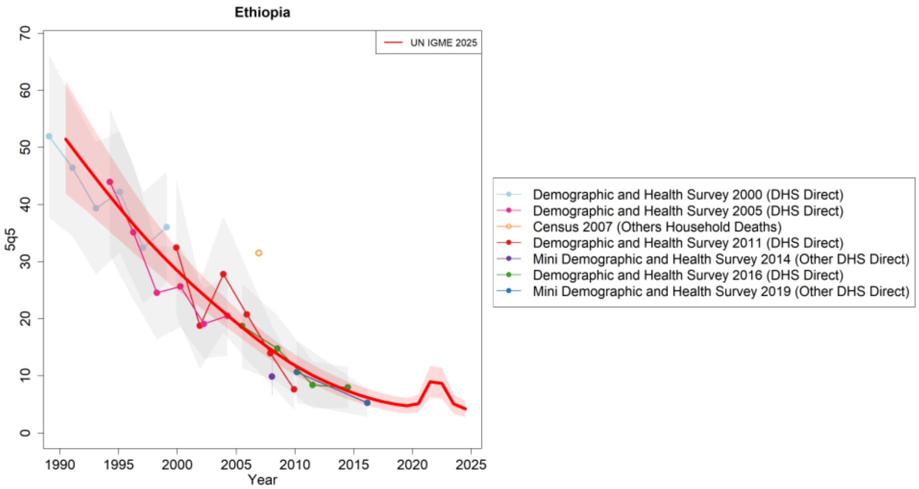

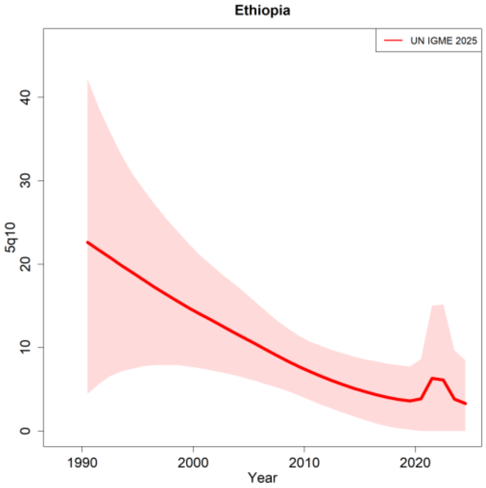

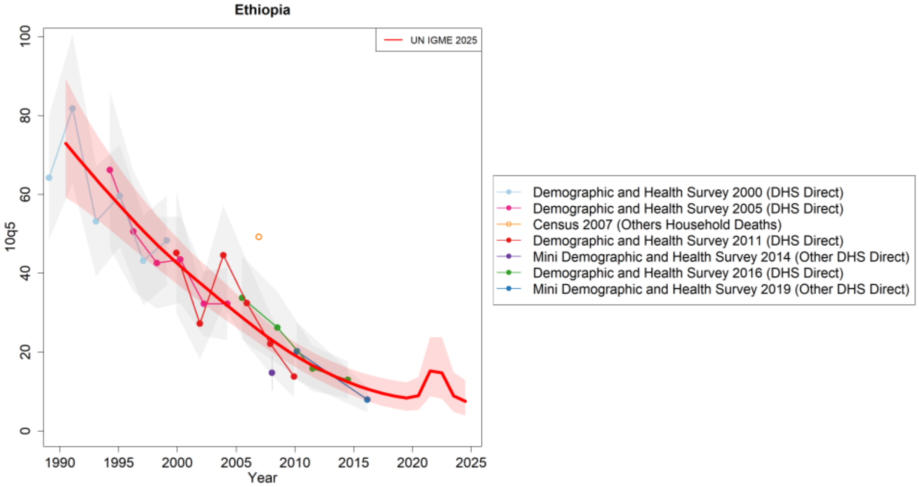

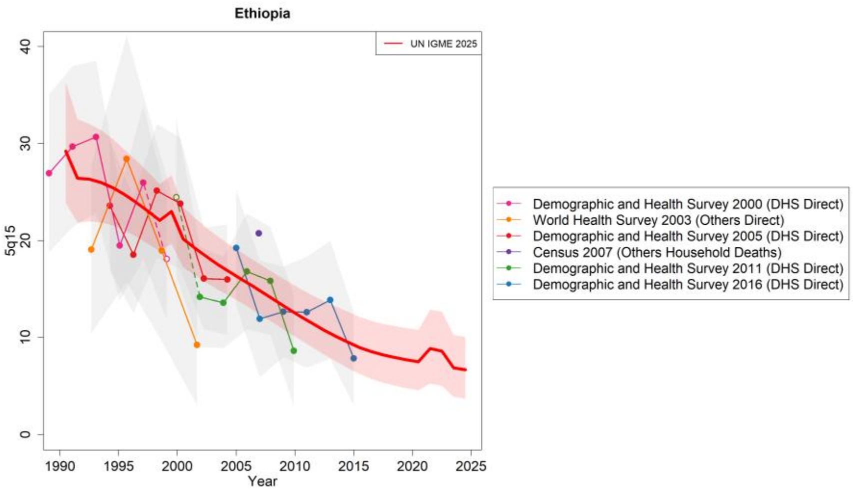
Ethiopia
UN IGME 2025
Demographic and Health Survey 2000 (DHS Direct)
World Health Survey 2003 (Others Direct)
Demographic and Health Survey 2005 (DHS Direct)
Census 2007 (Others Household Deaths)
Demographic and Health Survey 2011 (DHS Direct)
Demographic and Health Survey 2016 (DHS Direct)
5q15
Year

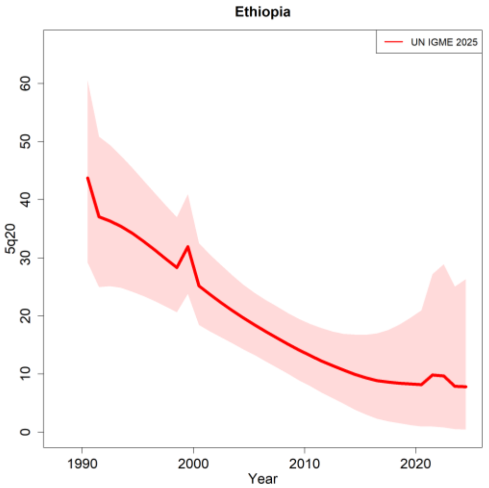
Ethiopia
UN IGME 2025
5q20
Year

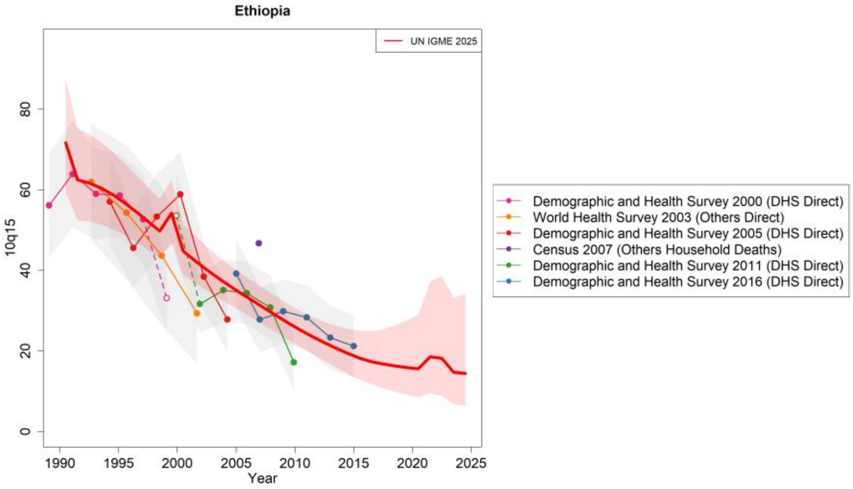
Ethiopia
UN IGME 2025
Demographic and Health Survey 2000 (DHS Direct)
World Health Survey 2003 (Others Direct)
Demographic and Health Survey 2005 (DHS Direct)
Census 2007 (Others Household Deaths)
Demographic and Health Survey 2011 (DHS Direct)
Demographic and Health Survey 2016 (DHS Direct)
10q15
Year

Fiji (FJI)

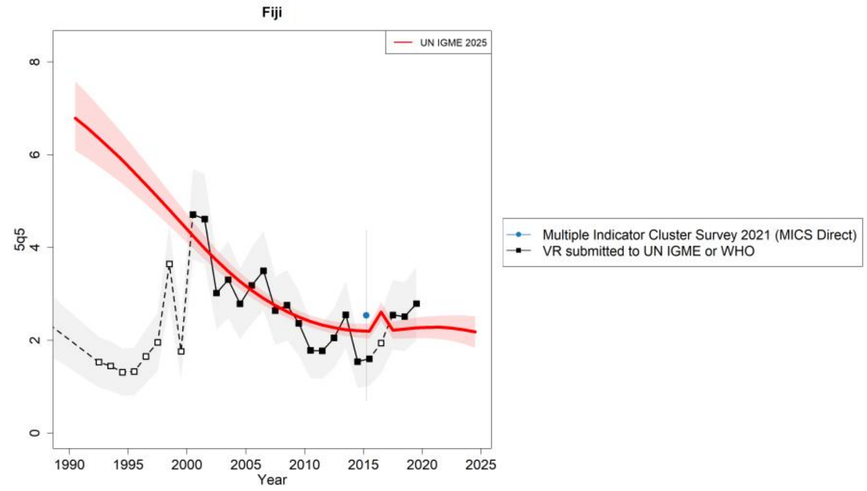
Fiji
UN IGME 2025
Multiple Indicator Cluster Survey 2021 (MICS Direct)
VR submitted to UN IGME or WHO
5q5
Year
0
2
4
6
8
1990
1995
2000
2005
2010
2015
2020
2025

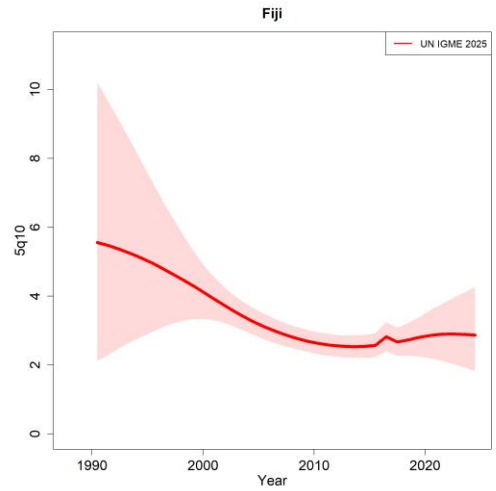
Fiji
UN IGME 2025
5q10
Year
0
2
4
6
8
10
1990
2000
2010
2020

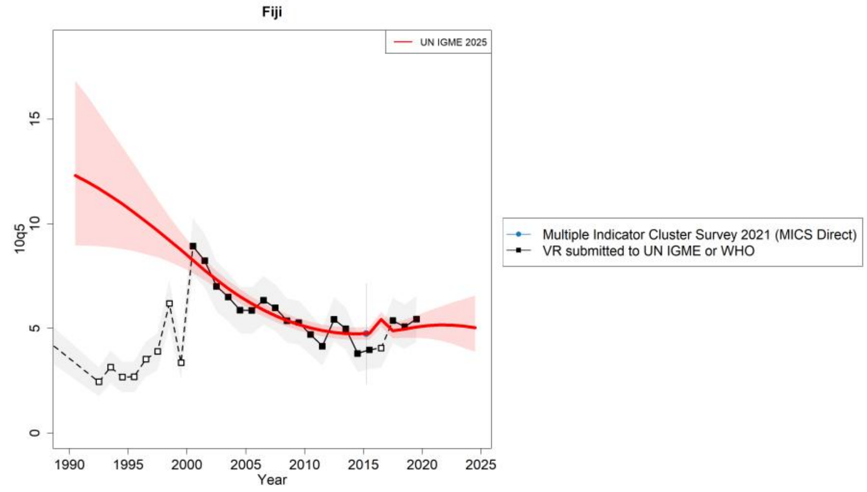
Fiji
UN IGME 2025
Multiple Indicator Cluster Survey 2021 (MICS Direct)
VR submitted to UN IGME or WHO
10q5
Year
0
5
10
15
1990
1995
2000
2005
2010
2015
2020
2025

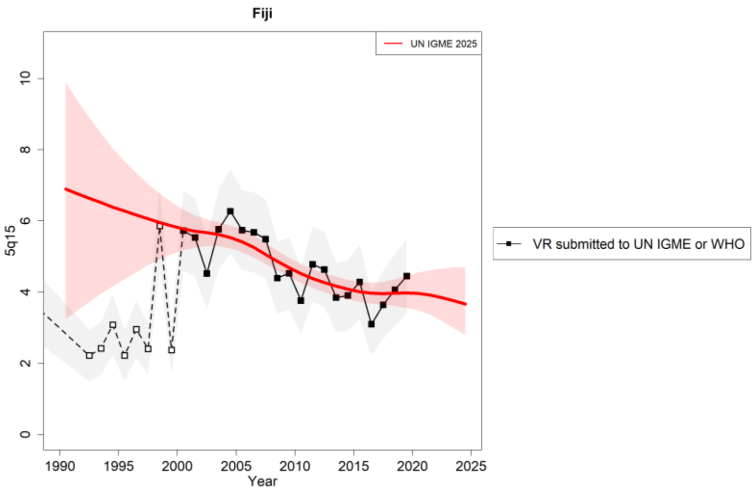
Fiji
UN IGME 2025
VR submitted to UN IGME or WHO
5q15
Year

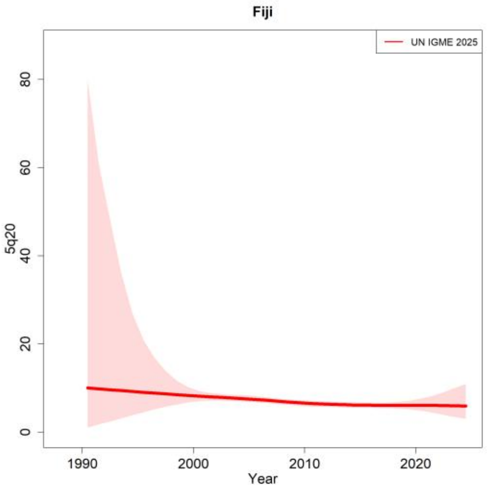
Fiji
UN IGME 2025
5q20
Year

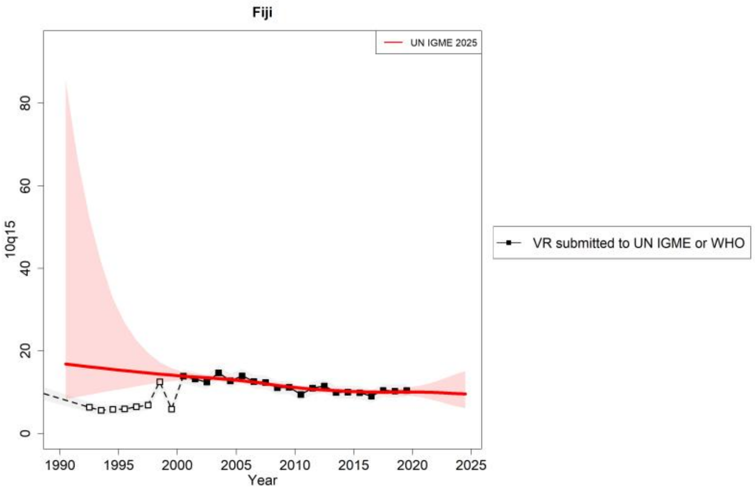
Fiji
UN IGME 2025
VR submitted to UN IGME or WHO
10q15
Year

Finland (FIN)

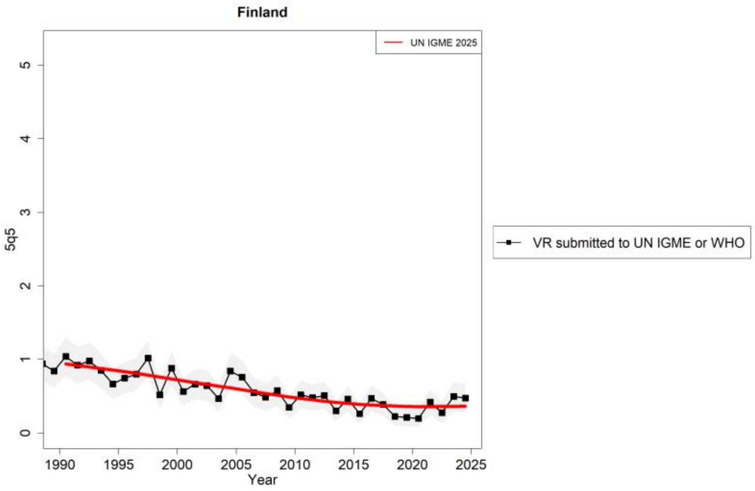
Finland
UN IGME 2025
5q5
Year
VR submitted to UN IGME or WHO

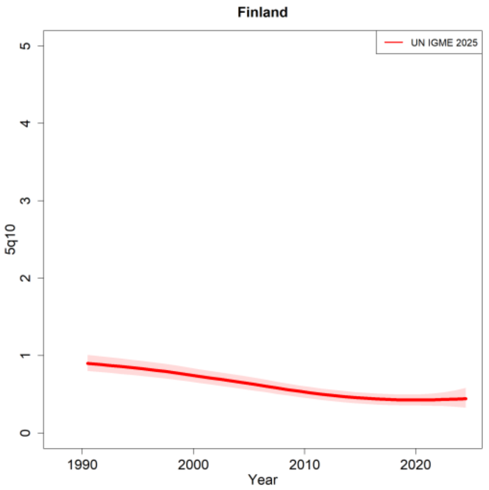
Finland
UN IGME 2025
5q10
Year

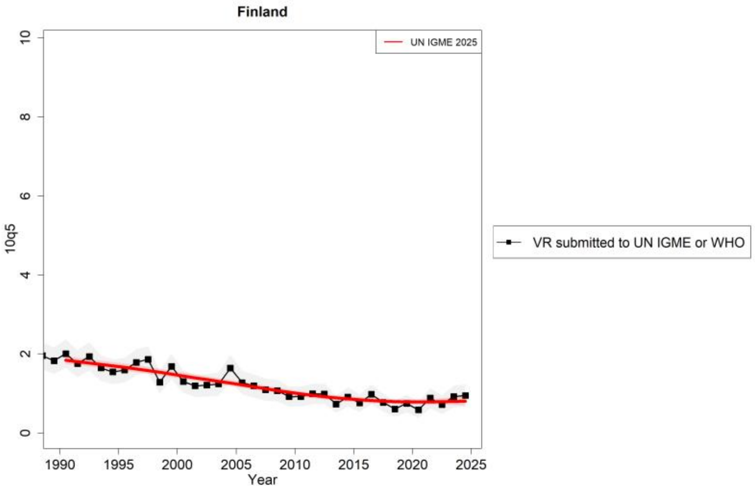
Finland
UN IGME 2025
10q5
Year
VR submitted to UN IGME or WHO

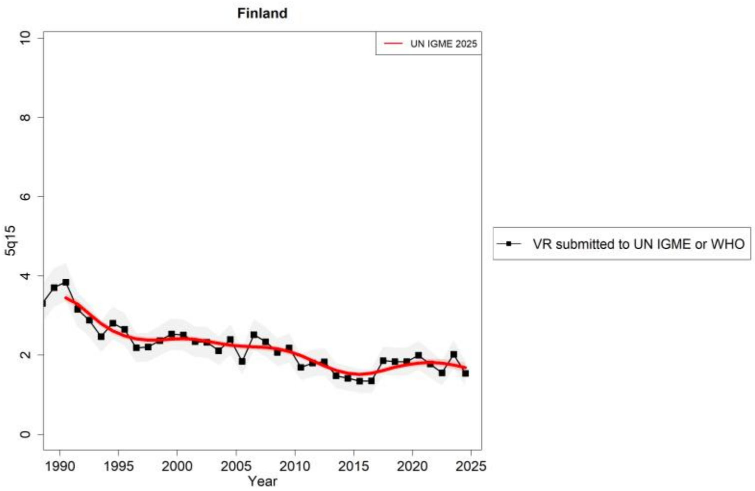
Finland
UN IGME 2025
VR submitted to UN IGME or WHO
5q15
0
2
4
6
8
10
1990
1995
2000
2005
2010
2015
2020
2025
Year

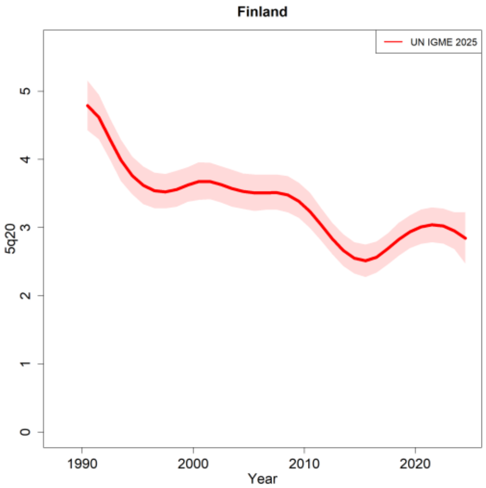
Finland
UN IGME 2025
5q20
0
1
2
3
4
5
1990
2000
2010
2020
Year

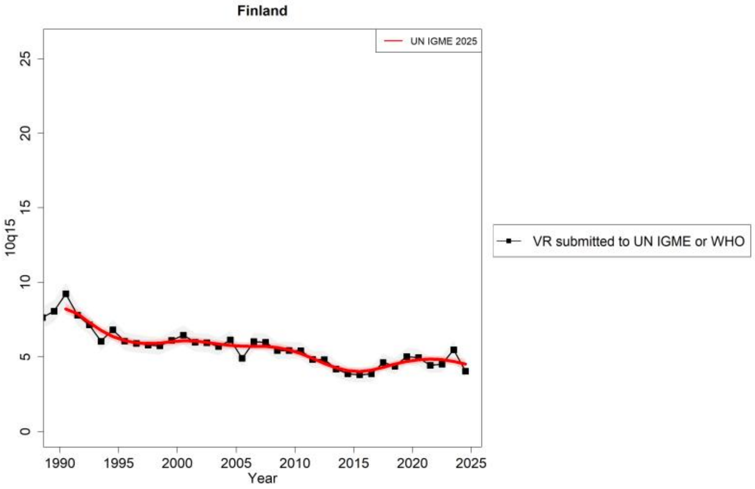
Finland
UN IGME 2025
VR submitted to UN IGME or WHO
10q15
0
5
10
15
20
25
1990
1995
2000
2005
2010
2015
2020
2025
Year

France (FRA)

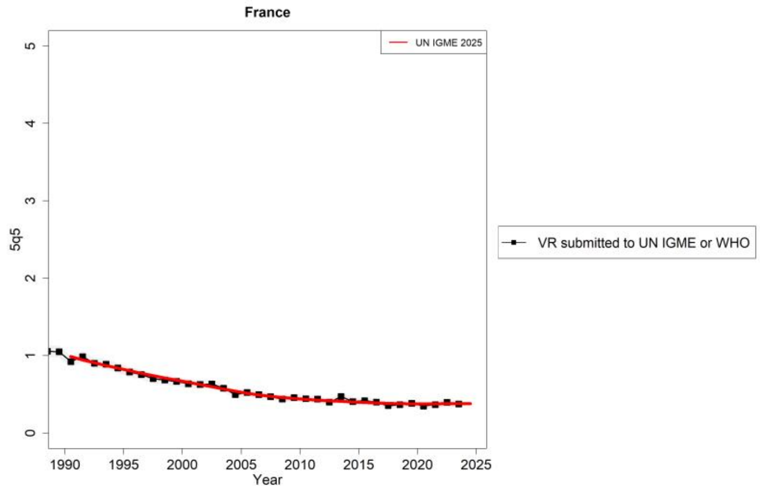
France
UN IGME 2025
VR submitted to UN IGME or WHO
5q5
0
1
2
3
4
5
1990
1995
2000
2005
2010
2015
2020
2025
Year

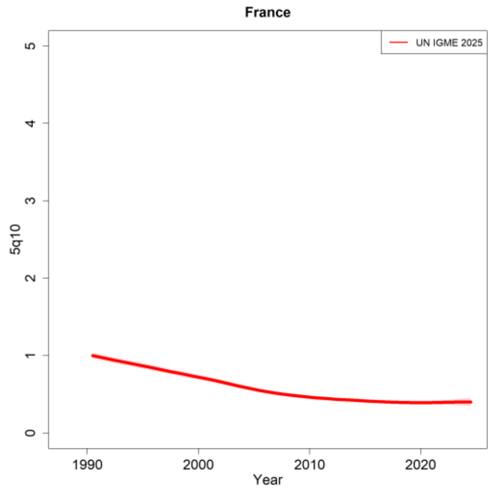
France
UN IGME 2025
5q10
0
1
2
3
4
5
1990
2000
2010
2020
Year

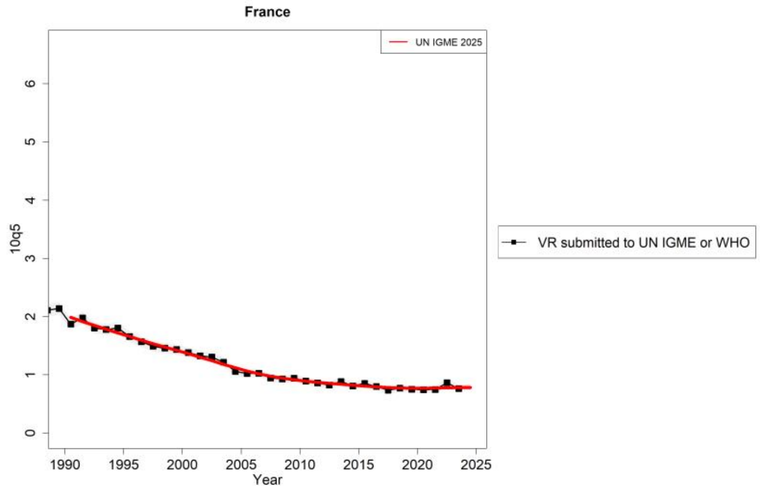
France
UN IGME 2025
VR submitted to UN IGME or WHO
10q5
0
1
2
3
4
5
6
1990
1995
2000
2005
2010
2015
2020
2025
Year

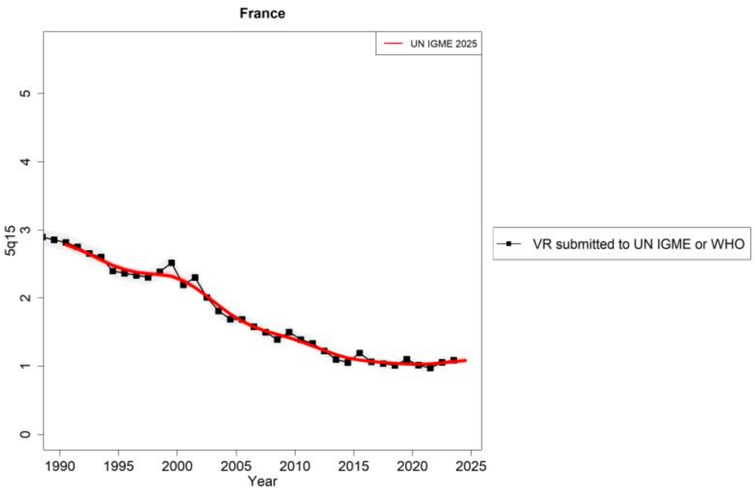
France
UN IGME 2025
VR submitted to UN IGME or WHO
5q15
0
1
2
3
4
5
1990
1995
2000
2005
2010
2015
2020
2025
Year

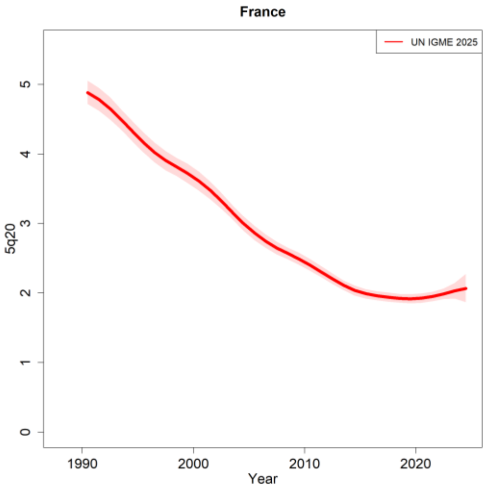
France
UN IGME 2025
5q20
0
1
2
3
4
5
1990
2000
2010
2020
Year

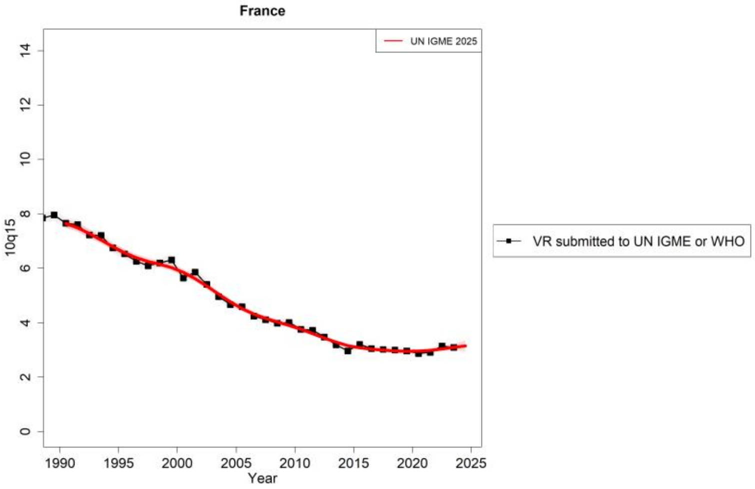
France
UN IGME 2025
VR submitted to UN IGME or WHO
10q15
0
2
4
6
8
10
12
14
1990
1995
2000
2005
2010
2015
2020
2025
Year

Gabon (GAB)

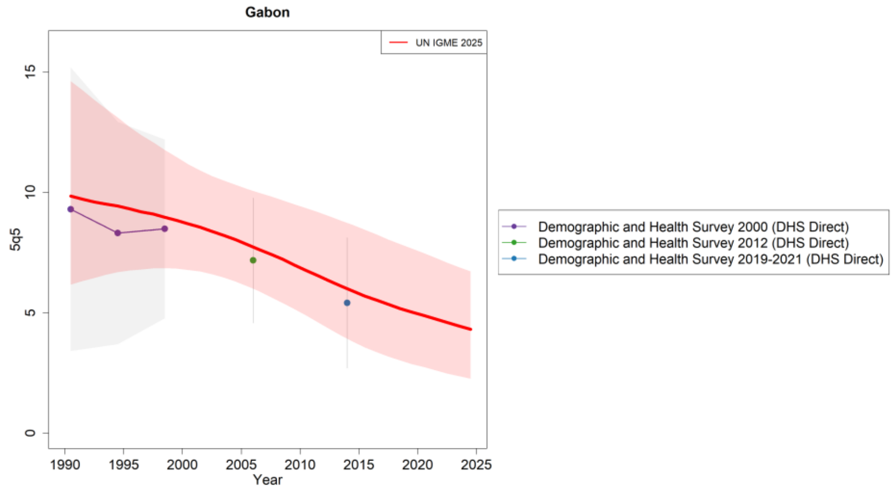
Gabon
UN IGME 2025
5q5
Year
Demographic and Health Survey 2000 (DHS Direct)
Demographic and Health Survey 2012 (DHS Direct)
Demographic and Health Survey 2019-2021 (DHS Direct)

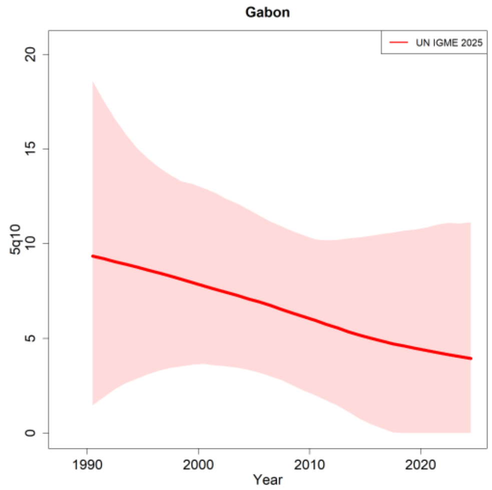
Gabon
UN IGME 2025
5q10
Year

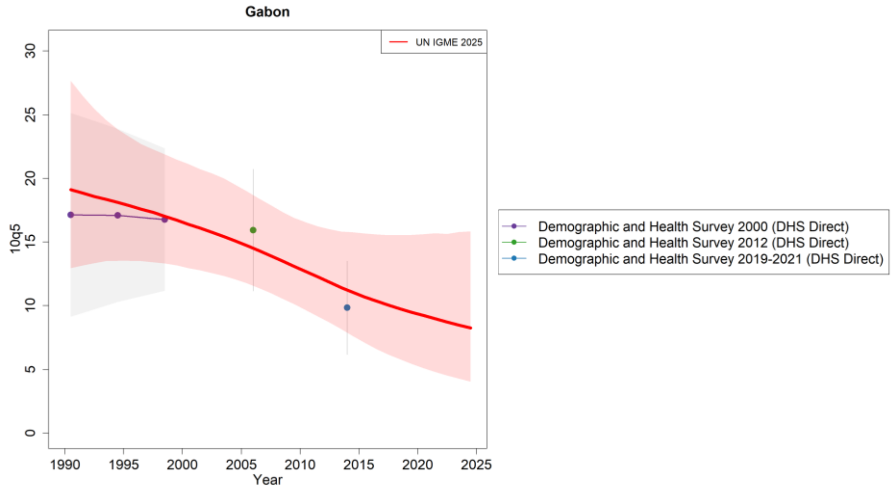
Gabon
UN IGME 2025
10q5
Year
Demographic and Health Survey 2000 (DHS Direct)
Demographic and Health Survey 2012 (DHS Direct)
Demographic and Health Survey 2019-2021 (DHS Direct)

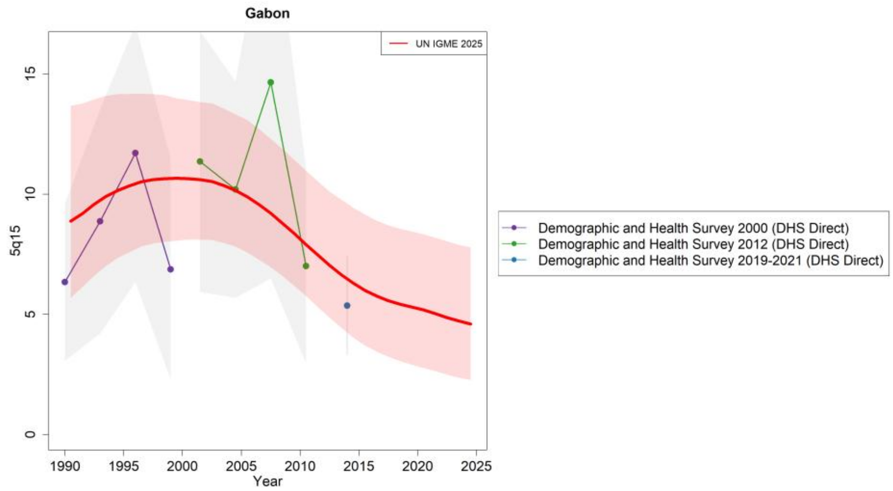
Gabon
UN IGME 2025
5q15
Year
Demographic and Health Survey 2000 (DHS Direct)
Demographic and Health Survey 2012 (DHS Direct)
Demographic and Health Survey 2019-2021 (DHS Direct)

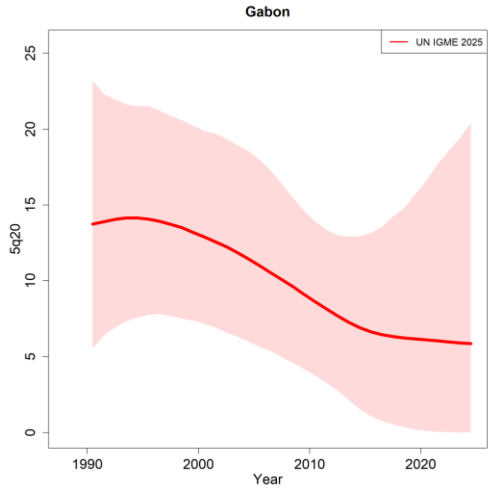
Gabon
UN IGME 2025
5q20
Year

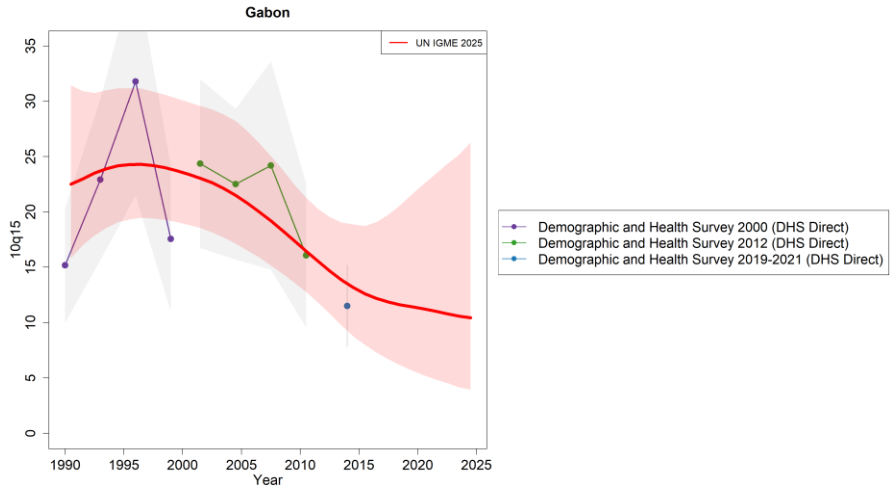
Gabon
UN IGME 2025
10q15
Year
Demographic and Health Survey 2000 (DHS Direct)
Demographic and Health Survey 2012 (DHS Direct)
Demographic and Health Survey 2019-2021 (DHS Direct)

Gambia (GMB)

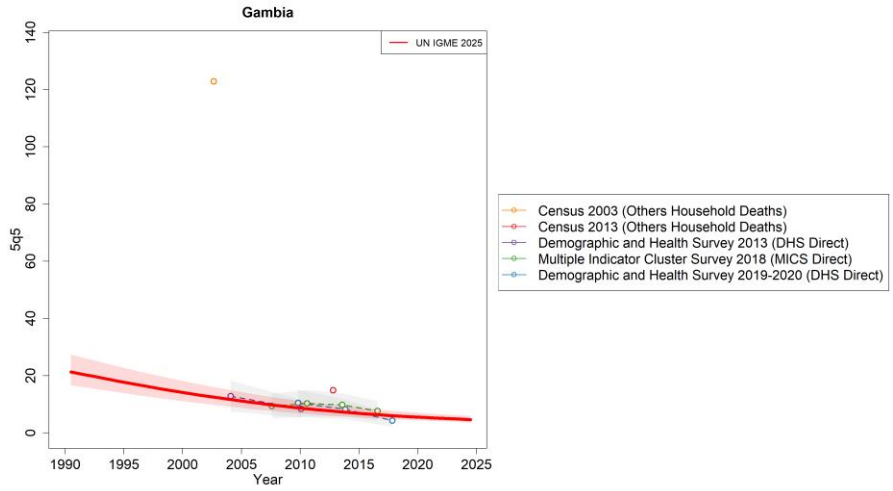
Gambia
UN IGME 2025
5q5
Year
Census 2003 (Others Household Deaths)
Census 2013 (Others Household Deaths)
Demographic and Health Survey 2013 (DHS Direct)
Multiple Indicator Cluster Survey 2018 (MICS Direct)
Demographic and Health Survey 2019-2020 (DHS Direct)

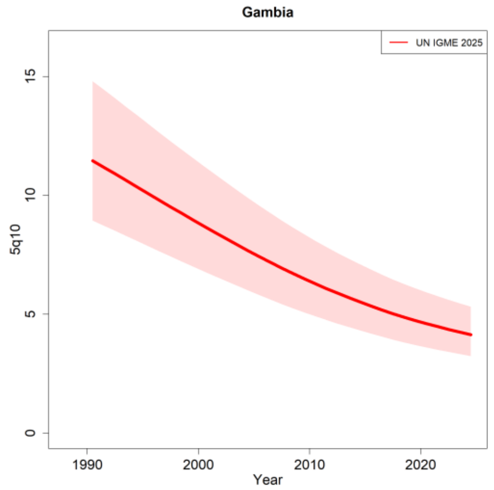
Gambia
UN IGME 2025
5q10
Year

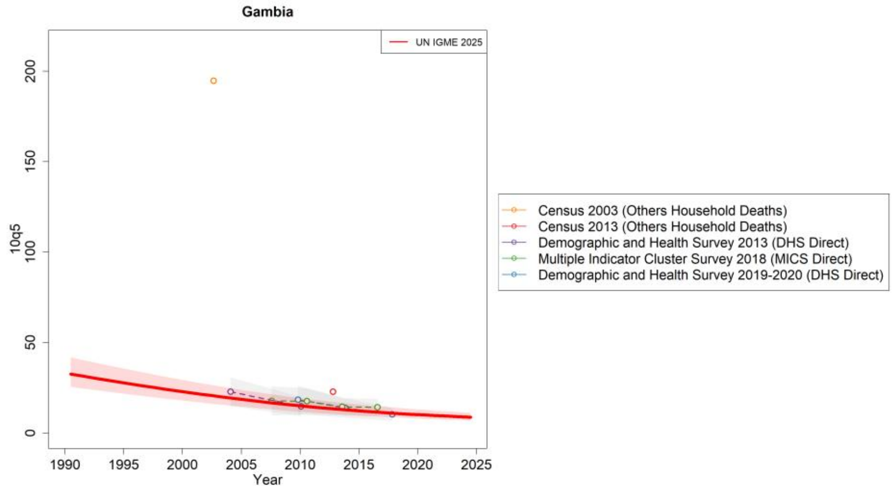
Gambia
UN IGME 2025
10q5
Year
Census 2003 (Others Household Deaths)
Census 2013 (Others Household Deaths)
Demographic and Health Survey 2013 (DHS Direct)
Multiple Indicator Cluster Survey 2018 (MICS Direct)
Demographic and Health Survey 2019-2020 (DHS Direct)

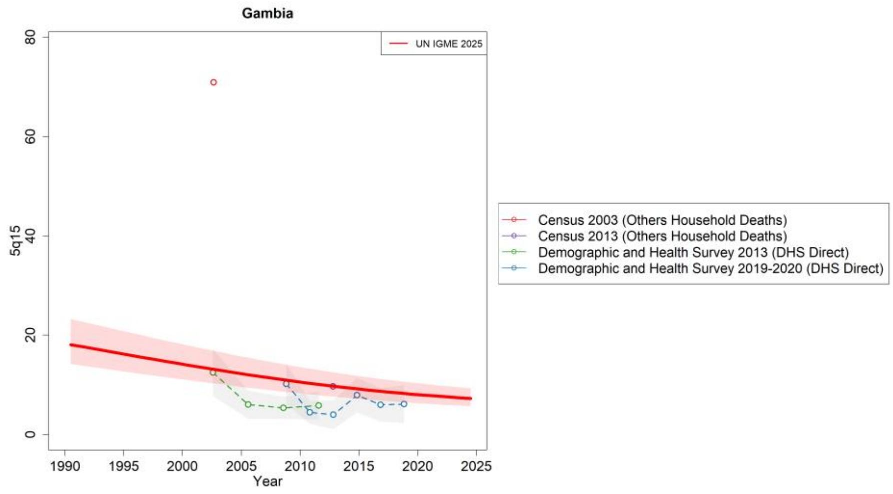
Gambia
UN IGME 2025
Census 2003 (Others Household Deaths)
Census 2013 (Others Household Deaths)
Demographic and Health Survey 2013 (DHS Direct)
Demographic and Health Survey 2019-2020 (DHS Direct)
5q15
Year
0
20
40
60
80
1990
1995
2000
2005
2010
2015
2020
2025

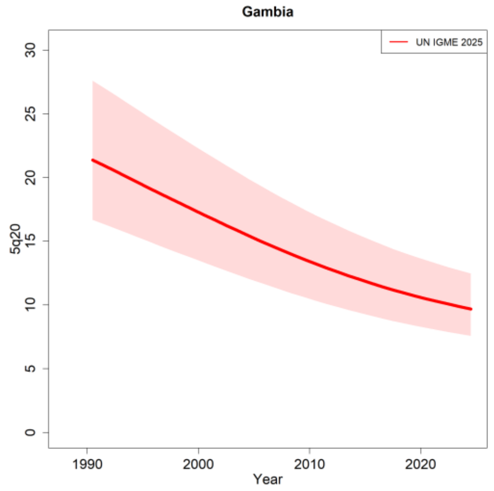
Gambia
UN IGME 2025
5q20
Year
0
5
10
15
20
25
30
1990
2000
2010
2020

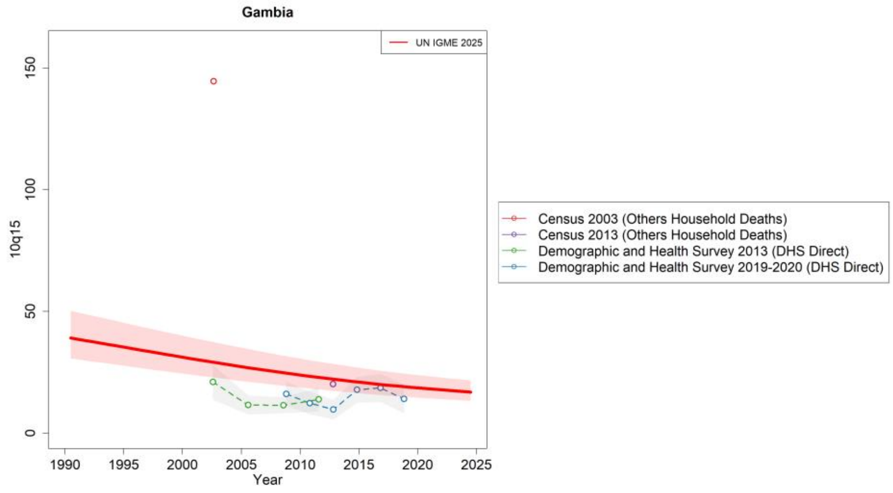
Gambia
UN IGME 2025
Census 2003 (Others Household Deaths)
Census 2013 (Others Household Deaths)
Demographic and Health Survey 2013 (DHS Direct)
Demographic and Health Survey 2019-2020 (DHS Direct)
10q15
Year
0
50
100
150
1990
1995
2000
2005
2010
2015
2020
2025

Georgia (GEO)

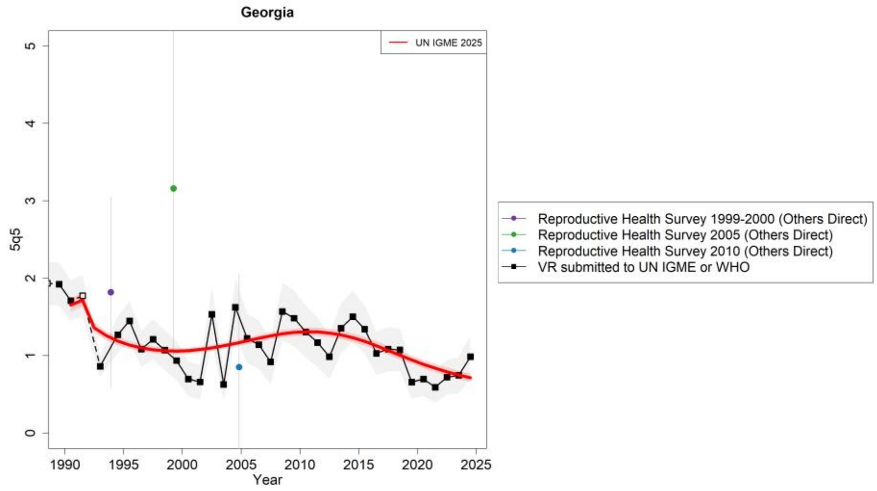

Georgia
UN IGME 2025
Reproductive Health Survey 1999-2000 (Others Direct)
Reproductive Health Survey 2005 (Others Direct)
Reproductive Health Survey 2010 (Others Direct)
VR submitted to UN IGME or WHO
5q5
Year

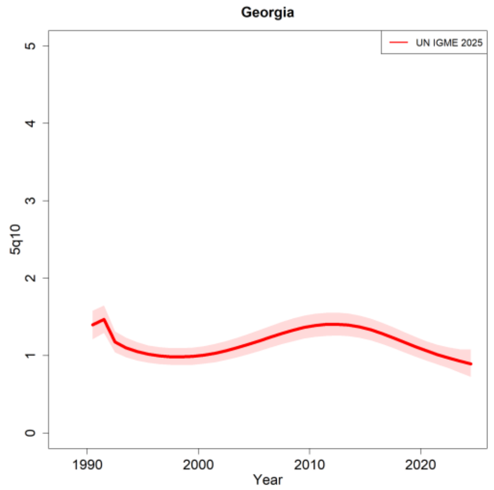

Georgia
UN IGME 2025
5q10
Year

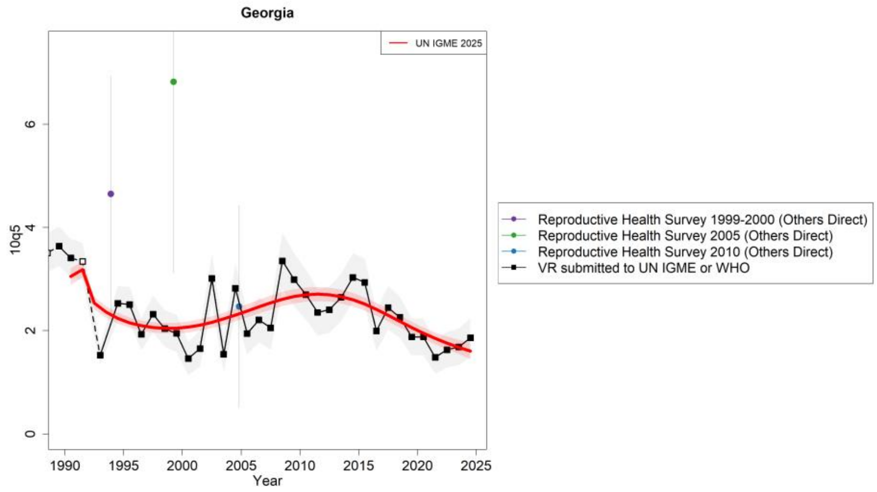

Georgia
UN IGME 2025
Reproductive Health Survey 1999-2000 (Others Direct)
Reproductive Health Survey 2005 (Others Direct)
Reproductive Health Survey 2010 (Others Direct)
VR submitted to UN IGME or WHO
10q5
Year

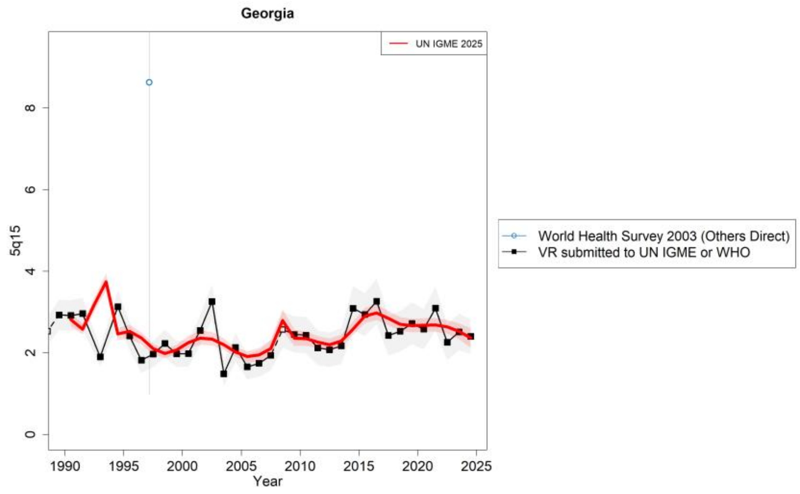
Georgia
UN IGME 2025
World Health Survey 2003 (Others Direct)
VR submitted to UN IGME or WHO
5q15
Year
0
2
4
6
8
1990
1995
2000
2005
2010
2015
2020
2025

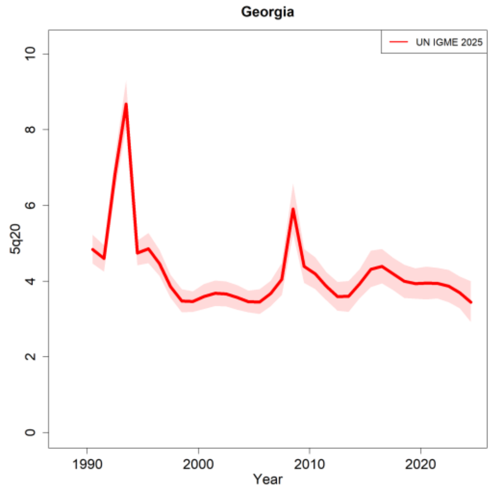
Georgia
UN IGME 2025
5q20
Year
0
2
4
6
8
10
1990
2000
2010
2020

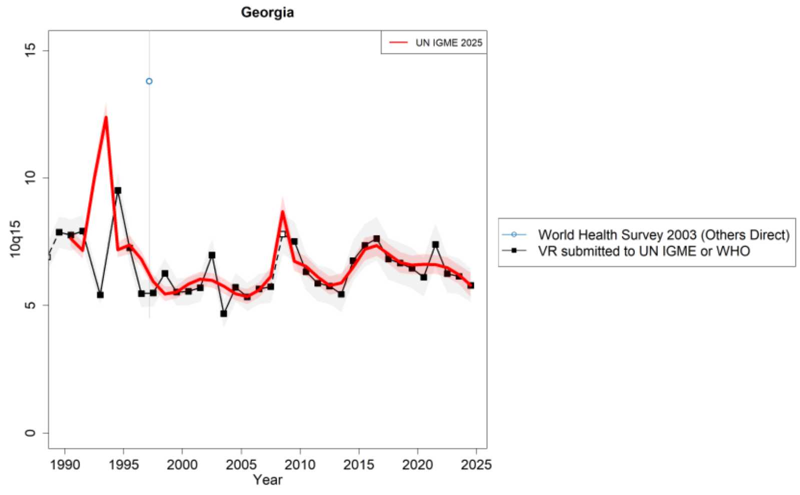
Georgia
UN IGME 2025
World Health Survey 2003 (Others Direct)
VR submitted to UN IGME or WHO
10q15
Year
0
5
10
15
1990
1995
2000
2005
2010
2015
2020
2025

Germany (DEU)

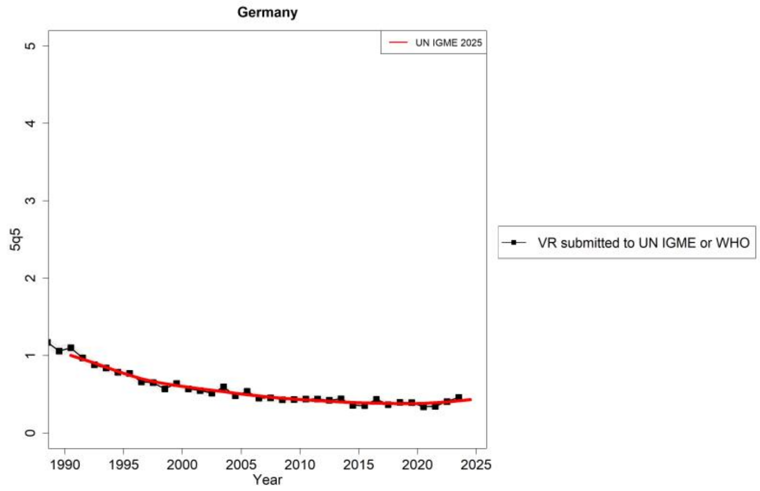
Germany
UN IGME 2025
VR submitted to UN IGME or WHO
5q5
0
1
2
3
4
5
1990
1995
2000
2005
2010
2015
2020
2025
Year

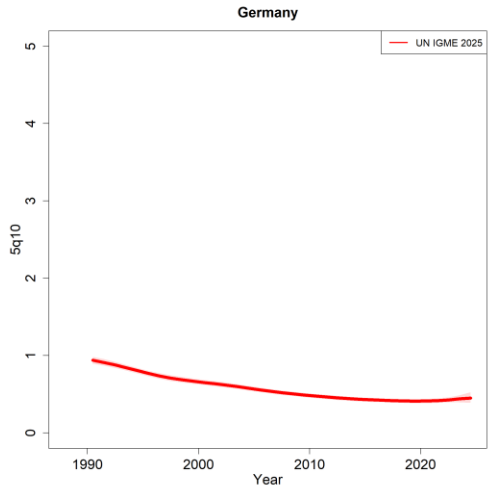
Germany
UN IGME 2025
5q10
0
1
2
3
4
5
1990
2000
2010
2020
Year

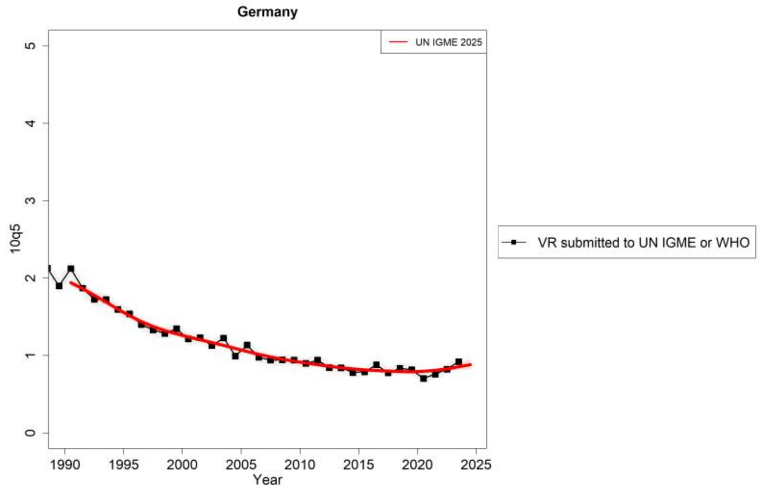
Germany
UN IGME 2025
VR submitted to UN IGME or WHO
10q5
0
1
2
3
4
5
1990
1995
2000
2005
2010
2015
2020
2025
Year

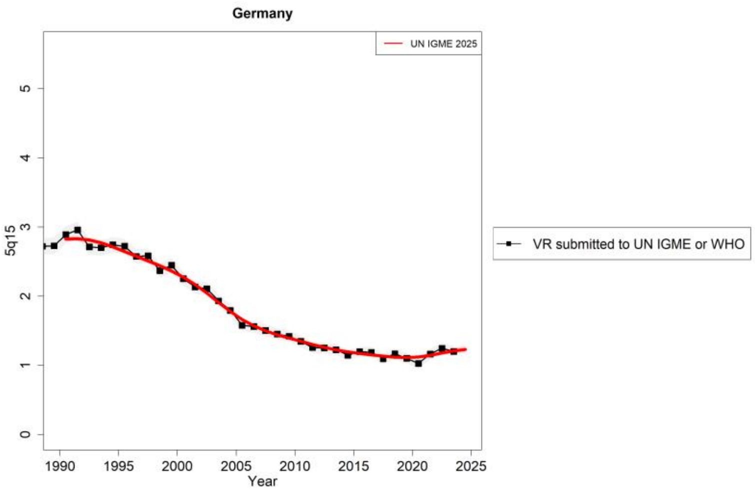
Germany
UN IGME 2025
VR submitted to UN IGME or WHO
5q15
Year

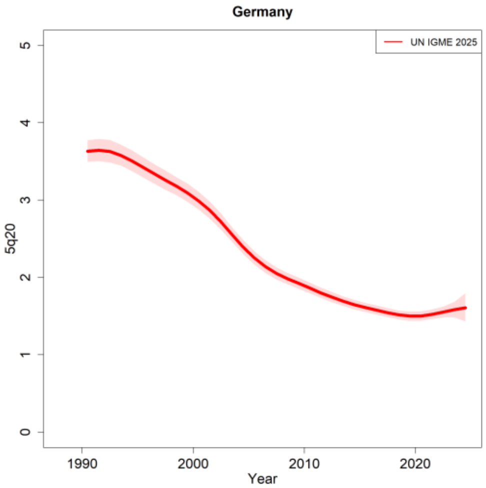
Germany
UN IGME 2025
5q20
Year

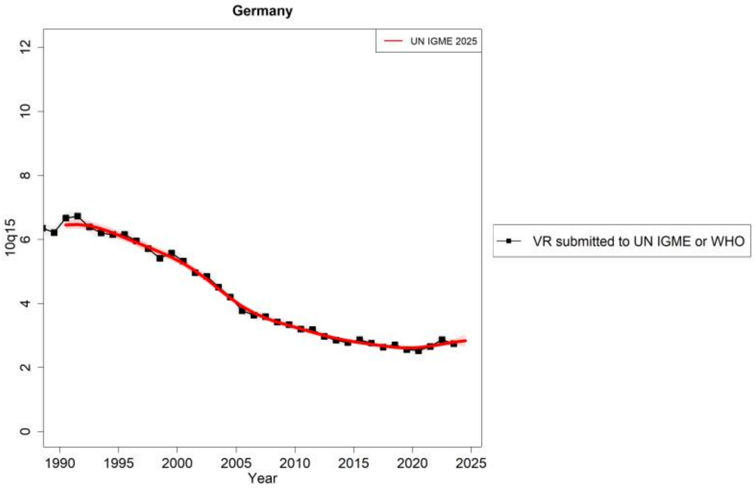
Germany
UN IGME 2025
VR submitted to UN IGME or WHO
10q15
Year

Ghana (GHA)

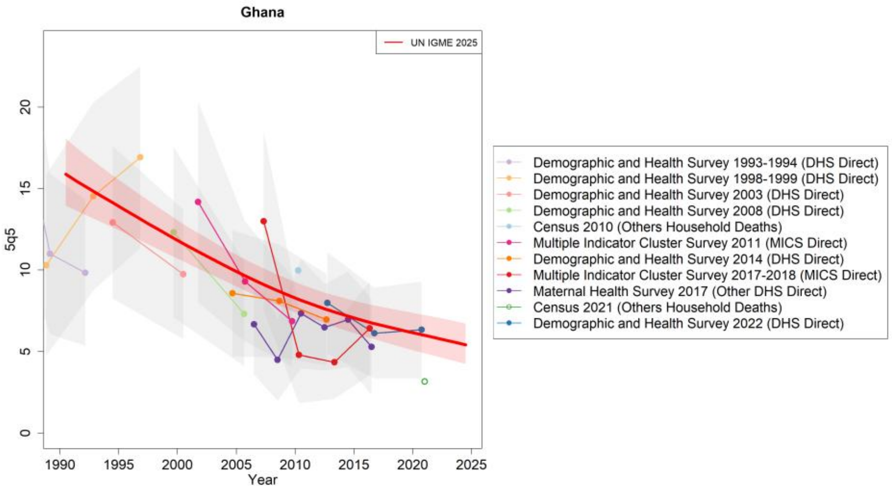
Ghana
UN IGME 2025
5q5
Year
Demographic and Health Survey 1993-1994 (DHS Direct)
Demographic and Health Survey 1998-1999 (DHS Direct)
Demographic and Health Survey 2003 (DHS Direct)
Demographic and Health Survey 2008 (DHS Direct)
Census 2010 (Others Household Deaths)
Multiple Indicator Cluster Survey 2011 (MICS Direct)
Demographic and Health Survey 2014 (DHS Direct)
Multiple Indicator Cluster Survey 2017-2018 (MICS Direct)
Maternal Health Survey 2017 (Other DHS Direct)
Census 2021 (Others Household Deaths)
Demographic and Health Survey 2022 (DHS Direct)

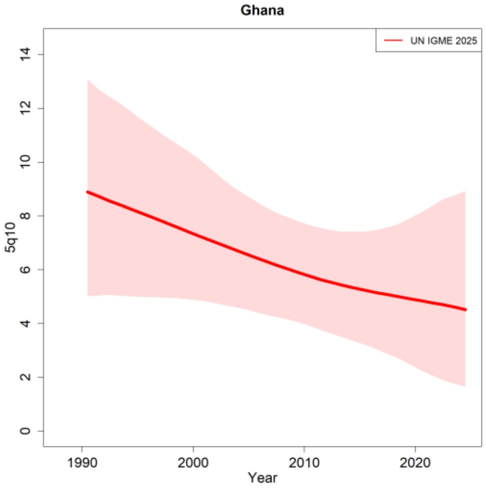
Ghana
UN IGME 2025
5q10
Year

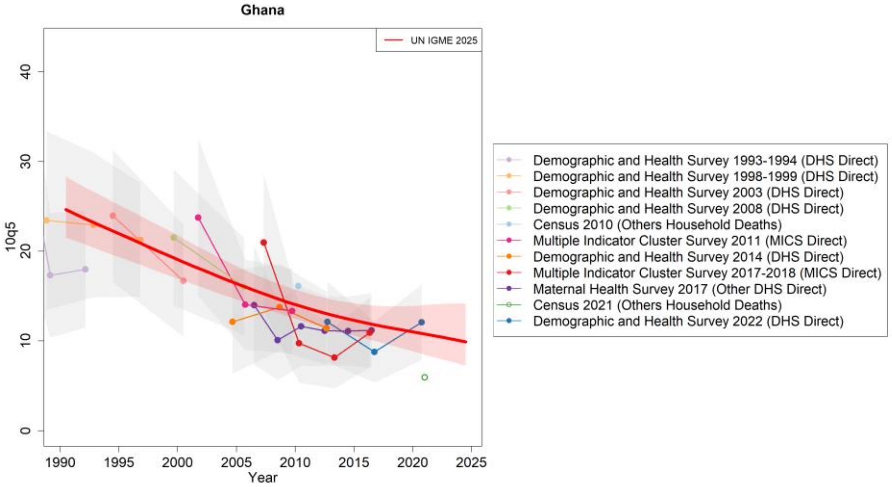
Ghana
UN IGME 2025
10q5
Year
Demographic and Health Survey 1993-1994 (DHS Direct)
Demographic and Health Survey 1998-1999 (DHS Direct)
Demographic and Health Survey 2003 (DHS Direct)
Demographic and Health Survey 2008 (DHS Direct)
Census 2010 (Others Household Deaths)
Multiple Indicator Cluster Survey 2011 (MICS Direct)
Demographic and Health Survey 2014 (DHS Direct)
Multiple Indicator Cluster Survey 2017-2018 (MICS Direct)
Maternal Health Survey 2017 (Other DHS Direct)
Census 2021 (Others Household Deaths)
Demographic and Health Survey 2022 (DHS Direct)

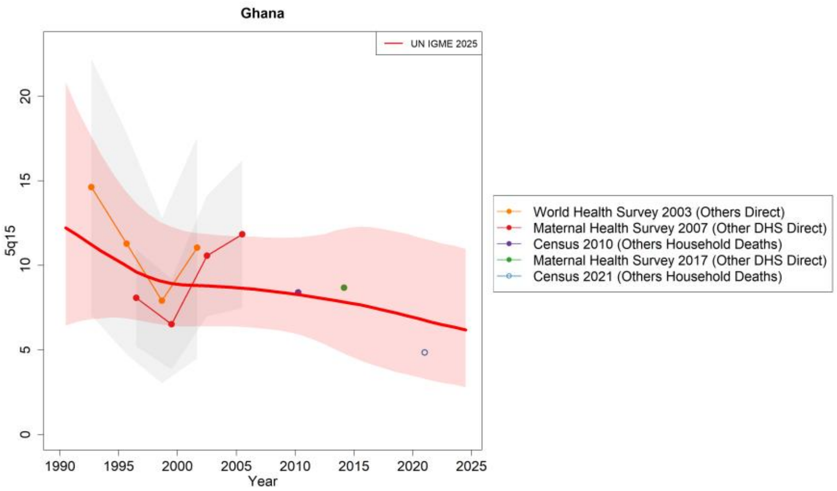

Ghana
UN IGME 2025
5q15
Year
World Health Survey 2003 (Others Direct)
Maternal Health Survey 2007 (Other DHS Direct)
Census 2010 (Others Household Deaths)
Maternal Health Survey 2017 (Other DHS Direct)
Census 2021 (Others Household Deaths)

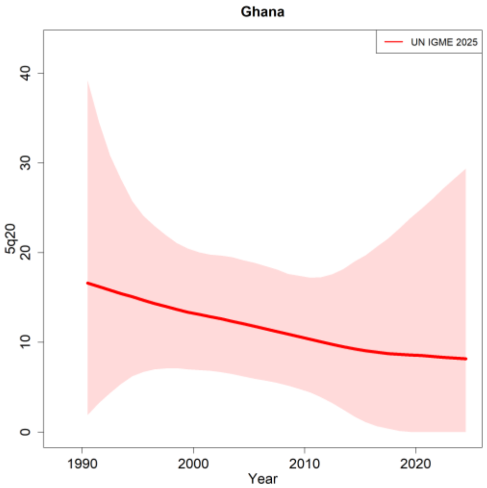

Ghana
UN IGME 2025
5q20
Year

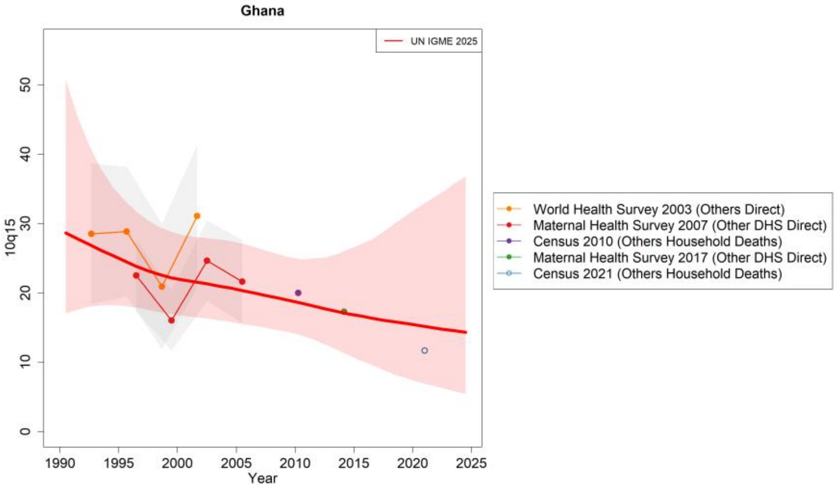

Ghana
UN IGME 2025
10q15
Year
World Health Survey 2003 (Others Direct)
Maternal Health Survey 2007 (Other DHS Direct)
Census 2010 (Others Household Deaths)
Maternal Health Survey 2017 (Other DHS Direct)
Census 2021 (Others Household Deaths)

Greece (GRC)

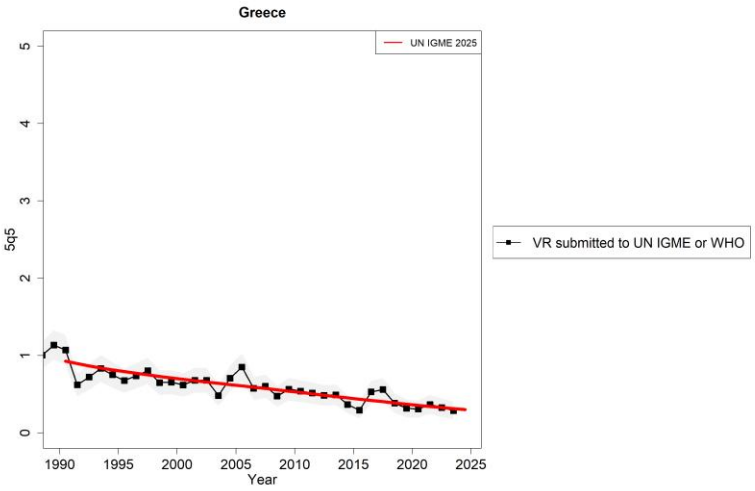
Greece
UN IGME 2025
5q5
Year
VR submitted to UN IGME or WHO

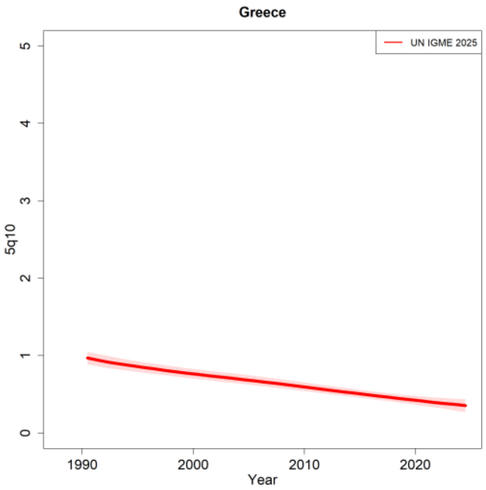
Greece
UN IGME 2025
5q10
Year

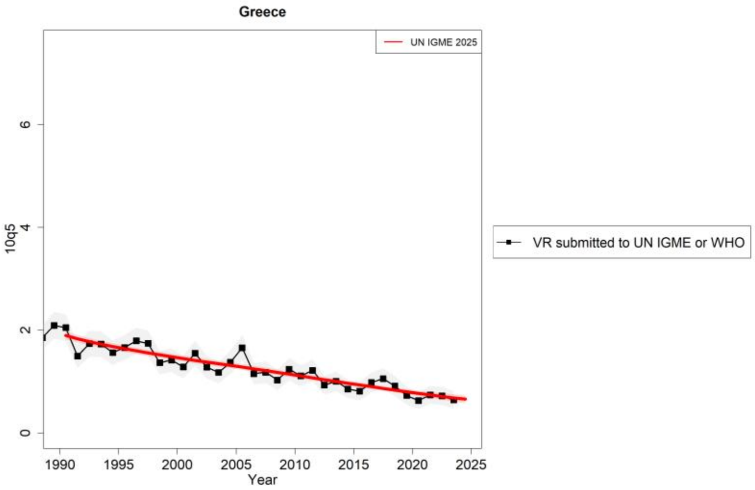
Greece
UN IGME 2025
10q5
Year
VR submitted to UN IGME or WHO

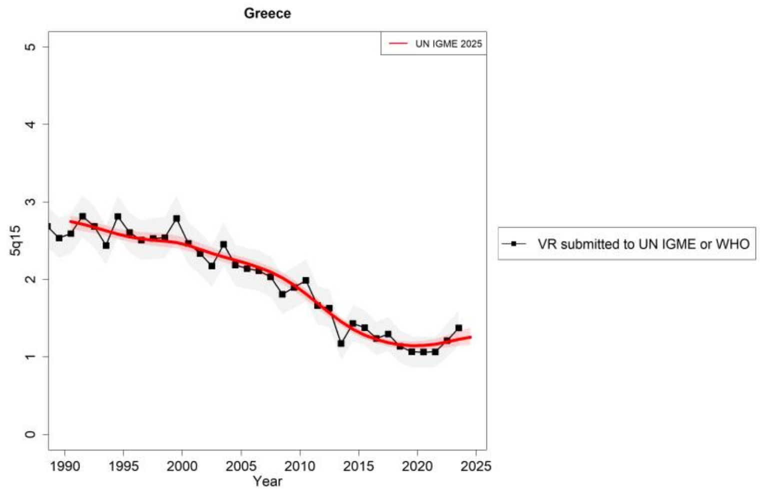
Greece
UN IGME 2025
VR submitted to UN IGME or WHO
5q15
Year
0
1
2
3
4
5
1990
1995
2000
2005
2010
2015
2020
2025

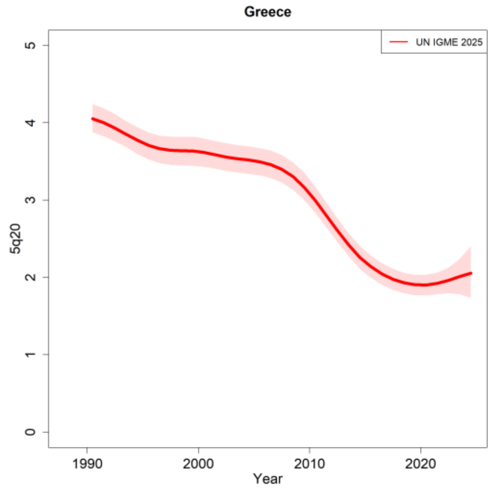
Greece
UN IGME 2025
5q20
Year
0
1
2
3
4
5
1990
2000
2010
2020

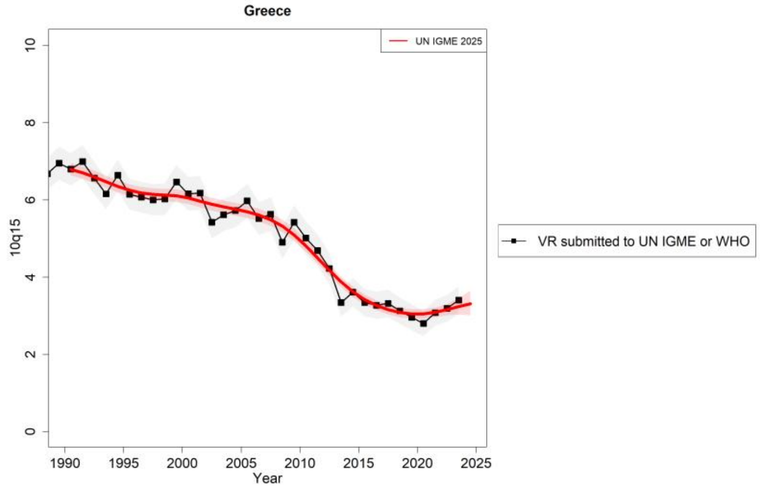
Greece
UN IGME 2025
VR submitted to UN IGME or WHO
10q15
Year
0
2
4
6
8
10
1990
1995
2000
2005
2010
2015
2020
2025

Grenada (GRD)

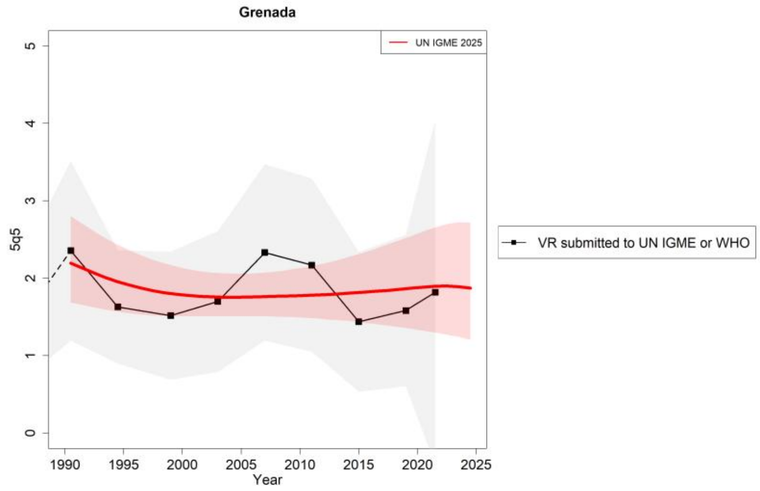
Grenada
UN IGME 2025
VR submitted to UN IGME or WHO
5q5
0
1
2
3
4
5
1990
1995
2000
2005
2010
2015
2020
2025
Year

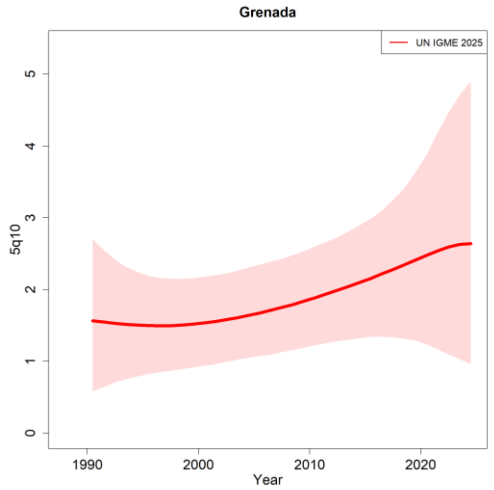
Grenada
UN IGME 2025
5q10
0
1
2
3
4
5
1990
2000
2010
2020
Year

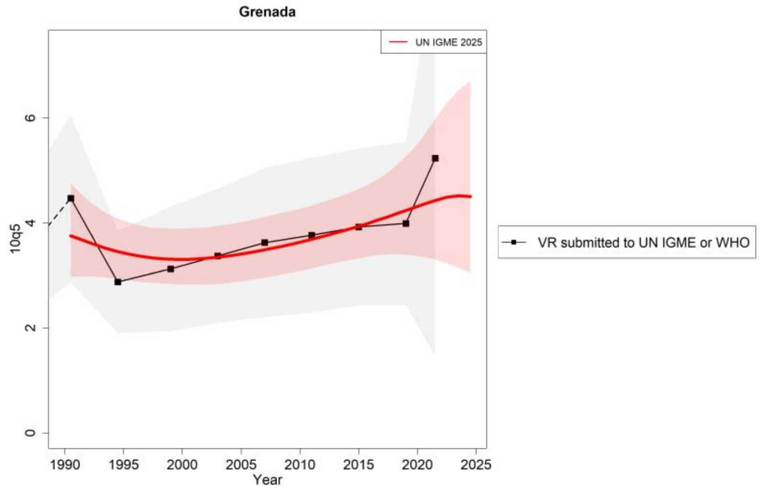
Grenada
UN IGME 2025
VR submitted to UN IGME or WHO
10q5
0
2
4
6
1990
1995
2000
2005
2010
2015
2020
2025
Year

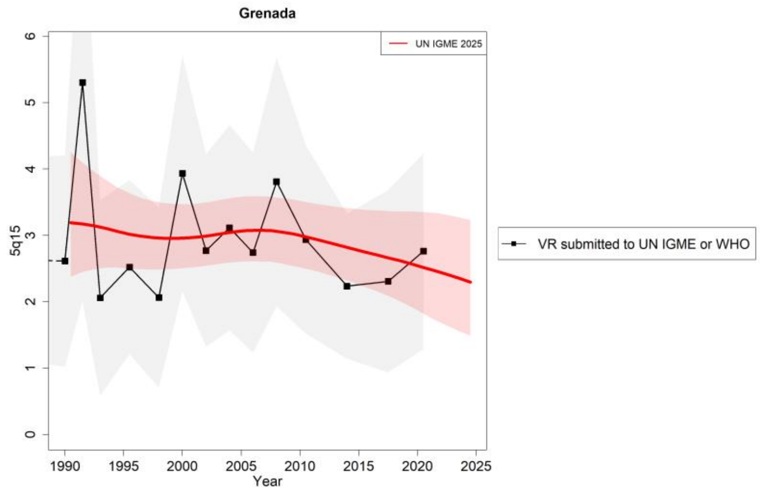
Grenada
UN IGME 2025
VR submitted to UN IGME or WHO
5q15
Year

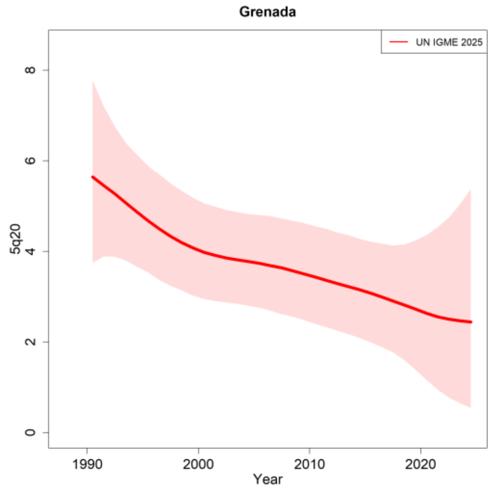
Grenada
UN IGME 2025
5q20
Year

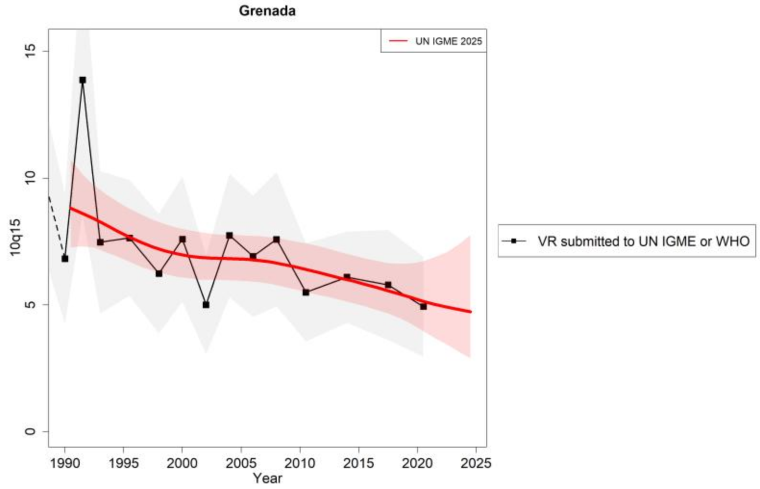
Grenada
UN IGME 2025
VR submitted to UN IGME or WHO
10q15
Year

Guatemala (GTM)

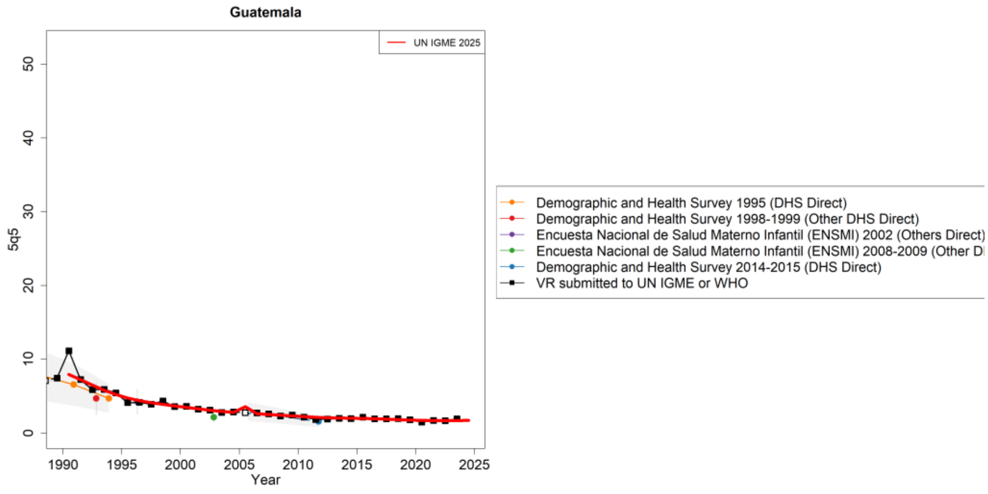

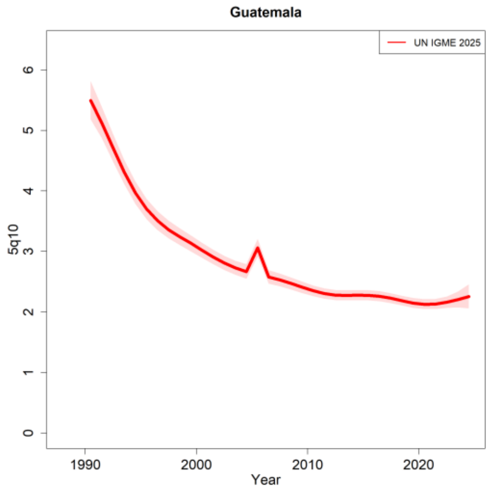

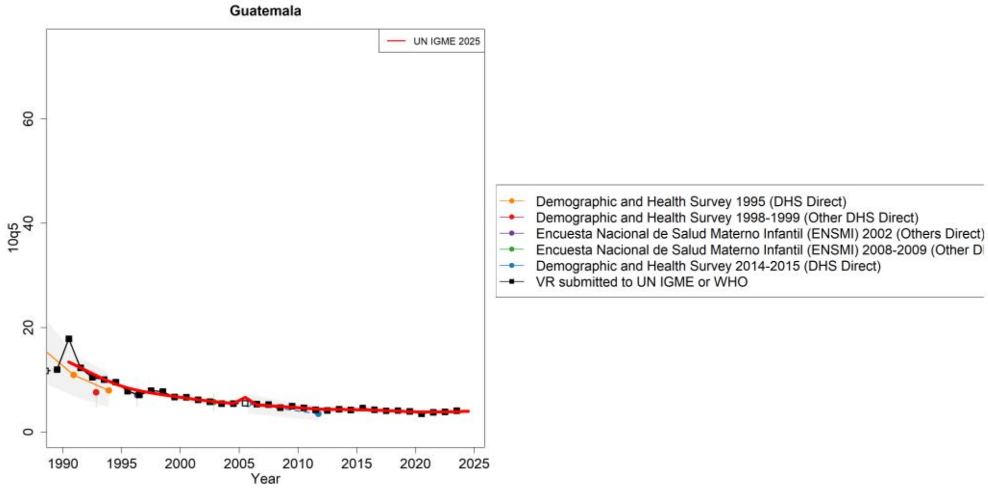

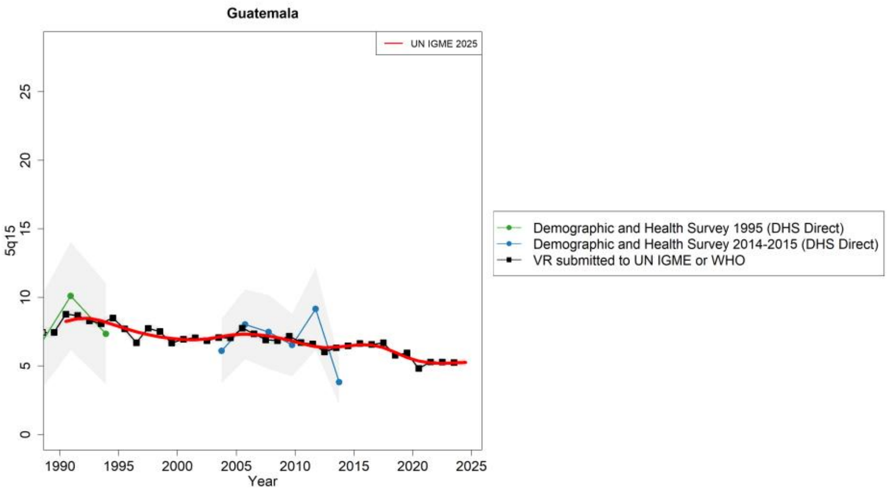
Guatemala
UN IGME 2025
Demographic and Health Survey 1995 (DHS Direct)
Demographic and Health Survey 2014-2015 (DHS Direct)
VR submitted to UN IGME or WHO
5q15
Year
0
5
10
15
20
25
1990
1995
2000
2005
2010
2015
2020
2025

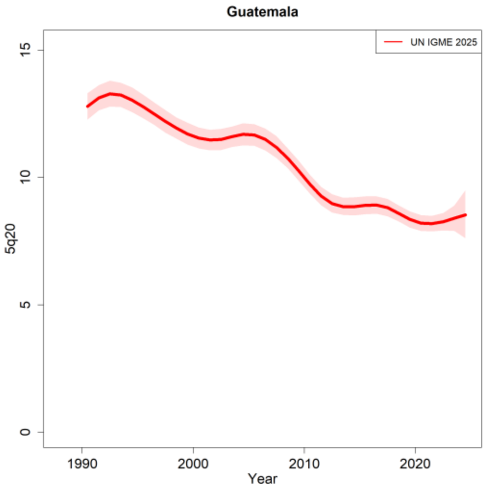
Guatemala
UN IGME 2025
5q20
Year
0
5
10
15
1990
2000
2010
2020

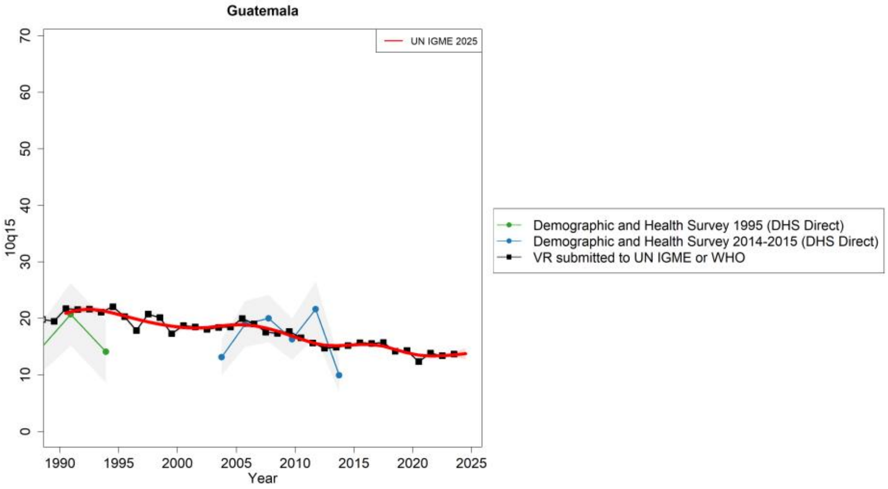
Guatemala
UN IGME 2025
Demographic and Health Survey 1995 (DHS Direct)
Demographic and Health Survey 2014-2015 (DHS Direct)
VR submitted to UN IGME or WHO
10q15
Year
0
10
20
30
40
50
60
70
1990
1995
2000
2005
2010
2015
2020
2025

Guinea (GIN)

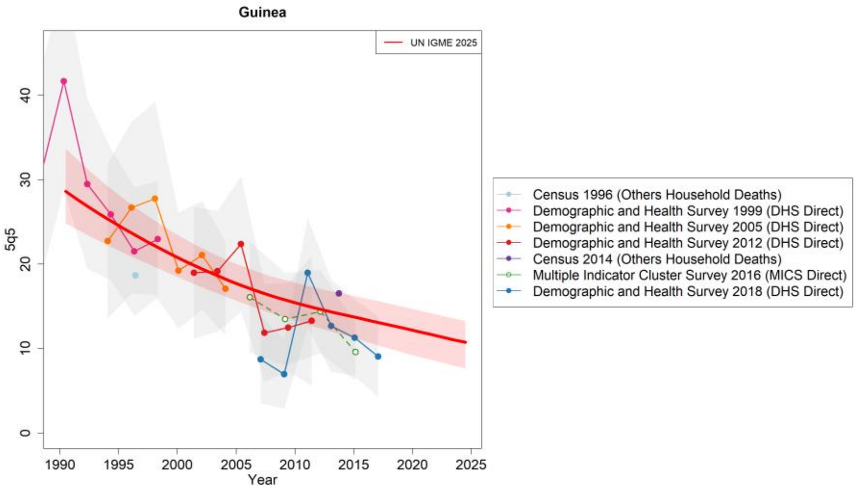
Guinea
UN IGME 2025
5q5
Year
Census 1996 (Others Household Deaths)
Demographic and Health Survey 1999 (DHS Direct)
Demographic and Health Survey 2005 (DHS Direct)
Demographic and Health Survey 2012 (DHS Direct)
Census 2014 (Others Household Deaths)
Multiple Indicator Cluster Survey 2016 (MICS Direct)
Demographic and Health Survey 2018 (DHS Direct)

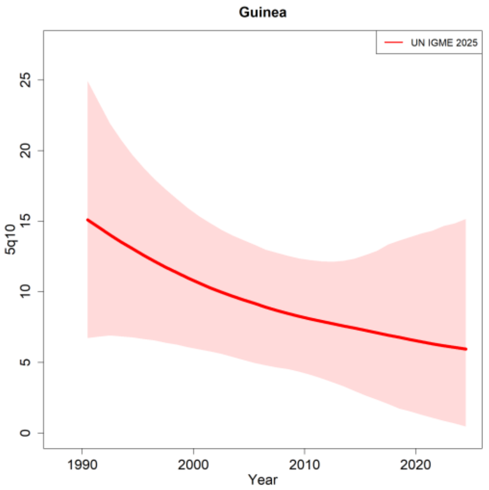
Guinea
UN IGME 2025
5q10
Year

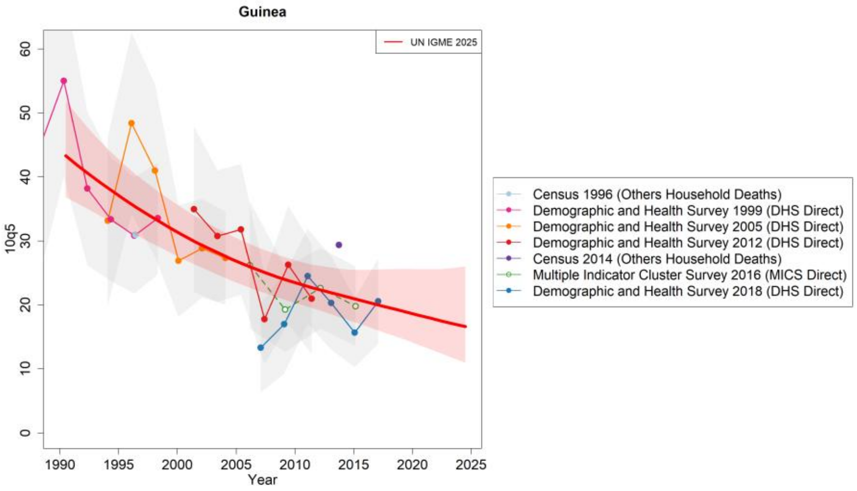
Guinea
UN IGME 2025
10q5
Year
Census 1996 (Others Household Deaths)
Demographic and Health Survey 1999 (DHS Direct)
Demographic and Health Survey 2005 (DHS Direct)
Demographic and Health Survey 2012 (DHS Direct)
Census 2014 (Others Household Deaths)
Multiple Indicator Cluster Survey 2016 (MICS Direct)
Demographic and Health Survey 2018 (DHS Direct)

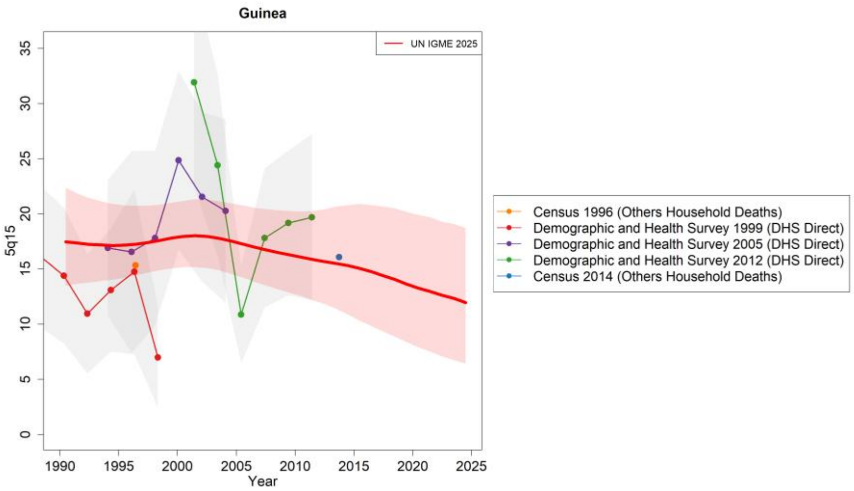
Guinea
UN IGME 2025
Census 1996 (Others Household Deaths)
Demographic and Health Survey 1999 (DHS Direct)
Demographic and Health Survey 2005 (DHS Direct)
Demographic and Health Survey 2012 (DHS Direct)
Census 2014 (Others Household Deaths)
5q15
Year
0
5
10
15
20
25
30
35
1990
1995
2000
2005
2010
2015
2020
2025

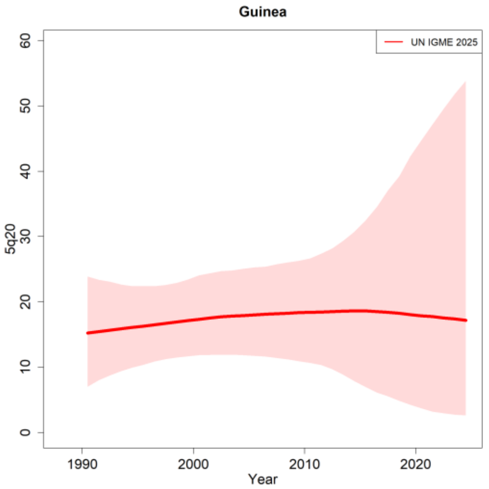
Guinea
UN IGME 2025
5q20
Year
0
10
20
30
40
50
60
1990
2000
2010
2020

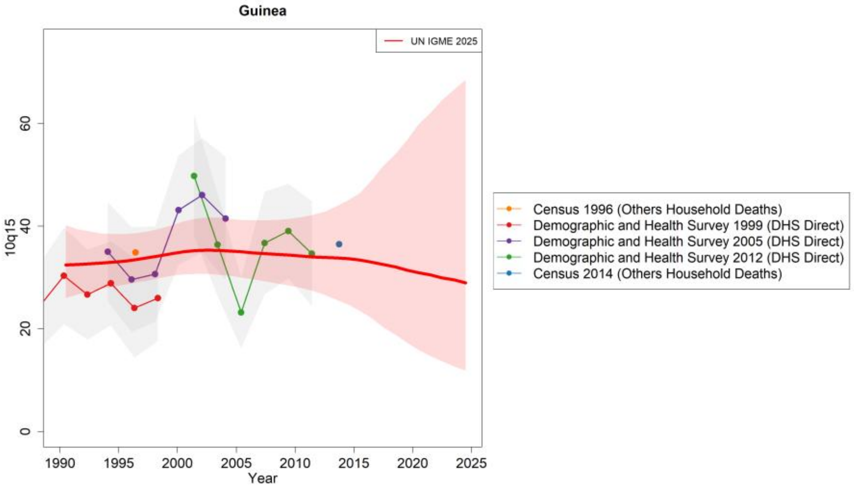
Guinea
UN IGME 2025
Census 1996 (Others Household Deaths)
Demographic and Health Survey 1999 (DHS Direct)
Demographic and Health Survey 2005 (DHS Direct)
Demographic and Health Survey 2012 (DHS Direct)
Census 2014 (Others Household Deaths)
10q15
Year
0
20
40
60
1990
1995
2000
2005
2010
2015
2020
2025

Guinea-Bissau (GNB)

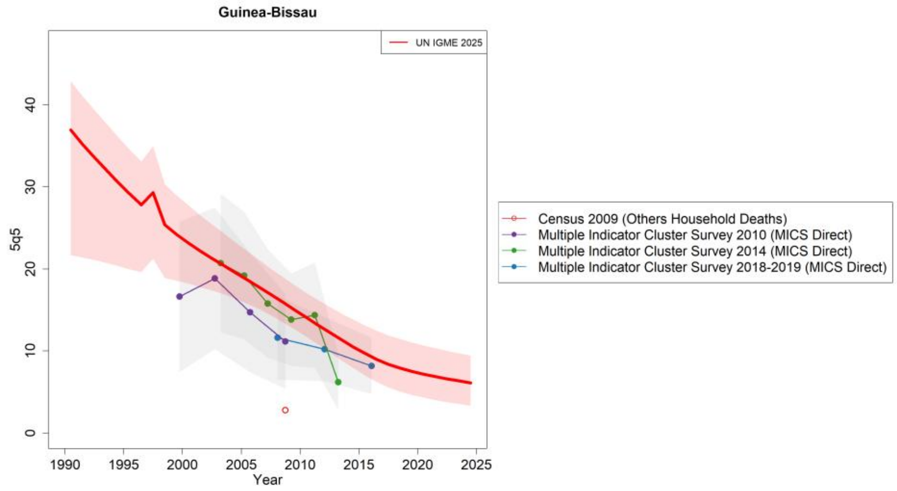

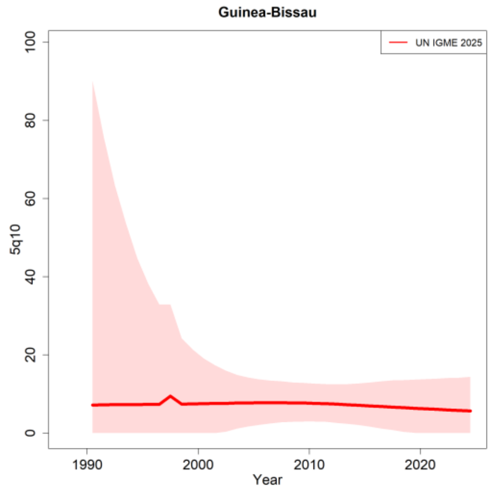

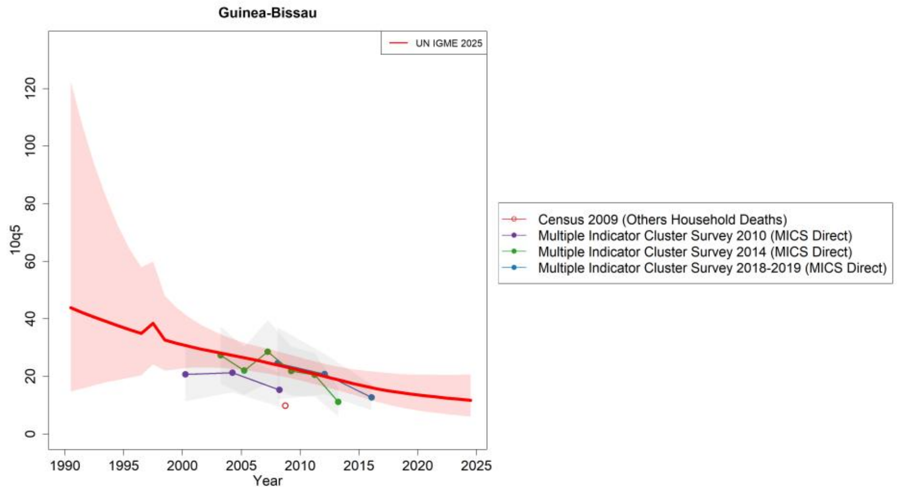

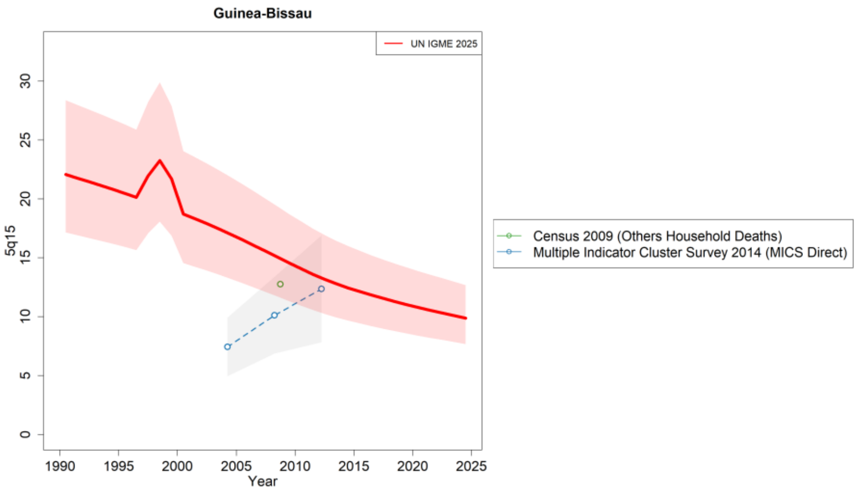
Guinea-Bissau
UN IGME 2025
Census 2009 (Others Household Deaths)
Multiple Indicator Cluster Survey 2014 (MICS Direct)
5q15
Year
0
5
10
15
20
25
30
1990
1995
2000
2005
2010
2015
2020
2025

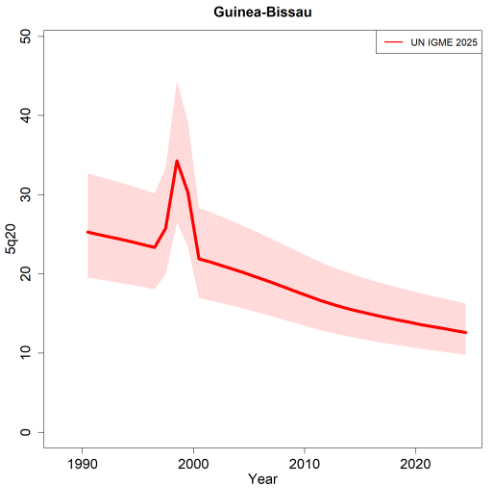
Guinea-Bissau
UN IGME 2025
5q20
Year
0
10
20
30
40
50
1990
2000
2010
2020

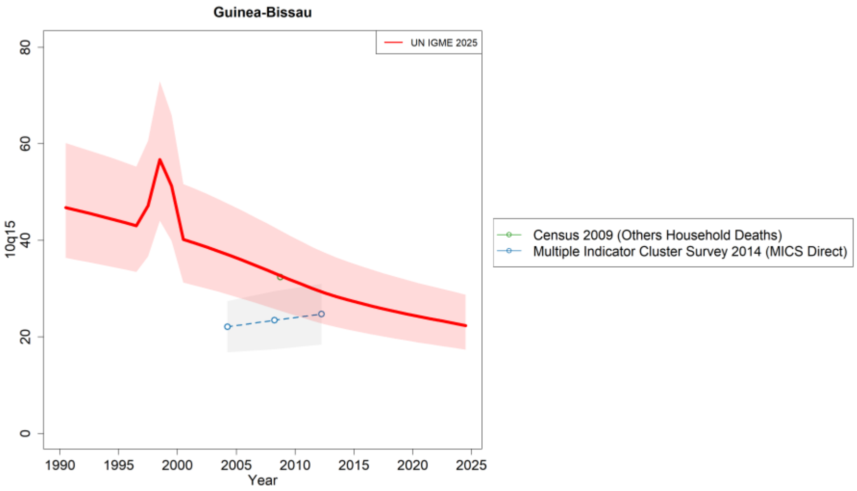
Guinea-Bissau
UN IGME 2025
Census 2009 (Others Household Deaths)
Multiple Indicator Cluster Survey 2014 (MICS Direct)
10q15
Year
0
20
40
60
80
1990
1995
2000
2005
2010
2015
2020
2025

Guyana (GUY)

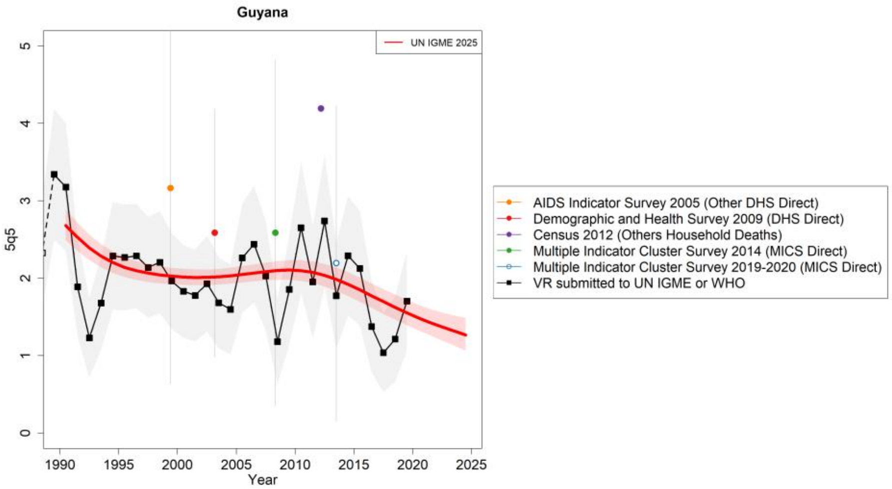
Guyana
UN IGME 2025
5q5
Year
AIDS Indicator Survey 2005 (Other DHS Direct)
Demographic and Health Survey 2009 (DHS Direct)
Census 2012 (Others Household Deaths)
Multiple Indicator Cluster Survey 2014 (MICS Direct)
Multiple Indicator Cluster Survey 2019-2020 (MICS Direct)
VR submitted to UN IGME or WHO

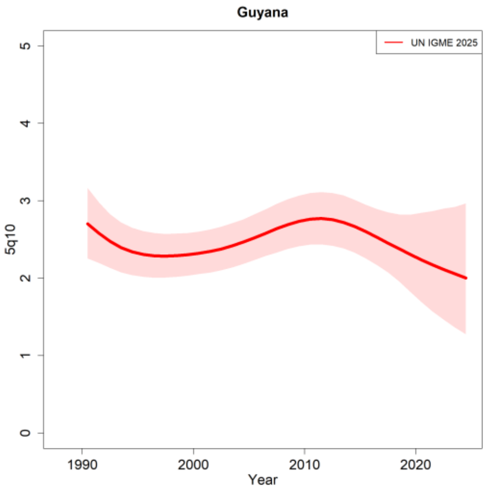
Guyana
UN IGME 2025
5q10
Year

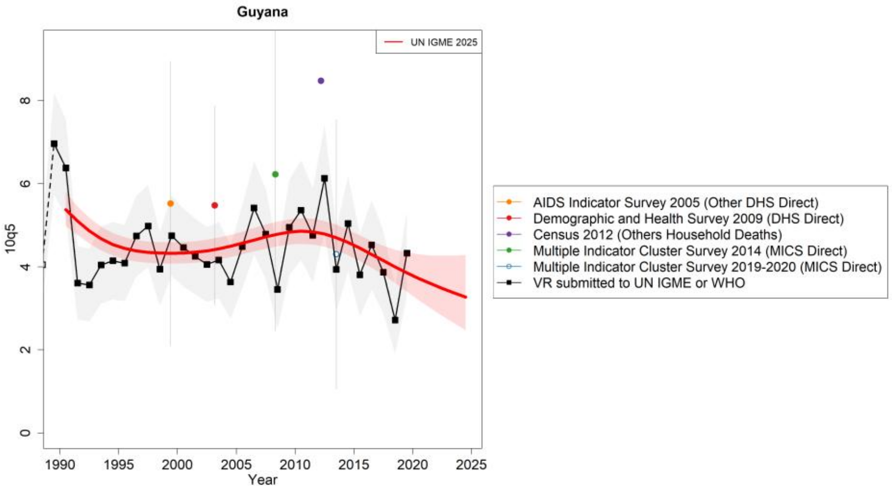
Guyana
UN IGME 2025
10q5
Year
AIDS Indicator Survey 2005 (Other DHS Direct)
Demographic and Health Survey 2009 (DHS Direct)
Census 2012 (Others Household Deaths)
Multiple Indicator Cluster Survey 2014 (MICS Direct)
Multiple Indicator Cluster Survey 2019-2020 (MICS Direct)
VR submitted to UN IGME or WHO

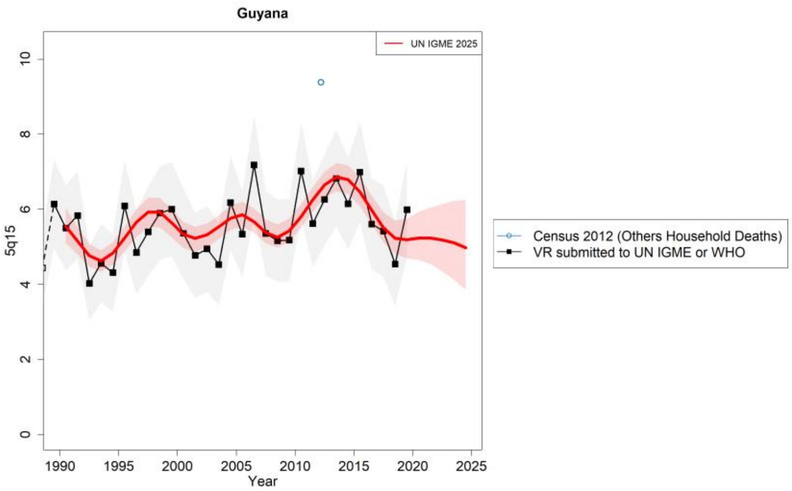
Guyana
UN IGME 2025
Census 2012 (Others Household Deaths)
VR submitted to UN IGME or WHO
5q15
Year

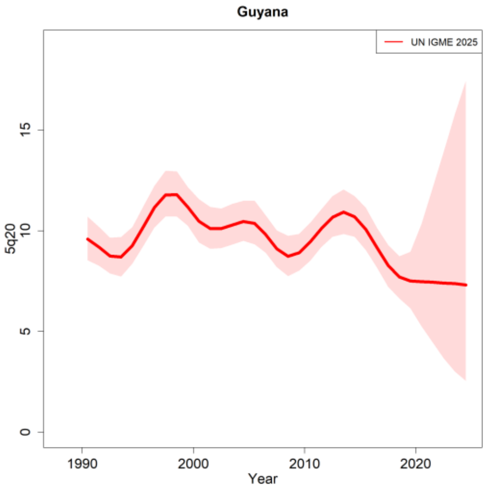
Guyana
UN IGME 2025
5q20
Year

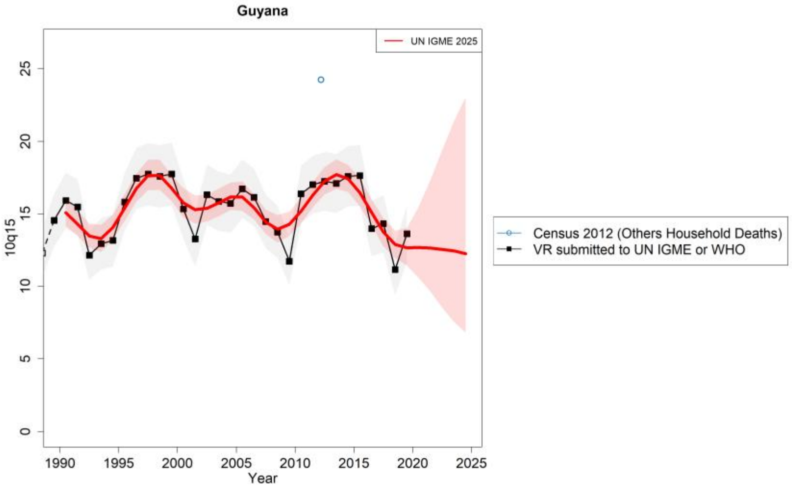
Guyana
UN IGME 2025
Census 2012 (Others Household Deaths)
VR submitted to UN IGME or WHO
10q15
Year

Haiti (HTI)

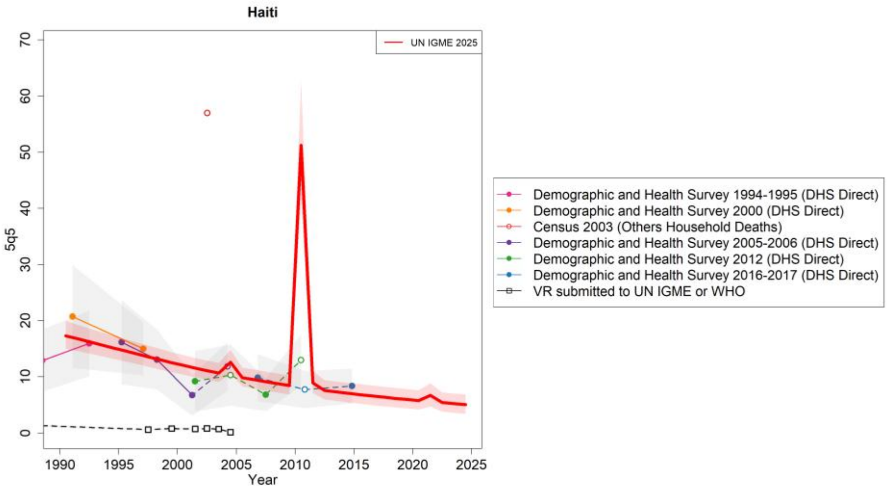
Haiti
UN IGME 2025
5q5
Year
Demographic and Health Survey 1994-1995 (DHS Direct)
Demographic and Health Survey 2000 (DHS Direct)
Census 2003 (Others Household Deaths)
Demographic and Health Survey 2005-2006 (DHS Direct)
Demographic and Health Survey 2012 (DHS Direct)
Demographic and Health Survey 2016-2017 (DHS Direct)
VR submitted to UN IGME or WHO

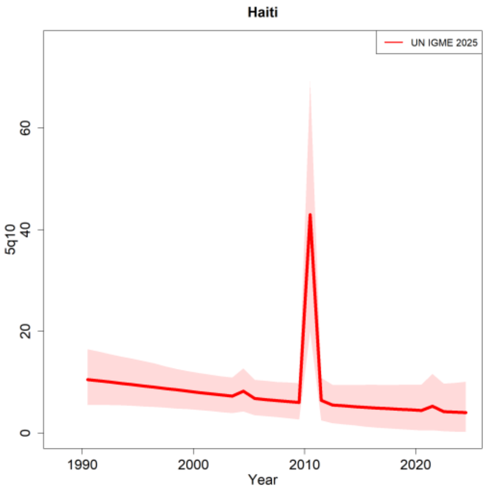
Haiti
UN IGME 2025
5q10
Year

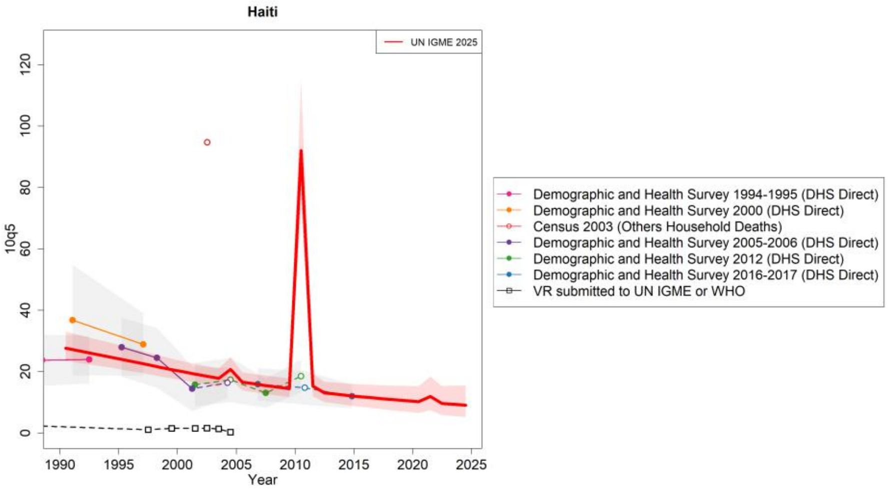
Haiti
UN IGME 2025
10q5
Year
Demographic and Health Survey 1994-1995 (DHS Direct)
Demographic and Health Survey 2000 (DHS Direct)
Census 2003 (Others Household Deaths)
Demographic and Health Survey 2005-2006 (DHS Direct)
Demographic and Health Survey 2012 (DHS Direct)
Demographic and Health Survey 2016-2017 (DHS Direct)
VR submitted to UN IGME or WHO

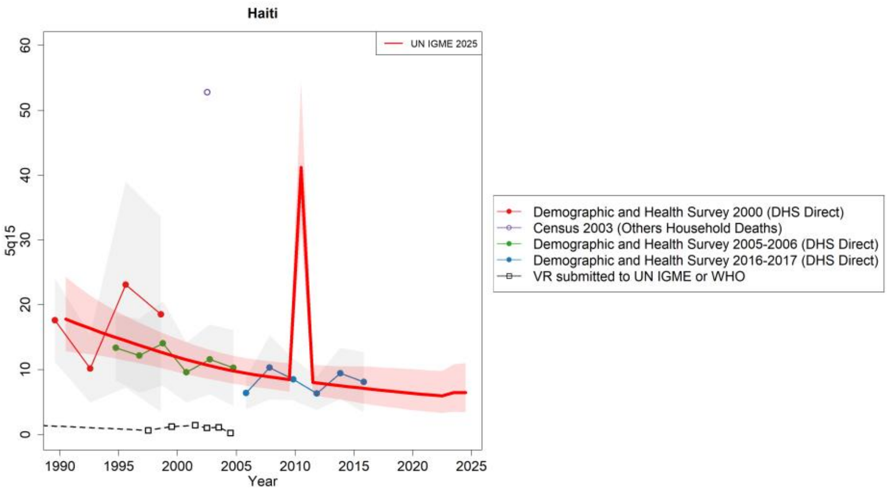
Haiti
UN IGME 2025
Demographic and Health Survey 2000 (DHS Direct)
Census 2003 (Others Household Deaths)
Demographic and Health Survey 2005-2006 (DHS Direct)
Demographic and Health Survey 2016-2017 (DHS Direct)
VR submitted to UN IGME or WHO
5q15
Year
0
10
20
30
40
50
60
1990
1995
2000
2005
2010
2015
2020
2025

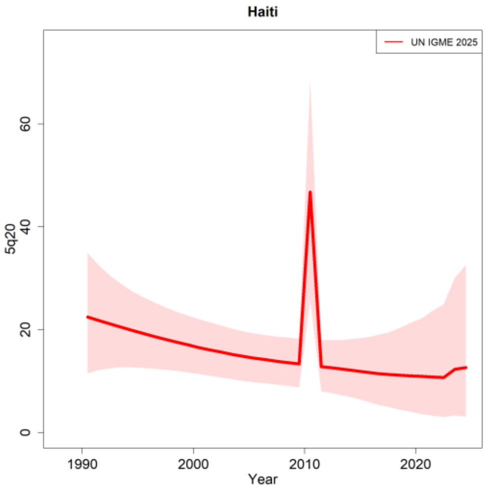
Haiti
UN IGME 2025
5q20
Year
0
20
40
60
1990
2000
2010
2020

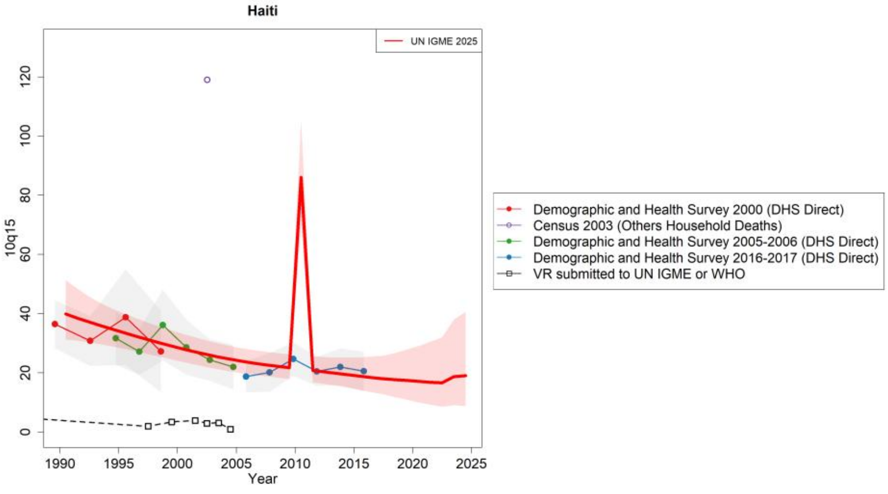
Haiti
UN IGME 2025
Demographic and Health Survey 2000 (DHS Direct)
Census 2003 (Others Household Deaths)
Demographic and Health Survey 2005-2006 (DHS Direct)
Demographic and Health Survey 2016-2017 (DHS Direct)
VR submitted to UN IGME or WHO
10q15
Year
0
20
40
60
80
100
120
1990
1995
2000
2005
2010
2015
2020
2025

Honduras (HND)

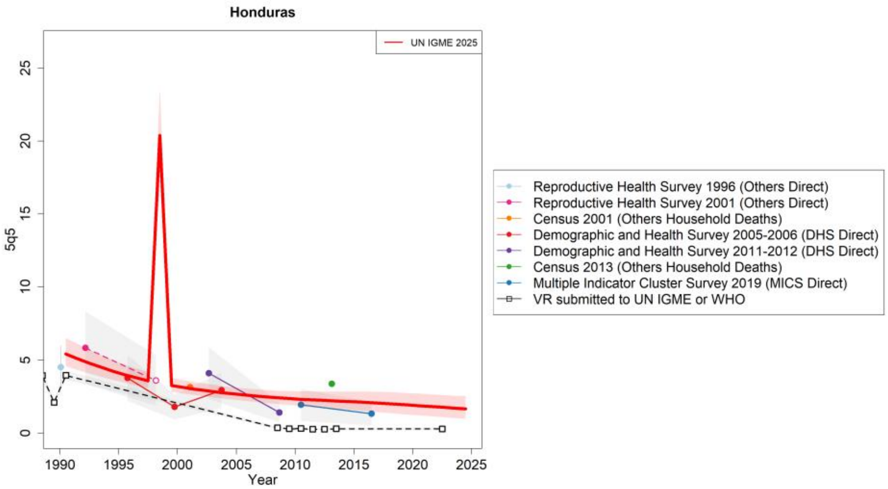

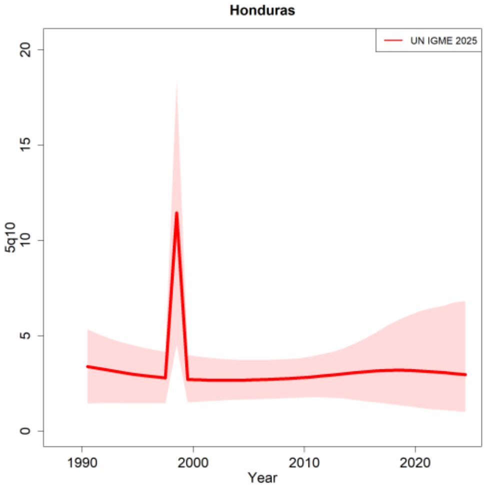

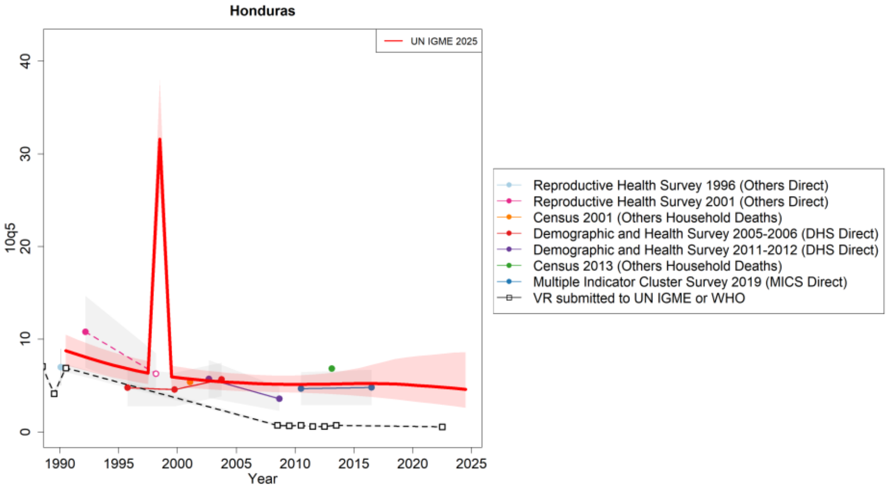

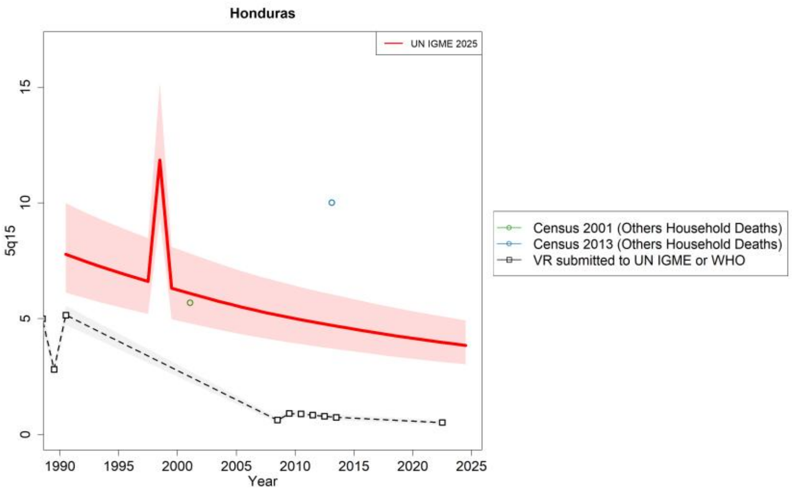
Honduras
UN IGME 2025
Census 2001 (Others Household Deaths)
Census 2013 (Others Household Deaths)
VR submitted to UN IGME or WHO
5q15
Year
0
5
10
15
1990
1995
2000
2005
2010
2015
2020
2025

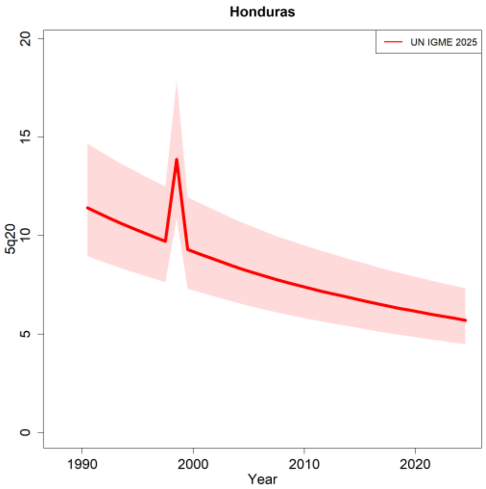
Honduras
UN IGME 2025
5q20
Year
0
5
10
15
20
1990
2000
2010
2020

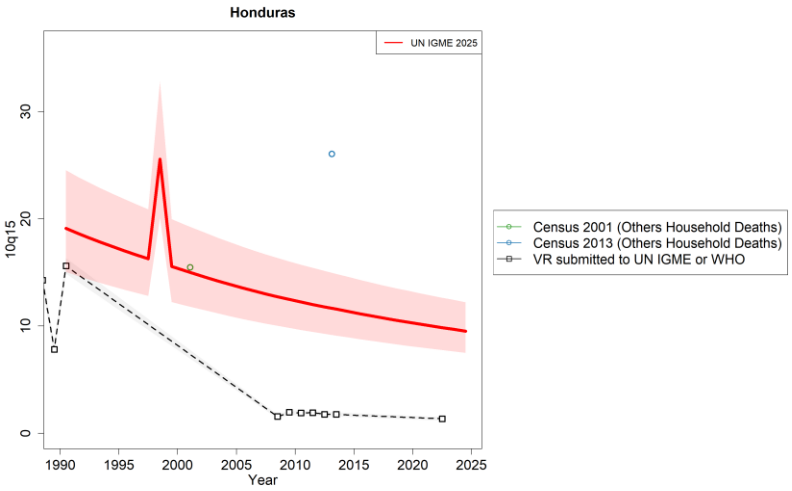
Honduras
UN IGME 2025
Census 2001 (Others Household Deaths)
Census 2013 (Others Household Deaths)
VR submitted to UN IGME or WHO
10q15
Year
0
10
20
30
1990
1995
2000
2005
2010
2015
2020
2025

Hungary (HUN)

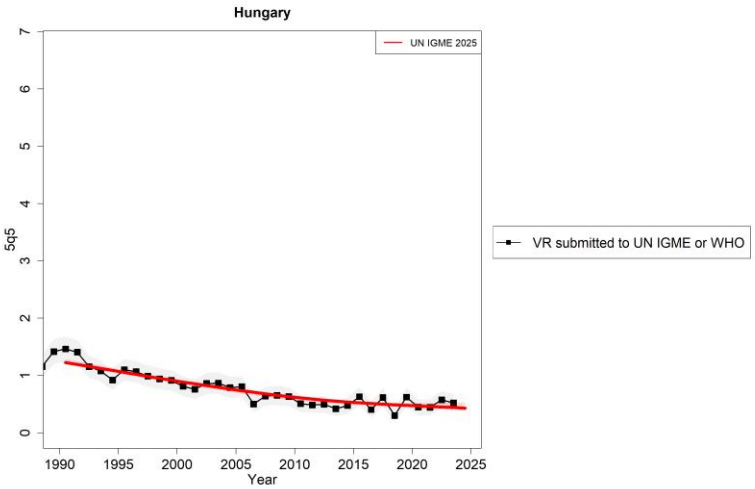
Hungary
UN IGME 2025
5q5
Year
VR submitted to UN IGME or WHO

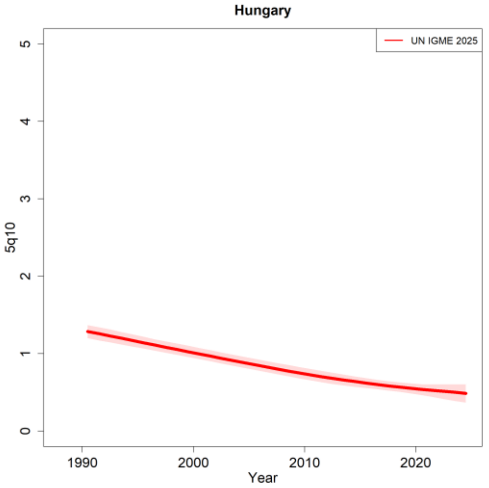
Hungary
UN IGME 2025
5q10
Year

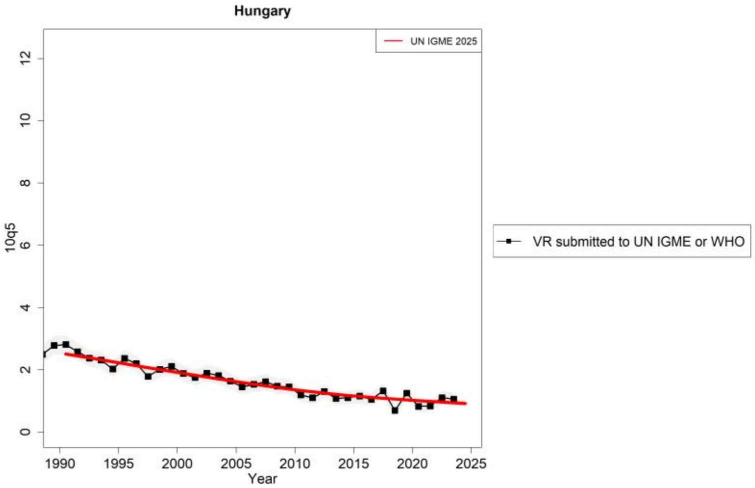
Hungary
UN IGME 2025
10q5
Year
VR submitted to UN IGME or WHO

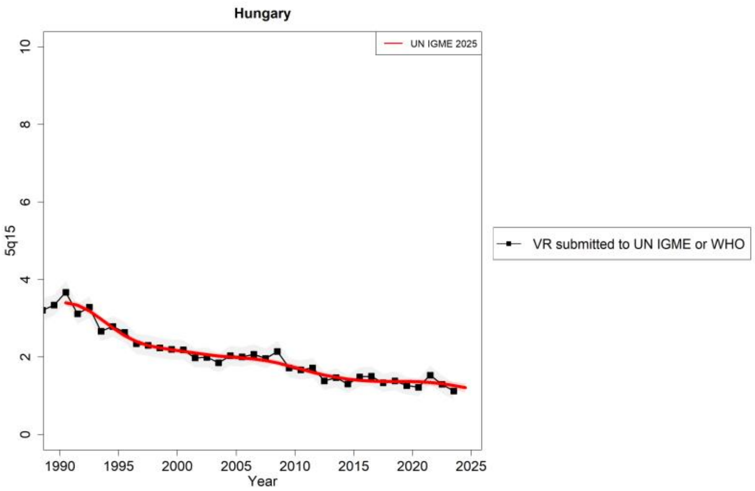
Hungary
UN IGME 2025
VR submitted to UN IGME or WHO
5q15
Year

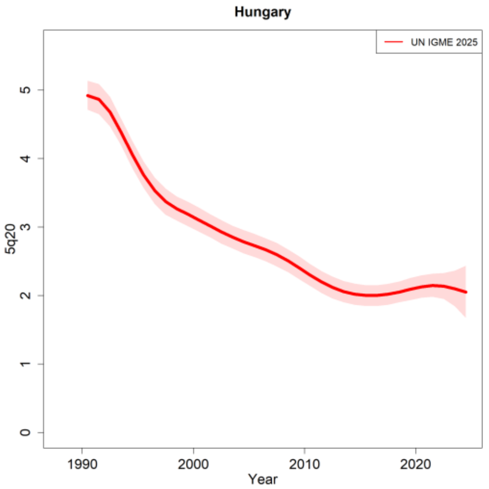
Hungary
UN IGME 2025
5q20
Year

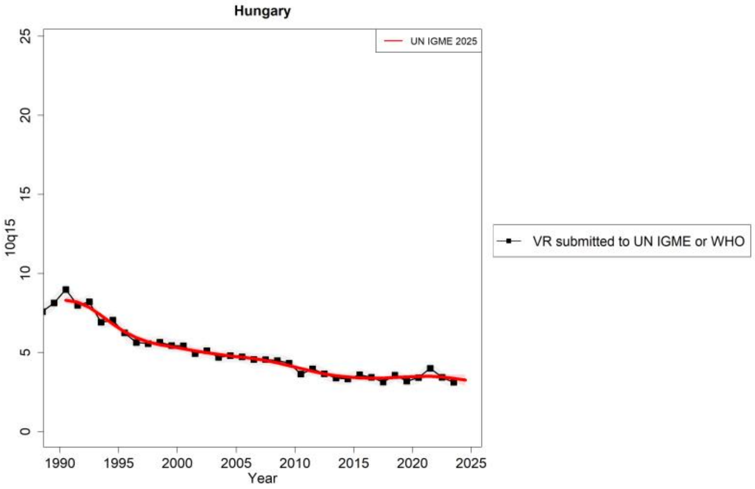
Hungary
UN IGME 2025
VR submitted to UN IGME or WHO
10q15
Year

Iceland (ISL)

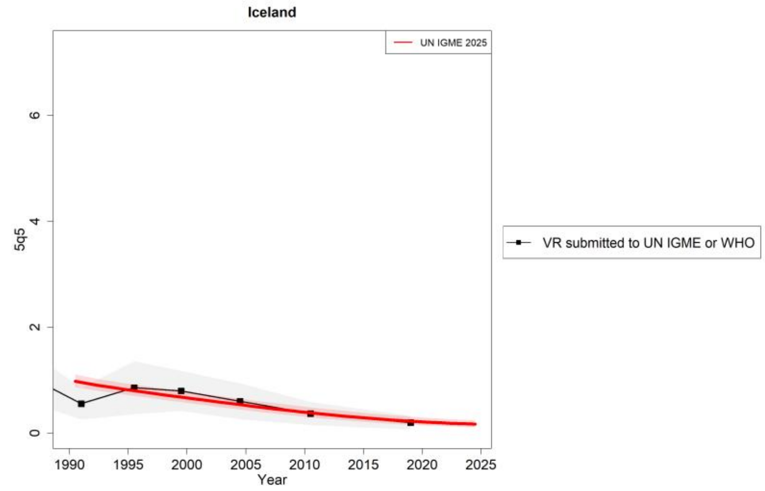
Iceland
UN IGME 2025
VR submitted to UN IGME or WHO
5q5
0
2
4
6
1990
1995
2000
2005
2010
2015
2020
2025
Year

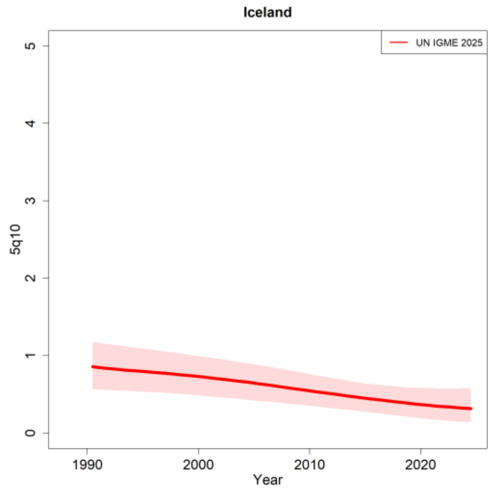
Iceland
UN IGME 2025
5q10
0
1
2
3
4
5
1990
2000
2010
2020
Year

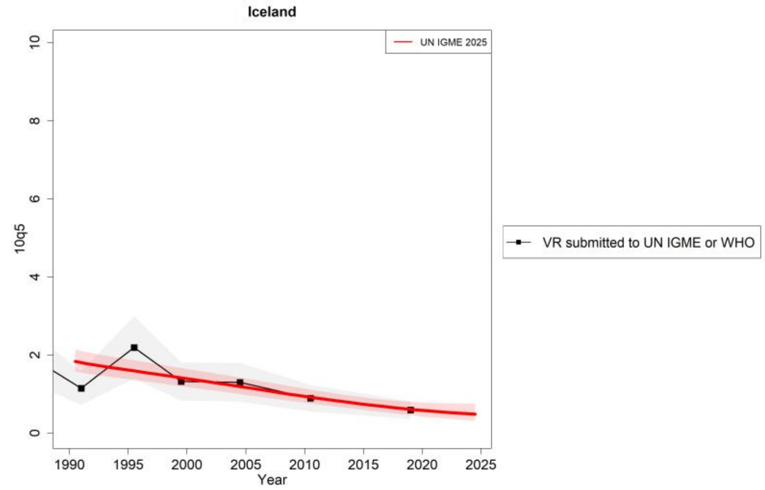
Iceland
UN IGME 2025
VR submitted to UN IGME or WHO
10q5
0
2
4
6
8
10
1990
1995
2000
2005
2010
2015
2020
2025
Year

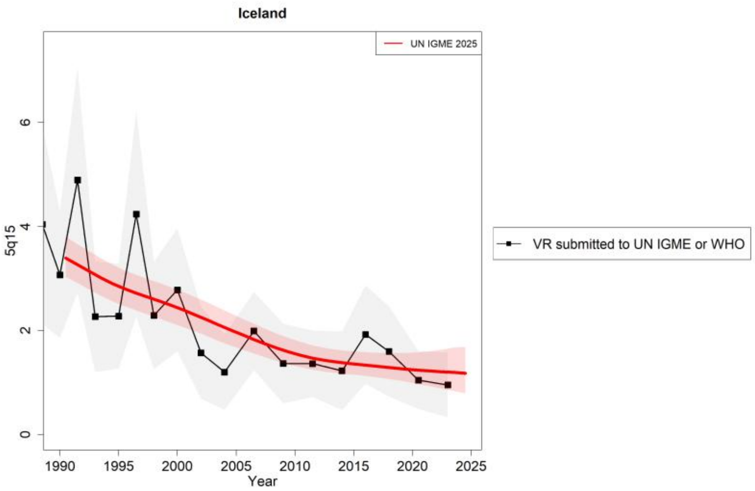
Iceland
UN IGME 2025
VR submitted to UN IGME or WHO
5q15
Year
0
2
4
6
1990
1995
2000
2005
2010
2015
2020
2025

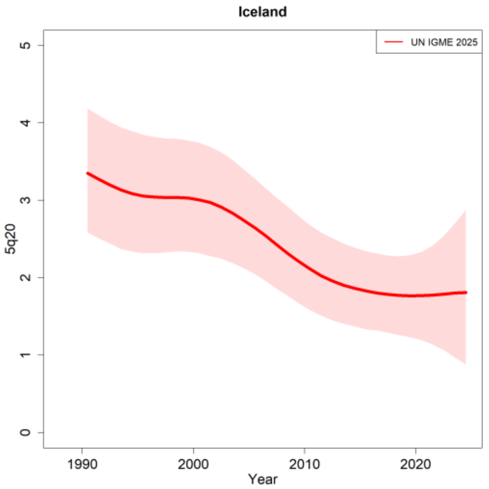
Iceland
UN IGME 2025
5q20
Year
0
1
2
3
4
5
1990
2000
2010
2020

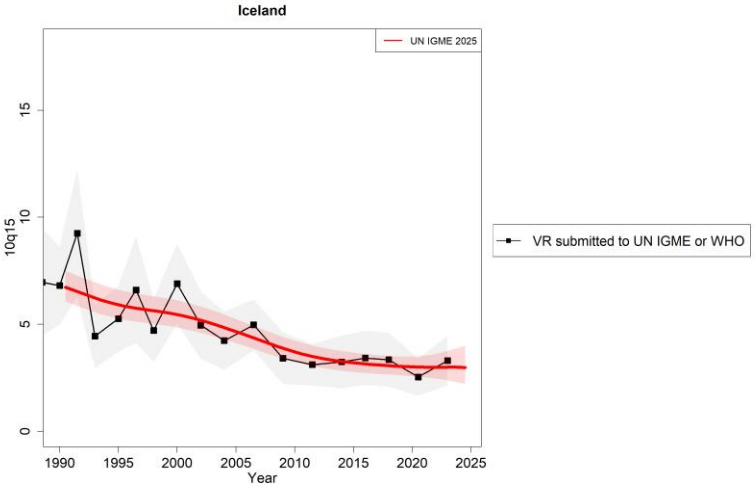
Iceland
UN IGME 2025
VR submitted to UN IGME or WHO
10q15
Year
0
5
10
15
1990
1995
2000
2005
2010
2015
2020
2025

India (IND)

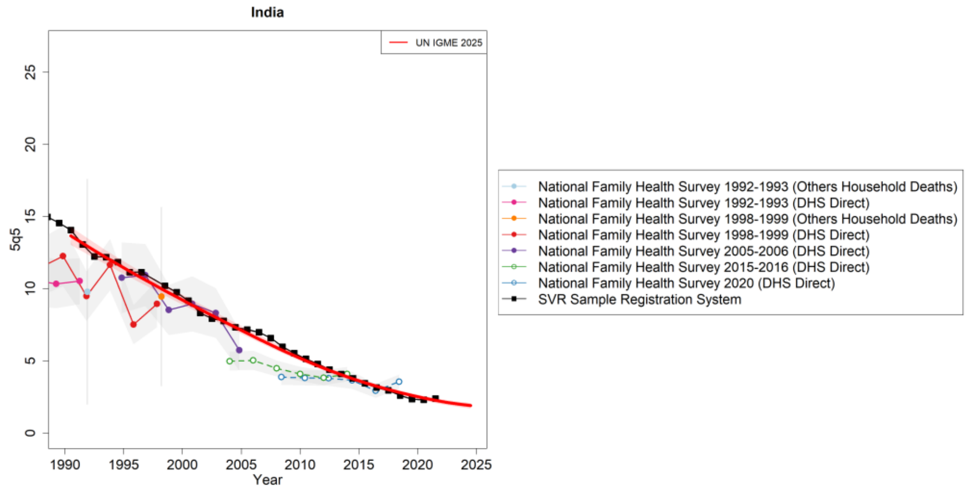

India
UN IGME 2025
5q5
Year
National Family Health Survey 1992-1993 (Others Household Deaths)
National Family Health Survey 1992-1993 (DHS Direct)
National Family Health Survey 1998-1999 (Others Household Deaths)
National Family Health Survey 1998-1999 (DHS Direct)
National Family Health Survey 2005-2006 (DHS Direct)
National Family Health Survey 2015-2016 (DHS Direct)
National Family Health Survey 2020 (DHS Direct)
SVR Sample Registration System

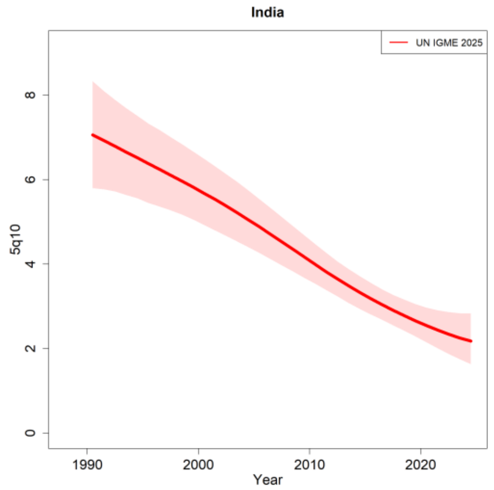

India
UN IGME 2025
5q10
Year

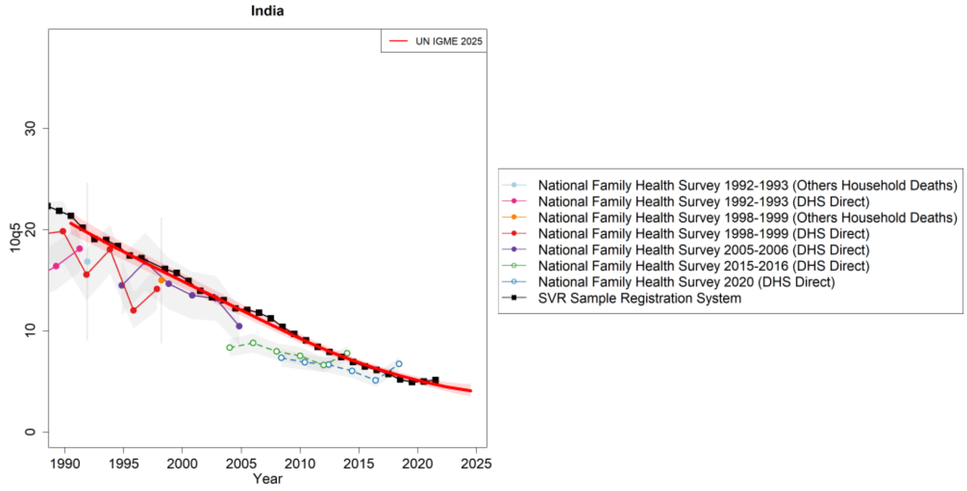

India
UN IGME 2025
10q5
Year
National Family Health Survey 1992-1993 (Others Household Deaths)
National Family Health Survey 1992-1993 (DHS Direct)
National Family Health Survey 1998-1999 (Others Household Deaths)
National Family Health Survey 1998-1999 (DHS Direct)
National Family Health Survey 2005-2006 (DHS Direct)
National Family Health Survey 2015-2016 (DHS Direct)
National Family Health Survey 2020 (DHS Direct)
SVR Sample Registration System

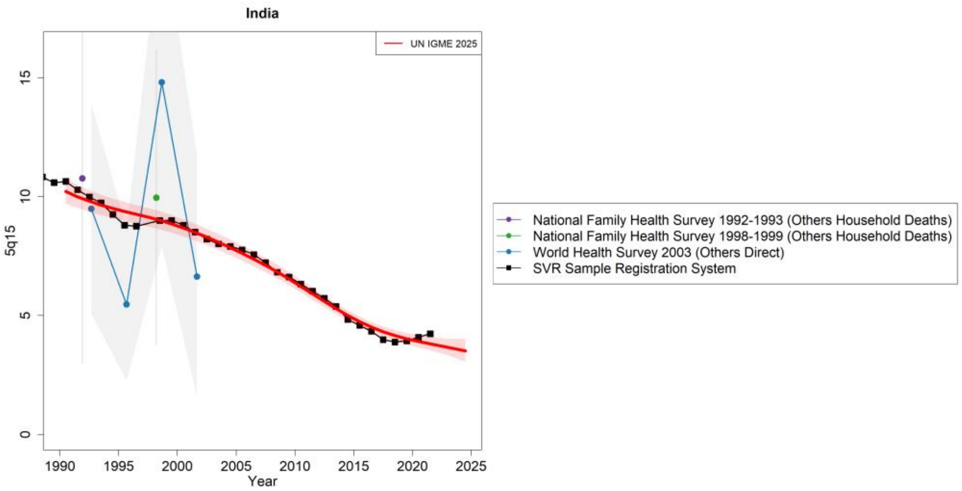
India
UN IGME 2025
5q15
Year
National Family Health Survey 1992-1993 (Others Household Deaths)
National Family Health Survey 1998-1999 (Others Household Deaths)
World Health Survey 2003 (Others Direct)
SVR Sample Registration System

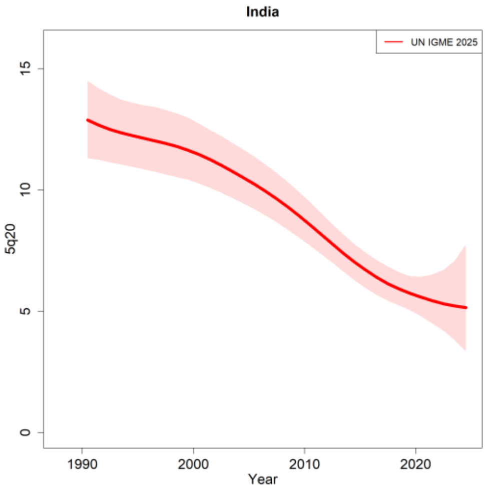
India
UN IGME 2025
5q20
Year

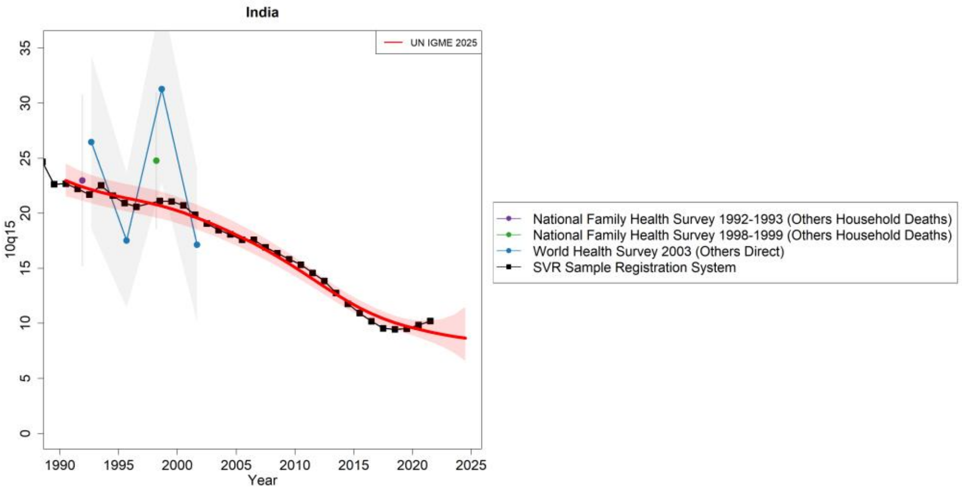
India
UN IGME 2025
10q15
Year
National Family Health Survey 1992-1993 (Others Household Deaths)
National Family Health Survey 1998-1999 (Others Household Deaths)
World Health Survey 2003 (Others Direct)
SVR Sample Registration System

Indonesia (IDN)

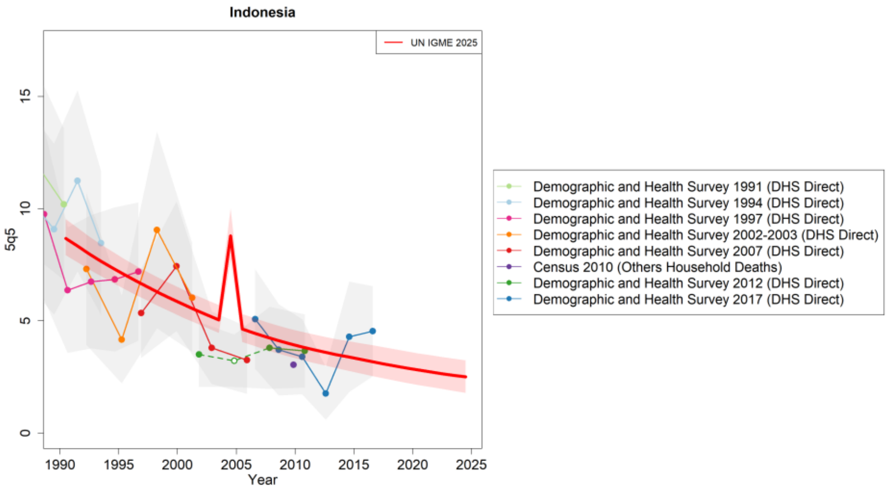

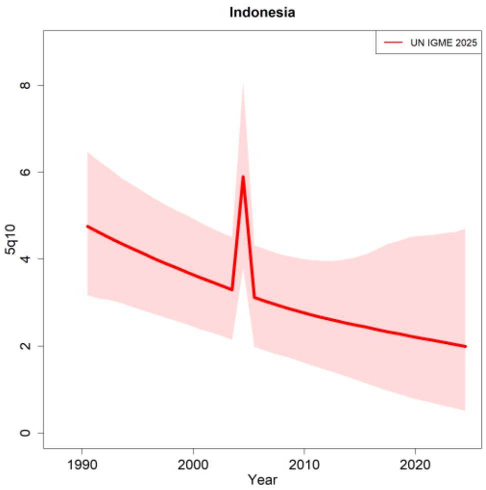

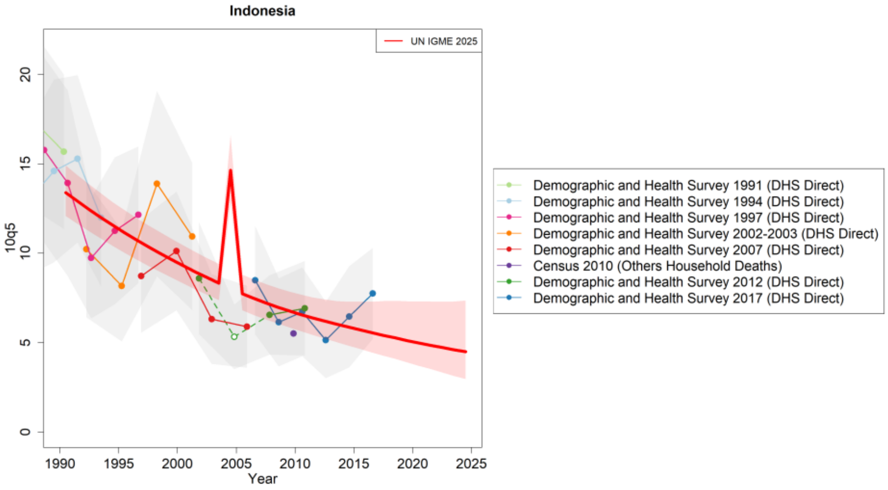

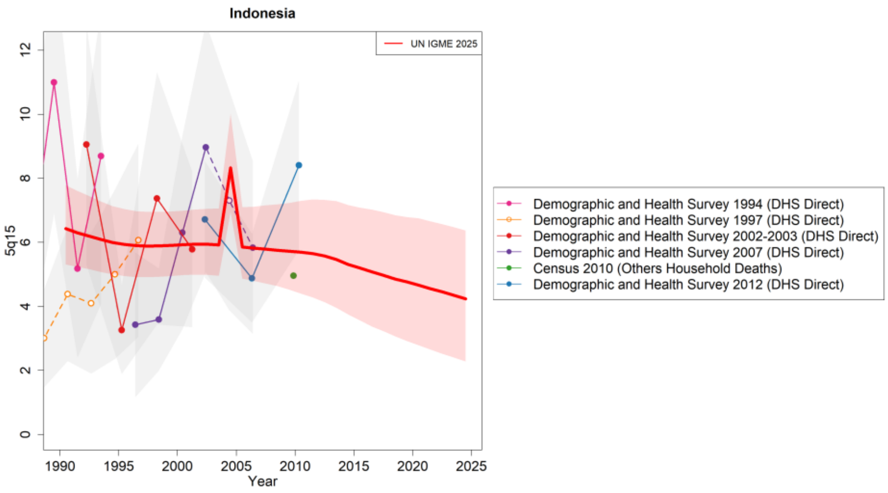
Indonesia
UN IGME 2025
5q15
Year
Demographic and Health Survey 1994 (DHS Direct)
Demographic and Health Survey 1997 (DHS Direct)
Demographic and Health Survey 2002-2003 (DHS Direct)
Demographic and Health Survey 2007 (DHS Direct)
Census 2010 (Others Household Deaths)
Demographic and Health Survey 2012 (DHS Direct)

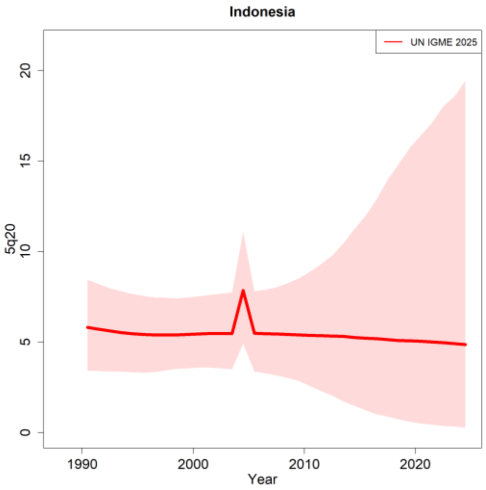
Indonesia
UN IGME 2025
5q20
Year

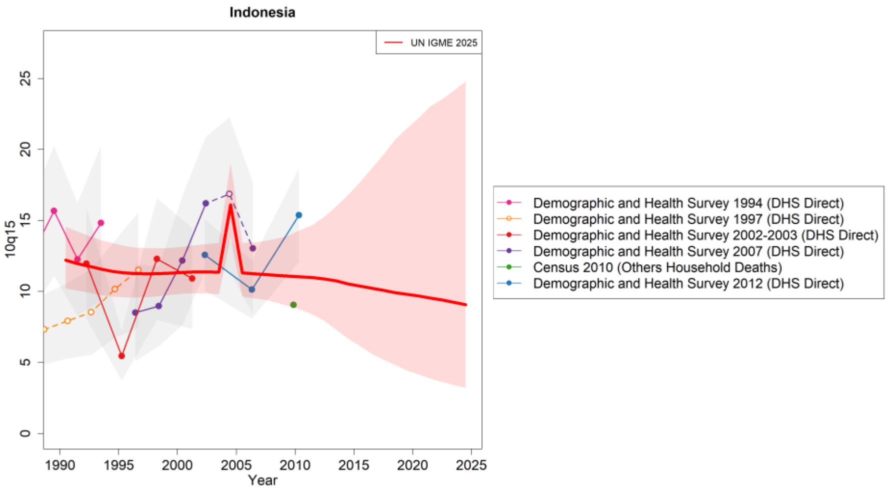
Indonesia
UN IGME 2025
10q15
Year
Demographic and Health Survey 1994 (DHS Direct)
Demographic and Health Survey 1997 (DHS Direct)
Demographic and Health Survey 2002-2003 (DHS Direct)
Demographic and Health Survey 2007 (DHS Direct)
Census 2010 (Others Household Deaths)
Demographic and Health Survey 2012 (DHS Direct)

Iran (Islamic Republic of) (IRN)

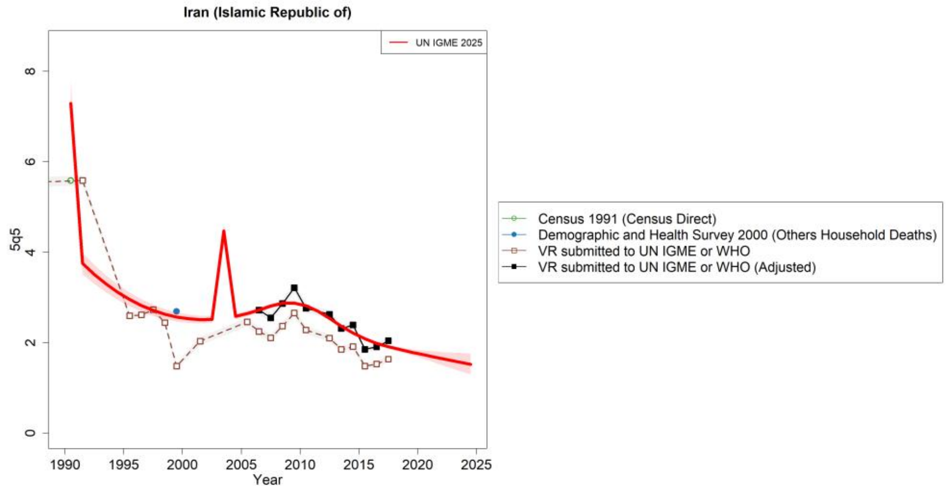

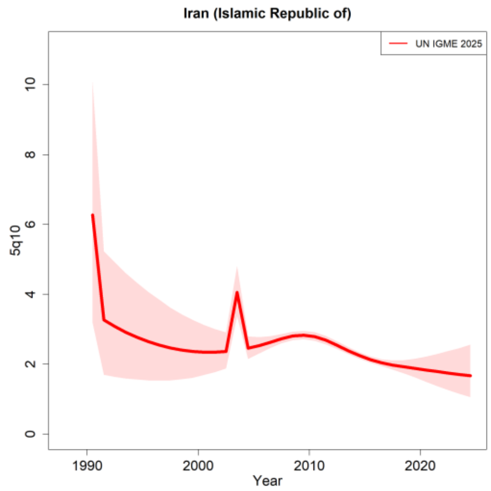

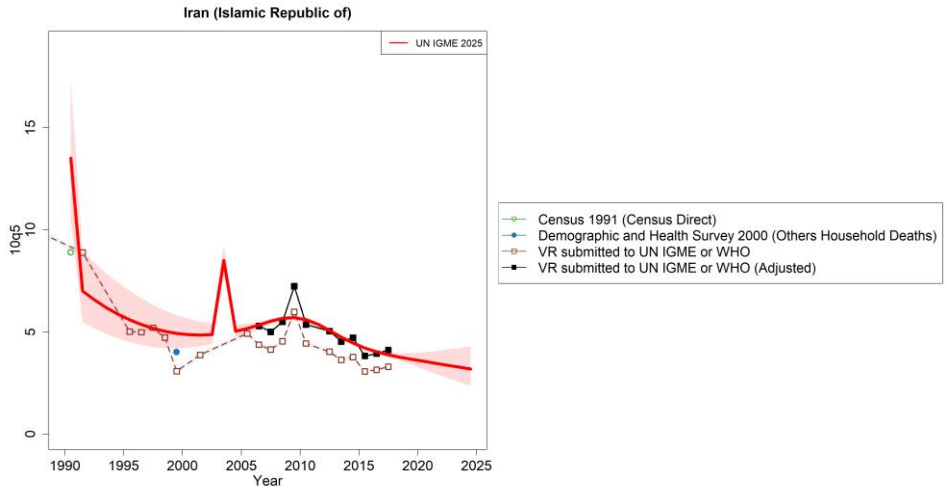

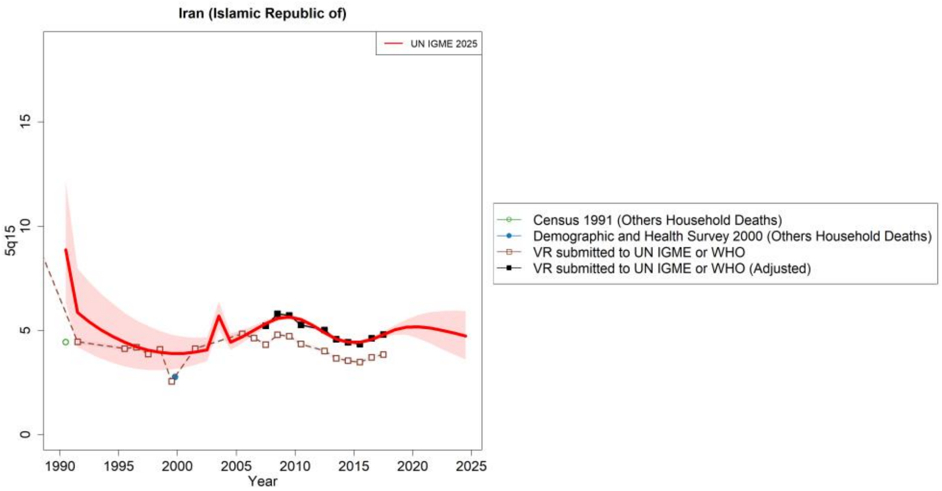

Iran (Islamic Republic of)
UN IGME 2025
Census 1991 (Others Household Deaths)
Demographic and Health Survey 2000 (Others Household Deaths)
VR submitted to UN IGME or WHO
VR submitted to UN IGME or WHO (Adjusted)
5q15
Year
0
5
10
15
1990
1995
2000
2005
2010
2015
2020
2025

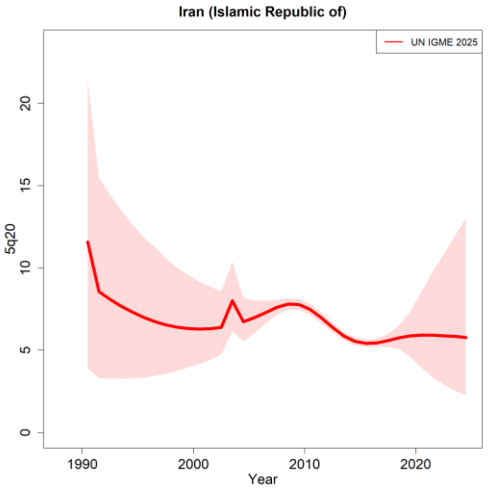

Iran (Islamic Republic of)
UN IGME 2025
5q20
Year
0
5
10
15
20
1990
2000
2010
2020

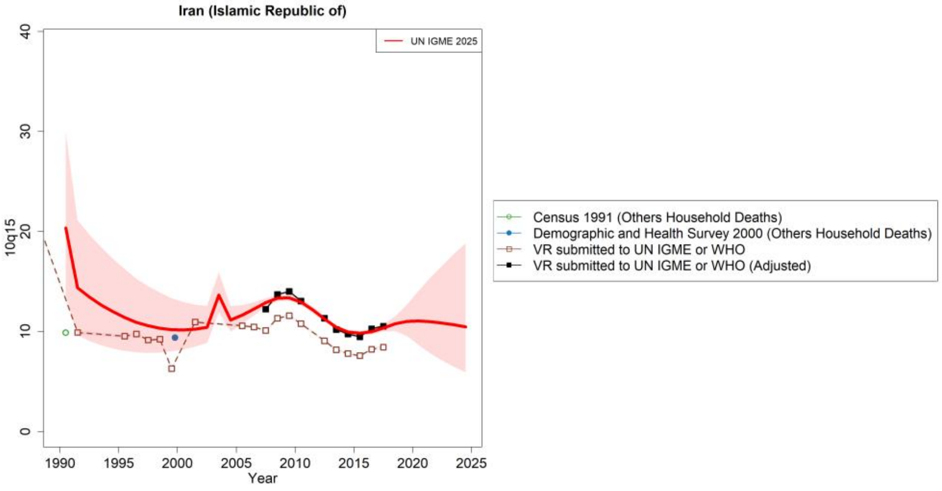

Iran (Islamic Republic of)
UN IGME 2025
Census 1991 (Others Household Deaths)
Demographic and Health Survey 2000 (Others Household Deaths)
VR submitted to UN IGME or WHO
VR submitted to UN IGME or WHO (Adjusted)
10q15
Year
0
10
20
30
40
1990
1995
2000
2005
2010
2015
2020
2025

Iraq (IRQ)

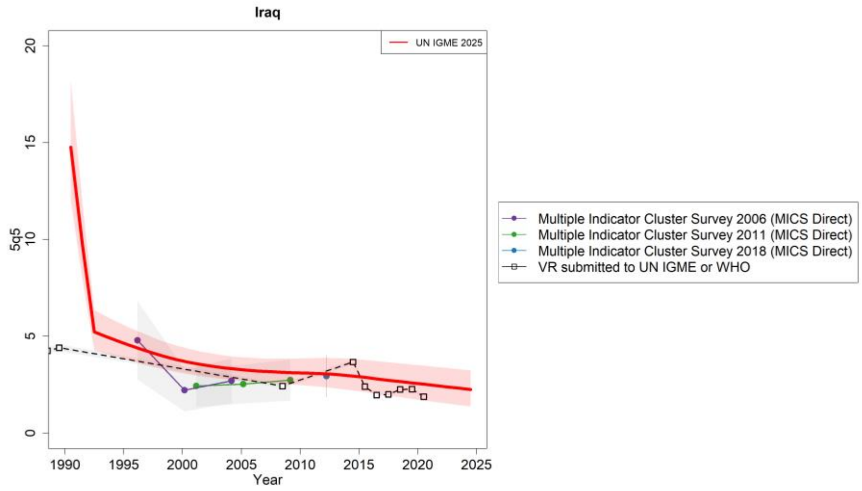
Iraq
UN IGME 2025
Multiple Indicator Cluster Survey 2006 (MICS Direct)
Multiple Indicator Cluster Survey 2011 (MICS Direct)
Multiple Indicator Cluster Survey 2018 (MICS Direct)
VR submitted to UN IGME or WHO
5q5
0
5
10
15
20
1990
1995
2000
2005
2010
2015
2020
2025
Year

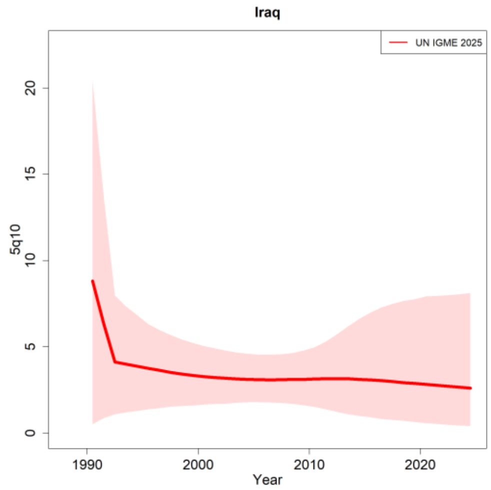
Iraq
UN IGME 2025
5q10
0
5
10
15
20
1990
2000
2010
2020
Year

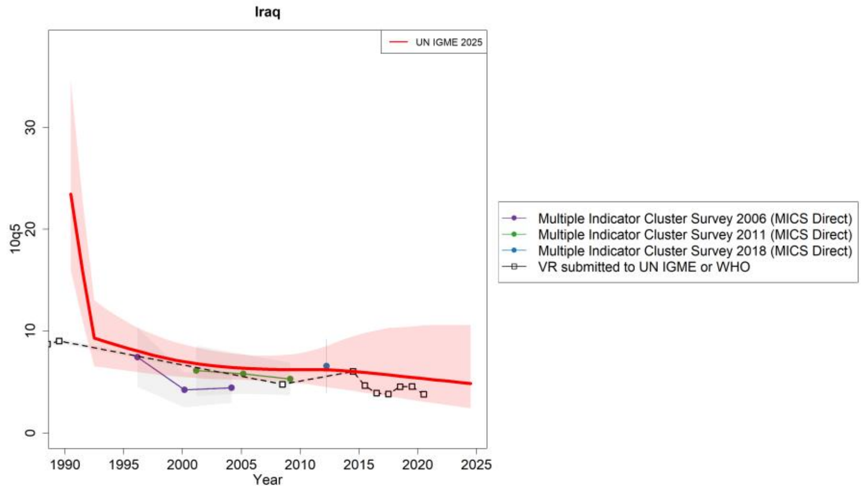
Iraq
UN IGME 2025
Multiple Indicator Cluster Survey 2006 (MICS Direct)
Multiple Indicator Cluster Survey 2011 (MICS Direct)
Multiple Indicator Cluster Survey 2018 (MICS Direct)
VR submitted to UN IGME or WHO
10q5
0
10
20
30
1990
1995
2000
2005
2010
2015
2020
2025
Year

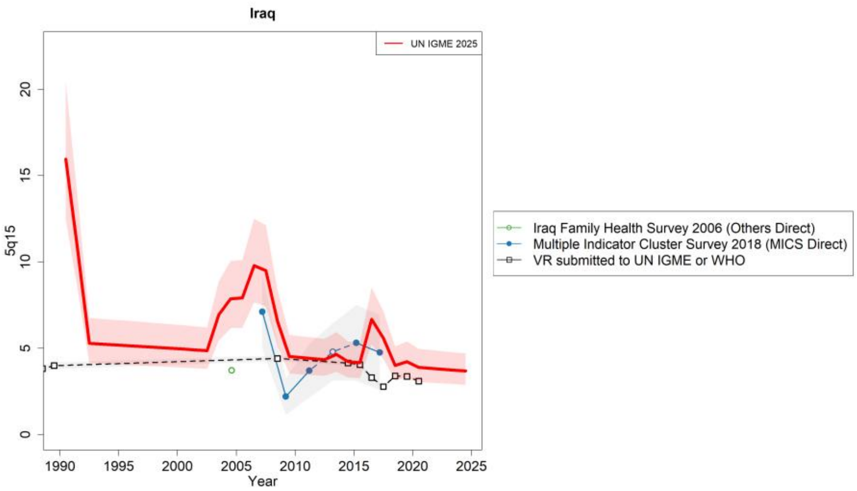
Iraq
UN IGME 2025
5q15
Year
Iraq Family Health Survey 2006 (Others Direct)
Multiple Indicator Cluster Survey 2018 (MICS Direct)
VR submitted to UN IGME or WHO

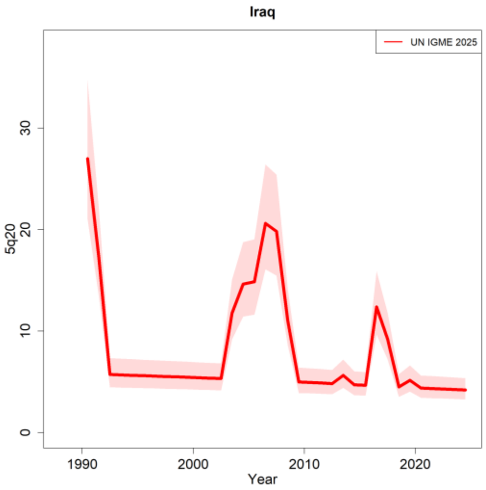
Iraq
UN IGME 2025
5q20
Year

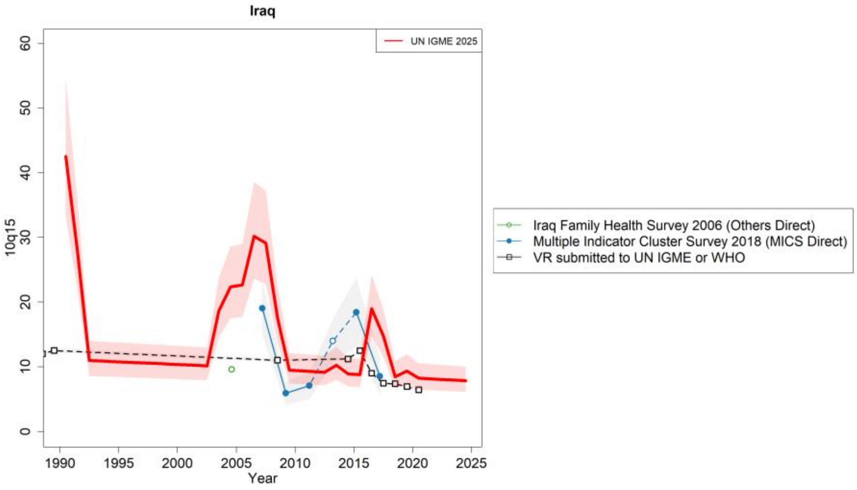
Iraq
UN IGME 2025
10q15
Year
Iraq Family Health Survey 2006 (Others Direct)
Multiple Indicator Cluster Survey 2018 (MICS Direct)
VR submitted to UN IGME or WHO

Ireland (IRL)

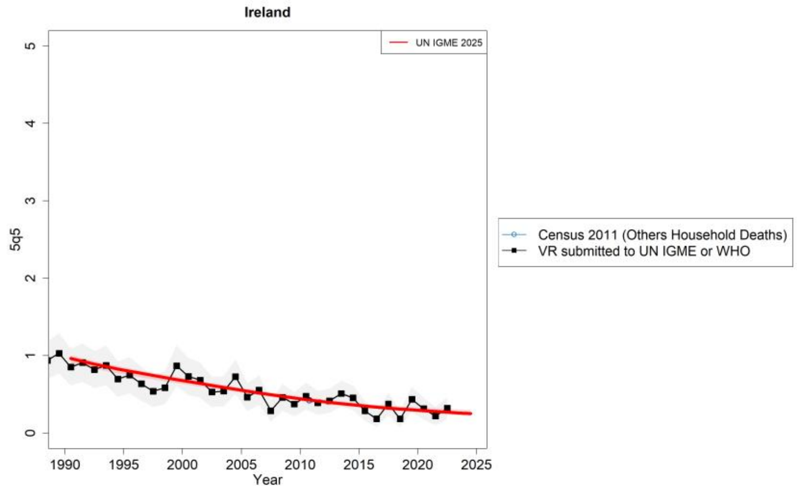

Ireland
UN IGME 2025
5q5
Year
Census 2011 (Others Household Deaths)
VR submitted to UN IGME or WHO

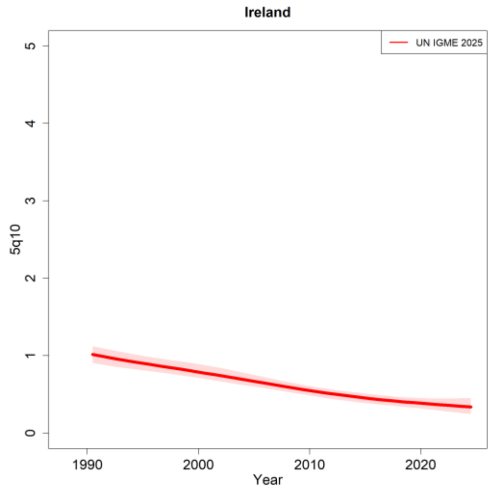

Ireland
UN IGME 2025
5q10
Year

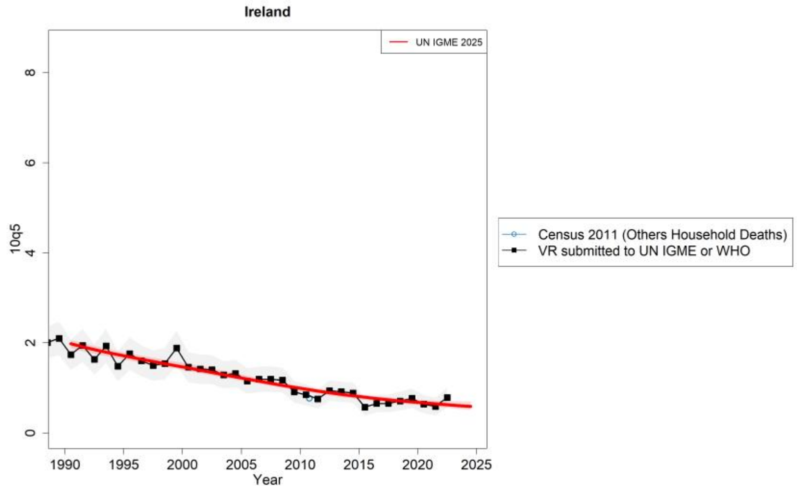

Ireland
UN IGME 2025
10q5
Year
Census 2011 (Others Household Deaths)
VR submitted to UN IGME or WHO

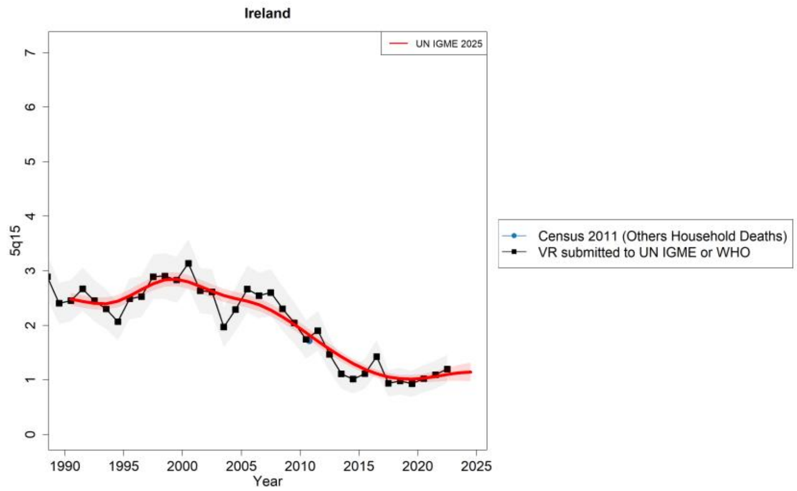
Ireland
UN IGME 2025
5q15
Year
Census 2011 (Others Household Deaths)
VR submitted to UN IGME or WHO

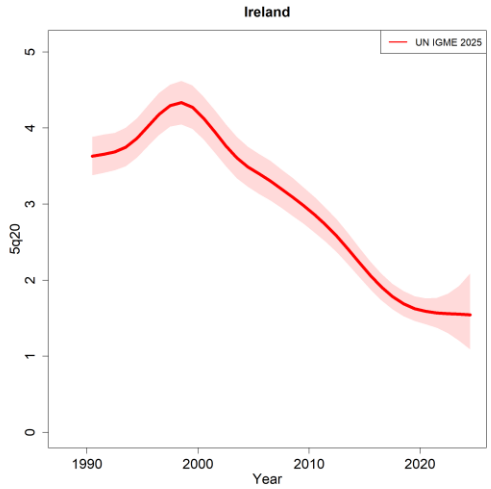
Ireland
UN IGME 2025
5q20
Year

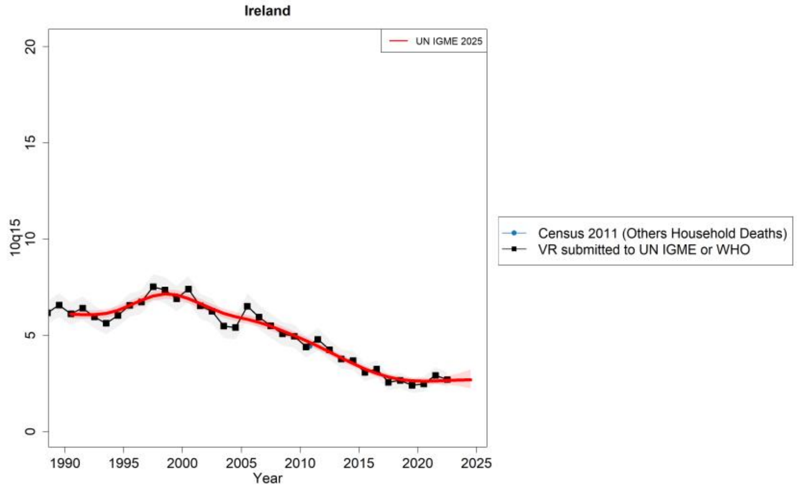
Ireland
UN IGME 2025
10q15
Year
Census 2011 (Others Household Deaths)
VR submitted to UN IGME or WHO

Israel (ISR)

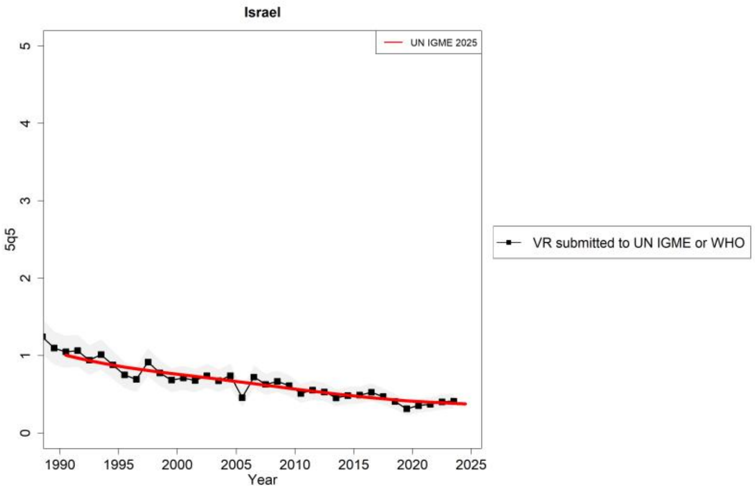
Israel
UN IGME 2025
VR submitted to UN IGME or WHO
5q5
Year
0
1
2
3
4
5
1990
1995
2000
2005
2010
2015
2020
2025

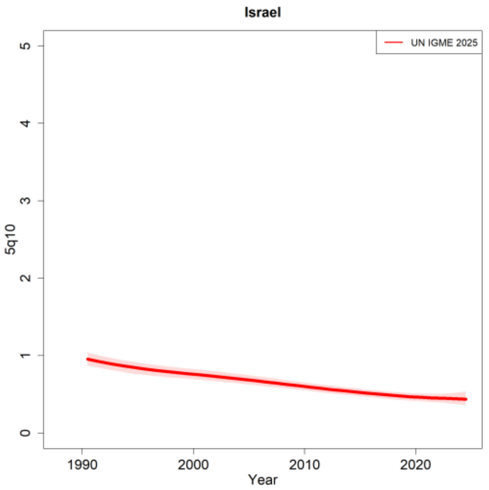
Israel
UN IGME 2025
5q10
Year
0
1
2
3
4
5
1990
2000
2010
2020

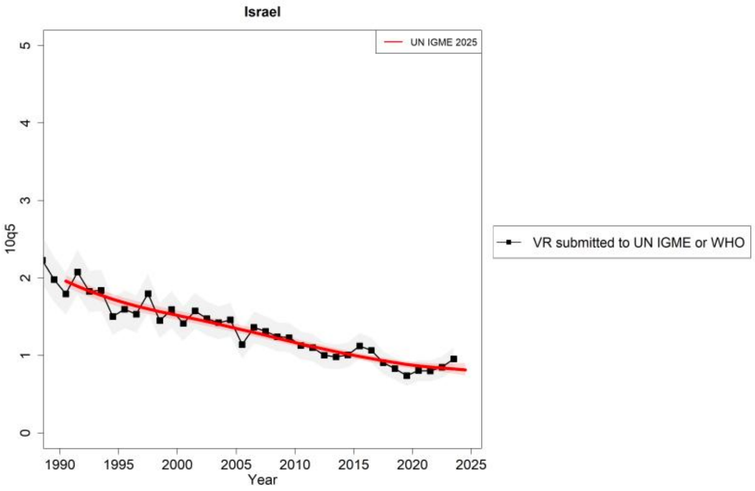
Israel
UN IGME 2025
VR submitted to UN IGME or WHO
10q5
Year
0
1
2
3
4
5
1990
1995
2000
2005
2010
2015
2020
2025

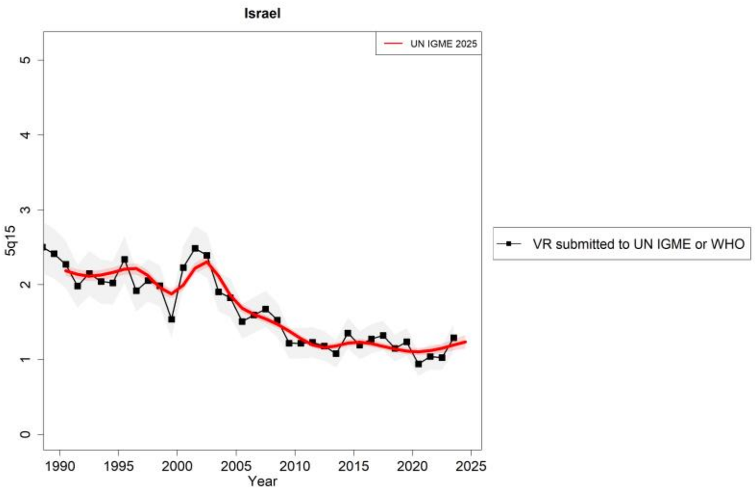
Israel
UN IGME 2025
VR submitted to UN IGME or WHO
5q15
Year
0
1
2
3
4
5
1990
1995
2000
2005
2010
2015
2020
2025

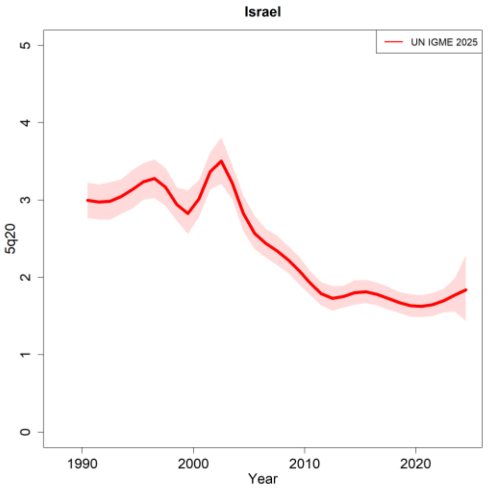
Israel
UN IGME 2025
5q20
Year
0
1
2
3
4
5
1990
2000
2010
2020

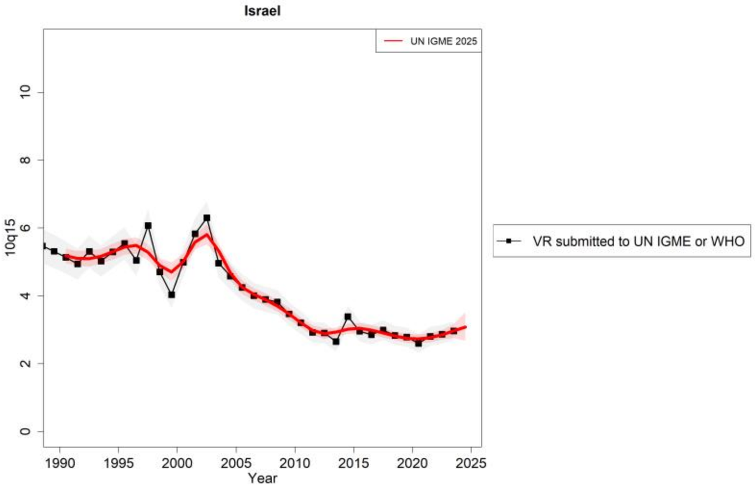
Israel
UN IGME 2025
VR submitted to UN IGME or WHO
10q15
Year
0
2
4
6
8
10
1990
1995
2000
2005
2010
2015
2020
2025

Italy (ITA)

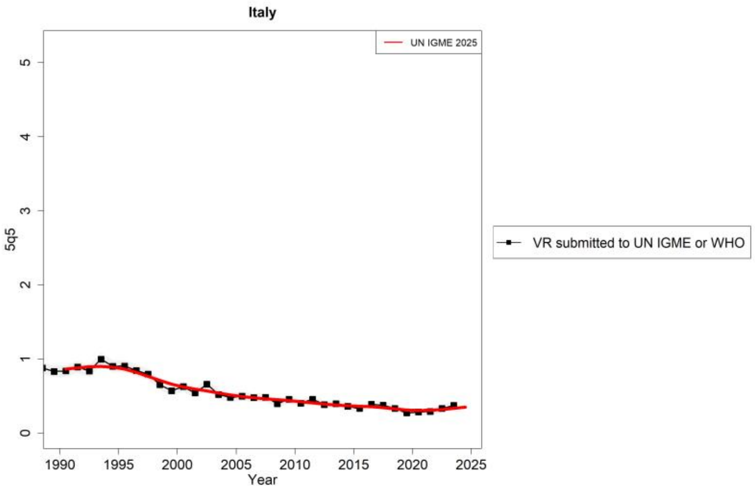
Italy
UN IGME 2025
VR submitted to UN IGME or WHO
5q5
0
1
2
3
4
5
1990
1995
2000
2005
2010
2015
2020
2025
Year

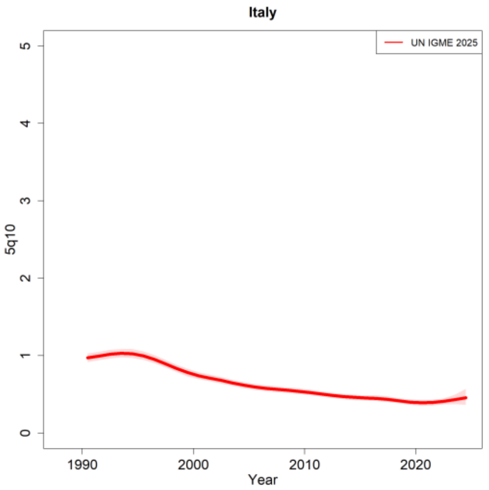
Italy
UN IGME 2025
5q10
0
1
2
3
4
5
1990
2000
2010
2020
Year

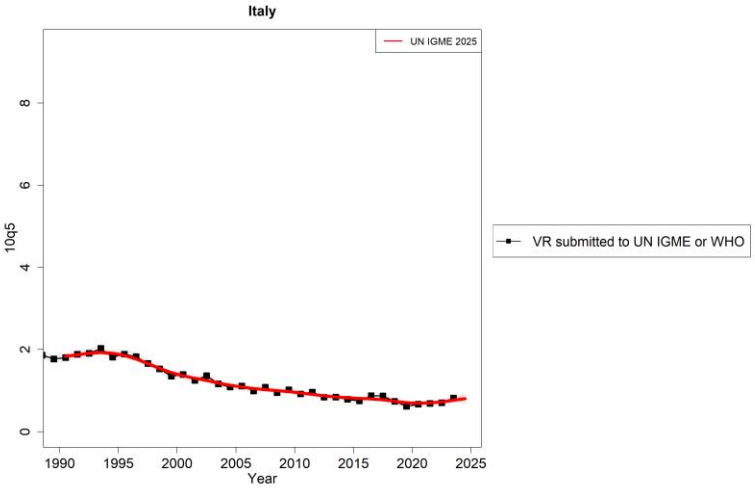
Italy
UN IGME 2025
VR submitted to UN IGME or WHO
10q5
0
2
4
6
8
1990
1995
2000
2005
2010
2015
2020
2025
Year

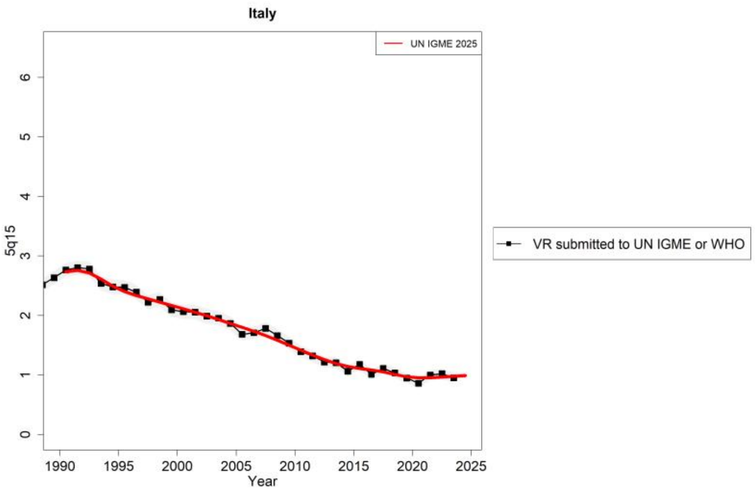
Italy
UN IGME 2025
VR submitted to UN IGME or WHO
5q15
Year

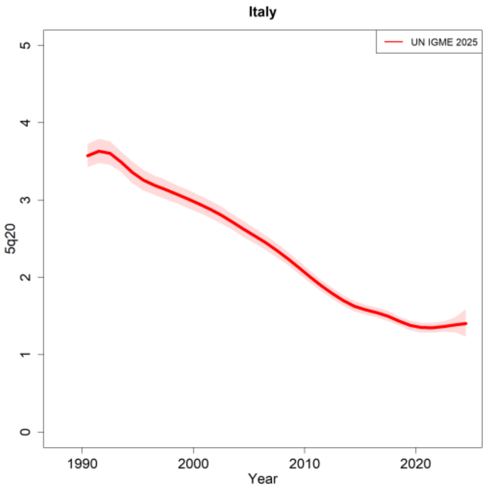
Italy
UN IGME 2025
5q20
Year

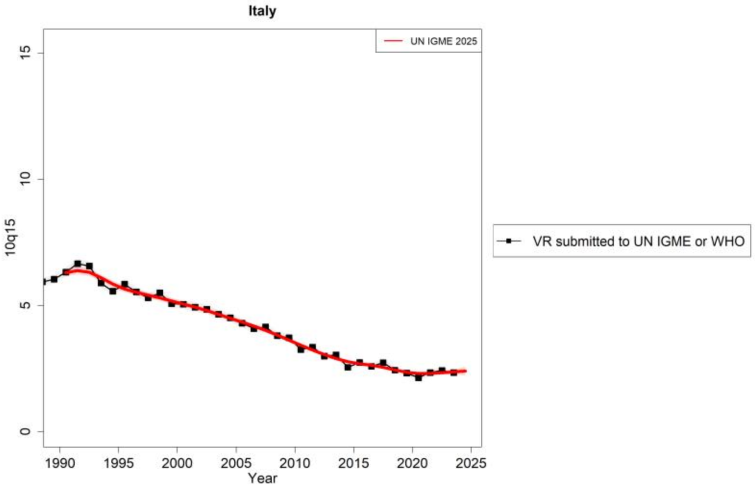
Italy
UN IGME 2025
VR submitted to UN IGME or WHO
10q15
Year

Jamaica (JAM)

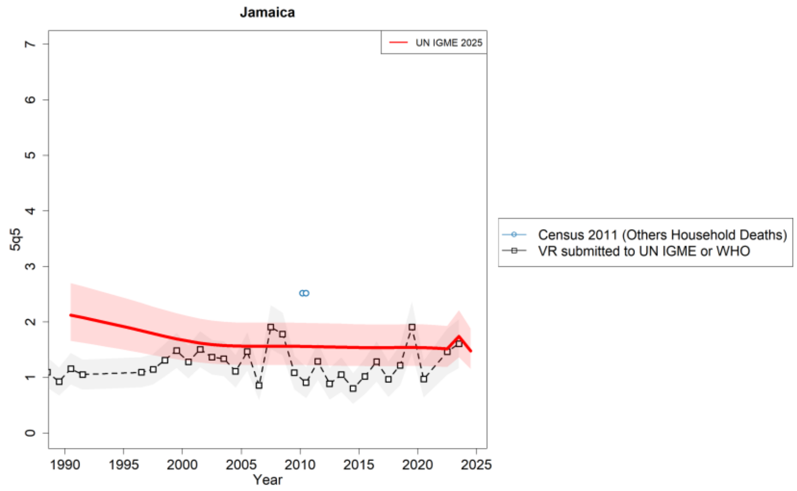
Jamaica
UN IGME 2025
Census 2011 (Others Household Deaths)
VR submitted to UN IGME or WHO
5q5
0
1
2
3
4
5
6
7
1990
1995
2000
2005
2010
2015
2020
2025
Year

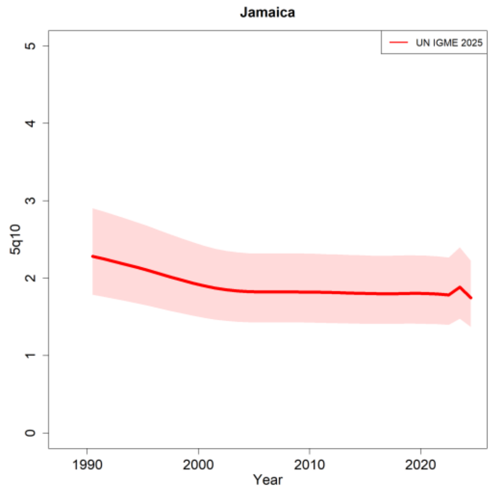
Jamaica
UN IGME 2025
5q10
0
1
2
3
4
5
1990
2000
2010
2020
Year

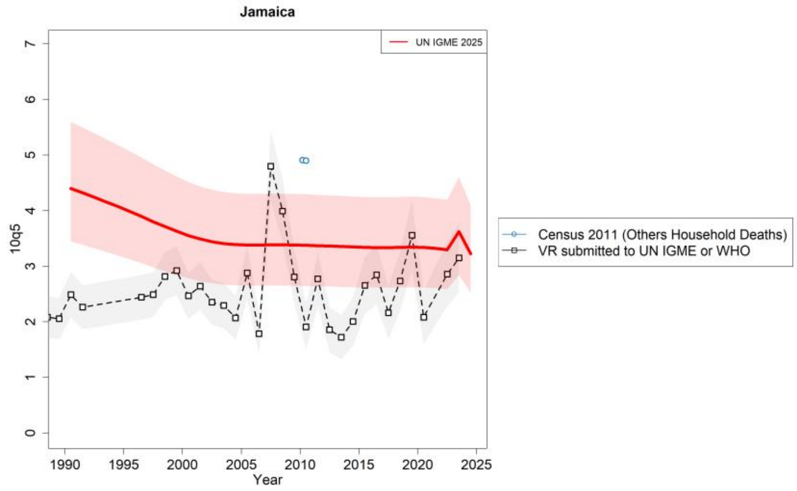
Jamaica
UN IGME 2025
Census 2011 (Others Household Deaths)
VR submitted to UN IGME or WHO
10q5
0
1
2
3
4
5
6
7
1990
1995
2000
2005
2010
2015
2020
2025
Year

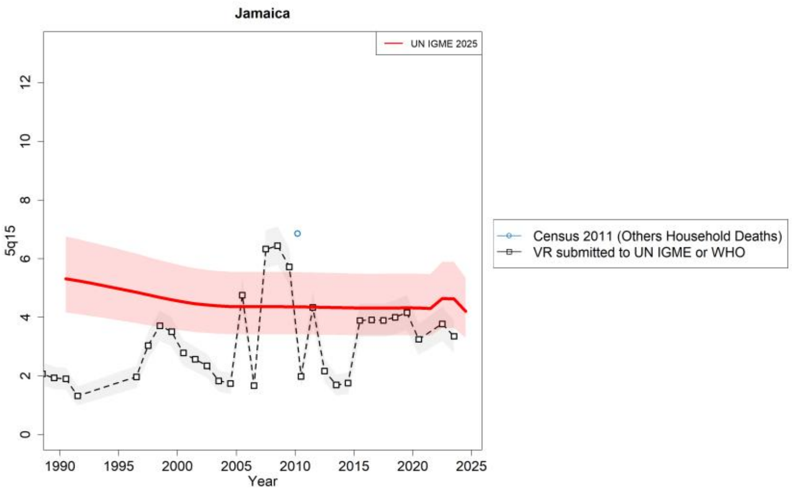
Jamaica
UN IGME 2025
Census 2011 (Others Household Deaths)
VR submitted to UN IGME or WHO
5q15
Year

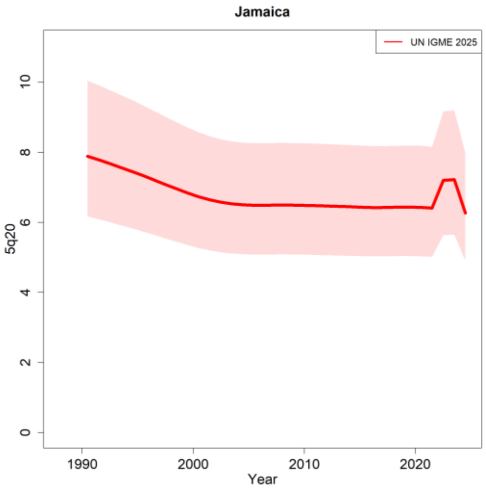
Jamaica
UN IGME 2025
5q20
Year

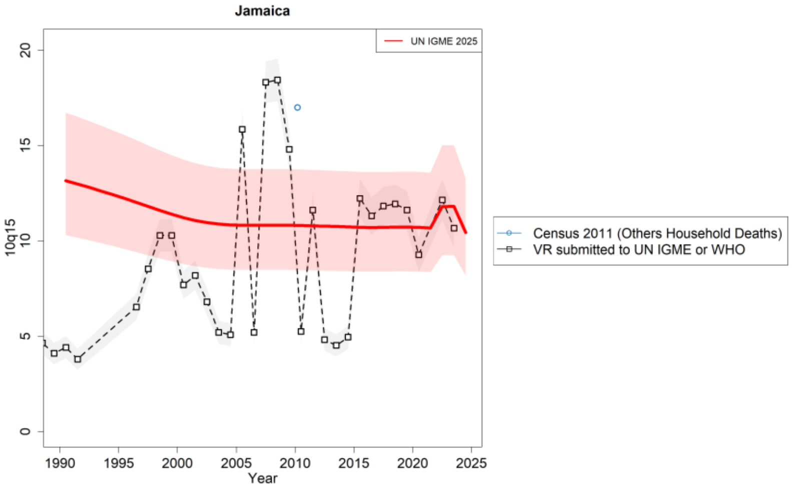
Jamaica
UN IGME 2025
Census 2011 (Others Household Deaths)
VR submitted to UN IGME or WHO
10q15
Year

Japan (JPN)

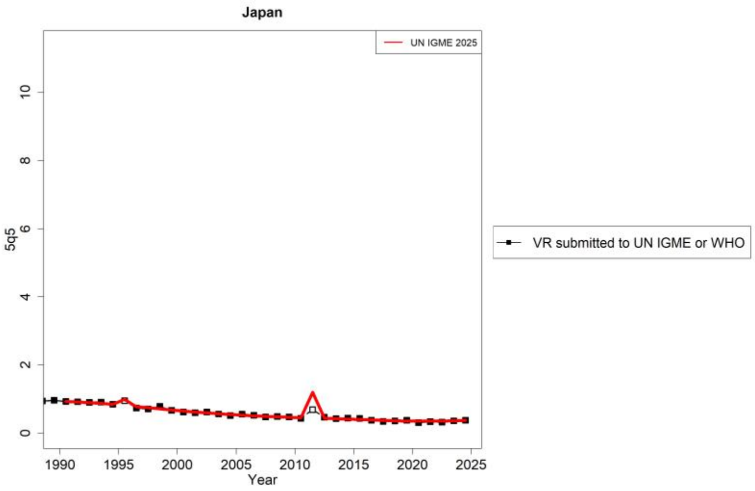
Japan
UN IGME 2025
VR submitted to UN IGME or WHO
5q5
0
2
4
6
8
10
1990
1995
2000
2005
2010
2015
2020
2025
Year

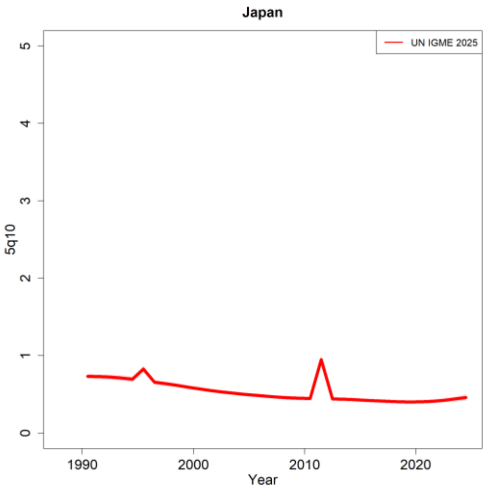
Japan
UN IGME 2025
5q10
0
1
2
3
4
5
1990
2000
2010
2020
Year

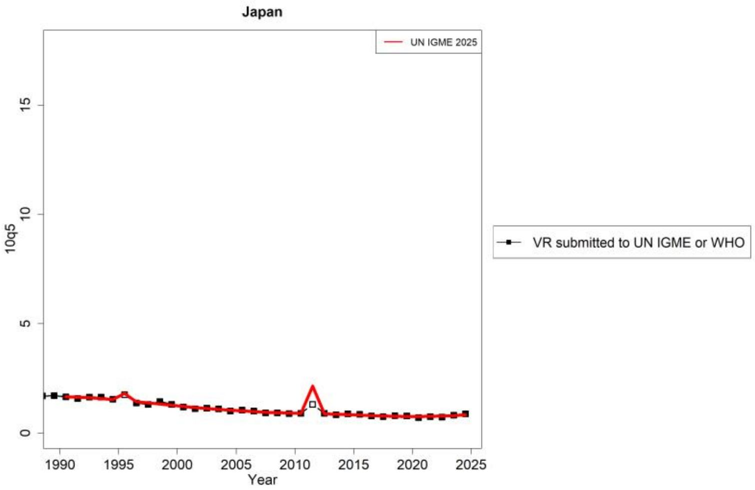
Japan
UN IGME 2025
VR submitted to UN IGME or WHO
10q5
0
5
10
15
1990
1995
2000
2005
2010
2015
2020
2025
Year

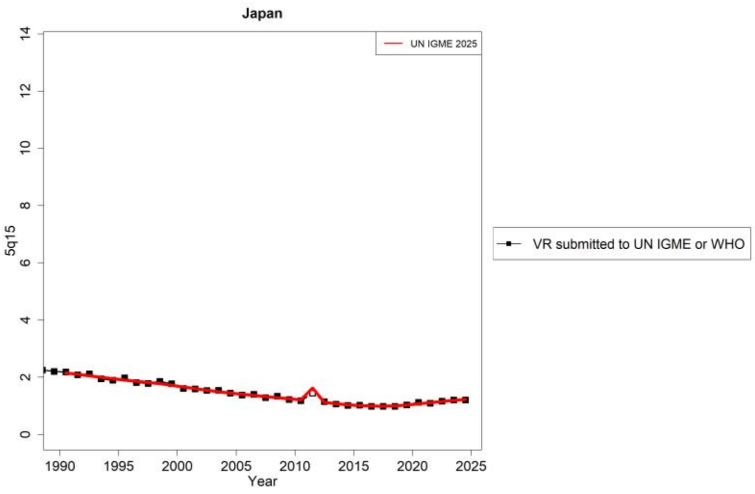
Japan
UN IGME 2025
VR submitted to UN IGME or WHO
5q15
Year

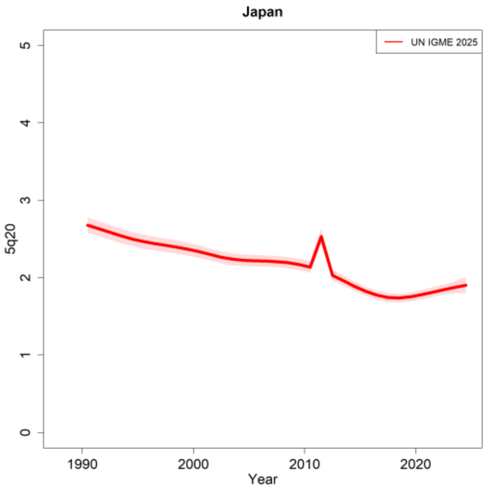
Japan
UN IGME 2025
5q20
Year

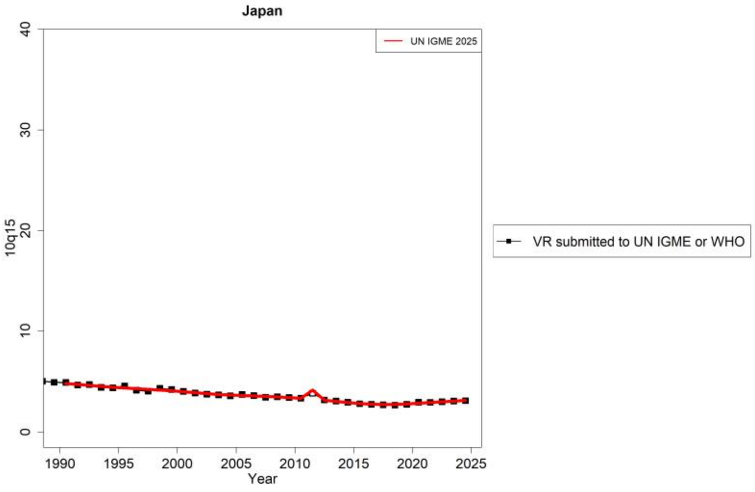
Japan
UN IGME 2025
VR submitted to UN IGME or WHO
10q15
Year

Jordan (JOR)

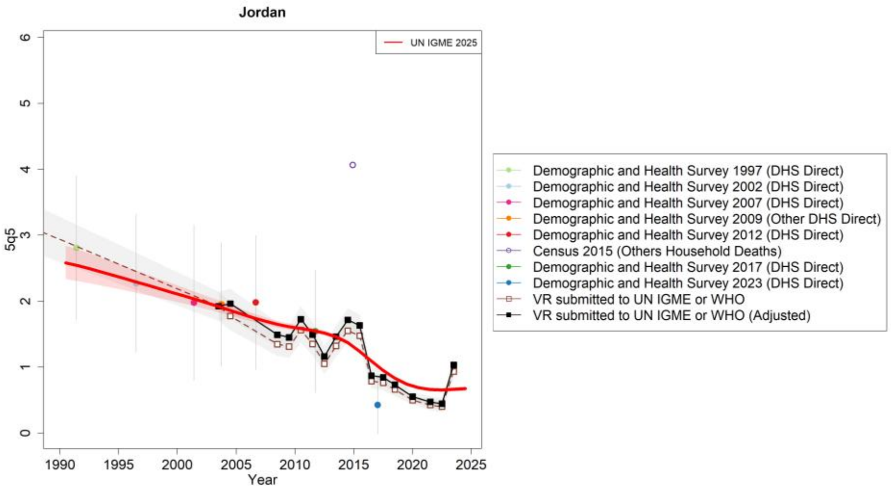
Jordan
UN IGME 2025
5q5
Year
Demographic and Health Survey 1997 (DHS Direct)
Demographic and Health Survey 2002 (DHS Direct)
Demographic and Health Survey 2007 (DHS Direct)
Demographic and Health Survey 2009 (Other DHS Direct)
Demographic and Health Survey 2012 (DHS Direct)
Census 2015 (Others Household Deaths)
Demographic and Health Survey 2017 (DHS Direct)
Demographic and Health Survey 2023 (DHS Direct)
VR submitted to UN IGME or WHO
VR submitted to UN IGME or WHO (Adjusted)

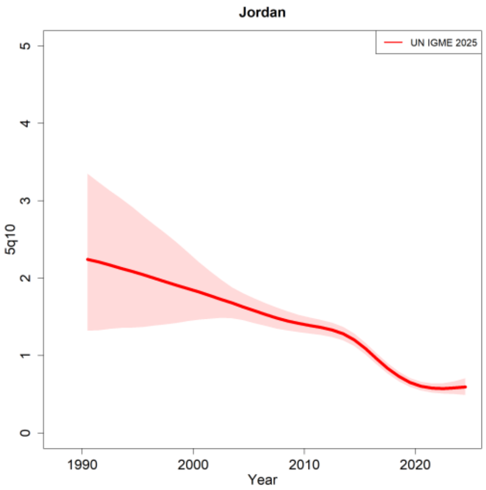
Jordan
UN IGME 2025
5q10
Year

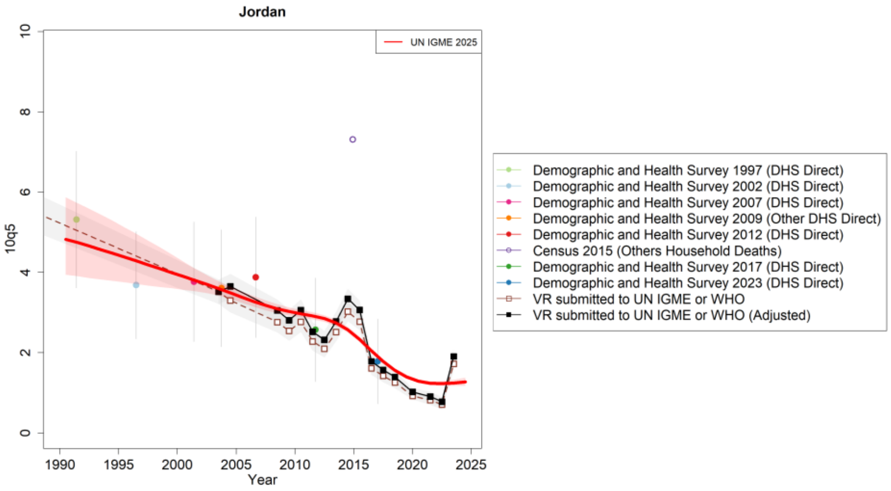
Jordan
UN IGME 2025
10q5
Year
Demographic and Health Survey 1997 (DHS Direct)
Demographic and Health Survey 2002 (DHS Direct)
Demographic and Health Survey 2007 (DHS Direct)
Demographic and Health Survey 2009 (Other DHS Direct)
Demographic and Health Survey 2012 (DHS Direct)
Census 2015 (Others Household Deaths)
Demographic and Health Survey 2017 (DHS Direct)
Demographic and Health Survey 2023 (DHS Direct)
VR submitted to UN IGME or WHO
VR submitted to UN IGME or WHO (Adjusted)

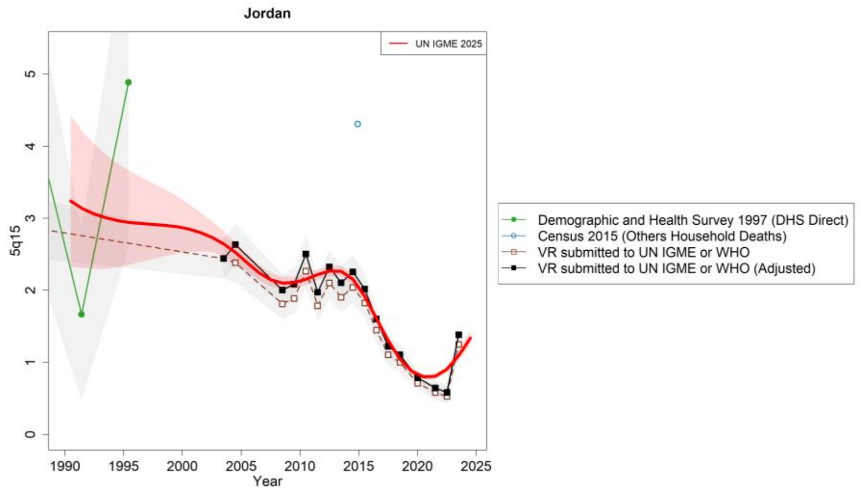
Jordan
UN IGME 2025
Demographic and Health Survey 1997 (DHS Direct)
Census 2015 (Others Household Deaths)
VR submitted to UN IGME or WHO
VR submitted to UN IGME or WHO (Adjusted)
5q15
Year

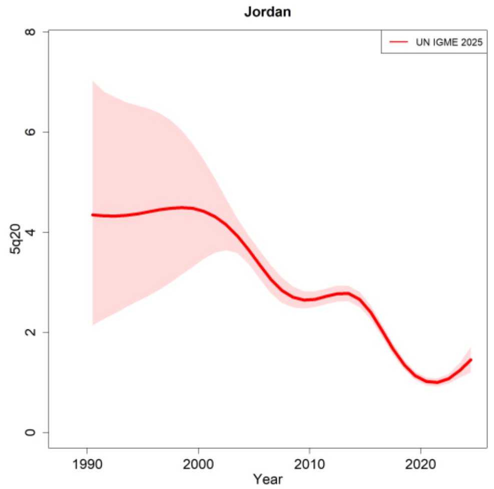
Jordan
UN IGME 2025
5q20
Year

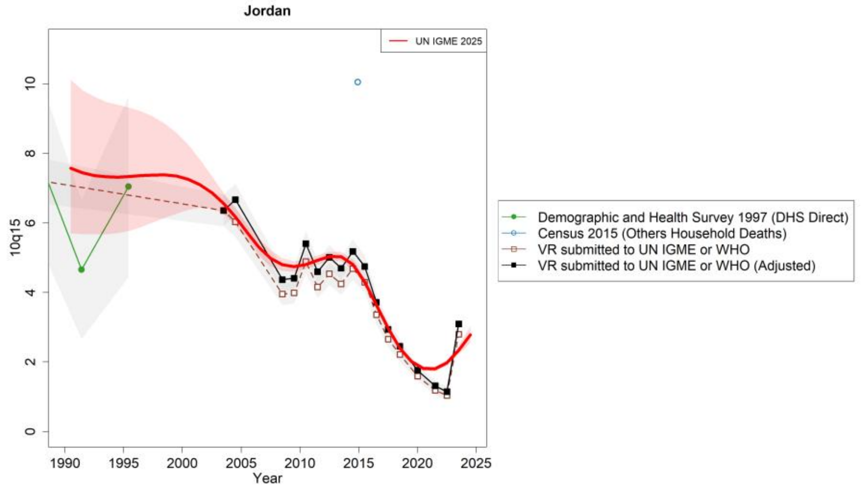
Jordan
UN IGME 2025
Demographic and Health Survey 1997 (DHS Direct)
Census 2015 (Others Household Deaths)
VR submitted to UN IGME or WHO
VR submitted to UN IGME or WHO (Adjusted)
10q15
Year

Kazakhstan (KAZ)

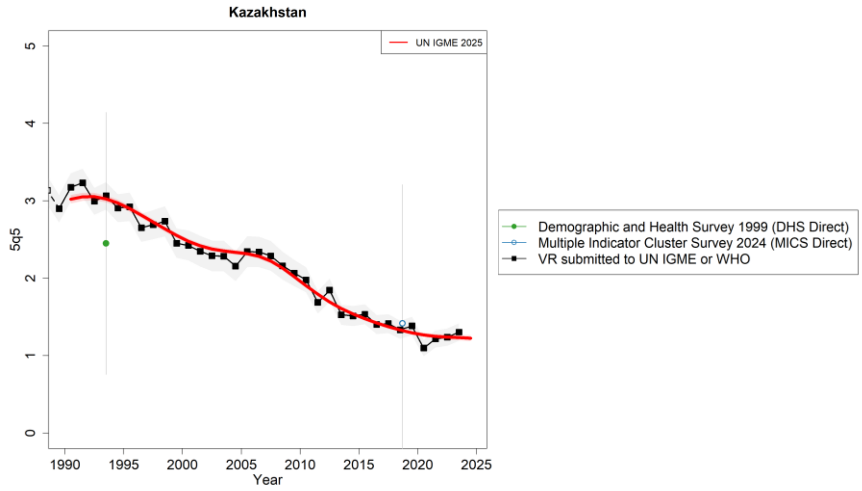

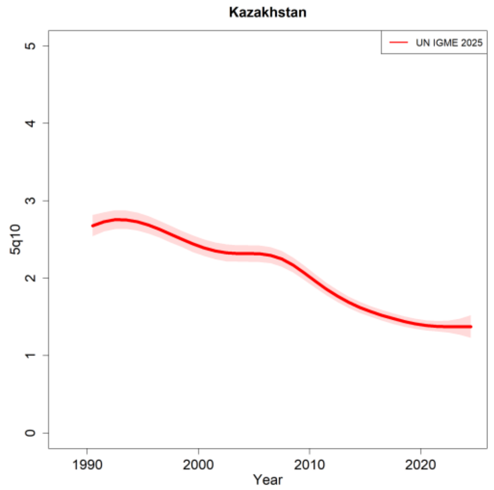

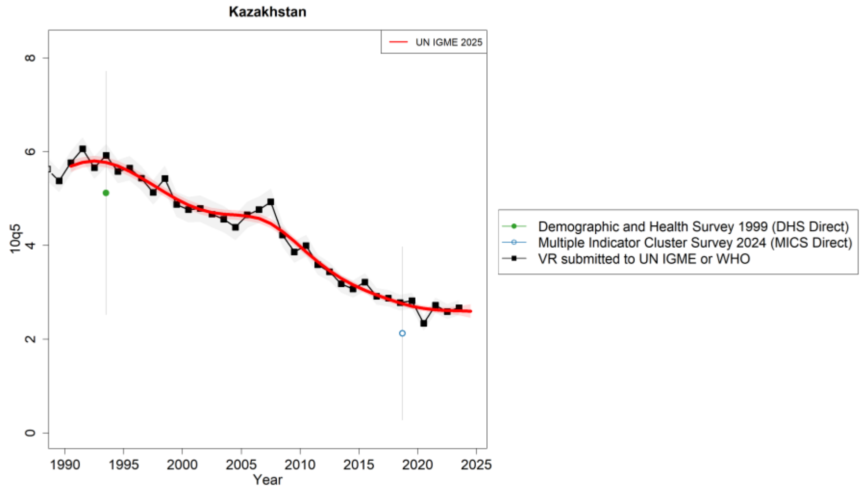

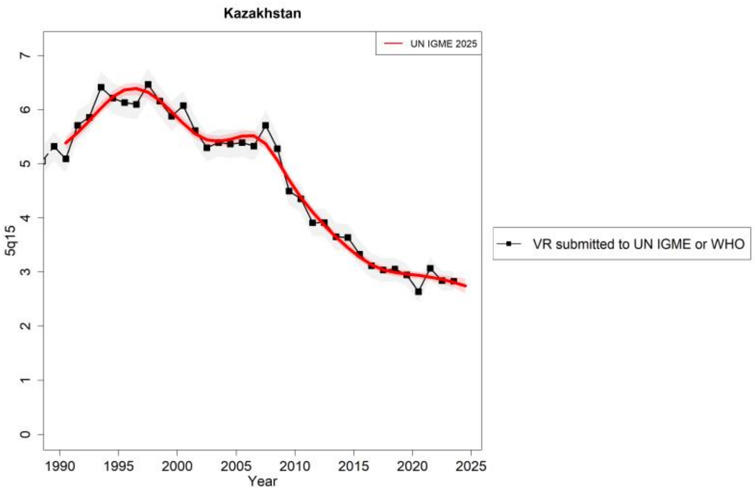
Kazakhstan
UN IGME 2025
VR submitted to UN IGME or WHO
5q15
Year

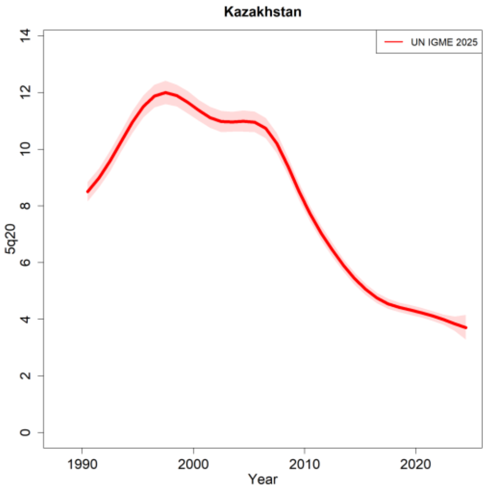
Kazakhstan
UN IGME 2025
5q20
Year

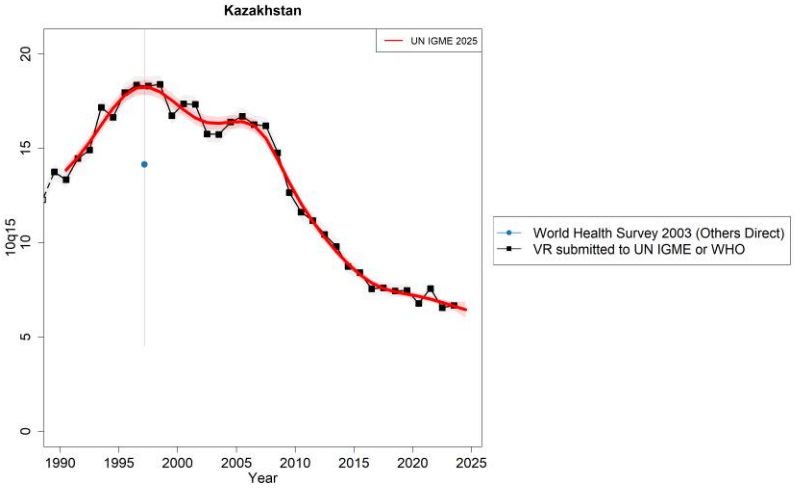
Kazakhstan
UN IGME 2025
World Health Survey 2003 (Others Direct)
VR submitted to UN IGME or WHO
10q15
Year

Kenya (KEN)

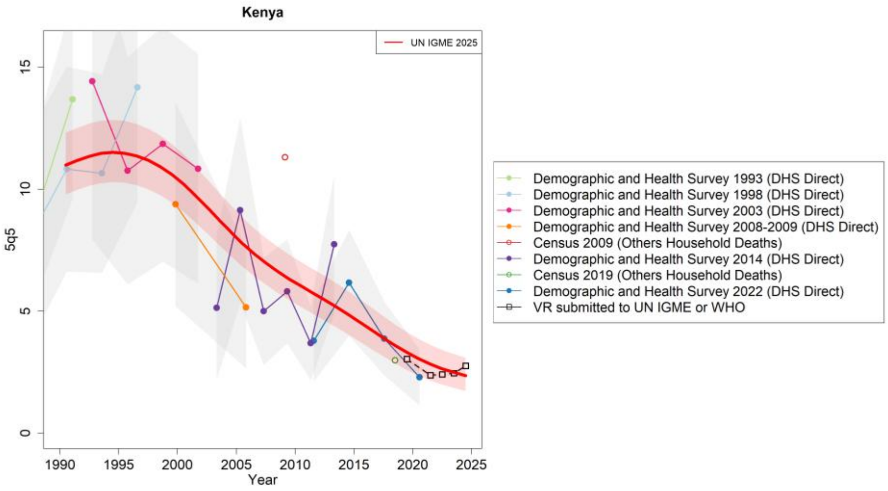
Kenya
UN IGME 2025
5q5
Year
Demographic and Health Survey 1993 (DHS Direct)
Demographic and Health Survey 1998 (DHS Direct)
Demographic and Health Survey 2003 (DHS Direct)
Demographic and Health Survey 2008-2009 (DHS Direct)
Census 2009 (Others Household Deaths)
Demographic and Health Survey 2014 (DHS Direct)
Census 2019 (Others Household Deaths)
Demographic and Health Survey 2022 (DHS Direct)
VR submitted to UN IGME or WHO

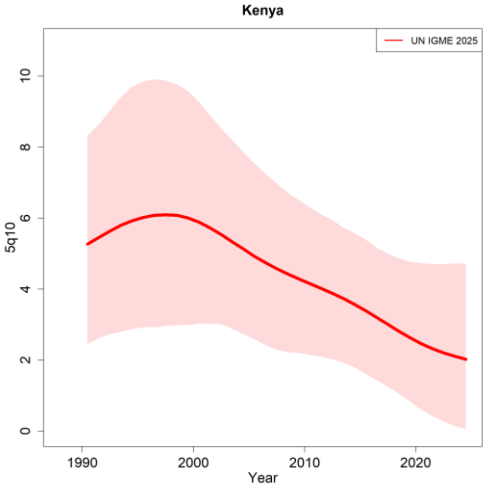
Kenya
UN IGME 2025
5q10
Year

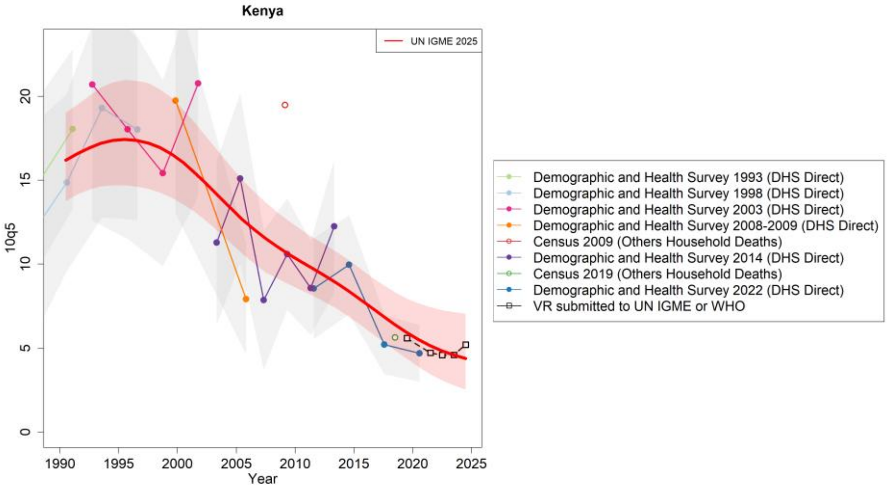
Kenya
UN IGME 2025
10q5
Year
Demographic and Health Survey 1993 (DHS Direct)
Demographic and Health Survey 1998 (DHS Direct)
Demographic and Health Survey 2003 (DHS Direct)
Demographic and Health Survey 2008-2009 (DHS Direct)
Census 2009 (Others Household Deaths)
Demographic and Health Survey 2014 (DHS Direct)
Census 2019 (Others Household Deaths)
Demographic and Health Survey 2022 (DHS Direct)
VR submitted to UN IGME or WHO

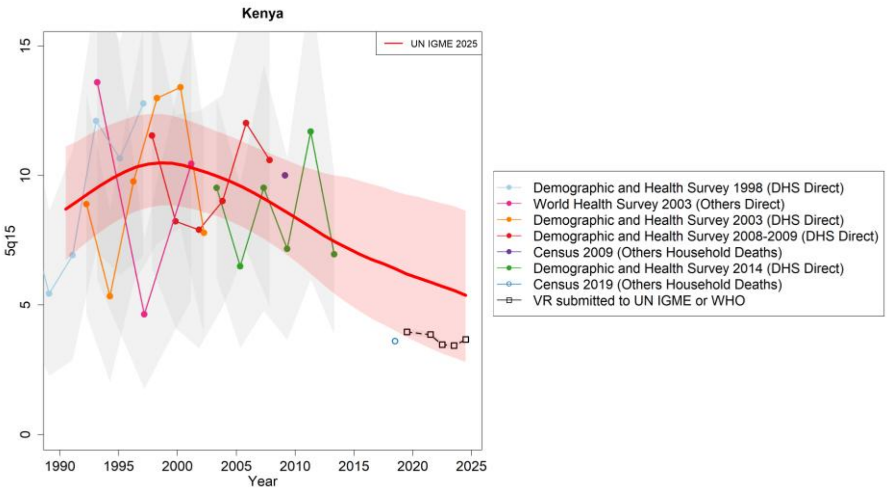
Kenya
UN IGME 2025
5q15
Year
Demographic and Health Survey 1998 (DHS Direct)
World Health Survey 2003 (Others Direct)
Demographic and Health Survey 2003 (DHS Direct)
Demographic and Health Survey 2008-2009 (DHS Direct)
Census 2009 (Others Household Deaths)
Demographic and Health Survey 2014 (DHS Direct)
Census 2019 (Others Household Deaths)
VR submitted to UN IGME or WHO

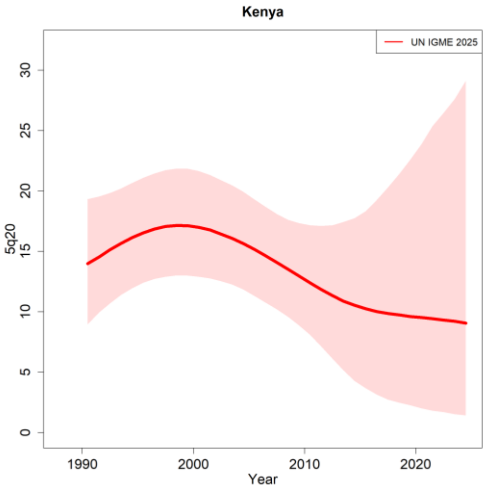
Kenya
UN IGME 2025
5q20
Year

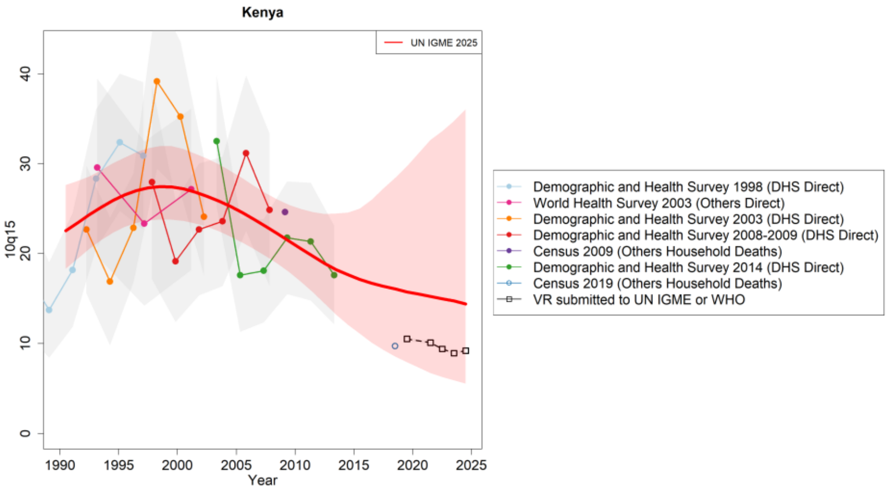
Kenya
UN IGME 2025
10q15
Year
Demographic and Health Survey 1998 (DHS Direct)
World Health Survey 2003 (Others Direct)
Demographic and Health Survey 2003 (DHS Direct)
Demographic and Health Survey 2008-2009 (DHS Direct)
Census 2009 (Others Household Deaths)
Demographic and Health Survey 2014 (DHS Direct)
Census 2019 (Others Household Deaths)
VR submitted to UN IGME or WHO

Kiribati (KIR)

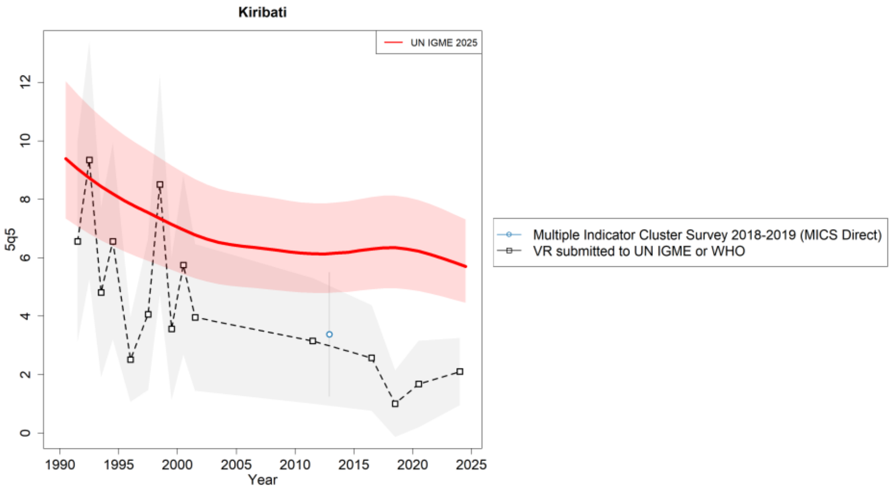

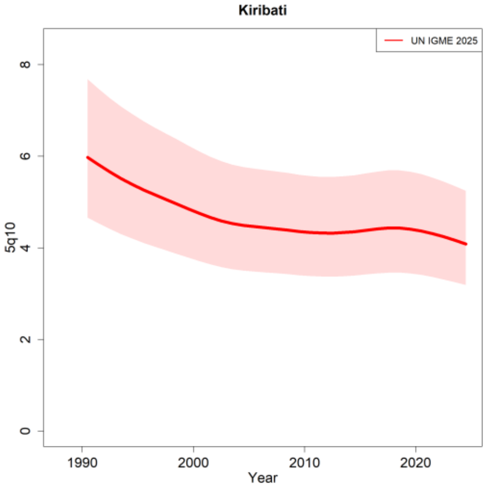

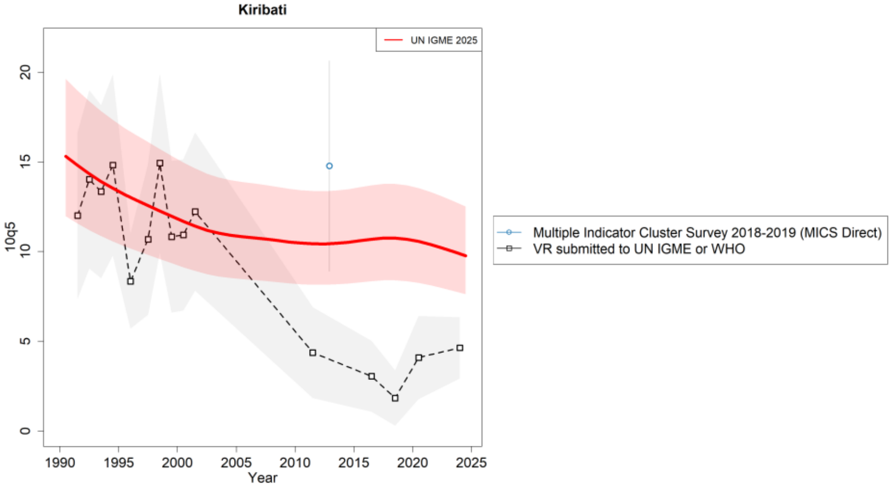

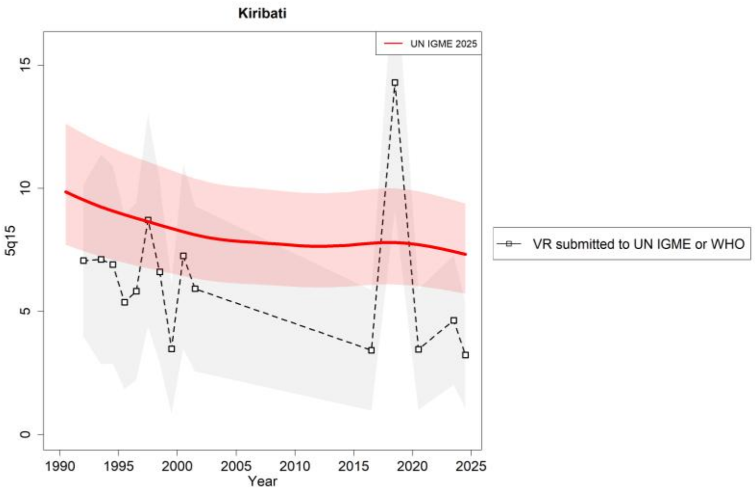
Kiribati
UN IGME 2025
VR submitted to UN IGME or WHO
5q15
Year

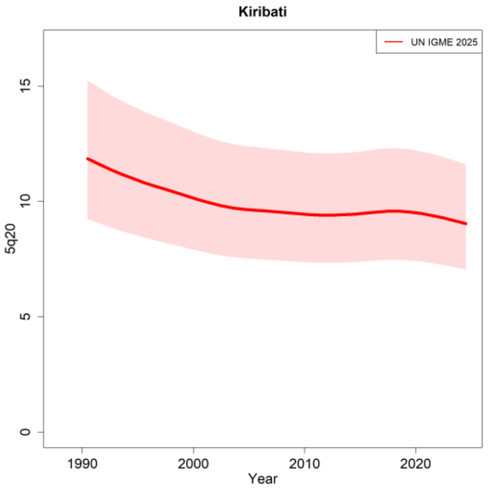
Kiribati
UN IGME 2025
5q20
Year

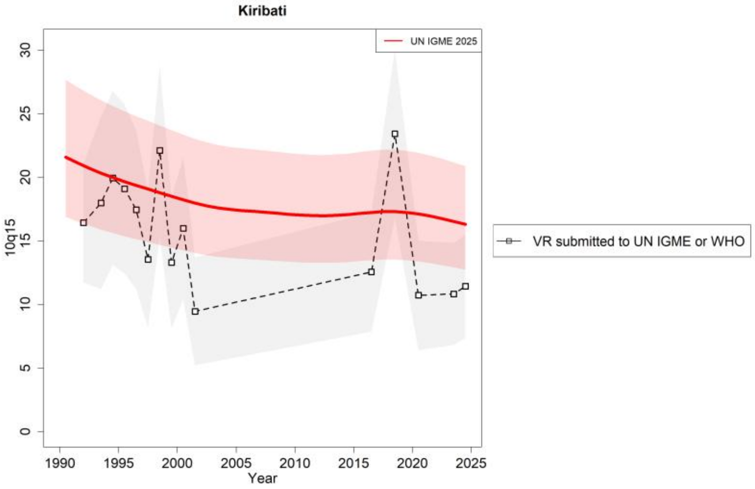
Kiribati
UN IGME 2025
VR submitted to UN IGME or WHO
10q15
Year

Kosovo (UNSCR 1244) (RKS)

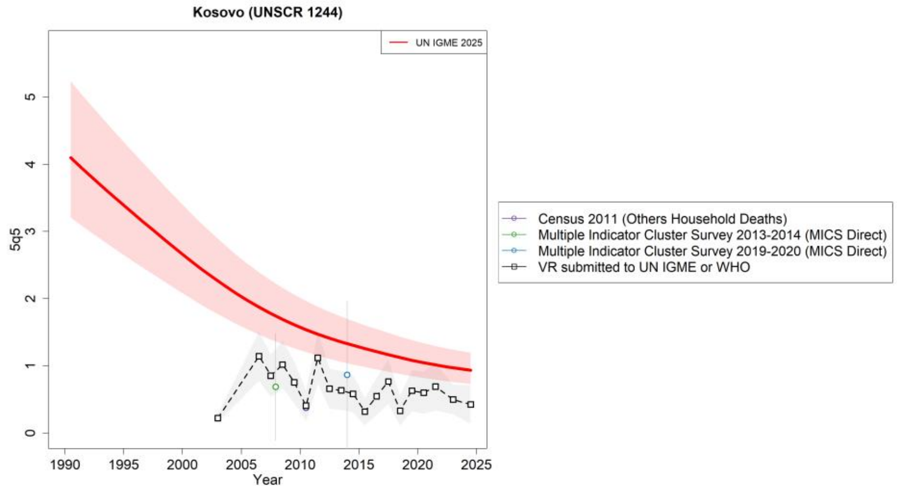

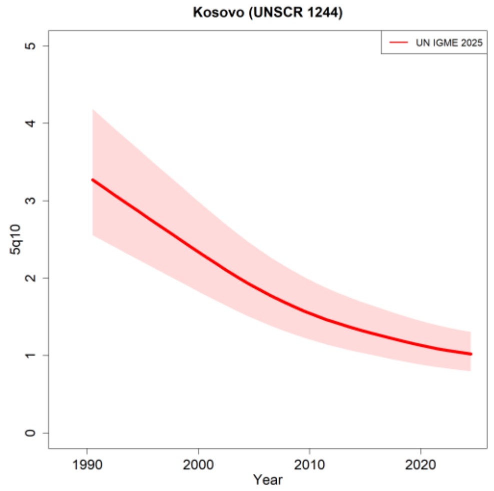

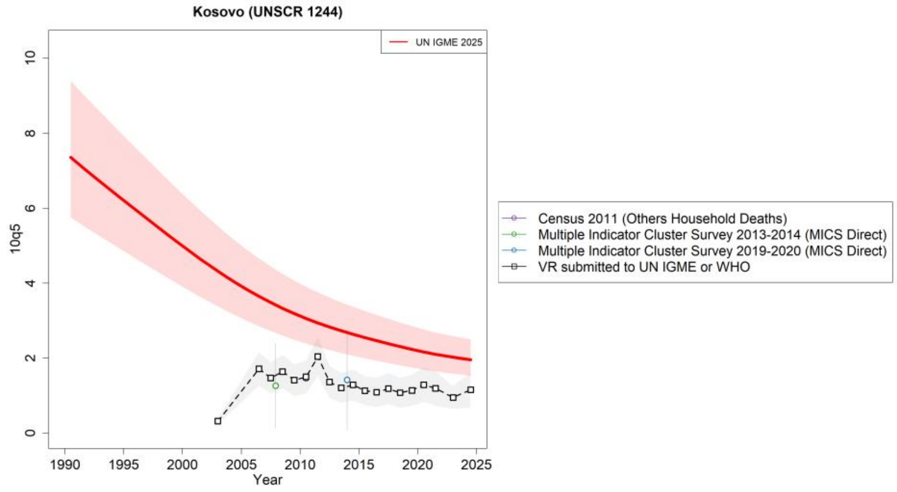

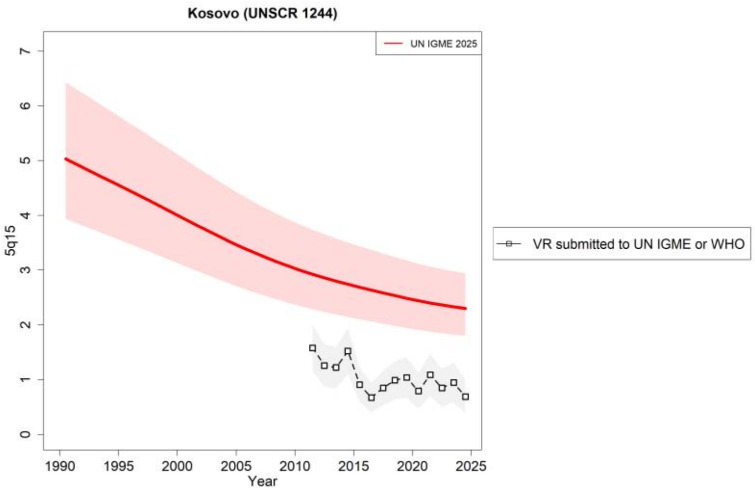
Kosovo (UNSCR 1244)
UN IGME 2025
VR submitted to UN IGME or WHO
5q15
Year

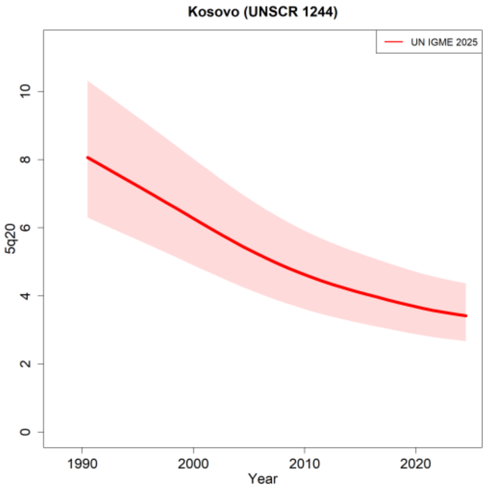
Kosovo (UNSCR 1244)
UN IGME 2025
5q20
Year

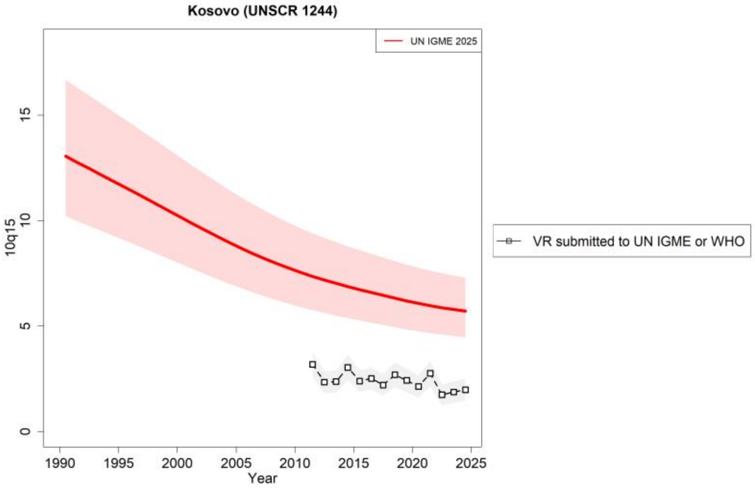
Kosovo (UNSCR 1244)
UN IGME 2025
VR submitted to UN IGME or WHO
10q15
Year

Kuwait (KWT)

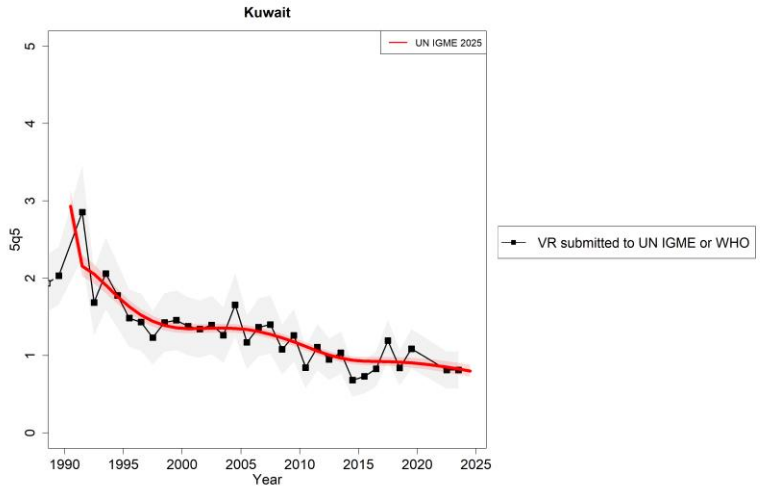
Kuwait
UN IGME 2025
VR submitted to UN IGME or WHO
5q5
Year
0
1
2
3
4
5
1990
1995
2000
2005
2010
2015
2020
2025

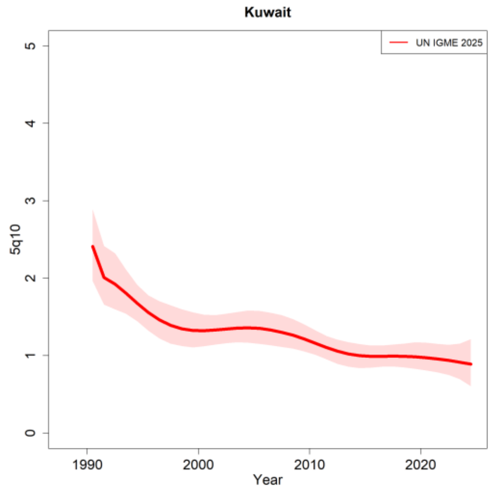
Kuwait
UN IGME 2025
5q10
Year
0
1
2
3
4
5
1990
2000
2010
2020

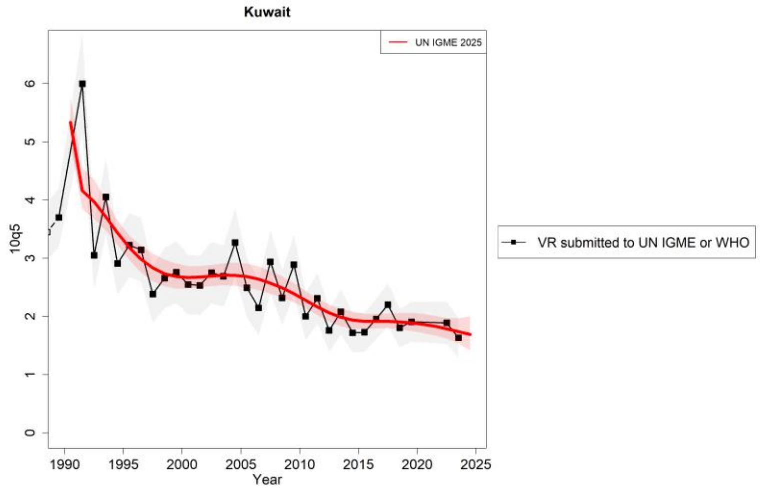
Kuwait
UN IGME 2025
VR submitted to UN IGME or WHO
10q5
Year
0
1
2
3
4
5
6
1990
1995
2000
2005
2010
2015
2020
2025

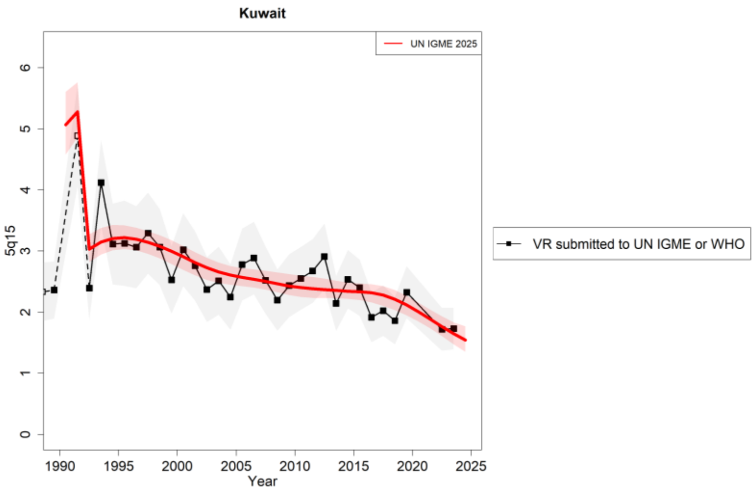
Kuwait
UN IGME 2025
VR submitted to UN IGME or WHO
5q15
Year

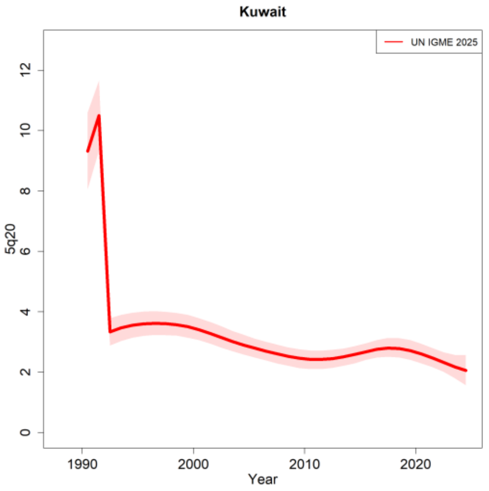
Kuwait
UN IGME 2025
5q20
Year

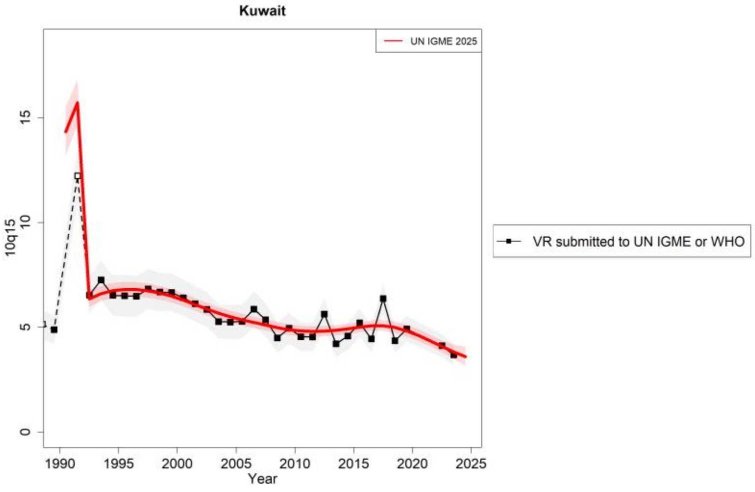
Kuwait
UN IGME 2025
VR submitted to UN IGME or WHO
10q15
Year

Kyrgyzstan (KGZ)

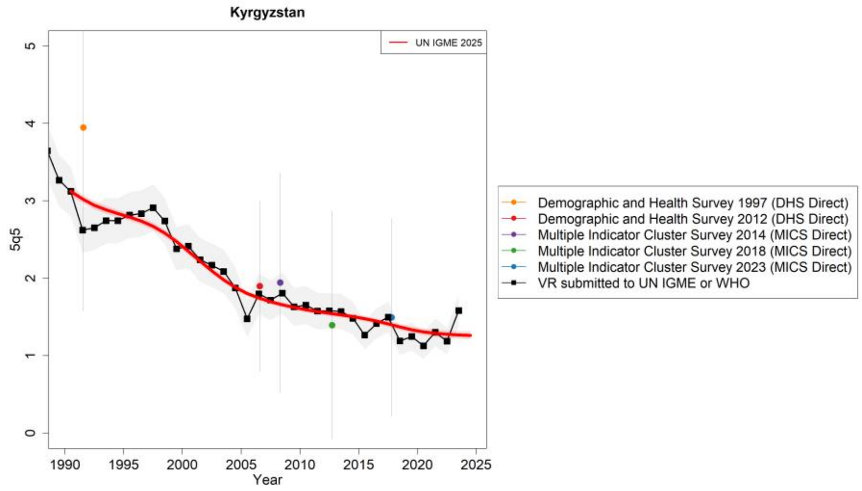

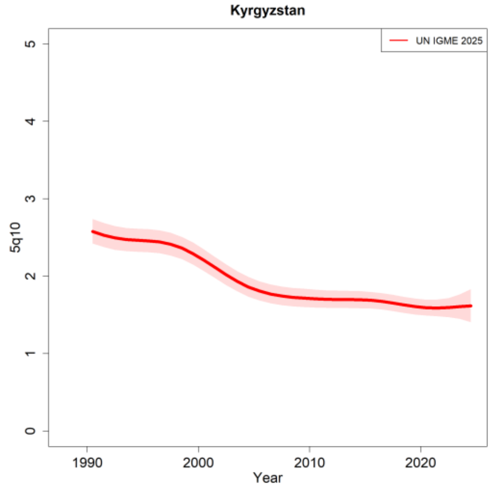

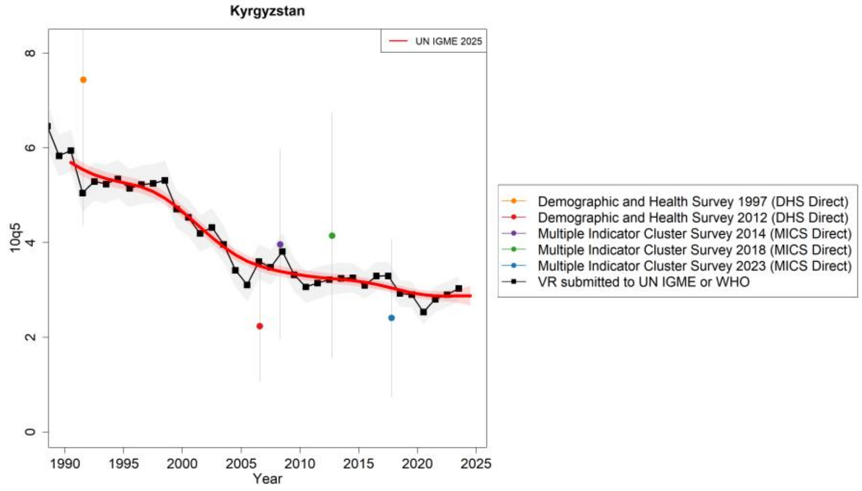

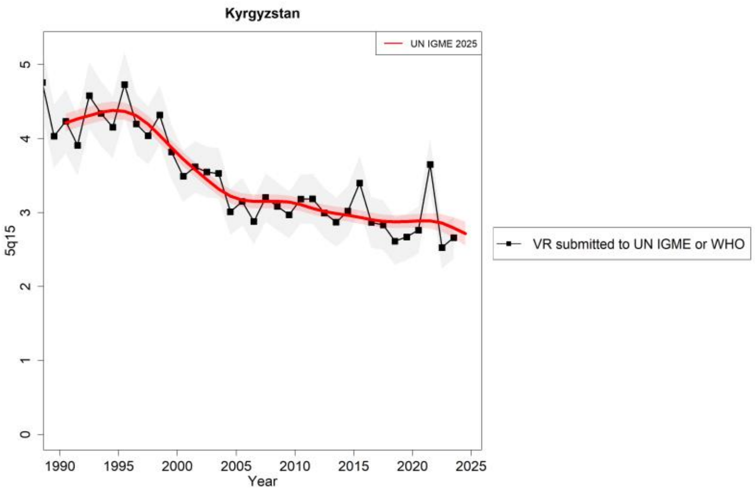
Kyrgyzstan
UN IGME 2025
VR submitted to UN IGME or WHO
5q15
Year
0
1
2
3
4
5
1990
1995
2000
2005
2010
2015
2020
2025

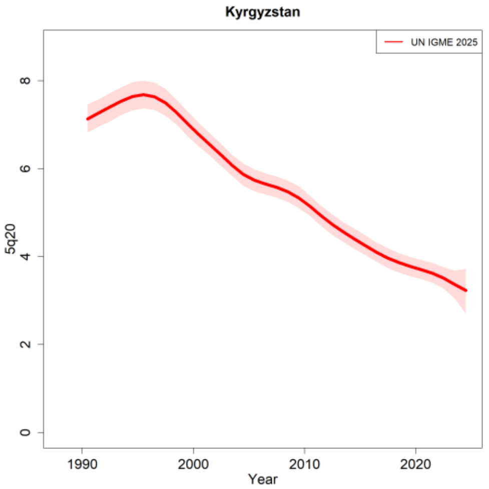
Kyrgyzstan
UN IGME 2025
5q20
Year
0
2
4
6
8
1990
2000
2010
2020

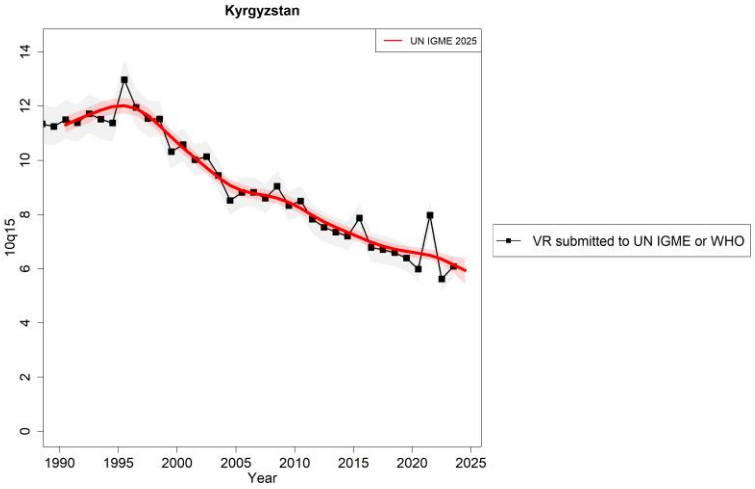
Kyrgyzstan
UN IGME 2025
VR submitted to UN IGME or WHO
10q15
Year
0
2
4
6
8
10
12
14
1990
1995
2000
2005
2010
2015
2020
2025

Lao People's Democratic Republic (LAO)

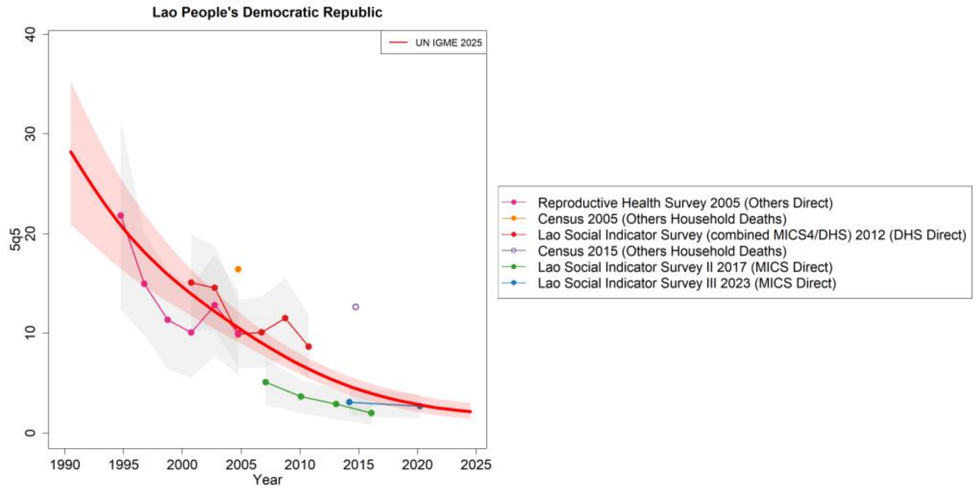

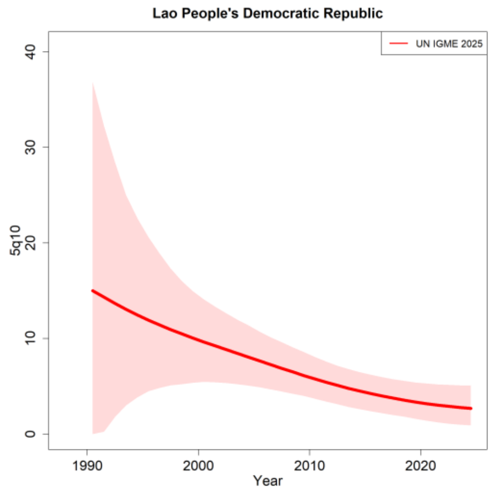

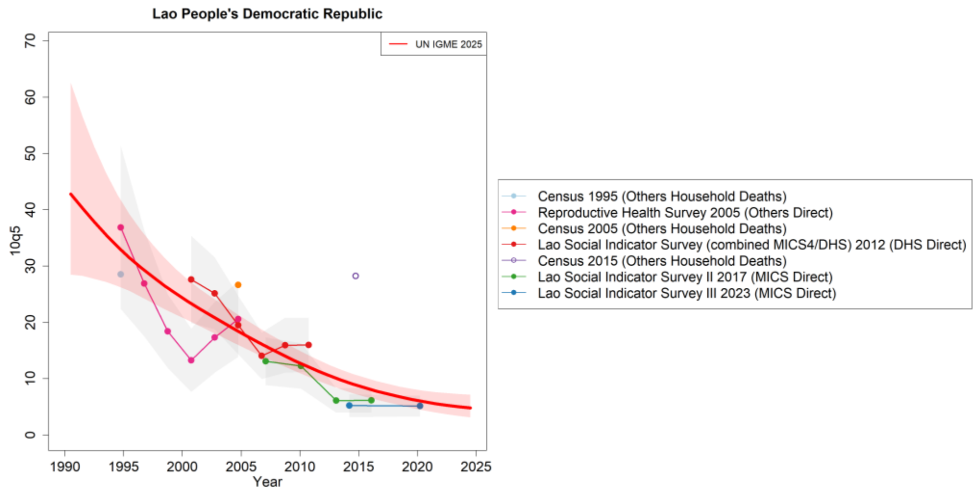

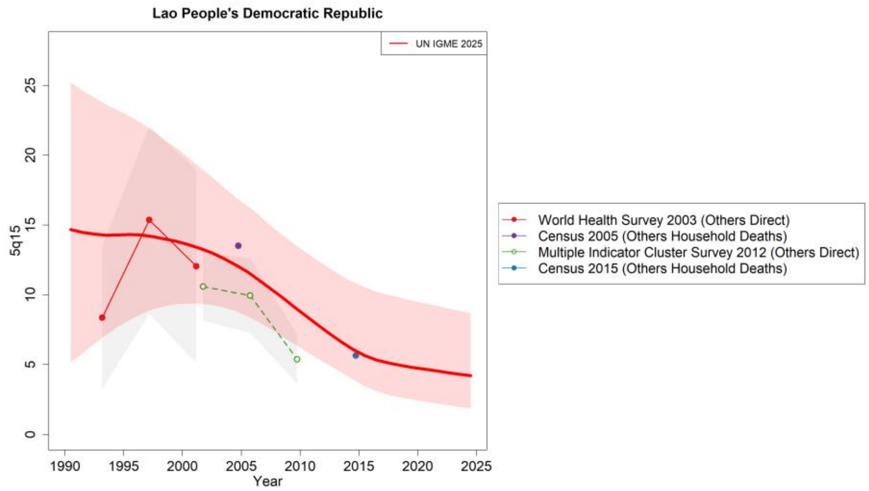
Lao People's Democratic Republic
UN IGME 2025
5q15
Year
World Health Survey 2003 (Others Direct)
Census 2005 (Others Household Deaths)
Multiple Indicator Cluster Survey 2012 (Others Direct)
Census 2015 (Others Household Deaths)

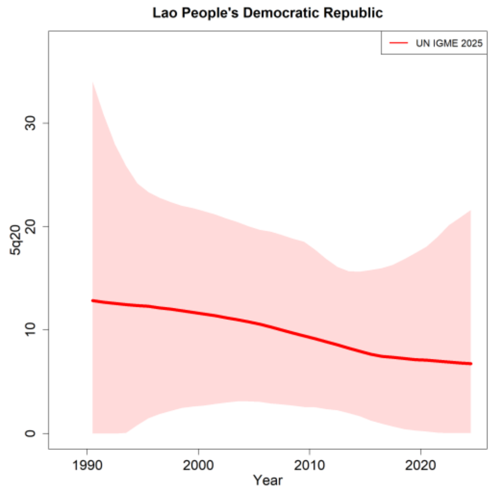
Lao People's Democratic Republic
UN IGME 2025
5q20
Year

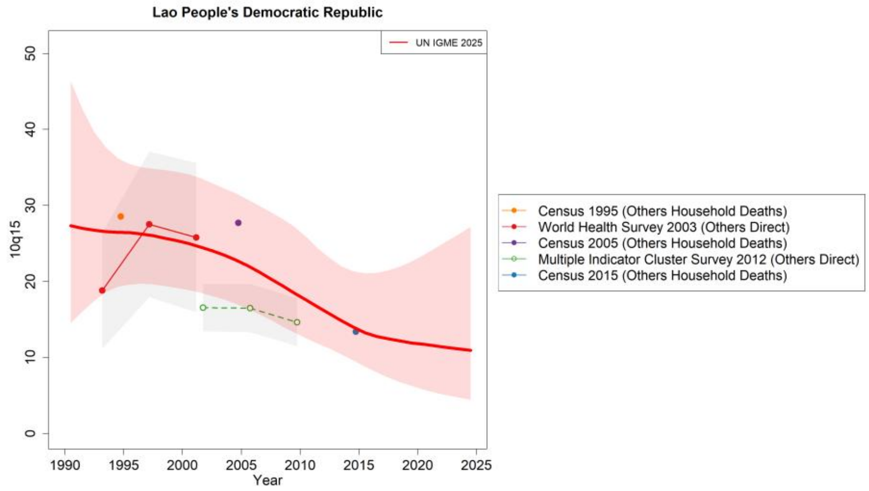
Lao People's Democratic Republic
UN IGME 2025
10q15
Year
Census 1995 (Others Household Deaths)
World Health Survey 2003 (Others Direct)
Census 2005 (Others Household Deaths)
Multiple Indicator Cluster Survey 2012 (Others Direct)
Census 2015 (Others Household Deaths)

Latvia (LVA)

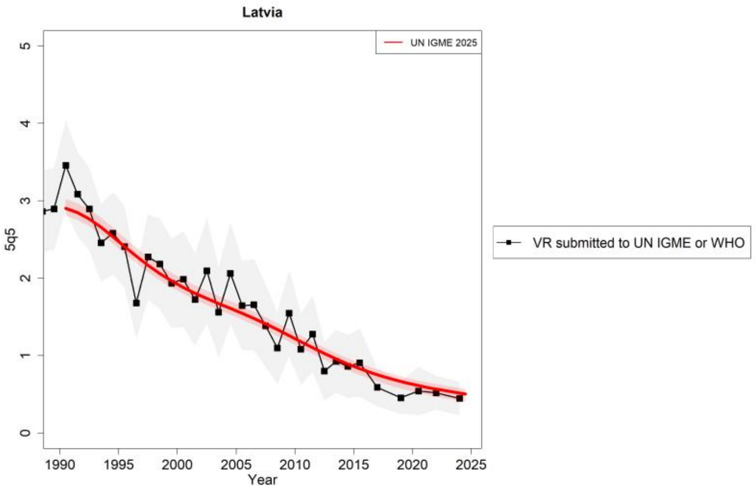
Latvia
UN IGME 2025
VR submitted to UN IGME or WHO
5q5
Year

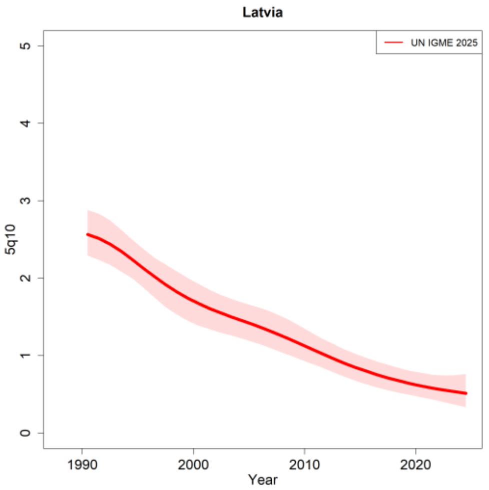
Latvia
UN IGME 2025
5q10
Year

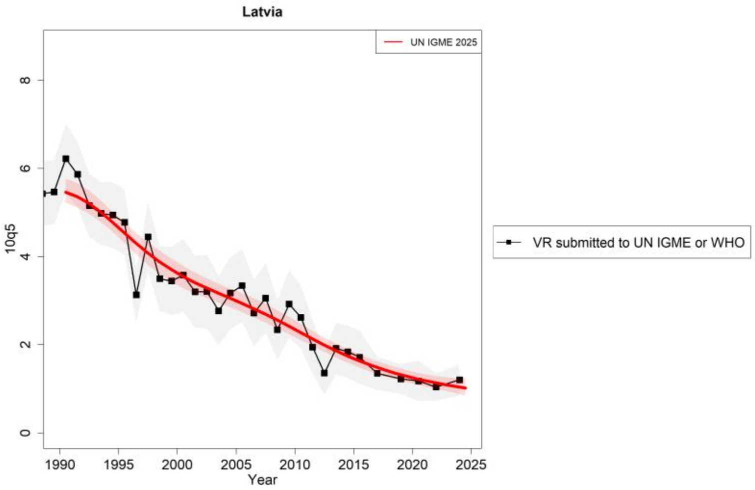
Latvia
UN IGME 2025
VR submitted to UN IGME or WHO
10q5
Year

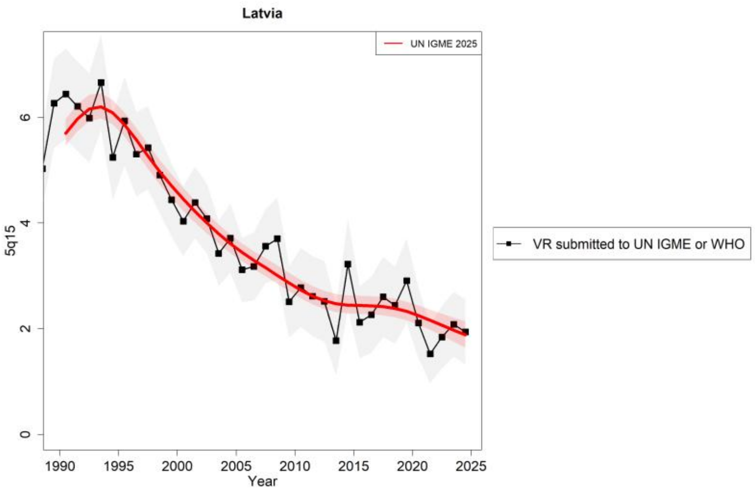
Latvia
UN IGME 2025
VR submitted to UN IGME or WHO
5q15
Year

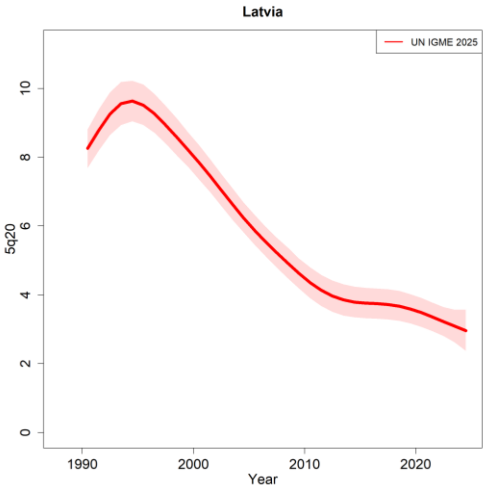
Latvia
UN IGME 2025
5q20
Year

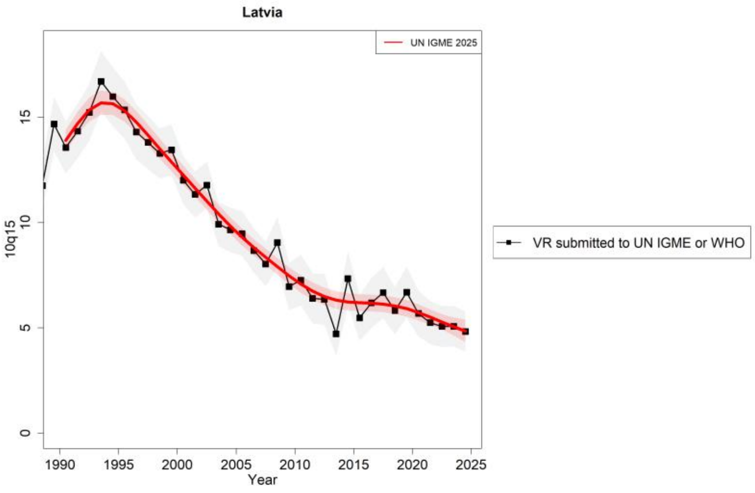
Latvia
UN IGME 2025
VR submitted to UN IGME or WHO
10q15
Year

Lebanon (LBN)

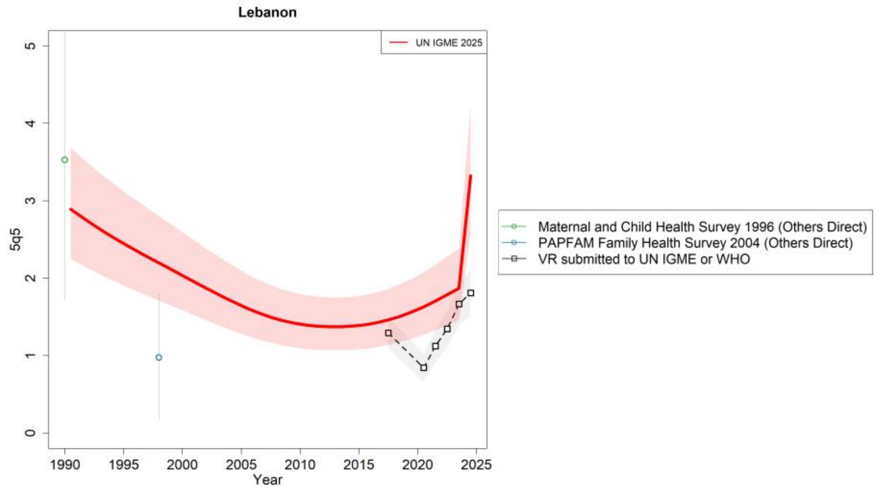
Lebanon
UN IGME 2025
5q5
Year
Maternal and Child Health Survey 1996 (Others Direct)
PAPFAM Family Health Survey 2004 (Others Direct)
VR submitted to UN IGME or WHO

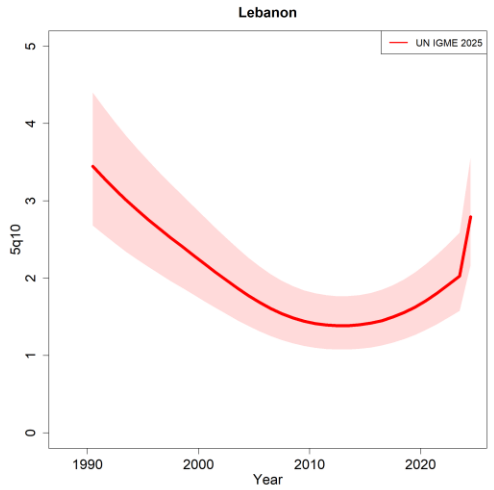
Lebanon
UN IGME 2025
5q10
Year

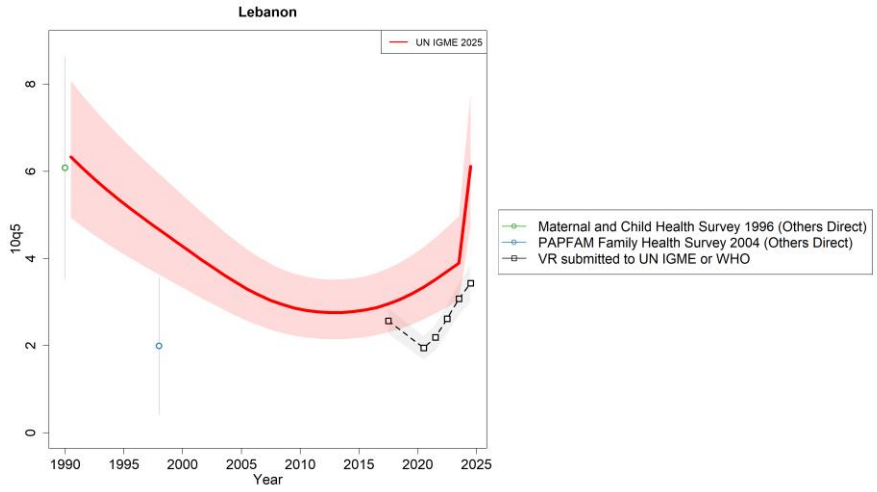
Lebanon
UN IGME 2025
10q5
Year
Maternal and Child Health Survey 1996 (Others Direct)
PAPFAM Family Health Survey 2004 (Others Direct)
VR submitted to UN IGME or WHO

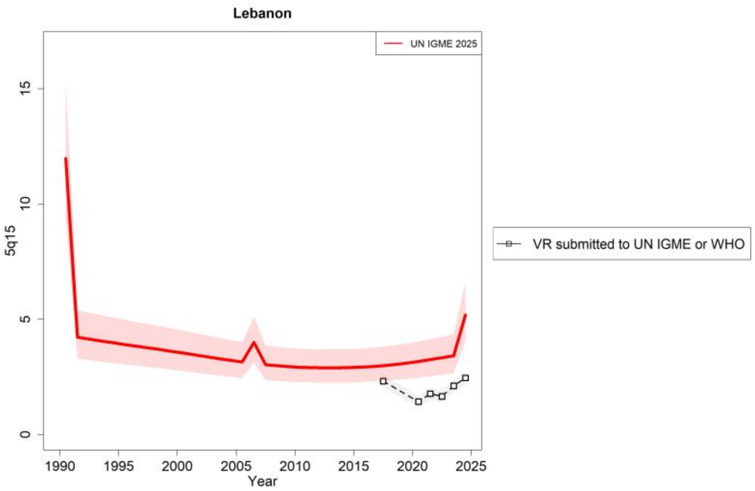
Lebanon
UN IGME 2025
VR submitted to UN IGME or WHO
5q15
0
5
10
15
1990
1995
2000
2005
2010
2015
2020
2025
Year

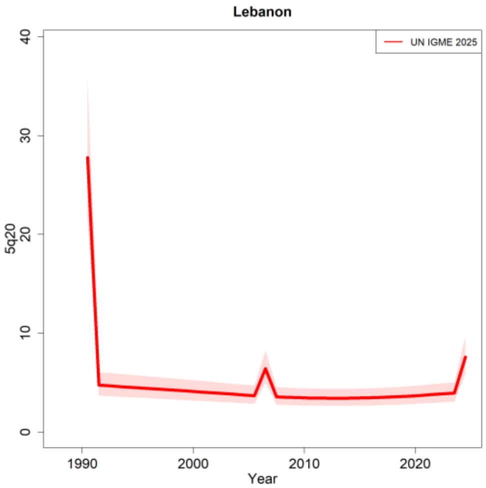
Lebanon
UN IGME 2025
5q20
0
10
20
30
40
1990
2000
2010
2020
Year

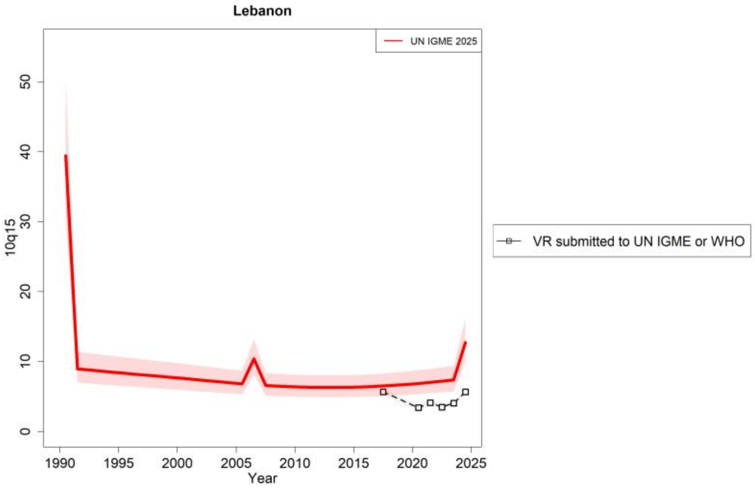
Lebanon
UN IGME 2025
VR submitted to UN IGME or WHO
10q15
0
10
20
30
40
50
1990
1995
2000
2005
2010
2015
2020
2025
Year

Lesotho (LSO)

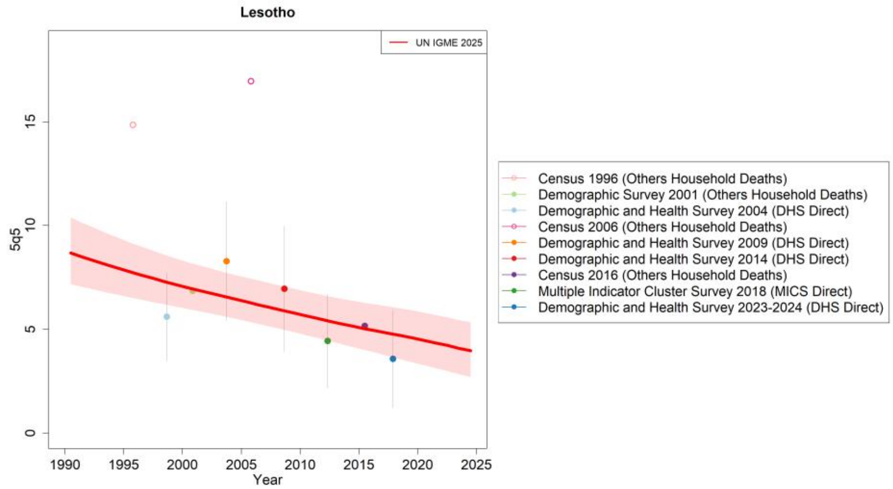
Lesotho
UN IGME 2025
5q5
Year
Census 1996 (Others Household Deaths)
Demographic Survey 2001 (Others Household Deaths)
Demographic and Health Survey 2004 (DHS Direct)
Census 2006 (Others Household Deaths)
Demographic and Health Survey 2009 (DHS Direct)
Demographic and Health Survey 2014 (DHS Direct)
Census 2016 (Others Household Deaths)
Multiple Indicator Cluster Survey 2018 (MICS Direct)
Demographic and Health Survey 2023-2024 (DHS Direct)

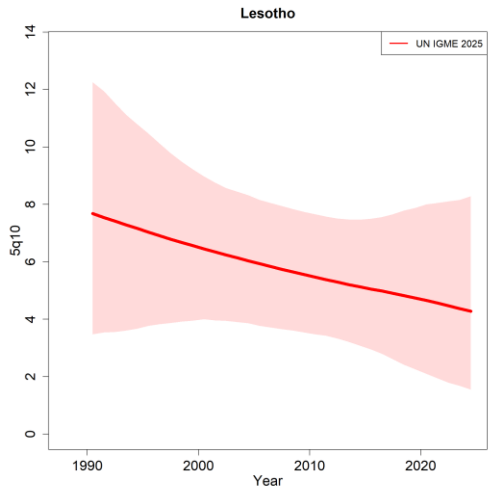
Lesotho
UN IGME 2025
5q10
Year

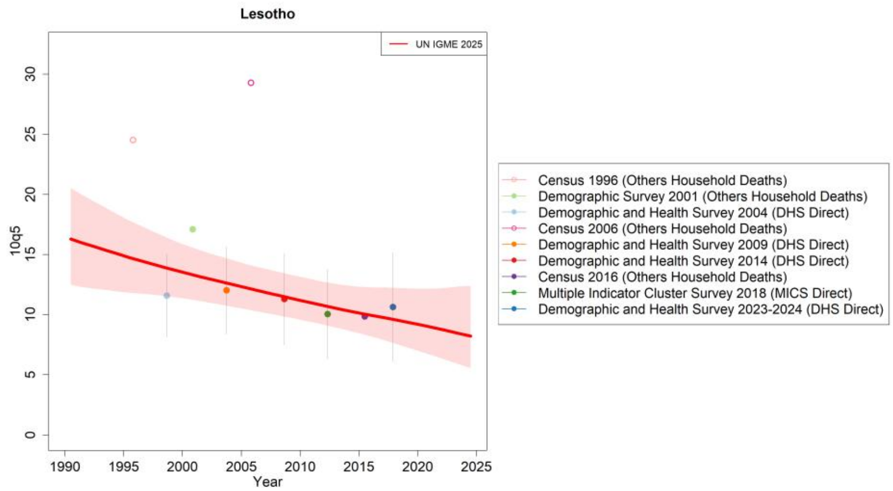
Lesotho
UN IGME 2025
10q5
Year
Census 1996 (Others Household Deaths)
Demographic Survey 2001 (Others Household Deaths)
Demographic and Health Survey 2004 (DHS Direct)
Census 2006 (Others Household Deaths)
Demographic and Health Survey 2009 (DHS Direct)
Demographic and Health Survey 2014 (DHS Direct)
Census 2016 (Others Household Deaths)
Multiple Indicator Cluster Survey 2018 (MICS Direct)
Demographic and Health Survey 2023-2024 (DHS Direct)

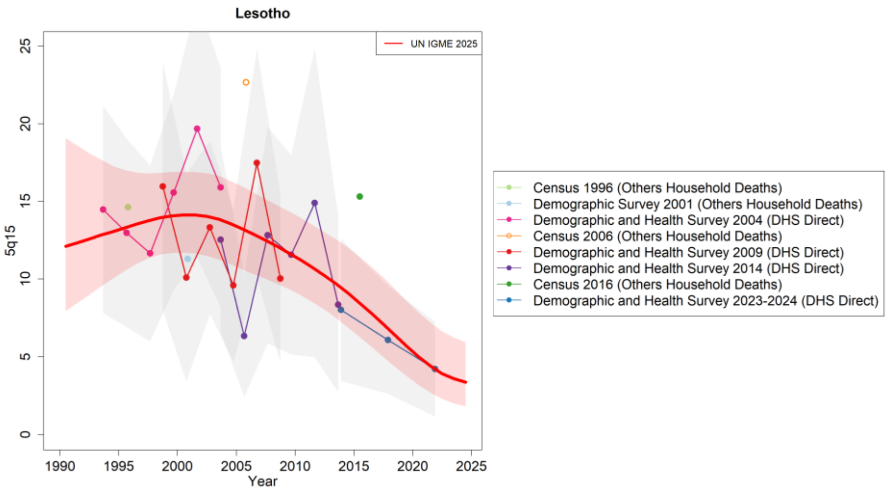
Lesotho
UN IGME 2025
5q15
Year
Census 1996 (Others Household Deaths)
Demographic Survey 2001 (Others Household Deaths)
Demographic and Health Survey 2004 (DHS Direct)
Census 2006 (Others Household Deaths)
Demographic and Health Survey 2009 (DHS Direct)
Demographic and Health Survey 2014 (DHS Direct)
Census 2016 (Others Household Deaths)
Demographic and Health Survey 2023-2024 (DHS Direct)

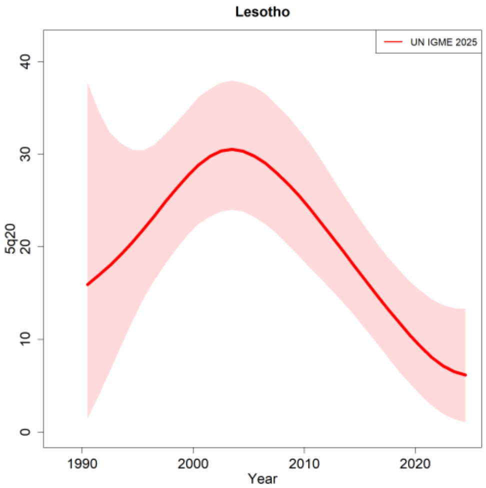
Lesotho
UN IGME 2025
5q20
Year

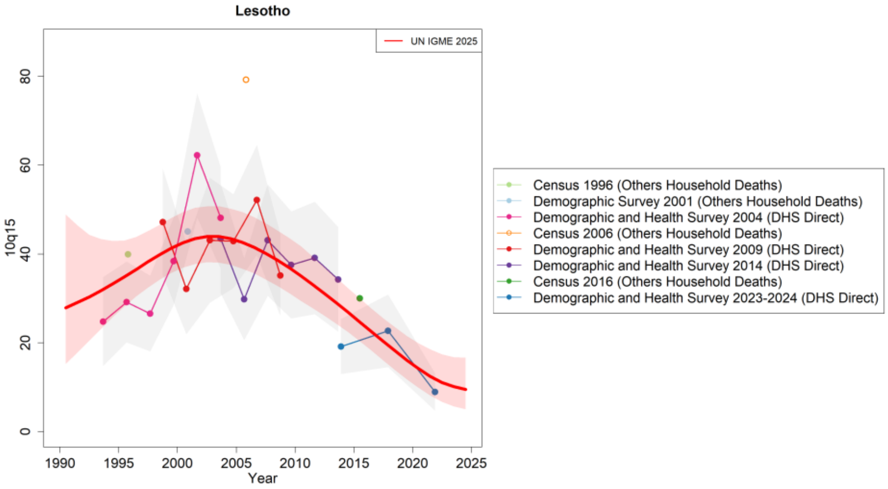
Lesotho
UN IGME 2025
10q15
Year
Census 1996 (Others Household Deaths)
Demographic Survey 2001 (Others Household Deaths)
Demographic and Health Survey 2004 (DHS Direct)
Census 2006 (Others Household Deaths)
Demographic and Health Survey 2009 (DHS Direct)
Demographic and Health Survey 2014 (DHS Direct)
Census 2016 (Others Household Deaths)
Demographic and Health Survey 2023-2024 (DHS Direct)

Liberia (LBR)

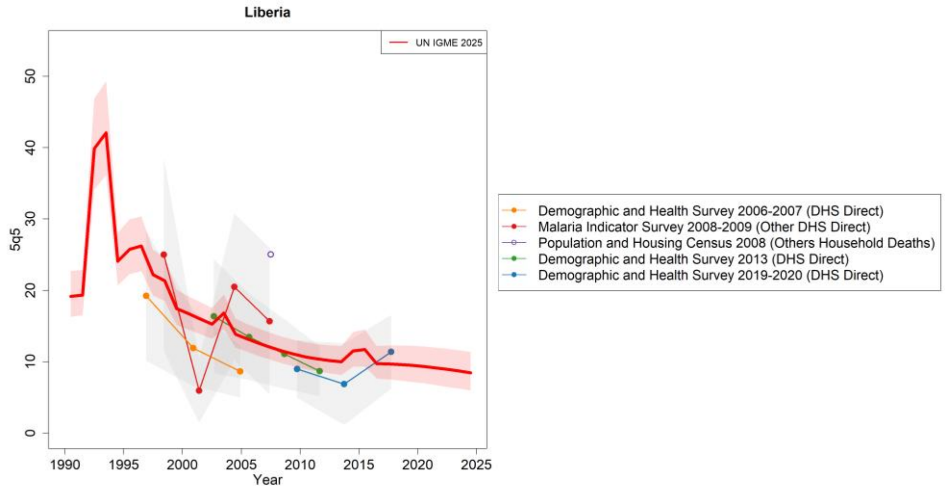
Liberia
UN IGME 2025
5q5
Year
Demographic and Health Survey 2006-2007 (DHS Direct)
Malaria Indicator Survey 2008-2009 (Other DHS Direct)
Population and Housing Census 2008 (Others Household Deaths)
Demographic and Health Survey 2013 (DHS Direct)
Demographic and Health Survey 2019-2020 (DHS Direct)

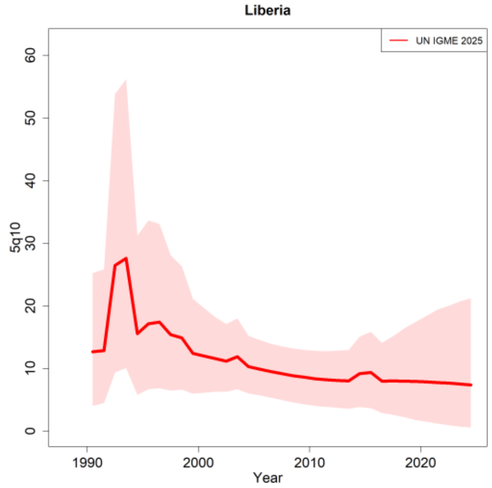
Liberia
UN IGME 2025
5q10
Year

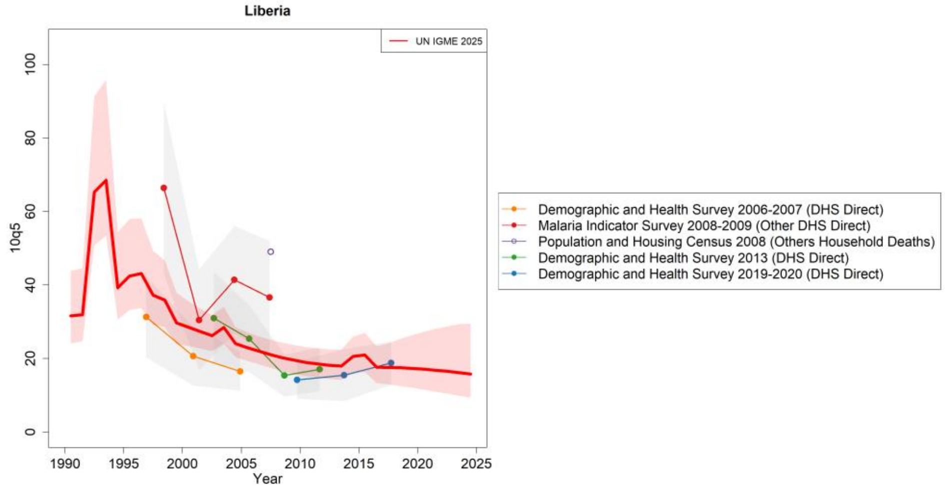
Liberia
UN IGME 2025
10q5
Year
Demographic and Health Survey 2006-2007 (DHS Direct)
Malaria Indicator Survey 2008-2009 (Other DHS Direct)
Population and Housing Census 2008 (Others Household Deaths)
Demographic and Health Survey 2013 (DHS Direct)
Demographic and Health Survey 2019-2020 (DHS Direct)

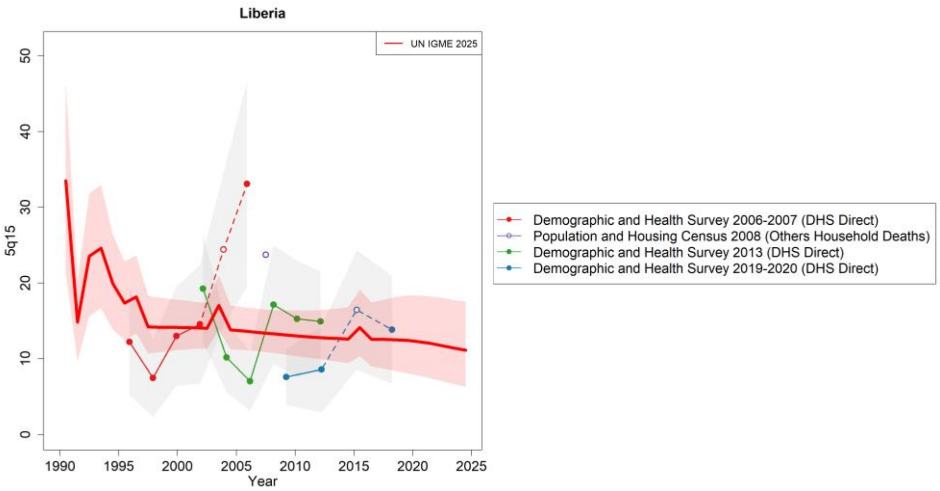
Liberia
UN IGME 2025
5q15
Year
Demographic and Health Survey 2006-2007 (DHS Direct)
Population and Housing Census 2008 (Others Household Deaths)
Demographic and Health Survey 2013 (DHS Direct)
Demographic and Health Survey 2019-2020 (DHS Direct)

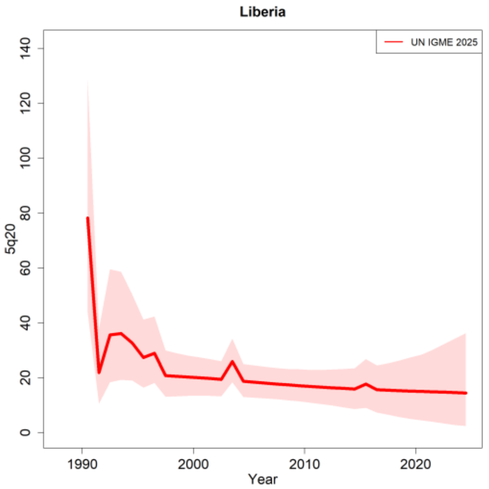
Liberia
UN IGME 2025
5q20
Year

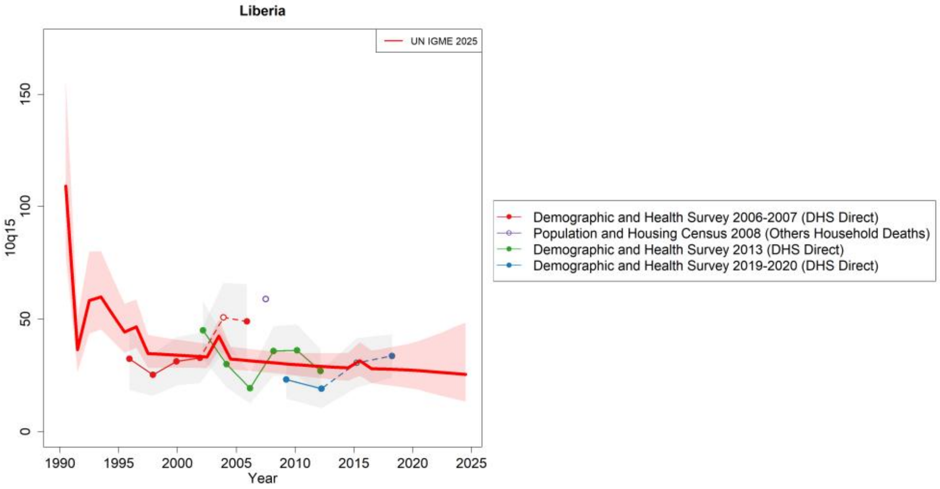
Liberia
UN IGME 2025
10q15
Year
Demographic and Health Survey 2006-2007 (DHS Direct)
Population and Housing Census 2008 (Others Household Deaths)
Demographic and Health Survey 2013 (DHS Direct)
Demographic and Health Survey 2019-2020 (DHS Direct)

Libya (LBY)

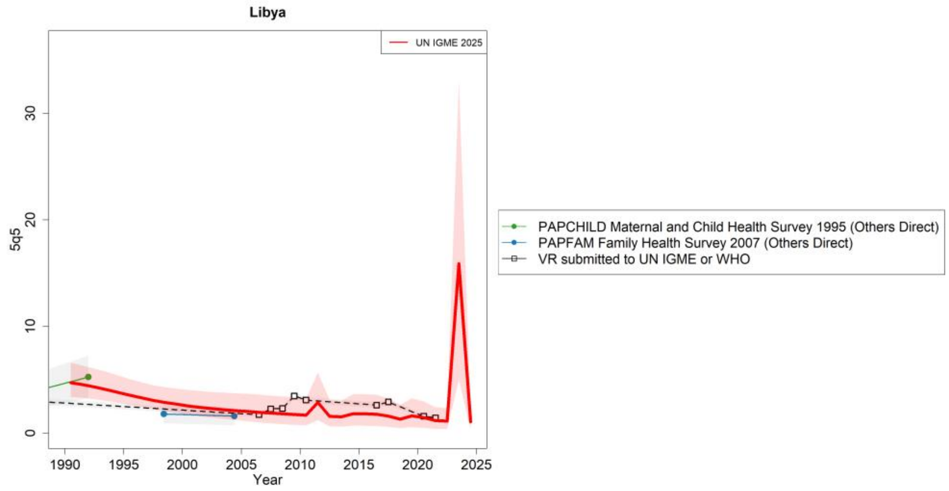
Libya
UN IGME 2025
5q5
0
10
20
30
1990
1995
2000
2005
2010
2015
2020
2025
Year
PAPCHILD Maternal and Child Health Survey 1995 (Others Direct)
PAPFAM Family Health Survey 2007 (Others Direct)
VR submitted to UN IGME or WHO

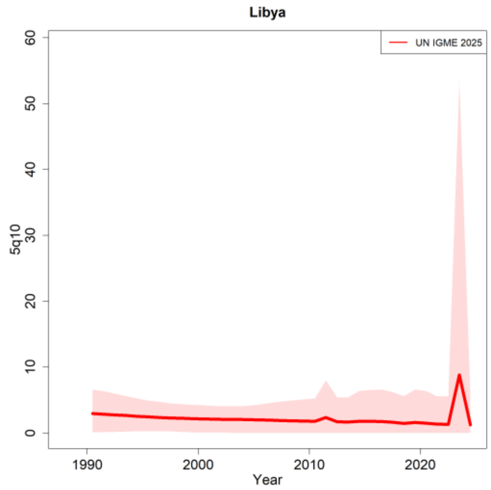
Libya
UN IGME 2025
5q10
0
10
20
30
40
50
60
1990
2000
2010
2020
Year

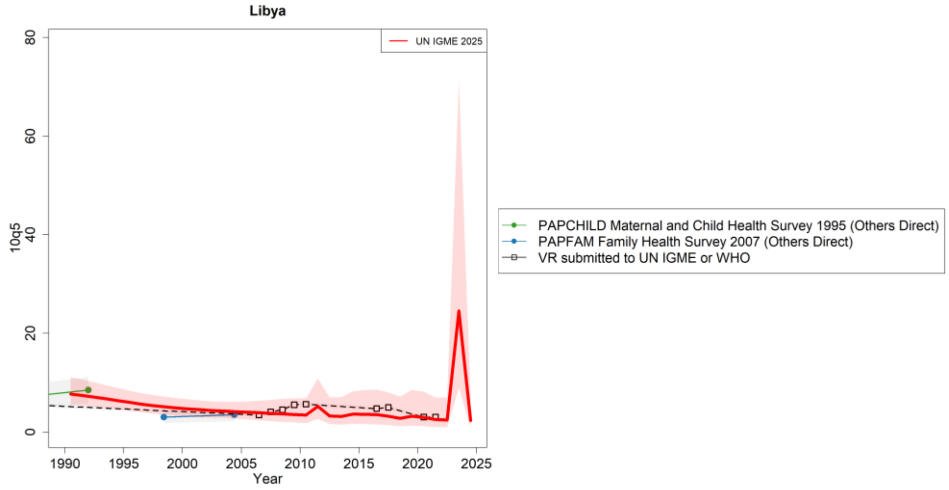
Libya
UN IGME 2025
10q5
0
20
40
60
80
1990
1995
2000
2005
2010
2015
2020
2025
Year
PAPCHILD Maternal and Child Health Survey 1995 (Others Direct)
PAPFAM Family Health Survey 2007 (Others Direct)
VR submitted to UN IGME or WHO

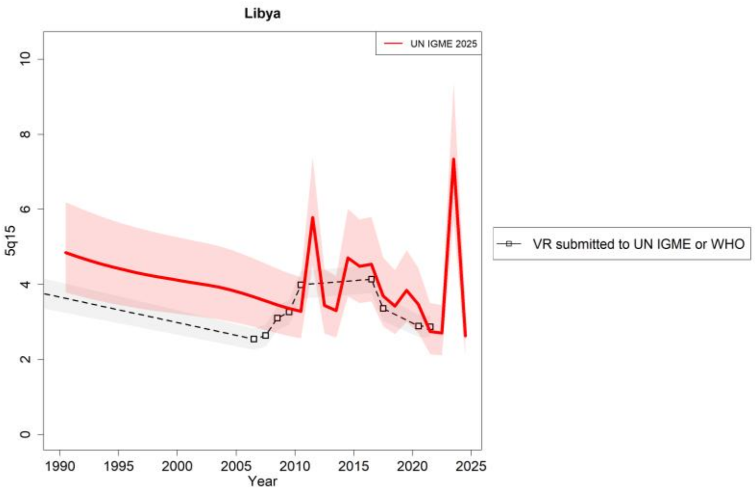
Libya
UN IGME 2025
VR submitted to UN IGME or WHO
5q15
Year

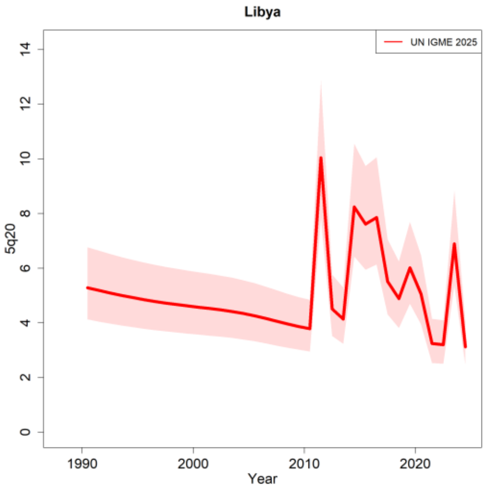
Libya
UN IGME 2025
5q20
Year

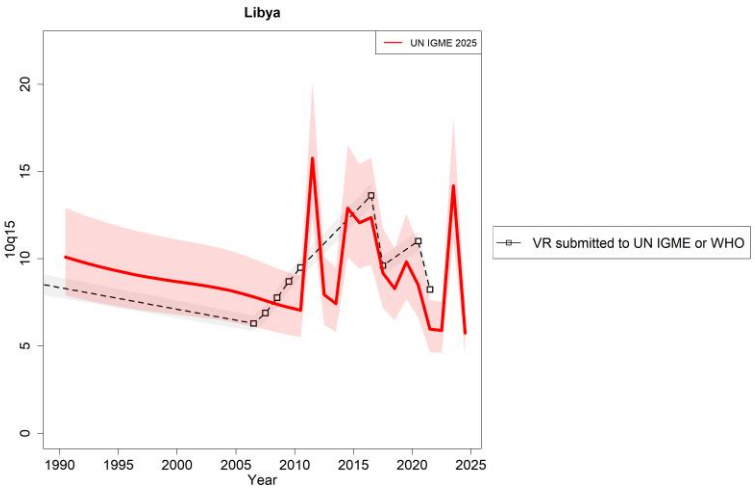
Libya
UN IGME 2025
VR submitted to UN IGME or WHO
10q15
Year

Lithuania (LTU)

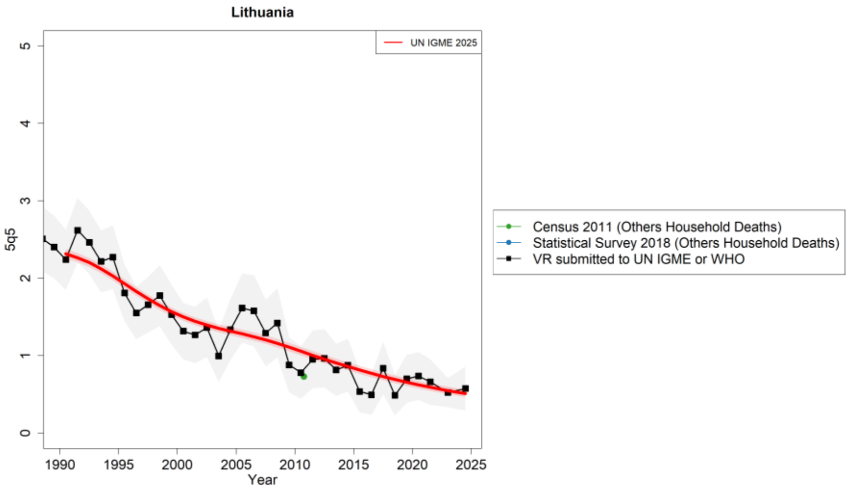

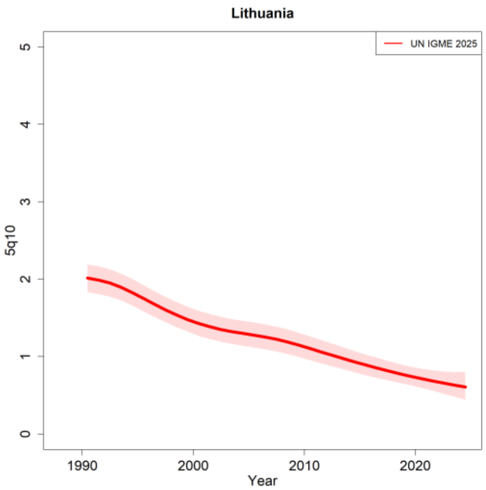

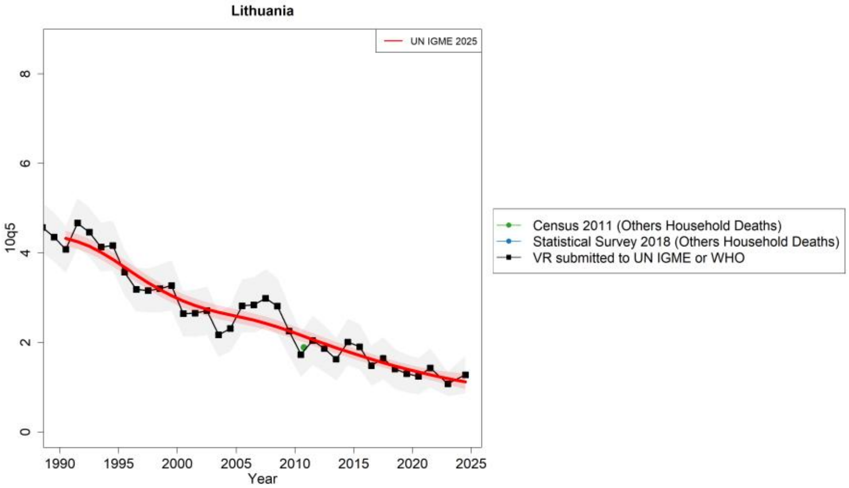

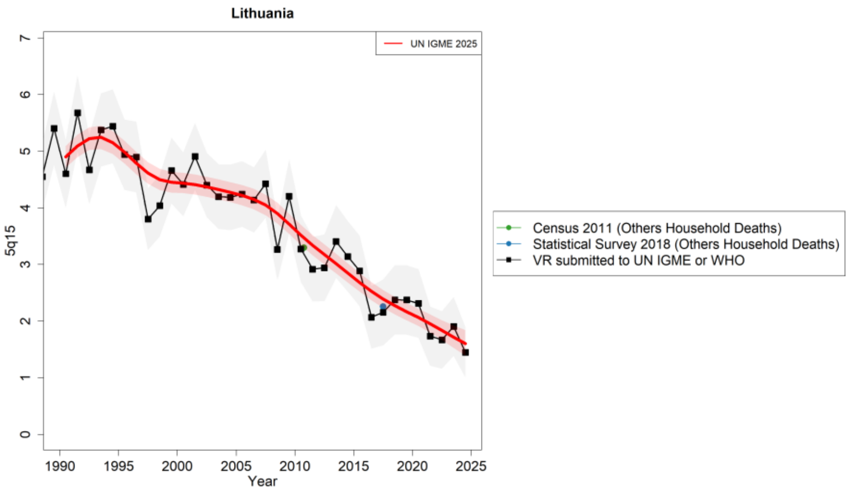
Lithuania
UN IGME 2025
5q15
Year
0
1
2
3
4
5
6
7
1990
1995
2000
2005
2010
2015
2020
2025
Census 2011 (Others Household Deaths)
Statistical Survey 2018 (Others Household Deaths)
VR submitted to UN IGME or WHO

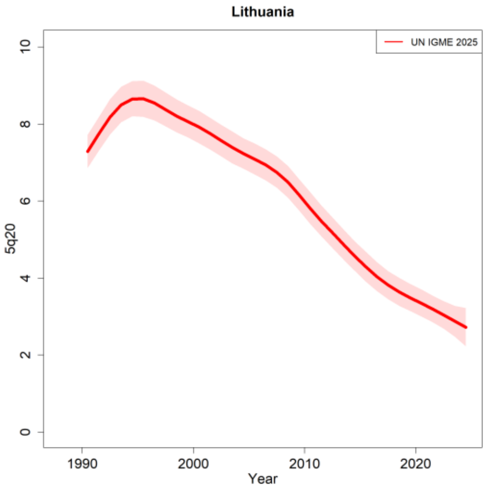
Lithuania
UN IGME 2025
5q20
Year
0
2
4
6
8
10
1990
2000
2010
2020

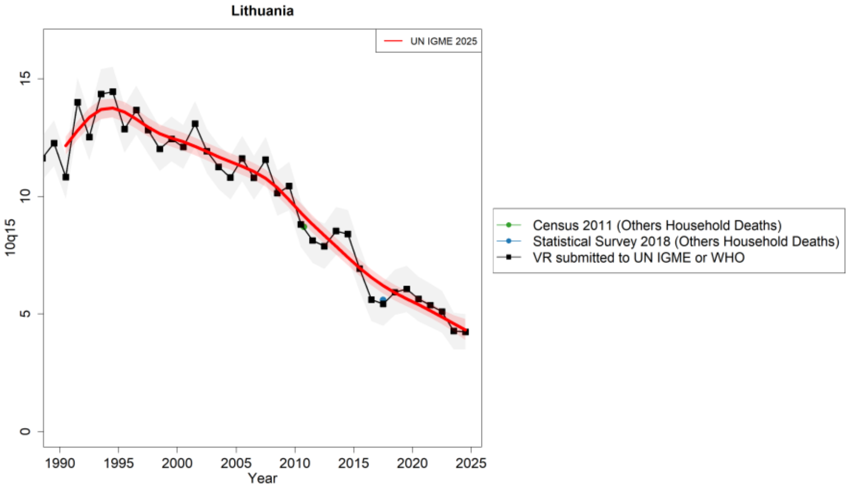
Lithuania
UN IGME 2025
10q15
Year
0
5
10
15
1990
1995
2000
2005
2010
2015
2020
2025
Census 2011 (Others Household Deaths)
Statistical Survey 2018 (Others Household Deaths)
VR submitted to UN IGME or WHO

Luxembourg (LUX)

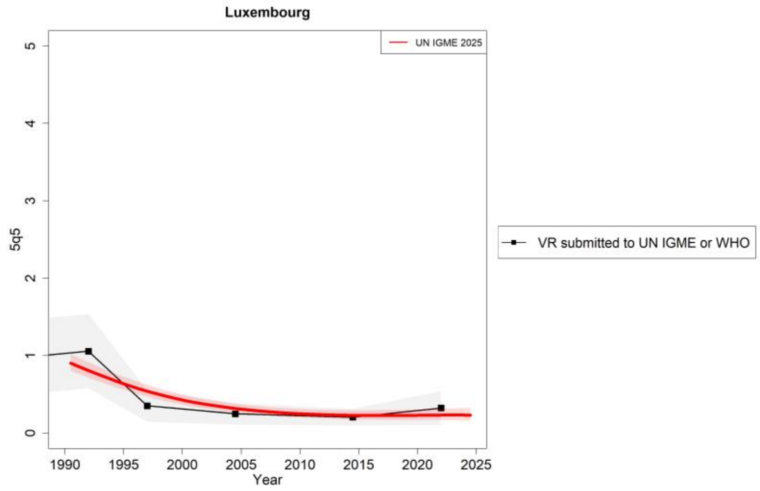

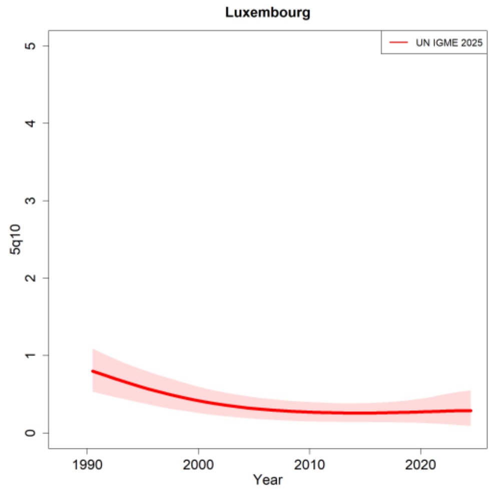

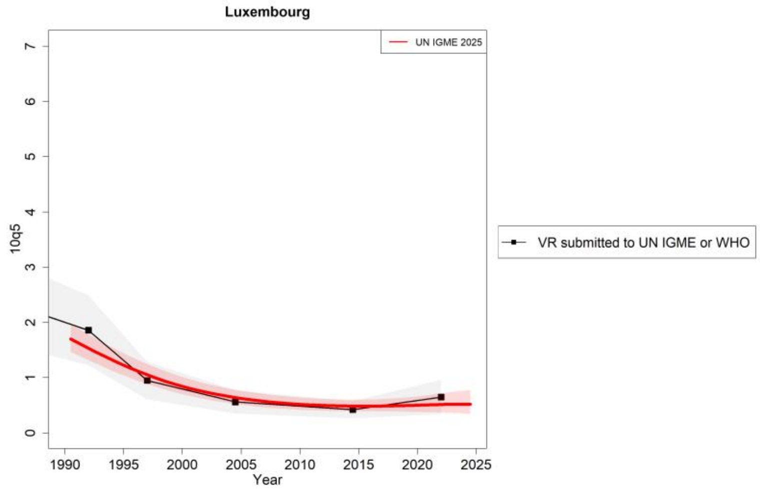

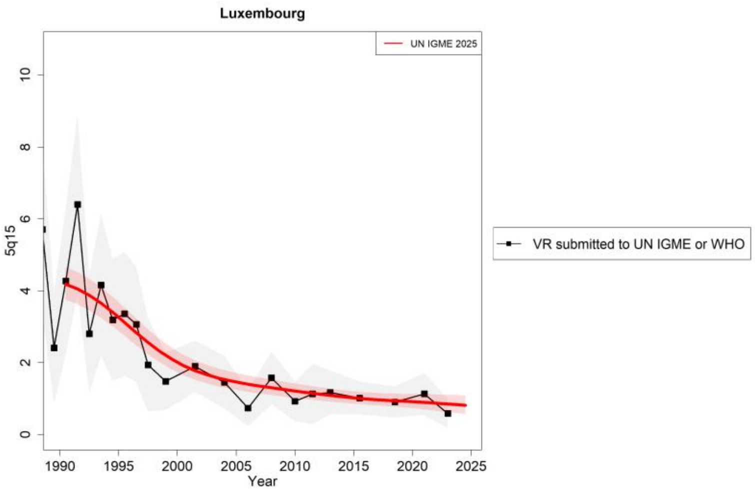
Luxembourg
UN IGME 2025
VR submitted to UN IGME or WHO
5q15
Year

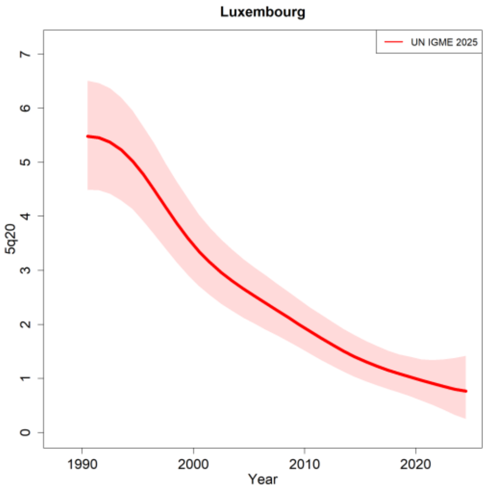
Luxembourg
UN IGME 2025
5q20
Year

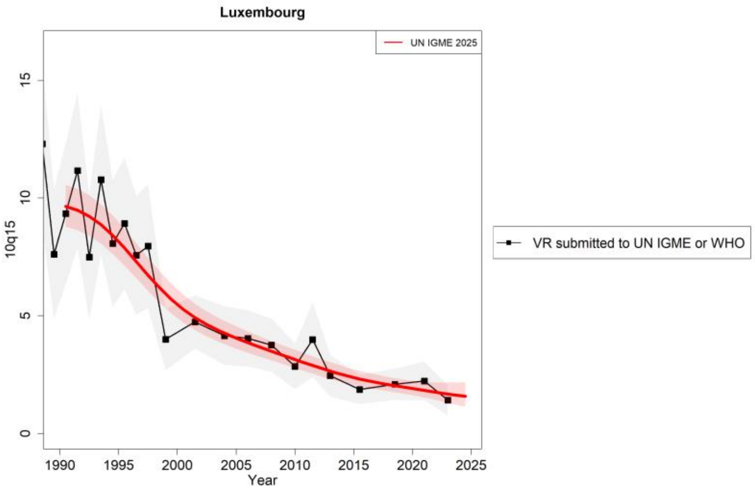
Luxembourg
UN IGME 2025
VR submitted to UN IGME or WHO
10q15
Year

Madagascar (MDG)

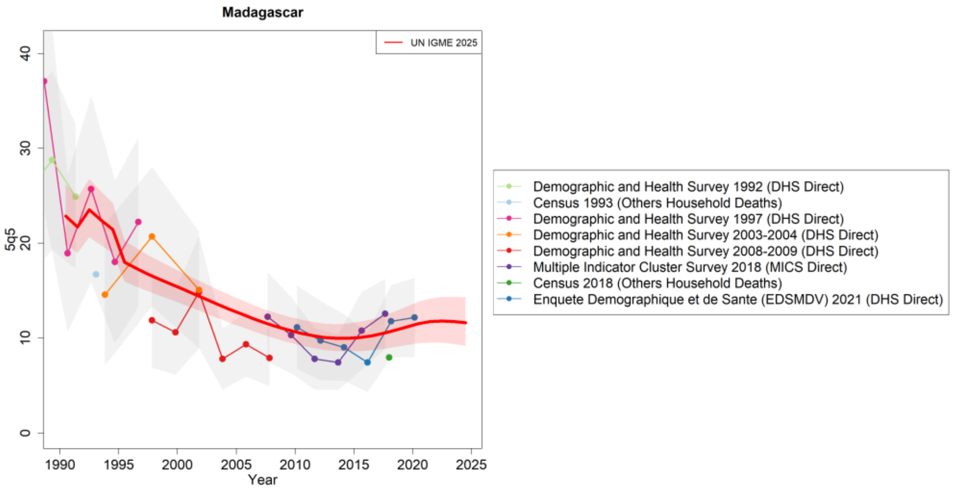

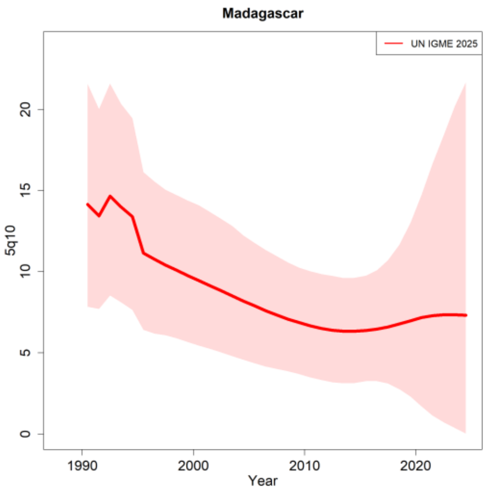

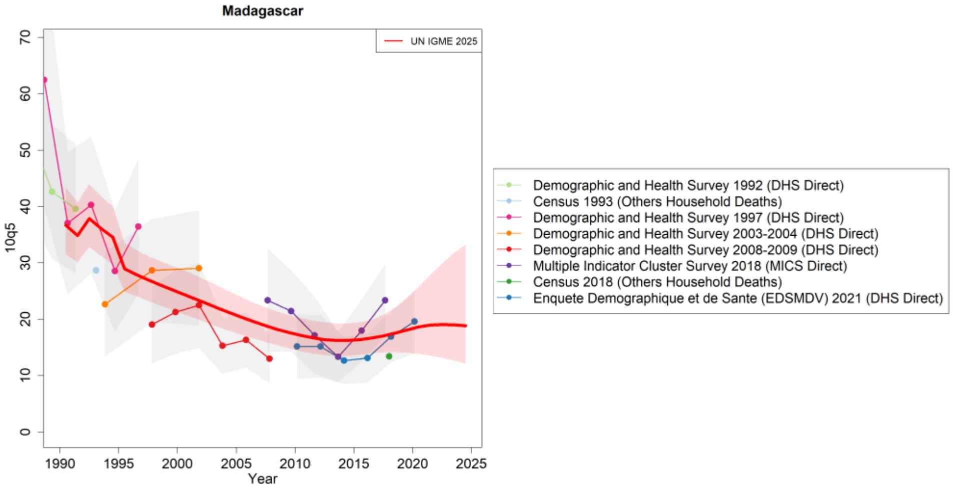

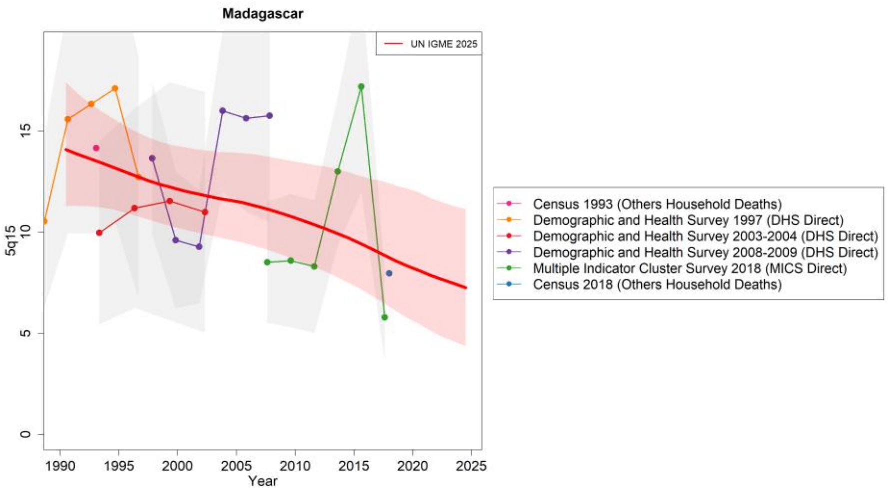
Madagascar
UN IGME 2025
5q15
Year
Census 1993 (Others Household Deaths)
Demographic and Health Survey 1997 (DHS Direct)
Demographic and Health Survey 2003-2004 (DHS Direct)
Demographic and Health Survey 2008-2009 (DHS Direct)
Multiple Indicator Cluster Survey 2018 (MICS Direct)
Census 2018 (Others Household Deaths)

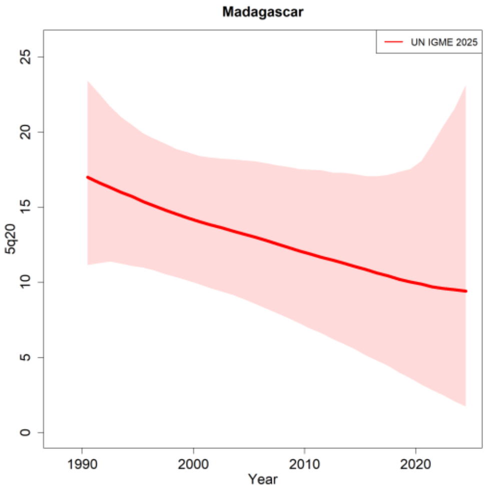
Madagascar
UN IGME 2025
5q20
Year

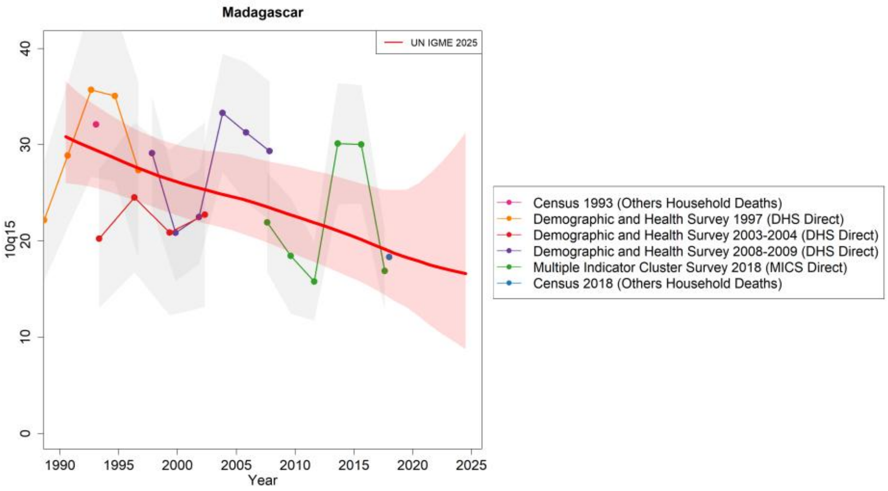
Madagascar
UN IGME 2025
10q15
Year
Census 1993 (Others Household Deaths)
Demographic and Health Survey 1997 (DHS Direct)
Demographic and Health Survey 2003-2004 (DHS Direct)
Demographic and Health Survey 2008-2009 (DHS Direct)
Multiple Indicator Cluster Survey 2018 (MICS Direct)
Census 2018 (Others Household Deaths)

Malawi (MWI)

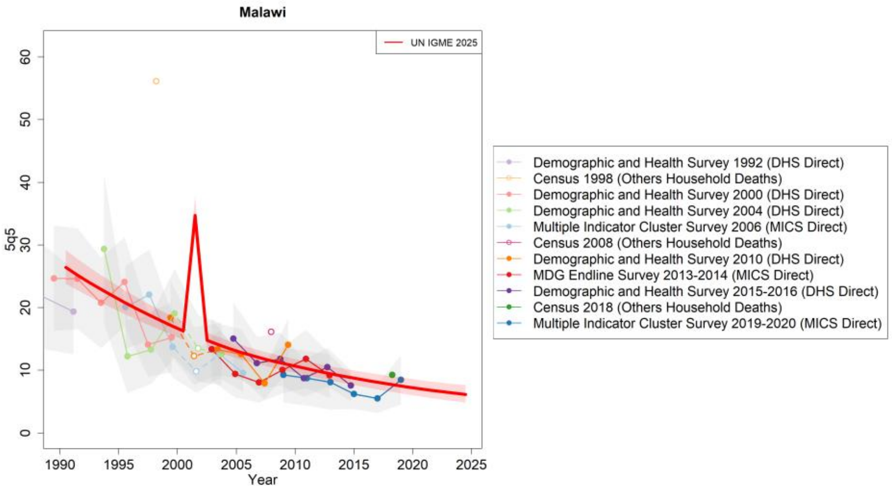
Malawi
UN IGME 2025
5q5
Year
Demographic and Health Survey 1992 (DHS Direct)
Census 1998 (Others Household Deaths)
Demographic and Health Survey 2000 (DHS Direct)
Demographic and Health Survey 2004 (DHS Direct)
Multiple Indicator Cluster Survey 2006 (MICS Direct)
Census 2008 (Others Household Deaths)
Demographic and Health Survey 2010 (DHS Direct)
MDG Endline Survey 2013-2014 (MICS Direct)
Demographic and Health Survey 2015-2016 (DHS Direct)
Census 2018 (Others Household Deaths)
Multiple Indicator Cluster Survey 2019-2020 (MICS Direct)

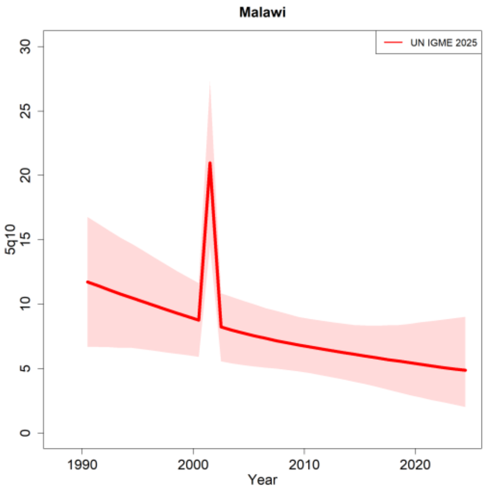
Malawi
UN IGME 2025
5q10
Year

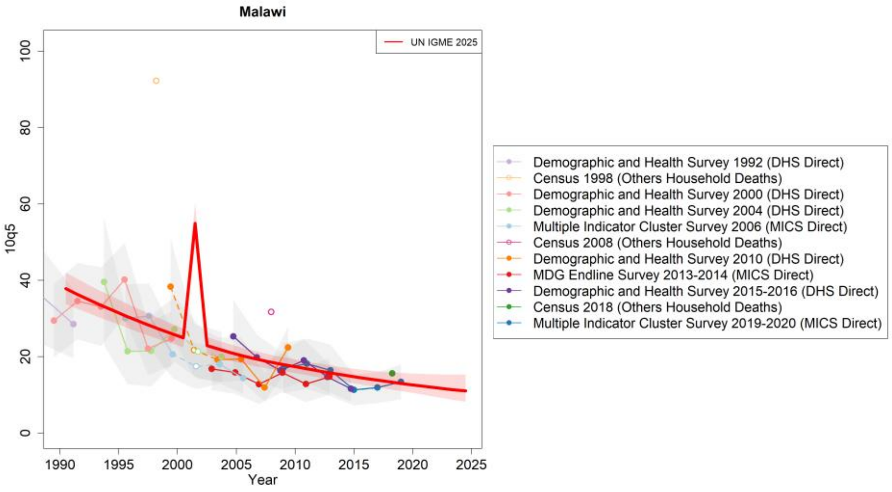
Malawi
UN IGME 2025
10q5
Year
Demographic and Health Survey 1992 (DHS Direct)
Census 1998 (Others Household Deaths)
Demographic and Health Survey 2000 (DHS Direct)
Demographic and Health Survey 2004 (DHS Direct)
Multiple Indicator Cluster Survey 2006 (MICS Direct)
Census 2008 (Others Household Deaths)
Demographic and Health Survey 2010 (DHS Direct)
MDG Endline Survey 2013-2014 (MICS Direct)
Demographic and Health Survey 2015-2016 (DHS Direct)
Census 2018 (Others Household Deaths)
Multiple Indicator Cluster Survey 2019-2020 (MICS Direct)

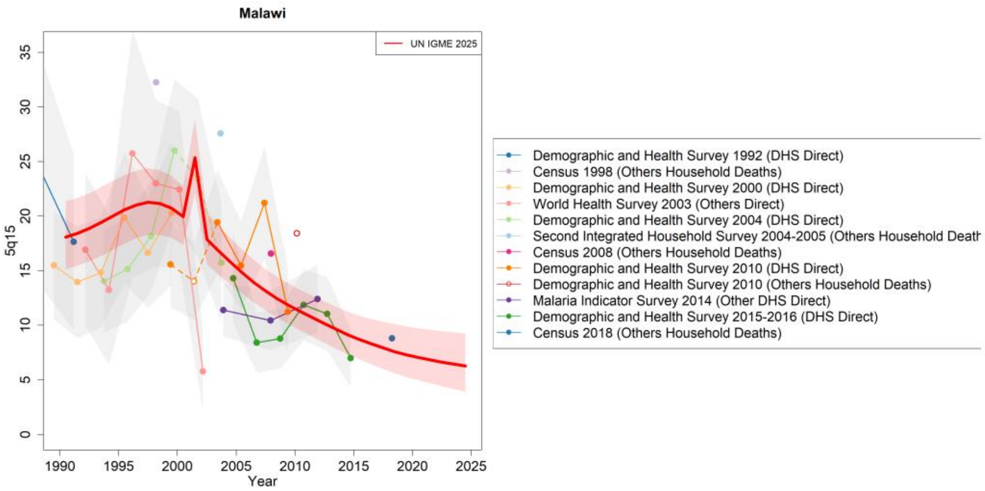
Malawi
UN IGME 2025
5q15
Year
Demographic and Health Survey 1992 (DHS Direct)
Census 1998 (Others Household Deaths)
Demographic and Health Survey 2000 (DHS Direct)
World Health Survey 2003 (Others Direct)
Demographic and Health Survey 2004 (DHS Direct)
Second Integrated Household Survey 2004-2005 (Others Household Deatl
Census 2008 (Others Household Deaths)
Demographic and Health Survey 2010 (DHS Direct)
Demographic and Health Survey 2010 (Others Household Deaths)
Malaria Indicator Survey 2014 (Other DHS Direct)
Demographic and Health Survey 2015-2016 (DHS Direct)
Census 2018 (Others Household Deaths)

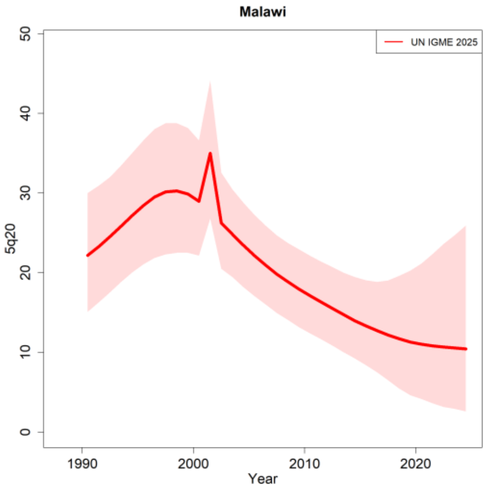
Malawi
UN IGME 2025
5q20
Year

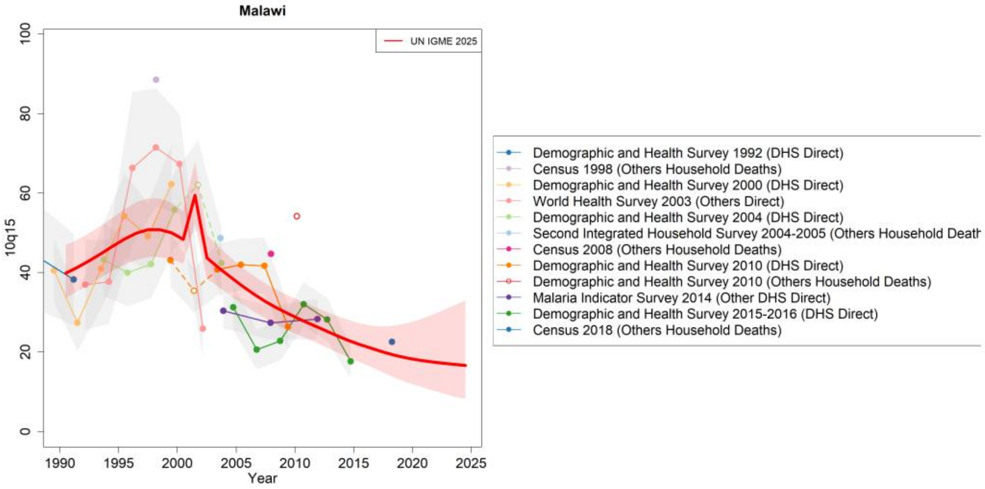
Malawi
UN IGME 2025
10q15
Year
Demographic and Health Survey 1992 (DHS Direct)
Census 1998 (Others Household Deaths)
Demographic and Health Survey 2000 (DHS Direct)
World Health Survey 2003 (Others Direct)
Demographic and Health Survey 2004 (DHS Direct)
Second Integrated Household Survey 2004-2005 (Others Household Deatl
Census 2008 (Others Household Deaths)
Demographic and Health Survey 2010 (DHS Direct)
Demographic and Health Survey 2010 (Others Household Deaths)
Malaria Indicator Survey 2014 (Other DHS Direct)
Demographic and Health Survey 2015-2016 (DHS Direct)
Census 2018 (Others Household Deaths)

Malaysia (MYS)

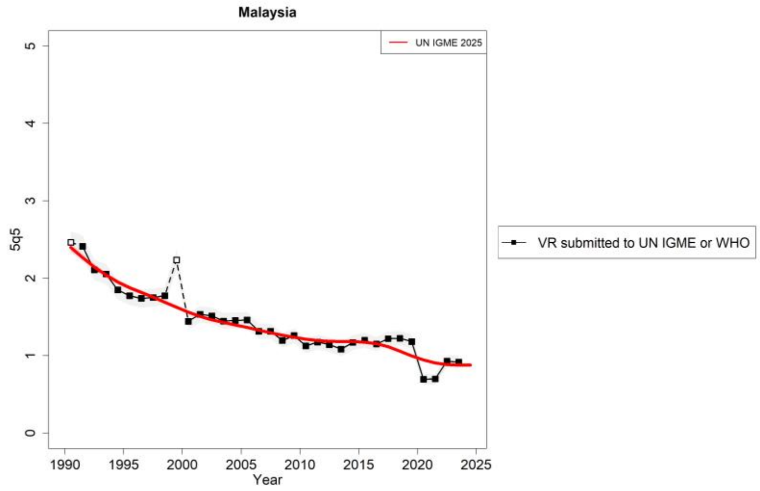

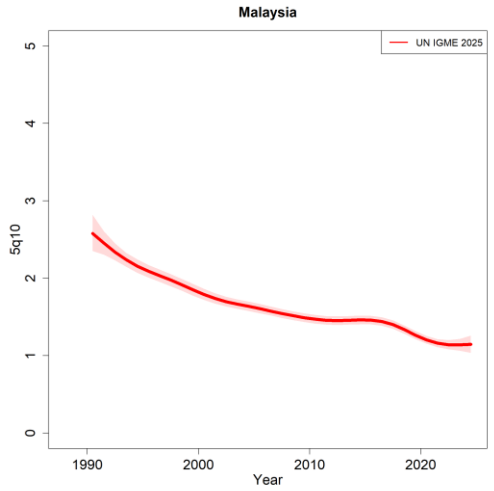

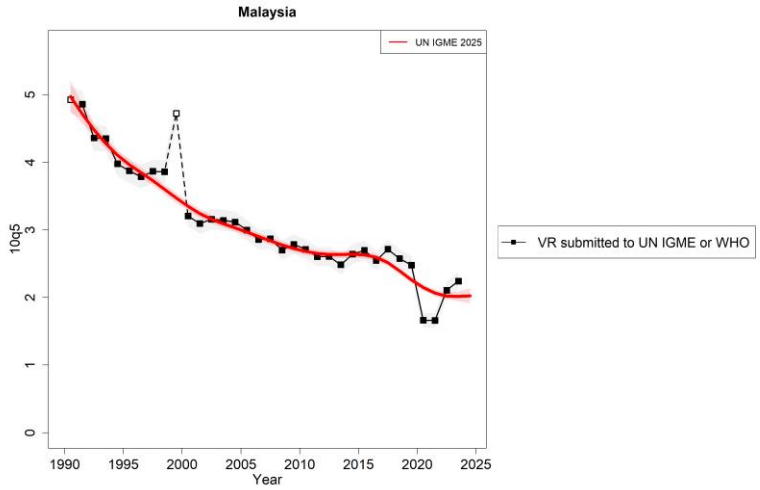

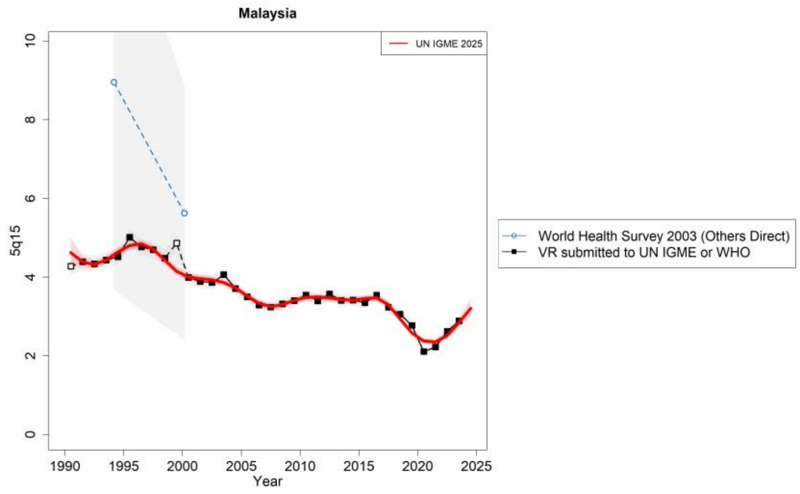
Malaysia
UN IGME 2025
World Health Survey 2003 (Others Direct)
VR submitted to UN IGME or WHO
5q15
Year
0
2
4
6
8
10
1990
1995
2000
2005
2010
2015
2020
2025

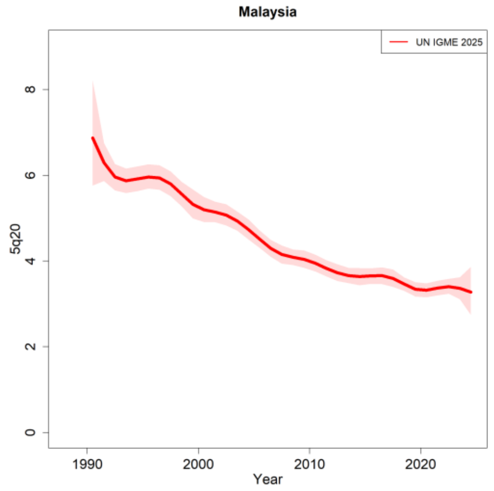
Malaysia
UN IGME 2025
5q20
Year
0
2
4
6
8
1990
2000
2010
2020

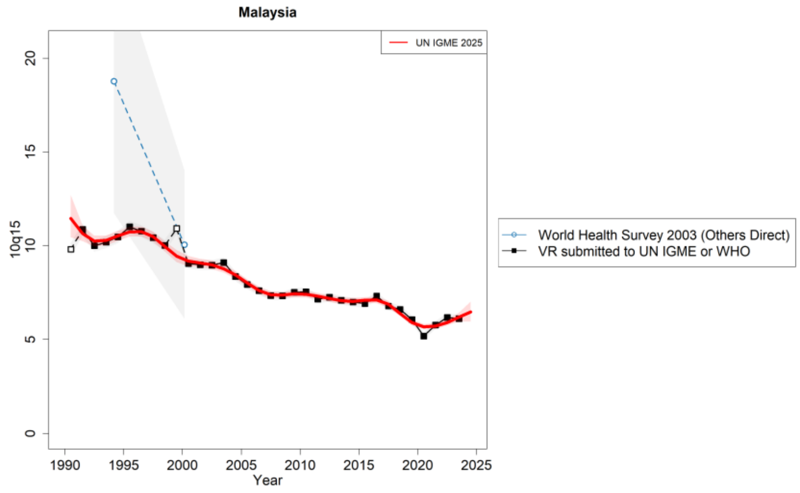
Malaysia
UN IGME 2025
World Health Survey 2003 (Others Direct)
VR submitted to UN IGME or WHO
10q15
Year
0
5
10
15
20
1990
1995
2000
2005
2010
2015
2020
2025

Maldives (MDV)

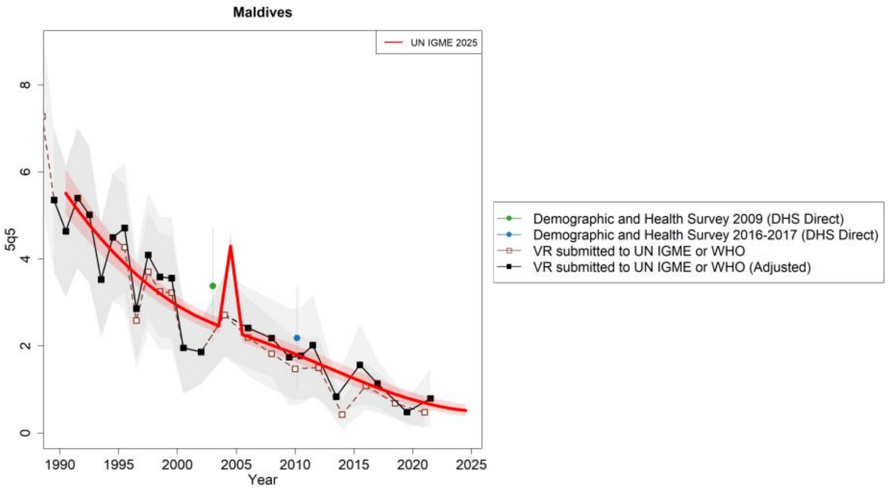

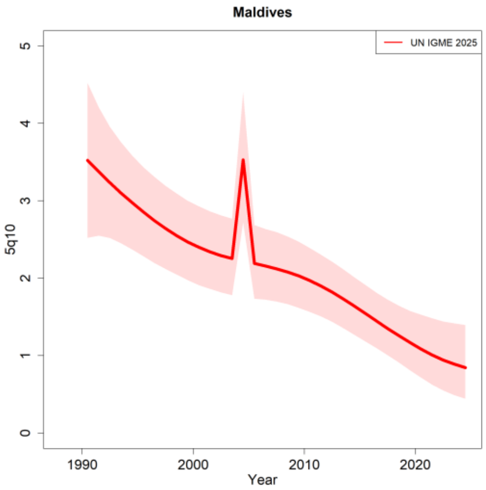

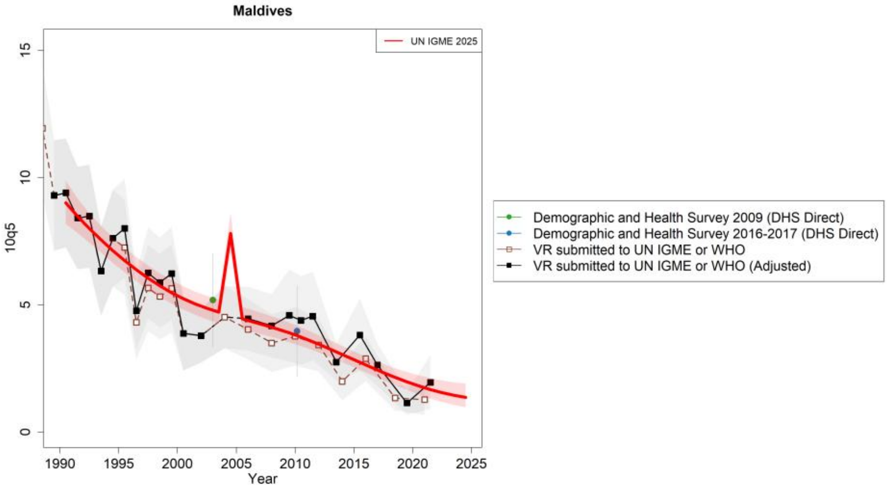

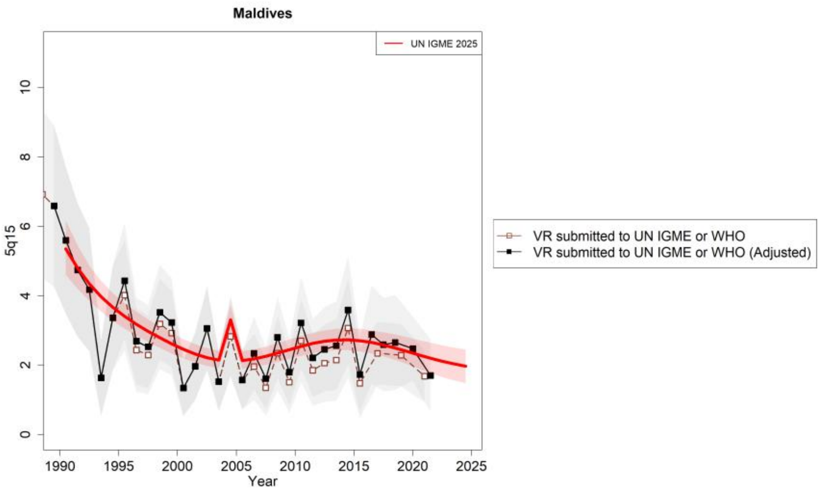
Maldives
UN IGME 2025
5q15
Year
VR submitted to UN IGME or WHO
VR submitted to UN IGME or WHO (Adjusted)

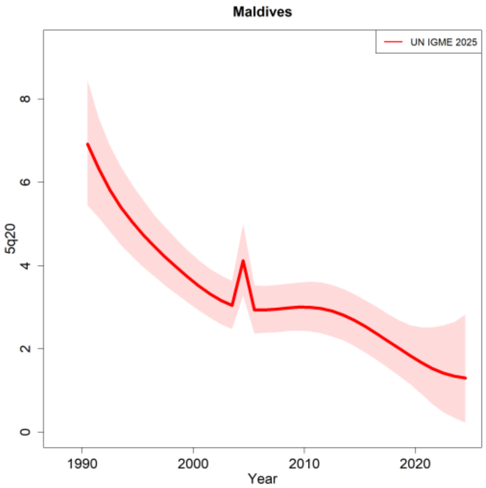
Maldives
UN IGME 2025
5q20
Year

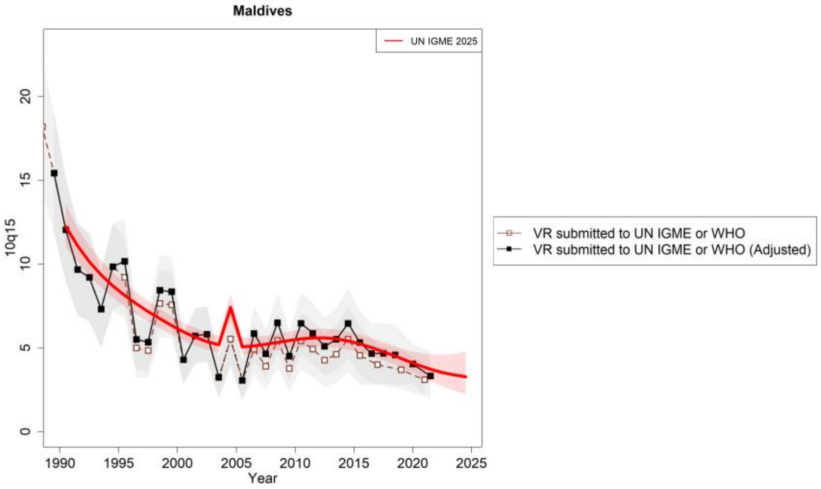
Maldives
UN IGME 2025
10q15
Year
VR submitted to UN IGME or WHO
VR submitted to UN IGME or WHO (Adjusted)

Mali (MLI)

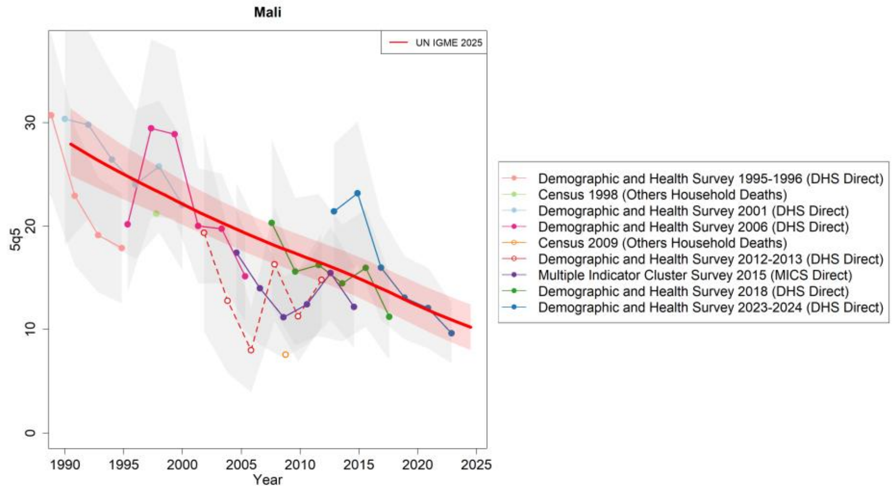
Mali
UN IGME 2025
5q5
Year
Demographic and Health Survey 1995-1996 (DHS Direct)
Census 1998 (Others Household Deaths)
Demographic and Health Survey 2001 (DHS Direct)
Demographic and Health Survey 2006 (DHS Direct)
Census 2009 (Others Household Deaths)
Demographic and Health Survey 2012-2013 (DHS Direct)
Multiple Indicator Cluster Survey 2015 (MICS Direct)
Demographic and Health Survey 2018 (DHS Direct)
Demographic and Health Survey 2023-2024 (DHS Direct)

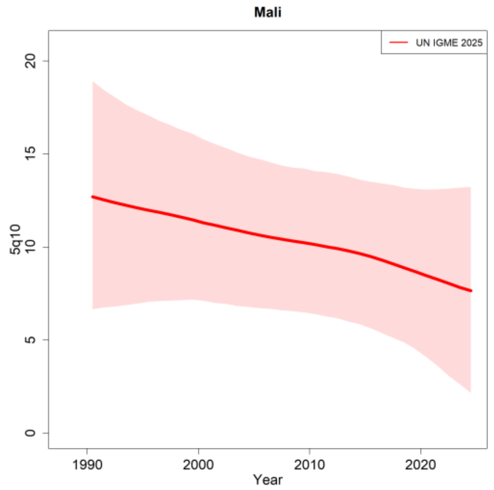
Mali
UN IGME 2025
5q10
Year

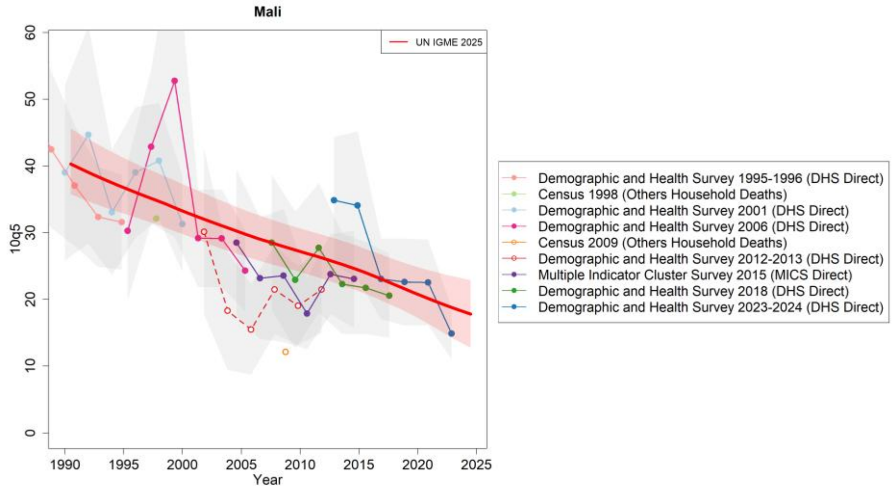
Mali
UN IGME 2025
10q5
Year
Demographic and Health Survey 1995-1996 (DHS Direct)
Census 1998 (Others Household Deaths)
Demographic and Health Survey 2001 (DHS Direct)
Demographic and Health Survey 2006 (DHS Direct)
Census 2009 (Others Household Deaths)
Demographic and Health Survey 2012-2013 (DHS Direct)
Multiple Indicator Cluster Survey 2015 (MICS Direct)
Demographic and Health Survey 2018 (DHS Direct)
Demographic and Health Survey 2023-2024 (DHS Direct)

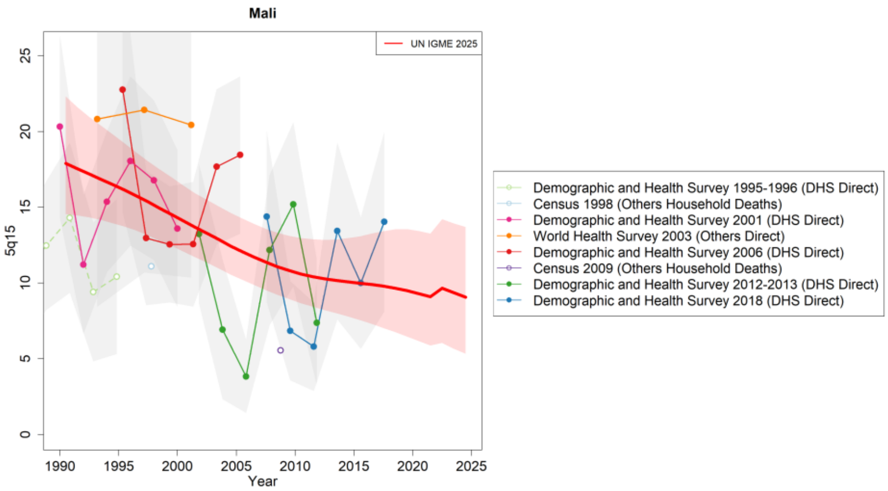
Mali
UN IGME 2025
5q15
Year
Demographic and Health Survey 1995-1996 (DHS Direct)
Census 1998 (Others Household Deaths)
Demographic and Health Survey 2001 (DHS Direct)
World Health Survey 2003 (Others Direct)
Demographic and Health Survey 2006 (DHS Direct)
Census 2009 (Others Household Deaths)
Demographic and Health Survey 2012-2013 (DHS Direct)
Demographic and Health Survey 2018 (DHS Direct)

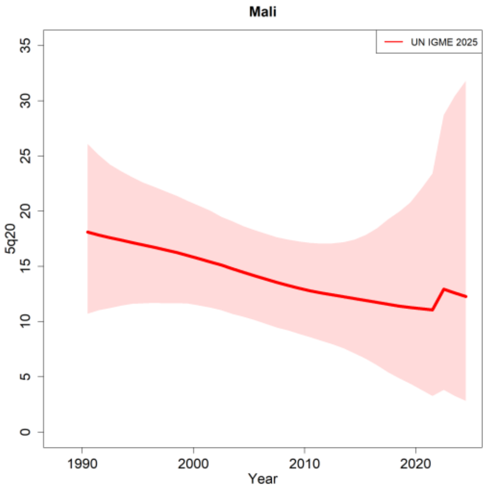
Mali
UN IGME 2025
5q20
Year

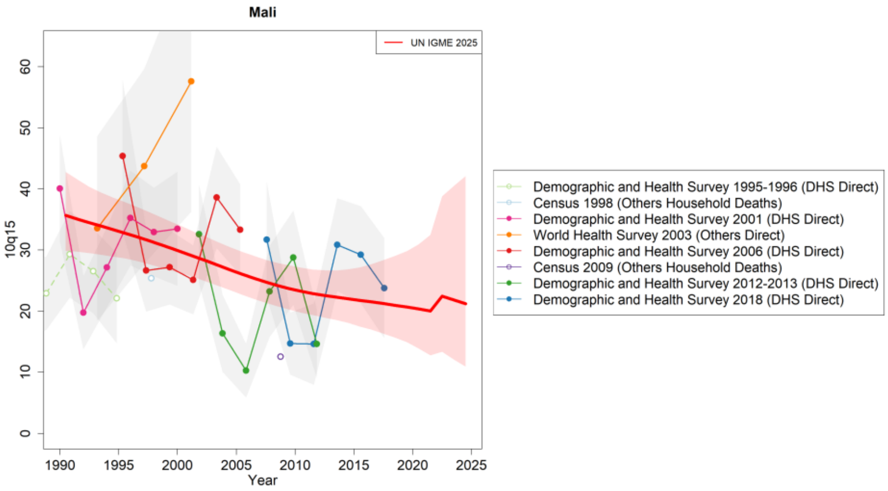
Mali
UN IGME 2025
10q15
Year
Demographic and Health Survey 1995-1996 (DHS Direct)
Census 1998 (Others Household Deaths)
Demographic and Health Survey 2001 (DHS Direct)
World Health Survey 2003 (Others Direct)
Demographic and Health Survey 2006 (DHS Direct)
Census 2009 (Others Household Deaths)
Demographic and Health Survey 2012-2013 (DHS Direct)
Demographic and Health Survey 2018 (DHS Direct)

Malta (MLT)

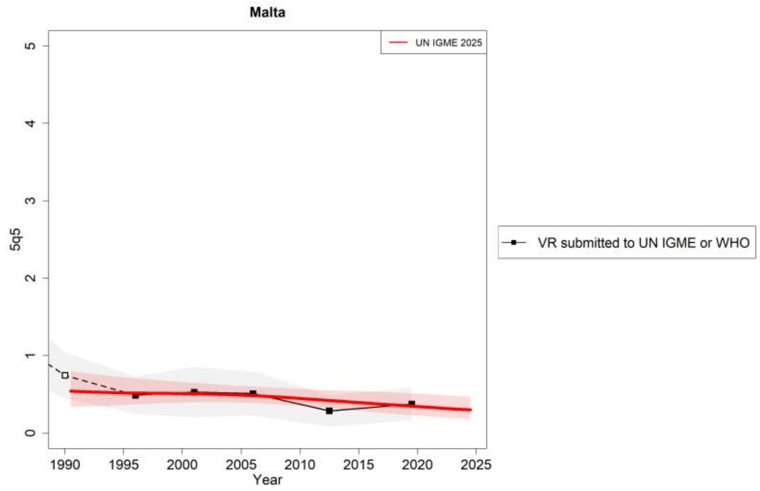
Malta
UN IGME 2025
VR submitted to UN IGME or WHO
5q5
0
1
2
3
4
5
1990
1995
2000
2005
2010
2015
2020
2025
Year

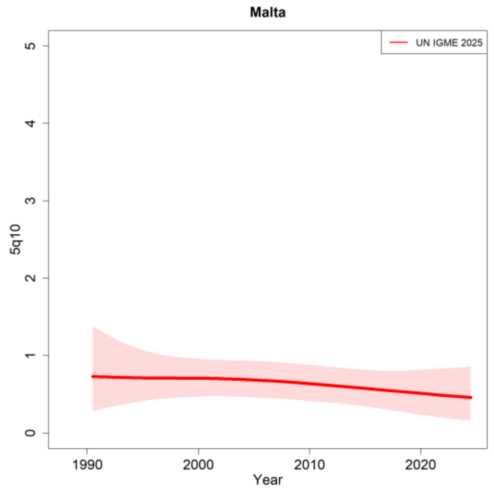
Malta
UN IGME 2025
5q10
0
1
2
3
4
5
1990
2000
2010
2020
Year

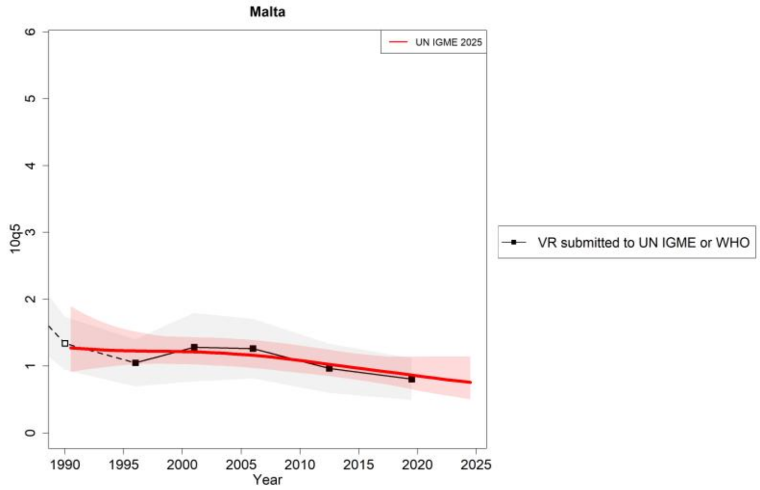
Malta
UN IGME 2025
VR submitted to UN IGME or WHO
10q5
0
1
2
3
4
5
6
1990
1995
2000
2005
2010
2015
2020
2025
Year

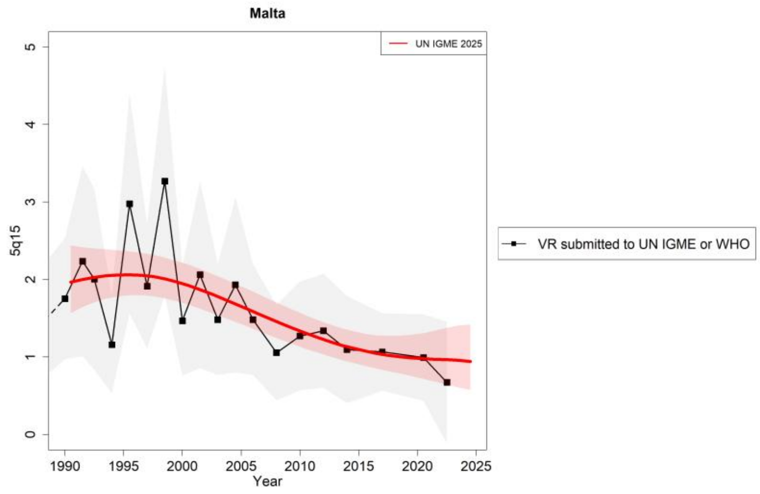
Malta
UN IGME 2025
VR submitted to UN IGME or WHO
5q15
0
1
2
3
4
5
1990
1995
2000
2005
2010
2015
2020
2025
Year

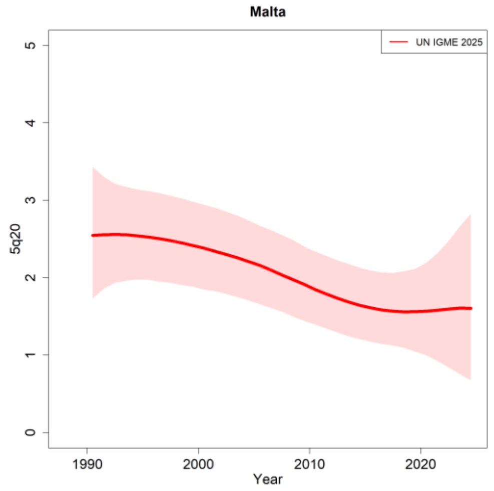
Malta
UN IGME 2025
5q20
0
1
2
3
4
5
1990
2000
2010
2020
Year

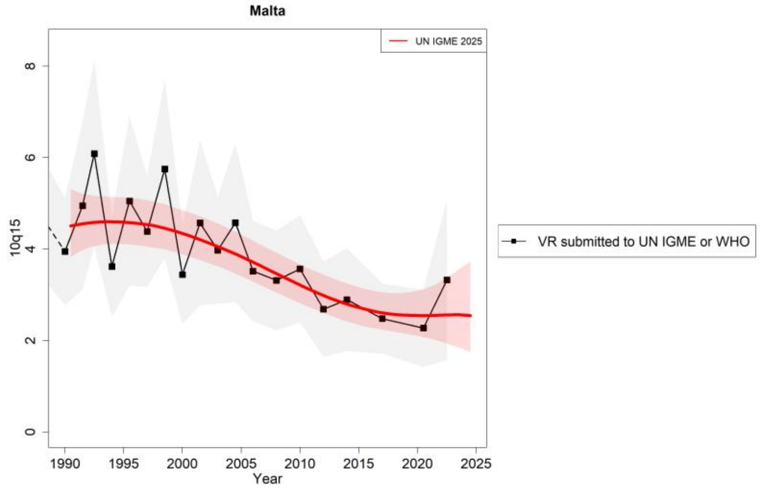
Malta
UN IGME 2025
VR submitted to UN IGME or WHO
10q15
0
2
4
6
8
1990
1995
2000
2005
2010
2015
2020
2025
Year

Marshall Islands (MHL)

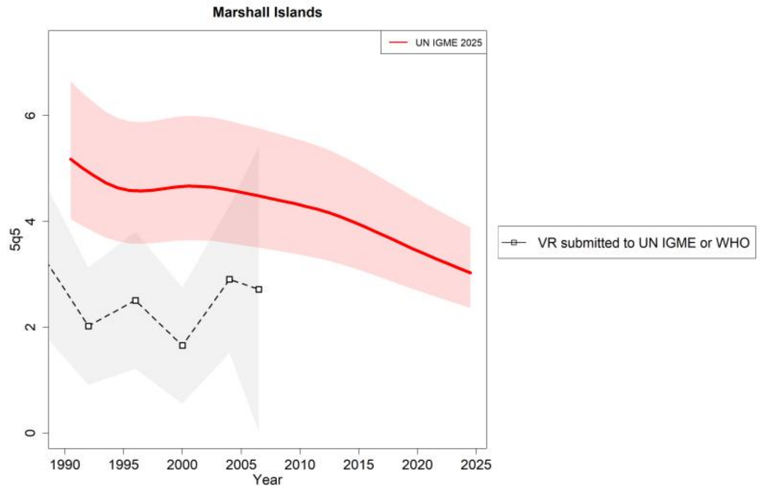

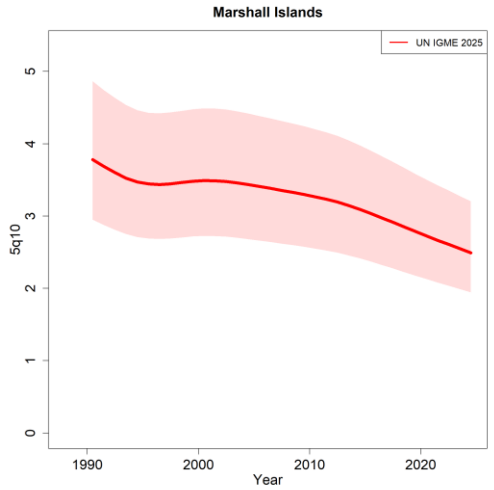

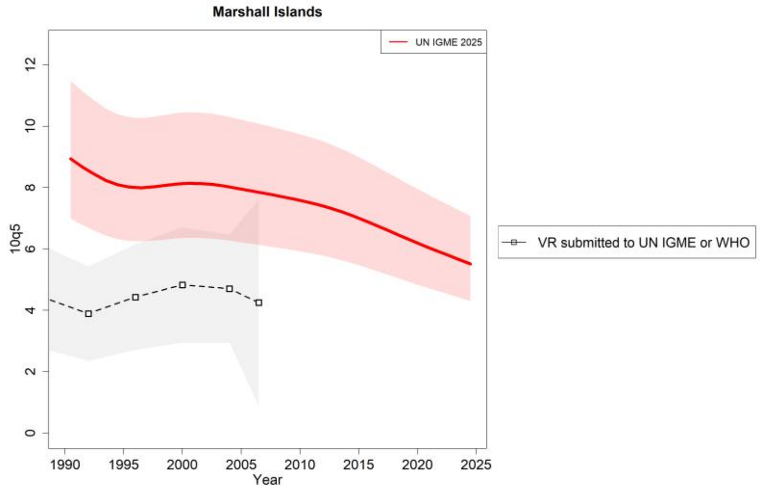

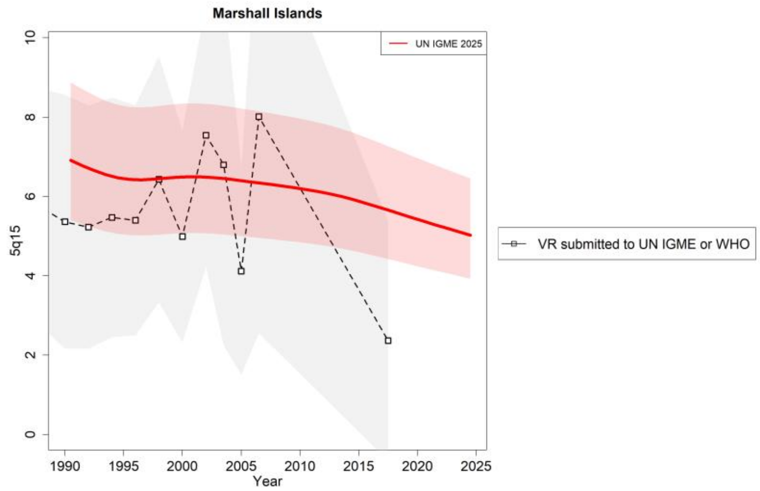
Marshall Islands
UN IGME 2025
VR submitted to UN IGME or WHO
5q15
Year
0
2
4
6
8
10
1990
1995
2000
2005
2010
2015
2020
2025

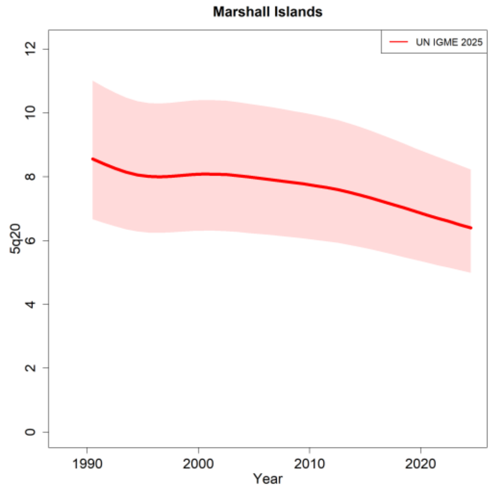
Marshall Islands
UN IGME 2025
5q20
Year
0
2
4
6
8
10
12
1990
2000
2010
2020

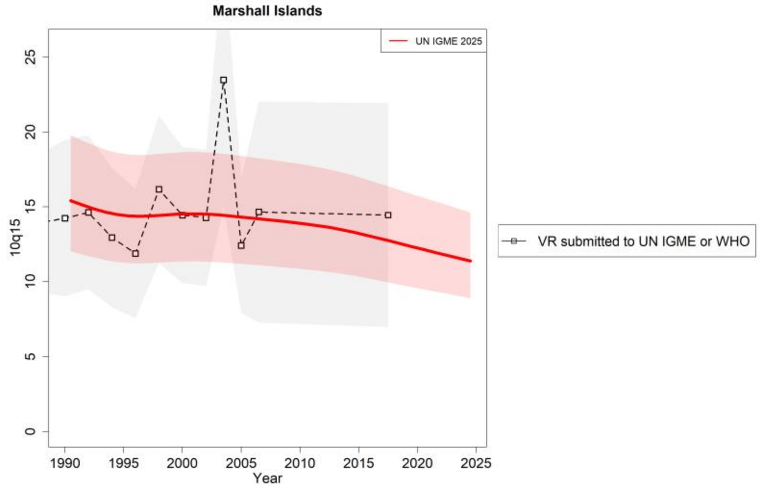
Marshall Islands
UN IGME 2025
VR submitted to UN IGME or WHO
10q15
Year
0
5
10
15
20
25
1990
1995
2000
2005
2010
2015
2020
2025

Mauritania (MRT)

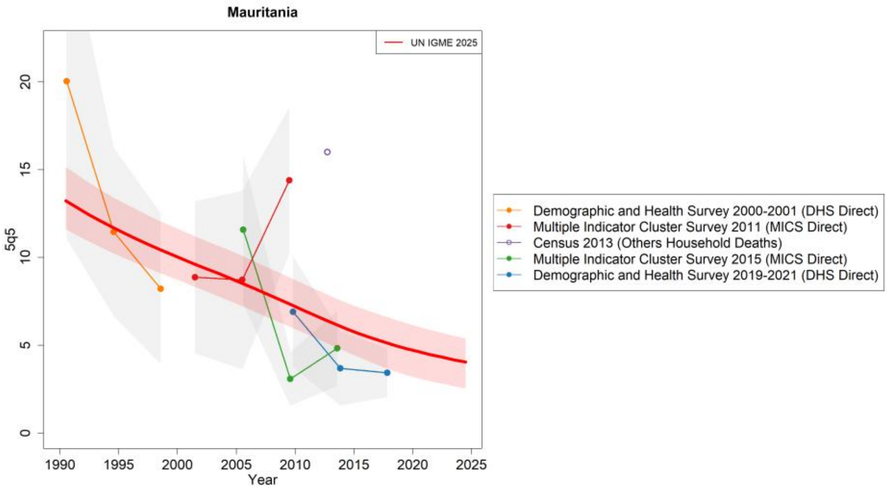

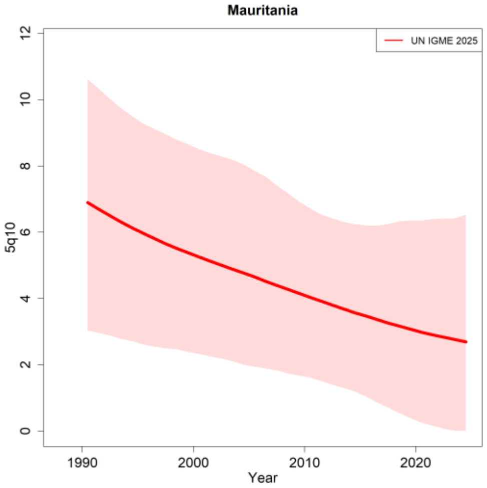

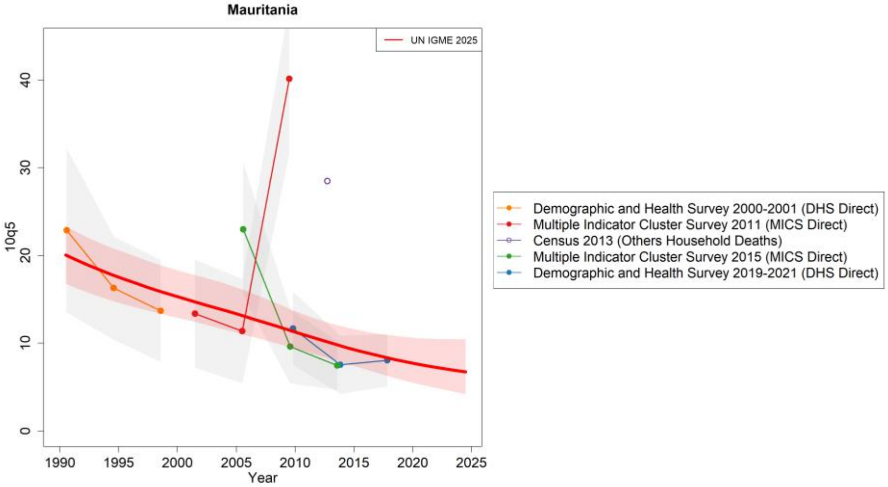

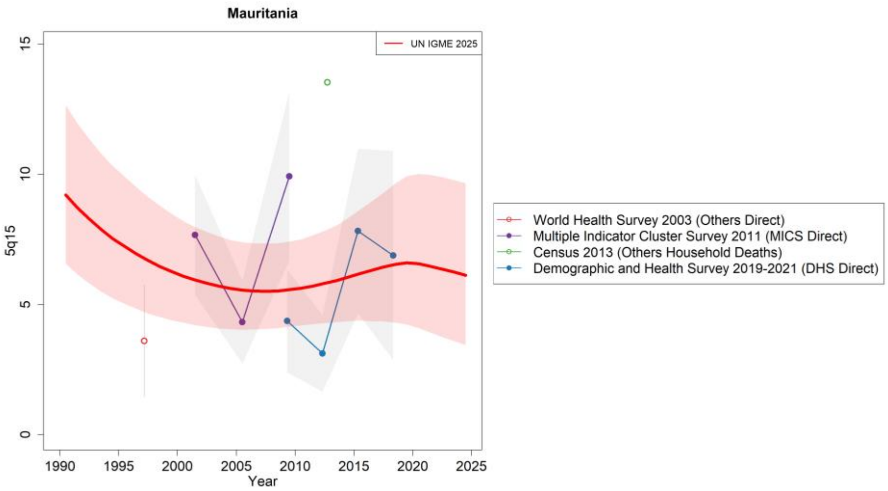
Mauritania
UN IGME 2025
5q15
Year
World Health Survey 2003 (Others Direct)
Multiple Indicator Cluster Survey 2011 (MICS Direct)
Census 2013 (Others Household Deaths)
Demographic and Health Survey 2019-2021 (DHS Direct)

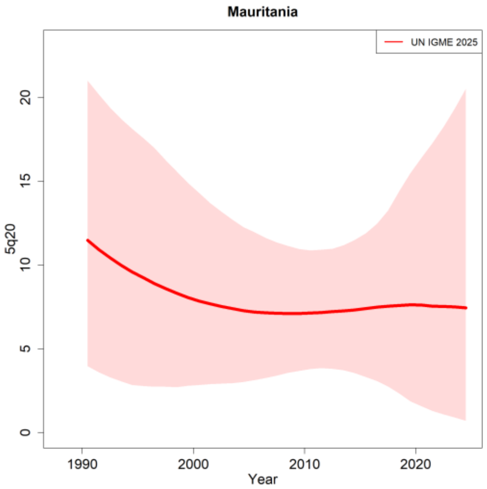
Mauritania
UN IGME 2025
5q20
Year

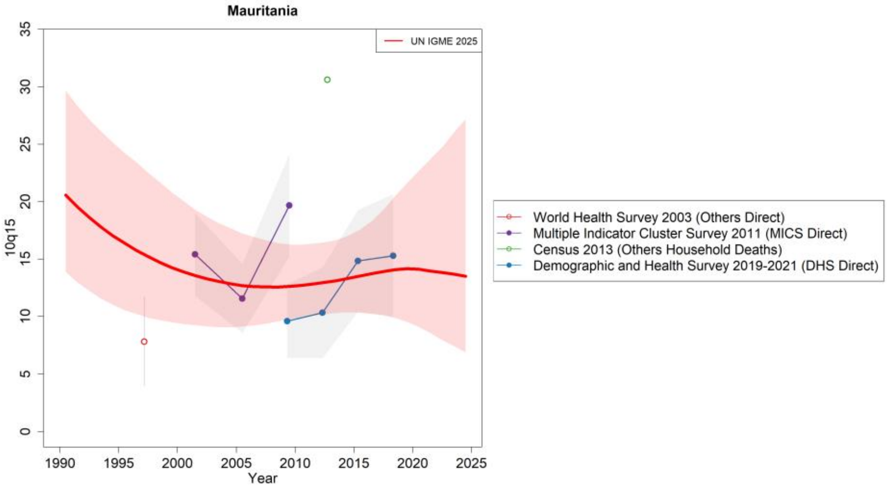
Mauritania
UN IGME 2025
10q15
Year
World Health Survey 2003 (Others Direct)
Multiple Indicator Cluster Survey 2011 (MICS Direct)
Census 2013 (Others Household Deaths)
Demographic and Health Survey 2019-2021 (DHS Direct)

Mauritius (MUS)

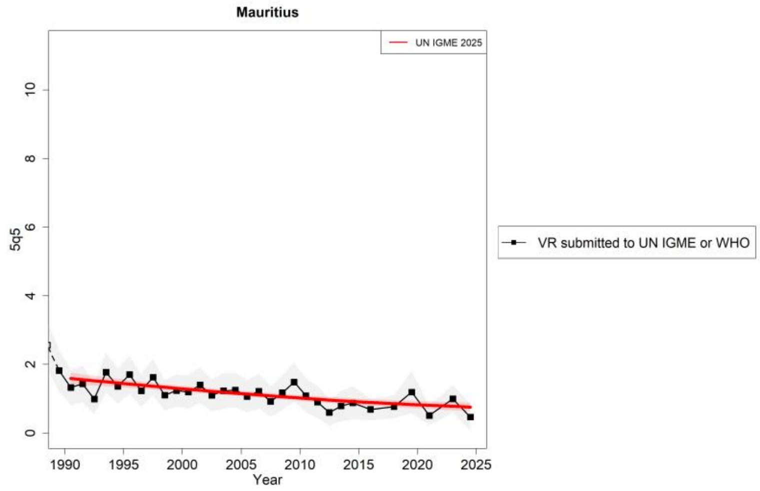

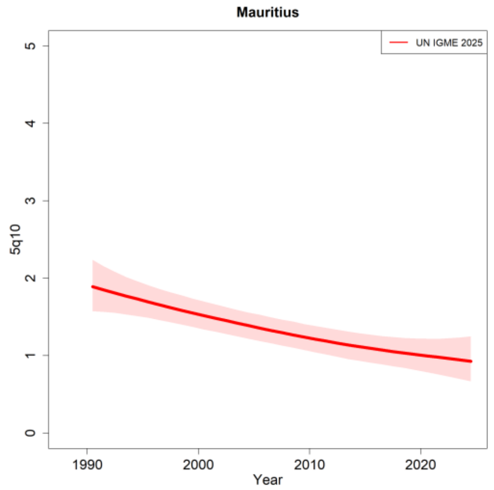

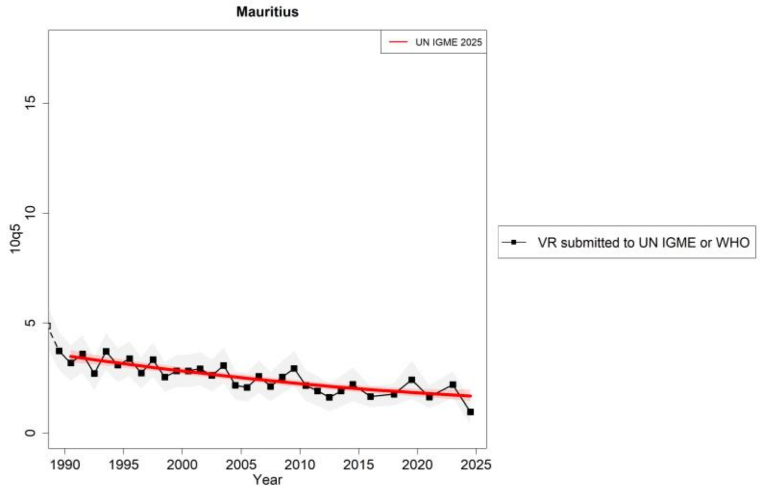

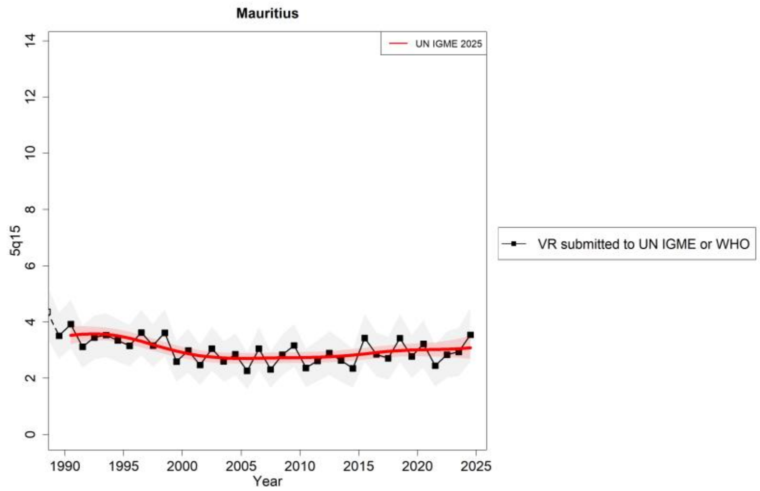
Mauritius
UN IGME 2025
VR submitted to UN IGME or WHO
5q15
0
2
4
6
8
10
12
14
1990
1995
2000
2005
2010
2015
2020
2025
Year

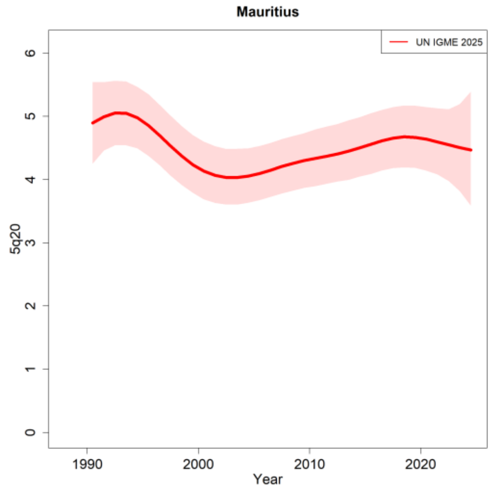
Mauritius
UN IGME 2025
5q20
0
1
2
3
4
5
6
1990
2000
2010
2020
Year

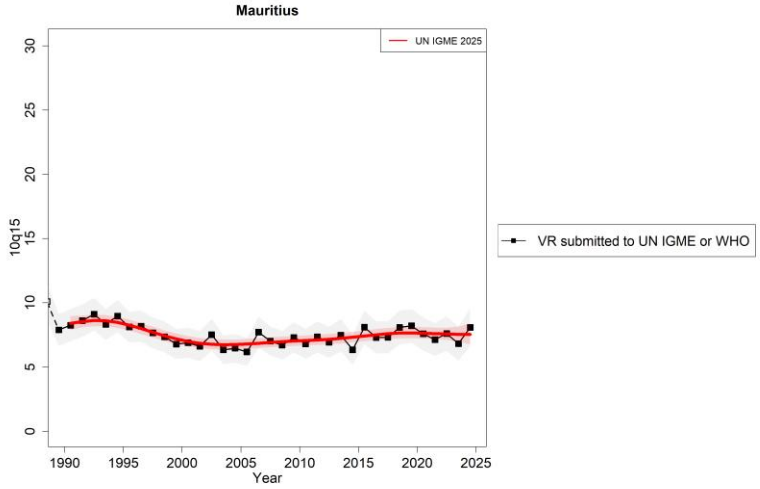
Mauritius
UN IGME 2025
VR submitted to UN IGME or WHO
10q15
0
5
10
15
20
25
30
1990
1995
2000
2005
2010
2015
2020
2025
Year

Mexico (MEX)

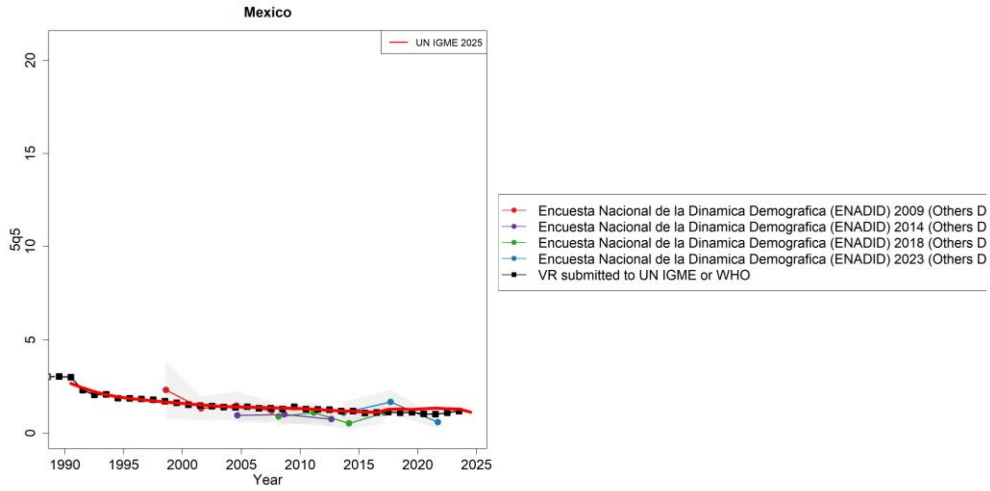
Mexico
UN IGME 2025
Encuesta Nacional de la Dinamica Demografica (ENADID) 2009 (Others D
Encuesta Nacional de la Dinamica Demografica (ENADID) 2014 (Others D
Encuesta Nacional de la Dinamica Demografica (ENADID) 2018 (Others D
Encuesta Nacional de la Dinamica Demografica (ENADID) 2023 (Others D
VR submitted to UN IGME or WHO
5q5
Year

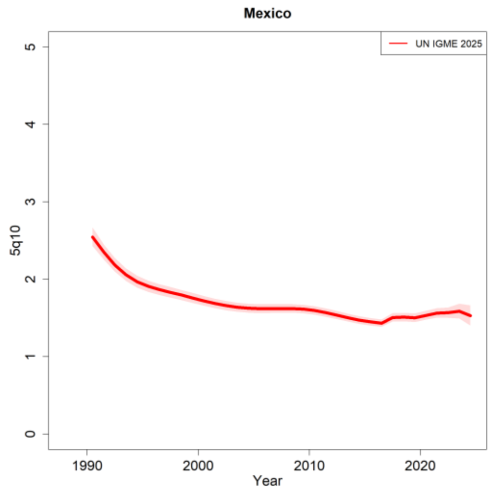
Mexico
UN IGME 2025
5q10
Year

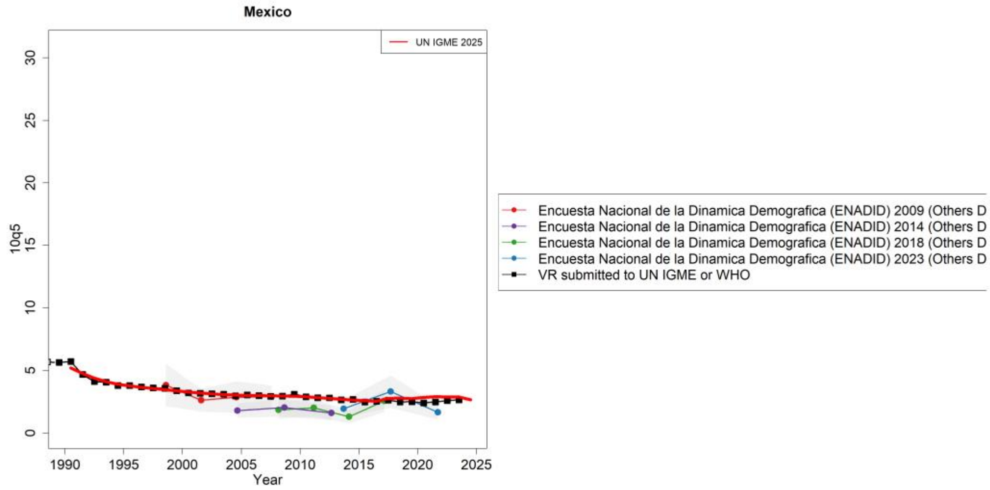
Mexico
UN IGME 2025
Encuesta Nacional de la Dinamica Demografica (ENADID) 2009 (Others D
Encuesta Nacional de la Dinamica Demografica (ENADID) 2014 (Others D
Encuesta Nacional de la Dinamica Demografica (ENADID) 2018 (Others D
Encuesta Nacional de la Dinamica Demografica (ENADID) 2023 (Others D
VR submitted to UN IGME or WHO
10q5
Year

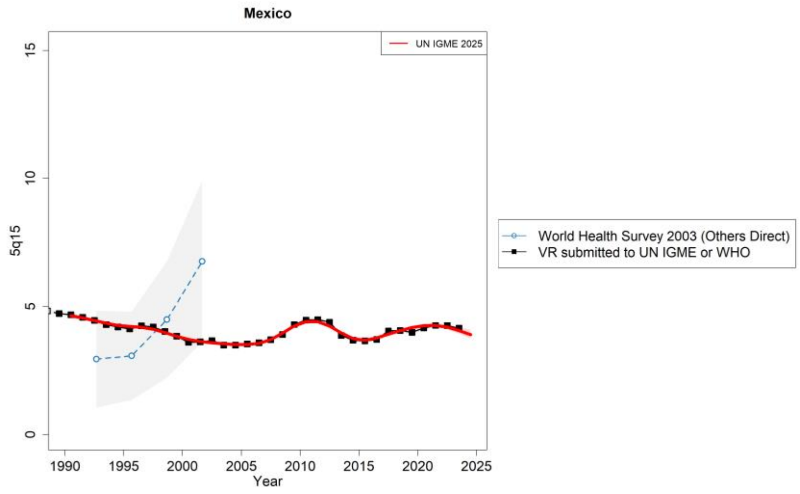
Mexico
UN IGME 2025
World Health Survey 2003 (Others Direct)
VR submitted to UN IGME or WHO
5q15
Year

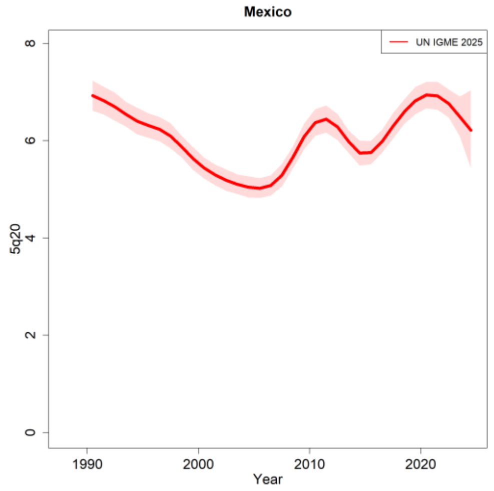
Mexico
UN IGME 2025
5q20
Year

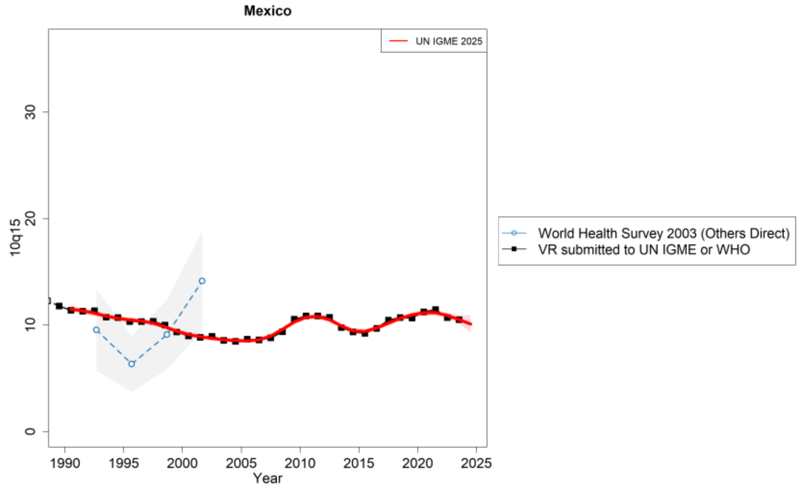
Mexico
UN IGME 2025
World Health Survey 2003 (Others Direct)
VR submitted to UN IGME or WHO
10q15
Year

Micronesia (Federated States of) (FSM)

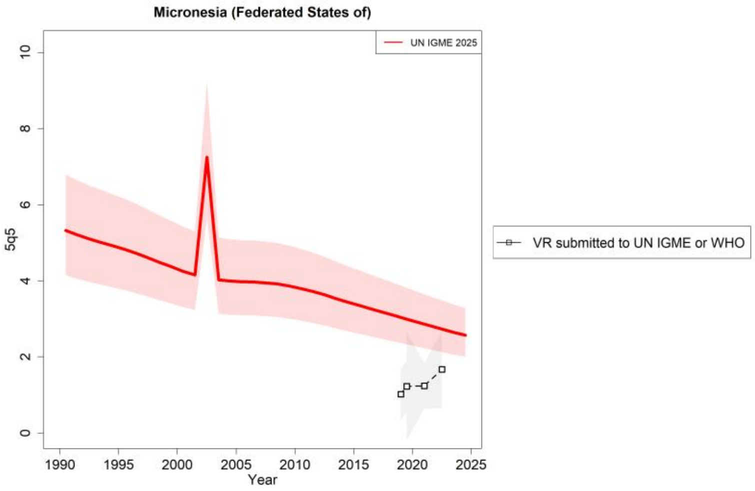

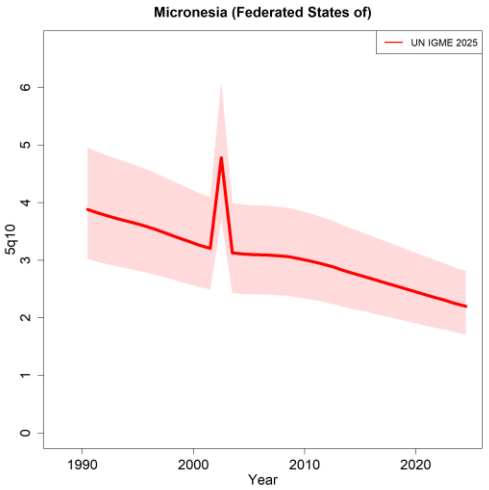

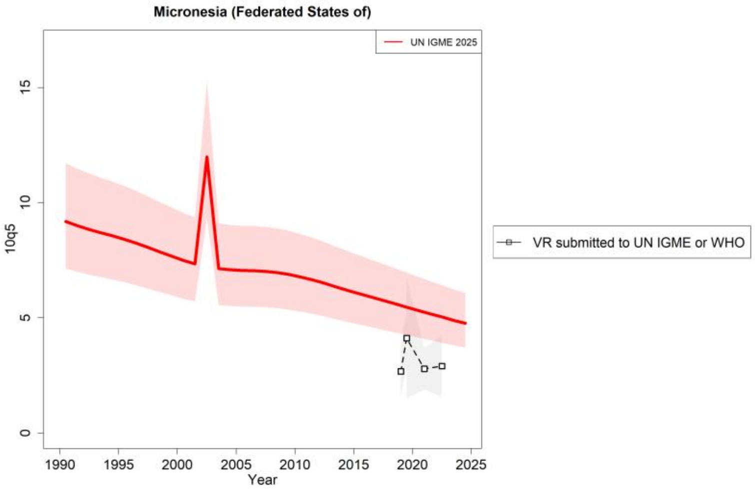

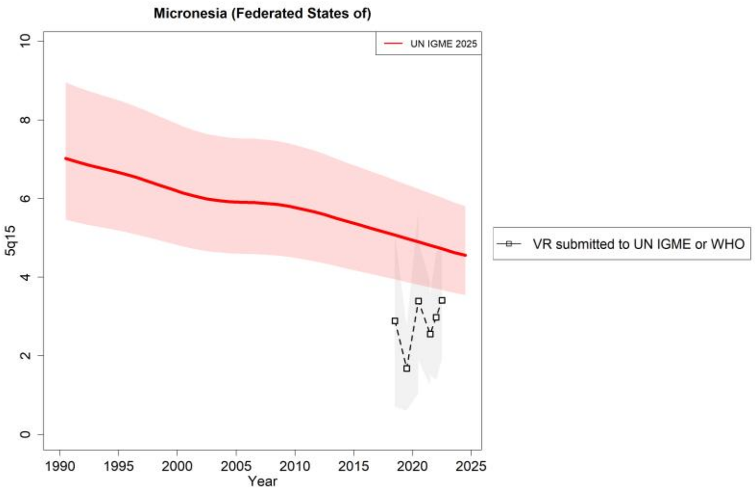
Micronesia (Federated States of)
UN IGME 2025
VR submitted to UN IGME or WHO
5q15
Year

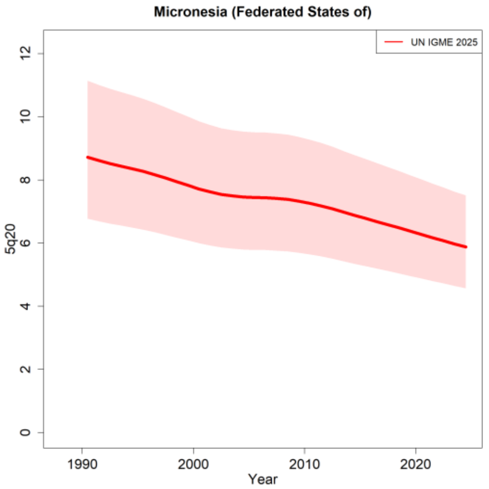
Micronesia (Federated States of)
UN IGME 2025
5q20
Year

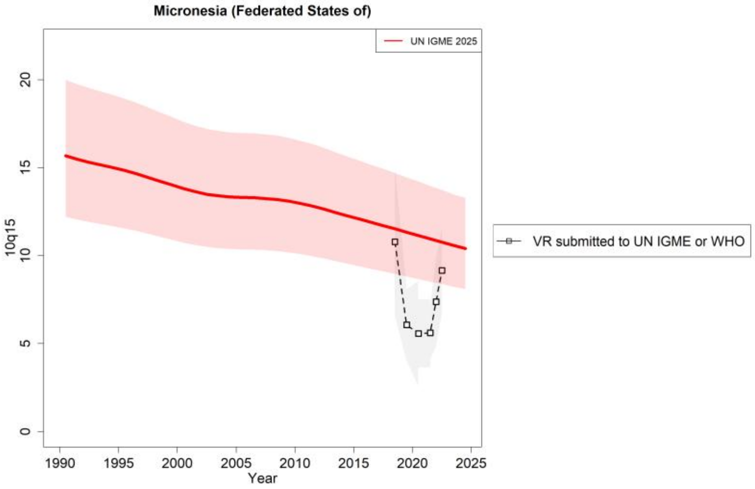
Micronesia (Federated States of)
UN IGME 2025
VR submitted to UN IGME or WHO
10q15
Year

Monaco (MCO)

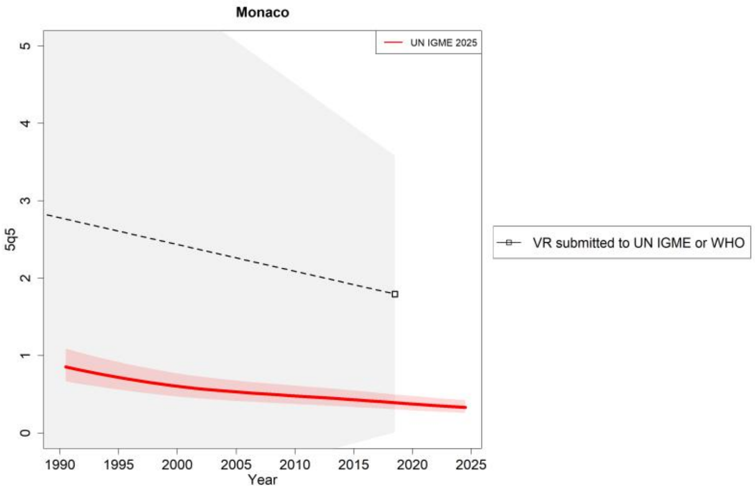
Monaco
UN IGME 2025
VR submitted to UN IGME or WHO
5q5
Year

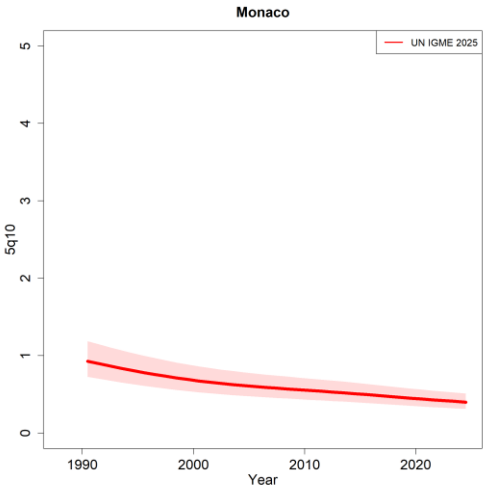
Monaco
UN IGME 2025
5q10
Year

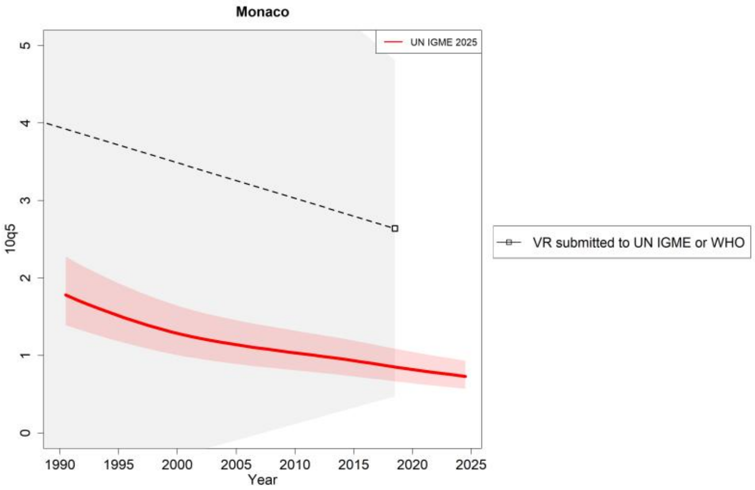
Monaco
UN IGME 2025
VR submitted to UN IGME or WHO
10q5
Year

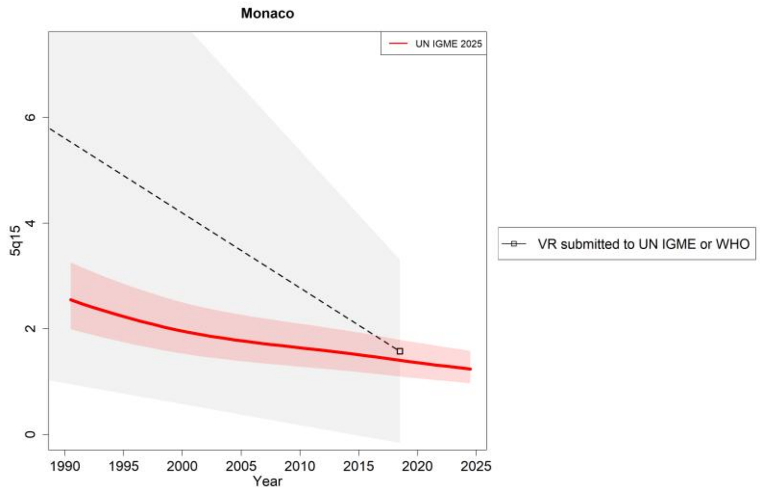
Monaco
UN IGME 2025
VR submitted to UN IGME or WHO
5q15
0
2
4
6
1990
1995
2000
2005
2010
2015
2020
2025
Year

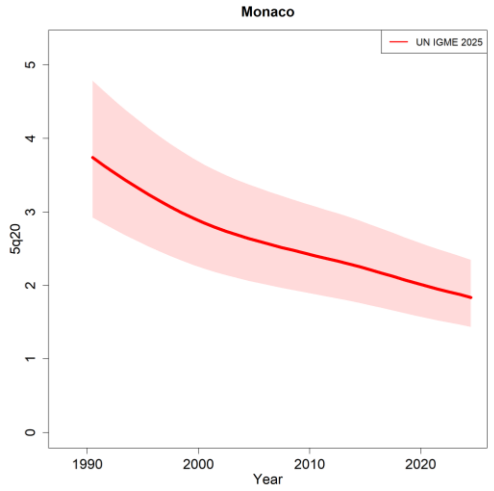
Monaco
UN IGME 2025
5q20
0
1
2
3
4
5
1990
2000
2010
2020
Year

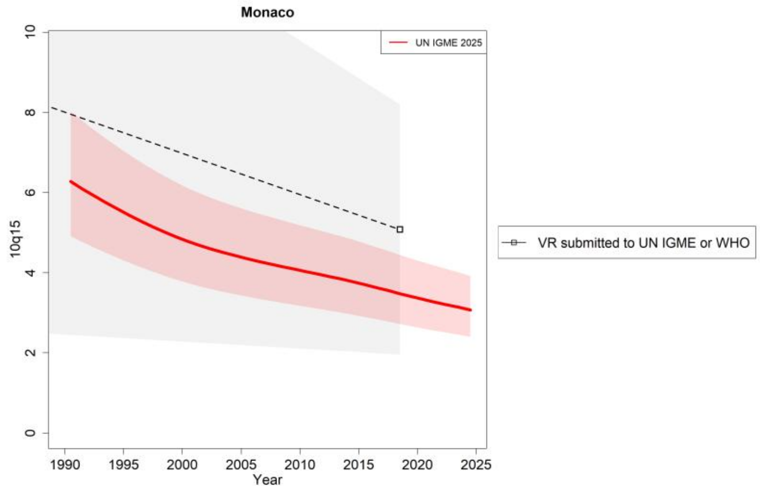
Monaco
UN IGME 2025
VR submitted to UN IGME or WHO
10q15
0
2
4
6
8
10
1990
1995
2000
2005
2010
2015
2020
2025
Year

Mongolia (MNG)

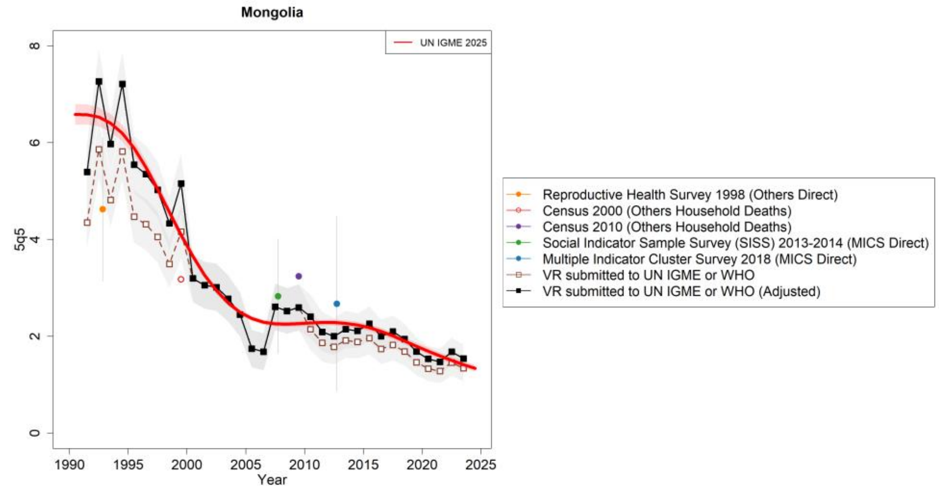

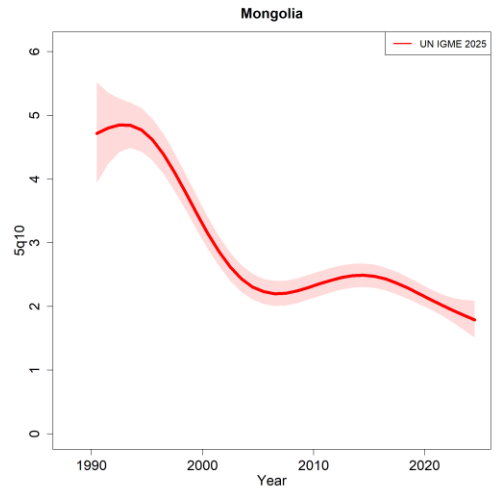

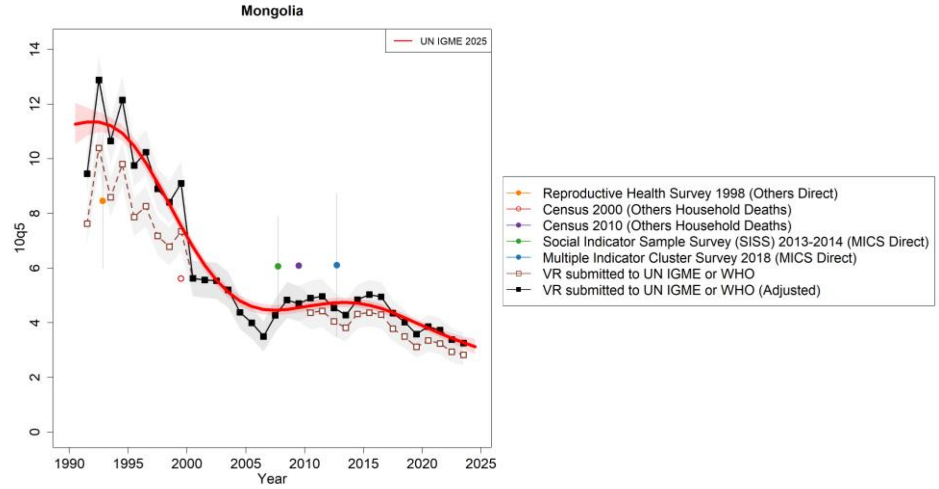

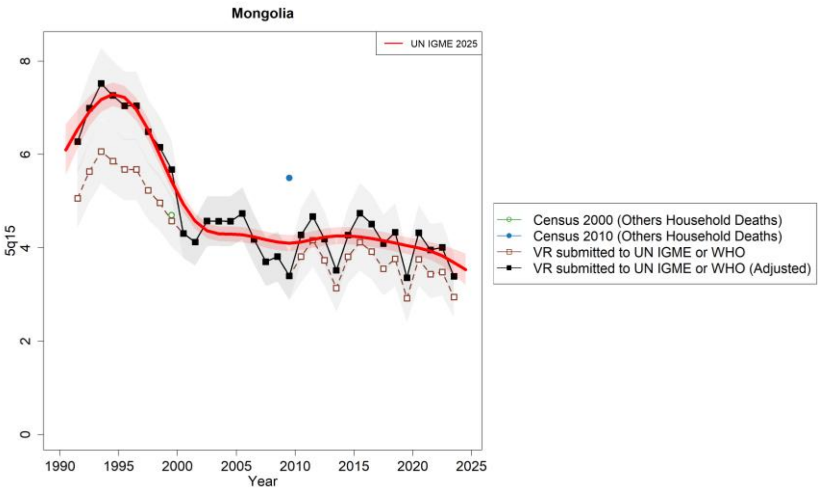
Mongolia
UN IGME 2025
5q15
Year
Census 2000 (Others Household Deaths)
Census 2010 (Others Household Deaths)
VR submitted to UN IGME or WHO
VR submitted to UN IGME or WHO (Adjusted)

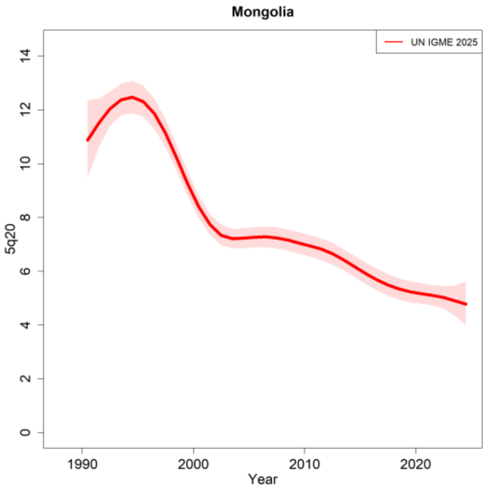
Mongolia
UN IGME 2025
5q20
Year

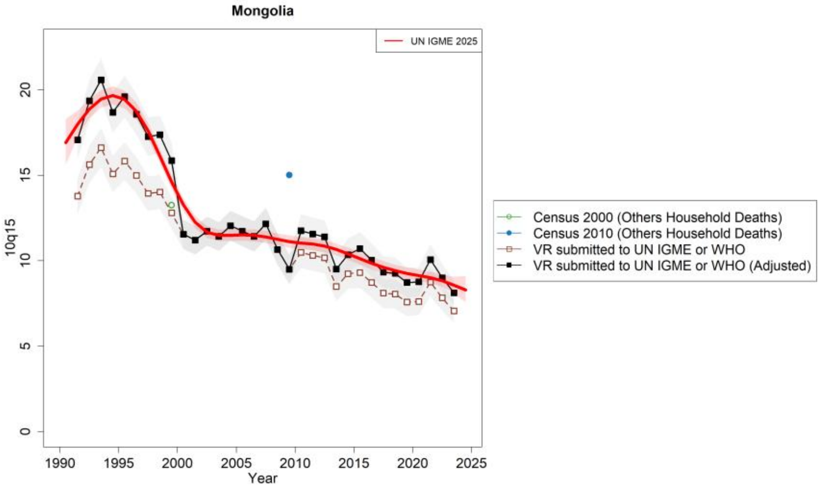
Mongolia
UN IGME 2025
10q15
Year
Census 2000 (Others Household Deaths)
Census 2010 (Others Household Deaths)
VR submitted to UN IGME or WHO
VR submitted to UN IGME or WHO (Adjusted)

Montenegro (MNE)

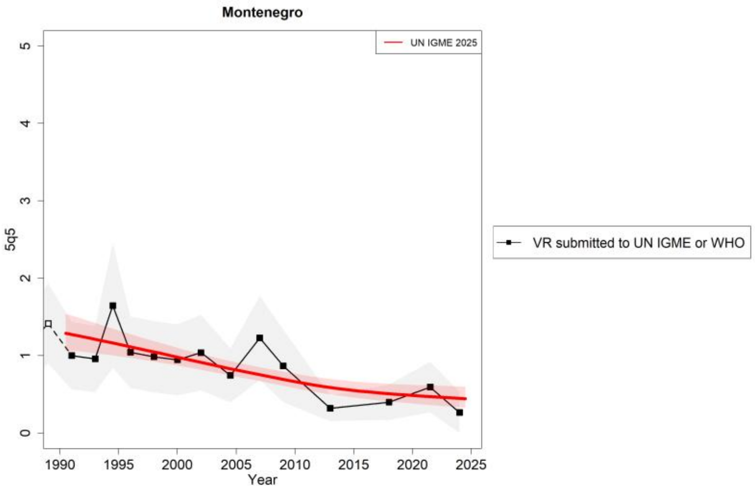

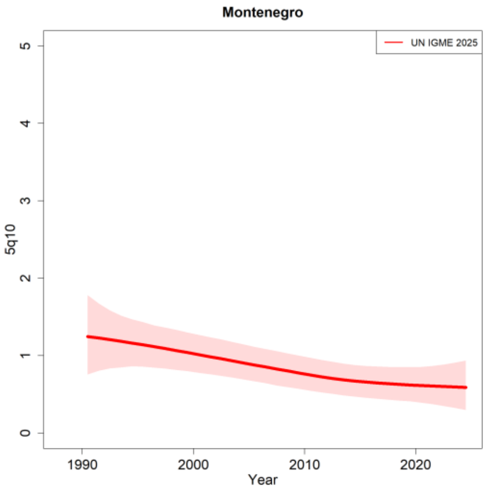

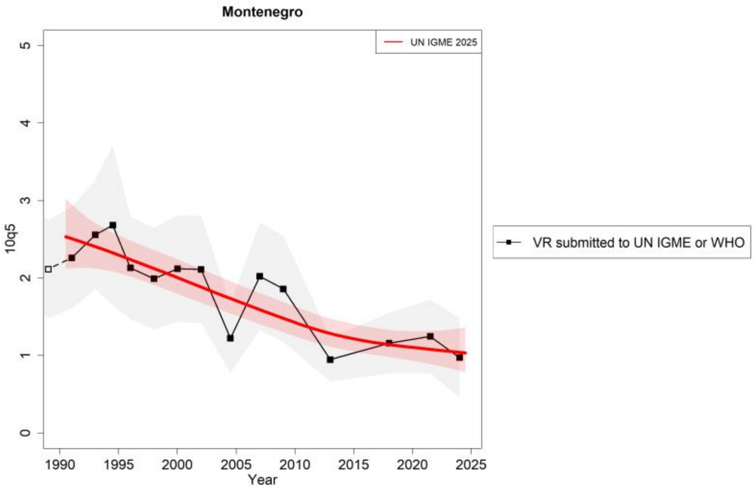

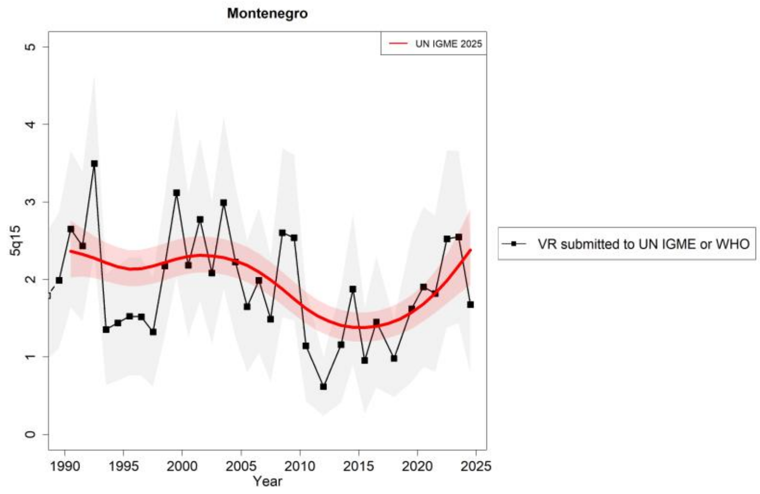
Montenegro
UN IGME 2025
VR submitted to UN IGME or WHO
5q15
Year
0
1
2
3
4
5
1990
1995
2000
2005
2010
2015
2020
2025

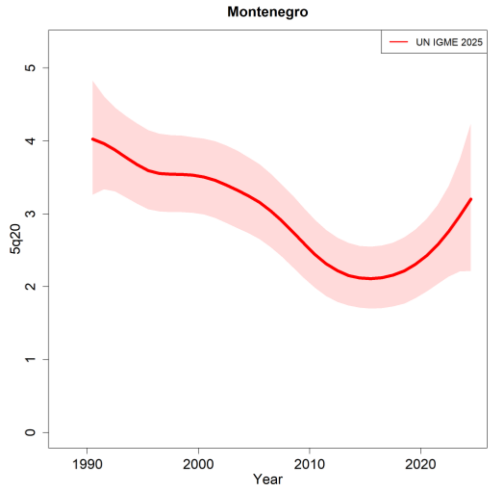
Montenegro
UN IGME 2025
5q20
Year
0
1
2
3
4
5
1990
2000
2010
2020

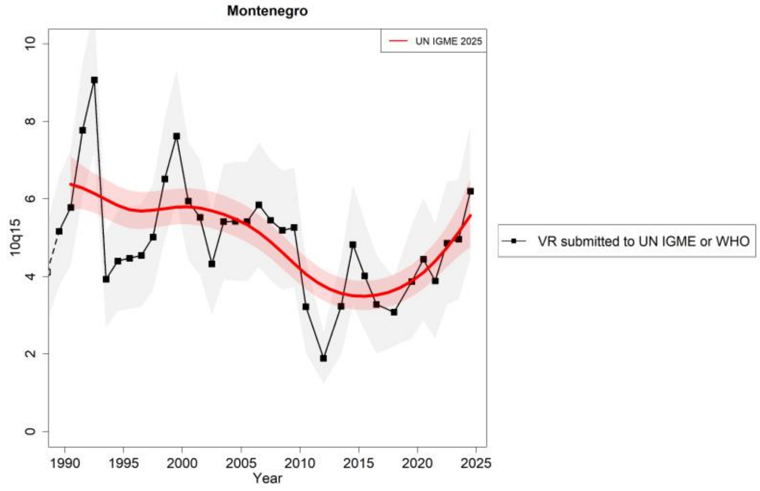
Montenegro
UN IGME 2025
VR submitted to UN IGME or WHO
10q15
Year
0
2
4
6
8
10
1990
1995
2000
2005
2010
2015
2020
2025

Montserrat (MSR)

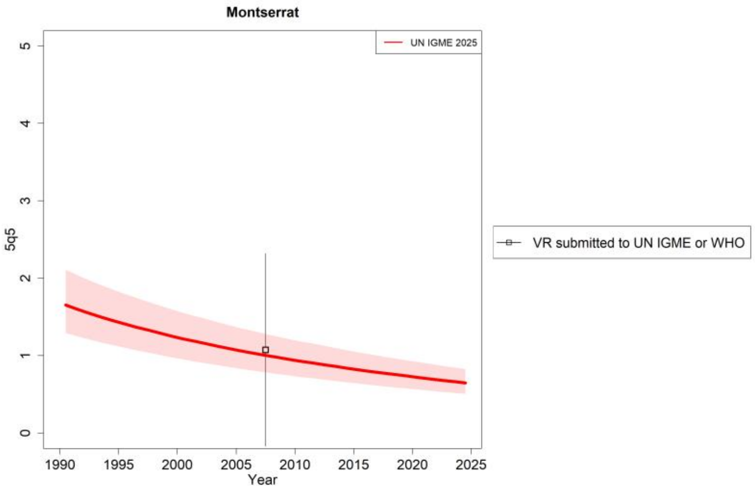

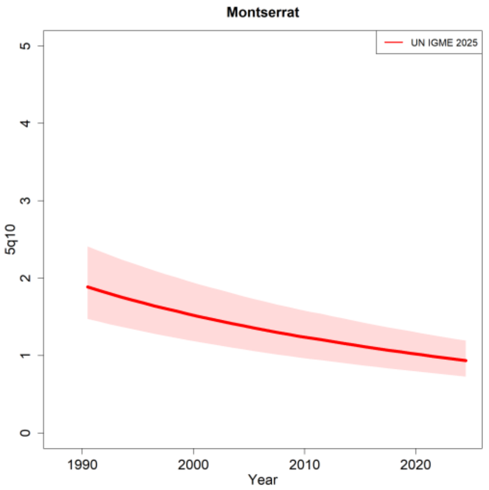

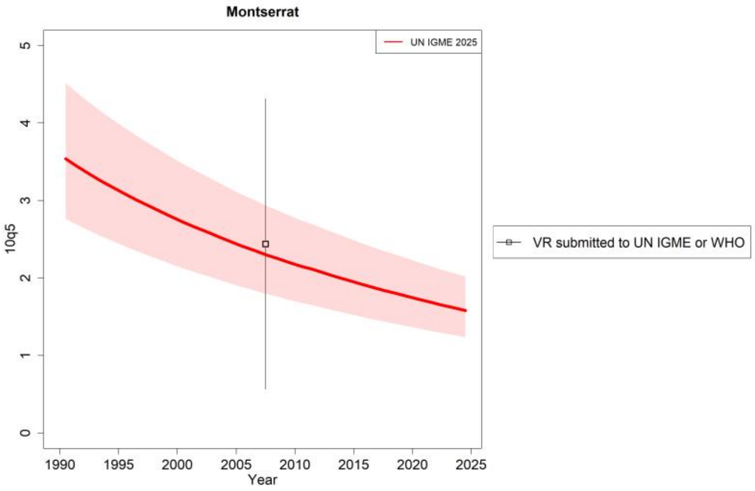

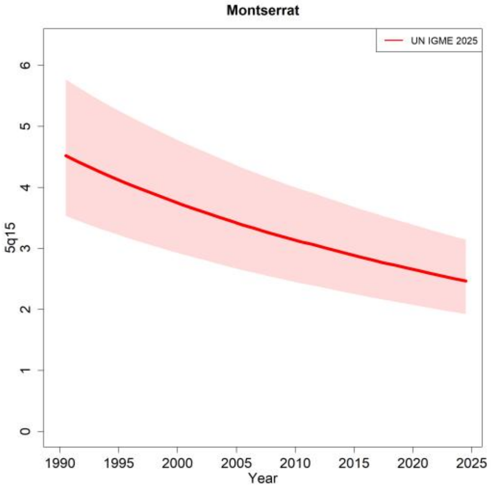
Montserrat
UN IGME 2025
5q15
Year
0
1
2
3
4
5
6
1990
1995
2000
2005
2010
2015
2020
2025

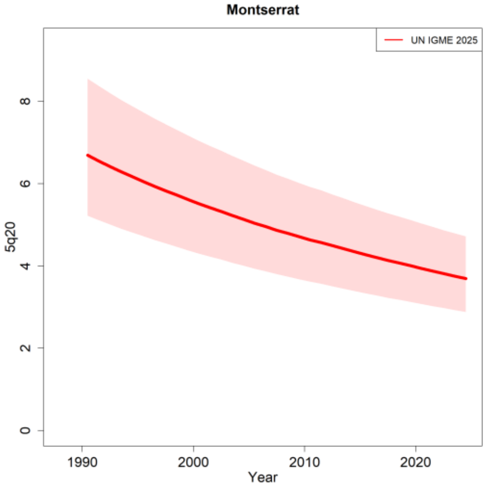
Montserrat
UN IGME 2025
5q20
Year
0
2
4
6
8
1990
2000
2010
2020

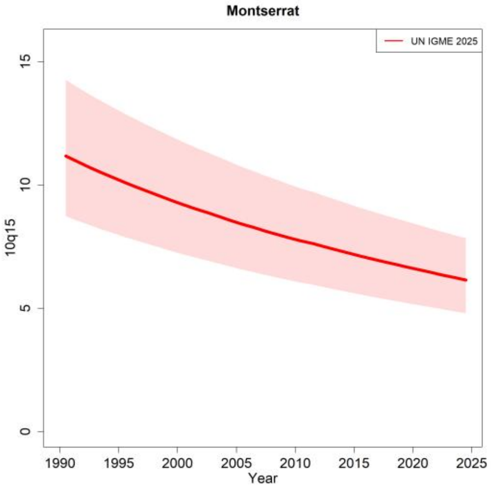
Montserrat
UN IGME 2025
10q15
Year
0
5
10
15
1990
1995
2000
2005
2010
2015
2020
2025

Morocco (MAR)

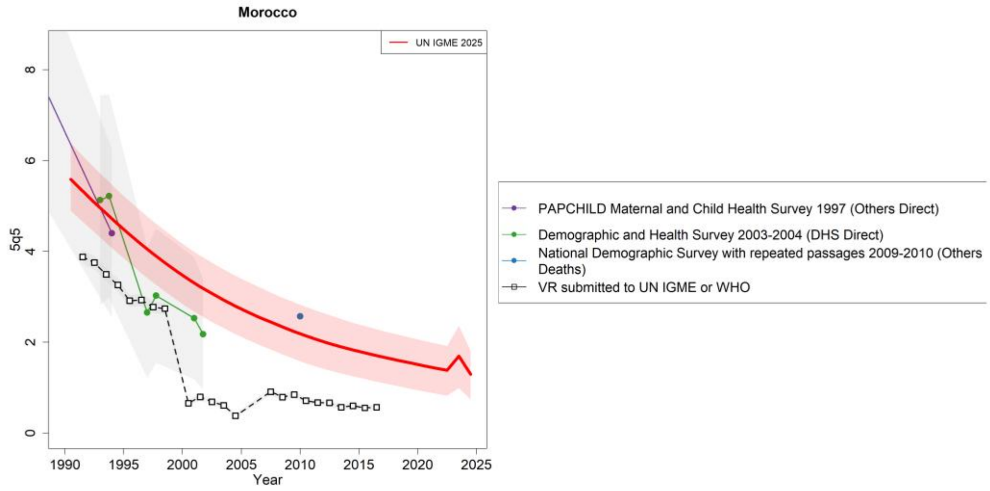
Morocco
UN IGME 2025
5q5
Year
PAPCHILD Maternal and Child Health Survey 1997 (Others Direct)
Demographic and Health Survey 2003-2004 (DHS Direct)
National Demographic Survey with repeated passages 2009-2010 (Others Deaths)
VR submitted to UN IGME or WHO

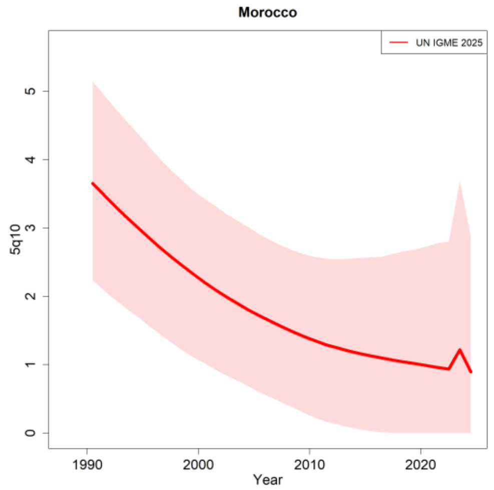
Morocco
UN IGME 2025
5q10
Year

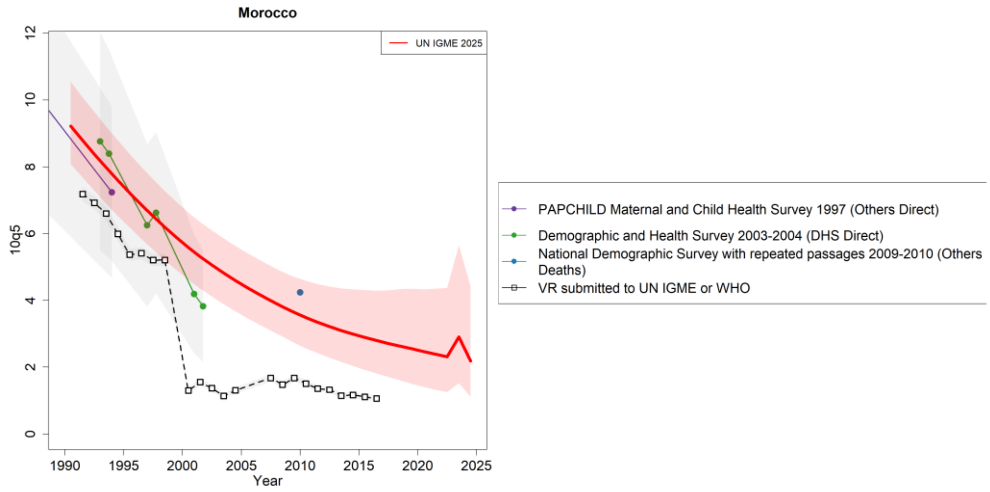
Morocco
UN IGME 2025
10q5
Year
PAPCHILD Maternal and Child Health Survey 1997 (Others Direct)
Demographic and Health Survey 2003-2004 (DHS Direct)
National Demographic Survey with repeated passages 2009-2010 (Others Deaths)
VR submitted to UN IGME or WHO

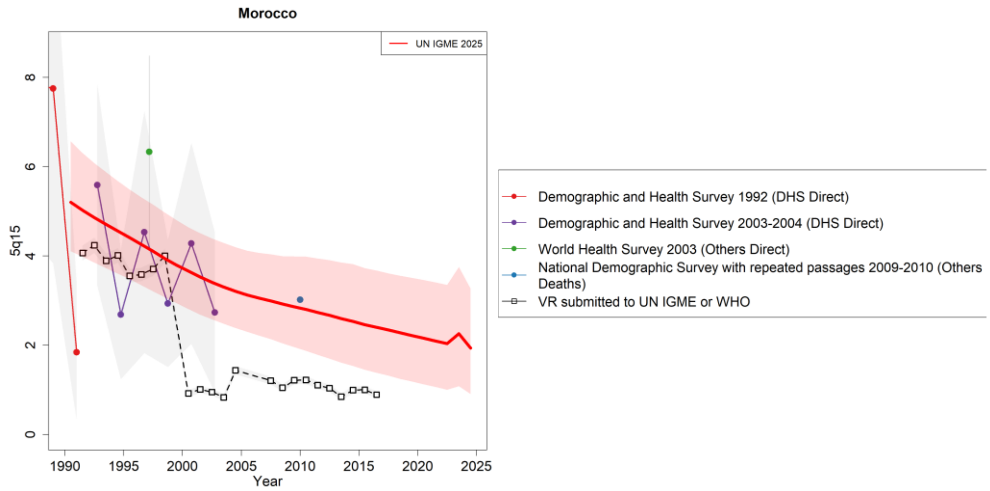
Morocco
UN IGME 2025
5q15
Year
Demographic and Health Survey 1992 (DHS Direct)
Demographic and Health Survey 2003-2004 (DHS Direct)
World Health Survey 2003 (Others Direct)
National Demographic Survey with repeated passages 2009-2010 (Others Deaths)
VR submitted to UN IGME or WHO

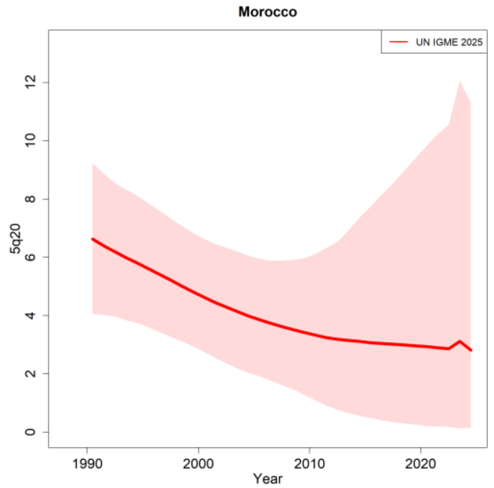
Morocco
UN IGME 2025
5q20
Year

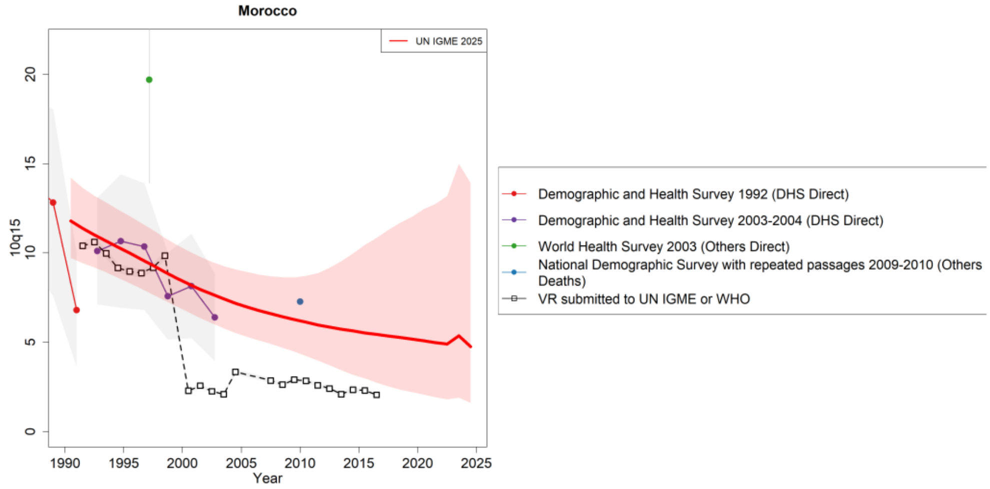
Morocco
UN IGME 2025
10q15
Year
Demographic and Health Survey 1992 (DHS Direct)
Demographic and Health Survey 2003-2004 (DHS Direct)
World Health Survey 2003 (Others Direct)
National Demographic Survey with repeated passages 2009-2010 (Others Deaths)
VR submitted to UN IGME or WHO

Mozambique (MOZ)

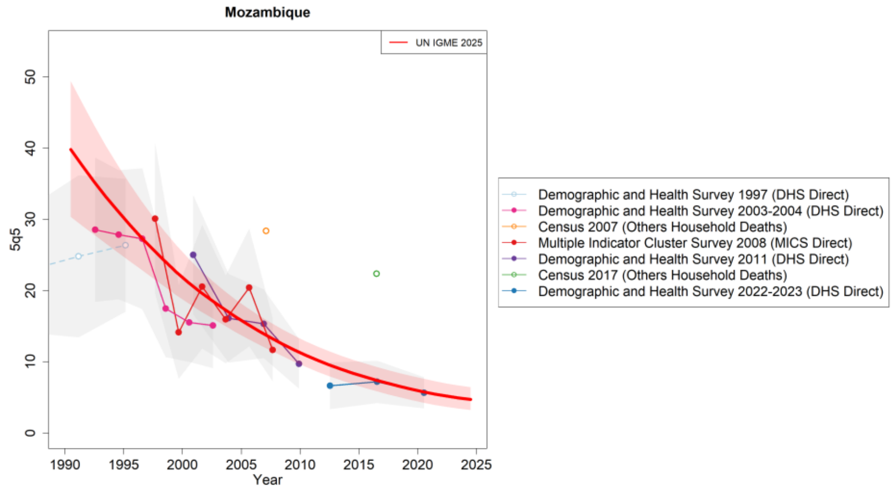

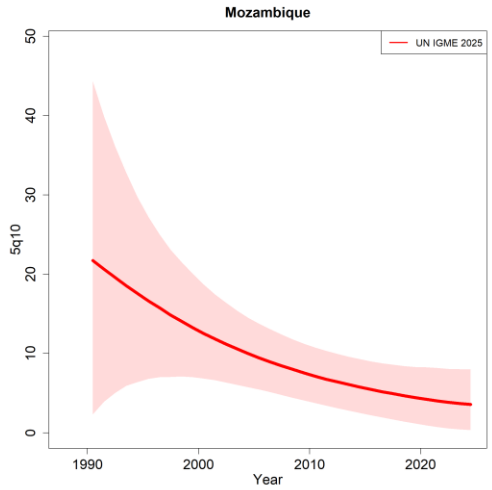

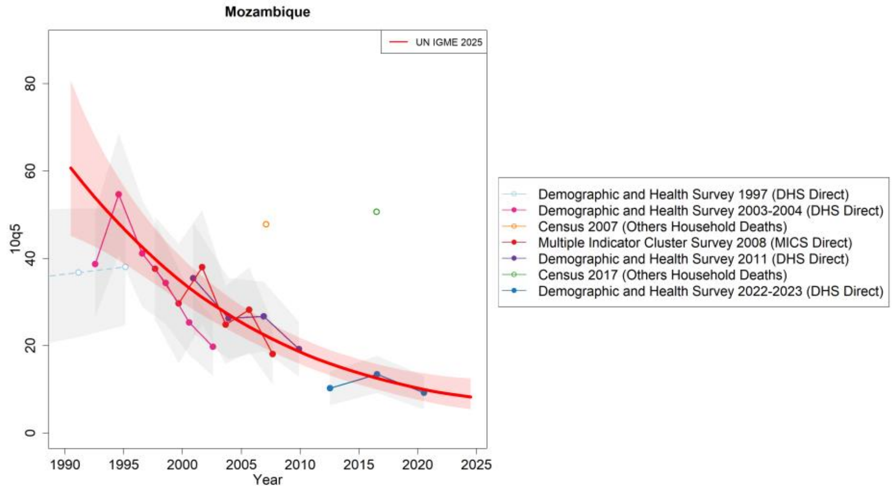

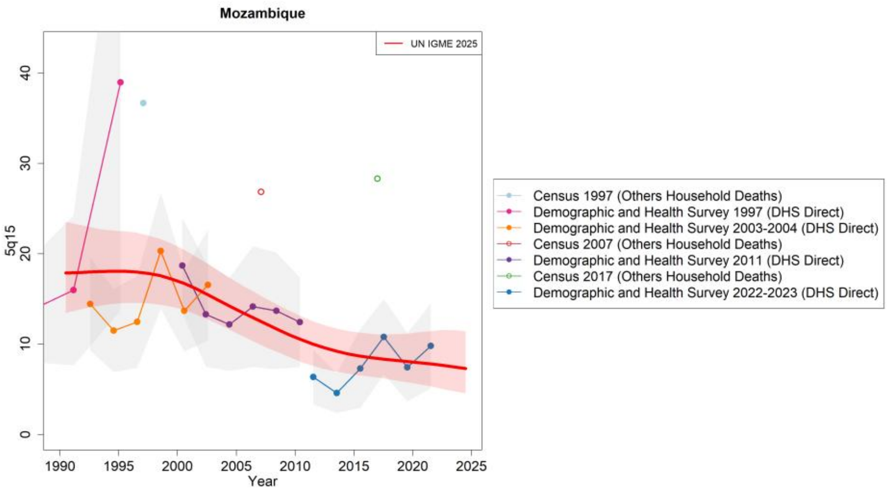
Mozambique
UN IGME 2025
5q15
Year
Census 1997 (Others Household Deaths)
Demographic and Health Survey 1997 (DHS Direct)
Demographic and Health Survey 2003-2004 (DHS Direct)
Census 2007 (Others Household Deaths)
Demographic and Health Survey 2011 (DHS Direct)
Census 2017 (Others Household Deaths)
Demographic and Health Survey 2022-2023 (DHS Direct)

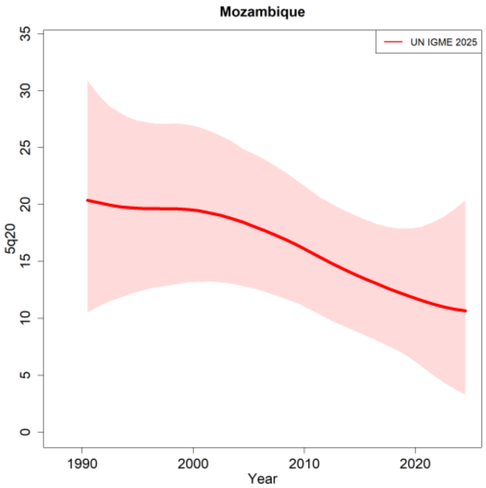
Mozambique
UN IGME 2025
5q20
Year

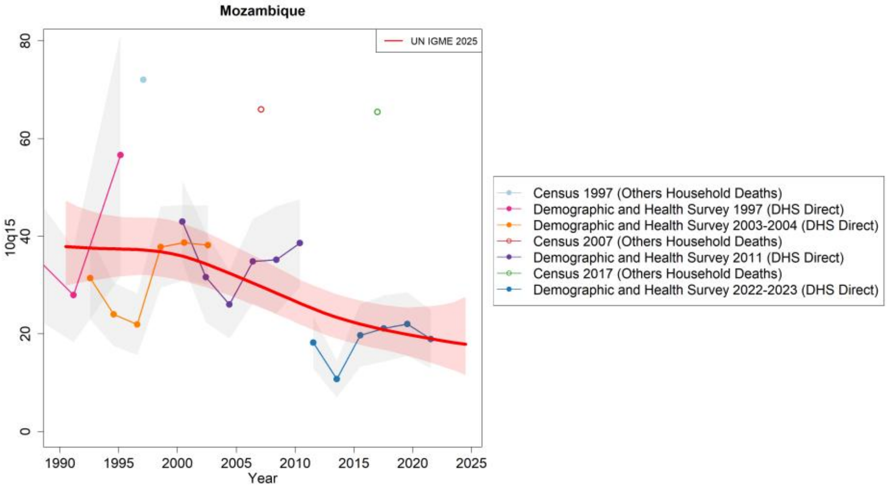
Mozambique
UN IGME 2025
10q15
Year
Census 1997 (Others Household Deaths)
Demographic and Health Survey 1997 (DHS Direct)
Demographic and Health Survey 2003-2004 (DHS Direct)
Census 2007 (Others Household Deaths)
Demographic and Health Survey 2011 (DHS Direct)
Census 2017 (Others Household Deaths)
Demographic and Health Survey 2022-2023 (DHS Direct)

Myanmar (MMR)

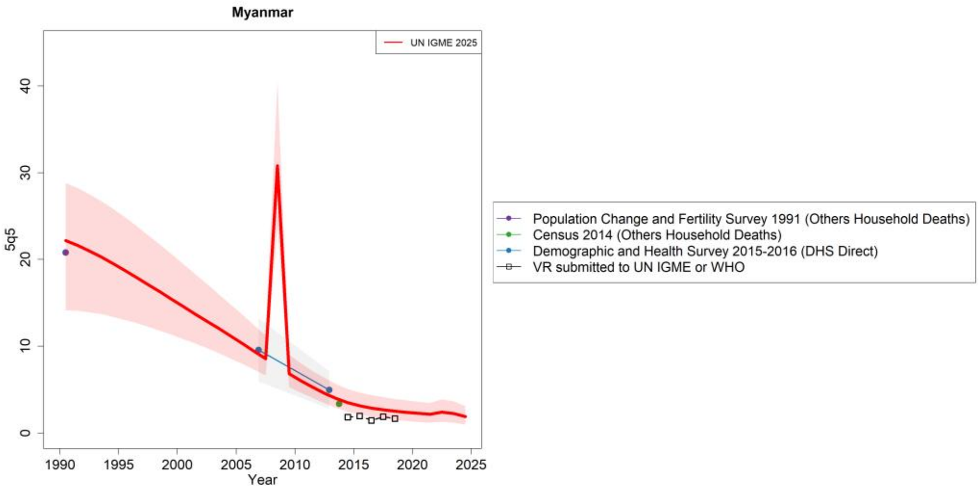
Myanmar
UN IGME 2025
5q5
Year
Population Change and Fertility Survey 1991 (Others Household Deaths)
Census 2014 (Others Household Deaths)
Demographic and Health Survey 2015-2016 (DHS Direct)
VR submitted to UN IGME or WHO

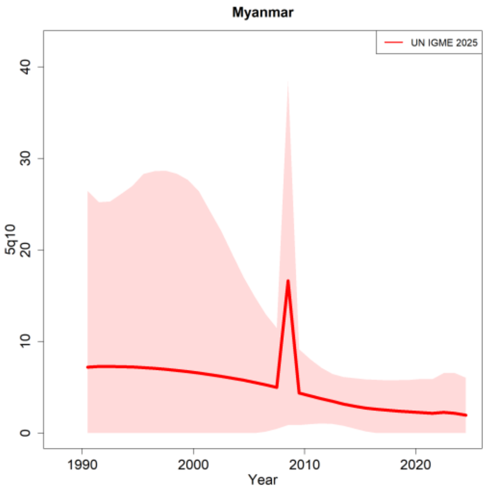
Myanmar
UN IGME 2025
5q10
Year

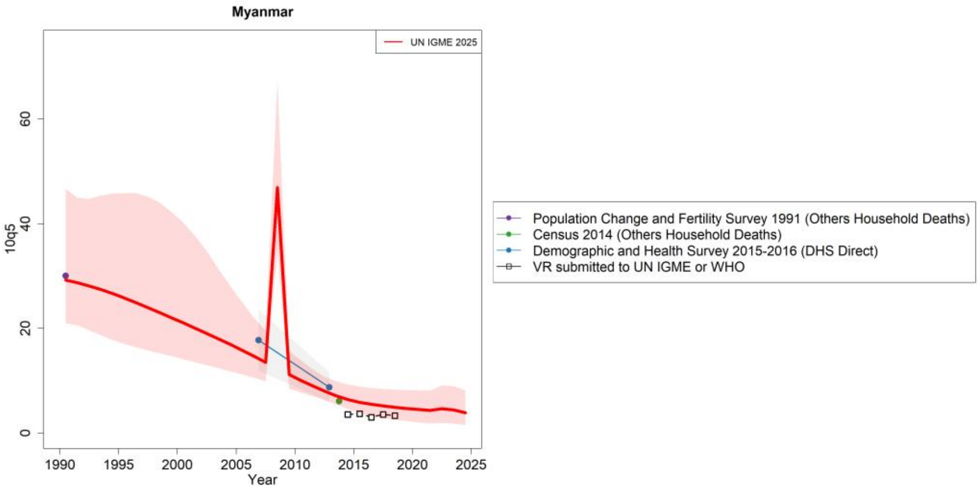
Myanmar
UN IGME 2025
10q5
Year
Population Change and Fertility Survey 1991 (Others Household Deaths)
Census 2014 (Others Household Deaths)
Demographic and Health Survey 2015-2016 (DHS Direct)
VR submitted to UN IGME or WHO

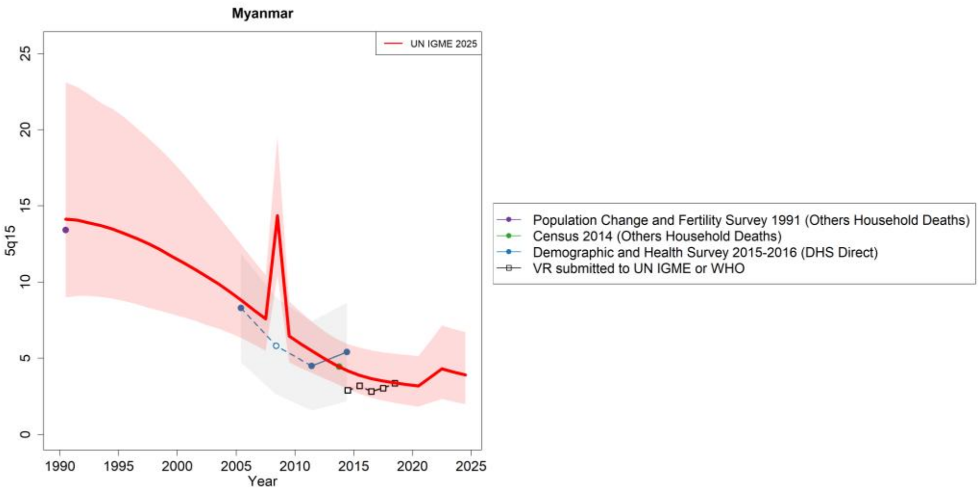
Myanmar
UN IGME 2025
5q15
Year
Population Change and Fertility Survey 1991 (Others Household Deaths)
Census 2014 (Others Household Deaths)
Demographic and Health Survey 2015-2016 (DHS Direct)
VR submitted to UN IGME or WHO

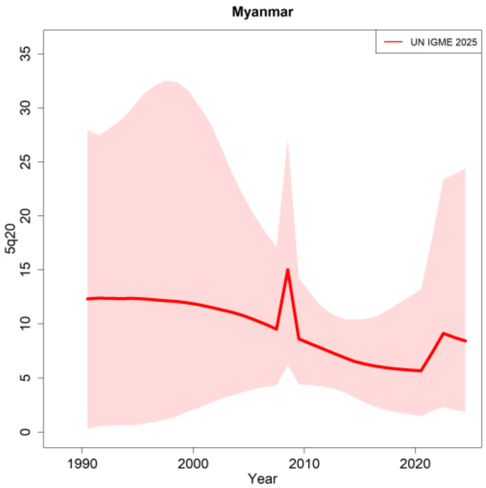
Myanmar
UN IGME 2025
5q20
Year

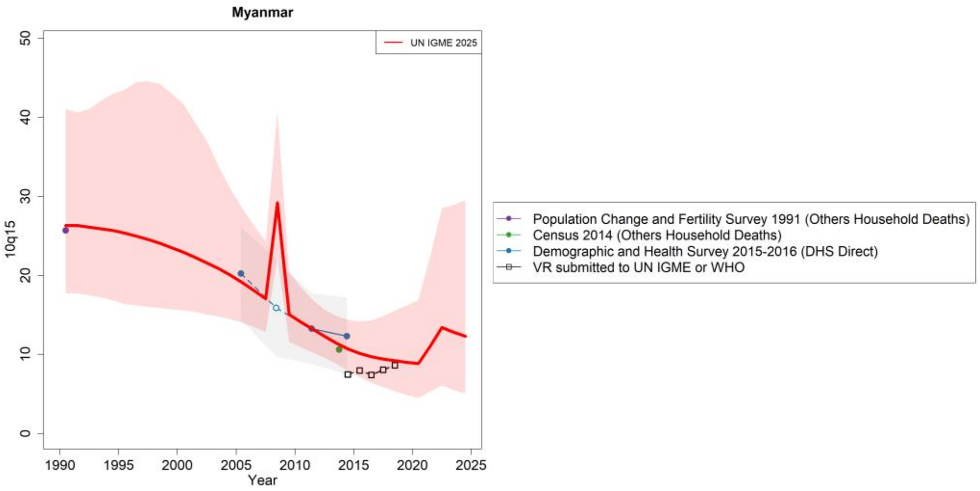
Myanmar
UN IGME 2025
10q15
Year
Population Change and Fertility Survey 1991 (Others Household Deaths)
Census 2014 (Others Household Deaths)
Demographic and Health Survey 2015-2016 (DHS Direct)
VR submitted to UN IGME or WHO

Namibia (NAM)

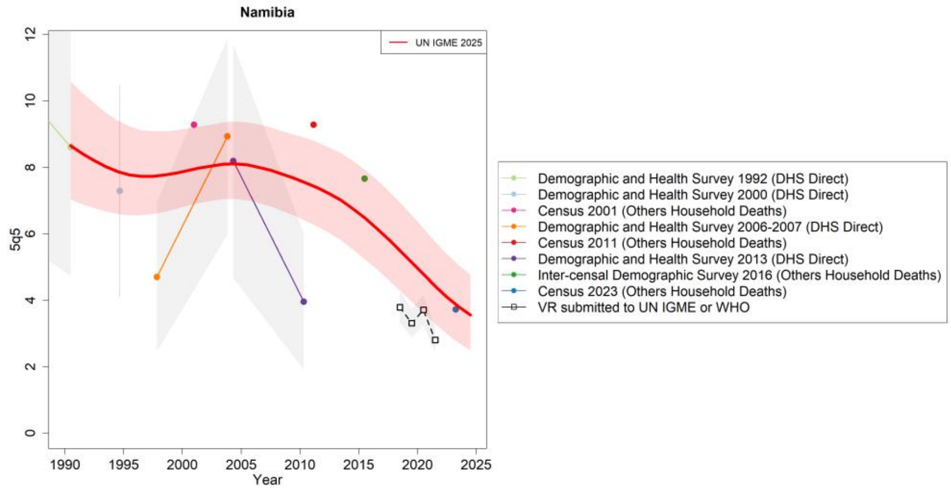
Namibia
UN IGME 2025
5q5
Year
Demographic and Health Survey 1992 (DHS Direct)
Demographic and Health Survey 2000 (DHS Direct)
Census 2001 (Others Household Deaths)
Demographic and Health Survey 2006-2007 (DHS Direct)
Census 2011 (Others Household Deaths)
Demographic and Health Survey 2013 (DHS Direct)
Inter-censal Demographic Survey 2016 (Others Household Deaths)
Census 2023 (Others Household Deaths)
VR submitted to UN IGME or WHO

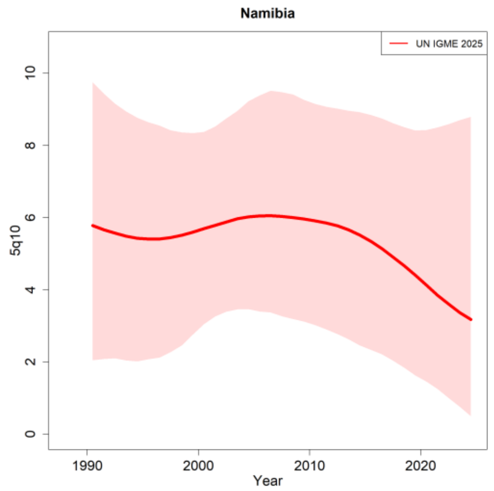
Namibia
UN IGME 2025
5q10
Year

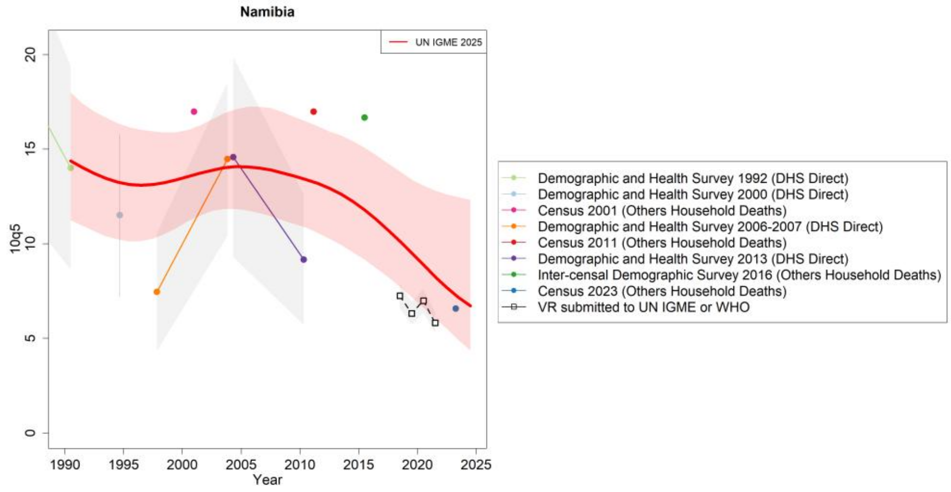
Namibia
UN IGME 2025
10q5
Year
Demographic and Health Survey 1992 (DHS Direct)
Demographic and Health Survey 2000 (DHS Direct)
Census 2001 (Others Household Deaths)
Demographic and Health Survey 2006-2007 (DHS Direct)
Census 2011 (Others Household Deaths)
Demographic and Health Survey 2013 (DHS Direct)
Inter-censal Demographic Survey 2016 (Others Household Deaths)
Census 2023 (Others Household Deaths)
VR submitted to UN IGME or WHO

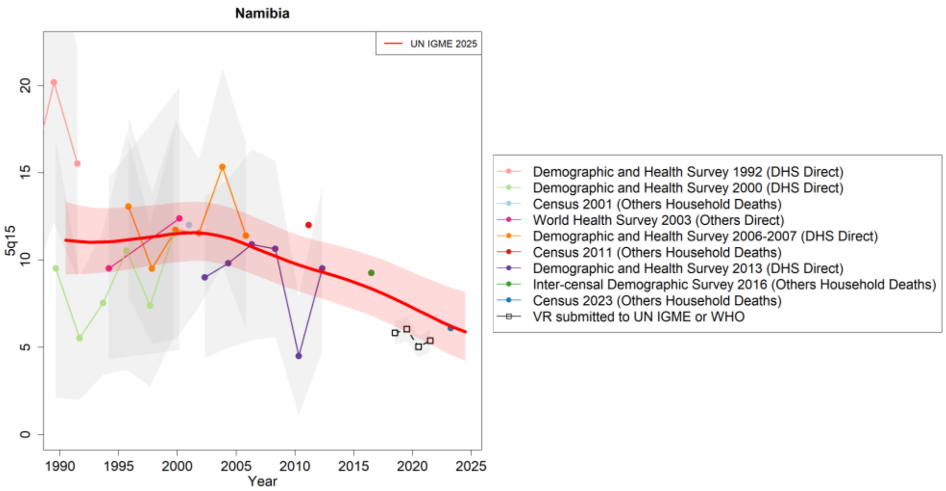
Namibia
UN IGME 2025
5q15
Year
Demographic and Health Survey 1992 (DHS Direct)
Demographic and Health Survey 2000 (DHS Direct)
Census 2001 (Others Household Deaths)
World Health Survey 2003 (Others Direct)
Demographic and Health Survey 2006-2007 (DHS Direct)
Census 2011 (Others Household Deaths)
Demographic and Health Survey 2013 (DHS Direct)
Inter-censal Demographic Survey 2016 (Others Household Deaths)
Census 2023 (Others Household Deaths)
VR submitted to UN IGME or WHO

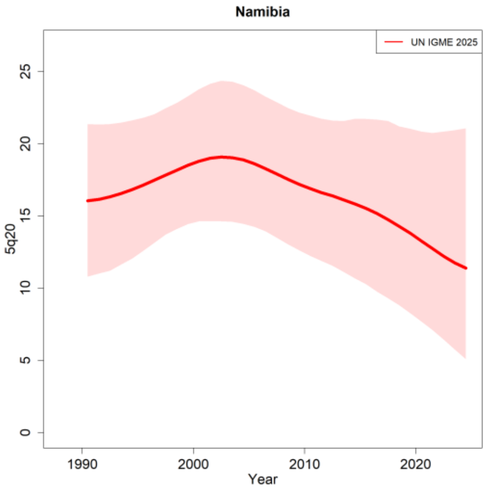
Namibia
UN IGME 2025
5q20
Year

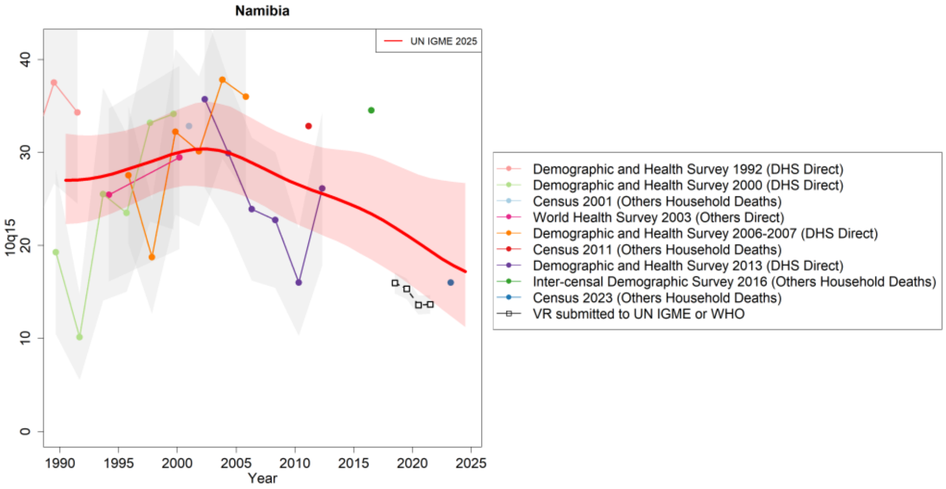
Namibia
UN IGME 2025
10q15
Year
Demographic and Health Survey 1992 (DHS Direct)
Demographic and Health Survey 2000 (DHS Direct)
Census 2001 (Others Household Deaths)
World Health Survey 2003 (Others Direct)
Demographic and Health Survey 2006-2007 (DHS Direct)
Census 2011 (Others Household Deaths)
Demographic and Health Survey 2013 (DHS Direct)
Inter-censal Demographic Survey 2016 (Others Household Deaths)
Census 2023 (Others Household Deaths)
VR submitted to UN IGME or WHO

Nauru (NRU)

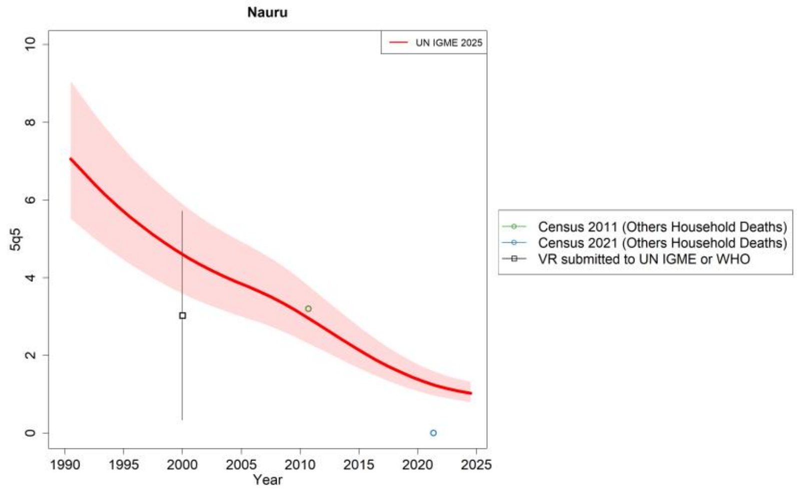
Nauru
UN IGME 2025
Census 2011 (Others Household Deaths)
Census 2021 (Others Household Deaths)
VR submitted to UN IGME or WHO
5q5
Year
0
2
4
6
8
10
1990
1995
2000
2005
2010
2015
2020
2025

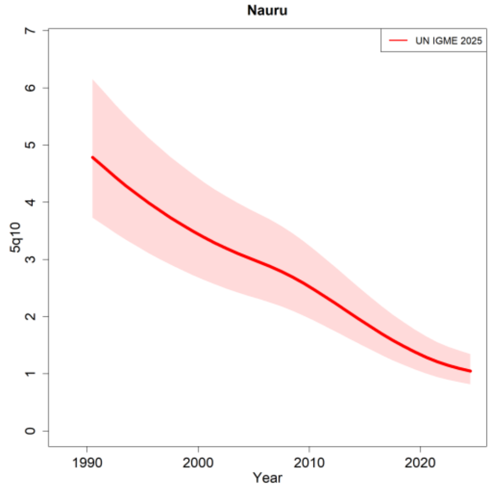
Nauru
UN IGME 2025
5q10
Year
0
1
2
3
4
5
6
7
1990
2000
2010
2020

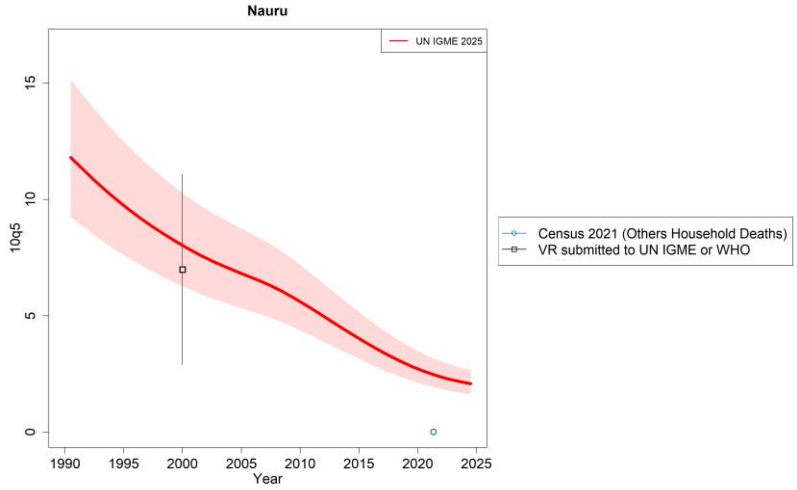
Nauru
UN IGME 2025
Census 2021 (Others Household Deaths)
VR submitted to UN IGME or WHO
10q5
Year
0
5
10
15
1990
1995
2000
2005
2010
2015
2020
2025

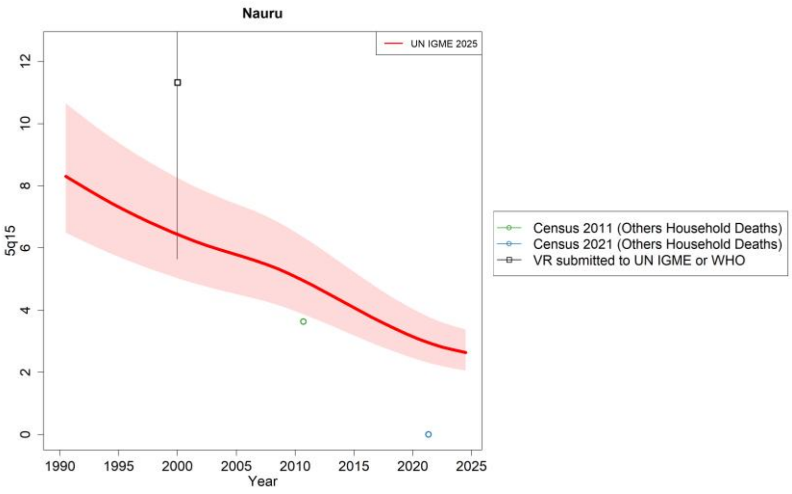
Nauru
UN IGME 2025
5q15
Year
Census 2011 (Others Household Deaths)
Census 2021 (Others Household Deaths)
VR submitted to UN IGME or WHO

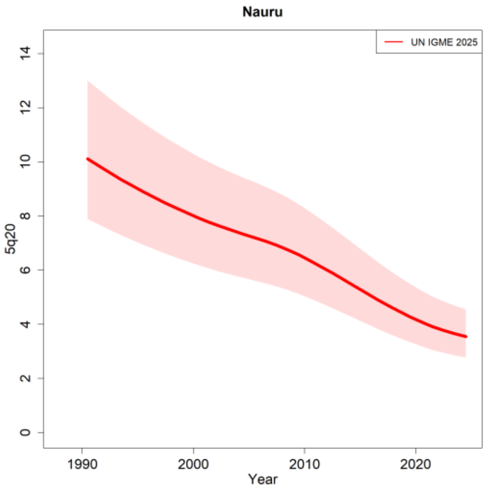
Nauru
UN IGME 2025
5q20
Year

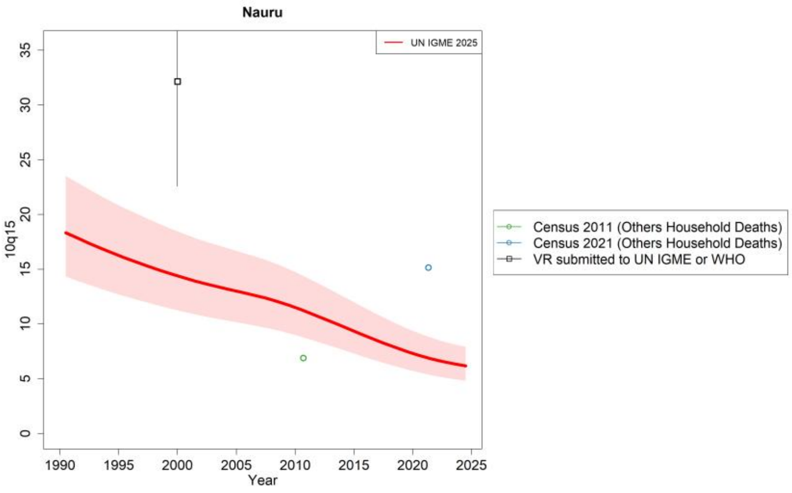
Nauru
UN IGME 2025
10q15
Year
Census 2011 (Others Household Deaths)
Census 2021 (Others Household Deaths)
VR submitted to UN IGME or WHO

Nepal (NPL)

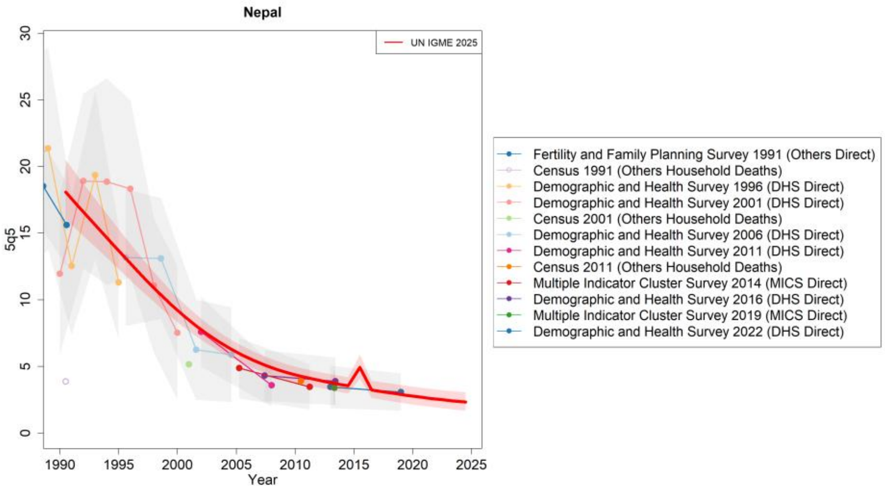
Nepal
UN IGME 2025
5q5
Year
Fertility and Family Planning Survey 1991 (Others Direct)
Census 1991 (Others Household Deaths)
Demographic and Health Survey 1996 (DHS Direct)
Demographic and Health Survey 2001 (DHS Direct)
Census 2001 (Others Household Deaths)
Demographic and Health Survey 2006 (DHS Direct)
Demographic and Health Survey 2011 (DHS Direct)
Census 2011 (Others Household Deaths)
Multiple Indicator Cluster Survey 2014 (MICS Direct)
Demographic and Health Survey 2016 (DHS Direct)
Multiple Indicator Cluster Survey 2019 (MICS Direct)
Demographic and Health Survey 2022 (DHS Direct)

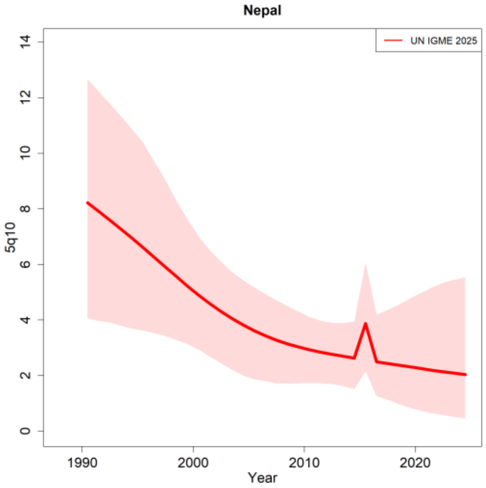
Nepal
UN IGME 2025
5q10
Year

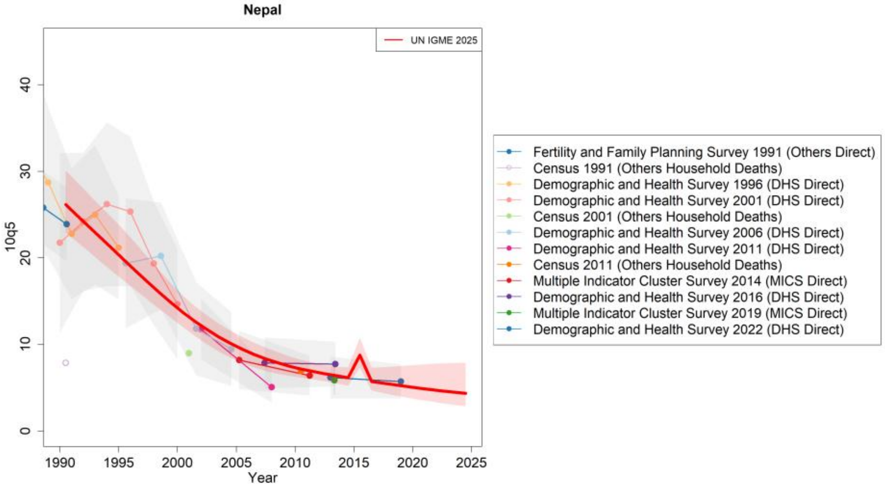
Nepal
UN IGME 2025
10q5
Year
Fertility and Family Planning Survey 1991 (Others Direct)
Census 1991 (Others Household Deaths)
Demographic and Health Survey 1996 (DHS Direct)
Demographic and Health Survey 2001 (DHS Direct)
Census 2001 (Others Household Deaths)
Demographic and Health Survey 2006 (DHS Direct)
Demographic and Health Survey 2011 (DHS Direct)
Census 2011 (Others Household Deaths)
Multiple Indicator Cluster Survey 2014 (MICS Direct)
Demographic and Health Survey 2016 (DHS Direct)
Multiple Indicator Cluster Survey 2019 (MICS Direct)
Demographic and Health Survey 2022 (DHS Direct)

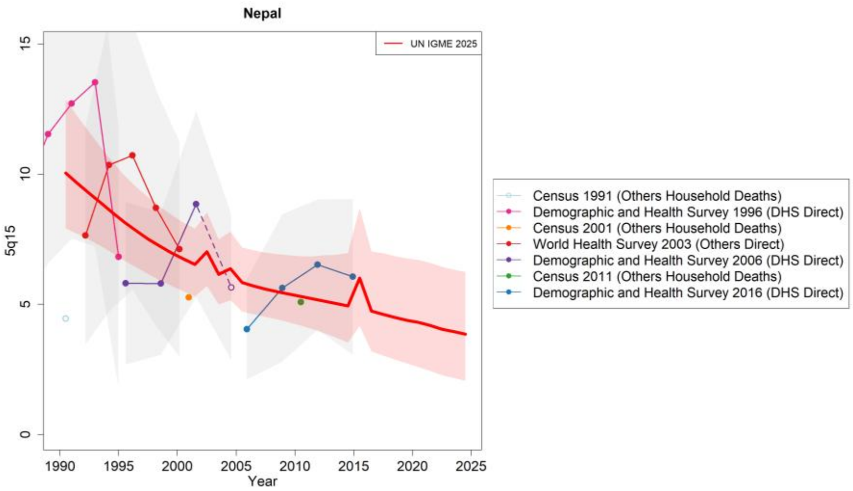
Nepal
UN IGME 2025
5q15
Year
Census 1991 (Others Household Deaths)
Demographic and Health Survey 1996 (DHS Direct)
Census 2001 (Others Household Deaths)
World Health Survey 2003 (Others Direct)
Demographic and Health Survey 2006 (DHS Direct)
Census 2011 (Others Household Deaths)
Demographic and Health Survey 2016 (DHS Direct)

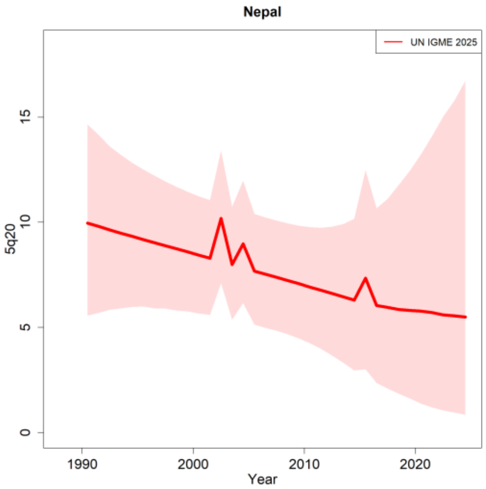
Nepal
UN IGME 2025
5q20
Year

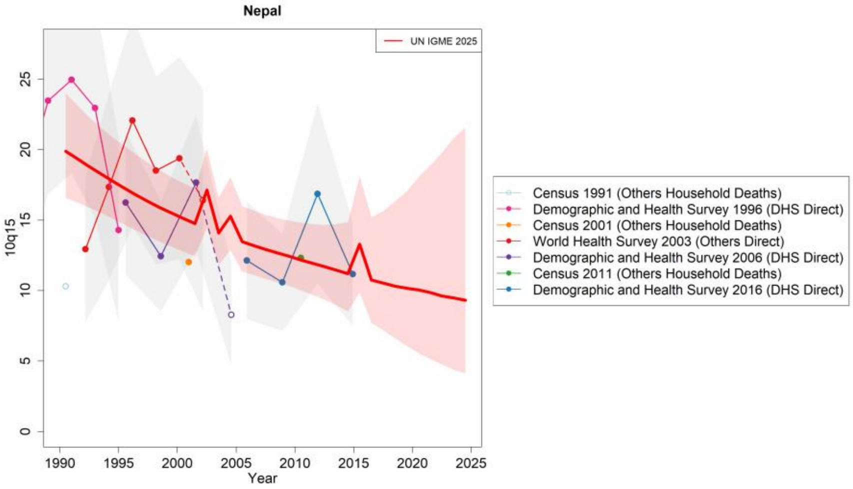
Nepal
UN IGME 2025
10q15
Year
Census 1991 (Others Household Deaths)
Demographic and Health Survey 1996 (DHS Direct)
Census 2001 (Others Household Deaths)
World Health Survey 2003 (Others Direct)
Demographic and Health Survey 2006 (DHS Direct)
Census 2011 (Others Household Deaths)
Demographic and Health Survey 2016 (DHS Direct)

Netherlands (Kingdom of the) (NLD)

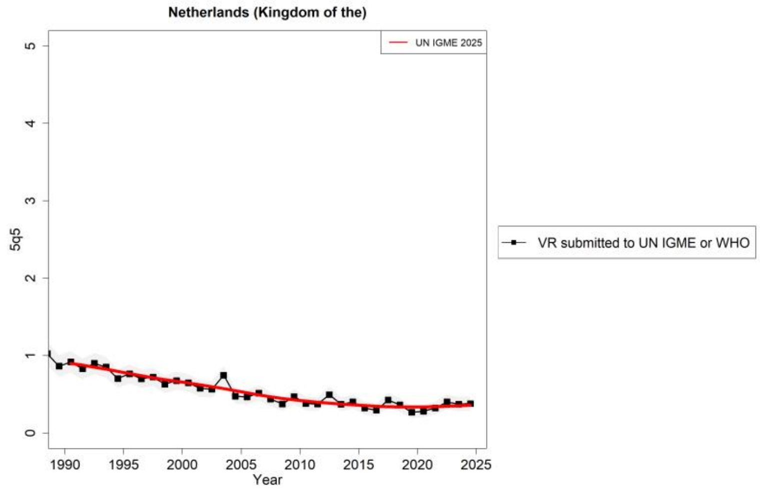

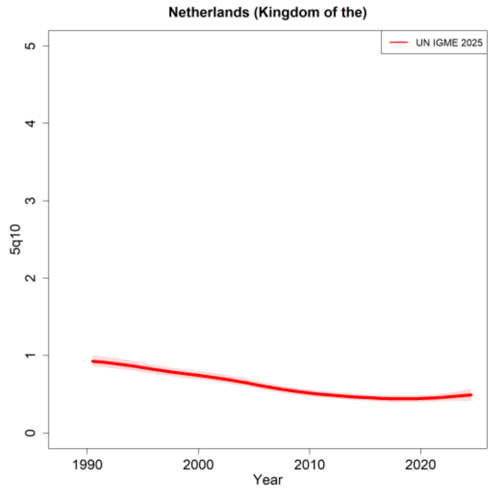

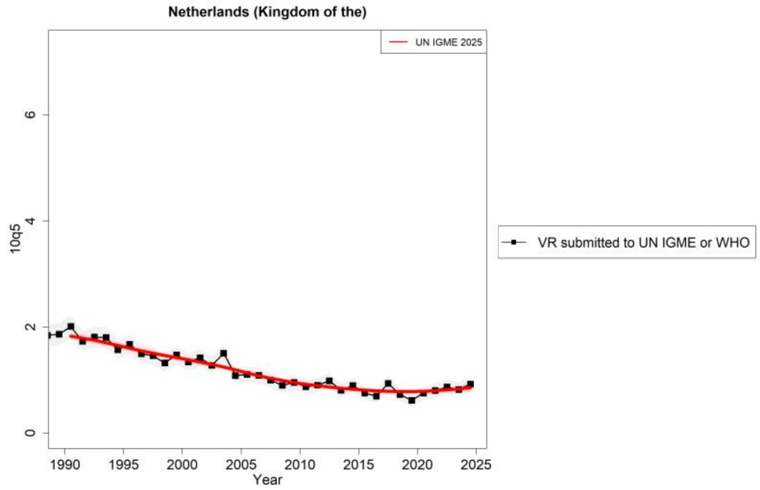

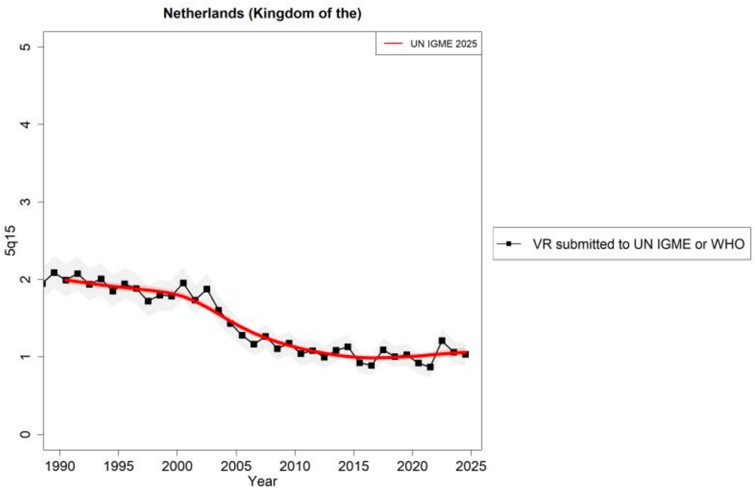
Netherlands (Kingdom of the)
UN IGME 2025
VR submitted to UN IGME or WHO
5q15
0
1
2
3
4
5
1990
1995
2000
2005
2010
2015
2020
2025
Year

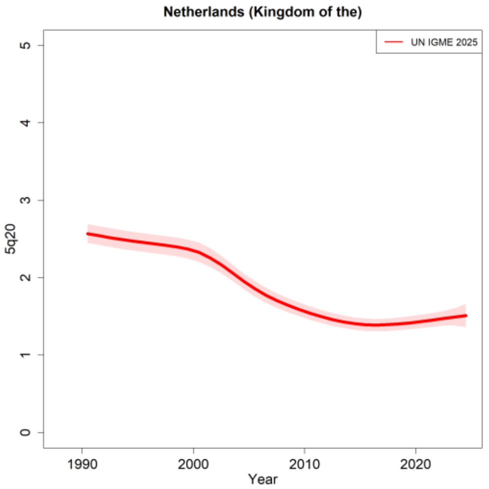
Netherlands (Kingdom of the)
UN IGME 2025
5q20
0
1
2
3
4
5
1990
2000
2010
2020
Year

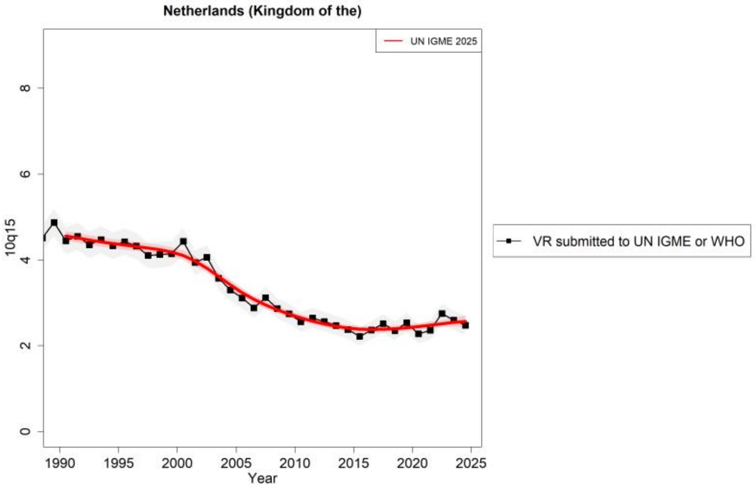
Netherlands (Kingdom of the)
UN IGME 2025
VR submitted to UN IGME or WHO
10q15
0
2
4
6
8
1990
1995
2000
2005
2010
2015
2020
2025
Year

New Zealand (NZL)

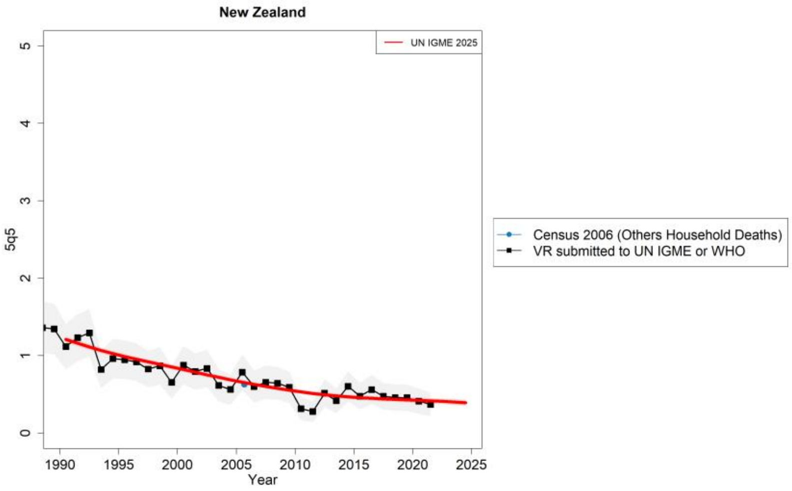

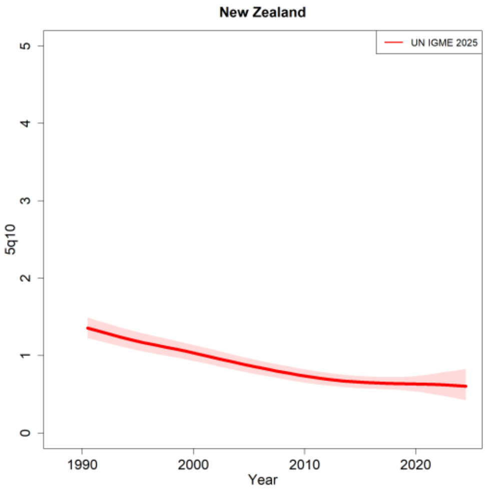

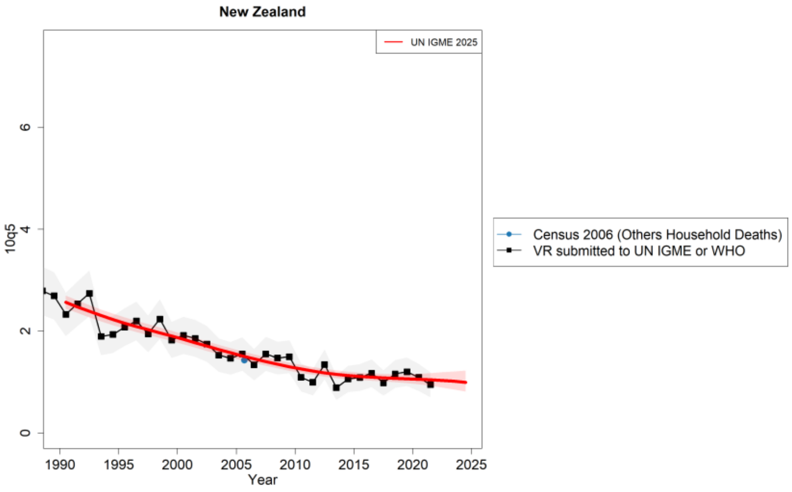

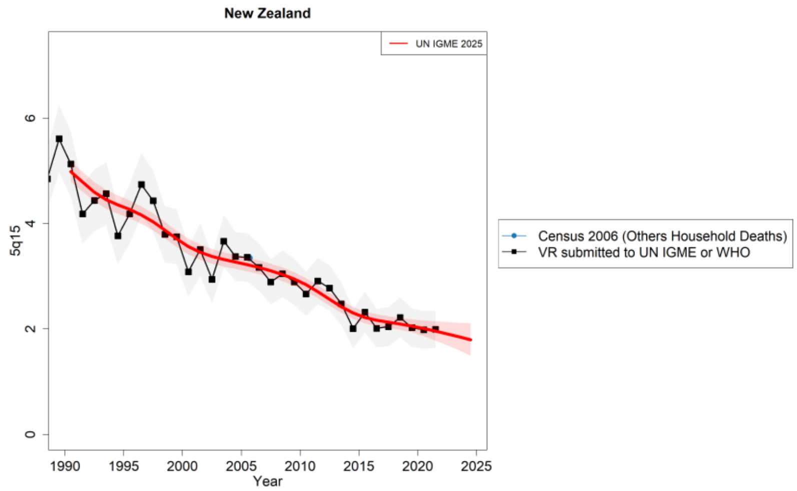
New Zealand
UN IGME 2025
5q15
Year
Census 2006 (Others Household Deaths)
VR submitted to UN IGME or WHO

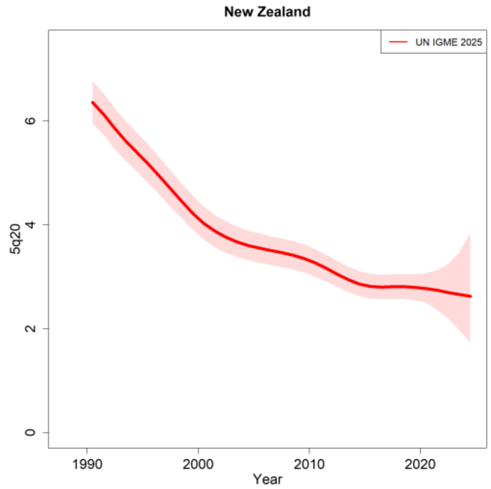
New Zealand
UN IGME 2025
5q20
Year

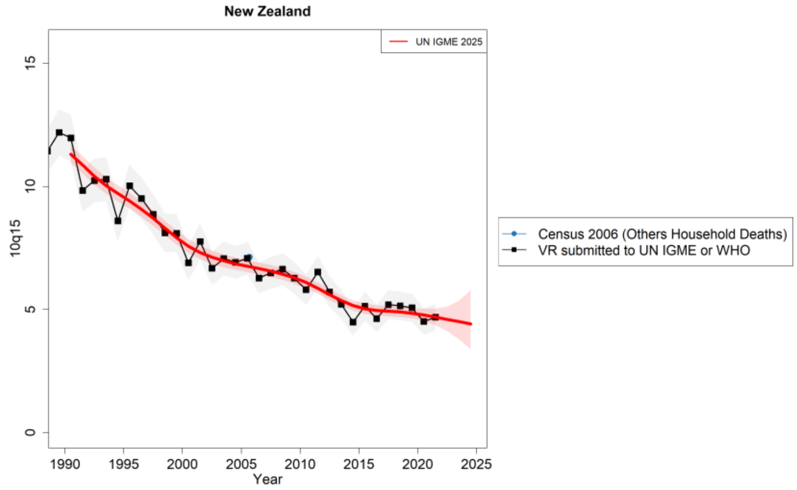
New Zealand
UN IGME 2025
10q15
Year
Census 2006 (Others Household Deaths)
VR submitted to UN IGME or WHO

Nicaragua (NIC)

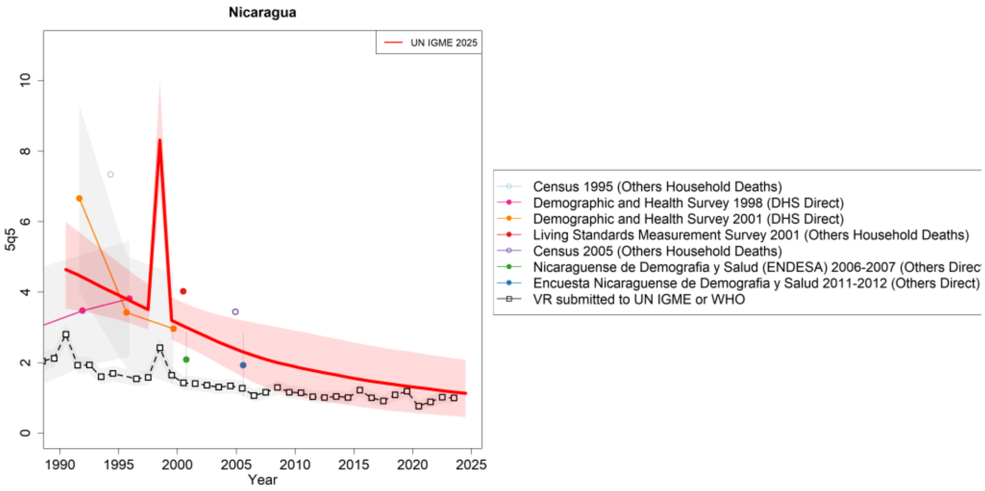

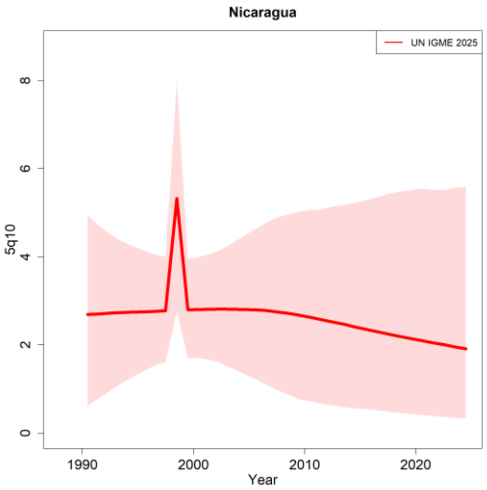

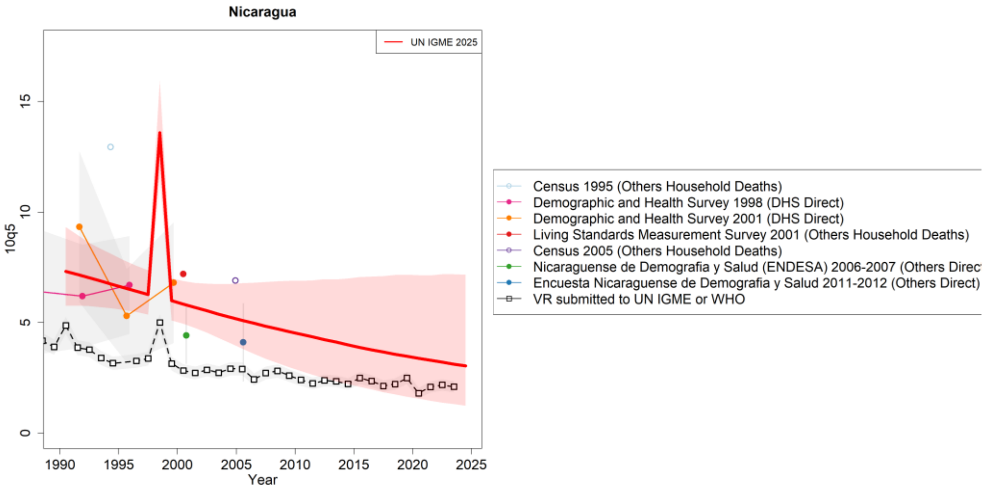

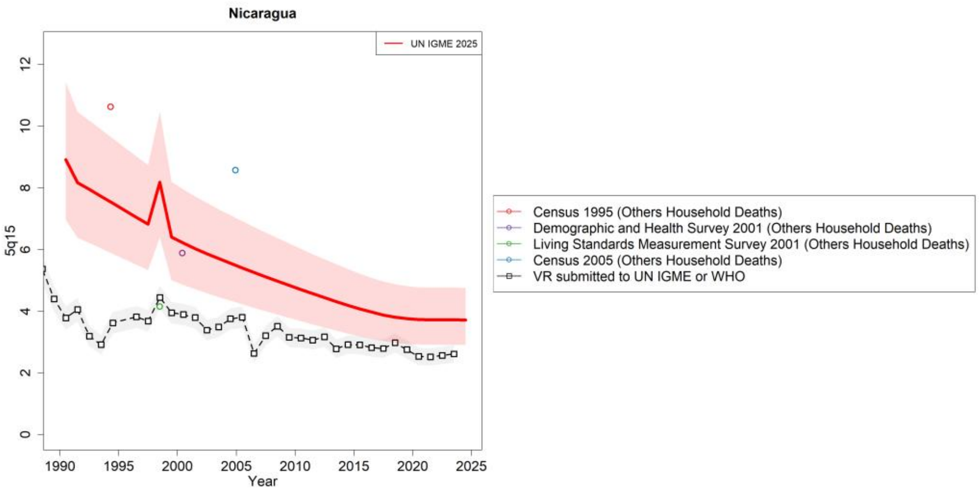
Nicaragua
UN IGME 2025
5q15
Year
Census 1995 (Others Household Deaths)
Demographic and Health Survey 2001 (Others Household Deaths)
Living Standards Measurement Survey 2001 (Others Household Deaths)
Census 2005 (Others Household Deaths)
VR submitted to UN IGME or WHO

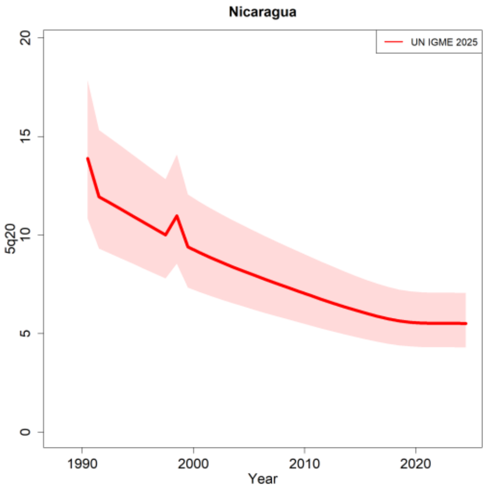
Nicaragua
UN IGME 2025
5q20
Year

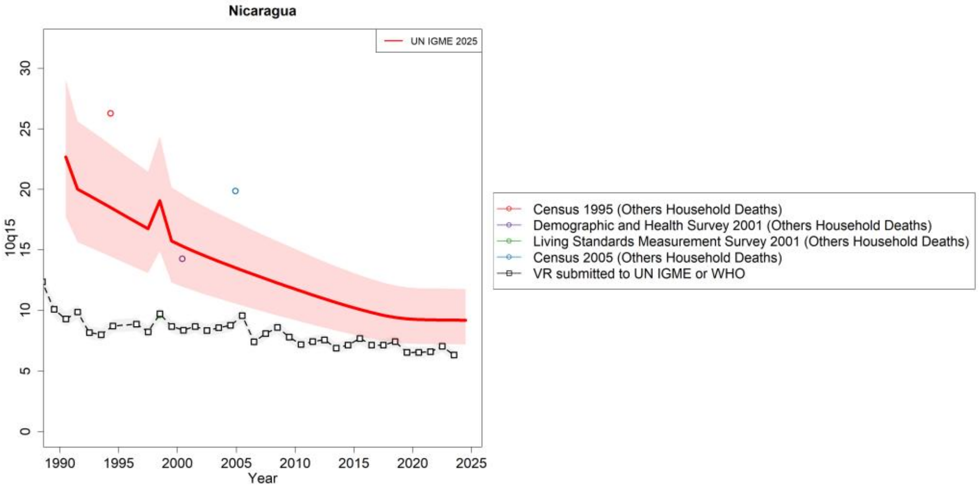
Nicaragua
UN IGME 2025
10q15
Year
Census 1995 (Others Household Deaths)
Demographic and Health Survey 2001 (Others Household Deaths)
Living Standards Measurement Survey 2001 (Others Household Deaths)
Census 2005 (Others Household Deaths)
VR submitted to UN IGME or WHO

Niger (NER)

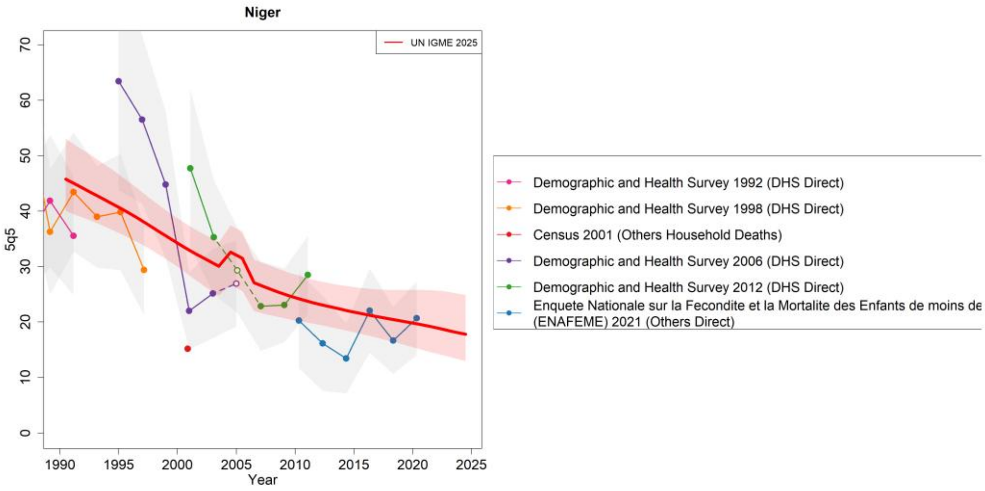
Niger
UN IGME 2025
5q5
Year
Demographic and Health Survey 1992 (DHS Direct)
Demographic and Health Survey 1998 (DHS Direct)
Census 2001 (Others Household Deaths)
Demographic and Health Survey 2006 (DHS Direct)
Demographic and Health Survey 2012 (DHS Direct)
Enquete Nationale sur la Fecondite et la Mortalite des Enfants de moins de
(ENAFEME) 2021 (Others Direct)

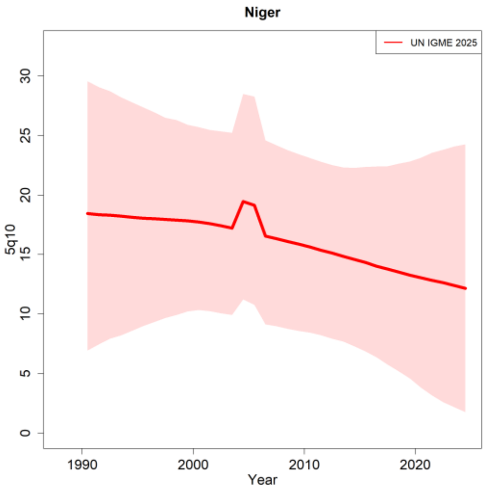
Niger
UN IGME 2025
5q10
Year

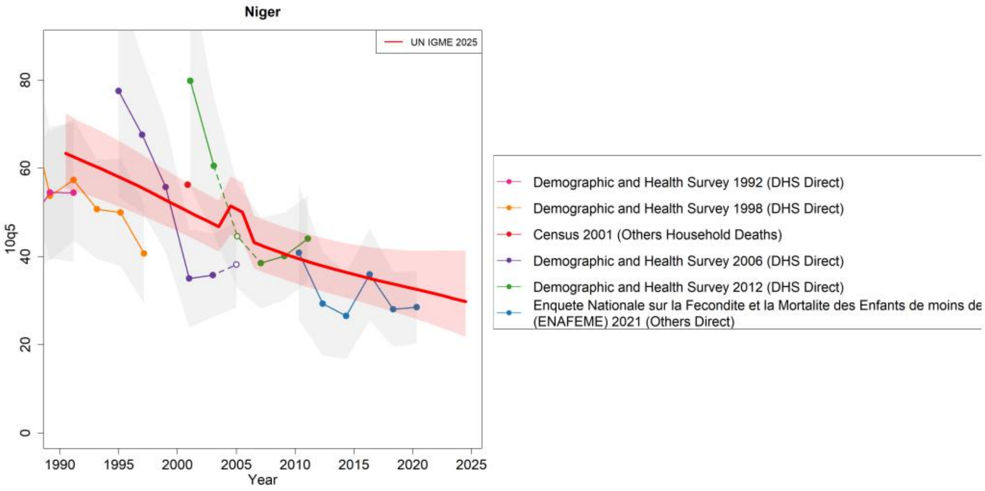
Niger
UN IGME 2025
10q5
Year
Demographic and Health Survey 1992 (DHS Direct)
Demographic and Health Survey 1998 (DHS Direct)
Census 2001 (Others Household Deaths)
Demographic and Health Survey 2006 (DHS Direct)
Demographic and Health Survey 2012 (DHS Direct)
Enquete Nationale sur la Fecondite et la Mortalite des Enfants de moins de
(ENAFEME) 2021 (Others Direct)

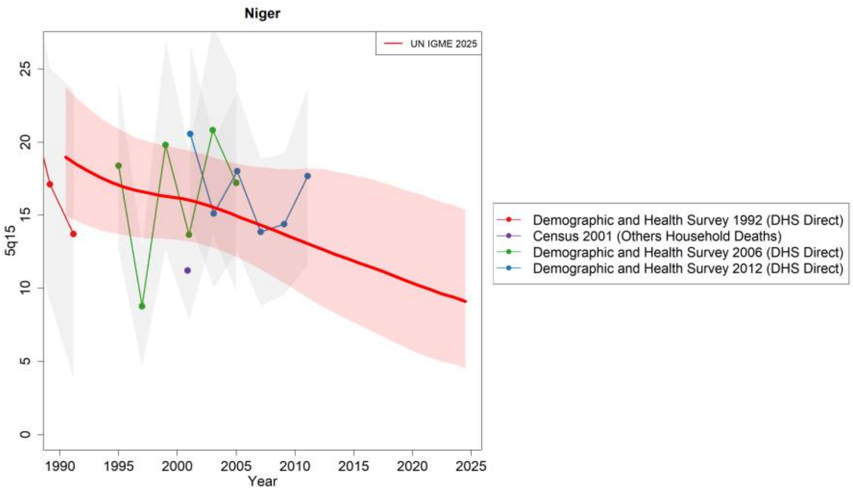
Niger
UN IGME 2025
Demographic and Health Survey 1992 (DHS Direct)
Census 2001 (Others Household Deaths)
Demographic and Health Survey 2006 (DHS Direct)
Demographic and Health Survey 2012 (DHS Direct)
5q15
Year

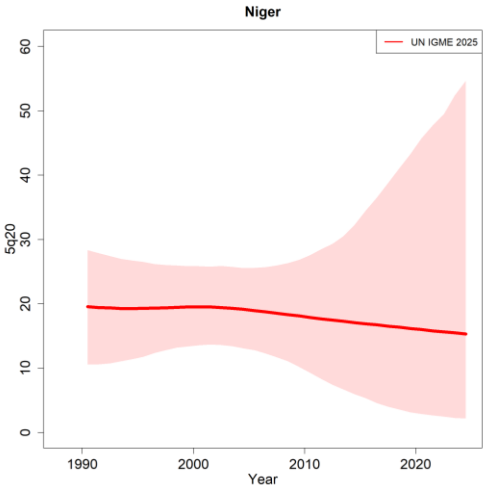
Niger
UN IGME 2025
5q20
Year

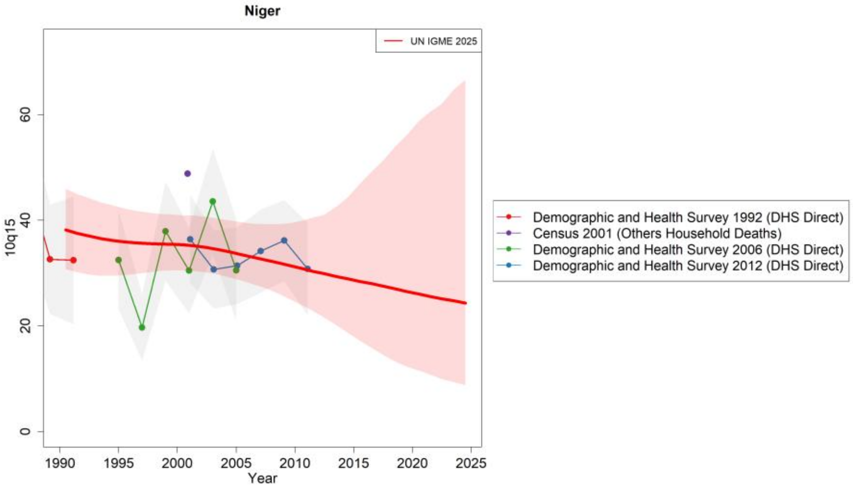
Niger
UN IGME 2025
Demographic and Health Survey 1992 (DHS Direct)
Census 2001 (Others Household Deaths)
Demographic and Health Survey 2006 (DHS Direct)
Demographic and Health Survey 2012 (DHS Direct)
10q15
Year

Nigeria (NGA)

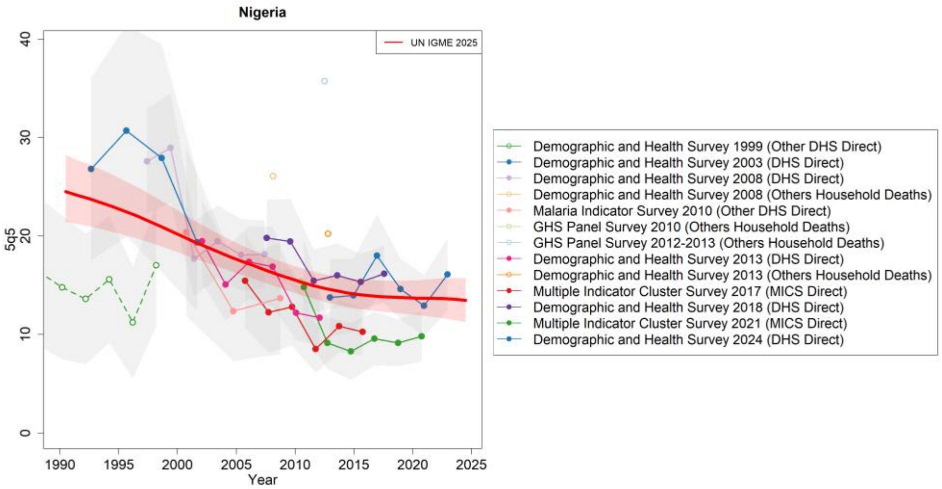
Nigeria
UN IGME 2025
5q5
Year
Demographic and Health Survey 1999 (Other DHS Direct)
Demographic and Health Survey 2003 (DHS Direct)
Demographic and Health Survey 2008 (DHS Direct)
Demographic and Health Survey 2008 (Others Household Deaths)
Malaria Indicator Survey 2010 (Other DHS Direct)
GHS Panel Survey 2010 (Others Household Deaths)
GHS Panel Survey 2012-2013 (Others Household Deaths)
Demographic and Health Survey 2013 (DHS Direct)
Demographic and Health Survey 2013 (Others Household Deaths)
Multiple Indicator Cluster Survey 2017 (MICS Direct)
Demographic and Health Survey 2018 (DHS Direct)
Multiple Indicator Cluster Survey 2021 (MICS Direct)
Demographic and Health Survey 2024 (DHS Direct)

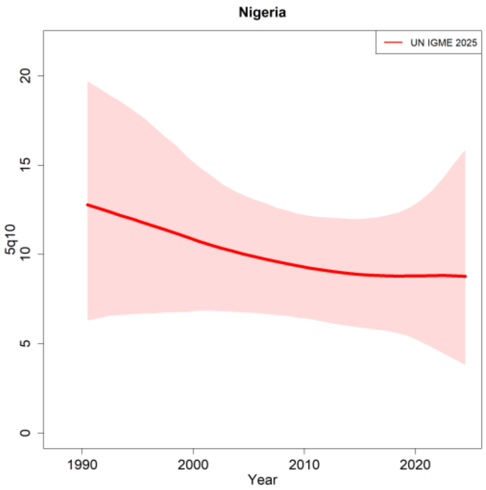
Nigeria
UN IGME 2025
5q10
Year

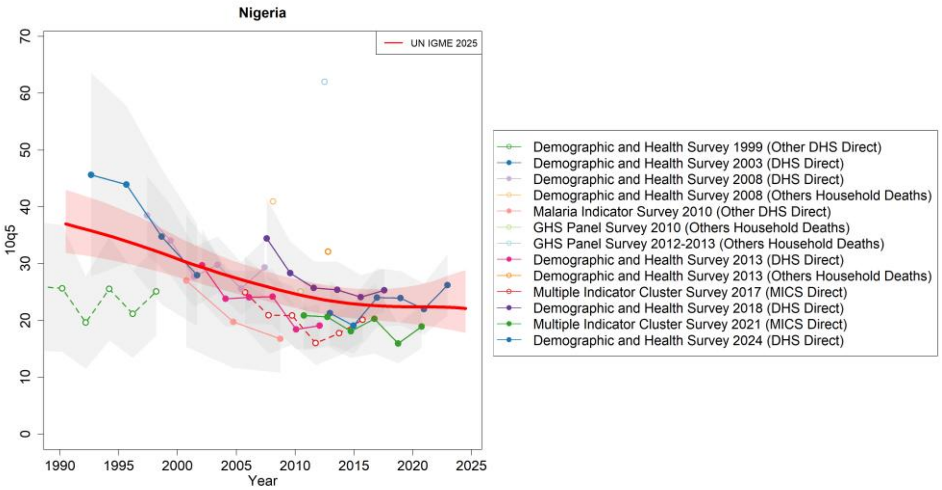
Nigeria
UN IGME 2025
10q5
Year
Demographic and Health Survey 1999 (Other DHS Direct)
Demographic and Health Survey 2003 (DHS Direct)
Demographic and Health Survey 2008 (DHS Direct)
Demographic and Health Survey 2008 (Others Household Deaths)
Malaria Indicator Survey 2010 (Other DHS Direct)
GHS Panel Survey 2010 (Others Household Deaths)
GHS Panel Survey 2012-2013 (Others Household Deaths)
Demographic and Health Survey 2013 (DHS Direct)
Demographic and Health Survey 2013 (Others Household Deaths)
Multiple Indicator Cluster Survey 2017 (MICS Direct)
Demographic and Health Survey 2018 (DHS Direct)
Multiple Indicator Cluster Survey 2021 (MICS Direct)
Demographic and Health Survey 2024 (DHS Direct)

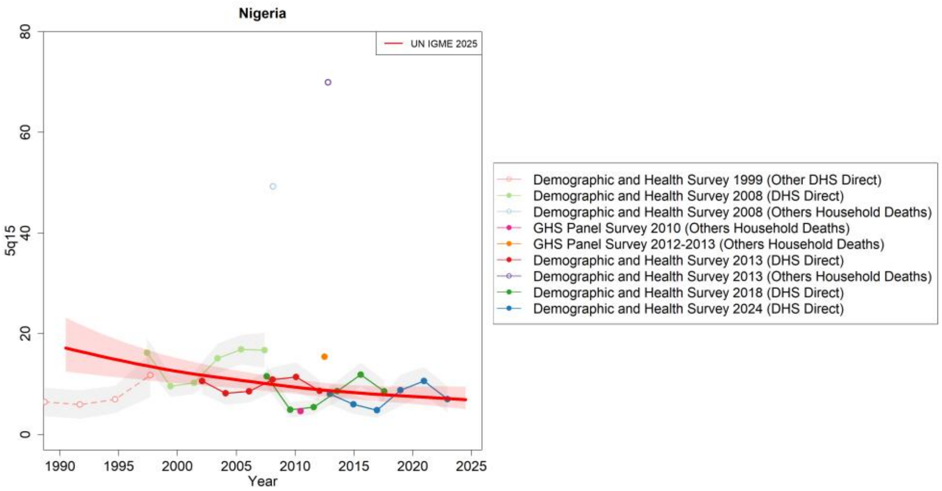
Nigeria
UN IGME 2025
5q15
0
20
40
60
80
1990
1995
2000
2005
2010
2015
2020
2025
Year
Demographic and Health Survey 1999 (Other DHS Direct)
Demographic and Health Survey 2008 (DHS Direct)
Demographic and Health Survey 2008 (Others Household Deaths)
GHS Panel Survey 2010 (Others Household Deaths)
GHS Panel Survey 2012-2013 (Others Household Deaths)
Demographic and Health Survey 2013 (DHS Direct)
Demographic and Health Survey 2013 (Others Household Deaths)
Demographic and Health Survey 2018 (DHS Direct)
Demographic and Health Survey 2024 (DHS Direct)

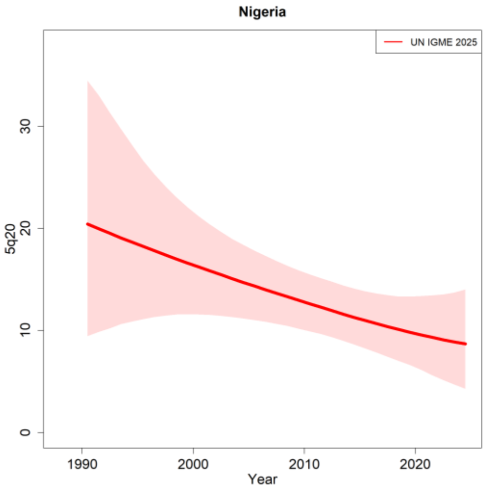
Nigeria
UN IGME 2025
5q20
0
10
20
30
1990
2000
2010
2020
Year

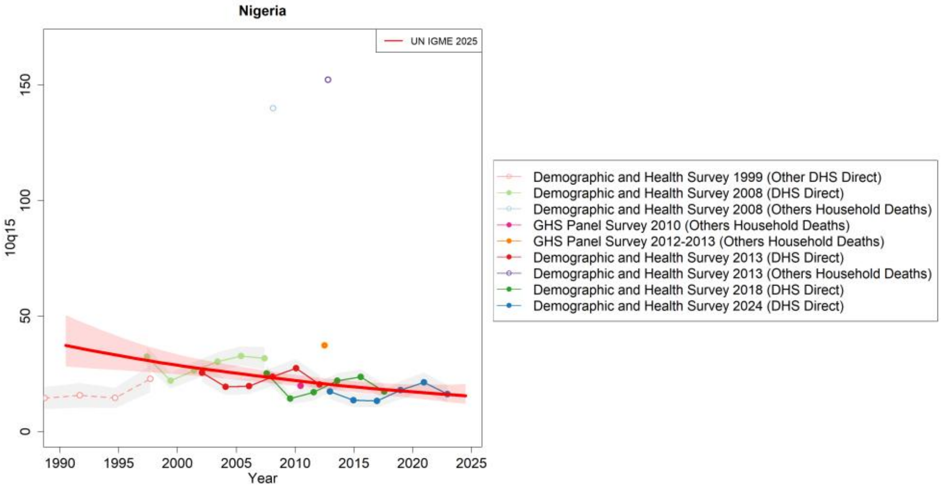
Nigeria
UN IGME 2025
10q15
0
50
100
150
1990
1995
2000
2005
2010
2015
2020
2025
Year
Demographic and Health Survey 1999 (Other DHS Direct)
Demographic and Health Survey 2008 (DHS Direct)
Demographic and Health Survey 2008 (Others Household Deaths)
GHS Panel Survey 2010 (Others Household Deaths)
GHS Panel Survey 2012-2013 (Others Household Deaths)
Demographic and Health Survey 2013 (DHS Direct)
Demographic and Health Survey 2013 (Others Household Deaths)
Demographic and Health Survey 2018 (DHS Direct)
Demographic and Health Survey 2024 (DHS Direct)

Niue (NIU)

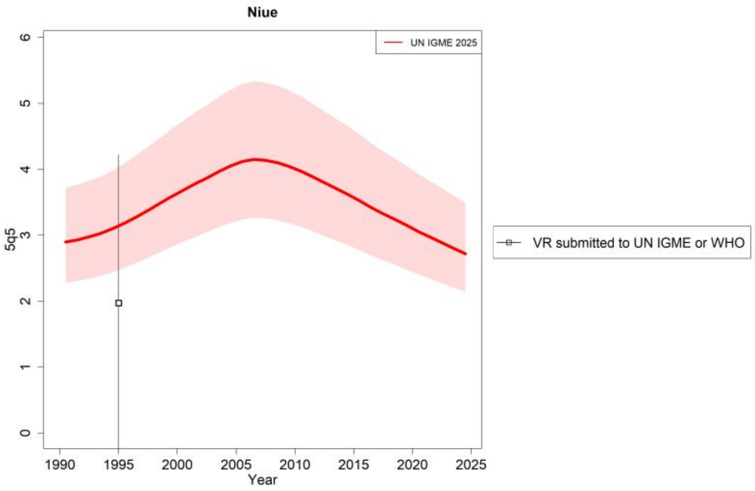
Niue
UN IGME 2025
VR submitted to UN IGME or WHO
5q5
Year

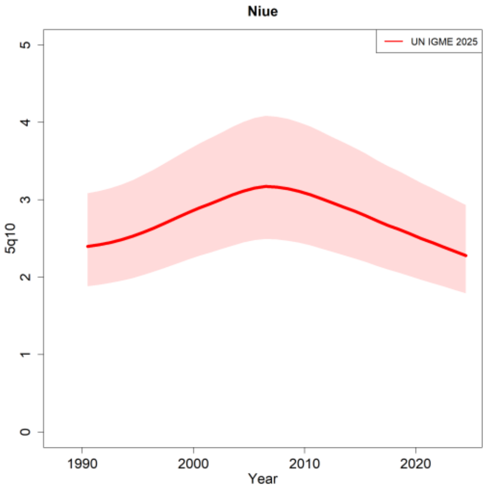
Niue
UN IGME 2025
5q10
Year

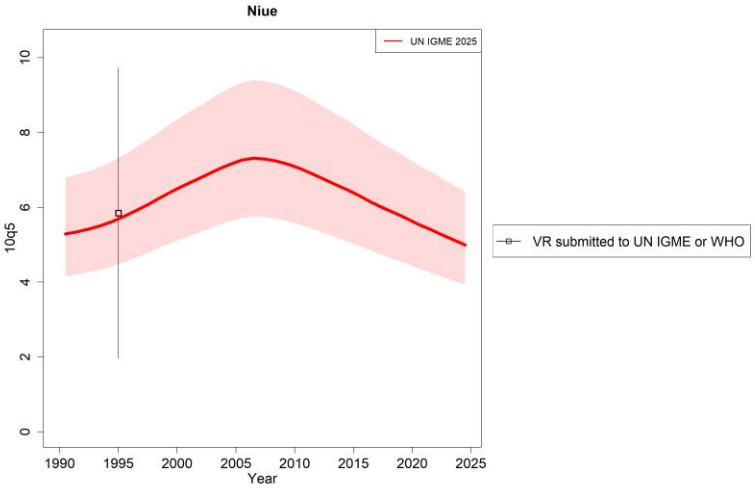
Niue
UN IGME 2025
VR submitted to UN IGME or WHO
10q5
Year

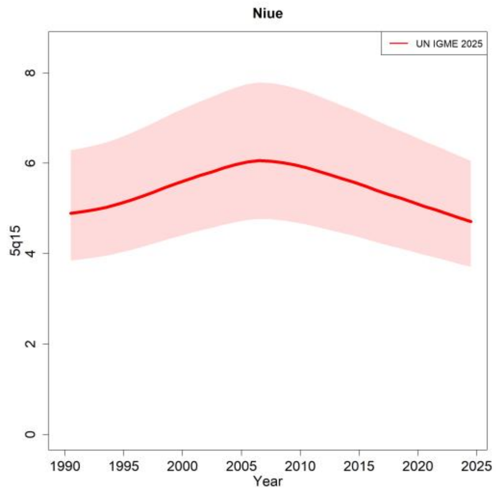
Niue
UN IGME 2025
5q15
Year
0
2
4
6
8
1990
1995
2000
2005
2010
2015
2020
2025

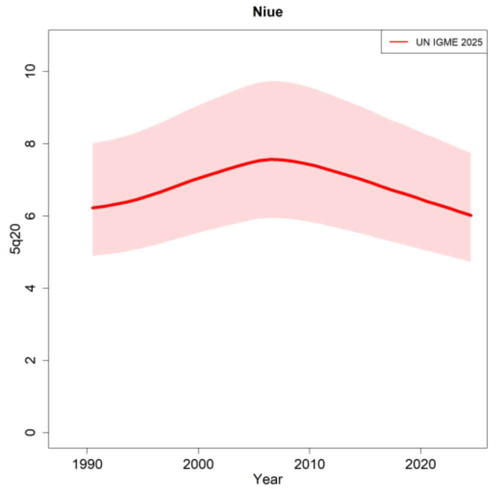
Niue
UN IGME 2025
5q20
Year
0
2
4
6
8
10
1990
2000
2010
2020

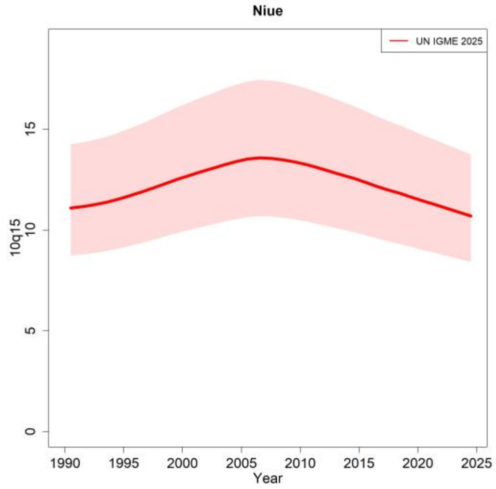
Niue
UN IGME 2025
10q15
Year
0
5
10
15
1990
1995
2000
2005
2010
2015
2020
2025

North Macedonia (MKD)

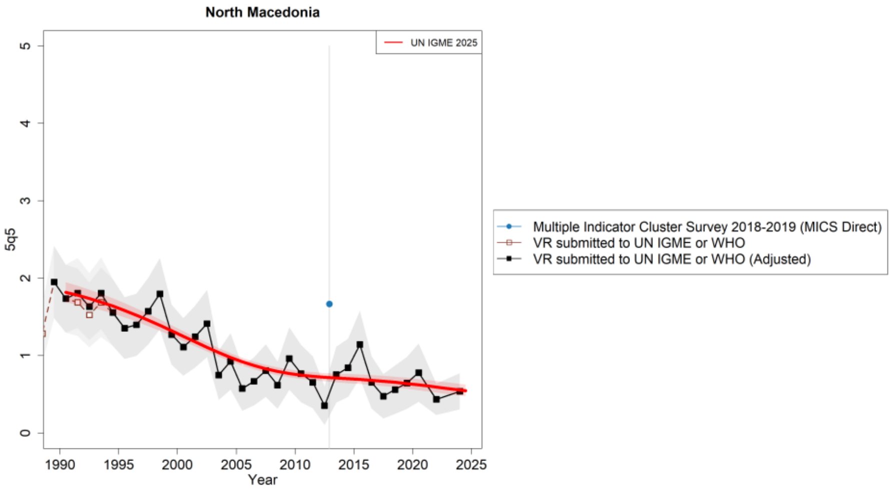

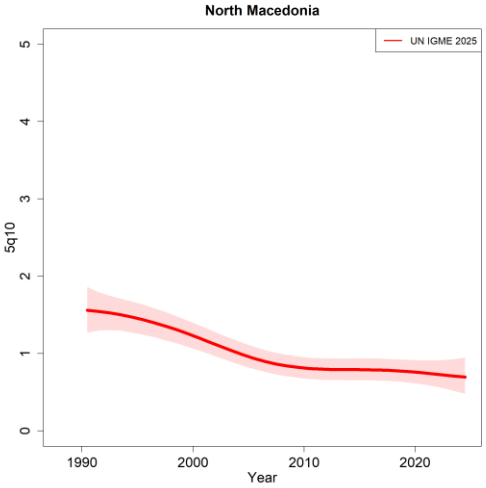

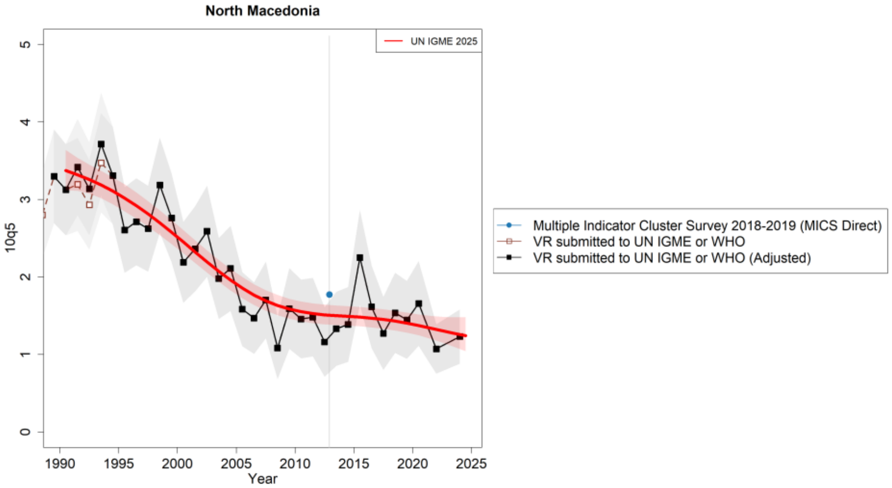

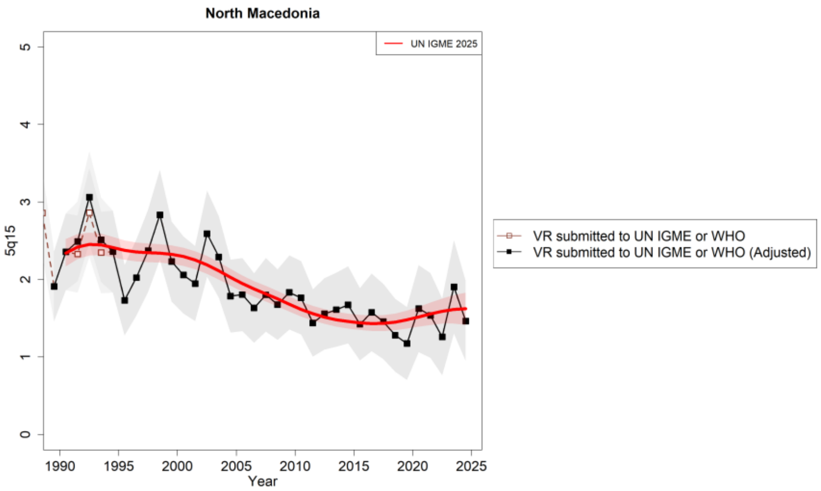
North Macedonia
UN IGME 2025
VR submitted to UN IGME or WHO
VR submitted to UN IGME or WHO (Adjusted)
5q15
Year
0
1
2
3
4
5
1990
1995
2000
2005
2010
2015
2020
2025

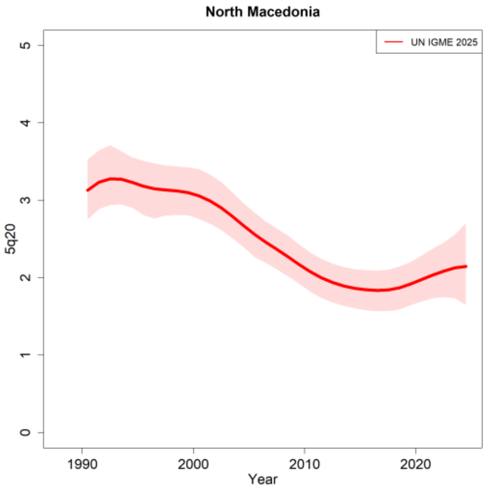
North Macedonia
UN IGME 2025
5q20
Year
0
1
2
3
4
5
1990
2000
2010
2020

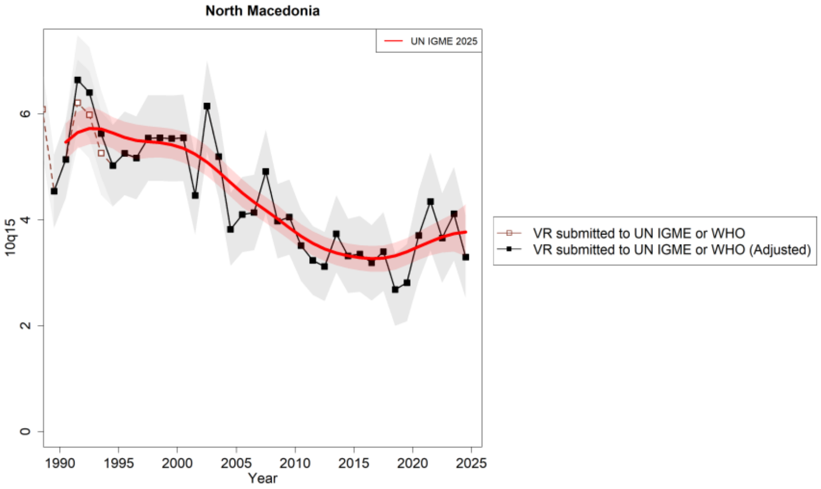
North Macedonia
UN IGME 2025
VR submitted to UN IGME or WHO
VR submitted to UN IGME or WHO (Adjusted)
10q15
Year
0
2
4
6
1990
1995
2000
2005
2010
2015
2020
2025

Norway (NOR)

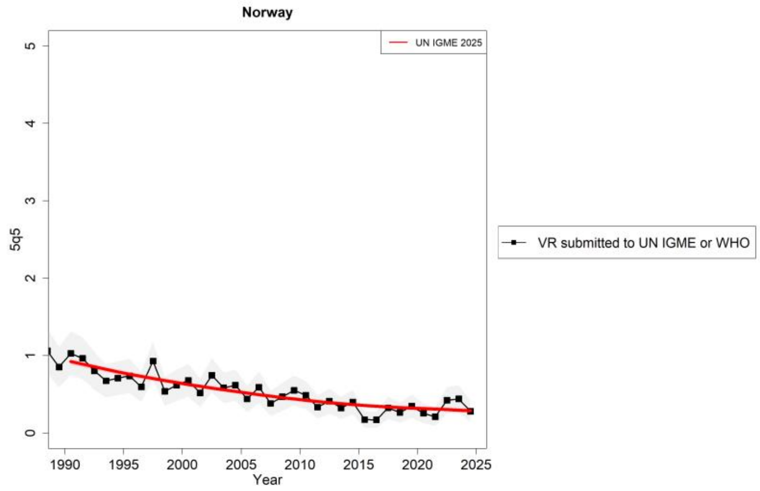

Norway
UN IGME 2025
VR submitted to UN IGME or WHO
5q5
Year

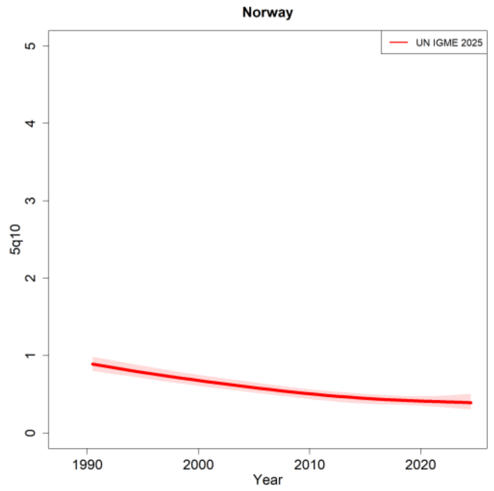

Norway
UN IGME 2025
5q10
Year

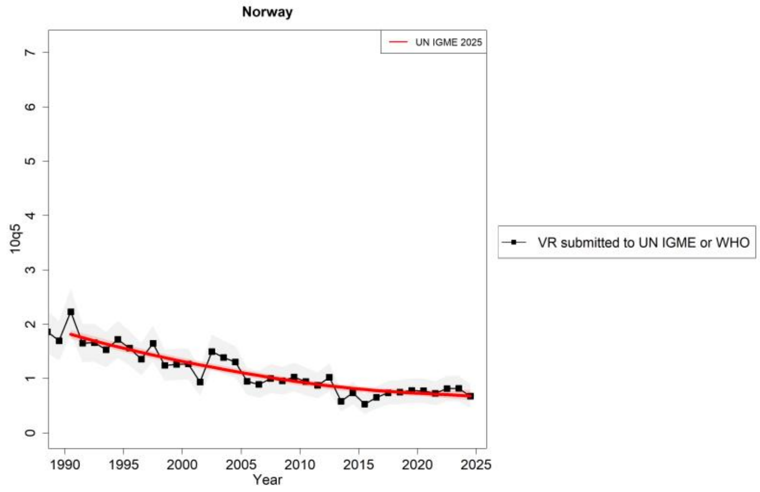

Norway
UN IGME 2025
VR submitted to UN IGME or WHO
10q5
Year

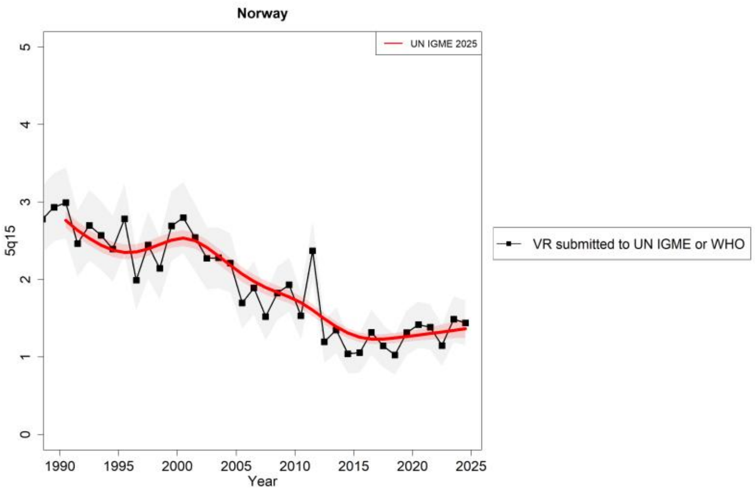
Norway
UN IGME 2025
VR submitted to UN IGME or WHO
5q15
Year

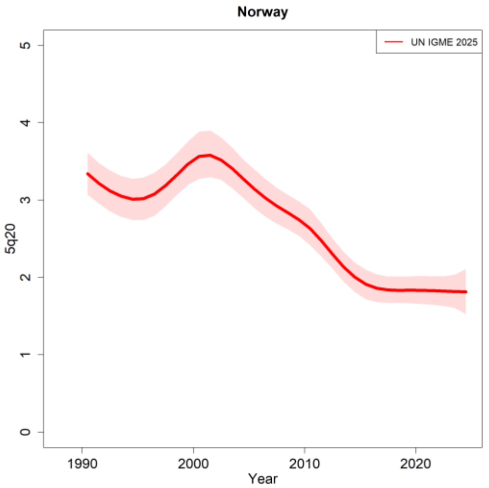
Norway
UN IGME 2025
5q20
Year

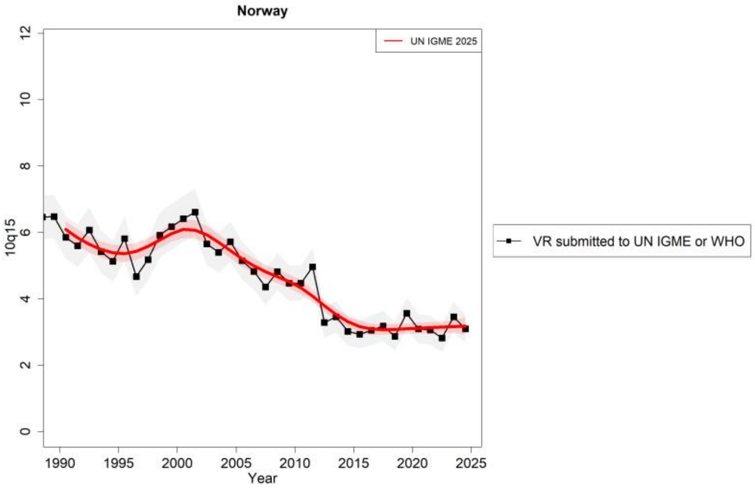
Norway
UN IGME 2025
VR submitted to UN IGME or WHO
10q15
Year

Oman (OMN)

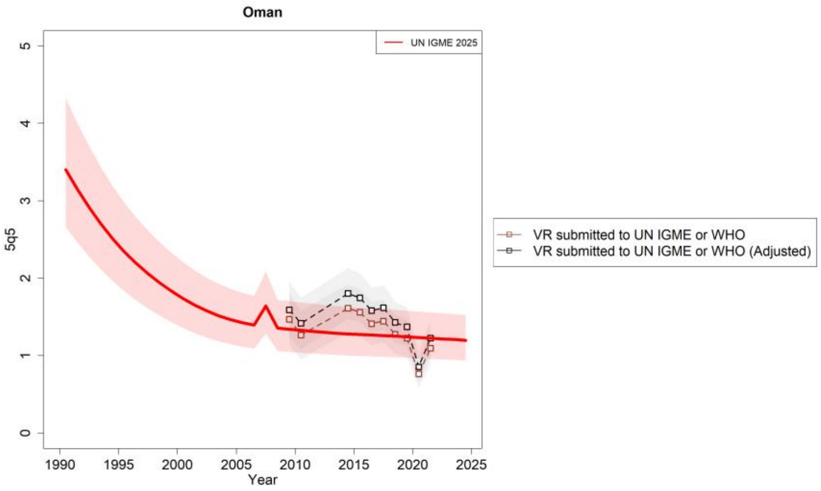

Oman
UN IGME 2025
VR submitted to UN IGME or WHO
VR submitted to UN IGME or WHO (Adjusted)
5q5
Year

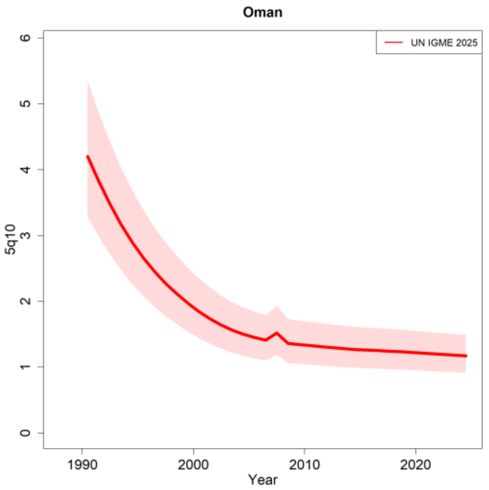

Oman
UN IGME 2025
5q10
Year

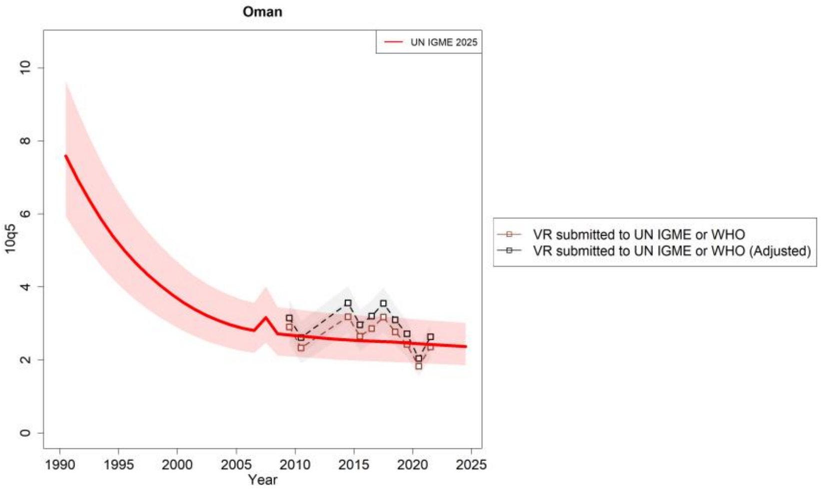

Oman
UN IGME 2025
VR submitted to UN IGME or WHO
VR submitted to UN IGME or WHO (Adjusted)
10q5
Year

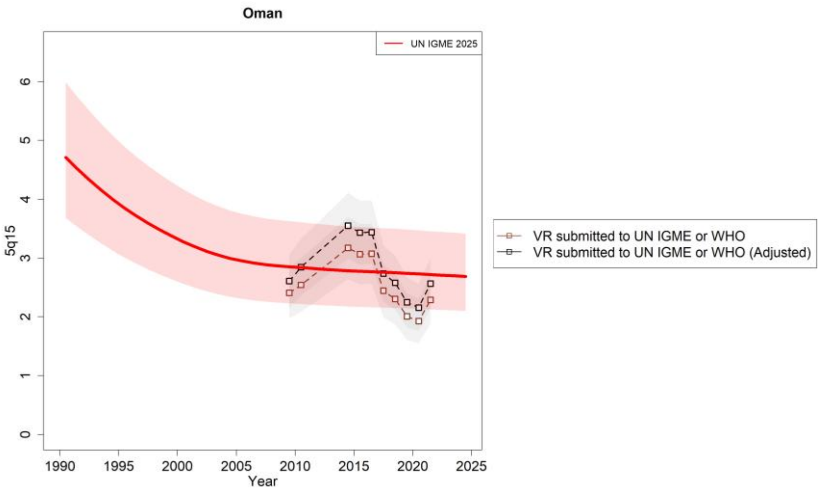
Oman
UN IGME 2025
VR submitted to UN IGME or WHO
VR submitted to UN IGME or WHO (Adjusted)
5q15
Year
0
1
2
3
4
5
6
1990
1995
2000
2005
2010
2015
2020
2025

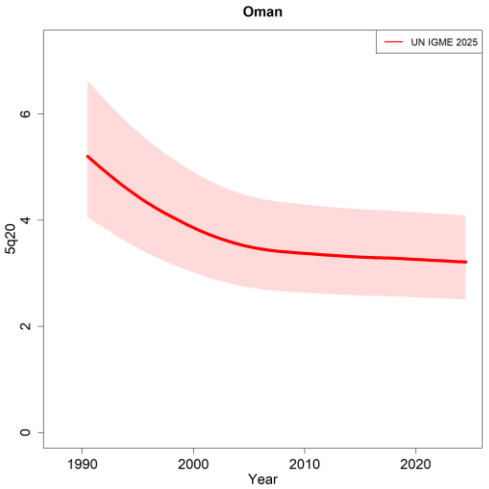
Oman
UN IGME 2025
5q20
Year
0
2
4
6
1990
2000
2010
2020

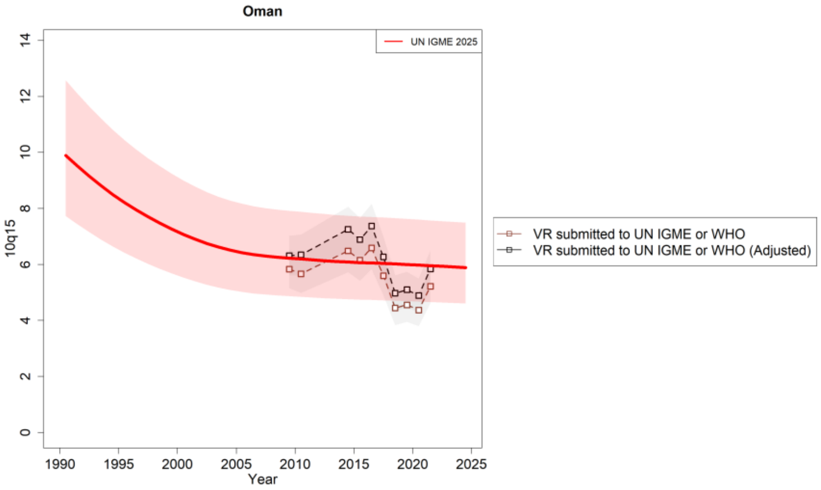
Oman
UN IGME 2025
VR submitted to UN IGME or WHO
VR submitted to UN IGME or WHO (Adjusted)
10q15
Year
0
2
4
6
8
10
12
14
1990
1995
2000
2005
2010
2015
2020
2025

Pakistan (PAK)

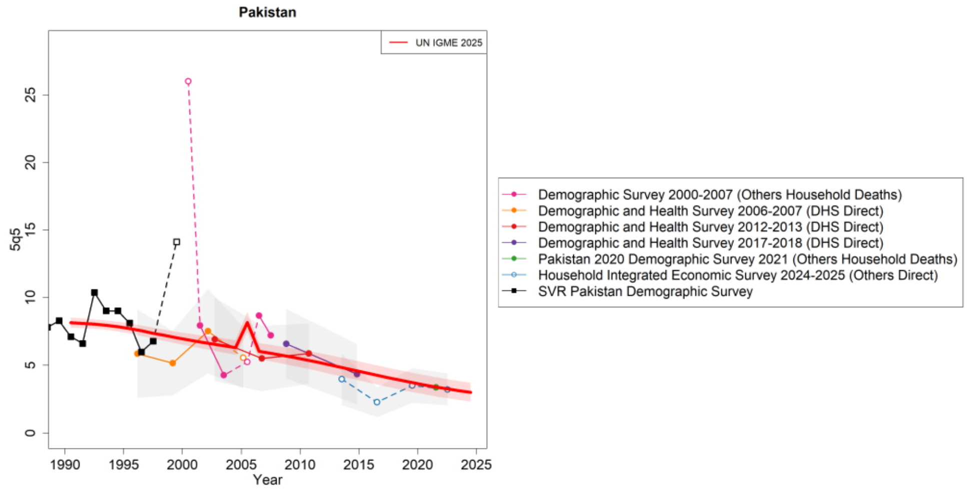

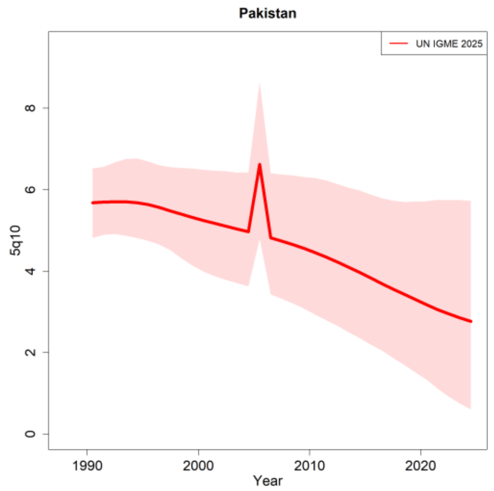

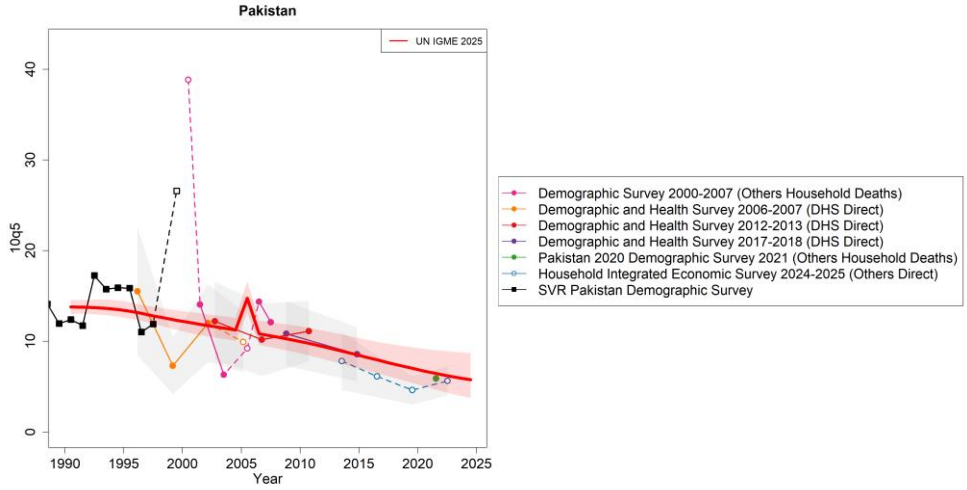

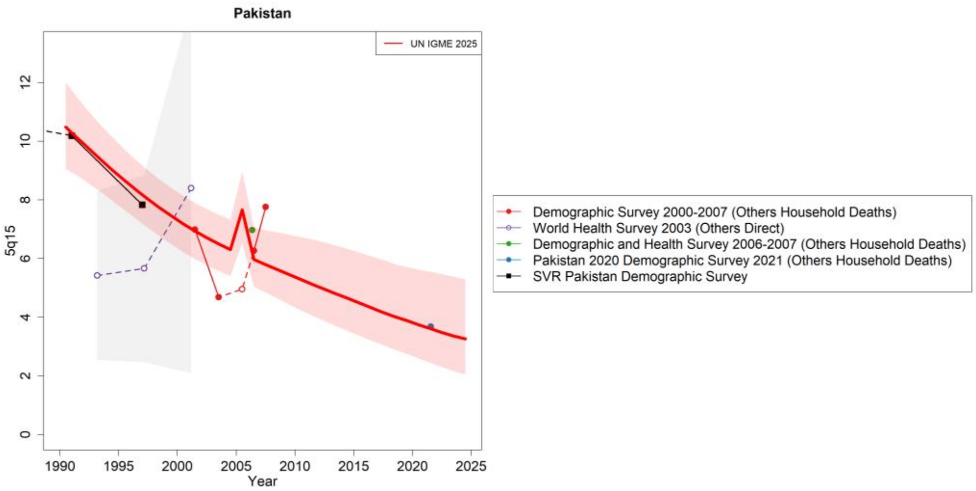
Pakistan
UN IGME 2025
5q15
Year
Demographic Survey 2000-2007 (Others Household Deaths)
World Health Survey 2003 (Others Direct)
Demographic and Health Survey 2006-2007 (Others Household Deaths)
Pakistan 2020 Demographic Survey 2021 (Others Household Deaths)
SVR Pakistan Demographic Survey

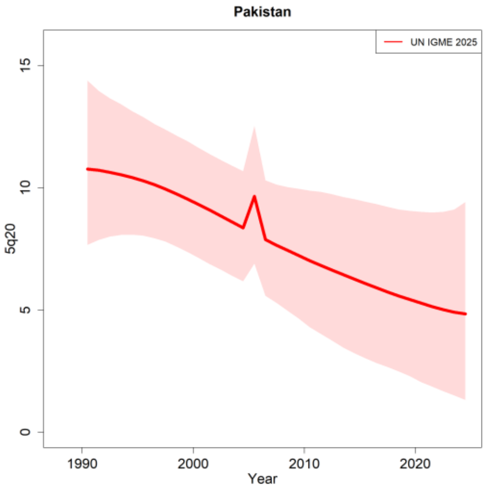
Pakistan
UN IGME 2025
5q20
Year

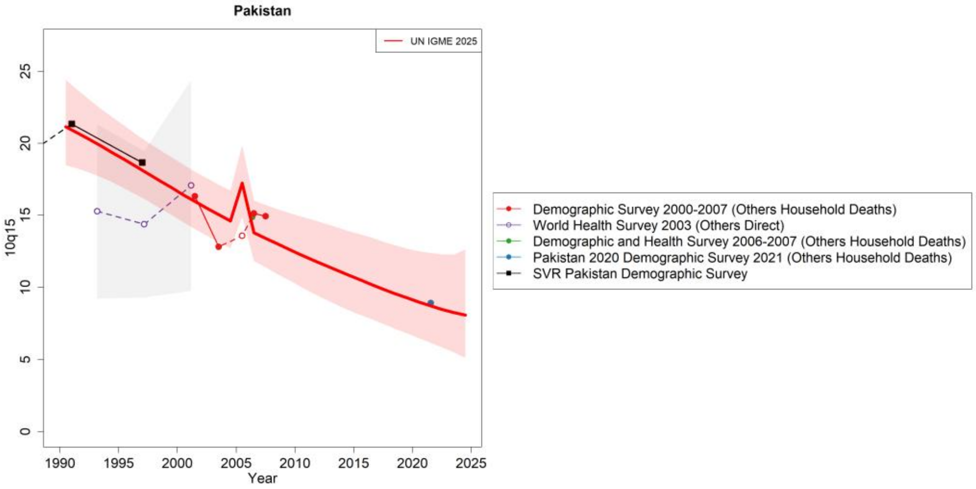
Pakistan
UN IGME 2025
10q15
Year
Demographic Survey 2000-2007 (Others Household Deaths)
World Health Survey 2003 (Others Direct)
Demographic and Health Survey 2006-2007 (Others Household Deaths)
Pakistan 2020 Demographic Survey 2021 (Others Household Deaths)
SVR Pakistan Demographic Survey

Palau (PLW)

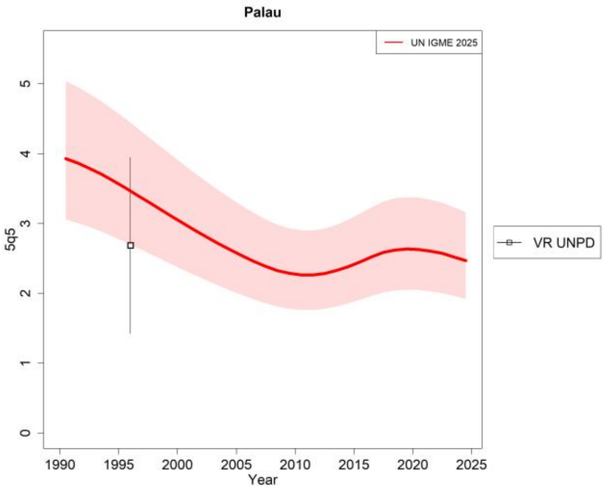
Palau
UN IGME 2025
VR UNPD
5q5
Year

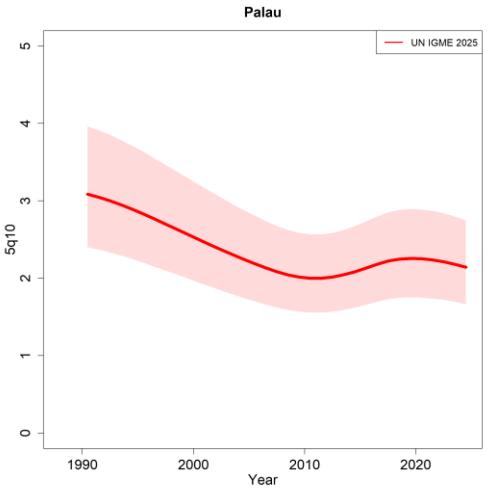
Palau
UN IGME 2025
5q10
Year

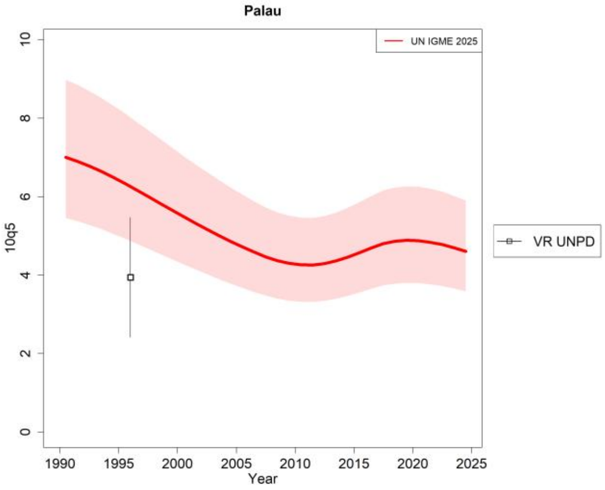
Palau
UN IGME 2025
VR UNPD
10q5
Year

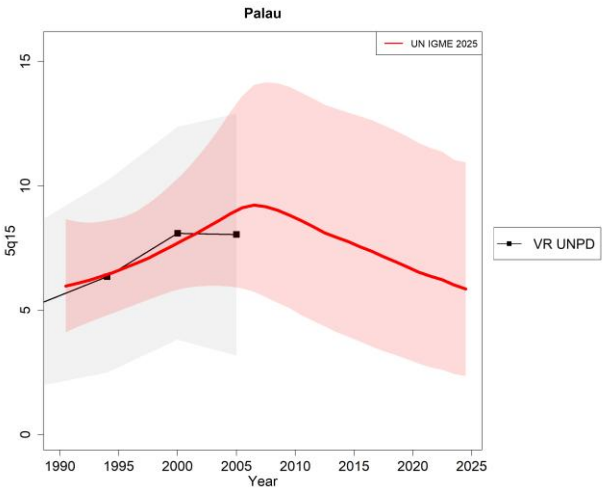
Palau
UN IGME 2025
VR UNPD
5q15
Year

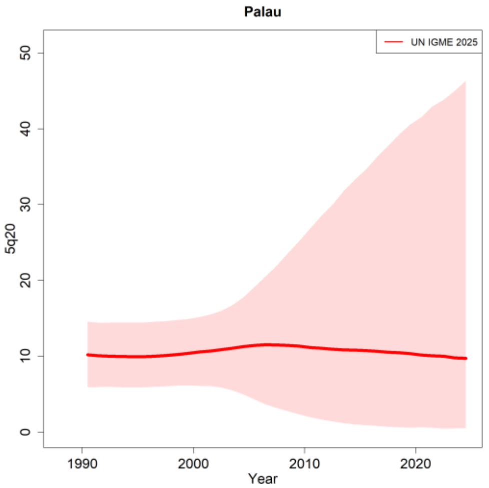
Palau
UN IGME 2025
5q20
Year

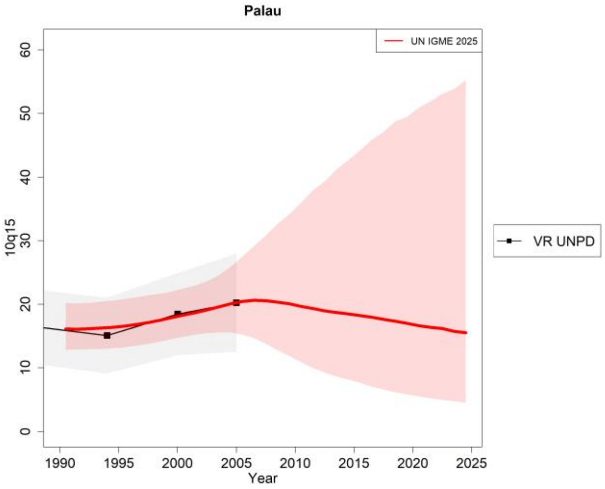
Palau
UN IGME 2025
VR UNPD
10q15
Year

Panama (PAN)

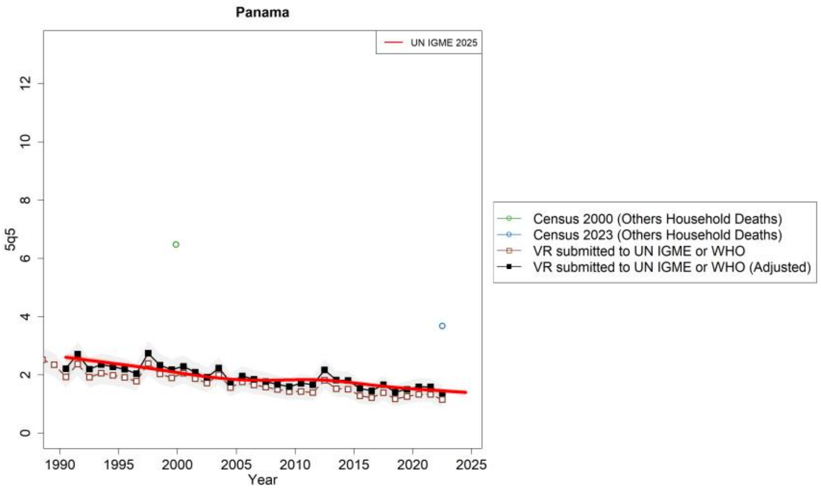
Panama
UN IGME 2025
5q5
Year
Census 2000 (Others Household Deaths)
Census 2023 (Others Household Deaths)
VR submitted to UN IGME or WHO
VR submitted to UN IGME or WHO (Adjusted)

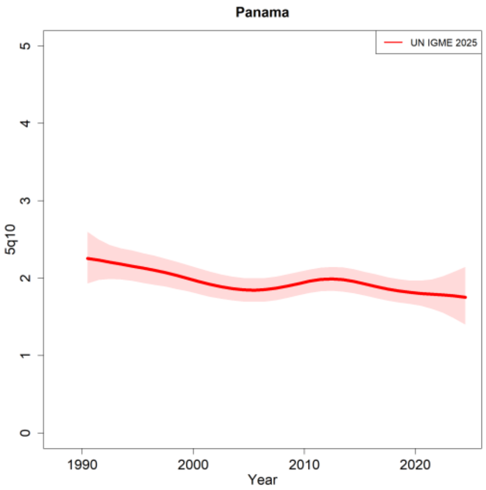
Panama
UN IGME 2025
5q10
Year

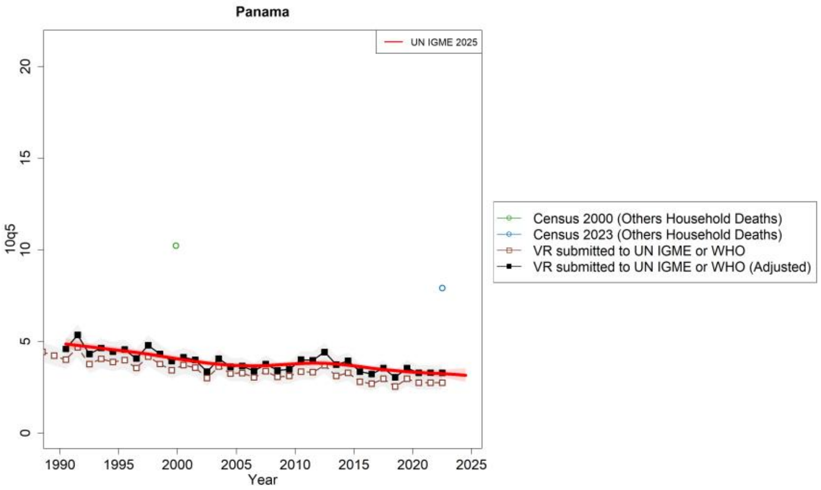
Panama
UN IGME 2025
10q5
Year
Census 2000 (Others Household Deaths)
Census 2023 (Others Household Deaths)
VR submitted to UN IGME or WHO
VR submitted to UN IGME or WHO (Adjusted)

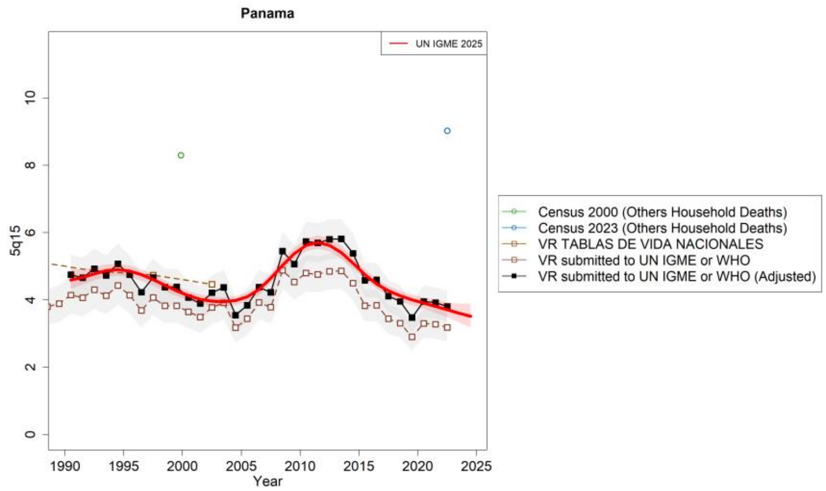
Panama
UN IGME 2025
Census 2000 (Others Household Deaths)
Census 2023 (Others Household Deaths)
VR TABLAS DE VIDA NACIONALES
VR submitted to UN IGME or WHO
VR submitted to UN IGME or WHO (Adjusted)
5q15
Year
0
2
4
6
8
10
1990
1995
2000
2005
2010
2015
2020
2025

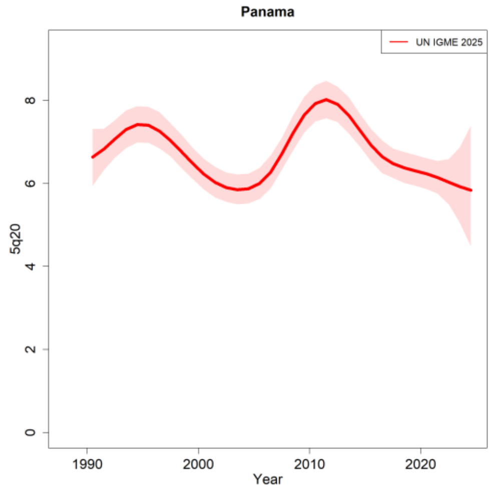
Panama
UN IGME 2025
5q20
Year
0
2
4
6
8
1990
2000
2010
2020

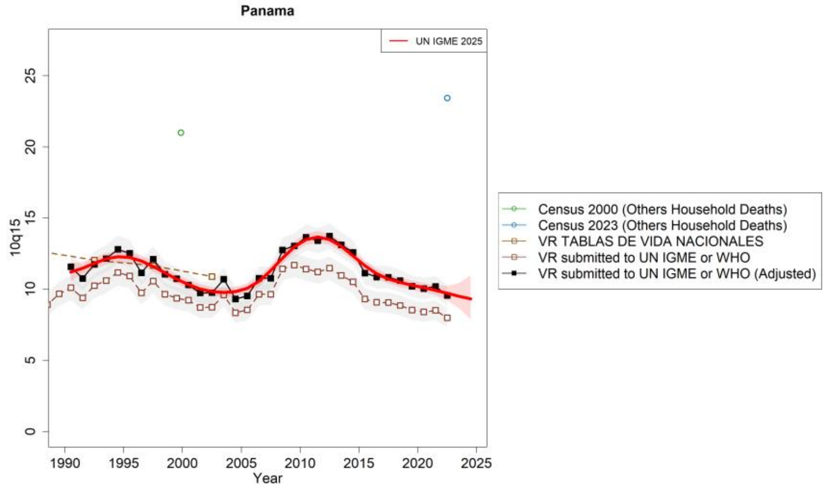
Panama
UN IGME 2025
Census 2000 (Others Household Deaths)
Census 2023 (Others Household Deaths)
VR TABLAS DE VIDA NACIONALES
VR submitted to UN IGME or WHO
VR submitted to UN IGME or WHO (Adjusted)
10q15
Year
0
5
10
15
20
25
1990
1995
2000
2005
2010
2015
2020
2025

Papua New Guinea (PNG)

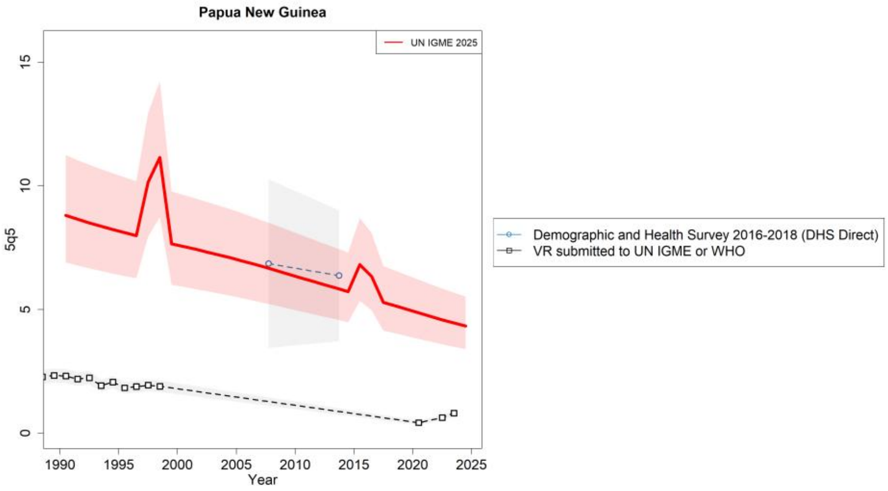

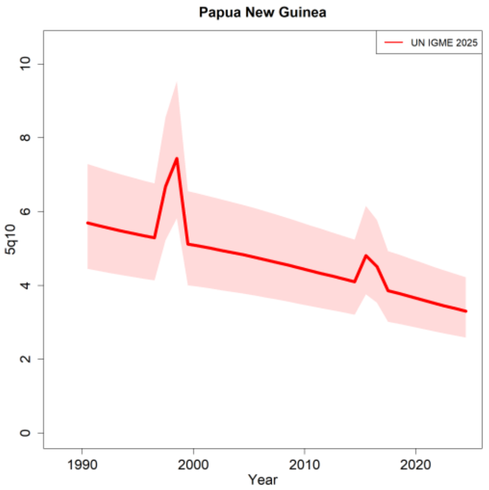

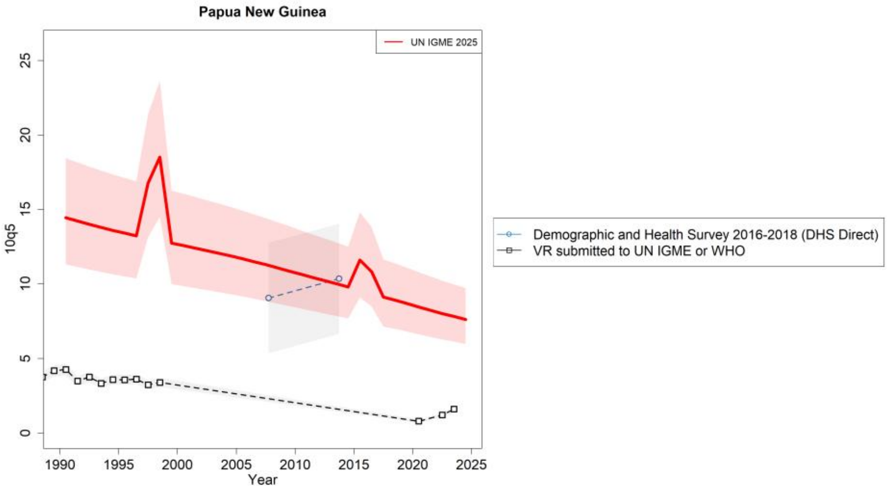

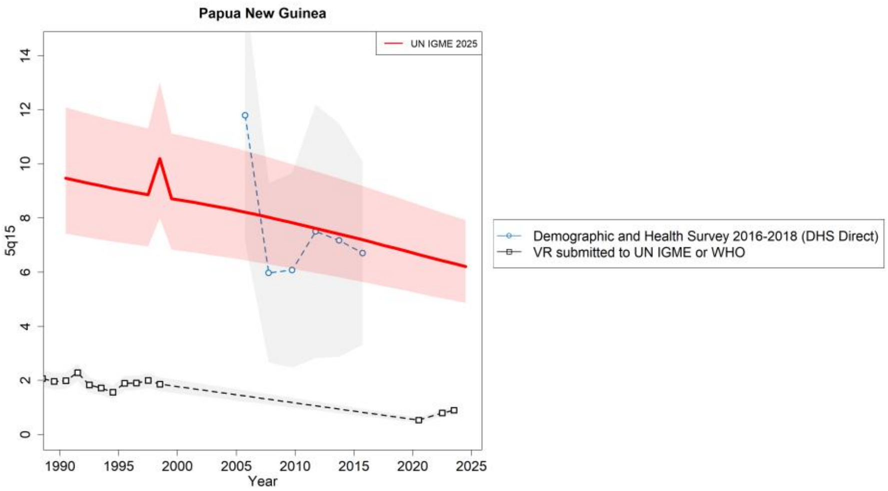
Papua New Guinea
UN IGME 2025
Demographic and Health Survey 2016-2018 (DHS Direct)
VR submitted to UN IGME or WHO
5q15
Year
0
2
4
6
8
10
12
14
1990
1995
2000
2005
2010
2015
2020
2025

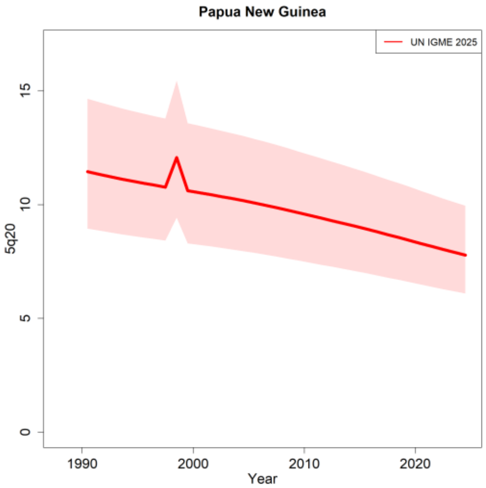
Papua New Guinea
UN IGME 2025
5q20
Year
0
5
10
15
1990
2000
2010
2020

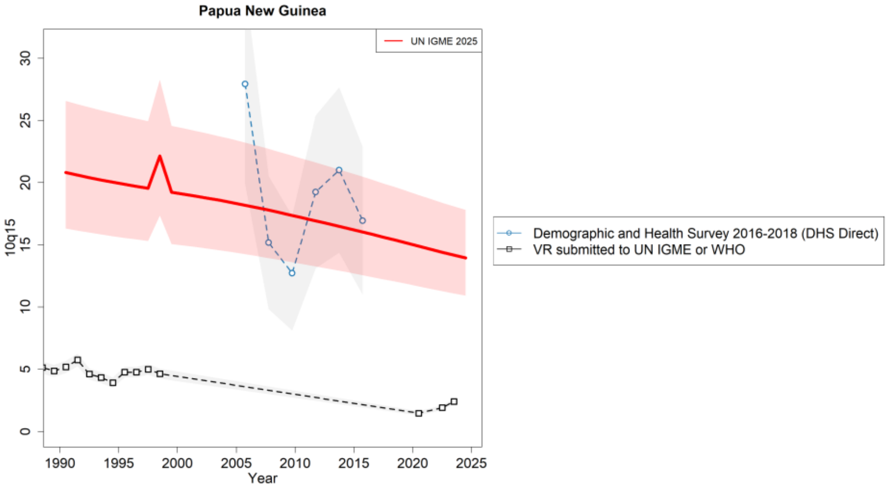
Papua New Guinea
UN IGME 2025
Demographic and Health Survey 2016-2018 (DHS Direct)
VR submitted to UN IGME or WHO
10q15
Year
0
5
10
15
20
25
30
1990
1995
2000
2005
2010
2015
2020
2025

Paraguay (PRY)

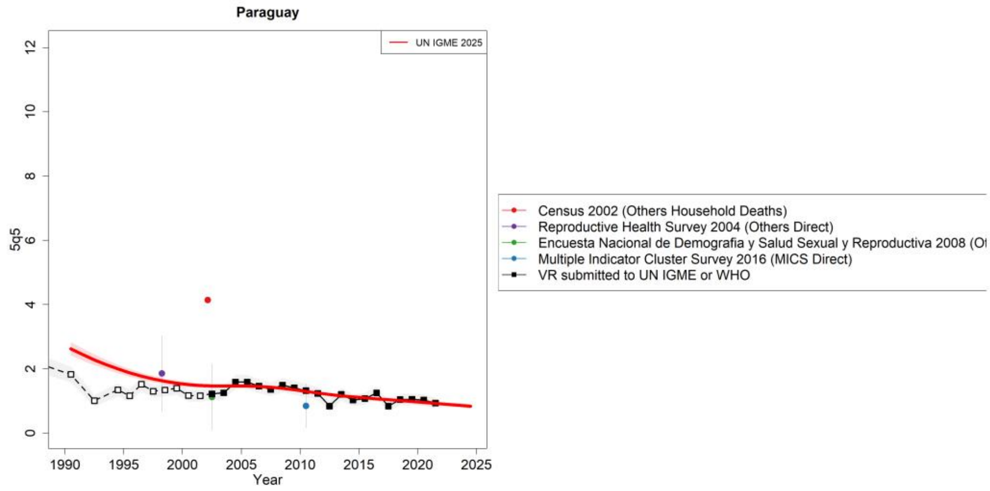

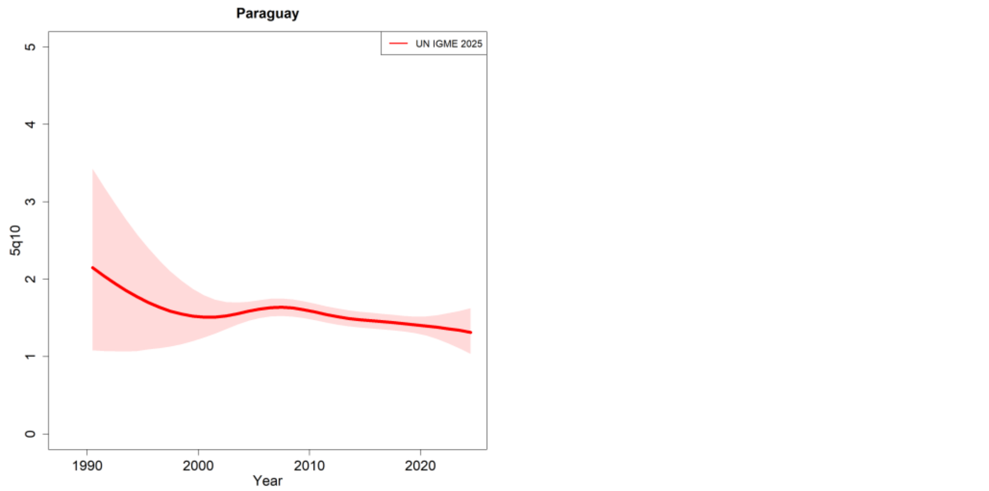

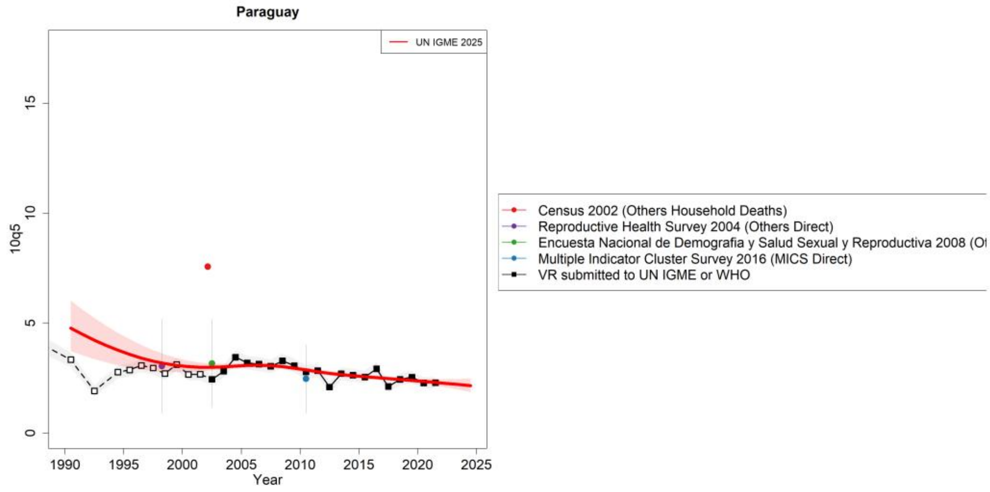

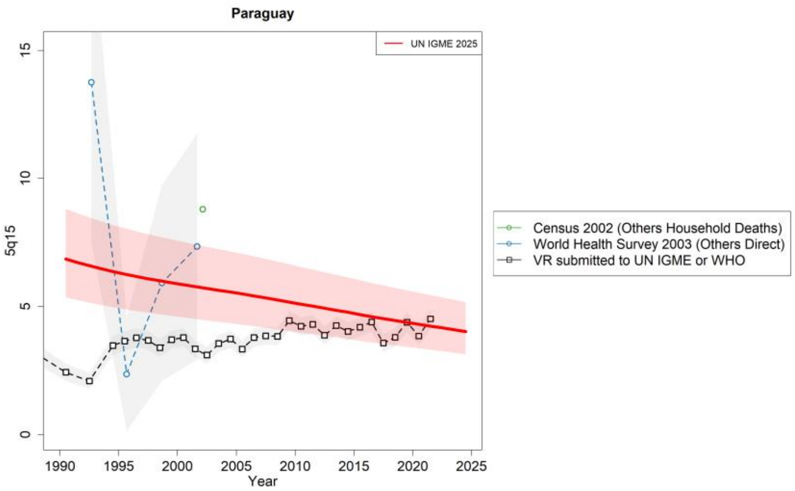
Paraguay
UN IGME 2025
Census 2002 (Others Household Deaths)
World Health Survey 2003 (Others Direct)
VR submitted to UN IGME or WHO
5q15
Year

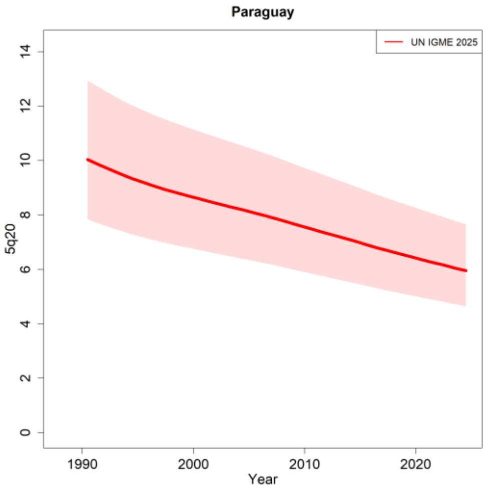
Paraguay
UN IGME 2025
5q20
Year

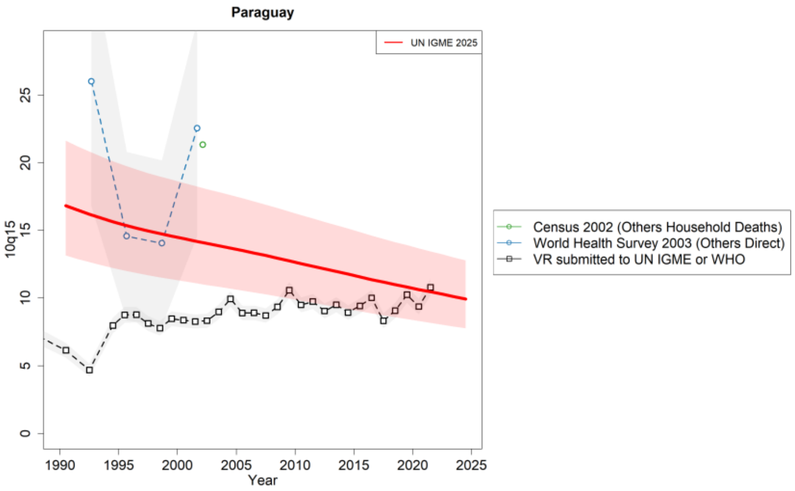
Paraguay
UN IGME 2025
Census 2002 (Others Household Deaths)
World Health Survey 2003 (Others Direct)
VR submitted to UN IGME or WHO
10q15
Year

Peru (PER)

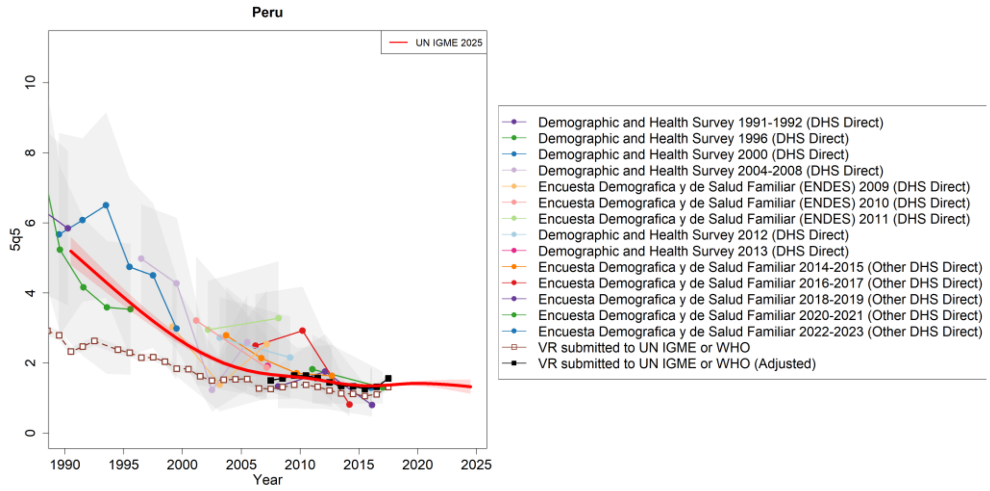
Peru
UN IGME 2025
5q5
Year
Demographic and Health Survey 1991-1992 (DHS Direct)
Demographic and Health Survey 1996 (DHS Direct)
Demographic and Health Survey 2000 (DHS Direct)
Demographic and Health Survey 2004-2008 (DHS Direct)
Encuesta Demografica y de Salud Familiar (ENDES) 2009 (DHS Direct)
Encuesta Demografica y de Salud Familiar (ENDES) 2010 (DHS Direct)
Encuesta Demografica y de Salud Familiar (ENDES) 2011 (DHS Direct)
Demographic and Health Survey 2012 (DHS Direct)
Demographic and Health Survey 2013 (DHS Direct)
Encuesta Demografica y de Salud Familiar 2014-2015 (Other DHS Direct)
Encuesta Demografica y de Salud Familiar 2016-2017 (Other DHS Direct)
Encuesta Demografica y de Salud Familiar 2018-2019 (Other DHS Direct)
Encuesta Demografica y de Salud Familiar 2020-2021 (Other DHS Direct)
Encuesta Demografica y de Salud Familiar 2022-2023 (Other DHS Direct)
VR submitted to UN IGME or WHO
VR submitted to UN IGME or WHO (Adjusted)

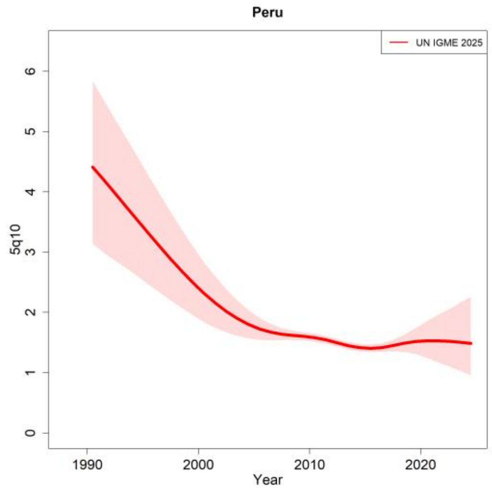
Peru
UN IGME 2025
5q10
Year

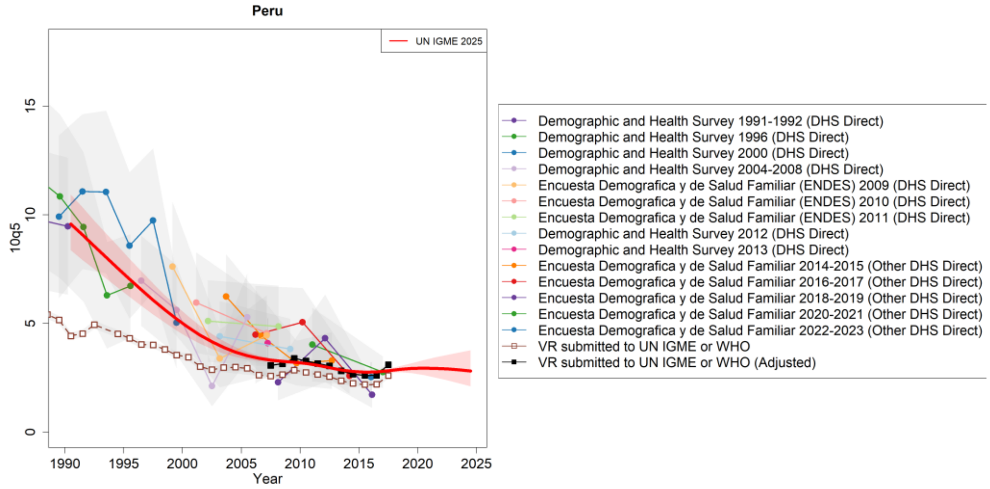
Peru
UN IGME 2025
10q5
Year
Demographic and Health Survey 1991-1992 (DHS Direct)
Demographic and Health Survey 1996 (DHS Direct)
Demographic and Health Survey 2000 (DHS Direct)
Demographic and Health Survey 2004-2008 (DHS Direct)
Encuesta Demografica y de Salud Familiar (ENDES) 2009 (DHS Direct)
Encuesta Demografica y de Salud Familiar (ENDES) 2010 (DHS Direct)
Encuesta Demografica y de Salud Familiar (ENDES) 2011 (DHS Direct)
Demographic and Health Survey 2012 (DHS Direct)
Demographic and Health Survey 2013 (DHS Direct)
Encuesta Demografica y de Salud Familiar 2014-2015 (Other DHS Direct)
Encuesta Demografica y de Salud Familiar 2016-2017 (Other DHS Direct)
Encuesta Demografica y de Salud Familiar 2018-2019 (Other DHS Direct)
Encuesta Demografica y de Salud Familiar 2020-2021 (Other DHS Direct)
Encuesta Demografica y de Salud Familiar 2022-2023 (Other DHS Direct)
VR submitted to UN IGME or WHO
VR submitted to UN IGME or WHO (Adjusted)

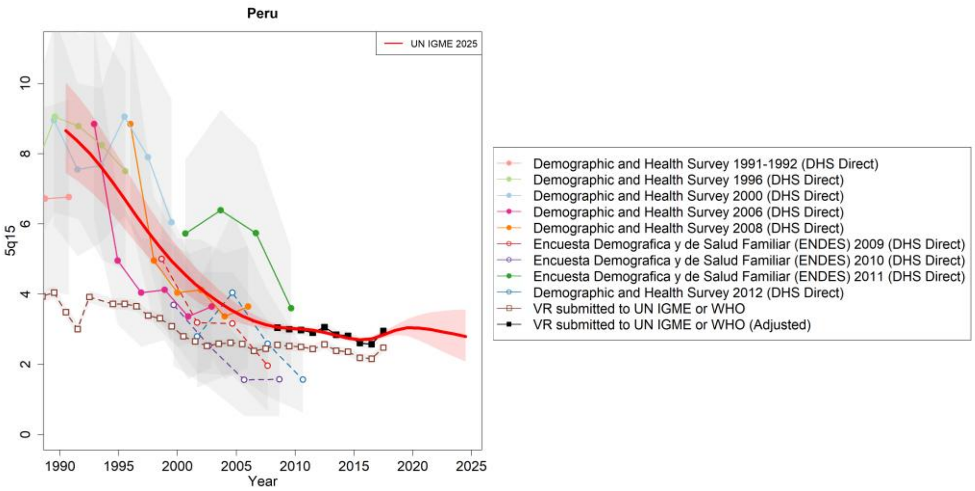
Peru
UN IGME 2025
5q15
Year
Demographic and Health Survey 1991-1992 (DHS Direct)
Demographic and Health Survey 1996 (DHS Direct)
Demographic and Health Survey 2000 (DHS Direct)
Demographic and Health Survey 2006 (DHS Direct)
Demographic and Health Survey 2008 (DHS Direct)
Encuesta Demografica y de Salud Familiar (ENDES) 2009 (DHS Direct)
Encuesta Demografica y de Salud Familiar (ENDES) 2010 (DHS Direct)
Encuesta Demografica y de Salud Familiar (ENDES) 2011 (DHS Direct)
Demographic and Health Survey 2012 (DHS Direct)
VR submitted to UN IGME or WHO
VR submitted to UN IGME or WHO (Adjusted)

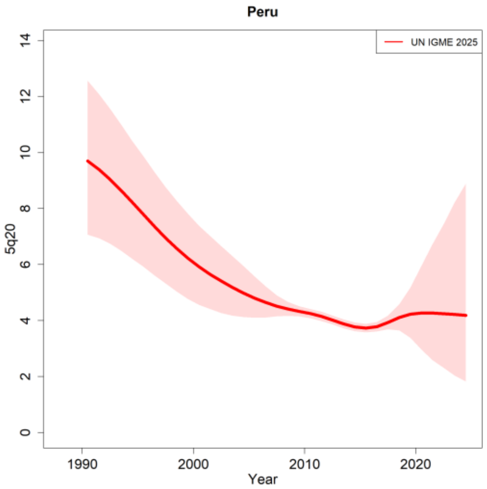
Peru
UN IGME 2025
5q20
Year

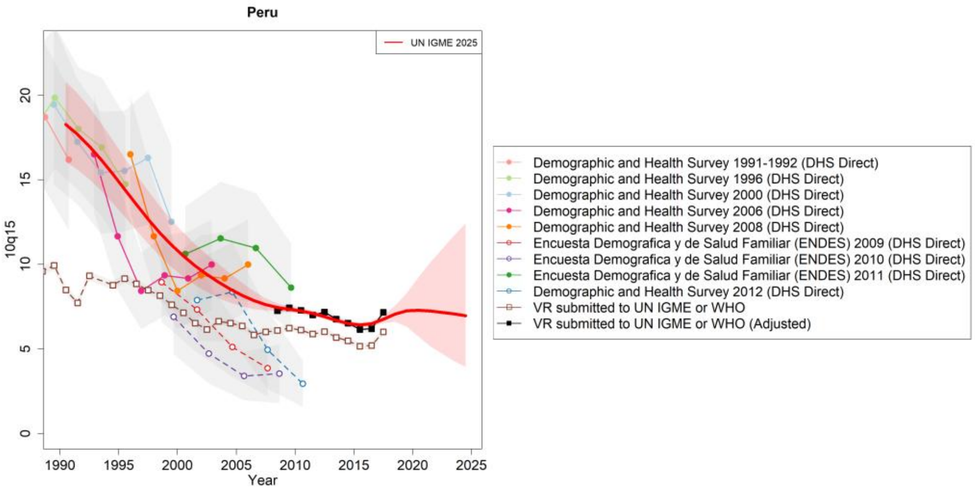
Peru
UN IGME 2025
10q15
Year
Demographic and Health Survey 1991-1992 (DHS Direct)
Demographic and Health Survey 1996 (DHS Direct)
Demographic and Health Survey 2000 (DHS Direct)
Demographic and Health Survey 2006 (DHS Direct)
Demographic and Health Survey 2008 (DHS Direct)
Encuesta Demografica y de Salud Familiar (ENDES) 2009 (DHS Direct)
Encuesta Demografica y de Salud Familiar (ENDES) 2010 (DHS Direct)
Encuesta Demografica y de Salud Familiar (ENDES) 2011 (DHS Direct)
Demographic and Health Survey 2012 (DHS Direct)
VR submitted to UN IGME or WHO
VR submitted to UN IGME or WHO (Adjusted)

Philippines (PHL)

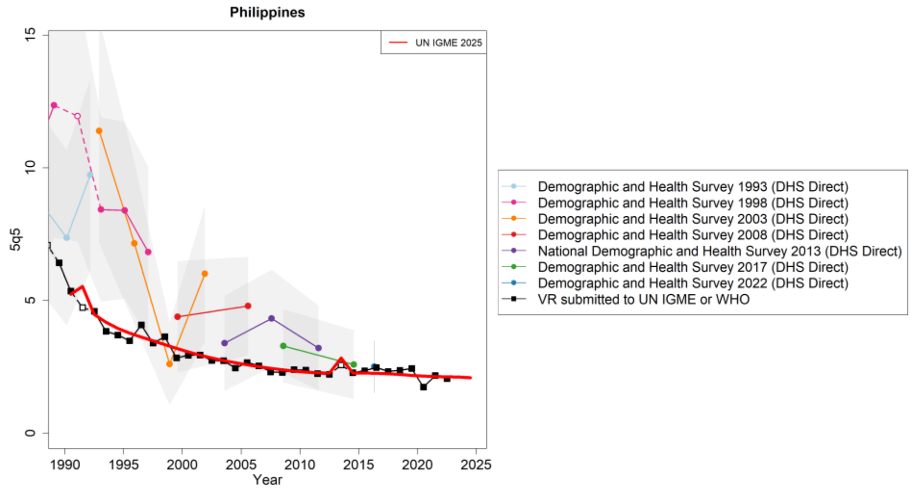

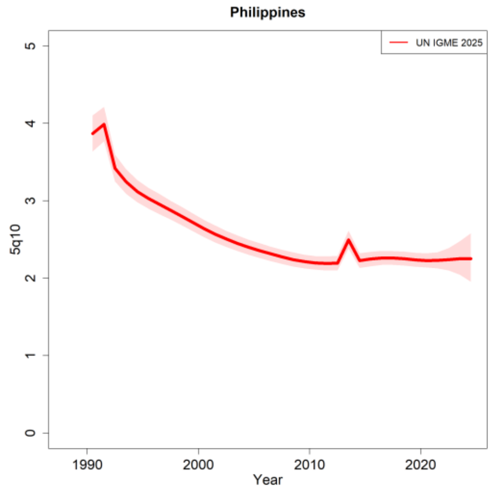

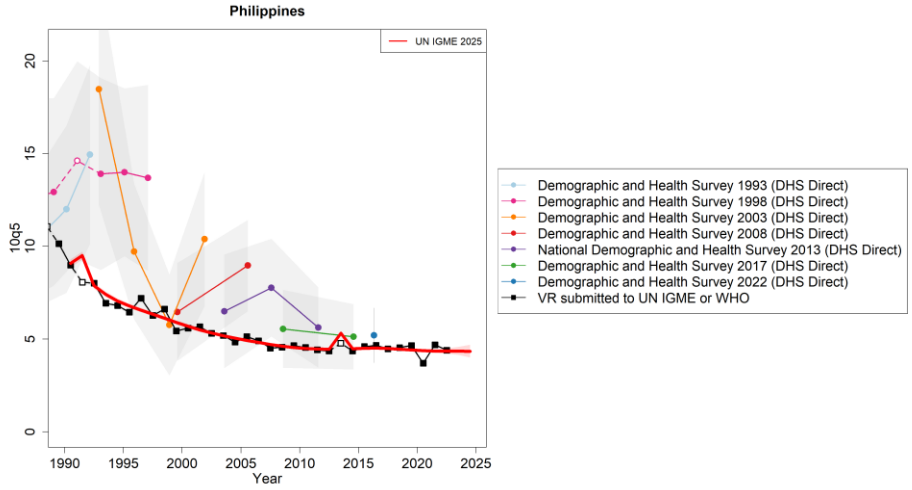

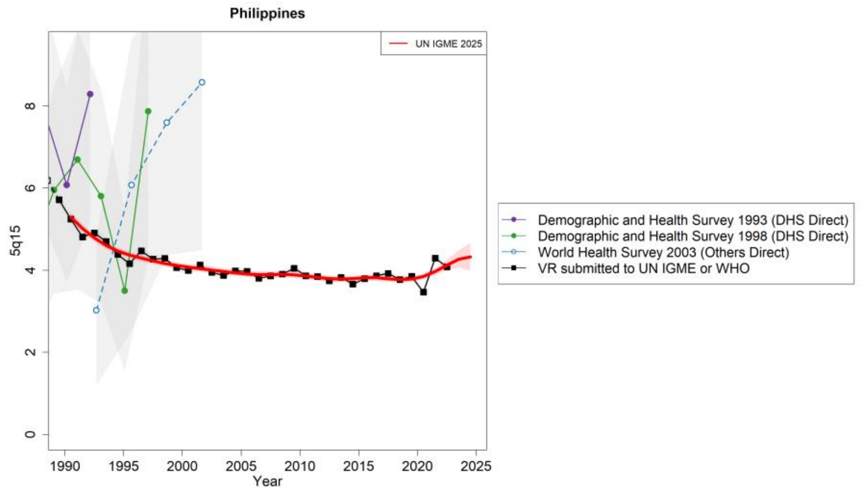
Philippines
UN IGME 2025
Demographic and Health Survey 1993 (DHS Direct)
Demographic and Health Survey 1998 (DHS Direct)
World Health Survey 2003 (Others Direct)
VR submitted to UN IGME or WHO
5q15
Year

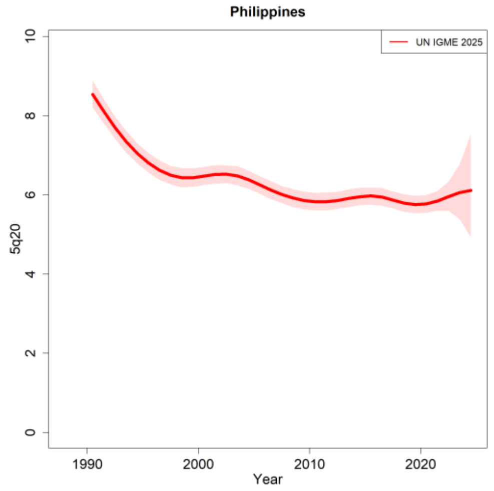
Philippines
UN IGME 2025
5q20
Year

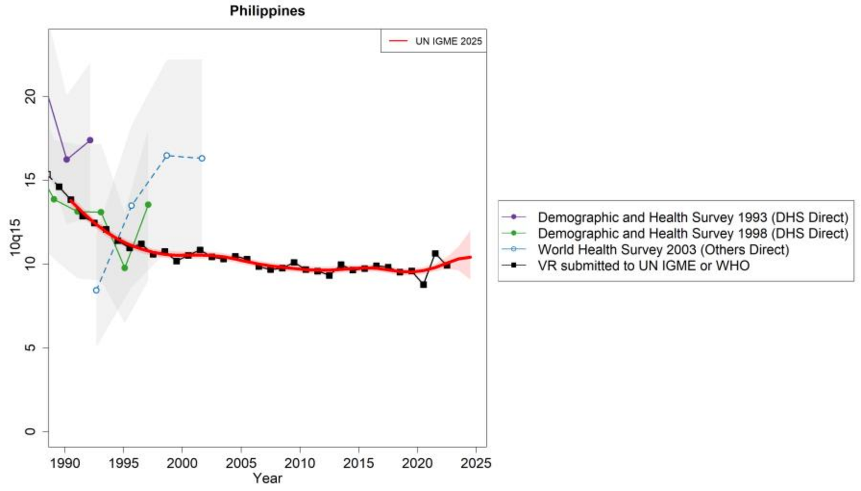
Philippines
UN IGME 2025
Demographic and Health Survey 1993 (DHS Direct)
Demographic and Health Survey 1998 (DHS Direct)
World Health Survey 2003 (Others Direct)
VR submitted to UN IGME or WHO
10q15
Year

Poland (POL)

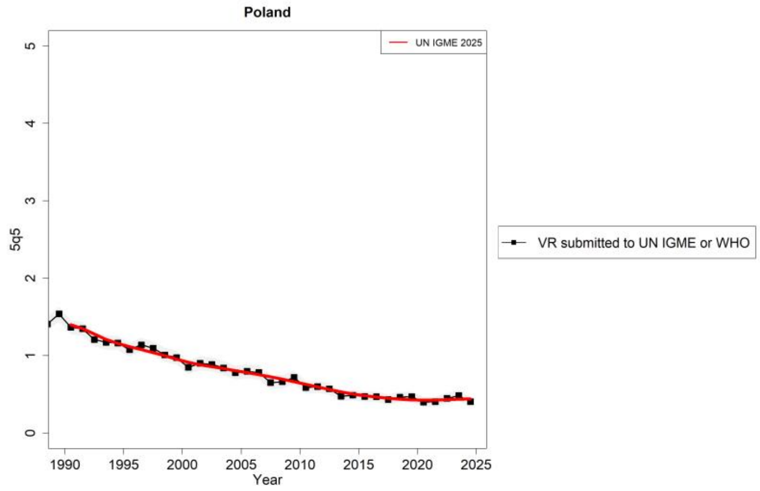
Poland
UN IGME 2025
VR submitted to UN IGME or WHO
5q5
Year

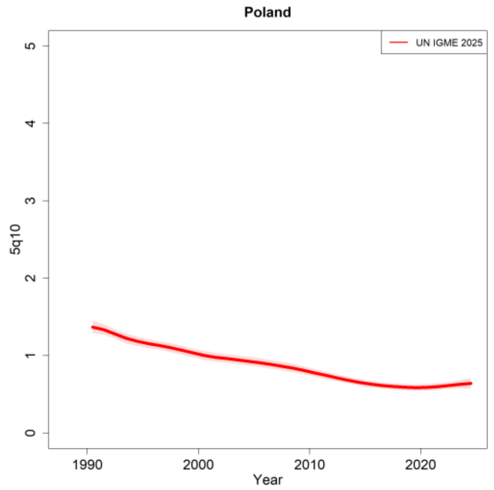
Poland
UN IGME 2025
5q10
Year

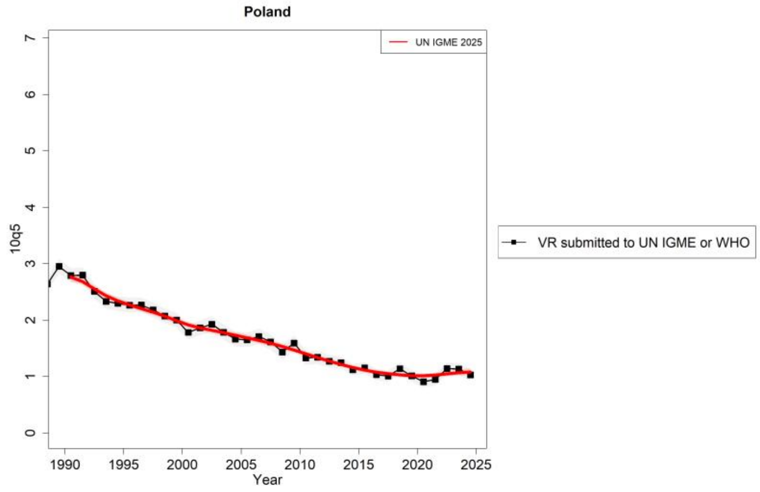
Poland
UN IGME 2025
VR submitted to UN IGME or WHO
10q5
Year

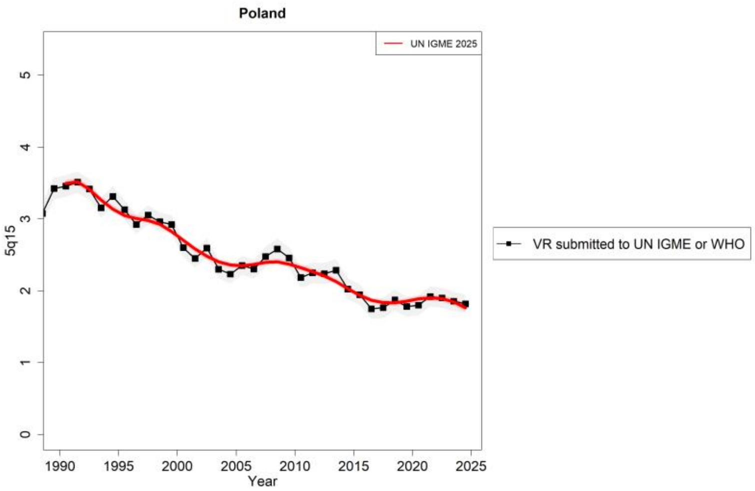
Poland
UN IGME 2025
VR submitted to UN IGME or WHO
5q15
Year

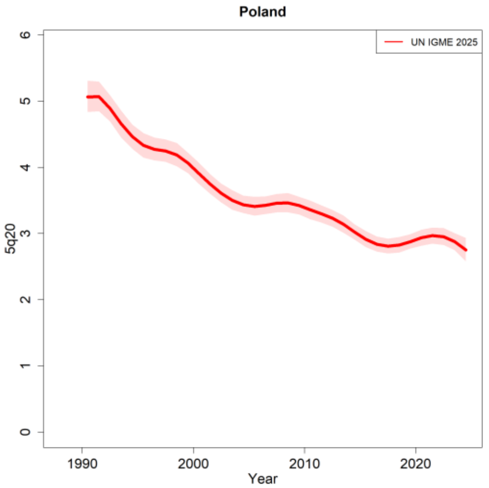
Poland
UN IGME 2025
5q20
Year

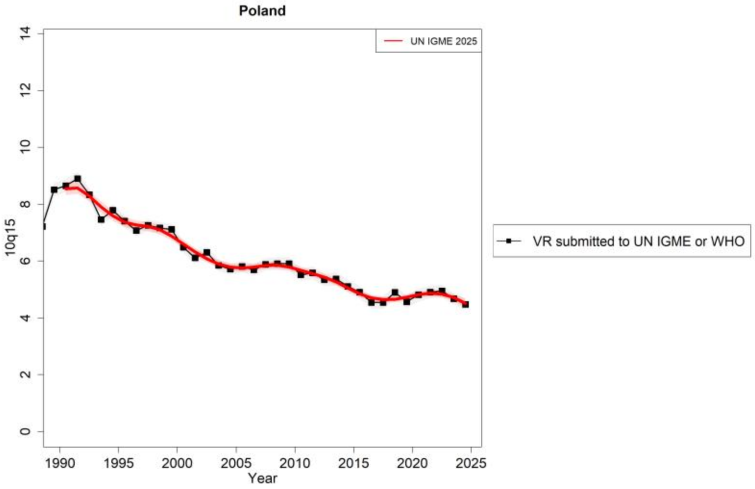
Poland
UN IGME 2025
VR submitted to UN IGME or WHO
10q15
Year

Portugal (PRT)

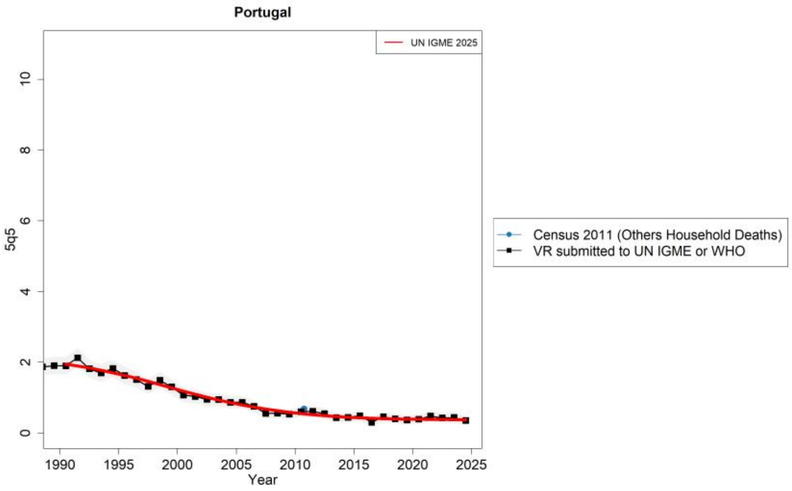

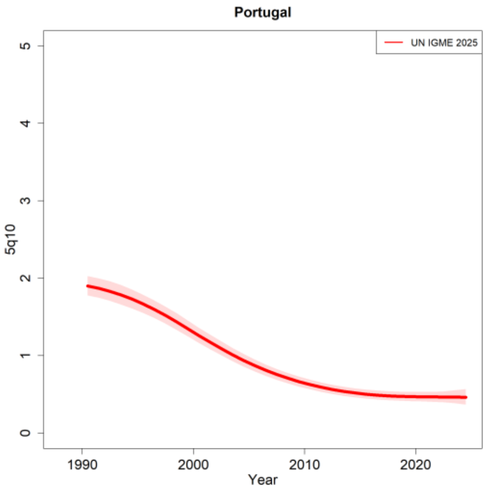

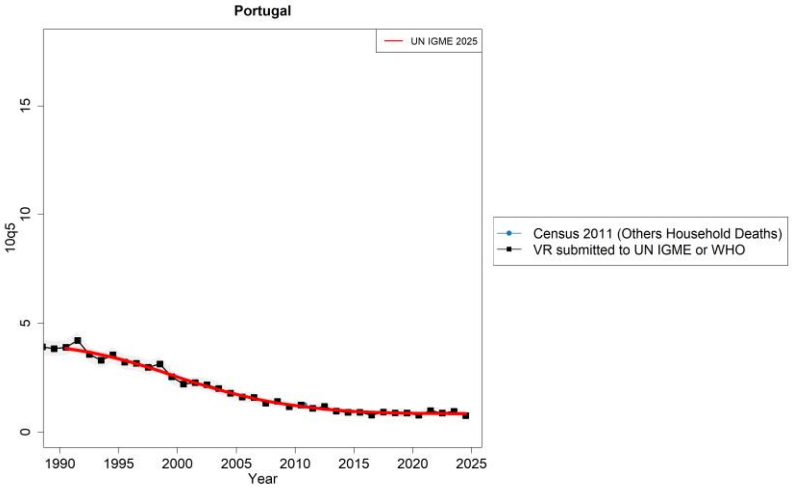

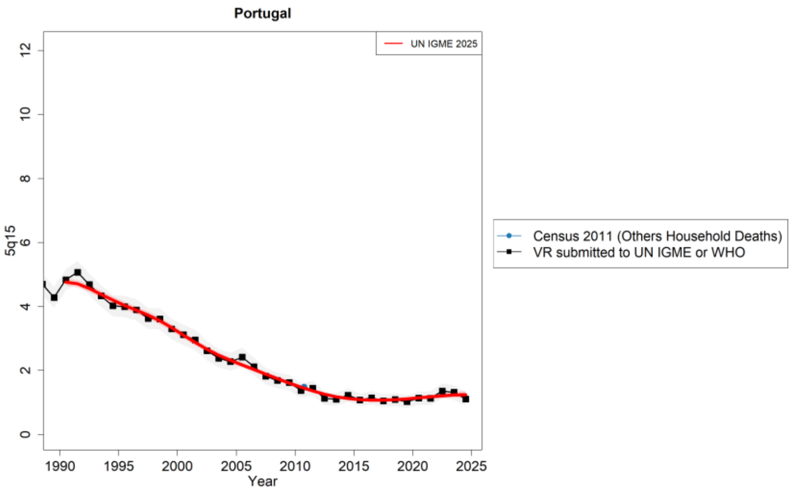
Portugal
UN IGME 2025
5q15
0
2
4
6
8
10
12
1990
1995
2000
2005
2010
2015
2020
2025
Year
Census 2011 (Others Household Deaths)
VR submitted to UN IGME or WHO

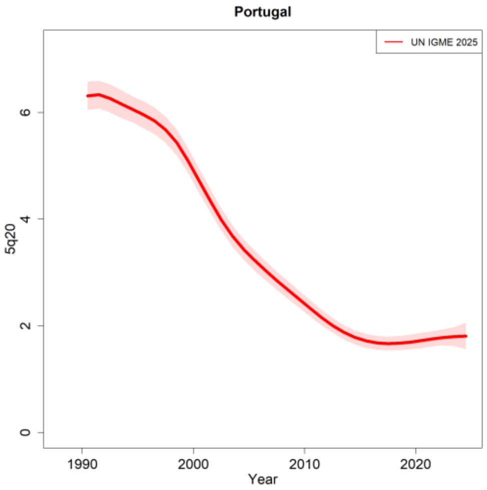
Portugal
UN IGME 2025
5q20
0
2
4
6
1990
2000
2010
2020
Year

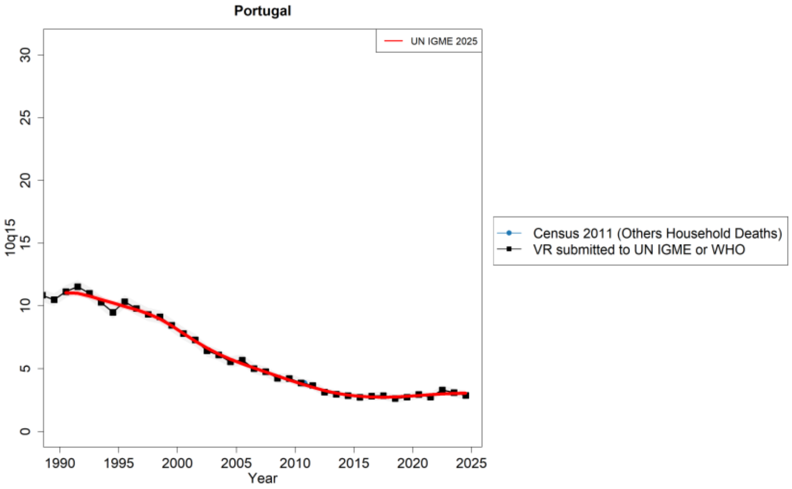
Portugal
UN IGME 2025
10q15
0
5
10
15
20
25
30
1990
1995
2000
2005
2010
2015
2020
2025
Year
Census 2011 (Others Household Deaths)
VR submitted to UN IGME or WHO

Qatar (QAT)

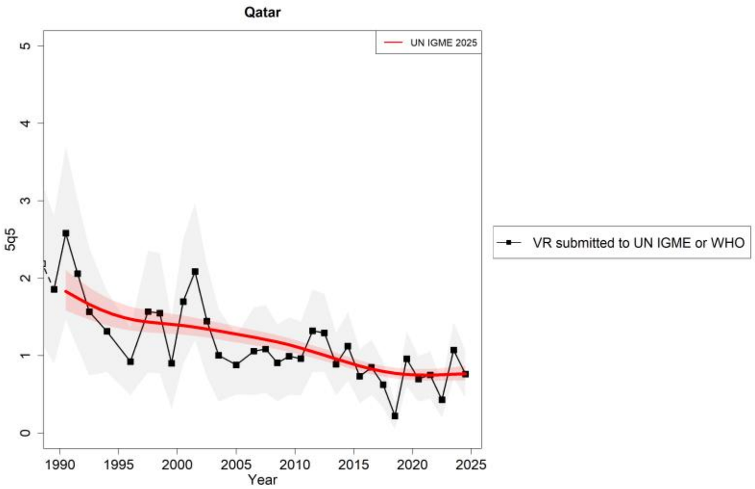
Qatar
UN IGME 2025
VR submitted to UN IGME or WHO
5q5
Year

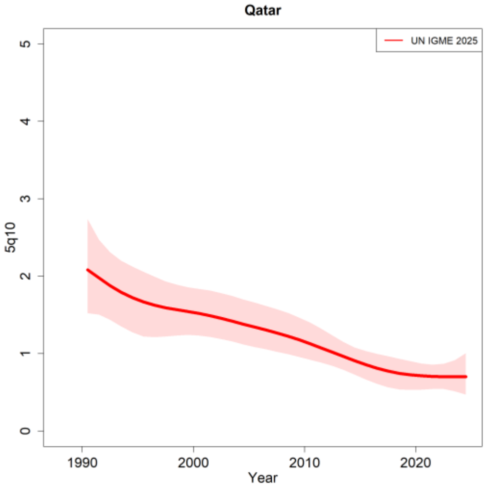
Qatar
UN IGME 2025
5q10
Year

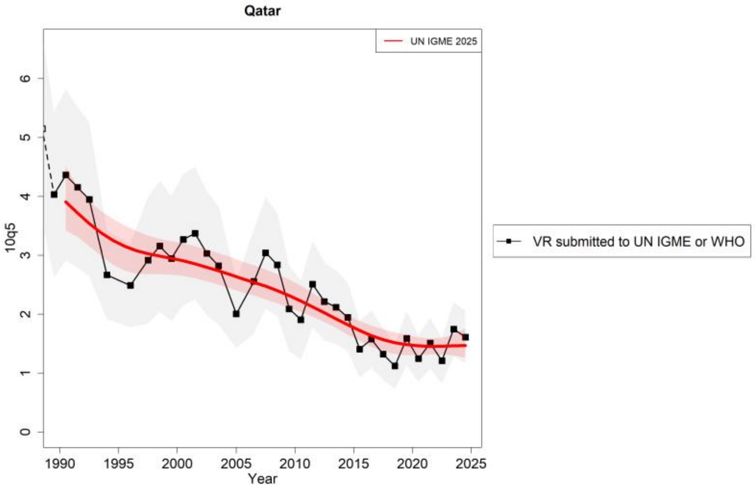
Qatar
UN IGME 2025
VR submitted to UN IGME or WHO
10q5
Year

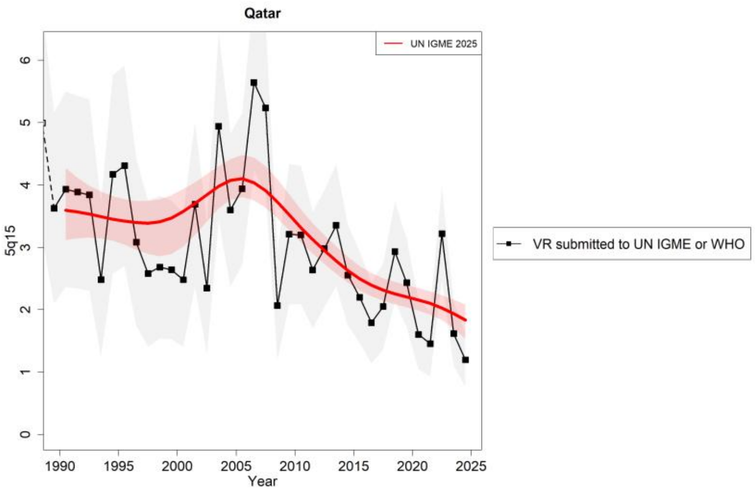
Qatar
UN IGME 2025
VR submitted to UN IGME or WHO
5q15
Year

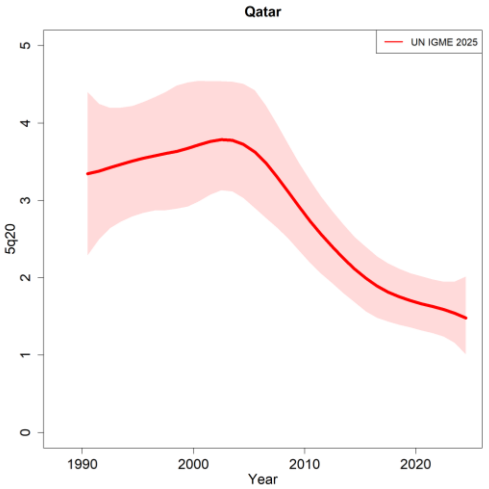
Qatar
UN IGME 2025
5q20
Year

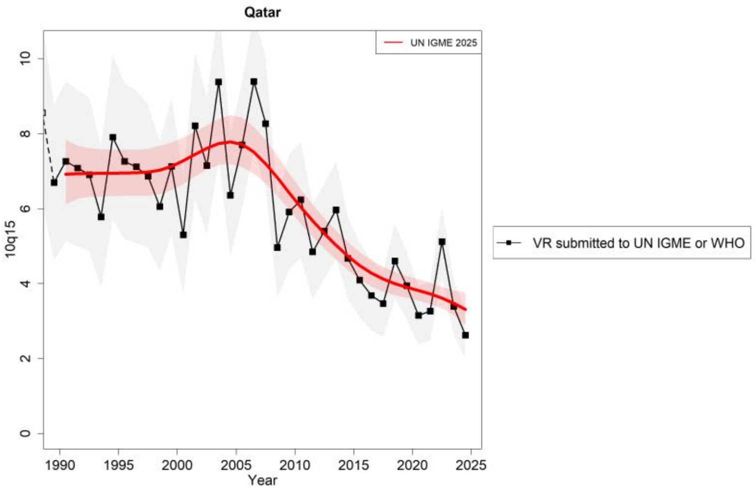
Qatar
UN IGME 2025
VR submitted to UN IGME or WHO
10q15
Year

Republic of Korea (KOR)

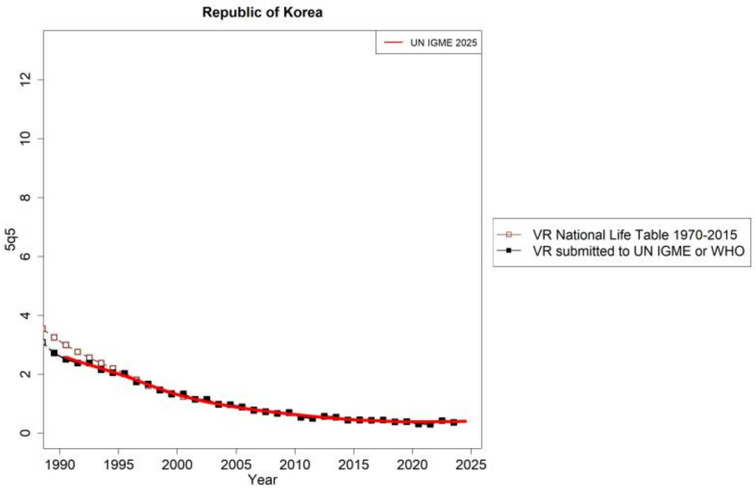

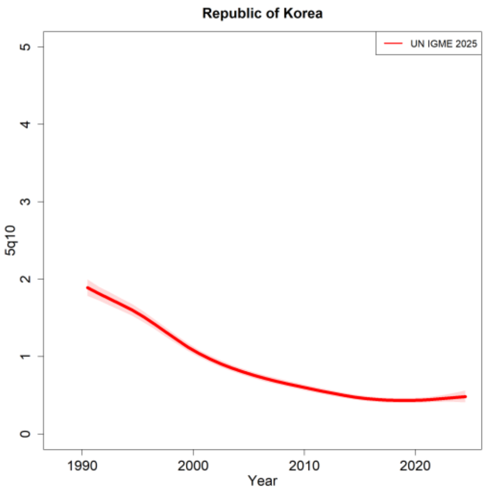

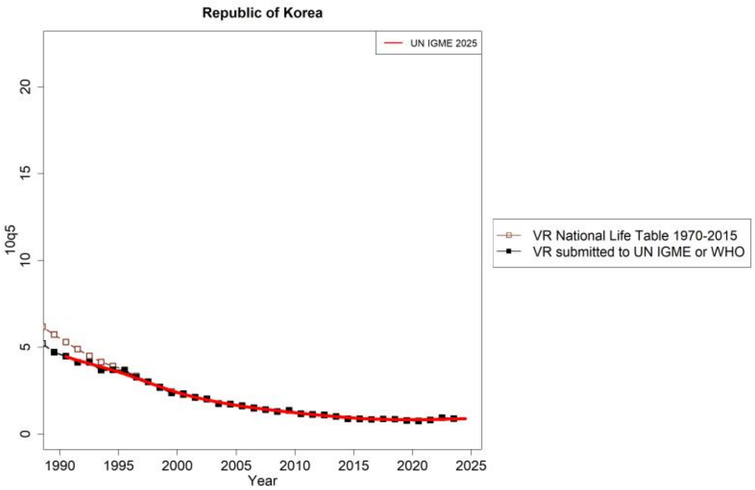

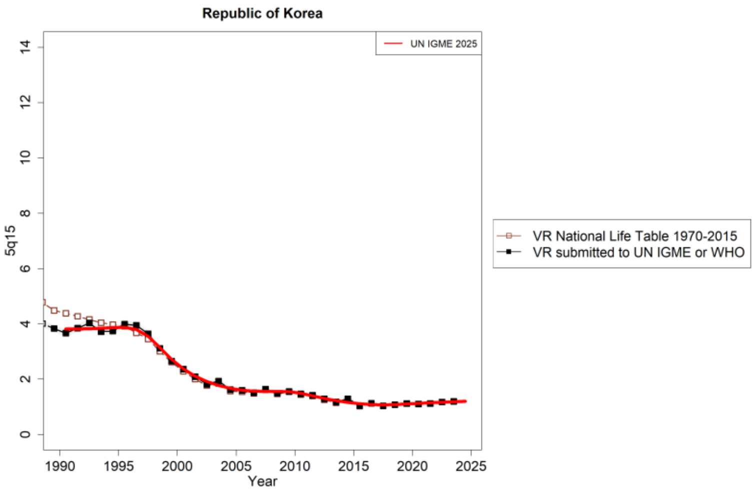
Republic of Korea
UN IGME 2025
VR National Life Table 1970-2015
VR submitted to UN IGME or WHO
5q15
Year

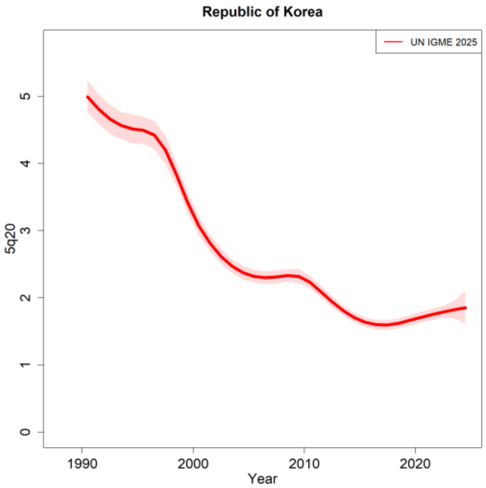
Republic of Korea
UN IGME 2025
5q20
Year

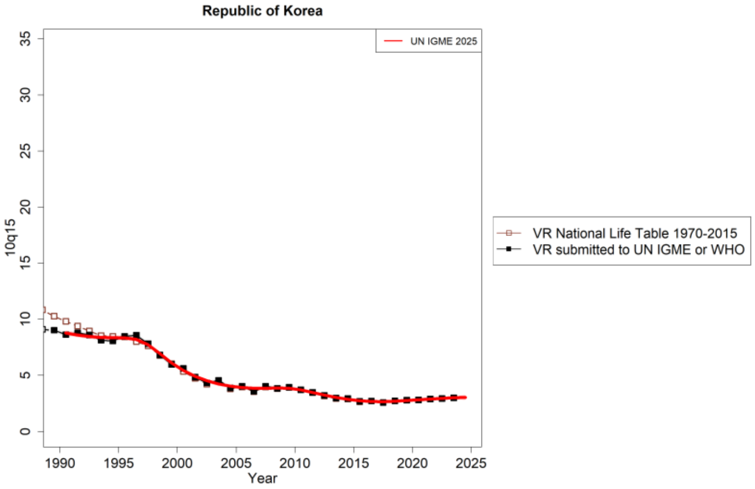
Republic of Korea
UN IGME 2025
VR National Life Table 1970-2015
VR submitted to UN IGME or WHO
10q15
Year

Republic of Moldova (MDA)

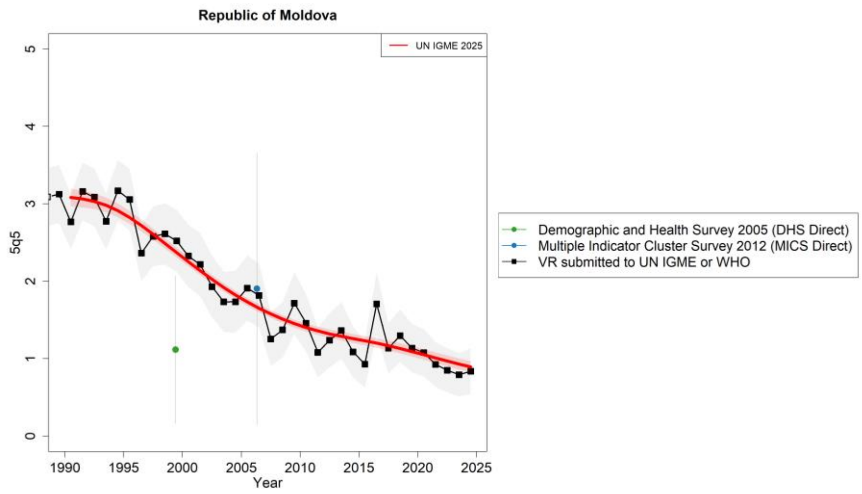

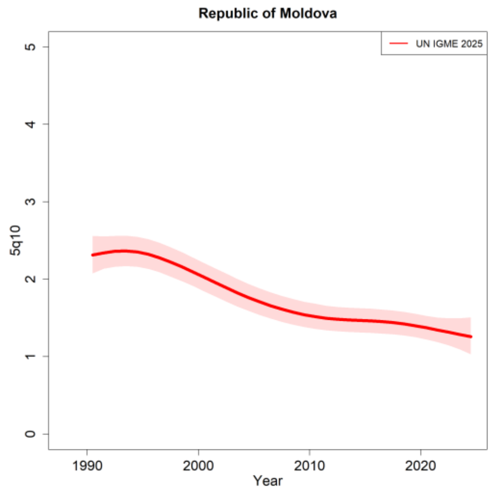

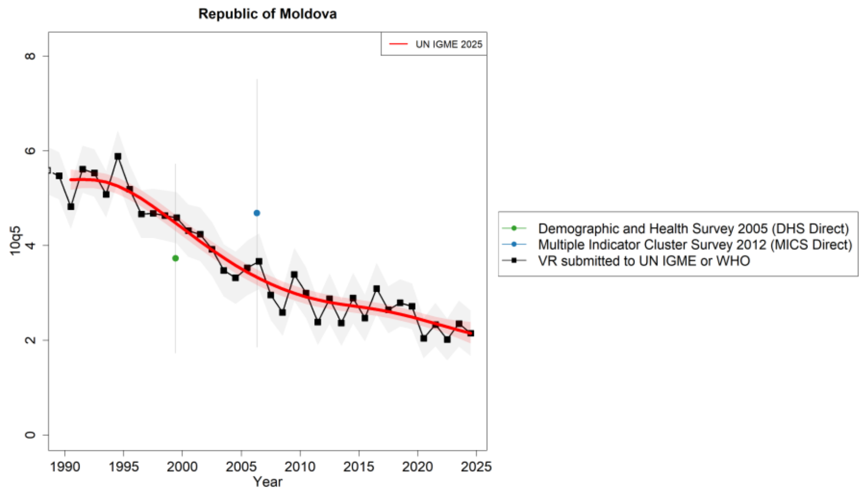

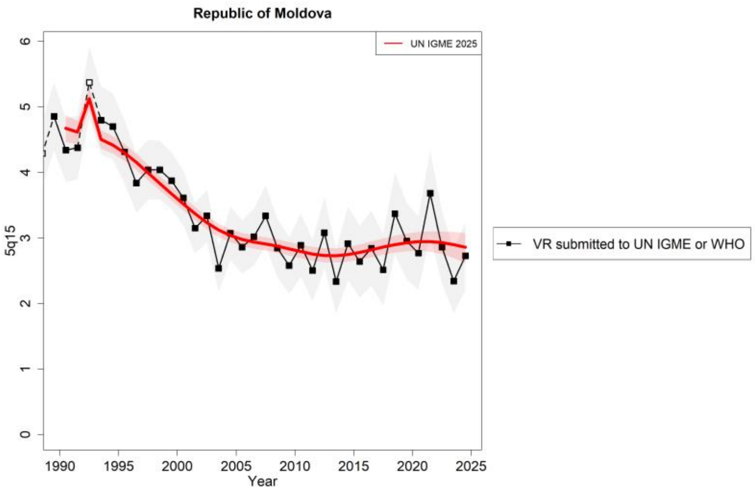

Republic of Moldova
UN IGME 2025
VR submitted to UN IGME or WHO
5q15
Year

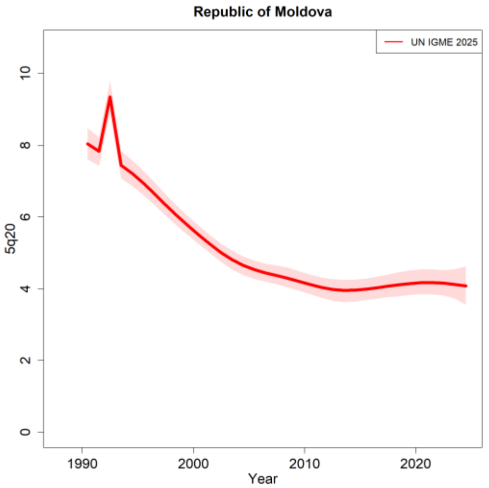

Republic of Moldova
UN IGME 2025
5q20
Year

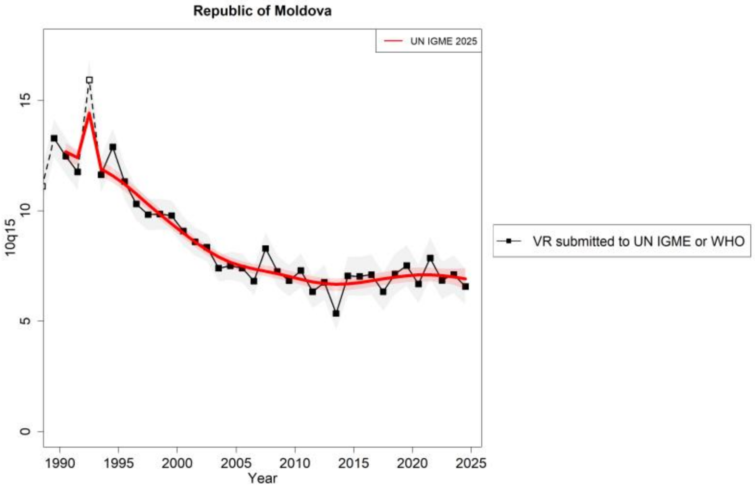

Republic of Moldova
UN IGME 2025
VR submitted to UN IGME or WHO
10q15
Year

Romania (ROU)

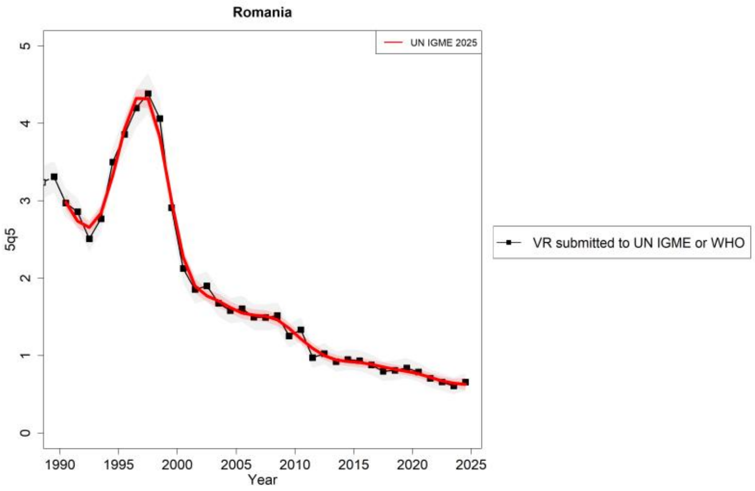
Romania
UN IGME 2025
VR submitted to UN IGME or WHO
5q5
Year

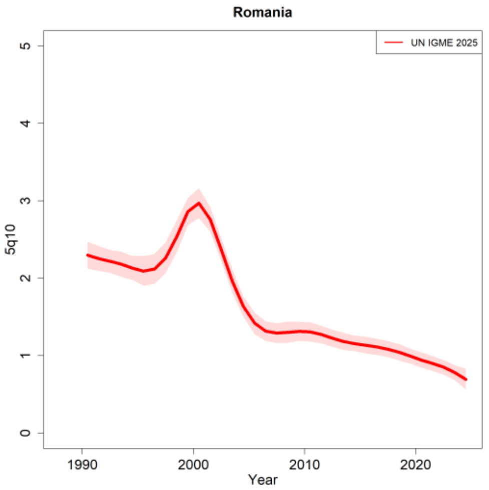
Romania
UN IGME 2025
5q10
Year

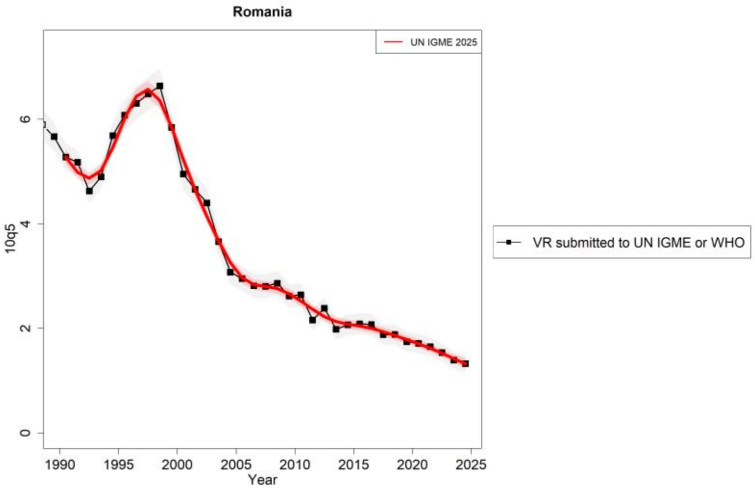
Romania
UN IGME 2025
VR submitted to UN IGME or WHO
10q5
Year

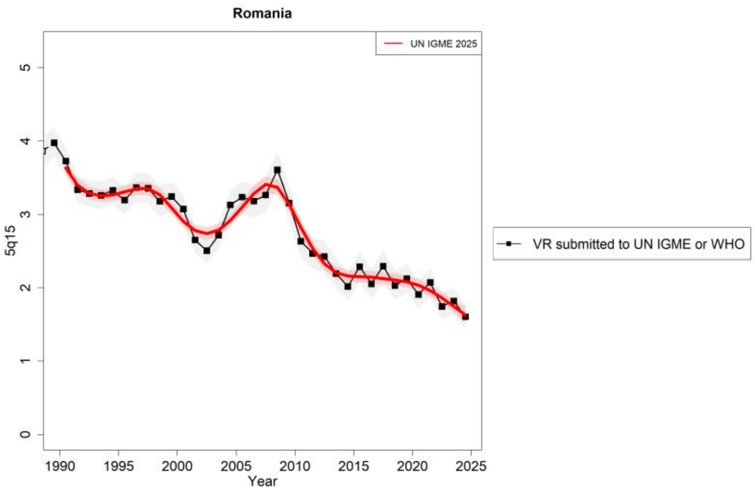
Romania
UN IGME 2025
VR submitted to UN IGME or WHO
5q15
Year

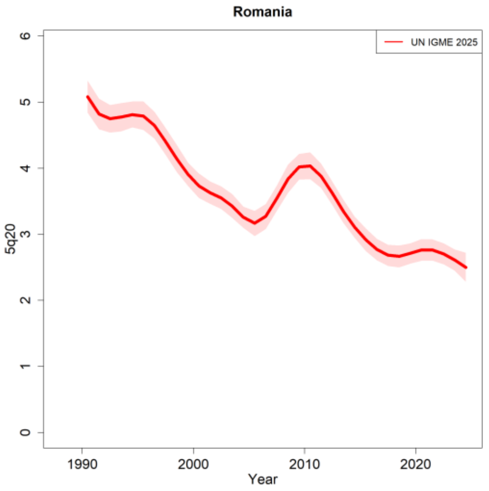
Romania
UN IGME 2025
5q20
Year

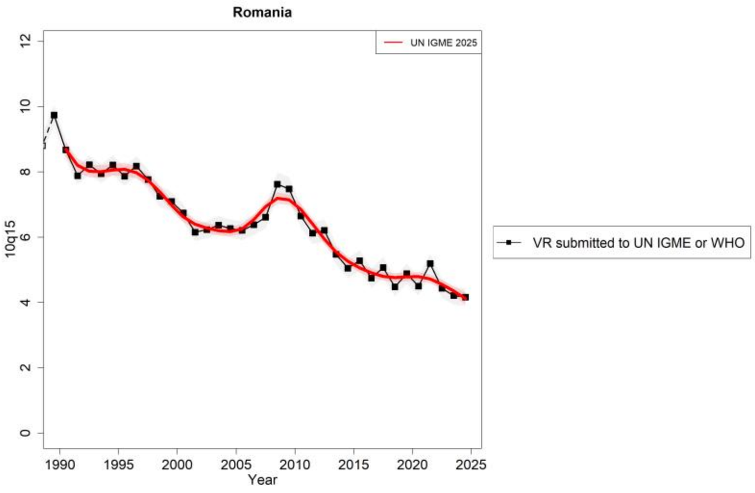
Romania
UN IGME 2025
VR submitted to UN IGME or WHO
10q15
Year

Russian Federation (RUS)

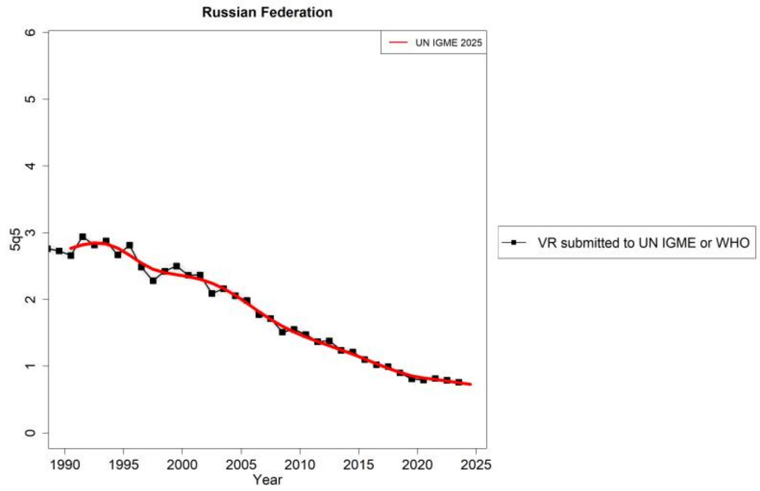

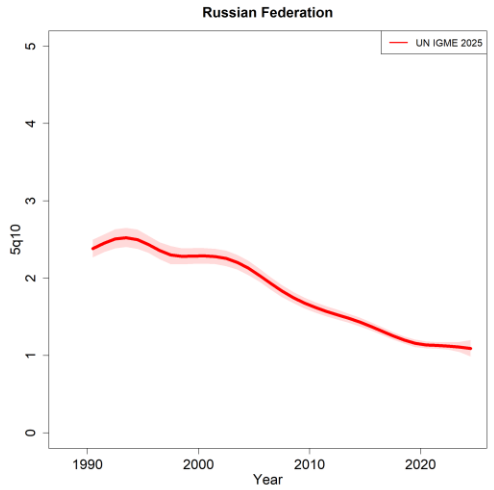

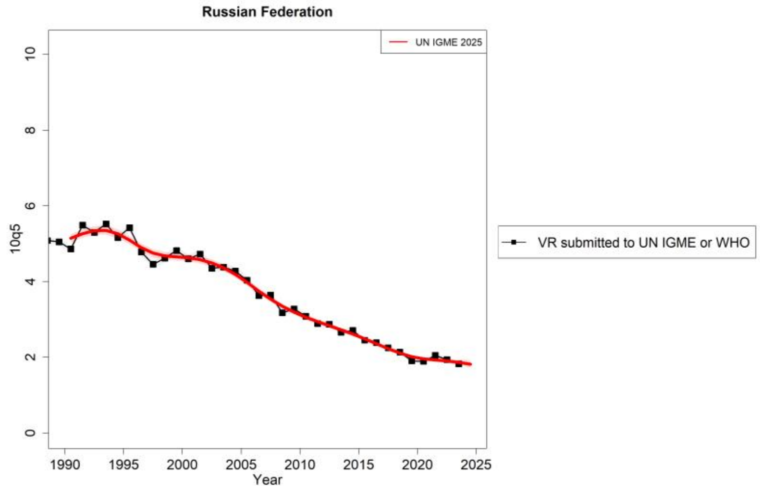

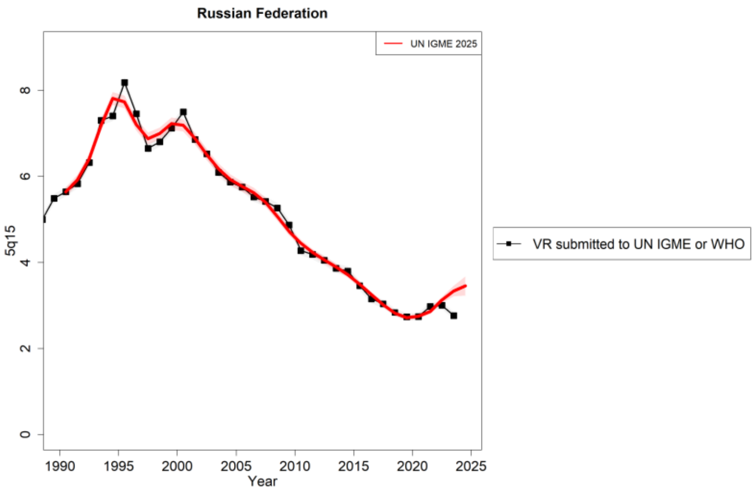
Russian Federation
UN IGME 2025
VR submitted to UN IGME or WHO
5q15
Year

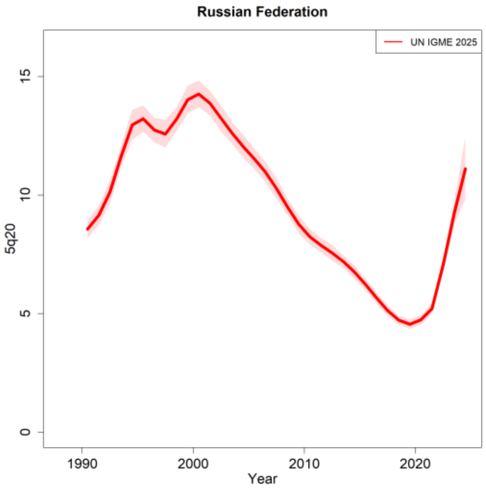
Russian Federation
UN IGME 2025
5q20
Year

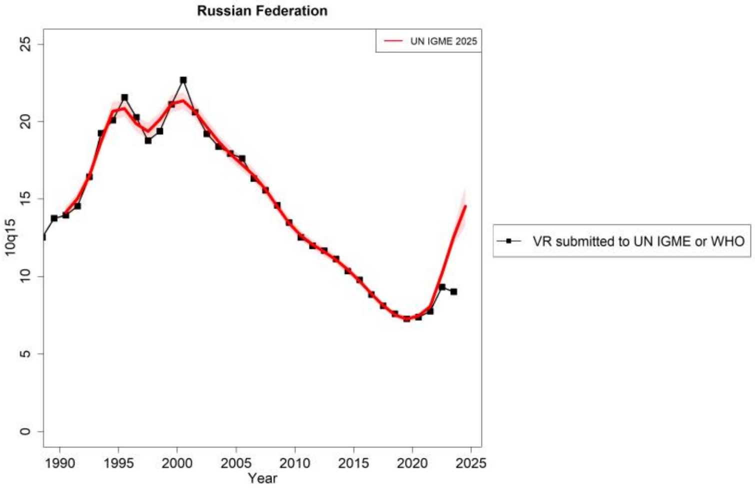
Russian Federation
UN IGME 2025
VR submitted to UN IGME or WHO
10q15
Year

Rwanda (RWA)

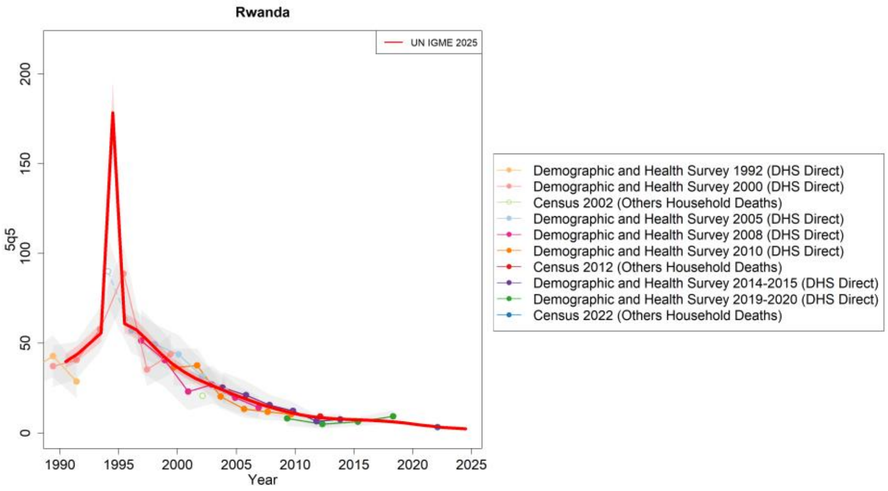
Rwanda
UN IGME 2025
5q5
Year
Demographic and Health Survey 1992 (DHS Direct)
Demographic and Health Survey 2000 (DHS Direct)
Census 2002 (Others Household Deaths)
Demographic and Health Survey 2005 (DHS Direct)
Demographic and Health Survey 2008 (DHS Direct)
Demographic and Health Survey 2010 (DHS Direct)
Census 2012 (Others Household Deaths)
Demographic and Health Survey 2014-2015 (DHS Direct)
Demographic and Health Survey 2019-2020 (DHS Direct)
Census 2022 (Others Household Deaths)

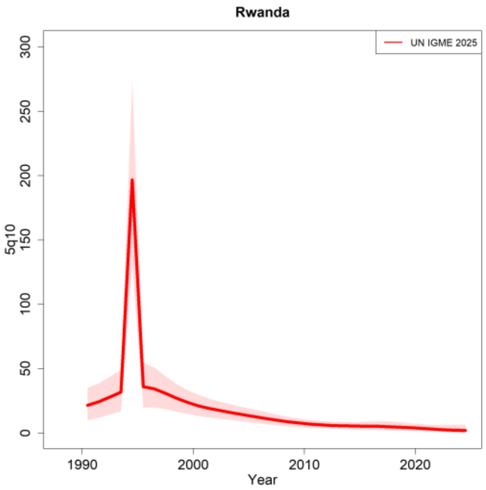
Rwanda
UN IGME 2025
5q10
Year

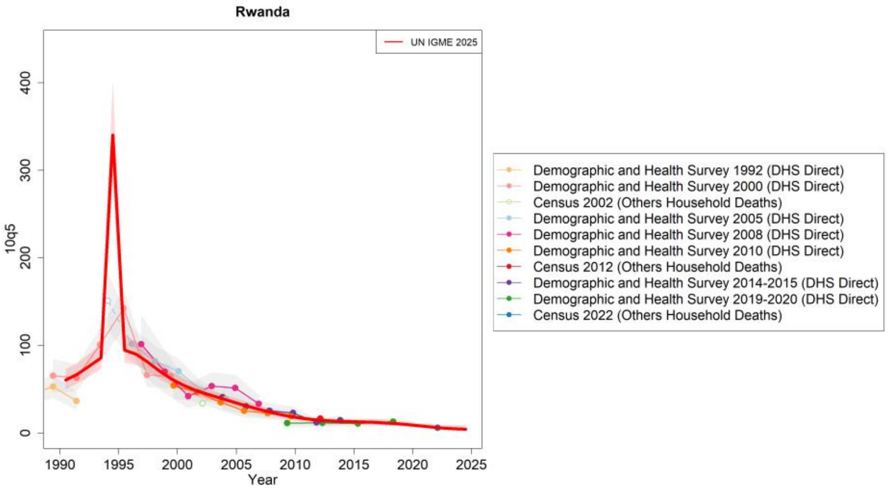
Rwanda
UN IGME 2025
10q5
Year
Demographic and Health Survey 1992 (DHS Direct)
Demographic and Health Survey 2000 (DHS Direct)
Census 2002 (Others Household Deaths)
Demographic and Health Survey 2005 (DHS Direct)
Demographic and Health Survey 2008 (DHS Direct)
Demographic and Health Survey 2010 (DHS Direct)
Census 2012 (Others Household Deaths)
Demographic and Health Survey 2014-2015 (DHS Direct)
Demographic and Health Survey 2019-2020 (DHS Direct)
Census 2022 (Others Household Deaths)

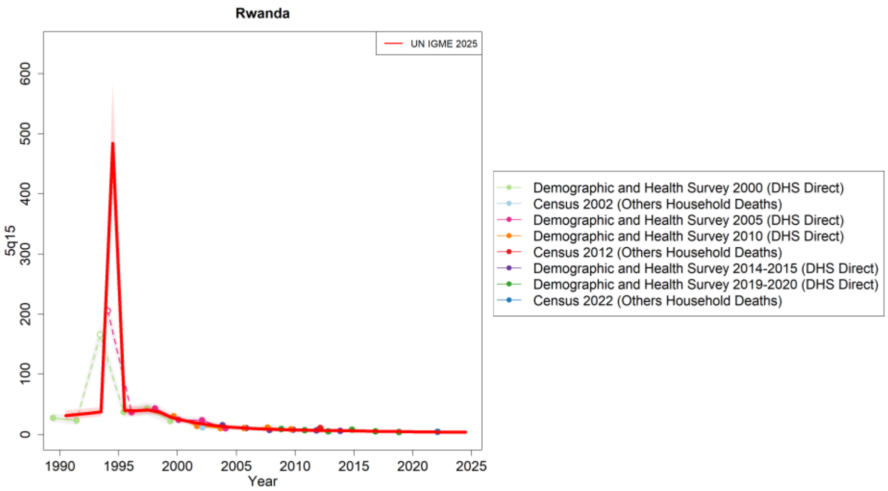
Rwanda
UN IGME 2025
5q15
Year
Demographic and Health Survey 2000 (DHS Direct)
Census 2002 (Others Household Deaths)
Demographic and Health Survey 2005 (DHS Direct)
Demographic and Health Survey 2010 (DHS Direct)
Census 2012 (Others Household Deaths)
Demographic and Health Survey 2014-2015 (DHS Direct)
Demographic and Health Survey 2019-2020 (DHS Direct)
Census 2022 (Others Household Deaths)

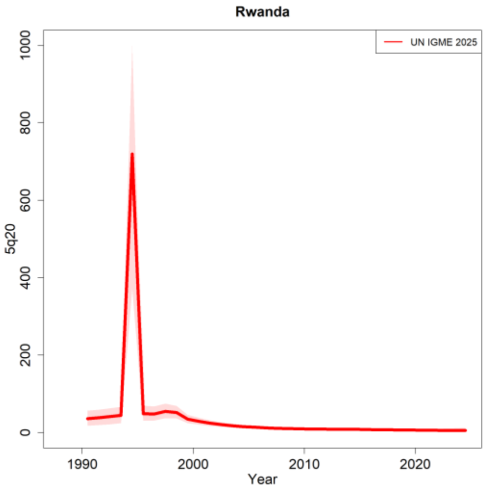
Rwanda
UN IGME 2025
5q20
Year

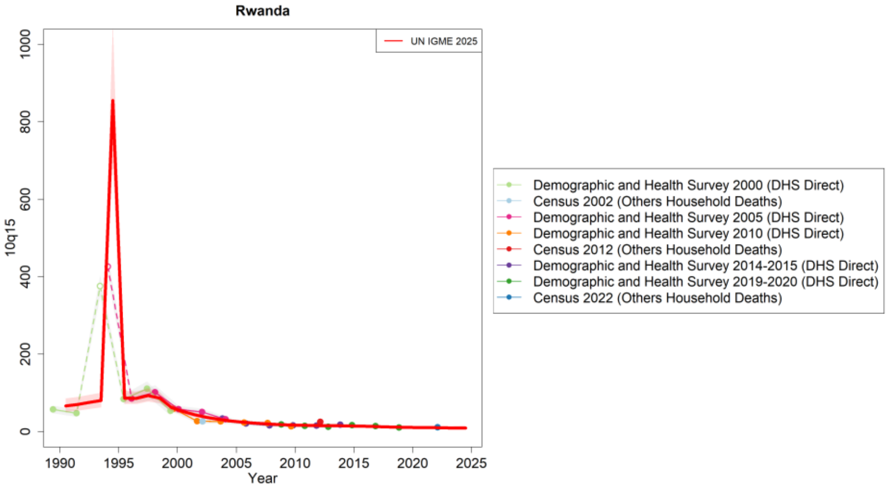
Rwanda
UN IGME 2025
10q15
Year
Demographic and Health Survey 2000 (DHS Direct)
Census 2002 (Others Household Deaths)
Demographic and Health Survey 2005 (DHS Direct)
Demographic and Health Survey 2010 (DHS Direct)
Census 2012 (Others Household Deaths)
Demographic and Health Survey 2014-2015 (DHS Direct)
Demographic and Health Survey 2019-2020 (DHS Direct)
Census 2022 (Others Household Deaths)

Saint Kitts and Nevis (KNA)

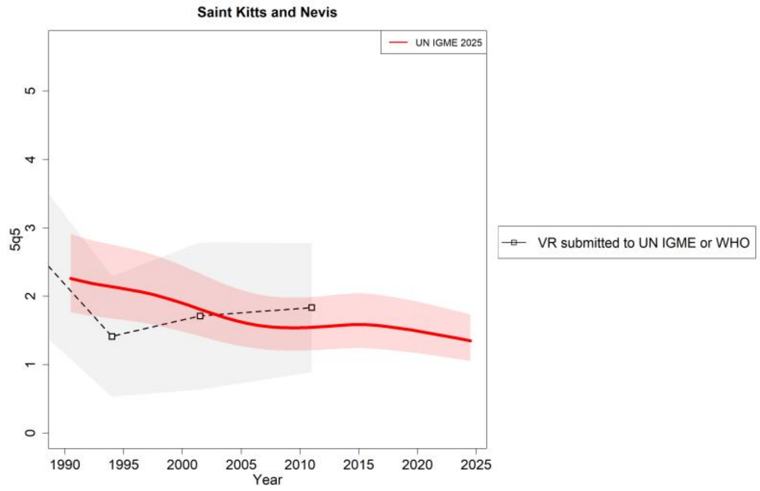

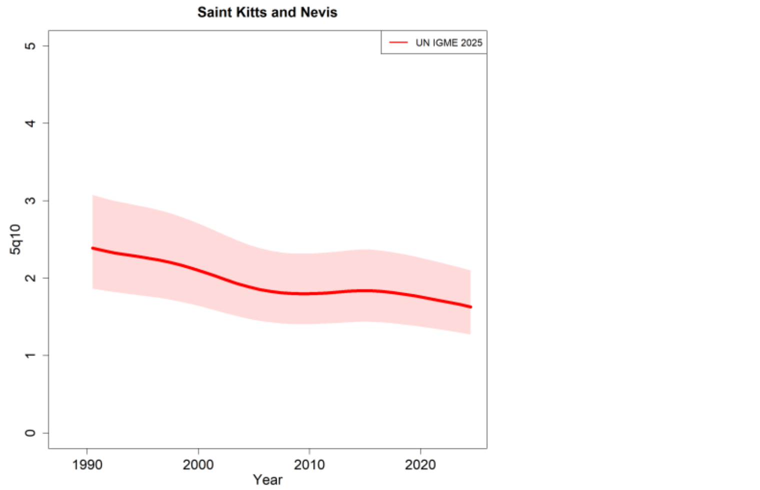

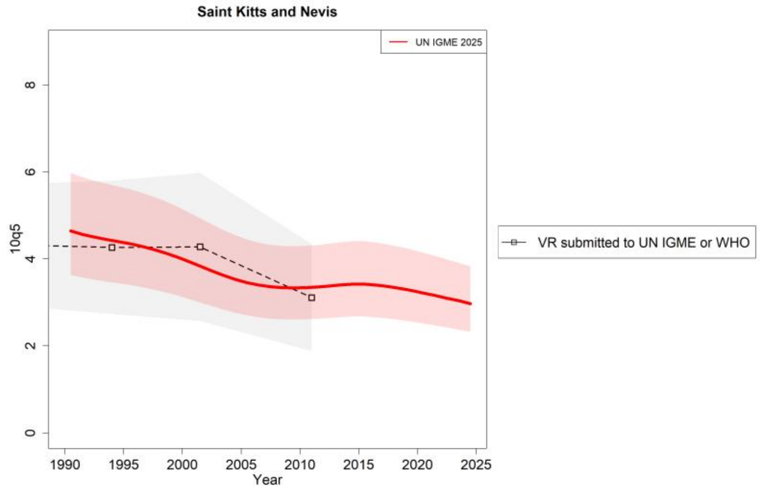

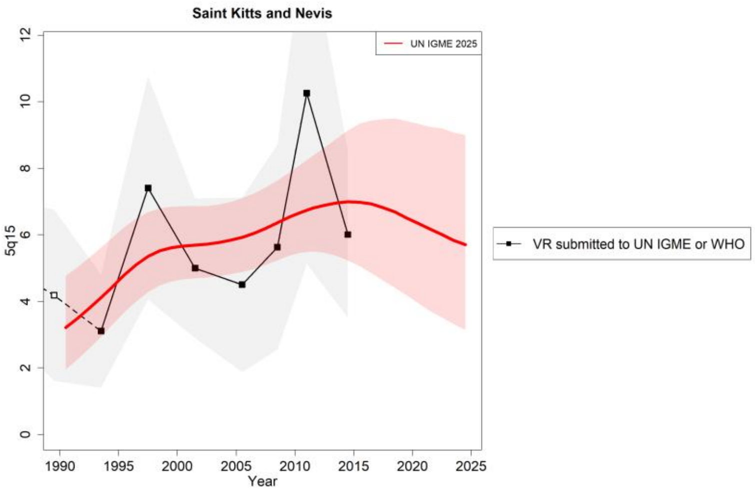
Saint Kitts and Nevis
UN IGME 2025
VR submitted to UN IGME or WHO
5q15
Year
0
2
4
6
8
10
12
1990
1995
2000
2005
2010
2015
2020
2025

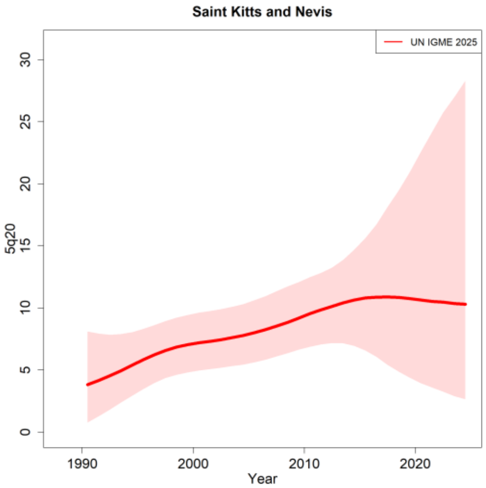
Saint Kitts and Nevis
UN IGME 2025
5q20
Year
0
5
10
15
20
25
30
1990
2000
2010
2020

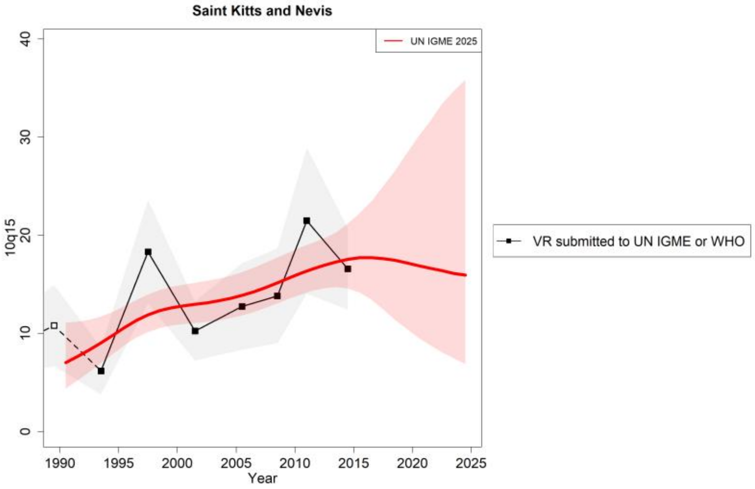
Saint Kitts and Nevis
UN IGME 2025
VR submitted to UN IGME or WHO
10q15
Year
0
10
20
30
40
1990
1995
2000
2005
2010
2015
2020
2025

Saint Lucia (LCA)

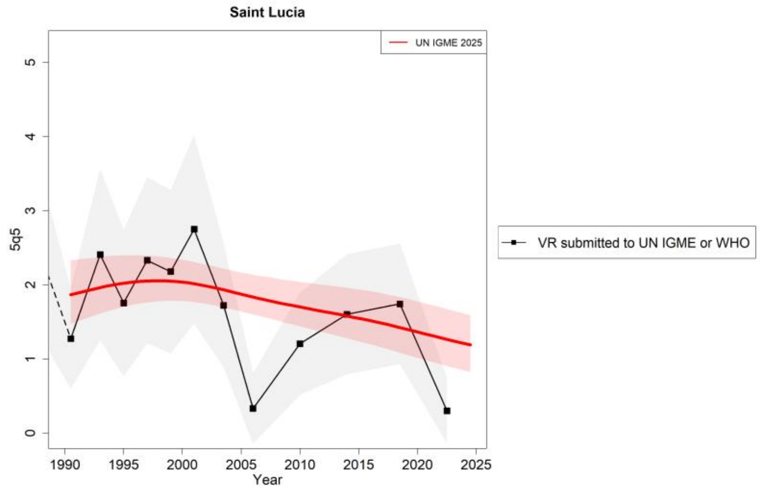

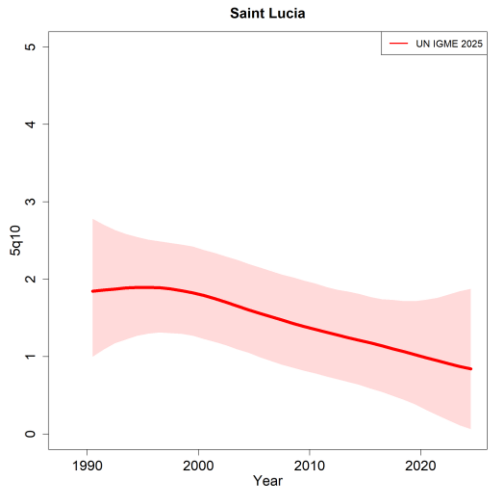

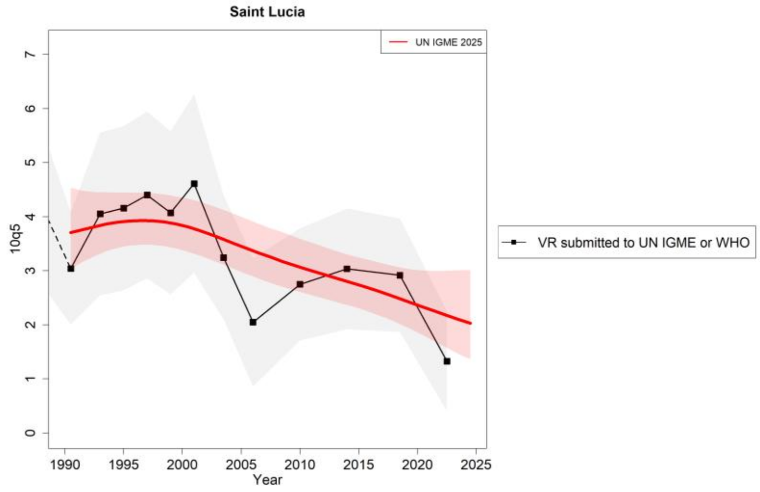

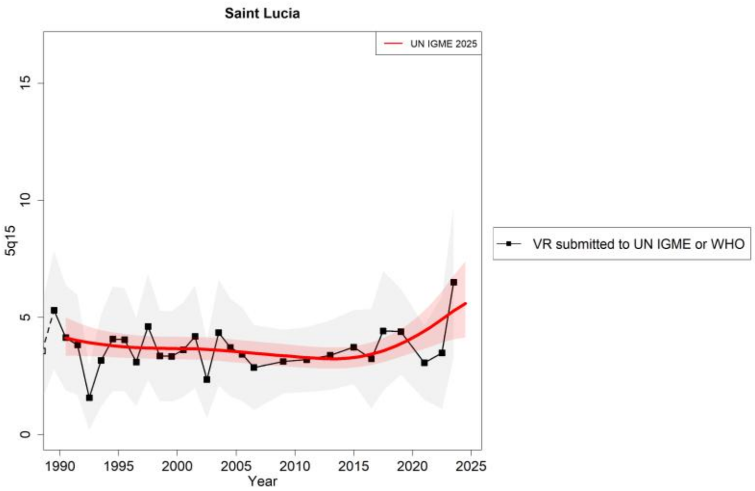
Saint Lucia
UN IGME 2025
VR submitted to UN IGME or WHO
5q15
0
5
10
15
1990
1995
2000
2005
2010
2015
2020
2025
Year

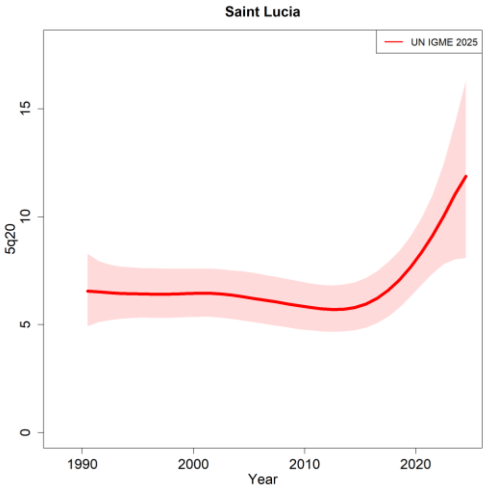
Saint Lucia
UN IGME 2025
5q20
0
5
10
15
1990
2000
2010
2020
Year

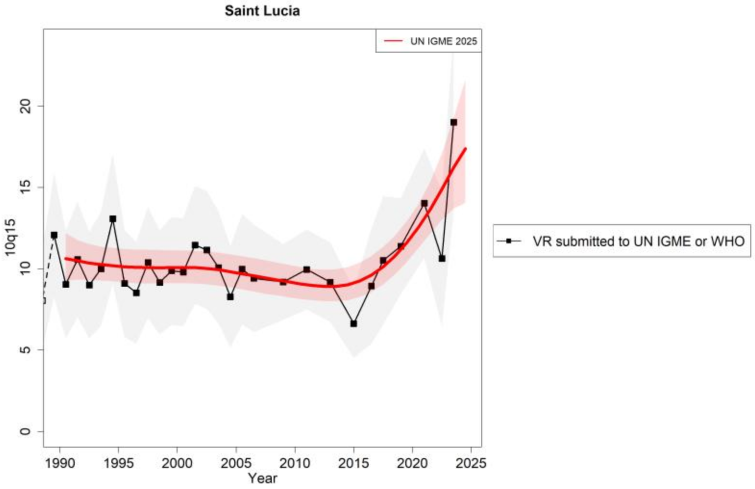
Saint Lucia
UN IGME 2025
VR submitted to UN IGME or WHO
10q15
0
5
10
15
20
1990
1995
2000
2005
2010
2015
2020
2025
Year

Saint Vincent and the Grenadines (VCT)

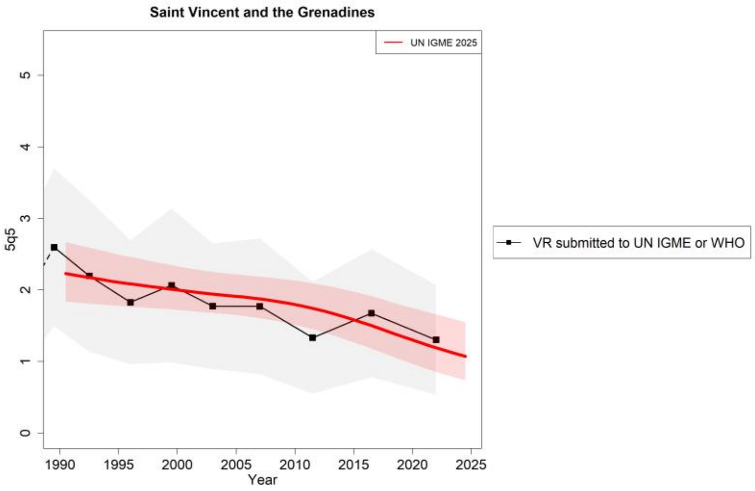

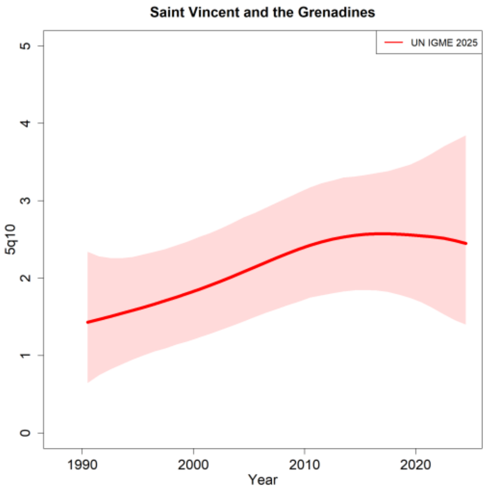

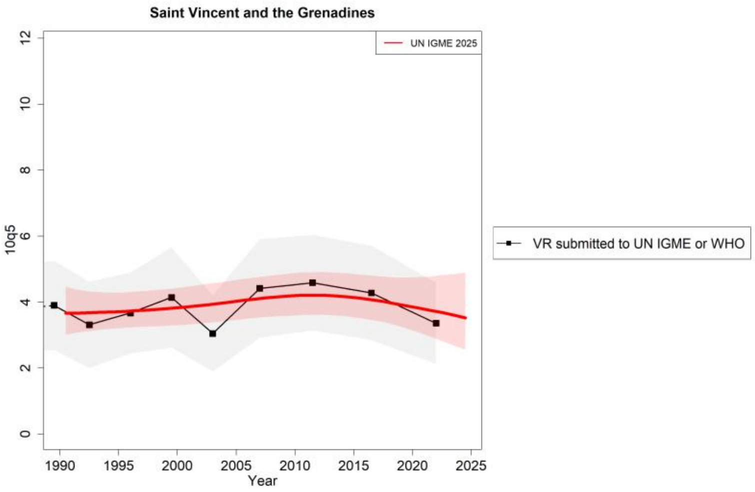

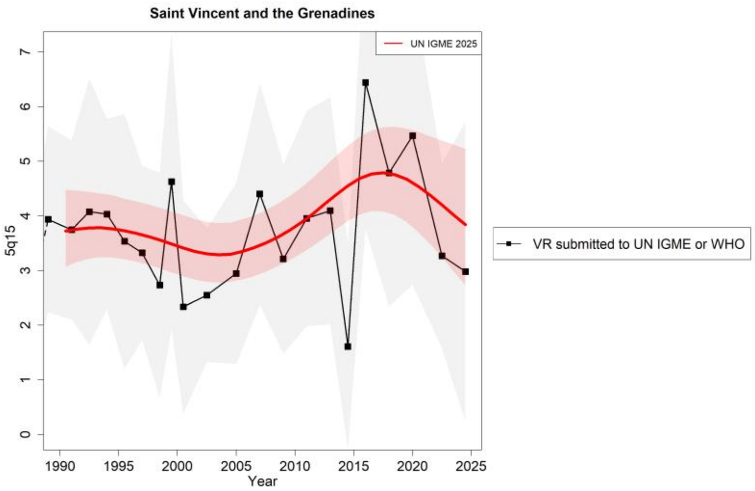
Saint Vincent and the Grenadines
UN IGME 2025
VR submitted to UN IGME or WHO
5q15
Year
0
1
2
3
4
5
6
7
1990
1995
2000
2005
2010
2015
2020
2025

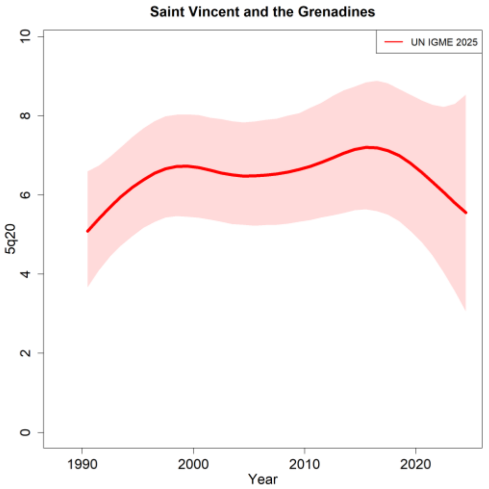
Saint Vincent and the Grenadines
UN IGME 2025
5q20
Year
0
2
4
6
8
10
1990
2000
2010
2020

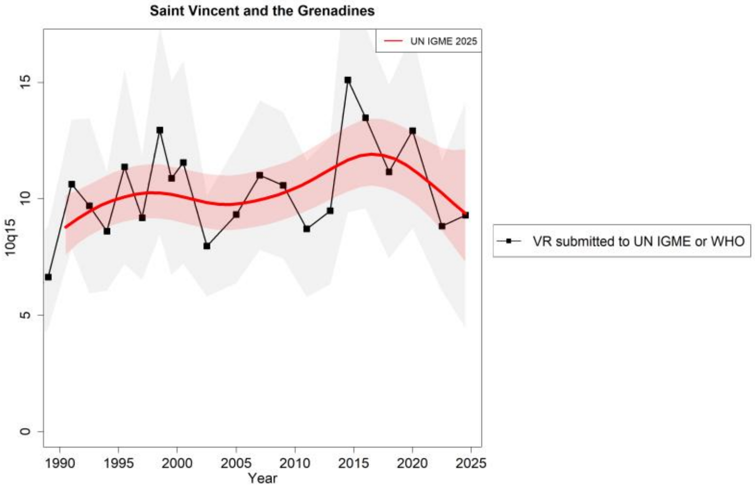
Saint Vincent and the Grenadines
UN IGME 2025
VR submitted to UN IGME or WHO
10q15
Year
0
5
10
15
1990
1995
2000
2005
2010
2015
2020
2025

Samoa (WSM)

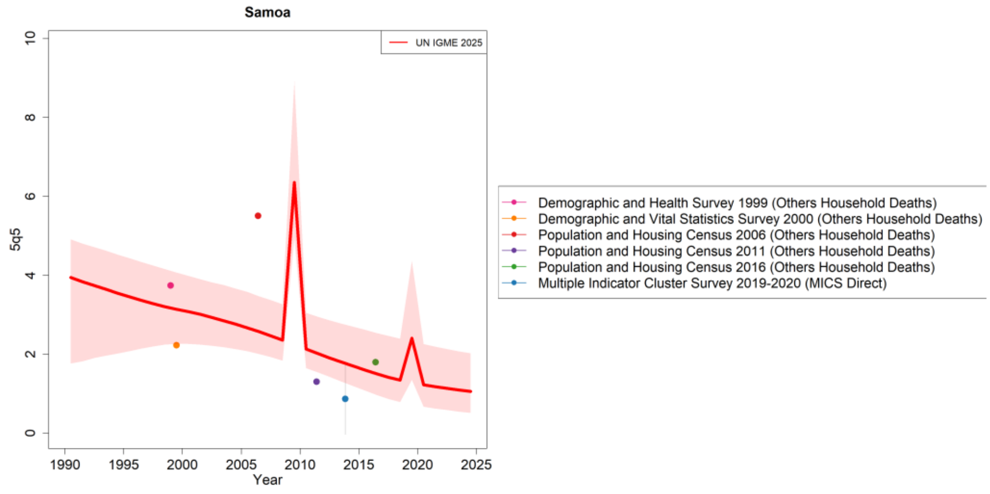
Samoa
UN IGME 2025
5q5
Year
Demographic and Health Survey 1999 (Others Household Deaths)
Demographic and Vital Statistics Survey 2000 (Others Household Deaths)
Population and Housing Census 2006 (Others Household Deaths)
Population and Housing Census 2011 (Others Household Deaths)
Population and Housing Census 2016 (Others Household Deaths)
Multiple Indicator Cluster Survey 2019-2020 (MICS Direct)

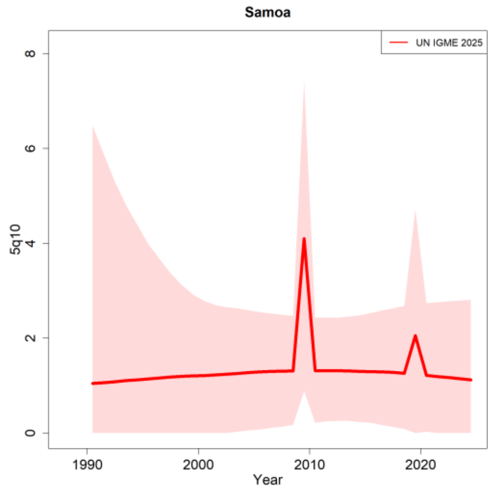
Samoa
UN IGME 2025
5q10
Year

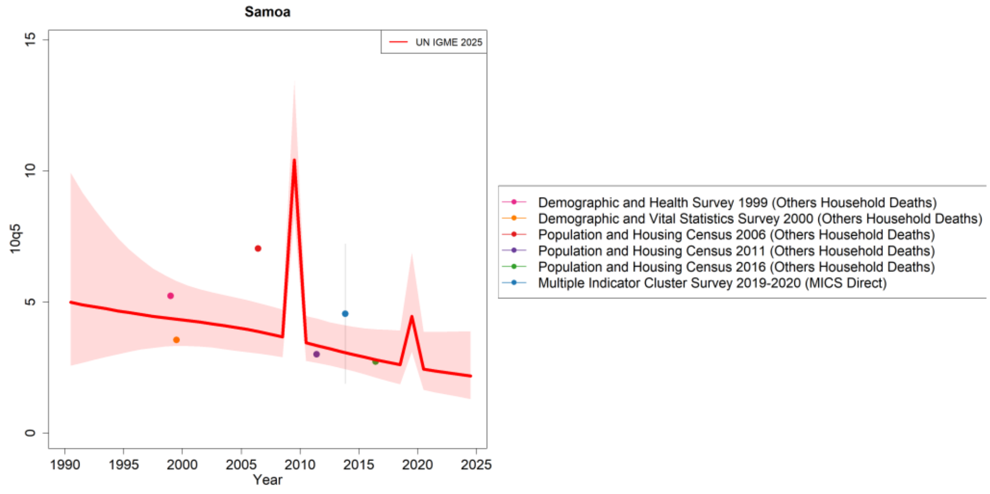
Samoa
UN IGME 2025
10q5
Year
Demographic and Health Survey 1999 (Others Household Deaths)
Demographic and Vital Statistics Survey 2000 (Others Household Deaths)
Population and Housing Census 2006 (Others Household Deaths)
Population and Housing Census 2011 (Others Household Deaths)
Population and Housing Census 2016 (Others Household Deaths)
Multiple Indicator Cluster Survey 2019-2020 (MICS Direct)

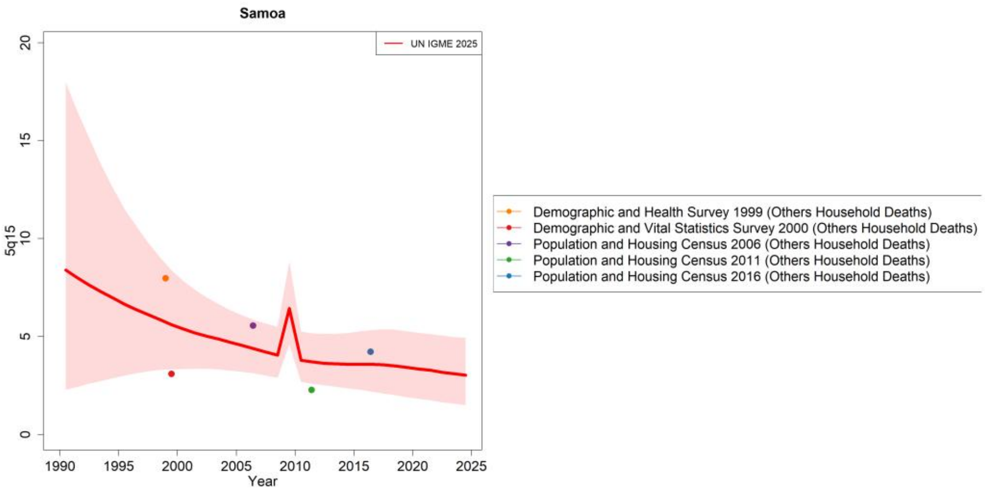
Samoa
UN IGME 2025
5q15
Year
Demographic and Health Survey 1999 (Others Household Deaths)
Demographic and Vital Statistics Survey 2000 (Others Household Deaths)
Population and Housing Census 2006 (Others Household Deaths)
Population and Housing Census 2011 (Others Household Deaths)
Population and Housing Census 2016 (Others Household Deaths)

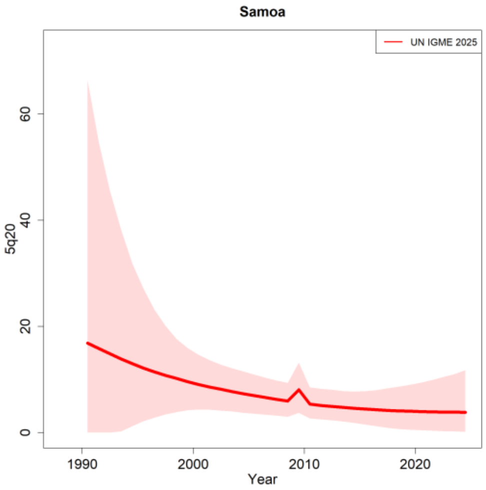
Samoa
UN IGME 2025
5q20
Year

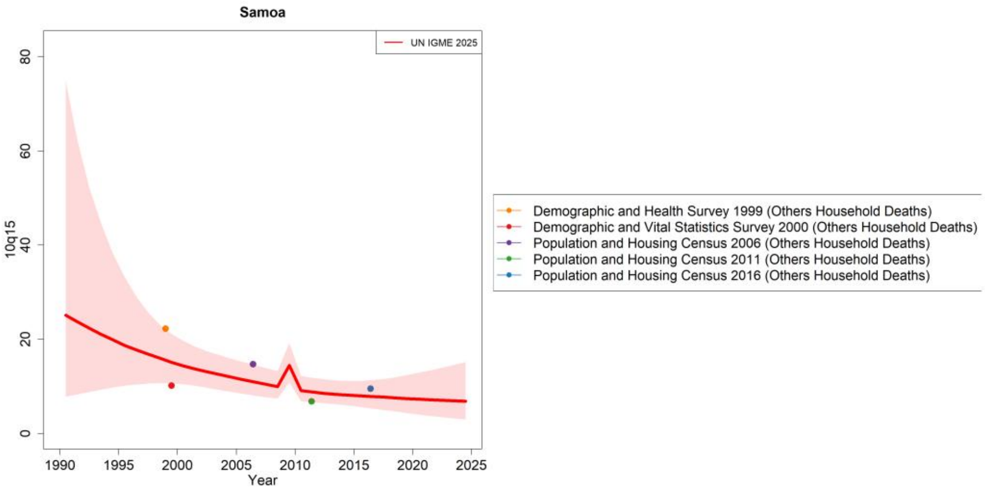
Samoa
UN IGME 2025
10q15
Year
Demographic and Health Survey 1999 (Others Household Deaths)
Demographic and Vital Statistics Survey 2000 (Others Household Deaths)
Population and Housing Census 2006 (Others Household Deaths)
Population and Housing Census 2011 (Others Household Deaths)
Population and Housing Census 2016 (Others Household Deaths)

San Marino (SMR)

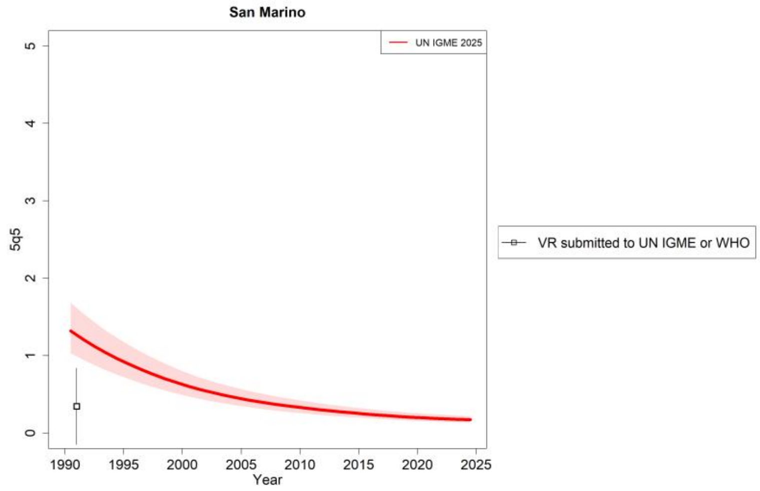

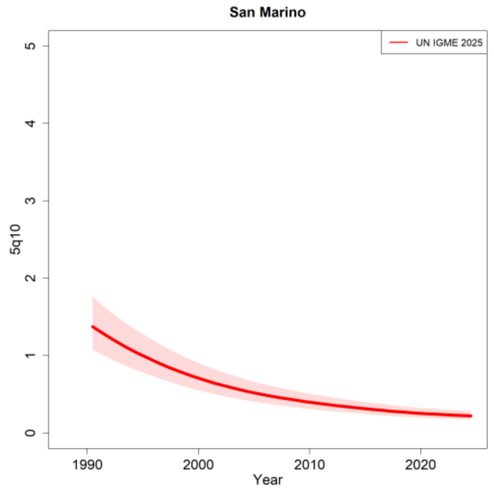

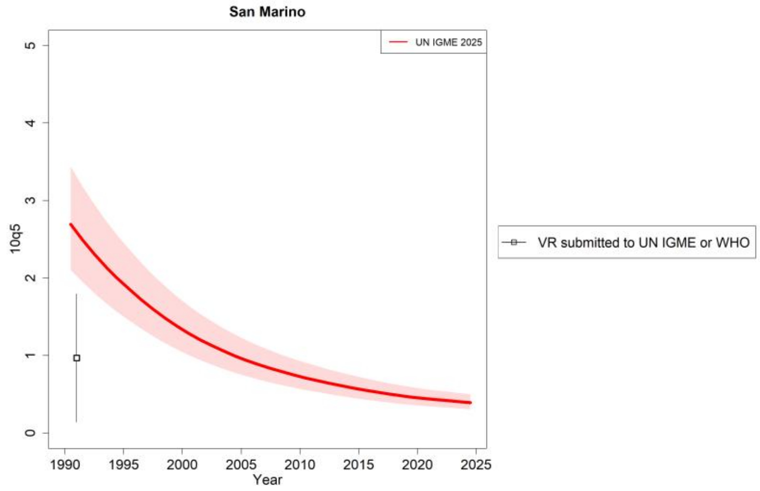

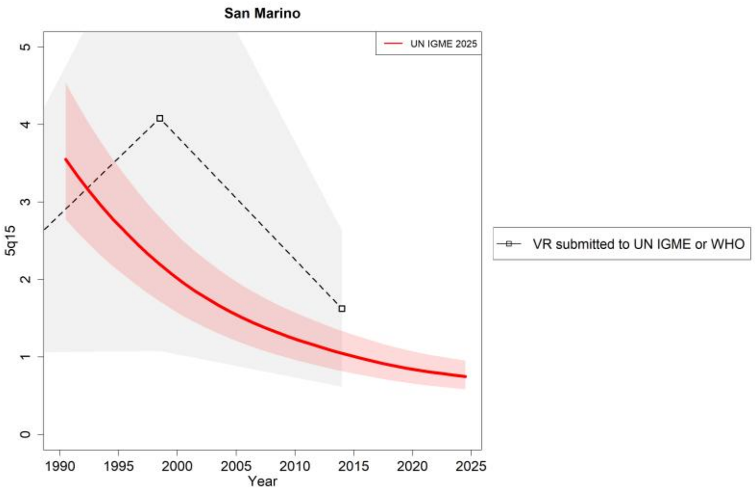
San Marino
UN IGME 2025
VR submitted to UN IGME or WHO
5q15
0
1
2
3
4
5
1990
1995
2000
2005
2010
2015
2020
2025
Year

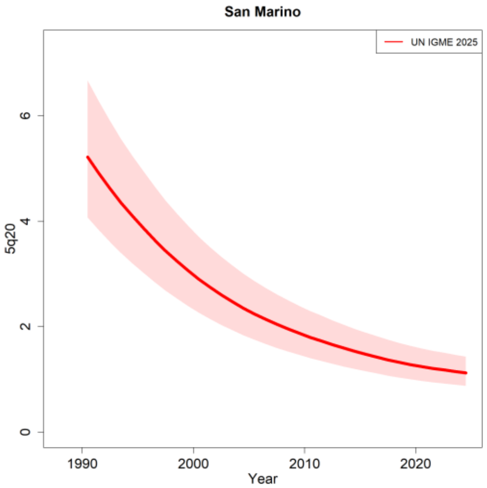
San Marino
UN IGME 2025
5q20
0
2
4
6
1990
2000
2010
2020
Year

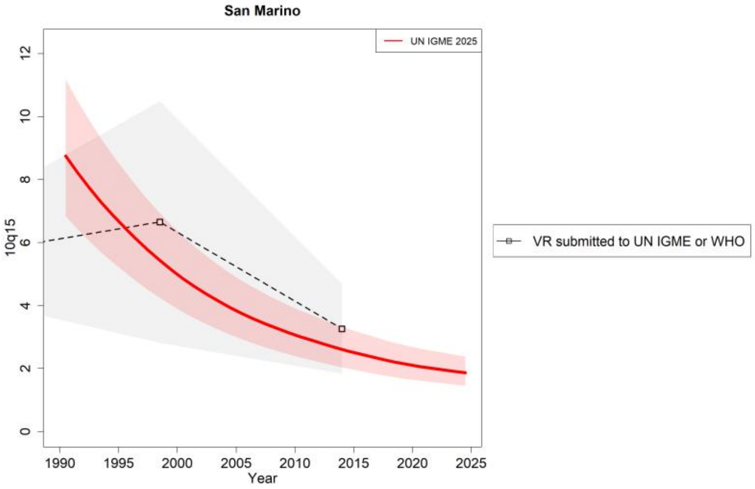
San Marino
UN IGME 2025
VR submitted to UN IGME or WHO
10q15
0
2
4
6
8
10
12
1990
1995
2000
2005
2010
2015
2020
2025
Year

Sao Tome and Principe (STP)

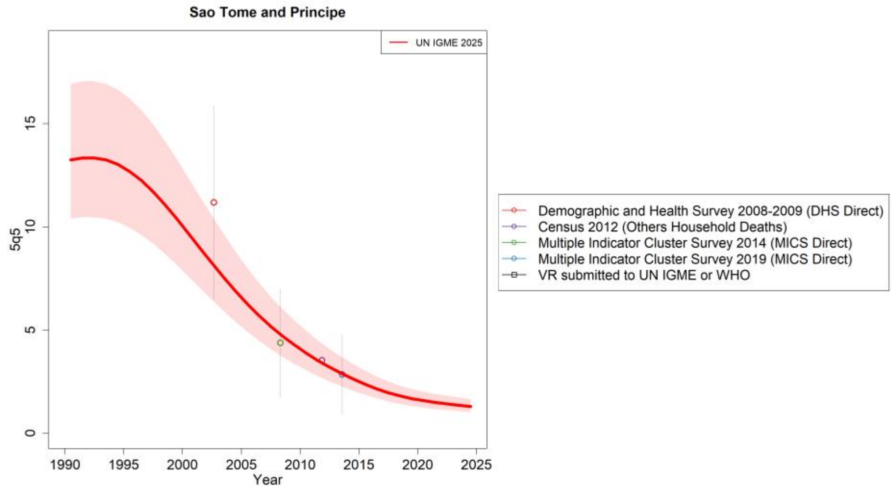

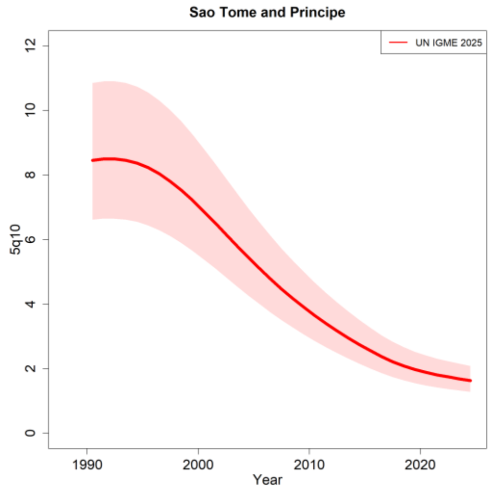

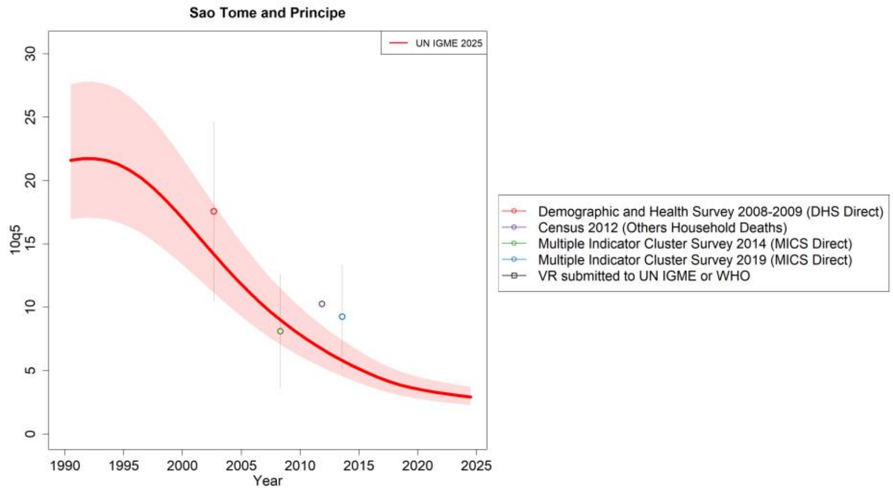

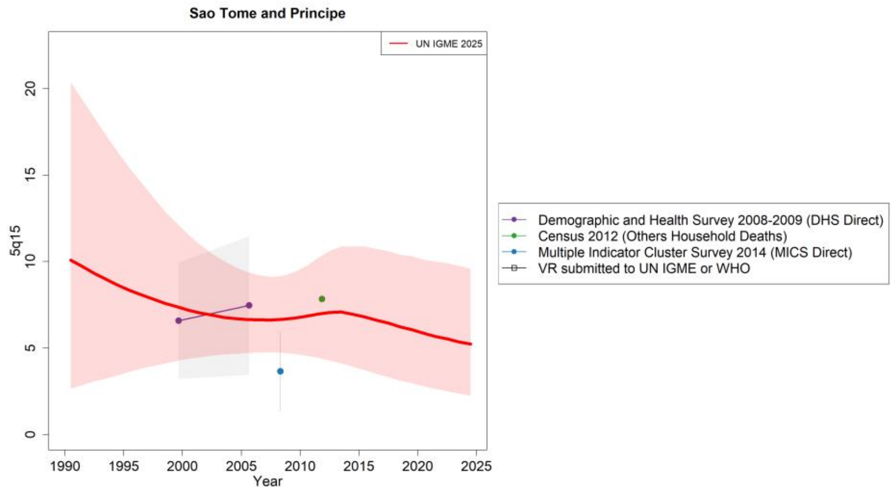
Sao Tome and Principe
UN IGME 2025
5q15
Year
Demographic and Health Survey 2008-2009 (DHS Direct)
Census 2012 (Others Household Deaths)
Multiple Indicator Cluster Survey 2014 (MICS Direct)
VR submitted to UN IGME or WHO

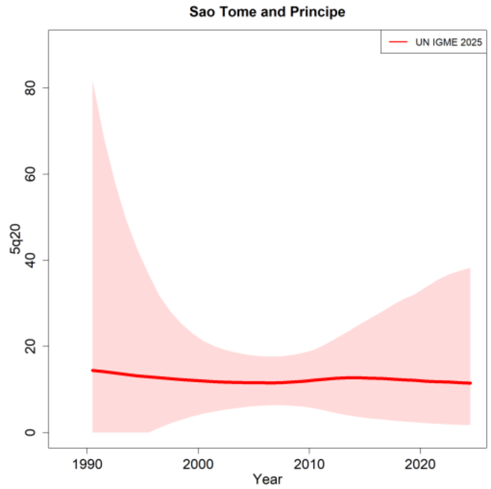
Sao Tome and Principe
UN IGME 2025
5q20
Year

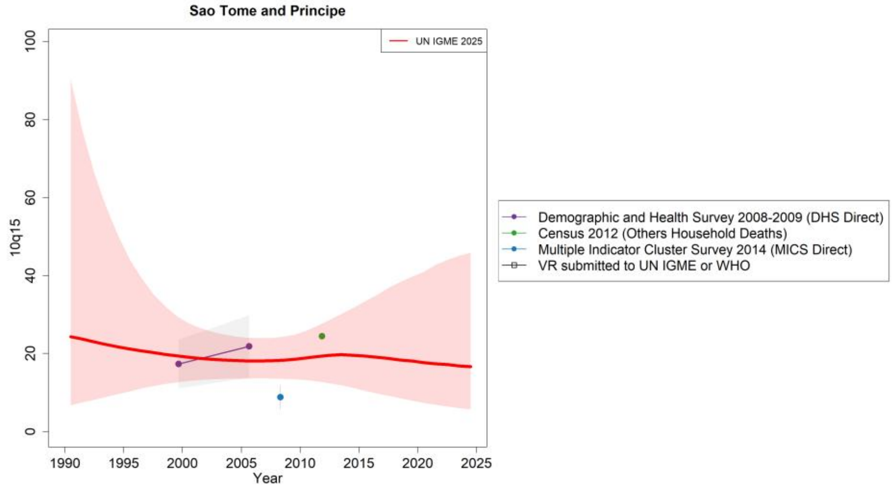
Sao Tome and Principe
UN IGME 2025
10q15
Year
Demographic and Health Survey 2008-2009 (DHS Direct)
Census 2012 (Others Household Deaths)
Multiple Indicator Cluster Survey 2014 (MICS Direct)
VR submitted to UN IGME or WHO

Saudi Arabia (SAU)

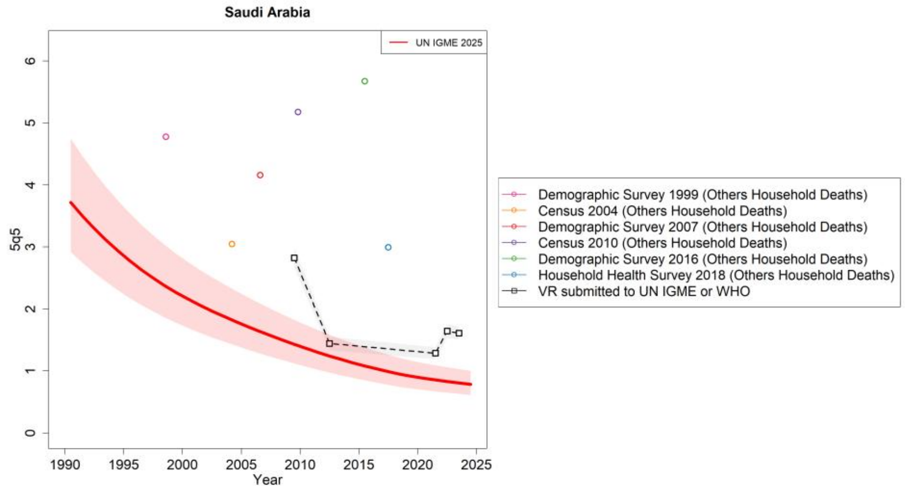

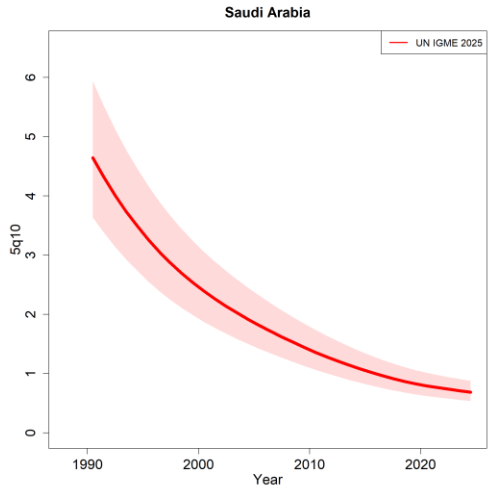

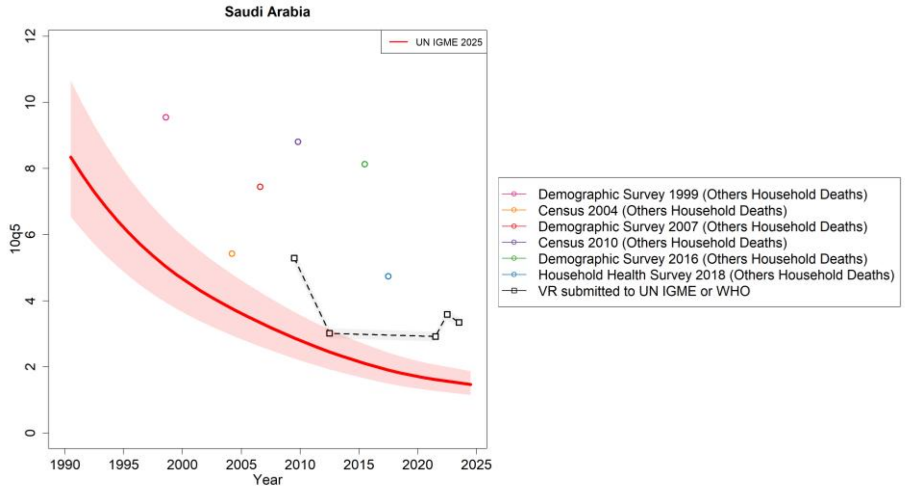

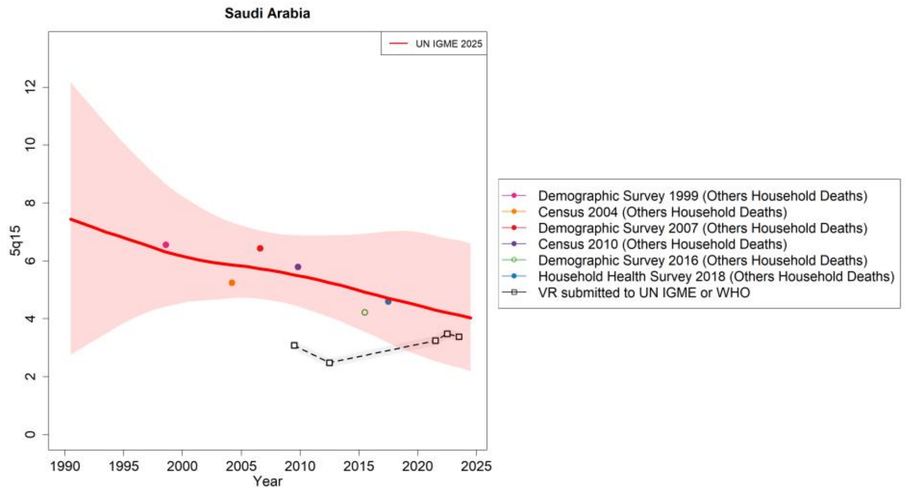
Saudi Arabia
UN IGME 2025
5q15
Year
Demographic Survey 1999 (Others Household Deaths)
Census 2004 (Others Household Deaths)
Demographic Survey 2007 (Others Household Deaths)
Census 2010 (Others Household Deaths)
Demographic Survey 2016 (Others Household Deaths)
Household Health Survey 2018 (Others Household Deaths)
VR submitted to UN IGME or WHO

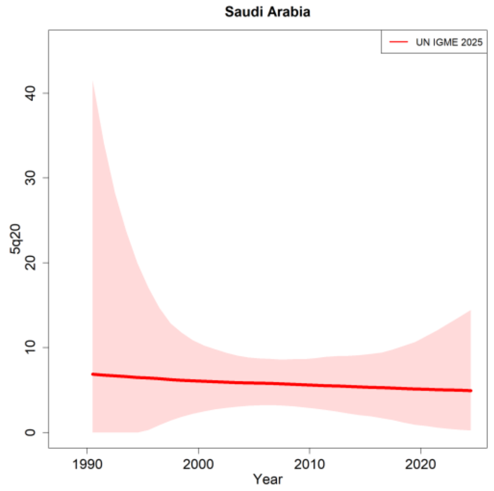
Saudi Arabia
UN IGME 2025
5q20
Year

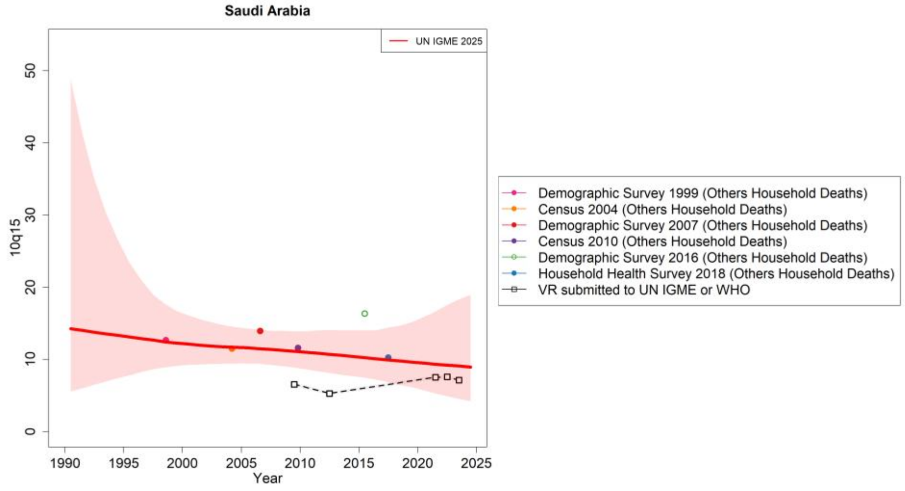
Saudi Arabia
UN IGME 2025
10q15
Year
Demographic Survey 1999 (Others Household Deaths)
Census 2004 (Others Household Deaths)
Demographic Survey 2007 (Others Household Deaths)
Census 2010 (Others Household Deaths)
Demographic Survey 2016 (Others Household Deaths)
Household Health Survey 2018 (Others Household Deaths)
VR submitted to UN IGME or WHO

Senegal (SEN)

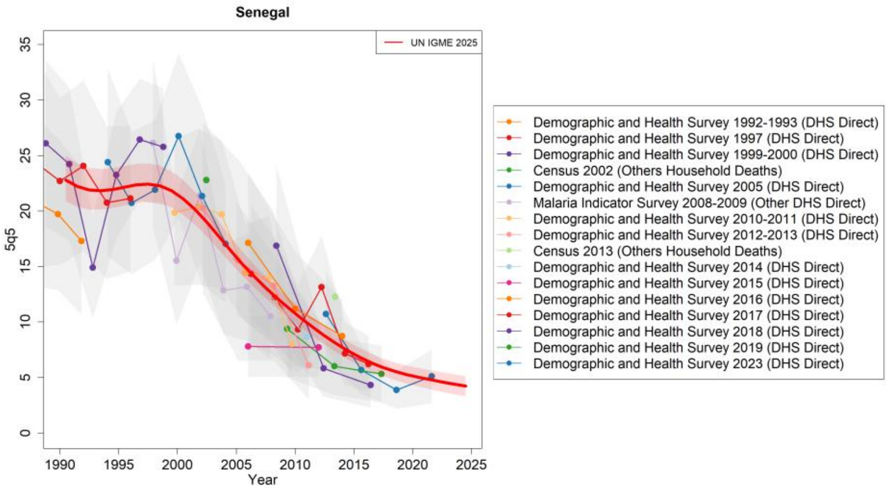
Senegal
UN IGME 2025
5q5
Year
Demographic and Health Survey 1992-1993 (DHS Direct)
Demographic and Health Survey 1997 (DHS Direct)
Demographic and Health Survey 1999-2000 (DHS Direct)
Census 2002 (Others Household Deaths)
Demographic and Health Survey 2005 (DHS Direct)
Malaria Indicator Survey 2008-2009 (Other DHS Direct)
Demographic and Health Survey 2010-2011 (DHS Direct)
Demographic and Health Survey 2012-2013 (DHS Direct)
Census 2013 (Others Household Deaths)
Demographic and Health Survey 2014 (DHS Direct)
Demographic and Health Survey 2015 (DHS Direct)
Demographic and Health Survey 2016 (DHS Direct)
Demographic and Health Survey 2017 (DHS Direct)
Demographic and Health Survey 2018 (DHS Direct)
Demographic and Health Survey 2019 (DHS Direct)
Demographic and Health Survey 2023 (DHS Direct)

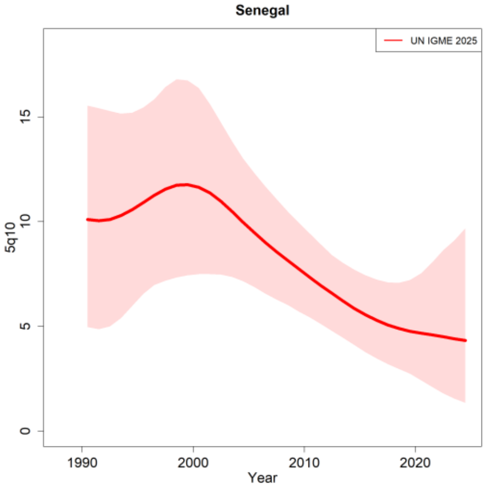
Senegal
UN IGME 2025
5q10
Year

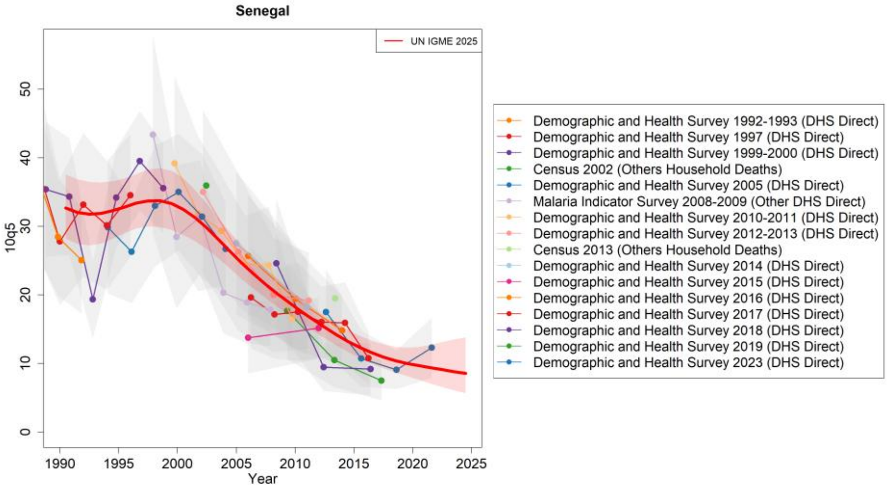
Senegal
UN IGME 2025
10q5
Year
Demographic and Health Survey 1992-1993 (DHS Direct)
Demographic and Health Survey 1997 (DHS Direct)
Demographic and Health Survey 1999-2000 (DHS Direct)
Census 2002 (Others Household Deaths)
Demographic and Health Survey 2005 (DHS Direct)
Malaria Indicator Survey 2008-2009 (Other DHS Direct)
Demographic and Health Survey 2010-2011 (DHS Direct)
Demographic and Health Survey 2012-2013 (DHS Direct)
Census 2013 (Others Household Deaths)
Demographic and Health Survey 2014 (DHS Direct)
Demographic and Health Survey 2015 (DHS Direct)
Demographic and Health Survey 2016 (DHS Direct)
Demographic and Health Survey 2017 (DHS Direct)
Demographic and Health Survey 2018 (DHS Direct)
Demographic and Health Survey 2019 (DHS Direct)
Demographic and Health Survey 2023 (DHS Direct)

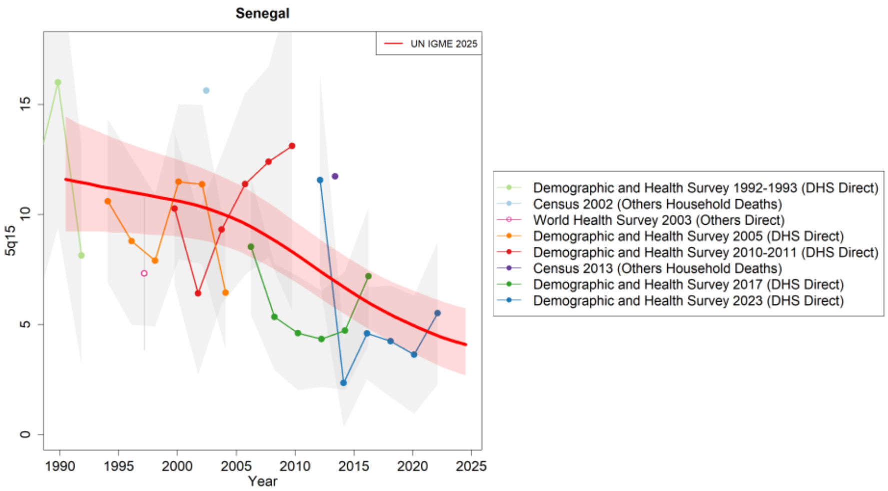

Senegal
UN IGME 2025
5q15
Year
0
5
10
15
1990
1995
2000
2005
2010
2015
2020
2025
Demographic and Health Survey 1992-1993 (DHS Direct)
Census 2002 (Others Household Deaths)
World Health Survey 2003 (Others Direct)
Demographic and Health Survey 2005 (DHS Direct)
Demographic and Health Survey 2010-2011 (DHS Direct)
Census 2013 (Others Household Deaths)
Demographic and Health Survey 2017 (DHS Direct)
Demographic and Health Survey 2023 (DHS Direct)

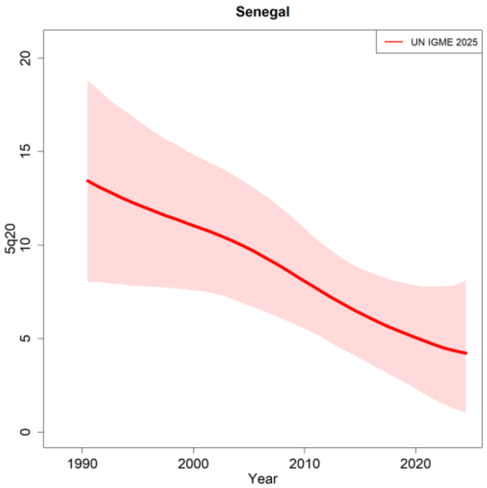

Senegal
UN IGME 2025
5q20
Year
0
5
10
15
20
1990
2000
2010
2020

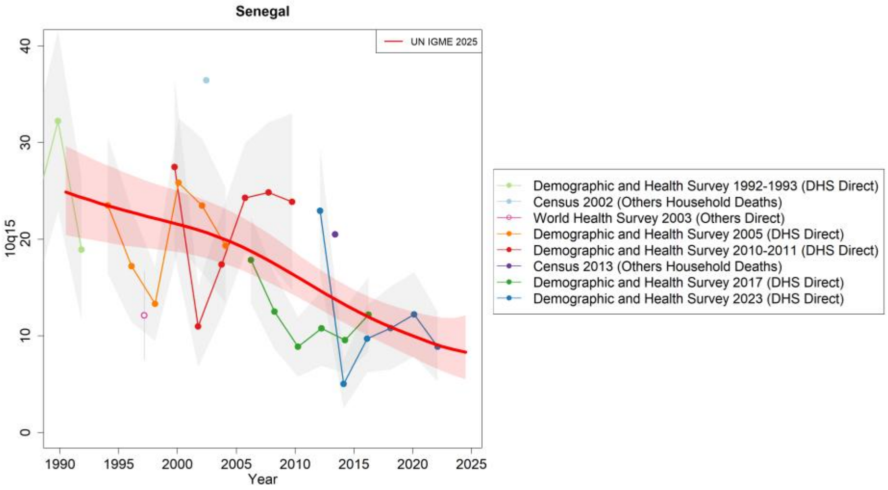

Senegal
UN IGME 2025
10q15
Year
0
10
20
30
40
1990
1995
2000
2005
2010
2015
2020
2025
Demographic and Health Survey 1992-1993 (DHS Direct)
Census 2002 (Others Household Deaths)
World Health Survey 2003 (Others Direct)
Demographic and Health Survey 2005 (DHS Direct)
Demographic and Health Survey 2010-2011 (DHS Direct)
Census 2013 (Others Household Deaths)
Demographic and Health Survey 2017 (DHS Direct)
Demographic and Health Survey 2023 (DHS Direct)

Serbia (SRB)

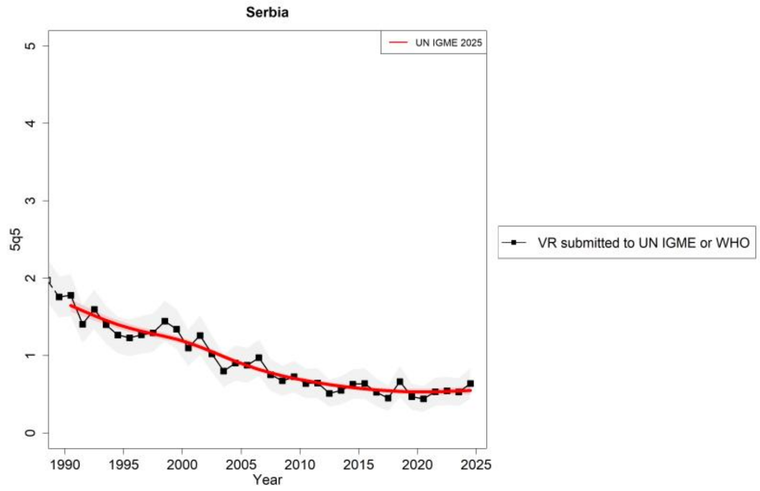
Serbia
UN IGME 2025
VR submitted to UN IGME or WHO
5q5
0
1
2
3
4
5
1990
1995
2000
2005
2010
2015
2020
2025
Year

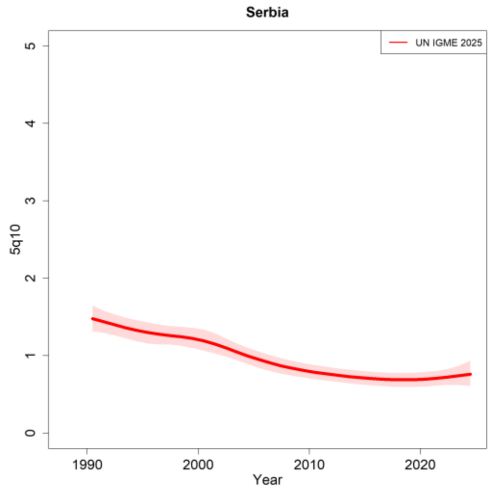
Serbia
UN IGME 2025
5q10
0
1
2
3
4
5
1990
2000
2010
2020
Year

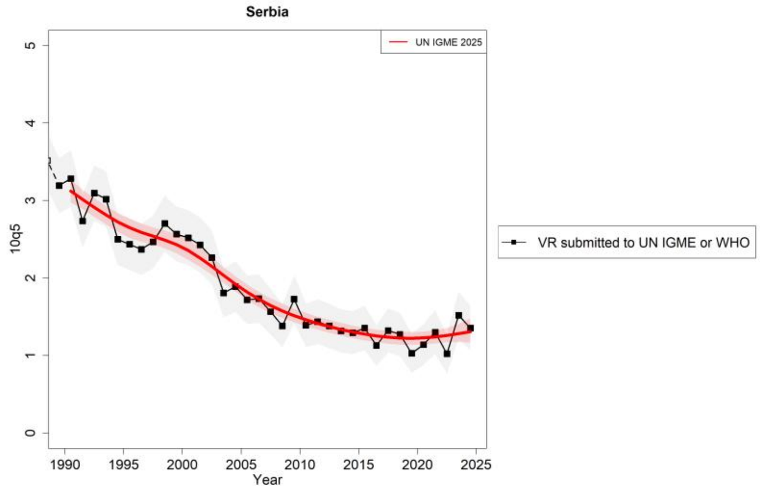
Serbia
UN IGME 2025
VR submitted to UN IGME or WHO
10q5
0
1
2
3
4
5
1990
1995
2000
2005
2010
2015
2020
2025
Year

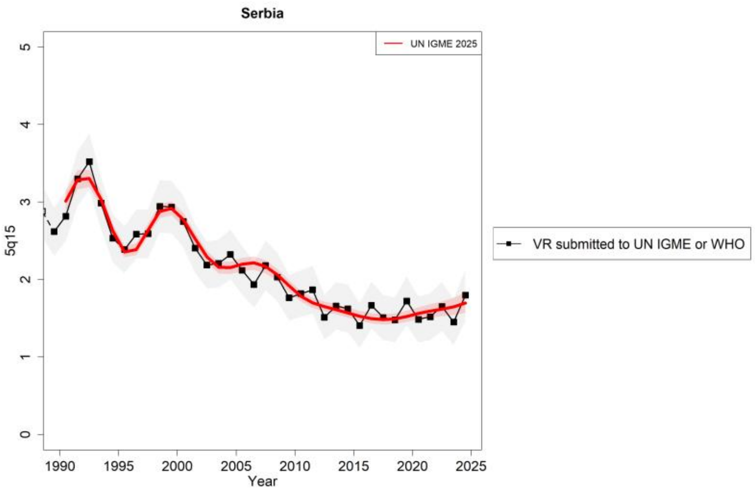
Serbia
UN IGME 2025
5q15
Year
VR submitted to UN IGME or WHO

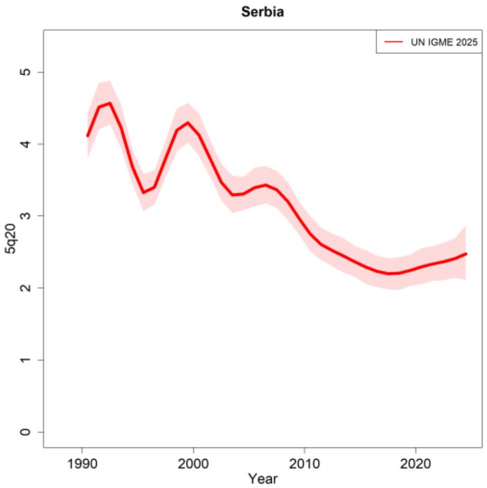
Serbia
UN IGME 2025
5q20
Year

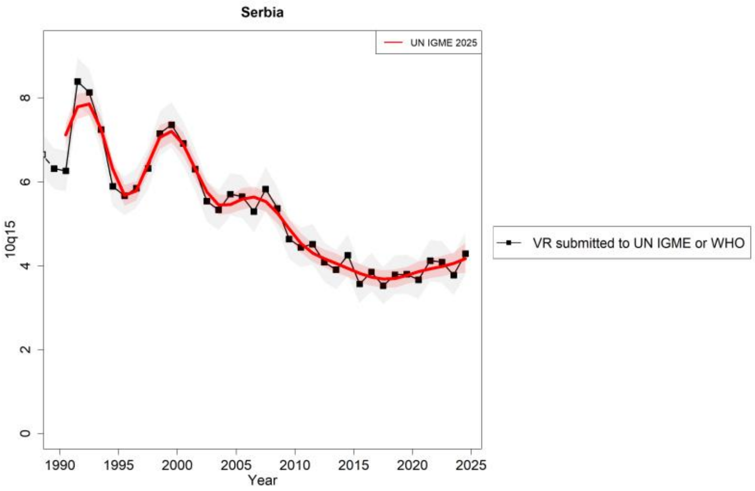
Serbia
UN IGME 2025
10q15
Year
VR submitted to UN IGME or WHO

Seychelles (SYC)

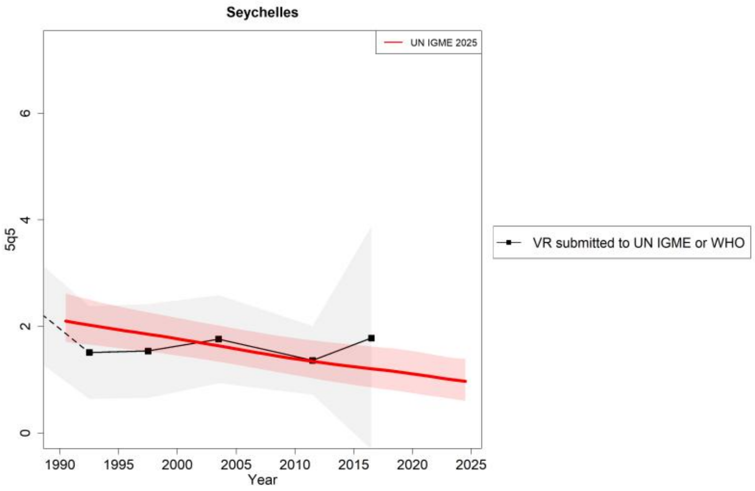

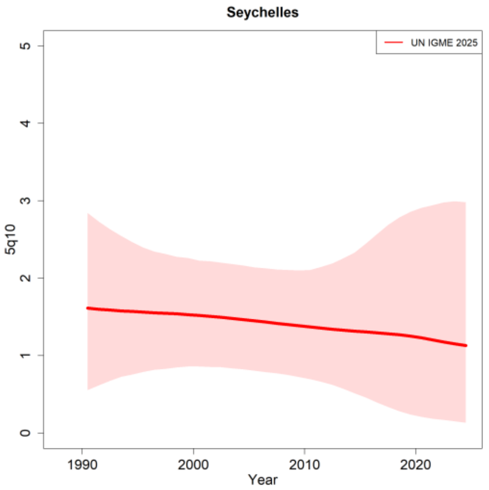

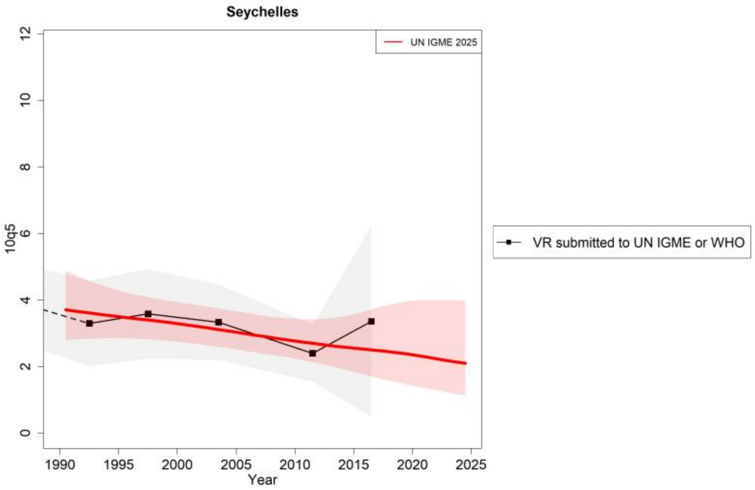

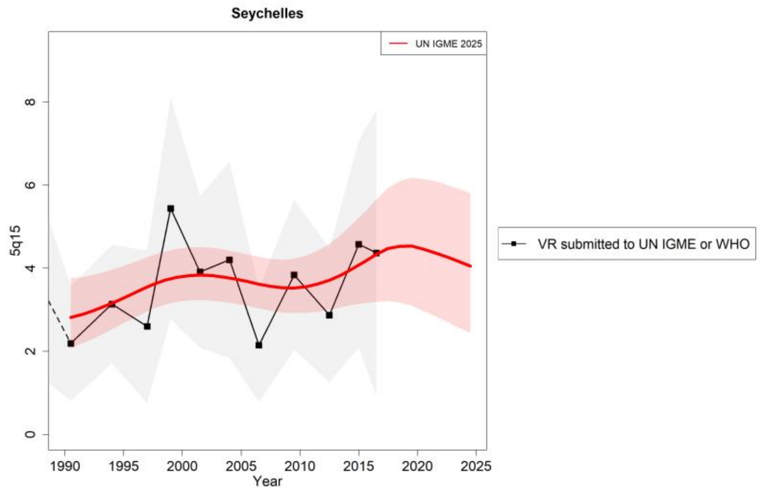
Seychelles
UN IGME 2025
VR submitted to UN IGME or WHO
5q15
0
2
4
6
8
1990
1995
2000
2005
2010
2015
2020
2025
Year

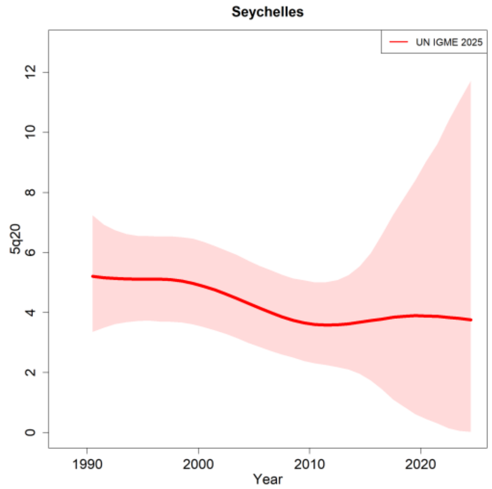
Seychelles
UN IGME 2025
5q20
0
2
4
6
8
10
12
1990
2000
2010
2020
Year

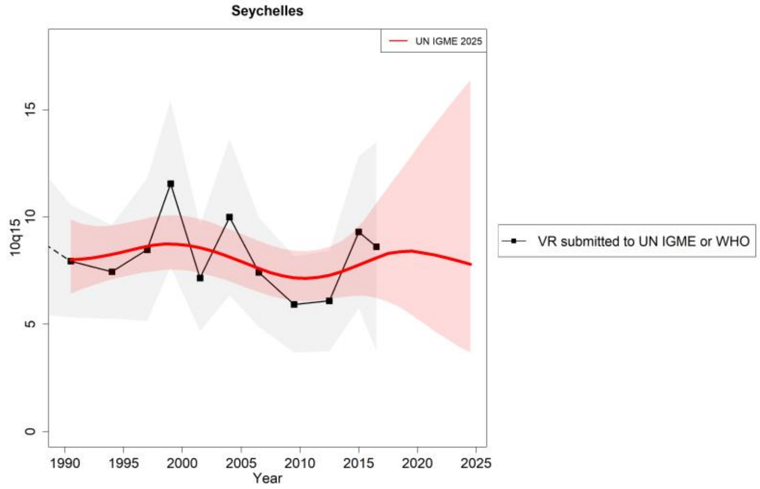
Seychelles
UN IGME 2025
VR submitted to UN IGME or WHO
10q15
0
5
10
15
1990
1995
2000
2005
2010
2015
2020
2025
Year

Sierra Leone (SLE)

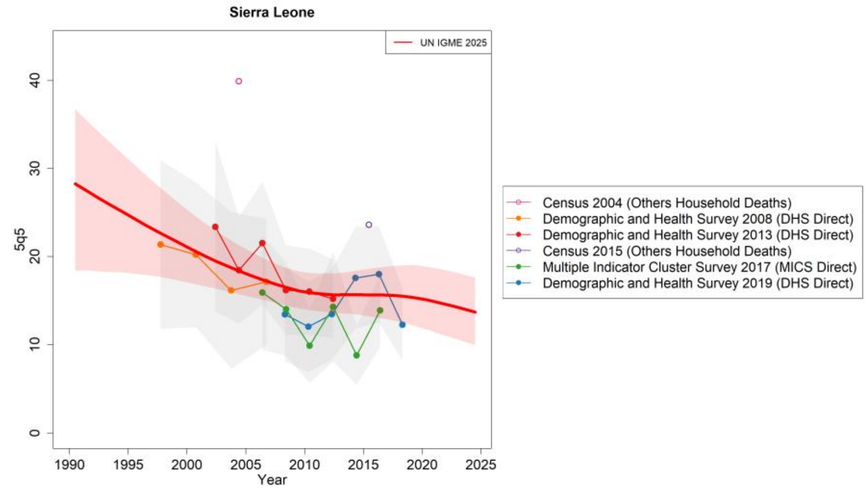

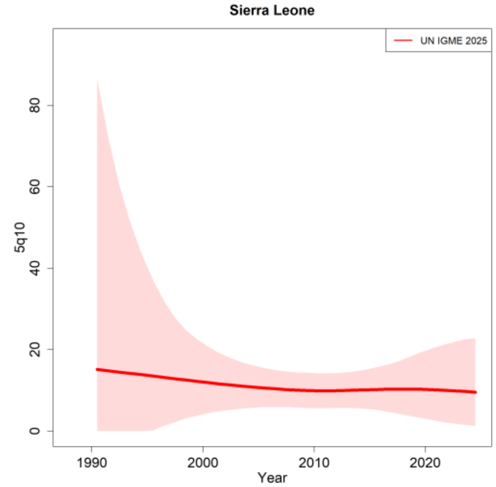

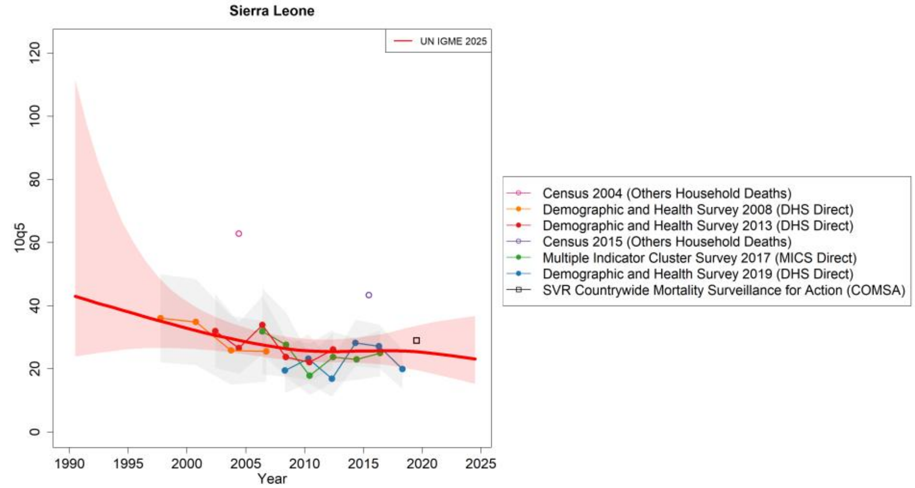

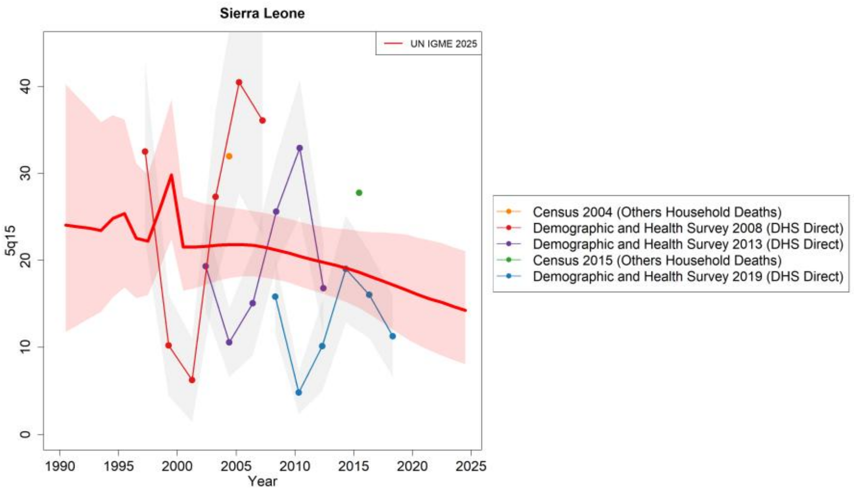
Sierra Leone
UN IGME 2025
5q15
Year
Census 2004 (Others Household Deaths)
Demographic and Health Survey 2008 (DHS Direct)
Demographic and Health Survey 2013 (DHS Direct)
Census 2015 (Others Household Deaths)
Demographic and Health Survey 2019 (DHS Direct)

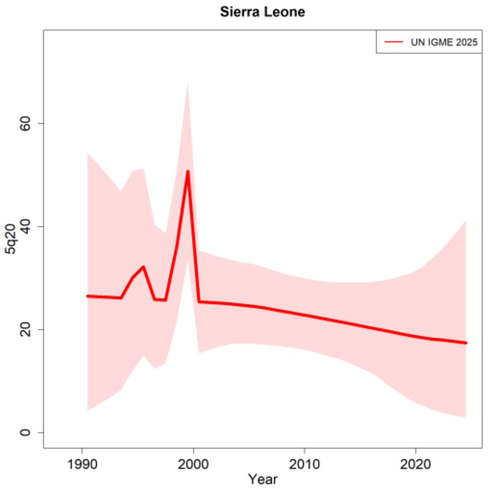
Sierra Leone
UN IGME 2025
5q20
Year

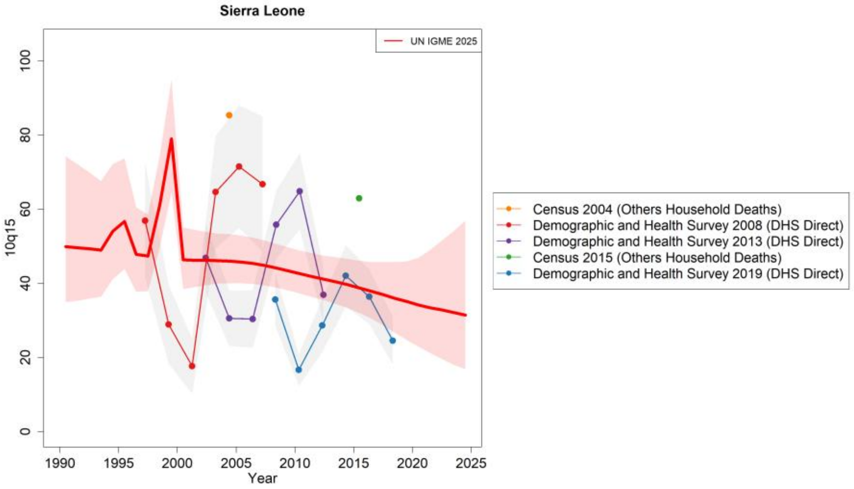
Sierra Leone
UN IGME 2025
10q15
Year
Census 2004 (Others Household Deaths)
Demographic and Health Survey 2008 (DHS Direct)
Demographic and Health Survey 2013 (DHS Direct)
Census 2015 (Others Household Deaths)
Demographic and Health Survey 2019 (DHS Direct)

Singapore (SGP)

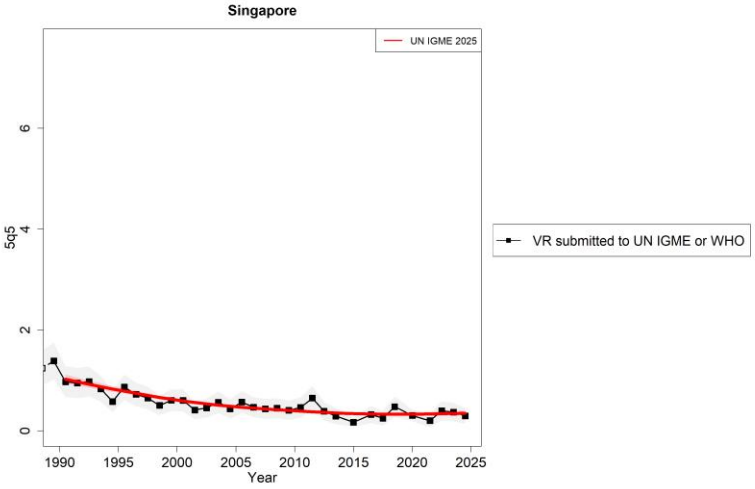

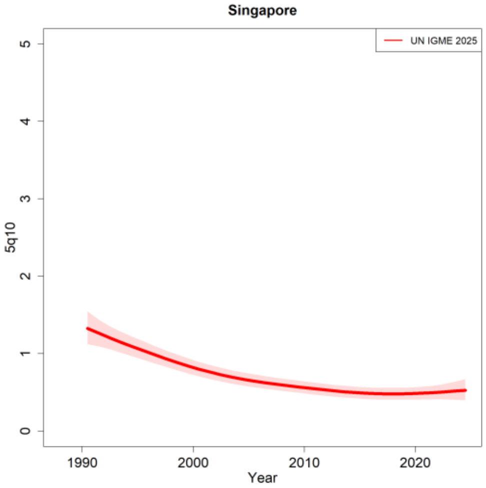

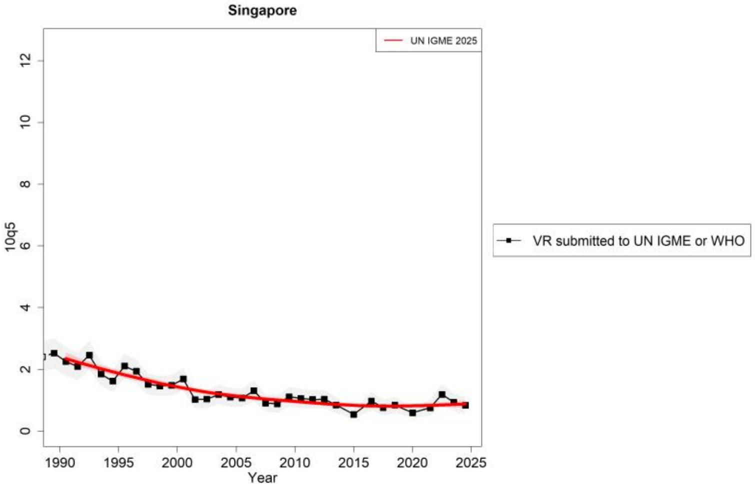

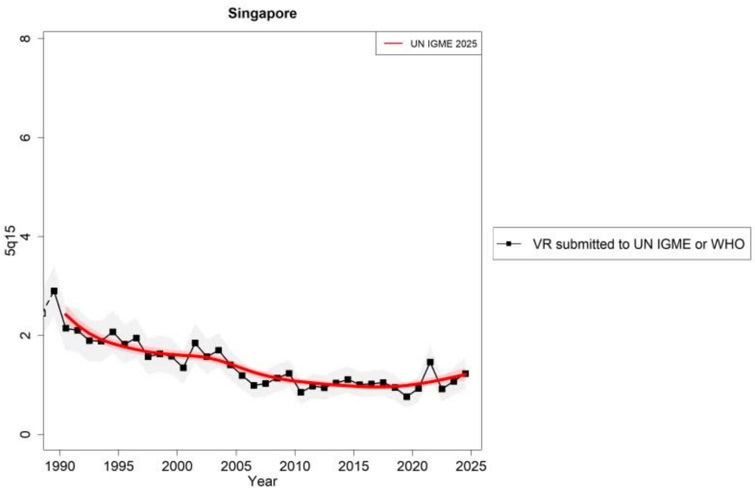
Singapore
UN IGME 2025
VR submitted to UN IGME or WHO
5q15
Year
0
2
4
6
8
1990
1995
2000
2005
2010
2015
2020
2025

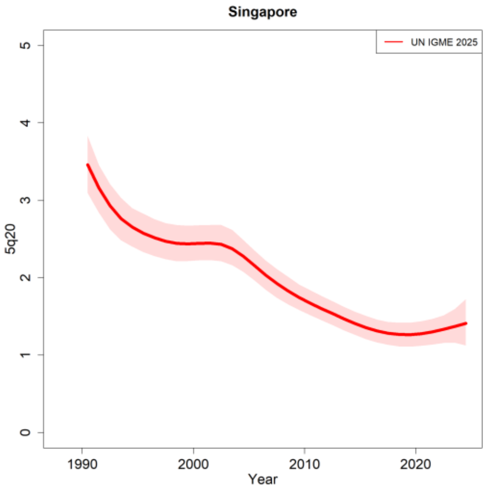
Singapore
UN IGME 2025
5q20
Year
0
1
2
3
4
5
1990
2000
2010
2020

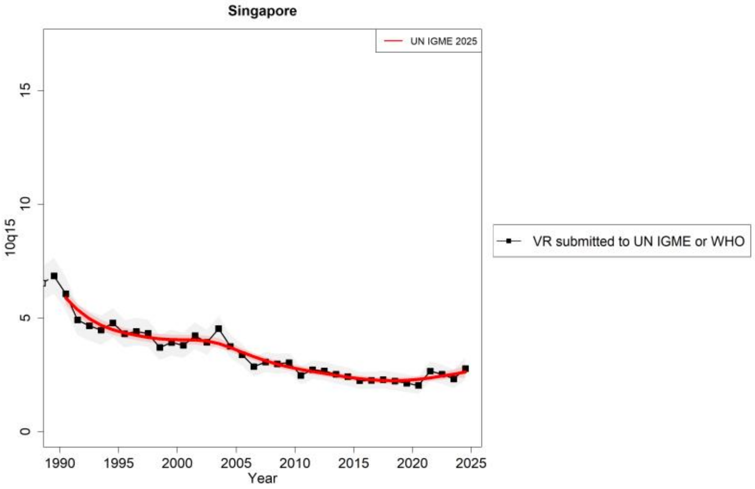
Singapore
UN IGME 2025
VR submitted to UN IGME or WHO
10q15
Year
0
5
10
15
1990
1995
2000
2005
2010
2015
2020
2025

Slovakia (SVK)

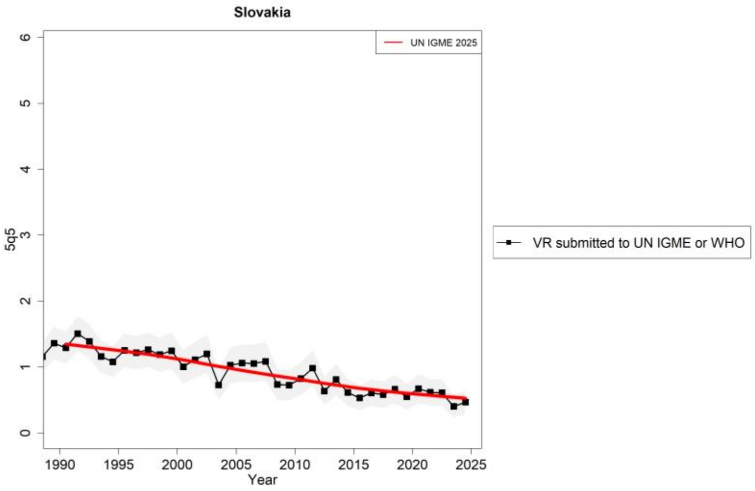

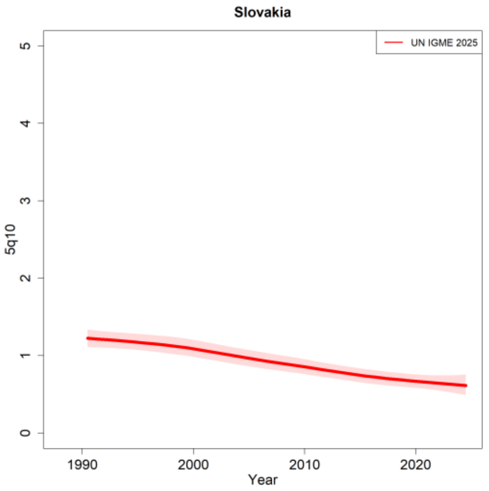

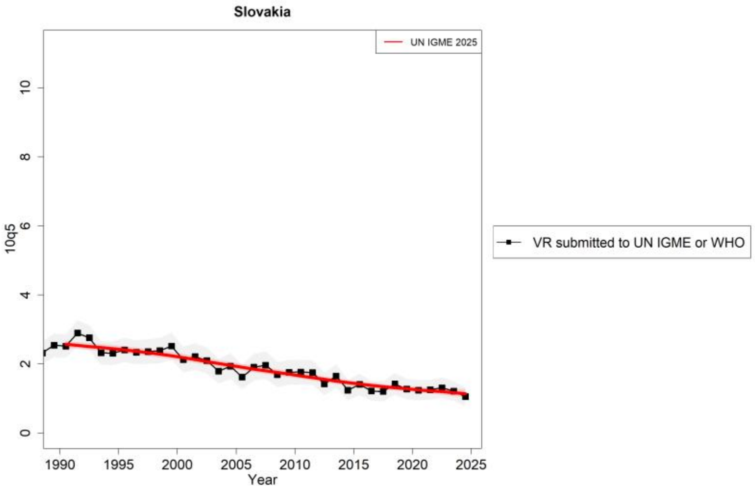

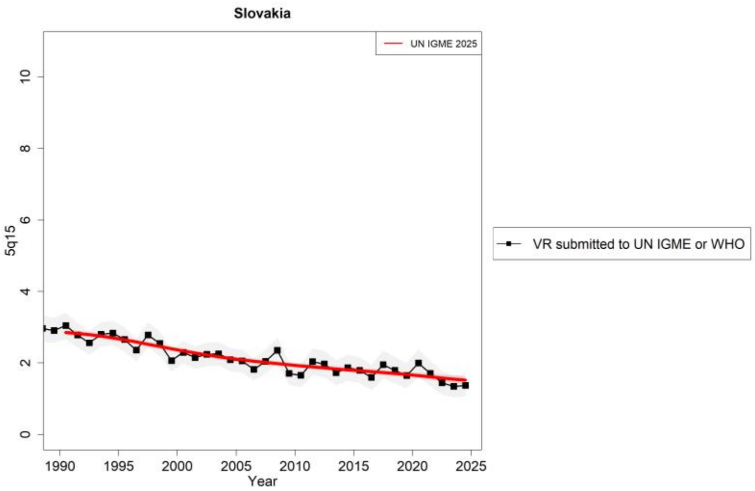
Slovakia
UN IGME 2025
VR submitted to UN IGME or WHO
5q15
Year
0
2
4
6
8
10
1990
1995
2000
2005
2010
2015
2020
2025

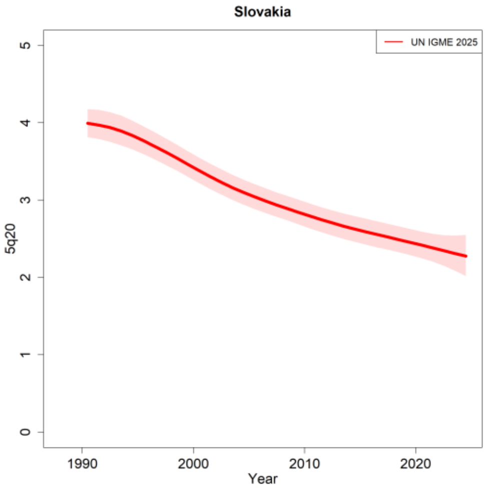
Slovakia
UN IGME 2025
5q20
Year
0
1
2
3
4
5
1990
2000
2010
2020

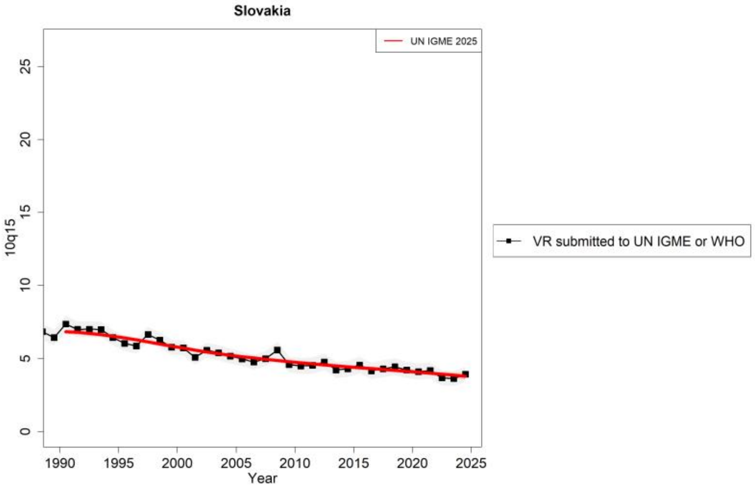
Slovakia
UN IGME 2025
VR submitted to UN IGME or WHO
10q15
Year
0
5
10
15
20
25
1990
1995
2000
2005
2010
2015
2020
2025

Slovenia (SVN)

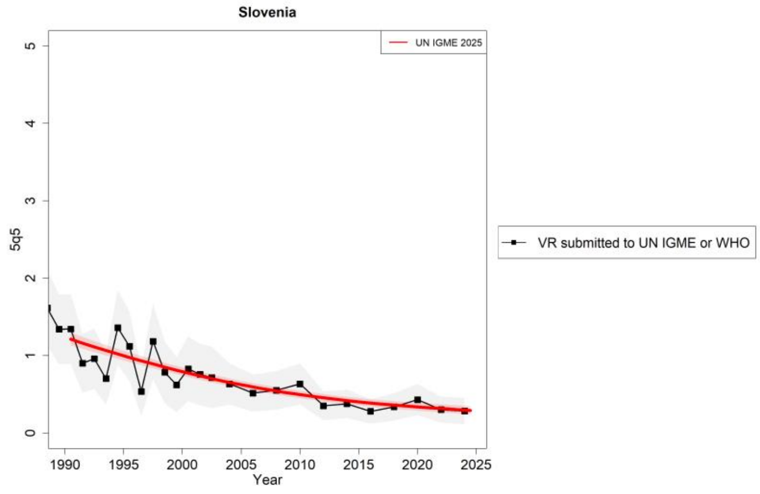

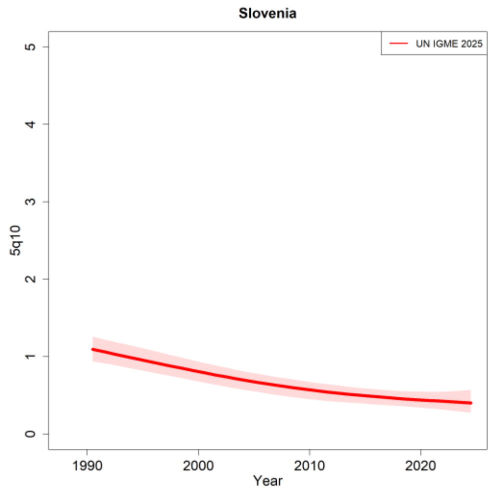

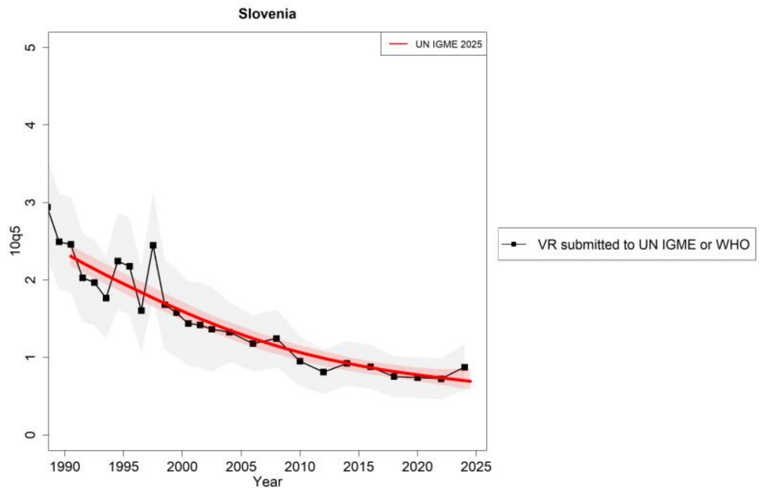

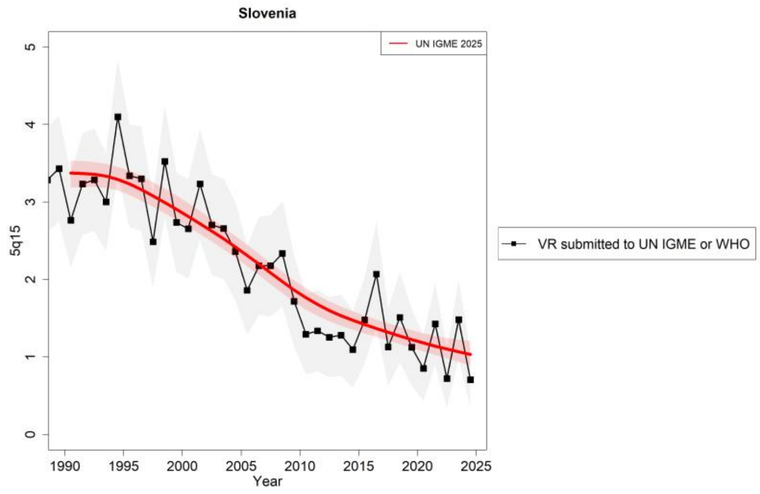
Slovenia
UN IGME 2025
VR submitted to UN IGME or WHO
5q15
Year
0
1
2
3
4
5
1990
1995
2000
2005
2010
2015
2020
2025

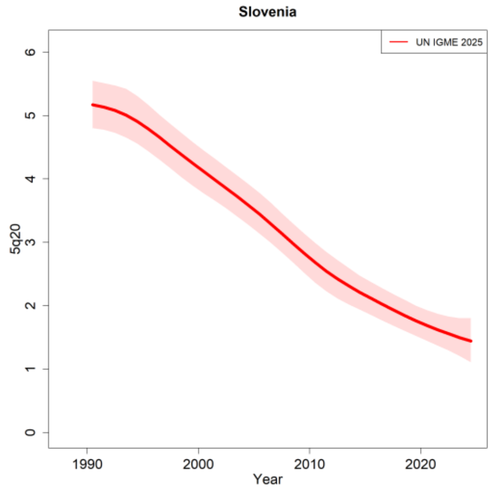
Slovenia
UN IGME 2025
5q20
Year
0
1
2
3
4
5
6
1990
2000
2010
2020

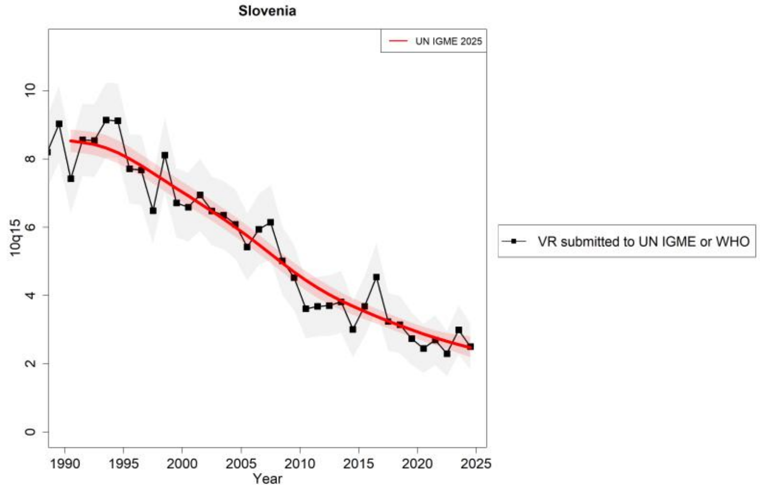
Slovenia
UN IGME 2025
VR submitted to UN IGME or WHO
10q15
Year
0
2
4
6
8
10
1990
1995
2000
2005
2010
2015
2020
2025

Solomon Islands (SLB)

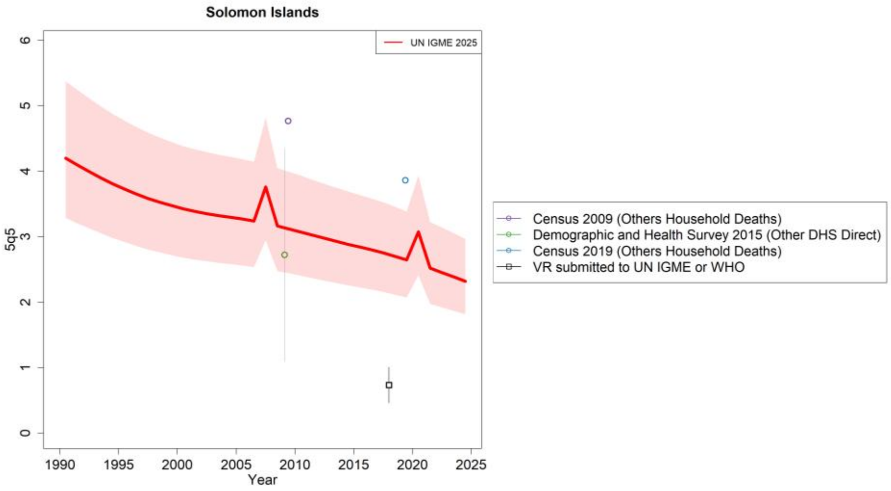

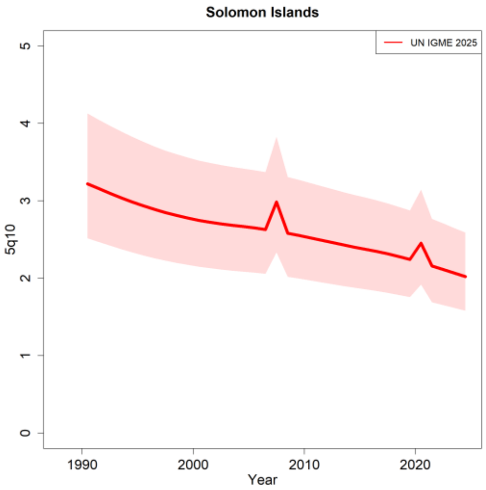

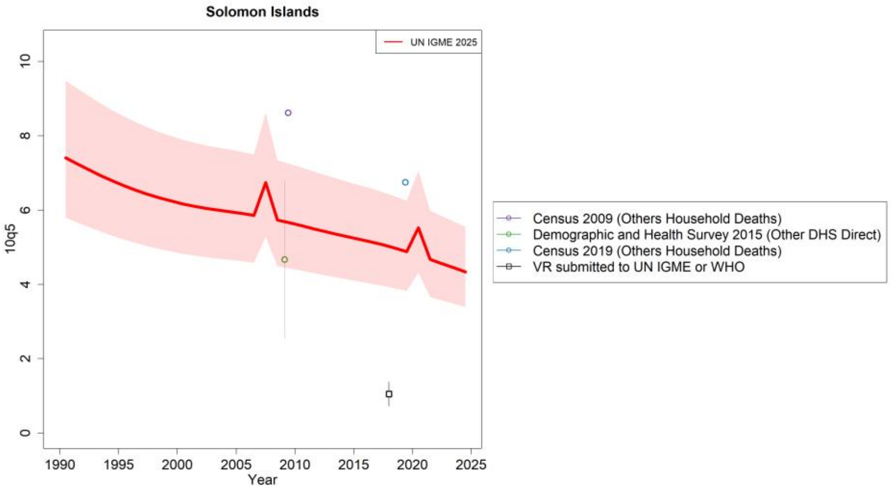

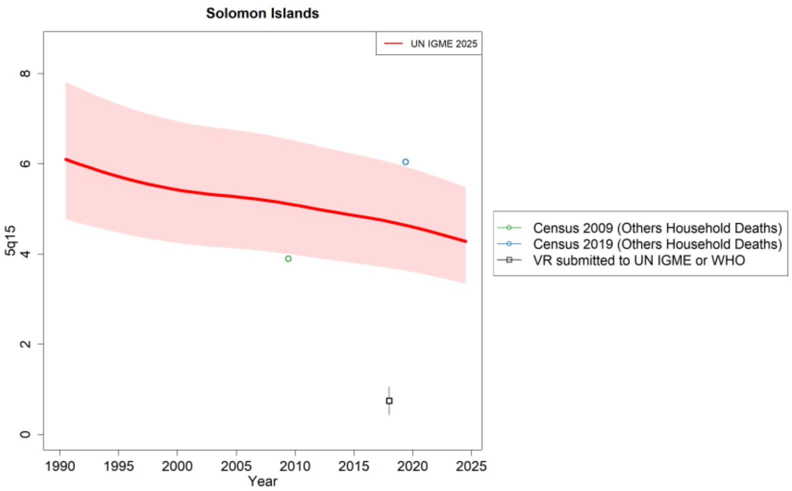
Solomon Islands
UN IGME 2025
Census 2009 (Others Household Deaths)
Census 2019 (Others Household Deaths)
VR submitted to UN IGME or WHO
5q15
Year
0
2
4
6
8
1990
1995
2000
2005
2010
2015
2020
2025

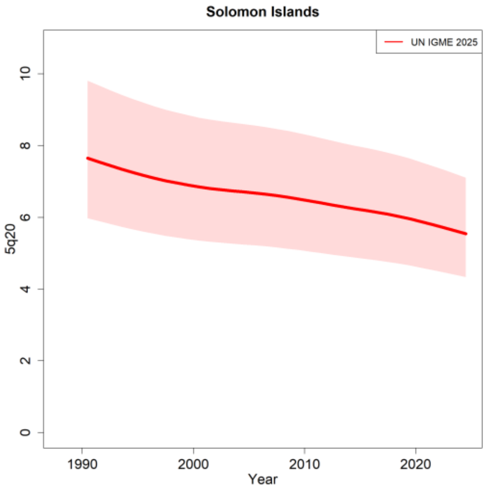
Solomon Islands
UN IGME 2025
5q20
Year
0
2
4
6
8
10
1990
2000
2010
2020

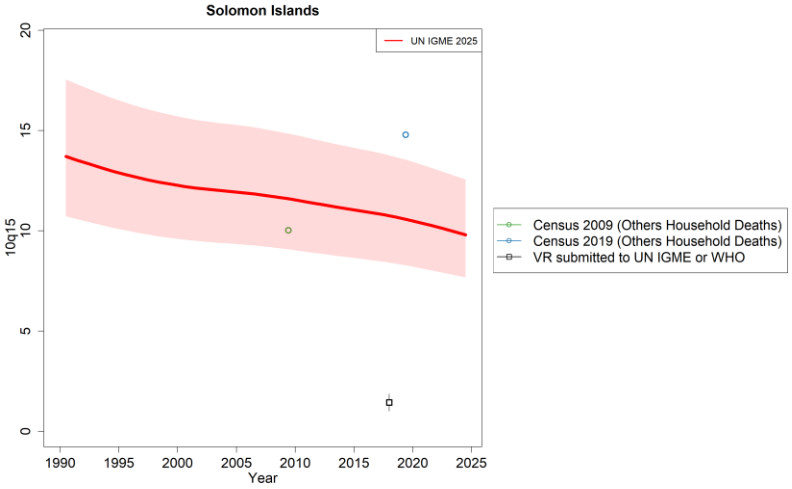
Solomon Islands
UN IGME 2025
Census 2009 (Others Household Deaths)
Census 2019 (Others Household Deaths)
VR submitted to UN IGME or WHO
10q15
Year
0
5
10
15
20
1990
1995
2000
2005
2010
2015
2020
2025

Somalia (SOM)

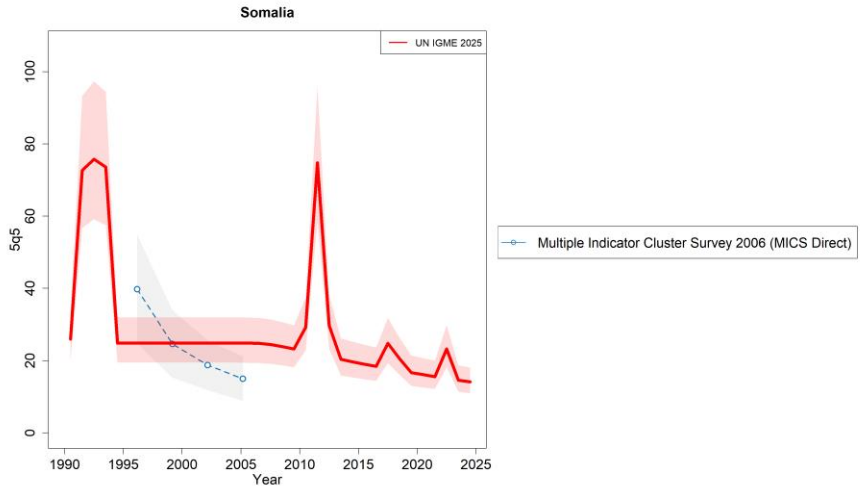
Somalia
UN IGME 2025
Multiple Indicator Cluster Survey 2006 (MICS Direct)
5q5
0
20
40
60
80
100
1990
1995
2000
2005
2010
2015
2020
2025
Year

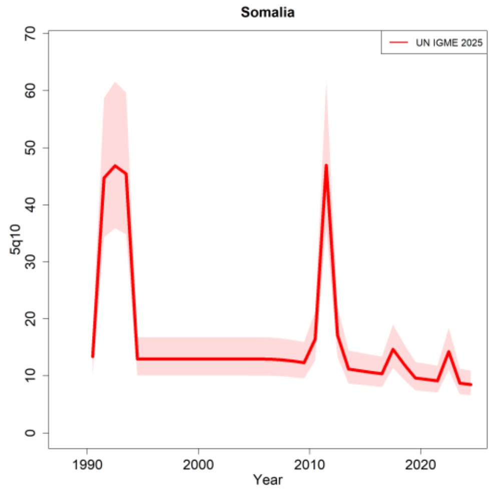
Somalia
UN IGME 2025
5q10
0
10
20
30
40
50
60
70
1990
2000
2010
2020
Year

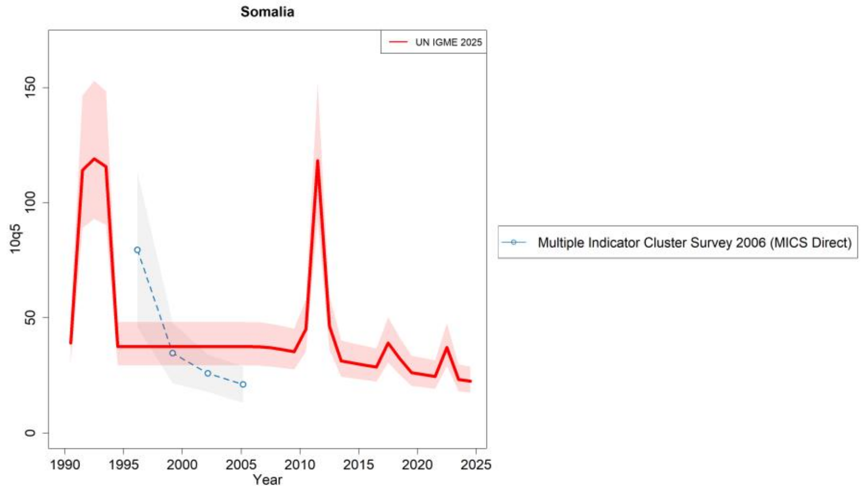
Somalia
UN IGME 2025
Multiple Indicator Cluster Survey 2006 (MICS Direct)
10q5
0
50
100
150
1990
1995
2000
2005
2010
2015
2020
2025
Year

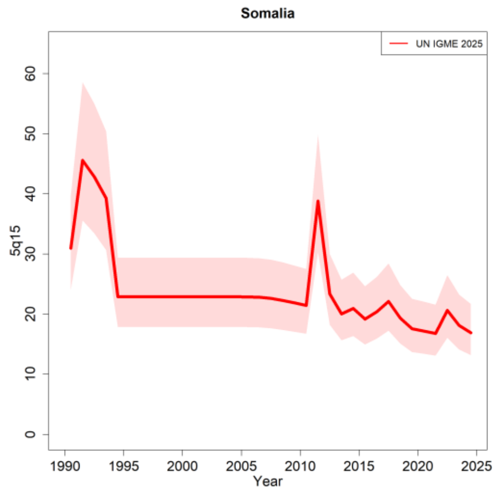
Somalia
UN IGME 2025
5q15
Year
0
10
20
30
40
50
60
1990
1995
2000
2005
2010
2015
2020
2025

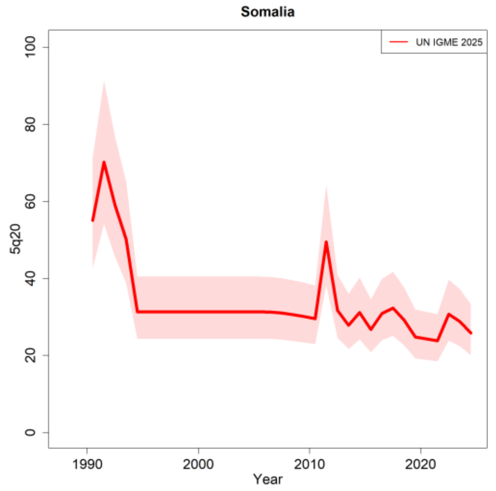
Somalia
UN IGME 2025
5q20
Year
0
20
40
60
80
100
1990
2000
2010
2020

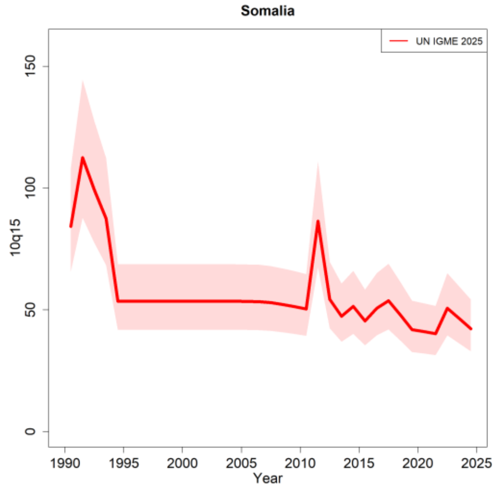
Somalia
UN IGME 2025
10q15
Year
0
50
100
150
1990
1995
2000
2005
2010
2015
2020
2025

South Africa (ZAF)

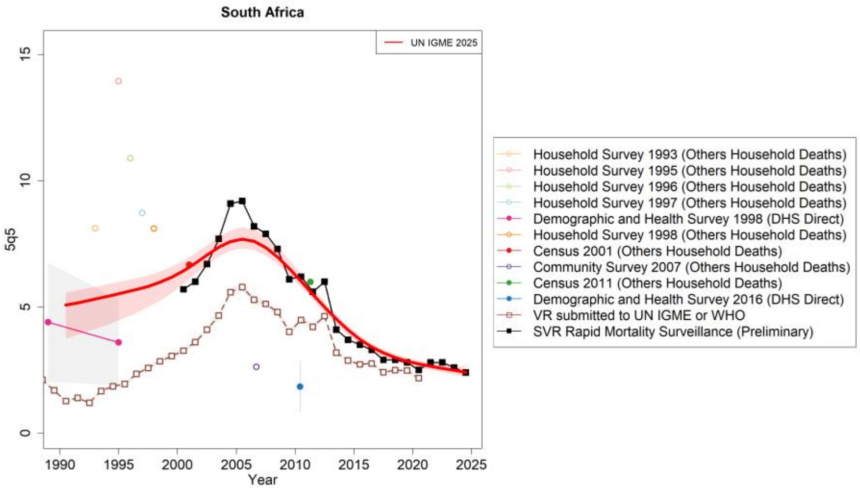

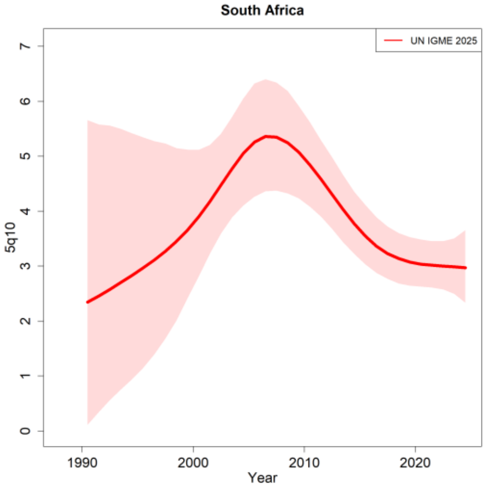

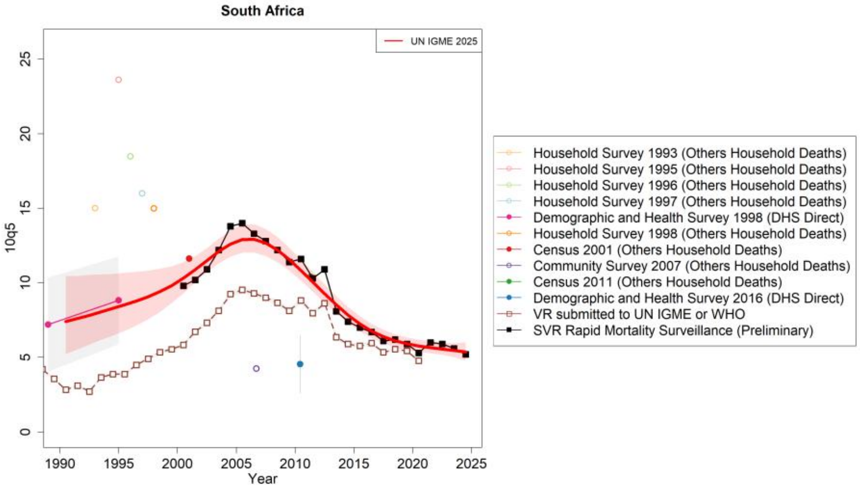

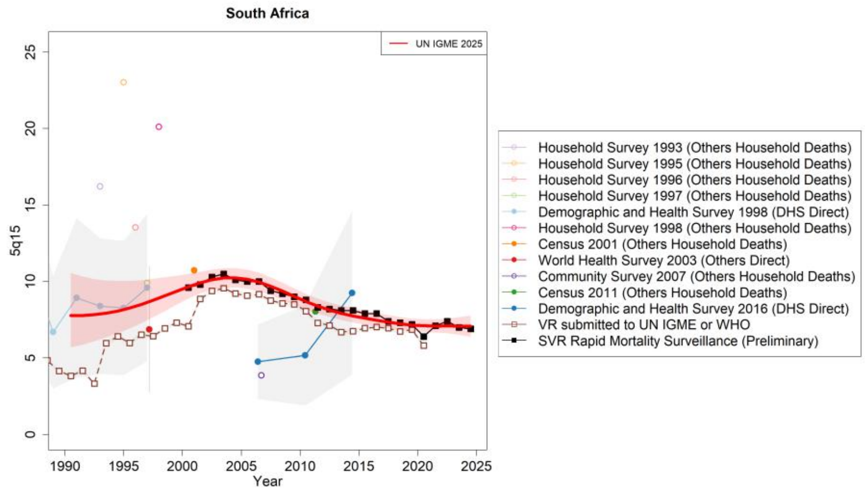
South Africa
UN IGME 2025
5q15
Year
Household Survey 1993 (Others Household Deaths)
Household Survey 1995 (Others Household Deaths)
Household Survey 1996 (Others Household Deaths)
Household Survey 1997 (Others Household Deaths)
Demographic and Health Survey 1998 (DHS Direct)
Household Survey 1998 (Others Household Deaths)
Census 2001 (Others Household Deaths)
World Health Survey 2003 (Others Direct)
Community Survey 2007 (Others Household Deaths)
Census 2011 (Others Household Deaths)
Demographic and Health Survey 2016 (DHS Direct)
VR submitted to UN IGME or WHO
SVR Rapid Mortality Surveillance (Preliminary)

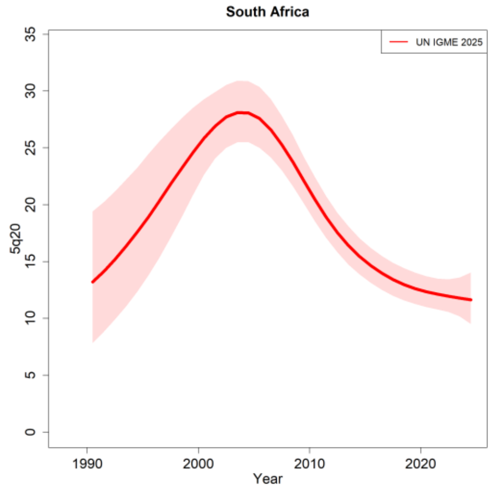
South Africa
UN IGME 2025
5q20
Year

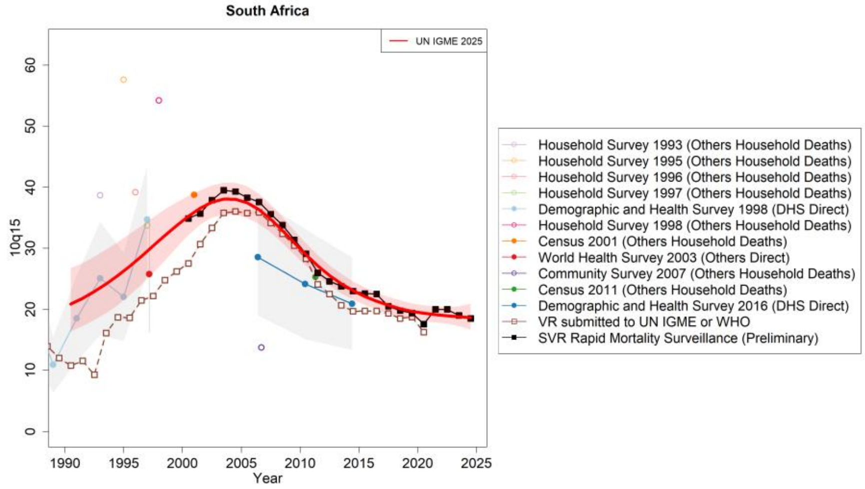
South Africa
UN IGME 2025
10q15
Year
Household Survey 1993 (Others Household Deaths)
Household Survey 1995 (Others Household Deaths)
Household Survey 1996 (Others Household Deaths)
Household Survey 1997 (Others Household Deaths)
Demographic and Health Survey 1998 (DHS Direct)
Household Survey 1998 (Others Household Deaths)
Census 2001 (Others Household Deaths)
World Health Survey 2003 (Others Direct)
Community Survey 2007 (Others Household Deaths)
Census 2011 (Others Household Deaths)
Demographic and Health Survey 2016 (DHS Direct)
VR submitted to UN IGME or WHO
SVR Rapid Mortality Surveillance (Preliminary)

South Sudan (SSD)

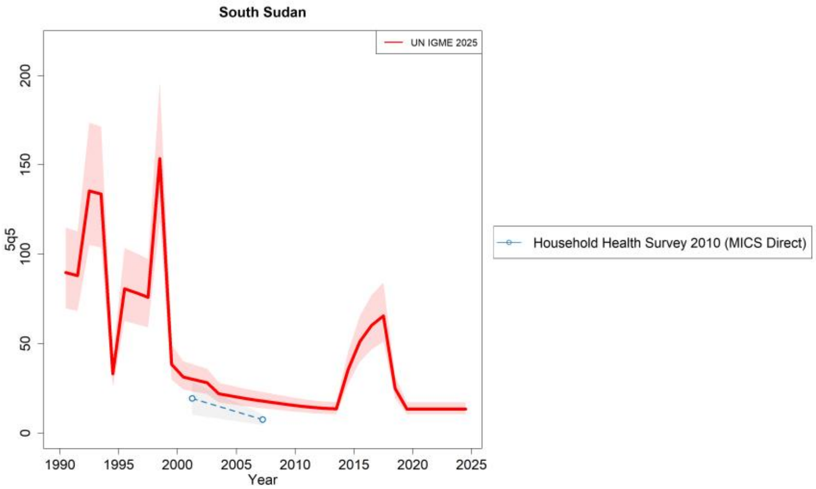

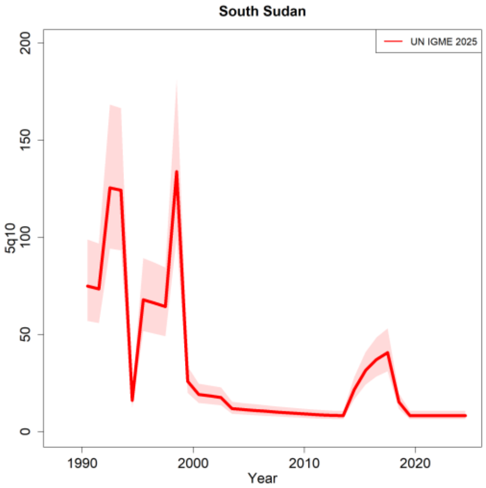

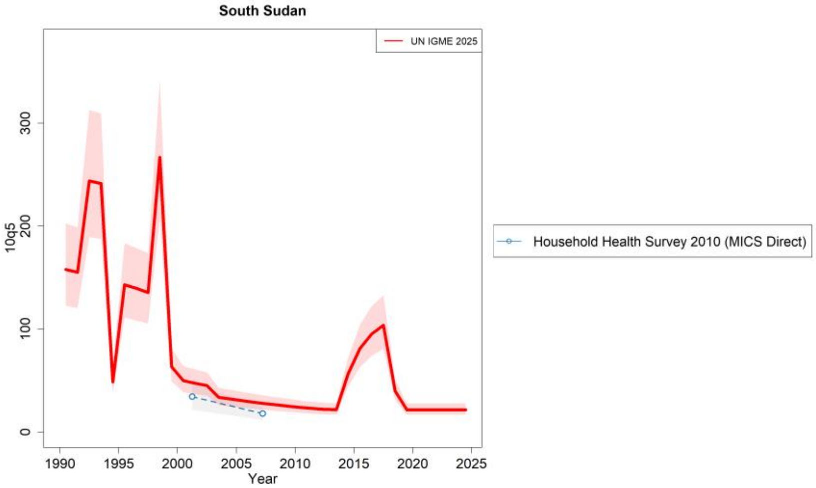

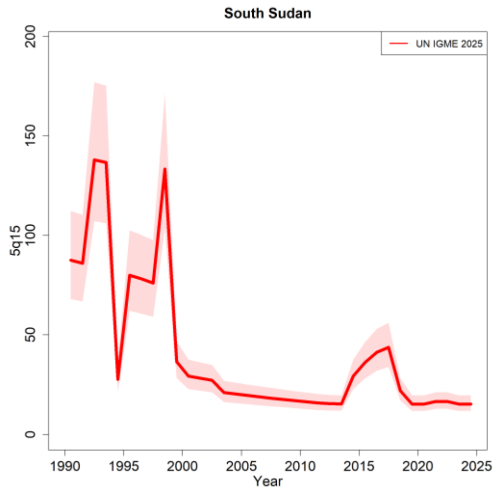
South Sudan
UN IGME 2025
5q15
0
50
100
150
200
1990
1995
2000
2005
2010
2015
2020
2025
Year

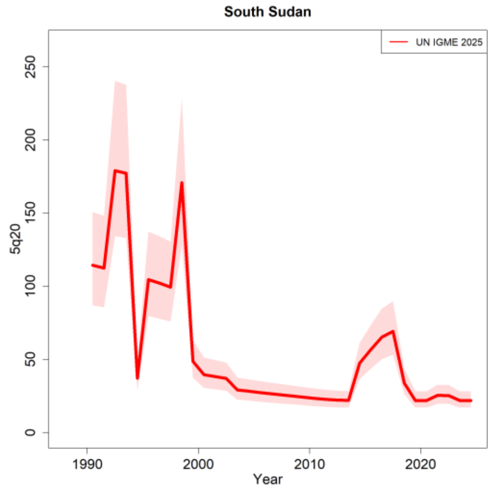
South Sudan
UN IGME 2025
5q20
0
50
100
150
200
250
1990
2000
2010
2020
Year

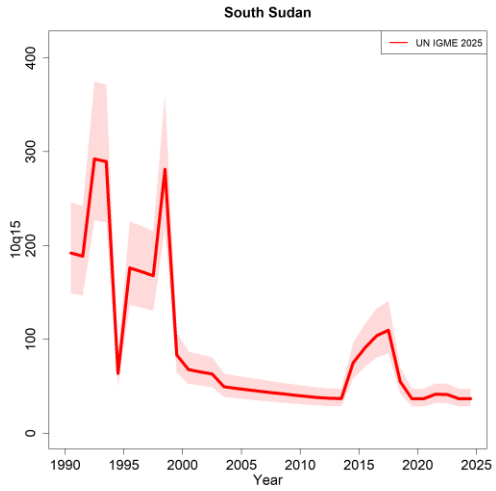
South Sudan
UN IGME 2025
10q15
0
100
200
300
400
1990
1995
2000
2005
2010
2015
2020
2025
Year

Spain (ESP)

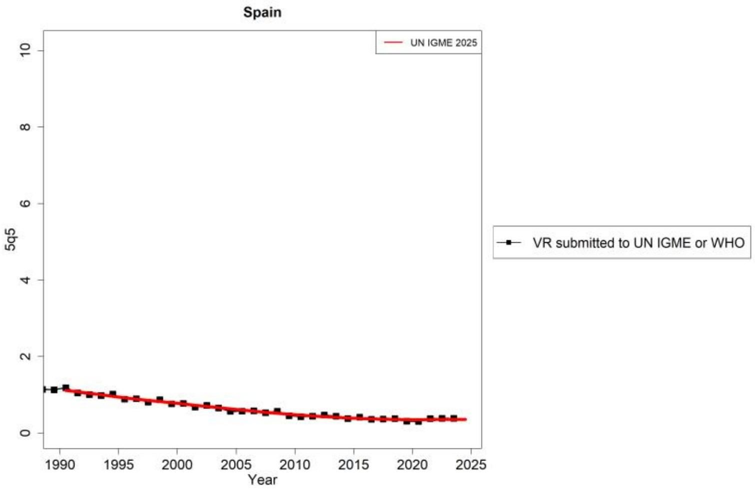
Spain
UN IGME 2025
VR submitted to UN IGME or WHO
5q5
0
2
4
6
8
10
1990
1995
2000
2005
2010
2015
2020
2025
Year

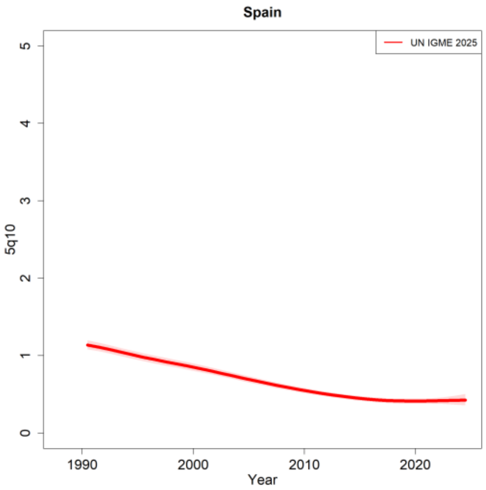
Spain
UN IGME 2025
5q10
0
1
2
3
4
5
1990
2000
2010
2020
Year

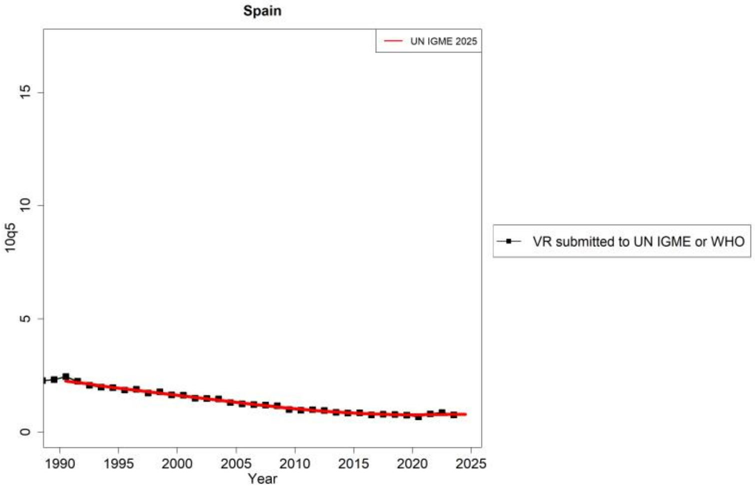
Spain
UN IGME 2025
VR submitted to UN IGME or WHO
10q5
0
5
10
15
1990
1995
2000
2005
2010
2015
2020
2025
Year

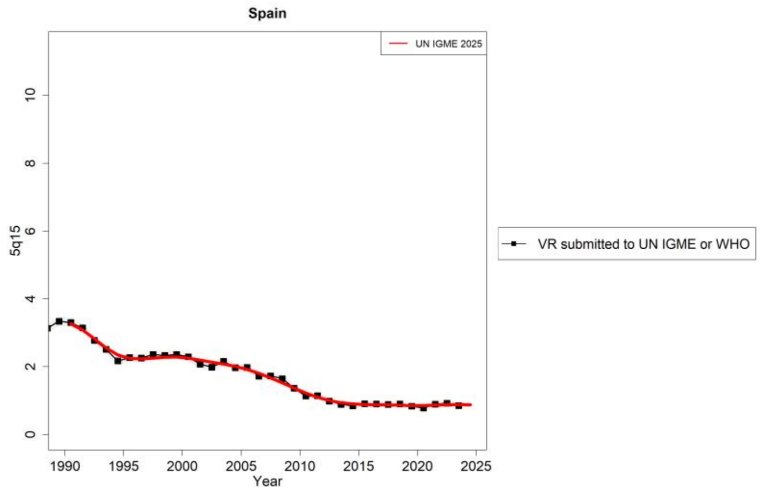
Spain
UN IGME 2025
VR submitted to UN IGME or WHO
5q15
Year

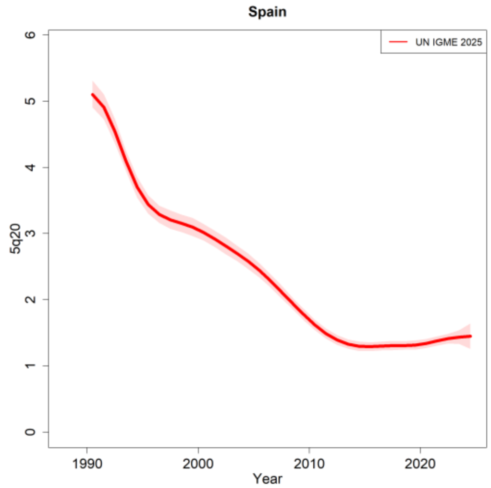
Spain
UN IGME 2025
5q20
Year

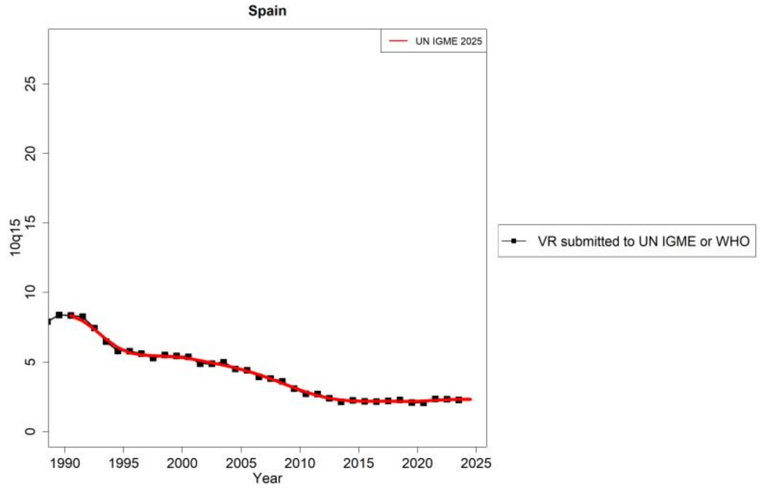
Spain
UN IGME 2025
VR submitted to UN IGME or WHO
10q15
Year

Sri Lanka (LKA)

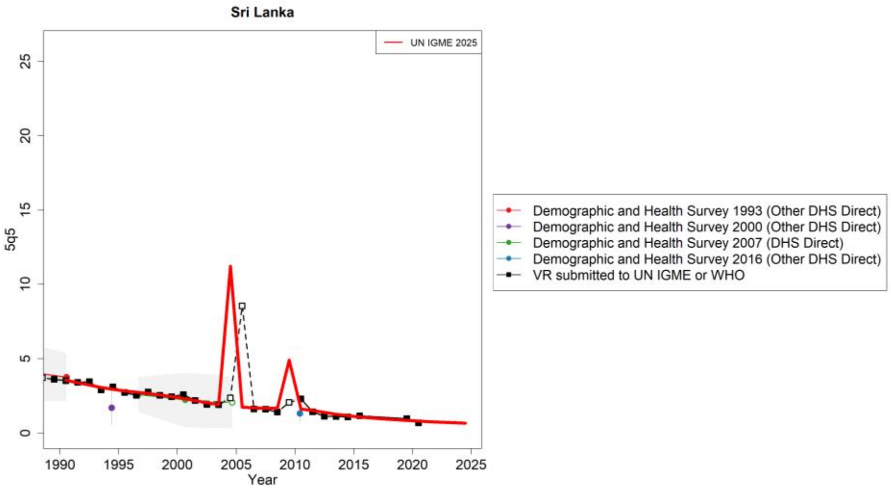

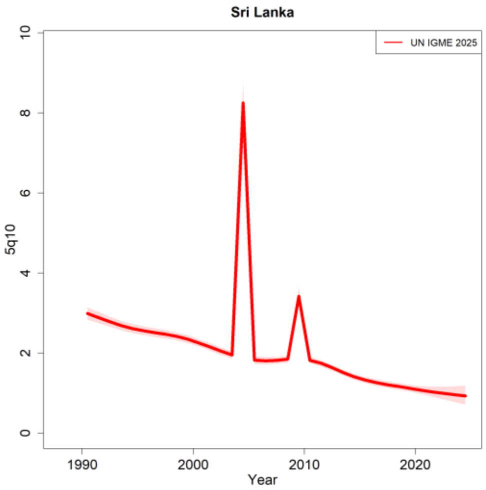

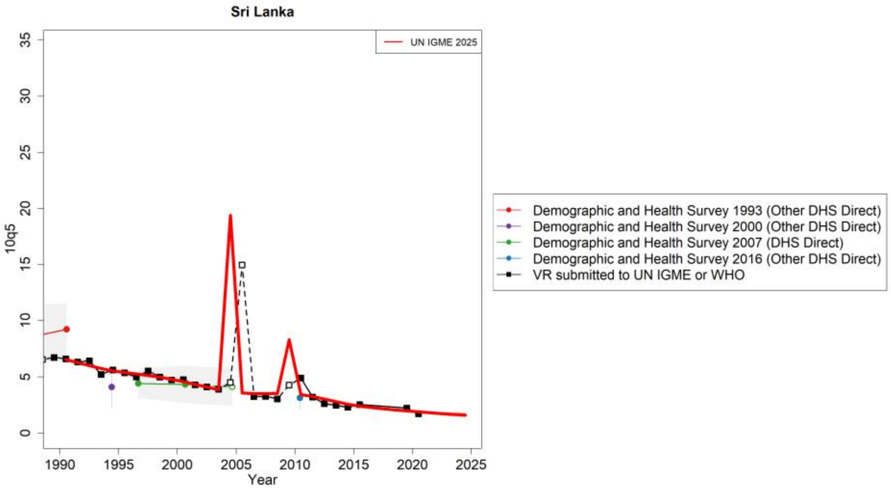

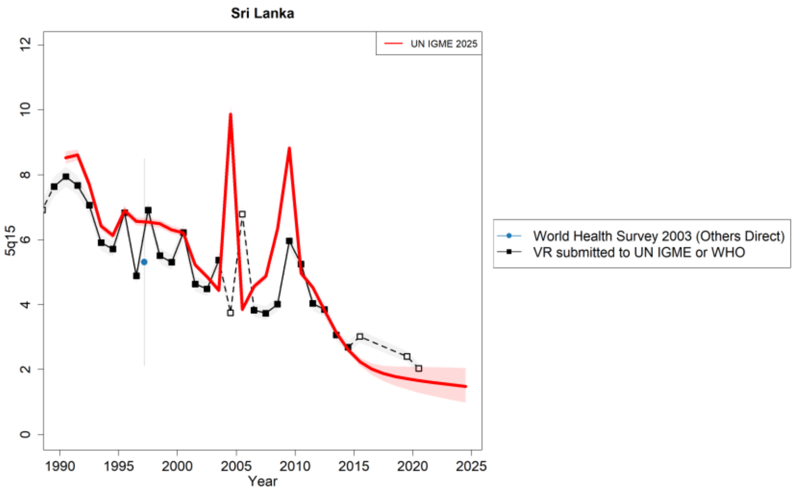
Sri Lanka
UN IGME 2025
World Health Survey 2003 (Others Direct)
VR submitted to UN IGME or WHO
5q15
Year
0
2
4
6
8
10
12
1990
1995
2000
2005
2010
2015
2020
2025

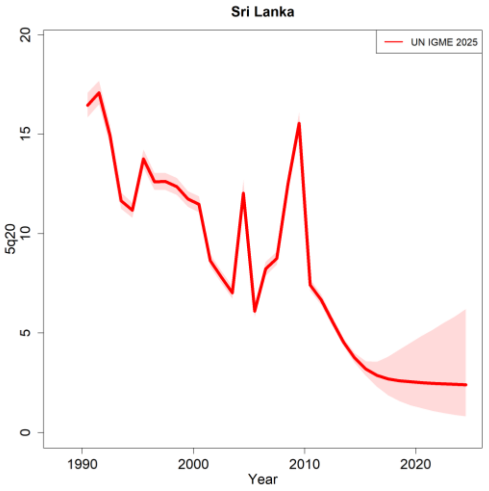
Sri Lanka
UN IGME 2025
5q20
Year
0
5
10
15
20
1990
2000
2010
2020

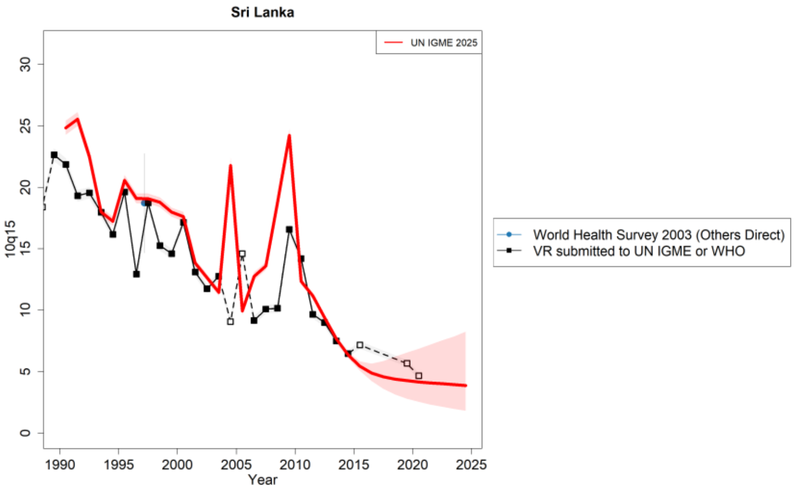
Sri Lanka
UN IGME 2025
World Health Survey 2003 (Others Direct)
VR submitted to UN IGME or WHO
10q15
Year
0
5
10
15
20
25
30
1990
1995
2000
2005
2010
2015
2020
2025

State of Palestine (PSE)

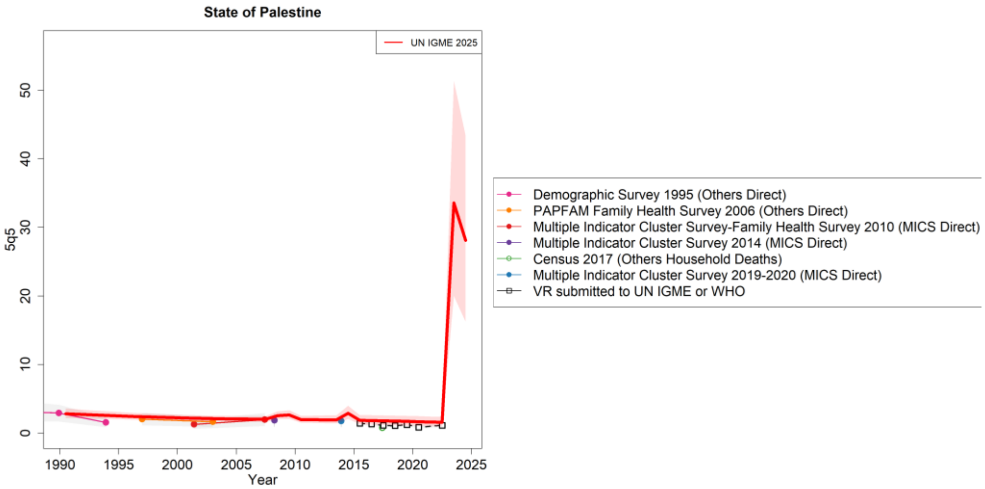

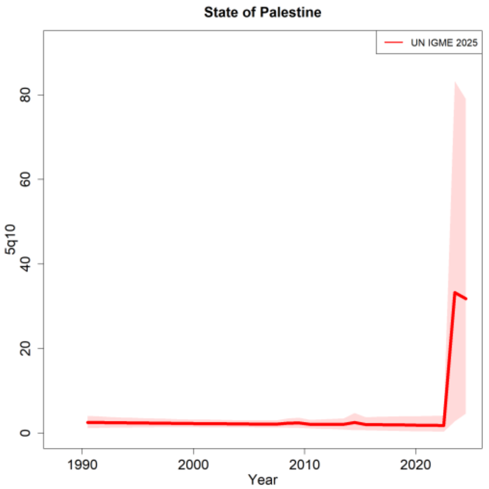

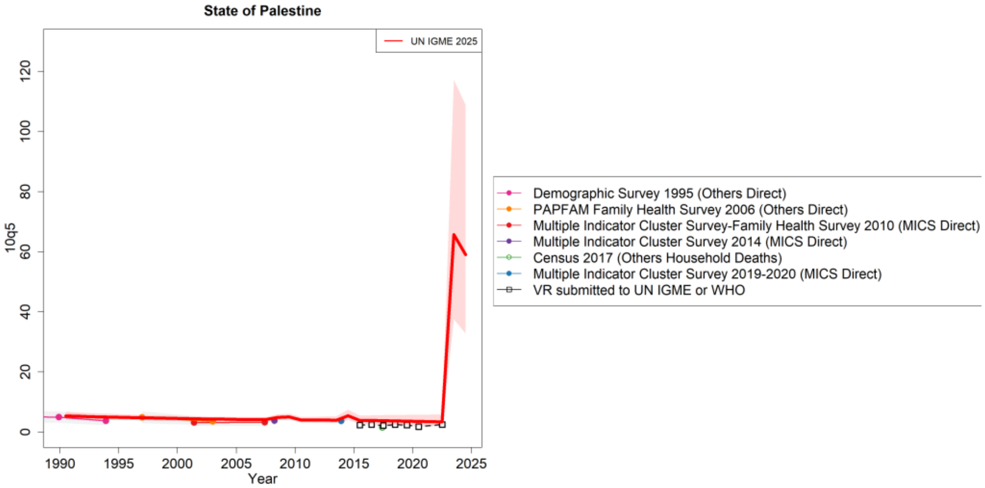

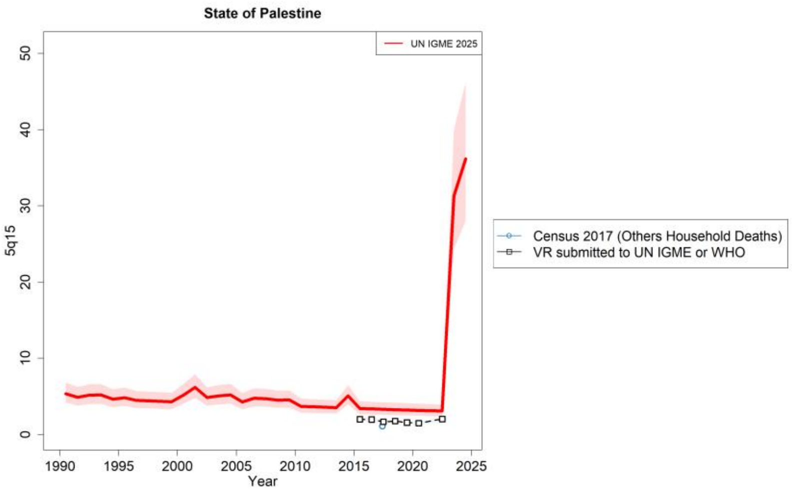
State of Palestine
UN IGME 2025
5q15
Year
Census 2017 (Others Household Deaths)
VR submitted to UN IGME or WHO

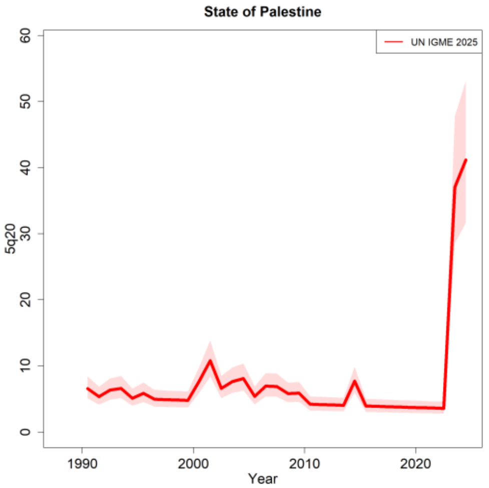
State of Palestine
UN IGME 2025
5q20
Year

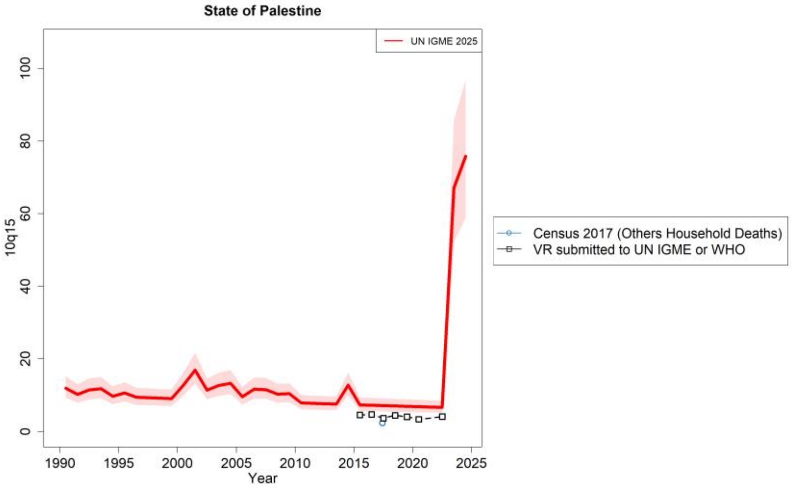
State of Palestine
UN IGME 2025
10q15
Year
Census 2017 (Others Household Deaths)
VR submitted to UN IGME or WHO

Sudan (SDN)

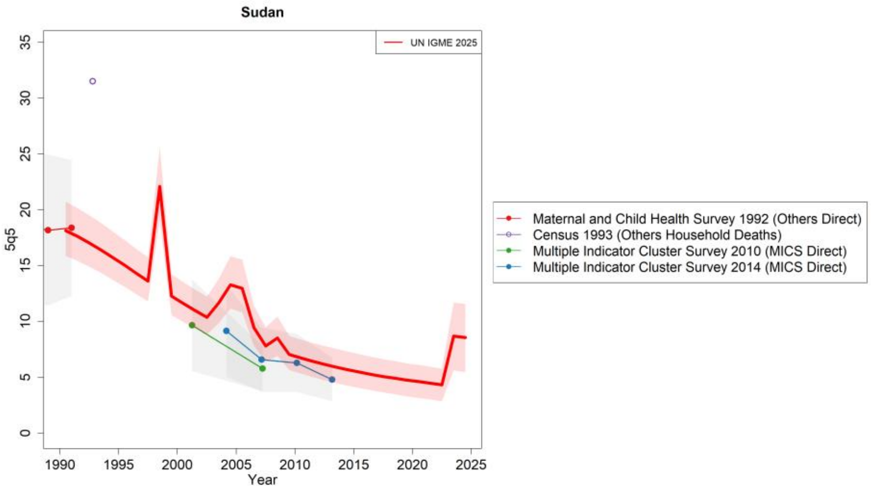
Sudan
UN IGME 2025
5q5
Year
Maternal and Child Health Survey 1992 (Others Direct)
Census 1993 (Others Household Deaths)
Multiple Indicator Cluster Survey 2010 (MICS Direct)
Multiple Indicator Cluster Survey 2014 (MICS Direct)

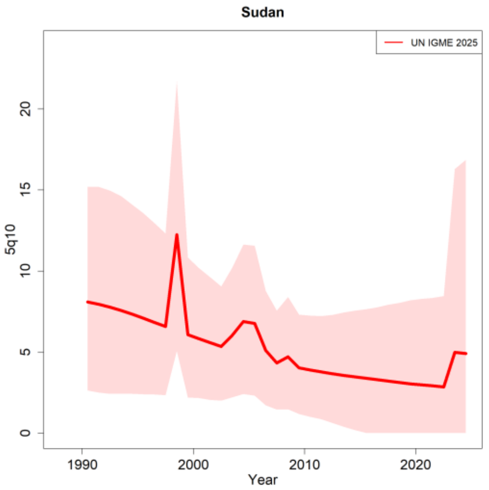
Sudan
UN IGME 2025
5q10
Year

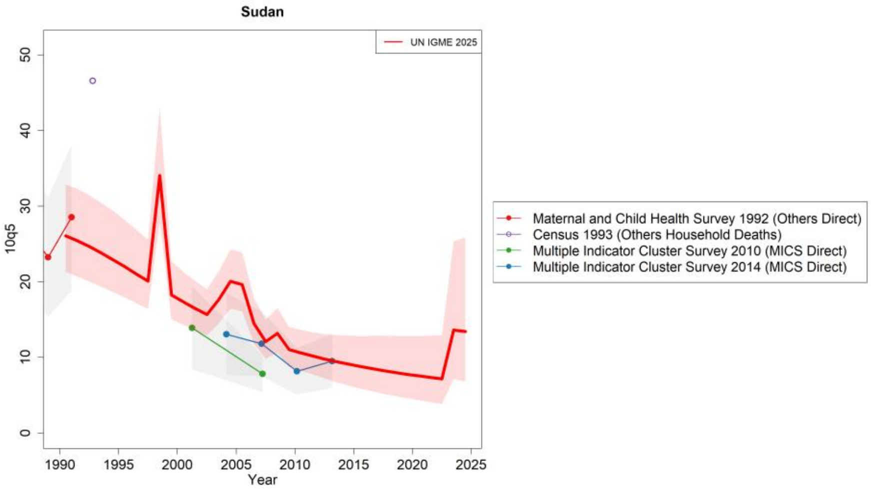
Sudan
UN IGME 2025
10q5
Year
Maternal and Child Health Survey 1992 (Others Direct)
Census 1993 (Others Household Deaths)
Multiple Indicator Cluster Survey 2010 (MICS Direct)
Multiple Indicator Cluster Survey 2014 (MICS Direct)

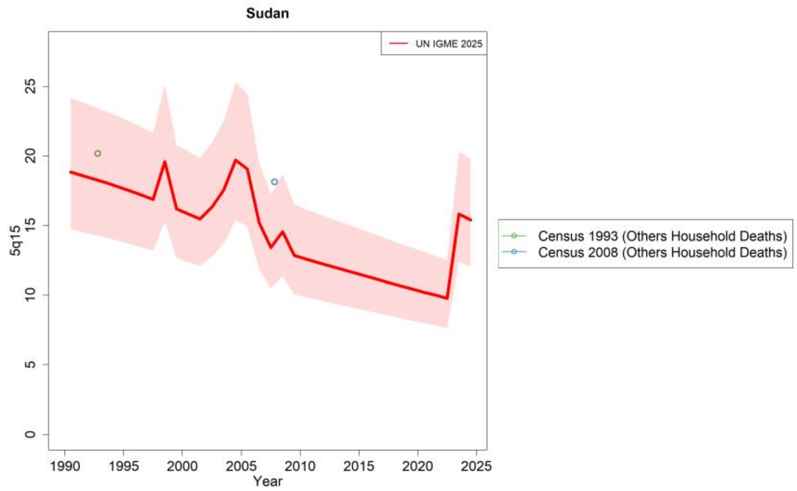
Sudan
UN IGME 2025
5q15
Year
Census 1993 (Others Household Deaths)
Census 2008 (Others Household Deaths)

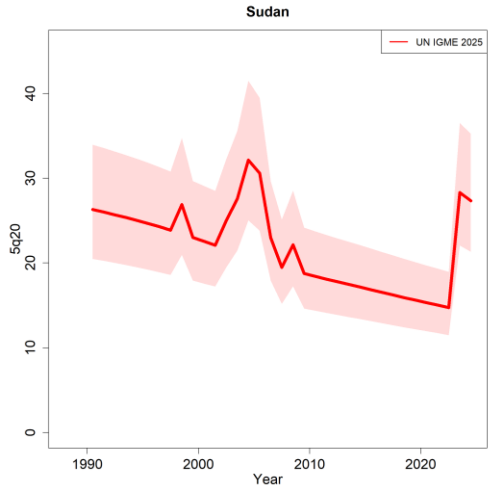
Sudan
UN IGME 2025
5q20
Year

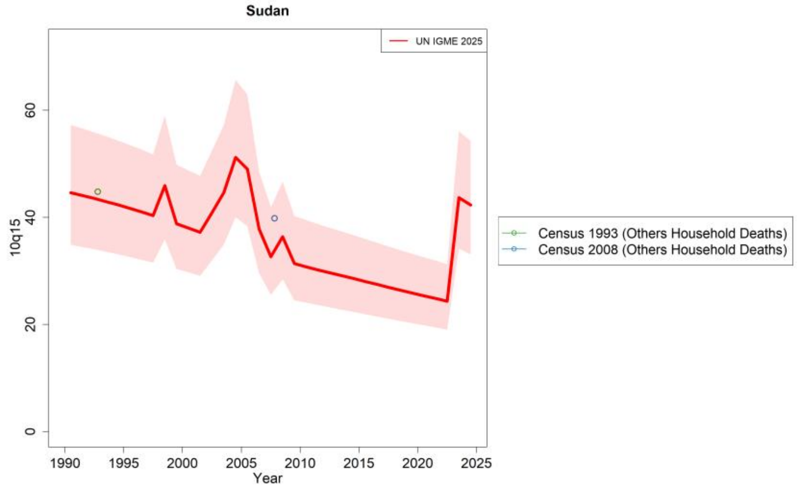
Sudan
UN IGME 2025
10q15
Year
Census 1993 (Others Household Deaths)
Census 2008 (Others Household Deaths)

Suriname (SUR)

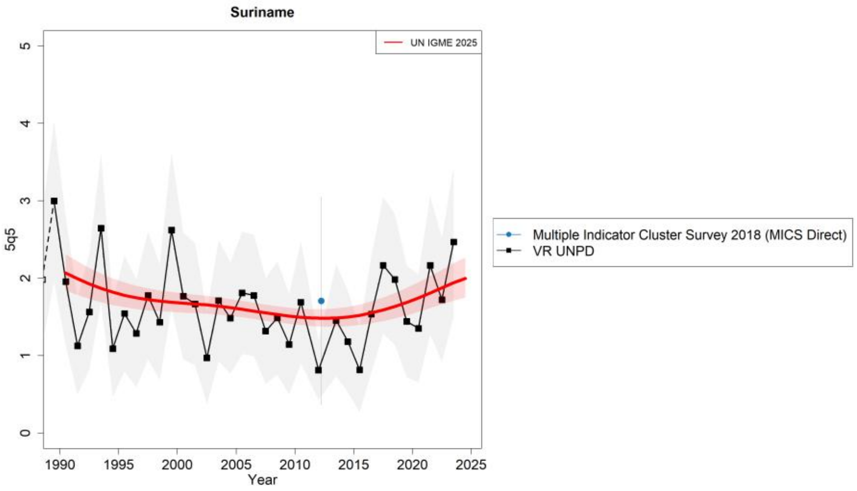

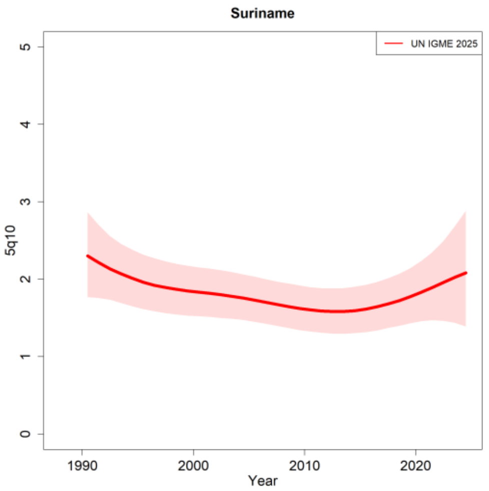

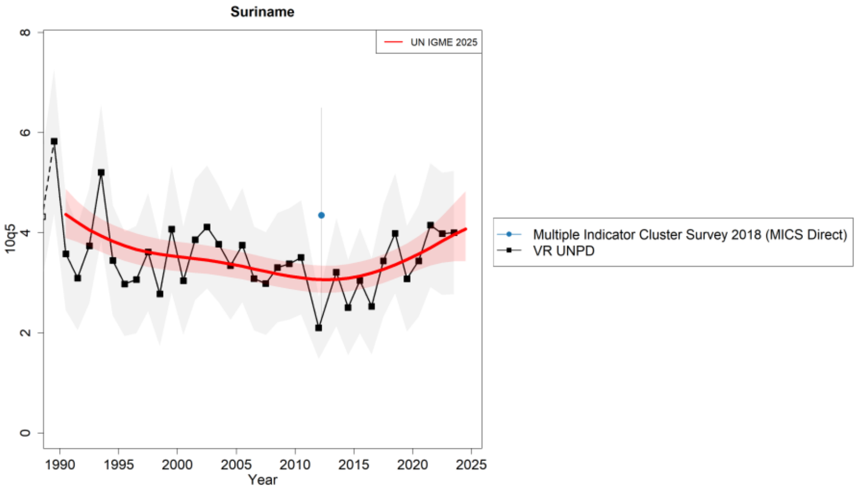

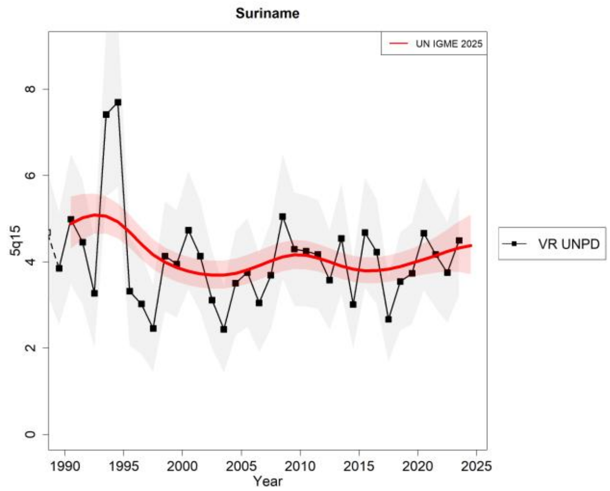
Suriname
UN IGME 2025
VR UNPD
5q15
Year

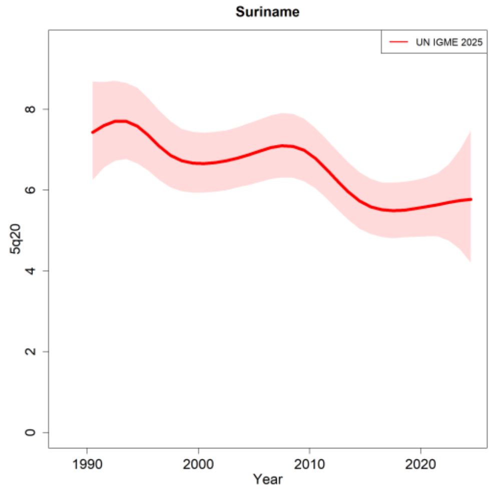
Suriname
UN IGME 2025
5q20
Year

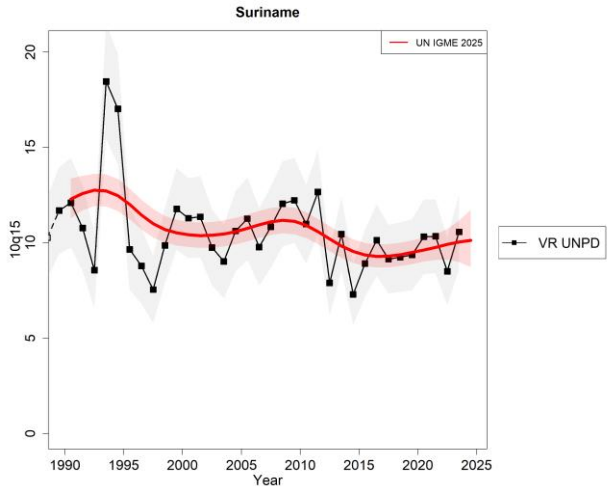
Suriname
UN IGME 2025
VR UNPD
10q15
Year

Sweden (SWE)

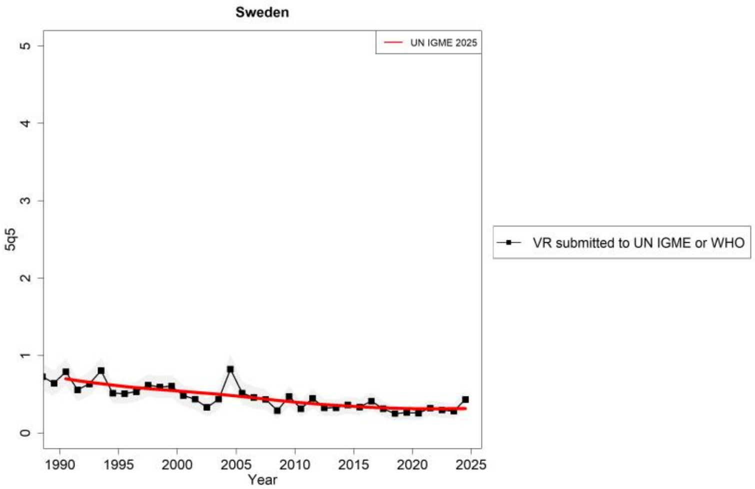
Sweden
UN IGME 2025
VR submitted to UN IGME or WHO
5q5
Year
0
1
2
3
4
5
1990
1995
2000
2005
2010
2015
2020
2025

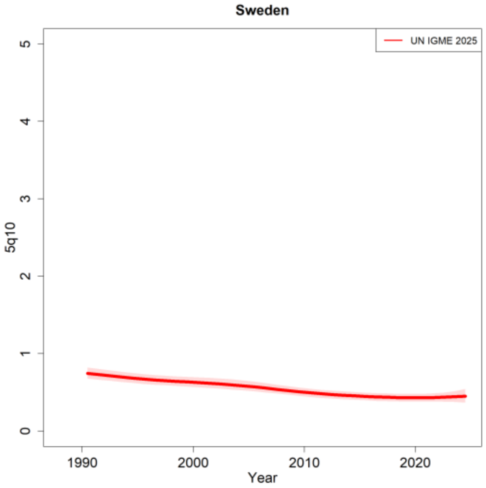
Sweden
UN IGME 2025
5q10
Year
0
1
2
3
4
5
1990
2000
2010
2020

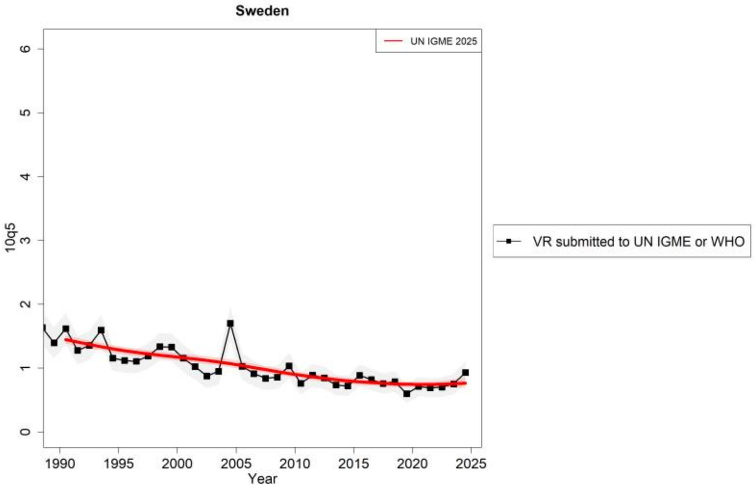
Sweden
UN IGME 2025
VR submitted to UN IGME or WHO
10q5
Year
0
1
2
3
4
5
6
1990
1995
2000
2005
2010
2015
2020
2025

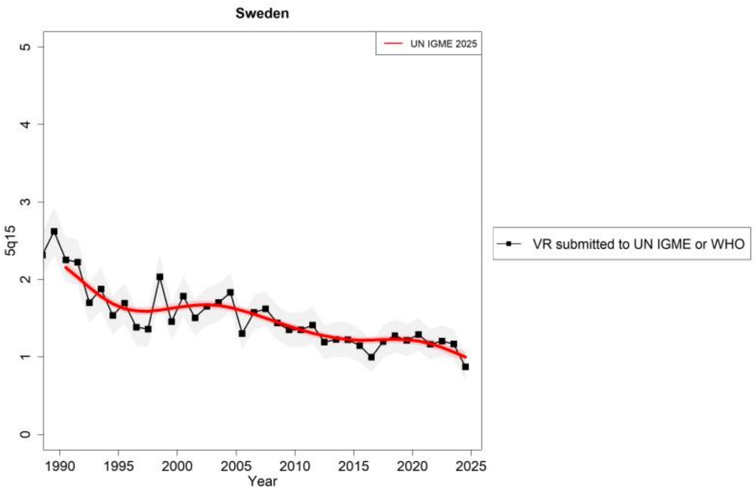
Sweden
UN IGME 2025
VR submitted to UN IGME or WHO
5q15
0
1
2
3
4
5
1990
1995
2000
2005
2010
2015
2020
2025
Year

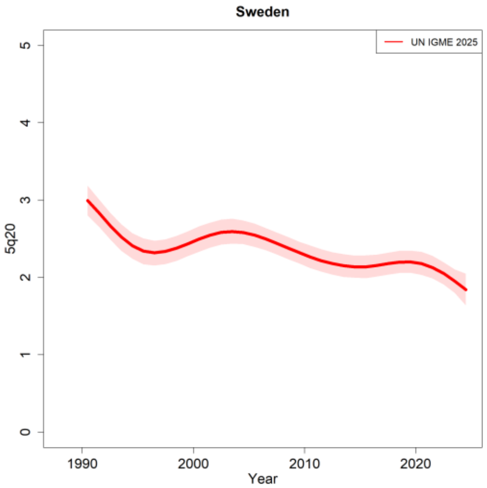
Sweden
UN IGME 2025
5q20
0
1
2
3
4
5
1990
2000
2010
2020
Year

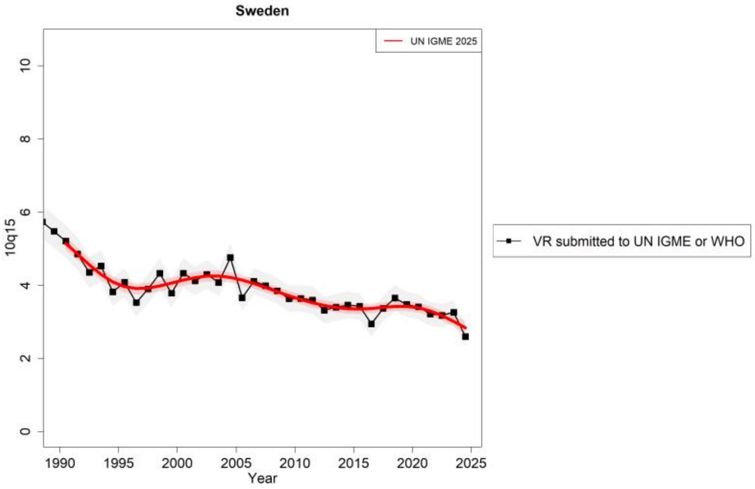
Sweden
UN IGME 2025
VR submitted to UN IGME or WHO
10q15
0
2
4
6
8
10
1990
1995
2000
2005
2010
2015
2020
2025
Year

Switzerland (CHE)

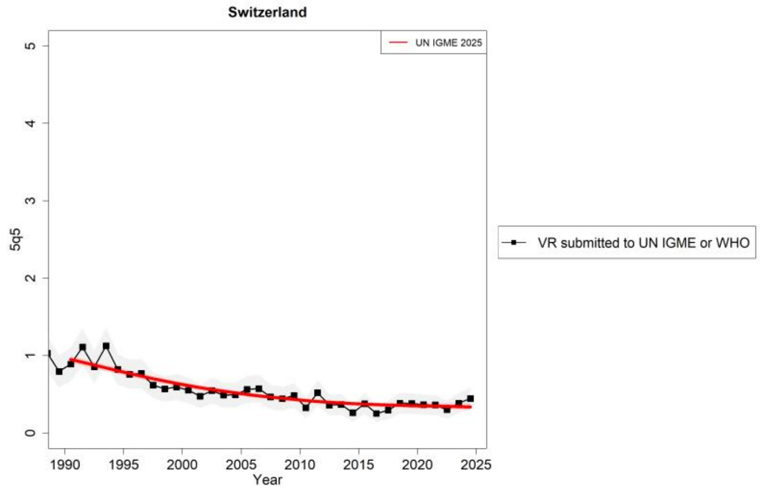

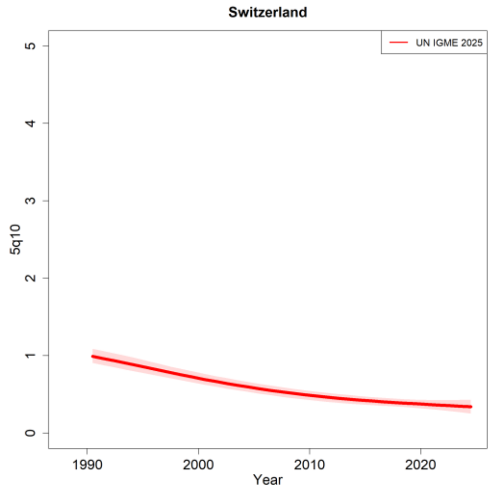

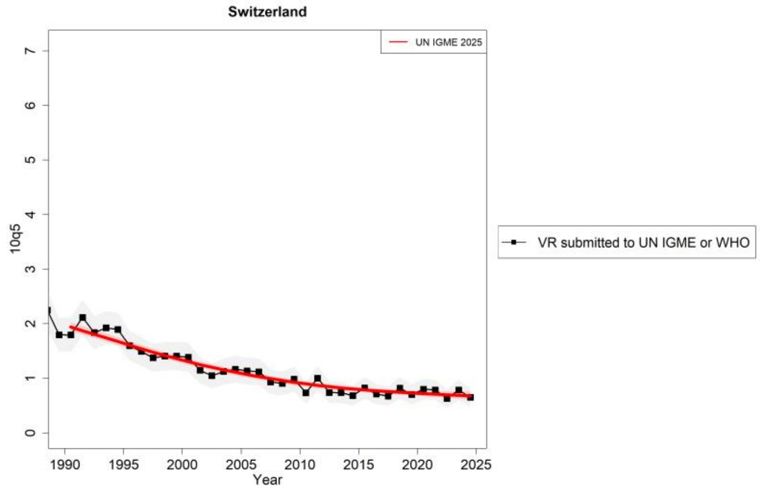

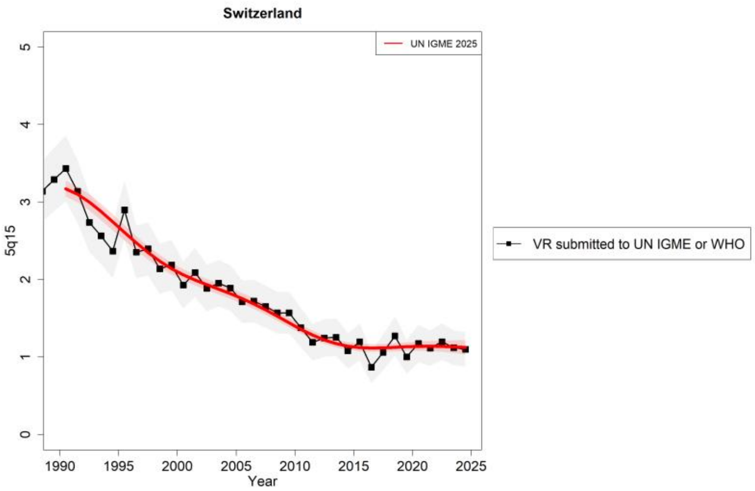
Switzerland
UN IGME 2025
VR submitted to UN IGME or WHO
5q15
0
1
2
3
4
5
1990
1995
2000
2005
2010
2015
2020
2025
Year

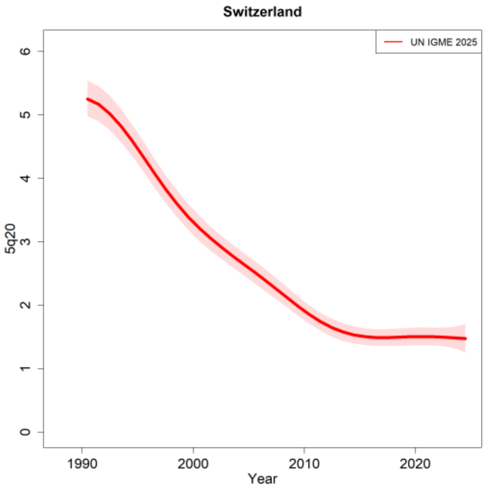
Switzerland
UN IGME 2025
5q20
0
1
2
3
4
5
6
1990
2000
2010
2020
Year

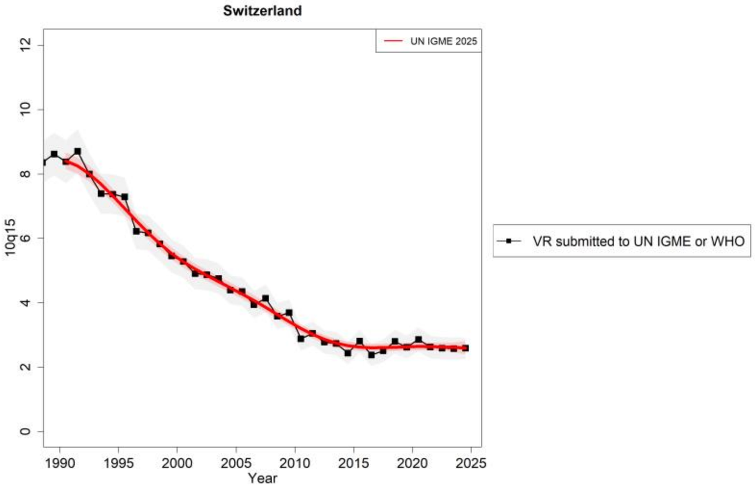
Switzerland
UN IGME 2025
VR submitted to UN IGME or WHO
10q15
0
2
4
6
8
10
12
1990
1995
2000
2005
2010
2015
2020
2025
Year

Syrian Arab Republic (SYR)

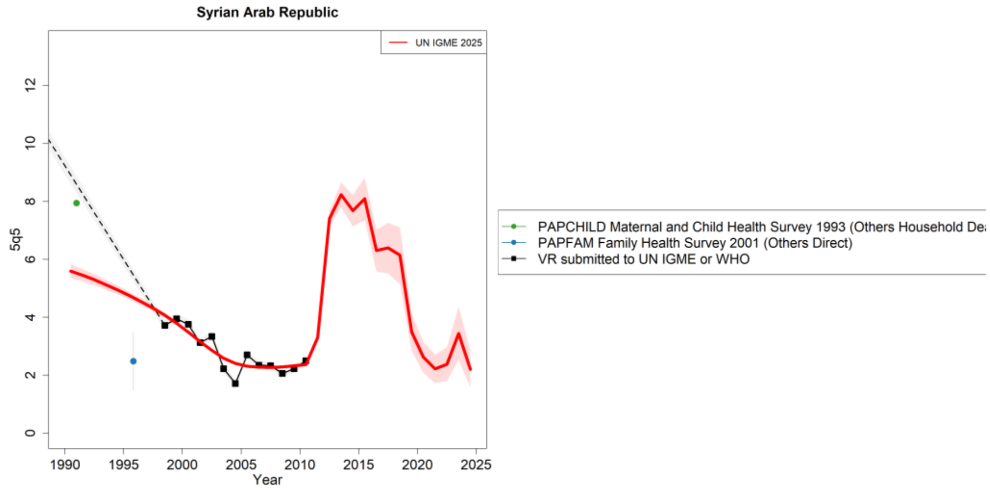

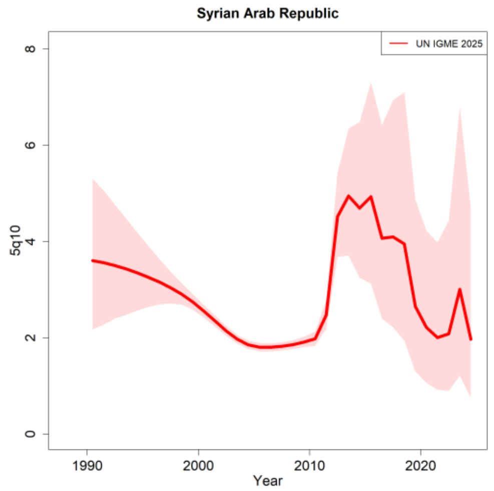

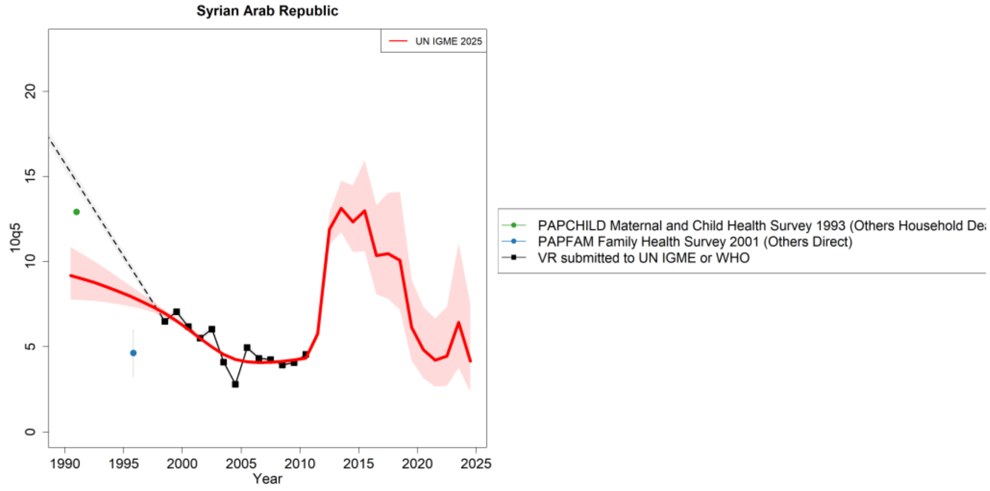

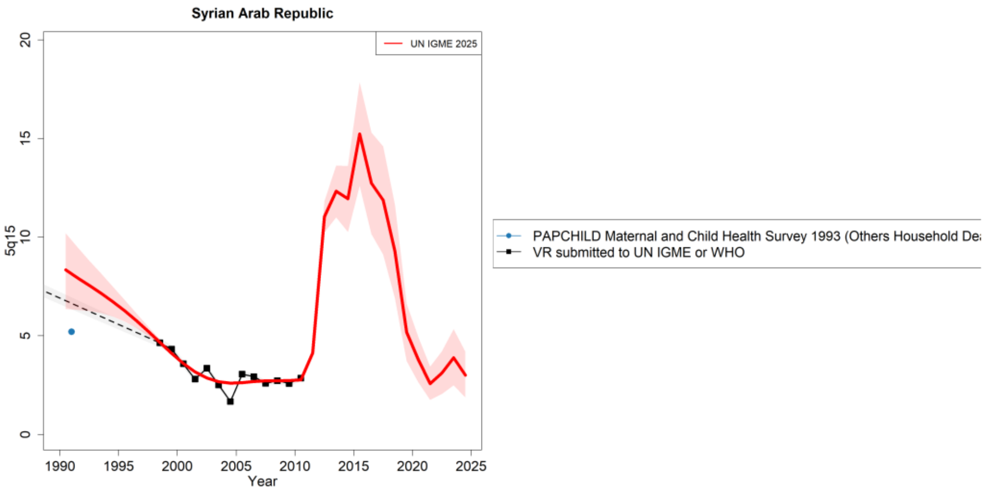
Syrian Arab Republic
UN IGME 2025
5q15
Year
PAPCHILD Maternal and Child Health Survey 1993 (Others Household De
VR submitted to UN IGME or WHO

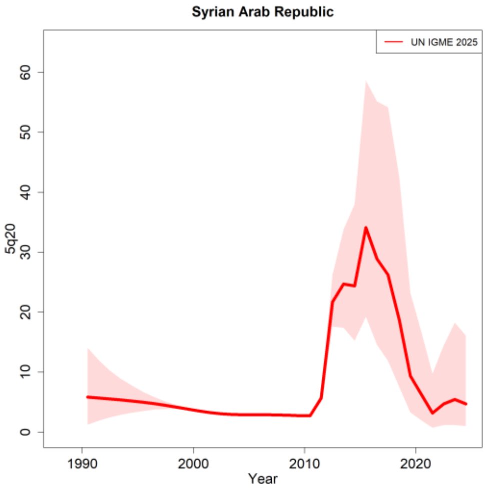
Syrian Arab Republic
UN IGME 2025
5q20
Year

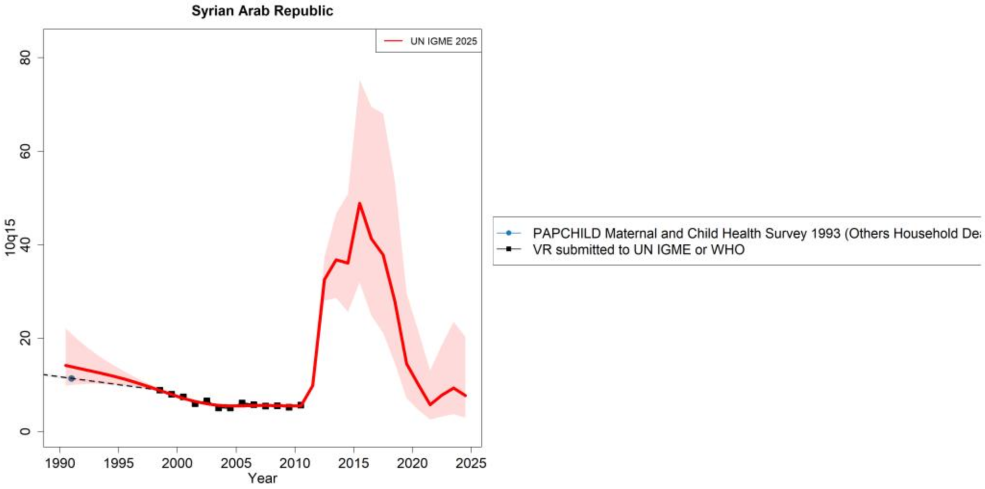
Syrian Arab Republic
UN IGME 2025
10q15
Year
PAPCHILD Maternal and Child Health Survey 1993 (Others Household De
VR submitted to UN IGME or WHO

Tajikistan (TJK)

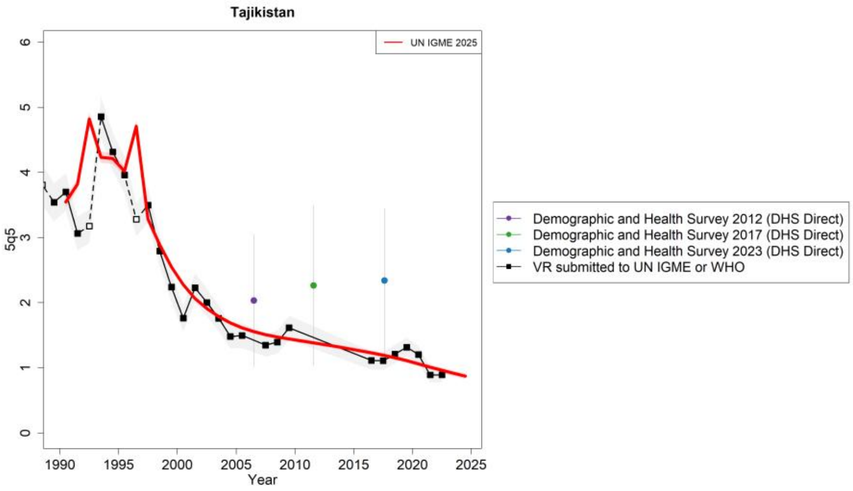

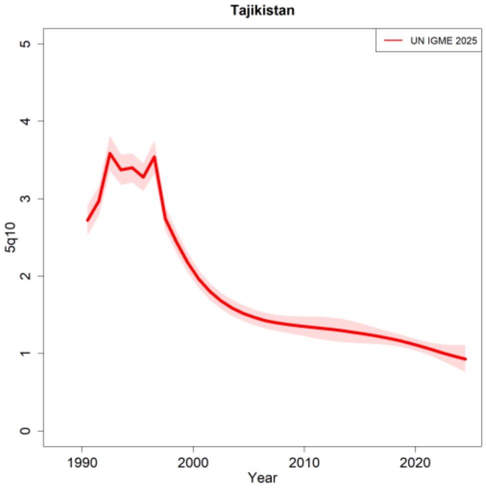

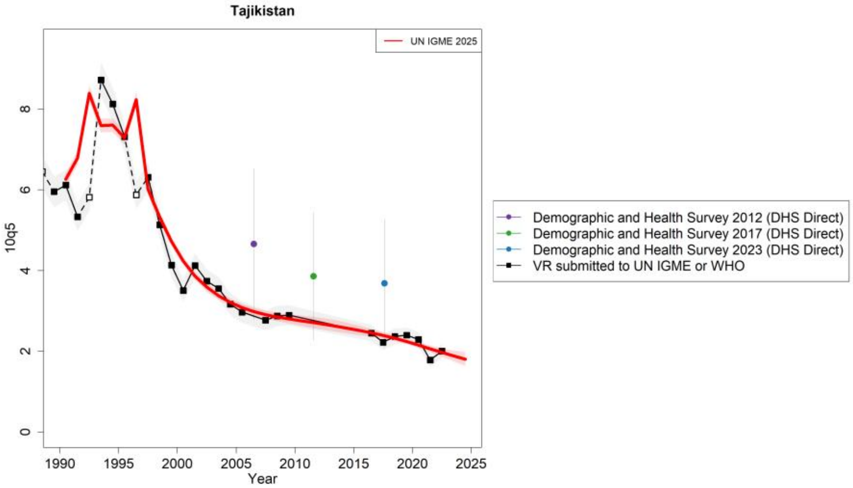

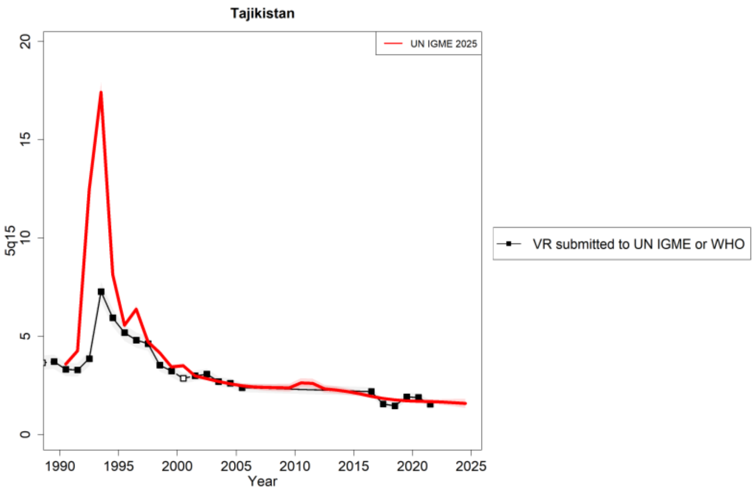
Tajikistan
UN IGME 2025
VR submitted to UN IGME or WHO
5q15
0
5
10
15
20
1990
1995
2000
2005
2010
2015
2020
2025
Year

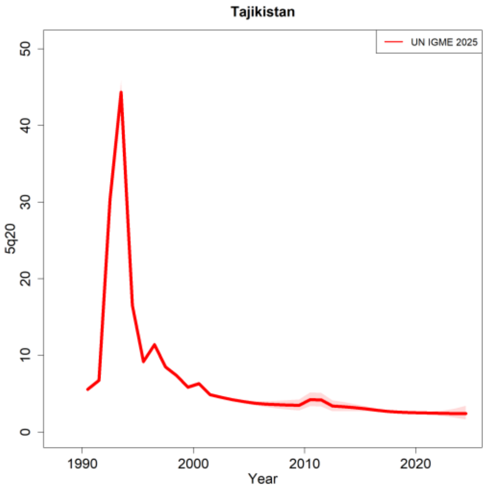
Tajikistan
UN IGME 2025
5q20
0
10
20
30
40
50
1990
2000
2010
2020
Year

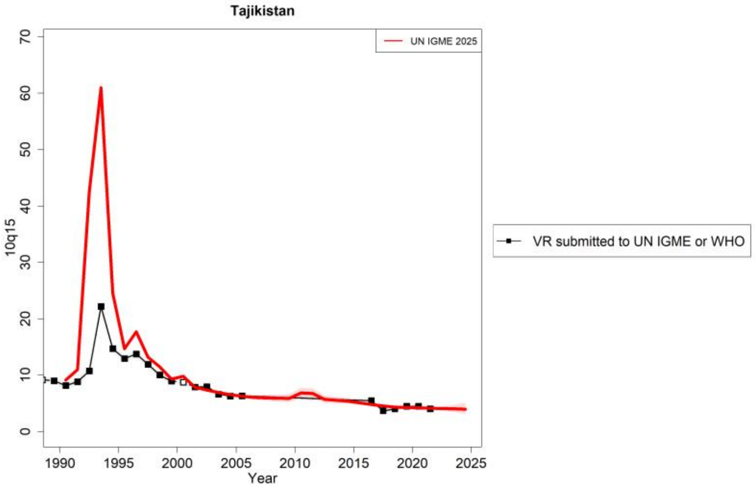
Tajikistan
UN IGME 2025
VR submitted to UN IGME or WHO
10q15
0
10
20
30
40
50
60
70
1990
1995
2000
2005
2010
2015
2020
2025
Year

Thailand (THA)

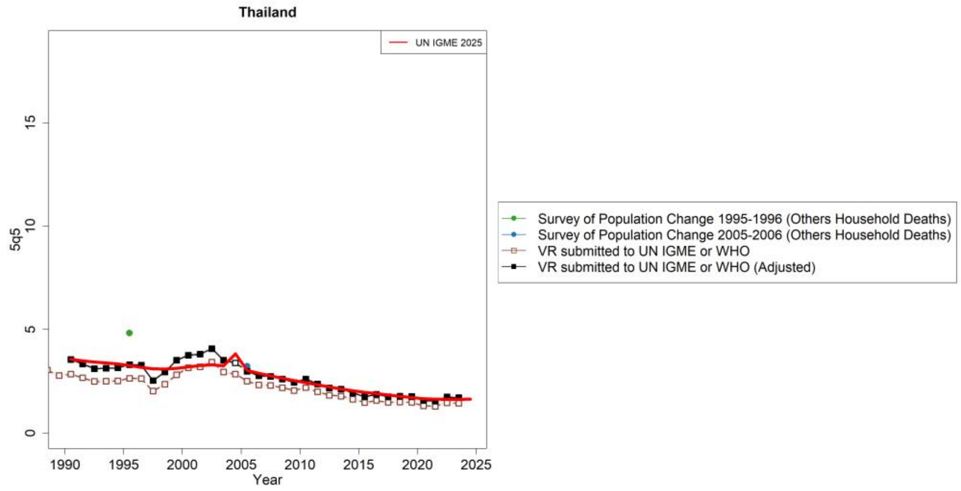

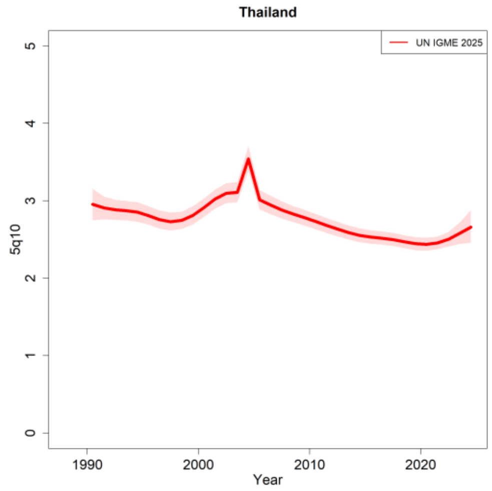

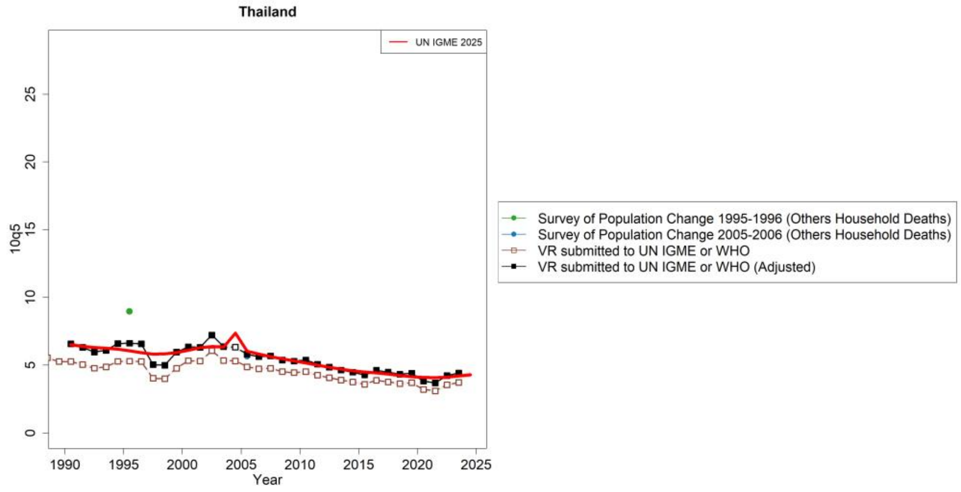

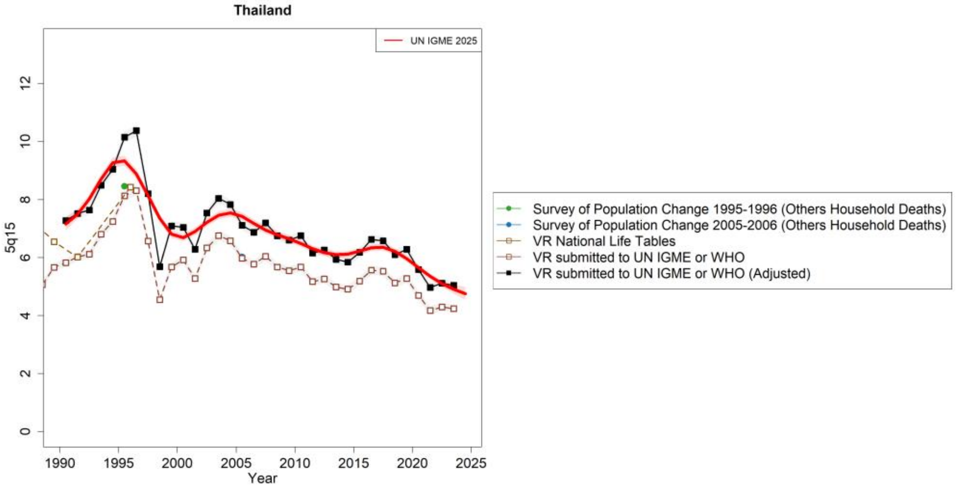
Thailand
UN IGME 2025
Survey of Population Change 1995-1996 (Others Household Deaths)
Survey of Population Change 2005-2006 (Others Household Deaths)
VR National Life Tables
VR submitted to UN IGME or WHO
VR submitted to UN IGME or WHO (Adjusted)
5q15
Year
0
2
4
6
8
10
12
1990
1995
2000
2005
2010
2015
2020
2025

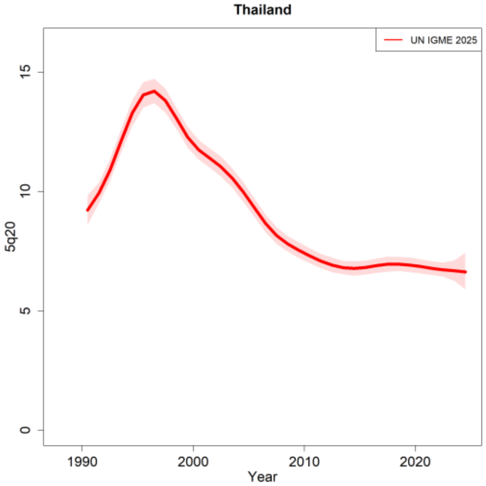
Thailand
UN IGME 2025
5q20
Year
0
5
10
15
1990
2000
2010
2020

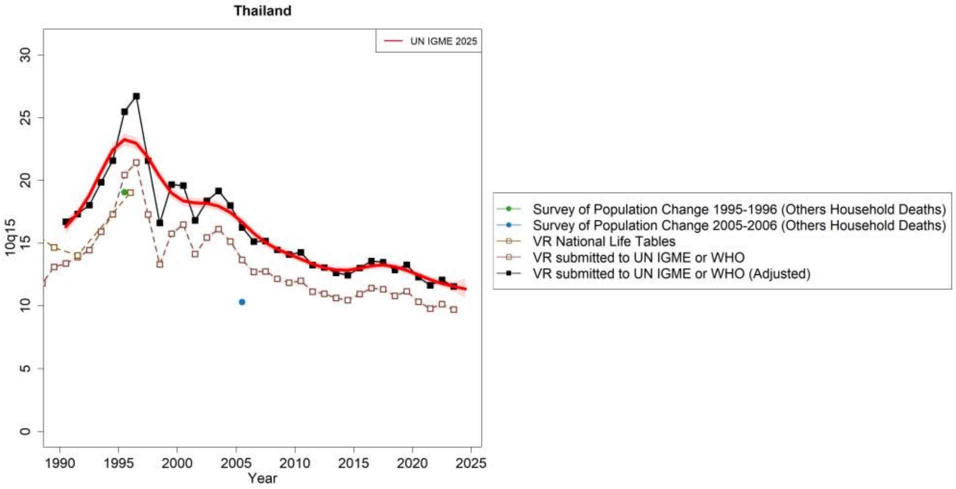
Thailand
UN IGME 2025
Survey of Population Change 1995-1996 (Others Household Deaths)
Survey of Population Change 2005-2006 (Others Household Deaths)
VR National Life Tables
VR submitted to UN IGME or WHO
VR submitted to UN IGME or WHO (Adjusted)
10q15
Year
0
5
10
15
20
25
30
1990
1995
2000
2005
2010
2015
2020
2025

Timor-Leste (TLS)

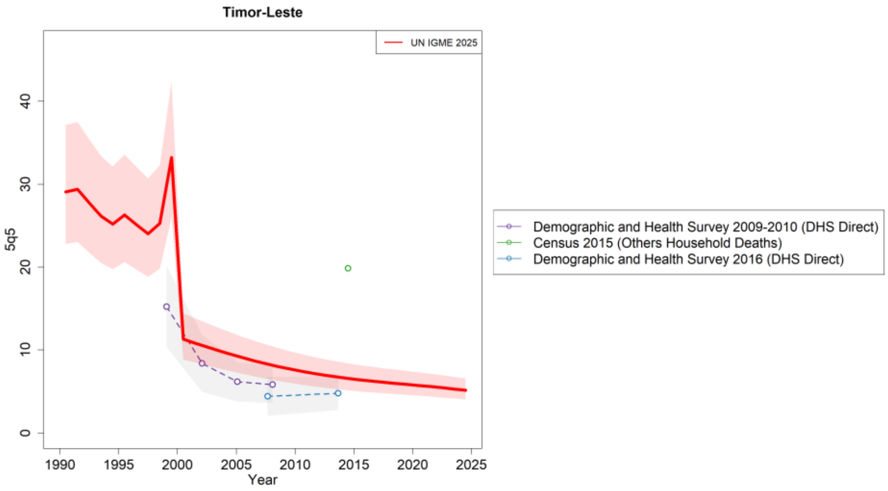

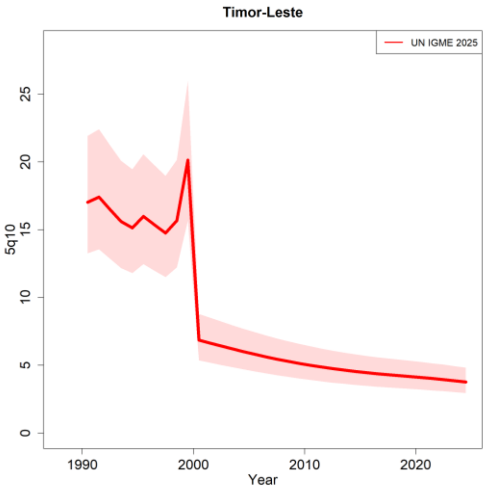

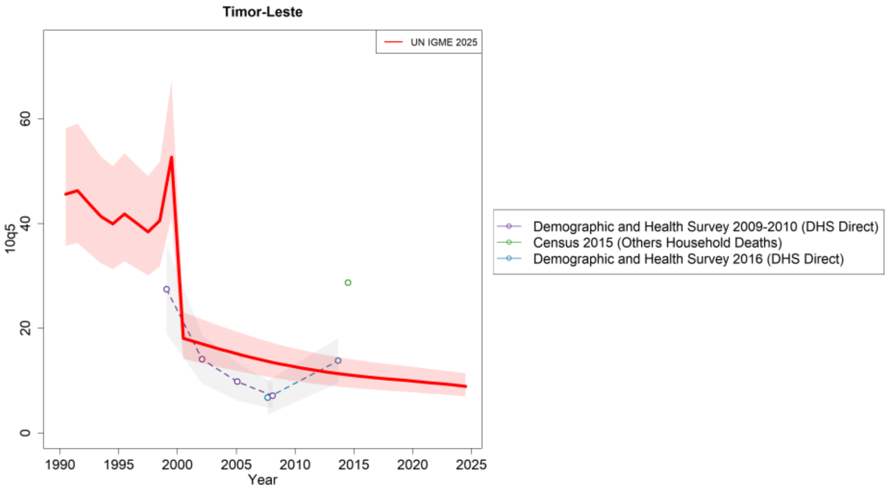

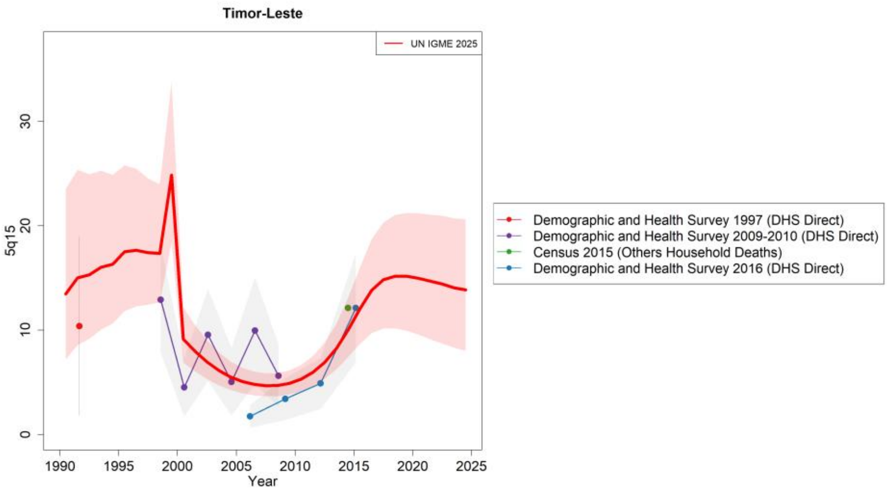
Timor-Leste
UN IGME 2025
5q15
Year
Demographic and Health Survey 1997 (DHS Direct)
Demographic and Health Survey 2009-2010 (DHS Direct)
Census 2015 (Others Household Deaths)
Demographic and Health Survey 2016 (DHS Direct)

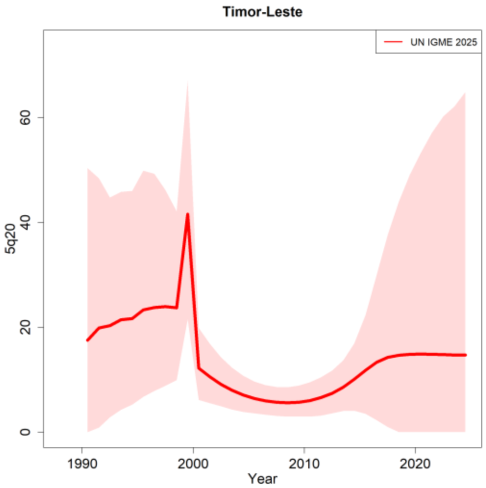
Timor-Leste
UN IGME 2025
5q20
Year

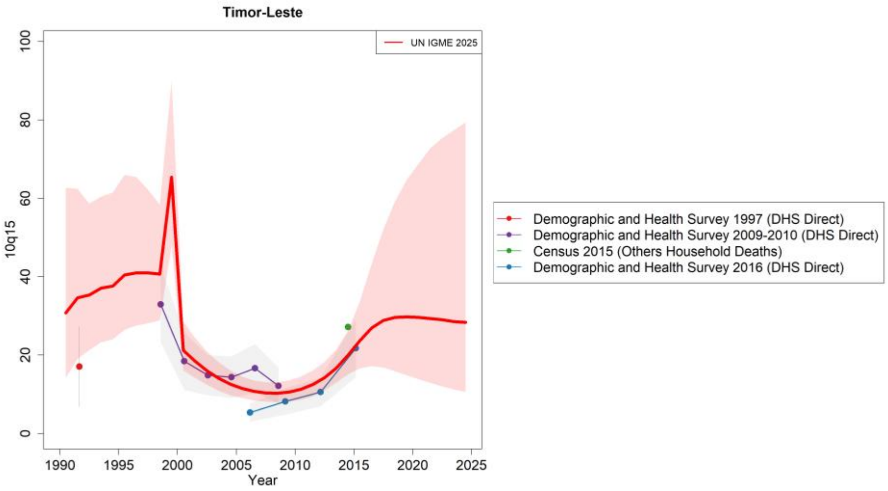
Timor-Leste
UN IGME 2025
10q15
Year
Demographic and Health Survey 1997 (DHS Direct)
Demographic and Health Survey 2009-2010 (DHS Direct)
Census 2015 (Others Household Deaths)
Demographic and Health Survey 2016 (DHS Direct)

Togo (TGO)

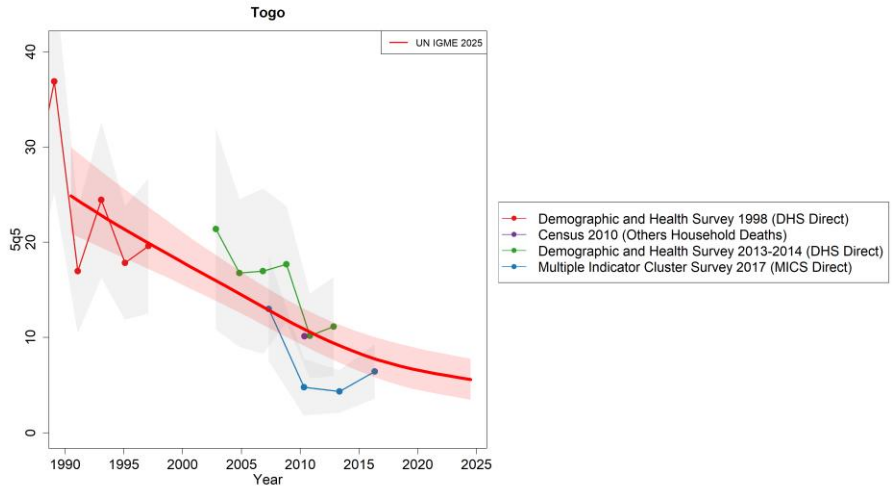

Togo
UN IGME 2025
5q5
Year
Demographic and Health Survey 1998 (DHS Direct)
Census 2010 (Others Household Deaths)
Demographic and Health Survey 2013-2014 (DHS Direct)
Multiple Indicator Cluster Survey 2017 (MICS Direct)

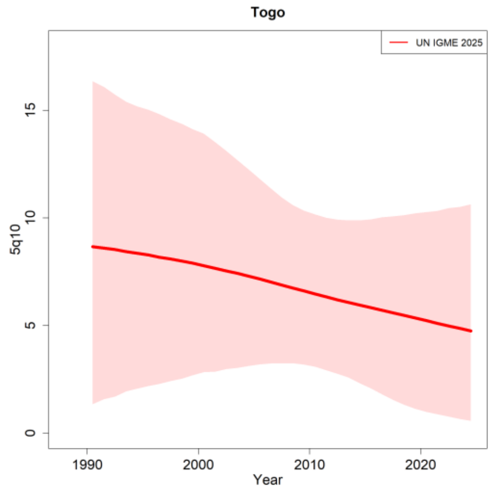

Togo
UN IGME 2025
5q10
Year

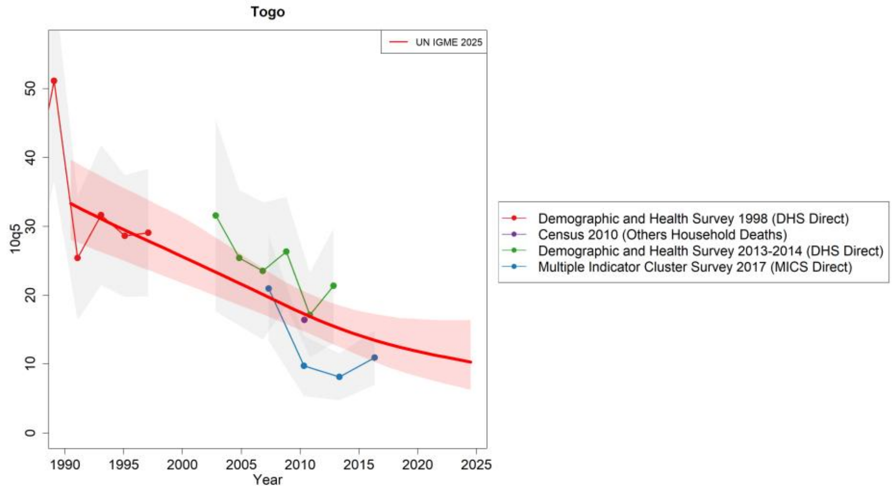

Togo
UN IGME 2025
10q5
Year
Demographic and Health Survey 1998 (DHS Direct)
Census 2010 (Others Household Deaths)
Demographic and Health Survey 2013-2014 (DHS Direct)
Multiple Indicator Cluster Survey 2017 (MICS Direct)

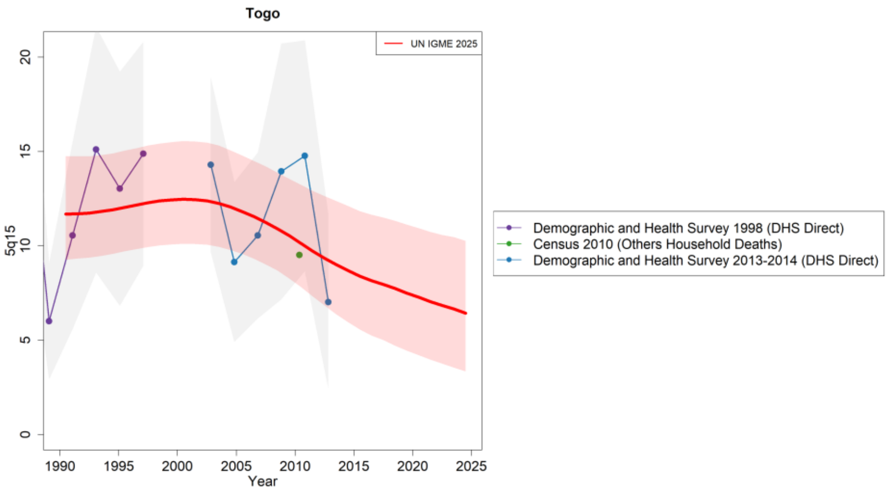
Togo
UN IGME 2025
5q15
Year
Demographic and Health Survey 1998 (DHS Direct)
Census 2010 (Others Household Deaths)
Demographic and Health Survey 2013-2014 (DHS Direct)

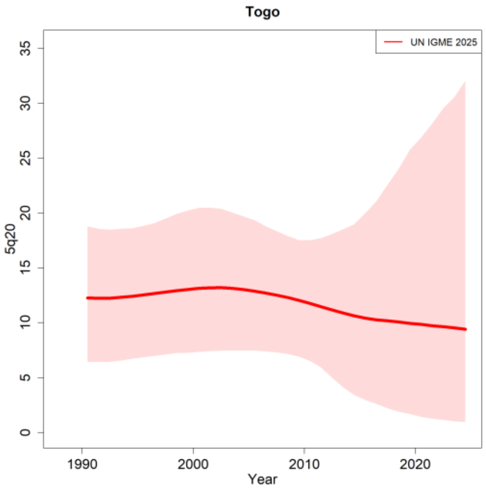
Togo
UN IGME 2025
5q20
Year

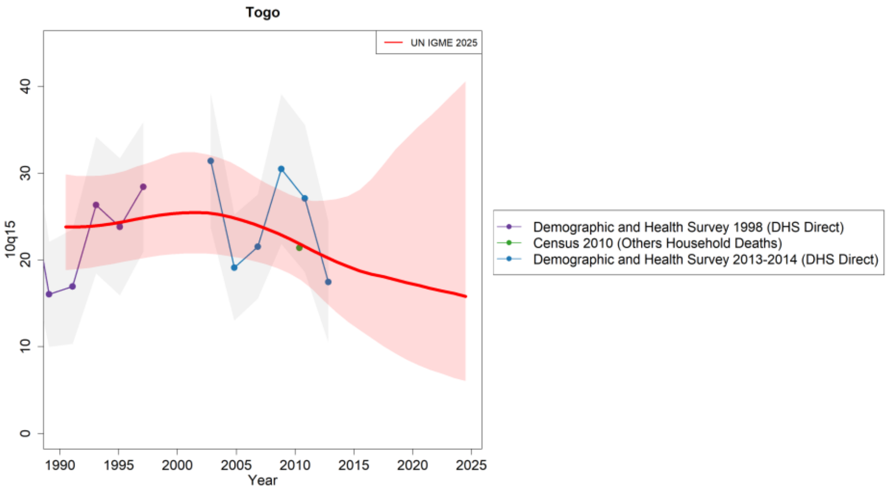
Togo
UN IGME 2025
10q15
Year
Demographic and Health Survey 1998 (DHS Direct)
Census 2010 (Others Household Deaths)
Demographic and Health Survey 2013-2014 (DHS Direct)

Tonga (TON)

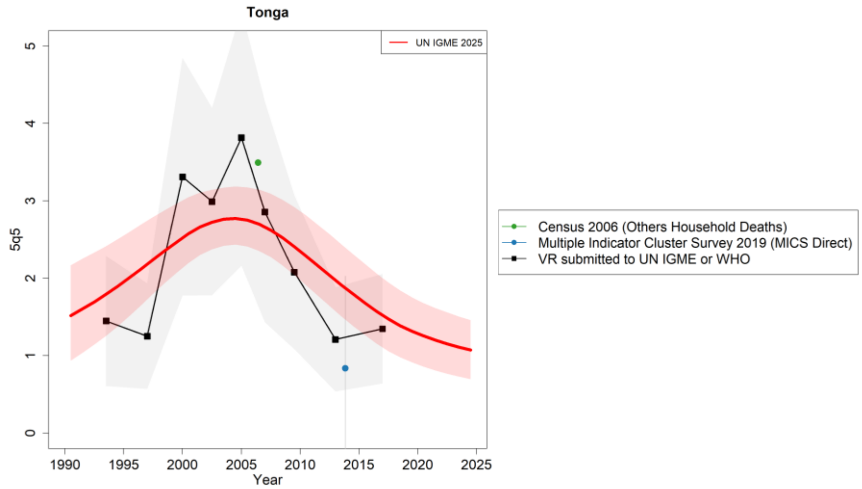
Tonga
UN IGME 2025
5q5
Year
Census 2006 (Others Household Deaths)
Multiple Indicator Cluster Survey 2019 (MICS Direct)
VR submitted to UN IGME or WHO

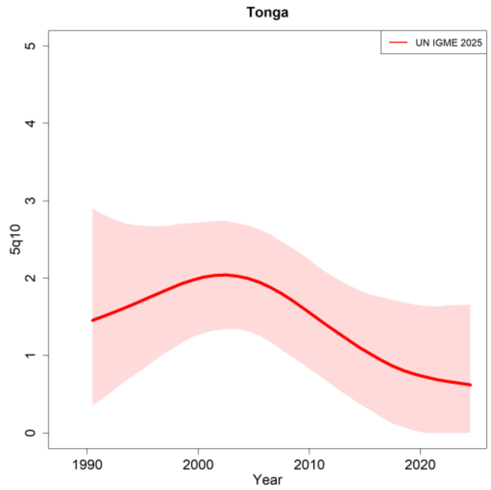
Tonga
UN IGME 2025
5q10
Year

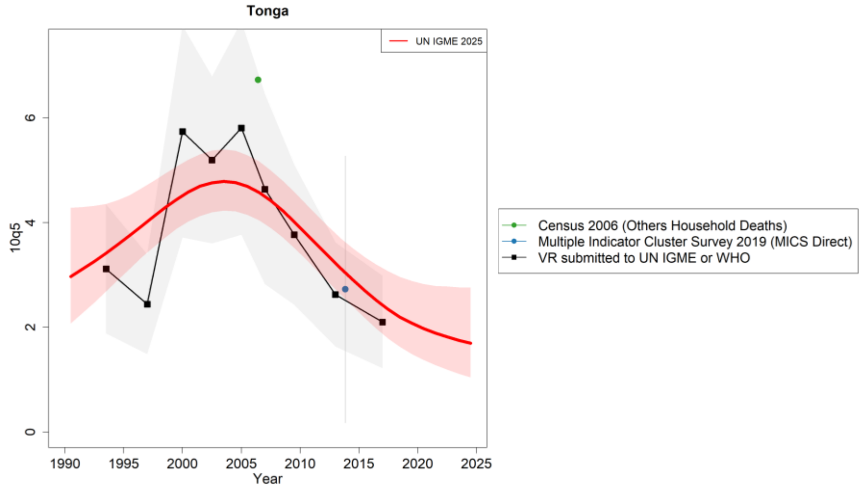
Tonga
UN IGME 2025
10q5
Year
Census 2006 (Others Household Deaths)
Multiple Indicator Cluster Survey 2019 (MICS Direct)
VR submitted to UN IGME or WHO

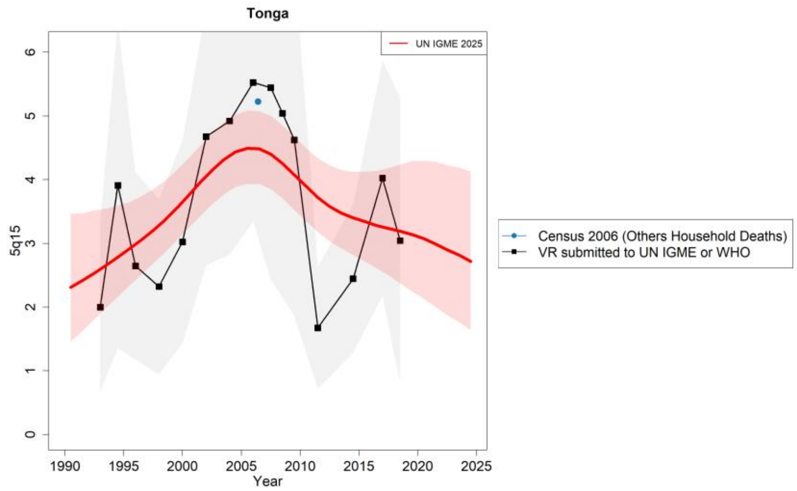
Tonga
UN IGME 2025
Census 2006 (Others Household Deaths)
VR submitted to UN IGME or WHO
5q15
Year

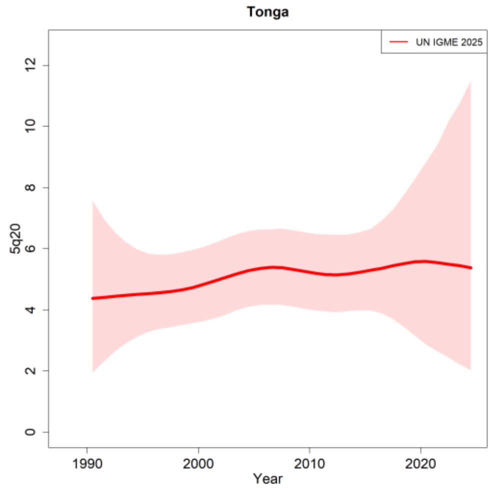
Tonga
UN IGME 2025
5q20
Year

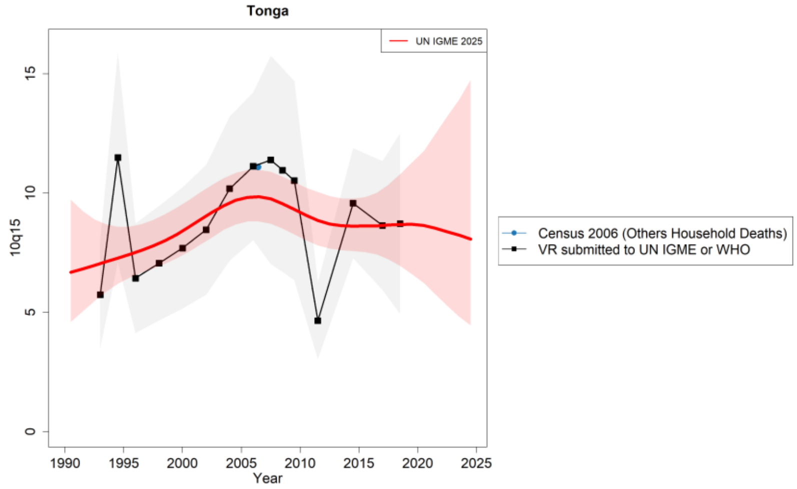
Tonga
UN IGME 2025
Census 2006 (Others Household Deaths)
VR submitted to UN IGME or WHO
10q15
Year

Trinidad and Tobago (TTO)

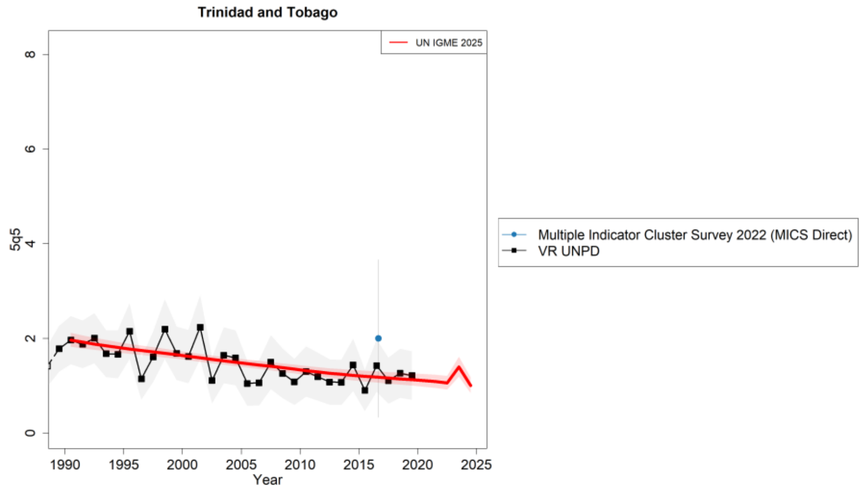

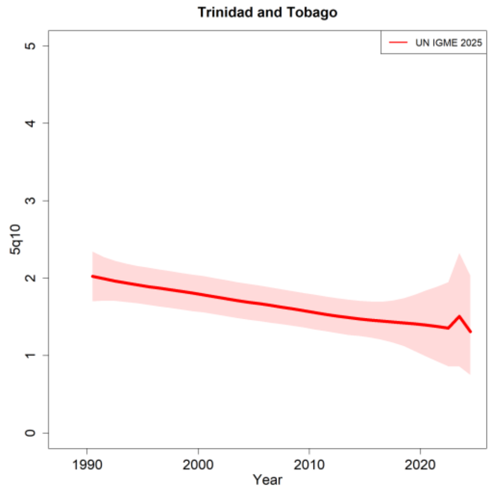

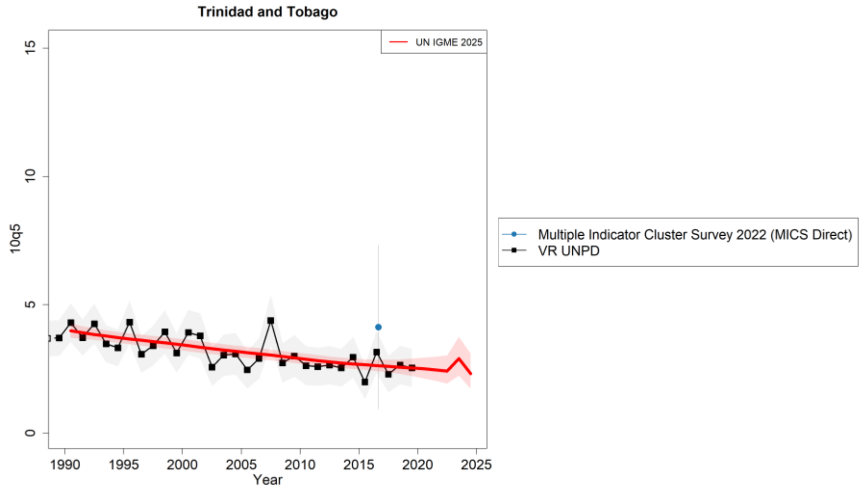

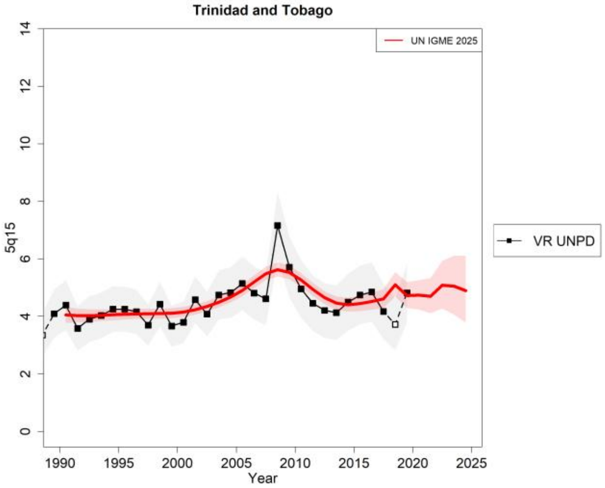
Trinidad and Tobago
UN IGME 2025
VR UNPD
5q15
Year
0
2
4
6
8
10
12
14
1990
1995
2000
2005
2010
2015
2020
2025

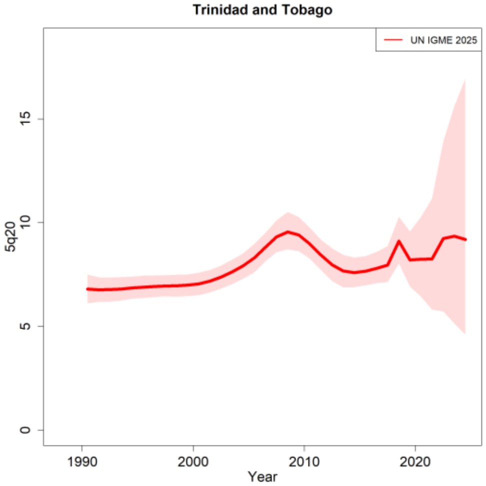
Trinidad and Tobago
UN IGME 2025
5q20
Year
0
5
10
15
1990
2000
2010
2020

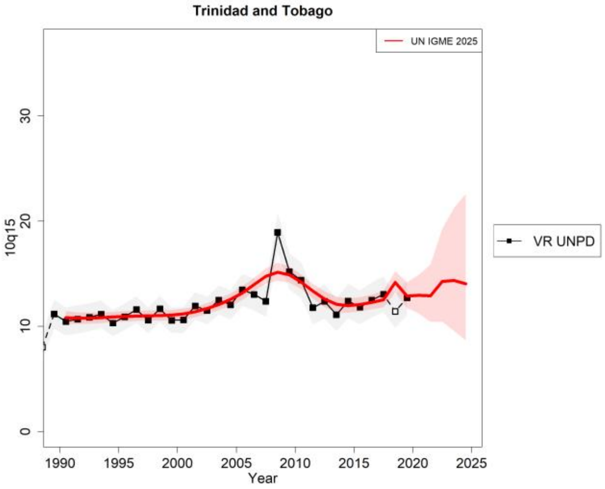
Trinidad and Tobago
UN IGME 2025
VR UNPD
10q15
Year
0
10
20
30
1990
1995
2000
2005
2010
2015
2020
2025

Tunisia (TUN)

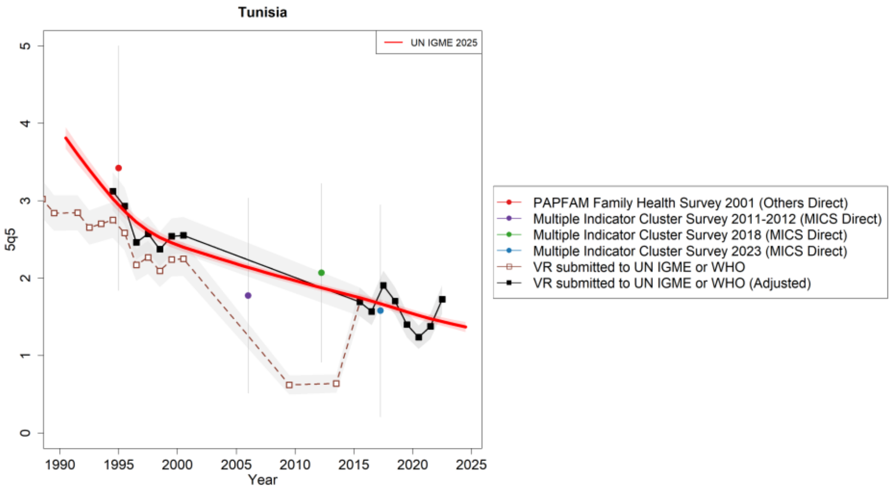
Tunisia
UN IGME 2025
5q5
Year
PAPFAM Family Health Survey 2001 (Others Direct)
Multiple Indicator Cluster Survey 2011-2012 (MICS Direct)
Multiple Indicator Cluster Survey 2018 (MICS Direct)
Multiple Indicator Cluster Survey 2023 (MICS Direct)
VR submitted to UN IGME or WHO
VR submitted to UN IGME or WHO (Adjusted)

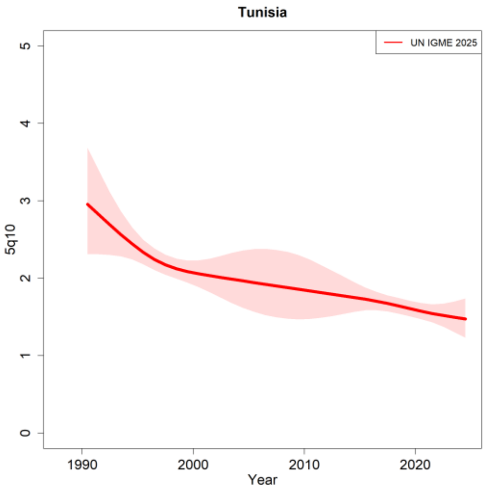
Tunisia
UN IGME 2025
5q10
Year

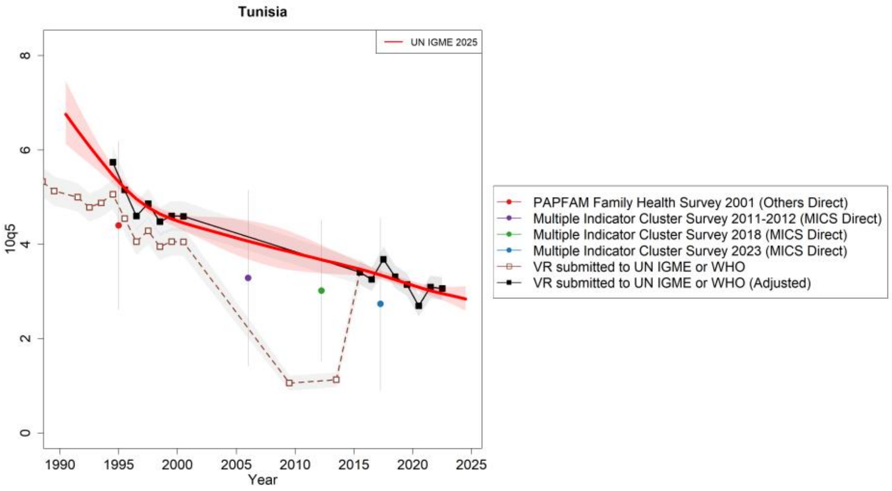
Tunisia
UN IGME 2025
10q5
Year
PAPFAM Family Health Survey 2001 (Others Direct)
Multiple Indicator Cluster Survey 2011-2012 (MICS Direct)
Multiple Indicator Cluster Survey 2018 (MICS Direct)
Multiple Indicator Cluster Survey 2023 (MICS Direct)
VR submitted to UN IGME or WHO
VR submitted to UN IGME or WHO (Adjusted)

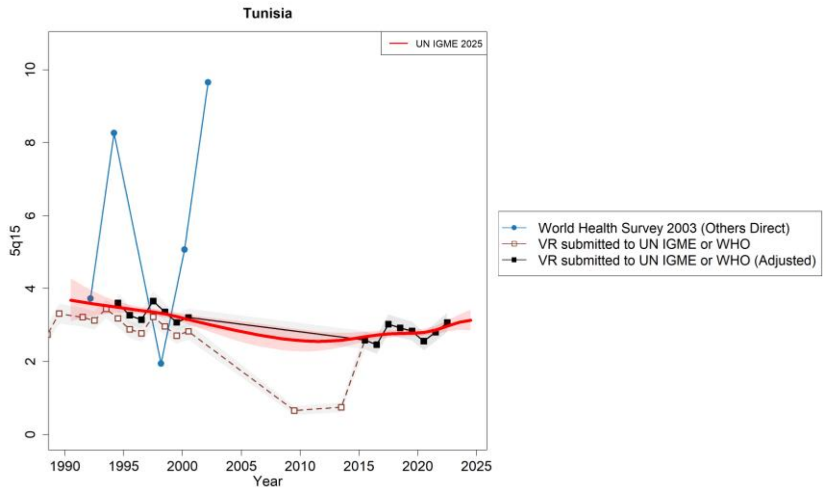
Tunisia
UN IGME 2025
World Health Survey 2003 (Others Direct)
VR submitted to UN IGME or WHO
VR submitted to UN IGME or WHO (Adjusted)
5q15
Year

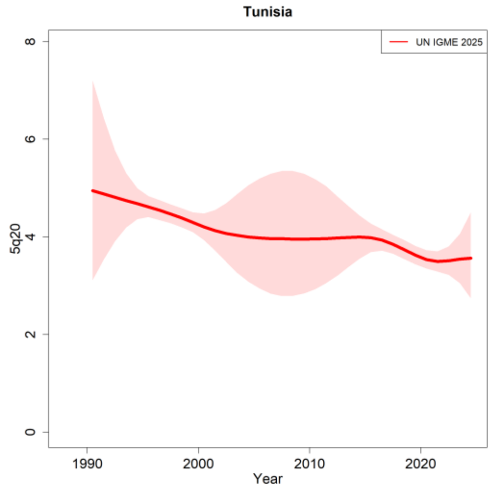
Tunisia
UN IGME 2025
5q20
Year

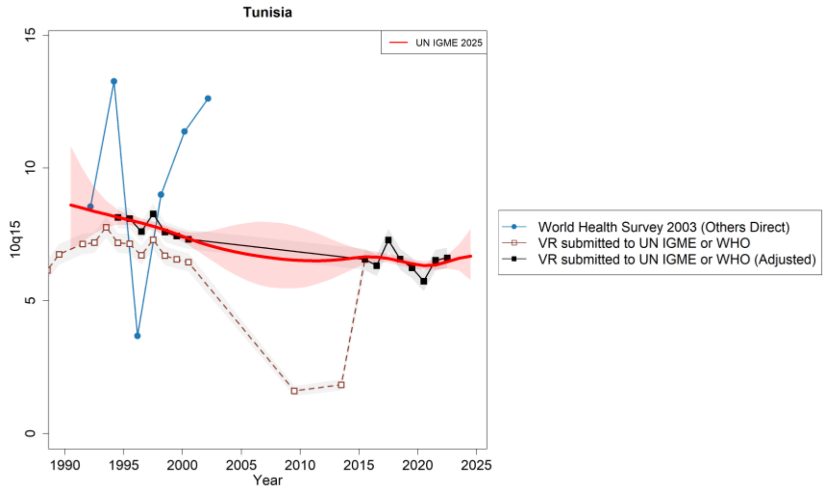
Tunisia
UN IGME 2025
World Health Survey 2003 (Others Direct)
VR submitted to UN IGME or WHO
VR submitted to UN IGME or WHO (Adjusted)
10q15
Year

Turkmenistan (TKM)

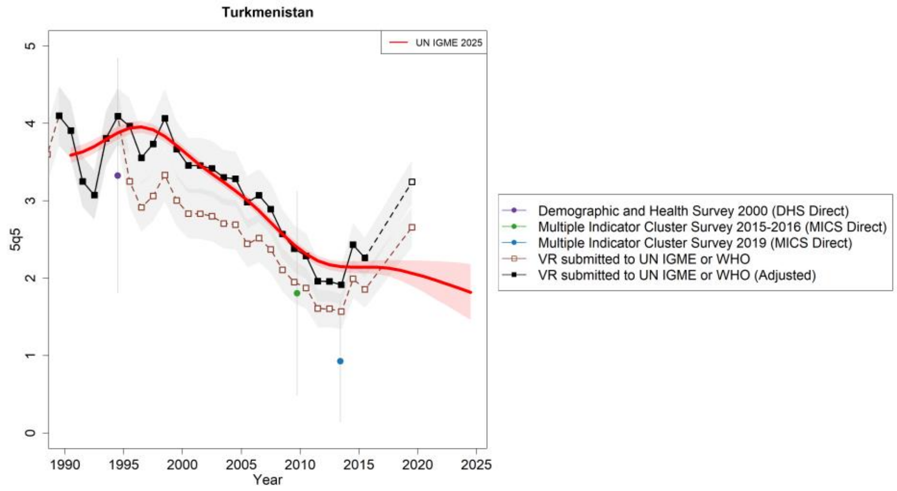

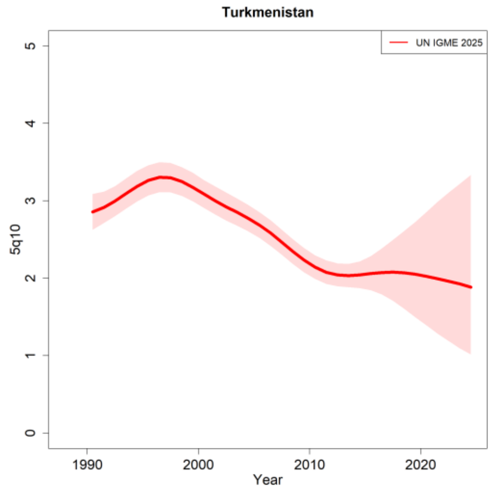

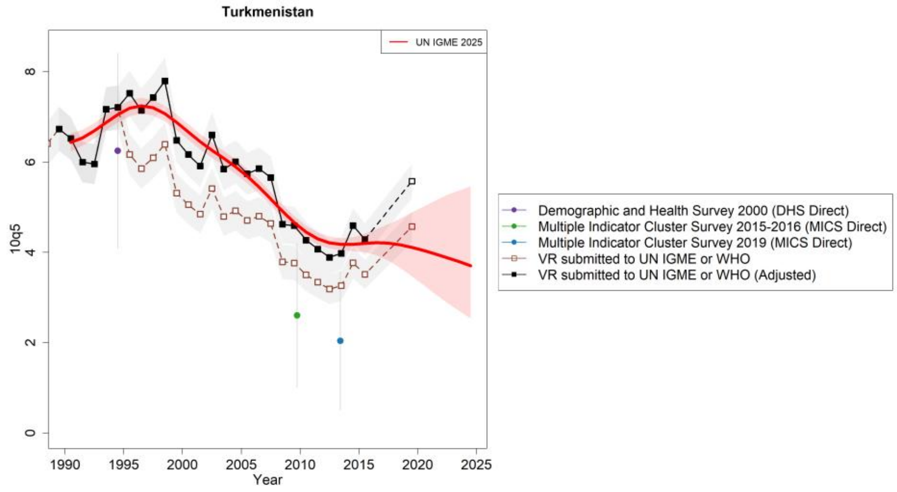

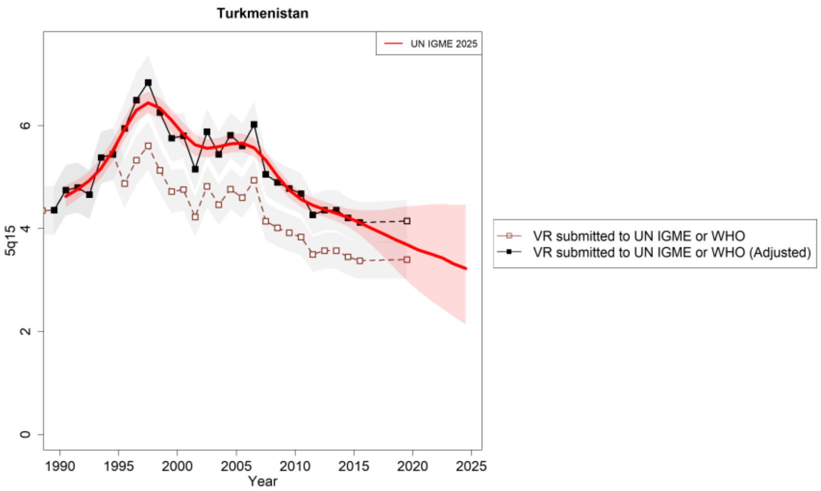
Turkmenistan
UN IGME 2025
VR submitted to UN IGME or WHO
VR submitted to UN IGME or WHO (Adjusted)
5q15
Year
0
2
4
6
1990
1995
2000
2005
2010
2015
2020
2025

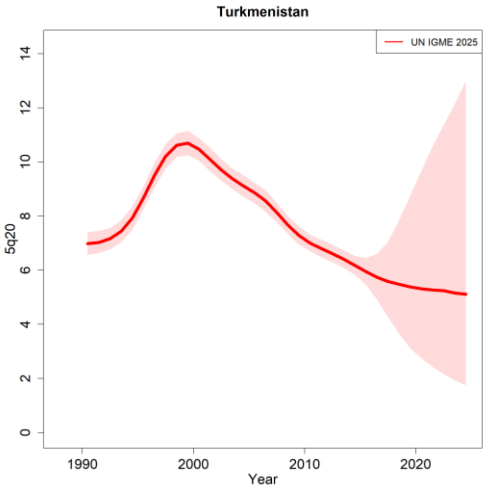
Turkmenistan
UN IGME 2025
5q20
Year
0
2
4
6
8
10
12
14
1990
2000
2010
2020

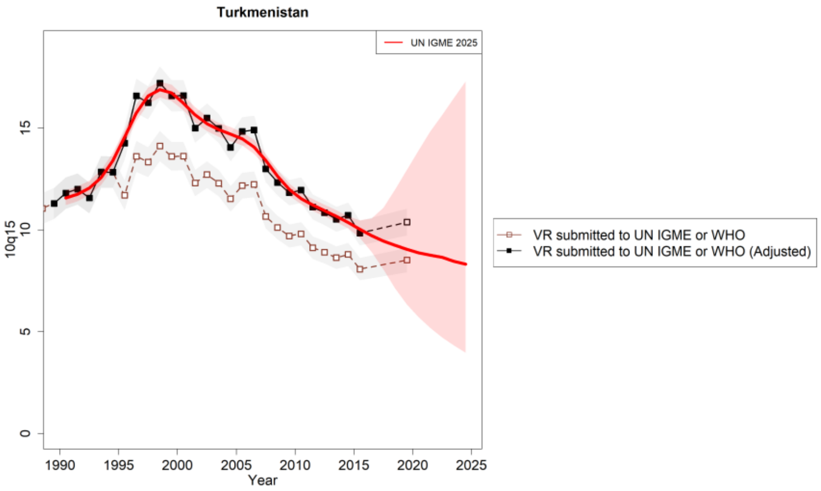
Turkmenistan
UN IGME 2025
VR submitted to UN IGME or WHO
VR submitted to UN IGME or WHO (Adjusted)
10q15
Year
0
5
10
15
1990
1995
2000
2005
2010
2015
2020
2025

Turks and Caicos Islands (TCA)

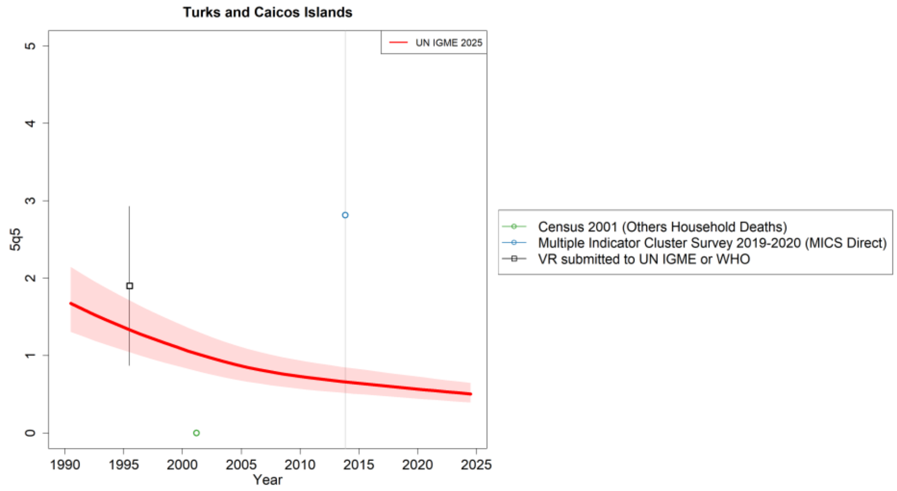

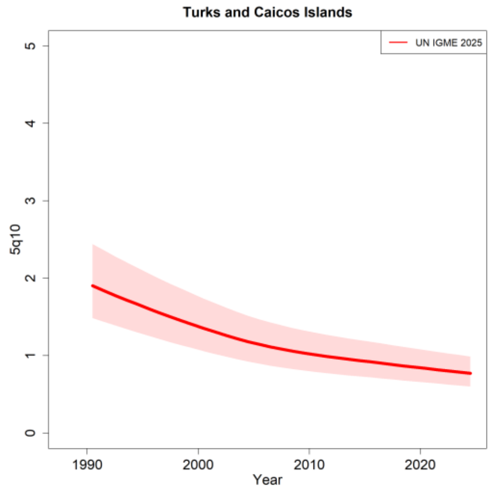

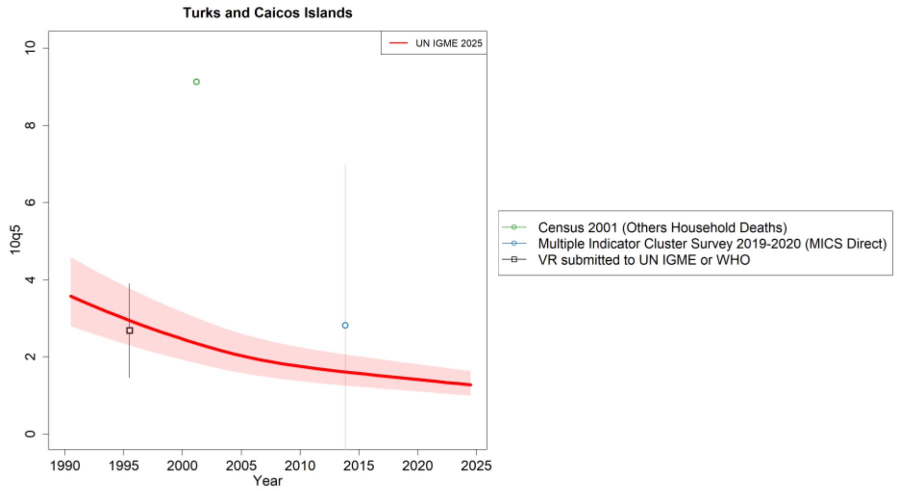

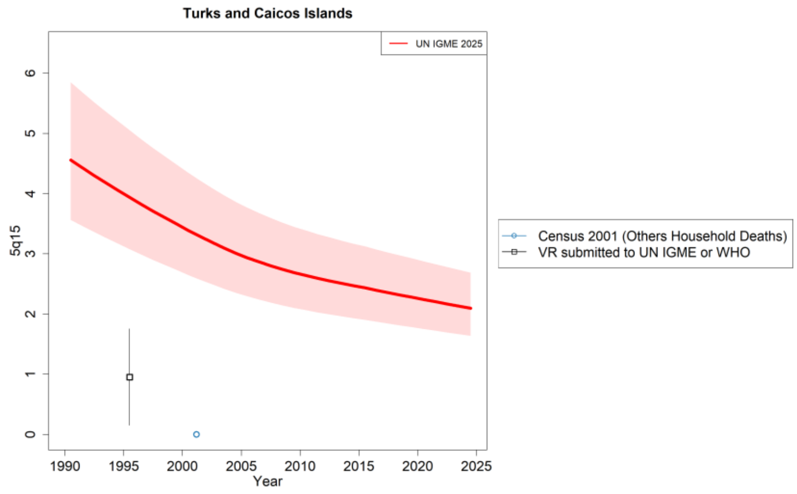
Turks and Caicos Islands
UN IGME 2025
5q15
Year
Census 2001 (Others Household Deaths)
VR submitted to UN IGME or WHO

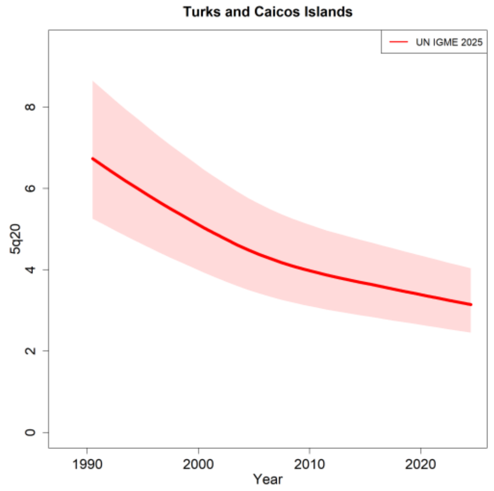
Turks and Caicos Islands
UN IGME 2025
5q20
Year

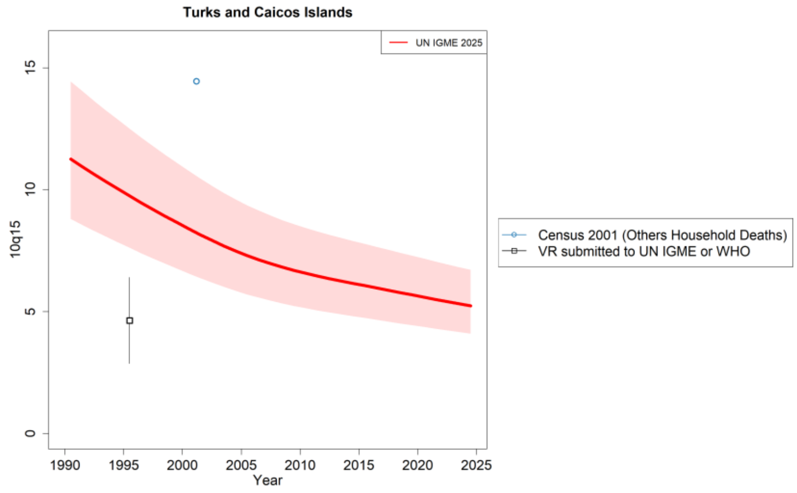
Turks and Caicos Islands
UN IGME 2025
10q15
Year
Census 2001 (Others Household Deaths)
VR submitted to UN IGME or WHO

Tuvalu (TUV)

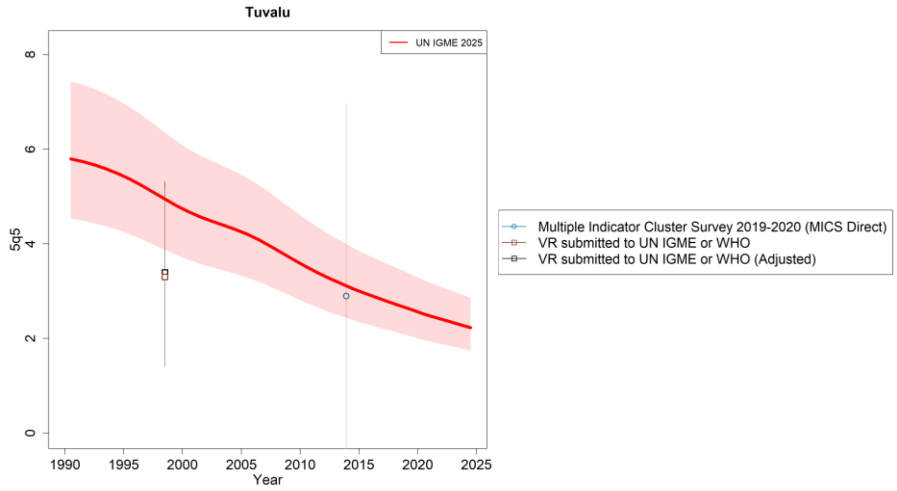
Tuvalu
UN IGME 2025
5q5
Year
Multiple Indicator Cluster Survey 2019-2020 (MICS Direct)
VR submitted to UN IGME or WHO
VR submitted to UN IGME or WHO (Adjusted)

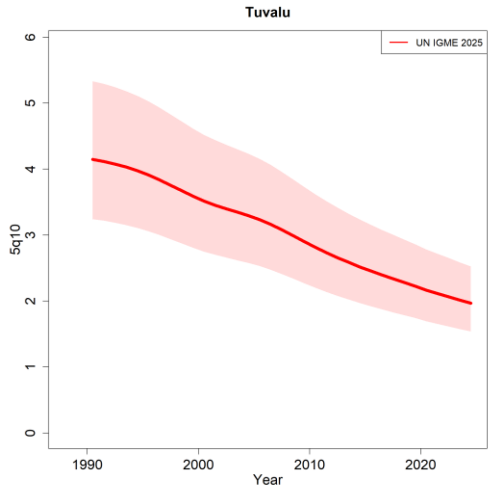
Tuvalu
UN IGME 2025
5q10
Year

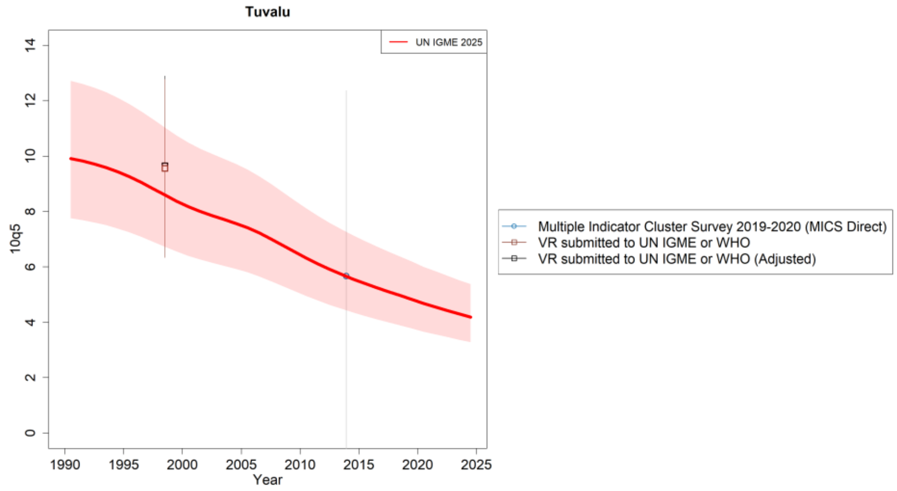
Tuvalu
UN IGME 2025
10q5
Year
Multiple Indicator Cluster Survey 2019-2020 (MICS Direct)
VR submitted to UN IGME or WHO
VR submitted to UN IGME or WHO (Adjusted)

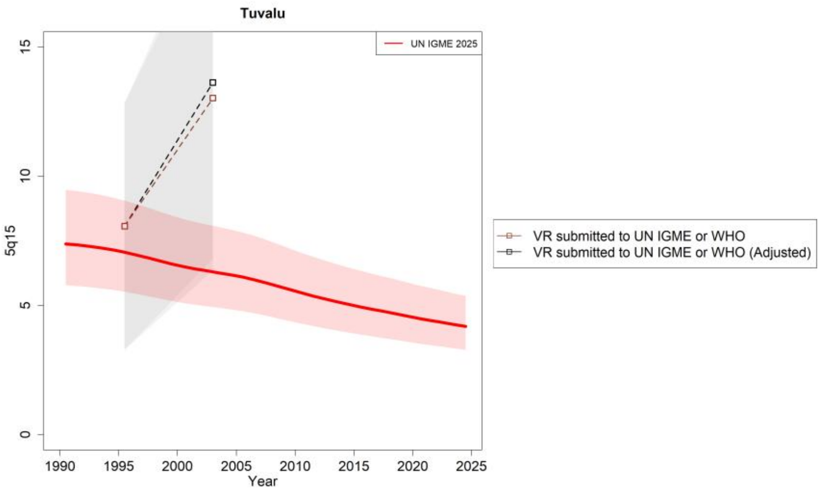
Tuvalu
UN IGME 2025
VR submitted to UN IGME or WHO
VR submitted to UN IGME or WHO (Adjusted)
5q15
Year

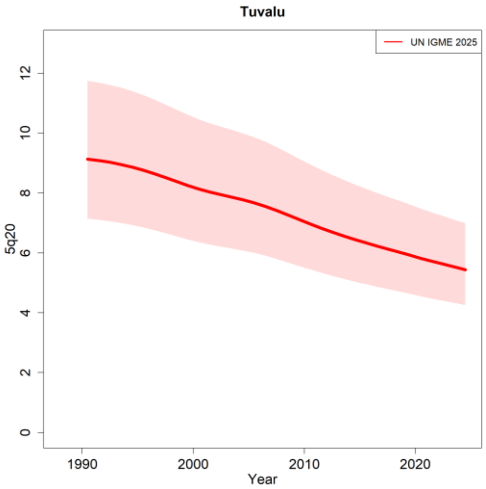
Tuvalu
UN IGME 2025
5q20
Year

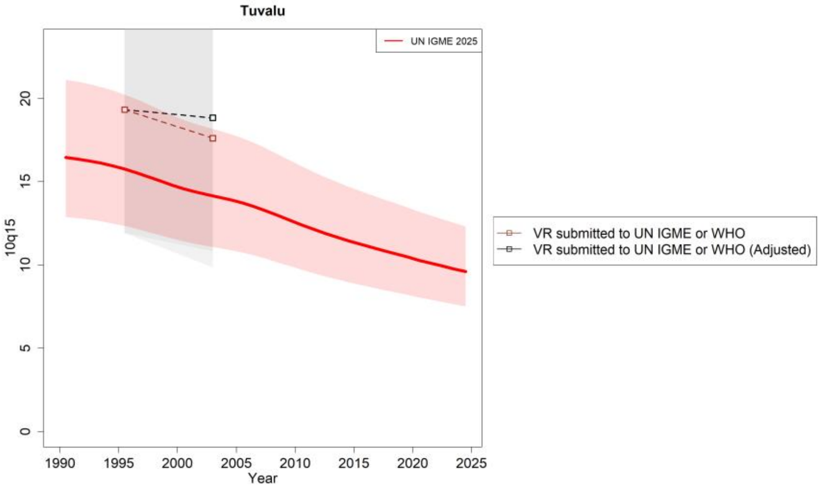
Tuvalu
UN IGME 2025
VR submitted to UN IGME or WHO
VR submitted to UN IGME or WHO (Adjusted)
10q15
Year

Türkiye (TUR)

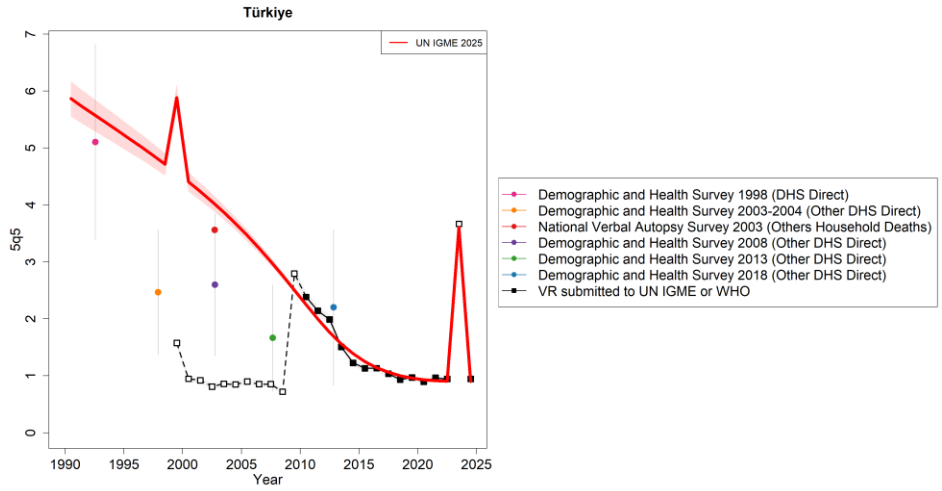

Türkiye
UN IGME 2025
5q5
Year
Demographic and Health Survey 1998 (DHS Direct)
Demographic and Health Survey 2003-2004 (Other DHS Direct)
National Verbal Autopsy Survey 2003 (Others Household Deaths)
Demographic and Health Survey 2008 (Other DHS Direct)
Demographic and Health Survey 2013 (Other DHS Direct)
Demographic and Health Survey 2018 (Other DHS Direct)
VR submitted to UN IGME or WHO

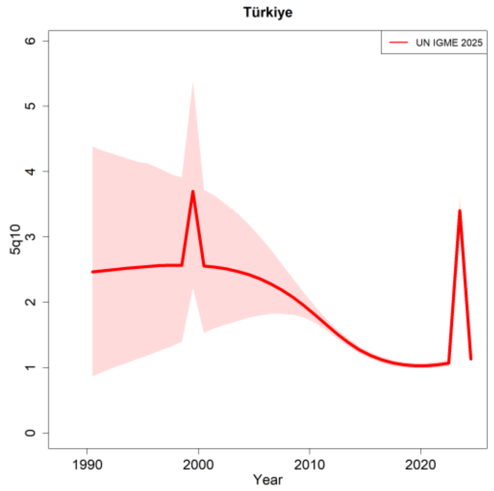

Türkiye
UN IGME 2025
5q10
Year

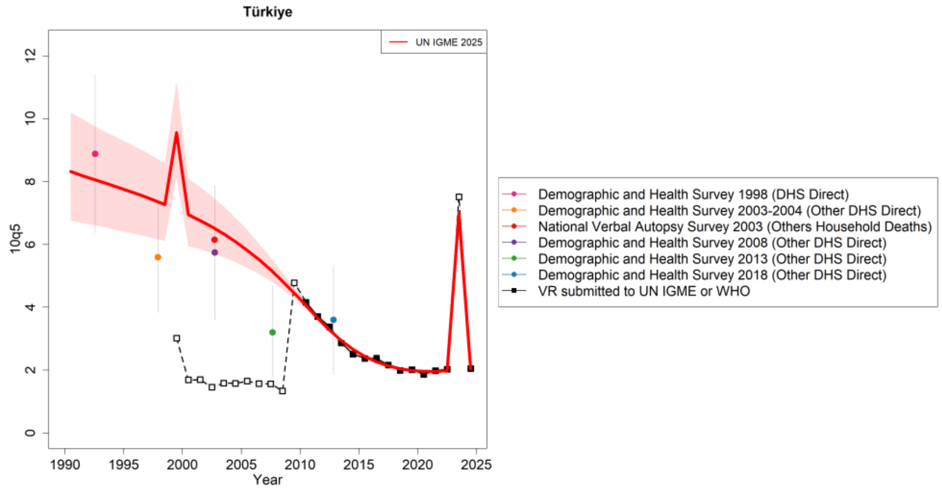

Türkiye
UN IGME 2025
10q5
Year
Demographic and Health Survey 1998 (DHS Direct)
Demographic and Health Survey 2003-2004 (Other DHS Direct)
National Verbal Autopsy Survey 2003 (Others Household Deaths)
Demographic and Health Survey 2008 (Other DHS Direct)
Demographic and Health Survey 2013 (Other DHS Direct)
Demographic and Health Survey 2018 (Other DHS Direct)
VR submitted to UN IGME or WHO

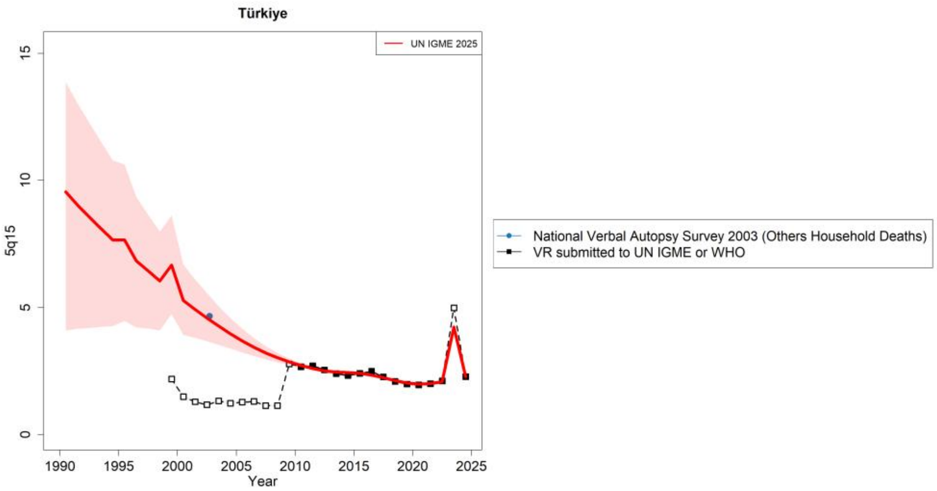
Türkiye
UN IGME 2025
5q15
Year
National Verbal Autopsy Survey 2003 (Others Household Deaths)
VR submitted to UN IGME or WHO

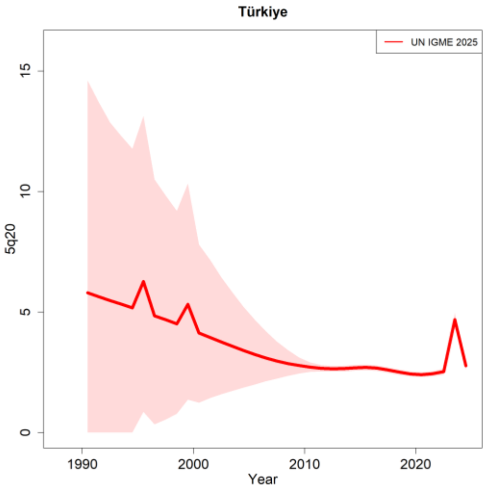
Türkiye
UN IGME 2025
5q20
Year

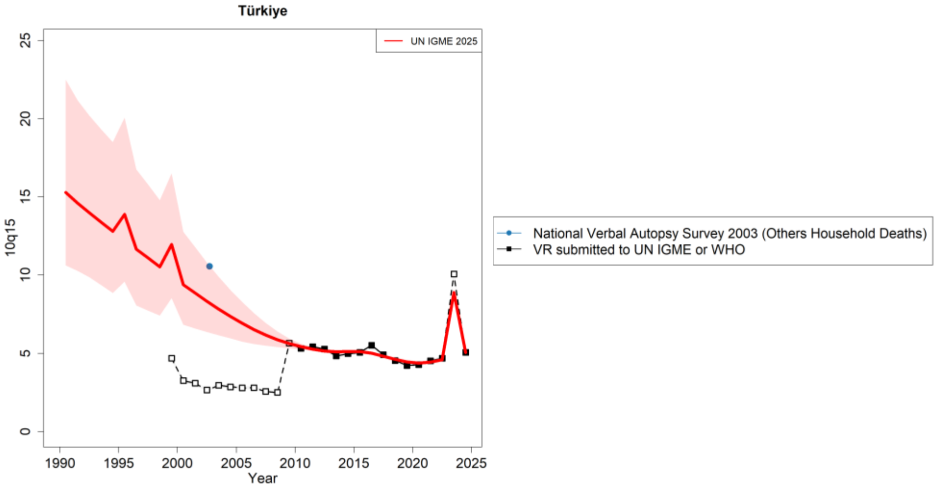
Türkiye
UN IGME 2025
10q15
Year
National Verbal Autopsy Survey 2003 (Others Household Deaths)
VR submitted to UN IGME or WHO

Uganda (UGA)

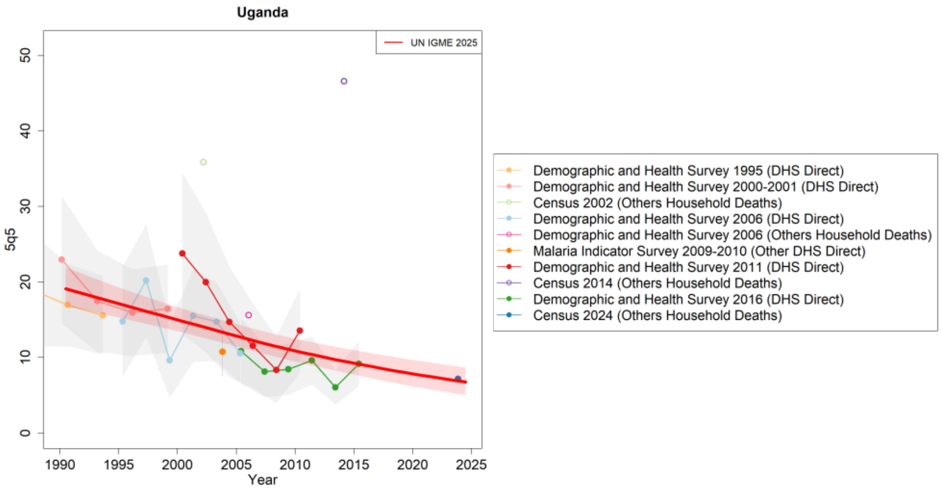
Uganda
UN IGME 2025
5q5
Year
Demographic and Health Survey 1995 (DHS Direct)
Demographic and Health Survey 2000-2001 (DHS Direct)
Census 2002 (Others Household Deaths)
Demographic and Health Survey 2006 (DHS Direct)
Demographic and Health Survey 2006 (Others Household Deaths)
Malaria Indicator Survey 2009-2010 (Other DHS Direct)
Demographic and Health Survey 2011 (DHS Direct)
Census 2014 (Others Household Deaths)
Demographic and Health Survey 2016 (DHS Direct)
Census 2024 (Others Household Deaths)

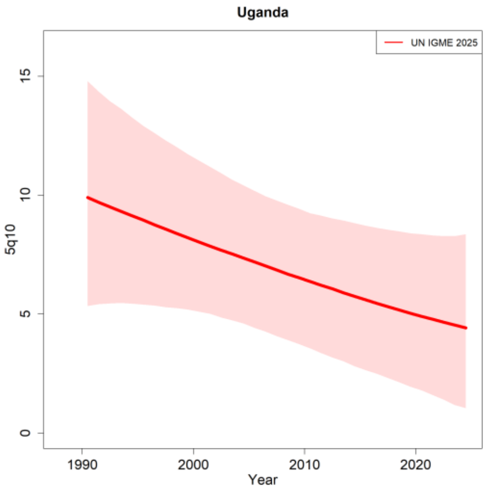
Uganda
UN IGME 2025
5q10
Year

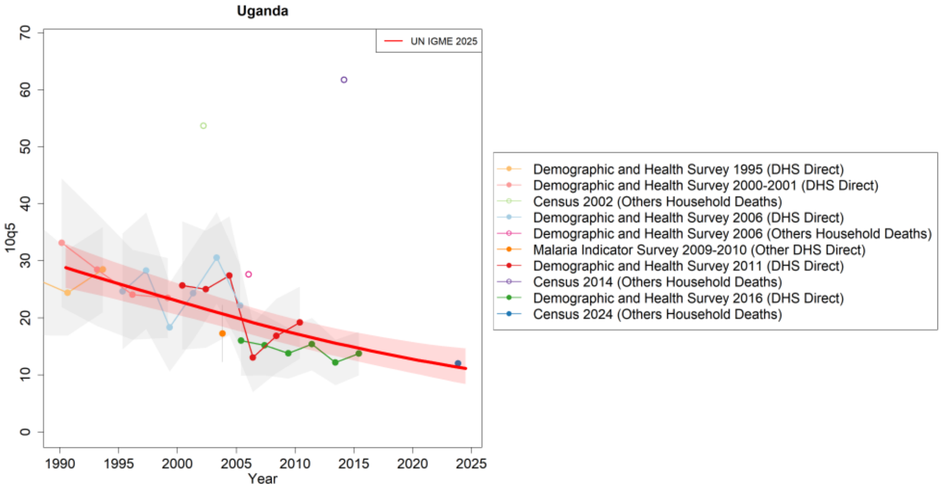
Uganda
UN IGME 2025
10q5
Year
Demographic and Health Survey 1995 (DHS Direct)
Demographic and Health Survey 2000-2001 (DHS Direct)
Census 2002 (Others Household Deaths)
Demographic and Health Survey 2006 (DHS Direct)
Demographic and Health Survey 2006 (Others Household Deaths)
Malaria Indicator Survey 2009-2010 (Other DHS Direct)
Demographic and Health Survey 2011 (DHS Direct)
Census 2014 (Others Household Deaths)
Demographic and Health Survey 2016 (DHS Direct)
Census 2024 (Others Household Deaths)

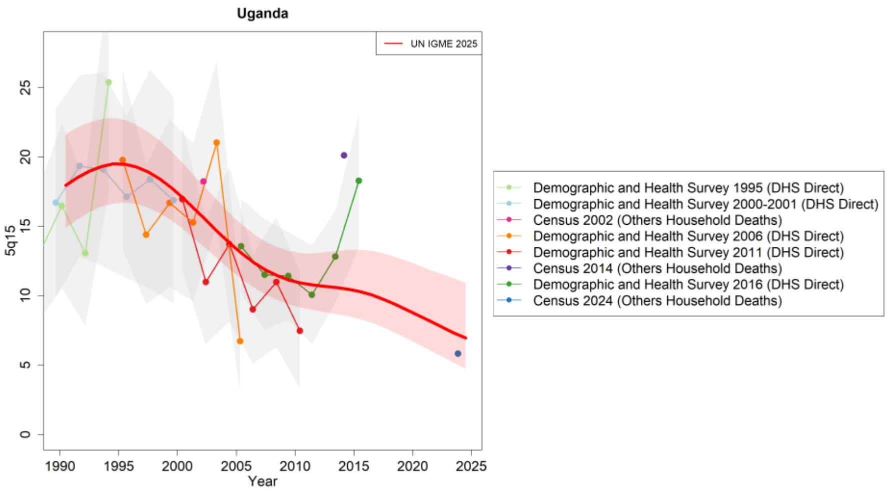
Uganda
UN IGME 2025
5q15
Year
Demographic and Health Survey 1995 (DHS Direct)
Demographic and Health Survey 2000-2001 (DHS Direct)
Census 2002 (Others Household Deaths)
Demographic and Health Survey 2006 (DHS Direct)
Demographic and Health Survey 2011 (DHS Direct)
Census 2014 (Others Household Deaths)
Demographic and Health Survey 2016 (DHS Direct)
Census 2024 (Others Household Deaths)

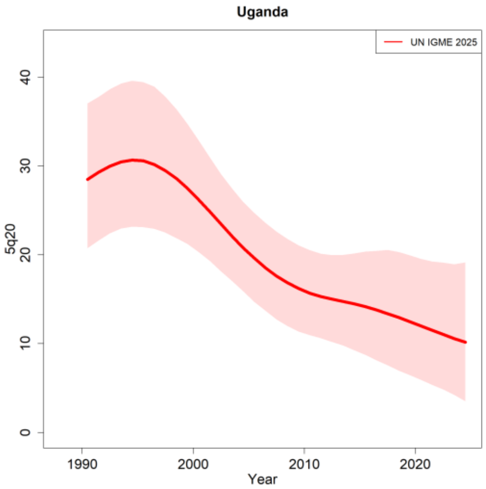
Uganda
UN IGME 2025
5q20
Year

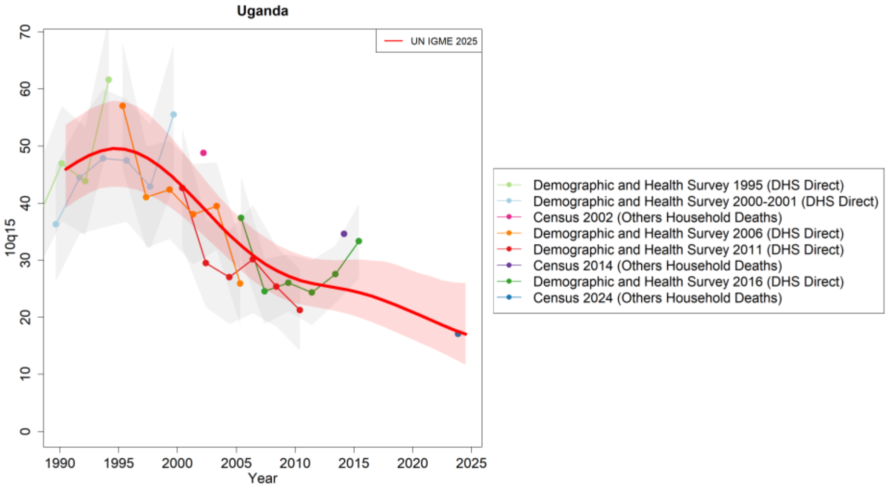
Uganda
UN IGME 2025
10q15
Year
Demographic and Health Survey 1995 (DHS Direct)
Demographic and Health Survey 2000-2001 (DHS Direct)
Census 2002 (Others Household Deaths)
Demographic and Health Survey 2006 (DHS Direct)
Demographic and Health Survey 2011 (DHS Direct)
Census 2014 (Others Household Deaths)
Demographic and Health Survey 2016 (DHS Direct)
Census 2024 (Others Household Deaths)

Ukraine (UKR)

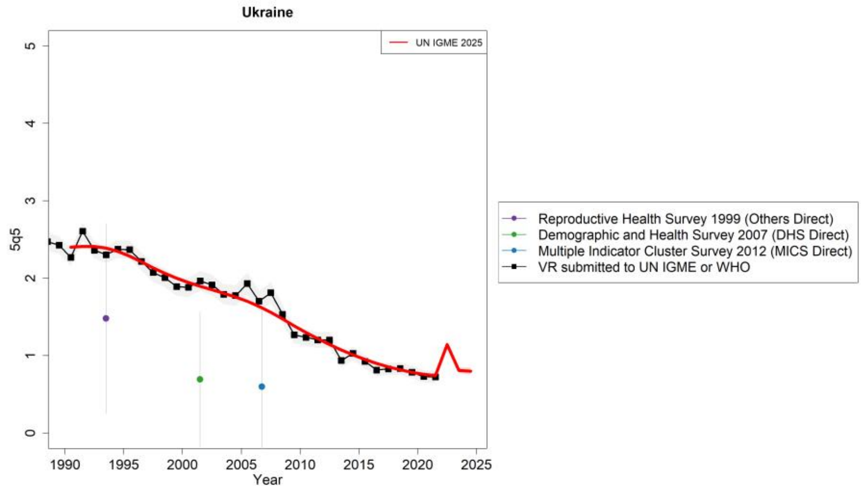
Ukraine
UN IGME 2025
5q5
Year
Reproductive Health Survey 1999 (Others Direct)
Demographic and Health Survey 2007 (DHS Direct)
Multiple Indicator Cluster Survey 2012 (MICS Direct)
VR submitted to UN IGME or WHO

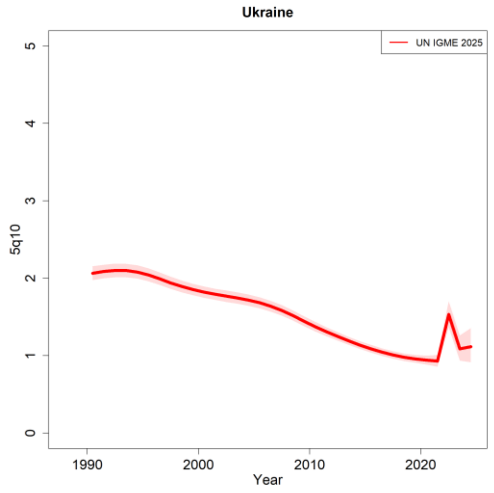
Ukraine
UN IGME 2025
5q10
Year

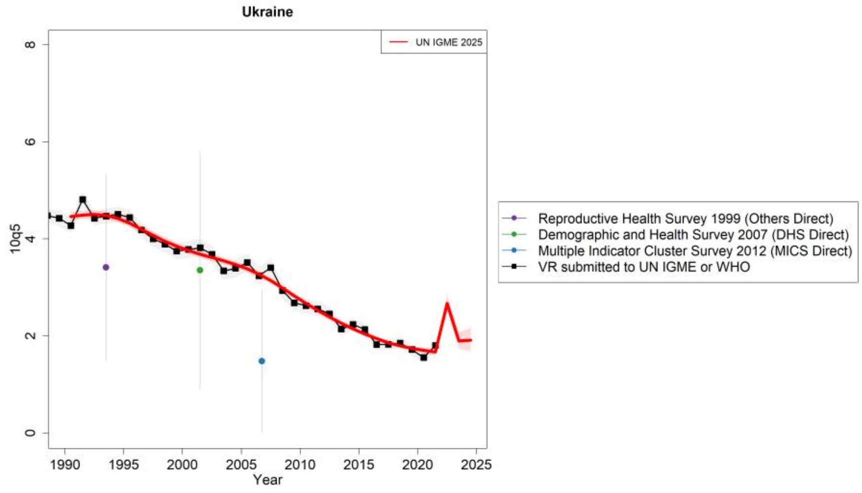
Ukraine
UN IGME 2025
10q5
Year
Reproductive Health Survey 1999 (Others Direct)
Demographic and Health Survey 2007 (DHS Direct)
Multiple Indicator Cluster Survey 2012 (MICS Direct)
VR submitted to UN IGME or WHO

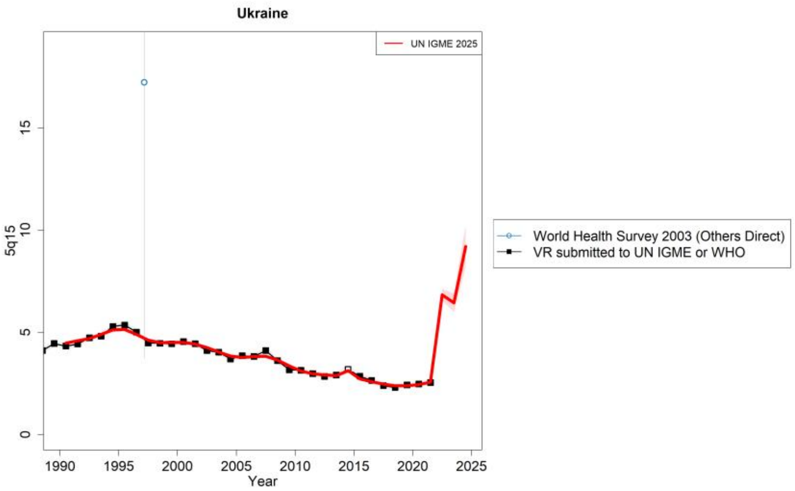
Ukraine
UN IGME 2025
World Health Survey 2003 (Others Direct)
VR submitted to UN IGME or WHO
5q15
Year

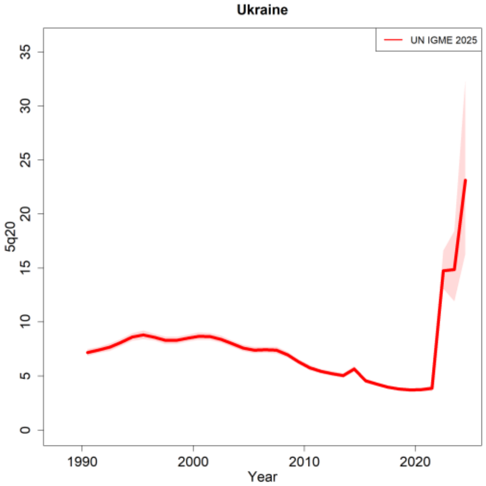
Ukraine
UN IGME 2025
5q20
Year

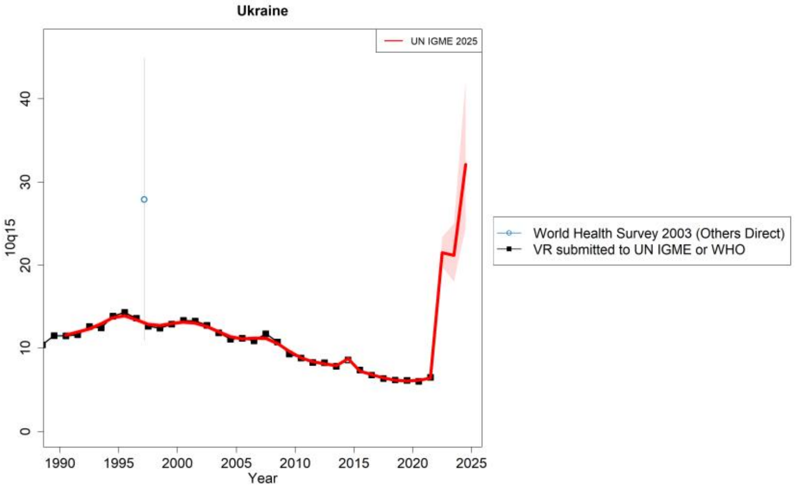
Ukraine
UN IGME 2025
World Health Survey 2003 (Others Direct)
VR submitted to UN IGME or WHO
10q15
Year

United Arab Emirates (ARE)

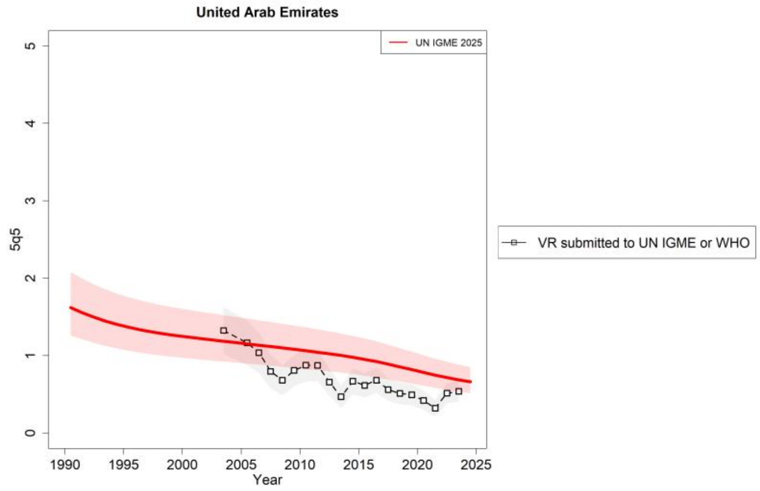

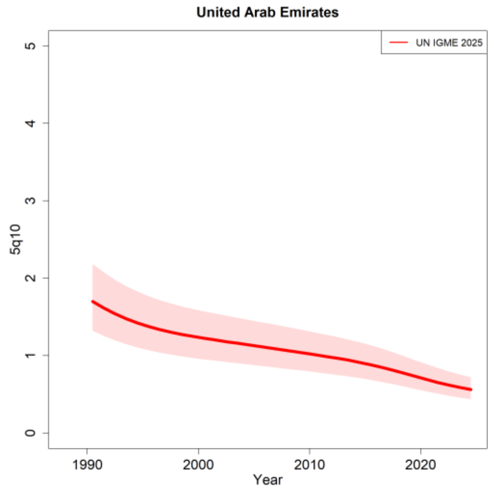

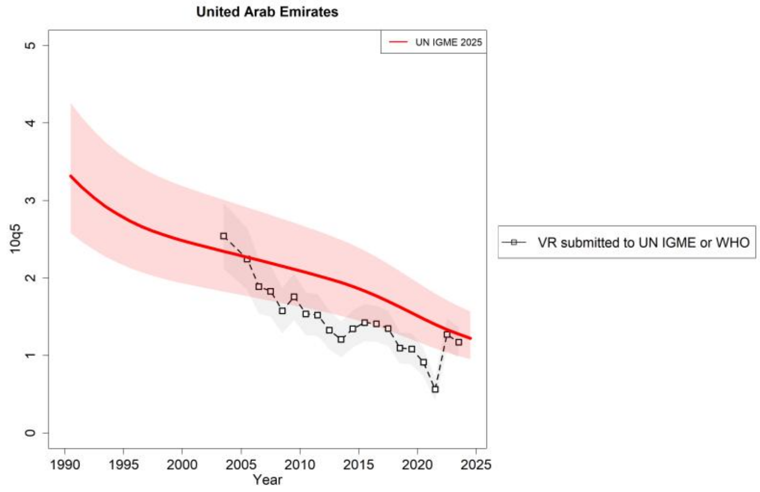

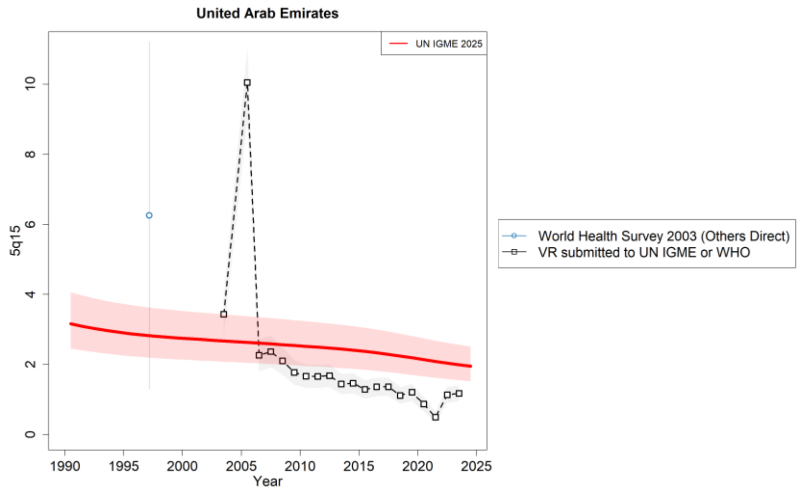
United Arab Emirates
UN IGME 2025
World Health Survey 2003 (Others Direct)
VR submitted to UN IGME or WHO
5q15
Year

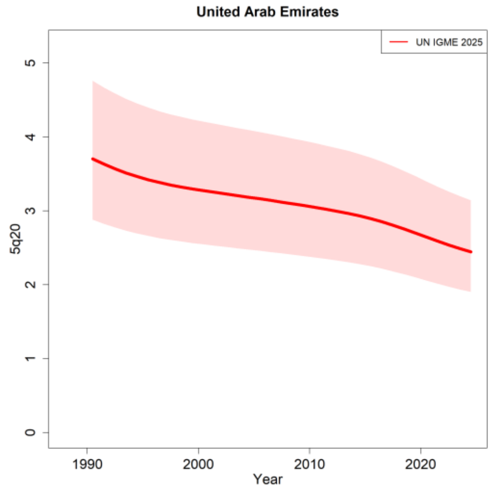
United Arab Emirates
UN IGME 2025
5q20
Year

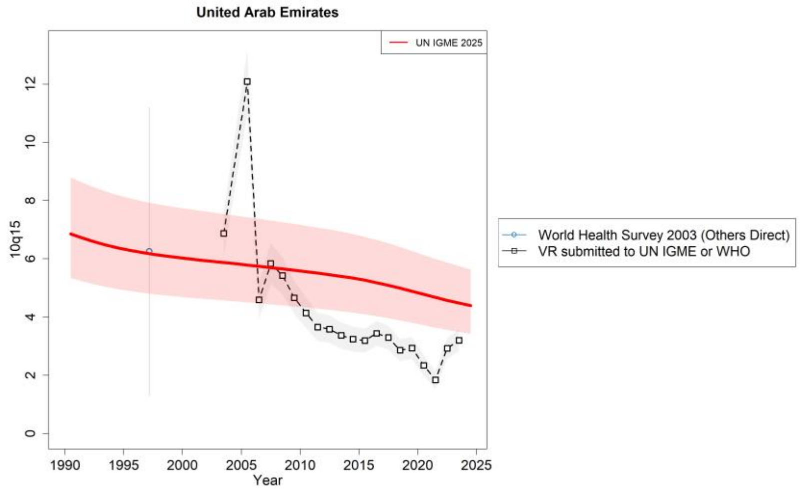
United Arab Emirates
UN IGME 2025
World Health Survey 2003 (Others Direct)
VR submitted to UN IGME or WHO
10q15
Year

United Kingdom (GBR)

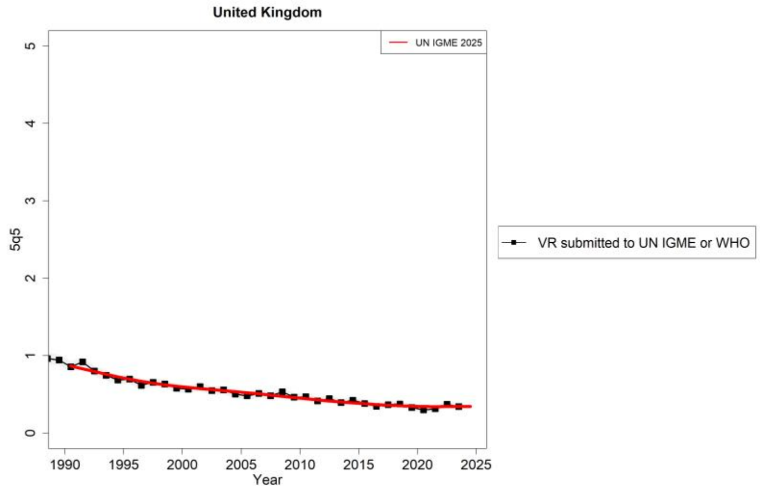

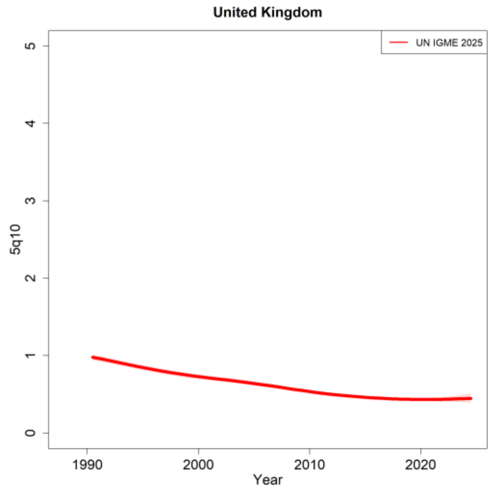

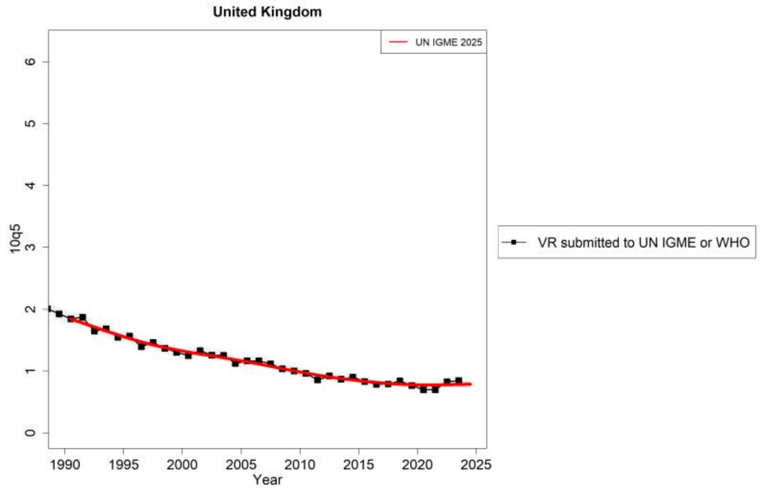

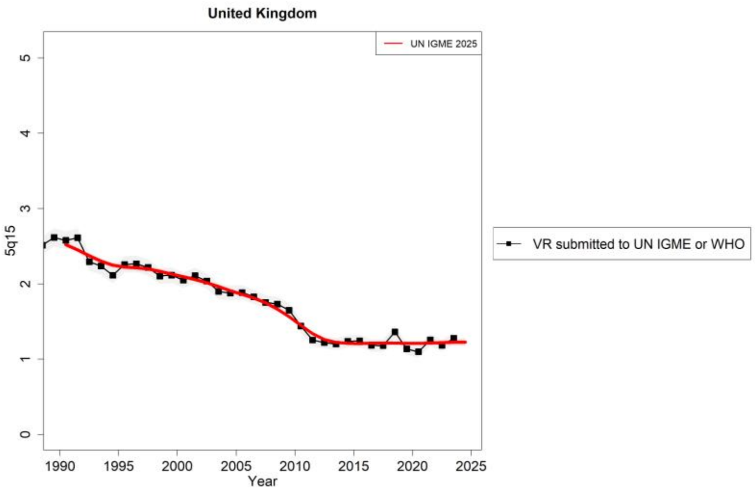
United Kingdom
UN IGME 2025
VR submitted to UN IGME or WHO
5q15
Year
0
1
2
3
4
5
1990
1995
2000
2005
2010
2015
2020
2025

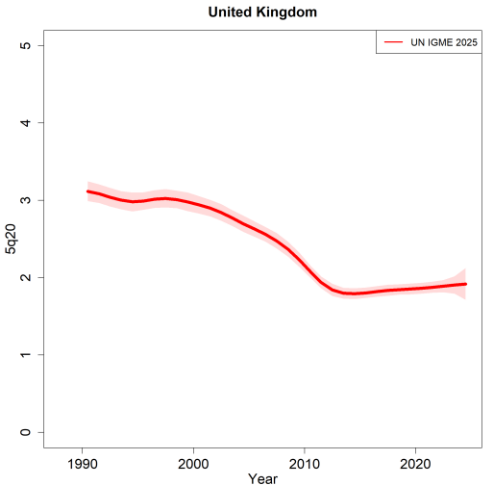
United Kingdom
UN IGME 2025
5q20
Year
0
1
2
3
4
5
1990
2000
2010
2020

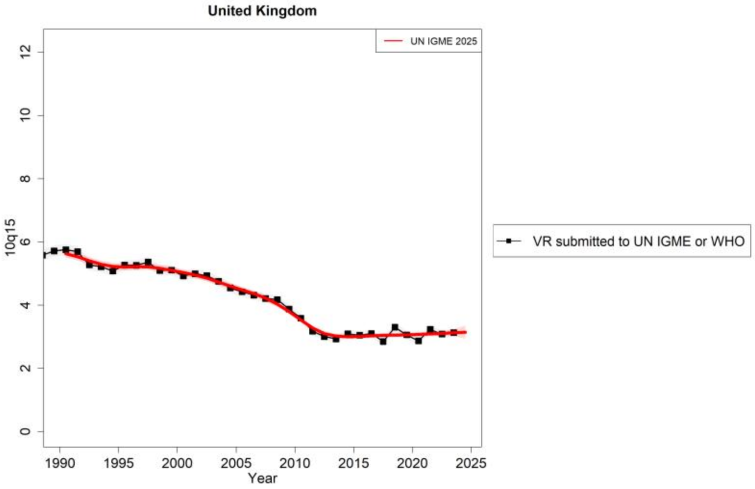
United Kingdom
UN IGME 2025
VR submitted to UN IGME or WHO
10q15
Year
0
2
4
6
8
10
12
1990
1995
2000
2005
2010
2015
2020
2025

United Republic of Tanzania (TZA)

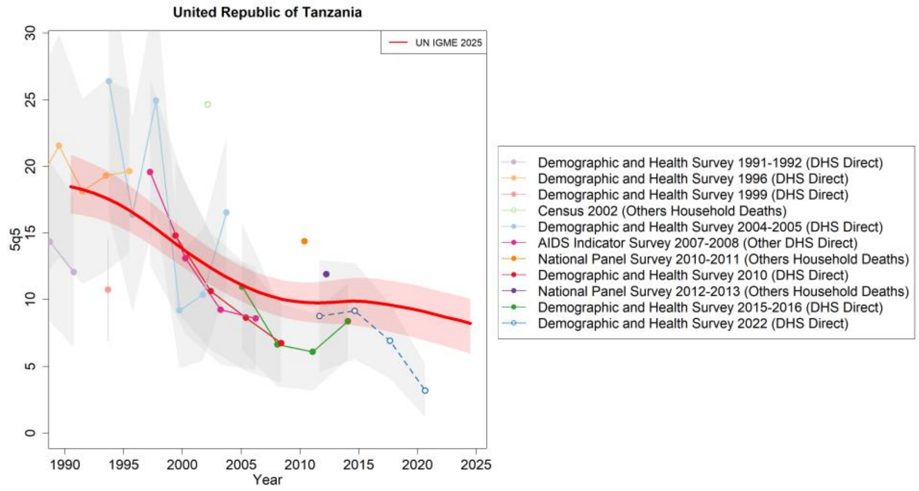

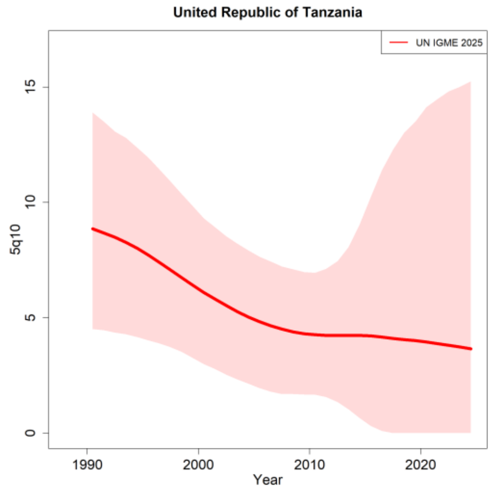

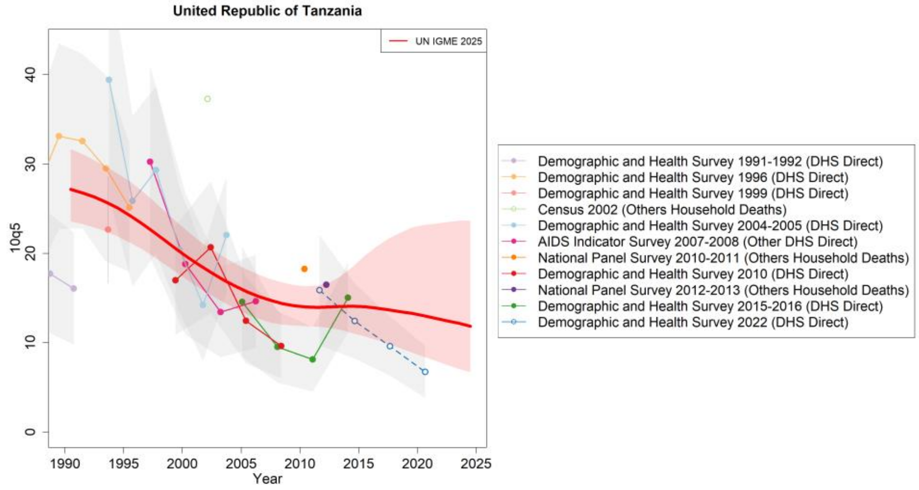

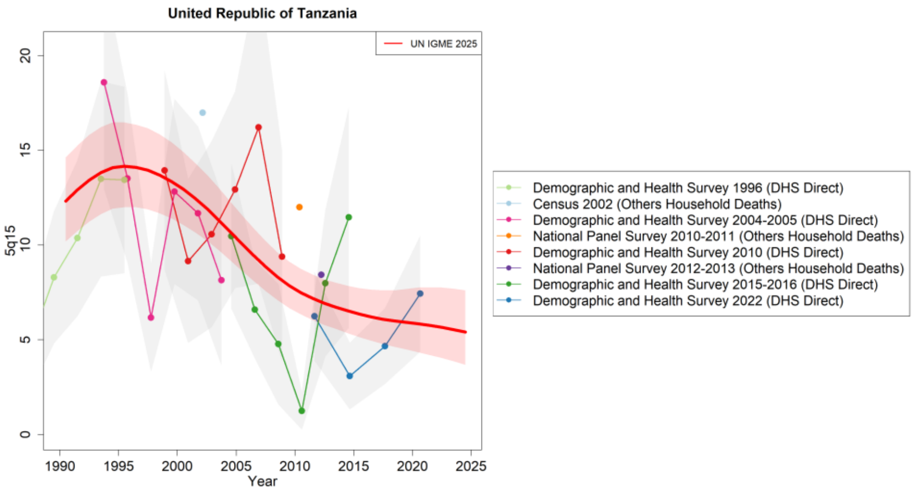
United Republic of Tanzania
UN IGME 2025
5q15
Year
Demographic and Health Survey 1996 (DHS Direct)
Census 2002 (Others Household Deaths)
Demographic and Health Survey 2004-2005 (DHS Direct)
National Panel Survey 2010-2011 (Others Household Deaths)
Demographic and Health Survey 2010 (DHS Direct)
National Panel Survey 2012-2013 (Others Household Deaths)
Demographic and Health Survey 2015-2016 (DHS Direct)
Demographic and Health Survey 2022 (DHS Direct)

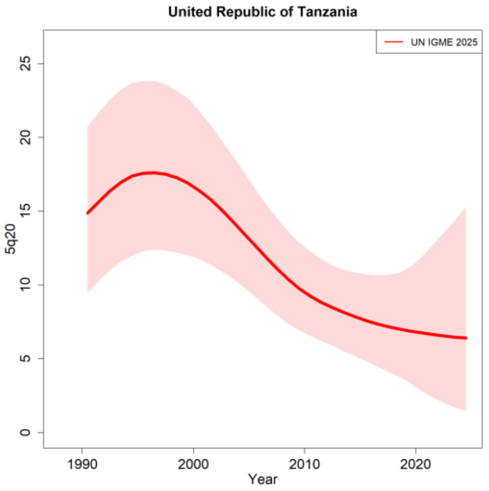
United Republic of Tanzania
UN IGME 2025
5q20
Year

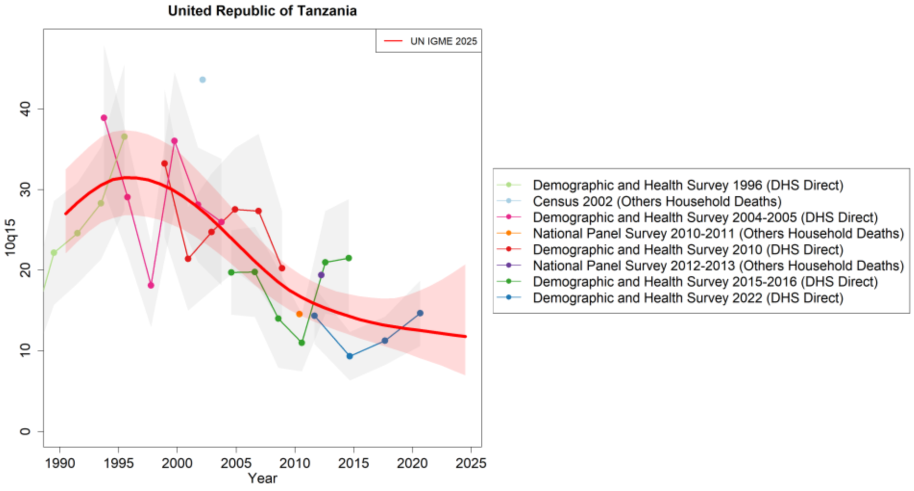
United Republic of Tanzania
UN IGME 2025
10q15
Year
Demographic and Health Survey 1996 (DHS Direct)
Census 2002 (Others Household Deaths)
Demographic and Health Survey 2004-2005 (DHS Direct)
National Panel Survey 2010-2011 (Others Household Deaths)
Demographic and Health Survey 2010 (DHS Direct)
National Panel Survey 2012-2013 (Others Household Deaths)
Demographic and Health Survey 2015-2016 (DHS Direct)
Demographic and Health Survey 2022 (DHS Direct)

United States (USA)

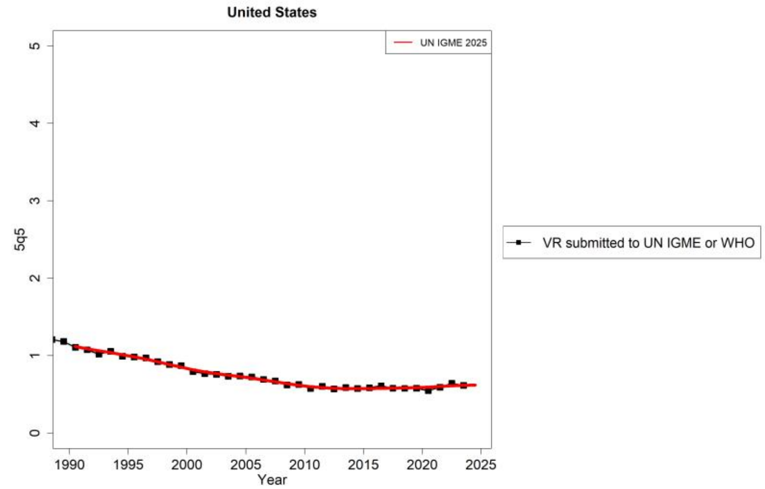

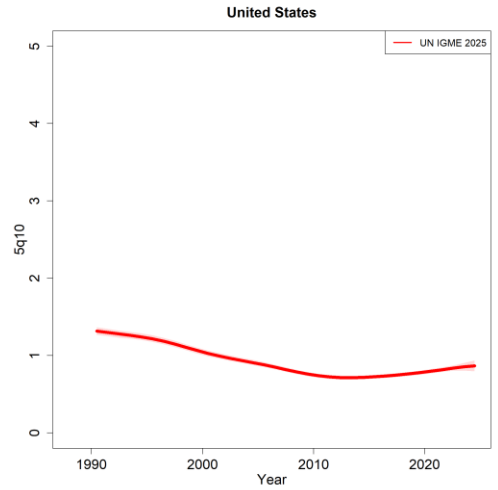

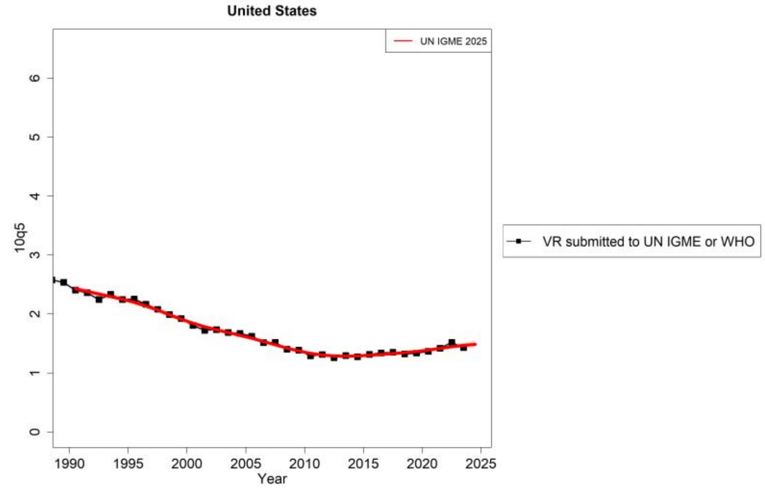

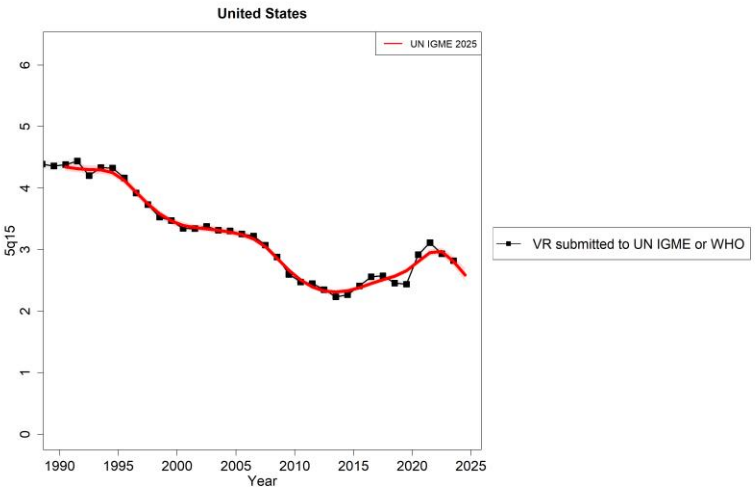
United States
UN IGME 2025
VR submitted to UN IGME or WHO
5q15
Year
0
1
2
3
4
5
6
1990
1995
2000
2005
2010
2015
2020
2025

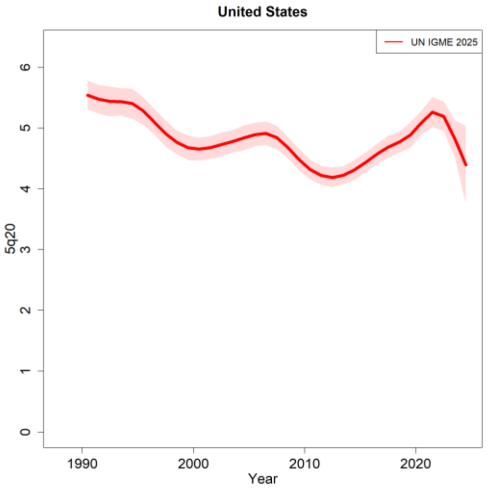
United States
UN IGME 2025
5q20
Year
0
1
2
3
4
5
6
1990
2000
2010
2020

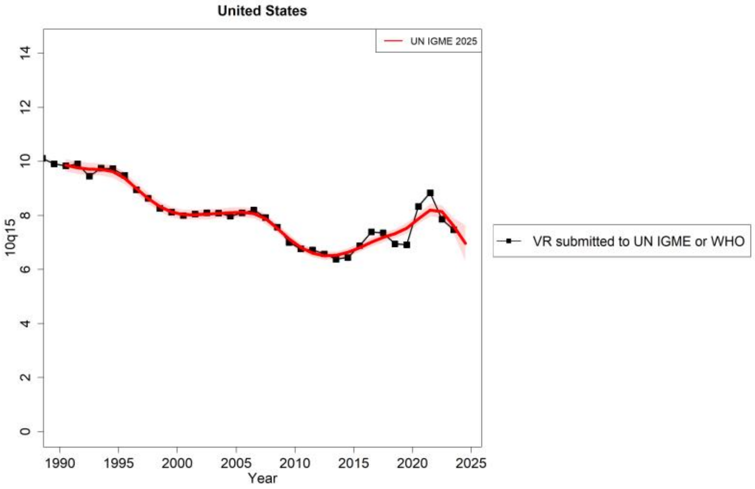
United States
UN IGME 2025
VR submitted to UN IGME or WHO
10q15
Year
0
2
4
6
8
10
12
14
1990
1995
2000
2005
2010
2015
2020
2025

Uruguay (URY)

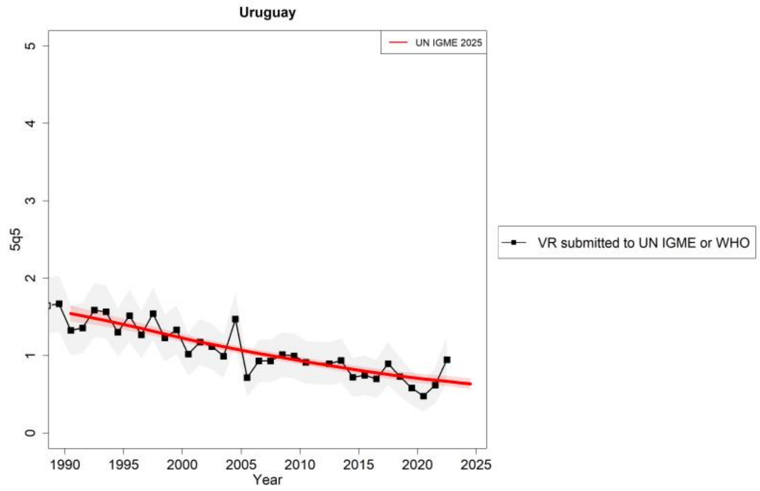
Uruguay
UN IGME 2025
VR submitted to UN IGME or WHO
5q5
Year

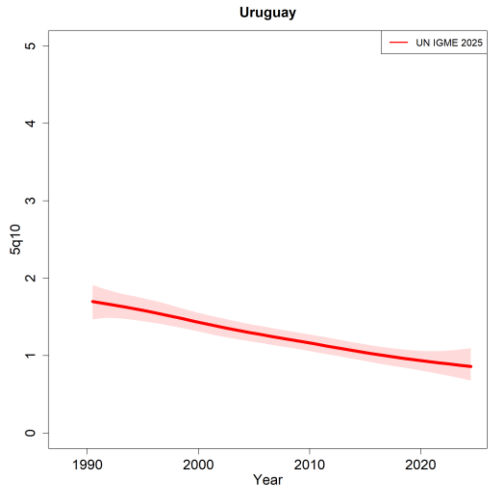
Uruguay
UN IGME 2025
5q10
Year

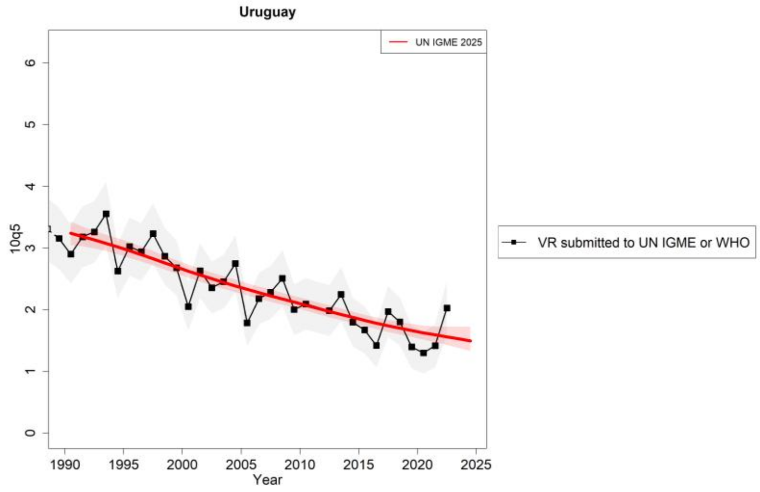
Uruguay
UN IGME 2025
VR submitted to UN IGME or WHO
10q5
Year

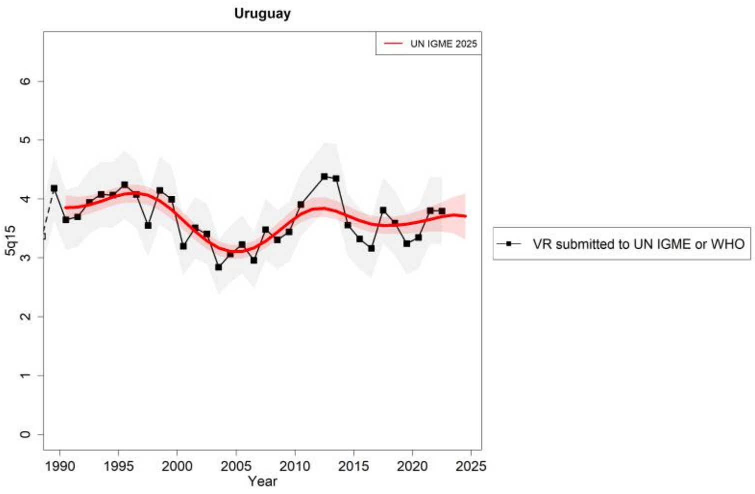
Uruguay
UN IGME 2025
VR submitted to UN IGME or WHO
5q15
Year

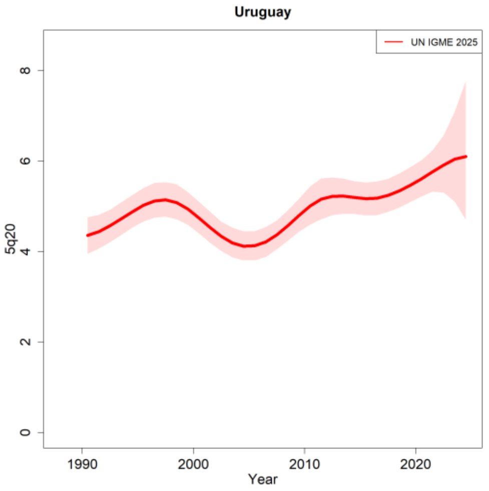
Uruguay
UN IGME 2025
5q20
Year

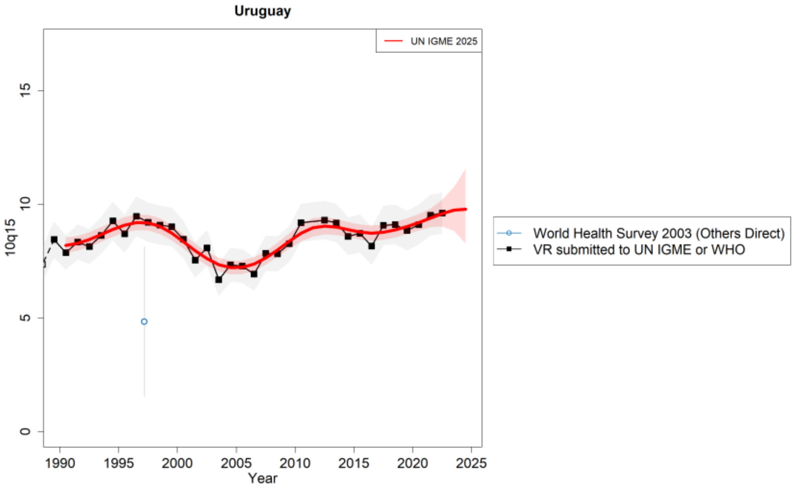
Uruguay
UN IGME 2025
World Health Survey 2003 (Others Direct)
VR submitted to UN IGME or WHO
10q15
Year

Uzbekistan (UZB)

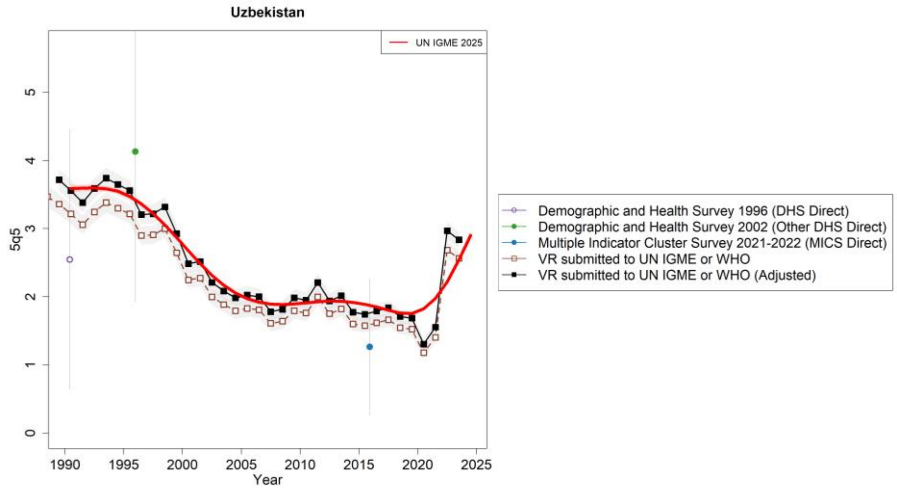

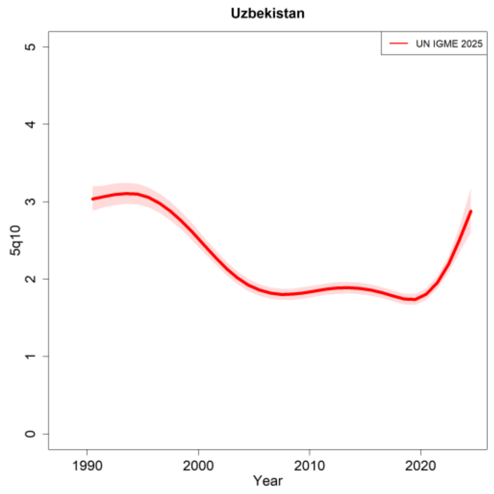

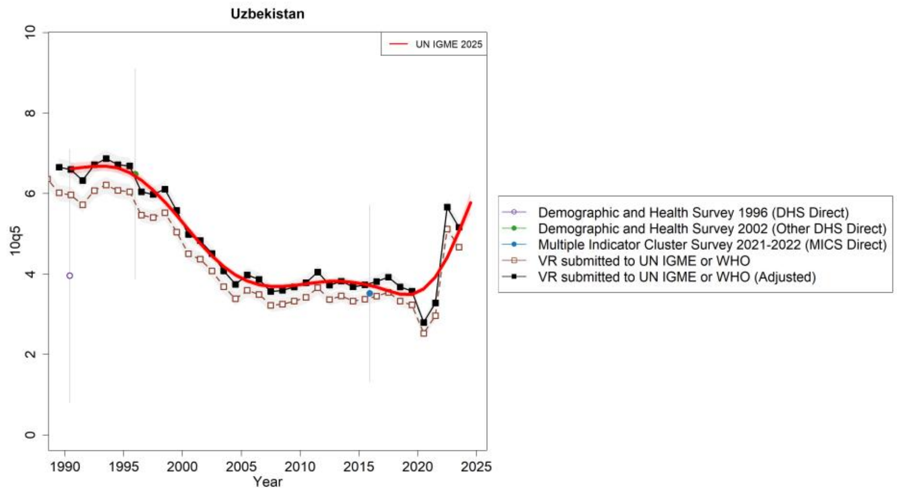

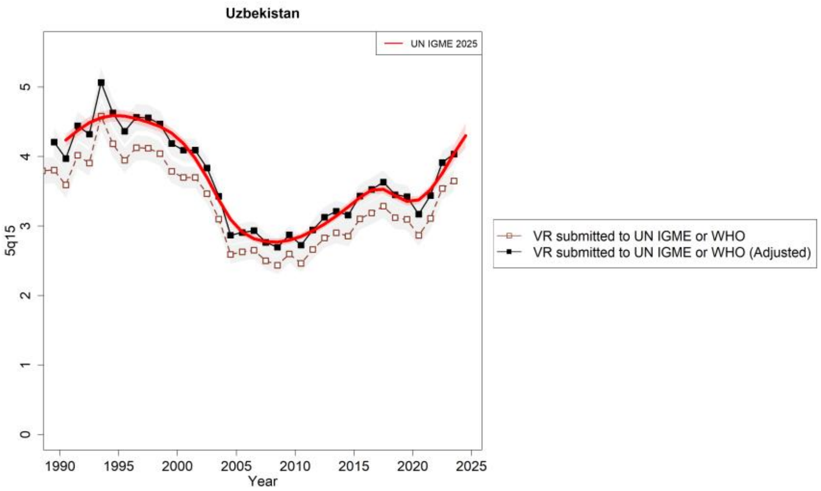
Uzbekistan
UN IGME 2025
VR submitted to UN IGME or WHO
VR submitted to UN IGME or WHO (Adjusted)
5q15
Year

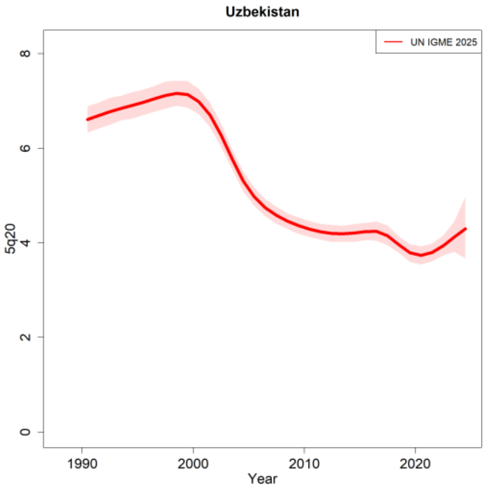
Uzbekistan
UN IGME 2025
5q20
Year

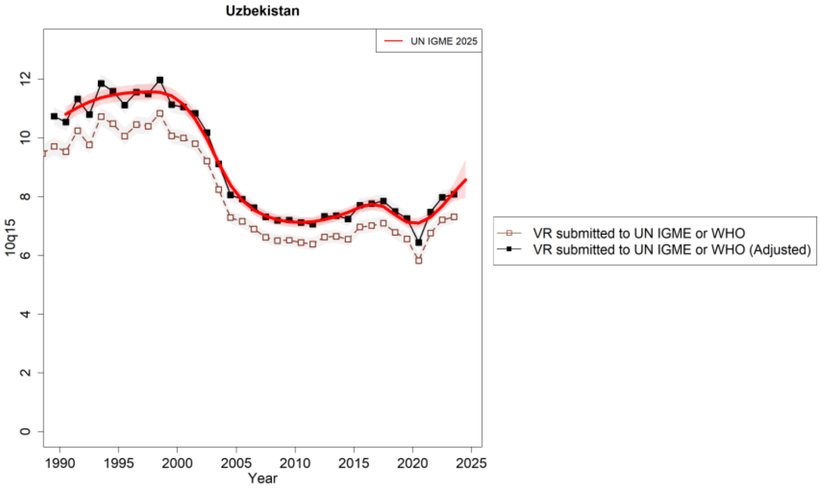
Uzbekistan
UN IGME 2025
VR submitted to UN IGME or WHO
VR submitted to UN IGME or WHO (Adjusted)
10q15
Year

Vanuatu (VUT)

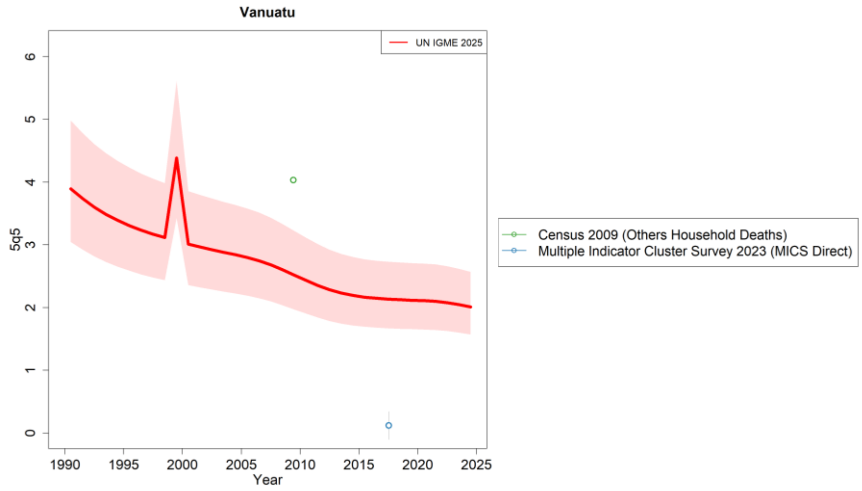
Vanuatu
UN IGME 2025
Census 2009 (Others Household Deaths)
Multiple Indicator Cluster Survey 2023 (MICS Direct)
5q5
0
1
2
3
4
5
6
1990
1995
2000
2005
2010
2015
2020
2025
Year

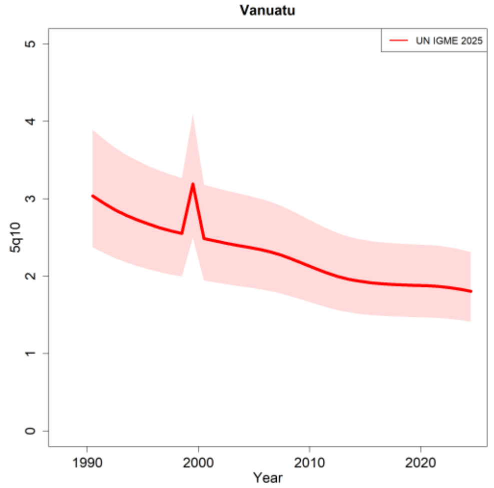
Vanuatu
UN IGME 2025
5q10
0
1
2
3
4
5
1990
2000
2010
2020
Year

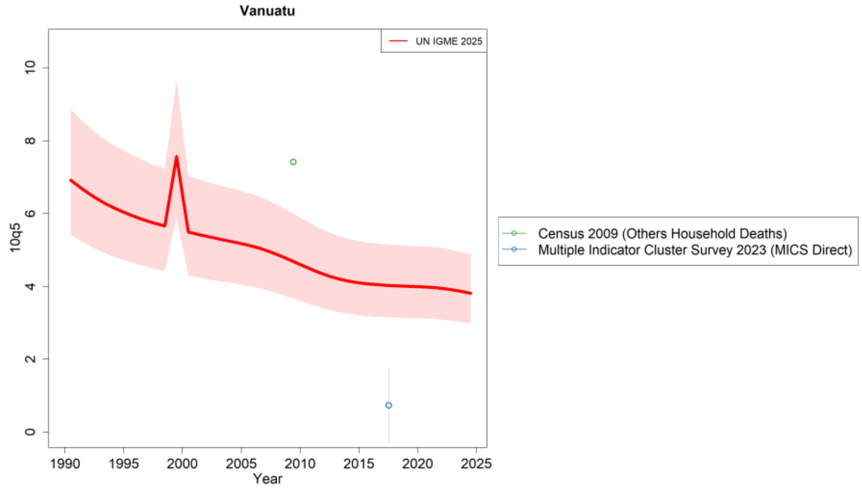
Vanuatu
UN IGME 2025
Census 2009 (Others Household Deaths)
Multiple Indicator Cluster Survey 2023 (MICS Direct)
10q5
0
2
4
6
8
10
1990
1995
2000
2005
2010
2015
2020
2025
Year

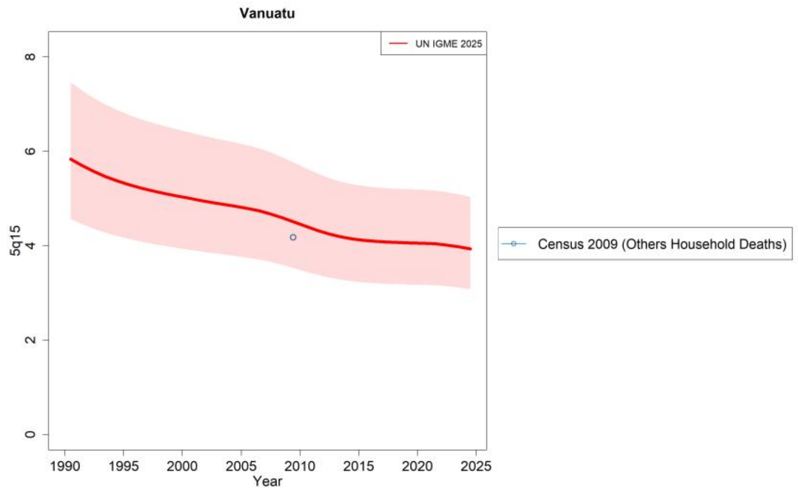
Vanuatu
UN IGME 2025
Census 2009 (Others Household Deaths)
5q15
Year
0
2
4
6
8
1990
1995
2000
2005
2010
2015
2020
2025

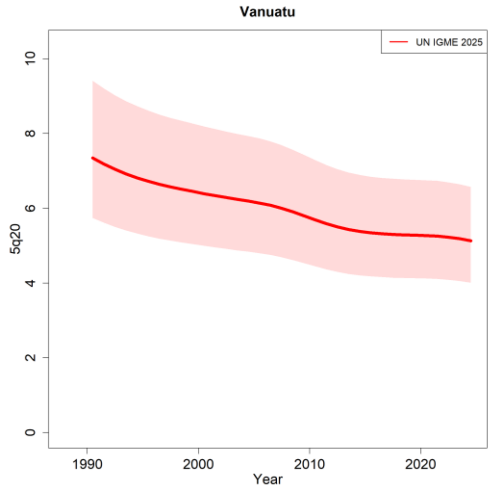
Vanuatu
UN IGME 2025
5q20
Year
0
2
4
6
8
10
1990
2000
2010
2020

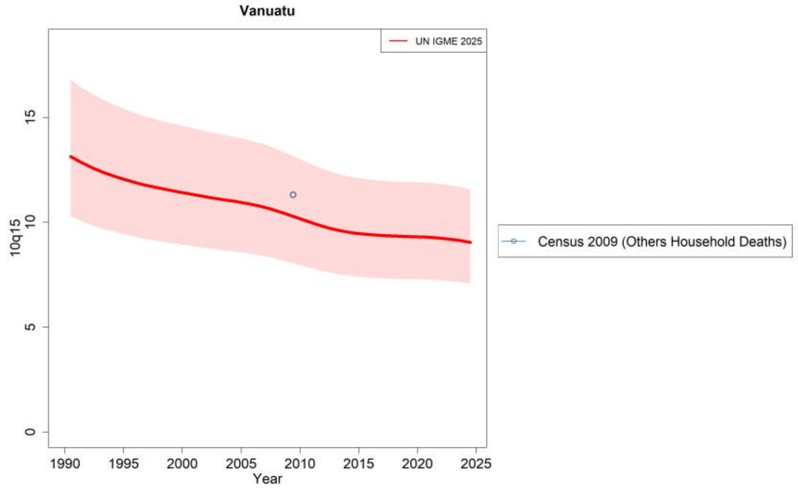
Vanuatu
UN IGME 2025
Census 2009 (Others Household Deaths)
10q15
Year
0
5
10
15
1990
1995
2000
2005
2010
2015
2020
2025

Venezuela (Bolivarian Republic of) (VEN)

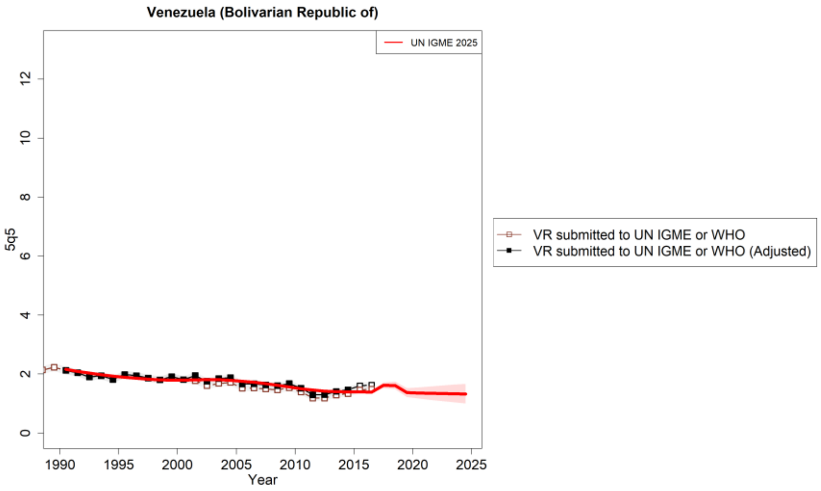

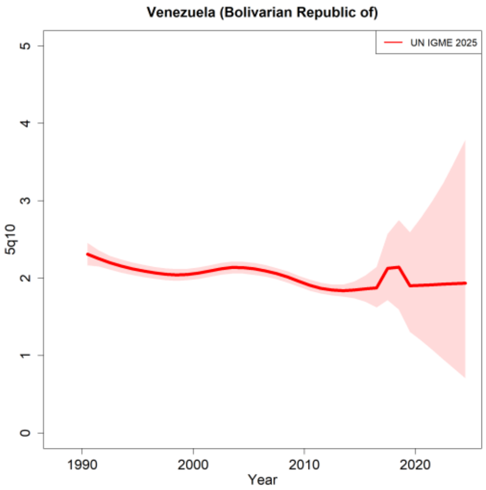

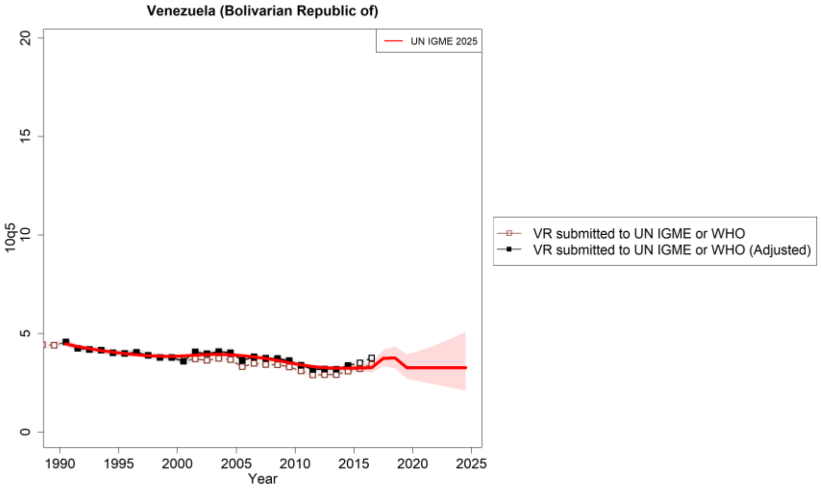

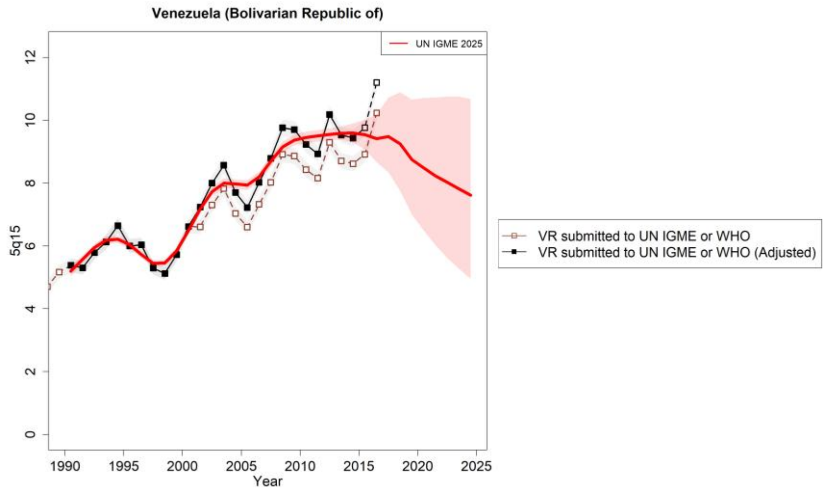
Venezuela (Bolivarian Republic of)
UN IGME 2025
5q15
Year
VR submitted to UN IGME or WHO
VR submitted to UN IGME or WHO (Adjusted)

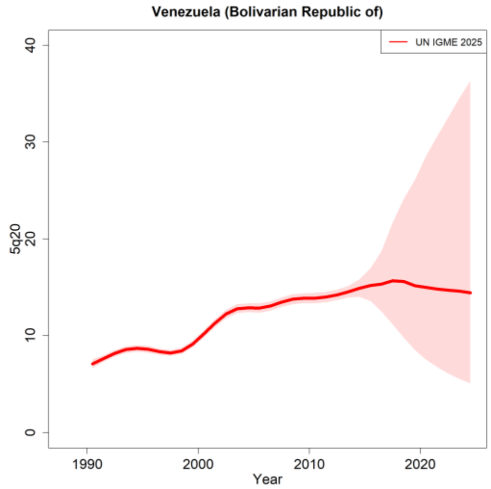
Venezuela (Bolivarian Republic of)
UN IGME 2025
5q20
Year

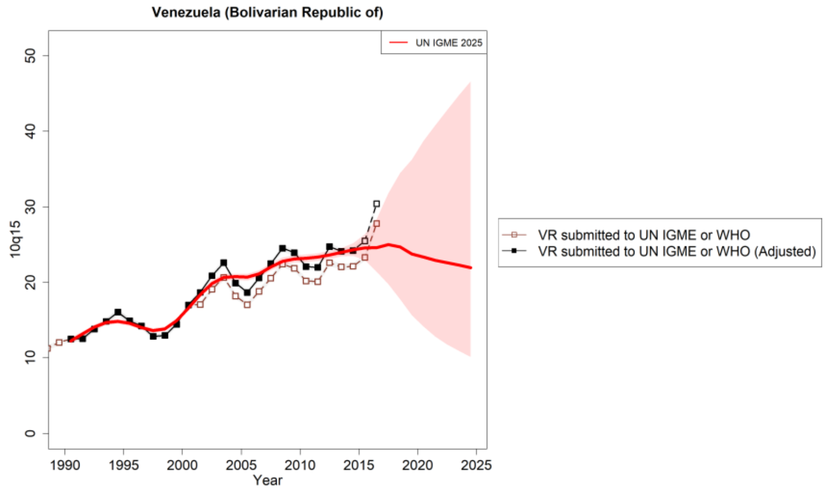
Venezuela (Bolivarian Republic of)
UN IGME 2025
10q15
Year
VR submitted to UN IGME or WHO
VR submitted to UN IGME or WHO (Adjusted)

Viet Nam (VNM)

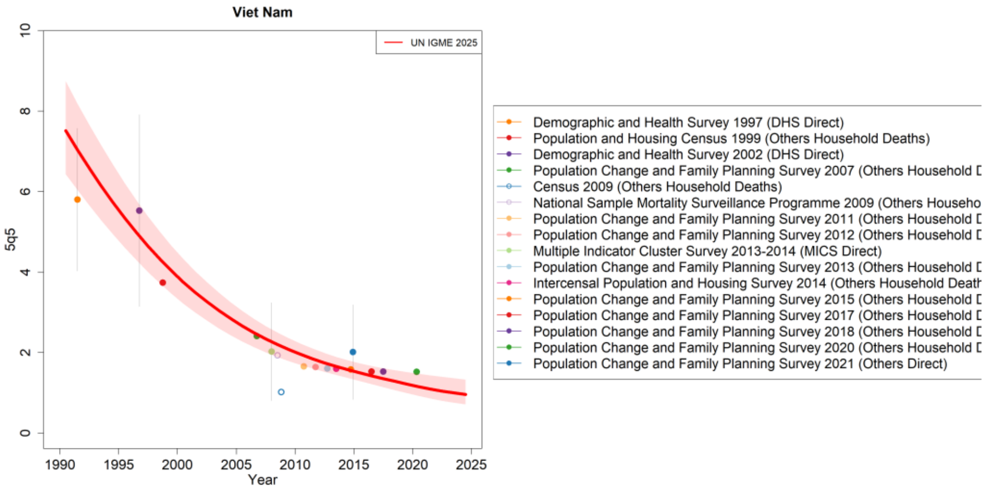
Viet Nam
UN IGME 2025
5q5
Year
Demographic and Health Survey 1997 (DHS Direct)
Population and Housing Census 1999 (Others Household Deaths)
Demographic and Health Survey 2002 (DHS Direct)
Population Change and Family Planning Survey 2007 (Others Household D
Census 2009 (Others Household Deaths)
National Sample Mortality Surveillance Programme 2009 (Others Househo
Population Change and Family Planning Survey 2011 (Others Household D
Population Change and Family Planning Survey 2012 (Others Household D
Multiple Indicator Cluster Survey 2013-2014 (MICS Direct)
Population Change and Family Planning Survey 2013 (Others Household D
Intercensal Population and Housing Survey 2014 (Others Household Death
Population Change and Family Planning Survey 2015 (Others Household D
Population Change and Family Planning Survey 2017 (Others Household D
Population Change and Family Planning Survey 2018 (Others Household D
Population Change and Family Planning Survey 2020 (Others Household D
Population Change and Family Planning Survey 2021 (Others Direct)

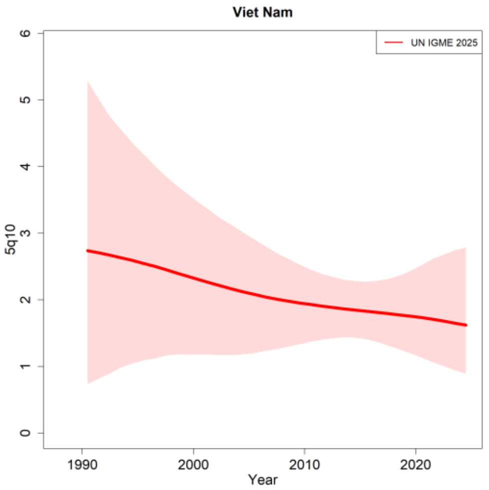
Viet Nam
UN IGME 2025
5q10
Year

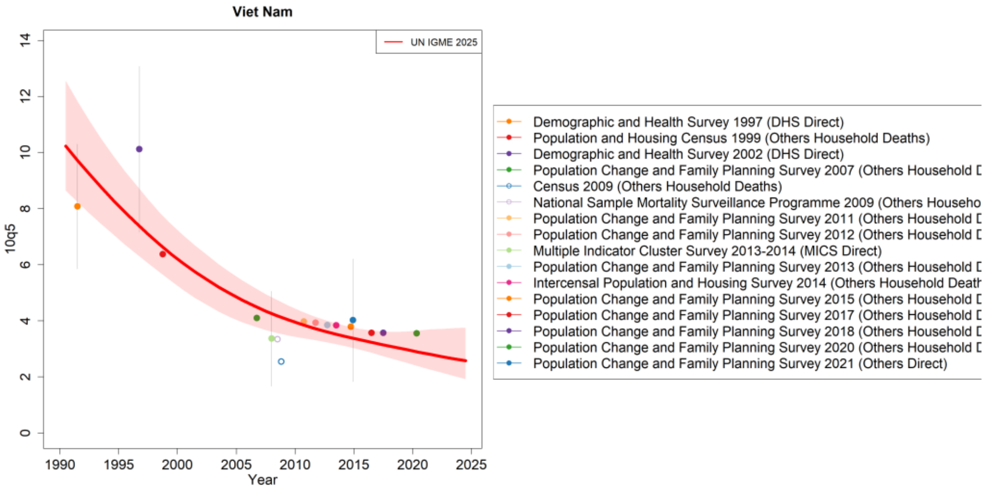
Viet Nam
UN IGME 2025
10q5
Year
Demographic and Health Survey 1997 (DHS Direct)
Population and Housing Census 1999 (Others Household Deaths)
Demographic and Health Survey 2002 (DHS Direct)
Population Change and Family Planning Survey 2007 (Others Household D
Census 2009 (Others Household Deaths)
National Sample Mortality Surveillance Programme 2009 (Others Househo
Population Change and Family Planning Survey 2011 (Others Household D
Population Change and Family Planning Survey 2012 (Others Household D
Multiple Indicator Cluster Survey 2013-2014 (MICS Direct)
Population Change and Family Planning Survey 2013 (Others Household D
Intercensal Population and Housing Survey 2014 (Others Household Death
Population Change and Family Planning Survey 2015 (Others Household D
Population Change and Family Planning Survey 2017 (Others Household D
Population Change and Family Planning Survey 2018 (Others Household D
Population Change and Family Planning Survey 2020 (Others Household D
Population Change and Family Planning Survey 2021 (Others Direct)

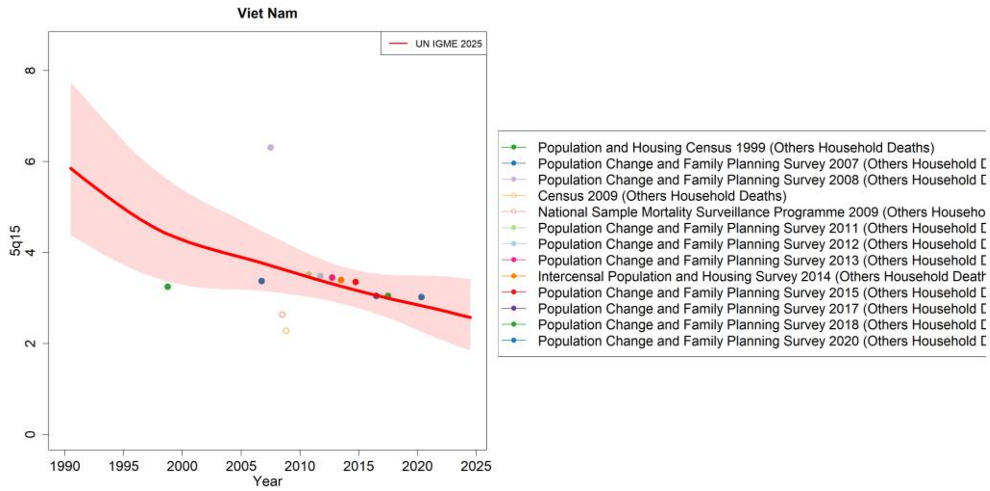
Viet Nam
UN IGME 2025
5q15
Year
Population and Housing Census 1999 (Others Household Deaths)
Population Change and Family Planning Survey 2007 (Others Household D
Population Change and Family Planning Survey 2008 (Others Household D
Census 2009 (Others Household Deaths)
National Sample Mortality Surveillance Programme 2009 (Others Househo
Population Change and Family Planning Survey 2011 (Others Household D
Population Change and Family Planning Survey 2012 (Others Household D
Population Change and Family Planning Survey 2013 (Others Household D
Intercensal Population and Housing Survey 2014 (Others Household Death
Population Change and Family Planning Survey 2015 (Others Household D
Population Change and Family Planning Survey 2017 (Others Household D
Population Change and Family Planning Survey 2018 (Others Household D
Population Change and Family Planning Survey 2020 (Others Household D

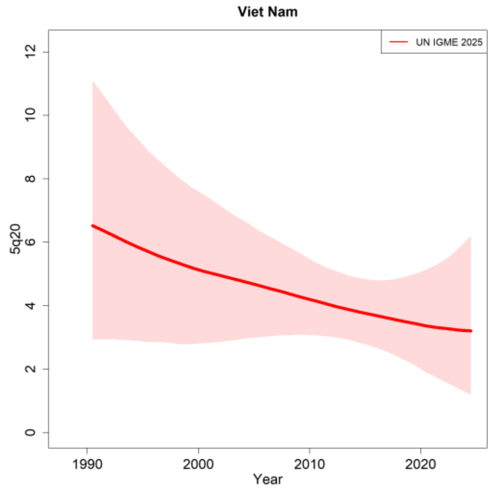
Viet Nam
UN IGME 2025
5q20
Year

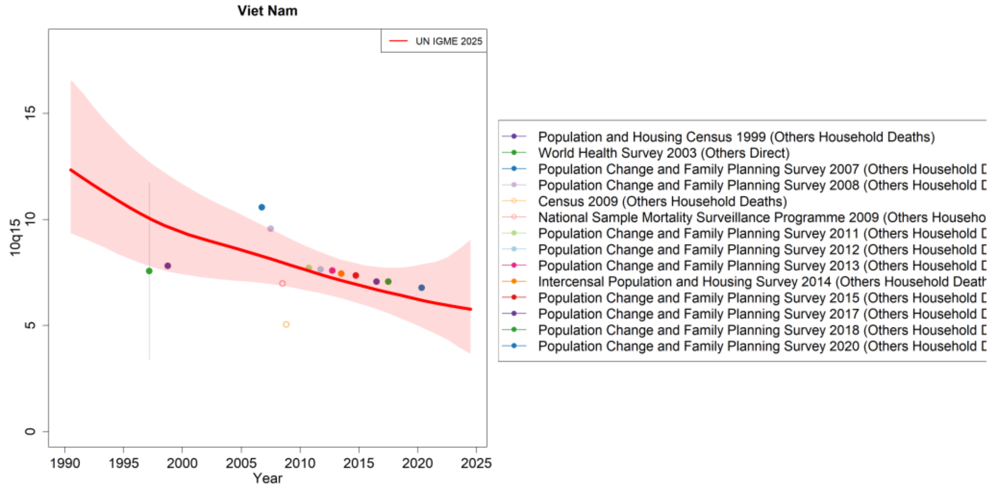
Viet Nam
UN IGME 2025
10q15
Year
Population and Housing Census 1999 (Others Household Deaths)
World Health Survey 2003 (Others Direct)
Population Change and Family Planning Survey 2007 (Others Household D
Population Change and Family Planning Survey 2008 (Others Household D
Census 2009 (Others Household Deaths)
National Sample Mortality Surveillance Programme 2009 (Others Househo
Population Change and Family Planning Survey 2011 (Others Household D
Population Change and Family Planning Survey 2012 (Others Household D
Population Change and Family Planning Survey 2013 (Others Household D
Intercensal Population and Housing Survey 2014 (Others Household Death
Population Change and Family Planning Survey 2015 (Others Household D
Population Change and Family Planning Survey 2017 (Others Household D
Population Change and Family Planning Survey 2018 (Others Household D
Population Change and Family Planning Survey 2020 (Others Household D

Yemen (YEM)

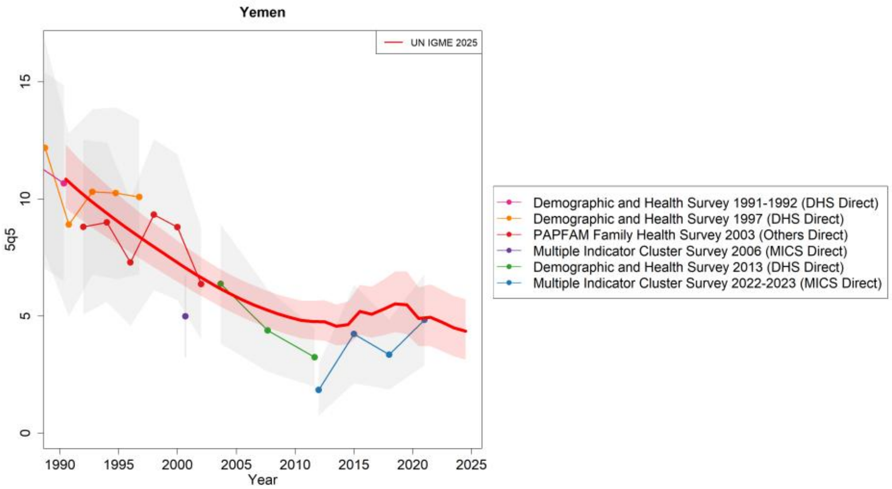
Yemen
UN IGME 2025
5q5
Year
Demographic and Health Survey 1991-1992 (DHS Direct)
Demographic and Health Survey 1997 (DHS Direct)
PAPFAM Family Health Survey 2003 (Others Direct)
Multiple Indicator Cluster Survey 2006 (MICS Direct)
Demographic and Health Survey 2013 (DHS Direct)
Multiple Indicator Cluster Survey 2022-2023 (MICS Direct)

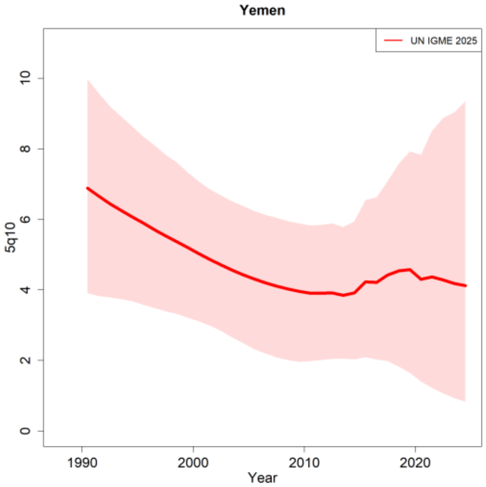
Yemen
UN IGME 2025
5q10
Year

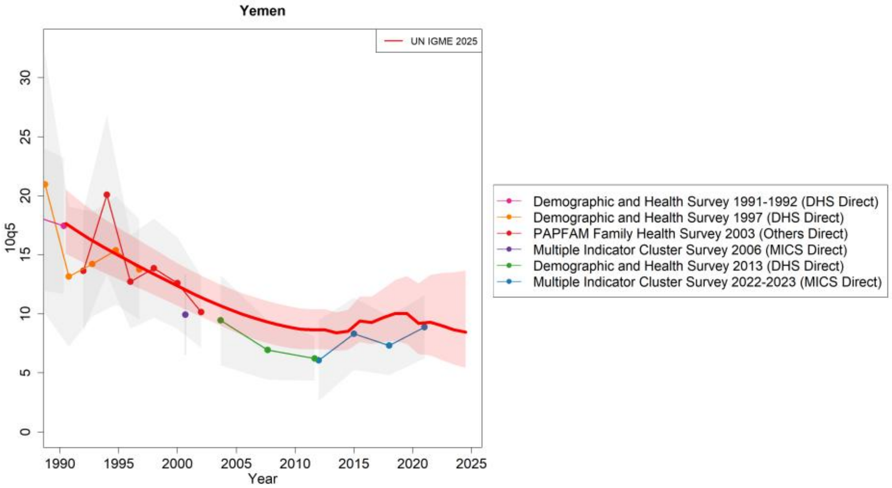
Yemen
UN IGME 2025
10q5
Year
Demographic and Health Survey 1991-1992 (DHS Direct)
Demographic and Health Survey 1997 (DHS Direct)
PAPFAM Family Health Survey 2003 (Others Direct)
Multiple Indicator Cluster Survey 2006 (MICS Direct)
Demographic and Health Survey 2013 (DHS Direct)
Multiple Indicator Cluster Survey 2022-2023 (MICS Direct)

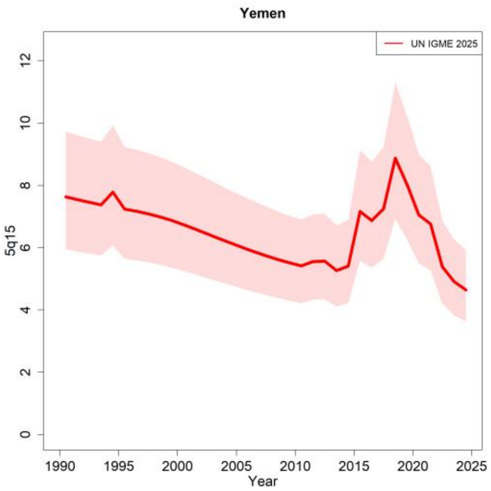
Yemen
UN IGME 2025
5q15
Year
0
2
4
6
8
10
12
1990
1995
2000
2005
2010
2015
2020
2025

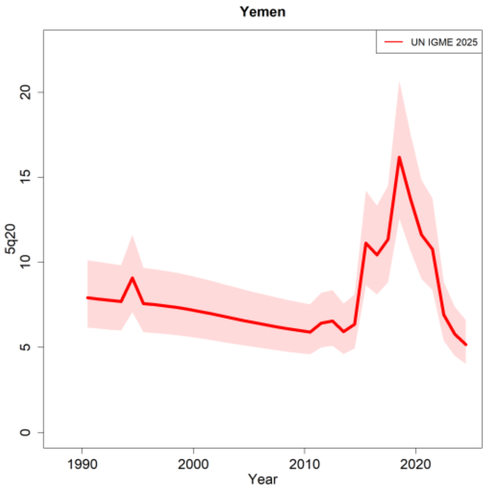
Yemen
UN IGME 2025
5q20
Year
0
5
10
15
20
1990
2000
2010
2020

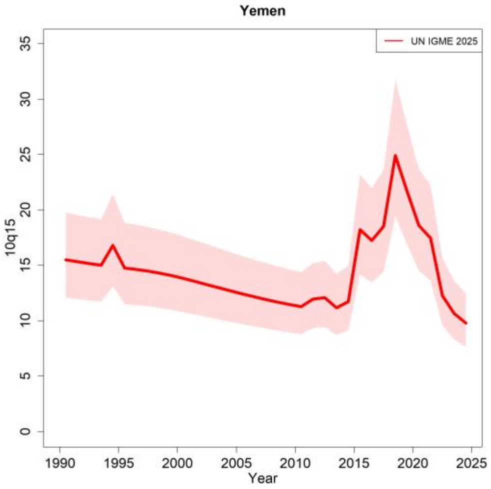
Yemen
UN IGME 2025
10q15
Year
0
5
10
15
20
25
30
35
1990
1995
2000
2005
2010
2015
2020
2025

Zambia (ZMB)

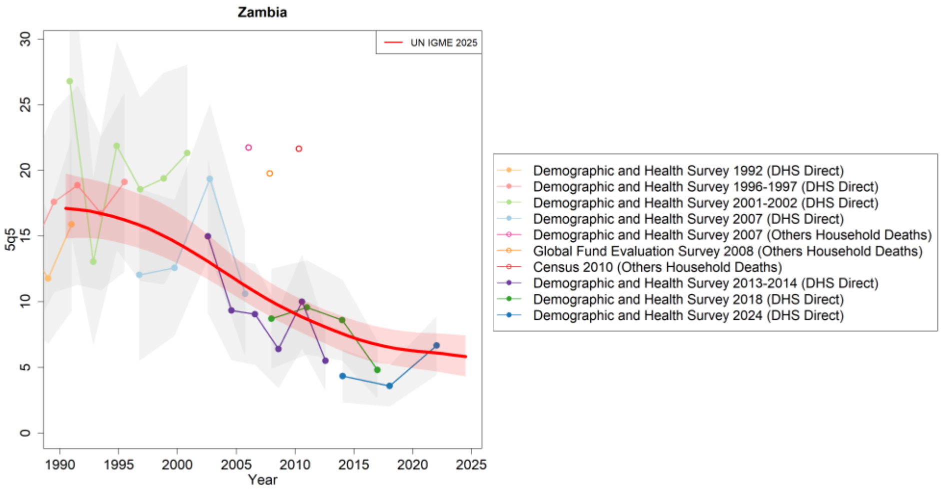
Zambia
UN IGME 2025
5q5
Year
Demographic and Health Survey 1992 (DHS Direct)
Demographic and Health Survey 1996-1997 (DHS Direct)
Demographic and Health Survey 2001-2002 (DHS Direct)
Demographic and Health Survey 2007 (DHS Direct)
Demographic and Health Survey 2007 (Others Household Deaths)
Global Fund Evaluation Survey 2008 (Others Household Deaths)
Census 2010 (Others Household Deaths)
Demographic and Health Survey 2013-2014 (DHS Direct)
Demographic and Health Survey 2018 (DHS Direct)
Demographic and Health Survey 2024 (DHS Direct)

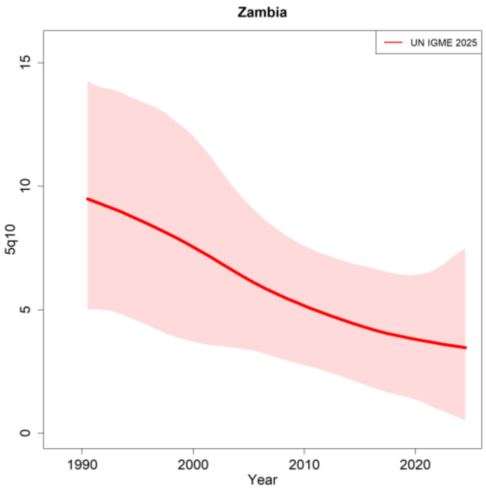
Zambia
UN IGME 2025
5q10
Year

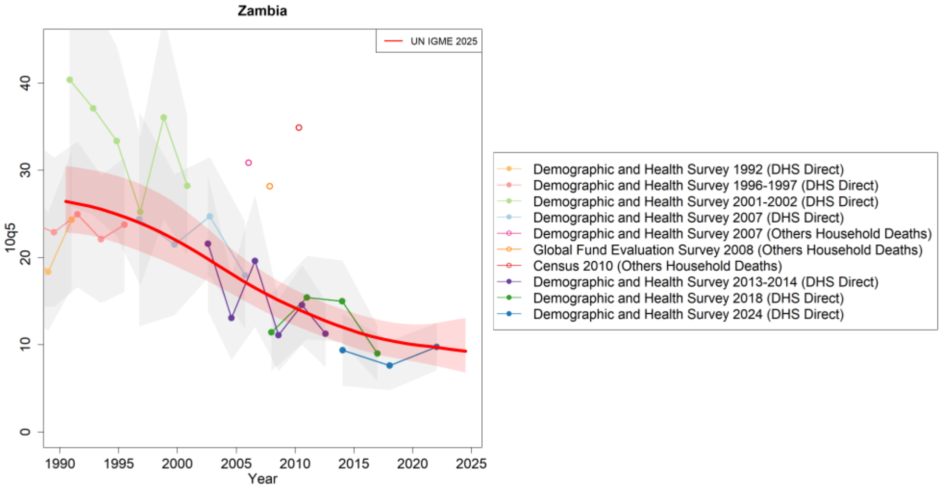
Zambia
UN IGME 2025
10q5
Year
Demographic and Health Survey 1992 (DHS Direct)
Demographic and Health Survey 1996-1997 (DHS Direct)
Demographic and Health Survey 2001-2002 (DHS Direct)
Demographic and Health Survey 2007 (DHS Direct)
Demographic and Health Survey 2007 (Others Household Deaths)
Global Fund Evaluation Survey 2008 (Others Household Deaths)
Census 2010 (Others Household Deaths)
Demographic and Health Survey 2013-2014 (DHS Direct)
Demographic and Health Survey 2018 (DHS Direct)
Demographic and Health Survey 2024 (DHS Direct)

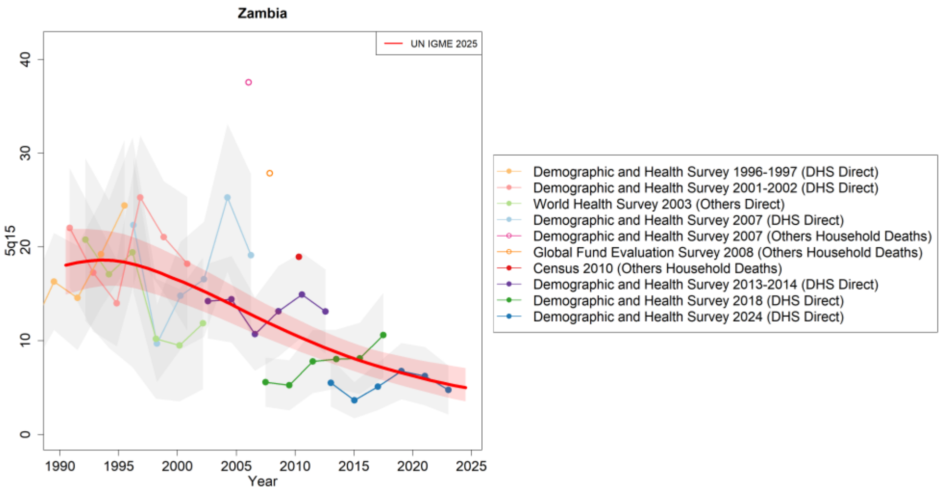
Zambia
UN IGME 2025
5q15
Year
Demographic and Health Survey 1996-1997 (DHS Direct)
Demographic and Health Survey 2001-2002 (DHS Direct)
World Health Survey 2003 (Others Direct)
Demographic and Health Survey 2007 (DHS Direct)
Demographic and Health Survey 2007 (Others Household Deaths)
Global Fund Evaluation Survey 2008 (Others Household Deaths)
Census 2010 (Others Household Deaths)
Demographic and Health Survey 2013-2014 (DHS Direct)
Demographic and Health Survey 2018 (DHS Direct)
Demographic and Health Survey 2024 (DHS Direct)

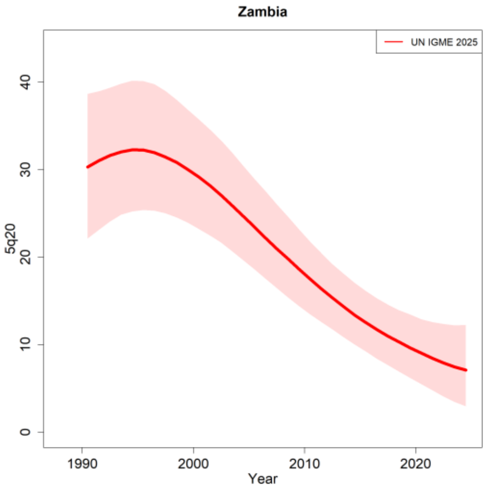
Zambia
UN IGME 2025
5q20
Year

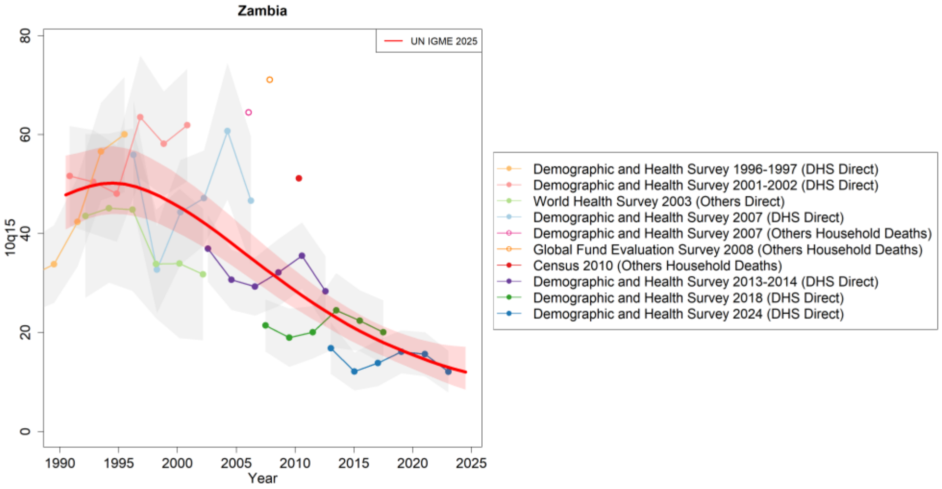
Zambia
UN IGME 2025
10q15
Year
Demographic and Health Survey 1996-1997 (DHS Direct)
Demographic and Health Survey 2001-2002 (DHS Direct)
World Health Survey 2003 (Others Direct)
Demographic and Health Survey 2007 (DHS Direct)
Demographic and Health Survey 2007 (Others Household Deaths)
Global Fund Evaluation Survey 2008 (Others Household Deaths)
Census 2010 (Others Household Deaths)
Demographic and Health Survey 2013-2014 (DHS Direct)
Demographic and Health Survey 2018 (DHS Direct)
Demographic and Health Survey 2024 (DHS Direct)

Zimbabwe (ZWE)

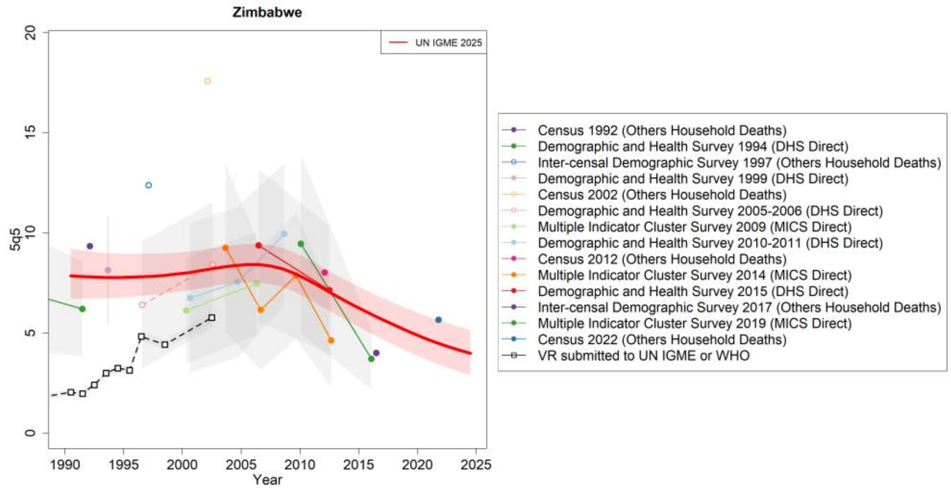

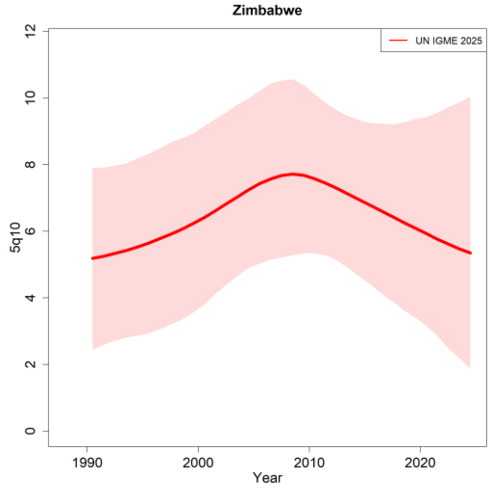

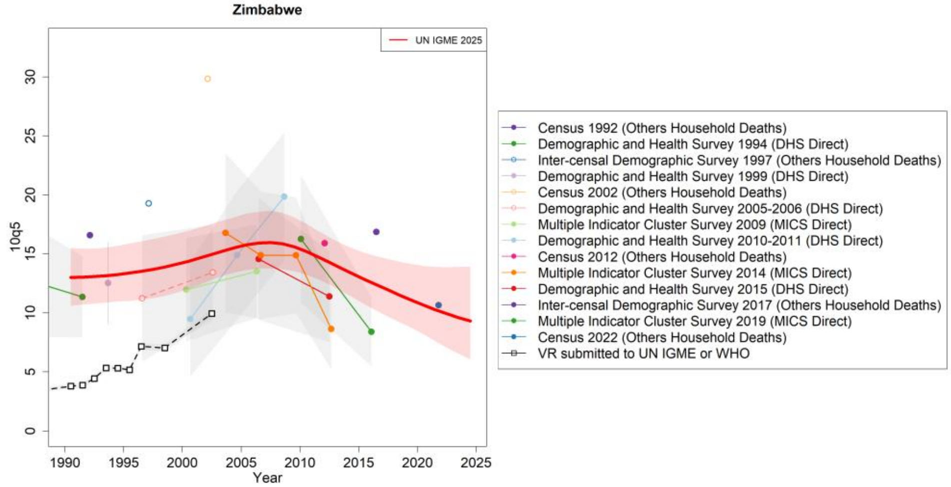

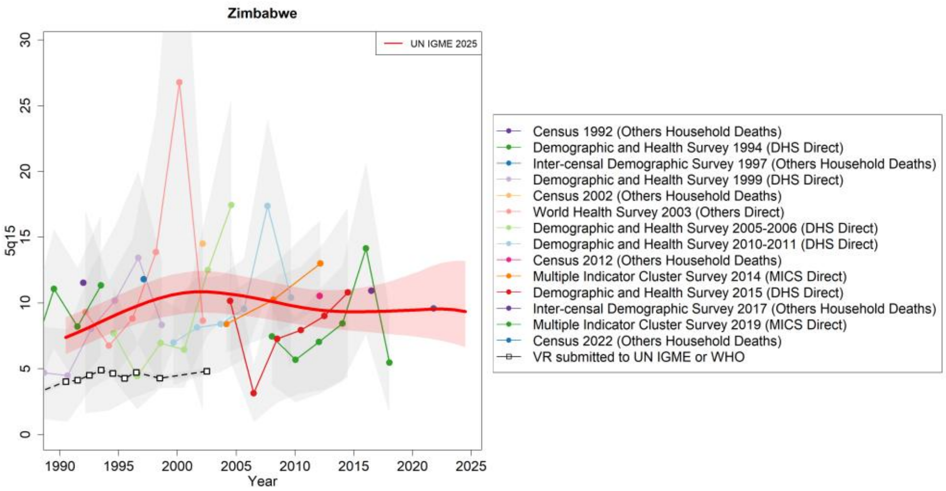
Zimbabwe
UN IGME 2025
5q15
Year
Census 1992 (Others Household Deaths)
Demographic and Health Survey 1994 (DHS Direct)
Inter-censal Demographic Survey 1997 (Others Household Deaths)
Demographic and Health Survey 1999 (DHS Direct)
Census 2002 (Others Household Deaths)
World Health Survey 2003 (Others Direct)
Demographic and Health Survey 2005-2006 (DHS Direct)
Demographic and Health Survey 2010-2011 (DHS Direct)
Census 2012 (Others Household Deaths)
Multiple Indicator Cluster Survey 2014 (MICS Direct)
Demographic and Health Survey 2015 (DHS Direct)
Inter-censal Demographic Survey 2017 (Others Household Deaths)
Multiple Indicator Cluster Survey 2019 (MICS Direct)
Census 2022 (Others Household Deaths)
VR submitted to UN IGME or WHO

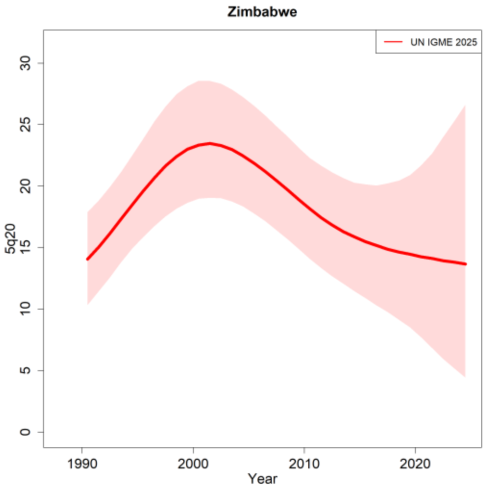
Zimbabwe
UN IGME 2025
5q20
Year

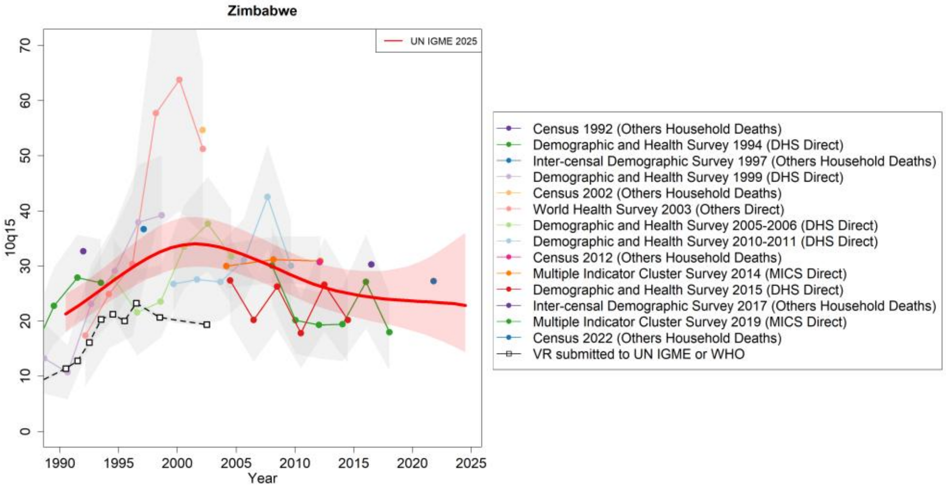
Zimbabwe
UN IGME 2025
10q15
Year
Census 1992 (Others Household Deaths)
Demographic and Health Survey 1994 (DHS Direct)
Inter-censal Demographic Survey 1997 (Others Household Deaths)
Demographic and Health Survey 1999 (DHS Direct)
Census 2002 (Others Household Deaths)
World Health Survey 2003 (Others Direct)
Demographic and Health Survey 2005-2006 (DHS Direct)
Demographic and Health Survey 2010-2011 (DHS Direct)
Census 2012 (Others Household Deaths)
Multiple Indicator Cluster Survey 2014 (MICS Direct)
Demographic and Health Survey 2015 (DHS Direct)
Inter-censal Demographic Survey 2017 (Others Household Deaths)
Multiple Indicator Cluster Survey 2019 (MICS Direct)
Census 2022 (Others Household Deaths)
VR submitted to UN IGME or WHO

[1] Moultrie T, Dorrington R, Hill A, Hill K, Timæus I, Zaba B. Tools for Demographic Estimation [Internet]. International Union for the Scientific Study of Population; 2013. Available from: https://demographicestimation.iussp.org/content/introduction-tools-demographic-estimation

[2] Riffle T, Lima E, Queiroz B. Riffe T, Lima E, Queiroz B (2017). DDM: Death Registration Coverage Estimation. R package version 1.0-0. 2017.

[3] United Nations Statistics Division. Population censuses' datasets (1995-present) [Internet]. New York: United Nations; [cited 2026 Jan 6]. Available from: https://unstats.un.org/unsd/demographic-social/products/dyb/dybcensusdata.cshtm

[4] HMD. Human Mortality Database. Max Planck Institute for Demographic Research (Germany), University of California, Berkeley (USA), and French Institute for Demographic Studies (France). (available at www.mortality.org).

[5] Masquelier B, et al. Global, regional, and national mortality trends in youth aged 15–24 years between 1990 and 2019: a systematic analysis. The Lancet Global Health, 2021;9(4).

[6] 1. Pedersen J, Liu J. Child mortality estimation: appropriate time periods for child mortality estimates from full birth histories. PLoS Med. 2012;9(8):e1001289.

[7] Timaeus IM, Jasseh M. Adult mortality in sub-Saharan Africa: evidence from Demographic and Health Surveys. Demography. 2004 Nov;41(4):757–72.

[8] 1. Reniers G, Masquelier B, Gerland P. Adult Mortality in Africa. In: Rogers RG, Crimmins EM, editors. International Handbook of Adult Mortality [Internet]. Dordrecht: Springer Netherlands; 2011 [cited 2026 Jan 6]. p. 151–70. (International Handbooks of Population; vol. 2). Available from: https://link.springer.com/10.1007/978-90-481-9996-9_7

[9] 1. Alkema L, Chou D, Hogan D, Zhang S, Moller AB, Gemmill A, et al. Global, regional, and national levels and trends in maternal mortality between 1990 and 2015, with scenario-based projections to 2030: a systematic analysis by the UN Maternal Mortality Estimation Inter-Agency Group. Lancet. 2016 Jan 30;387(10017):462–74.

[10] United Nations Inter-agency Group for Child Mortality Estimation (UN IGME). Levels & Trends in Child Mortality: Report 2024 – Estimates developed by the United Nations Inter-agency Group for Child Mortality Estimation. New York; 2025.

[11] United Nations Inter-agency Group for Child Mortality Estimation (UN IGME). Child Mortality Estimates [Internet]. New York: United Nations Children's Fund (UNICEF); c2025 [cited 2026 Jan 6]. Available from: https://childmortality.org/

[12] Masquelier B, Hug L, Sharrow D, You D, Hogan D, Hill K, et al. Global, regional, and national mortality trends in older children and young adolescents (5-14 years) from 1990 to 2016: an analysis of empirical data. Lancet Glob Health. 2018 Oct;6(10):e1087–99..

~~[13] Hill K, You D, Inoue M, Oestergaard MZ, Technical Advisory Group of United Nations Inter-agency Group for Child Mortality Estimation. Child mortality estimation: accelerated progress in reducing global child mortality, 1990-2010. PLoS Med. 2012;9(8):e1001303.~~

[14] Sawyer CC. Child mortality estimation: estimating sex differences in childhood mortality since the 1970s. PLoS Med. 2012;9(8):e1001287.

[15] Chao F, Masquelier B, You D, Hug L, Liu Y, Sharrow D, et al. Sex differences in mortality among children, adolescents, and young people aged 0-24 years: a systematic assessment of national, regional, and global trends from 1990 to 2021. Lancet Glob Health. 2023 Oct;11(10):e1519–30.

[16] Delforge D, Wathelet V, Below R, Sofia CL, Tonnelier M, Van Loenhout JAF, et al. EM-DAT: the Emergency Events Database. International Journal of Disaster Risk Reduction. 2025 June;124:105509

[17] Davies S, Pettersson T, Sollenberg M, Öberg M. Organized violence 1989–2024, and the challenges of identifying civilian victims. Journal of Peace Research. 2025 July;62(4):1223–40.

[18] Sundberg R, Melander E. Introducing the UCDP Georeferenced Event Dataset. Journal of Peace Research. 2013 July;50(4):523–32.

[19] Raleigh C, Linke R, Hegre H, Karlsen J. Introducing ACLED: An Armed Conflict Location and Event Dataset. Journal of Peace Research. 2010 Sept;47(5):651–60.

[20] Center for Systemic Peace/Integrated Network for Societal Conflict Research (INSCR) datasets.)
[21] Mathers C, Sohn H, Castanheira H, You D, Hug L, Pelletier F, et al. Age-Sex Patterns of Crisis Deaths: Towards a more standard mortality estimation approach [Internet]. New York,; 2023 [cited 2026 Jan 1]. (Working paper, United Nations Children's Fund). Available from: https://childmortality.org/wp-content/uploads/2023/09/UN-IGME-Working-Paper.-Age-sex-Patterns-of-Crisis-Deaths.pdf
[22] United Nations, Department of Economic and Social Affairs, Population Division. World Population Prospects 2024. 2024.
